# Proteomics of *Streptococcus gordonii* within a model developing oral microbial community

**DOI:** 10.1186/1471-2180-12-211

**Published:** 2012-09-18

**Authors:** Erik L Hendrickson, Tiansong Wang, Brittany C Dickinson, Sarah E Whitmore, Christopher J Wright, Richard J Lamont, Murray Hackett

**Affiliations:** 1 Department of Chemical Engineering, University of Washington, Box 355014, Seattle, WA, 98195, USA; 2 Department of Microbiology, University of Washington, Box 357242, Seattle, WA, 98195, USA; 3 Department of Oral Biology, University of Florida, Gainesville, FL, 32610, USA; 4 Center for Oral Health and Systemic Disease, University of Louisville, Louisville, KY, 40292, USA

**Keywords:** *Streptococcus gordonii*, Oral biofilm, Proteomics, Model community, *Porphyromonas gingivalis*, *Fusobacterium nucleatum*

## Abstract

**Background:**

*Streptococcus gordonii* is one of several species that can initiate the formation of oral biofilms that develop into the complex multispecies microbial communities referred to as dental plaque. It is in the context of dental plaque that periodontal pathogens such as *Porphyromonas gingivalis* cause disease. We have previously reported a whole cell quantitative proteomics investigation of *P. gingivalis* in a model dental plaque community of *S. gordonii*, *P. gingivalis*, and *Fusobacterium nucleatum*. Here we report the adaptation of *S. gordonii* to the same model.

**Results:**

1122 *S. gordonii* proteins were detected in *S. gordonii* control samples, 915 in communities with *F. nucleatum*, 849 with *P. gingivalis*, and 649 with all three organisms. Quantitative comparisons showed extensive proteome changes in association with *F. nucleatum* or *P. gingivalis* individually or both *P. gingivalis* and *F. nucleatum* together. The changes were species specific, though the *P. gingivalis* interaction may be dominant, indicated by large differences between the proteomes with *F. nucleatum* or *P. gingivalis* but limited changes between communities with *P. gingivalis* or both *P. gingivalis* and *F. nucleatum*. The results were inspected manually and an ontology analysis conducted using DAVID. Extensive changes were seen in nutrition pathways with increases in energy metabolism and changes in the resulting byproducts, while the acid and sugar repressed PTS (phosphoenolpyruvate dependent phosphotransferase system) sugar transport systems showed decreases. These results were seen across all the multispecies samples, though with different profiles according to the partner species. *F. nucleatum* association decreased proteins for the metabolic end products acetate and ethanol but increased lactate, the primary source of acidity from streptococcal cultures. *P. gingivalis* containing samples had a reduction in levels of proteins for ethanol and formate but increased proteins for both acetate and lactate production. The communities also showed increases in exopolysaccharide synthesis, amino acid biosynthesis, and oxidative stress protection and decreases in adhesion and transporter proteins.

**Conclusion:**

This study showed that *S. gordonii* demonstrates species specific responses during interactions with *F. nucleatum* or *P. gingivalis*. Extensive changes were seen in energy metabolism and byproduct production implicating nutrient transfer as an important community interaction.

## Background

Oral infections, such as caries and periodontal disease, are among the most common instances of bacterial pathogenesis in humans. Current models of oral disease development center around the microbial communities found in dental plaque biofilms. Development of the dental plaque biofilm involves competition and cooperation among hundreds of different organisms. Early colonizing organisms, dominated by streptococci such as *S. gordonii*[1], bind to a variety of host derived molecules coating oral surfaces known as the acquired pellicle. Secondary colonizing species then adhere to those bound to the pellicle. *Fusobacterium nucleatum* can bind these early colonizing organisms and later additions to the biofilm [2]. In addition, *F. nucleatum* is aerotolerant and metabolic activity can reduce the concentration of oxygen to levels that can be tolerated by more pathogenic organisms such as *P. gingivalis*[3]. *P. gingivalis* can bind to both *F. nucleatum* and *S. gordonii*[4,5], and these organisms are metabolically compatible when associated [3,6]. While destruction of periodontal tissue is generally associated with later colonizers like *P. gingivalis*, pathogenicity is expressed within the context of the microbial community.

It has recently been shown that nutrient transfer within a community can play an important role in pathogenicity [7]. Co-culture with *S. gordonii* resulted in increased virulence of the periodontal pathogen *Aggregatibacter actinomycetemcomitans*. The increase was dependent on the ability of *A. actinomycetemcomitans* to utilize L-lactate, a byproduct of *S. gordonii* energy metabolism, as an energy source. Furthermore, a mutant strain unable to utilize L-lactate showed significantly decreased virulence in the co-culture highlighting the importance of metabolite cross-feeding.

Oral microbial communities are also known for altering their local environment. The most striking example occurs in dental caries where species such as *Streptococcus mutans* significantly reduce the pH to a point where enamel is demineralized [8]. This shift in ecology also effects the development of the dental plaque, selecting for more aciduric organisms such as lactobacilli. While *S. gordonii* does not produce acid at the same levels or at lower pH as does *S. mutans*, *S. gordonii* has been found to produce acid down to pH 5.5 [9] and may also change the local ecology during formation of dental plaque.

The large number of species involved, the heterogeneity between hosts as well as within the oral cavity, and the small sample sizes that can be harvested from the oral cavity compared to laboratory grown samples, all present significant experimental challenges in examining microbial interactions in dental plaque development. In order to investigate these interactions in a more experimentally tractable system [10], we have developed a model of nascent community interactions [11] using three representative species of oral bacteria, *S. gordonii*, *F. nucleatum*, and *P. gingivalis.* We have previously reported our results for *P. gingivalis* protein expression, which showed extensive changes in 18 hour pellets with *S. gordonii* and *F. nucleatum*, especially in the cell envelope proteome and in vitamin synthesis pathways [11]. Here we report changes in *S. gordonii* protein levels in model nascent communities with *F. nucleatum*, *P. gingivalis*, and all three species combined.

## Results and discussion

**Figure 1:**
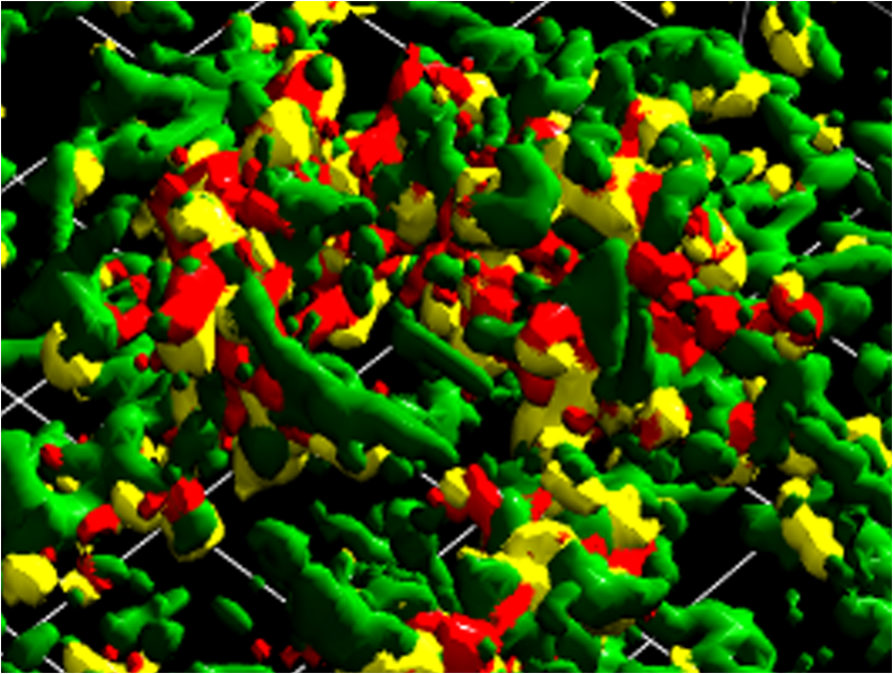
**Multispecies community of*S. gordonii*,*P. gingivalis*and*F. nucleatum*.** Confocal laser scanning analysis of heterotypic communities of *S. gordonii* (red), *F. nucleatum* (green) and *P. gingivalis* (yellow). Bacterial accumulations were analysed on an Olympus FV500 laser scanning confocal microscope. A series of 1 μm fluorescent slices were re-constructed using Volocity software. The area shown measures approximately 40 × 50 μm.

Bacteria in the oral cavity assemble into complex heterotypic communities that engage in multilevel signaling and response interactions [12,13]. Bacteria can communicate through direct contact; soluble secreted factors such as autoinducers; and detection and utilization of metabolic products of partner species [14,15]. Proteomic investigation of such communities in vitro presents numerous challenges including sample size and relevance to the in vivo situation. We have developed a model that includes elements from three major species of dental biofilms that represent early (*S. gordonii*) mid (*F. nucleatum*) and late (*P. gingivalis*) colonizers, organisms that form close associations with one another, as illustrated using scanning confocal microscopy (Figure 1). By generating pellets of these organisms, we have provided conditions under which they are in close contact, thus allowing signaling through contact dependent mechanisms and short range chemical mediators. This model also allows separation of the interaction stage of community development (our major interest) from community development through bacterial growth and division. By avoiding growth cycles influenced by nutrient diffusion, there is less opportunity for results to be confounded by differential protein expression due to different physiological microniches.

### Protein detection

The whole cell proteome of *S. gordonii* was measured either alone in a single species assembled 18 hour biofilm or in communities with *F. nucleatum* (SgFn), *P. gingivalis* (SgPg), or both *P. gingivalis* and *F. nucleatum* (SgPgFn). Table 1 shows the number of *S. gordonii* proteins identified by three or more unique peptides across two biological replicates of each sample. The number of identified proteins is lower in the mixed samples relative to the single species control as the percentage of the extracted proteins originating from *S. gordonii* is lower in the mixed community than in a pure Sg sample.

**Table 1:** ***S. gordonii*proteins detected in communities**

**Organism(s)**	**Proteins detected**
*S. gordonii*	1122
SgFn	915
SgPg	849
SgPgFn	649

Protein levels, as measured by spectral counting (see Methods), were compared among all samples. Proteins were considered significantly altered between conditions at *q* values of 0.005 and lower. Table 2 shows numbers of increased, decreased, and unchanged proteins for all six comparisons. Relative abundance calculations were only carried out for proteins detected in both conditions being compared, i.e. no artificial baselines in place of missing data were used. Therefore increased and decreased protein levels are also expressed as a percentage of the shared proteins detected in both states. The *S. gordonii* proteome undergoes substantial changes when exposed to Fn or Pg with 45 to 54% of the detected proteins showing altered levels compared to Sg alone (SgFn vs Sg, SgPg vs Sg, and SgPgFn vs Sg). While Sg showed many relative abundance changes with either Fn or Pg, the responses are distinct and species specific as seen in the large differences between the SgPg and SgFn preparations (SgPg vs SgFn). However, the response to Pg appears to be dominant. Sg in a community with both Pg and Fn shows significant differences compared to the SgFn community but is very similar to the SgPg community (SgPgFn vs SgFn, SgPgFn vs SgPg). This is in keeping with models of dental plaque development whereby the pathogenic potential alters as later colonizers become established [16]. A short format summary table of all data presented in this report can be found in Additional file 1. Additional files 2, 3, 4, 5, 6, 7 present the data in somewhat greater detail for each proteome quantitative comparison, including both raw and normalized spectral counts and associated statistics. Qualitative protein coverage information is summarized in Additional file 8. Additional file 9 shows a whole genome plot of the SgPgFn vs Sg comparison. Plots comparing spectral counts for technical replicates and spectral counts for each biological replicate are found in Additional file 10, as well as additional remarks about data reproducibility and the effects of normalization. The high correlations shown suggest that the detected changes are due primarily to differences between the conditions being compared rather than random variability in the measurements. The original FileMaker™ database from which additional files 1, 2, 3, 4, 5, 6, 7, 8 were derived is available from the corresponding author. The raw data has been archived in a remote secure location as part of the University of Washington’s *lolo* file retrieval system, and will also be made available through the United States Department of Energy’s Joint Genome Institute (JGI), and possibly other sites pending ongoing discussions in the proteomics community with respect to best practices for permanent archival storage.

**Table 2:** **Relative abundance changes observed for the*S. gordonii*expressed proteome**

**Comparison**	**Unchanged**	**Increased**	**Decreased**
SgFn vs *S. gordonii*	421	188 (24%)	160 (21%)
SgPg vs *S. gordonii*	389	212 (25%)	200 (26%)
SgPgFn vs *S. gordonii*	287	163 (26%)	174 (28%)
SgPg vs SgFn	375	161 (23%)	177 (25%)
SgPg Fn vs SgFn	327	111 (19%)	146 (25%)
SgPg Fn vs SgPg	556	15 (2%)	56 (9%)

### Energy metabolism and sugar transport

**Figure 2:**
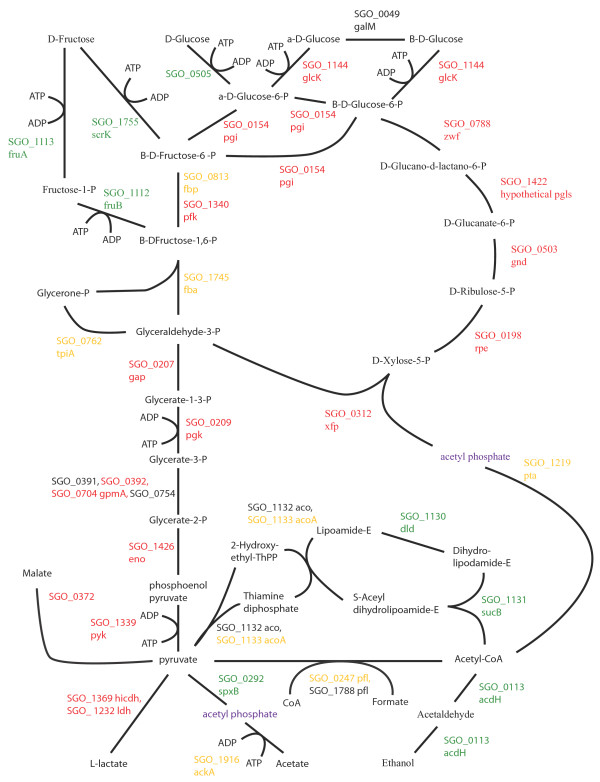
**SgFn vs Sg Energy metabolism and end products.** The diagram shows a schematic of the glycolysis and pentose phosphate pathways for Sg including the end products of the metabolism, formate, acetate, L-lactate, and ethanol for the *S. gordonii* with *F. nucleatum* sample compared to *S. gordonii*. Proteins catalyzing each step are shown by their *S. gordonii* SGO designation, some include a protein abbreviation. Red numbers indicate increased levels in the first condition compared to the second condition, green decreased levels, yellow no statistical change, and black undetected in at least one of the conditions. Abbreviations: acdH: alcohol-acetaldehyde dehydrogenase; ackA: acetate kinase A; acoA: acetoin dehydrogenase; dld: dihydrolipoamide dehydrogenase; eno: enolase; fba: fructose-1,6-bisphosphate aldolase; fbp: fructose-bisphosphatase; fruA: fructose specific phosphoenolpyruvate-dependent phosphotransferase systems component II; fruB: 1-phosphofructokinase; galM: aldose 1-epimerase; gap: glyceraldehydes-3-phosphate dehydrogenase; glcK: glucokinase; gnd: 6-phosphogluconate dehydrogenase; gpmA: 2,3-bisphosphoglycerate-dependent phosphoglycerate mutase; hicdh: L-2-hydroxyisocaproate dehydrogenase; ldh: lactate dehydrogenase; pfk: phosphofructokinase; pfl: pyruvate formate lyase; pgi: glucose-6-phosphate isomerase; pgk: phosphoglycerate kinase; pgls: 6-phosphogluconolactonase; pta: phosphate acetlytransferase; pyk: pyruvate kinase; rpe: ribulose-phosphate 3-epimerase; scrK: fructokinase; spxB: pyruvate oxidase; sucB: dihydrolipoamide S-acetyltransferase; tpiA: triosephosphate isomerase; xfp: D-xululose 5-phosphate/ D-fructose 6-phosphate phosphoketolase; zwf: glucose-6-phosphate 1-dehydrogenase.

**Figure 3:**
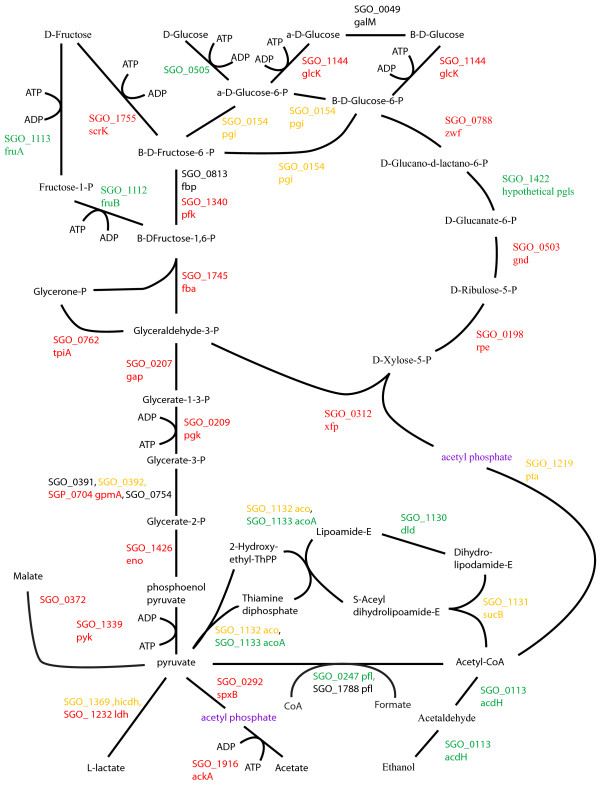
**SgPg vs Sg Energy metabolism and end products.** Labels, abbreviations and color coding as described for Figure 2, for the *S. gordonii* with *P. gingivalis* comparison to *S. gordonii.*

**Figure 4:**
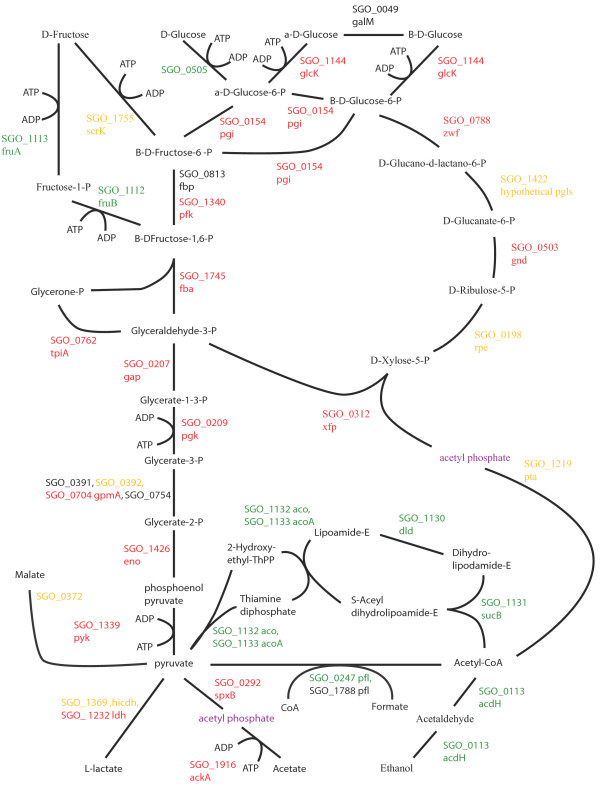
**SgPgFn vs Sg energy metabolism and end products.** Labels, abbreviations and color coding as described for Figure 2, for the *S. gordonii* with *P. gingivalis* and *F. nucleatum* comparison to *S. gordonii.*

**Figure 5:**
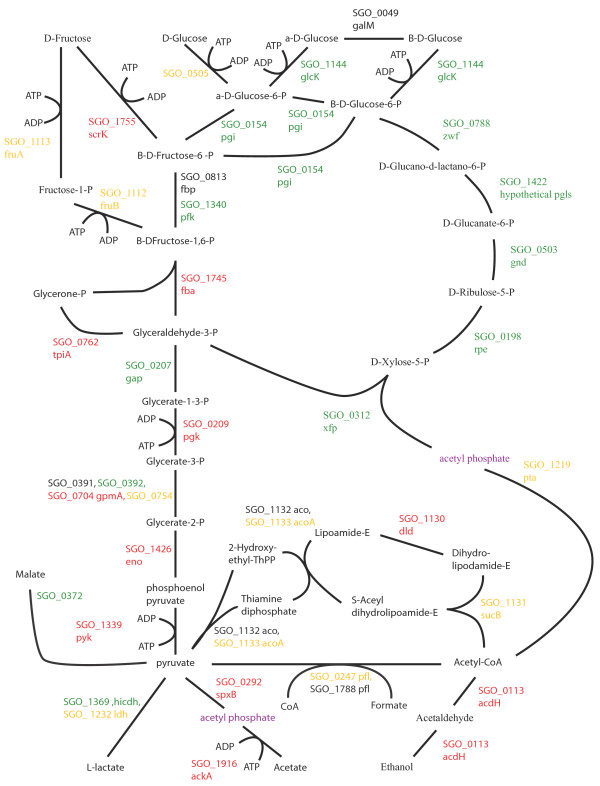
**SgPg vs SgFn Energy metabolism and end products.** Labels, abbreviations and color coding as described for Figure 2, for the *S. gordonii* with *P. gingivalis* comparison to *S. gordonii* with *F. nucleatum.*

**Figure 6:**
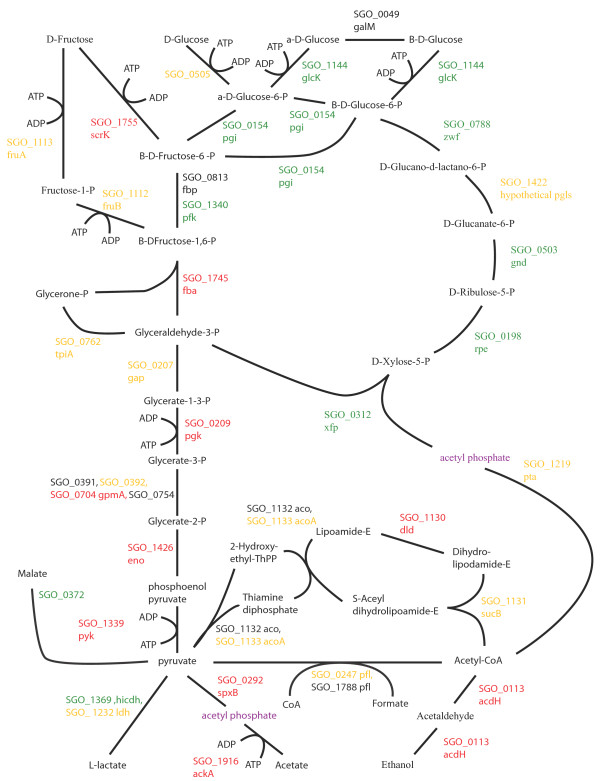
**SgPgFn vs SgFn Energy Metabolism and End Products.** Labels, abbreviations and color coding as described for Figure 2, for the *S. gordonii* with *P. gingivalis* and *F. nucleatum* comparison to *S. gordonii* with *F. nucleatum.*

**Figure 7:**
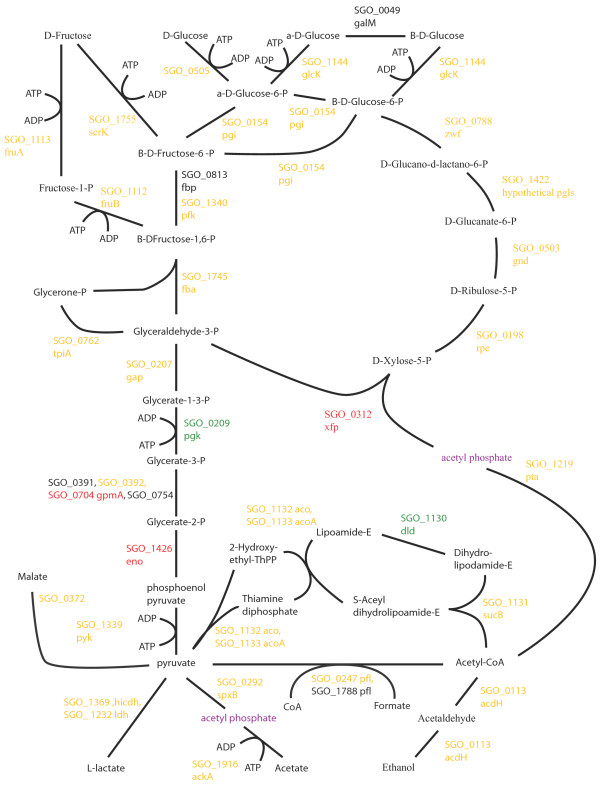
**SgPg Fn vs SgPg Energy metabolism and end products.** Labels, abbreviations and color coding as described for Figure 2, for the *S. gordonii* with *P. gingivalis* and *F. nucleatum* comparison to *S. gordonii* with *P. gingivalis.*

Changes to pathways for energy metabolism and sugar transport in the multispecies communities were consistent with a higher level of available energy metabolites and a lower pH. Oral streptococcal species primarily derive their energy from the breakdown of carbohydrates. Figures 2, 3, 4, 5, 6, 7 compare energy metabolism pathway proteins between the different communities (2 SgFn vs Sg, 3 SgPg vs Sg, 4 SgPgFn vs Sg, 5 SgPg vs SgFn, 6 SgPgFn vs SgFn, 7 SgPgFn vs SgPg). Compared to Sg alone the multispecies communities showed increased levels for both the glycolysis pathway and the pentose phosphate pathway, implying higher energy availability (Figures 2, 3, 4). The presence of Pg appeared to be dominant as SgPgFn was very similar to SgPg (Figure 7). Even though both pathways were increased in the presence of Fn or Pg there was a difference in emphasis (Figure 5). Sg in contact with Pg had larger increases in the glycolysis pathway while Sg with Fn had larger increases in the pentose phosphate pathway. SgFn and SgPg also showed increased levels of malate oxidoreductase that converts malate to pyruvate (Figures 2, 3) with SgFn showing a larger increase (Figure 5).

Higher levels of available sugars could explain the increase in energy pathways, but would require importing the sugars into the cell. Oral streptococci transport sugar using two primary systems: the phosphoenolpyruvate mediated phosphotransferase (PTS) system, which moves sugars across the membrane with concomitant phosphorylation; and the proton motive force (PMF) system [8,17], though the specific proteins for the PMF system have not yet been identified. Both systems are known to be regulated. While the lactose-PTS in *S. mutans* is induced by lactose, PTS activity is generally repressed under sugar excess. The PTS is also repressed at low pH while the PMF system is induced under low pH. Together the systems are believed to provide *Streptococcus* species with a high affinity scavenger system under sugar limited conditions, and a low affinity system taking advantage of the proton motive force available under low environmental pH.

**Figure 8:**
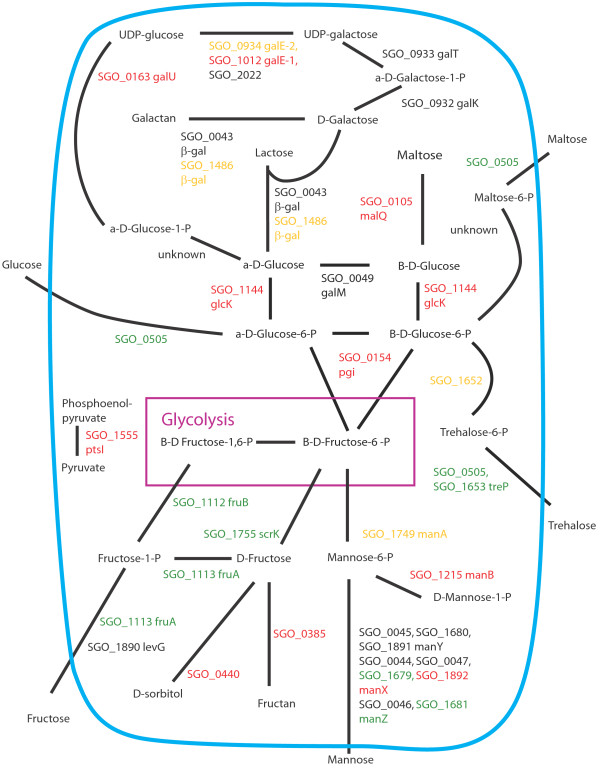
**SgFn vs Sg Sugar transport.** The diagram shows a schematic of sugar transport across the cell membrane and reactions feeding into the glycolysis pathway for Sg for the *S. gordonii* with *F. nucleatum* samples compared to *S. gordonii*. Proteins catalyzing each step are shown by their *S. gordonii* SGO designation, some include a protein abbreviation. The purple box represents the glycolysis pathway and the blue line the cell membrane. Red numbers indicate increased levels in the first condition compared to the second condition, green decreased levels, yellow no statistical change, and black undetected in at least one of the conditions. Abbreviations: β-gal: β-galactosidase; fruA: fructose specific phosphoenolpyruvate-dependent phosphotransferase systems component II; fruB: 1-phosphofructokinase; galE-1: UDP-glucose 4-epimerase; galE-2: UDP-glucose 4-epimerase; galK: galactokinase; galM: aldose 1-epimerase; galT: galactose-1-phosphate uridylytransferase; galU: UTP-glucose-1-phosphate uridylytransferase; glcK: glucokinase; malQ: 4-alpha-glucanotransferase; manA: mannose-6-phosphate isomerase; manB: phosphomannomutase; manX: mannose-specific phosphoenolpyruvate-dependent phosphotransferase systems component IIB; manY: mannose-specific phosphoenolpyruvate-dependent phosphotransferase systems component IIC; manZ: mannose-specific phosphoenolpyruvate-dependent phosphotransferase systems component IID; ptsI: phosphoenolpyruvate-protein phosphotransferase; scrK: fructokinase; treP: trehalose phosphoenolpyruvate-dependent phosphotransferase systems component II.

**Figure 9:**
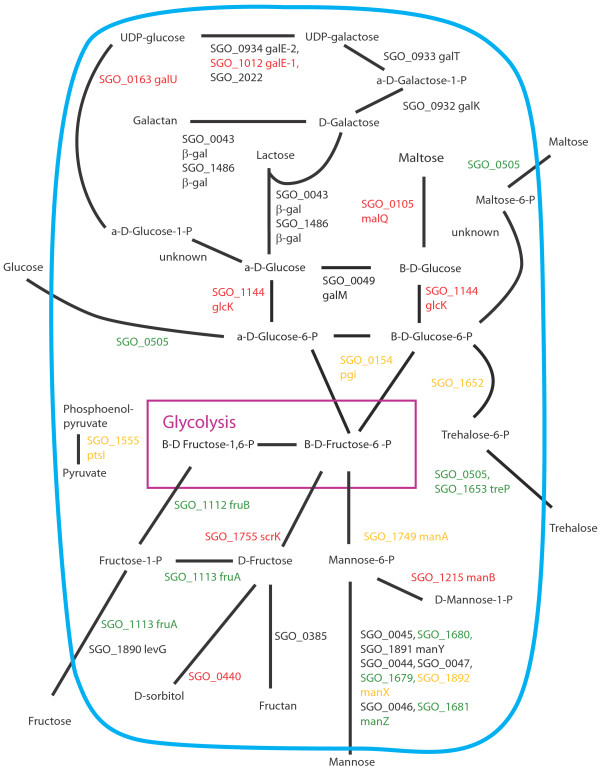
**SgPg vs Sg Sugar transport.** Labels, abbreviations and color coding as described for Figure 8, for the *S. gordonii* with *P. gingivalis* comparison to *S. gordonii.*

**Figure 10:**
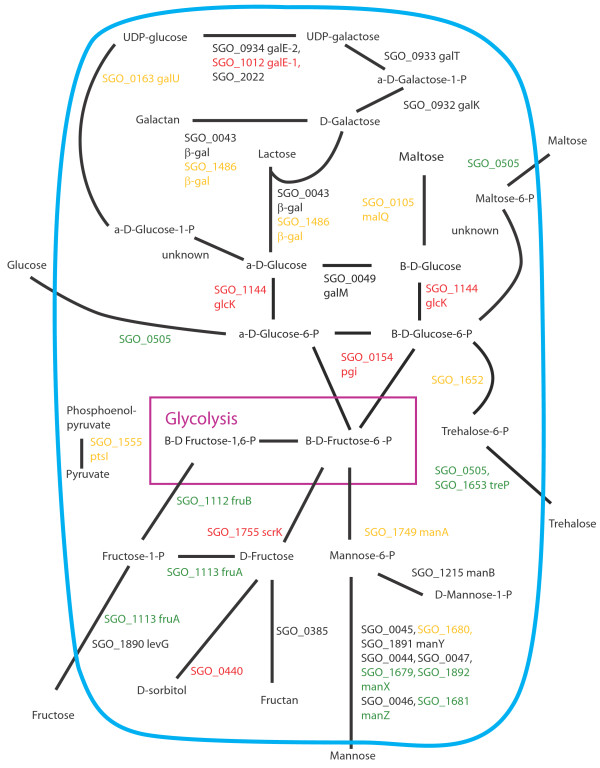
**SgPgFn vs Sg Energy metabolism and end products.** Labels, abbreviations and color coding as described for Figure 8, for the *S. gordonii* with *P. gingivalis* and *F. nucleatum* comparison to *S. gordonii.*

**Figure 11:**
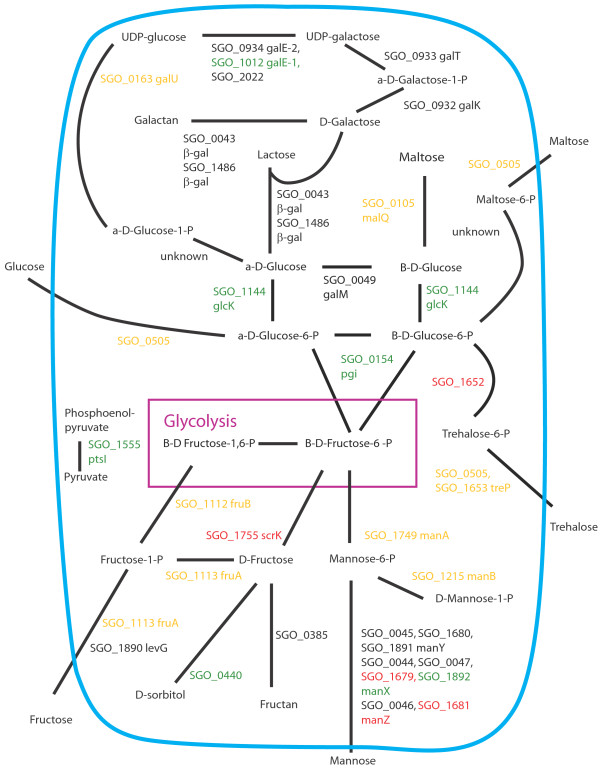
**SgPg vs SgFn Energy metabolism and end products.** Labels, abbreviations and color coding as described for Figure 8, for the *S. gordonii* with *P. gingivalis* comparison to *S. gordonii* with *F. nucleatum.*

**Figure 12:**
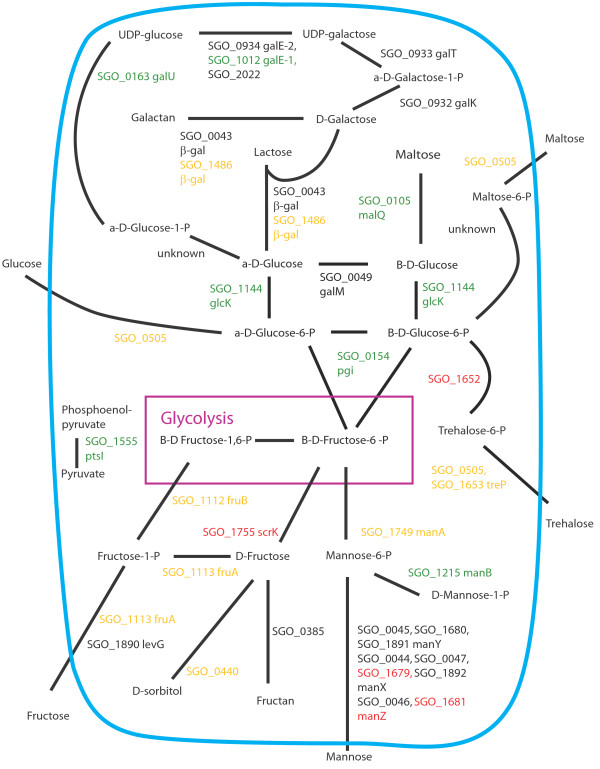
**SgPgFn vs SgFn Energy metabolism and end products.** Labels, abbreviations and color coding as described for Figure 8, for the *S. gordonii* with *P. gingivalis* and *F. nucleatum* comparison to *S. gordonii* with *F. nucleatum.*

**Figure 13:**
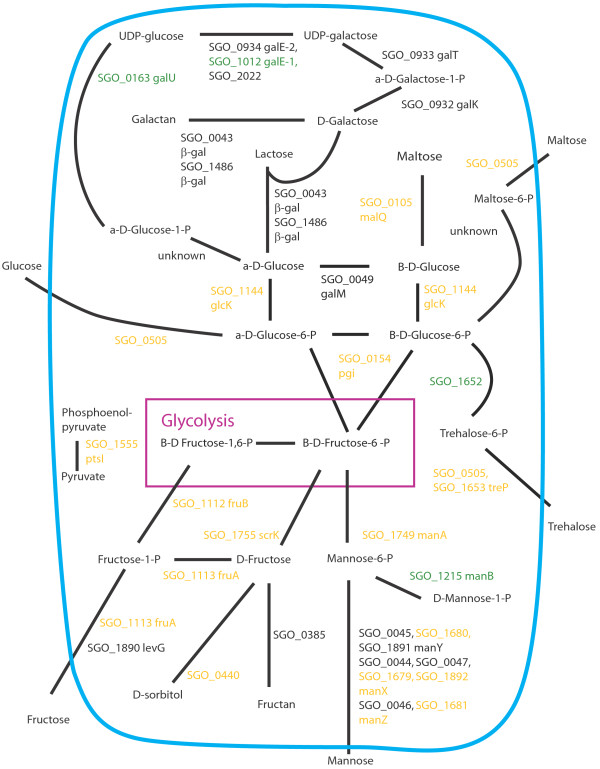
**SgPgFn vs SgPg Energy metabolism and end products.** Labels, abbreviations and color coding as described for Figure 8, for the *S. gordonii* with *P. gingivalis* and *F. nucleatum* comparison to *S. gordonii* with *P. gingivalis.*

Figures 8, 9, 10, 11, 12, 13 show comparisons between the communities for PTS transport systems and pathways feeding sugars into glycolysis. These are a subset of annotated systems including only those with detected proteins. The multispecies communities show a reduction in almost all detected PTS components compared to Sg alone (Figures 8, 9, 10). The exceptions are one protein in the multiple protein complex for transporting mannose, either SGO_1680 (SgPgFn vs Sg) or SGO_1892 (SgFn vs Sg, SgPg vs Sg) depending on the comparison, and SGO_1555, PtsI, the sugar non-specific component of the PTS that provides the phosphoryl group for the reaction to a carrier protein. These are increased in SgFn and show no change in SgPg and SgPgFn (Figures 8, 9, 10). Overall, the indication is a reduction in transport from the PTS system, consistent with sugar excess and/ or low pH.

In contrast to the PTS system proteins, many of the proteins feeding sugars into the glycolysis and pentose phosphate pathways show increased levels in mixed communities (Figures 8, 9, 10). This is consistent with the higher protein levels in the energy pathways as well as high levels of available sugar. The implication is that the second, low pH induced, pathway has high activity under the mixed community conditions. Induction of the second sugar transport system would again be consistent with a low pH environment. While Sg does not commonly reduce pH to levels where demineralization occurs, it can produce acid at pH’s as low as 5.5 and so could be responsible for a lower pH in the mixed communities [9]. It is important to note that these experiments were conducted in media without exogenous nutrients and thus Sg may be undergoing a programmed response to the presence of the other species, rather than a response to altered nutrient levels.

### Alcohols and acidic end products

In mixed species communities Sg showed an extensive shift in pathways for byproduct production. The end products of energy metabolism are often important components of pathogenicity and community development. Changes in pH can select for different organisms [3]. End products can also provide nutrients for other community members. *S. gordonii* has been shown to increase *A. actinomycetemcomitans* pathogenicity through metabolic cross-feeding of L-lactate [7].

Figures 2, 3, 4, 5, 6, 7 show the end products of Sg energy metabolism, formate, acetate, L-lactate, and ethanol. Compared to Sg alone, SgFn showed a reduction in the proteins for ethanol and acetate production and an increase in those for lactate (Figure 2). SgPg and SgPgFn also had an increase in proteins for lactate production and a decrease in the ethanol pathway (Figures 3, 4). However, neither was as strong as that seen in SgFn (Figure 5). In contrast, SgPg and SgPgFn displayed an increase rather than a decrease in the pathway to acetate (Figures 3, 4). These combinations also showed a decrease in the enzyme for decarboxylation of pyruvate that produces formate as a byproduct (Figures 3, 4).

Overall, exposure to Pg caused a shift away from ethanol and formate towards acetate and lactate, while SgFn shifted away from acetate and ethanol heavily towards lactate formation. While an asaccharolytic organism like Pg is unlikely to make use of L-lactate it is interesting to see a shift in all the mixed cultures towards lactate production. Given the increased *A. actinomycetemcomitans* pathogenicity in Sg co-culture from L-lactate transfer [7], shifting to higher lactate production might be a typical Sg response to the presence of other oral species.

The presence of excess sugars and rapid growth have also been associated with a shift towards lactate in *S. mutans*[18]. However, as mentioned above, the cultures were not provided with exogenous nutrients so the likelihood of rapid growth under our experimental conditions was low. Hence, these results are more consistent with *S. gordonii* utilizing the presence of other organisms as a proxy for nutritional availability in developing plaque.

### Adhesion

Proteins that enhance bacterial binding to dental surfaces and other bacteria are important for the formation of dental plaque [19]. Table 3 shows the protein ratios for adhesion proteins across the six comparisons. Almost all detected proteins showed statistically significant decreases compared to levels in Sg alone. This includes amylase binding protein, SGO_2105, which plays an important role in plaque formation by binding salivary amylase [20]. Streptococcal surface proteins (Ssp) A and B, SGO_0210 and SGO_0211, are important for binding Pg via the Mfa1 receptor [5]. Table 3 shows that SspA is down in SgPg vs Sg and SspB is down in SgFn vs Sg. Cell surface protein CshA, SGO_0854, has been shown to be important in binding the oral microbes *Actinomyces naeslundii* and *Streptococcus oralis* as well as the host adhesion target human fibronectin [21]. CshA was down in SgFn, SgPg, and SgPgFn compared to Sg. Mutations in CshB, SGO_1148, also decreased binding but reduced CshA levels and that may account for the binding differences [21]. CshB was down in SgFn vs Sg and undetected in the other samples. In contrast, the fibronectin binding protein SGO_0855 showed no statistical differences between samples. Streptococcal hemagglutinin, Hsa SGO_0966, which binds to erythrocytes and plays a role in infective endocarditis [22], was down-regulated in the one comparison where it was detected, SgFn vs Sg.

**Table 3:** Regulation of adhesion proteins

**Protein**	**SgFn vs Sg**	**SgPg vs Sg**	**SgPgFn vs Sg**	**SgPg vs SgFn**	**SgPgFn vs SgFn**	**SgPgFn vs SgPg**
SGO_0210	−1.0	**−5.4**	nd	**−4.5**	nd	nd
SGO_0211	**−4.1**	nd	nd	nd	nd	nd
SGO_0854	**−2.7**	**−5.3**	**−5.4**	**−2.6**	**−2.7**	−0.1
SGO_0855	−0.7	−0.5	nd	0.3	nd	nd
SGO_0966	**−1.3**	nd	nd	nd	nd	nd
SGO_1148	**−2.7**	nd	nd	nd	nd	nd
SGO_2105	**−1.9**	**−6.9**	**−6.4**	**−5.0**	**−4.4**	**0.6**

Bold: statistically significant difference, all ratios are log_2_.

nd: not detected in one or more of the compared samples.

While the short fimbriae of Pg have been found to bind to Sg via SspB, the long fimbriae bind to streptococci using the metabolic protein glyceraldehyde-3-phosphate dehydrogenase, Gap [23]. Sg GAPDH, SGO_0207, shows increased protein levels with SgFn, SgPg, and SgPgFn vs Sg and may indicate an increased need for Gap mediated inter-species adhesion in the mixed samples. However, Gap functions not only as an adhesin but is also part of the glycolysis pathway. The rest of the pathway showed increased levels and the increases in GAPDH may be related to its metabolic function rather than binding. This would be consistent with the reduced levels seen with the other adhesins.

Despite the inconsistent detection of many of the known adhesins, overall, adhesion protein levels appear to be down in the mixed species samples. This is consistent with earlier studies showing that after initial contact, organisms in communities down-regulate adhesin expression [24].

### Surface proteins and cell wall synthesis

In addition to proteins with known functions in binding, many proteins predicted to be located on the cell surface were found at significantly different levels in the community samples. Table 4 shows significant differences, mostly lower, in many of the detected surface proteins between the community samples and Sg alone. There are also numerous changes in proteins predicted to participate in cell wall biosynthesis (Table 4). Comparing community to Sg samples both increased and decreased protein levels are seen, though SgPgFn vs Sg was skewed towards reduced levels. Proteins for synthesis and attachment of the cell membrane sugar rhamnose show an interesting pattern. The results for these proteins are shown in Table 5. Rhamnose synthesizing proteins show generally increased levels with SgFn, SgPg, and SgPgFn compared to Sg alone with even higher levels in the Fn community than with Pg or PgFn. However, the rhamnosyltransferase, SGO_1026, which would attach rhamnose to the cell membrane, is down compared to Sg. One possible explanation is a shift between different rhamnosyltransferases. Sg has three, SGO_1021, SGO_1022, and SGO_1026. We failed to detect SGO_1021 or SGO_1022 in all but the Sg single species controls.

**Table 4:** Predicted surface and cell wall biosynthesis proteins

**Category**	**Change**	**SgFn vs*Sg***	**SgPg vs Sg**	**SgPgFn vs Sg**	**SgPg vs SgFn**	**SgPgFn vs SgFn**	**SgPgFn vs SgPg**
Other Surface Proteins ^a^	Total	27	16	12	17	12	12
	Unchanged	16	4	2	11	9	11
	Increase	3	3	1	2	1	0
	Decrease	8	9	9	4	2	1
Cell Wall Metabolism ^b^	Total	36	26	23	29	23	23
	Unchanged	16	15	8	17	15	18
	Increase	11	6	5	4	0	0
	Decrease	9	5	10	8	8	5

^a^ Covers SGO_0004, 0068, 0208, 0233, 0388, 0430, 0455, 0494, 0502, 0521, 0707, 0721, 0825, 0890, 1069, 1082, 1110, 1189, 1347, 1355, 1381, 1487, 1651, 1898, 2004, 2005, 2082.

^b^ Covers SGO_0054, 0056, 0057, 0327, 0393, 0515, 0586, 0631, 0671, 0672, 0763, 0804, 0886, 0948, 0969, 0975, 1009, 1010, 1011, 1013, 1020, 1025, 1026, 1400, 1446, 1447, 1623, 1624, 1638, 1676, 1717, 1768, 1854, 2010, 2104, 2037.

**Table 5:** Protein ratios for rhamnose synthesis and attachment

**Protein**	**SgFn vs Sg**	**SgPg vs Sg**	**SgPgFn vs Sg**	**SgPg vs SgFn**	**SgPgFn vs SgFn**	**SgPgFn vs SgPg**
SGO_1009	**2.1**	**1.5**	**1.1**	**−0.6**	**−0.9**	−0.4
SGO_1010	**0.8**	**0.9**	**0.9**	0.1	0.1	0
SGO_1011	**2.4**	**1.2**	0.6	**−1.2**	**−1.7**	−0.5
SGO_1020	**1.1**	0.7	**0.5**	−0.5	**−0.6**	−0.1
SGO_1026	**−1.9**	**−2.2**	**−3.2**	−0.2	**−1.3**	**−1.0**

Bold: statistically significant difference, all ratios are log_2_.

### Transport and export

As mentioned above, PTS sugar transport systems are almost all reduced in the mixed organism samples. The other transport and export proteins are also generally reduced in the mixed samples as shown in Table 6. Some exceptions show increases compared to Sg alone and are shown in detail in Table 7. Two, SGO_0006 and SGO_2100, are ABC transporter proteins with unknown substrates. SGO_1059 is a phosphate transport protein showing significantly lower levels in SgFn vs Sg but higher levels with SgPg or SgPgFn. Interestingly, the phosphate transport system regulatory protein, SGO_1060, is significantly down in SgFn and SgPgFn implying another level of regulation for SGO_1059. In contrast to the phosphate transporter, the predicted Trk potassium uptake system protein, SGO_1666, is up in SgFn but significantly reduced in SgPgFn.

**Table 6:** **Export and non-PTS transport proteins**^**a**^

	**SgFn vs Sg**	**SgPg vs Sg**	**SgPgFn vs Sg**	**SgPg vs SgFn**	**SgPgFn vs SgFn**	**SgPgFn vs SgPg**
Total	61	58	45	58	45	44
Unchanged	18	15	6	26	19	38
Increased	3	5	4	24	18	1
Decreased	40	38	35	8	8	5

^a^ Proteins covered SGO_0006, 0015, 0104, 0255, 0291, 0352, 0353, 0398, 0415, 0457, 0458, 0460, 0488, 0505, 0538, 0548, 0579, 0750, 0751, 0767, 0798, 0805, 0808, 0851, 0856, 0955, 0982, 1024, 1036, 1037, 1059, 1060, 1118, 1123, 1216, 1338, 1342, 1458,1465,1572, 1580, 1605, 1619, 1626, 1630, 1634, 1666, 1708, 1709, 1711, 1712, 1713, 1715, 1716, 1727, 1728, 1744, 1763, 1802, 1936, 2100.

**Table 7:** Protein ratios of selected export and transport proteins

**Protein**	**SgFn vs Sg**	**SgPg vs Sg**	**SgPgFn vs Sg**	**SgPg vs SgFn**	**SgPgFn vs SgFn**	**SgPgFn vs SgPg**
SGO_0006	0.2	**0.5**	0.1	0.3	−0.1	−0.4
SGO_0255	**−1.9**	**−1.6**	**−2.2**	0.3	−0.2	−0.1
SGO_0415	**−1.1**	**−1.0**	**−1.2**	0.1	−0.1	−0.1
SGO_1059	**−1.6**	**0.5**	**0.8**	**2.1**	**2.4**	**0.3**
SGO_1060	**−1.6**	−0.2	**−1.3**	**1.4**	0.4	**−1.0**
SGO_1123	**1.0**	**1.2**	**1.0**	**0.3**	0.0	−0.2
SGO_1216	**1.4**	**1.8**	**1.2**	0.4	−0.2	−0.6
SGO_1338	−0.7	−3.0	nd	**−2.3**	nd	nd
SGO_1666	**0.8**	−1.0	**−2.5**	**−1.8**	**−3.3**	−1.5
SGO_2100	**−0.9**	**2.6**	**2.7**	**3.5**	**3.6**	0.1

Bold: statistically significant difference, all ratios are log_2_.

nd: not detected in one or more of the compared samples.

The signal recognition particle protein, Ffh SGO_1123, is also increased in all of the mixed communities. Ffh binds to protein’s signal sequences when they emerge from the ribosome and is necessary for efficient extracytoplasmic protein export. Both SecA, SGO_0415, the only detected sec protein, and SGO_0255, one of two detected signal peptidases, showed significant reduction in the mixed communities (Table 7). SGO_1338, the other detected signal peptidase, showed reduced levels but did not make the statistical cutoff. The implication is that the mixed communities had an increase in integral membrane proteins, primarily those processed by Ffh and often SecA independent, but a decrease in periplasmic and extracellular proteins, primarily those processed via the sec pathway [25].

Bacteriocins, toxins that kill or inhibit closely related species, may experience increased export. The predicted bacteriocin transport accessory protein, SGO_1216, showed increased levels in all mixed communities. Bacteriocin production could be part of a strategy adopted by Sg to influence its mixed species environment, explaining the increase in all mixed organism samples. However, none of the other annotated bacteriocin proteins were detected. Also, SGO_1216 is not associated with the other bacteriocin proteins and may be a mis-annotation.

### Transcriptional regulation

Table 8 summarizes the results for predicted transcriptional regulators. Approximately a third of the detected regulators show statistically altered levels in the mixed communities. A subset of the regulatory proteins, those discussed below, is shown in Table 9. Most of these proteins have only a general prediction of transcriptional regulatory function, though they may be interesting targets for further investigation.

**Table 8:** **Transcriptional Regulators**^**a**^

	**SgFn vs Sg**	**SgPg vs Sg**	**SgPgFn vs Sg**	**SgPg vs SgFn**	**SgPgFn vs SgFn**	**SgPgFn vs SgPg**
Total	31	24	14	24	14	14
Unchanged	20	17	10	14	10	14
Increased	9	3	1	2	1	0
Decreased	2	4	3	8	3	0

^a^ Covers proteins SGO_0042, 0100, 0182, 0202, 0237, 0252, 0374, 0400, 0431, 0484, 0508, 0535, 0603, 0755, 0773, 0779, 0981, 1072, 1073, 1228, 1257, 1281, 1365, 1699, 1731, 1739, 1792, 1814, 1816, 1878, 1993.

**Table 9:** Protein Ratios of Selected Transcriptional Regulators and Regulated Proteins

**Protein**	**SgFn vs Sg**	**SgPg vs Sg**	**SgPgFn vs Sg**	**SgPg vs SgFn**	**SgPgFn vs SgFn**	**SgPgFn vs SgPg**
SGO_0237	**0.8**	**1.3**	0.2	0.5	−0.6	−1.1
SGO_0773	**−2.3**	**−2.4**	**−2.5**	−0.1	−0.2	−0.1
SGO_1072	**3.9**	1.3*	nd	**−2.6**	nd	nd
SGO_1073	−0.8	−2.1	nd	−1.3	nd	nd
SGO_1800	nd	**−2.2**	**−2.8**	nd	nd	−0.7
SGO_1801	nd	nd	nd	nd	nd	nd
SGO_1802	**−6.2**	**−2.7**	**−3.4**	**3.4**	**2.8**	−0.6
SGO_1816	**0.9**	0.1	nd	−0.7	nd	nd

Bold: statistically significant difference, all ratios are log_2_.

nd: not detected in one or more of the compared samples.

* insufficient detection to determine significance.

Two of those proteins with functional predictions from the annotation, SGO_0237 and SGO_0773, have homology to catabolite control protein A, CcpA. CcpA is a transcriptional regulator primarily involved in controlling carbon metabolism, especially catabolic repression [26], though a *ccpA* mutant in *S. mutans* was found to have 60% impairment in biofilm formation [27]. SGO_0237 shows increased levels in SgPg compared to Sg but SGO_0773 shows decreased levels in all mixed communities (Table 9). Given the reduction seen in PTS sugar transport and the formation of communities, a CcpA protein would be expected to be increased across all of the communities. It is unlikely that both SGO_0237 and SGO_0773 are functioning as classical CcpA regulatory proteins. The increased SGO_0237 may be the actual catabolite control protein A for Sg. However, the PTS transport systems do not seem to be responding to a traditional catabolic repression and the binding proteins that play an important role in biofilm formation are down as well. As with the binding proteins, CcpA may play an early role in biofilm formation and be reduced at 18 hours when the samples were collected. It is also possible that despite the homology neither protein acts like CcpA in Sg.

SGO_1816 encodes for ScaR, a manganese dependent regulator of a high affinity ABC manganese transporter, SGO_1800-1802 [28]. However, the name Sca actually refers to streptococcal coaggregation adherence because one of the regulated transporter proteins, ScaA, SGO_1801, was originally identified as an adhesin important for aggregation with *A. neaslundii*[29]. ScaA was not detected in any of the samples, though that is not unusual for a membrane protein, but ScaR showed increased levels in SgFn while the other members of the operon with ScaA, SGO_1800 and SGO_1802, showed reduced levels in all the mixed communities. It seems unlikely that Sg is seeing higher levels of manganese in the mixed communities to account for down-regulation of the ABC transporter. However, there are some indications that, like the PTS sugar transporters, Sg has a second manganese transport system driven by the proton motive force [28]. This would once again be consistent with a low pH environment. Also, we see a significant reduction in other adhesin proteins and the Sca operon may be down-regulated to reduce the adhesin ScaA.

SGO_1072 and SGO_1073 have homology to the sensor and kinase proteins of the two-component signaling-transducing system CiaR-CiaH from *S. pneumoniae*[30]. In *S. pneumoniae* Cia has been shown to regulate a number of genes involved in the biochemical make up of the cell wall, including activation of the genes for D-alanylation of lipoteichoic acid, *dlt*. Detection of the regulatory protein CiaR, SGO_1072, was poor and statistical significance could only be calculated for the SgFn vs Sg and SgPg vs SgFn comparisons. CiaR showed a significant increase in SgFn vs Sg and a decrease in SgPg vs SgFn implying a large increase in the presence of Fn. The sensor kinase, SGO_1073, remained statistically unchanged. Despite the high levels of CiaR in SgFn the Dtl proteins did not show any coherent change. CiaR may control a different set of genes in Sg than *S. pneumoniae*. Increases in the amounts of the regulator protein also do not necessarily cause regulatory effects. However, given the changes to cell wall biosynthesis proteins it is interesting that a cell wall biosynthetic regulator showed increased levels in the presence of Fn.

### Translation, ribosomal proteins, and tRNA synthetases

In a previous report on *P. gingivalis* results from these same experiments we noted that Pg had significant increases in translational machinery and ribosomal protein levels in a community with Sg and Fn [11]. Table 10 shows a summary of the translational machinery proteins, ribosomal and accessory proteins, and tRNA synthetases for Sg. The translational proteins showed some increase in the mixed communities with increases in approximately half of the detected proteins. SgFn vs Sg showed one reduced protein. The ribosomal proteins showed a general increase compared to Sg in the SgPg and SgPgFn communities, again approximately half of the detected proteins, with a small number showing a decrease. In contrast, ribosomal proteins in SgFn were mostly unchanged and most of the changed proteins showed decreased levels compared to Sg. Similar results were seen with tRNA synthetases where SgPg and SgPgFn showed a significant number of increased proteins and few or no decreased proteins. SgFn showed few changes of tRNA synthetase protein levels. Taken together the data imply that translation is increased in Sg, similar to what was seen with Pg when exposed to SgFn, but only in communities with Pg or PgFn and not with Fn alone. Hence Fn-Sg interactions may be less synergistic than occur in the three species community.

**Table 10:** Translation, ribosomal, and tRNA synthetase proteins

		**SgFn vs Sg**	**SgPg vs Sg**	**SgPgFn vs Sg**	**SgPg vs SgFn**	**SgPgFn vs SgFn**	**SgPgFn vs SgPg**
Translation^a^	Total	10	10	9	10	9	9
	Unchanged	5	5	5	5	5	9
	Increased	4	5	4	3	2	0
	Decreased	1	0	0	2	2	0
Ribosomal Proteins^b^	Total	58	57	53	57	53	52
	Unchanged	43	26	21	27	25	44
	Increased	5	28	30	28	28	5
	Decreased	10	2	2	2	0	3
tRNA Synthetases^c^	Total	22	22	21	22	21	21
	Unchanged	18	9	9	11	13	17
	Increased	2	13	9	8	6	0
	Decreased	2	0	3	3	2	4

^a^ covers SGO_0206, 0321, 0546, 0761, 1090, 1154, 1441, 1617, 1863, 2000.

^b^ covers SGO_0027, 0183, 0204, 0205, 0333, 0355, 0358, 0359, 0523, 0573, 0610, 0719, 0818, 0820, 0848, 1033, 1034, 1191, 1192, 1234, 1276, 1316, 1323, 1364, 1383, 1451, 1455, 1456, 1669, 1824, 1879, 1881, 1958, 1960, 1961, 1966, 1967, 1968, 1969, 1970, 1971, 1973, 1974, 1975, 1976, 1977, 1978, 1979, 1980, 1981, 1982, 1983, 1984, 1985, 1986, 2001, 2066, 2088.

^c^ covers SGO_0007, 0174, 0349, 0407, 0434, 0568, 0569, 0639, 0681, 0753, 0778, 0859, 0861, 1293, 1570, 1683, 1784, 1851, 1929, 2058, 2060, 2062.

### Stress proteins

A syntropic community might be expected to be less stressful to the organisms involved due to support from other species. One result of stressful conditions is DNA damage. Table 11 shows a summary of the DNA repair proteins. Most remain unchanged but a number of DNA repair proteins show reduced levels in the mixed communities, and in the SgPg and SgPgFn communities compared to SgFn. There is one striking exception however, recombinase A. RecA, SGO_ 2045, is significantly down in SgFn but up in SgPg and SgPgFn compared to Sg alone (Table 12). RecA is important for both DNA recombination and DNA repair. An increase in RecA but a decrease in other DNA repair proteins might indicate increased homologous recombination rather than DNA repair. However, the proteins associated with bacterial competence that we detected showed many significant reductions in all mixed pellets (Table 12).

**Table 11:** Stress proteins

		**SgFn vs Sg**	**SgPg vs Sg**	**SgPgFn vs Sg**	**SgPg vs SgFn**	**SgPgFn vs SgFn**	**SgPgFn vs SgPg**
DNA Repair ^a^	Total	21	17	12	17	12	11
	Unchanged	13	12	6	11	8	9
	Increased	2	2	1	1	1	0
	Decreased	6	3	5	5	3	2
Oxidative Stress ^b^	Total	7	6	6	6	6	6
	Unchanged	1	1	3	2	3	6
	Increased	6	5	3	2	1	0
	Decreased	0	0	0	2	2	0
Other Stress Proteins ^c^	Total	18	17	15	17	15	14
	Unchanged	9	8	5	8	8	10
	Increased	7	6	4	2	0	0
	Decreased	2	3	6	7	7	4

^a^ Covers SGO_0105, 0171, 0260, 0286, 0626, 0685, 0698, 0830, 1000, 1038, 1044, 1250, 1390, 1413, 1414, 1531, 1865, 2045, 2050, 2053, 2056.

^b^ Covers SGO_0263, 0278, 0749, 1599, 1685, 1803, 1990.

^c^ Covers SGO_0368, 0401, 0402, 0404, 0495. 0688, 0722, 1140, 1625, 1632, 1736, 1862, 1885, 1886, 1991, 1998, 2150.

**Table 12:** RecA and competence proteins

**Protein**	**SgFn vs Sg**	**SgPg vs Sg**	**SgPgFn vs Sg**	**SgPg vs SgFn**	**SgPgFn vs SgFn**	**SgPgFn vs SgPg**
SGO_0200	**−1.4**	**−1.2**	**−1.8**	**0.3**	−0.3	−0.6
SGO_0981	−1.1	−0.8	nd	0.3	Nd	nd
SGO_1924	nd	**−2.0**	**−2.5**	nd	Nd	−0.5
SGO_2045	**−2.3**	**0.8**	**0.9**	**3.2**	**3.2**	0.1
SGO_2097	nd	**−5.5**	**−6.6**	nd	Nd	−1.1
SGO_2145	nd	−0.3	**−0.3**	nd	Nd	0.0
SGO_2146	nd	**−1.7**	−2.7	nd	Nd	−1.0

Bold: statistically significant difference, all ratios are log_2_.

nd: not detected in one or more of the compared samples.

Sg also has a number of proteins to deal with oxidative stress. Most of these proteins showed increased levels in the mixed communities compared to Sg alone (Table 11). This may indicate an increased exposure to oxidative stress. However, while Sg can grow aerobically and anaerobically, other oral microbes like Pg are strict anaerobes. The increased protein levels may serve the purpose of providing oxygen protection for anaerobic community members.

Other stress response proteins include chaperones such as GroES, SGO_1886, and proteases such as Clp protease P (ClpP), SGO_1632, that degrades misfolded proteins. Table 11 summarizes the changes in other stress proteins. Both increased and decreased protein levels were seen in all of the multispecies samples compared to the Sg control, though there was a general trend towards lower levels in SgPg and even lower levels in SgPgFn compared to SgFn.

## Conclusions

Both dental caries and periodontal disease are community diseases that ensue from the action of complex multispecies biofilms. Both synergistic and competitive interactions occur among the biofilm constituent species, and biofilm development is a complex interaction involving attachment, recruitment, maturation and detachment. In this study we used quantitative whole cell proteomics to compare proteomes in a simplified model of dental plaque, from a mono-culture of the early colonizer *S. gordonii*, to a mixed community of *S. gordonii* with the intermediate colonizer *F. nucleatum*, to a three-species model nascent community of *S. gordonii*, *F. nucleatum*, and the late colonizing periodontal pathogen *P. gingivalis*.

*S. gordonii* displayed extensive changes in communities with *F. nucleatum* and *P. gingivalis*, especially related to pathways for metabolite utilization and production. The observed changes were species specific depending on the interaction partner. The *P. gingivalis* interaction appeared to be dominant as protein levels in *S. gordonii* paired with *P. gingivalis* and *F. nucleatum* were very similar to those observed with *P. gingivalis* only. All of the mixed species samples showed evidence of increased energy metabolism and decreased PTS sugar transport compared to *S. gordonii* alone, consistent with high metabolite availability in mixed communities in vivo. There was also a shift in end product pathways for energy metabolism, altering the products available from *S. gordonii* to the community away from ethanol and towards L-lactate. Such a shift would be consistent with the production of a more acidic environment in vivo.

While contact with both *F. nucleatum* and *P. gingivalis* resulted in extensive changes to the proteome of *S. gordonii*, the dominant *P. gingivalis* interaction was consistent with models whereby *P. gingivalis* can influence the virulence properties of the microbial community as a whole [31,32]. The mixed communities showed significant quantitative changes in 45 to 54% of the detected proteome compared to the *S. gordonii* single organism control. The *F. nucleatum* or *P. gingivalis* interactions appeared to be quite distinct, with approximately 48% of the detected proteome differing between the two two-species communities. However, only a small quantitative relative abundance difference, 11% of the detected proteome, occurred between pellets containing *P. gingivalis* and pellets with *P. gingivalis* and *F. nucleatum*, implying that in the present experimental model the contribution of *P. gingivalis* to a nascent heterotypic community supersedes that of other gram-negative anaerobes, such as *F. nucleatum*.

## Methods

### Bacteria and culture conditions

*Fusobacterium nucleatum* subsp. *nucleatum* ATCC 25586 and *Porphyromonas gingivalis* ATCC 33277 were grown anaerobically (85% N_2_, 10% H_2_, 5% CO_2_) at 37°C in trypticase soy broth supplemented with 1 mg/ml yeast extract, 1 μg/ml menadione and 5 μg/ml hemin (TSB)_._*S. gordonii* DL1 was grown anaerobically at 37°C in Todd-Hewitt broth (THB).

### Chemicals

HPLC grade acetonitrile was from Burdick & Jackson (Muskegon, MI, USA); high purity acetic acid (99.99%) and ammonium acetate (99.99%), from Aldrich (Milwaukee, WI, USA). High purity water was generated with a NANOpure UV system (Barnstead, Dubuque, IA, USA).

### Proteomics of model bacterial communities

Harvesting and pelleting of bacteria, proteomic analysis, mass spectrometry and statistical methods were handled as described in Kuboniwa *et al.*[11]. In brief, bacteria were cultured to mid-log phase, harvested by centrifugation and resuspended in pre-reduced PBS (rPBS). 1 x 10^9^ cells of *S. gordonii* were mixed with an equal number of *P. gingivalis*, *F. nucleatum*, or both as combinations of the species. *S. gordonii* cells alone were also used as a control. Two independent biological replicates from separate experiments comprised of at least two technical replicates were analyzed. Bacteria were centrifuged at 3000 g for 5 min, and pelleted mixtures of bacteria were held in 1 ml pre-reduced PBS in an anaerobic chamber at 37°C for 18 h [10]. Bacterial cells were lysed in resuspension buffer (15 mM Tris HCl pH 9.5, 0.02% Rapigest^tm^ Waters, Milford, MA) in a boiling water bath followed by sonication and bead beating and proteins were digested with trypsin then fractionated into five pre-fractions [33]. The 2D capillary HPLC/MS/MS analyses were conducted on a Thermo LTQ mass spectrometer (Thermo Fisher Corp. San Jose, CA, USA). Peptides were eluted with a seven step salt gradient (0, 10, 25, 50, 100, 250 and 500 mM ammonium acetate) followed by an acetonitrile gradient elution (Solvent A: 99.5% water, 0.5% acetic acid. Solvent B: 99.5% acetonitrile, 0.5% acetic acid). The MS^1^ scan range for all samples was 400–2000 *m/z*. Each MS^1^ scan was followed by 10 MS^2^ scans in a data dependent manner for the 10 most intense ions in the MS^1^ scan. Default parameters under Xcalibur 1.4 data acquisition software (Thermo Fisher) were used, with the exception of an isolation width of 3.0 *m/z* units and normalized collision energy of 40%.

### Data processing and protein identification

Data processing was handled as described in Kuboniwa *et al.*[11]. In brief, raw data were searched by SEQUEST [34] against a FASTA protein ORF database consisting of the *P. gingivalis* W83 (2006, TIGR-CMR [35]) [GenBank: AE015924], *S. gordonii* Challis NCTC7868 (2007, TIGR-CMR [36]) [GenBank: CP00725.1], *F. nucleatum* ATCC 25586 (2002, TIGR-CMR [37]) [GenBank: AE009951.1], bovine (2005, UC Santa Cruz), nrdb human subset (NCBI, as provided with Thermo Bioworks ver. 3.3) and the MGC (Mammalian Gene collection, 2004 curation, NIH-NCI [38]) concatenated with the reversed sequences. The reversed sequences were used for purposes of calculating a qualitative FDR using the published method [39,40]. The SEQUEST peptide level search results were filtered and grouped by protein using DTASelect [41], then input into a FileMaker script developed in-house [42,43] for further processing, including peak list generation. Only peptides that were unique to a given ORF were used in the calculations, ignoring tryptic fragments that were common to more than one ORF or more than one organism, or both. The qualitative peptide level FDR was controlled to approximately 5% for all conditions by selecting a minimum non-redundant spectral count (unique peptide) cut-off number appropriate to the complexity of each condition. Using our methods, this implies a protein level qualitative FDR in the range of approximately 0.01 to 2%, depending on the specific experiment. A minimum of three unique peptides were used for any qualitative protein identification. Substitution of a database based on *P. gingivalis* 33277 [GenBank: AP009380] rather than W83 had no substantive effect on the calculations [44], so the original W83 entries were retained in the database for purposes of the work described here.

### Protein abundance ratio calculations

Protein relative abundances were estimated on the basis of spectral count values for proteins meeting the requirements for qualitative identification described above [42,43]. For spectral counts, the redundant numbers of peptides uniquely associated with each ORF were taken from the DTAselect filter table (t = 0). Spectral counting is a frequency measurement that has been demonstrated in the literature to correlate with protein abundance [45]. To calculate protein abundance ratios, a normalization scheme was applied such that the total spectral counts for all *S. gordonii* proteins in each condition were set equal for each comparison. The normalized data for each abundance ratio comparison was tested for significance using a global paired *t*-test for each condition, the details of which have been published for this type of proteomics data in which all biological replicates are compared against each other [33,46], see also the explanatory notes in Kuboniwa *et al*. [11]. The testing procedure weighs deviation from the null hypothesis of zero abundance change and random scatter in the data to derive a probability or *p*-value that the observed change is a random event, i.e. that the null hypothesis of no abundance change is true. Each hypothesis test generated a *p*-value that in turn was used to generate a *q*-value as described [42,47], using the R package QVALUE [48]. The *q*-value in this context is a measure of quantitative FDR [49] that contains a correction for multiple hypothesis testing. A *q* cut-off value of 0.005 was used for all ratios reported in the relative abundance tables shown in Additional files 1, 2, 3, 4, 5, 6, 7. All statistical calculations were done using R (Ver. 2.5.0). Only proteins with data consisting of confirmed high scoring MS^2^ mass spectra (high scoring qualitative database matches as described above) present in both the numerator and denominator of the abundance ratio comparison were listed as significantly changed in the relative abundance data tables (see Additional files 1, 2, 3, 4, 5, 6, 7).

### Ontology analysis

An overall list of detected proteins, as well as lists of proteins that showed increased or decreased levels in the community comparisons, were prepared using Entrez gene identifiers. Ontology analyses were then conducted using the DAVID [50] functional annotation clustering feature with the default databases. Both increased and decreased protein level lists were analyzed using the overall list of detected proteins as the background. Potentially interesting clusters identified by DAVID were then examined manually.

### Confocal microscopy

*S. gordonii* stained with hexidium iodide 15 μg ml^-1^, (Molecular Probes, Carlsbad, CA), *F. nucleatum* stained 5- (and 6-) carboxyfluorescein (4 μg ml^-1^, Molecular Probes) and *P. gingivalis* (2 x 10^8^ cells of each species) were added together, centrifuged and incubated under anaerobic conditions for 18 h before removal of the supernatant and gentle re-suspension of the cells. The cell suspension (0.5 ml) was added to a glass coverslip before fixing with 4% paraformaldehyde. Detection of *P. gingivalis* was achieved using a specific anti-whole cell *P. gingivalis* antibody and anti-rabbit alexa 547 (Molecular Probes) conjugated secondary. Coverslips were imaged using an Olympus FV500 laser scanning confocal microscope. A series of XYZ image stacks were digitally reconstructed using Volocity image analysis program (Improvision, Waltham, MA).

## Abbreviations

ATCC: American type culture collection; DAVID: Database for annotation, visualization and integrated discovery; FDR: False discovery rate; Fn: *Fusobacterium nucleatum* ; LANL: Los Alamos National Laboratory; MS: Mass spectrometry; ORF: Open reading frame; Pg: *Porphyromonas gingivalis* ; PTS: Phosphoenolpyruvate dependent phosphotransferase system; Sg: *Streptococcus gordonii* ; TIGR-CMR: The Institute for Genomic Research Comprehensive Microbial Resource, now part of the J. Craig Venter Institute.

## Competing interests

The authors declare that they have no competing interests.

## Authors’ contributions

ELH calculated the protein abundance ratios, abundance change statistics, and performed the pathway and ontology analyses. TW performed the mass spectrometry measurements. BCD and SEW performed in vitro experiments. CJW performed the confocal microscopy. MH and RJL conceived the experiments. ELH, MH and RJL wrote the manuscript. All authors read and approved the manuscript.

## Supplementary Material

Additional file 1 **Summary.** This file contains a short summary of all the relative abundance ratios mentioned in this report. Prior to permanent archiving at JGI (http://www.jgi.doe.gov/) and LANL (http://semiglobe.lanl.gov/) with the mass spectral data in XML compatible format, summaries of the protein identifications in the form of tab-delimited text files will be available on a University of Washington server (http://depts.washington.edu/mhlab/), rather than on the BMC Microbiology web site due to their large size. Request a password from the corresponding author. These files include details such as SEQUEST scores, peptide sequence, percentage of peptide coverage by observed ions in the CID spectrum, spectral counts, and other information at the individual peptide and protein level as calculated using DTASelect [41]. Spectral counts and coverage information for each protein can also be found in the files listed below. Ratios for protein comparisons with statistically increased levels are shown in red highlight, ratios for statistically decreased levels are shown in green highlight. The pale red and green highlights indicate the *q*-values for statistically increased or decreased levels respectively. Click here for file

Additional file 2 **SgFn_vs_Sg.** A more detailed presentation of the relative abundance ratios for the comparison of SgFn and the Sg controls, including both raw and normalized spectral counts. Red and green highlights are used as in Additional file 1. Click here for file

Additional file 3 **SgPg_vs_Sg.** A more detailed presentation of the relative abundance ratios for the comparison of SgPg and the Sg controls, including both raw and normalized spectral counts. Red and green highlights are used as in Additional file 1. Click here for file

Additional file 4 **SgPgFn_vs_Sg.** A more detailed presentation of the relative abundance ratios for the comparison of SgPgFn and the Sg controls, including both raw and normalized spectral counts. Red and green highlights are used as in Additional file 1. Click here for file

Additional file 5 **SgPg_vs_SgFn.** A more detailed presentation of the relative abundance ratios for the comparison of SgPg and SgFn, including both raw and normalized spectral counts. Red and green highlights are used as in Additional file 1. Click here for file

Additional file 6 **SgPgFn_vs_SgFn.** A more detailed presentation of the relative abundance ratios for the comparison of SgPgFn and SgFn, including both raw and normalized spectral counts. Red and green highlights are used as in Additional file 1. Click here for file

Additional file 7 **SgPgFn_vs_SgPg.** A more detailed presentation of the relative abundance ratios for the comparison of SgPgFn and SgPg, including both raw and normalized spectral counts. Red and green highlights are used as in Additional file 1. Click here for file

Additional file 8 **Coverage.** Coverage statistics for individual proteins based on recovered tryptic fragments and the inferred sequences from the annotated genome for *S. gordonii*[36]. Gray shading indicates the percentage of the protein covered by the detected peptides. Black shading indicates the undetected percentage. Click here for file

Additional file 9 **Geneplot_SgPgFn_vs_Sg.** A genomic plot of all data collected for *S. gordonii* protein relative abundance calculations used in the comparison of SgPgFn and the Sg controls. The color code for each SGO number [36] follows that used in the data tables (see Additional files 1, 2, 3, 4, 5, 6, 7), where data was acquired. ORFs coded black were either not used in the annotation or no tryptic fragments were observed. Grey indicates qualitative detection only. Click here for file

Additional file 10 **Regressplots.pdf.** XY regression plots demonstrating the reproducibility of the spectral counting mass spectrometry data for the technical and biological replicates, with an explanatory note. Click here for file
