# Supplementary material for: Proteomics of Streptococcus gordonii within a model developing oral microbial community
Source: BMC Microbiol. 2012 Sep 18;12:211. doi: 10.1186/1471-2180-12-211 (PMC3534352; doi:10.1186/1471-2180-12-211)
Supplement: Additional file 1 — Summary. This file contains a short summary of all the relative abundance ratios mentioned in this report. Prior to permanent archiving at JGI (http://www.jgi.doe.gov/) and LANL (http://semiglobe.lanl.gov/) with the mass spectral data in XML compatible format, summaries of the protein identifications in the form of tab-delimited text files will be available on a University of Washington server (http://depts.washington.edu/mhlab/), rather than on the BMC Microbiology web site due to their large size. Request a password from the corresponding author. These files include details such as SEQUEST scores, peptide sequence, percentage of peptide coverage by observed ions in the CID spectrum, spectral counts, and other information at the individual peptide and protein level as calculated using DTASelect [41]. Spectral counts and coverage information for each protein can also be found in the files listed below. Ratios for protein comparisons with statistically increased levels are shown in red highlight, ratios for statistically decreased levels are shown in green highlight. The pale red and green highlights indicate the q-values for statistically increased or decreased levels respectively. [file 1471-2180-12-211-S1.pdf]

Spectral Counts
Sg Summary Table

Streptococcus gordonii

Hackett Laboratory UW

Summary Table | SgFn vs Sg | SgPg vs Sg | SgPgFn vs Sg | SgPg v SgFn | SgPgFn v SgFn | SgPgFn vs SgPg | Coverage

	SgFn vs Sg				*SgPg vs Sg*				*SgPgFn vs Sg*				*SgPg vs SgFn*				*SgPgFn vs SgFn*				*SgPgFn vs SgPg*			
Protein	Ratio	Sum	*q*-Val	*p*-Val	Ratio	Sum	*q*-Val	*p*-Val	Ratio	Sum	*q*-Val	*p*-Val	Ratio	Sum	*q*-Val	*p*-Val	Ratio	Sum	*q*-Val	*p*-Val	Ratio	Sum	*q*-Val	*p*-Val
**SGO_0001**	-0.902	7.046	0.0101	0.0398	-0.754	7.101	0.0159	0.0586	-1.455	6.882	0.0038	0.0189	0.148	6.069	0.0468	0.3152	-0.553	5.134	0.0078	0.0292	-0.702	5.838	0.0183	0.0240
	dnaA; chromosomal replication initiator protein DnaA																							
**SGO_0002**	0.640	7.258	0.0146	0.0633	0.550	7.204	0.0242	0.0969	-0.140	6.846	0.0591	0.5301	-0.090	6.961	0.0299	0.1843	-0.780	6.060	0.0016	0.0035	-0.691	6.614	0.0067	0.0043
	dnaN; DNA polymerase III, beta subunit																							
**SGO_0004**	-1.661	8.780	0.0020	0.0050	-1.979	8.713	0.0019	0.0040	-2.017	8.695	0.0009	0.0028	-0.318	7.080	0.0514	0.3501	-0.356	6.431	0.0274	0.1457	-0.038	6.879	0.2009	0.7232
	putative lipoprotein																							
**SGO_0005**		4.640				4.640				4.640														
	hypothetical protein SGO_0005																							
**SGO_0006**	0.158	7.655	0.0485	0.2750	0.462	7.817	0.0017	0.0033	0.100	7.626	0.0516	0.4541	0.304	7.378	0.0161	0.0882	-0.058	6.587	0.1084	0.7696	-0.362	7.354	0.0348	0.0653
	ABC transporter, ATP-binding protein																							
**SGO_0007**	-0.294	6.164	0.0417	0.2311	1.153	6.977	0.0003	0.0002	0.785	6.734	0.0005	0.0012	1.447	6.385	0.0006	0.0008	1.079	5.516	0.0015	0.0031	-0.368	6.754	0.0012	0.0001
	trpS; tryptophanyl-tRNA synthetase																							
**SGO_0008**	0.310	9.371	0.0149	0.0651	1.064	9.833	0.0005	0.0007	0.961	9.763	0.0004	0.0008	0.755	9.426	0.0009	0.0014	0.651	8.752	0.0013	0.0020	-0.103	9.703	0.0397	0.0811
	inosine-5'-monophosphate dehydrogenase																							
**SGO_0011**	-0.863	5.257	0.0270	0.1369	-1.064	5.482	0.0036	0.0090	-0.825	5.581	0.0082	0.0486	-0.201	4.002	0.0922	0.6812	0.038	3.580	0.1191	0.8484	0.238	4.509	0.1464	0.4687
	proteinase, M16 family																							
**SGO_0012**	1.455	4.510	0.0205	0.0948		1.807				1.807				3.756				3.145						
	mpp; peptidase, M16 family																							
**SGO_0013**		4.813			-0.643	5.212	0.0434	0.1881		4.813				2.648								2.648		
	hypothetical protein SGO_0013																							
**SGO_0015**	-2.771	6.426	0.0058	0.0196	-2.256	6.598	0.0001	0.0000	-2.738	6.428	0.0039	0.0197	0.514	3.987	0.0097	0.0458	0.033	2.445			-0.482	3.996	0.0283	0.0484
	ABC transporter (ATP-binding protein)																							
**SGO_0016**		3.787			-0.241	4.669	0.0423	0.1825		3.787				3.028								3.028		
	cbiO; ABC transporter, ATP-binding protein																							
**SGO_0017**		2.351				2.351				2.351														
	ABC-type putative cobalt transport system, permease																							
**SGO_0019**		2.807				2.807				2.807														
	sdhA; L-serine dehydratase, iron-sulfur-dependent, alpha subunit																							
**SGO_0020**	-0.115	4.222	0.1386	0.9320		2.585				2.585				3.149				2.538						
	sdhB; L-serine dehydratase, iron-sulfur-dependent, beta subunit																							
**SGO_0022**	3.662	7.860	0.0001	0.0000	1.241	5.854	0.0025	0.0057	0.104	5.176	0.0906	0.8591	-2.421	7.479	0.0000	0.0000	-3.558	6.740	0.0001	0.0000	-1.137	5.357	0.0037	0.0010
	trmU; tRNA (5-methylaminomethyl-2-thiouridylate)-methyltransferase																							

Log$_2$ Ratios

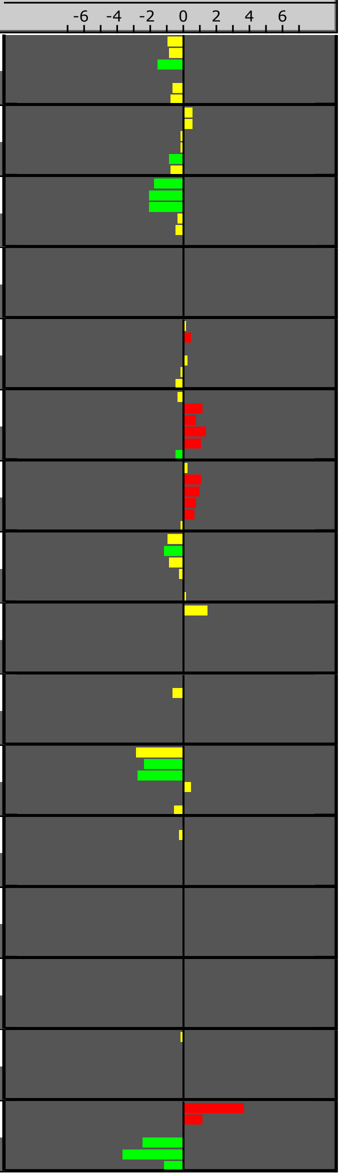

◉ Show detected proteins only
○ Show all proteins
☐ Filter by category:
ABC Transporter

Proteins found:
1179

Dot Plots
Dot Plots

Test	Cutoff
q-Value	.005
p-Value	

	Signif	Direction	Applies To
(red)	yes	+	ratios, bars
(yellow)	no	n/a	bars
(green)	yes	–	ratios, bars
(light red)	yes	+	*p*- , *q*-Values
(light green)	yes	–	

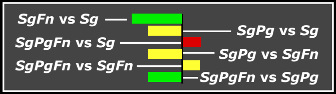

Hendrickson *et al.*

Streptococcus gordonii

Summary Table | SgFn vs Sg | SgPg vs Sg | SgPgFn vs Sg | SgPg v SgFn | SgPgFn v SgFn | SgPgFn vs SgPg | Coverage

Protein	Description	*SgFn vs Sg* Ratio	Sum	*q*-Val	*p*-Val	*SgPg vs Sg* Ratio	Sum	*q*-Val	*p*-Val	*SgPgFn vs Sg* Ratio	Sum	*q*-Val	*p*-Val	*SgPg vs SgFn* Ratio	Sum	*q*-Val	*p*-Val	*SgPgFn vs SgFn* Ratio	Sum	*q*-Val	*p*-Val	*SgPgFn vs SgPg* Ratio	Sum	*q*-Val	*p*-Val
SGO_0025	gidA; glucose inhibited division protein A	-0.570	5.781	0.0268	0.1355	0.338	6.207	0.0477	0.2100	0.343	6.230	0.0332	0.2749	0.908	5.502	0.0157	0.0846	0.913	4.918	0.0230	0.1173	0.005	5.892	0.2382	0.9134
SGO_0026	DHH subfamily 1 protein		7.420			-3.976	7.514	0.0005	0.0007	-4.015	7.463	0.0109	0.0718		3.020				1.272			-0.039	3.561	0.2247	0.8413
SGO_0027	rplI; ribosomal protein L9	-0.380	6.937	0.0100	0.0393	0.376	7.312	0.0046	0.0126	0.520	7.410	0.0109	0.0722	0.756	6.646	0.0011	0.0021	0.900	6.144	0.0071	0.0259	0.144	7.060	0.1271	0.3931
SGO_0028	dnaC; replicative DNA helicase	-1.442	5.255	0.0034	0.0101	-0.451	5.614	0.0507	0.2268	-0.610	5.208	0.0357	0.2993	0.991	4.452	0.0119	0.0590	0.832	3.141	0.0235	0.1219	-0.159	4.394	0.1982	0.7084
SGO_0030	aspB; aspartate transaminase	2.151	7.906	0.0001	0.0000	0.967	7.027	0.0008	0.0011	0.689	6.851	0.0018	0.0069	-1.184	7.625	0.0001	0.0000	-1.462	6.935	0.0001	0.0000	-0.278	6.784	0.0184	0.0244
SGO_0032	plsX; fatty acid/phospholipid synthesis protein PlsX	-0.944	7.088	0.0028	0.0077	0.024	7.492	0.1697	0.8926	-0.245	7.365	0.0180	0.1364	0.968	6.584	0.0006	0.0007	0.699	5.805	0.0031	0.0086	-0.269	6.860	0.0188	0.0254
SGO_0033	acpP; acyl carrier protein		3.725			0.994	5.306	0.0039	0.0102	1.403	5.651	0.0085	0.0517		4.205				4.086			0.409	5.472	0.0935	0.2631
SGO_0035	phosphoribosylformylglycinamidine synthase	2.367	5.314	0.0174	0.0779	-0.709	2.496				1.807			-3.076	4.751	0.0137	0.0713		4.058				0.585		
SGO_0042	transcription regulator, GntR family	0.918	6.144	0.0003	0.0002	0.355	5.804	0.0083	0.0260	0.243	5.283	0.0113	0.0752	-0.563	5.766	0.0019	0.0045	-0.675	4.801	0.0136	0.0616	-0.112	5.007	0.1627	0.5485
SGO_0044	PTS system, IIB component																								
SGO_0050	hypothetical protein SGO_0050		4.640				4.640				4.640														
SGO_0051	hypothetical protein SGO_0051		3.351				3.351				3.351														
SGO_0054	dltA; D-alanine-activating enzyme	1.565	8.469	0.0001	0.0001	0.238	7.609	0.0136	0.0480	-0.677	7.186	0.0010	0.0031	-1.327	8.019	0.0002	0.0001	-2.242	7.201	0.0001	0.0000	-0.915	6.823	0.0031	0.0007
SGO_0055	dltB; integral membrane protein		2.058				2.058				2.058														
SGO_0056	dltC; D-alanyl carrier protein	-0.711	3.805	0.0112	0.0452		3.420				3.420				1.196				0.585						
SGO_0057	dltD protein	-1.818	8.223	0.0010	0.0018	-2.098	8.163	0.0008	0.0012	-2.415	8.108	0.0005	0.0010	-0.280	6.397	0.0215	0.1239	-0.596	5.653	0.0078	0.0291	-0.316	6.092	0.0050	0.0020

Log$_2$ Ratios

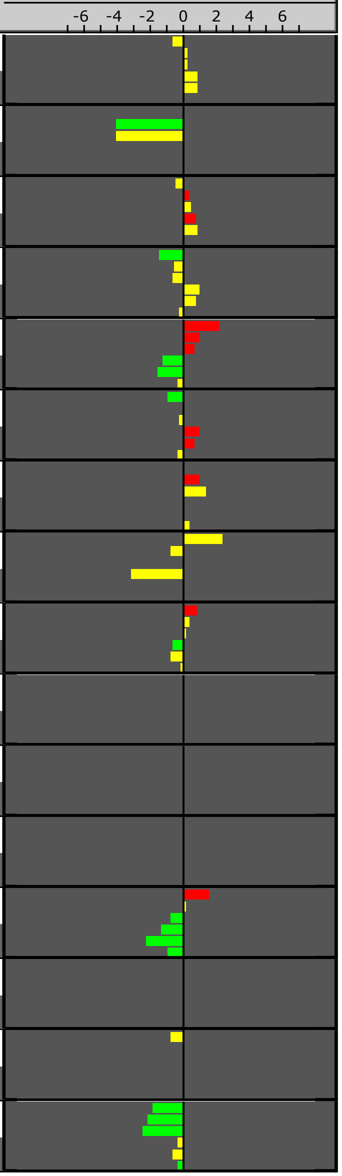

Show detected proteins only
Show all proteins
Filter by category:
ABC Transporter

Proteins found:
1179

Dot Plots
Dot Plots

Test	Cutoff
q-Value	.005
p-Value	

	Signif	Direction	Applies To
(red)	yes	+	ratios, bars
(yellow)	no	n/a	bars
(green)	yes	−	ratios, bars
(pink)	yes	+	*p*- , *q*-Values
(light green)	yes	−	

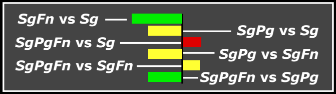

Hendrickson *et al.*

Streptococcus gordonii

Summary Table | SgFn vs Sg | SgPg vs Sg | SgPgFn vs Sg | SgPg v SgFn | SgPgFn v SgFn | SgPgFn vs SgPg | Coverage

Protein	Description	*SgFn vs Sg* Ratio	Sum	*q*-Val	*p*-Val	*SgPg vs Sg* Ratio	Sum	*q*-Val	*p*-Val	*SgPgFn vs Sg* Ratio	Sum	*q*-Val	*p*-Val	*SgPg vs SgFn* Ratio	Sum	*q*-Val	*p*-Val	*SgPgFn vs SgFn* Ratio	Sum	*q*-Val	*p*-Val	*SgPgFn vs SgPg* Ratio	Sum	*q*-Val	*p*-Val
**SGO_0059**	pXO1; hypothetical protein SGO_0059	-2.492	11.167	0.0067	0.0235	1.097	12.538	0.0073	0.0222	1.992	13.189	0.0006	0.0015	3.589	11.564	0.0007	0.0009	4.484	11.789	0.0004	0.0002	0.895	12.958	0.0058	0.0034
**SGO_0060**	hypothetical protein SGO_0060	-4.809	8.069	0.0112	0.0455		8.043				8.043				1.717				1.107						
**SGO_0062**	hypothetical protein SGO_0062		1.379				1.380				1.380														
**SGO_0063**	hypothetical protein SGO_0063	-1.969	7.830	0.0001	0.0000	-1.765	7.875	0.0002	0.0001	-1.618	7.907	0.0000	0.0000	0.204	6.135	0.0400	0.2615	0.350	5.597	0.0071	0.0258	0.146	6.304	0.1104	0.3266
**SGO_0064**	FtsK/SpoIIIE family protein	-2.780	8.948	0.0001	0.0000	-1.647	9.154	0.0003	0.0002	-2.484	8.988	0.0001	0.0000	1.133	7.144	0.0027	0.0079	0.296	6.006	0.0101	0.0407	-0.837	7.241	0.0120	0.0121
**SGO_0065**	hypothetical protein SGO_0065	0.041	7.328	0.0927	0.5936	0.339	7.484	0.0009	0.0013	0.159	7.386	0.0007	0.0020	0.298	6.991	0.0071	0.0294	0.118	6.285	0.0397	0.2305	-0.179	7.043	0.0094	0.0077
**SGO_0066**	D-3-phosphoglycerate dehydrogenase, putative		2.446				4.494				4.900			1.034	4.293	0.0278	0.1684	1.422	4.018	0.0352	0.2001	0.388	5.198	0.0661	0.1647
**SGO_0067**	protein with prophage function domain	-1.326	6.056	0.0003	0.0004	0.784	7.024	0.0059	0.0173	-0.170	6.487	0.0082	0.0489	2.110	6.156	0.0015	0.0035	1.156	4.815	0.0012	0.0019	-0.954	6.452	0.0122	0.0126
**SGO_0068**	lipoprotein, putative	-1.848	6.075	0.0116	0.0478	-0.510	6.655	0.0016	0.0031	-0.815	6.537	0.0004	0.0009	1.338	5.123	0.0075	0.0324	1.033	4.262	0.0063	0.0223	-0.305	5.719	0.0051	0.0023
**SGO_0069**	hypothetical protein SGO_0069		5.402			-0.728	6.084	0.0007	0.0009	-1.033	5.980	0.0006	0.0015		4.162				3.258			-0.305	5.023	0.0396	0.0796
**SGO_0070**	merozoite surface protein 1		5.452			0.264	6.588	0.0177	0.0667	0.351	6.637	0.0080	0.0470		5.199				4.678			0.087	6.244	0.1261	0.3884
**SGO_0075**	hypothetical protein SGO_0075		1.379				1.380				1.380														
**SGO_0078**	hypothetical protein SGO_0078	-2.233	5.972	0.0356	0.1899	-1.267	6.328	0.0090	0.0292		5.830			0.966	4.360	0.0387	0.2518		1.429				4.038		
**SGO_0079**	hypothetical protein SGO_0079		1.379				1.380				1.380														
**SGO_0080**	hypothetical protein SGO_0080	-1.239	7.315	0.0013	0.0026	-0.712	7.484	0.0011	0.0018	-0.691	7.490	0.0004	0.0007	0.527	6.346	0.0125	0.0629	0.548	5.745	0.0105	0.0445	0.021	6.581	0.2181	0.8060
**SGO_0081**	hypothetical protein SGO_0081	1.176	4.906	0.0041	0.0126	1.034	4.842	0.0181	0.0685		3.266			-0.142	4.788	0.0913	0.6734		3.224				3.740		

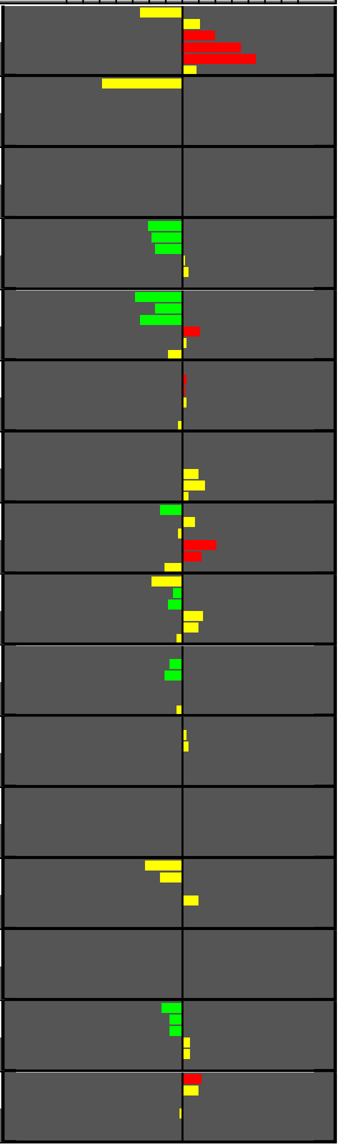

Show detected proteins only
Show all proteins
Filter by category:
ABC Transporter

Proteins found:
1179

Dot Plots
Dot Plots

Test	Cutoff
q-Value	.005
p-Value	

	Signif	Direction	Applies To
(red)	yes	+	ratios, bars
(yellow)	no	n/a	bars
(green)	yes	−	ratios, bars
(pink)	yes	+	*p*- , *q*-Values
(light green)	yes	−	

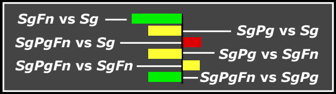

Hendrickson *et al.*

Streptococcus gordonii

Hackett Laboratory UW

Protein	Description	*SgFn vs Sg* Ratio	Sum	*q*-Val	*p*-Val	*SgPg vs Sg* Ratio	Sum	*q*-Val	*p*-Val	*SgPgFn vs Sg* Ratio	Sum	*q*-Val	*p*-Val	*SgPg vs SgFn* Ratio	Sum	*q*-Val	*p*-Val	*SgPgFn vs SgFn* Ratio	Sum	*q*-Val	*p*-Val	*SgPgFn vs SgPg* Ratio	Sum	*q*-Val	*p*-Val
SGO_0095	mccF; microcin immunity protein MccF, putative		6.401				5.271							-1.125	6.431	0.0009	0.0016		5.277				4.758		
SGO_0097	hypothetical protein SGO_0097		6.097				6.097				6.097														
SGO_0098	ribonucleotide reductase-like protein		4.012			0.979	6.324	0.0344	0.1448		4.012				5.487								5.487		
SGO_0099	pulA-2; pullulanase, type I		4.372				3.712				2.396			-0.483	4.566	0.0416	0.2746	-0.792	3.575	0.0732	0.4882	-0.310	3.686	0.0956	0.2721
SGO_0100	maltose operon transcription repressor	0.845	4.371	0.0570	0.3329	0.127	4.089	0.1827	0.9834		3.499			-0.717	3.403				2.107				2.000		
SGO_0101	malA; Maltodextrose utilization protein malA		4.874				4.874				4.874														
SGO_0104	Maltose/maltodextrin-binding protein precursor	-0.242	9.829	0.0207	0.0962	-0.689	9.636	0.0001	0.0000	-0.524	9.703	0.0008	0.0024	-0.447	8.985	0.0077	0.0334	-0.282	8.448	0.0219	0.1109	0.165	8.825	0.0450	0.0968
SGO_0105	malQ; 4-alpha-glucanotransferase	1.097	6.362	0.0042	0.0127	0.873	6.200	0.0011	0.0019	-0.043	5.689	0.0920	0.8746	-0.225	6.182	0.0330	0.2061	-1.140	5.228	0.0045	0.0140	-0.915	5.676	0.0067	0.0043
SGO_0106	glgP-2; maltodextrin phosphorylase	2.848	8.574	0.0005	0.0007	1.969	7.831	0.0002	0.0001	1.923	7.794	0.0001	0.0001	-0.879	8.496	0.0019	0.0044	-0.925	7.869	0.0018	0.0039	-0.046	7.963	0.0238	0.0370
SGO_0107	LPXTG cell wall surface protein, collagen binding domain										2.907								1.783				2.394		
SGO_0108	ruvB; Holliday junction DNA helicase RuvB	-1.035	6.092	0.0022	0.0058		5.505				5.505				3.998				3.387						
SGO_0110	phosphotyrosine protein phosphatase		2.231				2.898							0.668	3.090				1.107				2.385		
SGO_0111	MORN motif family protein		4.115				4.115				4.115														
SGO_0112	putative acyltransferase		5.366				5.366				5.366														
SGO_0113	acdH; alcohol-acetaldehyde dehydrogenase	-2.089	13.012	0.0001	0.0000	-1.505	13.143	0.0002	0.0001	-1.648	13.107	0.0001	0.0001	0.584	11.427	0.0014	0.0031	0.441	10.731	0.0011	0.0015	-0.143	11.620	0.0405	0.0836
SGO_0128	hypothetical protein SGO_0128		3.170				3.170				3.170														

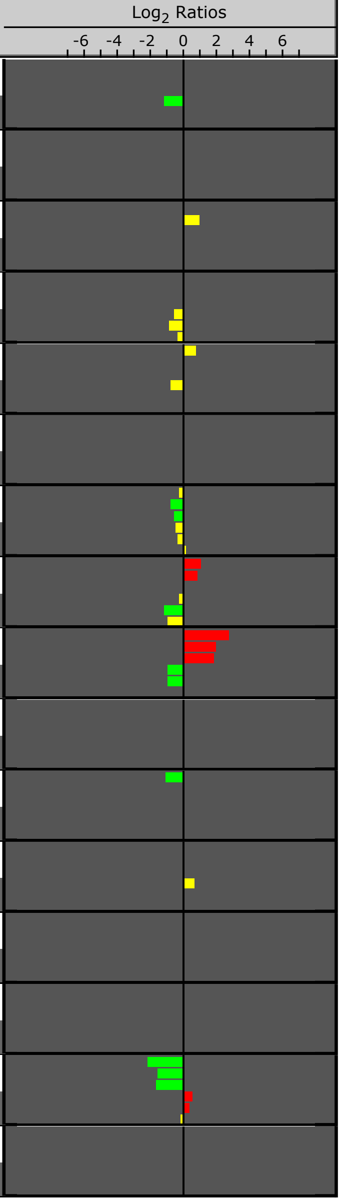

Show detected proteins only
Show all proteins
Filter by category:
ABC Transporter

Proteins found:
1179

Dot Plots
Dot Plots

Test	Cutoff
q-Value	.005
p-Value	

	Signif	Direction	Applies To
red	yes	+	ratios, bars
yellow	no	n/a	bars
green	yes	–	ratios, bars
pink	yes	+	*p*- , *q*-Values
light green	yes	–	

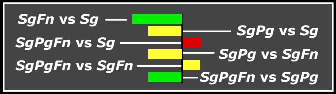

Hendrickson *et al.*

Protein	Description	*SgFn vs Sg*				*SgPg vs Sg*				*SgPgFn vs Sg*				*SgPg vs SgFn*				*SgPgFn vs SgFn*				*SgPgFn vs SgPg*			
		Ratio	Sum	*q*-Val	*p*-Val	Ratio	Sum	*q*-Val	*p*-Val	Ratio	Sum	*q*-Val	*p*-Val	Ratio	Sum	*q*-Val	*p*-Val	Ratio	Sum	*q*-Val	*p*-Val	Ratio	Sum	*q*-Val	*p*-Val
**SGO_0129**	atpI; v-type sodium ATP synthase, chain I		6.741			-3.290	6.881	0.0002	0.0001		6.741				2.937								2.937		
**SGO_0131**	v-type sodium ATP synthase, chain E	-0.135	6.282	0.0858	0.5442		5.425				5.425				4.611				4.000						
**SGO_0132**	ATP synthase (C/AC39) subunit		3.524			-1.088	4.528	0.0494	0.2205		3.524				3.020								3.020		
**SGO_0134**	acetyltransferase, GNAT family	-0.328	4.635	0.0580	0.3390	0.106	5.225	0.1182	0.5924		4.154			0.434	4.222	0.0641	0.4482		1.692				3.779		
**SGO_0135**	v-type sodium ATP synthase, subunit A	-0.017	8.321	0.1433	0.9713	-0.954	7.926	0.0003	0.0003	-1.286	7.825	0.0004	0.0008	-0.937	7.410	0.0031	0.0095	-1.269	6.698	0.0026	0.0068	-0.332	6.712	0.0532	0.1238
**SGO_0136**	v-type sodium ATP synthase, chain B	-0.696	8.587	0.0016	0.0037	-1.309	8.383	0.0007	0.0010	-1.600	8.309	0.0005	0.0011	-0.613	7.410	0.0019	0.0047	-0.904	6.696	0.0022	0.0052	-0.291	6.942	0.0664	0.1660
**SGO_0137**	V-type ATPase, D subunit		3.653				3.653				3.653														
**SGO_0138**	LysM domain protein	0.579	3.778				2.459				2.459				2.525				1.914						
**SGO_0139**	thrC; threonine synthase	0.506	7.391	0.0013	0.0027	0.581	7.434	0.0007	0.0010	-0.511	6.896	0.0090	0.0555	0.074	7.146	0.0368	0.2375	-1.017	6.088	0.0023	0.0056	-1.092	6.748	0.0063	0.0038
**SGO_0142**	hypothetical protein SGO_0142		1.585				1.585				1.585														
**SGO_0144**	hypothetical protein SGO_0144	-1.032	5.671	0.0020	0.0050	-2.511	5.213	0.0174	0.0651		5.092			-1.478	3.796	0.0265	0.1585		2.951				1.063		
**SGO_0145**	polI; DNA polymerase I	0.275	8.112	0.0341	0.1807	-0.566	7.699	0.0007	0.0009	-0.848	7.593	0.0004	0.0009	-0.841	7.372	0.0061	0.0248	-1.124	6.669	0.0048	0.0153	-0.282	6.743	0.0224	0.0329
**SGO_0146**	CoA-binding domain protein	1.056	6.377	0.0048	0.0152	0.659	6.116	0.0087	0.0278	0.244	5.991	0.0505	0.4436	-0.397	6.110	0.0195	0.1112	-0.812	5.412	0.0355	0.2024	-0.416	5.791	0.1828	0.6415
**SGO_0148**	hypothetical protein SGO_0148		4.387			-1.225	4.667	0.0139	0.0492		4.387				1.648								1.648		
**SGO_0150**	fucA1; alpha-L-fucosidase		1.379				1.380				1.380														
**SGO_0152**	tgt; queuine tRNA-ribosyltransferase	0.387	5.773	0.0519	0.2961	1.009	6.079	0.0003	0.0003	-0.431	5.291	0.0027	0.0119	0.622	5.762	0.0322	0.1998	-0.818	4.490	0.0268	0.1420	-1.441	5.438	0.0019	0.0003

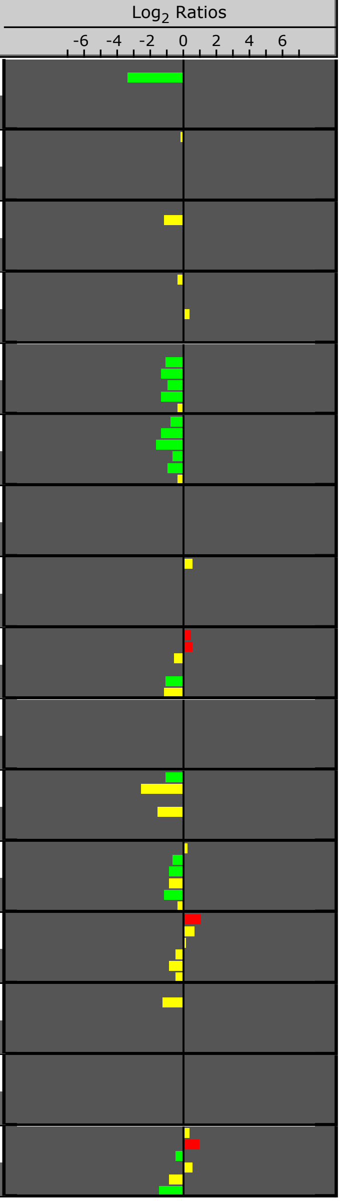

Show detected proteins only
Show all proteins
Filter by category:
ABC Transporter

Proteins found:
1179

Dot Plots
Dot Plots

Test	Cutoff
q-Value	.005
p-Value	

	Signif	Direction	Applies To
red	yes	+	ratios, bars
yellow	no	n/a	bars
green	yes	−	ratios, bars
pink	yes	+	*p*- , *q*-Values
light green	yes	−	

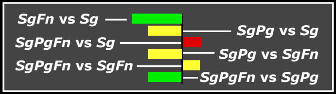

Hendrickson *et al.*

Streptococcus gordonii

Protein	SgFn vs Sg Ratio	SgFn vs Sg Sum	SgFn vs Sg *q*-Val	SgFn vs Sg *p*-Val	SgPg vs Sg Ratio	SgPg vs Sg Sum	SgPg vs Sg *q*-Val	SgPg vs Sg *p*-Val	SgPgFn vs Sg Ratio	SgPgFn vs Sg Sum	SgPgFn vs Sg *q*-Val	SgPgFn vs Sg *p*-Val	SgPg vs SgFn Ratio	SgPg vs SgFn Sum	SgPg vs SgFn *q*-Val	SgPg vs SgFn *p*-Val	SgPgFn vs SgFn Ratio	SgPgFn vs SgFn Sum	SgPgFn vs SgFn *q*-Val	SgPgFn vs SgFn *p*-Val	SgPgFn vs SgPg Ratio	SgPgFn vs SgPg Sum	SgPgFn vs SgPg *q*-Val	SgPgFn vs SgPg *p*-Val
**SGO_0154**	1.787	11.314	0.0007	0.0010	0.517	10.445	0.0109	0.0367	0.826	10.628	0.0007	0.0018	-1.270	10.934	0.0011	0.0022	-0.961	10.418	0.0014	0.0025	0.309	10.324	0.0256	0.0417
	pgi; glucose-6-phosphate isomerase																							
**SGO_0155**		8.348			-4.703	8.402	0.0009	0.0014		8.348				3.118								3.118		
	hypothetical protein SGO_0155																							
**SGO_0156**		7.444			-2.580	7.668	0.0004	0.0004	-2.949	7.620	0.0003	0.0003		4.357				3.370			-0.369	5.181	0.0319	0.0567
	hypothetical protein SGO_0156																							
**SGO_0157**		3.357				3.357				3.357														
	hypothetical protein SGO_0157																							
**SGO_0158**	2.802	8.548	0.0003	0.0002	1.379	7.403	0.0009	0.0014	1.833	7.756	0.0014	0.0050	-1.423	8.300	0.0006	0.0007	-0.969	7.832	0.0020	0.0047	0.454	7.674	0.0376	0.0735
	2,3,4,5-tetrahydropyridine-2-carboxylate N-succinyltransferase, putative																							
**SGO_0159**	1.294	3.787			-0.424	2.804				2.000			-1.718	3.164				2.170				1.063		
	hippurate hydrolase																							
**SGO_0163**	0.563	7.521	0.0030	0.0085	0.746	7.631	0.0010	0.0016	0.009	7.216	0.1026	0.9909	0.182	7.353	0.0069	0.0286	-0.555	6.398	0.0013	0.0022	-0.737	7.118	0.0007	0.0000
	galU; UTP-glucose-1-phosphate uridylyltransferase																							
**SGO_0164**	0.296	7.639	0.0213	0.0992	-0.280	7.349	0.0252	0.1009	-0.524	7.239	0.0018	0.0071	-0.577	7.010	0.0080	0.0354	-0.821	6.303	0.0038	0.0111	-0.244	6.572	0.0594	0.1433
	Glycerol-3-phosphate dehydrogenase [NAD(P)+] (NAD(P)H-dependent glycerol-3-phosphate dehydrogenase)																							
**SGO_0165**		4.275				4.275				4.275														
	ABC transporter, permease/ATP-binding protein SP2075																							
**SGO_0166**		6.237			-2.723	6.342	0.0082	0.0256		6.237				2.000								2.000		
	ABC transporter, permease/ATP-binding protein SP2073																							
**SGO_0167**		4.020				4.020				4.020														
	glutamine amidotransferase																							
**SGO_0168**	-0.600	4.566	0.0250	0.1212		4.156				4.156				2.039				1.429						
	hydrolase, NUDIX family																							
**SGO_0169**	1.929	6.595	0.0023	0.0059	0.502	5.611	0.0352	0.1490	0.646	5.680	0.0064	0.0364	-1.427	6.200	0.0032	0.0101	-1.283	5.622	0.0033	0.0094	0.144	5.383	0.1609	0.5407
	dut; dUTP diphosphatase																							
**SGO_0171**	-0.989	5.628	0.0409	0.2246	-0.402	6.118	0.0338	0.1424	-1.565	5.530	0.0219	0.1728	0.587	4.795	0.0400	0.2623	-0.576	2.911			-1.163	4.672	0.0664	0.1664
	radA; DNA repair protein RadA																							
**SGO_0172**	-0.682	6.005	0.0089	0.0337		5.614				5.614				3.418				2.807						
	conserved hypothetical protein TIGR00266																							
**SGO_0173**	-1.237	7.924	0.0003	0.0002	-3.121	7.570	0.0001	0.0000		7.412			-1.884	6.016	0.0008	0.0013		5.058				3.790		
	Carbonic anhydrase																							

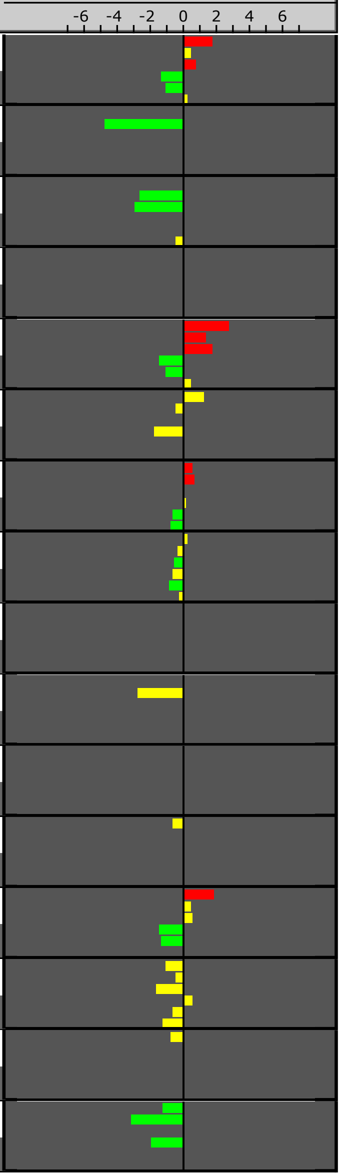

Show detected proteins only
Show all proteins
Filter by category:
ABC Transporter

Proteins found:
1179

Dot Plots
Dot Plots

Test	Cutoff
q-Value	.005
p-Value	

	Signif	Direction	Applies To
red	yes	+	ratios, bars
yellow	no	n/a	bars
green	yes	−	ratios, bars
light red	yes	+	*p*- , *q*-Values
light green	yes	−	*p*- , *q*-Values

SgFn vs Sg — SgPg vs Sg
SgPgFn vs Sg — SgPg vs SgFn
SgPgFn vs SgFn — SgPgFn vs SgPg

Hendrickson *et al.*

Streptococcus gordonii

Summary Table | SgFn vs Sg | SgPg vs Sg | SgPgFn vs Sg | SgPg v SgFn | SgPgFn v SgFn | SgPgFn vs SgPg | Coverage

Protein	Description	*SgFn vs Sg* Ratio	Sum	*q*-Val	*p*-Val	*SgPg vs Sg* Ratio	Sum	*q*-Val	*p*-Val	*SgPgFn vs Sg* Ratio	Sum	*q*-Val	*p*-Val	*SgPg vs SgFn* Ratio	Sum	*q*-Val	*p*-Val	*SgPgFn vs SgFn* Ratio	Sum	*q*-Val	*p*-Val	*SgPgFn vs SgPg* Ratio	Sum	*q*-Val	*p*-Val
SGO_0174	gltX; glutamyl-tRNA synthetase	0.758	8.458	0.0073	0.0266	1.114	8.678	0.0001	0.0000	0.684	8.400	0.0008	0.0023	0.356	8.459	0.0157	0.0841	-0.073	7.627	0.0875	0.6035	-0.429	8.419	0.0055	0.0029
SGO_0179	Membrane protein oxaA 1 precursor		2.585				2.585				2.585														
SGO_0180	jag; hypothetical protein SGO_0180	-0.946	6.660	0.0122	0.0506	-0.132	7.007	0.1414	0.7291	-1.123	6.647	0.0120	0.0809	0.815	6.068	0.0151	0.0803	-0.177	4.949	0.1287	0.9284	-0.991	6.054	0.0521	0.1201
SGO_0181	lipoprotein, putative		5.217				5.217				5.217														
SGO_0182	sapR; sakacin A production response regulator	0.723	4.937	0.0798	0.4978		2.700				2.700				4.080				3.469						
SGO_0188	hydrolase, TatD family	-0.017	5.264	0.1383	0.9279	-0.745	4.646	0.0473	0.2076		4.272			-0.729	4.120	0.0340	0.2137		3.132				2.000		
SGO_0189	primase-related protein		1.585				1.585				1.585														
SGO_0190	hypothetical protein SGO_0190		5.130			-1.685	5.563	0.0110	0.0370	-1.173	5.410	0.0326	0.2689		3.103				1.783			0.512	3.791	0.2040	0.7382
SGO_0191	hypothetical protein SGO_0191		4.351			-1.774	4.548	0.0060	0.0179		4.351				1.063								1.063		
SGO_0193	ksgA; dimethyladenosine transferase	0.004	4.805	0.1428	0.9653	0.844	5.139	0.0514	0.2304		3.820			0.840	4.614	0.0309	0.1910		2.666				3.887		
SGO_0197	predicted ribosome small subunit-dependent GTPase A		4.429				3.541				3.170			-0.890	4.539	0.0020	0.0048	-0.253	3.809	0.0515	0.3175	0.637	3.854	0.0520	0.1190
SGO_0198	rpe; ribulose-phosphate 3-epimerase	2.600	8.472	0.0001	0.0001	0.896	7.170	0.0011	0.0017	0.364	6.853	0.0122	0.0827	-1.704	8.123	0.0002	0.0001	-2.236	7.405	0.0002	0.0001	-0.532	6.794	0.0089	0.0068
SGO_0200	competence-induced protein Ccs50	-1.421	7.784	0.0001	0.0001	-1.166	7.858	0.0002	0.0001	-1.758	7.706	0.0003	0.0003	0.255	6.525	0.0010	0.0018	-0.337	5.633	0.0212	0.1059	-0.591	6.391	0.0165	0.0202
SGO_0201	cmp-binding-factor 1	1.296	8.289	0.0028	0.0079	-0.173	7.404	0.0277	0.1122	-1.090	7.048	0.0011	0.0034	-1.470	7.726	0.0022	0.0060	-2.387	6.927	0.0016	0.0032	-0.917	6.416	0.0048	0.0017
SGO_0202	pur operon repressor	0.149	5.698	0.0870	0.5536	-0.101	5.559	0.1063	0.5282		4.619			-0.250	5.128	0.0384	0.2496		3.648				3.983		
SGO_0203	hypothetical protein SGO_0203		2.700				2.700				2.700														

Log$_2$ Ratios

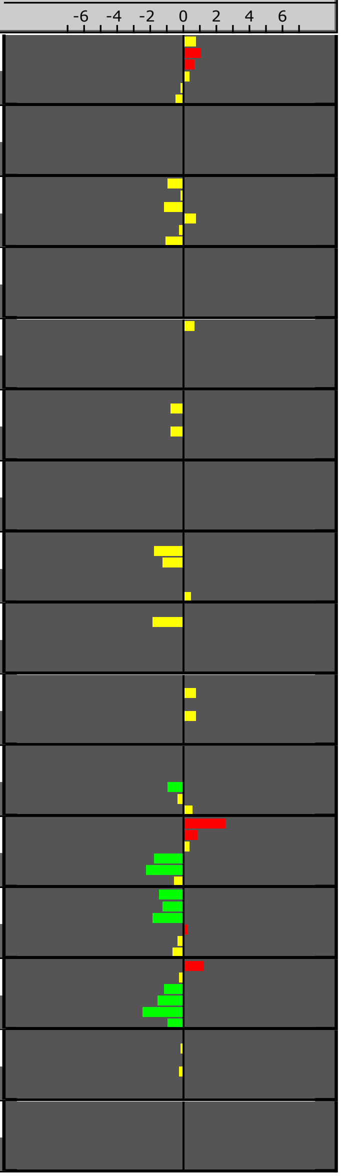

Show detected proteins only
Show all proteins
Filter by category:
ABC Transporter

Proteins found:
1179

Dot Plots
Dot Plots

Test	Cutoff
q-Value	.005
p-Value	

	Signif	Direction	Applies To
red	yes	+	ratios, bars
yellow	no	n/a	bars
green	yes	–	ratios, bars
pink	yes	+	*p*- , *q*-Values
light green	yes	–	

SgFn vs Sg — *SgPg vs Sg*
SgPgFn vs Sg — *SgPg vs SgFn*
SgPgFn vs SgFn — *SgPgFn vs SgPg*

Hendrickson *et al.*

Streptococcus gordonii

Protein	Description	SgFn vs Sg Ratio	SgFn vs Sg Sum	SgFn vs Sg *q*-Val	SgFn vs Sg *p*-Val	SgPg vs Sg Ratio	SgPg vs Sg Sum	SgPg vs Sg *q*-Val	SgPg vs Sg *p*-Val	SgPgFn vs Sg Ratio	SgPgFn vs Sg Sum	SgPgFn vs Sg *q*-Val	SgPgFn vs Sg *p*-Val	SgPg vs SgFn Ratio	SgPg vs SgFn Sum	SgPg vs SgFn *q*-Val	SgPg vs SgFn *p*-Val	SgPgFn vs SgFn Ratio	SgPgFn vs SgFn Sum	SgPgFn vs SgFn *q*-Val	SgPgFn vs SgFn *p*-Val	SgPgFn vs SgPg Ratio	SgPgFn vs SgPg Sum	SgPgFn vs SgPg *q*-Val	SgPgFn vs SgPg *p*-Val
SGO_0204	rpsL; ribosomal protein S12	-0.081	8.109	0.1339	0.8941	0.094	8.177	0.0899	0.4380	-0.064	8.092	0.0304	0.2491	0.174	7.649	0.0829	0.6053	0.016	6.952	0.1239	0.8855	-0.158	7.633	0.0830	0.2217
SGO_0205	rpsG; ribosomal protein S7	0.380	9.252	0.0236	0.1119	0.726	9.456	0.0085	0.0269	0.797	9.499	0.0036	0.0174	0.345	9.103	0.0281	0.1707	0.417	8.530	0.0214	0.1072	0.071	9.299	0.2016	0.7269
SGO_0206	fusA; translation elongation factor G	0.449	12.222	0.0005	0.0006	0.873	12.482	0.0002	0.0001	1.012	12.576	0.0006	0.0015	0.424	12.143	0.0008	0.0013	0.564	11.617	0.0024	0.0061	0.140	12.413	0.0590	0.1419
SGO_0207	gap; glyceraldehyde-3-phosphate dehydrogenase, type I	1.189	12.534	0.0005	0.0007	1.071	12.454	0.0007	0.0010	1.446	12.724	0.0010	0.0034	-0.118	12.434	0.0006	0.0008	0.257	12.020	0.0183	0.0872	0.375	12.579	0.0248	0.0399
SGO_0208	LPXTG cell wall surface protein, glycosyl hydrolase family	0.596	6.284	0.0337	0.1782		4.996				4.996				5.011				4.400						
SGO_0209	pgk; phosphoglycerate kinase	1.455	12.630	0.0002	0.0001	2.191	13.204	0.0002	0.0001	1.708	12.819	0.0001	0.0000	0.736	13.083	0.0007	0.0009	0.252	12.189	0.0031	0.0085	-0.484	13.183	0.0050	0.0022
SGO_0210	sspA; streptococcal surface protein A	-0.950	8.543	0.0059	0.0198	-5.424	7.976	0.0012	0.0020		7.943			-4.474	6.540	0.0009	0.0016		5.865				2.024		
SGO_0211	sspB; streptococcal surface protein B	-4.047	9.262	0.0003	0.0003		9.177				9.177				4.614				4.003						
SGO_0214	transcription regulator, MerR family		1.322				1.322				1.322														
SGO_0215	glnA; glutamine synthetase, type I	0.153	8.746	0.0017	0.0040	-0.087	8.628	0.0722	0.3407	-0.427	8.481	0.0090	0.0555	-0.240	8.195	0.0085	0.0389	-0.579	7.445	0.0059	0.0205	-0.339	7.921	0.0562	0.1327
SGO_0219	metallo-beta-lactamase superfamily protein 1	-0.136	8.107	0.0278	0.1417	0.237	8.297	0.0080	0.0251	-0.152	8.101	0.0183	0.1387	0.373	7.721	0.0007	0.0008	-0.016	6.905	0.1133	0.8056	-0.389	7.715	0.0050	0.0021
SGO_0220	Protein of unknown function (DUF1447) superfamily		3.446				4.294							-0.171	4.418	0.0848	0.6214		2.322				3.781		
SGO_0223	glycoproteinase family protein	0.818	4.028	0.0150	0.0659	1.487	4.380	0.0087	0.0279		3.118			0.669	3.791				1.807				3.087		
SGO_0230	Protein of unknown function, DUF536 family	-0.275	5.406	0.1056	0.6858	0.665	5.843	0.0050	0.0141	0.773	5.967	0.0163	0.1203	0.940	5.270	0.0117	0.0564	1.048	4.788	0.0233	0.1196	0.108	5.720	0.1672	0.5724
SGO_0231	glycerophosphoryl diester phosphodiesterase family protein		3.111				3.111				3.111														
SGO_0232	conserved hypothetical protein TIGR00103		1.807			1.663	4.738	0.0558	0.2527	1.561	4.606	0.0173	0.1289		4.022				3.258			-0.102	4.947	0.1775	0.6200

Log$_2$ Ratios

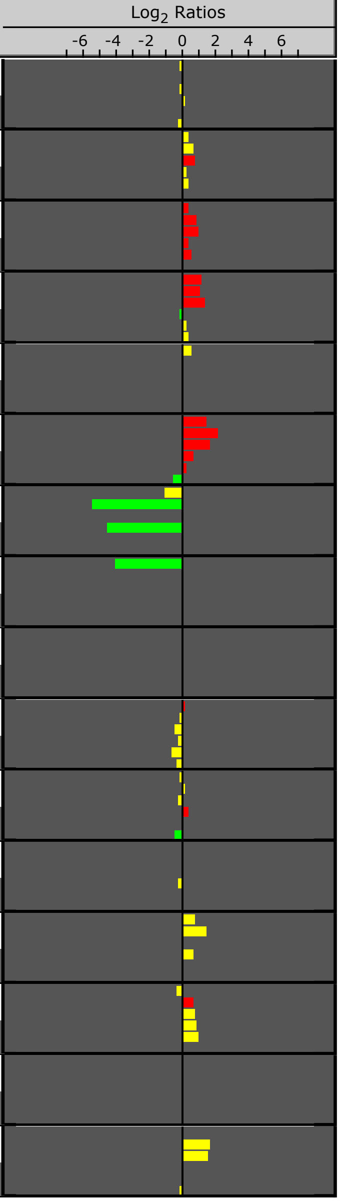

Show detected proteins only
Show all proteins
Filter by category:
ABC Transporter

Proteins found:
1179

Dot Plots
Dot Plots

Test	Cutoff
q-Value	.005
p-Value	

	Signif	Direction	Applies To
red	yes	+	ratios, bars
yellow	no	n/a	bars
green	yes	–	ratios, bars
light red	yes	+	*p*- , *q*-Values
light green	yes	–	

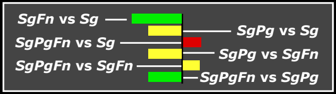

Hendrickson *et al.*

Spectral Counts
Sg Summary Table

Streptococcus gordonii

Hackett Laboratory UW

Summary Table | SgFn vs Sg | SgPg vs Sg | SgPgFn vs Sg | SgPg v SgFn | SgPgFn v SgFn | SgPgFn vs SgPg | Coverage

Protein	Description	SgFn vs Sg Ratio	SgFn vs Sg Sum	SgFn vs Sg *q*-Val	SgFn vs Sg *p*-Val	SgPg vs Sg Ratio	SgPg vs Sg Sum	SgPg vs Sg *q*-Val	SgPg vs Sg *p*-Val	SgPgFn vs Sg Ratio	SgPgFn vs Sg Sum	SgPgFn vs Sg *q*-Val	SgPgFn vs Sg *p*-Val	SgPg vs SgFn Ratio	SgPg vs SgFn Sum	SgPg vs SgFn *q*-Val	SgPg vs SgFn *p*-Val	SgPgFn vs SgFn Ratio	SgPgFn vs SgFn Sum	SgPgFn vs SgFn *q*-Val	SgPgFn vs SgFn *p*-Val	SgPgFn vs SgPg Ratio	SgPgFn vs SgPg Sum	SgPgFn vs SgPg *q*-Val	SgPgFn vs SgPg *p*-Val
SGO_0233	lipoprotein, putative	-1.071	6.570	0.0111	0.0442		5.976				5.976				4.491				3.881						
SGO_0234	pepX; X-Pro dipeptidyl-peptidase	0.223	7.887	0.0036	0.0108	-0.219	7.669	0.0216	0.0849	-0.741	7.449	0.0004	0.0009	-0.442	7.280	0.0043	0.0153	-0.964	6.469	0.0008	0.0008	-0.522	6.807	0.0178	0.0227
SGO_0235	glycerol uptake facilitator protein-like protein		6.742			-1.563	7.157	0.0048	0.0133		6.742				4.642								4.642		
SGO_0236	hypothetical protein SGO_0236		4.509				4.509				4.509														
SGO_0237	ccpA; CcpA protein (proteinase)	0.811	5.501	0.0302	0.1571	1.319	5.790	0.0038	0.0097	0.200	5.141	0.0774	0.7214	0.508	5.557	0.0255	0.1510	-0.611	4.436	0.0348	0.1970	-1.119	5.325	0.0099	0.0084
SGO_0240	mvaD; diphosphomevalonate decarboxylase		2.836				2.836				2.836														
SGO_0242	FMN-dependent dehydrogenase family protein	1.066	2.687				1.058				1.058				1.611				1.000						
SGO_0243	hydroxymethylglutaryl-CoA reductase, degradative		4.989				3.791				3.755			-1.190	4.998	0.0013	0.0029	-0.226	4.377	0.0612	0.3979	0.965	4.260	0.0166	0.0206
SGO_0244	hydroxymethylglutaryl-CoA synthase	2.219	5.446	0.0014	0.0029	2.252	5.461	0.0003	0.0002	2.049	4.551	0.0023	0.0095	0.032	5.664	0.1189	0.9011	-0.170	4.570	0.0834	0.5695	-0.203	5.193	0.0935	0.2633
SGO_0247	pfl; formate acetyltransferase	-0.232	11.349	0.0506	0.2878	-1.086	11.004	0.0005	0.0006	-0.986	11.037	0.0004	0.0007	-0.854	10.354	0.0067	0.0274	-0.755	9.779	0.0088	0.0343	0.100	9.898	0.0316	0.0559
SGO_0252	possible TetR-type transcriptional regulator	0.478	5.107	0.0169	0.0754	-0.542	4.617	0.0297	0.1218	-0.114	4.382	0.0432	0.3739	-1.020	4.417	0.0086	0.0395	-0.593	3.619	0.0510	0.3132	0.427	3.562	0.1586	0.5274
SGO_0253	hypothetical protein SGO_0253		5.655				5.655				5.655														
SGO_0254	helicase, RecD/TraA family		4.018			-0.810	4.643	0.0299	0.1228		4.018				2.622								2.622		
SGO_0255	Signal peptidase I	-1.915	7.565	0.0021	0.0053	-1.618	7.630	0.0027	0.0065	-2.151	7.519	0.0013	0.0045	0.297	5.938	0.0082	0.0371	-0.236	5.062	0.0282	0.1521	-0.533	5.836	0.0073	0.0051
SGO_0256	rnhC; ribonuclease HIII	0.549	4.962	0.0603	0.3566		4.191				4.191				3.177				2.566						
SGO_0258	hypothetical protein SGO_0258	1.231	3.328				1.585			1.585	3.585				2.302			0.355	2.880				2.657		

Log$_2$ Ratios

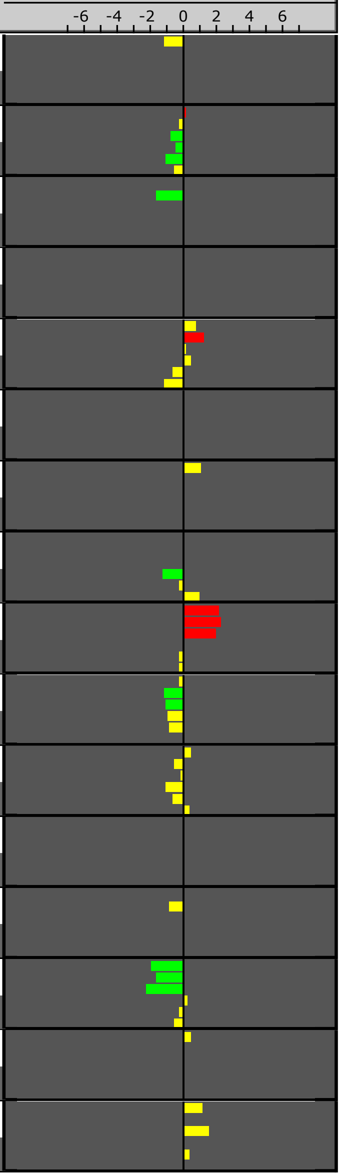

Show detected proteins only
Show all proteins
Filter by category:
ABC Transporter

Proteins found: 1179

Dot Plots
Dot Plots

Test	Cutoff
q-Value	.005
p-Value	

	Signif	Direction	Applies To
red	yes	+	ratios, bars
yellow	no	n/a	bars
green	yes	–	ratios, bars
pink	yes	+	*p*- , *q*-Values
light green	yes	–	

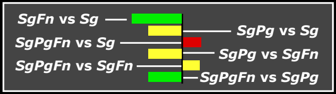

Hendrickson *et al.*

Protein	Description	*SgFn vs Sg* Ratio	Sum	*q*-Val	*p*-Val	*SgPg vs Sg* Ratio	Sum	*q*-Val	*p*-Val	*SgPgFn vs Sg* Ratio	Sum	*q*-Val	*p*-Val	*SgPg vs SgFn* Ratio	Sum	*q*-Val	*p*-Val	*SgPgFn vs SgFn* Ratio	Sum	*q*-Val	*p*-Val	*SgPgFn vs SgPg* Ratio	Sum	*q*-Val	*p*-Val
SGO_0260	DNA mismatch binding protein MutS2	-0.703	6.887	0.0044	0.0135	-0.106	7.135	0.0376	0.1609	-1.380	6.661	0.0004	0.0008	0.597	6.310	0.0062	0.0249	-0.677	5.083	0.0123	0.0543	-1.274	6.073	0.0045	0.0014
SGO_0261	acetyltransferase, GNAT family		1.585				1.585				1.585														
SGO_0262	dipeptidase	0.568	6.914	0.0032	0.0094	0.829	7.073	0.0008	0.0012	-0.877	6.228	0.0006	0.0017	0.261	6.793	0.0115	0.0553	-1.445	5.499	0.0010	0.0014	-1.706	6.302	0.0019	0.0003
SGO_0263	trx-1; thioredoxin	0.095	7.290	0.0900	0.5751	0.527	7.504	0.0012	0.0020	1.216	7.973	0.0040	0.0199	0.431	7.051	0.0237	0.1383	1.121	6.893	0.0087	0.0340	0.690	7.637	0.0306	0.0536
SGO_0268	mechanosensitive transport protein		5.055			-1.945	5.229	0.0268	0.1082		5.055				1.585								1.585		
SGO_0269	hypothetical protein SGO_0269		4.194				4.194				4.194														
SGO_0272	hypothetical protein SGO_0272	0.119	6.592	0.0674	0.4081	-0.248	6.402	0.0007	0.0009	0.120	6.586	0.0310	0.2548	-0.367	5.966	0.0163	0.0892	0.001	5.530	0.1304	0.9477	0.368	5.959	0.0212	0.0303
SGO_0276	gdhA; glutamate dehydrogenase (NADP)	0.718	9.428	0.0029	0.0083	0.383	9.225	0.0017	0.0034	0.460	9.271	0.0031	0.0143	-0.334	9.070	0.0083	0.0379	-0.258	8.496	0.0206	0.1025	0.077	8.931	0.1104	0.3263
SGO_0277	pyrA; Dihydroorotate dehydrogenase	0.354	6.057	0.0447	0.2509	-0.412	5.671	0.0422	0.1820		4.856			-0.766	5.384	0.0153	0.0815		4.110				3.945		
SGO_0278	msrA; Peptide methionine sulfoxide reductase msrA/msrB	1.709	3.094				1.000				1.000				2.196				1.585						
SGO_0279	lipoprotein, putative		3.902				3.902				3.902														
SGO_0280	trzA; ethylammeline chlorohydrolase	0.678	7.016	0.0256	0.1260	-0.591	6.340	0.0170	0.0629		5.584			-1.269	6.326	0.0124	0.0620		5.224				4.531		
SGO_0282	cell wall polysaccharide biosynthesis protein		5.347				5.347				5.347														
SGO_0284	transcriptional regulator		1.058				1.058				1.058														
SGO_0286	DNA mismatch repair protein MutS, putative	-1.447	5.729	0.0117	0.0484		5.487			-1.900	5.668	0.0073	0.0419		2.525			-0.453	2.705				2.072		
SGO_0288	GDSL-like lipase/acylhydrolase		3.231				3.007							-1.232	3.610	0.0111	0.0536		2.107				2.494		

Log$_2$ Ratios: -6 -4 -2 0 2 4 6

- Show detected proteins only
- Show all proteins
- Filter by category: ABC Transporter

Proteins found: 1179

Dot Plots
Dot Plots

Test	Cutoff
q-Value	.005
p-Value	

	Signif	Direction	Applies To
red	yes	+	ratios, bars
yellow	no	n/a	bars
green	yes	–	ratios, bars
pink	yes	+	*p*- , *q*-Values
light green	yes	–	*p*- , *q*-Values

Hendrickson *et al.*

Protein	*SgFn vs Sg* Ratio	Sum	*q*-Val	*p*-Val	*SgPg vs Sg* Ratio	Sum	*q*-Val	*p*-Val	*SgPgFn vs Sg* Ratio	Sum	*q*-Val	*p*-Val	*SgPg vs SgFn* Ratio	Sum	*q*-Val	*p*-Val	*SgPgFn vs SgFn* Ratio	Sum	*q*-Val	*p*-Val	*SgPgFn vs SgPg* Ratio	Sum	*q*-Val	*p*-Val	Description
**SGO_0290**		5.411			-1.343	5.871	0.0144	0.0519	-1.342	5.657	0.0373	0.3152		3.486				1.857			0.001	4.065	0.2458	0.9518	copper -translocating P-type ATPase
**SGO_0291**	-1.699	7.622	0.0005	0.0008	-1.402	7.697	0.0008	0.0011	-2.286	7.507	0.0004	0.0006	0.297	6.175	0.0019	0.0045	-0.587	5.151	0.0046	0.0146	-0.884	5.946	0.0057	0.0032	copper-translocating P-type ATPase
**SGO_0292**	-1.515	8.103	0.0001	0.0000	0.881	9.177	0.0004	0.0005	1.040	9.282	0.0003	0.0005	2.395	8.289	0.0002	0.0001	2.555	7.814	0.0003	0.0001	0.159	9.120	0.0391	0.0774	spxB; pyruvate oxidase
**SGO_0294**		4.271				4.271				4.271															ABC transporter, putative
**SGO_0297**	2.180	5.828	0.0348	0.1852		2.459				2.459				5.168				4.557							6-phospho-beta-glucosidase
**SGO_0299**		0.585				0.585				0.585															putative histidine kinase
**SGO_0301**										1.981								0.857				1.468			ABC transporter ATP-binding protein-like protein
**SGO_0306**		3.886				3.886				3.886															ABC transporter ATP-binding protein-like protein
**SGO_0307**		2.718				2.718				2.718															pabB; chorismate binding enzyme
**SGO_0310**	2.302	4.772	0.0011	0.0022	1.387	3.401	0.0144	0.0522		2.202			-0.916	4.329	0.0290	0.1782		3.382				2.063			metE; 5-methyltetrahydropteroyltriglutamate--homocysteine S-methyltransferase
**SGO_0312**	1.214	11.377	0.0002	0.0002	0.735	11.059	0.0001	0.0000	0.992	11.225	0.0001	0.0000	-0.479	11.127	0.0008	0.0013	-0.222	10.630	0.0039	0.0115	0.258	11.002	0.0042	0.0012	xfp; D-xylulose 5-phosphate/D-fructose 6-phosphate phosphoketolase
**SGO_0317**		8.639				8.639			-5.050	8.661	0.0082	0.0481						1.461				2.072			LPXTG cell wall surface protein, serine protease, subtilase family
**SGO_0321**	1.577	7.033	0.0012	0.0024	0.887	6.560	0.0098	0.0322	0.594	6.384	0.0162	0.1194	-0.690	6.785	0.0021	0.0052	-0.983	6.071	0.0015	0.0030	-0.292	6.267	0.0650	0.1584	polypeptide deformylase
**SGO_0323**		1.000			0.576	2.317				1.000				1.063								1.063			pseudouridine synthase rRNA-specific
**SGO_0324**		5.618				5.618				5.618															hypothetical protein SGO_0324
**SGO_0325**		3.058				3.058				3.058															probable membrane protein, putative

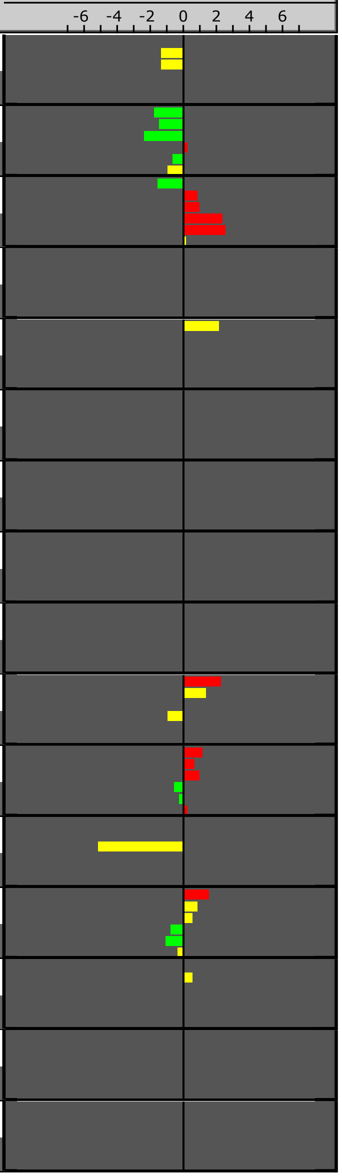

Show detected proteins only
Show all proteins
Filter by category:
ABC Transporter

Proteins found:
1179

Dot Plots
Dot Plots

Test	Cutoff
***q*-Value**	.005
p-Value	

	Signif	Direction	Applies To
(red)	yes	+	ratios, bars
(yellow)	no	n/a	bars
(green)	yes	−	ratios, bars
(pink)	yes	+	*p*- , *q*-Values
(light green)	yes	−	*p*- , *q*-Values

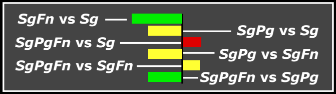

Hendrickson *et al.*

Streptococcus gordonii

Summary Table | SgFn vs Sg | SgPg vs Sg | SgPgFn vs Sg | SgPg v SgFn | SgPgFn v SgFn | SgPgFn vs SgPg | Coverage

Protein	Description	*SgFn vs Sg*				*SgPg vs Sg*				*SgPgFn vs Sg*				*SgPg vs SgFn*				*SgPgFn vs SgFn*				*SgPgFn vs SgPg*			
		Ratio	Sum	*q*-Val	*p*-Val	Ratio	Sum	*q*-Val	*p*-Val	Ratio	Sum	*q*-Val	*p*-Val	Ratio	Sum	*q*-Val	*p*-Val	Ratio	Sum	*q*-Val	*p*-Val	Ratio	Sum	*q*-Val	*p*-Val
SGO_0326	probable membrane protein, putative		6.681			-3.296	6.752	0.0097	0.0317		6.681				1.871								1.871		
SGO_0327	Lipopolysaccharide N-acetylglucosaminyltransferase	0.798	3.924	0.0200	0.0913	-0.856	3.376	0.0346	0.1462		3.025			-1.654	2.700				1.692				0.648		
SGO_0328	transmembrane protein, putative		4.383				4.383				4.383														
SGO_0329	hypothetical protein SGO_0329		6.839			-4.732	6.866	0.0187	0.0718		6.839				0.585								0.585		
SGO_0330	hypothetical protein SGO_0330		5.031				5.031				5.031														
SGO_0331	hypothetical protein SGO_0331		5.455				5.455				5.455														
SGO_0332	hypothetical protein SGO_0332		5.722				5.722				5.722														
SGO_0333	rpsO; ribosomal protein S15	-0.323	8.323	0.0334	0.1757	-0.089	8.419	0.0644	0.2989	0.039	8.488	0.0801	0.7503	0.234	7.759	0.0419	0.2768	0.362	7.225	0.0310	0.1718	0.128	7.930	0.1089	0.3178
SGO_0337	trx-2; thioredoxin		1.000			2.161	3.452				1.000				2.648								2.648		
SGO_0339	hypothetical protein SGO_0339	3.050	5.724	0.0138	0.0594	-0.424	2.389				1.585			-3.474	5.190	0.0118	0.0574		4.516				0.648		
SGO_0342	pepF-2; oligoendopeptidase	1.201	8.387	0.0004	0.0004	0.572	7.982	0.0036	0.0093	0.291	7.819	0.0031	0.0143	-0.629	8.075	0.0021	0.0050	-0.909	7.359	0.0009	0.0010	-0.280	7.594	0.0330	0.0606
SGO_0344	pnpA; polyribonucleotide nucleotidyltransferase	0.219	9.215	0.0124	0.0516	-0.124	9.039	0.0049	0.0139	-0.094	9.053	0.0059	0.0326	-0.343	8.646	0.0046	0.0170	-0.313	8.048	0.0061	0.0214	0.030	8.477	0.0735	0.1898
SGO_0345	cysE; serine O-acetyltransferase		3.663				3.663				3.663														
SGO_0348	reductase	-0.407	4.511			-1.348	4.537	0.0036	0.0090		3.700			-0.942	3.810	0.0043	0.0151		2.170				2.839		
SGO_0349	cysS; cysteinyl-tRNA synthetase	0.798	7.081	0.0084	0.0315	0.002	6.611	0.1832	0.9875	0.188	6.710	0.0171	0.1270	-0.796	6.568	0.0072	0.0301	-0.611	6.029	0.0125	0.0552	0.186	6.196	0.0419	0.0871
SGO_0352	ABC transporter, ATP-binding protein SP1580	-0.349	7.992	0.0015	0.0032	-0.745	7.834	0.0012	0.0022	-0.788	7.816	0.0003	0.0003	-0.396	7.112	0.0042	0.0144	-0.438	6.480	0.0007	0.0007	-0.043	6.880	0.1733	0.6020

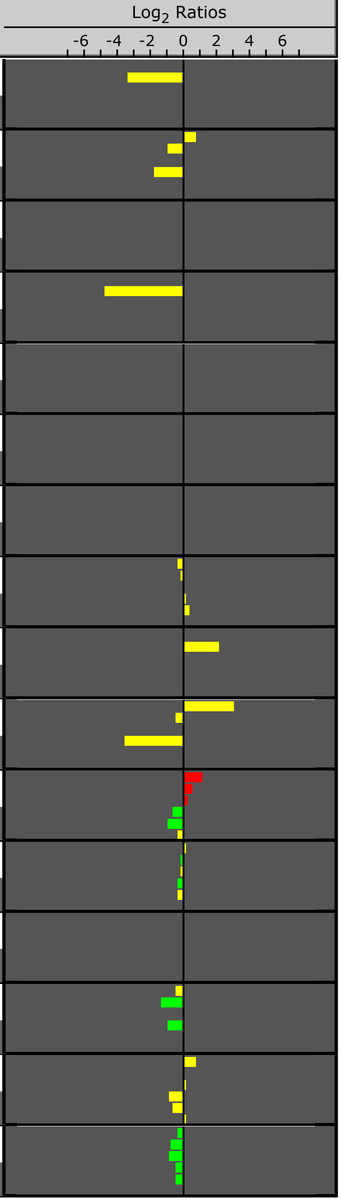

Show detected proteins only
Show all proteins
Filter by category:
ABC Transporter

Proteins found:
1179

Dot Plots
Dot Plots

Test	Cutoff
q-Value	.005
p-Value	

	Signif	Direction	Applies To
(red)	yes	+	ratios, bars
(yellow)	no	n/a	bars
(green)	yes	−	ratios, bars
(pink)	yes	+	*p*- , *q*-Values
(light green)	yes	−	

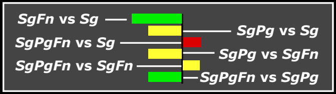

Hendrickson *et al.*

Protein	Description	SgFn vs Sg Ratio	Sum	*q*-Val	*p*-Val	SgPg vs Sg Ratio	Sum	*q*-Val	*p*-Val	SgPgFn vs Sg Ratio	Sum	*q*-Val	*p*-Val	SgPg vs SgFn Ratio	Sum	*q*-Val	*p*-Val	SgPgFn vs SgFn Ratio	Sum	*q*-Val	*p*-Val	SgPgFn vs SgPg Ratio	Sum	*q*-Val	*p*-Val
**SGO_0353**	transport protein	1.406	4.544	0.0136	0.0580	0.905	4.160	0.0171	0.0639		2.619			-0.501	4.341	0.0414	0.2728		2.979				3.040		
**SGO_0355**	RNA methyltransferase, TrmH family, group 3	0.730	5.564	0.0047	0.0149	0.966	5.720	0.0046	0.0127		4.154			0.236	5.496	0.0349	0.2222		3.759				4.612		
**SGO_0356**	hypothetical protein SGO_0356		1.379				1.380				1.380														
**SGO_0357**	degV; DegV family fatty acid binding protein	1.098	7.238	0.0022	0.0058	1.449	7.485	0.0013	0.0025	1.313	7.388	0.0012	0.0041	0.351	7.343	0.0025	0.0070	0.214	6.658	0.0091	0.0360	-0.137	7.442	0.0627	0.1523
**SGO_0358**	rplM; ribosomal protein L13	0.443	9.550	0.0112	0.0451	0.122	9.371	0.0784	0.3765	0.343	9.490	0.0083	0.0498	-0.321	9.085	0.0082	0.0369	-0.100	8.576	0.0520	0.3226	0.221	9.027	0.0116	0.0115
**SGO_0359**	rpsI; ribosomal protein S9	-0.649	8.262	0.0156	0.0689	-0.466	8.319	0.0137	0.0485	0.022	8.546	0.0981	0.9422	0.184	7.477	0.0730	0.5246	0.671	7.145	0.0101	0.0418	0.488	7.813	0.0120	0.0122
**SGO_0361**	immunity repressor protein	0.296	4.951	0.0570	0.3323	-0.553	4.546	0.0329	0.1384		3.789			-0.849	4.222	0.0132	0.0679		2.972				2.740		
**SGO_0368**	merA; mercury(II) reductase	1.733	4.471	0.0048	0.0150		2.345			1.075	3.371	0.0148	0.1064		3.583			-0.659	3.359	0.0554	0.3497		1.883		
**SGO_0371**	putative transcriptional regulator		2.000				2.000				2.000														
**SGO_0372**	malate oxidoreductase	1.711	6.450	0.0017	0.0040	1.166	6.049	0.0026	0.0062	0.792	5.806	0.0059	0.0319	-0.545	6.289	0.0048	0.0177	-0.919	5.539	0.0025	0.0064	-0.374	5.811	0.0055	0.0029
**SGO_0374**	Response regulator of the LytR/AlgR family	0.739	6.858	0.0028	0.0079	-0.169	6.358	0.0363	0.1547	-1.123	6.002	0.0022	0.0094	-0.908	6.283	0.0019	0.0047	-1.862	5.419	0.0013	0.0022	-0.954	5.376	0.0145	0.0161
**SGO_0376**	ABC transporter ATP-binding protein		3.322				3.322			-0.926	3.932								1.272				1.883		
**SGO_0378**	hypothetical protein SGO_0378		4.294												3.781				3.170						
**SGO_0379**	Protein of unknown function (DUF421) family		4.594				4.594				4.594														
**SGO_0380**	hypothetical protein SGO_0380		3.459				3.459				3.459														
**SGO_0384**	putative carboxylate-amine/thiol ligase	-0.291	5.820	0.0649	0.3905	-0.385	5.773	0.0578	0.2637	-0.243	5.827	0.0418	0.3577	-0.094	5.081	0.0912	0.6706	0.048	4.532	0.1293	0.9361	0.142	5.090	0.1416	0.4514

Log$_2$ Ratios

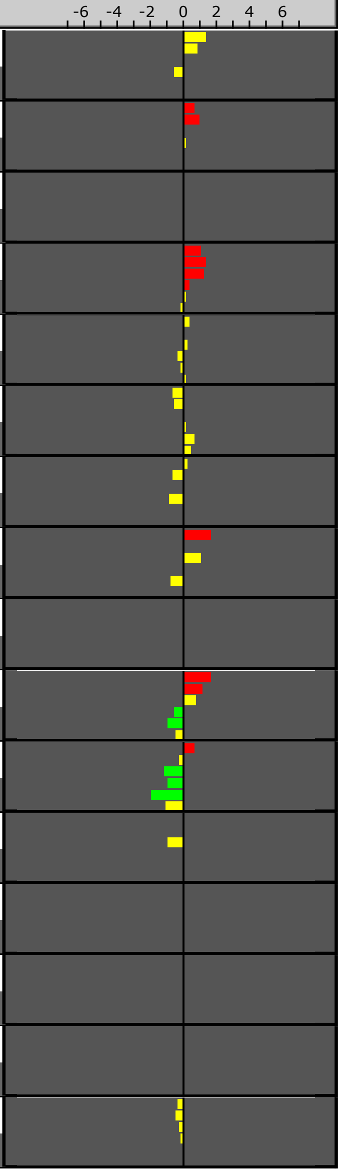

Show detected proteins only
Show all proteins
Filter by category:
ABC Transporter

Proteins found:
1179

Dot Plots
Dot Plots

Test	Cutoff
q-Value	.005
p-Value	

	Signif	Direction	Applies To
red	yes	+	ratios, bars
yellow	no	n/a	bars
green	yes	–	ratios, bars
light red	yes	+	*p*- , *q*-Values
light green	yes	–	

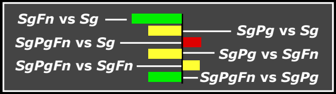

Hendrickson *et al.*

Streptococcus gordonii

Summary Table | SgFn vs Sg | SgPg vs Sg | SgPgFn vs Sg | SgPg v SgFn | SgPgFn v SgFn | SgPgFn vs SgPg | Coverage

Protein	Description	SgFn vs Sg Ratio	Sum	*q*-Val	*p*-Val	SgPg vs Sg Ratio	Sum	*q*-Val	*p*-Val	SgPgFn vs Sg Ratio	Sum	*q*-Val	*p*-Val	SgPg vs SgFn Ratio	Sum	*q*-Val	*p*-Val	SgPgFn vs SgFn Ratio	Sum	*q*-Val	*p*-Val	SgPgFn vs SgPg Ratio	Sum	*q*-Val	*p*-Val
SGO_0385	exo-beta-D-fructosidase	0.670	7.728	0.0026	0.0070		6.351				6.351				6.514				5.903						
SGO_0387	hypothetical protein SGO_0387		4.194				4.194				4.194														
SGO_0388	LPXTG cell wall surface protein, zinc carboxypeptidase family	0.224	7.351	0.0555	0.3217		6.251				6.251				5.931				5.320						
SGO_0389	possible phosphoserine phosphatase	3.458	5.613	0.0418	0.2326		1.000				1.000				5.039				4.429						
SGO_0390	glycerol-3-phosphate dehydrogenase (NAD(P)+)	1.938	8.321	0.0014	0.0028	1.564	8.027	0.0010	0.0015	1.196	7.757	0.0003	0.0005	-0.374	8.298	0.0161	0.0876	-0.742	7.537	0.0050	0.0165	-0.369	7.920	0.0236	0.0363
SGO_0392	phosphoglycerate mutase	3.821	6.458	0.0090	0.0344	2.875	5.566	0.0232	0.0922	2.271	4.127			-0.946	6.501	0.0011	0.0021	-1.550	5.511	0.0113	0.0485	-0.604	5.365	0.0830	0.2216
SGO_0393	D-alanyl-D-alanine carboxypeptidase	-0.858	5.430	0.0702	0.4299		5.105				5.105				2.611				2.000						
SGO_0396	Transcriptional regulator, PadR family		2.322				2.322				2.322														
SGO_0398	ABC transporter ATP-binding protein	-1.902	7.059	0.0021	0.0053	-2.695	6.928	0.0017	0.0033	-2.749	6.912	0.0009	0.0028	-0.794	4.991	0.0183	0.1028	-0.848	4.338	0.0119	0.0517	-0.054	4.508	0.2021	0.7300
SGO_0400	hrcA; heat-inducible transcription repressor HrcA	0.608	4.887	0.0421	0.2346		3.423				3.423				3.724				3.113						
SGO_0401	grpE; co-chaperone GrpE	0.727	7.007	0.0294	0.1511	0.166	6.709	0.1742	0.9248	0.875	7.096	0.0122	0.0835	-0.561	6.510	0.0076	0.0327	0.148	6.218	0.0233	0.1210	0.708	6.598	0.0127	0.0135
SGO_0402	dnaK; DnaK chaperone protein	1.202	11.615	0.0001	0.0001	0.461	11.142	0.0013	0.0022	0.487	11.157	0.0007	0.0019	-0.741	11.257	0.0002	0.0001	-0.715	10.656	0.0003	0.0001	0.026	10.853	0.1603	0.5363
SGO_0404	dnaJ; DnaJ chaparone protein	-2.009	7.978	0.0052	0.0171	-2.045	7.970	0.0057	0.0167	-2.460	7.910	0.0032	0.0147	-0.036	6.073	0.0706	0.5026	-0.451	5.300	0.0313	0.1735	-0.415	5.889	0.0769	0.2007
SGO_0407	truA; tRNA pseudouridine synthase A	1.455	4.510	0.0205	0.0948	-0.231	2.696				1.807			-1.686	3.963	0.0161	0.0876		3.145				1.063		
SGO_0408	zmpB; zinc metalloproteinase B		8.890				8.890			-4.983	8.913	0.0027	0.0117						1.783				2.394		
SGO_0409	pyridoxine kinase	7.517	9.176	0.0184	0.0834	1.598	3.467	0.0233	0.0929		0.643			-5.919	8.682	0.0007	0.0010		8.048				2.735		

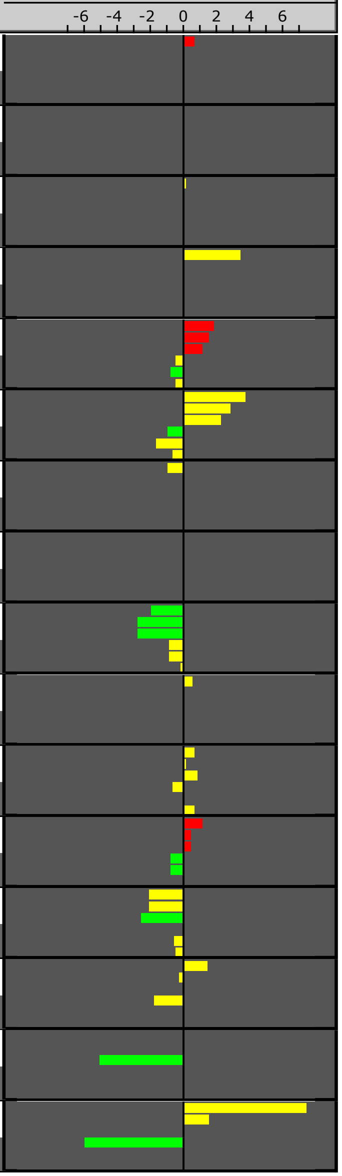

Show detected proteins only
Show all proteins
Filter by category:
ABC Transporter

Proteins found: 1179

Dot Plots
Dot Plots

Test	Cutoff
q-Value	.005
p-Value	

	Signif	Direction	Applies To
(red)	yes	+	ratios, bars
(yellow)	no	n/a	bars
(green)	yes	–	ratios, bars
(pink)	yes	+	*p*- , *q*-Values
(light green)	yes	–	

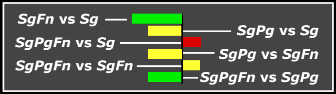

Spectral Counts
Sg Summary Table

Streptococcus gordonii

Hackett Laboratory UW

Summary Table | SgFn vs Sg | SgPg vs Sg | SgPgFn vs Sg | SgPg v SgFn | SgPgFn v SgFn | SgPgFn vs SgPg | Coverage

Protein	Description	*SgFn vs Sg* Ratio	Sum	*q*-Val	*p*-Val	*SgPg vs Sg* Ratio	Sum	*q*-Val	*p*-Val	*SgPgFn vs Sg* Ratio	Sum	*q*-Val	*p*-Val	*SgPg vs SgFn* Ratio	Sum	*q*-Val	*p*-Val	*SgPgFn vs SgFn* Ratio	Sum	*q*-Val	*p*-Val	*SgPgFn vs SgPg* Ratio	Sum	*q*-Val	*p*-Val
**SGO_0411**	conserved hypothetical protein TIGR01440		8.854				4.454				3.566			-4.261	8.407	0.0072	0.0303	-4.144	7.766	0.0451	0.2683	0.118	4.564	0.1587	0.5290
**SGO_0412**	tig; trigger factor	0.157	10.717	0.0298	0.1538	0.831	11.109	0.0005	0.0006	1.089	11.280	0.0002	0.0002	0.673	10.654	0.0006	0.0008	0.932	10.208	0.0004	0.0003	0.259	11.086	0.0051	0.0025
**SGO_0413**	DNA-directed RNA polymerase delta chain	2.206	7.129	0.0003	0.0004	2.022	7.008	0.0040	0.0107	1.445	6.540	0.0007	0.0018	-0.184	7.251	0.0657	0.4612	-0.761	6.382	0.0007	0.0007	-0.577	6.900	0.0368	0.0718
**SGO_0415**	secA; preprotein translocase, SecA subunit	-1.134	10.674	0.0005	0.0007	-1.044	10.702	0.0005	0.0006	-1.184	10.658	0.0003	0.0005	0.090	9.530	0.0349	0.2222	-0.050	8.850	0.0760	0.5089	-0.140	9.505	0.0226	0.0337
**SGO_0416**	phospho-2-dehydro-3-deoxyheptonate aldolase	1.455	7.692	0.0001	0.0001	0.754	7.228	0.0087	0.0280	-0.211	6.715	0.0597	0.5367	-0.702	7.428	0.0028	0.0085	-1.666	6.527	0.0009	0.0010	-0.964	6.657	0.0227	0.0340
**SGO_0418**	alr; alanine racemase		5.266				3.870							-1.405	5.217	0.0004	0.0004		4.142				3.357		
**SGO_0425**	ansB; asparaginase	2.236	3.572			1.326	2.868				1.058			-0.910	3.396				2.170				1.871		
**SGO_0426**	Cof family protein	0.886	6.136	0.0062	0.0214	-0.316	5.480	0.0492	0.2190	-0.210	5.146	0.0650	0.5907	-1.201	5.513	0.0025	0.0071	-1.096	4.682	0.0291	0.1586	0.105	4.407	0.1979	0.7048
**SGO_0427**	universal stress protein family	3.220	7.650	0.0005	0.0006	1.411	6.151	0.0006	0.0008	1.454	6.180	0.0001	0.0001	-1.809	7.352	0.0008	0.0012	-1.766	6.750	0.0009	0.0012	0.043	6.199	0.1767	0.6150
**SGO_0429**	aspartate transaminase	-0.087	9.283	0.0341	0.1809	-0.208	9.238	0.0673	0.3144	-0.434	9.126	0.0012	0.0042	-0.121	8.678	0.0795	0.5757	-0.347	7.952	0.0016	0.0034	-0.226	8.510	0.0878	0.2406
**SGO_0430**	LPXTG cell wall surface protein	-2.297	9.650	0.0001	0.0000		9.382				9.382				6.577				5.967						
**SGO_0431**	GTP-sensing transcriptional pleiotropic repressor codY	0.352	4.954	0.0932	0.5974	0.147	5.310	0.1464	0.7587		4.268			-0.204	4.493	0.0326	0.2026		2.429				3.837		
**SGO_0432**	entB; isochorismatase family protein	-1.338	6.161	0.0267	0.1349	0.535	7.190	0.0066	0.0198	0.234	7.019	0.0184	0.1401	1.873	6.100	0.0126	0.0633	1.572	5.226	0.0121	0.0526	-0.301	6.773	0.0214	0.0309
**SGO_0434**	aspS-2; aspartyl-tRNA synthetase	-0.624	7.577	0.0152	0.0669	-0.559	7.587	0.0084	0.0265	-0.962	7.443	0.0026	0.0113	0.066	6.756	0.1206	0.9195	-0.338	5.963	0.0483	0.2909	-0.404	6.589	0.0524	0.1213
**SGO_0435**	gatC; glutamyl-tRNA(Gln) amidotransferase, C subunit	-0.058	6.298	0.0814	0.5104	-0.003	6.327	0.1779	0.9549	0.062	6.359	0.0647	0.5867	0.055	5.782	0.0639	0.4458	0.120	5.204	0.0298	0.1626	0.065	5.843	0.1504	0.4844
**SGO_0436**	gatA; glutamyl-tRNA(Gln) amidotransferase, A subunit	-0.868	9.074	0.0011	0.0021	-0.300	9.301	0.0091	0.0294	-0.390	9.261	0.0025	0.0108	0.568	8.373	0.0013	0.0027	0.478	7.708	0.0009	0.0010	-0.090	8.584	0.0541	0.1266

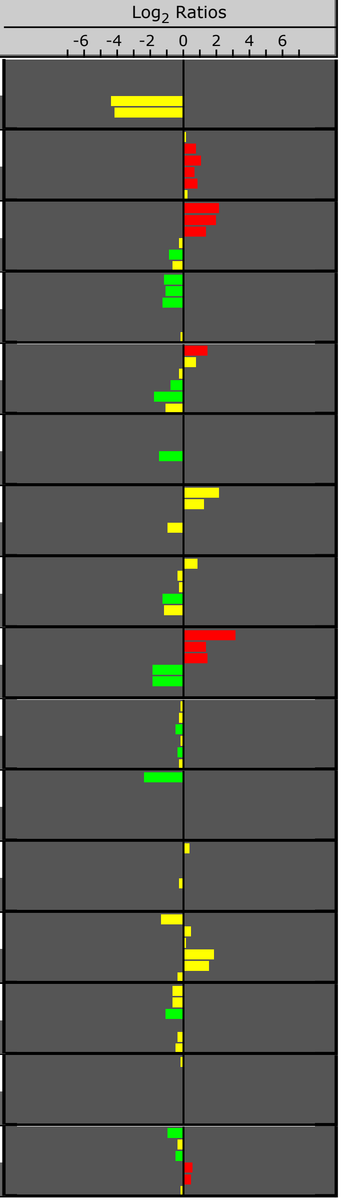

Show detected proteins only
Show all proteins
Filter by category:
ABC Transporter

Proteins found:
1179

Dot Plots
Dot Plots

Test	Cutoff
***q*-Value**	.005
p-Value	

	Signif	Direction	Applies To
(red)	yes	+	ratios, bars
(yellow)	no	n/a	bars
(green)	yes	−	ratios, bars
(light red)	yes	+	*p-* , *q*-Values
(light green)	yes	−	

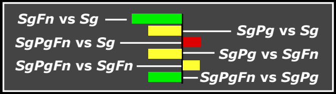

Hendrickson *et al.*

Streptococcus gordonii

Protein	*SgFn vs Sg*				*SgPg vs Sg*				*SgPgFn vs Sg*				*SgPg vs SgFn*				*SgPgFn vs SgFn*				*SgPgFn vs SgPg*				Description
	Ratio	Sum	*q*-Val	*p*-Val	Ratio	Sum	*q*-Val	*p*-Val	Ratio	Sum	*q*-Val	*p*-Val	Ratio	Sum	*q*-Val	*p*-Val	Ratio	Sum	*q*-Val	*p*-Val	Ratio	Sum	*q*-Val	*p*-Val	
**SGO_0437**	-0.052	8.674	0.1104	0.7212	0.549	8.996	0.0018	0.0038	0.341	8.903	0.0263	0.2113	0.601	8.467	0.0037	0.0121	0.393	7.762	0.0355	0.2029	-0.208	8.655	0.1558	0.5048	gatB; glutamyl-tRNA(Gln) amidotransferase, B subunit
**SGO_0440**	4.334	7.132	0.0002	0.0002	1.808	4.908	0.0017	0.0035	1.671	4.100	0.0035	0.0167	-2.526	6.782	0.0004	0.0003	-2.663	6.048	0.0122	0.0534	-0.137	4.572	0.1770	0.6170	L-iditol 2-dehydrogenase BH3949
**SGO_0444**		3.087				3.087				3.087															HAD superfamily (subfamily IIIA) phosphatase, TIGR01668
**SGO_0445**	0.991	7.156	0.0005	0.0006	0.374	6.781	0.0137	0.0486	-0.388	6.403	0.0100	0.0638	-0.617	6.781	0.0019	0.0044	-1.379	5.916	0.0006	0.0005	-0.762	6.115	0.0125	0.0132	GTP-binding protein
**SGO_0446**		3.585				3.585				3.585															conserved hypothetical protein TIGR00253
**SGO_0447**	0.891	5.986	0.0080	0.0300	-0.822	5.135	0.0144	0.0518		4.453			-1.713	5.261	0.0044	0.0155		4.251				3.212			nadD; nicotinate (nicotinamide) nucleotide adenylyltransferase
**SGO_0448**	1.496	5.978	0.0028	0.0078	0.170	5.137	0.1330	0.6801	-1.036	4.384	0.0375	0.3180	-1.326	5.495	0.0019	0.0046	-2.532	4.521	0.0206	0.1025	-1.206	3.960	0.0358	0.0691	conserved hypothetical protein TIGR00488
**SGO_0449**		1.585				1.585				1.585															isochorismatase family protein
**SGO_0450**	1.587	4.981	0.0111	0.0443	1.018	4.508	0.0199	0.0773		2.170			-0.570	4.989	0.0013	0.0029		3.635				3.677			iojap-related protein
**SGO_0451**						3.791								3.278								3.278			methyltransferase
**SGO_0453**		7.985				7.985				7.985															lipoprotein, putative
**SGO_0454**	0.166	7.715	0.0699	0.4277	0.236	7.740	0.0327	0.1365	-0.263	7.542	0.0681	0.6242	0.070	7.311	0.1040	0.7740	-0.429	6.514	0.0563	0.3573	-0.499	7.151	0.0878	0.2397	conserved hypothetical protein TIGR01033
**SGO_0455**	-5.504	8.234	0.0133	0.0563	-2.675	8.428	0.0004	0.0005	-3.589	8.338	0.0003	0.0004	2.829	5.127	0.0111	0.0532	1.915	3.733	0.0398	0.2313	-0.914	5.663	0.0166	0.0204	lipoprotein, putative
**SGO_0456**	1.256	5.488	0.0003	0.0003		3.732				3.732				4.469				3.858							ILL5; amino acid aminohydrolase
**SGO_0457**	-3.171	9.200	0.0001	0.0000	-1.812	9.410	0.0002	0.0001	-1.713	9.432	0.0001	0.0001	1.359	7.198	0.0002	0.0001	1.458	6.659	0.0001	0.0000	0.099	7.774	0.0397	0.0810	ABC transporter, substrate-binding protein SP0148
**SGO_0458**	-2.885	10.776	0.0004	0.0004	-1.995	10.913	0.0005	0.0005	-1.927	10.927	0.0003	0.0006	0.889	8.708	0.0015	0.0034	0.958	8.142	0.0015	0.0029	0.069	9.113	0.0419	0.0868	hlpA; lipoprotein

Log$_2$ Ratios

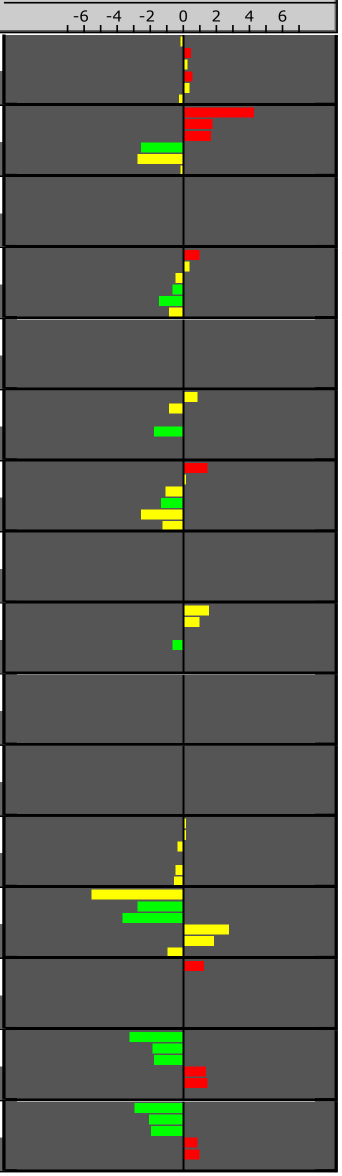

◉ Show detected proteins only
○ Show all proteins
☐ Filter by category:
ABC Transporter

Proteins found:
1179

Dot Plots
Dot Plots

Test	Cutoff
***q*-Value**	**.005**
p-Value	

	Signif	Direction	Applies To
red	yes	+	ratios, bars
yellow	no	n/a	bars
green	yes	–	ratios, bars
pink	yes	+	*p*- , *q*-Values
light green	yes	–	

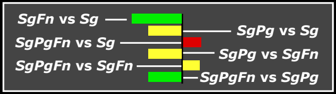

Hendrickson *et al.*

Streptococcus gordonii

Protein	Description	*SgFn vs Sg* Ratio	Sum	*q*-Val	*p*-Val	*SgPg vs Sg* Ratio	Sum	*q*-Val	*p*-Val	*SgPgFn vs Sg* Ratio	Sum	*q*-Val	*p*-Val
SGO_0459	succinyl-diaminopimelate desuccinylase	0.112	5.970	0.1341	0.8978	-1.190	5.463	0.0169	0.0627		4.950		
SGO_0460	ABC transporter, ATP-binding protein SP0151	-2.640	7.625	0.0007	0.0010	-2.214	7.691	0.0008	0.0013	-2.331	7.671	0.0005	0.0012
SGO_0463	cydD; putative ABC transporter (ATP-binding protein)		2.723				2.723				2.723		
SGO_0468	hypothetical protein SGO_0468	1.460	7.214	0.0095	0.0367	-0.141	6.233	0.1049	0.5199	-0.209	6.214	0.0553	0.4919
SGO_0469	integral membrane protein		4.352				4.352				4.352		
SGO_0476	rhodanese family protein	-1.320	4.817	0.0152	0.0670	1.201	6.277	0.0008	0.0012	1.297	6.342	0.0002	0.0002
SGO_0480	hypothetical protein SGO_0480	6.692	9.969	0.0405	0.2217	2.150	5.482	0.0231	0.0917	0.737	3.585		
SGO_0483	hypothetical protein SGO_0483	0.666	6.247	0.0052	0.0168	0.911	6.419	0.0108	0.0362	0.979	6.445	0.0011	0.0035
SGO_0484	sensor histidine kinase	-0.367	4.981	0.0680	0.4142		4.515				4.515		
SGO_0488	ABC transporter, ATP-binding protein SP0483	-2.214	6.635	0.0018	0.0041	-2.054	6.608	0.0002	0.0001		6.297		
SGO_0491	gidB; methyltransferase GidB		4.342				3.343						
SGO_0494	lemA; LemA-like protein	3.418	8.065	0.0010	0.0018	1.064	6.136	0.0009	0.0013		4.516		
SGO_0495	htpx; heat shock protein	0.441	5.691	0.0257	0.1271	-0.397	4.932	0.0987	0.4863		4.481		
SGO_0497	gtfG; glucosyltransferase G		9.226								1.981		
SGO_0500	rggD; putative transcriptional regulator RggD		2.058				2.058				2.058		
SGO_0501	Uncharacterized ACR, COG1399	-1.705	5.727	0.0055	0.0183	0.653	6.885	0.0004	0.0004	0.967	7.084	0.0000	0.0000

Protein	*SgPg vs SgFn* Ratio	Sum	*q*-Val	*p*-Val	*SgPgFn vs SgFn* Ratio	Sum	*q*-Val	*p*-Val	*SgPgFn vs SgPg* Ratio	Sum	*q*-Val	*p*-Val
SGO_0459	-1.303	4.977	0.0023	0.0064		3.866				3.209		
SGO_0460	0.425	5.479	0.0025	0.0072	0.308	4.801	0.0014	0.0024	-0.117	5.619	0.0524	0.1210
SGO_0463												
SGO_0468	-1.602	6.634	0.0051	0.0196	-1.669	6.013	0.0058	0.0201	-0.067	5.563	0.2181	0.8046
SGO_0469												
SGO_0476	2.521	5.363	0.0119	0.0583	2.617	4.838	0.0082	0.0313	0.096	6.290	0.0979	0.2804
SGO_0480	-4.542	9.506	0.0050	0.0191	-5.955	8.849	0.0388	0.2230	-1.413	5.063	0.0499	0.1131
SGO_0483	0.245	6.162	0.0367	0.2361	0.313	5.573	0.0011	0.0015	0.069	6.312	0.2214	0.8252
SGO_0484		2.611				2.000						
SGO_0488	0.160	4.799	0.1077	0.8086		3.254				3.730		
SGO_0491	-1.048	4.414	0.0033	0.0108		3.218				2.830		
SGO_0494	-2.354	7.679	0.0013	0.0025		6.813				5.055		
SGO_0495	-0.838	4.717	0.0118	0.0577		3.751				2.523		
SGO_0497		8.713			-6.245	8.111	0.0022	0.0054		1.468		
SGO_0500												
SGO_0501	2.358	5.796	0.0062	0.0253	2.672	5.473	0.0013	0.0023	0.314	6.827	0.0037	0.0010

Log$_2$ Ratios: -6 -4 -2 0 2 4 6

Show detected proteins only
Show all proteins
Filter by category:
ABC Transporter

Proteins found: 1179

Dot Plots
Dot Plots

Test	Cutoff
q-Value	.005
p-Value	

	Signif	Direction	Applies To
red	yes	+	ratios, bars
yellow	no	n/a	bars
green	yes	–	ratios, bars
pink	yes	+	*p*- , *q*-Values
light green	yes	–	

SgFn vs Sg; SgPgFn vs Sg; SgPgFn vs SgFn; SgPg vs Sg; SgPg vs SgFn; SgPgFn vs SgPg

Hendrickson *et al.*

Streptococcus gordonii

Protein	*SgFn vs Sg* Ratio	Sum	*q*-Val	*p*-Val	*SgPg vs Sg* Ratio	Sum	*q*-Val	*p*-Val	*SgPgFn vs Sg* Ratio	Sum	*q*-Val	*p*-Val	*SgPg vs SgFn* Ratio	Sum	*q*-Val	*p*-Val	*SgPgFn vs SgFn* Ratio	Sum	*q*-Val	*p*-Val	*SgPgFn vs SgPg* Ratio	Sum	*q*-Val	*p*-Val
**SGO_0502**	-1.901	9.535	0.0003	0.0003	-0.710	9.878	0.0016	0.0032	-1.046	9.757	0.0002	0.0002	1.192	8.502	0.0023	0.0064	0.856	7.662	0.0028	0.0076	-0.336	8.810	0.0263	0.0431
	floL; flotillin-like protein																							
**SGO_0503**	1.705	11.551	0.0001	0.0001	0.873	10.962	0.0003	0.0002	1.201	11.183	0.0002	0.0003	-0.832	11.295	0.0004	0.0003	-0.504	10.811	0.0012	0.0018	0.328	10.994	0.0086	0.0064
	gnd; 6-phosphogluconate dehydrogenase, decarboxylating																							
**SGO_0505**	-3.594	10.971	0.0001	0.0000	-3.384	10.986	0.0001	0.0000	-3.763	10.957	0.0001	0.0000	0.210	7.872	0.0512	0.3479	-0.169	7.069	0.0565	0.3604	-0.379	7.786	0.0264	0.0435
	PTS system, IIBC component																							
**SGO_0506**		3.000			-1.165	3.532				3.000				1.322								1.322		
	rgfB; RgfB																							
**SGO_0508**	-0.432	6.099	0.0118	0.0489	-0.519	6.409	0.0010	0.0016	-0.317	6.498	0.0061	0.0338	-0.087	5.228	0.0712	0.5085	0.116	4.756	0.0856	0.5856	0.202	5.722	0.0616	0.1493
	nrdR; transcriptional regulator, NrdR family																							
**SGO_0509**		4.188			-1.245	4.446	0.0735	0.3492		4.188				1.322								1.322		
	Replication initiation and membrane attachment protein (DnaB) superfamily																							
**SGO_0510**	1.340	5.852	0.0052	0.0170	1.688	6.091	0.0010	0.0015	1.171	5.714	0.0011	0.0038	0.348	6.032	0.0275	0.1659	-0.169	5.157	0.0612	0.3971	-0.517	5.948	0.0158	0.0183
	dnaI; primosomal protein DnaI																							
**SGO_0511**	1.306	5.805	0.0113	0.0460	0.194	5.117	0.1773	0.9470	0.216	5.138	0.0919	0.8722	-1.112	5.300	0.0054	0.0209	-1.090	4.703	0.0072	0.0263	0.022	4.638	0.2139	0.7868
	NADPH-flavin oxidoreductase -like protein																							
**SGO_0512**	0.201	7.827	0.0613	0.3659	0.530	8.008	0.0100	0.0333	0.771	8.160	0.0043	0.0222	0.329	7.571	0.0043	0.0149	0.571	7.105	0.0060	0.0210	0.241	7.855	0.0443	0.0950
	GTP-binding protein engA																							
**SGO_0513**	0.668	4.254	0.0524	0.3005		2.170				2.170				3.353				2.742						
	Snf2 family protein																							
**SGO_0514**						3.791								3.278								3.278		
	hypothetical protein SGO_0514																							
**SGO_0515**	0.612	8.759	0.0031	0.0092	0.105	8.471	0.0142	0.0507	0.490	8.689	0.0047	0.0250	-0.507	8.288	0.0038	0.0126	-0.122	7.853	0.0635	0.4155	0.385	8.219	0.0246	0.0388
	murC; UDP-N-acetylmuramate--alanine ligase																							
**SGO_0518**	-2.664	8.637	0.0001	0.0000	-1.438	8.879	0.0001	0.0001	-1.560	8.849	0.0002	0.0001	1.227	6.988	0.0002	0.0001	1.104	6.297	0.0015	0.0027	-0.123	7.418	0.0956	0.2702
	aminodeoxychorismate lyase-like protein																							
**SGO_0519**	0.050	7.158	0.1070	0.6966	0.526	7.415	0.0087	0.0280	0.177	7.214	0.0112	0.0744	0.476	6.929	0.0132	0.0679	0.127	6.121	0.0722	0.4804	-0.349	6.975	0.0315	0.0556
	greA; transcription elongation factor greA																							
**SGO_0521**	-0.573	5.942	0.0557	0.3239	-1.082	6.092	0.0080	0.0248		5.531			-0.509	4.706	0.0488	0.3295		2.807				3.945		
	Membrane protein oxaA 2 precursor																							
**SGO_0523**	2.159	6.704	0.0005	0.0007	0.164	5.107	0.1254	0.6344		3.392			-1.995	6.366	0.0002	0.0001		5.427				4.070		
	spoU rRNA Methylase family protein																							

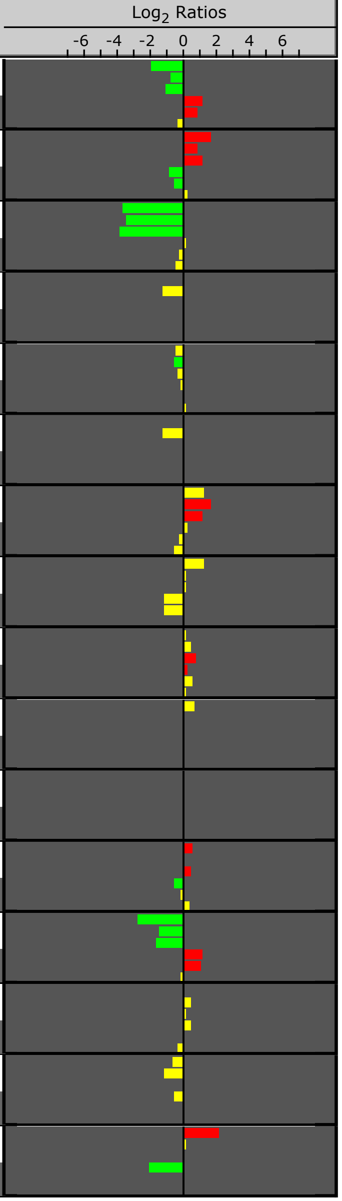

Show detected proteins only
Show all proteins
Filter by category:
ABC Transporter

Proteins found:
1179

Dot Plots
Dot Plots

Test	Cutoff
q-Value	.005
p-Value	

	Signif	Direction	Applies To
(red)	yes	+	ratios, bars
(yellow)	no	n/a	bars
(green)	yes	–	ratios, bars
(pink)	yes	+	*p*- , *q*-Values
(light green)	yes	–	

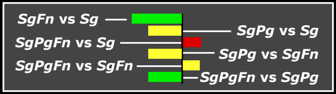

Hendrickson *et al.*

Streptococcus gordonii

Protein	Description	*SgFn vs Sg* Ratio	Sum	*q*-Val	*p*-Val	*SgPg vs Sg* Ratio	Sum	*q*-Val	*p*-Val	*SgPgFn vs Sg* Ratio	Sum	*q*-Val	*p*-Val	*SgPg vs SgFn* Ratio	Sum	*q*-Val	*p*-Val	*SgPgFn vs SgFn* Ratio	Sum	*q*-Val	*p*-Val	*SgPgFn vs SgPg* Ratio	Sum	*q*-Val	*p*-Val
**SGO_0526**	ilvB; acetolactate synthase, large subunit, biosynthetic type	0.727	6.089	0.0277	0.1410	0.991	6.225	0.0078	0.0239	-0.429	5.467	0.0284	0.2290	0.264	5.998	0.0611	0.4254	-1.156	4.798	0.0101	0.0413	-1.420	5.561	0.0050	0.0022
**SGO_0527**	ilvN; acetolactate synthase, small subunit	0.008	5.759	0.1306	0.8672	-0.395	5.587	0.0288	0.1179	-1.584	4.978	0.0063	0.0350	-0.403	5.078	0.0196	0.1122	-1.592	3.861	0.0089	0.0352	-1.189	4.162	0.0867	0.2343
**SGO_0528**	ilvC; ketol-acid reductoisomerase	1.026	8.815	0.0020	0.0049	0.581	8.528	0.0018	0.0039	0.292	8.366	0.0128	0.0887	-0.445	8.520	0.0078	0.0339	-0.734	7.797	0.0048	0.0157	-0.289	8.143	0.0423	0.0885
**SGO_0529**	ilvA; threonine dehydratase	1.907	5.080	0.0065	0.0226	1.401	4.686	0.0093	0.0302	1.149	3.915	0.0332	0.2748	-0.507	4.959	0.0259	0.1541	-0.759	3.952	0.0542	0.3391	-0.252	4.168	0.1566	0.5093
**SGO_0530**	Cof family protein	-0.427	5.304	0.0251	0.1236	-1.277	5.023	0.0059	0.0174		4.486			-0.849	4.253	0.0220	0.1276		2.972				2.826		
**SGO_0533**	conserved hypothetical protein TIGR00150	2.481	3.362			0.871	2.143				0.643			-1.611	3.019				2.000				1.000		
**SGO_0535**	putative transcriptional regulator LytR	-2.494	6.747	0.0178	0.0800	-2.237	6.905	0.0008	0.0011	-2.636	6.736	0.0117	0.0784	0.257	4.384	0.0652	0.4574	-0.143	2.930			-0.400	4.343	0.1153	0.3452
**SGO_0536**	hypothetical protein SGO_0536		6.082			-0.177	6.995	0.0193	0.0745	0.019	7.090	0.0847	0.7979		5.390				4.975			0.196	6.491	0.0198	0.0277
**SGO_0537**	HIT family protein	1.242	6.570	0.0008	0.0013	1.124	6.487	0.0008	0.0011	0.678	6.200	0.0029	0.0130	-0.118	6.485	0.0126	0.0637	-0.564	5.678	0.0016	0.0033	-0.446	6.219	0.0051	0.0025
**SGO_0538**	ABC transporter, ATP-binding protein SP0522	-0.908	6.861	0.0075	0.0275	-2.541	6.463	0.0012	0.0020		6.232			-1.633	5.245	0.0054	0.0210		4.236				3.195		
**SGO_0539**	ABC transporter, permease protein		4.667				4.667				4.667														
**SGO_0540**	hypothetical protein SGO_0540	1.134	7.814	0.0003	0.0003	0.148	7.214	0.0131	0.0456	-0.100	7.089	0.0204	0.1589	-0.986	7.348	0.0004	0.0003	-1.234	6.659	0.0004	0.0002	-0.248	6.654	0.0094	0.0078
**SGO_0541**	methyltransferase, putative	-0.545	5.797	0.0064	0.0223	-1.602	5.480	0.0030	0.0073		5.047			-1.057	4.580	0.0057	0.0224		3.371				3.020		
**SGO_0542**	hypothetical protein SGO_0542		2.700			-0.865	3.332				2.700				1.322								1.322		
**SGO_0543**	nusA; transcription termination factor NusA	-1.692	7.929	0.0001	0.0000	-0.585	8.276	0.0002	0.0001	-0.940	8.146	0.0003	0.0004	1.107	6.991	0.0000	0.0000	0.752	6.151	0.0016	0.0032	-0.355	7.276	0.0105	0.0098
**SGO_0544**	Protein of unknown function (DUF448) superfamily		2.000				2.000				2.000														

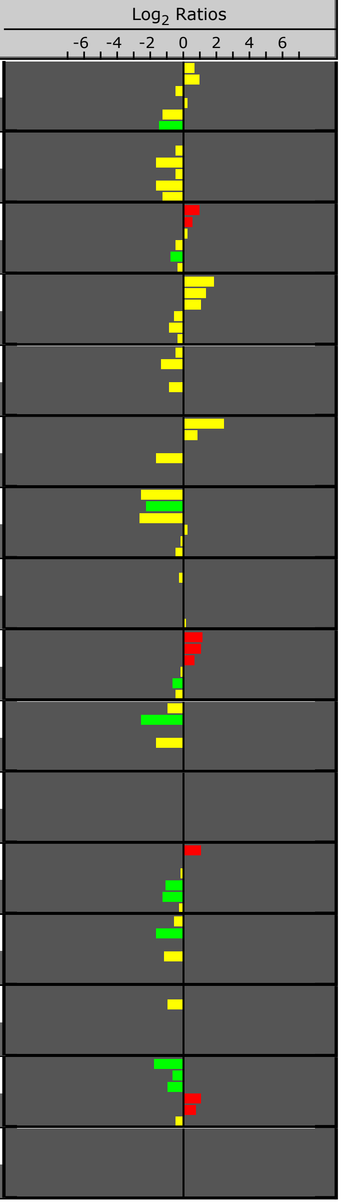

Show detected proteins only
Show all proteins
Filter by category:
ABC Transporter

Proteins found:
1179

Dot Plots
Dot Plots

Test	Cutoff
q-Value	.005
p-Value	

	Signif	Direction	Applies To
(red)	yes	+	ratios, bars
(yellow)	no	n/a	bars
(green)	yes	–	ratios, bars
(light red)	yes	+	*p*- , *q*-Values
(light green)	yes	–	

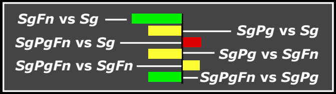

Hendrickson *et al.*

Protein	SgFn vs Sg Ratio	SgFn vs Sg Sum	SgFn vs Sg *q*-Val	SgFn vs Sg *p*-Val	SgPg vs Sg Ratio	SgPg vs Sg Sum	SgPg vs Sg *q*-Val	SgPg vs Sg *p*-Val	SgPgFn vs Sg Ratio	SgPgFn vs Sg Sum	SgPgFn vs Sg *q*-Val	SgPgFn vs Sg *p*-Val	SgPg vs SgFn Ratio	SgPg vs SgFn Sum	SgPg vs SgFn *q*-Val	SgPg vs SgFn *p*-Val	SgPgFn vs SgFn Ratio	SgPgFn vs SgFn Sum	SgPgFn vs SgFn *q*-Val	SgPgFn vs SgFn *p*-Val	SgPgFn vs SgPg Ratio	SgPgFn vs SgPg Sum	SgPgFn vs SgPg *q*-Val	SgPgFn vs SgPg *p*-Val	Description
SGO_0545		1.807				1.807				1.807															ribosomal protein L7A family
SGO_0546	-0.307	8.766	0.0250	0.1222	-0.138	8.842	0.0729	0.3461	-0.475	8.700	0.0122	0.0836	0.170	8.171	0.0265	0.1583	-0.168	7.401	0.0612	0.3987	-0.338	8.102	0.0476	0.1054	infB; Translation initiation factor IF-2
SGO_0547						1.098								0.585								0.585			rbfA; ribosome-binding factor A
SGO_0548	-4.413	7.683	0.0137	0.0590	-2.975	7.822	0.0004	0.0004	-3.700	7.756	0.0003	0.0004	1.438	4.402	0.0127	0.0642	0.714	3.205	0.0101	0.0417	-0.725	4.840	0.0050	0.0019	Na/Pi-cotransporter family protein
SGO_0549	-0.156	2.789			3.756	5.724				1.865			3.912	5.200				0.585				5.108			nagA; N-acetylglucosamine-6-phosphate deacetylase
SGO_0552	3.347	7.019	0.0005	0.0007	2.595	6.355	0.0004	0.0004	2.390	6.185	0.0004	0.0009	-0.752	7.028	0.0022	0.0058	-0.957	6.345	0.0017	0.0037	-0.205	6.502	0.0184	0.0242	oxidoreductase, aldo/keto reductase family
SGO_0554	0.061	6.972	0.1305	0.8645	-0.957	6.566	0.0297	0.1216	-1.664	6.199	0.0395	0.3367	-1.018	6.007	0.0028	0.0083	-1.725	5.015	0.0204	0.1000	-0.707	4.799	0.0921	0.2571	hsdR; type I site-specific deoxyribonuclease
SGO_0556		3.279				3.279				3.279															DNA helicase-like protein, putative
SGO_0557		1.807				1.807				1.807															HsdS specificity protein of type I restriction-modification system
SGO_0558	0.494	3.590			0.356	4.186	0.1015	0.5010	0.263	3.460			-0.137	3.826	0.1100	0.8286	-0.230	2.581			-0.093	3.750	0.2118	0.7732	hypothetical protein SGO_0558
SGO_0560	-1.604	4.640	0.0073	0.0263	0.516	5.703	0.0024	0.0054	-0.051	5.401	0.0810	0.7600	2.120	4.582	0.0118	0.0572	1.553	3.483	0.0247	0.1292	-0.567	5.174	0.0151	0.0173	hsdM; type I restriction-modification system, M subunit
SGO_0565	2.010	9.293	0.0008	0.0013	0.214	8.069	0.0140	0.0500	0.010	7.962	0.0921	0.8777	-1.797	8.825	0.0009	0.0015	-2.000	8.171	0.0010	0.0013	-0.204	7.560	0.0246	0.0387	adhA; alcohol dehydrogenase
SGO_0566	0.575	8.380	0.0109	0.0434		7.073				7.073				7.120				6.510							sgc; serine protease challisin
SGO_0568	-1.401	6.686	0.0013	0.0025	0.047	7.232	0.0949	0.4663	-0.300	7.076	0.0184	0.1403	1.448	6.209	0.0014	0.0031	1.101	5.369	0.0073	0.0268	-0.346	6.590	0.0499	0.1129	glyQ; glycyl-tRNA synthetase, alpha subunit
SGO_0569	0.390	8.405	0.0137	0.0586	0.474	8.445	0.0001	0.0001	0.301	8.362	0.0214	0.1686	0.084	8.115	0.0814	0.5918	-0.089	7.432	0.1037	0.7297	-0.173	8.079	0.1338	0.4182	glyS; glycyl-tRNA synthetase, beta subunit
SGO_0570		3.170			-0.455	4.468	0.0111	0.0375		3.170				3.202								3.202			hypothetical protein SGO_0570

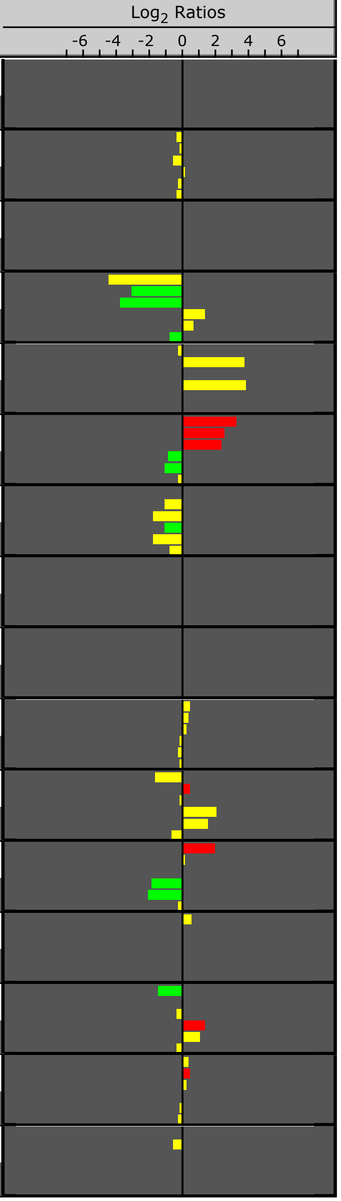

Show detected proteins only
Show all proteins
Filter by category:
ABC Transporter

Proteins found:
1179

Dot Plots
Dot Plots

Test	Cutoff
q-Value	.005
p-Value	

	Signif	Direction	Applies To
red	yes	+	ratios, bars
yellow	no	n/a	bars
green	yes	–	ratios, bars
light red	yes	+	*p*- , *q*-Values
light green	yes	–	

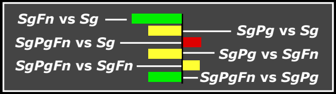

Streptococcus gordonii

Protein	Description	*SgFn vs Sg* Ratio	Sum	*q*-Val	*p*-Val	*SgPg vs Sg* Ratio	Sum	*q*-Val	*p*-Val	*SgPgFn vs Sg* Ratio	Sum	*q*-Val	*p*-Val	*SgPg vs SgFn* Ratio	Sum	*q*-Val	*p*-Val	*SgPgFn vs SgFn* Ratio	Sum	*q*-Val	*p*-Val	*SgPgFn vs SgPg* Ratio	Sum	*q*-Val	*p*-Val
SGO_0571	hypothetical protein SGO_0571		1.322				1.322				1.322														
SGO_0573	mraW; S-adenosyl-methyltransferase MraW	0.013	6.729	0.1339	0.8953	-0.163	6.619	0.0727	0.3439	-1.240	6.233	0.0046	0.0239	-0.176	6.126	0.0588	0.4067	-1.254	5.136	0.0101	0.0414	-1.078	5.593	0.0120	0.0121
SGO_0575	pbp2X; penicillin-binding protein 2X		6.904			-3.188	7.054	0.0001	0.0001		6.904				3.202								3.202		
SGO_0576	mraY; phospho-N-acetylmuramoyl-pentapeptide-transferase		3.087				3.087				3.087														
SGO_0577	ATP-dependent RNA helicase	-0.084	6.915	0.1306	0.8687	0.720	7.329	0.0064	0.0189	0.118	7.003	0.0712	0.6568	0.804	6.796	0.0075	0.0319	0.202	5.854	0.0868	0.5957	-0.602	6.863	0.0238	0.0368
SGO_0578	amino acid ABC transporter permease protein		6.489				6.489				6.489														
SGO_0579	amino acid ABC transporter ATP binding protein	-3.048	6.975	0.0001	0.0000	-2.330	7.076	0.0002	0.0001		6.811			0.718	4.668	0.0075	0.0323		2.639				3.991		
SGO_0581	trxB; thioredoxin-disulfide reductase	-0.401	5.554	0.0381	0.2059	0.719	6.131	0.0084	0.0264	-0.136	5.652	0.0353	0.2946	1.121	5.490	0.0046	0.0168	0.265	4.348	0.0563	0.3569	-0.856	5.563	0.0121	0.0124
SGO_0582	nicotinate phosphoribosyltransferase, putative	3.236	8.876	0.0000	0.0000	1.890	7.748	0.0025	0.0058	1.063	7.143	0.0042	0.0212	-1.346	8.699	0.0006	0.0007	-2.174	7.897	0.0002	0.0001	-0.827	7.536	0.0224	0.0331
SGO_0583	nadE; NAD+ synthetase	2.312	6.694	0.0022	0.0058	1.345	5.936	0.0033	0.0083	0.732	5.536	0.0130	0.0915	-0.967	6.490	0.0045	0.0163	-1.580	5.703	0.0027	0.0071	-0.613	5.640	0.0045	0.0014
SGO_0585	pepC; aminopeptidase C	2.877	8.679	0.0002	0.0002	0.921	7.164	0.0065	0.0193	0.528	6.916	0.0092	0.0570	-1.956	8.313	0.0004	0.0004	-2.349	7.628	0.0004	0.0002	-0.393	6.850	0.0426	0.0898
SGO_0586	pbp1a; penicillin-binding protein 1A	-1.742	8.755	0.0002	0.0001	-1.402	8.842	0.0003	0.0003	-1.931	8.713	0.0001	0.0001	0.341	7.309	0.0144	0.0754	-0.188	6.424	0.0303	0.1667	-0.529	7.227	0.0163	0.0197
SGO_0589	methylase	1.676	7.845	0.0038	0.0113	1.105	7.429	0.0068	0.0204	0.840	7.241	0.0028	0.0124	-0.571	7.688	0.0196	0.1119	-0.835	6.970	0.0101	0.0417	-0.265	7.239	0.0979	0.2806
SGO_0590	Methyltransferase	0.366	6.294	0.0250	0.1225	-0.529	5.876	0.0308	0.1271	-1.104	5.408	0.0263	0.2109	-0.895	5.577	0.0044	0.0159	-1.470	4.571	0.0188	0.0905	-0.575	4.491	0.1229	0.3763
SGO_0591	hypothetical protein SGO_0591	-2.414	8.988	0.0000	0.0000	-2.495	8.975	0.0000	0.0000	-3.040	8.906	0.0000	0.0000	-0.081	6.773	0.0005	0.0005	-0.626	5.925	0.0013	0.0021	-0.545	6.487	0.0058	0.0034
SGO_0592	luxS; autoinducer-2 production protein LuxS	0.770	5.593	0.0015	0.0034	1.171	5.856	0.0003	0.0002	0.243	4.825	0.0276	0.2220	0.401	5.626	0.0027	0.0078	-0.527	4.232	0.0281	0.1510	-0.928	5.148	0.0264	0.0438

Log$_2$ Ratios

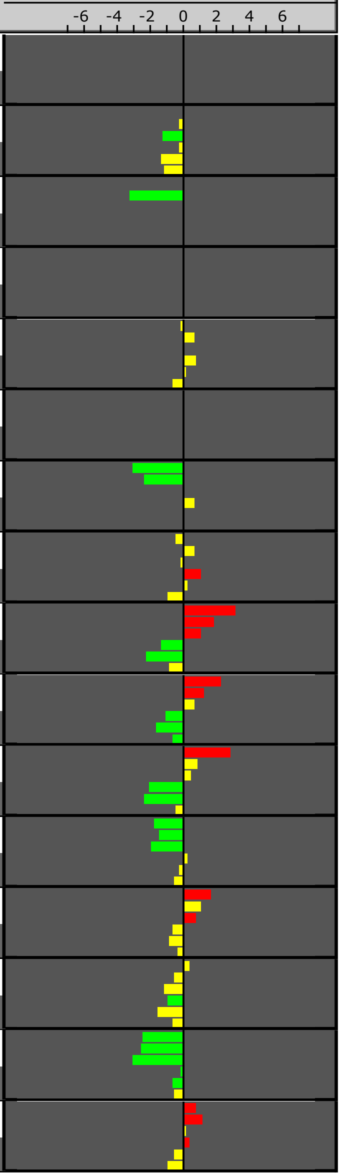

Show detected proteins only
Show all proteins
Filter by category:
ABC Transporter

Proteins found:
1179

Dot Plots
Dot Plots

Test	Cutoff
q-Value	.005
p-Value	

	Signif	Direction	Applies To
red	yes	+	ratios, bars
yellow	no	n/a	bars
green	yes	–	ratios, bars
light red	yes	+	*p*- , *q*-Values
light green	yes	–	

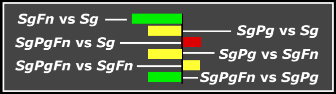

Hendrickson *et al.*

Protein	*SgFn vs Sg* Ratio	Sum	*q*-Val	*p*-Val	*SgPg vs Sg* Ratio	Sum	*q*-Val	*p*-Val	*SgPgFn vs Sg* Ratio	Sum	*q*-Val	*p*-Val	*SgPg vs SgFn* Ratio	Sum	*q*-Val	*p*-Val	*SgPgFn vs SgFn* Ratio	Sum	*q*-Val	*p*-Val	*SgPgFn vs SgPg* Ratio	Sum	*q*-Val	*p*-Val
**SGO_0593**	-3.236	8.876	0.0001	0.0000	-2.047	9.043	0.0002	0.0001	-2.992	8.903	0.0001	0.0001	1.189	6.701	0.0013	0.0027	0.245	5.513	0.0449	0.2657	-0.945	6.782	0.0089	0.0068
HD/KH domain protein																								
**SGO_0594**	0.562	6.622	0.0052	0.0169	-0.071	6.280	0.1194	0.6001	-0.356	6.147	0.0097	0.0616	-0.633	6.078	0.0028	0.0086	-0.918	5.361	0.0014	0.0025	-0.285	5.592	0.0336	0.0621
gmk; Guanylate kinase (GMP kinase)																								
**SGO_0595**	-1.365	6.899	0.0004	0.0006	-0.368	7.248	0.0003	0.0002	-0.733	7.101	0.0004	0.0006	0.997	6.131	0.0011	0.0021	0.632	5.291	0.0054	0.0183	-0.365	6.369	0.0086	0.0063
DNA-directed RNA polymerase, omega subunit																								
**SGO_0596**	1.535	4.408	0.0093	0.0359		1.643				1.643				3.665				3.054						
priA; primosomal protein N''																								
**SGO_0597**	2.095	6.308	0.0012	0.0024	1.413	5.785	0.0025	0.0057	0.482	5.192	0.0290	0.2349	-0.682	6.173	0.0032	0.0104	-1.613	5.275	0.0013	0.0022	-0.930	5.400	0.0057	0.0031
fmt; methionyl-tRNA formyltransferase																								
**SGO_0598**	0.617	4.435	0.0067	0.0238	0.767	5.065	0.0098	0.0324		3.614			0.150	4.423	0.0894	0.6563		2.107				3.895		
sun; sun protein																								
**SGO_0599**	1.085	7.939	0.0005	0.0006	-0.624	7.019	0.0012	0.0022	-0.816	6.954	0.0019	0.0077	-1.709	7.255	0.0004	0.0003	-1.901	6.606	0.0006	0.0005	-0.192	6.080	0.1225	0.3743
phosphoprotein phosphatase																								
**SGO_0600**	-2.104	8.114	0.0001	0.0001	-2.409	8.059	0.0001	0.0000	-3.243	7.883	0.0037	0.0182	-0.306	6.059	0.0240	0.1406	-1.139	4.891	0.0315	0.1751	-0.833	5.248	0.0549	0.1291
serine/threonine protein kinase																								
**SGO_0602**		4.115				4.115				4.115														
histidine kinase																								
**SGO_0603**	0.459	5.665	0.0023	0.0062	-0.916	5.074	0.0148	0.0537		4.419			-1.375	4.866	0.0031	0.0096		3.751				3.106		
response regulator																								
**SGO_0604**	1.785	8.952	0.0001	0.0001	0.579	8.119	0.0028	0.0068	0.298	7.959	0.0102	0.0659	-1.206	8.588	0.0001	0.0000	-1.487	7.899	0.0001	0.0000	-0.281	7.730	0.0121	0.0124
hydrolase, haloacid dehalogenase family/peptidyl-prolyl cis-trans isomerase, cyclophilin type																								
**SGO_0606**	-0.606	9.178	0.0004	0.0005	-0.319	9.299	0.0034	0.0086	-0.436	9.248	0.0006	0.0014	0.287	8.481	0.0032	0.0103	0.171	7.806	0.0019	0.0043	-0.116	8.560	0.0452	0.0978
cysK; cysteine synthase A																								
**SGO_0608**		4.069				4.069				4.069														
comFA; competence ComFA-like protein																								
**SGO_0610**	1.162	8.870	0.0022	0.0058	0.727	8.578	0.0016	0.0031	0.525	8.478	0.0148	0.1065	-0.434	8.621	0.0095	0.0444	-0.637	7.943	0.0136	0.0613	-0.202	8.303	0.1450	0.4632
ribosomal subunit interface protein																								
**SGO_0626**	-1.694	5.610	0.0047	0.0149	-1.293	5.901	0.0004	0.0005	-1.685	5.612	0.0032	0.0150	0.400	4.071	0.0495	0.3347	0.009	2.590			-0.391	4.073	0.1141	0.3401
recX; Regulatory protein recX																								
**SGO_0628**		1.322				1.322				1.322														
ymdC; 3''-aminoglycoside phosphotransferase-like protein (kanamycin kinase)																								

Log$_2$ Ratios

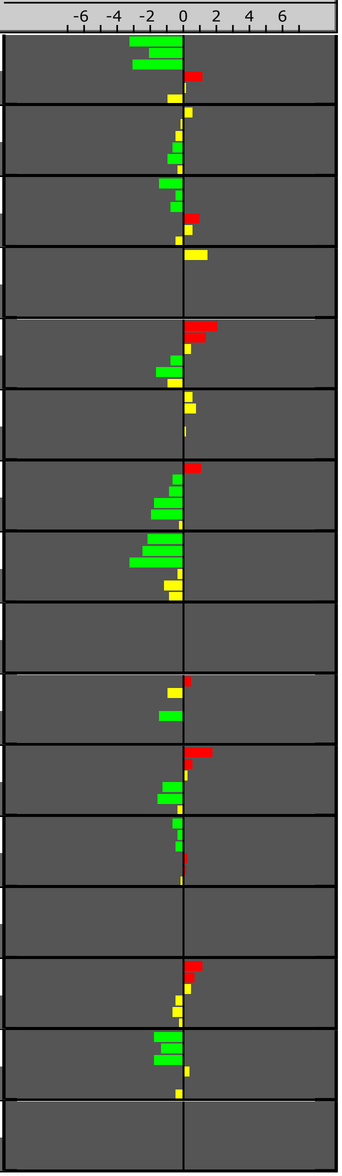

Show detected proteins only
Show all proteins
Filter by category:
ABC Transporter

Proteins found:
1179

Dot Plots
Dot Plots

Test	Cutoff
***q*-Value**	**.005**
p-Value	

	Signif	Direction	Applies To
(red)	yes	+	ratios, bars
(yellow)	no	n/a	bars
(green)	yes	–	ratios, bars
(light red)	yes	+	*p*- , *q*-Values
(light green)	yes	–	*p*- , *q*-Values

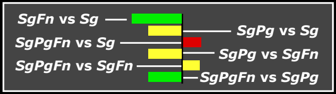

Hendrickson *et al.*

Streptococcus gordonii

	SgFn vs Sg				*SgPg vs Sg*				*SgPgFn vs Sg*				*SgPg vs SgFn*				*SgPgFn vs SgFn*				*SgPgFn vs SgPg*			
Protein	Ratio	Sum	*q*-Val	*p*-Val	Ratio	Sum	*q*-Val	*p*-Val	Ratio	Sum	*q*-Val	*p*-Val	Ratio	Sum	*q*-Val	*p*-Val	Ratio	Sum	*q*-Val	*p*-Val	Ratio	Sum	*q*-Val	*p*-Val
**SGO_0629**		3.342			-0.706	3.714	0.0791	0.3819		3.342				1.063								1.063		
	rumA-1; 23S rRNA (uracil-5-)-methyltransferase RumA																							
**SGO_0631**	0.803	3.099			3.710	6.407	0.0052	0.0151	2.433	5.206	0.0084	0.0510	2.906	5.932	0.0044	0.0161	1.630	4.171	0.0136	0.0613	-1.276	6.339	0.0012	0.0001
	alpha-glycerophosphate oxidase																							
**SGO_0632**						5.243				4.816				4.730				3.692			-0.428	5.532	0.0101	0.0091
	glpK; glycerol kinase																							
**SGO_0635**	1.553	3.976				2.000				2.000				3.039				2.429						
	hypothetical protein SGO_0635																							
**SGO_0636**		2.930				2.930				2.930														
	hypothetical protein SGO_0636																							
**SGO_0639**	0.072	8.074	0.0676	0.4110	0.402	8.251	0.0020	0.0044	-0.081	8.001	0.0655	0.5956	0.330	7.771	0.0060	0.0237	-0.153	6.917	0.0555	0.3511	-0.483	7.708	0.0168	0.0211
	valS; valyl-tRNA synthetase																							
**SGO_0640**		3.285			-0.116	4.207	0.1194	0.5995	2.061	4.870	0.0095	0.0595		2.612				3.162			2.176	4.306	0.0195	0.0266
	modification methylase																							
**SGO_0641**	0.022	6.939	0.1313	0.8744	0.103	6.975	0.0807	0.3916	-0.590	6.661	0.0043	0.0221	0.081	6.473	0.0774	0.5594	-0.612	5.552	0.0071	0.0258	-0.693	6.206	0.0057	0.0032
	ATPase, histidine kinase-, DNA gyrase B-, and HSP90-like domain protein protein																							
**SGO_0642**	-1.789	6.945	0.0020	0.0050	-1.357	7.044	0.0027	0.0063	1.284	8.338	0.0006	0.0014	0.432	5.530	0.0425	0.2821	3.073	6.884	0.0005	0.0003	2.641	7.544	0.0019	0.0003
	hypothetical protein SGO_0642																							
**SGO_0643**	0.835	5.351	0.0028	0.0078	-0.367	4.720	0.0490	0.2176	-0.469	4.323	0.0371	0.3127	-1.201	4.722	0.0021	0.0051	-1.303	3.846	0.0181	0.0853	-0.102	3.563	0.2074	0.7545
	cytosine-specific methyltransferase																							
**SGO_0644**	-0.099	5.966	0.0866	0.5505	1.368	6.862	0.0033	0.0081	0.731	6.424	0.0024	0.0102	1.467	6.326	0.0028	0.0083	0.830	5.269	0.0045	0.0138	-0.638	6.594	0.0262	0.0428
	hypothetical protein SGO_0644																							
**SGO_0649**		2.322				2.322				2.322														
	hypothetical protein SGO_0649																							
**SGO_0652**	-3.825	7.262	0.0001	0.0000	-3.768	7.285	0.0002	0.0001	-3.269	7.309	0.0001	0.0000	0.057	3.991	0.0810	0.5877	0.556	3.540	0.0164	0.0761	0.499	4.282	0.1697	0.5840
	hypothetical protein SGO_0652																							
**SGO_0653**		3.322			-0.400	4.660	0.0528	0.2379		3.322				3.420								3.420		
	conserved hypothetical protein of unknown function (DUF1027)																							
**SGO_0654**	-3.313	7.518	0.0183	0.0826	-0.815	8.095	0.0039	0.0102	-1.356	7.923	0.0011	0.0036	2.498	6.237	0.0141	0.0739	1.957	5.141	0.0205	0.1015	-0.541	6.872	0.0183	0.0237
	radical SAM enzyme, Cfr family																							
**SGO_0656**	0.786	6.720	0.0347	0.1846	-0.846	5.780	0.0012	0.0020	-0.917	5.799	0.0092	0.0574	-1.633	5.978	0.0164	0.0904	-1.703	5.379	0.0202	0.0985	-0.070	4.817	0.2354	0.8955
	trpB-2; tryptophan synthase, beta subunit																							

Log$_2$ Ratios

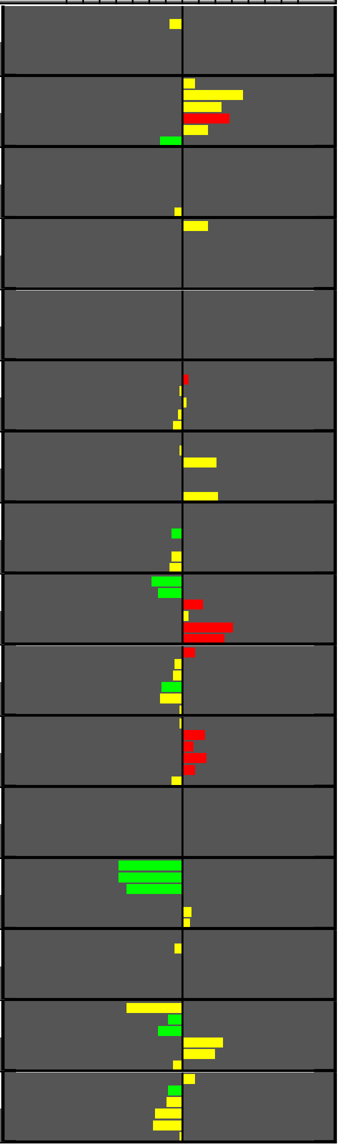

Show detected proteins only
Show all proteins
Filter by category:
ABC Transporter

Proteins found:
1179

Dot Plots
Dot Plots

Test	Cutoff
q-Value	.005
p-Value	

	Signif	Direction	Applies To
red	yes	+	ratios, bars
yellow	no	n/a	bars
green	yes	–	ratios, bars
light red	yes	+	*p*- , *q*-Values
light green	yes	–	

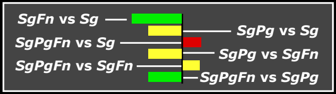

Hendrickson *et al.*

Protein	Description	*SgFn vs Sg* Ratio	Sum	*q*-Val	*p*-Val	*SgPg vs Sg* Ratio	Sum	*q*-Val	*p*-Val	*SgPgFn vs Sg* Ratio	Sum	*q*-Val	*p*-Val	*SgPg vs SgFn* Ratio	Sum	*q*-Val	*p*-Val	*SgPgFn vs SgFn* Ratio	Sum	*q*-Val	*p*-Val	*SgPgFn vs SgPg* Ratio	Sum	*q*-Val	*p*-Val
SGO_0657	trpE; anthranilate synthase component I		1.322				1.322				1.322														
SGO_0662	trpB-1; tryptophan synthase, beta subunit	1.838	4.612	0.0045	0.0139		1.585				1.585				3.910				3.299						
SGO_0663	trpA-2; tryptophan synthase, alpha subunit		1.000				1.000				1.000														
SGO_0665	non-heme iron-containing ferritin	-0.806	10.143	0.0079	0.0292	-0.422	10.290	0.0218	0.0858	-0.506	10.265	0.0144	0.1037	0.384	9.366	0.0119	0.0585	0.300	8.721	0.0397	0.2299	-0.084	9.511	0.2049	0.7441
SGO_0667	rhodanese family protein		4.807			-1.961	5.142	0.0011	0.0019		4.807				2.360								2.360		
SGO_0669	typA; GTP-binding protein TypA	0.790	8.186	0.0019	0.0045	0.694	8.122	0.0002	0.0001	0.640	8.090	0.0006	0.0015	-0.097	7.966	0.0429	0.2855	-0.151	7.331	0.0325	0.1814	-0.054	7.888	0.0984	0.2826
SGO_0671	murD; UDP-N-acetylmuramoylalanine--D-glutamate ligase	1.635	7.976	0.0003	0.0004	0.437	7.176	0.0084	0.0267	-0.744	6.613	0.0005	0.0010	-1.199	7.584	0.0008	0.0012	-2.380	6.703	0.0004	0.0002	-1.181	6.393	0.0058	0.0033
SGO_0672	murG; undecaprenyl-PP-MurNAc-pentapeptide-UDPGlcNAc GlcNAc transferase	-0.119	7.180	0.0814	0.5114	-1.494	6.719	0.0073	0.0224	-3.241	6.316	0.0161	0.1181	-1.375	6.114	0.0055	0.0216	-3.122	5.069	0.0180	0.0847	-1.747	4.555	0.1156	0.3479
SGO_0673	DivIB; cell division protein DivIB	-1.650	6.736	0.0020	0.0049	-2.932	6.419	0.0241	0.0961	-2.920	6.420	0.0136	0.0962	-1.282	4.462	0.0387	0.2526	-1.270	3.853	0.0431	0.2539	0.012	2.877		
SGO_0674	ftsA; cell division protein FtsA	0.569	9.226	0.0003	0.0004	0.359	9.105	0.0012	0.0021	0.429	9.145	0.0011	0.0037	-0.210	8.869	0.0037	0.0120	-0.140	8.291	0.0145	0.0659	0.070	8.796	0.0972	0.2773
SGO_0675	ftsZ; cell division protein FtsZ	0.657	9.878	0.0130	0.0548	0.507	9.775	0.0084	0.0266	0.860	9.992	0.0013	0.0044	-0.149	9.573	0.0515	0.3515	0.204	9.139	0.0478	0.2859	0.353	9.672	0.0028	0.0006
SGO_0676	conserved hypothetical protein TIGR00044	-0.268	5.946	0.0808	0.5048	0.129	6.116	0.1080	0.5380	-0.086	5.997	0.0553	0.4930	0.397	5.507	0.0465	0.3128	0.182	4.768	0.0997	0.6987	-0.215	5.555	0.0878	0.2400
SGO_0677	ylmF protein	-0.741	8.345	0.0045	0.0138	-0.679	8.372	0.0077	0.0236	-0.787	8.346	0.0071	0.0406	0.062	7.443	0.0778	0.5626	-0.046	6.798	0.1359	0.9911	-0.108	7.445	0.2139	0.7877
SGO_0680	cell division protein DivIVA	0.018	8.598	0.1210	0.7945	0.819	9.042	0.0017	0.0035	0.360	8.769	0.0084	0.0506	0.801	8.548	0.0049	0.0186	0.341	7.668	0.0356	0.2039	-0.460	8.674	0.0226	0.0338
SGO_0681	ileS; isoleucyl-tRNA synthetase	-0.268	8.346	0.0217	0.1021	0.809	8.932	0.0014	0.0026	0.282	8.622	0.0122	0.0830	1.077	8.323	0.0001	0.0000	0.551	7.377	0.0007	0.0008	-0.526	8.524	0.0026	0.0005
SGO_0684	hypothetical protein SGO_0684	0.170	8.754	0.0141	0.0608	-1.462	8.112	0.0000	0.0000	-0.754	8.337	0.0001	0.0000	-1.632	7.728	0.0004	0.0004	-0.924	7.324	0.0010	0.0012	0.708	7.087	0.0016	0.0002

Log_2 Ratios

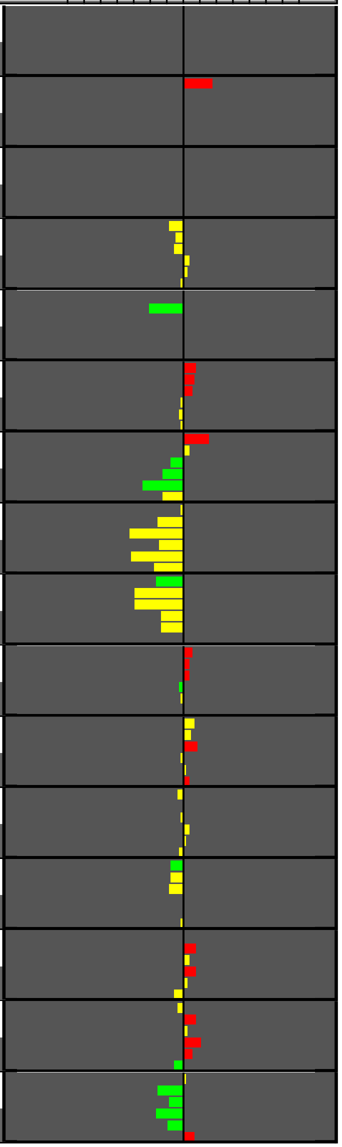

Show detected proteins only
Show all proteins
Filter by category:
ABC Transporter

Proteins found:
1179

Dot Plots
Dot Plots

Test	Cutoff
q-Value	.005
p-Value	

	Signif	Direction	Applies To
(red)	yes	+	ratios, bars
(yellow)	no	n/a	bars
(green)	yes	–	ratios, bars
(pink)	yes	+	*p*- , *q*-Values
(light green)	yes	–	

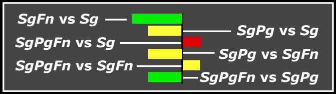

Hendrickson *et al.*

Streptococcus gordonii

Protein	Description	*SgFn vs Sg*				*SgPg vs Sg*				*SgPgFn vs Sg*				*SgPg vs SgFn*				*SgPgFn vs SgFn*				*SgPgFn vs SgPg*			
		Ratio	Sum	*q*-Val	*p*-Val	Ratio	Sum	*q*-Val	*p*-Val	Ratio	Sum	*q*-Val	*p*-Val	Ratio	Sum	*q*-Val	*p*-Val	Ratio	Sum	*q*-Val	*p*-Val	Ratio	Sum	*q*-Val	*p*-Val
**SGO_0685**	MutT/nudix family protein	0.646	3.529				2.170				2.170				2.302				1.692						
**SGO_0688**	ATP dependent Clp protease, ATP-binding subunit, ClpE	0.228	8.363	0.0550	0.3181	-0.104	8.168	0.0159	0.0586	-0.604	7.948	0.0002	0.0003	-0.332	7.804	0.0336	0.2103	-0.832	6.996	0.0118	0.0511	-0.500	7.374	0.0031	0.0007
**SGO_0689**	hypothetical protein SGO_0689		3.170				3.170				3.170														
**SGO_0690**	folD; methenyltetrahydrofolate cyclohydrolase	1.809	5.251	0.0084	0.0320	0.338	3.866	0.1779	0.9520		3.260			-1.471	4.553	0.0349	0.2212		3.709				1.807		
**SGO_0693**	xseA; exodeoxyribonuclease VII, large subunit	0.753	6.673	0.0237	0.1130	-2.268	5.513	0.0024	0.0053	-2.019	5.369	0.0151	0.1091	-3.021	5.697	0.0055	0.0215	-2.773	4.999	0.0401	0.2338	0.248	3.226	0.2508	0.9789
**SGO_0694**	xseB; exodeoxyribonuclease VII, small subunit		3.248			0.972	4.814				3.248				3.707								3.707		
**SGO_0695**	geranyltranstransferase		2.931				3.321							0.389	3.626				1.807				2.807		
**SGO_0696**	hemolysin-like protein		3.029				3.029				3.029														
**SGO_0697**	transcription regulator		2.339			0.936	3.253	0.0636	0.2947		2.339				1.648								1.648		
**SGO_0698**	recN; DNA repair protein RecN	1.171	6.257	0.0039	0.0118	0.402	5.779	0.0587	0.2686		4.604			-0.769	5.857	0.0019	0.0043		4.580				4.421		
**SGO_0699**	Serine/threonine protein phosphatase	1.280	5.382	0.0018	0.0043	0.342	4.783	0.0207	0.0805		3.609			-0.938	4.974	0.0023	0.0060		3.759				3.426		
**SGO_0700**	DegV family protein	-0.380	5.870	0.0479	0.2708	0.522	6.310	0.0140	0.0498	-0.170	5.972	0.0741	0.6885	0.903	5.663	0.0060	0.0237	0.210	4.677	0.0820	0.5588	-0.692	5.746	0.0354	0.0673
**SGO_0701**	hup; DNA-binding histone-like protein HU	0.805	12.498	0.0064	0.0221	1.056	12.649	0.0002	0.0001	0.991	12.614	0.0019	0.0078	0.251	12.458	0.0282	0.1725	0.186	11.819	0.0600	0.3839	-0.065	12.543	0.1866	0.6611
**SGO_0704**	gpmA; 2,3-bisphosphoglycerate-dependent phosphoglycerate mutase	1.491	10.896	0.0009	0.0016	1.791	11.120	0.0004	0.0004	2.431	11.636	0.0001	0.0001	0.300	11.092	0.0038	0.0127	0.940	10.868	0.0004	0.0002	0.640	11.588	0.0012	0.0001
**SGO_0705**	Protein of unknown function (DUF1250) superfamily		1.807				1.807				1.807														
**SGO_0706**	phoH-like protein	2.946	6.245	0.0001	0.0001	1.431	5.021	0.0017	0.0035	1.279	4.295	0.0196	0.1513	-1.515	5.980	0.0001	0.0000	-1.667	5.147	0.0054	0.0186	-0.152	4.568	0.0575	0.1368

Log$_2$ Ratios

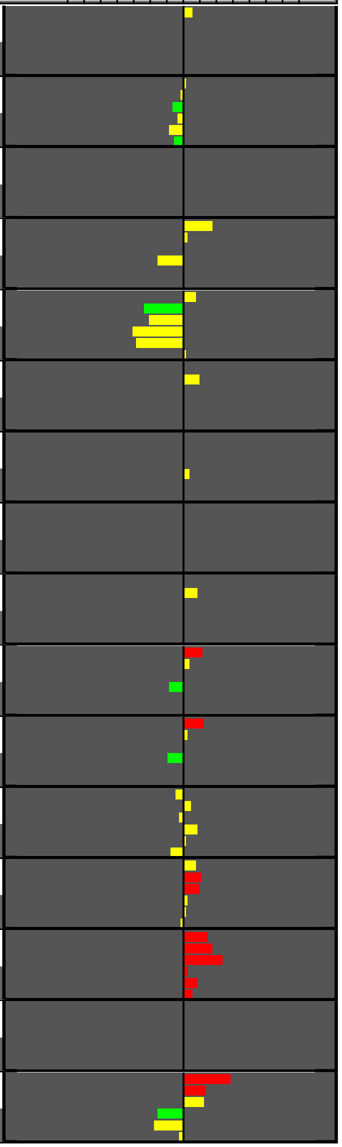

Show detected proteins only
Show all proteins
Filter by category:
ABC Transporter

Proteins found:
1179

Dot Plots
Dot Plots

Test	Cutoff
q-Value	.005
p-Value	

	Signif	Direction	Applies To
(red)	yes	+	ratios, bars
(yellow)	no	n/a	bars
(green)	yes	–	ratios, bars
(pink)	yes	+	*p*- , *q*-Values
(light green)	yes	–	

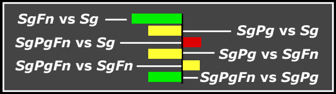

Hendrickson *et al.*

Streptococcus gordonii

Protein	SgFn vs Sg				SgPg vs Sg				SgPgFn vs Sg				SgPg vs SgFn				SgPgFn vs SgFn				SgPgFn vs SgPg				Description
	Ratio	Sum	*q*-Val	*p*-Val	Ratio	Sum	*q*-Val	*p*-Val	Ratio	Sum	*q*-Val	*p*-Val	Ratio	Sum	*q*-Val	*p*-Val	Ratio	Sum	*q*-Val	*p*-Val	Ratio	Sum	*q*-Val	*p*-Val	
**SGO_0707**	0.131	9.804	0.0405	0.2218	-5.103	8.777	0.0000	0.0000	-4.949	8.783	0.0000	0.0000	-5.234	8.398	0.0005	0.0005	-5.080	7.793	0.0006	0.0005	0.154	4.307	0.1982	0.7097	LPXTG cell wall surface protein
**SGO_0708**	3.041	11.420	0.0000	0.0000	0.702	9.607	0.0002	0.0001	0.711	9.617	0.0018	0.0071	-2.339	11.001	0.0000	0.0000	-2.330	10.393	0.0001	0.0000	0.009	9.410	0.2272	0.8553	ald; alanine dehydrogenase
**SGO_0711**	0.408	4.363				3.145				3.145				3.039				2.429							conserved hypothetical protein TIGR00043
**SGO_0713**	1.330	7.661	0.0001	0.0001	0.586	7.177	0.0059	0.0173	0.405	7.070	0.0056	0.0304	-0.744	7.343	0.0012	0.0024	-0.925	6.666	0.0007	0.0007	-0.181	6.841	0.0893	0.2463	sgg; GTP-binding protein Era
**SGO_0715**		2.322			-0.001	3.321				2.322				1.807								1.807			mutM; formamidopyrimidine-DNA glycosylase
**SGO_0719**	-0.545	6.065	0.0077	0.0282	-2.061	5.620	0.0009	0.0013		5.311			-1.516	4.686	0.0013	0.0029		3.644				2.735			rnr; ribonuclease R
**SGO_0721**	0.551	5.573	0.0013	0.0026	0.112	5.337	0.0947	0.4647	0.287	5.501	0.0435	0.3781	-0.439	5.111	0.0072	0.0300	-0.265	4.636	0.1044	0.7373	0.174	5.042	0.1577	0.5139	abpB-like dipeptidase lipoprotein
**SGO_0722**	-0.229	5.726	0.0706	0.4330	0.624	6.153	0.0651	0.3027		4.921			0.853	5.475	0.0002	0.0001		3.376				4.839			tehB; tellurite resistance protein TehB
**SGO_0724**	-1.628	6.596	0.0007	0.0011	-2.408	6.437	0.0004	0.0003		6.186			-0.780	4.723	0.0077	0.0334		3.454				3.275			dipeptidase
**SGO_0728**		2.000				2.000				2.000															ABC transporter, ATP-binding protein SP1653
**SGO_0729**		3.022				3.022				3.022															ABC transporter permease protein
**SGO_0731**		4.120				4.120				4.120															RRF2 family protein
**SGO_0736**	0.917	6.466	0.0009	0.0015	-1.037	5.520	0.0033	0.0081	-1.951	5.113	0.0111	0.0738	-1.954	5.676	0.0005	0.0005	-2.868	4.823	0.0101	0.0416	-0.914	3.749	0.0956	0.2697	hprK; HPr(Ser) kinase/phosphatase
**SGO_0737**		4.348				4.348				4.348															lgt; prolipoprotein diacylglyceryl transferase
**SGO_0738**		6.938			-3.482	7.062	0.0005	0.0006		6.938				2.941								2.941			Bacterial protein of unknown function (DUF948) family
**SGO_0739**	-0.567	4.807	0.0334	0.1759	0.984	5.964	0.0021	0.0046	0.854	5.875	0.0006	0.0015	1.551	5.087	0.0174	0.0968	1.421	4.361	0.0109	0.0466	-0.130	5.795	0.0902	0.2502	hypothetical protein SGO_0739

Log $_2$ Ratios

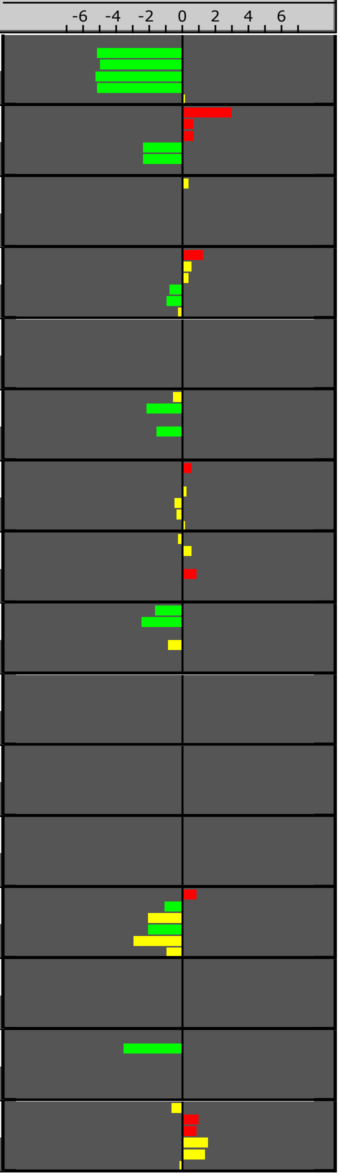

Show detected proteins only
Show all proteins
Filter by category:
ABC Transporter

Proteins found:
1179

Dot Plots
Dot Plots

Test	Cutoff
q-Value	.005
p-Value	

	Signif	Direction	Applies To
(red)	yes	+	ratios, bars
(yellow)	no	n/a	bars
(green)	yes	–	ratios, bars
(light red)	yes	+	*p*- , *q*-Values
(light green)	yes	–	

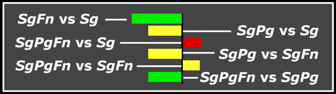

Hendrickson *et al.*

Streptococcus gordonii

Protein	SgFn vs Sg Ratio	Sum	*q*-Val	*p*-Val	SgPg vs Sg Ratio	Sum	*q*-Val	*p*-Val	SgPgFn vs Sg Ratio	Sum	*q*-Val	*p*-Val	SgPg vs SgFn Ratio	Sum	*q*-Val	*p*-Val	SgPgFn vs SgFn Ratio	Sum	*q*-Val	*p*-Val	SgPgFn vs SgPg Ratio	Sum	*q*-Val	*p*-Val	Description
SGO_0742	0.753	6.494	0.0004	0.0005	0.310	6.232	0.0039	0.0104	0.136	5.701	0.0463	0.4048	-0.443	6.102	0.0000	0.0000	-0.616	5.103	0.0023	0.0056	-0.174	5.394	0.0184	0.0243	peptidase, U32 family
SGO_0743	-2.254	7.696	0.0001	0.0000	-1.241	7.933	0.0005	0.0007	-1.304	7.921	0.0007	0.0019	1.012	6.258	0.0031	0.0096	0.950	5.620	0.0085	0.0330	-0.062	6.660	0.2296	0.8666	peptidase, U32 family
SGO_0745	1.409	5.643	0.0205	0.0948	2.842	7.443	0.0002	0.0001	2.462	7.139	0.0015	0.0057	1.433	6.982	0.0106	0.0504	1.053	6.078	0.0389	0.2241	-0.380	7.571	0.0455	0.0988	hypothetical protein SGO_0745
SGO_0749	2.313	7.691	0.0032	0.0094	1.249	6.844	0.0018	0.0038	0.577	6.407	0.0069	0.0395	-1.065	7.467	0.0068	0.0278	-1.737	6.673	0.0041	0.0126	-0.672	6.517	0.0067	0.0042	glutathione reductase
SGO_0750	-5.485	10.400	0.0005	0.0007	-1.521	10.797	0.0013	0.0023	-2.349	10.625	0.0005	0.0012	3.963	8.415	0.0001	0.0000	3.135	7.045	0.0004	0.0002	-0.828	8.970	0.0037	0.0010	efflux transporter, RND family, MFP subunit subfamily
SGO_0751	-3.035	7.519	0.0002	0.0002	0.115	8.412	0.0791	0.3822	0.176	8.439	0.0142	0.1021	3.150	7.118	0.0009	0.0015	3.211	6.554	0.0004	0.0003	0.061	7.986	0.1979	0.7045	ABC transporter, ATP-binding protein SP0786
SGO_0752		7.159			-1.394	7.621	0.0017	0.0034	-2.977	7.247	0.0130	0.0914		5.241				2.046			-1.583	5.463	0.0073	0.0049	ABC transporter, ATP-binding protein
SGO_0753	-0.738	8.826	0.0084	0.0316	0.038	9.150	0.1267	0.6427	-0.113	9.075	0.0303	0.2481	0.776	8.336	0.0061	0.0244	0.624	7.633	0.0121	0.0526	-0.152	8.582	0.0514	0.1174	lysS; lysyl-tRNA synthetase
SGO_0754		3.038				1.835							-1.203	3.045				1.914				1.322			phosphoglycerate mutase family protein
SGO_0755	0.217	5.320	0.0517	0.2947	0.647	5.566	0.0123	0.0424	-0.282	4.691	0.0355	0.2972	0.430	5.150	0.0253	0.1495	-0.500	3.740	0.0565	0.3592	-0.930	4.679	0.0843	0.2256	regulatory protein
SGO_0757		6.945				6.945				6.945															peptidase, U32 family
SGO_0760	0.523	9.217	0.0019	0.0046	0.615	9.269	0.0000	0.0000	0.431	9.163	0.0019	0.0079	0.092	8.988	0.0338	0.2120	-0.093	8.287	0.0546	0.3434	-0.184	8.944	0.0275	0.0469	ppc; phosphoenolpyruvate carboxylase
SGO_0761	0.986	13.763	0.0007	0.0011	0.462	13.435	0.0010	0.0017	0.656	13.549	0.0001	0.0000	-0.524	13.422	0.0022	0.0058	-0.330	12.893	0.0045	0.0138	0.194	13.234	0.0122	0.0126	tuf; translation elongation factor Tu
SGO_0762	-0.128	10.618	0.0596	0.3510	0.868	11.183	0.0047	0.0131	1.175	11.402	0.0039	0.0197	0.996	10.630	0.0038	0.0129	1.303	10.244	0.0054	0.0182	0.307	11.217	0.0902	0.2505	tpiA; triosephosphate isomerase
SGO_0763	-0.883	8.224	0.0007	0.0010	-1.037	8.173	0.0008	0.0011	-1.499	8.053	0.0010	0.0030	-0.153	7.128	0.0151	0.0803	-0.615	6.340	0.0148	0.0673	-0.462	6.864	0.0656	0.1614	murA-1; UDP-N-acetylglucosamine 1-carboxyvinyltransferase
SGO_0765		4.959				4.959				4.959															endA; DNA-entry nuclease

Log_2 Ratios

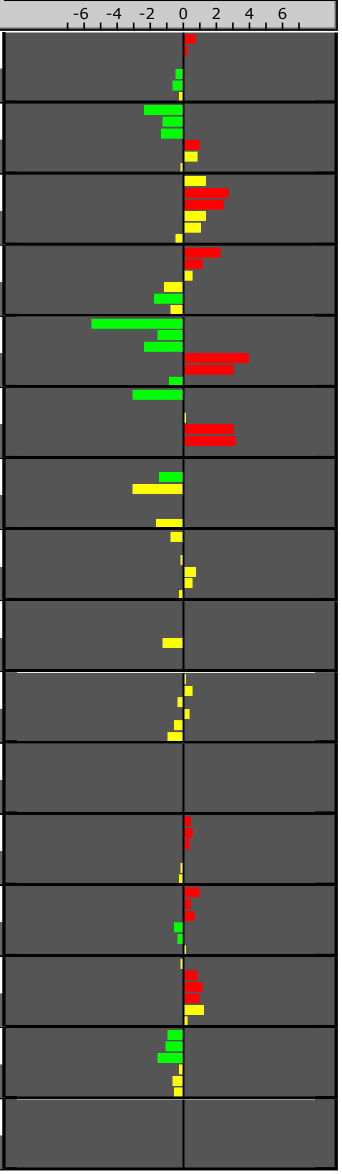

Show detected proteins only
Show all proteins
Filter by category:
ABC Transporter

Proteins found:
1179

Dot Plots
Dot Plots

Test	Cutoff
q-Value	.005
p-Value	

	Signif	Direction	Applies To
red	yes	+	ratios, bars
yellow	no	n/a	bars
green	yes	–	ratios, bars
light red	yes	+	*p*- , *q*-Values
light green	yes	–	*p*- , *q*-Values

SgFn vs Sg
SgPgFn vs Sg
SgPgFn vs SgFn
SgPg vs Sg
SgPg vs SgFn
SgPgFn vs SgPg

Hendrickson *et al.*

Spectral Counts
Sg Summary Table

Streptococcus gordonii

Hackett Laboratory UW

Summary Table | SgFn vs Sg | SgPg vs Sg | SgPgFn vs Sg | SgPg v SgFn | SgPgFn v SgFn | SgPgFn vs SgPg | Coverage

Protein	Description	*SgFn vs Sg*				*SgPg vs Sg*				*SgPgFn vs Sg*				*SgPg vs SgFn*				*SgPgFn vs SgFn*				*SgPgFn vs SgPg*			
		Ratio	Sum	*q*-Val	*p*-Val	Ratio	Sum	*q*-Val	*p*-Val	Ratio	Sum	*q*-Val	*p*-Val	Ratio	Sum	*q*-Val	*p*-Val	Ratio	Sum	*q*-Val	*p*-Val	Ratio	Sum	*q*-Val	*p*-Val
**SGO_0767**	transport protein	0.649	5.851	0.0207	0.0965	-0.180	5.377	0.0674	0.3152		4.456			-0.829	5.279	0.0144	0.0755		4.038				3.781		
**SGO_0771**	pepq; proline dipeptidase	1.357	8.365	0.0016	0.0037	1.279	8.306	0.0013	0.0023	0.699	7.925	0.0046	0.0243	-0.078	8.325	0.0501	0.3396	-0.658	7.462	0.0020	0.0046	-0.580	8.022	0.0012	0.0001
**SGO_0773**	ccpA; catabolite control protein A	-2.320	10.237	0.0002	0.0001	-2.406	10.223	0.0002	0.0001	-2.492	10.209	0.0001	0.0001	-0.087	8.100	0.0601	0.4177	-0.172	7.448	0.0253	0.1330	-0.086	8.013	0.1168	0.3541
**SGO_0774**	glycosyl transferase, group 1 family protein	-1.831	7.074	0.0009	0.0018	-1.574	7.142	0.0017	0.0036	-1.853	6.904	0.0142	0.1017	0.257	5.516	0.0357	0.2284	-0.022	4.332	0.1048	0.7413	-0.279	5.141	0.1579	0.5182
**SGO_0775**	glycosyl transferase, group 1	-0.533	6.511	0.0127	0.0537	-0.719	6.433	0.0052	0.0149	-1.162	6.276	0.0009	0.0028	-0.186	5.622	0.0562	0.3873	-0.629	4.816	0.0103	0.0434	-0.443	5.309	0.0248	0.0395
**SGO_0778**	thrS; threonyl-tRNA synthetase	-0.632	9.020	0.0003	0.0002	-0.132	9.237	0.0068	0.0204	-0.413	9.110	0.0003	0.0004	0.500	8.428	0.0005	0.0005	0.220	7.659	0.0011	0.0016	-0.280	8.523	0.0045	0.0014
**SGO_0779**	response regulator	1.732	7.769	0.0006	0.0008	0.258	6.808	0.0709	0.3336	-1.147	6.246	0.0082	0.0488	-1.473	7.317	0.0007	0.0008	-2.878	6.457	0.0005	0.0004	-1.405	5.902	0.0158	0.0186
**SGO_0780**	histidine kinase	-1.119	5.893	0.0058	0.0196	-2.189	5.620	0.0017	0.0034		5.337			-1.070	4.283	0.0082	0.0369		3.126				2.617		
**SGO_0781**	vicX; Zn-dependent hydrolase (beta-lactamase superfamily)	-1.851	5.277	0.0213	0.0999		5.091				5.091				1.717				1.107						
**SGO_0782**	Protein of unknown function (DUF454) family		3.665				3.665				3.665														
**SGO_0784**	smc; chromosome segregation protein SMC	-1.644	8.363	0.0015	0.0033	-1.759	8.327	0.0011	0.0018	-2.876	8.139	0.0005	0.0010	-0.115	6.752	0.0695	0.4919	-1.232	5.715	0.0055	0.0190	-1.117	6.222	0.0007	0.0000
**SGO_0785**	Cof family protein	-0.328	4.940	0.0462	0.2608	-0.875	4.804	0.0299	0.1232		4.457			-0.548	3.363				2.000				2.063		
**SGO_0786**	Cof family protein	-1.050	4.921	0.0257	0.1273	0.800	6.061	0.0010	0.0015	0.377	5.816	0.0142	0.1012	1.850	5.077	0.0050	0.0193	1.427	4.116	0.0282	0.1522	-0.423	5.701	0.0238	0.0369
**SGO_0787**	ftsY; cell division protein FtsY	-0.721	7.421	0.0055	0.0182	-0.273	7.598	0.0142	0.0509	-0.953	7.335	0.0016	0.0064	0.448	6.741	0.0087	0.0402	-0.232	5.787	0.0490	0.2959	-0.680	6.644	0.0078	0.0056
**SGO_0788**	zwf; glucose-6-phosphate 1-dehydrogenase	2.261	8.231	0.0012	0.0024	1.244	7.437	0.0003	0.0002	1.161	7.380	0.0002	0.0002	-1.017	8.020	0.0028	0.0083	-1.100	7.383	0.0027	0.0071	-0.083	7.373	0.0165	0.0200
**SGO_0792**	hypothetical protein SGO_0792	-1.830	6.869	0.0014	0.0031	-0.585	7.246	0.0105	0.0350	-0.372	7.330	0.0097	0.0613	1.245	5.922	0.0025	0.0071	1.458	5.456	0.0009	0.0011	0.213	6.515	0.0667	0.1684

Log$_2$ Ratios

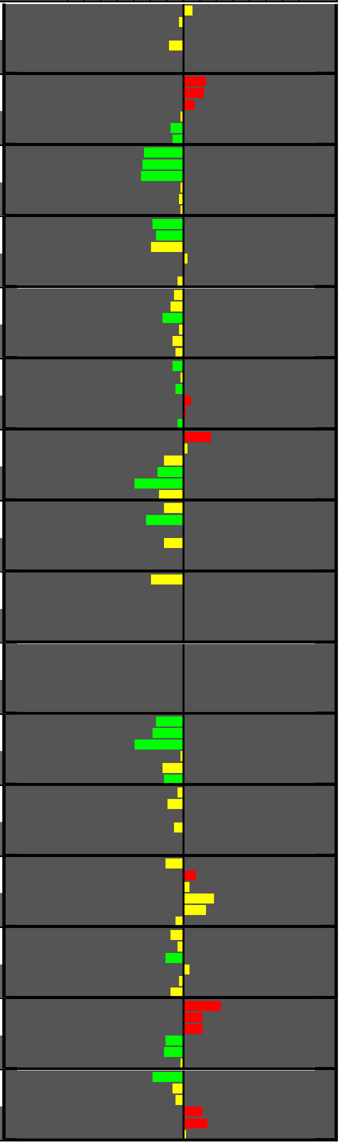

Show detected proteins only
Show all proteins
Filter by category:
ABC Transporter

Proteins found:
1179

Dot Plots
Dot Plots

Test	Cutoff
q-Value	.005
p-Value	

	Signif	Direction	Applies To
red	yes	+	ratios, bars
yellow	no	n/a	bars
green	yes	−	ratios, bars
light red	yes	+	*p*- , *q*-Values
light green	yes	−	

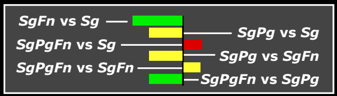

Hendrickson *et al.*

Summary Table | SgFn vs Sg | SgPg vs Sg | SgPgFn vs Sg | SgPg v SgFn | SgPgFn v SgFn | SgPgFn vs SgPg | Coverage

Protein	Description	*SgFn vs Sg* Ratio	Sum	*q*-Val	*p*-Val	*SgPg vs Sg* Ratio	Sum	*q*-Val	*p*-Val	*SgPgFn vs Sg* Ratio	Sum	*q*-Val	*p*-Val	*SgPg vs SgFn* Ratio	Sum	*q*-Val	*p*-Val	*SgPgFn vs SgFn* Ratio	Sum	*q*-Val	*p*-Val	*SgPgFn vs SgPg* Ratio	Sum	*q*-Val	*p*-Val
**SGO_0794**	metallo-beta-lactamase family protein	0.304	7.978	0.0206	0.0955	-0.049	7.789	0.1049	0.5188	-0.592	7.547	0.0015	0.0055	-0.353	7.442	0.0119	0.0588	-0.896	6.619	0.0029	0.0081	-0.543	7.003	0.0048	0.0017
**SGO_0795**	tributyrin esterase	2.063	5.813	0.0022	0.0058	1.371	5.264	0.0002	0.0001	0.525	4.707	0.0008	0.0022	-0.692	5.681	0.0081	0.0360	-1.539	4.805	0.0031	0.0086	-0.846	4.916	0.0016	0.0002
**SGO_0797**	RocB protein, putative	-0.613	4.048				3.322				3.322				2.196				1.585						
**SGO_0798**	ABC transporter, ATP-binding protein SP1381	-1.060	7.828	0.0009	0.0017	-1.256	7.768	0.0009	0.0013	-1.346	7.742	0.0005	0.0011	-0.196	6.593	0.0045	0.0162	-0.286	5.940	0.0007	0.0007	-0.090	6.447	0.0451	0.0973
**SGO_0800**	polysaccharide deacetylase family protein	-2.065	6.313	0.0072	0.0261	-1.446	6.450	0.0120	0.0409	-1.735	6.211	0.0329	0.2721	0.619	4.705	0.0019	0.0047	0.330	3.454	0.0202	0.0988	-0.289	4.477	0.0893	0.2470
**SGO_0801**	hom; homoserine dehydrogenase	0.572	8.591	0.0014	0.0028	1.139	8.955	0.0003	0.0002	0.695	8.684	0.0080	0.0468	0.567	8.645	0.0005	0.0004	0.123	7.802	0.0693	0.4577	-0.444	8.709	0.0357	0.0679
**SGO_0802**	thrB; homoserine kinase	0.760	6.777	0.0003	0.0003	0.767	6.782	0.0003	0.0002	-0.222	6.252	0.0335	0.2779	0.008	6.597	0.0826	0.6016	-0.982	5.582	0.0015	0.0031	-0.990	6.198	0.0056	0.0030
**SGO_0803**	hypothetical protein SGO_0803		7.539			-1.866	7.889	0.0002	0.0001	-2.851	7.738	0.0002	0.0002		5.159				3.654			-0.985	5.780	0.0183	0.0238
**SGO_0804**	murB; UDP-N-acetylenolpyruvoylglucosamine reductase	0.475	5.314	0.0954	0.6141	-0.227	4.969	0.0921	0.4499		4.183			-0.701	4.607	0.0102	0.0483		3.311				3.205		
**SGO_0805**	potA; spermidine/putrescine ABC transporter, ATP-binding subunit	-1.847	6.699	0.0005	0.0007	-2.598	6.570	0.0004	0.0005		6.344			-0.751	4.677	0.0092	0.0427		3.382				3.272		
**SGO_0806**	potB; spermidine/putrescine ABC transporter, permease protein		4.271				4.271				4.271														
**SGO_0808**	potD; spermidine/putrescine ABC transporter	-3.175	6.779	0.0410	0.2259	-3.301	6.773	0.0501	0.2239		6.705			-0.125	2.871				1.322				1.807		
**SGO_0812**	hypothetical protein SGO_0812	1.605	6.051	0.0014	0.0029	-0.235	4.916	0.0150	0.0547		4.031			-1.840	5.482	0.0012	0.0023		4.519				3.278		
**SGO_0813**	fructose-bisphosphatase	0.162	3.519	0.0963	0.6216		1.807				1.807				2.480				1.869						
**SGO_0814**	aminotransferase, class-V		2.170			0.665	3.541			0.226	3.287				2.322				1.272			-0.439	3.119		
**SGO_0815**	thiI; thiamine biosynthesis protein ThiI	-1.223	7.201	0.0008	0.0014	-0.231	7.574	0.0182	0.0693	-0.667	7.389	0.0012	0.0041	0.991	6.530	0.0013	0.0026	0.556	5.645	0.0041	0.0124	-0.436	6.739	0.0128	0.0137

Log_2 Ratios

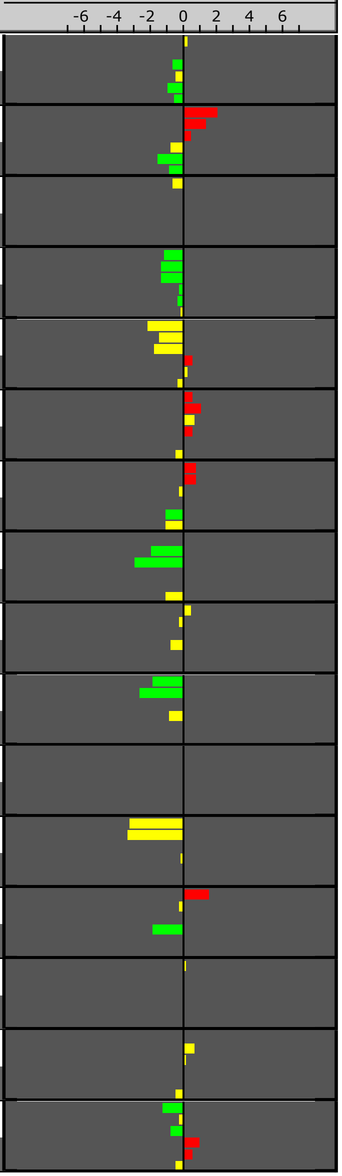

Show detected proteins only
Show all proteins
Filter by category:
ABC Transporter

Proteins found: 1179

Dot Plots
Dot Plots

Test	Cutoff
q-Value	.005
p-Value	

Colour	Signif	Direction	Applies To
red	yes	+	ratios, bars
yellow	no	n/a	bars
green	yes	–	ratios, bars
light red	yes	+	*p*- , *q*-Values
light green	yes	–	*p*- , *q*-Values

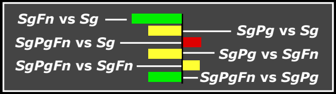

Hendrickson *et al.*

Streptococcus gordonii

Protein	*SgFn vs Sg* Ratio	Sum	*q*-Val	*p*-Val	*SgPg vs Sg* Ratio	Sum	*q*-Val	*p*-Val	*SgPgFn vs Sg* Ratio	Sum	*q*-Val	*p*-Val	*SgPg vs SgFn* Ratio	Sum	*q*-Val	*p*-Val	*SgPgFn vs SgFn* Ratio	Sum	*q*-Val	*p*-Val	*SgPgFn vs SgPg* Ratio	Sum	*q*-Val	*p*-Val
**SGO_0817** hypothetical protein SGO_0817		3.556				3.556				3.556														
**SGO_0818** rplU; ribosomal protein L21	-0.991	7.586	0.0392	0.2131	2.010	9.582	0.0005	0.0006	2.156	9.701	0.0004	0.0007	3.001	8.832	0.0110	0.0524	3.147	8.361	0.0134	0.0601	0.146	9.821	0.0805	0.2119
**SGO_0820** rpmA; ribosomal protein L27	-0.660	7.245	0.0675	0.4089	0.025	7.846	0.1704	0.8971	0.074	7.887	0.0933	0.8915	0.685	6.704	0.0229	0.1335	0.734	6.156	0.0515	0.3181	0.049	7.356	0.1989	0.7149
**SGO_0822** lipoprotein, putative		3.621				3.621				3.621														
**SGO_0823** Cof family protein	0.742	4.129	0.0368	0.1974	-0.165	2.920				2.000			-0.907	3.580	0.0282	0.1720		2.630				1.322		
**SGO_0824** lepA; GTP-binding protein LepA	0.590	7.350	0.0082	0.0310	1.254	7.781	0.0010	0.0017	0.727	7.433	0.0035	0.0168	0.663	7.458	0.0000	0.0000	0.136	6.548	0.0078	0.0297	-0.527	7.513	0.0019	0.0003
**SGO_0825** lipoprotein, putative	-2.331	6.193	0.0250	0.1222	-1.794	6.419	0.0019	0.0042		6.057			0.536	4.160	0.0096	0.0448		1.585				3.732		
**SGO_0830** uvrC; excinuclease ABC, C subunit	1.409	3.045	0.0116	0.0479	0.755	2.710	0.0282	0.1150		1.832			-0.654	2.427				1.107				1.063		
**SGO_0832** hypothetical protein SGO_0832	-3.238	6.446	0.0184	0.0838	-2.252	6.649	0.0010	0.0016	-2.454	6.497	0.0130	0.0915	0.985	3.942	0.0356	0.2272	0.783	2.444			-0.202	4.135	0.1685	0.5790
**SGO_0833** phosphoesterase-related gene		5.529				3.120							-2.479	5.265	0.0007	0.0009		4.405				2.606		
**SGO_0834** hypothetical protein SGO_0834	-1.079	4.671	0.0550	0.3178		4.377				4.377				1.717				1.107						
**SGO_0835** nitroreductase	2.378	8.920	0.0001	0.0000	1.275	8.064	0.0004	0.0004	1.176	8.008	0.0025	0.0110	-1.103	8.702	0.0000	0.0000	-1.203	8.066	0.0008	0.0008	-0.100	8.008	0.1676	0.5747
**SGO_0836** pepV; dipeptidase PepV	1.291	9.572	0.0004	0.0004	0.793	9.237	0.0004	0.0004	0.471	9.046	0.0053	0.0283	-0.497	9.336	0.0013	0.0026	-0.820	8.604	0.0015	0.0029	-0.322	8.917	0.0227	0.0344
**SGO_0841** oxidoreductase	4.736	7.573	0.0111	0.0444		1.807				1.807				7.033				6.422						
**SGO_0842** rhodanese family protein	2.744	6.552	0.0028	0.0078	0.073	4.710	0.1825	0.9811	0.388	4.835	0.0615	0.5534	-2.671	6.038	0.0025	0.0072	-2.356	5.463	0.0027	0.0073	0.314	4.316	0.1271	0.3929
**SGO_0848** rpmE; ribosomal protein L31	-0.727	7.350	0.0071	0.0254	1.269	8.420	0.0000	0.0000	1.395	8.511	0.0001	0.0001	1.996	7.739	0.0004	0.0003	2.122	7.230	0.0005	0.0003	0.126	8.471	0.0199	0.0280

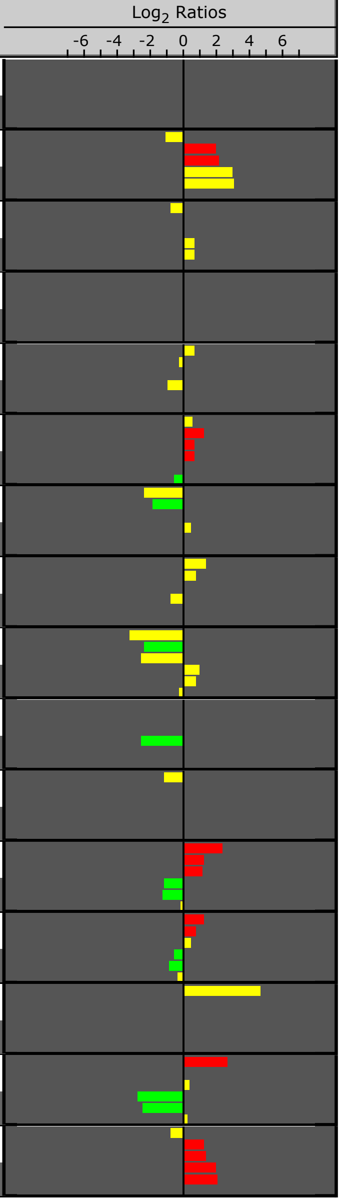

Show detected proteins only
Show all proteins
Filter by category:
ABC Transporter

Proteins found: 1179

Dot Plots
Dot Plots

Test	Cutoff
q-Value	.005
p-Value	

	Signif	Direction	Applies To
red	yes	+	ratios, bars
yellow	no	n/a	bars
green	yes	–	ratios, bars
light red	yes	+	*p*- , *q*-Values
light green	yes	–	

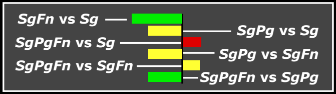

Hendrickson *et al.*

Streptococcus gordonii

Protein	Description	*SgFn vs Sg* Ratio	Sum	*q*-Val	*p*-Val	*SgPg vs Sg* Ratio	Sum	*q*-Val	*p*-Val	*SgPgFn vs Sg* Ratio	Sum	*q*-Val	*p*-Val	*SgPg vs SgFn* Ratio	Sum	*q*-Val	*p*-Val	*SgPgFn vs SgFn* Ratio	Sum	*q*-Val	*p*-Val	*SgPgFn vs SgPg* Ratio	Sum	*q*-Val	*p*-Val
**SGO_0849**	DHH subfamily 1 protein	0.752	6.415	0.0126	0.0528	0.055	6.011	0.1464	0.7609		4.979			-0.697	5.926	0.0132	0.0684		4.626				4.529		
**SGO_0850**	flavodoxin	0.386	6.736	0.0152	0.0673	1.562	7.506	0.0004	0.0004	2.383	8.256	0.0063	0.0348	1.176	7.105	0.0011	0.0020	1.997	7.200	0.0101	0.0422	0.821	8.115	0.0532	0.1238
**SGO_0851**	putative permease	1.157	4.915	0.0416	0.2304	0.281	4.984	0.1267	0.6444		3.898			-0.875	4.486	0.0156	0.0837		2.807				3.551		
**SGO_0852**	TPR domain protein	0.346	5.338	0.0035	0.0103	-0.862	4.813	0.0092	0.0300		4.156			-1.209	4.527	0.0025	0.0071		3.376				2.848		
**SGO_0853**	budA; alpha-acetolactate decarboxylase	-0.298	5.096	0.0590	0.3462	-0.638	4.965	0.0412	0.1775		4.288			-0.340	4.208	0.0129	0.0661		2.751				3.036		
**SGO_0854**	cshA; surface-associated protein CshA	-2.737	9.701	0.0002	0.0001	-5.332	9.527	0.0001	0.0000	-5.415	9.525	0.0001	0.0000	-2.595	6.521	0.0044	0.0160	-2.678	5.896	0.0048	0.0152	-0.083	4.618	0.1668	0.5698
**SGO_0855**	fbpA; fibronectin-binding protein A	-0.735	5.591	0.0050	0.0161	-0.453	5.699	0.0089	0.0287		4.908			0.282	4.813	0.0271	0.1633		3.061				3.941		
**SGO_0856**	ABC transporter, substrate binding protein	-3.308	8.206	0.0001	0.0000	-1.459	8.517	0.0003	0.0003	-2.301	8.333	0.0001	0.0000	1.850	6.458	0.0011	0.0021	1.007	5.228	0.0013	0.0022	-0.843	6.741	0.0099	0.0084
**SGO_0858**	ABC transporter, ATP-binding protein		3.727				3.727				3.727														
**SGO_0859**	pheS; phenylalanyl-tRNA synthetase, alpha subunit	0.405	7.841	0.0093	0.0357	-0.216	7.523	0.0440	0.1920	-0.807	7.283	0.0037	0.0179	-0.621	7.234	0.0007	0.0010	-1.212	6.420	0.0006	0.0004	-0.591	6.628	0.0101	0.0091
**SGO_0861**	pheT; phenylalanyl-tRNA synthetase, beta subunit	-0.152	8.497	0.0448	0.2521	0.743	8.986	0.0008	0.0012	0.727	8.977	0.0010	0.0033	0.895	8.418	0.0007	0.0009	0.878	7.798	0.0013	0.0023	-0.016	8.788	0.2193	0.8146
**SGO_0862**	hypothetical protein SGO_0862		3.475				3.475				3.475														
**SGO_0868**	hypothetical protein SGO_0868		1.058				1.058				1.058														
**SGO_0877**	permease domain protein		1.585				1.585				1.585														
**SGO_0885**	cobyric acid synthase	0.700	6.064	0.0149	0.0652	-0.639	5.374	0.0115	0.0390	-0.567	5.403	0.0086	0.0525	-1.339	5.333	0.0042	0.0146	-1.267	4.744	0.0049	0.0163	0.072	4.529	0.1023	0.2951
**SGO_0886**	Mur ligase family protein	0.806	5.586	0.0240	0.1154	-0.777	4.851	0.0434	0.1880		4.225			-1.583	4.790	0.0009	0.0015		3.751				2.830		

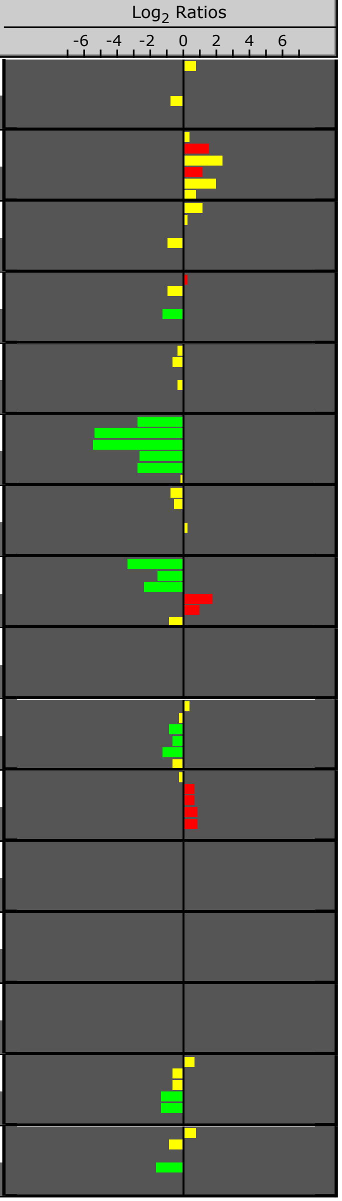

Show detected proteins only
Show all proteins
Filter by category:
ABC Transporter

Proteins found:
1179

Dot Plots
Dot Plots

Test	Cutoff
q-Value	.005
p-Value	

	Signif	Direction	Applies To
red	yes	+	ratios, bars
yellow	no	n/a	bars
green	yes	−	ratios, bars
light red	yes	+	*p*- , *q*-Values
light green	yes	−	

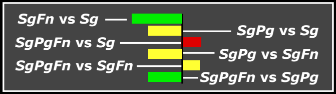

Hendrickson *et al.*

Streptococcus gordonii

Summary Table | SgFn vs Sg | SgPg vs Sg | SgPgFn vs Sg | SgPg v SgFn | SgPgFn v SgFn | SgPgFn vs SgPg | Coverage

Protein	Description	*SgFn vs Sg* Ratio	Sum	*q*-Val	*p*-Val	*SgPg vs Sg* Ratio	Sum	*q*-Val	*p*-Val	*SgPgFn vs Sg* Ratio	Sum	*q*-Val	*p*-Val	*SgPg vs SgFn* Ratio	Sum	*q*-Val	*p*-Val	*SgPgFn vs SgFn* Ratio	Sum	*q*-Val	*p*-Val	*SgPgFn vs SgPg* Ratio	Sum	*q*-Val	*p*-Val
**SGO_0887**	conserved hypothetical protein TIGR00159		7.303				7.303				7.303														
**SGO_0888**	hypothetical protein SGO_0888		5.203			-1.894	5.554	0.0025	0.0055	-2.014	5.369	0.0175	0.1316		2.830				1.046			-0.120	3.360	0.2049	0.7437
**SGO_0889**	glmM; phosphoglucosamine mutase	-0.553	6.939	0.0003	0.0003	0.013	7.195	0.1403	0.7211	-0.844	6.839	0.0020	0.0083	0.566	6.433	0.0003	0.0003	-0.291	5.387	0.0344	0.1939	-0.857	6.334	0.0094	0.0079
**SGO_0890**	LPXTG cell wall surface protein, collagen binding domain	-1.796	8.439	0.0006	0.0010		8.059				8.059				5.817				5.206						
**SGO_0893**	GTP-binding protein	0.773	7.832	0.0005	0.0008	0.666	7.769	0.0040	0.0106	0.522	7.678	0.0010	0.0033	-0.107	7.603	0.0440	0.2940	-0.251	6.921	0.0001	0.0000	-0.144	7.478	0.0694	0.1766
**SGO_0899**	putative hydrolase or acyltransferase		3.334				3.334				3.334														
**SGO_0901**	DNA-directed DNA polymerase III	0.594	6.466	0.0011	0.0021	0.470	6.395	0.0052	0.0150	-0.769	5.823	0.0046	0.0238	-0.125	6.162	0.0425	0.2819	-1.363	5.098	0.0016	0.0033	-1.239	5.624	0.0092	0.0073
**SGO_0904**	purR; galactose operon repressor GalR		1.058				1.058				1.058														
**SGO_0905**	lipoprotein, putative		4.857				4.857				4.857														
**SGO_0906**	leuA; 2-isopropylmalate synthase	3.270	8.067	0.0000	0.0000	1.999	6.978	0.0001	0.0000	1.830	6.859	0.0013	0.0047	-1.270	7.911	0.0000	0.0000	-1.439	7.258	0.0005	0.0004	-0.169	7.067	0.1085	0.3157
**SGO_0907**	leuB; 3-isopropylmalate dehydrogenase		2.828			0.546	3.597	0.0772	0.3701		2.828				1.807								1.807		
**SGO_0909**	leuC; 3-isopropylmalate dehydratase, large subunit		4.074				2.683							-0.372	4.027	0.0561	0.3851		2.951				2.170		
**SGO_0911**	hypothetical protein SGO_0911	1.606	7.986	0.0001	0.0000	1.860	8.180	0.0001	0.0000	1.485	7.903	0.0009	0.0027	0.254	8.195	0.0012	0.0024	-0.121	7.397	0.0554	0.3488	-0.375	8.145	0.0196	0.0271
**SGO_0915**	proV; ABC-type proline/glycine betaine transporter		4.909			-1.047	5.481	0.0012	0.0021		4.909				3.357								3.357		
**SGO_0916**	proWX; ABC transporter membrane-spanning permease		5.255				5.255				5.255														
**SGO_0932**	galK; galactokinase		2.845				2.845				2.845														

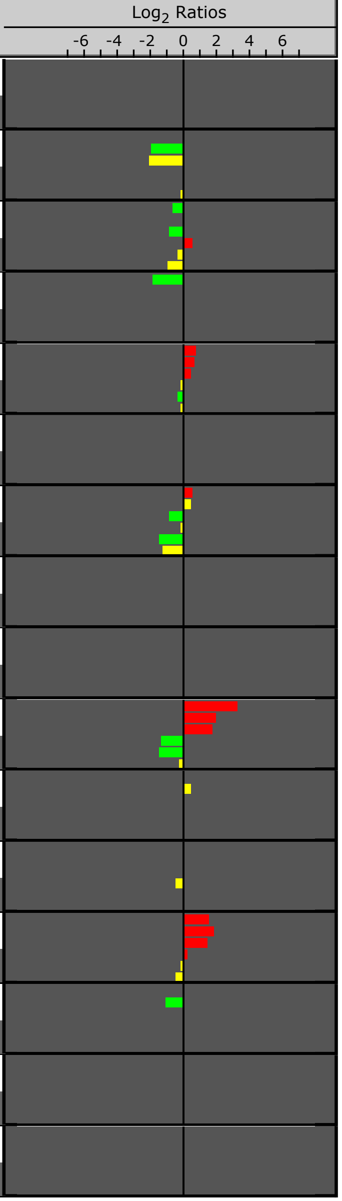

- Show detected proteins only
- Show all proteins
- Filter by category: ABC Transporter

Proteins found: 1179

Dot Plots

Dot Plots

Test	Cutoff
q-Value	.005
p-Value	

	Signif	Direction	Applies To
red	yes	+	ratios, bars
yellow	no	n/a	bars
green	yes	–	ratios, bars
light red	yes	+	*p*- , *q*-Values
light green	yes	–	

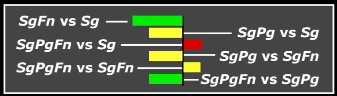

Hendrickson *et al.*

Streptococcus gordonii

Protein	*SgFn vs Sg* Ratio	Sum	*q*-Val	*p*-Val	*SgPg vs Sg* Ratio	Sum	*q*-Val	*p*-Val	*SgPgFn vs Sg* Ratio	Sum	*q*-Val	*p*-Val	*SgPg vs SgFn* Ratio	Sum	*q*-Val	*p*-Val	*SgPgFn vs SgFn* Ratio	Sum	*q*-Val	*p*-Val	*SgPgFn vs SgPg* Ratio	Sum	*q*-Val	*p*-Val
**SGO_0934**	-0.121	3.967	0.0745	0.4594		2.459				2.459				2.829				2.218						
	galE-2; UDP-glucose 4-epimerase																							
**SGO_0936**		2.036				2.036			1.188	3.105	0.0184	0.1408						1.046				1.657		
	phosphoenolpyruvate synthase, putative																							
**SGO_0938**										2.718								1.594				2.205		
	Phosphatidylserine decarboxylase proenzyme 2																							
**SGO_0940**		3.894				3.894			-0.447	4.331	0.0526	0.4634						1.272				1.883		
	ppsA; phosphoenolpyruvate synthase																							
**SGO_0941**		1.322				1.322				1.322														
	hypothetical protein SGO_0941																							
**SGO_0942**		5.201				5.201				5.201														
	zmpC; zinc metalloproteinase C																							
**SGO_0944**		5.012				5.012				5.012														
	ABC transporter, ATP-binding protein																							
**SGO_0946**	0.714	7.288	0.0005	0.0007	-0.217	6.786	0.0285	0.1162	-0.622	6.632	0.0098	0.0625	-0.931	6.698	0.0008	0.0011	-1.336	5.975	0.0016	0.0033	-0.405	5.997	0.0811	0.2141
	Deblocking aminopeptidase																							
**SGO_0948**	0.086	6.180	0.0837	0.5279		5.141				5.141				4.705				4.094						
	pgdA; peptidoglycan N-acetylglucosamine deacetylase A																							
**SGO_0949**	-0.475	5.295	0.0377	0.2032	-0.476	5.268	0.0151	0.0552	-0.757	4.862	0.0284	0.2286	-0.001	4.522	0.1088	0.8182	-0.281	3.421	0.0875	0.6042	-0.280	3.985	0.0869	0.2354
	deaD; DEAD RNA helicase																							
**SGO_0950**	1.300	5.753	0.0324	0.1703	-0.275	4.609	0.1251	0.6319	-0.774	3.834			-1.575	5.402	0.0029	0.0089	-2.074	4.525	0.0255	0.1345	-0.499	3.856	0.1382	0.4363
	oxidoreductase																							
**SGO_0951**	0.064	6.011	0.1114	0.7288	-0.138	5.905	0.0714	0.3362	-0.083	5.935	0.0662	0.6035	-0.202	5.426	0.0329	0.2049	-0.146	4.844	0.0608	0.3924	0.056	5.347	0.1726	0.5963
	udk; uridine kinase																							
**SGO_0953**						5.155								4.642								4.642		
	endoribonuclease L-PSP, putative																							
**SGO_0954**	0.510	6.460	0.0041	0.0126	0.095	6.263	0.1169	0.5847	0.496	6.475	0.0164	0.1211	-0.415	6.016	0.0367	0.2361	-0.015	5.584	0.1274	0.9148	0.400	6.031	0.1104	0.3269
	ATP-binding protein																							
**SGO_0955**	-0.040	4.797	0.1102	0.7184	0.411	4.555	0.0759	0.3630		3.830			0.451	4.001	0.0127	0.0640		2.639				2.700		
	transporter																							
**SGO_0957**	-2.699	5.625	0.0124	0.0518		5.518				5.518				1.302				0.692						
	hypothetical protein SGO_0957																							

Log_2 Ratios

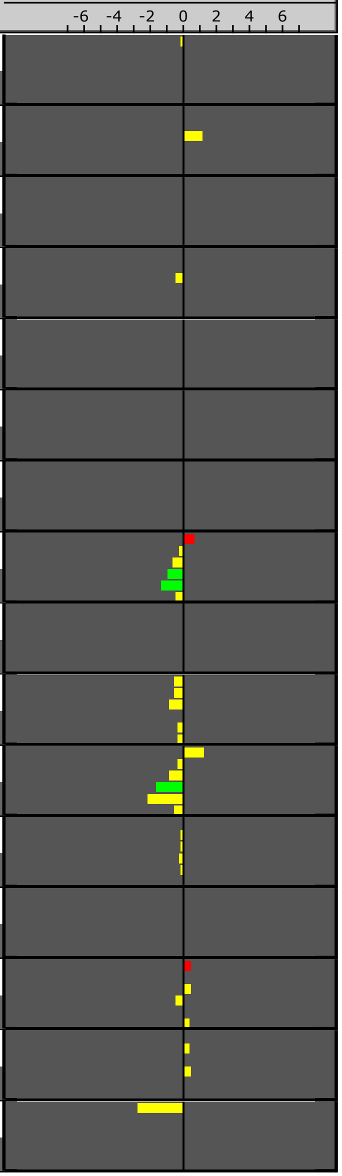

Show detected proteins only
Show all proteins
Filter by category:
ABC Transporter

Proteins found:
1179

Dot Plots
Dot Plots

Test	Cutoff
q-Value	.005
p-Value	

	Signif	Direction	Applies To
red	yes	+	ratios, bars
yellow	no	n/a	bars
green	yes	–	ratios, bars
light red	yes	+	*p*- , *q*-Values
light green	yes	–	

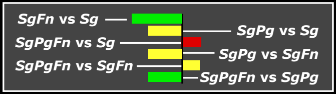

Streptococcus gordonii

Protein	*SgFn vs Sg* Ratio	Sum	*q*-Val	*p*-Val	*SgPg vs Sg* Ratio	Sum	*q*-Val	*p*-Val	*SgPgFn vs Sg* Ratio	Sum	*q*-Val	*p*-Val	*SgPg vs SgFn* Ratio	Sum	*q*-Val	*p*-Val	*SgPgFn vs SgFn* Ratio	Sum	*q*-Val	*p*-Val	*SgPgFn vs SgPg* Ratio	Sum	*q*-Val	*p*-Val	Description
**SGO_0959**	2.052	5.945	0.0046	0.0144	0.527	4.866	0.0492	0.2185		3.624			-1.525	5.534	0.0049	0.0184		4.499				3.560			copper homeostasis protein CutC
**SGO_0961**		3.776				3.776				3.776															ABC transporter, permease/ATP-binding protein
**SGO_0962**		1.841				1.841				1.841															ABC transporter, permease/ATP-binding protein
**SGO_0966**	-1.271	6.593	0.0003	0.0003		6.093				6.093				4.307				3.696							hsa; streptococcal hemagglutinin
**SGO_0969**	0.191	3.585	0.0379	0.2044		2.934				2.934				1.611				1.000							nss; nucleotide sugar synthetase-like protein
**SGO_0971**		2.609				2.609				2.609															asp1; accessory secretory protein
**SGO_0972**		1.585				1.585				1.585															asp2; accessory sescretory protein
**SGO_0975**		3.272				3.272				3.272															gtaA; glycosyl transferase, group 1 SP1758
**SGO_0979**		3.279				3.279				3.279															degV family protein
**SGO_0980**		0.643				0.643				0.643															transcriptional regulator, TetR family domain protein
**SGO_0981**	-1.139	4.303	0.0531	0.3053	-0.793	4.371	0.0772	0.3697		4.020			0.346	2.486				0.692				1.648			comE operon protein 2 family
**SGO_0982**	-2.577	8.302	0.0002	0.0002	-2.052	8.392	0.0004	0.0003	-3.500	8.201	0.0002	0.0002	0.524	6.278	0.0049	0.0186	-0.924	4.987	0.0004	0.0002	-1.448	5.968	0.0050	0.0022	amino acid ABC transporter, amino acid-binding protein
**SGO_0983**		5.795			-3.281	5.868	0.0096	0.0314		5.795				1.000								1.000			amino acid ABC transporter, ATP-binding protein SP0709
**SGO_0986**		3.992				3.992				3.992															hypothetical protein SGO_0986
**SGO_0987**	0.344	7.742	0.0021	0.0052	0.274	7.714	0.0366	0.1563	0.420	7.786	0.0008	0.0021	-0.070	7.365	0.0982	0.7269	0.076	6.818	0.0052	0.0176	0.146	7.404	0.1271	0.3950	metK; S-adenosylmethionine synthetase
**SGO_0991**		6.865			1.525	8.820				6.865				7.877								7.877			hypothetical protein SGO_0991

Log$_2$ Ratios

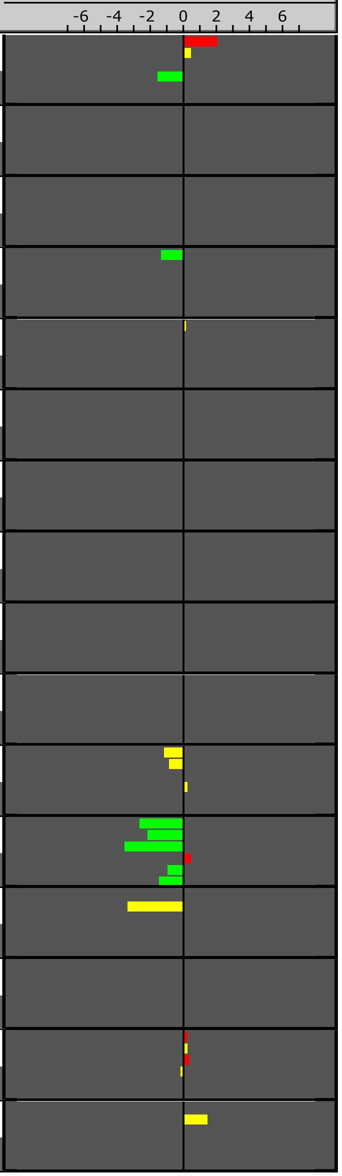

Show detected proteins only
Show all proteins
Filter by category:
ABC Transporter

Proteins found: 1179

Dot Plots
Dot Plots

Test	Cutoff
q-Value	.005
p-Value	

	Signif	Direction	Applies To
red	yes	+	ratios, bars
yellow	no	n/a	bars
green	yes	–	ratios, bars
light red	yes	+	*p*- , *q*-Values
light green	yes	–	

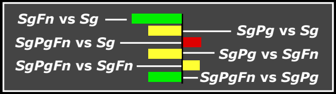

Hendrickson *et al.*

		SgFn vs Sg				*SgPg vs Sg*				*SgPgFn vs Sg*				*SgPg vs SgFn*				*SgPgFn vs SgFn*				*SgPgFn vs SgPg*			
Protein	Description	Ratio	Sum	*q*-Val	*p*-Val	Ratio	Sum	*q*-Val	*p*-Val	Ratio	Sum	*q*-Val	*p*-Val	Ratio	Sum	*q*-Val	*p*-Val	Ratio	Sum	*q*-Val	*p*-Val	Ratio	Sum	*q*-Val	*p*-Val
**SGO_0993**	GTP-binding protein HflX		3.772				2.098				2.396			-0.644	3.652	0.0469	0.3163	-0.346	3.118	0.0694	0.4602	0.298	2.742		
**SGO_0994**	hypothetical protein SGO_0994		2.718			0.996	4.243	0.0136	0.0479		2.718				3.114								3.114		
**SGO_0995**	metallo-beta-lactamase superfamily protein	1.419	6.066	0.0067	0.0234	0.171	5.395	0.1330	0.6801	-0.740	4.614	0.0526	0.4645	-1.248	5.629	0.0117	0.0566	-2.159	4.605	0.0271	0.1436	-0.910	4.300	0.1485	0.4762
**SGO_0996**	oxidoreductase, short-chain dehydrogenase/reductase family		3.351				3.351				3.351														
**SGO_0997**	hypothetical protein SGO_0997	-0.144	5.713	0.0609	0.3621	-1.025	5.451	0.0317	0.1312		4.781			-0.881	4.852	0.0342	0.2148		3.519				3.508		
**SGO_1000**	recJ; single-stranded-DNA-specific exonuclease RecJ	2.968	5.438	0.0074	0.0270	1.958	4.529	0.0329	0.1379		1.380			-1.010	5.423	0.0013	0.0025		4.224				3.844		
**SGO_1001**	apt; adenine phosphoribosyltransferase	1.289	8.028	0.0064	0.0223	1.111	7.883	0.0025	0.0058	0.748	7.654	0.0066	0.0376	-0.178	7.924	0.0588	0.4071	-0.540	7.161	0.0225	0.1147	-0.363	7.652	0.0482	0.1077
**SGO_1002**	metA; homoserine O-succinyltransferase		1.000				1.000				1.000														
**SGO_1003**	DNA replication protein DnaD	-0.915	5.035	0.0023	0.0059	0.078	5.464	0.1208	0.6096		4.424			0.993	4.573	0.0021	0.0054		2.376				3.989		
**SGO_1004**	glutathione S-transferase family protein	0.470	3.945	0.0697	0.4252	1.083	4.433	0.0635	0.2929		2.000			0.613	4.345	0.0244	0.1438		2.387				3.625		
**SGO_1005**	Bcl-2 family protein	0.530	5.015	0.0122	0.0505	0.994	5.306	0.0039	0.0102	0.682	5.098	0.0013	0.0047	0.464	4.993	0.0129	0.0658	0.152	4.203	0.0501	0.3034	-0.311	5.052	0.0394	0.0786
**SGO_1006**	conserved hypothetical protein TIGR00486	0.802	2.778			0.787	3.497	0.0576	0.2617		1.322			-0.015	3.203	0.1268	0.9734		1.000				2.622		
**SGO_1007**	oxidoreductase, DadA family protein SP1608						2.854								2.341								2.341		
**SGO_1009**	rfbA-1; glucose-1-phosphate thymidylyltransferase	2.074	9.379	0.0003	0.0003	1.501	8.934	0.0003	0.0002	1.149	8.693	0.0021	0.0088	-0.573	9.300	0.0019	0.0047	-0.925	8.563	0.0020	0.0047	-0.352	8.825	0.0358	0.0687
**SGO_1010**	rmlC; dTDP-4-keto-6-deoxyglucose-3,5-epimerase	0.815	7.342	0.0017	0.0041	0.919	7.414	0.0033	0.0084	0.915	7.403	0.0001	0.0001	0.105	7.238	0.0678	0.4775	0.100	6.619	0.0511	0.3146	-0.004	7.284	0.2348	0.8913
**SGO_1011**	rfbB-1; dTDP-glucose 4,6-dehydratase	2.376	9.181	0.0022	0.0055	1.150	8.223	0.0022	0.0049	0.641	7.895	0.0064	0.0362	-1.226	8.918	0.0039	0.0133	-1.735	8.174	0.0028	0.0076	-0.510	7.930	0.0092	0.0074

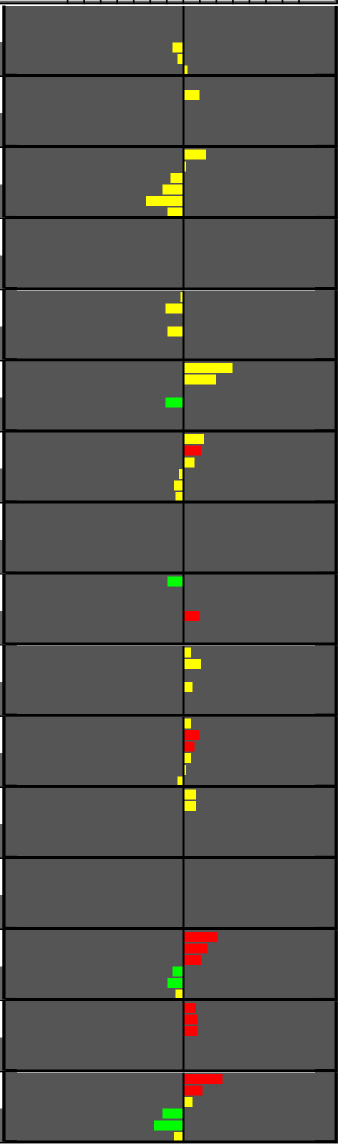

Show detected proteins only
Show all proteins
Filter by category:
ABC Transporter

Proteins found: 1179

Dot Plots
Dot Plots

Test	Cutoff
q-Value	.005
p-Value	

	Signif	Direction	Applies To
red	yes	+	ratios, bars
yellow	no	n/a	bars
green	yes	–	ratios, bars
pink	yes	+	*p*- , *q*-Values
light green	yes	–	*p*- , *q*-Values

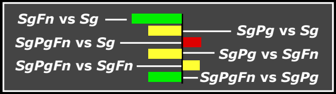

Hendrickson *et al.*

Spectral Counts
Sg Summary Table

Streptococcus gordonii

Hackett Laboratory UW

Summary Table | SgFn vs Sg | SgPg vs Sg | SgPgFn vs Sg | SgPg v SgFn | SgPgFn v SgFn | SgPgFn vs SgPg | Coverage

Protein	Description	*SgFn vs Sg*				*SgPg vs Sg*				*SgPgFn vs Sg*				*SgPg vs SgFn*				*SgPgFn vs SgFn*				*SgPgFn vs SgPg*			
		Ratio	Sum	*q*-Val	*p*-Val	Ratio	Sum	*q*-Val	*p*-Val	Ratio	Sum	*q*-Val	*p*-Val	Ratio	Sum	*q*-Val	*p*-Val	Ratio	Sum	*q*-Val	*p*-Val	Ratio	Sum	*q*-Val	*p*-Val
**SGO_1012**	galE-1; UDP-glucose 4-epimerase	1.633	9.235	0.0001	0.0000	0.993	8.779	0.0001	0.0000	0.585	8.521	0.0002	0.0002	-0.640	9.034	0.0002	0.0001	-1.048	8.277	0.0001	0.0000	-0.408	8.489	0.0012	0.0001
**SGO_1013**	Glycosyltransferase involved in cell wall biogenesis	-2.184	6.159	0.0202	0.0930	-2.901	6.102	0.0214	0.0839	-1.144	6.302	0.0175	0.1311	-0.717	2.988			1.040	3.303			1.757	3.716		
**SGO_1016**	putative glycosyltransferase	-1.977	5.619	0.0303	0.1580	-1.298	5.936	0.0047	0.0130	-1.705	5.652	0.0215	0.1691	0.679	4.010	0.0282	0.1722	0.272	2.464			-0.407	4.079	0.0910	0.2534
**SGO_1018**	hypothetical protein SGO_1018		3.669				3.669				3.669														
**SGO_1019**	glycosyl transferase	-2.526	6.462	0.0035	0.0105	-2.504	6.579	0.0001	0.0000	-3.361	6.410	0.0023	0.0096	0.022	3.918	0.1180	0.8916	-0.835	2.334			-0.856	3.694	0.0874	0.2378
**SGO_1020**	rfbD; dTDP-4-dehydrorhamnose reductase	1.139	7.938	0.0003	0.0002	0.660	7.639	0.0101	0.0334	0.517	7.544	0.0030	0.0137	-0.479	7.674	0.0085	0.0391	-0.622	6.999	0.0020	0.0046	-0.143	7.350	0.1361	0.4272
**SGO_1021**	rgpA; rhamnosyltransferase, putative		5.625				5.625				5.625														
**SGO_1022**	rhamnosyltransferase		5.593				5.593				5.593														
**SGO_1024**	putative polysaccharide ABC transporter	-2.758	6.575	0.0023	0.0063	-0.897	6.994	0.0133	0.0467		6.371			1.861	5.327	0.0044	0.0154		2.528				4.969		
**SGO_1025**	rgp; glycosyltransferase	-1.071	6.306	0.0103	0.0406	-0.018	6.700	0.1714	0.9072	-0.576	6.464	0.0131	0.0922	1.052	5.779	0.0087	0.0402	0.495	4.844	0.0527	0.3287	-0.558	5.938	0.0438	0.0934
**SGO_1026**	rhamnosyltransferase	-1.947	8.073	0.0048	0.0151	-2.178	8.025	0.0046	0.0126	-3.207	7.890	0.0021	0.0088	-0.231	6.138	0.0308	0.1900	-1.260	5.145	0.0030	0.0084	-1.030	5.584	0.0017	0.0002
**SGO_1027**	hypothetical protein SGO_1027		5.739				5.739				5.739														
**SGO_1028**	hypothetical protein SGO_1028		4.110				4.110				4.110														
**SGO_1030**	hypothetical protein SGO_1030		4.129				4.129				4.129														
**SGO_1031**	cmk; cytidylate kinase	-0.494	5.534	0.0028	0.0079	-0.051	5.735	0.1206	0.6069	-0.973	5.369	0.0025	0.0107	0.443	4.994	0.0067	0.0276	-0.479	3.944	0.0246	0.1287	-0.922	4.825	0.0158	0.0184
**SGO_1032**	infC; translation initiation factor IF-3		4.700			-0.408	5.564	0.0853	0.4151	-0.623	5.422	0.0007	0.0018		3.902				2.955			-0.215	4.744	0.1382	0.4357

Log$_2$ Ratios: -6 -4 -2 0 2 4 6

Show detected proteins only
Show all proteins
Filter by category:
ABC Transporter

Proteins found:
1179

Dot Plots
Dot Plots

Test	Cutoff
***q*-Value**	**.005**
p-Value	

	Signif	Direction	Applies To
red	yes	+	ratios, bars
yellow	no	n/a	bars
green	yes	–	ratios, bars
light red	yes	+	*p-* , *q*-Values
light green	yes	–	

SgFn vs Sg
SgPgFn vs Sg
SgPgFn vs SgFn
SgPg vs Sg
SgPg vs SgFn
SgPgFn vs SgPg

Hendrickson *et al.*

Streptococcus gordonii

Protein	SgFn vs Sg				SgPg vs Sg				SgPgFn vs Sg				SgPg vs SgFn				SgPgFn vs SgFn				SgPgFn vs SgPg				Description
	Ratio	Sum	*q*-Val	*p*-Val	Ratio	Sum	*q*-Val	*p*-Val	Ratio	Sum	*q*-Val	*p*-Val	Ratio	Sum	*q*-Val	*p*-Val	Ratio	Sum	*q*-Val	*p*-Val	Ratio	Sum	*q*-Val	*p*-Val	
**SGO_1033**	-0.590	7.556	0.0312	0.1631		6.858			-0.863	7.498	0.0208	0.1629		5.660			-0.273	5.973	0.0937	0.6504		5.504			rpmI; ribosomal protein L35
**SGO_1034**	0.007	9.370	0.1242	0.8190	1.019	9.964	0.0000	0.0000	0.317	9.575	0.0337	0.2807	1.012	9.453	0.0001	0.0000	0.310	8.455	0.0481	0.2893	-0.702	9.592	0.0321	0.0574	rplT; ribosomal protein L20
**SGO_1035**	1.252	6.060	0.0022	0.0058	0.688	5.690	0.0127	0.0439	0.599	5.642	0.0130	0.0914	-0.564	5.768	0.0013	0.0027	-0.653	5.126	0.0027	0.0070	-0.089	5.418	0.1403	0.4457	gloA; lactoylglutathione lyase
**SGO_1036**	-0.270	7.310	0.0668	0.4036	-1.212	6.966	0.0083	0.0259	-2.329	6.684	0.0011	0.0035	-0.942	6.292	0.0161	0.0871	-2.060	5.362	0.0045	0.0142	-1.118	5.294	0.0358	0.0685	amino acid ABC transporter, ATP-binding protein SP1242
**SGO_1037**	-2.158	5.957	0.0010	0.0019	-0.955	6.229	0.0002	0.0001		5.628			1.204	4.743	0.0083	0.0375		2.538				4.162			glutamine ABC transporter permease and substrate binding protein
**SGO_1038**	1.093	5.266	0.0097	0.0377	0.889	5.145	0.0229	0.0908	0.907	5.145	0.0101	0.0649	-0.205	5.088	0.0722	0.5173	-0.187	4.477	0.0694	0.4605	0.018	4.993	0.2552	0.9977	uvrB; excinuclease ABC, B subunit
**SGO_1039**		3.272				3.272				3.272															hypothetical protein SGO_1039
**SGO_1041**	1.365	4.298			0.719	4.572	0.0490	0.2172		2.459			-0.646	4.507	0.0268	0.1605		2.700				3.680			hypothetical protein SGO_1041
**SGO_1044**	2.231	3.094			2.045	3.796	0.0095	0.0310		0.585			-0.186	3.767	0.0278	0.1681		1.692				3.118			MutT/nudix family protein
**SGO_1047**	-2.388	5.361	0.0294	0.1515	0.514	6.503	0.0197	0.0765	-0.691	5.932	0.0115	0.0768	2.902	5.310	0.0170	0.0942	1.697	3.631	0.0356	0.2046	-1.205	5.745	0.0107	0.0100	hypothetical protein SGO_1047
**SGO_1049**		3.011			2.154	5.188	0.0027	0.0065	1.047	3.872	0.0552	0.4899		4.315				1.594			-1.108	4.615	0.0348	0.0652	tRNA pseudouridine synthase B
**SGO_1050**	1.494	3.254			1.530	4.146	0.0605	0.2783	1.263	3.088			0.036	3.963	0.1129	0.8522	-0.230	2.581			-0.267	3.894	0.1782	0.6234	ribF; riboflavin biosynthesis protein RibF
**SGO_1051**						3.576				1.981				3.063				0.857			-1.595	3.476			negative regulator of proteolysis
**SGO_1054**		3.033			0.144	3.662	0.1254	0.6353		3.033				1.648								1.648			NOL1/NOP2/sun family protein
**SGO_1055**		6.557			-1.995	6.887	0.0009	0.0014		6.557				4.083								4.083			phosphate ABC transporter, phosphate-binding protein
**SGO_1057**		2.585				2.585				2.585															pstA; phosphate ABC transporter, permease protein PstA

Log$_2$ Ratios

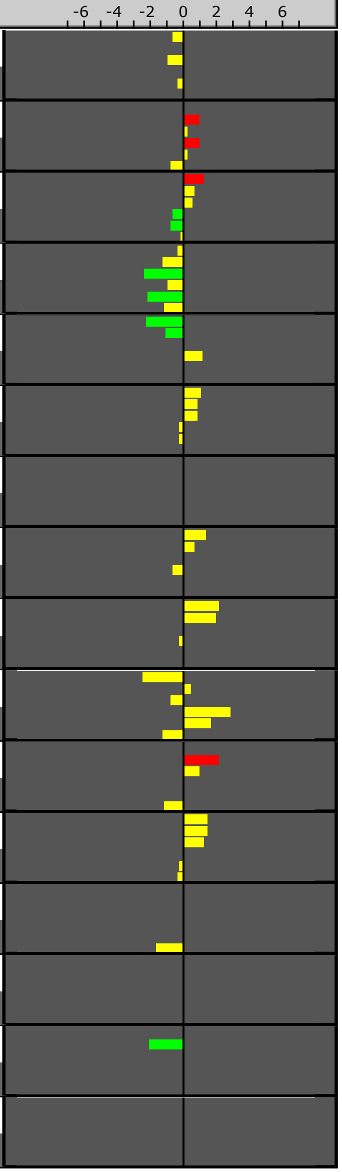

- Show detected proteins only
- Show all proteins
- Filter by category: ABC Transporter

Proteins found: 1179

Dot Plots
Dot Plots

Test	Cutoff
q-Value	.005
p-Value	

	Signif	Direction	Applies To
red	yes	+	ratios, bars
yellow	no	n/a	bars
green	yes	–	ratios, bars
light red	yes	+	*p*- , *q*-Values
light green	yes	–	*p*- , *q*-Values

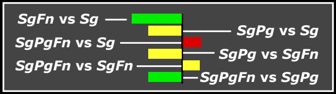

Streptococcus gordonii

Hackett Laboratory UW

	SgFn vs Sg				*SgPg vs Sg*				*SgPgFn vs Sg*				*SgPg vs SgFn*				*SgPgFn vs SgFn*				*SgPgFn vs SgPg*			
Protein	Ratio	Sum	*q*-Val	*p*-Val	Ratio	Sum	*q*-Val	*p*-Val	Ratio	Sum	*q*-Val	*p*-Val	Ratio	Sum	*q*-Val	*p*-Val	Ratio	Sum	*q*-Val	*p*-Val	Ratio	Sum	*q*-Val	*p*-Val
**SGO_1058**		6.104			-1.604	6.514	0.0002	0.0001	-0.384	6.571	0.0169	0.1253		3.985				3.594			1.220	5.099	0.0073	0.0050
	pstB; Phosphate import ATP-binding protein pstB 2 (Phosphate-transporting ATPase 2) (ABC phosphate transporter 2)																							
**SGO_1059**	-1.598	5.982	0.0001	0.0001	0.545	6.866	0.0004	0.0004	0.849	7.054	0.0001	0.0000	2.143	5.896	0.0001	0.0001	2.447	5.537	0.0001	0.0000	0.304	6.760	0.0048	0.0016
	pstB; Phosphate import ATP-binding protein pstB 1 (Phosphate-transporting ATPase 1) (ABC phosphate transporter 1)																							
**SGO_1060**	-1.641	7.306	0.0009	0.0016	-0.234	7.789	0.0257	0.1035	-1.276	7.399	0.0007	0.0018	1.407	6.618	0.0009	0.0014	0.365	5.333	0.0135	0.0606	-1.042	6.723	0.0043	0.0012
	phosphate transport system regulatory protein																							
**SGO_1065**	-0.762	6.548	0.0014	0.0031	-0.937	6.481	0.0002	0.0001	-0.993	6.465	0.0004	0.0009	-0.175	5.520	0.0316	0.1956	-0.231	4.888	0.0351	0.1995	-0.056	5.401	0.1849	0.6538
	hypothetical protein SGO_1065																							
**SGO_1066**	-0.160	6.695	0.0523	0.2992	-1.426	6.237	0.0027	0.0065		5.779			-1.266	5.599	0.0009	0.0016		4.482				3.844		
	hypothetical protein SGO_1066																							
**SGO_1067**	0.317	2.974				1.807				1.807				1.611				1.000						
	hypothetical protein SGO_1067																							
**SGO_1069**	0.425	8.560	0.0023	0.0062	0.674	8.712	0.0038	0.0100	0.331	8.508	0.0047	0.0248	0.250	8.375	0.0127	0.0644	-0.094	7.585	0.0233	0.1204	-0.344	8.334	0.0248	0.0395
	membrane alanyl aminopeptidase																							
**SGO_1072**	3.899	6.269	0.0019	0.0046	1.254	3.081				1.322			-2.645	5.819	0.0021	0.0051		5.097				2.063		
	ciaR; Transcriptional regulatory protein CiaR																							
**SGO_1073**	-0.816	5.409	0.0184	0.0834	-2.149	5.203	0.0133	0.0468		5.049			-1.332	3.200				2.107				1.385		
	Sensor protein CiaH																							
**SGO_1075**	2.151	5.320	0.0250	0.1226	0.098	3.050				2.000			-2.053	4.817	0.0216	0.1250		4.044				1.585		
	alpha-amylase precursor																							
**SGO_1077**	-0.574	5.673	0.1462	0.9937	-0.543	5.427	0.0040	0.0106		4.671			0.031	4.917	0.0724	0.5199		3.552				3.620		
	coaA; pantothenate kinase																							
**SGO_1078**		5.392				4.180							-1.252	5.397	0.0030	0.0090		4.268				3.667		
	methyltransferase domain protein																							
**SGO_1079**	0.866	7.696	0.0238	0.1139	-0.010	7.139	0.1705	0.8989	-0.826	6.789	0.0056	0.0302	-0.876	7.174	0.0185	0.1042	-1.692	6.333	0.0101	0.0421	-0.816	6.258	0.0107	0.0101
	pdp; pyrimidine-nucleoside phosphorylase																							
**SGO_1080**	-0.018	8.282	0.1194	0.7822	0.227	8.409	0.0119	0.0406	-0.127	8.229	0.0176	0.1329	0.245	7.887	0.0045	0.0165	-0.109	7.094	0.0180	0.0847	-0.354	7.838	0.0064	0.0039
	deoC; deoxyribose-phosphate aldolase																							
**SGO_1081**		3.660			-0.569	4.373	0.0588	0.2694	0.464	4.360	0.0716	0.6617		2.500				1.857			1.033	3.484	0.0735	0.1892
	cdd; cytidine deaminase																							
**SGO_1082**	-1.703	12.818	0.0002	0.0001	-0.805	13.082	0.0002	0.0001	-1.016	13.028	0.0024	0.0103	0.898	11.735	0.0011	0.0019	0.686	11.027	0.0202	0.0984	-0.212	12.037	0.1609	0.5401
	lipoprotein																							

Log$_2$ Ratios

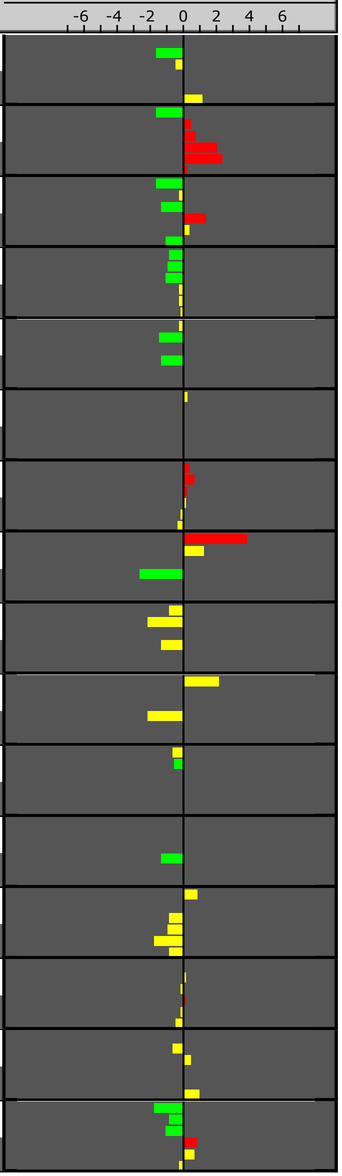

Show detected proteins only
Show all proteins
Filter by category:
ABC Transporter

Proteins found: 1179

Dot Plots
Dot Plots

Test	Cutoff
q-Value	.005
p-Value	

	Signif	Direction	Applies To
(red)	yes	+	ratios, bars
(yellow)	no	n/a	bars
(green)	yes	–	ratios, bars
(pink)	yes	+	*p*- , *q*-Values
(light green)	yes	–	

SgFn vs Sg
SgPgFn vs Sg
SgPgFn vs SgFn
SgPg vs Sg
SgPg vs SgFn
SgPgFn vs SgPg

Hendrickson *et al.*

Streptococcus gordonii

Protein	Description	*SgFn vs Sg* Ratio	Sum	*q*-Val	*p*-Val	*SgPg vs Sg* Ratio	Sum	*q*-Val	*p*-Val	*SgPgFn vs Sg* Ratio	Sum	*q*-Val	*p*-Val	*SgPg vs SgFn* Ratio	Sum	*q*-Val	*p*-Val	*SgPgFn vs SgFn* Ratio	Sum	*q*-Val	*p*-Val	*SgPgFn vs SgPg* Ratio	Sum	*q*-Val	*p*-Val
SGO_1083	sugar ABC transporter, ATP-binding protein SP0846		7.517				8.625				7.979			1.096	8.662	0.0021	0.0055	0.463	7.643	0.0001	0.0000	-0.634	8.825	0.0148	0.0168
SGO_1088	transcription regulator, LysR family		2.718			0.884	4.195	0.0275	0.1112	0.367	3.396	0.0737	0.6830		3.040				0.857			-0.517	3.458	0.1390	0.4396
SGO_1090	pseudouridine synthase, RluA family	-0.494	5.182	0.0601	0.3543	-0.245	5.633	0.0727	0.3447		4.751			0.249	4.491	0.0722	0.5174		2.107				3.991		
SGO_1091	hypothetical protein SGO_1091		2.022				2.022				2.022														
SGO_1096	butA; acetoin dehydrogenase	6.559	13.354	0.0001	0.0000	3.057	10.001	0.0001	0.0000	2.511	9.539	0.0010	0.0031	-3.502	12.948	0.0001	0.0001	-4.048	12.301	0.0002	0.0001	-0.546	10.083	0.0167	0.0208
SGO_1097	proB; glutamate 5-kinase	2.446	3.689				1.000				1.000				2.933				2.322						
SGO_1098	proA; gamma-glutamyl phosphate reductase	3.518	6.387	0.0016	0.0037	2.384	5.365	0.0434	0.1888	1.476	4.525	0.0115	0.0771	-1.135	6.369	0.0019	0.0043	-2.043	5.516	0.0001	0.0000	-0.908	5.326	0.0271	0.0453
SGO_1099	proC; pyrroline-5-carboxylate reductase	2.177	5.178	0.0740	0.4560		1.322				1.322				4.561				3.950						
SGO_1100	phnA; alkylphosphonate utilization operon protein PhnA		3.807				3.807				3.807														
SGO_1103	carA; carbamoyl-phosphate synthase, small subunit		3.944				3.944				3.944														
SGO_1104	carB; carbamoyl-phosphate synthase, large subunit	-0.461	4.724	0.0114	0.0463		4.277			0.289	4.965	0.0092	0.0571		2.302			0.750	3.115				3.053		
SGO_1107	PyrR bifunctional protein	-1.932	4.963	0.0370	0.1991	-0.775	5.151	0.0690	0.3237	-1.162	5.074	0.0311	0.2563	1.157	2.994			0.770	2.127			-0.387	3.279		
SGO_1109	pyrB; aspartate carbamoyltransferase	-1.562	7.655	0.0005	0.0008	-0.059	8.205	0.0928	0.4547	-0.527	7.996	0.0029	0.0129	1.503	7.095	0.0001	0.0000	1.035	6.157	0.0011	0.0017	-0.468	7.446	0.0077	0.0055
SGO_1110	surface antigen SCP-like domain	-2.330	6.283	0.0068	0.0242		6.147				6.147				2.302				1.692						
SGO_1111	fruR; phosphotransferase system repressor	-2.986	6.930	0.0008	0.0012	-2.353	7.016	0.0010	0.0016	-3.352	6.828	0.0127	0.0882	0.633	4.603	0.0021	0.0054	-0.366	3.112	0.0209	0.1038	-0.999	4.208	0.0466	0.1029
SGO_1112	fruB; 1-phosphofructokinase	-2.373	7.196	0.0001	0.0000	-1.735	7.327	0.0004	0.0004	-3.355	7.010	0.0028	0.0123	0.638	5.431	0.0121	0.0598	-0.983	3.774	0.0214	0.1074	-1.620	4.938	0.0735	0.1901

Log$_2$ Ratios

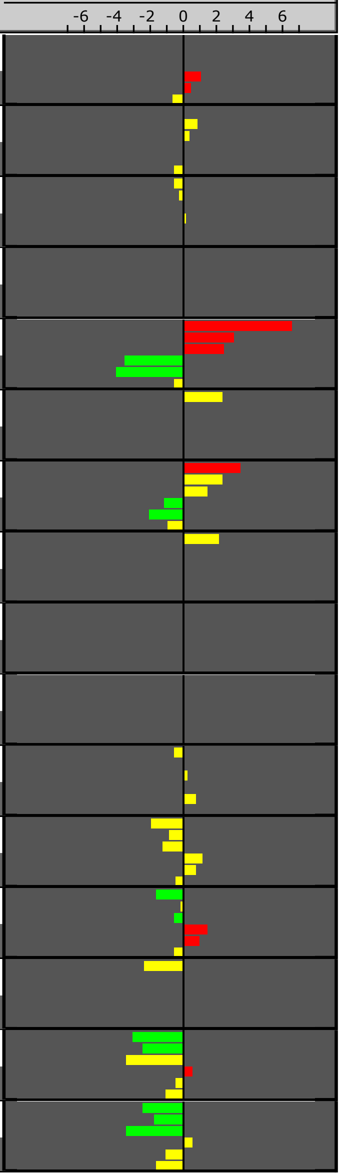

Show detected proteins only
Show all proteins
Filter by category:
ABC Transporter

Proteins found:
1179

Dot Plots
Dot Plots

Test	Cutoff
q-Value	.005
p-Value	

	Signif	Direction	Applies To
red	yes	+	ratios, bars
yellow	no	n/a	bars
green	yes	–	ratios, bars
light red	yes	+	*p*- , *q*-Values
light green	yes	–	

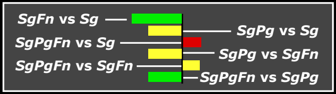

Hendrickson *et al.*

Streptococcus gordonii

	SgFn vs Sg				*SgPg vs Sg*				*SgPgFn vs Sg*				*SgPg vs SgFn*				*SgPgFn vs SgFn*				*SgPgFn vs SgPg*			
Protein	Ratio	Sum	*q*-Val	*p*-Val	Ratio	Sum	*q*-Val	*p*-Val	Ratio	Sum	*q*-Val	*p*-Val	Ratio	Sum	*q*-Val	*p*-Val	Ratio	Sum	*q*-Val	*p*-Val	Ratio	Sum	*q*-Val	*p*-Val
**SGO_1113**	-4.663	11.177	0.0000	0.0000	-3.955	11.208	0.0000	0.0000	-3.884	11.211	0.0000	0.0000	0.709	7.396	0.0246	0.1456	0.779	6.817	0.0182	0.0864	0.070	7.700	0.2139	0.7865
	fruA; PTS system, fructose specific IIABC components																							
**SGO_1114**	-2.537	6.777	0.0003	0.0002	-1.137	7.080	0.0005	0.0005	-2.238	6.815	0.0001	0.0001	1.400	5.373	0.0022	0.0060	0.299	4.065	0.0612	0.3976	-1.101	5.443	0.0051	0.0023
	Protein of unknown function (DUF1149) superfamily																							
**SGO_1115**	2.947	4.930			1.302	4.389	0.0357	0.1516		1.807			-1.645	4.960	0.0095	0.0441		3.630				3.612		
	DegV family protein																							
**SGO_1116**	2.419	6.592	0.0002	0.0001	2.860	6.972	0.0002	0.0001	2.399	6.582	0.0006	0.0013	0.440	7.068	0.0022	0.0059	-0.021	6.214	0.1274	0.9159	-0.461	7.063	0.0163	0.0196
	dapB; dihydrodipicolinate reductase																							
**SGO_1117**		4.237				3.130							-0.898	4.274	0.0269	0.1619		3.113				2.617		
	pcnA; polynucleotide adenylyltransferase																							
**SGO_1118**	1.378	4.957	0.0183	0.0825	-0.081	3.858	0.1344	0.6881		2.322			-1.459	4.639	0.0008	0.0011		3.580				2.735		
	ABC transporter, ATP-binding protein SP1553																							
**SGO_1119**		2.036				2.036				2.036														
	Abi-alpha protein, putative																							
**SGO_1120**	1.559	8.789	0.0013	0.0025	1.050	8.420	0.0004	0.0005	0.698	8.203	0.0049	0.0263	-0.509	8.618	0.0052	0.0203	-0.861	7.879	0.0041	0.0122	-0.352	8.177	0.0319	0.0570
	guaA; GMP synthase																							
**SGO_1121**		2.807			-0.972	3.402				2.807				1.322								1.322		
	transcription regulator, GntR family																							
**SGO_1123**	0.954	7.773	0.0005	0.0007	1.244	7.970	0.0002	0.0001	1.001	7.805	0.0004	0.0010	0.290	7.812	0.0038	0.0129	0.048	7.074	0.0814	0.5514	-0.243	7.834	0.0198	0.0276
	ffh; signal recognition particle protein																							
**SGO_1124**		3.025				3.025				3.025														
	cell surface hydrolase																							
**SGO_1128**		2.000				2.000				2.000														
	bacteriocin-associated integral membrane protein subfamily																							
**SGO_1129**	-0.262	6.672	0.0032	0.0095	-0.806	6.457	0.0025	0.0058	-0.672	6.501	0.0006	0.0016	-0.544	5.782	0.0047	0.0173	-0.410	5.221	0.0013	0.0022	0.133	5.556	0.1407	0.4477
	lplA; lipoate protein ligase A																							
**SGO_1130**	-3.046	7.970	0.0000	0.0000	-0.666	8.509	0.0002	0.0001	-0.959	8.402	0.0001	0.0000	2.380	6.880	0.0001	0.0000	2.087	6.027	0.0001	0.0000	-0.293	7.485	0.0045	0.0014
	dihydrolipoamide dehydrogenase																							
**SGO_1131**	-2.627	6.185	0.0028	0.0079	-0.208	6.974	0.0108	0.0364	-0.836	6.722	0.0014	0.0050	2.419	5.484	0.0116	0.0560	1.791	4.327	0.0283	0.1530	-0.628	6.083	0.0172	0.0217
	sucB; dihydrolipoamide S-acetyltransferase																							
**SGO_1132**		5.344			-0.631	6.047	0.0181	0.0688	-1.908	5.520	0.0242	0.1926		4.160				1.272			-1.277	4.431	0.0092	0.0072
	acetoin dehydrogenase																							

Log$_2$ Ratios

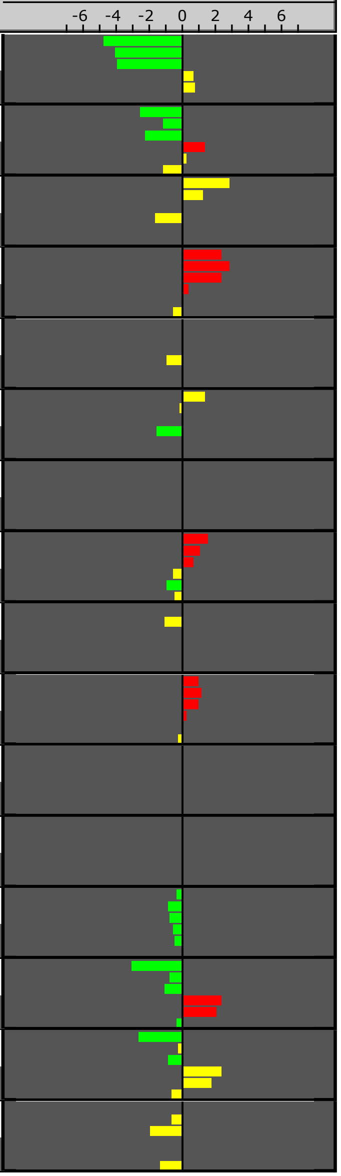

Show detected proteins only
Show all proteins
Filter by category:
ABC Transporter

Proteins found: 1179

Dot Plots
Dot Plots

Test	Cutoff
q-Value	.005
p-Value	

	Signif	Direction	Applies To
red	yes	+	ratios, bars
yellow	no	n/a	bars
green	yes	–	ratios, bars
pink	yes	+	*p*- , *q*-Values
light green	yes	–	

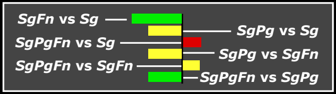

Spectral Counts
Sg Summary Table

Streptococcus gordonii

Hackett Laboratory UW

Summary Table | SgFn vs Sg | SgPg vs Sg | SgPgFn vs Sg | SgPg v SgFn | SgPgFn v SgFn | SgPgFn vs SgPg | Coverage

Protein	Description	*SgFn vs Sg* Ratio	Sum	*q*-Val	*p*-Val	*SgPg vs Sg* Ratio	Sum	*q*-Val	*p*-Val	*SgPgFn vs Sg* Ratio	Sum	*q*-Val	*p*-Val	*SgPg vs SgFn* Ratio	Sum	*q*-Val	*p*-Val	*SgPgFn vs SgFn* Ratio	Sum	*q*-Val	*p*-Val	*SgPgFn vs SgPg* Ratio	Sum	*q*-Val	*p*-Val
SGO_1133	acoA; acetoin dehydrogenase	-0.959	6.337	0.0317	0.1660	-0.813	6.658	0.0048	0.0135	-1.214	6.539	0.0027	0.0117	0.146	5.214	0.0706	0.5025	-0.255	4.367	0.0958	0.6663	-0.401	5.511	0.0753	0.1952
SGO_1134	hypothetical protein SGO_1134	2.321	6.119	0.0067	0.0238	-0.566	3.898	0.0626	0.2887	0.121	4.103	0.0782	0.7301	-2.886	5.443	0.0357	0.2287	-2.199	4.884	0.0423	0.2490	0.687	2.769		
SGO_1139	GTP-binding protein	0.405	6.420	0.0116	0.0478	1.267	6.969	0.0006	0.0007	0.580	6.523	0.0034	0.0164	0.862	6.582	0.0002	0.0001	0.176	5.568	0.0074	0.0275	-0.687	6.647	0.0017	0.0002
SGO_1140	clpX; ATP-dependent Clp protease, ATP-binding subunit ClpX	0.128	8.431	0.0388	0.2102	0.482	8.625	0.0017	0.0033	-0.066	8.332	0.0435	0.3780	0.354	8.166	0.0015	0.0034	-0.194	7.274	0.0080	0.0304	-0.548	8.083	0.0007	0.0000
SGO_1141	folA; dihydrofolate reductase	2.133	6.005	0.0060	0.0203	0.710	4.941	0.0144	0.0518	0.201	4.191	0.0695	0.6387	-1.422	5.648	0.0080	0.0352	-1.932	4.762	0.0407	0.2387	-0.510	4.166	0.1108	0.3287
SGO_1143	thyA; thymidylate synthase	0.391	5.180	0.0534	0.3075	1.268	5.705	0.0018	0.0037	0.799	4.852	0.0299	0.2442	0.877	5.319	0.0041	0.0140	0.407	3.951	0.0709	0.4709	-0.469	5.119	0.0256	0.0415
SGO_1144	glcK; glucokinase	2.268	9.727	0.0002	0.0001	1.912	9.437	0.0000	0.0000	1.707	9.282	0.0004	0.0009	-0.356	9.774	0.0022	0.0056	-0.562	9.079	0.0024	0.0060	-0.205	9.487	0.0330	0.0604
SGO_1145	cell surface hydrolase		3.781				3.781				3.781														
SGO_1148	cshB; surface-associated protein CshB	-2.650	8.527	0.0003	0.0003		8.314				8.314				5.151				4.540						
SGO_1149	Pneumococcal vaccine antigen A-like protein		3.830				3.830				3.830														
SGO_1150	hypothetical protein SGO_1150	0.600	4.826	0.0397	0.2158	1.321	5.394	0.0177	0.0671	0.585	4.130			0.721	5.305	0.0023	0.0062	-0.014	3.870	0.1271	0.9104	-0.736	4.996	0.0458	0.1000
SGO_1151	glyA; serine hydroxymethyltransferase	2.287	9.772	0.0000	0.0000	1.725	9.323	0.0000	0.0000	1.614	9.238	0.0001	0.0000	-0.562	9.736	0.0001	0.0000	-0.674	9.082	0.0002	0.0001	-0.112	9.374	0.0151	0.0172
SGO_1154	prfA; peptide chain release factor 1	0.128	7.465	0.0957	0.6170	0.348	7.523	0.0322	0.1340	0.523	7.625	0.0085	0.0520	0.220	7.107	0.1010	0.7485	0.396	6.592	0.0614	0.4010	0.176	7.252	0.0328	0.0595
SGO_1155	tdk; thymidine kinase	-1.087	5.504	0.0219	0.1031	0.258	6.054	0.1207	0.6079	0.196	5.567	0.0922	0.8799	1.345	5.132	0.0082	0.0365	1.283	3.830	0.0310	0.1716	-0.062	5.189	0.2203	0.8197
SGO_1159	guaC; guanosine monophosphate reductase	2.423	4.557	0.0157	0.0696	1.630	3.910	0.0590	0.2706		1.000			-0.794	4.599	0.0100	0.0472		3.305				3.191		
SGO_1163	Iron permease FTR1 family										1.981								0.857				1.468		

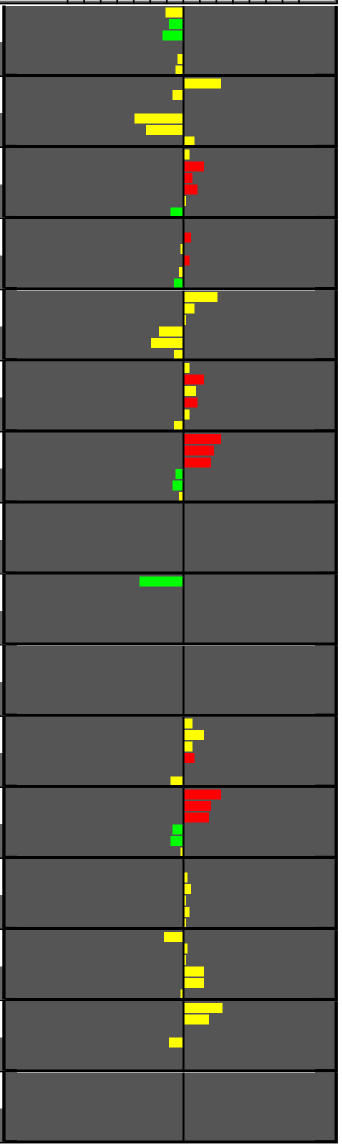

Show detected proteins only
Show all proteins
Filter by category:
ABC Transporter

Proteins found: 1179

Dot Plots
Dot Plots

Test	Cutoff
q-Value	.005
p-Value	

Color	Signif	Direction	Applies To
red	yes	+	ratios, bars
yellow	no	n/a	bars
green	yes	–	ratios, bars
pink	yes	+	*p*- , *q*-Values
light green	yes	–	

SgFn vs Sg — SgPg vs Sg
SgPgFn vs Sg — SgPg vs SgFn
SgPgFn vs SgFn — SgPgFn vs SgPg

Hendrickson *et al.*

Streptococcus gordonii

Hackett Laboratory UW

	SgFn vs Sg				*SgPg vs Sg*				*SgPgFn vs Sg*				*SgPg vs SgFn*				*SgPgFn vs SgFn*				*SgPgFn vs SgPg*			
Protein	Ratio	Sum	*q*-Val	*p*-Val	Ratio	Sum	*q*-Val	*p*-Val	Ratio	Sum	*q*-Val	*p*-Val	Ratio	Sum	*q*-Val	*p*-Val	Ratio	Sum	*q*-Val	*p*-Val	Ratio	Sum	*q*-Val	*p*-Val
**SGO_1166**		3.272				3.272				3.272														
voltage-gated chloride channel family protein																								
**SGO_1167**	-1.132	7.433	0.0004	0.0005	0.927	8.426	0.0002	0.0001	-0.299	7.772	0.0371	0.3123	2.059	7.614	0.0000	0.0000	0.833	6.143	0.0129	0.0571	-1.225	7.832	0.0061	0.0037
nox; NADH oxidase																								
**SGO_1169**	-0.745	6.887	0.0056	0.0186	0.631	7.557	0.0079	0.0244	0.557	7.504	0.0004	0.0009	1.376	6.806	0.0027	0.0078	1.302	6.132	0.0011	0.0015	-0.074	7.287	0.1579	0.5196
NADPH-dependent FMN reductase																								
**SGO_1170**	-2.073	8.329	0.0001	0.0000	-0.581	8.759	0.0002	0.0001	-0.745	8.696	0.0002	0.0002	1.493	7.367	0.0002	0.0001	1.328	6.638	0.0006	0.0005	-0.164	7.848	0.0268	0.0445
NADPH-dependent FMN reductase																								
**SGO_1171**	-3.650	7.609	0.0001	0.0001	-4.040	7.574	0.0001	0.0000		7.489			-0.390	4.216	0.0429	0.2851		2.839				2.937		
thioredoxin family protein																								
**SGO_1174**		3.417				3.417				3.417														
histidine kinase																								
**SGO_1176**		0.585				0.585				0.585														
peptide methionine sulfoxide reductase-like protein																								
**SGO_1182**		1.058				1.058				1.058														
LPXTG cell wall surface protein																								
**SGO_1184**		3.412				3.412				3.412														
GdmH																								
**SGO_1185**	-0.148	4.148	0.1009	0.6525	1.029	5.215	0.0048	0.0134	0.351	4.801	0.0232	0.1848	1.176	4.409	0.0260	0.1546	0.498	3.270	0.0501	0.3038	-0.678	4.825	0.0219	0.0318
acetyltransferase, GNAT family																								
**SGO_1186**		5.718				5.718				5.718														
ABC transporter, permease/ATP-binding protein																								
**SGO_1187**		6.301				6.301				6.301														
ABC transporter, permease/ATP-binding protein																								
**SGO_1189**	-2.586	9.364	0.0003	0.0004	-3.706	9.249	0.0003	0.0003	-3.464	9.278	0.0003	0.0004	-1.120	6.592	0.0013	0.0026	-0.877	6.105	0.0154	0.0708	0.243	6.125	0.1382	0.4349
lipoprotein, putative																								
**SGO_1191**	-1.030	11.163	0.0007	0.0010	0.125	11.651	0.0327	0.1371	0.717	11.990	0.0008	0.0022	1.155	10.733	0.0002	0.0001	1.746	10.556	0.0004	0.0002	0.592	11.525	0.0050	0.0022
rplL; ribosomal protein L7/L12																								
**SGO_1192**	-0.243	9.189	0.0417	0.2316	0.701	9.702	0.0111	0.0375	-0.130	9.230	0.0106	0.0690	0.944	9.110	0.0071	0.0297	0.112	7.996	0.0872	0.5997	-0.831	9.141	0.0196	0.0270
BL5; 50S ribosomal protein L10																								
**SGO_1193**	1.446	6.759	0.0004	0.0005	0.996	6.452	0.0037	0.0096	-0.784	5.522	0.0004	0.0007	-0.451	6.593	0.0082	0.0371	-2.230	5.464	0.0004	0.0003	-1.780	5.724	0.0061	0.0036
gid; Glucose inhibited division protein A																								

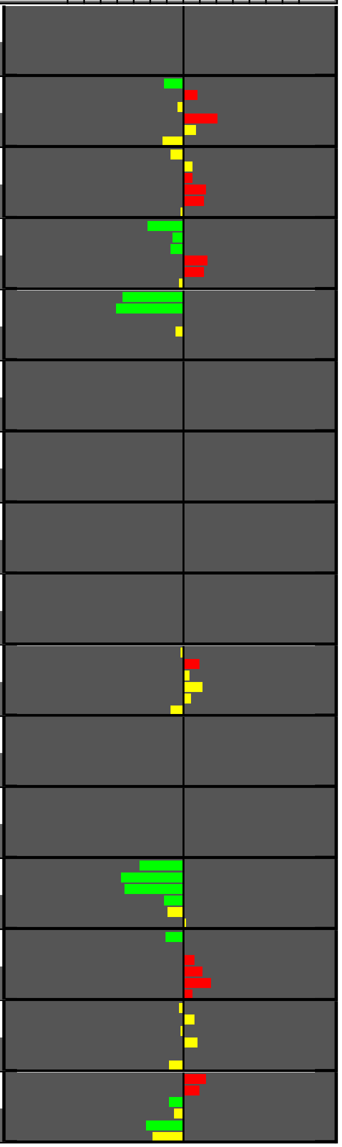

- Show detected proteins only
- Show all proteins
- Filter by category:
- ABC Transporter

Proteins found:
1179

Dot Plots
Dot Plots

Test	Cutoff
q-Value	.005
p-Value	

	Signif	Direction	Applies To
red	yes	+	ratios, bars
yellow	no	n/a	bars
green	yes	–	ratios, bars
pink	yes	+	*p*- , *q*-Values
light green	yes	–	

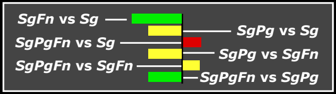

Streptococcus gordonii

Protein	Description	SgFn vs Sg Ratio	SgFn vs Sg Sum	SgFn vs Sg *q*-Val	SgFn vs Sg *p*-Val	SgPg vs Sg Ratio	SgPg vs Sg Sum	SgPg vs Sg *q*-Val	SgPg vs Sg *p*-Val	SgPgFn vs Sg Ratio	SgPgFn vs Sg Sum	SgPgFn vs Sg *q*-Val	SgPgFn vs Sg *p*-Val	SgPg vs SgFn Ratio	SgPg vs SgFn Sum	SgPg vs SgFn *q*-Val	SgPg vs SgFn *p*-Val	SgPgFn vs SgFn Ratio	SgPgFn vs SgFn Sum	SgPgFn vs SgFn *q*-Val	SgPgFn vs SgFn *p*-Val	SgPgFn vs SgPg Ratio	SgPgFn vs SgPg Sum	SgPgFn vs SgPg *q*-Val	SgPgFn vs SgPg *p*-Val
SGO_1196	satD; SatD-like protein		2.227				2.227			1.358	4.061								2.461				3.072		
SGO_1197	topA; DNA topoisomerase I	-1.919	7.885	0.0011	0.0021	-1.218	8.057	0.0018	0.0038	-1.756	7.913	0.0006	0.0017	0.702	6.511	0.0076	0.0330	0.163	5.590	0.0770	0.5164	-0.539	6.562	0.0140	0.0154
SGO_1198	DNA processing Smf protein		8.008			-0.387	8.825	0.0055	0.0161	-0.145	8.938	0.0296	0.2408		7.103				6.739			0.242	8.232	0.0289	0.0498
SGO_1199	acetyltransferase, CysE/LacA/LpxA/NodL family		2.934			-0.356	3.409	0.0322	0.1336		2.934				1.063								1.063		
SGO_1202	GTP-binding protein	0.838	5.772	0.0078	0.0290	0.592	5.619	0.0154	0.0563		4.275			-0.246	5.510	0.0543	0.3719		4.017				4.383		
SGO_1203	anaerobic ribonucleotide reductase	1.365	7.031	0.0011	0.0022	0.746	6.612	0.0027	0.0066	0.435	6.426	0.0071	0.0404	-0.619	6.766	0.0034	0.0112	-0.930	6.041	0.0020	0.0048	-0.311	6.275	0.0295	0.0509
SGO_1205	dapA; dihydrodipicolinate synthase	1.087	5.095	0.0245	0.1182	0.892	4.892	0.0147	0.0533	0.719	4.781	0.0129	0.0899	-0.195	4.889	0.0680	0.4795	-0.368	4.202	0.0507	0.3092	-0.173	4.641	0.1033	0.2994
SGO_1206	asd; aspartate-semialdehyde dehydrogenase	0.207	7.665	0.0140	0.0604	-0.353	7.392	0.0040	0.0107	0.164	7.642	0.0130	0.0915	-0.559	6.998	0.0013	0.0029	-0.043	6.619	0.0817	0.5556	0.517	6.972	0.0053	0.0027
SGO_1208	Hypothetical FAD-dependent oxidoreductase, putative		3.604				3.604				3.604														
SGO_1210	fhs-1; formate--tetrahydrofolate ligase	1.322	6.338	0.0019	0.0045	0.250	5.689	0.0723	0.3419	-0.802	4.885	0.0098	0.0626	-1.072	5.922	0.0057	0.0223	-2.125	4.887	0.0218	0.1097	-1.052	4.625	0.1103	0.3245
SGO_1213	coaC; phosphopantothenoylcysteine decarboxylase	-1.696	5.911	0.0075	0.0273	-2.296	5.647	0.0472	0.2066		5.513			-0.601	3.733	0.0674	0.4737		2.734				1.648		
SGO_1215	manB; phosphomannomutase	0.991	10.269	0.0005	0.0007	0.815	10.153	0.0002	0.0001	0.239	9.813	0.0014	0.0051	-0.176	10.082	0.0080	0.0351	-0.753	9.229	0.0009	0.0010	-0.577	9.730	0.0000	0.0000
SGO_1216	bta; Possible bacteriocin transport accessory protein	1.372	6.913	0.0009	0.0017	1.790	7.228	0.0010	0.0016	1.150	6.754	0.0004	0.0009	0.419	7.152	0.0080	0.0349	-0.222	6.206	0.0131	0.0582	-0.641	7.060	0.0103	0.0094
SGO_1219	pta; phosphate acetyltransferase	0.989	8.278	0.0179	0.0802	0.089	7.683	0.0652	0.3035	-0.058	7.613	0.0681	0.6240	-0.901	7.792	0.0163	0.0896	-1.048	7.136	0.0161	0.0745	-0.147	7.143	0.0656	0.1621
SGO_1221	ppnK; Probable inorganic polyphosphate/ATP-NAD kinase	-2.962	7.363	0.0009	0.0017		7.145				7.145				4.019				3.408						
SGO_1222	GTP pyrophosphokinase-like protein	-1.021	4.154	0.0070	0.0250	-0.323	4.321	0.0170	0.0631		3.836			0.698	2.693				0.692				2.000		

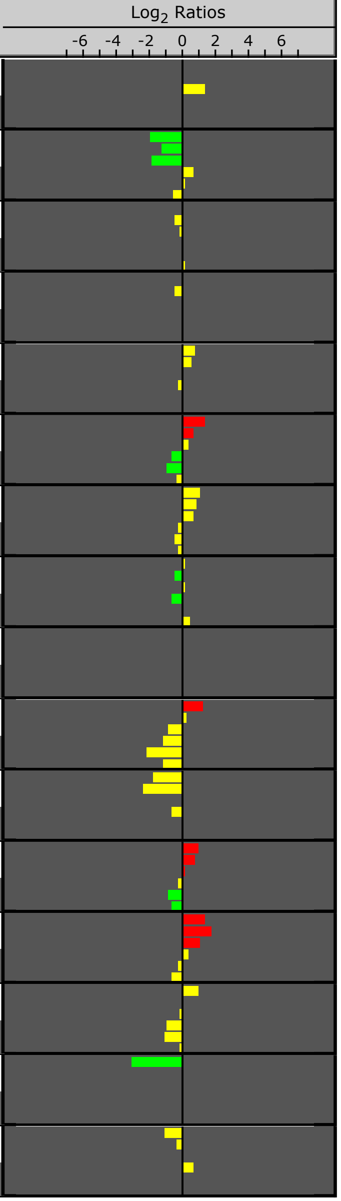

Show detected proteins only
Show all proteins
Filter by category:
ABC Transporter

Proteins found:
1179

Dot Plots
Dot Plots

Test	Cutoff
q-Value	.005
p-Value	

Color	Signif	Direction	Applies To
red	yes	+	ratios, bars
yellow	no	n/a	bars
green	yes	–	ratios, bars
light red	yes	+	*p*- , *q*-Values
light green	yes	–	*p*- , *q*-Values

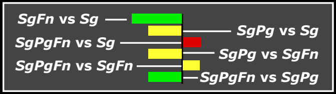

Hendrickson *et al.*

Protein	Description	*SgFn vs Sg* Ratio	Sum	*q*-Val	*p*-Val	*SgPg vs Sg* Ratio	Sum	*q*-Val	*p*-Val	*SgPgFn vs Sg* Ratio	Sum	*q*-Val	*p*-Val	*SgPg vs SgFn* Ratio	Sum	*q*-Val	*p*-Val	*SgPgFn vs SgFn* Ratio	Sum	*q*-Val	*p*-Val	*SgPgFn vs SgPg* Ratio	Sum	*q*-Val	*p*-Val
SGO_1224	Ribose-phosphate pyrophosphokinase 2	-0.808	8.161	0.0001	0.0001	0.647	8.869	0.0008	0.0012	0.425	8.740	0.0033	0.0157	1.455	8.092	0.0003	0.0002	1.233	7.325	0.0011	0.0015	-0.222	8.539	0.0384	0.0755
SGO_1225	pyridoxal-phosphate dependent aminotransferase	0.444	5.289	0.0259	0.1290	0.546	5.333	0.0081	0.0252	0.465	5.279	0.0018	0.0070	0.103	5.035	0.1024	0.7602	0.021	4.378	0.1295	0.9390	-0.082	5.027	0.1579	0.5157
SGO_1226	hypothetical protein SGO_1226	0.554	6.560	0.0306	0.1599	0.436	6.473	0.0366	0.1558	0.231	6.360	0.0532	0.4709	-0.118	6.216	0.0701	0.4969	-0.323	5.511	0.0327	0.1832	-0.205	6.039	0.0203	0.0288
SGO_1228	DNA-binding protein BH0551	1.028	5.079	0.0021	0.0053	0.534	4.773	0.0171	0.0636		3.494			-0.494	4.758	0.0045	0.0167		3.371				3.494		
SGO_1230	srtA; sortase A		6.030			-2.690	6.240	0.0003	0.0003		6.030				2.844								2.844		
SGO_1231	gyrA; DNA gyrase, A subunit	-0.634	8.407	0.0061	0.0209	-1.323	8.182	0.0024	0.0054	-1.868	8.032	0.0004	0.0008	-0.689	7.258	0.0135	0.0698	-1.234	6.445	0.0028	0.0078	-0.544	6.626	0.0495	0.1114
SGO_1232	L-lactate dehydrogenase	2.961	10.386	0.0001	0.0000	2.941	10.367	0.0001	0.0000	3.026	10.443	0.0000	0.0000	-0.020	10.687	0.0601	0.4179	0.065	10.119	0.0078	0.0295	0.085	10.720	0.0113	0.0108
SGO_1234	rpsA; 30S ribosomal protein S1	-0.416	10.488	0.0136	0.0581	0.414	10.895	0.0030	0.0073	0.581	10.993	0.0008	0.0023	0.829	10.222	0.0014	0.0032	0.997	9.720	0.0011	0.0016	0.167	10.658	0.0048	0.0016
SGO_1237	hypothetical protein SGO_1237		5.031			0.373	6.228	0.0055	0.0159	0.822	6.561	0.0153	0.1106		4.888				4.824			0.449	6.187	0.0848	0.2281
SGO_1238	ilvE; branched-chain amino acid aminotransferase	1.196	7.844	0.0074	0.0267	0.021	7.106	0.1464	0.7601	-0.322	6.950	0.0122	0.0839	-1.175	7.336	0.0066	0.0269	-1.517	6.634	0.0056	0.0194	-0.343	6.447	0.0305	0.0530
SGO_1239	parC; DNA topoisomerase IV, A subunit	-2.435	6.547	0.0006	0.0010	-1.117	6.822	0.0003	0.0003	-2.990	6.446	0.0001	0.0001	1.318	5.182	0.0052	0.0202	-0.555	3.569	0.0403	0.2359	-1.874	4.994	0.0012	0.0001
SGO_1241	aminoglycoside adenylyltransferase		2.946				2.946				2.946														
SGO_1242	lipoprotein, putative		7.113			-2.894	7.207	0.0175	0.0659	-2.541	7.232	0.0106	0.0688		2.700				2.442			0.352	3.887		
SGO_1244	hypothetical protein SGO_1244		4.043			0.288	5.085	0.1646	0.8635		4.043				3.612								3.612		
SGO_1245	parE; DNA topoisomerase IV, B subunit		5.347			-1.278	5.846	0.0026	0.0059		5.347				3.560								3.560		
SGO_1246	hypothetical protein SGO_1246		3.940				3.940				3.940														

Log_2 Ratios

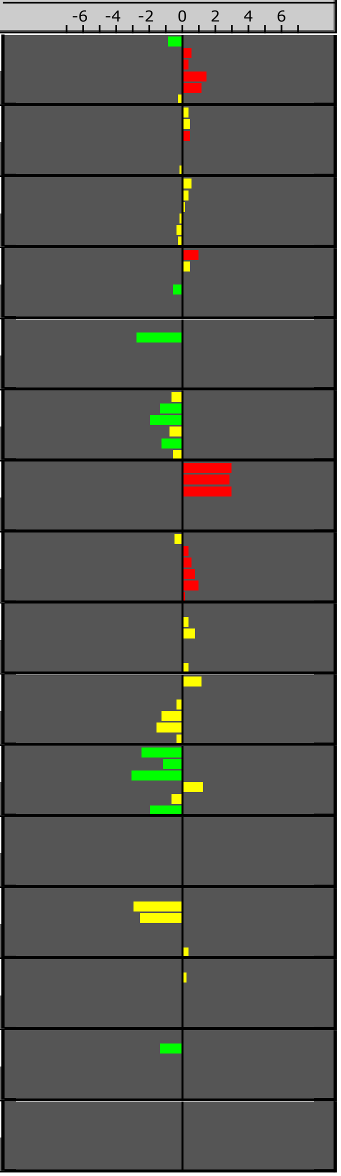

Show detected proteins only
Show all proteins
Filter by category:
ABC Transporter

Proteins found: 1179

Dot Plots
Dot Plots

Test	Cutoff
***q*-Value**	**.005**
p-Value	

	Signif	Direction	Applies To
red	yes	+	ratios, bars
yellow	no	n/a	bars
green	yes	−	ratios, bars
pink	yes	+	*p*- , *q*-Values
light green	yes	−	

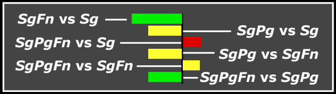

Protein	*SgFn vs Sg* Ratio	Sum	*q*-Val	*p*-Val	*SgPg vs Sg* Ratio	Sum	*q*-Val	*p*-Val	*SgPgFn vs Sg* Ratio	Sum	*q*-Val	*p*-Val	*SgPg vs SgFn* Ratio	Sum	*q*-Val	*p*-Val	*SgPgFn vs SgFn* Ratio	Sum	*q*-Val	*p*-Val	*SgPgFn vs SgPg* Ratio	Sum	*q*-Val	*p*-Val
**SGO_1247** 5'-nucleotidase family protein	-1.887	9.490	0.0001	0.0000		9.144				9.144				6.747				6.136						
**SGO_1248** pyrC; dihydroorotase	1.629	6.962	0.0102	0.0400	0.664	6.259	0.0221	0.0871	-0.668	5.583	0.0094	0.0593	-0.965	6.635	0.0161	0.0872	-2.297	5.699	0.0069	0.0249	-1.332	5.511	0.0136	0.0148
**SGO_1250** ung; uracil-DNA glycosylase	0.002	5.377	0.1241	0.8174		4.412				4.412				3.829				3.218						
**SGO_1251** hypothetical protein SGO_1251		1.841				1.841				1.841														
**SGO_1252** integral membrane protein, putative		1.322				1.322				1.322														
**SGO_1253** pyrE; orotate phosphoribosyltransferase	-0.416	6.262	0.0462	0.2609	0.695	6.813	0.0038	0.0099	0.357	6.621	0.0183	0.1387	1.111	6.169	0.0032	0.0103	0.773	5.347	0.0134	0.0603	-0.339	6.446	0.0353	0.0668
**SGO_1254** pyrF; orotidine 5'-phosphate decarboxylase		4.699			-1.011	5.018	0.0244	0.0978		4.699				2.170								2.170		
**SGO_1255** pyrd; dihydroorotate dehydrogenase B		2.824			-0.177	3.313	0.1403	0.7201		2.824				1.000								1.000		
**SGO_1257** probable transcriptional regulator (LysR family)	1.286	5.778	0.0098	0.0384	0.296	5.116	0.0467	0.2042		3.977			-0.991	5.347	0.0106	0.0504		4.167				3.730		
**SGO_1258** NAD-dependent deacetylase (Regulatory protein SIR2-like protein)	-0.144	4.608	0.0885	0.5643		3.718				3.718				2.976				2.366						
**SGO_1260** deoD; purine nucleoside phosphorylase	-0.908	7.764	0.0001	0.0001	0.150	8.225	0.0058	0.0171	0.150	8.227	0.0123	0.0845	1.058	7.351	0.0002	0.0002	1.059	6.742	0.0009	0.0010	0.001	7.787	0.2478	0.9628
**SGO_1261** glutamine amidotransferase, class I		1.807				1.807				1.807														
**SGO_1263** purine nucleoside phosphorylase I, inosine and guanosine-specific	-1.173	8.218	0.0007	0.0011	0.330	8.861	0.0049	0.0137	-0.436	8.496	0.0106	0.0693	1.503	7.938	0.0002	0.0001	0.737	6.819	0.0083	0.0316	-0.766	8.178	0.0100	0.0088
**SGO_1264** deoB; phosphopentomutase	0.143	10.075	0.0658	0.3970	0.019	10.003	0.1710	0.9034	-0.125	9.941	0.0572	0.5115	-0.124	9.567	0.0535	0.3653	-0.269	8.897	0.0403	0.2361	-0.144	9.434	0.1271	0.3944
**SGO_1265** rpiA; ribose 5-phosphate isomerase	-0.125	7.240	0.0847	0.5356	0.958	7.844	0.0077	0.0238	0.677	7.670	0.0125	0.0861	1.083	7.260	0.0003	0.0002	0.802	6.467	0.0014	0.0026	-0.281	7.570	0.0190	0.0257
**SGO_1266** trmE; tRNA modification GTPase TrmE	0.968	6.266	0.0005	0.0007	0.882	6.214	0.0025	0.0058	0.563	6.020	0.0059	0.0320	-0.086	6.119	0.0707	0.5041	-0.405	5.365	0.0117	0.0504	-0.319	5.932	0.0570	0.1353

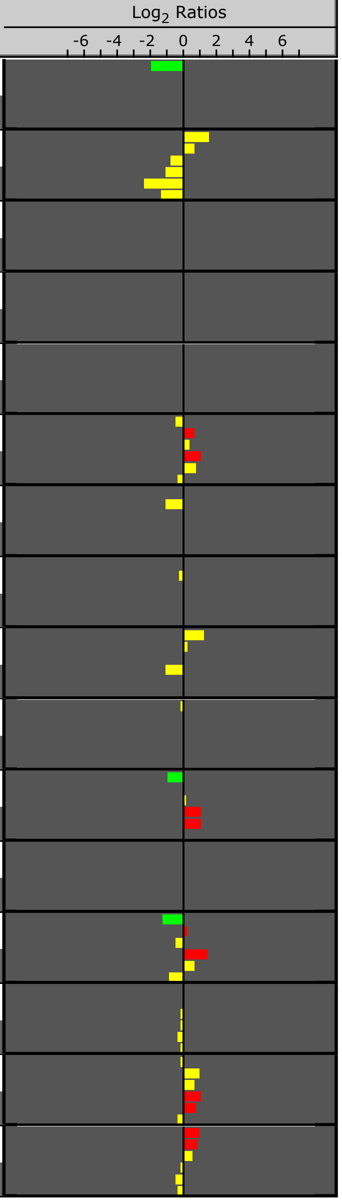

Show detected proteins only
Show all proteins
Filter by category:
ABC Transporter

Proteins found:
1179

Dot Plots
Dot Plots

Test	Cutoff
q-Value	.005
p-Value	

	Signif	Direction	Applies To
(red)	yes	+	ratios, bars
(yellow)	no	n/a	bars
(green)	yes	–	ratios, bars
(pink)	yes	+	*p*- , *q*-Values
(light green)	yes	–	

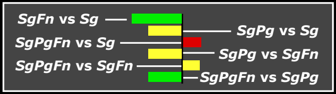

Hendrickson *et al.*

Streptococcus gordonii

Summary Table | SgFn vs Sg | SgPg vs Sg | SgPgFn vs Sg | SgPg v SgFn | SgPgFn v SgFn | SgPgFn vs SgPg | Coverage

Protein	Description	*SgFn vs Sg*				*SgPg vs Sg*				*SgPgFn vs Sg*				*SgPg vs SgFn*				*SgPgFn vs SgFn*				*SgPgFn vs SgPg*			
		Ratio	Sum	*q*-Val	*p*-Val	Ratio	Sum	*q*-Val	*p*-Val	Ratio	Sum	*q*-Val	*p*-Val	Ratio	Sum	*q*-Val	*p*-Val	Ratio	Sum	*q*-Val	*p*-Val	Ratio	Sum	*q*-Val	*p*-Val
**SGO_1267**	thioredoxin family protein		5.406			-1.825	5.596	0.0185	0.0707		5.406				2.063								2.063		
**SGO_1273**	rpoD; RNA polymerase sigma factor	0.309	7.412	0.0431	0.2413	1.903	8.469	0.0001	0.0000	1.987	8.558	0.0018	0.0070	1.594	8.036	0.0006	0.0008	1.678	7.509	0.0037	0.0108	0.084	8.669	0.1635	0.5535
**SGO_1274**	dnaG; DNA primase		3.105			0.563	4.342	0.0490	0.2164		3.105				3.032								3.032		
**SGO_1276**	rpsU; ribosomal protein S21	-1.596	7.134	0.0001	0.0000	0.281	7.872	0.0142	0.0507	0.481	7.989	0.0059	0.0324	1.877	6.842	0.0007	0.0010	2.077	6.395	0.0013	0.0021	0.200	7.602	0.0874	0.2374
**SGO_1280**	putative membrane-associated Zn-dependent protease		3.549				3.549				3.549														
**SGO_1281**	penicillinase repressor, putative	-0.896	4.066	0.0337	0.1781	0.982	5.300	0.0047	0.0128	-0.127	4.265	0.0675	0.6167	1.879	4.379	0.0216	0.1249	0.770	2.127			-1.109	4.494	0.0665	0.1670
**SGO_1283**	oxidoreductase	-0.373	7.916	0.0112	0.0452	0.742	8.503	0.0003	0.0003	0.335	8.261	0.0009	0.0029	1.115	7.864	0.0006	0.0008	0.708	6.989	0.0018	0.0041	-0.407	8.123	0.0016	0.0002
**SGO_1284**	thioredoxin-disulfide reductase	-0.375	5.791	0.0754	0.4661	0.132	6.048	0.1080	0.5381	0.103	5.968	0.0455	0.3963	0.508	5.424	0.0537	0.3677	0.479	4.727	0.0518	0.3205	-0.029	5.586	0.1982	0.7089
**SGO_1290**	ABC transporter, permease/ATP-binding protein		4.483				4.483				4.483														
**SGO_1291**	ABC transporter, permease/ATP-binding protein SP1358		3.115				3.115				3.115														
**SGO_1293**	asnS; asparaginyl-tRNA synthetase	0.127	7.656	0.0294	0.1508	0.430	7.822	0.0034	0.0085	0.069	7.646	0.0692	0.6353	0.303	7.365	0.0076	0.0328	-0.058	6.586	0.1292	0.9336	-0.362	7.357	0.0731	0.1879
**SGO_1297**	aspC; aspartate aminotransferase	2.252	7.245	0.0019	0.0046	0.992	6.280	0.0004	0.0005	1.112	6.360	0.0001	0.0000	-1.260	6.958	0.0033	0.0109	-1.140	6.383	0.0042	0.0127	0.120	6.240	0.0340	0.0635
**SGO_1298**	hypothetical protein SGO_1298		3.425				3.425				3.425														
**SGO_1305**	substrate-binding protein MsmE		5.520			-0.420	6.325	0.0004	0.0004	-1.367	5.998	0.0004	0.0007		4.586				3.048			-0.947	5.196	0.0053	0.0026
**SGO_1312**	pepT; peptidase T	3.698	7.531	0.0000	0.0000	3.727	7.558	0.0001	0.0000	3.152	7.040	0.0004	0.0009	0.028	7.925	0.0440	0.2936	-0.546	7.056	0.0025	0.0065	-0.574	7.684	0.0079	0.0057
**SGO_1314**	metal binding lipoprotein		1.807				1.807				1.807														

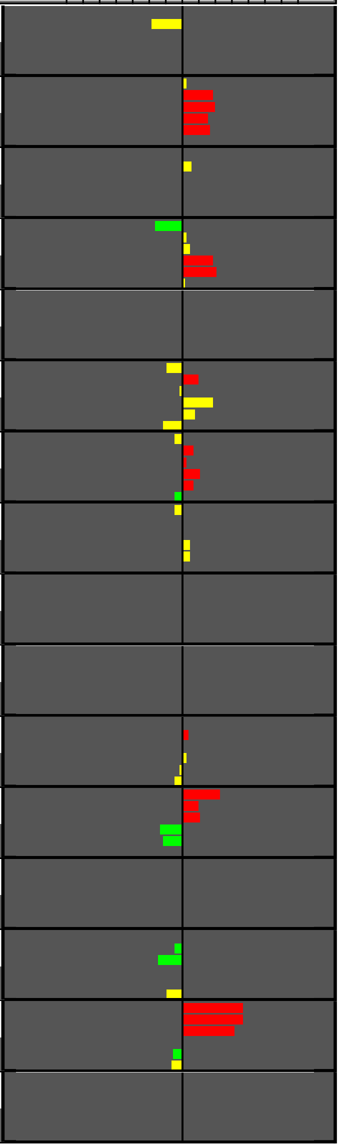

Show detected proteins only
Show all proteins
Filter by category:
ABC Transporter

Proteins found:
1179

Dot Plots
Dot Plots

Test	Cutoff
q-Value	.005
p-Value	

Color	Signif	Direction	Applies To
red	yes	+	ratios, bars
yellow	no	n/a	bars
green	yes	–	ratios, bars
light red	yes	+	*p*- , *q*-Values
light green	yes	–	*p*- , *q*-Values

SgFn vs Sg — *SgPg vs Sg*
SgPgFn vs Sg — *SgPg vs SgFn*
SgPgFn vs SgFn — *SgPgFn vs SgPg*

Hendrickson *et al.*

Streptococcus gordonii

Protein	Description	SgFn vs Sg Ratio	Sum	*q*-Val	*p*-Val	SgPg vs Sg Ratio	Sum	*q*-Val	*p*-Val	SgPgFn vs Sg Ratio	Sum	*q*-Val	*p*-Val	SgPg vs SgFn Ratio	Sum	*q*-Val	*p*-Val	SgPgFn vs SgFn Ratio	Sum	*q*-Val	*p*-Val	SgPgFn vs SgPg Ratio	Sum	*q*-Val	*p*-Val
**SGO_1315**	trmD; tRNA (guanine-N1)-methyltransferase	1.317	3.611			0.030	3.428	0.1661	0.8725		1.807			-1.287	3.485	0.0152	0.0809		2.000				2.348		
**SGO_1316**	16S rRNA processing protein RimM	-0.094	4.561	0.0829	0.5220	0.266	4.747	0.0494	0.2206		3.619			0.360	4.180	0.0050	0.0190		2.376				3.351		
**SGO_1323**	rpsP; ribosomal protein S16	-0.001	7.305	0.1420	0.9590	0.691	7.691	0.0033	0.0082	1.424	8.182	0.0002	0.0003	0.692	7.175	0.0021	0.0051	1.425	7.057	0.0003	0.0002	0.733	7.891	0.0043	0.0012
**SGO_1324**	hypothetical protein SGO_1324		5.595				5.595				5.595														
**SGO_1327**	HAD-superfamily subfamily IIA hydrolase, TIGR01457	-0.272	5.984	0.0425	0.2370	0.123	6.174	0.1330	0.6792	-0.794	5.775	0.0079	0.0463	0.395	5.518	0.0009	0.0015	-0.522	4.460	0.0011	0.0014	-0.917	5.317	0.0026	0.0005
**SGO_1328**	hypothetical protein SGO_1328		1.585				1.585				1.585														
**SGO_1330**	transporter		3.772				3.772				3.772														
**SGO_1331**	HlyB/MsbA family ABC transporter		1.807				1.807				1.807														
**SGO_1336**	pcrA; ATP-dependent DNA helicase PcrA	0.376	6.355	0.0261	0.1308	0.050	6.164	0.0802	0.3879	-0.743	5.818	0.0010	0.0032	-0.326	5.865	0.0282	0.1730	-1.119	4.961	0.0062	0.0218	-0.793	5.338	0.0073	0.0051
**SGO_1337**	cation efflux family protein		1.585				1.585				1.585														
**SGO_1338**	Signal peptidase I	-0.729	6.239	0.0050	0.0161	-3.036	5.648	0.0253	0.1018		5.564			-2.307	4.446	0.0012	0.0024		3.696				1.000		
**SGO_1339**	pyk; pyruvate kinase	0.635	11.550	0.0014	0.0030	1.066	11.826	0.0001	0.0001	0.963	11.757	0.0003	0.0003	0.431	11.551	0.0019	0.0043	0.328	10.883	0.0048	0.0154	-0.104	11.699	0.0426	0.0899
**SGO_1340**	Phosphofructokinase	1.240	10.517	0.0002	0.0001	0.758	10.195	0.0001	0.0000	0.630	10.116	0.0002	0.0003	-0.483	10.273	0.0008	0.0011	-0.610	9.611	0.0008	0.0008	-0.128	9.949	0.0114	0.0111
**SGO_1341**	dnaE; DNA-directed DNA polymerase III alpha chain	0.393	5.498	0.0437	0.2450	-1.076	4.546	0.0154	0.0566		4.239			-1.469	4.431	0.0448	0.3009		3.594				1.648		
**SGO_1342**	ABC transporter, ATP-binding protein SP1715	-0.526	10.529	0.0026	0.0071	-1.281	10.262	0.0001	0.0001	-1.216	10.281	0.0001	0.0000	-0.755	9.403	0.0030	0.0092	-0.690	8.816	0.0033	0.0095	0.066	9.004	0.1095	0.3209
**SGO_1343**	ABC transporter, ATP-binding protein SP1715		5.350			-1.773	5.547	0.0085	0.0271		5.350				2.063								2.063		

Log$_2$ Ratios

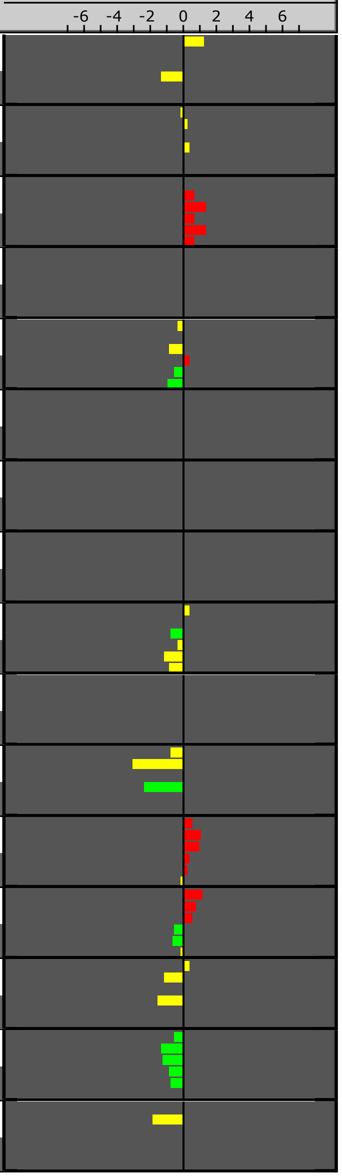

Show detected proteins only
Show all proteins
Filter by category:
ABC Transporter

Proteins found:
1179

Dot Plots
Dot Plots

Test	Cutoff
q-Value	.005
p-Value	

	Signif	Direction	Applies To
(red)	yes	+	ratios, bars
(yellow)	no	n/a	bars
(green)	yes	–	ratios, bars
(pink)	yes	+	*p*- , *q*-Values
(light green)	yes	–	

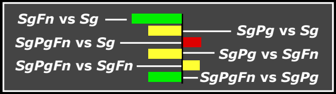

Streptococcus gordonii

Summary Table | SgFn vs Sg | SgPg vs Sg | SgPgFn vs Sg | SgPg v SgFn | SgPgFn v SgFn | SgPgFn vs SgPg | Coverage

Protein	Description	*SgFn vs Sg* Ratio	Sum	*q*-Val	*p*-Val	*SgPg vs Sg* Ratio	Sum	*q*-Val	*p*-Val	*SgPgFn vs Sg* Ratio	Sum	*q*-Val	*p*-Val
SGO_1347	membrane associated lipoprotein	-3.010	6.238	0.0236	0.1122		6.152				6.152		
SGO_1355	lipoprotein, putative	0.352	4.188	0.0603	0.3566		3.483				3.483		
SGO_1360	ABC transporter domain protein		2.832				2.832				2.832		
SGO_1361	ADP-ribosylhydrolase	-0.470	3.485				2.700				2.700		
SGO_1364	rumA-2; 23S rRNA (uracil-5-)-methyltransferase RumA	-0.705	6.491	0.0080	0.0301	-0.053	6.770	0.1267	0.6436	-2.201	5.952	0.0174	0.1299
SGO_1365	transcription regulator yrfE	-0.327	6.965	0.0064	0.0221	-3.071	6.289	0.0001	0.0001	-2.531	6.236	0.0012	0.0040
SGO_1366	pheA; prephenate dehydratase						3.719						
SGO_1367	aroK; shikimate kinase	1.206	5.508	0.0015	0.0034	-0.645	4.492	0.0027	0.0065		3.781		
SGO_1368	aroA; 3-phosphoshikimate 1-carboxyvinyltransferase	2.491	5.517	0.0015	0.0034	1.405	4.704	0.0175	0.0655		3.098		
SGO_1369	L-2-hydroxyisocaproate dehydrogenase	3.346	8.392	0.0001	0.0000	-0.018	5.905	0.1710	0.9038	-0.928	5.247	0.0072	0.0414
SGO_1370	Protein of unknown function (DUF964) superfamily	1.319	6.800	0.0022	0.0058	1.245	6.740	0.0010	0.0015	1.637	7.025	0.0003	0.0004
SGO_1372	aroC; chorismate synthase	0.153	4.305	0.0774	0.4805	1.553	5.640	0.0003	0.0002	0.923	4.628	0.0109	0.0717
SGO_1373	aroB; 3-dehydroquinate synthase	0.481	4.716	0.0699	0.4266	0.379	5.146	0.0473	0.2080	0.062	4.562	0.1026	0.9899
SGO_1374	aroE; shikimate 5-dehydrogenase		3.196			0.232	4.333	0.0655	0.3054		3.196		
SGO_1375	aroD; 3-dehydroquinate dehydratase, type I	-0.075	5.682	0.0821	0.5164	0.563	6.044	0.0214	0.0838	0.006	5.308	0.1015	0.9771
SGO_1376	Methyltransferase		3.982				3.982				3.982		

Protein	*SgPg vs SgFn* Ratio	Sum	*q*-Val	*p*-Val	*SgPgFn vs SgFn* Ratio	Sum	*q*-Val	*p*-Val	*SgPgFn vs SgPg* Ratio	Sum	*q*-Val	*p*-Val
SGO_1347		1.611				1.000						
SGO_1355		2.302				1.692						
SGO_1360												
SGO_1361		1.717				1.107						
SGO_1364	0.652	5.934	0.0013	0.0028	-1.496	4.199	0.0205	0.1008	-2.148	5.375	0.0143	0.0158
SGO_1365	-2.745	5.496	0.0007	0.0010	-2.204	4.820	0.0169	0.0791	0.541	3.376	0.1526	0.4924
SGO_1366		3.205								3.205		
SGO_1367	-1.851	4.827	0.0009	0.0014		3.866				2.617		
SGO_1368	-1.086	5.261	0.0005	0.0005		4.094				3.617		
SGO_1369	-3.364	7.878	0.0001	0.0000	-4.275	7.170	0.0063	0.0223	-0.910	4.724	0.0565	0.1337
SGO_1370	-0.075	6.763	0.0826	0.6023	0.318	6.355	0.0193	0.0935	0.392	6.928	0.0178	0.0228
SGO_1372	1.399	4.953	0.0083	0.0379	0.770	3.127			-0.629	5.106	0.0352	0.0663
SGO_1373	-0.102	4.405	0.0224	0.1302	-0.419	3.082			-0.318	4.272	0.0284	0.0487
SGO_1374		2.945								2.945		
SGO_1375	0.638	5.497	0.0111	0.0536	0.081	4.126	0.0571	0.3649	-0.556	5.207	0.1164	0.3516
SGO_1376												

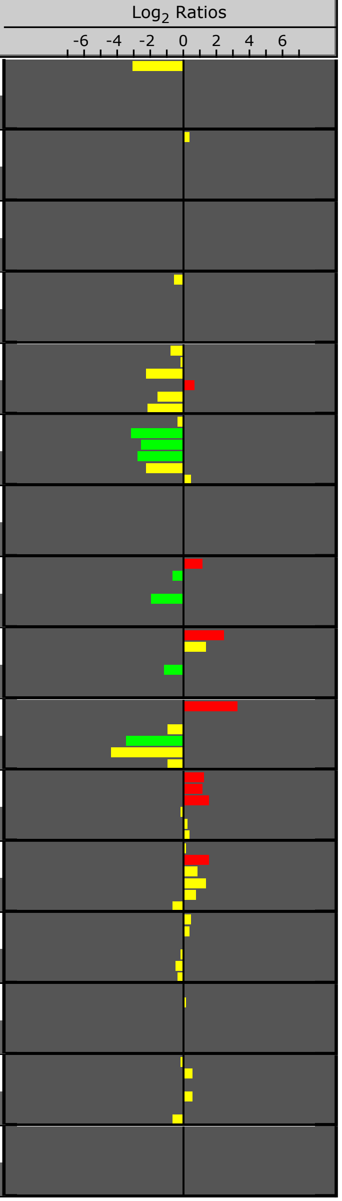

Show detected proteins only
Show all proteins
Filter by category:
ABC Transporter

Proteins found: 1179

Dot Plots
Dot Plots

Test	Cutoff
q-Value	.005
p-Value	

Color	Signif	Direction	Applies To
red	yes	+	ratios, bars
yellow	no	n/a	bars
green	yes	–	ratios, bars
light red	yes	+	*p*- , *q*-Values
light green	yes	–	

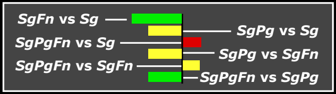

Hendrickson *et al.*

Spectral Counts<br>*Sg Summary Table*	*Streptococcus gordonii*	Hackett Laboratory UW

Protein	Description	*SgFn vs Sg* Ratio	Sum	*q*-Val	*p*-Val	*SgPg vs Sg* Ratio	Sum	*q*-Val	*p*-Val	*SgPgFn vs Sg* Ratio	Sum	*q*-Val	*p*-Val
SGO_1377	sulfatase	-1.512	8.293	0.0003	0.0003	-2.205	8.142	0.0003	0.0002	-3.781	7.966	0.0002	0.0001
SGO_1381	csn1; CRISPR-associated protein, Csn1 family	0.018	6.733	0.1240	0.8153	-0.506	6.495	0.0150	0.0546	-0.907	6.330	0.0003	0.0006
SGO_1383	rplS; ribosomal protein L19	0.097	8.709	0.0639	0.3823	0.692	9.044	0.0016	0.0032	0.813	9.118	0.0003	0.0004
SGO_1386	chorismate mutase	3.748	6.408	0.0246	0.1188	1.156	4.082	0.0635	0.2939		1.585		
SGO_1388	pulA-1; pullulanase, type I	-0.919	5.475	0.0302	0.1570		4.827				4.827		
SGO_1389	conserved hypothetical protein TIGR00147	-0.596	6.153	0.0095	0.0368		5.417				5.417		
SGO_1390	ligA; DNA ligase, NAD-dependent	-0.644	6.055	0.0041	0.0125	-0.884	5.960	0.0007	0.0009	-0.819	5.995	0.0031	0.0140
SGO_1394	hypothetical protein SGO_1394		3.124				4.059						
SGO_1397	map; methionine aminopeptidase, type I	0.483	6.995	0.0216	0.1016	0.382	6.937	0.0390	0.1674	-0.134	6.668	0.0535	0.4738
SGO_1398	hypothetical protein SGO_1398	-1.383	5.608	0.0018	0.0044	-1.419	5.593	0.0015	0.0030		5.134		
SGO_1400	murA-2; UDP-N-acetylglucosamine 1-carboxyvinyltransferase	-0.055	6.325	0.1011	0.6549	-0.556	6.098	0.0023	0.0052	-1.006	5.997	0.0111	0.0740
SGO_1405	hisA; phosphoribosylformimino-5-aminoimidazole carboxamide ribotide isomerase						1.835						
SGO_1413	first chain of major exonuclease RexA	0.215	5.654	0.0954	0.6144	-0.695	5.167	0.0323	0.1348		4.510		
SGO_1414	rexB; putative exonuclease RexB	1.306	5.724	0.0082	0.0306	-0.773	4.582	0.0176	0.0664	0.673	5.281	0.0084	0.0507
SGO_1415	LPXTG cell wall surface protein, X-prolyl dipeptidylaminopeptidase, putative		2.481				2.481				2.481		
SGO_1416	putative histidine kinase		1.585				1.585				1.585		

Protein	*SgPg vs SgFn* Ratio	Sum	*q*-Val	*p*-Val	*SgPgFn vs SgFn* Ratio	Sum	*q*-Val	*p*-Val	*SgPgFn vs SgPg* Ratio	Sum	*q*-Val	*p*-Val
SGO_1377	-0.693	6.527	0.0002	0.0001	-2.269	5.508	0.0005	0.0004	-1.576	5.577	0.0051	0.0025
SGO_1381	-0.525	6.005	0.0188	0.1061	-0.925	5.231	0.0052	0.0172	-0.400	5.525	0.0533	0.1243
SGO_1383	0.595	8.571	0.0030	0.0093	0.716	8.032	0.0013	0.0023	0.121	8.897	0.0576	0.1374
SGO_1386	-2.592	6.069	0.0018	0.0042		5.232				3.288		
SGO_1388		3.495				2.884						
SGO_1389		4.315				3.705						
SGO_1390	-0.240	5.074	0.0349	0.2218	-0.176	4.509	0.0817	0.5555	0.065	4.991	0.1897	0.6735
SGO_1394	-0.126	4.153	0.1186	0.8978		2.000				3.546		
SGO_1397	-0.101	6.641	0.0692	0.4893	-0.617	5.803	0.0048	0.0153	-0.516	6.352	0.0165	0.0202
SGO_1398	-0.036	4.232	0.1077	0.8090		2.648				3.205		
SGO_1400	-0.501	5.556	0.0058	0.0230	-0.951	4.842	0.0202	0.0980	-0.450	5.155	0.1586	0.5277
SGO_1405		1.322								1.322		
SGO_1413	-0.910	4.834	0.0232	0.1353		3.661				3.202		
SGO_1414	-2.079	5.022	0.0038	0.0129	-0.633	4.814	0.0183	0.0876	1.446	4.504	0.0057	0.0031
SGO_1415												
SGO_1416												

Log_2 Ratios

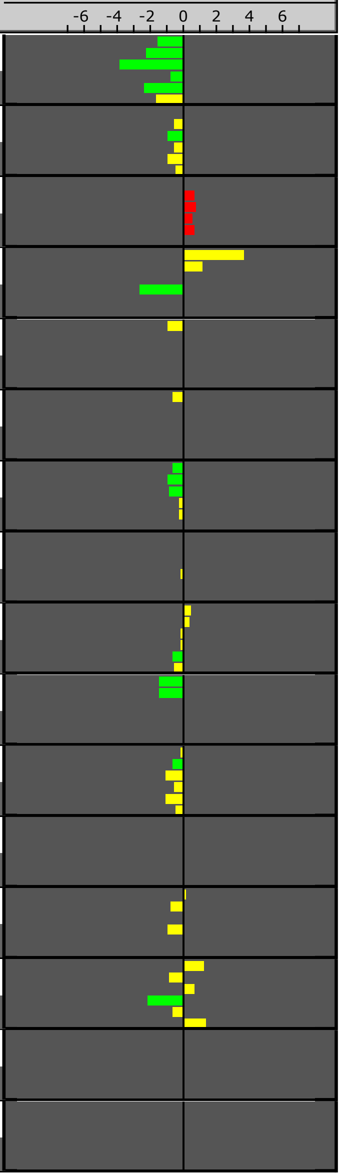

Show detected proteins only
Show all proteins
Filter by category:
ABC Transporter

Proteins found:
1179

Dot Plots
Dot Plots

Test	Cutoff
q-Value	.005
p-Value	

	Signif	Direction	Applies To
red	yes	+	ratios, bars
yellow	no	n/a	bars
green	yes	–	ratios, bars
light red	yes	+	*p*- , *q*-Values
light green	yes	–	

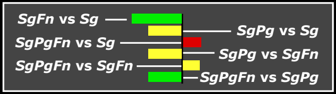

Hendrickson *et al.*

Streptococcus gordonii

Hackett Laboratory UW

Summary Table | SgFn vs Sg | SgPg vs Sg | SgPgFn vs Sg | SgPg v SgFn | SgPgFn v SgFn | SgPgFn vs SgPg | Coverage

	SgFn vs Sg				*SgPg vs Sg*				*SgPgFn vs Sg*				*SgPg vs SgFn*				*SgPgFn vs SgFn*				*SgPgFn vs SgPg*			
Protein	Ratio	Sum	*q*-Val	*p*-Val	Ratio	Sum	*q*-Val	*p*-Val	Ratio	Sum	*q*-Val	*p*-Val	Ratio	Sum	*q*-Val	*p*-Val	Ratio	Sum	*q*-Val	*p*-Val	Ratio	Sum	*q*-Val	*p*-Val
**SGO_1417**						2.973								2.459								2.459		
	response regulator protein																							
**SGO_1422**	1.421	7.816	0.0049	0.0157	-0.278	6.778	0.0007	0.0009	0.474	7.184	0.0121	0.0817	-1.700	7.234	0.0037	0.0120	-0.947	6.844	0.0101	0.0413	0.752	6.562	0.0229	0.0351
	hypothetical protein SGO_1422																							
**SGO_1426**	0.877	13.890	0.0009	0.0017	1.751	14.510	0.0001	0.0000	2.044	14.741	0.0001	0.0000	0.874	14.250	0.0003	0.0003	1.167	13.835	0.0002	0.0001	0.293	14.775	0.0037	0.0009
	eno; enolase																							
**SGO_1431**	-1.232	10.435	0.0057	0.0190	-1.476	10.366	0.0048	0.0136	-1.818	10.285	0.0024	0.0100	-0.244	9.039	0.0119	0.0586	-0.585	8.282	0.0028	0.0076	-0.341	8.748	0.0026	0.0005
	EzrA; Septation ring formation regulator ezrA																							
**SGO_1432**	-0.233	7.339	0.0479	0.2714	0.327	7.630	0.0534	0.2414	-0.175	7.394	0.0706	0.6504	0.560	7.005	0.0084	0.0385	0.058	6.137	0.0965	0.6723	-0.502	7.053	0.0667	0.1681
	gyrB; DNA gyrase, B subunit																							
**SGO_1434**	1.420	4.000	0.0260	0.1301	1.419	4.025	0.0515	0.2317	1.074	2.957			-0.001	4.256	0.1190	0.9037	-0.346	3.106	0.0544	0.3410	-0.345	3.738	0.1566	0.5087
	thiJ; 4-methyl-5(beta-hydroxyethyl)-thiazole monophosphate synthesis protein																							
**SGO_1437**		3.669				3.669				3.669														
	DnaQ family exonuclease/DinG family helicase, putative																							
**SGO_1439**	-1.950	7.156	0.0001	0.0000	-1.888	7.169	0.0001	0.0000	-1.087	7.383	0.0003	0.0006	0.062	5.394	0.0593	0.4113	0.863	5.252	0.0027	0.0072	0.801	5.885	0.0103	0.0094
	ftsX; cell division protein FtsX																							
**SGO_1440**	-0.192	8.633	0.0018	0.0041	-1.350	8.204	0.0001	0.0000	-2.111	8.027	0.0001	0.0000	-1.158	7.555	0.0000	0.0000	-1.919	6.748	0.0000	0.0000	-0.761	6.532	0.0017	0.0002
	cell-division ATP-binding protein FtsE																							
**SGO_1441**	1.397	6.689	0.0036	0.0106	0.860	6.328	0.0120	0.0413	0.530	6.167	0.0258	0.2063	-0.537	6.479	0.0212	0.1216	-0.867	5.769	0.0186	0.0894	-0.331	6.072	0.1586	0.5279
	prfB; peptide chain release factor 2																							
**SGO_1446**	0.050	6.385	0.1420	0.9578	0.547	6.652	0.0188	0.0726	-1.757	5.755	0.0038	0.0190	0.497	6.145	0.0048	0.0181	-1.806	4.641	0.0016	0.0034	-2.303	5.644	0.0026	0.0006
	murF; UDP-N-acetylmuramoyl-tripeptide--D-alanyl-D-alanine ligase																							
**SGO_1447**	0.192	6.727	0.0003	0.0002	0.694	7.021	0.0026	0.0059	-0.234	6.516	0.0002	0.0002	0.502	6.584	0.0037	0.0122	-0.426	5.499	0.0000	0.0000	-0.928	6.423	0.0057	0.0032
	ddlA; D-Ala-D-Ala ligase																							
**SGO_1449**		7.043			-2.494	7.277	0.0006	0.0007		7.043				4.030								4.030		
	pbp2b; penicillin-binding protein 2B																							
**SGO_1450**	0.488	5.729	0.1066	0.6933	0.312	5.654	0.1740	0.9221	-0.049	5.584	0.0945	0.9050	-0.175	5.241	0.0703	0.4995	-0.537	4.565	0.0809	0.5462	-0.361	5.099	0.2139	0.7870
	hypothetical protein SGO_1450																							
**SGO_1451**	-0.022	6.949	0.1451	0.9850	0.844	7.426	0.0011	0.0018	1.596	7.952	0.0003	0.0004	0.866	6.911	0.0024	0.0066	1.618	6.827	0.0008	0.0008	0.752	7.695	0.0032	0.0008
	frr; ribosome recycling factor																							
**SGO_1452**	0.222	6.908	0.0294	0.1514	1.012	7.403	0.0077	0.0237	0.769	7.227	0.0011	0.0036	0.790	6.962	0.0074	0.0313	0.547	6.184	0.0004	0.0002	-0.243	7.184	0.0848	0.2275
	pyrH; uridylate kinase																							

Log$_2$ Ratios

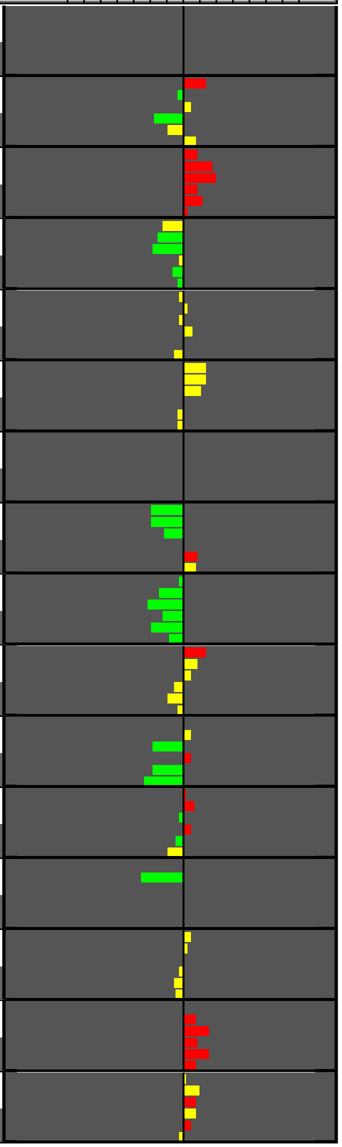

- Show detected proteins only
- Show all proteins
- Filter by category: ABC Transporter

Proteins found: 1179

Dot Plots | Dot Plots

Test	Cutoff
q-Value	.005
p-Value	

	Signif	Direction	Applies To
red	yes	+	ratios, bars
yellow	no	n/a	bars
green	yes	−	ratios, bars
pink	yes	+	*p*- , *q*-Values
light green	yes	−	

SgFn vs Sg — *SgPg vs Sg*
SgPgFn vs Sg — *SgPg vs SgFn*
SgPgFn vs SgFn — *SgPgFn vs SgPg*

Hendrickson *et al.*

Streptococcus gordonii

Hackett Laboratory UW

Summary Table | SgFn vs Sg | SgPg vs Sg | SgPgFn vs Sg | SgPg v SgFn | SgPgFn v SgFn | SgPgFn vs SgPg | Coverage

Protein	Description	*SgFn vs Sg* Ratio	Sum	*q*-Val	*p*-Val	*SgPg vs Sg* Ratio	Sum	*q*-Val	*p*-Val	*SgPgFn vs Sg* Ratio	Sum	*q*-Val	*p*-Val	*SgPg vs SgFn* Ratio	Sum	*q*-Val	*p*-Val	*SgPgFn vs SgFn* Ratio	Sum	*q*-Val	*p*-Val	*SgPgFn vs SgPg* Ratio	Sum	*q*-Val	*p*-Val
SGO_1453	glycosyl transferase, family 8 SP1766		2.231				4.100				5.420			1.869	3.936			2.151	4.446	0.0325	0.1818	0.282	5.393	0.1618	0.5445
SGO_1454	amino acid permease family protein		4.989				4.989				4.989														
SGO_1455	rplA; ribosomal protein L1	0.360	9.165	0.0027	0.0075	1.547	9.960	0.0027	0.0064	1.539	9.954	0.0016	0.0063	1.187	9.545	0.0031	0.0097	1.180	8.929	0.0033	0.0095	-0.007	10.022	0.2492	0.9697
SGO_1456	rplK; ribosomal protein L11	-0.623	7.371	0.0080	0.0297	0.563	7.956	0.0124	0.0428	1.340	8.456	0.0002	0.0002	1.186	7.235	0.0044	0.0159	1.963	7.189	0.0006	0.0004	0.777	8.133	0.0094	0.0079
SGO_1458	aha1; cation-transporting ATPase yfgQ	-1.358	7.397	0.0062	0.0213	-3.777	7.025	0.0025	0.0057	-3.490	6.985	0.0199	0.1543	-2.418	5.291	0.0023	0.0064	-2.131	4.587	0.0298	0.1627	0.287	3.299	0.1503	0.4830
SGO_1459	glyoxylase family protein		5.269				1.161							-3.101	4.837	0.0133	0.0686		4.145				0.648		
SGO_1460	DNA translocase ftsK	-2.935	6.888	0.0009	0.0018	-1.693	7.097	0.0017	0.0034	-2.301	6.850	0.0154	0.1116	1.242	5.000	0.0001	0.0000	0.634	3.467	0.0102	0.0427	-0.608	4.901	0.0251	0.0404
SGO_1463	peptidyl-prolyl cis-trans isomerase	-2.423	7.258	0.0002	0.0002	-1.582	7.422	0.0003	0.0002	-1.721	7.388	0.0001	0.0001	0.841	5.569	0.0059	0.0232	0.702	4.869	0.0085	0.0325	-0.139	5.848	0.1013	0.2915
SGO_1464	uncharacterized probable metal-binding protein		7.031			-1.171	7.558	0.0021	0.0045	-1.964	7.359	0.0007	0.0020		5.336				3.939			-0.793	5.996	0.0050	0.0018
SGO_1465	ABC transporter, ATP-binding protein SP0770	-1.851	6.642	0.0016	0.0037	0.016	7.297	0.1771	0.9448	-0.670	6.990	0.0048	0.0254	1.867	6.138	0.0013	0.0026	1.182	5.013	0.0011	0.0016	-0.686	6.485	0.0158	0.0184
SGO_1466	MTA/SAH nucleosidase	-0.133	5.958	0.0658	0.3968	-0.616	5.754	0.0142	0.0510		5.034			-0.483	5.149	0.0044	0.0157		3.755				3.893		
SGO_1468	MutT/nudix family protein		1.585				1.585				1.585														
SGO_1469	glmU; UDP-N-acetylglucosamine pyrophosphorylase	-0.341	7.526	0.0005	0.0007	0.365	7.880	0.0011	0.0018	0.313	7.853	0.0043	0.0221	0.705	7.228	0.0004	0.0003	0.653	6.587	0.0017	0.0038	-0.052	7.514	0.1588	0.5306
SGO_1472	acetyltransferase, GNAT family	1.625	4.942	0.0553	0.3203	0.998	4.385	0.0738	0.3513	0.170	3.088			-0.627	4.934	0.0371	0.2402	-1.454	3.841	0.0537	0.3353	-0.828	3.906	0.1256	0.3862
SGO_1475	lipoprotein, putative		4.513				4.513				4.513														
SGO_1483	ABC transporter, permease protein, probable SP0913		1.643				1.643				1.643														

Log$_2$ Ratios

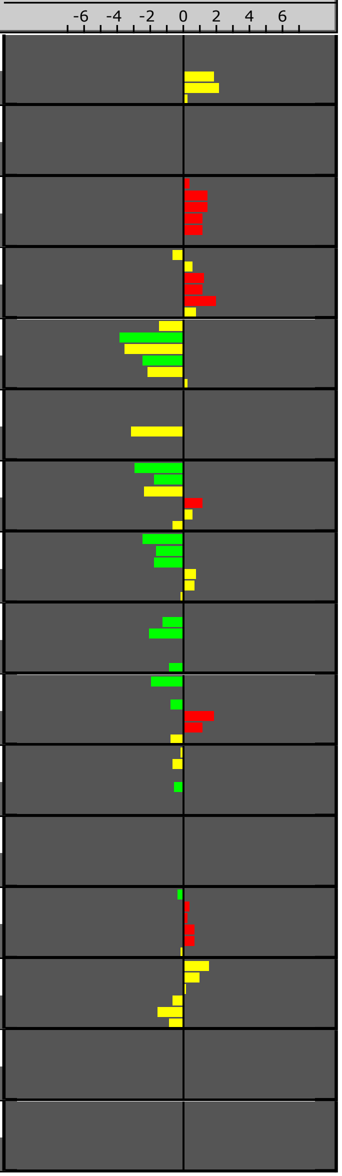

Show detected proteins only
Show all proteins
Filter by category:
ABC Transporter

Proteins found:
1179

Dot Plots
Dot Plots

Test	Cutoff
q-Value	.005
p-Value	

Color	Signif	Direction	Applies To
red	yes	+	ratios, bars
yellow	no	n/a	bars
green	yes	–	ratios, bars
light red	yes	+	*p-* , *q*-Values
light green	yes	–	*p-* , *q*-Values

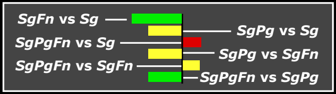

Hendrickson *et al.*

Protein	Description	*SgFn vs Sg* Ratio	Sum	*q*-Val	*p*-Val	*SgPg vs Sg* Ratio	Sum	*q*-Val	*p*-Val	*SgPgFn vs Sg* Ratio	Sum	*q*-Val	*p*-Val	*SgPg vs SgFn* Ratio	Sum	*q*-Val	*p*-Val	*SgPgFn vs SgFn* Ratio	Sum	*q*-Val	*p*-Val	*SgPgFn vs SgPg* Ratio	Sum	*q*-Val	*p*-Val
SGO_1484	ABC transporter, ATP-binding protein						3.135								2.622								2.622		
SGO_1486	beta-galactosidase	2.124	3.744				1.322			1.330	3.932	0.0225	0.1786		2.933			-0.794	3.441	0.0286	0.1547		3.161		
SGO_1487	LPXTG cell wall surface protein, Cna protein B-type domain	-0.501	7.346	0.0093	0.0359	-3.730	6.626	0.0185	0.0708		6.572			-3.229	5.637	0.0167	0.0923		4.952				1.322		
SGO_1493	hypothetical protein SGO_1493	-1.485	7.194	0.0009	0.0015	-2.649	6.864	0.0185	0.0704		6.754			-1.164	5.045	0.0191	0.1084		4.145				2.585		
SGO_1495	putative transcriptional regulator		4.523				4.523				4.523														
SGO_1498	ABC transporter, permease protein, probable SP0913		1.643				1.643				1.643														
SGO_1504	histidine kinase		3.785				3.785				3.785														
SGO_1507	hypothetical protein SGO_1507		5.334				5.334				5.334														
SGO_1512	lacG; 6-phospho-beta-galactosidase	1.099	4.069	0.0412	0.2271	0.741	3.060				1.643			-0.358	3.726	0.0645	0.4512		2.648				1.871		
SGO_1513	lacE; PTS system, lactose-specific IIBC component		1.807				1.807				1.807														
SGO_1516	lacD-2; tagatose 1,6-diphosphate aldolase		4.452			1.216	6.199	0.0050	0.0144	0.291	5.601	0.0057	0.0306		5.175				3.613			-0.925	5.776	0.0172	0.0219
SGO_1518	lacB-2; galactose-6-phosphate isomerase, LacB subunit		3.417			0.892	4.926	0.0015	0.0030		3.417				3.788								3.788		
SGO_1520	PTS system, IIC component		3.058				3.058				3.058														
SGO_1521	PTS system IIB component (galactitol/fructose specific)		2.170				2.170				2.170														
SGO_1523	lacD-1; tagatose 1,6-diphosphate aldolase																								
SGO_1527	Lactose phosphotransferase system repressor		1.865				1.865				1.865														

Log$_2$ Ratios: -6 -4 -2 0 2 4 6

Show detected proteins only
Show all proteins
Filter by category:
ABC Transporter

Proteins found: 1179

Dot Plots
Dot Plots

Test	Cutoff
q-Value	.005
p-Value	

	Signif	Direction	Applies To
red	yes	+	ratios, bars
yellow	no	n/a	bars
green	yes	–	ratios, bars
pink	yes	+	*p*- , *q*-Values
light green	yes	–	

SgFn vs Sg, SgPgFn vs Sg, SgPgFn vs SgFn, SgPg vs Sg, SgPg vs SgFn, SgPgFn vs SgPg

Hendrickson *et al.*

Streptococcus gordonii

Hackett Laboratory UW

Summary Table | SgFn vs Sg | SgPg vs Sg | SgPgFn vs Sg | SgPg v SgFn | SgPgFn v SgFn | SgPgFn vs SgPg | Coverage

Protein	*SgFn vs Sg* Ratio	Sum	*q*-Val	*p*-Val	*SgPg vs Sg* Ratio	Sum	*q*-Val	*p*-Val	*SgPgFn vs Sg* Ratio	Sum	*q*-Val	*p*-Val	*SgPg vs SgFn* Ratio	Sum	*q*-Val	*p*-Val	*SgPgFn vs SgFn* Ratio	Sum	*q*-Val	*p*-Val	*SgPgFn vs SgPg* Ratio	Sum	*q*-Val	*p*-Val
**SGO_1529**	-1.110	6.071	0.0007	0.0011	-1.199	6.053	0.0017	0.0037		5.519			-0.089	4.876	0.1076	0.8056		3.294				3.846		
	hypothetical protein SGO_1529																							
**SGO_1530**	-0.620	8.210	0.0111	0.0443	0.080	8.516	0.1062	0.5268	-0.321	8.323	0.0092	0.0575	0.700	7.741	0.0048	0.0180	0.299	6.897	0.0330	0.1849	-0.402	7.851	0.0073	0.0049
	methionine-tRNA ligase																							
**SGO_1531**	0.311	5.961	0.0299	0.1548	0.988	6.363	0.0013	0.0023	0.273	5.947	0.0295	0.2398	0.677	5.972	0.0060	0.0243	-0.038	4.984	0.1282	0.9234	-0.715	5.962	0.0212	0.0304
	xth; exodeoxyribonuclease III																							
**SGO_1534**	1.195	5.714	0.0220	0.1038	1.089	5.713	0.0490	0.2161	1.284	5.196	0.0428	0.3687	-0.106	5.567	0.1294	0.9952	0.089	4.573	0.0468	0.2795	0.195	5.182	0.2345	0.8886
	ArsC family																							
**SGO_1536**	-0.922	6.083	0.0065	0.0228	-0.019	6.475	0.1779	0.9526	-0.798	6.124	0.0053	0.0282	0.903	5.581	0.0127	0.0647	0.124	4.493	0.0921	0.6369	-0.779	5.622	0.0396	0.0794
	conserved hypothetical protein TIGR00096																							
**SGO_1538**		3.266				3.266				3.266														
	DNA-directed DNA polymerase III, delta'' chain																							
**SGO_1539**	0.151	6.166	0.0639	0.3839	0.530	6.365	0.0031	0.0075	0.614	6.430	0.0085	0.0520	0.379	5.928	0.0213	0.1226	0.463	5.379	0.0278	0.1490	0.084	6.150	0.1727	0.5986
	tmk; thymidylate kinase																							
**SGO_1540**		4.984			-2.147	5.138	0.0124	0.0428		4.984				1.322								1.322		
	YitT family protein																							
**SGO_1541**	-0.363	8.032	0.0624	0.3731	-1.636	7.569	0.0014	0.0025	-2.232	7.454	0.0008	0.0022	-1.273	6.843	0.0107	0.0508	-1.869	6.097	0.0086	0.0335	-0.596	5.759	0.0271	0.0458
	atpC; ATP synthase F1, epsilon subunit																							
**SGO_1542**	0.619	11.040	0.0001	0.0001	-1.008	10.281	0.0002	0.0001	-1.022	10.277	0.0002	0.0002	-1.626	10.208	0.0000	0.0000	-1.641	9.594	0.0001	0.0000	-0.014	9.171	0.2260	0.8480
	atpD; ATP synthase F1, beta subunit																							
**SGO_1543**	0.383	8.135	0.0131	0.0553	-1.291	7.418	0.0002	0.0001	-2.555	7.153	0.0001	0.0001	-1.674	7.196	0.0013	0.0029	-2.938	6.372	0.0011	0.0015	-1.264	5.625	0.0026	0.0005
	atpG; ATP synthase F1, gamma subunit																							
**SGO_1544**	-0.415	10.250	0.0008	0.0014	-0.726	10.124	0.0001	0.0001	-0.675	10.144	0.0002	0.0003	-0.311	9.368	0.0025	0.0072	-0.260	8.780	0.0054	0.0184	0.051	9.229	0.1101	0.3231
	atpA; ATP synthase F1, alpha subunit																							
**SGO_1545**	0.015	8.292	0.1306	0.8681	-1.669	7.677	0.0004	0.0004	-2.474	7.521	0.0002	0.0003	-1.684	7.176	0.0007	0.0008	-2.489	6.412	0.0006	0.0005	-0.805	5.751	0.0010	0.0001
	atpH; ATP synthase F1, delta subunit																							
**SGO_1546**	-1.156	8.155	0.0064	0.0223	-1.026	8.163	0.0016	0.0031	-1.162	8.114	0.0003	0.0005	0.130	7.047	0.1240	0.9498	-0.007	6.360	0.1038	0.7314	-0.137	6.983	0.1164	0.3521
	atpF; ATP synthase F0, B subunit																							
**SGO_1550**	0.171	7.857	0.0596	0.3503	0.394	7.975	0.0120	0.0408	-0.160	7.692	0.0416	0.3558	0.224	7.536	0.0179	0.1003	-0.330	6.657	0.0181	0.0857	-0.554	7.392	0.0092	0.0072
	glgP-1; glycogen phosphorylase																							
**SGO_1551**	2.324	7.034	0.0017	0.0041	0.606	5.888	0.1122	0.5606	0.335	5.734	0.0978	0.9376	-1.718	6.594	0.0009	0.0014	-1.989	5.919	0.0006	0.0006	-0.272	5.395	0.1285	0.4007
	glgA; Glycogen synthase																							

Log$_2$ Ratios

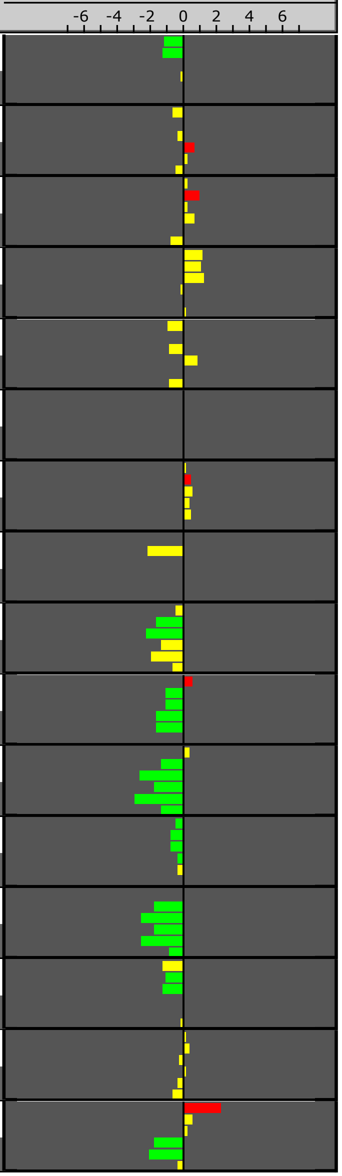

- Show detected proteins only
- Show all proteins
- Filter by category: ABC Transporter

Proteins found: 1179

Dot Plots

Dot Plots

Test	Cutoff
q-Value	.005
p-Value	

	Signif	Direction	Applies To
(red)	yes	+	ratios, bars
(yellow)	no	n/a	bars
(green)	yes	–	ratios, bars
(pink)	yes	+	*p*- , *q*-Values
(light green)	yes	–	

SgFn vs Sg, SgPgFn vs Sg, SgPgFn vs SgFn, SgPg vs Sg, SgPg vs SgFn, SgPgFn vs SgPg

Hendrickson *et al.*

Streptococcus gordonii

Hackett Laboratory UW

Protein	*SgFn vs Sg* Ratio	Sum	*q*-Val	*p*-Val	*SgPg vs Sg* Ratio	Sum	*q*-Val	*p*-Val	*SgPgFn vs Sg* Ratio	Sum	*q*-Val	*p*-Val	*SgPg vs SgFn* Ratio	Sum	*q*-Val	*p*-Val	*SgPgFn vs SgFn* Ratio	Sum	*q*-Val	*p*-Val	*SgPgFn vs SgPg* Ratio	Sum	*q*-Val	*p*-Val
**SGO_1552**	1.390	7.624	0.0016	0.0035	0.946	7.357	0.0188	0.0722	1.172	7.482	0.0036	0.0174	-0.443	7.456	0.0307	0.1890	-0.218	6.928	0.0481	0.2886	0.226	7.345	0.1627	0.5498
	glgD; glucose-1-phosphate adenylyltransferase, GlgD subunit																							
**SGO_1553**	1.980	7.868	0.0023	0.0062	1.515	7.492	0.0004	0.0005	1.201	7.268	0.0007	0.0018	-0.465	7.805	0.0137	0.0711	-0.779	7.072	0.0074	0.0272	-0.314	7.391	0.0050	0.0021
	glgC; glucose-1-phosphate adenylyltransferase																							
**SGO_1554**	2.537	8.474	0.0005	0.0007	1.332	7.526	0.0014	0.0027	1.318	7.518	0.0012	0.0042	-1.204	8.243	0.0008	0.0013	-1.219	7.629	0.0011	0.0015	-0.014	7.510	0.2269	0.8526
	glgB; 1,4-alpha-glucan branching enzyme																							
**SGO_1555**	0.328	11.311	0.0025	0.0069	0.101	11.189	0.0357	0.1513	-0.270	11.011	0.0082	0.0487	-0.227	10.842	0.0007	0.0011	-0.598	10.074	0.0012	0.0019	-0.372	10.552	0.0100	0.0088
	ptsI; phosphoenolpyruvate-protein phosphotransferase																							
**SGO_1556**	0.623	11.703	0.0038	0.0116	0.249	11.490	0.0302	0.1244	1.139	12.044	0.0021	0.0088	-0.374	11.298	0.0151	0.0799	0.516	11.147	0.0126	0.0555	0.890	11.617	0.0158	0.0186
	phosphocarrier protein HPr																							
**SGO_1557**		4.029				4.029				4.029														
	NrdH-redoxin																							
**SGO_1558**	0.723	8.951	0.0056	0.0187	0.059	8.566	0.0833	0.4048	-0.012	8.530	0.0856	0.8069	-0.663	8.459	0.0051	0.0197	-0.735	7.821	0.0049	0.0160	-0.072	8.045	0.0087	0.0065
	nrdE; ribonucleoside-diphosphate reductase large chain																							
**SGO_1559**	0.091	7.735	0.0544	0.3137	0.935	8.231	0.0007	0.0009	1.078	8.326	0.0002	0.0002	0.844	7.748	0.0005	0.0005	0.987	7.230	0.0001	0.0000	0.143	8.182	0.0200	0.0282
	ribonucleoside-diphosphate reductase, beta subunit																							
**SGO_1561**		3.674				3.674				3.674														
	putative zinc metallopeptidase																							
**SGO_1562**		4.516				4.516				4.516														
	hypothetical protein SGO_1562																							
**SGO_1564**		6.147			-0.168	7.022	0.0959	0.4718		6.147				5.371								5.371		
	transcription regulator, probable -related protein																							
**SGO_1570**	-0.511	9.201	0.0126	0.0527	0.166	9.513	0.0562	0.2548	-0.251	9.309	0.0180	0.1358	0.677	8.785	0.0044	0.0160	0.260	7.934	0.0345	0.1952	-0.417	8.885	0.0185	0.0248
	alaS; alanyl-tRNA synthetase																							
**SGO_1571**		4.888				4.888				4.888														
	Protein of unknown function (DUF567) superfamily																							
**SGO_1572**	-4.463	9.699	0.0000	0.0000		9.632			-5.725	9.645	0.0016	0.0062		4.734			-1.262	4.383	0.0507	0.3099		2.394		
	proteinase maturation protein, putative																							
**SGO_1573**	1.276	3.945			0.121	3.873	0.1440	0.7432		2.170			-1.155	3.882	0.0245	0.1448		2.322				2.830		
	O-methyltransferase family protein																							
**SGO_1574**	0.665	8.801	0.0015	0.0034	1.082	9.069	0.0001	0.0000	0.810	8.890	0.0002	0.0002	0.417	8.805	0.0019	0.0047	0.145	8.046	0.0233	0.1197	-0.272	8.868	0.0050	0.0020
	pepF-1; oligoendopeptidase F																							

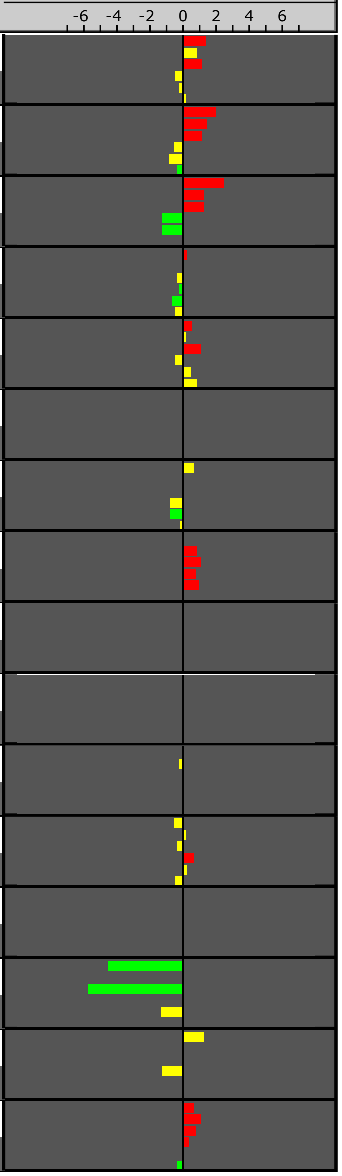

◉ Show detected proteins only
○ Show all proteins
☐ Filter by category:
ABC Transporter

Proteins found: 1179

Dot Plots
Dot Plots

Test	Cutoff
q-Value	.005
p-Value	

	Signif	Direction	Applies To
red	yes	+	ratios, bars
yellow	no	n/a	bars
green	yes	–	ratios, bars
light red	yes	+	*p*- , *q*-Values
light green	yes	–	*p*- , *q*-Values

Hendrickson *et al.*

Streptococcus gordonii

Hackett Laboratory UW

Summary Table | SgFn vs Sg | SgPg vs Sg | SgPgFn vs Sg | SgPg v SgFn | SgPgFn v SgFn | SgPgFn vs SgPg | Coverage

Protein	Description	SgFn vs Sg Ratio	SgFn vs Sg Sum	SgFn vs Sg *q*-Val	SgFn vs Sg *p*-Val	SgPg vs Sg Ratio	SgPg vs Sg Sum	SgPg vs Sg *q*-Val	SgPg vs Sg *p*-Val	SgPgFn vs Sg Ratio	SgPgFn vs Sg Sum	SgPgFn vs Sg *q*-Val	SgPgFn vs Sg *p*-Val	SgPg vs SgFn Ratio	SgPg vs SgFn Sum	SgPg vs SgFn *q*-Val	SgPg vs SgFn *p*-Val	SgPgFn vs SgFn Ratio	SgPgFn vs SgFn Sum	SgPgFn vs SgFn *q*-Val	SgPgFn vs SgFn *p*-Val	SgPgFn vs SgPg Ratio	SgPgFn vs SgPg Sum	SgPgFn vs SgPg *q*-Val	SgPgFn vs SgPg *p*-Val
SGO_1576	ptcC; PTS system, IIC component		2.832				2.832				2.832														
SGO_1580	PTS system, Lactose/Cellobiose specific IIB subunit		3.178				4.634				4.506			1.452	4.570	0.0007	0.0009	1.302	3.866	0.0039	0.0118	-0.150	5.058	0.1529	0.4943
SGO_1585	D-Alanyl-D-Alanine carboxypeptidase		6.024			-2.854	6.245	0.0039	0.0104	-2.399	6.151	0.0222	0.1761		2.920				1.461			0.456	3.557	0.2327	0.8803
SGO_1587	queA; S-adenosylmethionine:tRNA ribosyltransferase-isomerase	0.505	6.189	0.0397	0.2167	0.437	6.170	0.0692	0.3251	-0.217	5.809	0.0458	0.3997	-0.068	5.865	0.1190	0.9045	-0.722	4.947	0.0075	0.0278	-0.654	5.537	0.0426	0.0902
SGO_1588	arcR; arginine repressor		3.890				3.890				3.890														
SGO_1589	arcT; putative transaminase/peptidase	2.726	6.450	0.0001	0.0000		3.546				3.546				5.730				5.120						
SGO_1590	arcD; arginine-ornithine antiporter	-1.292	7.433	0.0008	0.0014		6.934				6.934				5.146				4.535						
SGO_1591	arcC; carbamate kinase	1.169	8.010	0.0009	0.0015	-1.436	6.786	0.0047	0.0131	-1.727	6.702	0.0018	0.0070	-2.605	7.184	0.0002	0.0001	-2.896	6.530	0.0001	0.0000	-0.291	5.239	0.0865	0.2333
SGO_1592	arcB; ornithine carbamoyltransferase	2.366	11.142	0.0009	0.0016	-0.713	9.197	0.0000	0.0000	-0.397	9.329	0.0032	0.0149	-3.079	10.535	0.0008	0.0012	-2.763	9.962	0.0010	0.0014	0.316	8.455	0.0271	0.0457
SGO_1593	arcA; arginine deiminase	1.771	10.509	0.0002	0.0001	-0.870	8.998	0.0009	0.0015	-0.377	9.191	0.0021	0.0090	-2.641	9.840	0.0002	0.0001	-2.148	9.308	0.0002	0.0001	0.493	8.251	0.0030	0.0007
SGO_1594	Crp/Fnr family protein		3.486				3.486				3.486														
SGO_1598	hypothetical protein SGO_1598		2.836				2.836				2.836														
SGO_1599	sodA; manganese-dependent superoxide dismutase	2.054	10.580	0.0002	0.0002	0.417	9.437	0.0020	0.0044	0.277	9.360	0.0057	0.0308	-1.637	10.158	0.0003	0.0002	-1.777	9.514	0.0004	0.0002	-0.140	9.050	0.0520	0.1195
SGO_1600	DNA polymerase III, delta chain		3.975				3.975				3.975														
SGO_1601	celB; DNA internalization-related competence protein celB		2.604				2.604				2.604														
SGO_1604	acyltransferase family protein	-1.738	6.821	0.0006	0.0009	-2.382	6.699	0.0006	0.0007	-3.267	6.516	0.0101	0.0647	-0.644	4.907	0.0028	0.0085	-1.529	3.806	0.0025	0.0063	-0.886	3.902	0.0769	0.2010

Log$_2$ Ratios

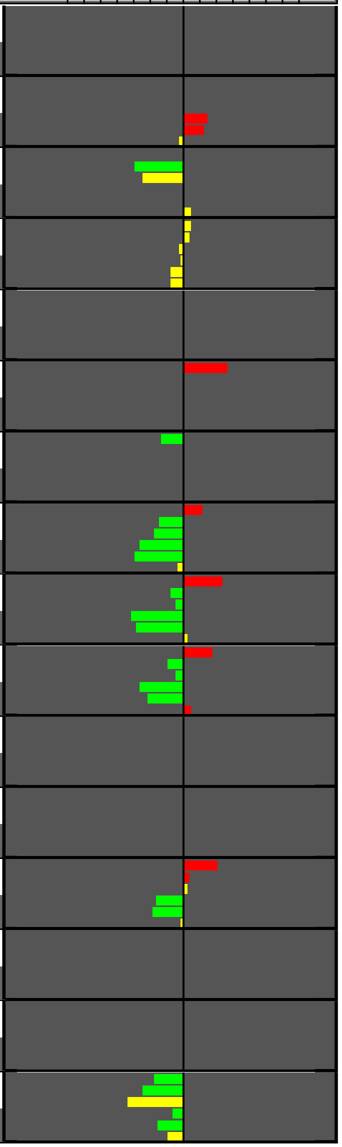

Show detected proteins only
Show all proteins
Filter by category:
ABC Transporter

Proteins found:
1179

Dot Plots
Dot Plots

Test	Cutoff
q-Value	.005
p-Value	

	Signif	Direction	Applies To
(red)	yes	+	ratios, bars
(yellow)	no	n/a	bars
(green)	yes	−	ratios, bars
(light red)	yes	+	*p*- , *q*-Values
(light green)	yes	−	

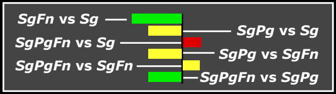

Hendrickson *et al.*

Spectral Counts
Sg Summary Table

Streptococcus gordonii

Hackett Laboratory UW

Summary Table | SgFn vs Sg | SgPg vs Sg | SgPgFn vs Sg | SgPg v SgFn | SgPgFn v SgFn | SgPgFn vs SgPg | Coverage

Protein	*SgFn vs Sg*				*SgPg vs Sg*				*SgPgFn vs Sg*				*SgPg vs SgFn*				*SgPgFn vs SgFn*				*SgPgFn vs SgPg*				Description
	Ratio	Sum	*q*-Val	*p*-Val	Ratio	Sum	*q*-Val	*p*-Val	Ratio	Sum	*q*-Val	*p*-Val	Ratio	Sum	*q*-Val	*p*-Val	Ratio	Sum	*q*-Val	*p*-Val	Ratio	Sum	*q*-Val	*p*-Val	
**SGO_1605**	-3.386	6.286	0.0004	0.0005	-1.656	6.552	0.0008	0.0012	-2.978	6.243	0.0106	0.0686	1.730	4.362	0.0005	0.0005	0.408	2.373	0.0156	0.0719	-1.322	4.245	0.0396	0.0791	P-type ATPase, metal cation transport
**SGO_1606**		4.275			-0.708	4.993	0.0157	0.0575		4.275				3.129								3.129			DNA methyltransferase signature protein
**SGO_1609**	-2.177	7.848	0.0001	0.0000	-0.739	8.236	0.0002	0.0001	-0.912	8.175	0.0003	0.0004	1.437	6.760	0.0001	0.0000	1.265	6.028	0.0008	0.0009	-0.173	7.225	0.0366	0.0712	ATP-dependent RNA helicase, DEAD/DEAH box family
**SGO_1614**		4.239				4.239				4.239															magnesium and cobalt transporter CorA family
**SGO_1617**	0.495	6.407	0.0360	0.1921	0.372	6.364	0.0788	0.3791	0.146	6.211	0.0823	0.7733	-0.123	6.060	0.1068	0.7985	-0.349	5.319	0.0224	0.1137	-0.226	5.887	0.1256	0.3856	prfC; peptide chain release factor 3
**SGO_1619**	-2.956	9.529	0.0009	0.0016	-1.786	9.723	0.0017	0.0033	-1.811	9.715	0.0009	0.0029	1.170	7.584	0.0024	0.0068	1.145	6.949	0.0009	0.0012	-0.025	8.040	0.2193	0.8120	cation-transporting ATPase, E1-E2 family
**SGO_1621**	-0.202	5.929	0.0227	0.1077	-0.354	5.860	0.0078	0.0242	-1.626	5.242	0.0106	0.0693	-0.151	5.237	0.0278	0.1678	-1.424	3.946	0.0167	0.0775	-1.273	4.429	0.0358	0.0691	HD domain protein
**SGO_1622**	1.367	6.408	0.0013	0.0026	-0.155	5.499	0.0807	0.3914	0.164	5.675	0.0620	0.5594	-1.521	5.844	0.0006	0.0006	-1.203	5.334	0.0019	0.0044	0.319	5.077	0.0728	0.1866	Cof family protein
**SGO_1623**	-0.490	6.963	0.0014	0.0030	-0.997	6.773	0.0003	0.0003	-1.620	6.404	0.0071	0.0405	-0.507	5.952	0.0012	0.0023	-1.129	4.870	0.0131	0.0586	-0.623	5.081	0.0248	0.0397	murM; MurM
**SGO_1624**	0.137	6.102	0.1018	0.6598	-0.926	5.632	0.0115	0.0389	-0.601	5.436	0.0431	0.3719	-1.064	5.206	0.0060	0.0240	-0.738	4.411	0.0501	0.3047	0.326	4.258	0.0656	0.1623	murN; MurN protein
**SGO_1625**	-0.506	8.384	0.0006	0.0009	-1.236	8.124	0.0001	0.0000	-2.134	7.916	0.0001	0.0001	-0.730	7.277	0.0008	0.0013	-1.628	6.398	0.0008	0.0009	-0.898	6.496	0.0094	0.0077	acetoin utilization putative/CBS domain protein
**SGO_1626**	-2.040	6.616	0.0213	0.0998	-0.735	7.128	0.0047	0.0132	-0.741	7.124	0.0024	0.0104	1.305	5.463	0.0170	0.0947	1.299	4.843	0.0101	0.0412	-0.006	6.193	0.2367	0.9031	branched-chain amino acid ABC transporter, ATP-binding protein
**SGO_1627**		6.853			-1.163	7.382	0.0038	0.0099	-1.098	7.407	0.0031	0.0142		5.163				4.633			0.065	6.204	0.1828	0.6418	branched-chain amino acid ABC transporter, ATP-binding protein
**SGO_1630**	-3.259	10.217	0.0000	0.0000	-1.166	10.605	0.0001	0.0000	-1.610	10.482	0.0001	0.0000	2.093	8.698	0.0000	0.0000	1.649	7.739	0.0001	0.0000	-0.444	9.189	0.0012	0.0001	branched-chain amino acid ABC transporter, amino acid-binding protein
**SGO_1631**		3.428				3.428				3.428															hypothetical protein SGO_1631
**SGO_1632**	1.202	8.084	0.0031	0.0090	-0.250	7.229	0.0036	0.0092	0.368	7.633	0.0375	0.3174	-1.451	7.500	0.0023	0.0064	-0.833	7.137	0.0239	0.1245	0.618	7.022	0.0768	0.1997	clpP; ATP-dependent Clp protease, proteolytic subunit ClpP

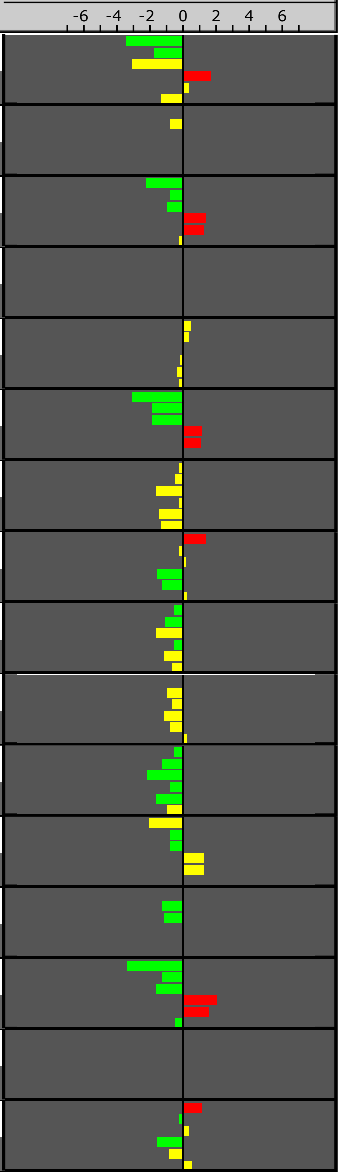

◉ Show detected proteins only
○ Show all proteins
☐ Filter by category:
ABC Transporter

Proteins found:
1179

Dot Plots
Dot Plots

Test	Cutoff
***q*-Value**	**.005**
p-Value	

	Signif	Direction	Applies To
(red)	yes	+	ratios, bars
(yellow)	no	n/a	bars
(green)	yes	−	ratios, bars
(pink)	yes	+	*p*- , *q*-Values
(light green)	yes	−	

SgFn vs Sg
SgPgFn vs Sg
SgPgFn vs SgFn
SgPg vs Sg
SgPg vs SgFn
SgPgFn vs SgPg

Hendrickson *et al.*

Streptococcus gordonii

Protein	SgFn vs Sg Ratio	SgFn vs Sg Sum	SgFn vs Sg *q*-Val	SgFn vs Sg *p*-Val	SgPg vs Sg Ratio	SgPg vs Sg Sum	SgPg vs Sg *q*-Val	SgPg vs Sg *p*-Val	SgPgFn vs Sg Ratio	SgPgFn vs Sg Sum	SgPgFn vs Sg *q*-Val	SgPgFn vs Sg *p*-Val	SgPg vs SgFn Ratio	SgPg vs SgFn Sum	SgPg vs SgFn *q*-Val	SgPg vs SgFn *p*-Val	SgPgFn vs SgFn Ratio	SgPgFn vs SgFn Sum	SgPgFn vs SgFn *q*-Val	SgPgFn vs SgFn *p*-Val	SgPgFn vs SgPg Ratio	SgPgFn vs SgPg Sum	SgPgFn vs SgPg *q*-Val	SgPgFn vs SgPg *p*-Val
**SGO_1633**	1.254	8.358	0.0018	0.0044	0.909	8.114	0.0001	0.0000	0.812	8.067	0.0046	0.0242	-0.346	8.175	0.0121	0.0602	-0.442	7.533	0.0195	0.0946	-0.097	7.949	0.1841	0.6500
upp; uracil phosphoribosyltransferase																								
**SGO_1634**	-4.978	9.104	0.0000	0.0000	-5.111	9.102	0.0000	0.0000		9.059			-0.133	4.535	0.1063	0.7935		2.958				3.500		
magnesium-translocating P-type ATPase																								
**SGO_1638**	1.909	5.438	0.0023	0.0061	1.421	5.055	0.0014	0.0027		3.205			-0.488	5.348	0.0125	0.0623		3.968				4.073		
murE; UDP-N-acetylmuramoylalanyl-D-glutamate--2,6-diaminopimelate ligase																								
**SGO_1648**	0.910	8.722	0.0009	0.0016	1.546	9.176	0.0020	0.0044	1.285	8.978	0.0004	0.0007	0.636	8.950	0.0052	0.0203	0.375	8.178	0.0014	0.0026	-0.261	9.108	0.0541	0.1270
ppx1; inorganic pyrophosphatase, manganese-dependent																								
**SGO_1649**	-0.303	6.470	0.0116	0.0477	-2.242	5.905	0.0010	0.0017	-2.213	5.763	0.0122	0.0831	-1.939	5.143	0.0006	0.0007	-1.910	4.362	0.0027	0.0073	0.029	3.498	0.2429	0.9375
act; pyruvate formate-lyase-activating enzyme																								
**SGO_1650**		7.977				7.977				7.977														
LPXTG cell wall surface protein, collagen binding domain																								
**SGO_1651**	-2.162	9.827	0.0000	0.0000		9.536				9.536				6.862				6.251						
LPXTG cell wall surface protein, nuclease/phosphatase domain																								
**SGO_1652**	-0.823	6.776	0.0058	0.0195	0.184	7.218	0.0578	0.2631	-0.012	7.118	0.0881	0.8335	1.008	6.371	0.0005	0.0005	0.811	5.633	0.0010	0.0012	-0.197	6.691	0.0007	0.0000
intracellular glycosyl hydrolase																								
**SGO_1653**	-2.067	8.997	0.0002	0.0002	-2.054	8.998	0.0002	0.0001	-2.355	8.961	0.0004	0.0007	0.013	7.115	0.1226	0.9373	-0.288	6.406	0.0813	0.5500	-0.301	7.020	0.1579	0.5204
trehalose PTS enzyme II																								
**SGO_1655**	-1.918	7.001	0.0023	0.0060	-2.739	6.857	0.0016	0.0030		6.653			-0.821	4.901	0.0162	0.0886		3.653				3.417		
CBS domain protein/possible hemolysin																								
**SGO_1657**		4.486				4.486				4.486														
membrane protein																								
**SGO_1661**	0.772	4.627	0.0794	0.4944		3.822				3.822				2.887				2.277						
radical SAM protein, TIGR01212 family																								
**SGO_1665**		2.585				2.585				2.585														
rRNA methylase																								
**SGO_1666**	0.784	7.821	0.0022	0.0057	-0.970	6.980	0.0056	0.0162	-2.475	6.630	0.0011	0.0038	-1.754	7.016	0.0001	0.0000	-3.259	6.176	0.0001	0.0000	-1.505	5.335	0.0052	0.0026
trkA; potassium uptake protein, Trk family																								
**SGO_1669**	-0.888	7.256	0.0099	0.0386	-0.728	7.307	0.0126	0.0436	-0.722	7.304	0.0063	0.0352	0.160	6.308	0.0762	0.5497	0.166	5.693	0.0746	0.4987	0.006	6.372	0.2463	0.9553
ribosomal large subunit pseudouridine synthase B																								
**SGO_1670**		2.379				2.379				2.379														
scpB; segregation and condensation protein B																								

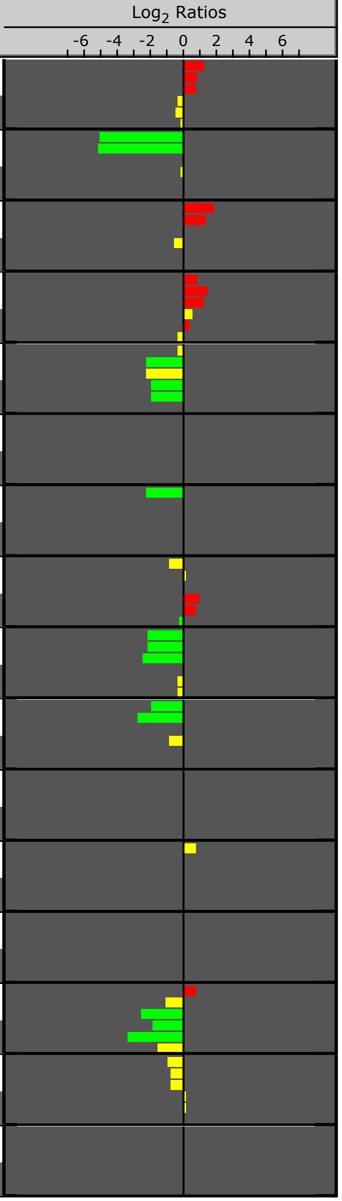

Show detected proteins only
Show all proteins
Filter by category:
ABC Transporter

Proteins found: 1179

Dot Plots
Dot Plots

Test	Cutoff
q-Value	.005
p-Value	

	Signif	Direction	Applies To
red	yes	+	ratios, bars
yellow	no	n/a	bars
green	yes	–	ratios, bars
light red	yes	+	*p*- , *q*-Values
light green	yes	–	*p*- , *q*-Values

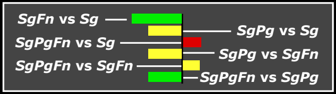

Protein	*SgFn vs Sg* Ratio	Sum	*q*-Val	*p*-Val	*SgPg vs Sg* Ratio	Sum	*q*-Val	*p*-Val	*SgPgFn vs Sg* Ratio	Sum	*q*-Val	*p*-Val	*SgPg vs SgFn* Ratio	Sum	*q*-Val	*p*-Val	*SgPgFn vs SgFn* Ratio	Sum	*q*-Val	*p*-Val	*SgPgFn vs SgPg* Ratio	Sum	*q*-Val	*p*-Val
**SGO_1674**	0.340	5.823	0.0050	0.0159	-0.643	5.361	0.0040	0.0107		4.646			-0.983	5.062	0.0007	0.0010		3.858				3.494		
	phosphodiesterase, MJ0936 family																							
**SGO_1675**	0.255	6.690	0.0603	0.3569	-0.654	6.276	0.0214	0.0834	-0.814	6.224	0.0104	0.0676	-0.908	5.903	0.0027	0.0079	-1.069	5.244	0.0031	0.0088	-0.161	5.315	0.1661	0.5658
	HAM1 protein-like protein																							
**SGO_1676**	0.999	5.350	0.0014	0.0030	1.692	5.845	0.0003	0.0002	1.047	5.395	0.0034	0.0164	0.694	5.633	0.0003	0.0002	0.048	4.668	0.0972	0.6784	-0.645	5.659	0.0113	0.0108
	murI; glutamate racemase																							
**SGO_1678**	1.280	6.005	0.0026	0.0070	0.029	5.328	0.1365	0.6999	-0.494	4.666	0.0386	0.3278	-1.251	5.548	0.0083	0.0379	-1.774	4.577	0.0233	0.1208	-0.523	4.291	0.1651	0.5609
	lysA; diaminopimelate decarboxylase																							
**SGO_1679**	-2.373	10.552	0.0000	0.0000	-1.278	10.798	0.0004	0.0003	-1.444	10.749	0.0001	0.0000	1.095	9.069	0.0023	0.0062	0.929	8.341	0.0012	0.0019	-0.165	9.433	0.0933	0.2616
	phosphotransferase system enzyme II																							
**SGO_1680**		7.797			-2.691	8.003	0.0007	0.0010	-1.867	7.981	0.0139	0.0989		4.584				3.796			0.823	5.498	0.0228	0.0348
	phosphotransferase system enzyme II																							
**SGO_1681**	-2.678	10.136	0.0001	0.0000	-1.826	10.284	0.0000	0.0000	-1.822	10.287	0.0001	0.0001	0.852	8.227	0.0011	0.0020	0.855	7.626	0.0043	0.0131	0.003	8.594	0.2354	0.8967
	PTS system, mannose/fructose/sorbose family, IID component																							
**SGO_1683**	0.183	8.135	0.0676	0.4105	0.563	8.319	0.0012	0.0021	0.372	8.211	0.0043	0.0219	0.380	7.907	0.0346	0.2185	0.189	7.196	0.0866	0.5936	-0.191	7.970	0.0457	0.0994
	serS; seryl-tRNA synthetase																							
**SGO_1684**		4.578			1.477	6.486	0.0012	0.0020	1.486	6.497	0.0013	0.0044		5.526				4.931			0.009	6.534	0.2382	0.9134
	acyl-CoA dehydrogenase family																							
**SGO_1685**	1.132	6.834	0.0008	0.0013	2.779	8.132	0.0001	0.0000	3.050	8.386	0.0008	0.0023	1.647	7.820	0.0001	0.0000	1.918	7.432	0.0017	0.0038	0.271	8.571	0.0582	0.1392
	putative peroxidase / antioxidase																							
**SGO_1687**	-0.326	7.903	0.0149	0.0652	-0.031	8.036	0.1317	0.6708	0.122	8.112	0.0045	0.0231	0.295	7.374	0.0188	0.1060	0.448	6.848	0.0070	0.0253	0.153	7.586	0.0385	0.0760
	accA; acetyl-CoA carboxylase, carboxyl transferase, alpha subunit																							
**SGO_1688**	0.081	6.923	0.1412	0.9507	-0.423	6.692	0.0434	0.1883	-1.406	6.366	0.0063	0.0357	-0.504	6.188	0.0000	0.0000	-1.487	5.254	0.0006	0.0005	-0.983	5.513	0.0057	0.0032
	accD; acetyl-CoA carboxylase, carboxyl transferase, beta subunit																							
**SGO_1689**	1.308	7.985	0.0008	0.0013	0.770	7.623	0.0018	0.0039	0.660	7.554	0.0016	0.0063	-0.538	7.735	0.0021	0.0051	-0.648	7.080	0.0015	0.0031	-0.110	7.385	0.0381	0.0748
	accC; acetyl-CoA carboxylase, biotin carboxylase																							
**SGO_1690**		5.237				5.878				3.396			0.686	6.080	0.0188	0.1070	-0.770	4.468	0.0601	0.3863	-1.456	5.603	0.0713	0.1821
	fabZ; beta-hydroxyacyl-(acyl-carrier-protein) dehydratase FabZ																							
**SGO_1691**	-0.102	7.422	0.1200	0.7870	1.379	8.276	0.0017	0.0036	0.766	7.873	0.0089	0.0549	1.481	7.734	0.0013	0.0027	0.867	6.712	0.0097	0.0386	-0.614	8.006	0.0102	0.0092
	accB; acetyl-CoA carboxylase, biotin carboxyl carrier protein																							
**SGO_1692**	2.086	10.166	0.0004	0.0004	1.429	9.656	0.0001	0.0000	0.957	9.329	0.0002	0.0002	-0.657	10.055	0.0015	0.0034	-1.129	9.280	0.0009	0.0011	-0.472	9.471	0.0019	0.0003
	3-oxoacyl-[acyl-carrier-protein] synthase																							

Log$_2$ Ratios: -6 -4 -2 0 2 4 6

Show detected proteins only
Show all proteins
Filter by category:
ABC Transporter

Proteins found: 1179

Dot Plots
Dot Plots

Test	Cutoff
q-Value	.005
p-Value	

	Signif	Direction	Applies To
red	yes	+	ratios, bars
yellow	no	n/a	bars
green	yes	−	ratios, bars
light red	yes	+	*p*- , *q*-Values
light green	yes	−	

SgFn vs Sg — SgPg vs Sg
SgPgFn vs Sg — SgPg vs SgFn
SgPgFn vs SgFn — SgPgFn vs SgPg

Hendrickson *et al.*

Streptococcus gordonii

Summary Table | SgFn vs Sg | SgPg vs Sg | SgPgFn vs Sg | SgPg v SgFn | SgPgFn v SgFn | SgPgFn vs SgPg | Coverage

Protein	*SgFn vs Sg* Ratio	Sum	*q*-Val	*p*-Val	*SgPg vs Sg* Ratio	Sum	*q*-Val	*p*-Val	*SgPgFn vs Sg* Ratio	Sum	*q*-Val	*p*-Val	*SgPg vs SgFn* Ratio	Sum	*q*-Val	*p*-Val	*SgPgFn vs SgFn* Ratio	Sum	*q*-Val	*p*-Val	*SgPgFn vs SgPg* Ratio	Sum	*q*-Val	*p*-Val	Description
SGO_1693	1.153	7.330	0.0003	0.0003	0.871	7.143	0.0014	0.0026	0.329	6.844	0.0297	0.2420	-0.282	7.146	0.0033	0.0107	-0.824	6.332	0.0053	0.0180	-0.542	6.773	0.0397	0.0812	fabG; 3-oxoacyl-(acyl-carrier-protein) reductase
SGO_1694	0.376	6.814	0.0294	0.1515	1.047	7.221	0.0027	0.0064	0.254	6.757	0.0396	0.3378	0.671	6.845	0.0067	0.0276	-0.122	5.819	0.1009	0.7086	-0.793	6.806	0.0204	0.0291	fabD; malonyl CoA-acyl carrier protein transacylase
SGO_1695	0.088	9.310	0.0294	0.1501	0.870	9.770	0.0028	0.0067	0.383	9.478	0.0104	0.0673	0.782	9.289	0.0030	0.0092	0.295	8.393	0.0233	0.1203	-0.487	9.411	0.0306	0.0534	enoyl-acyl carrier protein(ACP) reductase
SGO_1698		4.505			-0.756	5.106	0.0515	0.2316		4.505				3.040								3.040			3-oxoacyl-[acyl-carrier-protein] synthase III
SGO_1699	1.460	6.054	0.0002	0.0002	0.913	5.685	0.0055	0.0160	0.250	4.823	0.0440	0.3827	-0.547	5.849	0.0043	0.0152	-1.210	4.762	0.0048	0.0154	-0.663	4.951	0.0888	0.2445	transcriptional regulator, MarR family
SGO_1700	2.949	8.270	0.0006	0.0010	0.135	6.207	0.0097	0.0318	-1.156	5.429	0.0016	0.0063	-2.813	7.773	0.0007	0.0010	-4.105	7.012	0.0167	0.0776	-1.291	5.028	0.0264	0.0438	enoyl-CoA hydratase/isomerase family protein
SGO_1701	0.868	7.787	0.0013	0.0027	0.897	7.802	0.0001	0.0000	0.450	7.536	0.0079	0.0461	0.028	7.657	0.1026	0.7627	-0.419	6.844	0.0121	0.0527	-0.447	7.468	0.0184	0.0246	aspartate kinase
SGO_1708	-2.226	9.714	0.0047	0.0146	-1.595	9.842	0.0073	0.0220	-1.843	9.787	0.0037	0.0183	0.630	8.003	0.0022	0.0059	0.383	7.250	0.0103	0.0432	-0.248	8.165	0.0330	0.0603	amiF; Oligopeptide transport ATP-binding protein amiF
SGO_1709	-3.054	9.803	0.0000	0.0000	-1.584	10.055	0.0001	0.0000	-1.751	10.017	0.0001	0.0001	1.471	7.990	0.0006	0.0007	1.303	7.267	0.0024	0.0059	-0.168	8.469	0.1043	0.3029	amiE; Oligopeptide transport ATP-binding protein
SGO_1710		6.608			-2.268	6.883	0.0003	0.0002	-1.800	6.971	0.0002	0.0002		3.844				3.682			0.468	5.086	0.0227	0.0342	amiD; Oligopeptide transport system permease protein
SGO_1711	-2.691	8.366	0.0007	0.0012	-1.467	8.602	0.0014	0.0026	-1.236	8.671	0.0013	0.0049	1.224	6.684	0.0002	0.0001	1.455	6.248	0.0017	0.0038	0.231	7.297	0.0594	0.1436	hppB; Oligopeptide transport system permease
SGO_1712	-1.923	10.854	0.0003	0.0003	-1.011	11.090	0.0001	0.0000	-0.769	11.175	0.0001	0.0001	0.912	9.612	0.0027	0.0079	1.154	9.163	0.0016	0.0033	0.242	10.111	0.0051	0.0024	hppA; oligopeptide-binding lipoprotein
SGO_1713	-1.214	10.762	0.0008	0.0013	-1.386	10.708	0.0004	0.0004	-1.142	10.780	0.0003	0.0005	-0.172	9.436	0.0368	0.2370	0.072	8.945	0.0937	0.6499	0.244	9.467	0.0073	0.0050	hppG; oligopeptide-binding lipoprotein
SGO_1715	-1.347	9.552	0.0001	0.0000	-1.406	9.535	0.0001	0.0000	-1.403	9.537	0.0001	0.0000	-0.060	8.185	0.0447	0.2998	-0.056	7.577	0.0676	0.4455	0.004	8.157	0.2404	0.9264	hppH; oligopeptide-binding lipoprotein
SGO_1716	-0.893	9.573	0.0026	0.0072	-0.939	9.553	0.0017	0.0034	-0.817	9.616	0.0059	0.0323	-0.045	8.522	0.0912	0.6716	0.076	8.001	0.0978	0.6839	0.121	8.584	0.1586	0.5254	oligopeptide binding protein
SGO_1717	-2.333	6.693	0.0016	0.0037	-3.837	6.483	0.0278	0.1132		6.434			-1.505	3.809	0.0180	0.1005		2.965				1.063			pbp3; penicillin-binding protein 3

Log_2 Ratios

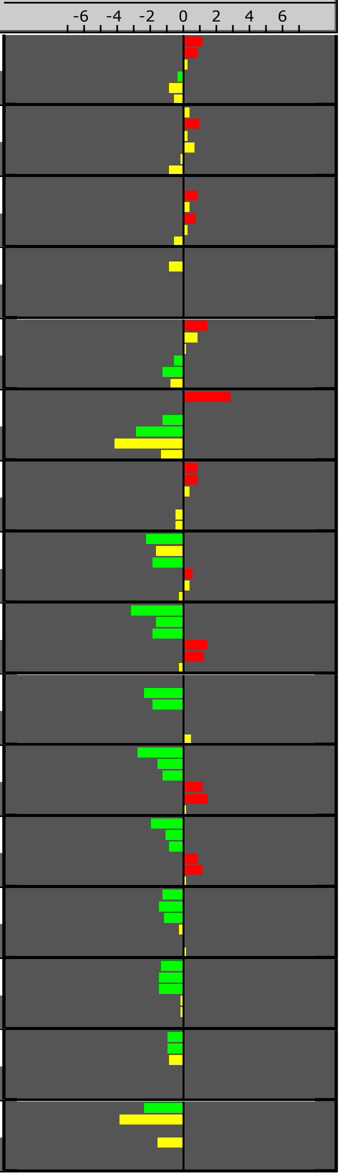

Show detected proteins only
Show all proteins
Filter by category:
ABC Transporter

Proteins found:
1179

Dot Plots
Dot Plots

Test	Cutoff
q-Value	.005
p-Value	

	Signif	Direction	Applies To
red	yes	+	ratios, bars
yellow	no	n/a	bars
green	yes	−	ratios, bars
pink	yes	+	*p*- , *q*-Values
light green	yes	−	

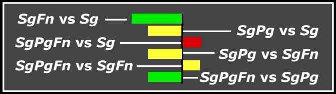

Hendrickson *et al.*

Streptococcus gordonii

Protein	Description	SgFn vs Sg Ratio	Sum	*q*-Val	*p*-Val	SgPg vs Sg Ratio	Sum	*q*-Val	*p*-Val	SgPgFn vs Sg Ratio	Sum	*q*-Val	*p*-Val	SgPg vs SgFn Ratio	Sum	*q*-Val	*p*-Val	SgPgFn vs SgFn Ratio	Sum	*q*-Val	*p*-Val	SgPgFn vs SgPg Ratio	Sum	*q*-Val	*p*-Val
SGO_1718	sufB-1; FeS assembly protein SufB	-0.376	7.137	0.0406	0.2227	0.192	7.395	0.0742	0.3537	-0.096	7.275	0.0867	0.8195	0.568	6.721	0.0097	0.0454	0.280	5.976	0.0608	0.3913	-0.288	6.851	0.1156	0.3481
SGO_1719	SUF system FeS assembly protein, NifU family		2.946				2.946				2.946														
SGO_1720	aminotransferase, class-V		3.975			1.646	5.980	0.0008	0.0011	0.593	5.293	0.0194	0.1486		5.053				3.429			-1.053	5.634	0.0073	0.0051
SGO_1721	sufD; FeS assembly protein SufD	0.391	7.322	0.0192	0.0878	0.589	7.430	0.0033	0.0083	0.411	7.324	0.0029	0.0128	0.197	7.090	0.0384	0.2496	0.020	6.386	0.1345	0.9787	-0.178	7.092	0.0300	0.0521
SGO_1722	sufC; FeS assembly ATPase SufC	0.722	6.904	0.0017	0.0039	0.437	6.734	0.0079	0.0243	0.109	6.599	0.0647	0.5864	-0.285	6.570	0.0075	0.0318	-0.613	5.854	0.0224	0.1139	-0.328	6.304	0.1360	0.4258
SGO_1724	mecA; Adapter protein mec	0.591	4.101	0.0794	0.4935	-0.127	3.848	0.1462	0.7567		3.339			-0.717	2.988				1.692				1.585		
SGO_1725	bacA; bacitracin resistance protein		4.038				4.038				4.038														
SGO_1726	hypothetical protein SGO_1726		4.724				4.724				4.724														
SGO_1727	amino acid ABC transporter, amino acid-binding/permease protein	-1.722	7.502	0.0003	0.0003	-0.949	7.719	0.0005	0.0007	-1.039	7.688	0.0002	0.0003	0.773	6.329	0.0041	0.0141	0.683	5.661	0.0048	0.0156	-0.089	6.612	0.1273	0.3962
SGO_1728	glnQ; glutamine ABC transporter ATP-binding protein	-0.925	7.589	0.0068	0.0241	-1.549	7.411	0.0042	0.0114	-1.188	7.505	0.0032	0.0153	-0.625	6.253	0.0044	0.0160	-0.263	5.786	0.0020	0.0047	0.361	6.099	0.0393	0.0781
SGO_1729	hypothetical protein SGO_1729		9.102				6.111				5.593			-2.961	8.759	0.0025	0.0072	-3.517	8.099	0.0025	0.0064	-0.556	6.362	0.0426	0.0903
SGO_1730	SPFH domain/Band 7 family	0.138	10.208	0.0639	0.3830	-1.409	9.596	0.0011	0.0019	-1.177	9.659	0.0006	0.0016	-1.547	9.192	0.0021	0.0053	-1.314	8.639	0.0024	0.0058	0.232	8.334	0.0701	0.1788
SGO_1731	DNA-binding response regulator	0.649	4.113	0.0366	0.1959	2.963	5.857	0.0014	0.0026	2.608	5.540	0.0004	0.0007	2.314	5.420	0.0013	0.0029	1.960	4.510	0.0006	0.0004	-0.354	5.984	0.0428	0.0909
SGO_1732	histidine kinase		4.686			0.137	5.243	0.1748	0.9291		4.686				3.087								3.087		
SGO_1735	hypothetical protein SGO_1735	-0.145	9.384	0.0589	0.3452	0.067	9.482	0.0791	0.3811	-0.245	9.332	0.0086	0.0525	0.211	8.904	0.0244	0.1434	-0.100	8.136	0.0649	0.4266	-0.312	8.854	0.0071	0.0047
SGO_1736	alkaline shock protein	1.192	6.293	0.0068	0.0244	0.919	6.090	0.0042	0.0115	-0.151	5.089	0.0568	0.5072	-0.273	6.124	0.0440	0.2941	-1.343	4.908	0.0394	0.2279	-1.070	5.272	0.0656	0.1608

Log$_2$ Ratios

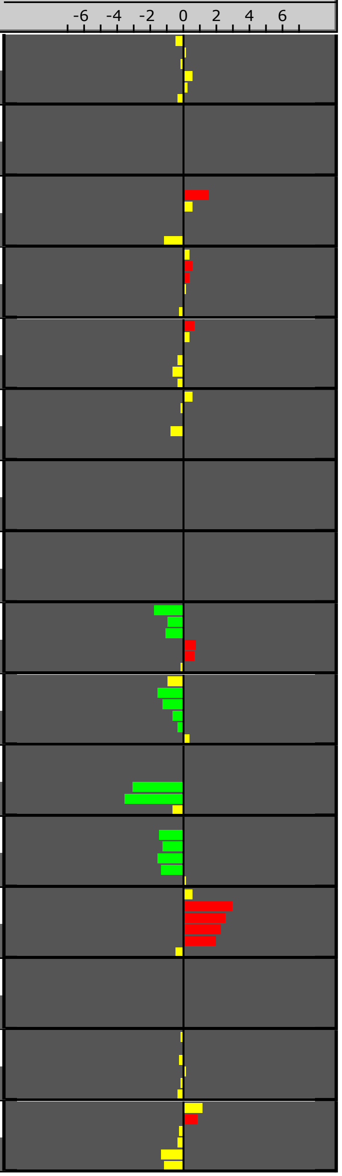

- Show detected proteins only
- Show all proteins
- Filter by category: ABC Transporter

Proteins found: 1179

Dot Plots
Dot Plots

Test	Cutoff
q-Value	.005
p-Value	

Colour	Signif	Direction	Applies To
red	yes	+	ratios, bars
yellow	no	n/a	bars
green	yes	−	ratios, bars
pink	yes	+	*p*- , *q*-Values
light green	yes	−	*p*- , *q*-Values

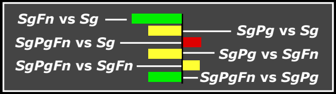

Hendrickson *et al.*

Summary Table | SgFn vs Sg | SgPg vs Sg | SgPgFn vs Sg | SgPg v SgFn | SgPgFn v SgFn | SgPgFn vs SgPg | Coverage

Protein	SgFn vs Sg Ratio	SgFn vs Sg Sum	SgFn vs Sg *q*-Val	SgFn vs Sg *p*-Val	SgPg vs Sg Ratio	SgPg vs Sg Sum	SgPg vs Sg *q*-Val	SgPg vs Sg *p*-Val	SgPgFn vs Sg Ratio	SgPgFn vs Sg Sum	SgPgFn vs Sg *q*-Val	SgPgFn vs Sg *p*-Val	SgPg vs SgFn Ratio	SgPg vs SgFn Sum	SgPg vs SgFn *q*-Val	SgPg vs SgFn *p*-Val	SgPgFn vs SgFn Ratio	SgPgFn vs SgFn Sum	SgPgFn vs SgFn *q*-Val	SgPgFn vs SgFn *p*-Val	SgPgFn vs SgPg Ratio	SgPgFn vs SgPg Sum	SgPgFn vs SgPg *q*-Val	SgPgFn vs SgPg *p*-Val
**SGO_1737** rpmB; ribosomal protein L28		5.867				5.867			2.134	7.515	0.0078	0.0454						5.836				6.447		
**SGO_1738** hypothetical protein SGO_1738	-0.253	4.817	0.0847	0.5362		4.320				4.320				2.525				1.914						
**SGO_1739** LytTr DNA-binding domain family	-1.358	4.332	0.0238	0.1138		4.076				4.076				1.196				0.585						
**SGO_1744** ABC transporter, ATP-binding protein SP0636	-0.225	4.863	0.0260	0.1304		4.350				4.350				2.611				2.000						
**SGO_1745** fba; fructose-1,6-bisphosphate aldolase, class II	0.223	11.665	0.0272	0.1384	1.138	12.225	0.0005	0.0006	1.424	12.436	0.0013	0.0044	0.915	11.782	0.0003	0.0002	1.201	11.373	0.0022	0.0053	0.286	12.325	0.0441	0.0943
**SGO_1747** hypothetical protein SGO_1747		3.401				2.553				2.981			-1.902	3.525	0.0129	0.0660	-0.419	3.082			1.483	3.270	0.0461	0.1009
**SGO_1748** pyrG; CTP synthase	-0.482	4.963	0.0203	0.0936	0.671	5.923	0.0190	0.0733	0.167	5.622	0.0423	0.3624	1.153	5.008	0.0403	0.2643	0.649	3.985	0.0510	0.3132	-0.504	5.487	0.0660	0.1637
**SGO_1749** manA; mannose-6-phosphate isomerase, class I	0.564	6.940	0.0184	0.0837	0.588	6.953	0.0186	0.0713	-0.386	6.448	0.0200	0.1552	0.024	6.688	0.1201	0.9143	-0.950	5.665	0.0052	0.0173	-0.974	6.290	0.0133	0.0144
**SGO_1750** ABC-type multidrug/protein/lipid transport system, ATPase component		5.518				5.518				5.518														
**SGO_1751** ABC-type multidrug/protein/lipid transport system, ATPase component		5.926			-2.262	6.233	0.0014	0.0025		5.926				3.338								3.338		
**SGO_1753** hypothetical protein SGO_1753		2.643				2.643				2.643														
**SGO_1755** scrK; fructokinase	-0.956	6.493	0.0018	0.0044	0.917	7.427	0.0037	0.0097	0.304	7.053	0.0095	0.0602	1.873	6.648	0.0011	0.0021	1.260	5.573	0.0006	0.0006	-0.613	7.025	0.0163	0.0196
**SGO_1756** hypothetical protein SGO_1756		2.170				2.170				2.170														
**SGO_1757** glmS; glucosamine--fructose-6-phosphate aminotransferase	-0.656	8.406	0.0149	0.0651	0.303	8.837	0.0214	0.0836	-0.347	8.518	0.0137	0.0974	0.959	8.074	0.0028	0.0085	0.309	7.076	0.0451	0.2675	-0.650	8.174	0.0050	0.0019
**SGO_1760** DNA-binding response regulator		3.231				2.098							-1.133	3.260				2.107				1.585		
**SGO_1761** histidine kinase		4.631				4.631				4.631														

Log$_2$ Ratios

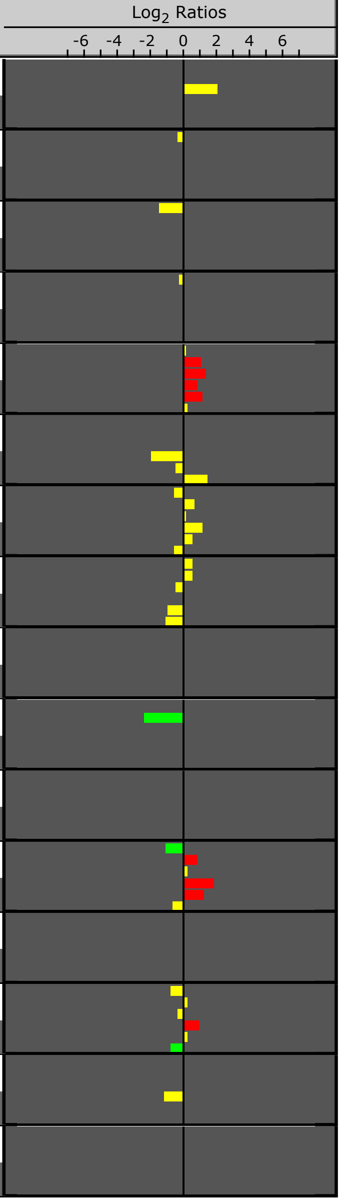

Show detected proteins only
Show all proteins
Filter by category:
ABC Transporter

Proteins found: 1179

Dot Plots
Dot Plots

Test	Cutoff
q-Value	.005
p-Value	

Color	Signif	Direction	Applies To
red	yes	+	ratios, bars
yellow	no	n/a	bars
green	yes	–	ratios, bars
light red	yes	+	*p*- , *q*-Values
light green	yes	–	*p*- , *q*-Values

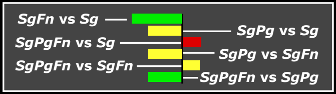

Hendrickson *et al.*

Streptococcus gordonii

Hackett Laboratory
UW

Summary Table | SgFn vs Sg | SgPg vs Sg | SgPgFn vs Sg | SgPg v SgFn | SgPgFn v SgFn | SgPgFn vs SgPg | Coverage

Protein	*SgFn vs Sg* Ratio	Sum	*q*-Val	*p*-Val	*SgPg vs Sg* Ratio	Sum	*q*-Val	*p*-Val	*SgPgFn vs Sg* Ratio	Sum	*q*-Val	*p*-Val	*SgPg vs SgFn* Ratio	Sum	*q*-Val	*p*-Val	*SgPgFn vs SgFn* Ratio	Sum	*q*-Val	*p*-Val	*SgPgFn vs SgPg* Ratio	Sum	*q*-Val	*p*-Val
**SGO_1763**	-0.583	7.656	0.0056	0.0188	0.005	7.920	0.1768	0.9410	0.099	7.963	0.0335	0.2787	0.588	7.148	0.0069	0.0284	0.682	6.589	0.0020	0.0048	0.094	7.454	0.1393	0.4415
	ABC transporter, substrate-binding protein SP0092																							
**SGO_1768**	3.248	6.256	0.0004	0.0004	0.808	4.468	0.0490	0.2172		2.946			-2.439	5.865	0.0010	0.0018		4.979				3.338		
	glycosyl hydrolase, family 38																							
**SGO_1772**		3.858				3.858				3.858														
	integral membrane protein																							
**SGO_1774**	1.620	6.691	0.0002	0.0002	0.032	5.695	0.1520	0.7951	0.393	5.898	0.0210	0.1646	-1.588	6.190	0.0005	0.0006	-1.227	5.684	0.0015	0.0028	0.361	5.406	0.0878	0.2411
	alcohol dehydrogenase, zinc-containing																							
**SGO_1780**	1.231	3.065				1.322				1.322				2.039				1.429						
	hypothetical protein SGO_1780																							
**SGO_1784**	0.353	8.245	0.0126	0.0527	0.586	8.378	0.0027	0.0063	0.812	8.516	0.0005	0.0011	0.233	8.016	0.0129	0.0663	0.459	7.530	0.0023	0.0057	0.226	8.242	0.0100	0.0089
	leuS; leucyl-tRNA synthetase																							
**SGO_1792**	0.124	2.648				1.585				1.585				1.196				0.585						
	transcription regulator																							
**SGO_1799**	2.086	9.301	0.0001	0.0000	1.049	8.530	0.0008	0.0012	0.779	8.359	0.0031	0.0142	-1.037	9.053	0.0001	0.0000	-1.307	8.362	0.0004	0.0002	-0.270	8.319	0.0358	0.0694
	endopeptidase O																							
**SGO_1800**		8.137			-2.160	8.427	0.0005	0.0007	-2.837	8.327	0.0003	0.0005		5.459				4.184			-0.676	6.165	0.0092	0.0071
	troB; manganese ABC transporter, ATP-binding protein SP1648																							
**SGO_1802**	-6.178	11.051	0.0003	0.0003	-2.784	11.227	0.0004	0.0004	-3.417	11.160	0.0003	0.0003	3.394	7.883	0.0013	0.0025	2.760	6.705	0.0014	0.0025	-0.634	8.464	0.0272	0.0462
	Metal ABC transporter substrate-binding lipoprotein precursor																							
**SGO_1803**	1.164	6.483	0.0010	0.0018	1.645	6.828	0.0001	0.0000	1.530	6.761	0.0021	0.0088	0.481	6.697	0.0022	0.0059	0.366	6.034	0.0255	0.1343	-0.115	6.871	0.1727	0.5975
	tpx; thioredoxin peroxidase																							
**SGO_1804**	1.496	4.317	0.0079	0.0293	2.632	5.330	0.0096	0.0315		1.585			1.136	5.246	0.0003	0.0002		2.958				4.705		
	hutI; imidazolonepropionase																							
**SGO_1805**	1.066	2.272			4.930	6.617	0.0277	0.1125	3.472	5.269	0.0288	0.2332	3.863	6.129	0.0204	0.1167	2.406	4.208	0.0435	0.2567	-1.458	6.549	0.0146	0.0165
	hutU; urocanate hydratase																							
**SGO_1806**						5.242				4.674				4.729				3.550			-0.584	5.473	0.0226	0.0335
	ftcD; glutamate formiminotransferase																							
**SGO_1807**						4.350								3.837								3.837		
	serine cycle enzyme, putative																							
**SGO_1808**		3.816				3.454				4.970			-1.367	4.133	0.0081	0.0358	0.110	4.382	0.1057	0.7490	1.477	4.890	0.0163	0.0196
	fhs-2; formate--tetrahydrofolate ligase																							

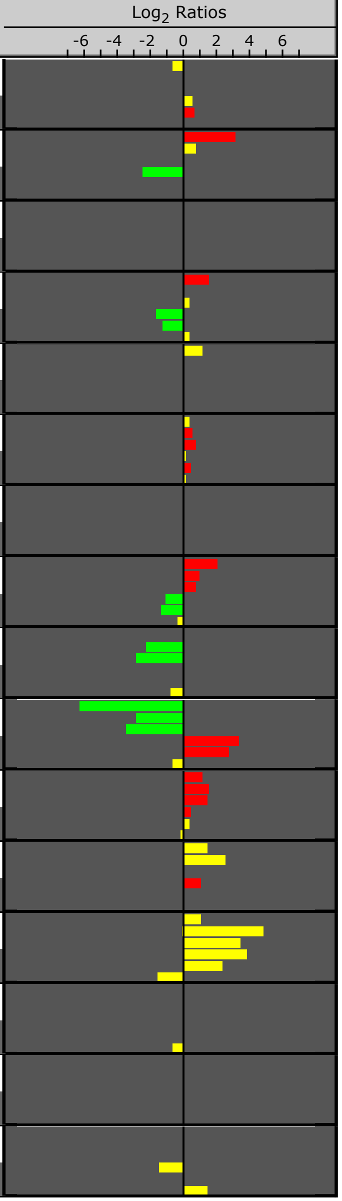

Show detected proteins only
Show all proteins
Filter by category:
ABC Transporter

Proteins found:
1179

Dot Plots
Dot Plots

Test	Cutoff
q-Value	.005
p-Value	

	Signif	Direction	Applies To
red	yes	+	ratios, bars
yellow	no	n/a	bars
green	yes	−	ratios, bars
light red	yes	+	*p*- , *q*-Values
light green	yes	−	

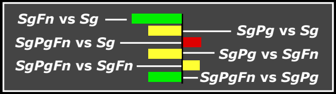

Hendrickson *et al.*

Protein	SgFn vs Sg Ratio	Sum	*q*-Val	*p*-Val	SgPg vs Sg Ratio	Sum	*q*-Val	*p*-Val	SgPgFn vs Sg Ratio	Sum	*q*-Val	*p*-Val	SgPg vs SgFn Ratio	Sum	*q*-Val	*p*-Val	SgPgFn vs SgFn Ratio	Sum	*q*-Val	*p*-Val	SgPgFn vs SgPg Ratio	Sum	*q*-Val	*p*-Val	Description
SGO_1811	1.763	4.707	0.0030	0.0087	1.223	5.013	0.0001	0.0000	0.311	3.971	0.0125	0.0864	-0.540	4.773	0.0042	0.0146	-1.453	3.363			-0.913	4.325	0.0131	0.0141	hutH; histidine ammonia-lyase
SGO_1813	0.124	3.063				2.000				2.000				1.611				1.000							hutG; formimidoylglutamase
SGO_1814	1.632	5.980	0.0028	0.0079		3.940				3.940				5.065				4.454							putative regulatory protein
SGO_1816	0.877	5.790	0.0044	0.0135	0.145	4.910	0.0440	0.1916		4.275			-0.731	5.031	0.0357	0.2282		4.044				2.907			scaR; ScaR Manganese-dependent regulator of scaCBA
SGO_1822	0.126	7.384	0.0609	0.3615	-1.768	6.687	0.0004	0.0004	-1.916	6.495	0.0097	0.0614	-1.893	6.278	0.0011	0.0022	-2.042	5.490	0.0212	0.1059	-0.149	4.569	0.0583	0.1398	relA; GTP diphosphokinase
SGO_1823						3.722								3.209								3.209			conserved hypothetical protein TIGR00046
SGO_1824	0.490	5.870	0.0226	0.1071	0.820	6.059	0.0028	0.0069	0.344	5.781	0.0208	0.1628	0.331	5.742	0.0167	0.0927	-0.145	4.892	0.0667	0.4389	-0.476	5.675	0.0116	0.0115	prmA; ribosomal protein L11 methyltransferase
SGO_1827	0.838	5.024	0.0259	0.1289	-0.256	4.992	0.0635	0.2934		4.111			-1.094	4.383	0.0183	0.1026		2.807				3.348			hypothetical protein SGO_1827
SGO_1828	0.994	5.002	0.0116	0.0472	0.056	4.482	0.1520	0.7952	0.528	4.191	0.0428	0.3679	-0.938	4.525	0.0147	0.0777	-0.466	3.724	0.0639	0.4192	0.472	3.741	0.1647	0.5585	ATPase, AAA family
SGO_1834	5.178	8.827	0.0008	0.0014	2.739	6.583	0.0043	0.0117	3.228	6.972	0.0001	0.0001	-2.439	8.524	0.0013	0.0027	-1.950	7.992	0.0015	0.0030	0.489	7.108	0.0493	0.1104	hypothetical protein SGO_1834
SGO_1835	2.787	4.567			0.863	3.800	0.0170	0.0631	2.701	4.492			-1.923	4.470	0.0032	0.0102	-0.086	4.206			1.837	4.414	0.0114	0.0111	hypothetical protein SGO_1835
SGO_1843	0.982	8.061	0.0002	0.0001	0.981	8.067	0.0026	0.0060	0.922	8.025	0.0010	0.0032	-0.001	7.961	0.1224	0.9344	-0.060	7.319	0.0775	0.5213	-0.059	7.934	0.1835	0.6466	pepS; aminopeptidase PepS
SGO_1844	0.367	5.531	0.0241	0.1161	0.775	5.772	0.0050	0.0141	-0.266	5.225	0.0432	0.3728	0.408	5.399	0.0074	0.0314	-0.633	4.302	0.0107	0.0454	-1.041	5.171	0.0100	0.0089	cbxX/cfqX family protein
SGO_1847	0.427	6.251	0.0392	0.2126	0.057	6.008	0.1039	0.5132	-0.806	5.342	0.0155	0.1128	-0.370	5.761	0.0377	0.2442	-1.233	4.620	0.0507	0.3100	-0.863	4.872	0.0339	0.0631	polC; DNA polymerase III, alpha subunit, Gram-positive type
SGO_1848		7.289			-3.095	7.448	0.0004	0.0005	-3.131	7.451	0.0003	0.0006		3.677				3.092			-0.036	4.690	0.2404	0.9247	lipoprotein, putative
SGO_1849		7.582				7.582				7.582															hypothetical protein SGO_1849

Log$_2$ Ratios

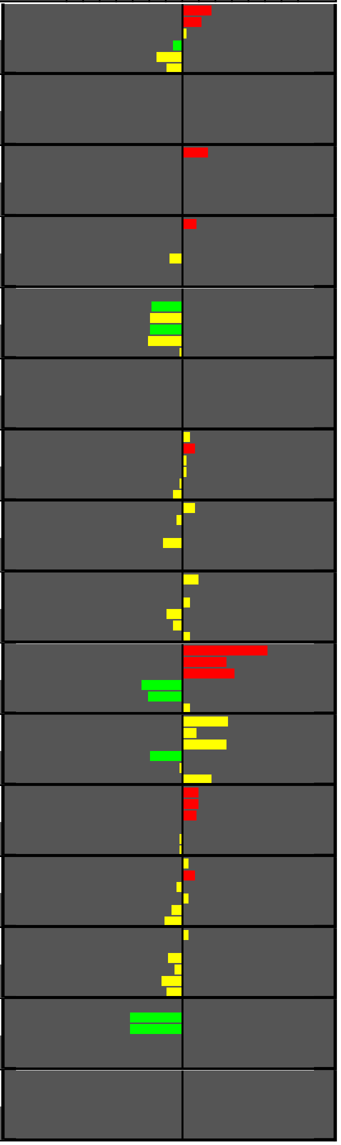

Show detected proteins only
Show all proteins
Filter by category:
ABC Transporter

Proteins found:
1179

Dot Plots
Dot Plots

Test	Cutoff
q-Value	.005
p-Value	

	Signif	Direction	Applies To
red	yes	+	ratios, bars
yellow	no	n/a	bars
green	yes	–	ratios, bars
light red	yes	+	*p*- , *q*-Values
light green	yes	–	

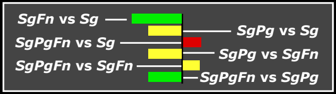

Hendrickson *et al.*

Streptococcus gordonii

Protein	*SgFn vs Sg* Ratio	Sum	*q*-Val	*p*-Val	*SgPg vs Sg* Ratio	Sum	*q*-Val	*p*-Val	*SgPgFn vs Sg* Ratio	Sum	*q*-Val	*p*-Val	*SgPg vs SgFn* Ratio	Sum	*q*-Val	*p*-Val	*SgPgFn vs SgFn* Ratio	Sum	*q*-Val	*p*-Val	*SgPgFn vs SgPg* Ratio	Sum	*q*-Val	*p*-Val	Description
SGO_1851	0.331	8.944	0.0137	0.0588	0.365	8.961	0.0066	0.0196	0.354	8.954	0.0037	0.0181	0.035	8.603	0.1049	0.7821	0.024	7.987	0.1208	0.8624	-0.011	8.612	0.2296	0.8672	proS; prolyl-tRNA synthetase
SGO_1852		6.376			-1.841	6.734	0.0003	0.0003	-1.808	6.568	0.0073	0.0423		4.036				2.442			0.033	4.627	0.2454	0.9485	membrane-associated zinc metalloprotease, putative
SGO_1854	-1.824	6.877	0.0003	0.0003	-1.307	7.017	0.0015	0.0028	-1.185	7.051	0.0011	0.0035	0.517	5.493	0.0244	0.1434	0.638	4.952	0.0188	0.0905	0.121	5.798	0.1989	0.7144	uppS; undecaprenyl diphosphate synthase
SGO_1855		7.640			-1.217	8.163	0.0022	0.0049		7.640				5.932								5.932			preprotein translocase, YajC subunit
SGO_1856	2.577	8.999	0.0055	0.0181	-0.896	6.760	0.0005	0.0007	-0.872	6.771	0.0009	0.0027	-3.472	8.392	0.0042	0.0144	-3.449	7.784	0.0046	0.0145	0.023	5.751	0.2247	0.8416	ATP-dependent proteinase ATP-binding chain
SGO_1857		4.723			-2.113	4.877	0.0396	0.1700		4.723				1.063								1.063			PTS system, IIABC components
SGO_1858	2.081	4.249	0.0188	0.0855	1.448	3.696	0.0223	0.0881		1.000			-0.633	4.293	0.0040	0.0137		2.965				2.941			beta-fructofuranosidase/sucrose 6 phoshate hydrolase
SGO_1860	-0.612	9.299	0.0202	0.0926	-1.332	9.034	0.0020	0.0044	-1.747	8.928	0.0009	0.0028	-0.721	8.138	0.0156	0.0833	-1.135	7.389	0.0101	0.0407	-0.414	7.507	0.0146	0.0165	5'-nucleotidase, lipoprotein e(P4) family
SGO_1861		1.865			2.171	5.189	0.0108	0.0360	0.305	3.026				4.524				1.046			-1.866	4.710	0.0246	0.0386	nusB; transcription antitermination factor NusB
SGO_1862	2.719	8.218	0.0003	0.0004	0.941	6.840	0.0011	0.0017	1.008	6.883	0.0004	0.0008	-1.778	7.869	0.0006	0.0006	-1.712	7.273	0.0007	0.0007	0.067	6.752	0.0956	0.2722	alkaline shock protein
SGO_1863	-0.622	8.044	0.0044	0.0134	0.394	8.526	0.0017	0.0034	0.360	8.507	0.0017	0.0065	1.017	7.780	0.0009	0.0014	0.982	7.147	0.0012	0.0019	-0.035	8.179	0.1094	0.3198	efp; Elongation factor P (EF-P)
SGO_1864	1.965	8.210	0.0001	0.0001	0.586	7.241	0.0040	0.0105	0.554	7.258	0.0189	0.1444	-1.379	7.834	0.0001	0.0000	-1.411	7.231	0.0014	0.0026	-0.032	7.000	0.2382	0.9116	X-Pro aminopeptidase
SGO_1865	-0.360	7.560	0.0050	0.0160	-1.073	7.288	0.0003	0.0003	-1.655	7.125	0.0002	0.0001	-0.713	6.543	0.0014	0.0032	-1.295	5.738	0.0008	0.0010	-0.582	5.878	0.0007	0.0000	uvrA; excinuclease ABC, A subunit
SGO_1866						2.384				3.718				1.871				2.594			1.334	3.687			corA; magnesium and cobalt transporter
SGO_1867	-3.307	7.508	0.0003	0.0003	-0.785	8.025	0.0012	0.0021	-0.450	8.165	0.0084	0.0500	2.522	6.302	0.0002	0.0001	2.857	5.997	0.0015	0.0030	0.335	7.252	0.0477	0.1060	hypothetical protein SGO_1867
SGO_1869		6.100				6.100				6.100															hypothetical protein SGO_1869

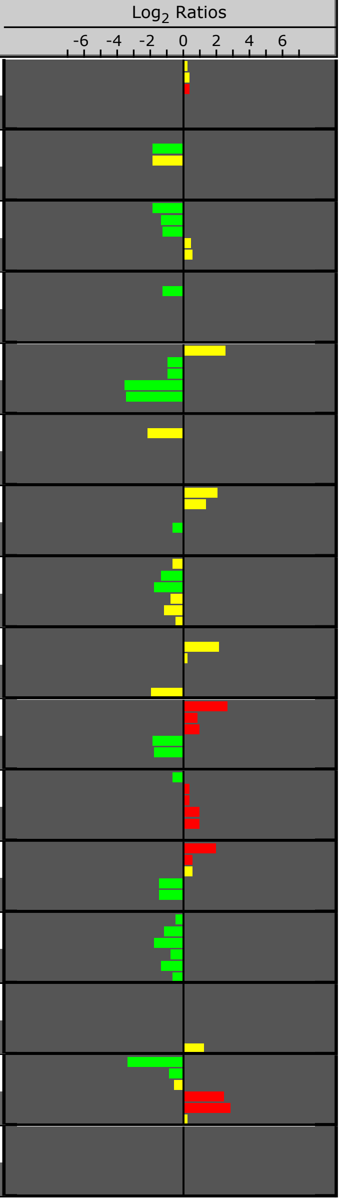

Show detected proteins only
Show all proteins
Filter by category:
ABC Transporter

Proteins found:
1179

Dot Plots
Dot Plots

Test	Cutoff
q-Value	.005
p-Value	

	Signif	Direction	Applies To
(red)	yes	+	ratios, bars
(yellow)	no	n/a	bars
(green)	yes	−	ratios, bars
(pink)	yes	+	*p*- , *q*-Values
(light green)	yes	−	

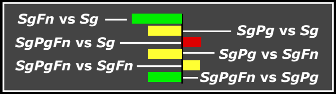

Hendrickson *et al.*

	SgFn vs Sg				*SgPg vs Sg*				*SgPgFn vs Sg*				*SgPg vs SgFn*				*SgPgFn vs SgFn*				*SgPgFn vs SgPg*				
Protein	Ratio	Sum	*q*-Val	*p*-Val	Ratio	Sum	*q*-Val	*p*-Val	Ratio	Sum	*q*-Val	*p*-Val	Ratio	Sum	*q*-Val	*p*-Val	Ratio	Sum	*q*-Val	*p*-Val	Ratio	Sum	*q*-Val	*p*-Val	Description
**SGO_1870**		6.281			-2.154	6.573	0.0017	0.0036		6.281				3.612								3.612			hypothetical protein SGO_1870
**SGO_1871**		5.486				5.486				5.486															lipoprotein, putative
**SGO_1872**		5.232			-2.042	5.394	0.0376	0.1612		5.232				1.648								1.648			hypothetical protein SGO_1872
**SGO_1878**	0.367	4.391	0.0261	0.1310		3.674				3.674				2.525				1.914							transcriptional regulator, TetR family domain protein
**SGO_1879**	0.354	7.776	0.0886	0.5656	0.485	7.843	0.0748	0.3573	0.700	7.966	0.0195	0.1505	0.132	7.433	0.0562	0.3870	0.347	6.937	0.0108	0.0460	0.215	7.603	0.0228	0.0348	rpsR; ribosomal protein S18
**SGO_1880**	-0.115	7.779	0.0501	0.2847	1.077	8.471	0.0008	0.0011	0.689	8.219	0.0009	0.0027	1.192	7.919	0.0004	0.0003	0.803	7.049	0.0001	0.0000	-0.388	8.214	0.0069	0.0045	ssb-1; single-strand binding protein
**SGO_1881**	-0.382	9.153	0.0271	0.1376	0.807	9.782	0.0045	0.0123	0.575	9.632	0.0010	0.0031	1.188	9.153	0.0034	0.0109	0.957	8.379	0.0031	0.0087	-0.231	9.505	0.0656	0.1618	rpsF; ribosomal protein S6
**SGO_1882**	1.171	5.749	0.0060	0.0204	1.566	6.032	0.0038	0.0099	0.473	5.296	0.0155	0.1125	0.395	5.908	0.0260	0.1553	-0.698	4.773	0.0085	0.0331	-1.092	5.641	0.0125	0.0131	folE; GTP cyclohydrolase I
**SGO_1884**		4.239				4.239				4.239															hypothetical protein SGO_1884
**SGO_1885**	3.284	12.092	0.0000	0.0000	0.544	9.965	0.0001	0.0000	0.330	9.842	0.0005	0.0013	-2.739	11.639	0.0000	0.0000	-2.954	11.002	0.0000	0.0000	-0.214	9.596	0.0050	0.0022	groL; 60 kDa chaperonin/groEL protein
**SGO_1886**	1.601	7.386	0.0137	0.0590	-0.062	6.280	0.1835	0.9903	-0.907	5.912	0.0036	0.0171	-1.663	6.874	0.0125	0.0626	-2.507	6.104	0.0097	0.0386	-0.844	5.401	0.0466	0.1028	groES; chaperonin, 10 kDa
**SGO_1887**		4.785				4.785				4.785															ABC transporter, ATP-binding protein
**SGO_1888**		3.936				3.936				3.936															ABC transporter, permease protein
**SGO_1890**		4.754				4.754				4.754															PTS system, fructose(mannose)-specific IID
**SGO_1891**		5.077				5.077				5.077															PTS system, IIC component
**SGO_1892**	2.240	8.409	0.0031	0.0089	-0.655	6.579	0.0076	0.0233	-0.914	6.496	0.0045	0.0231	-2.895	7.801	0.0024	0.0068	-3.155	7.166	0.0025	0.0065	-0.259	5.592	0.1219	0.3717	PTS system, fructose(mannose)-specific IIB

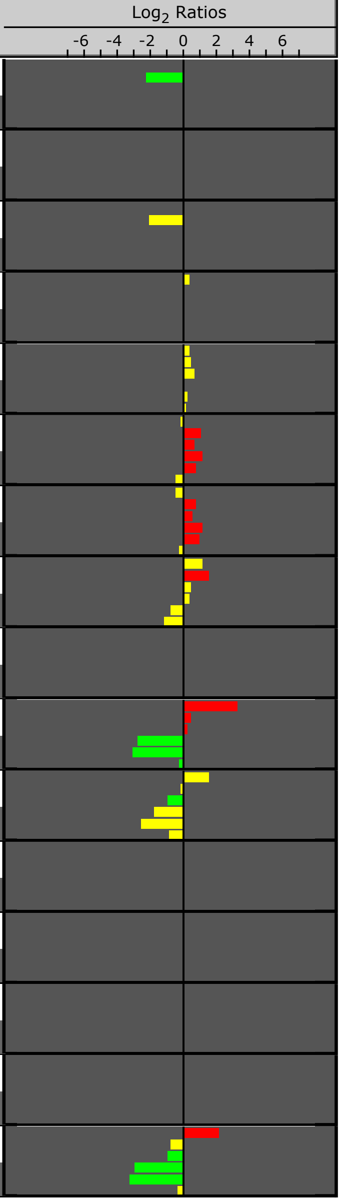

Show detected proteins only
Show all proteins
Filter by category:
ABC Transporter

Proteins found: 1179

Dot Plots
Dot Plots

Test	Cutoff
***q*-Value**	**.005**
p-Value	

	Signif	Direction	Applies To
red	yes	+	ratios, bars
yellow	no	n/a	bars
green	yes	–	ratios, bars
pink	yes	+	*p*- , *q*-Values
light green	yes	–	*p*- , *q*-Values

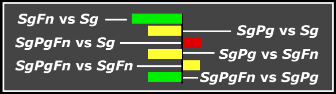

Hendrickson *et al.*

Streptococcus gordonii

Protein	SgFn vs Sg				SgPg vs Sg				SgPgFn vs Sg				SgPg vs SgFn				SgPgFn vs SgFn				SgPgFn vs SgPg			
	Ratio	Sum	*q*-Val	*p*-Val	Ratio	Sum	*q*-Val	*p*-Val	Ratio	Sum	*q*-Val	*p*-Val	Ratio	Sum	*q*-Val	*p*-Val	Ratio	Sum	*q*-Val	*p*-Val	Ratio	Sum	*q*-Val	*p*-Val
**SGO_1894** sugar-binding cell envelope protein		3.832				3.832				3.832														
**SGO_1895** response regulator		2.202				2.202				2.202														
**SGO_1897** rbsB; ribose ABC transporter (ribose-binding component) CAC1453		3.776				3.776				3.776														
**SGO_1898** glutamyl aminopeptidase	2.530	9.291	0.0004	0.0005	0.820	7.993	0.0003	0.0002	0.145	7.601	0.0155	0.1136	-1.710	8.931	0.0007	0.0009	-2.385	8.189	0.0006	0.0006	-0.675	7.534	0.0026	0.0006
**SGO_1899** hypothetical protein SGO_1899		2.700				2.700				2.700														
**SGO_1900** thioredoxin family protein		1.322			0.545	3.292	0.0127	0.0442		1.322				2.354								2.354		
**SGO_1901** tRNA binding domain	-0.053	6.001	0.1383	0.9288	1.151	6.682	0.0048	0.0136	0.799	6.435	0.0009	0.0026	1.204	6.178	0.0069	0.0283	0.852	5.322	0.0102	0.0424	-0.352	6.462	0.0503	0.1141
**SGO_1902** ssb; single-stranded DNA-binding protein		8.290			1.319	10.098	0.0015	0.0028	0.981	9.865	0.0016	0.0059		9.099				8.151			-0.339	9.940	0.0402	0.0826
**SGO_1903** ATP-dependent Zn protease	0.470	8.382	0.0109	0.0434	-0.014	8.113	0.1490	0.7754	0.042	8.146	0.0725	0.6714	-0.484	7.863	0.0087	0.0402	-0.428	7.280	0.0167	0.0781	0.056	7.626	0.1705	0.5878
**SGO_1907** sensor protein		2.170				2.170				2.170														
**SGO_1912** Bifunctional folate synthesis protein	2.439	4.026	0.0117	0.0484	1.890	4.365	0.0007	0.0009		2.189			-0.548	4.283	0.0072	0.0301		2.429				3.491		
**SGO_1914** folP; dihydropteroate synthase	1.677	4.472	0.0060	0.0203	0.948	3.872	0.0322	0.1338	1.000	3.170			-0.729	4.433	0.0011	0.0020	-0.677	3.531	0.0094	0.0372	0.052	3.628	0.2193	0.8139
**SGO_1916** ackA; acetate kinase	0.061	8.349	0.0764	0.4729	0.394	8.528	0.0030	0.0074	0.287	8.469	0.0046	0.0243	0.333	8.040	0.0002	0.0001	0.227	7.371	0.0018	0.0041	-0.106	8.144	0.0163	0.0194
**SGO_1917** hypothetical protein SGO_1917	0.787	5.009	0.0052	0.0167	0.982	5.136	0.0039	0.0104	1.025	5.153	0.0001	0.0001	0.195	4.942	0.0506	0.3436	0.237	4.345	0.0275	0.1467	0.042	5.049	0.2247	0.8395
**SGO_1923** comYB; competence protein		2.865				2.865				2.865														
**SGO_1924** comYA; competence protein comYA		8.254			-2.018	8.576	0.0002	0.0001	-2.489	8.495	0.0001	0.0001		5.740				4.667			-0.471	6.528	0.0494	0.1109

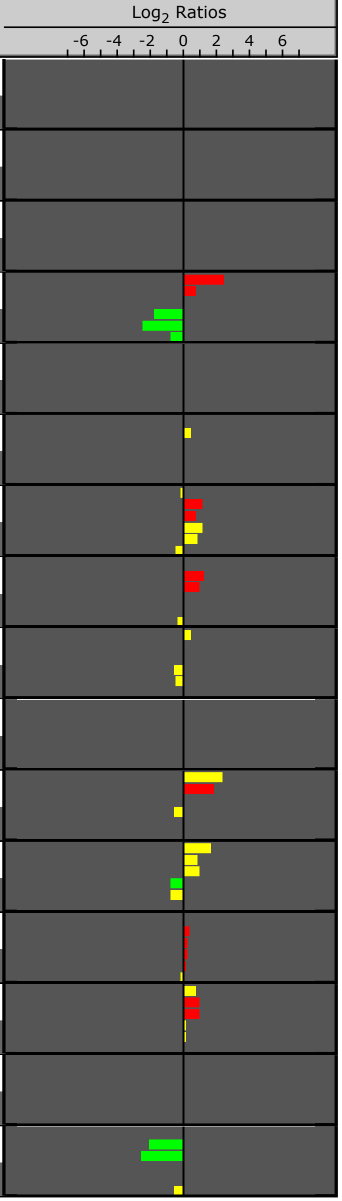

Show detected proteins only
Show all proteins
Filter by category:
ABC Transporter

Proteins found: 1179

Dot Plots
Dot Plots

Test	Cutoff
q-Value	.005
p-Value	

	Signif	Direction	Applies To
(red)	yes	+	ratios, bars
(yellow)	no	n/a	bars
(green)	yes	–	ratios, bars
(pink)	yes	+	*p*- , *q*-Values
(light green)	yes	–	

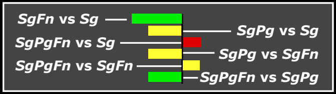

Spectral Counts
Sg Summary Table

Streptococcus gordonii

Hackett Laboratory UW

Summary Table | SgFn vs Sg | SgPg vs Sg | SgPgFn vs Sg | SgPg v SgFn | SgPgFn v SgFn | SgPgFn vs SgPg | Coverage

Protein	Description	*SgFn vs Sg* Ratio	Sum	*q*-Val	*p*-Val	*SgPg vs Sg* Ratio	Sum	*q*-Val	*p*-Val	*SgPgFn vs Sg* Ratio	Sum	*q*-Val	*p*-Val	*SgPg vs SgFn* Ratio	Sum	*q*-Val	*p*-Val	*SgPgFn vs SgFn* Ratio	Sum	*q*-Val	*p*-Val	*SgPgFn vs SgPg* Ratio	Sum	*q*-Val	*p*-Val
SGO_1926	rpoC; DNA-directed RNA polymerase, beta chain	-0.191	10.388	0.0027	0.0076	0.180	10.573	0.0033	0.0081	0.220	10.594	0.0018	0.0071	0.371	9.973	0.0006	0.0007	0.411	9.385	0.0008	0.0009	0.039	10.167	0.0956	0.2716
SGO_1927	rpoB; DNA-directed RNA polymerase, beta subunit	-0.581	10.200	0.0164	0.0731	-0.010	10.434	0.1496	0.7797	0.140	10.512	0.0173	0.1287	0.571	9.680	0.0141	0.0739	0.722	9.162	0.0101	0.0410	0.150	9.994	0.0423	0.0883
SGO_1928	pbp1b; penicillin-binding protein 1B		7.371			-2.965	7.544	0.0003	0.0003	-3.385	7.438	0.0087	0.0531		3.889				1.857			-0.421	4.347	0.0219	0.0317
SGO_1929	tyrS; tyrosyl-tRNA synthetase	-0.009	8.729	0.1251	0.8259	1.093	9.384	0.0010	0.0015	0.438	8.971	0.0031	0.0143	1.102	8.867	0.0008	0.0013	0.446	7.843	0.0035	0.0103	-0.655	9.026	0.0087	0.0065
SGO_1930	rrmA; Ribosomal RNA large subunit methyltransferase A		2.926			1.085	4.520	0.0051	0.0145		2.926				3.426								3.426		
SGO_1931	lipoprotein, putative						1.161								0.648								0.648		
SGO_1934	copper-translocating P-type ATPase		5.277				5.277			-1.691	5.485	0.0037	0.0184						1.461				2.072		
SGO_1935	negative transcriptional regulator, CopY		2.496				2.496				2.496														
SGO_1936	adcA; metal-binding (Mn) permease precursor, lipoprotein	-3.425	8.816	0.0000	0.0000	-2.189	8.974	0.0001	0.0000	-2.739	8.890	0.0001	0.0000	1.236	6.497	0.0004	0.0004	0.686	5.528	0.0033	0.0095	-0.550	6.741	0.0107	0.0101
SGO_1938	adcC; ATP-binding protein, Mn		5.090			0.029	6.096	0.1771	0.9448		5.090				4.589								4.589		
SGO_1939	adcR; repressor protein adcR						2.321								1.807								1.807		
SGO_1957	hypothetical protein SGO_1957	-0.388	5.582	0.0561	0.3268	-2.186	4.917	0.0534	0.2415		4.774			-1.798	4.035	0.0345	0.2178		3.236				1.000		
SGO_1958	rplQ; ribosomal protein L17	-1.095	8.424	0.0024	0.0066	2.075	10.247	0.0004	0.0003	1.594	9.873	0.0005	0.0012	3.171	9.577	0.0002	0.0002	2.689	8.543	0.0006	0.0004	-0.481	10.205	0.0116	0.0114
SGO_1959	rpoA; DNA-directed RNA polymerase, alpha subunit	0.029	9.157	0.1289	0.8527	0.482	9.402	0.0056	0.0164	0.337	9.318	0.0063	0.0352	0.453	8.899	0.0029	0.0089	0.308	8.205	0.0048	0.0153	-0.145	9.035	0.0322	0.0578
SGO_1960	rpsK; ribosomal protein S11	-0.204	7.854	0.0710	0.4360	0.545	8.234	0.0033	0.0080	0.585	8.257	0.0012	0.0041	0.749	7.652	0.0050	0.0192	0.789	7.066	0.0045	0.0141	0.040	7.986	0.1586	0.5270
SGO_1961	rpsM; ribosomal protein S13p/S18e	-1.173	8.386	0.0006	0.0010	1.499	9.790	0.0003	0.0003	0.380	9.056	0.0015	0.0056	2.673	9.052	0.0002	0.0001	1.553	7.536	0.0006	0.0004	-1.119	9.386	0.0026	0.0006

Log_2 Ratios: -6 -4 -2 0 2 4 6

Show detected proteins only
Show all proteins
Filter by category:
ABC Transporter

Proteins found: 1179

Dot Plots
Dot Plots

Test	Cutoff
q-Value	.005
p-Value	

	Signif	Direction	Applies To
red	yes	+	ratios, bars
yellow	no	n/a	bars
green	yes	–	ratios, bars
pink	yes	+	*p*- , *q*-Values
light green	yes	–	*p*- , *q*-Values

SgFn vs Sg — *SgPg vs Sg*
SgPgFn vs Sg — *SgPg vs SgFn*
SgPgFn vs SgFn — *SgPgFn vs SgPg*

Hendrickson *et al.*

Streptococcus gordonii

Protein	Description	*SgFn vs Sg* Ratio	Sum	*q*-Val	*p*-Val	*SgPg vs Sg* Ratio	Sum	*q*-Val	*p*-Val	*SgPgFn vs Sg* Ratio	Sum	*q*-Val	*p*-Val	*SgPg vs SgFn* Ratio	Sum	*q*-Val	*p*-Val	*SgPgFn vs SgFn* Ratio	Sum	*q*-Val	*p*-Val	*SgPgFn vs SgPg* Ratio	Sum	*q*-Val	*p*-Val	Log_2 Ratios
SGO_1962	rpmJ; ribosomal protein L36						5.444				5.204				4.930				4.080			0.805	5.815	0.0818	0.2172	
SGO_1964	adk; Adenylate kinase (ATP-AMP transphosphorylase)	-0.394	8.593	0.0246	0.1193	0.047	8.784	0.0231	0.0918	0.431	8.993	0.0013	0.0045	0.441	8.106	0.0161	0.0875	0.825	7.728	0.0045	0.0139	0.384	8.500	0.0076	0.0054	
SGO_1965	Preprotein translocase secY subunit		6.268			-1.375	6.740	0.0004	0.0005	-1.253	6.773	0.0003	0.0003		4.386				3.889			0.121	5.444	0.0928	0.2595	
SGO_1966	rplO; ribosomal protein L15	0.711	8.581	0.0250	0.1218	1.359	8.989	0.0068	0.0207	1.278	8.907	0.0009	0.0028	0.648	8.744	0.0282	0.1723	0.567	8.065	0.0299	0.1641	-0.081	8.956	0.1835	0.6466	
SGO_1967	50S ribosomal protein L30 -related protein	-0.238	7.392	0.0767	0.4756	1.872	8.702	0.0003	0.0002	0.975	8.055	0.0016	0.0058	2.111	8.152	0.0006	0.0007	1.214	6.873	0.0039	0.0118	-0.897	8.463	0.0054	0.0028	
SGO_1968	rpsE; ribosomal protein S5	-0.621	9.665	0.0078	0.0289	0.640	10.290	0.0024	0.0053	0.201	10.039	0.0195	0.1505	1.261	9.565	0.0006	0.0007	0.822	8.659	0.0017	0.0037	-0.439	9.855	0.0048	0.0017	
SGO_1969	rplR; ribosomal protein L18	-0.461	9.203	0.0251	0.1230	1.259	10.158	0.0003	0.0003	1.285	10.176	0.0002	0.0003	1.721	9.530	0.0005	0.0005	1.746	8.939	0.0006	0.0005	0.025	10.151	0.0183	0.0240	
SGO_1970	BL10; 50S ribosomal protein L6	-1.812	9.837	0.0001	0.0001	-0.442	10.269	0.0004	0.0004	-0.307	10.335	0.0120	0.0805	1.370	8.992	0.0003	0.0002	1.504	8.491	0.0022	0.0051	0.135	9.595	0.1111	0.3304	
SGO_1971	rpsH; ribosomal protein S8	-0.883	9.958	0.0005	0.0007	0.875	10.836	0.0004	0.0005	1.268	11.111	0.0015	0.0056	1.758	10.068	0.0002	0.0001	2.151	9.780	0.0014	0.0024	0.393	10.912	0.0327	0.0590	
SGO_1972	rpsN; ribosomal protein S14p/S29e		5.340				5.340				5.340															
SGO_1973	BL6; 50S ribosomal protein L5	-0.823	9.445	0.0007	0.0011	0.314	9.963	0.0026	0.0060	0.582	10.121	0.0019	0.0079	1.138	9.139	0.0002	0.0001	1.406	8.721	0.0009	0.0011	0.268	9.741	0.0248	0.0399	
SGO_1974	rplX; ribosomal protein L24	-1.058	8.620	0.0046	0.0142	-0.172	8.962	0.0595	0.2735	0.659	9.413	0.0039	0.0194	0.886	7.983	0.0021	0.0053	1.717	7.964	0.0011	0.0016	0.831	8.830	0.0071	0.0046	
SGO_1975	rplN; ribosomal protein L14	-0.135	8.279	0.0814	0.5114	0.740	8.750	0.0003	0.0003	1.293	9.123	0.0005	0.0012	0.875	8.196	0.0021	0.0051	1.428	7.967	0.0013	0.0021	0.553	8.865	0.0092	0.0073	
SGO_1976	BS16; 30S ribosomal protein	0.116	7.182	0.1315	0.8772	2.575	8.892	0.0008	0.0012	1.712	8.214	0.0030	0.0137	2.459	8.387	0.0007	0.0009	1.596	7.102	0.0038	0.0111	-0.862	8.785	0.0140	0.0153	
SGO_1977	rpmC; ribosomal protein L29	-0.463	5.951	0.0523	0.2993	0.014	6.513	0.1779	0.9553	0.375	6.717	0.0222	0.1763	0.476	5.445	0.0390	0.2551	0.837	5.125	0.0457	0.2725	0.361	6.208	0.0684	0.1734	
SGO_1978	rplP; ribosomal protein L16	-0.239	7.841	0.0607	0.3602	1.442	8.829	0.0015	0.0028	0.912	8.468	0.0040	0.0201	1.680	8.238	0.0001	0.0000	1.151	7.242	0.0002	0.0001	-0.529	8.605	0.0007	0.0000	

Log_2 Ratios axis: -6 -4 -2 0 2 4 6

Show detected proteins only
Show all proteins
Filter by category:
ABC Transporter

Proteins found:
1179

Dot Plots
Dot Plots

Test	Cutoff
q-Value	.005
p-Value	

	Signif	Direction	Applies To
red	yes	+	ratios, bars
yellow	no	n/a	bars
green	yes	–	ratios, bars
pink	yes	+	*p*- , *q*-Values
light green	yes	–	

SgFn vs Sg
SgPgFn vs Sg
SgPgFn vs SgFn
SgPg vs Sg
SgPg vs SgFn
SgPgFn vs SgPg

Hendrickson *et al.*

Streptococcus gordonii

Protein	Description	SgFn vs Sg Ratio	SgFn vs Sg Sum	SgFn vs Sg *q*-Val	SgFn vs Sg *p*-Val	SgPg vs Sg Ratio	SgPg vs Sg Sum	SgPg vs Sg *q*-Val	SgPg vs Sg *p*-Val	SgPgFn vs Sg Ratio	SgPgFn vs Sg Sum	SgPgFn vs Sg *q*-Val	SgPgFn vs Sg *p*-Val	SgPg vs SgFn Ratio	SgPg vs SgFn Sum	SgPg vs SgFn *q*-Val	SgPg vs SgFn *p*-Val	SgPgFn vs SgFn Ratio	SgPgFn vs SgFn Sum	SgPgFn vs SgFn *q*-Val	SgPgFn vs SgFn *p*-Val	SgPgFn vs SgPg Ratio	SgPgFn vs SgPg Sum	SgPgFn vs SgPg *q*-Val	SgPgFn vs SgPg *p*-Val
SGO_1979	rpsC; ribosomal protein S3	-1.001	10.336	0.0006	0.0010	-0.999	10.334	0.0003	0.0002	-0.865	10.382	0.0004	0.0010	0.002	9.240	0.1243	0.9528	0.136	8.699	0.0521	0.3237	0.134	9.307	0.0661	0.1645
SGO_1980	rplV; ribosomal protein L22	-0.272	8.907	0.0105	0.0417	0.869	9.533	0.0003	0.0003	1.192	9.751	0.0003	0.0005	1.141	8.930	0.0002	0.0002	1.464	8.550	0.0005	0.0003	0.323	9.561	0.0094	0.0076
SGO_1981	rpsS; ribosomal protein S19	-0.372	9.339	0.0070	0.0251	-0.198	9.418	0.0320	0.1328	-0.058	9.485	0.0642	0.5800	0.174	8.715	0.0107	0.0510	0.314	8.180	0.0049	0.0163	0.140	8.873	0.0680	0.1722
SGO_1982	rplB; ribosomal protein L2	0.174	9.007	0.0740	0.4556	0.327	9.069	0.0196	0.0760	0.562	9.205	0.0042	0.0214	0.153	8.650	0.0843	0.6166	0.388	8.167	0.0310	0.1712	0.235	8.829	0.0419	0.0871
SGO_1983	rplW; ribosomal protein L23	-1.445	8.527	0.0028	0.0079	-0.214	8.948	0.0580	0.2650	-0.221	8.939	0.0161	0.1179	1.231	7.880	0.0079	0.0346	1.224	7.256	0.0069	0.0248	-0.007	8.326	0.2399	0.9214
SGO_1984	rplD; ribosomal protein L4/L1 family	0.348	9.327	0.0089	0.0341	0.821	9.611	0.0017	0.0035	1.443	10.038	0.0004	0.0009	0.473	9.229	0.0014	0.0030	1.096	9.013	0.0008	0.0008	0.622	9.793	0.0066	0.0041
SGO_1985	rplC; ribosomal protein L3	1.375	9.340	0.0021	0.0053	2.375	10.178	0.0072	0.0217	2.068	9.860	0.0001	0.0000	0.999	9.986	0.0136	0.0706	0.693	9.127	0.0024	0.0061	-0.307	10.243	0.1025	0.2963
SGO_1986	rpsJ; ribosomal protein S10	-0.327	7.486	0.0238	0.1144	0.450	7.881	0.0131	0.0458	0.996	8.217	0.0008	0.0022	0.777	7.231	0.0011	0.0020	1.323	6.986	0.0002	0.0001	0.546	7.865	0.0050	0.0018
SGO_1988	hydrolase, haloacid dehalogenase-like family	1.168	5.598	0.0404	0.2208		4.572				4.572				4.110				3.499						
SGO_1989	purA; adenylosuccinate synthetase	0.601	9.267	0.0015	0.0034	1.403	9.800	0.0003	0.0002	1.422	9.815	0.0003	0.0004	0.802	9.478	0.0007	0.0008	0.821	8.880	0.0010	0.0013	0.019	9.834	0.2083	0.7590
SGO_1990	glutamate--cysteine ligase, putative/amino acid ligase, putative	3.661	6.835	0.0005	0.0008	1.608	5.146	0.0122	0.0419	1.153	4.157	0.0196	0.1515	-2.053	6.535	0.0013	0.0027	-2.509	5.718	0.0181	0.0858	-0.456	4.657	0.1579	0.5196
SGO_1991	hslO; 33 kDa chaperonin /Heat shock protein 33-like protein	2.067	6.131	0.0009	0.0016	-0.388	4.635	0.0674	0.3156		3.874			-2.454	5.522	0.0001	0.0000		4.668				2.835		
SGO_1993	possible transcriptional regulator	-1.499	6.875	0.0024	0.0063	-0.453	7.226	0.0175	0.0657	-0.658	7.153	0.0087	0.0534	1.046	6.030	0.0009	0.0014	0.841	5.299	0.0076	0.0285	-0.205	6.372	0.1151	0.3438
SGO_1994	lipoprotein, putative		1.379				1.380				1.380														
SGO_1995	MutT/nudix family protein	0.615	5.975	0.0425	0.2374		4.654			-0.155	5.158	0.0782	0.7315		4.724			-0.770	4.468	0.0601	0.3863		2.883		
SGO_1998	clpB; ATP-dependent Clp proteinase, ATP-binding chain	-0.114	7.258	0.0299	0.1545	-0.061	7.292	0.1442	0.7455	-0.905	6.939	0.0016	0.0059	0.053	6.720	0.0848	0.6222	-0.791	5.739	0.0021	0.0049	-0.844	6.394	0.0172	0.0218

Log$_2$ Ratios

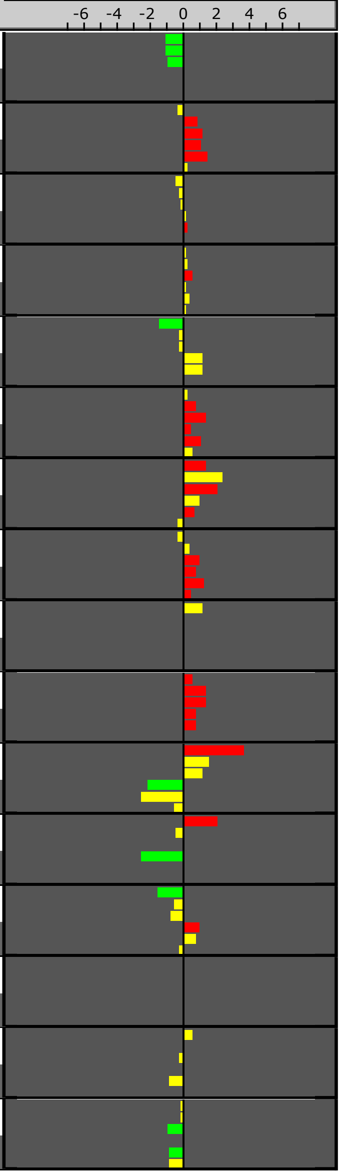

Show detected proteins only
Show all proteins
Filter by category:
ABC Transporter

Proteins found: 1179

Dot Plots
Dot Plots

Test	Cutoff
q-Value	.005
p-Value	

Color	Signif	Direction	Applies To
red	yes	+	ratios, bars
yellow	no	n/a	bars
green	yes	–	ratios, bars
light red	yes	+	*p*- , *q*-Values
light green	yes	–	

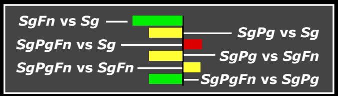

Hendrickson *et al.*

Spectral Counts
Sg Summary Table

Streptococcus gordonii

Hackett Laboratory UW

Summary Table | SgFn vs Sg | SgPg vs Sg | SgPgFn vs Sg | SgPg v SgFn | SgPgFn v SgFn | SgPgFn vs SgPg | Coverage

Protein	*SgFn vs Sg*				*SgPg vs Sg*				*SgPgFn vs Sg*				*SgPg vs SgFn*				*SgPgFn vs SgFn*				*SgPgFn vs SgPg*				Description
	Ratio	Sum	*q*-Val	*p*-Val	Ratio	Sum	*q*-Val	*p*-Val	Ratio	Sum	*q*-Val	*p*-Val	Ratio	Sum	*q*-Val	*p*-Val	Ratio	Sum	*q*-Val	*p*-Val	Ratio	Sum	*q*-Val	*p*-Val	
**SGO_2000**	0.216	10.871	0.0174	0.0778	0.557	11.062	0.0011	0.0018	0.572	11.072	0.0014	0.0052	0.341	10.640	0.0032	0.0103	0.356	10.040	0.0060	0.0208	0.015	10.808	0.2122	0.7760	tsf; translation elongation factor Ts
**SGO_2001**	-0.063	10.783	0.0858	0.5436	0.163	10.895	0.0136	0.0480	0.252	10.944	0.0063	0.0353	0.226	10.354	0.0122	0.0605	0.316	9.794	0.0097	0.0386	0.089	10.507	0.0813	0.2151	rpsB; ribosomal protein S2
**SGO_2004**	-2.079	7.088	0.0011	0.0023		6.780				6.780				4.196				3.585							LPXTG cell wall surface protein
**SGO_2005**	-5.995	9.561	0.0089	0.0340	-4.964	9.595	0.0002	0.0001	-4.242	9.625	0.0001	0.0001	1.031	4.389	0.0158	0.0855	1.753	4.401	0.0290	0.1574	0.722	5.492	0.0219	0.0321	LPXTG cell wall surface protein
**SGO_2006**		3.279				3.279				3.279															thioredoxin signature protein
**SGO_2007**	0.413	7.410	0.0001	0.0001	-0.015	7.182	0.1504	0.7846	0.048	7.214	0.0366	0.3079	-0.429	6.891	0.0009	0.0016	-0.365	6.308	0.0012	0.0019	0.063	6.694	0.1174	0.3566	nusG; transcription termination/antitermination factor NusG
**SGO_2008**		5.872				5.872				5.872															preprotein translocase secE component -related protein
**SGO_2010**	-1.834	6.616	0.0002	0.0002		6.259				6.259				3.916				3.305							pbp2a; penicillin-binding protein 2A
**SGO_2013**	-5.238	9.019	0.0000	0.0000		8.980				8.980				3.259				2.648							putative N-acetylmuramidase/lysin
**SGO_2015**		0.585				0.585				0.585															possible polysaccharide transport protein
**SGO_2016**		2.170				2.170				2.170															nucleotide sugar dehydratase, putative
**SGO_2017**		2.700			-0.539	3.456				2.700				1.648								1.648			nucleotidyl transferase, putative
**SGO_2019**		5.006			-0.881	5.633	0.0003	0.0002		5.006				3.614								3.614			licD; licD3 protein
**SGO_2020**		2.585				2.585				2.585															glycosyltransferase
**SGO_2021**		5.271				5.271				5.271															Extracellular polysaccharide glycosyltransferase
**SGO_2022**		2.000				2.000				2.000															UDP-glucose 4-epimerase BH3715

Log$_2$ Ratios

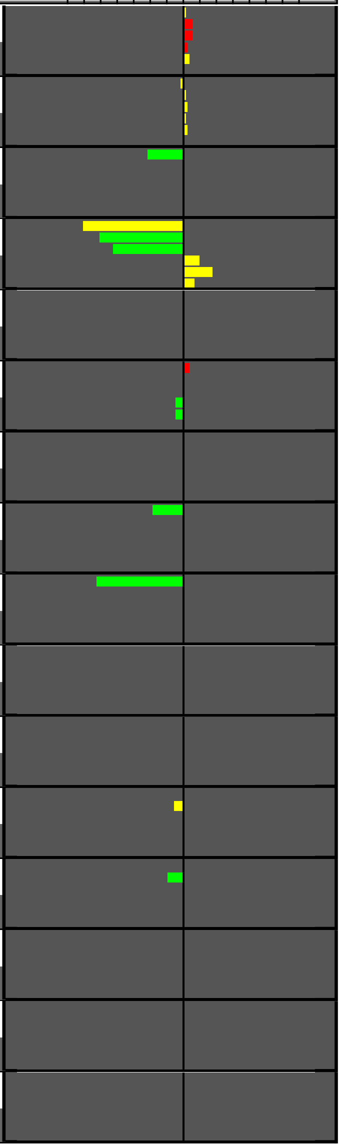

- Show detected proteins only
- Show all proteins
- Filter by category:

ABC Transporter

Proteins found: 1179

Dot Plots

Dot Plots

Test	Cutoff
q-Value	.005
p-Value	

	Signif	Direction	Applies To
red	yes	+	ratios, bars
yellow	no	n/a	bars
green	yes	–	ratios, bars
pink	yes	+	*p*- , *q*-Values
light green	yes	–	

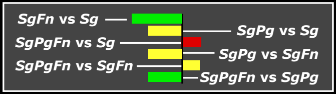

Hendrickson *et al.*

Streptococcus gordonii

	SgFn vs Sg				*SgPg vs Sg*				*SgPgFn vs Sg*				*SgPg vs SgFn*				*SgPgFn vs SgFn*				*SgPgFn vs SgPg*				
Protein	Ratio	Sum	*q*-Val	*p*-Val	Ratio	Sum	*q*-Val	*p*-Val	Ratio	Sum	*q*-Val	*p*-Val	Ratio	Sum	*q*-Val	*p*-Val	Ratio	Sum	*q*-Val	*p*-Val	Ratio	Sum	*q*-Val	*p*-Val	Description
**SGO_2023**		5.835				5.835				5.835															galactosyltransferase
**SGO_2024**		6.956			-1.523	7.385	0.0014	0.0026	-1.812	7.317	0.0008	0.0021		4.912				4.018			-0.289	5.777	0.0339	0.0630	Extracellular polysaccharide biosynthesis
**SGO_2025**		6.005			-0.304	6.869	0.0257	0.1036	-1.636	6.420	0.0005	0.0011		5.206				3.296			-1.332	5.698	0.0123	0.0128	wze; putative autophosphorylating protein tyrosine kinase
**SGO_2026**		6.124			-0.991	6.710	0.0019	0.0042		6.124				4.614								4.614			wzd; polysaccharide export protein, MPA1 family
**SGO_2027**		2.170				2.170				2.170															wzh; wzh/Putative phosphotyrosine-protein phosphatase
**SGO_2028**		6.039			-2.941	6.130	0.0049	0.0138		6.039				1.585								1.585			wzg; transcriptional regulator
**SGO_2033**	0.426	8.356	0.0017	0.0041	-0.854	7.763	0.0003	0.0002	-1.279	7.627	0.0002	0.0003	-1.280	7.538	0.0003	0.0003	-1.704	6.817	0.0004	0.0002	-0.425	6.565	0.0099	0.0085	nrdD; ribonucleoside-triphosphate reductase
**SGO_2034**	-1.249	5.669	0.0365	0.1951	-1.989	5.567	0.0362	0.1540		5.398			-0.740	3.288				2.000				1.871			hypothetical protein SGO_2034
**SGO_2037**	-0.611	6.209	0.0059	0.0199		5.472				5.472				4.374				3.763							cardiolipin synthase
**SGO_2039**		3.546			-1.419	3.789	0.0442	0.1931		3.546				0.585								0.585			dihydrofolate synthase
**SGO_2041**		3.189			1.335	4.956	0.0032	0.0078	0.282	3.847	0.0764	0.7107		3.941				1.272			-1.052	4.251	0.0482	0.1073	conserved hypothetical protein TIGR00250
**SGO_2042**	0.631	7.574	0.0260	0.1299	2.054	8.549	0.0006	0.0008	2.436	8.867	0.0006	0.0013	1.423	8.197	0.0021	0.0052	1.805	7.874	0.0017	0.0037	0.382	8.928	0.0313	0.0550	Bacterial protein of unknown function (DUF965) superfamily
**SGO_2043**		5.470			-2.954	5.560	0.0127	0.0442		5.470				1.000								1.000			lipoprotein, putative
**SGO_2045**	-2.332	9.653	0.0005	0.0006	0.828	10.862	0.0006	0.0008	0.911	10.917	0.0007	0.0018	3.160	9.855	0.0000	0.0000	3.243	9.321	0.0003	0.0001	0.083	10.745	0.0818	0.2174	recA; recA protein
**SGO_2046**	0.441	5.691	0.0257	0.1271	0.321	5.625	0.0568	0.2580	-0.649	5.179	0.0131	0.0925	-0.120	5.303	0.0188	0.1070	-1.090	4.310	0.0010	0.0012	-0.970	4.841	0.0050	0.0022	cinA; competence induced protein
**SGO_2049**		1.000				1.000				1.000															tagI; DNA-3-methyladenine glycosylase I

Log2 Ratios

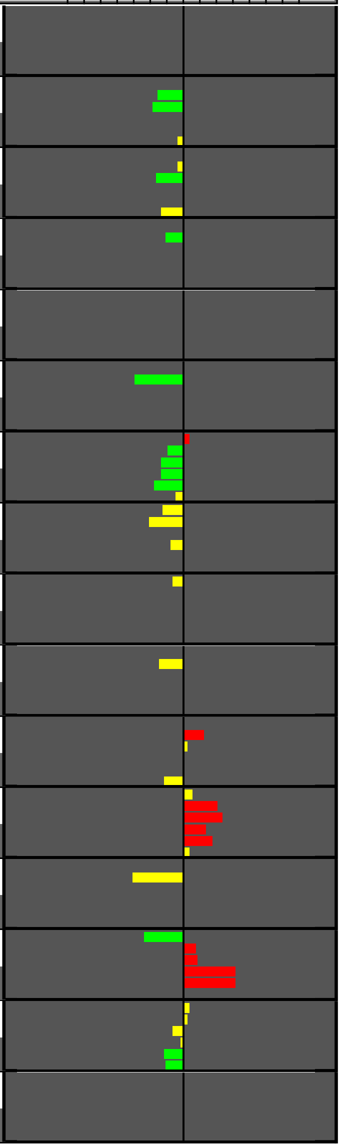

Show detected proteins only
Show all proteins
Filter by category:
ABC Transporter

Proteins found: 1179

Dot Plots
Dot Plots

Test	Cutoff
q-Value	.005
p-Value	

	Signif	Direction	Applies To
red	yes	+	ratios, bars
yellow	no	n/a	bars
green	yes	−	ratios, bars
light red	yes	+	*p*- , *q*-Values
light green	yes	−	

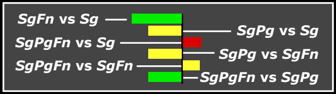

Streptococcus gordonii

Hackett Laboratory UW

Protein	SgFn vs Sg Ratio	Sum	q-Val	p-Val	SgPg vs Sg Ratio	Sum	q-Val	p-Val	SgPgFn vs Sg Ratio	Sum	q-Val	p-Val	SgPg vs SgFn Ratio	Sum	q-Val	p-Val	SgPgFn vs SgFn Ratio	Sum	q-Val	p-Val	SgPgFn vs SgPg Ratio	Sum	q-Val	p-Val	Description
SGO_2050	0.126	3.423	0.1429	0.9676	1.259	4.533	0.0163	0.0602		2.849			1.133	3.770	0.0411	0.2701		0.692				3.483			ruvA; Holliday junction DNA helicase RuvA
SGO_2052		3.000				3.000				3.000															hypothetical protein SGO_2052
SGO_2053	-0.307	6.428	0.0257	0.1272	-1.164	6.120	0.0053	0.0152	-1.264	6.077	0.0018	0.0070	-0.856	5.395	0.0050	0.0190	-0.957	4.735	0.0014	0.0024	-0.100	4.861	0.1661	0.5663	DNA mismatch repair protein hexB
SGO_2056	0.988	7.171	0.0049	0.0156	-0.084	6.562	0.1403	0.7223	-0.777	6.303	0.0176	0.1321	-1.072	6.628	0.0033	0.0109	-1.765	5.849	0.0027	0.0072	-0.693	5.734	0.0656	0.1610	mutS; DNA mismatch repair protein MutS
SGO_2057		5.049				5.049				5.049															argR; arginine repressor
SGO_2058	0.776	10.075	0.0002	0.0001	0.451	9.878	0.0008	0.0011	0.830	10.111	0.0006	0.0014	-0.325	9.745	0.0011	0.0020	0.054	9.316	0.0612	0.3967	0.379	9.776	0.0103	0.0095	argS; arginyl-tRNA synthetase
SGO_2059		2.058				2.058				2.058															hypothetical protein SGO_2059
SGO_2060	0.598	7.874	0.0093	0.0357	0.996	8.121	0.0014	0.0027	-0.098	7.496	0.0584	0.5233	0.399	7.832	0.0038	0.0130	-0.696	6.704	0.0031	0.0087	-1.094	7.569	0.0012	0.0001	aspS-1; aspartyl-tRNA synthetase
SGO_2062	1.266	8.848	0.0012	0.0025	0.686	8.464	0.0046	0.0126	0.880	8.587	0.0018	0.0073	-0.579	8.567	0.0028	0.0084	-0.386	8.038	0.0077	0.0286	0.193	8.348	0.0506	0.1152	hisS; histidyl-tRNA synthetase
SGO_2064	1.217	8.670	0.0002	0.0002	1.647	8.986	0.0005	0.0006	1.405	8.804	0.0002	0.0003	0.430	8.872	0.0029	0.0089	0.188	8.126	0.0073	0.0270	-0.242	8.955	0.0271	0.0454	ilvD; dihydroxy-acid dehydratase
SGO_2065																									rpmF; ribosomal protein L32
SGO_2066	-0.335	6.614	0.0814	0.5094	1.393	7.977	0.0019	0.0041	1.056	7.749	0.0037	0.0182	1.727	7.190	0.0158	0.0853	1.390	6.300	0.0278	0.1487	-0.337	7.834	0.0466	0.1024	rpmG; ribosomal protein L33
SGO_2070	-0.754	6.302	0.0416	0.2298	-0.247	6.477	0.0921	0.4498	-0.962	6.219	0.0204	0.1592	0.507	5.531	0.0345	0.2179	-0.208	4.559	0.0776	0.5231	-0.715	5.430	0.0332	0.0611	hypothetical protein SGO_2070
SGO_2073										1.981								0.857				1.468			FtsK/SpoIIIE family subfamily, putative
SGO_2080		5.162			-0.970	5.772	0.0103	0.0343		5.162				3.723								3.723			transcription activator plcR
SGO_2081		6.027			-2.513	6.148	0.0099	0.0328		6.027				2.000								2.000			lipoprotein, putative

Log$_2$ Ratios (-6 -4 -2 0 2 4 6)

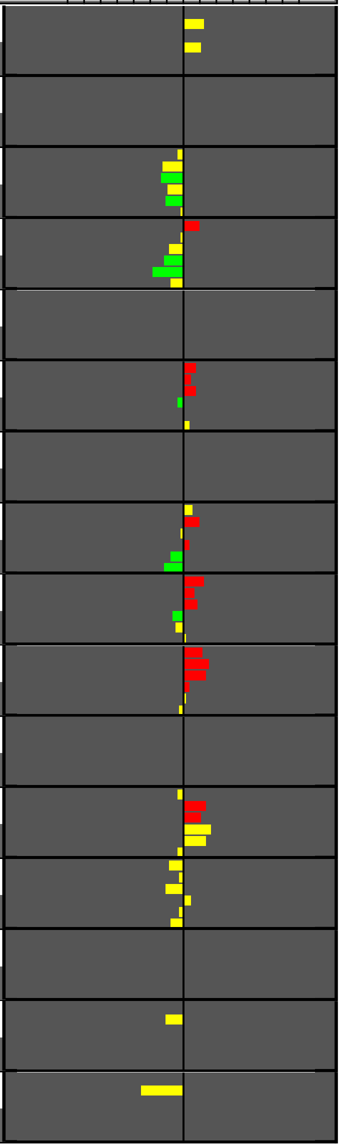

- Show detected proteins only
- Show all proteins
- Filter by category: ABC Transporter

Proteins found: 1179

Dot Plots
Dot Plots

Test	Cutoff
q-Value	.005
p-Value	

	Signif	Direction	Applies To
red	yes	+	ratios, bars
yellow	no	n/a	bars
green	yes	–	ratios, bars
light red	yes	+	p- , q-Values
light green	yes	–	

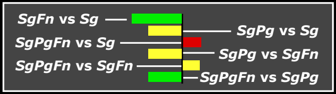

Summary Table | SgFn vs Sg | SgPg vs Sg | SgPgFn vs Sg | SgPg v SgFn | SgPgFn v SgFn | SgPgFn vs SgPg | Coverage

Protein	Description	*SgFn vs Sg*				*SgPg vs Sg*				*SgPgFn vs Sg*				*SgPg vs SgFn*				*SgPgFn vs SgFn*				*SgPgFn vs SgPg*			
		Ratio	Sum	*q*-Val	*p*-Val	Ratio	Sum	*q*-Val	*p*-Val	Ratio	Sum	*q*-Val	*p*-Val	Ratio	Sum	*q*-Val	*p*-Val	Ratio	Sum	*q*-Val	*p*-Val	Ratio	Sum	*q*-Val	*p*-Val
**SGO_2082**	lipoprotein, putative	-2.782	5.615	0.0263	0.1323		5.515				5.515				1.196				0.585						
**SGO_2084**	NAD(P)H dehydrogenase, quinone family	0.089	5.219	0.0952	0.6113	-0.180	4.686	0.0926	0.4531		4.161			-0.269	4.253	0.0745	0.5363		3.151				2.459		
**SGO_2085**	purB; adenylosuccinate lyase	-0.782	8.011	0.0004	0.0004	-0.211	8.247	0.0036	0.0090	-0.164	8.270	0.0050	0.0265	0.571	7.367	0.0003	0.0003	0.619	6.785	0.0006	0.0006	0.047	7.648	0.0778	0.2038
**SGO_2086**	hypothetical protein SGO_2086		4.929				4.929				4.929														
**SGO_2094**	general stress protein GSP-781		5.483				5.483				5.483														
**SGO_2096**	comB; transport protein ComB		8.836				8.836				8.836														
**SGO_2097**	comA; ATP-binding Transport protein ComA		9.971			-5.519	10.002	0.0001	0.0000	-6.574	9.979	0.0032	0.0151		3.939				1.272			-1.055	4.250	0.0195	0.0268
**SGO_2098**	rpsD; ribosomal protein S4	0.507	11.004	0.0078	0.0287	0.885	11.230	0.0001	0.0001	0.782	11.174	0.0034	0.0159	0.377	10.921	0.0072	0.0303	0.275	10.262	0.0340	0.1914	-0.102	11.049	0.1579	0.5181
**SGO_2099**	ABC transporter ATP-binding protein						3.098								2.585								2.585		
**SGO_2100**	ABC transporter substrate-binding protein	-0.870	6.617	0.0014	0.0029	2.646	8.845	0.0002	0.0001	2.702	8.891	0.0001	0.0000	3.516	8.239	0.0002	0.0001	3.572	7.678	0.0001	0.0000	0.055	9.145	0.1191	0.3624
**SGO_2102**	hypothetical protein SGO_2102						5.180				2.170				4.667				1.046			-1.959	4.836	0.0805	0.2118
**SGO_2103**	hydrolase, haloacid dehalogenase-like family	0.861	3.079				1.585				1.585				1.933				1.322						
**SGO_2104**	srtB; sortase B	-1.959	9.563	0.0003	0.0002	-1.730	9.606	0.0001	0.0000	-1.198	9.753	0.0005	0.0011	0.230	7.893	0.0562	0.3877	0.762	7.600	0.0117	0.0501	0.532	8.288	0.0328	0.0594
**SGO_2105**	abpA; amylase-binding protein AbpA	-1.919	12.653	0.0005	0.0008	-6.934	12.327	0.0003	0.0003	-6.361	12.333	0.0002	0.0003	-5.016	9.922	0.0000	0.0000	-4.443	9.332	0.0000	0.0000	0.573	6.179	0.0050	0.0019
**SGO_2106**	ribose-phosphate diphosphokinase	0.371	8.552	0.0141	0.0613	0.037	8.367	0.1120	0.5587	-0.130	8.283	0.0006	0.0015	-0.334	8.056	0.0167	0.0923	-0.501	7.373	0.0085	0.0332	-0.167	7.791	0.0396	0.0799
**SGO_2107**	general stress protein GSP-781		3.785				3.785				3.785														

Log$_2$ Ratios

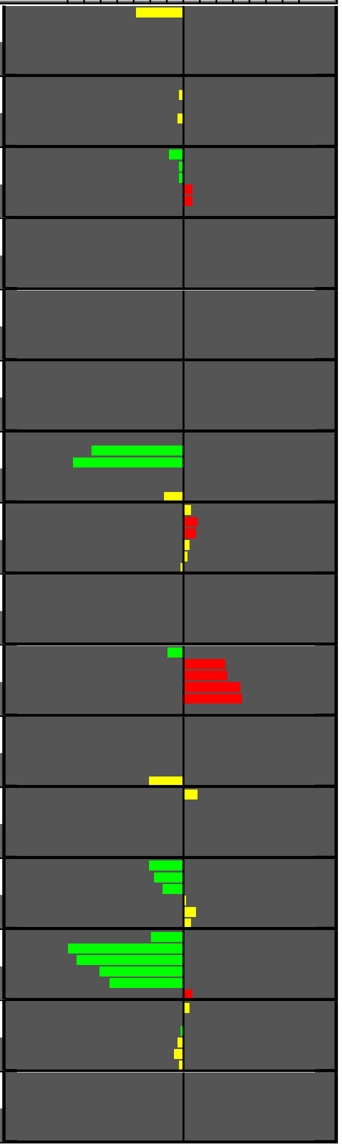

Show detected proteins only
Show all proteins
Filter by category:
ABC Transporter

Proteins found: 1179

Dot Plots
Dot Plots

Test	Cutoff
q-Value	.005
p-Value	

	Signif	Direction	Applies To
(red)	yes	+	ratios, bars
(yellow)	no	n/a	bars
(green)	yes	–	ratios, bars
(light red)	yes	+	*p*- , *q*-Values
(light green)	yes	–	

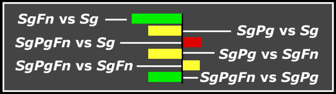

Streptococcus gordonii

	SgFn vs Sg				*SgPg vs Sg*				*SgPgFn vs Sg*				*SgPg vs SgFn*				*SgPgFn vs SgFn*				*SgPgFn vs SgPg*			
Protein	Ratio	Sum	*q*-Val	*p*-Val	Ratio	Sum	*q*-Val	*p*-Val	Ratio	Sum	*q*-Val	*p*-Val	Ratio	Sum	*q*-Val	*p*-Val	Ratio	Sum	*q*-Val	*p*-Val	Ratio	Sum	*q*-Val	*p*-Val
**SGO_2108**		6.472			-2.938	6.651	0.0001	0.0000		6.472				3.036								3.036		
	mreC; cell shape-determining protein MreC																							
**SGO_2133**	-0.914	10.415	0.0008	0.0014	-1.361	10.272	0.0002	0.0001	-1.206	10.318	0.0002	0.0002	-0.447	9.169	0.0060	0.0240	-0.292	8.626	0.0149	0.0680	0.155	9.003	0.0195	0.0267
	Cell division protein ftsH-like protein																							
**SGO_2134**	1.391	8.622	0.0005	0.0008	-0.013	7.764	0.1588	0.8316	-0.203	7.684	0.0476	0.4172	-1.405	8.099	0.0001	0.0000	-1.595	7.444	0.0006	0.0004	-0.190	7.151	0.1153	0.3458
	hpt; hypoxanthine phosphoribosyltransferase																							
**SGO_2138**		2.322				2.322				2.322														
	Septum formation initiator family																							
**SGO_2139**		1.379				1.380				1.380														
	S4 RNA-binding domain protein																							
**SGO_2140**		3.667				3.667				3.667														
	transcription-repair coupling factor																							
**SGO_2141**		1.000				1.000				1.000														
	pth; peptidyl-tRNA hydrolase																							
**SGO_2142**	-0.098	7.739	0.0609	0.3630	0.906	8.309	0.0011	0.0018	0.662	8.157	0.0025	0.0106	1.004	7.762	0.0008	0.0011	0.760	6.995	0.0026	0.0067	-0.244	8.063	0.0397	0.0812
	GTP-binding protein																							
**SGO_2145**		8.707			-0.255	9.587	0.0085	0.0270	-0.254	9.586	0.0013	0.0045		7.942				7.329			0.001	8.941	0.2492	0.9711
	comE; competence response regulator ComE																							
**SGO_2146**		6.837			-1.707	7.223	0.0003	0.0003	-2.665	6.947	0.0073	0.0420		4.618				2.046			-0.958	4.948	0.0402	0.0827
	comD; histidine protein kinase ComD																							
**SGO_2150**	-0.999	8.352	0.0006	0.0008	-1.324	8.249	0.0002	0.0001	-2.772	7.969	0.0002	0.0001	-0.325	7.102	0.0081	0.0362	-1.773	6.029	0.0014	0.0025	-1.449	6.392	0.0050	0.0020
	degP; serine protease																							

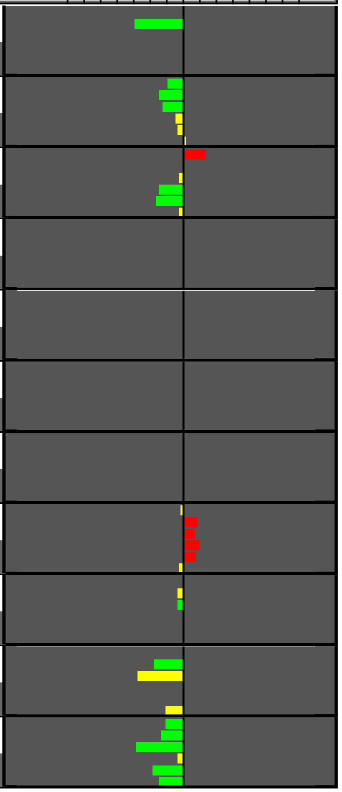

Show detected proteins only
Show all proteins
Filter by category:
ABC Transporter

Proteins found: 1179

Dot Plots
Dot Plots

Test	Cutoff
q-Value	.005
p-Value	

	Signif	Direction	Applies To
(red)	yes	+	ratios, bars
(yellow)	no	n/a	bars
(green)	yes	−	ratios, bars
(pink)	yes	+	*p*- , *q*-Values
(light green)	yes	−	

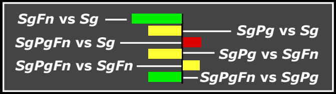

Hendrickson *et al.*
