# Supplementary material for: Proteomics of Streptococcus gordonii within a model developing oral microbial community
Source: BMC Microbiol. 2012 Sep 18;12:211. doi: 10.1186/1471-2180-12-211 (PMC3534352; doi:10.1186/1471-2180-12-211)
Supplement: Additional file 2 — SgFn_vs_Sg. A more detailed presentation of the relative abundance ratios for the comparison of SgFn and the Sg controls, including both raw and normalized spectral counts. Red and green highlights are used as in Additional file 1. [file 1471-2180-12-211-S2.pdf]

Protein	SgFn vs Sg Log$_2$ Ratio	Log$_2$ Sum	*q*-Value	*p*-Value	Raw *SgFn*	Raw *Sg*	Normalized *SgFn*	Normalized *Sg*	Description
SGO_0001	-0.902	7.046	0.0101	0.0398	9.500	32.000	20.7045	33.3027	dnaA; chromosomal replication initiator protein DnaA
					10.500	53.500	24.6409	53.5000	
SGO_0002	0.640	7.258	0.0146	0.0633	20.000	21.000	43.5884	21.8549	dnaN; DNA polymerase III, beta subunit
					20.500	39.500	48.1085	39.5000	
SGO_0004	-1.661	8.780	0.0020	0.0050	29.500	137.000	64.2929	142.5772	putative lipoprotein
					18.000	190.500	42.2416	190.5000	
SGO_0006	0.158	7.655	0.0485	0.2750	28.000	44.000	61.0238	45.7912	ABC transporter, ATP-binding protein
					19.500	49.000	45.7618	49.0000	
SGO_0007	-0.294	6.164	0.0417	0.2311	9.000	17.000	19.6148	17.6921	trpS; tryptophanyl-tRNA synthetase
					5.500	21.500	12.9072	21.5000	
SGO_0008	0.310	9.371	0.0149	0.0651	77.000	156.500	167.8155	162.8711	inosine-5'-monophosphate dehydrogenase
					84.500	133.000	198.3010	133.0000	
SGO_0011	-0.863	5.257	0.0270	0.1369		13.000		13.5292	proteinase, M16 family
					3.500	16.500	8.2136	16.5000	
SGO_0012	1.455	4.510	0.0205	0.0948	4.000		8.7177		mpp; peptidase, M16 family
					4.500	3.500	10.5604	3.5000	
SGO_0015	-2.771	6.426	0.0058	0.0196		39.500		41.1080	ABC transporter (ATP-binding protein)
					2.500	39.000	5.8669	39.0000	
SGO_0020	-0.115	4.222	0.1386	0.9320	1.500		3.2691		sdhB; L-serine dehydratase, iron-sulfur-dependent, beta subunit
					4.000	6.000	9.3870	6.0000	
SGO_0022	3.662	7.860	0.0001	0.0000	50.000	6.000	108.9711	6.2443	trmU; tRNA (5-methylaminomethyl-2-thiouridylate)-methyltransferase
					45.000	11.500	105.6041	11.5000	
SGO_0025	-0.570	5.781	0.0268	0.1355	3.500	14.500	7.6280	15.0903	gidA; glucose inhibited division protein A
					6.500	17.000	15.2539	17.0000	

Log$_2$ Ratios: -6 -4 -2 0 2 4 6

Show detected proteins only
Show all proteins
Filter by category:
ABC Transporter

Proteins found: 769

Test	Cutoff
q-Value	.005
p-Value	

	Signif	Direction	Applies To
red	yes	+	ratios, bars
yellow	no	n/a	bars
green	yes	−	ratios, bars
pink	yes	+	*p*- , *q*-Values
light green	yes	−	*p*- , *q*-Values

Dot Plots Dot Plots

Hendrickson *et al.*

Protein	SgFn vs Sg: Log_2 Ratio	Log_2 Sum	*q*-Value	*p*-Value	Raw: *SgFn*	Raw: *Sg*	Normalized: *SgFn*	Normalized: *Sg*	Description
SGO_0027	-0.380	6.937	0.0100	0.0393	11.000	30.500	23.9736	31.7416	rplI; ribosomal protein L9
					12.500	37.500	29.3345	37.5000	
SGO_0028	-1.442	5.255	0.0034	0.0101	2.000	11.000	4.3588	11.4478	dnaC; replicative DNA helicase
					2.500	16.500	5.8669	16.5000	
SGO_0030	2.151	7.906	0.0001	0.0000	44.500	19.000	96.9843	19.7735	aspB; aspartate transaminase
					42.000	24.500	98.5638	24.5000	
SGO_0032	-0.944	7.088	0.0028	0.0077	9.500	38.000	20.7045	39.5470	plsX; fatty acid/phospholipid synthesis protein PlsX
					11.000	50.000	25.8143	50.0000	
SGO_0035	2.367	5.314	0.0174	0.0779	7.500		16.3457		phosphoribosylformylglycinamidine synthase
					8.500	3.500	19.9474	3.5000	
SGO_0042	0.918	6.144	0.0003	0.0002	11.000	11.500	23.9736	11.9682	transcription regulator, GntR family
					9.500	12.500	22.2942	12.5000	
SGO_0054	1.565	8.469	0.0001	0.0001	59.000	40.000	128.5859	41.6284	dltA; D-alanine-activating enzyme
					58.000	48.000	136.1119	48.0000	
SGO_0056	-0.711	3.805	0.0112	0.0452	1.500	5.000	3.2691	5.2035	dltC; D-alanyl carrier protein
						5.500		5.5000	
SGO_0057	-1.818	8.223	0.0010	0.0018	17.500	99.500	38.1399	103.5506	dltD protein
					12.000	129.000	28.1611	129.0000	
SGO_0059	-2.492	11.167	0.0067	0.0235	93.500	1257.500	203.7760	1308.6923	pXO1; hypothetical protein SGO_0059
					56.500	653.500	132.5918	653.5000	
SGO_0060	-4.809	8.069	0.0112	0.0455		118.000		122.8037	hypothetical protein SGO_0060
					2.000	141.000	4.6935	141.0000	
SGO_0063	-1.969	7.830	0.0001	0.0000	9.500	86.000	20.7045	89.5010	hypothetical protein SGO_0063
					11.000	91.500	25.8143	91.5000	

Show detected proteins only
Show all proteins
Filter by category:
ABC Transporter

Proteins found: 769

Test	Cutoff
***q*-Value**	**.005**
p-Value	

	Signif	Direction	Applies To
red	yes	+	ratios, bars
yellow	no	n/a	bars
green	yes	−	ratios, bars
pink	yes	+	*p*- , *q*-Values
light green	yes	−	*p*- , *q*-Values

Dot Plots Dot Plots

Protein	SgFn vs Sg: Log_2 Ratio	Log_2 Sum	*q*-Value	*p*-Value	Raw: *SgFn*	Raw: *Sg*	Normalized: *SgFn*	Normalized: *Sg*	Description
SGO_0064	-2.780	8.948	0.0001	0.0000	16.000	214.000	34.8708	222.7119	FtsK/SpoIIIE family protein
					12.000	208.000	28.1611	208.0000	
SGO_0065	0.041	7.328	0.0927	0.5936	17.000	37.500	37.0502	39.0266	hypothetical protein SGO_0065
					19.000	40.000	44.5884	40.0000	
SGO_0067	-1.326	6.056	0.0003	0.0004	5.000	23.000	10.8971	23.9363	protein with prophage function domain
					3.500	23.500	8.2136	23.5000	
SGO_0068	-1.848	6.075	0.0116	0.0478		30.000		31.2213	lipoprotein, putative
					3.500	28.000	8.2136	28.0000	
SGO_0078	-2.233	5.972	0.0356	0.1899		34.000		35.3841	hypothetical protein SGO_0078
					2.500	21.500	5.8669	21.5000	
SGO_0080	-1.239	7.315	0.0013	0.0026	13.500	51.000	29.4222	53.0762	hypothetical protein SGO_0080
					8.000	58.000	18.7741	58.0000	
SGO_0081	1.176	4.906	0.0041	0.0126	4.500	3.000	9.8074	3.1221	hypothetical protein SGO_0081
					4.500	6.500	10.5604	6.5000	
SGO_0100	0.845	4.371	0.0570	0.3329		7.500		7.8053	maltose operon transcription repressor
					4.000	3.500	9.3870	3.5000	
SGO_0104	-0.242	9.829	0.0207	0.0962	108.000	234.000	235.3776	243.5261	Maltose/maltodextrin-binding protein precursor
					78.000	247.500	183.0470	247.5000	
SGO_0105	1.097	6.362	0.0042	0.0127	15.000	14.000	32.6913	14.5699	malQ; 4-alpha-glucanotransferase
					10.000	11.500	23.4676	11.5000	
SGO_0106	2.848	8.574	0.0005	0.0007	84.500	19.000	184.1612	19.7735	glgP-2; maltodextrin phosphorylase
					64.000	27.000	150.1924	27.0000	
SGO_0108	-1.035	6.092	0.0022	0.0058	4.000	22.500	8.7177	23.4160	ruvB; Holliday junction DNA helicase RuvB
					6.000	22.000	14.0805	22.0000	

Log_2 Ratios: -6 -4 -2 0 2 4 6

Show detected proteins only
Show all proteins
Filter by category:
ABC Transporter

Proteins found: 769

Test	Cutoff
***q*-Value** / *p*-Value	.005

	Signif	Direction	Applies To
red	yes	+	ratios, bars
yellow	no	n/a	bars
green	yes	–	ratios, bars
pink	yes	+	*p*- , *q*-Values
light green	yes	–	*p*- , *q*-Values

Dot Plots Dot Plots

Protein	SgFn vs Sg Log2 Ratio	Log2 Sum	*q*-Value	*p*-Value	Raw SgFn	Raw Sg	Normalized SgFn	Normalized Sg	Description
SGO_0113	-2.089	13.012	0.0001	0.0000	375.000	3329.000	817.2833	3464.5223	acdH; alcohol-acetaldehyde dehydrogenase
					321.500	3222.500	754.4823	3222.5000	
SGO_0131	-0.135	6.282	0.0858	0.5442	9.000	11.000	19.6148	11.4478	v-type sodium ATP synthase, chain E
					6.500	31.500	15.2539	31.5000	
SGO_0134	-0.328	4.635	0.0580	0.3390		7.500		7.8053	acetyltransferase, GNAT family
					3.000	10.000	7.0403	10.0000	
SGO_0135	-0.017	8.321	0.1433	0.9713	41.500	77.500	90.4460	80.6550	v-type sodium ATP synthase, subunit A
					29.500	79.500	69.2293	79.5000	
SGO_0136	-0.696	8.587	0.0016	0.0037	35.500	106.000	77.3695	110.3152	v-type sodium ATP synthase, chain B
					29.500	127.500	69.2293	127.5000	
SGO_0138	0.579	3.778							LysM domain protein
					3.500	5.500	8.2136	5.5000	
SGO_0139	0.506	7.391	0.0013	0.0027	21.500	32.000	46.8576	33.3027	thrC; threonine synthase
					22.000	36.000	51.6287	36.0000	
SGO_0144	-1.032	5.671	0.0020	0.0050	4.500	15.000	9.8074	15.6106	hypothetical protein SGO_0144
					3.000	18.500	7.0403	18.5000	
SGO_0145	0.275	8.112	0.0341	0.1807	41.500	61.500	90.4460	64.0036	polI; DNA polymerase I
					26.500	60.000	62.1891	60.0000	
SGO_0146	1.056	6.377	0.0048	0.0152	15.000	11.000	32.6913	11.4478	CoA-binding domain protein
					10.000	15.500	23.4676	15.5000	
SGO_0152	0.387	5.773	0.0519	0.2961	10.500	10.500	22.8839	10.9275	tgt; queuine tRNA-ribosyltransferase
					4.000	11.500	9.3870	11.5000	
SGO_0154	1.787	11.314	0.0007	0.0010	493.000	309.500	1074.4550	322.0996	pgi; glucose-6-phosphate isomerase
					382.500	251.500	897.6345	251.5000	

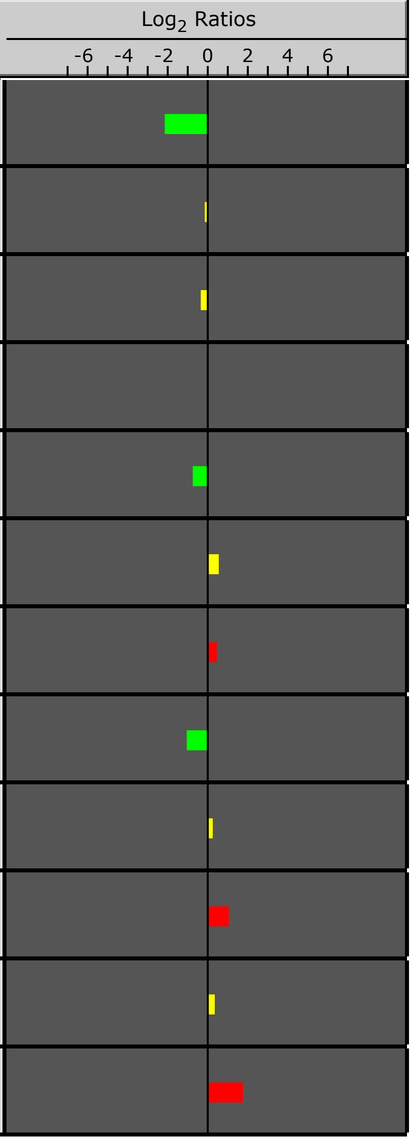

Show detected proteins only
Show all proteins
Filter by category:
ABC Transporter

Proteins found: 769

Test	Cutoff
q-Value	.005
p-Value	

	Signif	Direction	Applies To
red	yes	+	ratios, bars
yellow	no	n/a	bars
green	yes	−	ratios, bars
pink	yes	+	*p*- , *q*-Values
light green	yes	−	*p*- , *q*-Values

Dot Plots Dot Plots

Protein	SgFn vs Sg Log$_2$ Ratio	Log$_2$ Sum	*q*-Value	*p*-Value	Raw *SgFn*	Raw *Sg*	Normalized *SgFn*	Normalized *Sg*	Description
SGO_0158	2.802	8.548	0.0003	0.0002	70.000	24.500	152.5595	25.4974	2,3,4,5-tetrahydropyridine-2-carboxylate N-succinyltransferase, putative
					74.500	21.500	174.8334	21.5000	
SGO_0159	1.294	3.787			4.500		9.8074		hippurate hydrolase
						4.000		4.0000	
SGO_0163	0.563	7.521	0.0030	0.0085	26.500	32.000	57.7547	33.3027	galU; UTP-glucose-1-phosphate uridylyltransferase
					22.000	41.000	51.6287	41.0000	
SGO_0164	0.296	7.639	0.0213	0.0992	22.000	40.000	47.9473	41.6284	Glycerol-3-phosphate dehydrogenase [NAD(P)+] (NAD(P)H-dependent glycerol-3-phosphate dehydrogenase)
					26.500	47.500	62.1891	47.5000	
SGO_0168	-0.600	4.566	0.0250	0.1212		8.000		8.3257	hydrolase, NUDIX family
					2.500	9.500	5.8669	9.5000	
SGO_0169	1.929	6.595	0.0023	0.0059	20.500	7.500	44.6782	7.8053	dut; dUTP diphosphatase
					13.500	12.500	31.6812	12.5000	
SGO_0171	-0.989	5.628	0.0409	0.2246	4.500	15.500	9.8074	16.1310	radA; DNA repair protein RadA
						23.500		23.5000	
SGO_0172	-0.682	6.005	0.0089	0.0337	7.000	24.000	15.2560	24.9770	conserved hypothetical protein TIGR00266
						24.000		24.0000	
SGO_0173	-1.237	7.924	0.0003	0.0002	15.000	80.000	32.6913	83.2568	Carbonic anhydrase
					17.000	87.000	39.8949	87.0000	
SGO_0174	0.758	8.458	0.0073	0.0266	60.000	60.500	130.7653	62.9629	gltX; glutamyl-tRNA synthetase
					39.000	66.500	91.5235	66.5000	
SGO_0180	-0.946	6.660	0.0122	0.0506	6.500	24.000	14.1662	24.9770	jag; hypothetical protein SGO_0180
					8.500	42.000	19.9474	42.0000	
SGO_0182	0.723	4.937	0.0798	0.4978	3.000		6.5383		sapR; sakacin A production response regulator
					7.500	6.500	17.6007	6.5000	

Log$_2$ Ratios: -6 -4 -2 0 2 4 6

Show detected proteins only
Show all proteins
Filter by category:
ABC Transporter

Proteins found: 769

Test	Cutoff
q-Value	.005
p-Value	

	Signif	Direction	Applies To
red	yes	+	ratios, bars
yellow	no	n/a	bars
green	yes	–	ratios, bars
pink	yes	+	*p*- , *q*-Values
light green	yes	–	*p*- , *q*-Values

Dot Plots Dot Plots

Hendrickson *et al.*

Protein	SgFn vs Sg Log$_2$ Ratio	Log$_2$ Sum	*q*-Value	*p*-Value	Raw *SgFn*	Raw *Sg*	Normalized *SgFn*	Normalized *Sg*	Description
SGO_0188	-0.017	5.264	0.1383	0.9279	5.000	8.000	10.8971	8.3257	hydrolase, TatD family
					3.500	11.000	8.2136	11.0000	
SGO_0193	0.004	4.805	0.1428	0.9653	1.500	3.000	3.2691	3.1221	ksgA; dimethyladenosine transferase
					4.500	11.000	10.5604	11.0000	
SGO_0198	2.600	8.472	0.0001	0.0001	67.000	27.500	146.0213	28.6195	rpe; ribulose-phosphate 3-epimerase
					67.500	22.000	158.4061	22.0000	
SGO_0200	-1.421	7.784	0.0001	0.0001	13.500	74.500	29.4222	77.5329	competence-induced protein Ccs50
					13.000	83.000	30.5078	83.0000	
SGO_0201	1.296	8.289	0.0028	0.0079	43.000	47.000	93.7151	48.9134	cmp-binding-factor 1
					55.000	41.000	129.0716	41.0000	
SGO_0202	0.149	5.698	0.0870	0.5536	5.000	14.000	10.8971	14.5699	pur operon repressor
					7.000	10.000	16.4273	10.0000	
SGO_0204	-0.081	8.109	0.1339	0.8941	39.500	63.500	86.0872	66.0851	rpsL; ribosomal protein S12
					21.500	73.500	50.4553	73.5000	
SGO_0205	0.380	9.252	0.0236	0.1119	92.500	113.000	201.5965	117.6002	rpsG; ribosomal protein S7
					61.500	146.000	144.3255	146.0000	
SGO_0206	0.449	12.222	0.0005	0.0006	652.000	972.500	1420.9831	1012.0901	fusA; translation elongation factor G
					569.500	1007.500	1336.4780	1007.5000	
SGO_0207	1.189	12.534	0.0005	0.0007	934.000	748.500	2035.5801	778.9711	gap; glyceraldehyde-3-phosphate dehydrogenase, type I
					884.000	1043.000	2074.5331	1043.0000	
SGO_0208	0.596	6.284	0.0337	0.1782	12.500	10.000	27.2428	10.4071	LPXTG cell wall surface protein, glycosyl hydrolase family
					8.000	21.500	18.7741	21.5000	
SGO_0209	1.455	12.630	0.0002	0.0001	1110.000	845.500	2419.1584	879.9200	pgk; phosphoglycerate kinase
					949.000	814.500	2227.0723	814.5000	

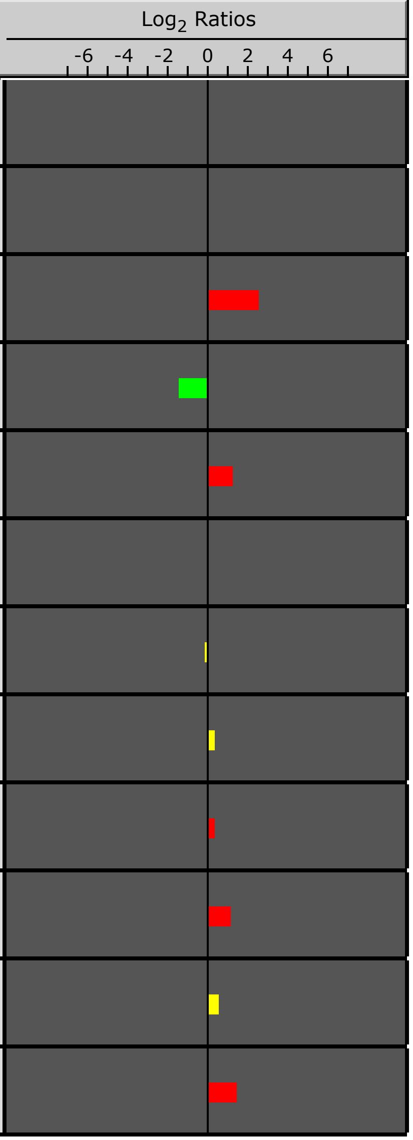

Show detected proteins only
Show all proteins
Filter by category:
ABC Transporter

Proteins found: 769

Test	Cutoff
***q*-Value**	.005
p-Value	

	Signif	Direction	Applies To
(red)	yes	+	ratios, bars
(yellow)	no	n/a	bars
(green)	yes	−	ratios, bars
(pink)	yes	+	*p*- , *q*-Values
(light green)	yes	−	*p*- , *q*-Values

Dot Plots Dot Plots

Protein	SgFn vs Sg $\log_2$ Ratio	$\log_2$ Sum	*q*-Value	*p*-Value	Raw *SgFn*	Raw *Sg*	Normalized *SgFn*	Normalized *Sg*	Description
**SGO_0210**	-0.950	8.543	0.0059	0.0198	33.500	98.500	73.0106	102.5099	sspA; streptococcal surface protein A
					23.000	143.500	53.9754	143.5000	
**SGO_0211**	-4.047	9.262	0.0003	0.0003	8.500	254.000	18.5251	264.3402	sspB; streptococcal surface protein B
					7.000	314.500	16.4273	314.5000	
**SGO_0215**	0.153	8.746	0.0017	0.0040	51.500	95.500	112.2402	99.3878	glnA; glutamine synthetase, type I
					48.500	104.000	113.8177	104.0000	
**SGO_0219**	-0.136	8.107	0.0278	0.1417	29.000	74.500	63.2032	77.5329	metallo-beta-lactamase superfamily protein 1
					29.000	67.000	68.0559	67.0000	
**SGO_0223**	0.818	4.028	0.0150	0.0659	3.500	4.500	7.6280	4.6832	glycoproteinase family protein
						4.000		4.0000	
**SGO_0230**	-0.275	5.406	0.1056	0.6858	6.500	9.000	14.1662	9.3664	Protein of unknown function, DUF536 family
					2.500	13.000	5.8669	13.0000	
**SGO_0233**	-1.071	6.570	0.0111	0.0442	4.500	35.000	9.8074	36.4248	lipoprotein, putative
					9.500	26.500	22.2942	26.5000	
**SGO_0234**	0.223	7.887	0.0036	0.0108	30.500	53.000	66.4724	55.1576	pepX; X-Pro dipeptidyl-peptidase
					26.000	54.000	61.0157	54.0000	
**SGO_0237**	0.811	5.501	0.0302	0.1571	4.500	5.000	9.8074	5.2035	ccpA; CcpA protein (proteinase)
					8.000	11.500	18.7741	11.5000	
**SGO_0242**	1.066	2.687			2.000	2.000	4.3588	2.0814	FMN-dependent dehydrogenase family protein
**SGO_0244**	2.219	5.446	0.0014	0.0029	9.500	3.500	20.7045	3.6425	hydroxymethylglutaryl-CoA synthase
					6.500	4.000	15.2539	4.0000	
**SGO_0247**	-0.232	11.349	0.0506	0.2878	223.500	709.000	487.1008	737.8631	pfl; formate acetyltransferase
					308.500	659.000	723.9745	659.0000	

$\log_2$ Ratios: -6 -4 -2 0 2 4 6

Show detected proteins only
Show all proteins
Filter by category:
ABC Transporter

Proteins found: 769

Test	Cutoff
***q*-Value**	.005
p-Value	

	Signif	Direction	Applies To
red	yes	+	ratios, bars
yellow	no	n/a	bars
green	yes	−	ratios, bars
pink	yes	+	*p*- , *q*-Values
light green	yes	−	*p*- , *q*-Values

Dot Plots Dot Plots

Hendrickson *et al.*

Protein	SgFn vs Sg				Raw		Normalized		Description
	Log_2 Ratio	Log_2 Sum	*q*-Value	*p*-Value	*SgFn*	*Sg*	*SgFn*	*Sg*	
SGO_0252	0.478	5.107	0.0169	0.0754	5.500	6.500	11.9868	6.7646	possible TetR-type transcriptional regulator
					3.500	7.500	8.2136	7.5000	
SGO_0255	-1.915	7.565	0.0021	0.0053	10.000	84.500	21.7942	87.9400	Signal peptidase I
					7.500	62.000	17.6007	62.0000	
SGO_0256	0.549	4.962	0.0603	0.3566		6.500		6.7646	rnhC; ribonuclease HIII
					5.500	11.500	12.9072	11.5000	
SGO_0258	1.231	3.328							hypothetical protein SGO_0258
					3.000	3.000	7.0403	3.0000	
SGO_0260	-0.703	6.887	0.0044	0.0135	8.500	33.500	18.5251	34.8638	DNA mismatch binding protein MutS2
					11.500	38.000	26.9877	38.0000	
SGO_0262	0.568	6.914	0.0032	0.0094	18.000	24.500	39.2296	25.4974	dipeptidase
					14.000	23.000	32.8546	23.0000	
SGO_0263	0.095	7.290	0.0900	0.5751	14.000	34.000	30.5119	35.3841	trx-1; thioredoxin
					22.000	39.000	51.6287	39.0000	
SGO_0272	0.119	6.592	0.0674	0.4081	13.500	22.500	29.4222	23.4160	hypothetical protein SGO_0272
					9.000	22.500	21.1208	22.5000	
SGO_0276	0.718	9.428	0.0029	0.0083	108.500	132.500	236.4673	137.8940	gdhA; glutamate dehydrogenase (NADP)
					82.000	122.000	192.4341	122.0000	
SGO_0277	0.354	6.057	0.0447	0.2509	6.500	11.500	14.1662	11.9682	pyrA; Dihydroorotate dehydrogenase
					10.000	17.000	23.4676	17.0000	
SGO_0278	1.709	3.094			3.000		6.5383		msrA; Peptide methionine sulfoxide reductase msrA/msrB
						2.000		2.0000	
SGO_0280	0.678	7.016	0.0256	0.1260	25.000	24.000	54.4856	24.9770	trzA; ethylammeline chlorohydrolase
					11.500	23.000	26.9877	23.0000	

Log_2 Ratios: -6 -4 -2 0 2 4 6

Show detected proteins only
Show all proteins
Filter by category:
ABC Transporter

Proteins found: 769

Test	Cutoff
***q*-Value**	**.005**
p-Value	

	Signif	Direction	Applies To
red	yes	+	ratios, bars
yellow	no	n/a	bars
green	yes	−	ratios, bars
pink	yes	+	*p-* , *q*-Values
light green	yes	−	*p-* , *q*-Values

Dot Plots Dot Plots

Protein	SgFn vs Sg: Log_2 Ratio	Log_2 Sum	*q*-Value	*p*-Value	Raw: *SgFn*	Raw: *Sg*	Normalized: *SgFn*	Normalized: *Sg*	Description
SGO_0286	-1.447	5.729	0.0117	0.0484		20.500		21.3345	DNA mismatch repair protein MutS, putative
					3.500	23.500	8.2136	23.5000	
SGO_0291	-1.699	7.622	0.0005	0.0008	11.000	78.500	23.9736	81.6957	copper-translocating P-type ATPase
					9.500	69.000	22.2942	69.0000	
SGO_0292	-1.515	8.103	0.0001	0.0000	15.500	100.000	33.7810	104.0710	spxB; pyruvate oxidase
					16.000	99.500	37.5481	99.5000	
SGO_0297	2.180	5.828	0.0348	0.1852	9.000		19.6148		6-phospho-beta-glucosidase
					13.500	5.500	31.6812	5.5000	
SGO_0310	2.302	4.772	0.0011	0.0022	4.500	2.500	9.8074	2.6018	metE; 5-methyltetrahydropteroyltriglutamate--homocysteine S-methyltransferase
					5.500	2.000	12.9072	2.0000	
SGO_0312	1.214	11.377	0.0002	0.0002	444.000	387.000	967.6634	402.7546	xfp; D-xylulose 5-phosphate/D-fructose 6-phosphate phosphoketolase
					380.000	398.000	891.7676	398.0000	
SGO_0321	1.577	7.033	0.0012	0.0024	23.500	11.500	51.2164	11.9682	polypeptide deformylase
					19.500	22.000	45.7618	22.0000	
SGO_0327	0.798	3.924	0.0200	0.0913		3.500		3.6425	Lipopolysaccharide N-acetylglucosaminyltransferase
					3.000	4.500	7.0403	4.5000	
SGO_0333	-0.323	8.323	0.0334	0.1757	39.500	77.500	86.0872	80.6550	rpsO; ribosomal protein S15
					24.500	96.000	57.4955	96.0000	
SGO_0339	3.050	5.724	0.0138	0.0594	10.500		22.8839		hypothetical protein SGO_0339
					11.500	3.000	26.9877	3.0000	
SGO_0342	1.201	8.387	0.0004	0.0004	56.500	47.500	123.1373	49.4337	pepF-2; oligoendopeptidase
					47.000	52.000	110.2976	52.0000	
SGO_0344	0.219	9.215	0.0124	0.0516	79.000	128.500	172.1743	133.7312	pnpA; polyribonucleotide nucleotidyltransferase
					63.000	140.500	147.8457	140.5000	

Log_2 Ratios: -6 -4 -2 0 2 4 6

Show detected proteins only
Show all proteins
Filter by category:
ABC Transporter

Proteins found: 769

Test	Cutoff
***q*-Value**	**.005**
p-Value	

Color	Signif	Direction	Applies To
red	yes	+	ratios, bars
yellow	no	n/a	bars
green	yes	−	ratios, bars
pink	yes	+	*p*- , *q*-Values
light green	yes	−	*p*- , *q*-Values

Dot Plots Dot Plots

Protein	SgFn vs Sg Log$_2$ Ratio	Log$_2$ Sum	*q*-Value	*p*-Value	Raw *SgFn*	Raw *Sg*	Normalized *SgFn*	Normalized *Sg*	Description	Log$_2$ Ratios (-6 -4 -2 0 2 4 6)
SGO_0348	-0.407	4.511			4.500		9.8074		reductase	
						13.000		13.0000		
SGO_0349	0.798	7.081	0.0084	0.0315	16.000	22.000	34.8708	22.8956	cysS; cysteinyl-tRNA synthetase	
					22.000	26.000	51.6287	26.0000		
SGO_0352	-0.349	7.992	0.0015	0.0032	25.000	66.000	54.4856	68.6868	ABC transporter, ATP-binding protein SP1580	
					24.500	74.000	57.4955	74.0000		
SGO_0353	1.406	4.544	0.0136	0.0580	2.500	3.500	5.4486	3.6425	transport protein	
					5.000	2.500	11.7338	2.5000		
SGO_0355	0.730	5.564	0.0047	0.0149	6.000	7.500	13.0765	7.8053	RNA methyltransferase, TrmH family, group 3	
					7.000	10.000	16.4273	10.0000		
SGO_0357	1.098	7.238	0.0022	0.0058	23.000	17.500	50.1267	18.2124	degV; DegV family fatty acid binding protein	
					22.000	31.000	51.6287	31.0000		
SGO_0358	0.443	9.550	0.0112	0.0451	108.500	133.000	236.4673	138.4144	rplM; ribosomal protein L13	
					83.000	180.000	194.7808	180.0000		
SGO_0359	-0.649	8.262	0.0156	0.0689	34.500	76.000	75.1901	79.0939	rpsI; ribosomal protein S9	
					19.500	107.000	45.7618	107.0000		
SGO_0361	0.296	4.951	0.0570	0.3323	3.000	8.000	6.5383	8.3257	immunity repressor protein	
					4.500	5.500	10.5604	5.5000		
SGO_0368	1.733	4.471	0.0048	0.0150	3.000	2.000	6.5383	2.0814	merA; mercury(II) reductase	
					4.500	3.000	10.5604	3.0000		
SGO_0372	1.711	6.450	0.0017	0.0040	17.000	13.000	37.0502	13.5292	malate oxidoreductase	
					12.500	7.500	29.3345	7.5000		
SGO_0374	0.739	6.858	0.0028	0.0079	15.000	22.500	32.6913	23.4160	Response regulator of the LytR/AlgR family	
					17.000	20.000	39.8949	20.0000		

Show detected proteins only
Show all proteins
Filter by category:
ABC Transporter

Proteins found: 769

Test	Cutoff
***q*-Value**	**.005**
p-Value	

	Signif	Direction	Applies To
(red)	yes	+	ratios, bars
(yellow)	no	n/a	bars
(green)	yes	−	ratios, bars
(pink)	yes	+	*p*- , *q*-Values
(light green)	yes	−	*p*- , *q*-Values

Dot Plots Dot Plots

Hendrickson *et al.*

Protein	SgFn vs Sg Log_2 Ratio	Log_2 Sum	*q*-Value	*p*-Value	Raw *SgFn*	Raw *Sg*	Normalized *SgFn*	Normalized *Sg*	Description
SGO_0384	-0.291	5.820	0.0649	0.3905	4.500	10.500	9.8074	10.9275	putative carboxylate-amine/thiol ligase
					6.500	20.500	15.2539	20.5000	
SGO_0385	0.670	7.728	0.0026	0.0070	27.000	39.500	58.8444	41.1080	exo-beta-D-fructosidase
					30.500	40.500	71.5761	40.5000	
SGO_0388	0.224	7.351	0.0555	0.3217	19.500	29.000	42.4987	30.1806	LPXTG cell wall surface protein, zinc carboxypeptidase family
					19.000	46.000	44.5884	46.0000	
SGO_0389	3.458	5.613	0.0418	0.2326	7.000		15.2560		possible phosphoserine phosphatase
					13.500	2.000	31.6812	2.0000	
SGO_0390	1.938	8.321	0.0014	0.0028	66.500	30.000	144.9316	31.2213	glycerol-3-phosphate dehydrogenase (NAD (P)+)
					46.500	34.500	109.1242	34.5000	
SGO_0392	3.821	6.458	0.0090	0.0344	18.500		40.3193		phosphoglycerate mutase
					19.000	3.000	44.5884	3.0000	
SGO_0393	-0.858	5.430	0.0702	0.4299	4.000	10.000	8.7177	10.4071	D-alanyl-D-alanine carboxypeptidase
						24.000		24.0000	
SGO_0398	-1.902	7.059	0.0021	0.0053	5.000	58.500	10.8971	60.8815	ABC transporter ATP-binding protein
					7.500	44.000	17.6007	44.0000	
SGO_0400	0.608	4.887	0.0421	0.2346	6.500	5.500	14.1662	5.7239	hrcA; heat-inducible transcription repressor HrcA
					2.000	5.000	4.6935	5.0000	
SGO_0401	0.727	7.007	0.0294	0.1511	16.500	14.000	35.9605	14.5699	grpE; co-chaperone GrpE
					17.500	37.000	41.0682	37.0000	
SGO_0402	1.202	11.615	0.0001	0.0001	492.500	434.500	1073.3653	452.1883	dnaK; DnaK chaperone protein
					474.000	499.000	1112.3628	499.0000	
SGO_0404	-2.009	7.978	0.0052	0.0171	10.500	71.000	22.8839	73.8904	dnaJ; DnaJ chaparone protein
					11.000	129.500	25.8143	129.5000	

Log_2 Ratios: -6 -4 -2 0 2 4 6

Show detected proteins only
Show all proteins
Filter by category:
ABC Transporter

Proteins found: 769

Test	Cutoff
***q*-Value**	**.005**
p-Value	

	Signif	Direction	Applies To
red	yes	+	ratios, bars
yellow	no	n/a	bars
green	yes	–	ratios, bars
pink	yes	+	*p*- , *q*-Values
light green	yes	–	*p*- , *q*-Values

Dot Plots Dot Plots

Protein	SgFn vs Sg Log2 Ratio	SgFn vs Sg Log2 Sum	SgFn vs Sg *q*-Value	SgFn vs Sg *p*-Value	Raw *SgFn*	Raw *Sg*	Normalized *SgFn*	Normalized *Sg*	Description
SGO_0407	1.455	4.510	0.0205	0.0948	4.000		8.7177		truA; tRNA pseudouridine synthase A
					4.500	3.500	10.5604	3.5000	
SGO_0409	7.517	9.176	0.0184	0.0834	115.000	1.500	250.6335	1.5611	pyridoxine kinase
					139.000		326.1992		
SGO_0412	0.157	10.717	0.0298	0.1538	216.500	353.500	471.8449	367.8908	tig; trigger factor
					177.000	428.500	415.3760	428.5000	
SGO_0413	2.206	7.129	0.0003	0.0004	25.000	15.500	54.4856	16.1310	DNA-directed RNA polymerase delta chain
					25.500	9.500	59.8423	9.5000	
SGO_0415	-1.134	10.674	0.0005	0.0007	125.500	569.500	273.5175	592.6841	secA; preprotein translocase, SecA subunit
					101.500	529.500	238.1958	529.5000	
SGO_0416	1.455	7.692	0.0001	0.0001	35.000	24.000	76.2798	24.9770	phospho-2-dehydro-3-deoxyheptonate aldolase
					32.000	30.500	75.0962	30.5000	
SGO_0425	2.236	3.572			4.500	2.000	9.8074	2.0814	ansB; asparaginase
SGO_0426	0.886	6.136	0.0062	0.0214	9.000	9.500	19.6148	9.8867	Cof family protein
					11.000	15.000	25.8143	15.0000	
SGO_0427	3.220	7.650	0.0005	0.0006	37.500	9.500	81.7283	9.8867	universal stress protein family
					42.500	9.500	99.7372	9.5000	
SGO_0429	-0.087	9.283	0.0341	0.1809	67.000	162.000	146.0213	168.5950	aspartate transaminase
					66.500	152.500	156.0593	152.5000	
SGO_0430	-2.297	9.650	0.0001	0.0000	34.000	314.000	74.1003	326.7828	LPXTG cell wall surface protein
					26.500	340.500	62.1891	340.5000	
SGO_0431	0.352	4.954	0.0932	0.5974		6.500		6.7646	GTP-sensing transcriptional pleiotropic repressor codY
					5.000	12.500	11.7338	12.5000	

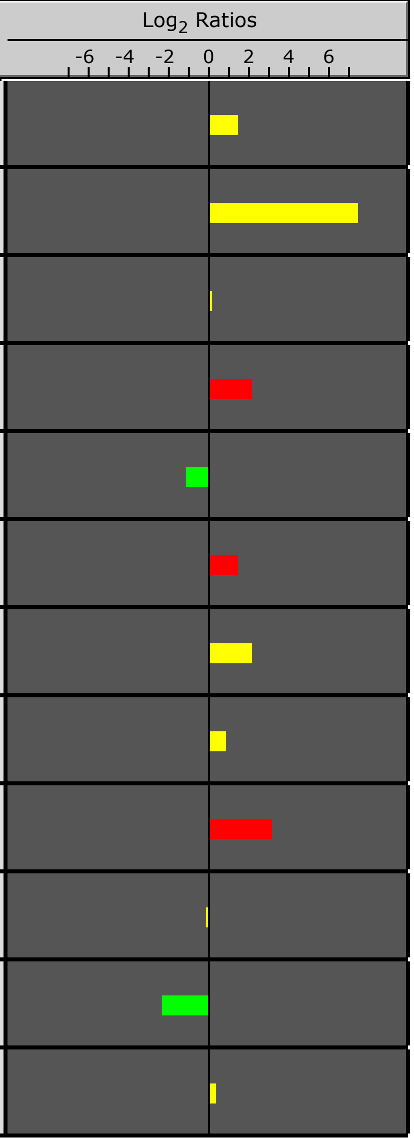

Show detected proteins only
Show all proteins
Filter by category:
ABC Transporter

Proteins found: 769

Test	Cutoff
***q*-Value**	**.005**
p-Value	

	Signif	Direction	Applies To
(red)	yes	+	ratios, bars
(yellow)	no	n/a	bars
(green)	yes	−	ratios, bars
(pink)	yes	+	*p*- , *q*-Values
(light green)	yes	−	*p*- , *q*-Values

Dot Plots Dot Plots

Hendrickson *et al.*

Streptococcus gordonii

Protein	SgFn vs Sg Log$_2$ Ratio	Log$_2$ Sum	*q*-Value	*p*-Value	Raw *SgFn*	Raw *Sg*	Normalized *SgFn*	Normalized *Sg*	Description
SGO_0432	-1.338	6.161	0.0267	0.1349		32.500		33.8231	entB; isochorismatase family protein
					5.000	26.000	11.7338	26.0000	
SGO_0434	-0.624	7.577	0.0152	0.0669	13.000	49.000	28.3325	50.9948	aspS-2; aspartyl-tRNA synthetase
					20.500	63.500	48.1085	63.5000	
SGO_0435	-0.058	6.298	0.0814	0.5104	8.500	17.500	18.5251	18.2124	gatC; glutamyl-tRNA(Gln) amidotransferase, C subunit
					8.500	22.000	19.9474	22.0000	
SGO_0436	-0.868	9.074	0.0011	0.0021	46.000	179.500	100.2534	186.8074	gatA; glutamyl-tRNA(Gln) amidotransferase, A subunit
					38.500	161.500	90.3501	161.5000	
SGO_0437	-0.052	8.674	0.1104	0.7212	52.000	100.000	113.3299	104.0710	gatB; glutamyl-tRNA(Gln) amidotransferase, B subunit
					37.500	103.000	88.0034	103.0000	
SGO_0440	4.334	7.132	0.0002	0.0002	28.500	3.000	62.1135	3.1221	L-iditol 2-dehydrogenase BH3949
					30.500	3.500	71.5761	3.5000	
SGO_0445	0.991	7.156	0.0005	0.0006	22.500	21.000	49.0370	21.8549	GTP-binding protein
					19.500	26.000	45.7618	26.0000	
SGO_0447	0.891	5.986	0.0080	0.0300	11.500	10.000	25.0634	10.4071	nadD; nicotinate (nicotinamide) nucleotide adenylyltransferase
					7.000	11.500	16.4273	11.5000	
SGO_0448	1.496	5.978	0.0028	0.0078	12.000	10.500	26.1531	10.9275	conserved hypothetical protein TIGR00488
					8.500	6.000	19.9474	6.0000	
SGO_0450	1.587	4.981	0.0111	0.0443	6.500		14.1662		iojap-related protein
					5.500	4.500	12.9072	4.5000	
SGO_0454	0.166	7.715	0.0699	0.4277	21.000	54.000	45.7679	56.1983	conserved hypothetical protein TIGR01033
					28.000	42.500	65.7092	42.5000	
SGO_0455	-5.504	8.234	0.0133	0.0563	1.500	155.500	3.2691	161.8303	lipoprotein, putative
						136.000		136.0000	

Log$_2$ Ratios: -6 -4 -2 0 2 4 6

Show detected proteins only
Show all proteins
Filter by category: ABC Transporter

Proteins found: 769

Test	Cutoff
q-Value	.005
p-Value	

	Signif	Direction	Applies To
red	yes	+	ratios, bars
yellow	no	n/a	bars
green	yes	−	ratios, bars
pink	yes	+	*p*- , *q*-Values
light green	yes	−	*p*- , *q*-Values

Dot Plots Dot Plots

Streptococcus gordonii

Protein	SgFn vs Sg Log_2 Ratio	Log_2 Sum	*q*-Value	*p*-Value	Raw *SgFn*	Raw *Sg*	Normalized *SgFn*	Normalized *Sg*	Description
SGO_0456	1.256	5.488	0.0003	0.0003	7.500	7.000	16.3457	7.2850	ILL5; amino acid aminohydrolase
					6.500	6.000	15.2539	6.0000	
SGO_0457	-3.171	9.200	0.0001	0.0000	13.500	245.000	29.4222	254.9739	ABC transporter, substrate-binding protein SP0148
					12.500	274.500	29.3345	274.5000	
SGO_0458	-2.885	10.776	0.0004	0.0004	56.500	801.500	123.1373	834.1288	hlpA; lipoprotein
					37.500	708.500	88.0034	708.5000	
SGO_0459	0.112	5.970	0.1341	0.8978	6.500	10.000	14.1662	10.4071	succinyl-diaminopimelate desuccinylase
					7.500	20.500	17.6007	20.5000	
SGO_0460	-2.640	7.625	0.0007	0.0010	6.000	91.000	13.0765	94.7046	ABC transporter, ATP-binding protein SP0151
					6.000	75.500	14.0805	75.5000	
SGO_0468	1.460	7.214	0.0095	0.0367	31.000	12.000	67.5621	12.4885	hypothetical protein SGO_0468
					17.000	28.500	39.8949	28.5000	
SGO_0476	-1.320	4.817	0.0152	0.0670		12.000		12.4885	rhodanese family protein
					2.000	11.000	4.6935	11.0000	
SGO_0480	6.692	9.969	0.0405	0.2217	146.500		319.2853		hypothetical protein SGO_0480
					289.000	4.500	678.2127	4.5000	
SGO_0483	0.666	6.247	0.0052	0.0168	11.000	17.000	23.9736	17.6921	hypothetical protein SGO_0483
					9.500	12.000	22.2942	12.0000	
SGO_0484	-0.367	4.981	0.0680	0.4142	4.000	9.000	8.7177	9.3664	sensor histidine kinase
						13.500		13.5000	
SGO_0488	-2.214	6.635	0.0018	0.0041	2.000	39.500	4.3588	41.1080	ABC transporter, ATP-binding protein SP0483
					7.000	37.500	16.4273	37.5000	
SGO_0494	3.418	8.065	0.0010	0.0018	64.500	9.500	140.5727	9.8867	lemA; LemA-like protein
					44.500	13.000	104.4307	13.0000	

Log2 Ratios: -6 -4 -2 0 2 4 6

Show detected proteins only
Show all proteins
Filter by category:
ABC Transporter

Proteins found: 769

Test	Cutoff
q-Value	.005
p-Value	

	Signif	Direction	Applies To
red	yes	+	ratios, bars
yellow	no	n/a	bars
green	yes	−	ratios, bars
pink	yes	+	*p*- , *q*-Values
light green	yes	−	*p*- , *q*-Values

Dot Plots Dot Plots

Protein	SgFn vs Sg Log_2 Ratio	SgFn vs Sg Log_2 Sum	SgFn vs Sg *q*-Value	SgFn vs Sg *p*-Value	Raw *SgFn*	Raw *Sg*	Normalized *SgFn*	Normalized *Sg*	Description
SGO_0495	0.441	5.691	0.0257	0.1271	7.000	8.000	15.2560	8.3257	htpx; heat shock protein
					6.000	14.000	14.0805	14.0000	
SGO_0501	-1.705	5.727	0.0055	0.0183		22.500		23.4160	Uncharacterized ACR, COG1399
					3.000	22.500	7.0403	22.5000	
SGO_0502	-1.901	9.535	0.0003	0.0003	44.000	291.000	95.8946	302.8465	floL; flotillin-like protein
					27.000	280.000	63.3624	280.0000	
SGO_0503	1.705	11.551	0.0001	0.0001	545.500	350.000	1188.8747	364.2484	gnd; 6-phosphogluconate dehydrogenase, decarboxylating
					472.000	340.000	1107.6692	340.0000	
SGO_0505	-3.594	10.971	0.0001	0.0000	42.500	911.000	92.6254	948.0865	PTS system, IIBC component
					27.000	902.500	63.3624	902.5000	
SGO_0508	-0.432	6.099	0.0118	0.0489	8.500	24.500	18.5251	25.4974	nrdR; transcriptional regulator, NrdR family
						24.500		24.5000	
SGO_0510	1.340	5.852	0.0052	0.0170	11.500	6.500	25.0634	6.7646	dnaI; primosomal protein DnaI
					7.000	9.500	16.4273	9.5000	
SGO_0511	1.306	5.805	0.0113	0.0460	7.000	4.500	15.2560	4.6832	NADPH-flavin oxidoreductase -like protein
					10.000	12.500	23.4676	12.5000	
SGO_0512	0.201	7.827	0.0613	0.3659	30.000	61.000	65.3827	63.4833	GTP-binding protein engA
					23.500	43.000	55.1488	43.0000	
SGO_0513	0.668	4.254	0.0524	0.3005	4.000		8.7177		Snf2 family protein
					2.500	4.500	5.8669	4.5000	
SGO_0515	0.612	8.759	0.0031	0.0092	66.000	85.500	143.8419	88.9807	murC; UDP-N-acetylmuramate--alanine ligase
					50.500	82.000	118.5112	82.0000	
SGO_0518	-2.664	8.637	0.0001	0.0000	12.500	170.000	27.2428	176.9206	aminodeoxychorismate lyase-like protein
					11.500	167.000	26.9877	167.0000	

Log_2 Ratios: -6 -4 -2 0 2 4 6

Show detected proteins only
Show all proteins
Filter by category: ABC Transporter

Proteins found: 769

Test	Cutoff
***q*-Value**	**.005**
p-Value	

	Signif	Direction	Applies To
red	yes	+	ratios, bars
yellow	no	n/a	bars
green	yes	−	ratios, bars
light red	yes	+	*p*- , *q*-Values
light green	yes	−	*p*- , *q*-Values

Dot Plots Dot Plots

Protein	SgFn vs Sg Log_2 Ratio	Log_2 Sum	*q*-Value	*p*-Value	Raw *SgFn*	Raw *Sg*	Normalized *SgFn*	Normalized *Sg*	Description
SGO_0519	0.050	7.158	0.1070	0.6966	19.500	31.000	42.4987	32.2620	greA; transcription elongation factor greA
					13.000	37.500	30.5078	37.5000	
SGO_0521	-0.573	5.942	0.0557	0.3239	7.000	18.000	15.2560	18.7328	Membrane protein oxaA 2 precursor
						27.500		27.5000	
SGO_0523	2.159	6.704	0.0005	0.0007	21.500		46.8576		spoU rRNA Methylase family protein
					20.000	10.500	46.9351	10.5000	
SGO_0526	0.727	6.089	0.0277	0.1410	7.000	8.500	15.2560	8.8460	ilvB; acetolactate synthase, large subunit, biosynthetic type
					11.500	17.000	26.9877	17.0000	
SGO_0527	0.008	5.759	0.1306	0.8672	6.000	12.500	13.0765	13.0089	ilvN; acetolactate synthase, small subunit
					6.000	14.000	14.0805	14.0000	
SGO_0528	1.026	8.815	0.0020	0.0049	77.500	68.000	168.9052	70.7683	ilvC; ketol-acid reductoisomerase
					57.000	77.000	133.7651	77.0000	
SGO_0529	1.907	5.080	0.0065	0.0226	4.500	2.000	9.8074	2.0814	ilvA; threonine dehydratase
					7.000	5.500	16.4273	5.5000	
SGO_0530	-0.427	5.304	0.0251	0.1236	3.000	10.000	6.5383	10.4071	Cof family protein
					4.500	12.000	10.5604	12.0000	
SGO_0533	2.481	3.362			4.000	1.500	8.7177	1.5611	conserved hypothetical protein TIGR00150
SGO_0535	-2.494	6.747	0.0178	0.0800	4.000	42.500	8.7177	44.2302	putative transcriptional regulator LytR
						54.500		54.5000	
SGO_0537	1.242	6.570	0.0008	0.0013	16.000	11.500	34.8708	11.9682	HIT family protein
					13.500	16.500	31.6812	16.5000	
SGO_0538	-0.908	6.861	0.0075	0.0275	7.000	41.000	15.2560	42.6691	ABC transporter, ATP-binding protein SP0522
					11.000	32.500	25.8143	32.5000	

Log_2 Ratios: -6 -4 -2 0 2 4 6

Show detected proteins only
Show all proteins
Filter by category:
ABC Transporter

Proteins found: 769

Test	Cutoff
q-Value	.005
p-Value	

Color	Signif	Direction	Applies To
red	yes	+	ratios, bars
yellow	no	n/a	bars
green	yes	−	ratios, bars
pink	yes	+	*p*- , *q*-Values
light green	yes	−	*p*- , *q*-Values

Dot Plots Dot Plots

Protein	SgFn vs Sg Log$_2$ Ratio	Log$_2$ Sum	*q*-Value	*p*-Value	Raw *SgFn*	Raw *Sg*	Normalized *SgFn*	Normalized *Sg*	Description
SGO_0540	1.134	7.814	0.0003	0.0003	37.000	35.500	80.6386	36.9452	hypothetical protein SGO_0540
					31.500	33.500	73.9228	33.5000	
SGO_0541	-0.545	5.797	0.0064	0.0223	5.500	14.000	11.9868	14.5699	methyltransferase, putative
					4.500	18.500	10.5604	18.5000	
SGO_0543	-1.692	7.929	0.0001	0.0000	13.500	87.500	29.4222	91.0621	nusA; transcription termination factor NusA
					12.000	95.000	28.1611	95.0000	
SGO_0546	-0.307	8.766	0.0250	0.1222	40.500	132.500	88.2666	137.8940	infB; Translation initiation factor IF-2
					45.000	103.500	105.6041	103.5000	
SGO_0548	-4.413	7.683	0.0137	0.0590		105.000		109.2745	Na/Pi-cotransporter family protein
					2.000	91.500	4.6935	91.5000	
SGO_0549	-0.156	2.789			1.500	3.500	3.2691	3.6425	nagA; N-acetylglucosamine-6-phosphate deacetylase
SGO_0552	3.347	7.019	0.0005	0.0007	29.500	3.500	64.2929	3.6425	oxidoreductase, aldo/keto reductase family
					22.500	9.000	52.8020	9.0000	
SGO_0554	0.061	6.972	0.1305	0.8645	12.500	18.000	27.2428	18.7328	hsdR; type I site-specific deoxyribonuclease
					14.500	45.500	34.0280	45.5000	
SGO_0558	0.494	3.590							hypothetical protein SGO_0558
					3.000	5.000	7.0403	5.0000	
SGO_0560	-1.604	4.640	0.0073	0.0263		10.000		10.4071	hsdM; type I restriction-modification system, M subunit
					1.500	11.000	3.5201	11.0000	
SGO_0565	2.010	9.293	0.0008	0.0013	128.000	56.500	278.9660	58.8001	adhA; alcohol dehydrogenase
					95.500	65.500	224.1153	65.5000	
SGO_0566	0.575	8.380	0.0109	0.0434	40.500	53.000	88.2666	55.1576	sgc; serine protease challisin
					47.000	79.500	110.2976	79.5000	

Log$_2$ Ratios: -6 -4 -2 0 2 4 6

Show detected proteins only
Show all proteins
Filter by category:
ABC Transporter

Proteins found: 769

Test	Cutoff
q-Value	.005
p-Value	

Colour	Signif	Direction	Applies To
red	yes	+	ratios, bars
yellow	no	n/a	bars
green	yes	−	ratios, bars
pink	yes	+	*p*- , *q*-Values
light green	yes	−	*p*- , *q*-Values

Dot Plots Dot Plots

Protein	SgFn vs Sg Log_2 Ratio	Log_2 Sum	*q*-Value	*p*-Value	Raw *SgFn*	Raw *Sg*	Normalized *SgFn*	Normalized *Sg*	Description
SGO_0568	-1.401	6.686	0.0013	0.0025	8.500	34.500	18.5251	35.9045	glyQ; glycyl-tRNA synthetase, alpha subunit
					4.500	38.000	10.5604	38.0000	
SGO_0569	0.390	8.405	0.0137	0.0586	38.000	70.000	82.8180	72.8497	glyS; glycyl-tRNA synthetase, beta subunit
					47.000	73.000	110.2976	73.0000	
SGO_0573	0.013	6.729	0.1339	0.8953	15.500	20.500	33.7810	21.3345	mraW; S-adenosyl-methyltransferase MraW
					8.500	31.000	19.9474	31.0000	
SGO_0577	-0.084	6.915	0.1306	0.8687	17.500	23.500	38.1399	24.4567	ATP-dependent RNA helicase
					9.000	37.000	21.1208	37.0000	
SGO_0579	-3.048	6.975	0.0001	0.0000	3.000	55.500	6.5383	57.7594	amino acid ABC transporter ATP binding protein
					3.000	54.500	7.0403	54.5000	
SGO_0581	-0.401	5.554	0.0381	0.2059	3.500	11.000	7.6280	11.4478	trxB; thioredoxin-disulfide reductase
					5.500	15.000	12.9072	15.0000	
SGO_0582	3.236	8.876	0.0000	0.0000	96.000	18.000	209.2245	18.7328	nicotinate phosphoribosyltransferase, putative
					91.500	27.000	214.7283	27.0000	
SGO_0583	2.312	6.694	0.0022	0.0058	23.000	5.500	50.1267	5.7239	nadE; NAD+ synthetase
					15.000	12.500	35.2014	12.5000	
SGO_0585	2.877	8.679	0.0002	0.0002	77.500	19.500	168.9052	20.2938	pepC; aminopeptidase C
					81.500	29.500	191.2607	29.5000	
SGO_0586	-1.742	8.755	0.0002	0.0001	20.500	154.500	44.6782	160.7896	pbp1a; penicillin-binding protein 1A
					23.500	171.500	55.1488	171.5000	
SGO_0589	1.676	7.845	0.0038	0.0113	49.000	24.000	106.7917	24.9770	methylase
					29.500	29.000	69.2293	29.0000	
SGO_0590	0.366	6.294	0.0250	0.1225	11.000	13.500	23.9736	14.0496	Methyltransferase
					8.500	20.500	19.9474	20.5000	

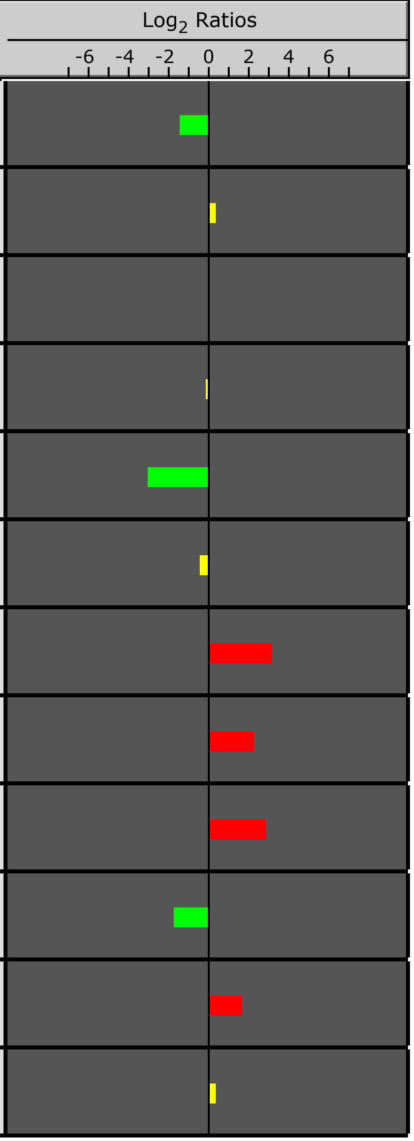

Show detected proteins only
Show all proteins
Filter by category:
ABC Transporter

Proteins found: 769

Test	Cutoff
q-Value	.005
p-Value	

	Signif	Direction	Applies To
red	yes	+	ratios, bars
yellow	no	n/a	bars
green	yes	−	ratios, bars
pink	yes	+	*p*- , *q*-Values
light green	yes	−	*p*- , *q*-Values

Dot Plots Dot Plots

Hendrickson *et al.*

Protein	SgFn vs Sg				Raw		Normalized		Description
	Log_2 Ratio	Log_2 Sum	*q*-Value	*p*-Value	*SgFn*	*Sg*	*SgFn*	*Sg*	
SGO_0591	-2.414	8.988	0.0000	0.0000	18.500	208.000	40.3193	216.4676	hypothetical protein SGO_0591
					17.000	211.000	39.8949	211.0000	
SGO_0592	0.770	5.593	0.0015	0.0034	7.500	8.000	16.3457	8.3257	luxS; autoinducer-2 production protein LuxS
					6.000	9.500	14.0805	9.5000	
SGO_0593	-3.236	8.876	0.0001	0.0000	9.000	196.000	19.6148	203.9791	HD/KH domain protein
					11.000	220.500	25.8143	220.5000	
SGO_0594	0.562	6.622	0.0052	0.0169	14.500	21.500	31.6016	22.3753	gmk; Guanylate kinase (GMP kinase)
					11.500	17.500	26.9877	17.5000	
SGO_0595	-1.365	6.899	0.0004	0.0006	9.000	40.500	19.6148	42.1487	DNA-directed RNA polymerase, omega subunit
					6.000	43.500	14.0805	43.5000	
SGO_0596	1.535	4.408	0.0093	0.0359	4.000	3.000	8.7177	3.1221	priA; primosomal protein N''
					4.000		9.3870		
SGO_0597	2.095	6.308	0.0012	0.0024	13.000	5.000	28.3325	5.2035	fmt; methionyl-tRNA formyltransferase
					15.000	10.500	35.2014	10.5000	
SGO_0598	0.617	4.435	0.0067	0.0238		6.000		6.2443	sun; sun protein
					4.000	6.000	9.3870	6.0000	
SGO_0599	1.085	7.939	0.0005	0.0006	40.500	39.000	88.2666	40.5877	phosphoprotein phosphatase
					33.500	38.000	78.6164	38.0000	
SGO_0600	-2.104	8.114	0.0001	0.0001	14.000	105.000	30.5119	109.2745	serine/threonine protein kinase
					9.500	115.000	22.2942	115.0000	
SGO_0603	0.459	5.665	0.0023	0.0062	7.000	9.500	15.2560	9.8867	response regulator
					6.000	11.500	14.0805	11.5000	
SGO_0604	1.785	8.952	0.0001	0.0001	87.000	47.500	189.6097	49.4337	hydrolase, haloacid dehalogenase family/peptidyl-prolyl cis-trans isomerase, cyclophilin type
					82.500	62.500	193.6074	62.5000	

Log_2 Ratios: -6 -4 -2 0 2 4 6

Show detected proteins only
Show all proteins
Filter by category:
ABC Transporter

Proteins found: 769

Test	Cutoff
q-Value	.005
p-Value	

	Signif	Direction	Applies To
red	yes	+	ratios, bars
yellow	no	n/a	bars
green	yes	−	ratios, bars
pink	yes	+	*p*- , *q*-Values
light green	yes	−	*p*- , *q*-Values

Dot Plots Dot Plots

Hendrickson *et al.*

Protein	SgFn vs Sg Log_2 Ratio	Log_2 Sum	*q*-Value	*p*-Value	Raw SgFn	Raw Sg	Normalized SgFn	Normalized Sg	Description
SGO_0606	-0.606	9.178	0.0004	0.0005	52.000	162.000	113.3299	168.5950	cysK; cysteine synthase A
					49.500	181.000	116.1645	181.0000	
SGO_0610	1.162	8.870	0.0022	0.0058	84.000	75.000	183.0714	78.0532	ribosomal subunit interface protein
					60.000	66.000	140.8054	66.0000	
SGO_0626	-1.694	5.610	0.0047	0.0149	3.000	20.000	6.5383	20.8142	recX; Regulatory protein recX
						21.500		21.5000	
SGO_0631	0.803	3.099			2.500	3.000	5.4486	3.1221	alpha-glycerophosphate oxidase
SGO_0635	1.553	3.976							hypothetical protein SGO_0635
					5.000	4.000	11.7338	4.0000	
SGO_0639	0.072	8.074	0.0676	0.4110	29.000	65.500	63.2032	68.1665	valS; valyl-tRNA synthetase
					32.000	63.000	75.0962	63.0000	
SGO_0641	0.022	6.939	0.1313	0.8744	16.000	32.500	34.8708	33.8231	ATPase, histidine kinase-, DNA gyrase B-, and HSP90-like domain protein protein
					11.500	27.000	26.9877	27.0000	
SGO_0642	-1.789	6.945	0.0020	0.0050	4.500	39.500	9.8074	41.1080	hypothetical protein SGO_0642
					8.000	53.500	18.7741	53.5000	
SGO_0643	0.835	5.351	0.0028	0.0078	5.500	6.000	11.9868	6.2443	cytosine-specific methyltransferase
					6.000	8.500	14.0805	8.5000	
SGO_0644	-0.099	5.966	0.0866	0.5505	8.000	16.000	17.4354	16.6514	hypothetical protein SGO_0644
					5.500	15.500	12.9072	15.5000	
SGO_0652	-3.825	7.262	0.0001	0.0000	2.500	71.000	5.4486	73.8904	hypothetical protein SGO_0652
					2.000	69.500	4.6935	69.5000	
SGO_0654	-3.313	7.518	0.0183	0.0826	4.000	74.000	8.7177	77.0125	radical SAM enzyme, Cfr family
						97.500		97.5000	

Log_2 Ratios: -6 -4 -2 0 2 4 6

Show detected proteins only
Show all proteins
Filter by category:
ABC Transporter

Proteins found: 769

Test	Cutoff
q-Value	.005
p-Value	

Colour	Signif	Direction	Applies To
red	yes	+	ratios, bars
yellow	no	n/a	bars
green	yes	−	ratios, bars
pink	yes	+	*p*- , *q*-Values
light green	yes	−	*p*- , *q*-Values

Dot Plots Dot Plots

Protein	SgFn vs Sg: Log_2 Ratio	Log_2 Sum	*q*-Value	*p*-Value	Raw: *SgFn*	Raw: *Sg*	Normalized: *SgFn*	Normalized: *Sg*	Description
SGO_0656	0.786	6.720	0.0347	0.1846	8.000	17.000	17.4354	17.6921	trpB-2; tryptophan synthase, beta subunit
					22.500	17.500	52.8020	17.5000	
SGO_0662	1.838	4.612	0.0045	0.0139	5.000		10.8971		trpB-1; tryptophan synthase, beta subunit
					4.500	3.000	10.5604	3.0000	
SGO_0665	-0.806	10.143	0.0079	0.0292	107.500	405.500	234.2879	422.0077	non-heme iron-containing ferritin
					75.000	298.500	176.0068	298.5000	
SGO_0669	0.790	8.186	0.0019	0.0045	38.500	49.000	83.9077	50.9948	typA; GTP-binding protein TypA
					43.000	55.500	100.9105	55.5000	
SGO_0671	1.635	7.976	0.0003	0.0004	46.500	30.500	101.3431	31.7416	murD; UDP-N-acetylmuramoylalanine--D-glutamate ligase
					38.000	29.500	89.1768	29.5000	
SGO_0672	-0.119	7.180	0.0814	0.5114	14.000	42.500	30.5119	44.2302	murG; undecaprenyl-PP-MurNAc-pentapeptide-UDPGlcNAc GlcNAc transferase
					16.500	31.500	38.7215	31.5000	
SGO_0673	-1.650	6.736	0.0020	0.0049	4.500	33.500	9.8074	34.8638	DivIB; cell division protein DivIB
					7.000	45.500	16.4273	45.5000	
SGO_0674	0.569	9.226	0.0003	0.0004	84.500	114.000	184.1612	118.6409	ftsA; cell division protein FtsA
					74.000	122.500	173.6600	122.5000	
SGO_0675	0.657	9.878	0.0130	0.0548	155.500	145.500	338.9001	151.4232	ftsZ; cell division protein FtsZ
					101.000	213.500	237.0224	213.5000	
SGO_0676	-0.268	5.946	0.0808	0.5048	4.500	13.500	9.8074	14.0496	conserved hypothetical protein TIGR00044
					8.000	19.000	18.7741	19.0000	
SGO_0677	-0.741	8.345	0.0045	0.0138	26.500	111.000	57.7547	115.5188	ylmF protein
					27.000	88.500	63.3624	88.5000	
SGO_0680	0.018	8.598	0.1210	0.7945	55.000	93.500	119.8682	97.3063	cell division protein DivIVA
					33.000	93.000	77.4430	93.0000	

Log_2 Ratios: -6 -4 -2 0 2 4 6

Show detected proteins only
Show all proteins
Filter by category: ABC Transporter

Proteins found: 769

Test	Cutoff
q-Value	.005
p-Value	

Color	Signif	Direction	Applies To
red	yes	+	ratios, bars
yellow	no	n/a	bars
green	yes	–	ratios, bars
pink	yes	+	*p*- , *q*-Values
light green	yes	–	*p*- , *q*-Values

Dot Plots Dot Plots

Protein	SgFn vs Sg Log_2 Ratio	Log_2 Sum	*q*-Value	*p*-Value	Raw *SgFn*	Raw *Sg*	Normalized *SgFn*	Normalized *Sg*	Description
SGO_0681	-0.268	8.346	0.0217	0.1021	33.500	74.500	73.0106	77.5329	ileS; isoleucyl-tRNA synthetase
					31.500	101.000	73.9228	101.0000	
SGO_0684	0.170	8.754	0.0141	0.0608	49.000	97.000	106.7917	100.9488	hypothetical protein SGO_0684
					52.000	102.000	122.0314	102.0000	
SGO_0685	0.646	3.529							MutT/nudix family protein
					3.000	4.500	7.0403	4.5000	
SGO_0688	0.228	8.363	0.0550	0.3181	31.000	73.500	67.5621	76.4922	ATP dependent Clp protease, ATP-binding subunit, ClpE
					48.000	72.500	112.6443	72.5000	
SGO_0690	1.809	5.251	0.0084	0.0320	5.000	2.000	10.8971	2.0814	folD; methenyltetrahydrofolate cyclohydrolase
					7.500	7.500	17.6007	7.5000	
SGO_0693	0.753	6.673	0.0237	0.1130	10.000	20.000	21.7942	20.8142	xseA; exodeoxyribonuclease VII, large subunit
					18.500	16.000	43.4150	16.0000	
SGO_0698	1.171	6.257	0.0039	0.0118	11.000	8.000	23.9736	8.3257	recN; DNA repair protein RecN
					12.000	16.000	28.1611	16.0000	
SGO_0699	1.280	5.382	0.0018	0.0043	6.000	5.000	13.0765	5.2035	Serine/threonine protein phosphatase
					7.000	7.000	16.4273	7.0000	
SGO_0700	-0.380	5.870	0.0479	0.2708	7.500	18.500	16.3457	19.2531	DegV family protein
					4.000	13.500	9.3870	13.5000	
SGO_0701	0.805	12.498	0.0064	0.0221	705.500	1072.000	1537.5822	1115.6407	hup; DNA-binding histone-like protein HU
					919.000	974.000	2156.6696	974.0000	
SGO_0704	1.491	10.896	0.0009	0.0016	341.500	193.000	744.2726	200.8570	gpmA; 2,3-bisphosphoglycerate-dependent phosphoglycerate mutase
					278.500	306.500	653.5718	306.5000	
SGO_0706	2.946	6.245	0.0001	0.0001	15.000	3.000	32.6913	3.1221	phoH-like protein
					14.500	6.000	34.0280	6.0000	

Log_2 Ratios: -6 -4 -2 0 2 4 6

Show detected proteins only
Show all proteins
Filter by category:
ABC Transporter

Proteins found: 769

Test	Cutoff
***q*-Value**	**.005**
p-Value	

	Signif	Direction	Applies To
(red)	yes	+	ratios, bars
(yellow)	no	n/a	bars
(green)	yes	−	ratios, bars
(pink)	yes	+	*p*- , *q*-Values
(light green)	yes	−	*p*- , *q*-Values

Dot Plots Dot Plots

Hendrickson *et al.*

Streptococcus gordonii

Hackett Laboratory UW

Protein	SgFn vs Sg Log_2 Ratio	Log_2 Sum	*q*-Value	*p*-Value	Raw *SgFn*	Raw *Sg*	Normalized *SgFn*	Normalized *Sg*	Description	Log_2 Ratios (-6 -4 -2 0 2 4 6)
SGO_0707	0.131	9.804	0.0405	0.2218	118.500	206.000	258.2615	214.3862	LPXTG cell wall surface protein	
					89.500	211.000	210.0347	211.0000		
SGO_0708	3.041	11.420	0.0000	0.0000	564.500	137.000	1230.2837	142.5772	ald; alanine dehydrogenase	
					516.500	154.500	1212.0999	154.5000		
SGO_0711	0.408	4.363				8.500		8.8460	conserved hypothetical protein TIGR00043	
					5.000		11.7338			
SGO_0713	1.330	7.661	0.0001	0.0001	32.500	29.500	70.8312	30.7009	sgg; GTP-binding protein Era	
					31.500	27.000	73.9228	27.0000		
SGO_0719	-0.545	6.065	0.0077	0.0282	5.500	17.000	11.9868	17.6921	rnr; ribonuclease R	
					6.500	22.000	15.2539	22.0000		
SGO_0721	0.551	5.573	0.0013	0.0026	6.500	8.500	14.1662	8.8460	abpB-like dipeptidase lipoprotein	
					6.000	10.500	14.0805	10.5000		
SGO_0722	-0.229	5.726	0.0706	0.4330	5.000	7.500	10.8971	7.8053	tehB; tellurite resistance protein TehB	
					5.000	22.500	11.7338	22.5000		
SGO_0724	-1.628	6.596	0.0007	0.0011	4.500	32.500	9.8074	33.8231	dipeptidase	
					6.000	39.000	14.0805	39.0000		
SGO_0736	0.917	6.466	0.0009	0.0015	12.500	16.000	27.2428	16.6514	hprK; HPr(Ser) kinase/phosphatase	
					13.000	14.000	30.5078	14.0000		
SGO_0739	-0.567	4.807	0.0334	0.1759		11.000		11.4478	hypothetical protein SGO_0739	
					3.000	9.500	7.0403	9.5000		
SGO_0742	0.753	6.494	0.0004	0.0005	13.000	15.000	28.3325	15.6106	peptidase, U32 family	
					12.000	18.000	28.1611	18.0000		
SGO_0743	-2.254	7.696	0.0001	0.0000	9.000	80.000	19.6148	83.2568	peptidase, U32 family	
					7.000	88.000	16.4273	88.0000		

Show detected proteins only
Show all proteins
Filter by category:
ABC Transporter

Proteins found: 769

Test	Cutoff
***q*-Value**	**.005**
p-Value	

	Signif	Direction	Applies To
red	yes	+	ratios, bars
yellow	no	n/a	bars
green	yes	−	ratios, bars
pink	yes	+	*p*- , *q*-Values
light green	yes	−	*p*- , *q*-Values

Dot Plots Dot Plots

Hendrickson *et al.*

Protein	SgFn vs Sg Log_2 Ratio	Log_2 Sum	*q*-Value	*p*-Value	Raw *SgFn*	Raw *Sg*	Normalized *SgFn*	Normalized *Sg*	Description
SGO_0745	1.409	5.643	0.0205	0.0948		8.000		8.3257	hypothetical protein SGO_0745
					12.000	13.500	28.1611	13.5000	
SGO_0749	2.313	7.691	0.0032	0.0094	30.500	13.000	66.4724	13.5292	glutathione reductase
					45.000	21.000	105.6041	21.0000	
SGO_0750	-5.485	10.400	0.0005	0.0007	6.000	708.000	13.0765	736.8224	efflux transporter, RND family, MFP subunit subfamily
					7.000	584.500	16.4273	584.5000	
SGO_0751	-3.035	7.519	0.0002	0.0002	3.500	82.500	7.6280	85.8585	ABC transporter, ATP-binding protein SP0786
					5.500	77.000	12.9072	77.0000	
SGO_0753	-0.738	8.826	0.0084	0.0316	50.000	144.000	108.9711	149.8622	lysS; lysyl-tRNA synthetase
					27.500	130.500	64.5358	130.5000	
SGO_0755	0.217	5.320	0.0517	0.2947	4.000	8.000	8.7177	8.3257	regulatory protein
					5.500	10.000	12.9072	10.0000	
SGO_0760	0.523	9.217	0.0019	0.0046	75.000	117.000	163.4567	121.7630	ppc; phosphoenolpyruvate carboxylase
					80.000	122.000	187.7405	122.0000	
SGO_0761	0.986	13.763	0.0007	0.0011	1976.000	2223.000	4306.5379	2313.4975	tuf; translation elongation factor Tu
					2103.500	2341.000	4936.4031	2341.0000	
SGO_0762	-0.128	10.618	0.0596	0.3510	150.500	384.500	328.0030	400.1528	tpiA; triosephosphate isomerase
					181.500	417.000	425.9364	417.0000	
SGO_0763	-0.883	8.224	0.0007	0.0010	24.500	87.500	53.3958	91.0621	murA-1; UDP-N-acetylglucosamine 1-carboxyvinyltransferase
					22.000	103.000	51.6287	103.0000	
SGO_0767	0.649	5.851	0.0207	0.0965	10.500	11.000	22.8839	11.4478	transport protein
					5.500	10.500	12.9072	10.5000	
SGO_0771	1.357	8.365	0.0016	0.0037	58.500	56.000	127.4962	58.2797	pepq; proline dipeptidase
					46.000	36.000	107.9508	36.0000	

Log_2 Ratios: -6 -4 -2 0 2 4 6

Show detected proteins only
Show all proteins
Filter by category:
ABC Transporter

Proteins found: 769

Test	Cutoff
***q*-Value**	**.005**
p-Value	

	Signif	Direction	Applies To
(red)	yes	+	ratios, bars
(yellow)	no	n/a	bars
(green)	yes	−	ratios, bars
(pink)	yes	+	*p*- , *q*-Values
(light green)	yes	−	*p*- , *q*-Values

Dot Plots Dot Plots

Protein	SgFn vs Sg Log$_2$ Ratio	Log$_2$ Sum	*q*-Value	*p*-Value	Raw *SgFn*	Raw *Sg*	Normalized *SgFn*	Normalized *Sg*	Description
SGO_0773	-2.320	10.237	0.0002	0.0001	42.500	508.000	92.6254	528.6805	ccpA; catabolite control protein A
					46.500	476.500	109.1242	476.5000	
SGO_0774	-1.831	7.074	0.0009	0.0018	6.500	56.500	14.1662	58.8001	glycosyl transferase, group 1 family protein
					6.500	46.500	15.2539	46.5000	
SGO_0775	-0.533	6.511	0.0127	0.0537	7.000	28.500	15.2560	29.6602	glycosyl transferase, group 1
					9.500	24.000	22.2942	24.0000	
SGO_0778	-0.632	9.020	0.0003	0.0002	45.500	148.000	99.1637	154.0250	thrS; threonyl-tRNA synthetase
					44.500	161.500	104.4307	161.5000	
SGO_0779	1.732	7.769	0.0006	0.0008	40.500	19.500	88.2666	20.2938	response regulator
					33.500	31.000	78.6164	31.0000	
SGO_0780	-1.119	5.893	0.0058	0.0196	5.500	22.500	11.9868	23.4160	histidine kinase
					3.000	17.000	7.0403	17.0000	
SGO_0781	-1.851	5.277	0.0213	0.0999		14.500		15.0903	vicX; Zn-dependent hydrolase (beta-lactamase superfamily)
					2.000	19.000	4.6935	19.0000	
SGO_0784	-1.644	8.363	0.0015	0.0033	14.500	105.500	31.6016	109.7949	smc; chromosome segregation protein SMC
					21.000	138.500	49.2819	138.5000	
SGO_0785	-0.328	4.940	0.0462	0.2608	4.000	11.500	8.7177	11.9682	Cof family protein
						10.000		10.0000	
SGO_0786	-1.050	4.921	0.0257	0.1273		10.500		10.9275	Cof family protein
					2.500	13.500	5.8669	13.5000	
SGO_0787	-0.721	7.421	0.0055	0.0182	17.500	56.000	38.1399	58.2797	ftsY; cell division protein FtsY
					11.500	48.000	26.9877	48.0000	
SGO_0788	2.261	8.231	0.0012	0.0024	49.000	27.000	106.7917	28.0992	zwf; glucose-6-phosphate 1-dehydrogenase
					60.500	23.500	141.9788	23.5000	

Log$_2$ Ratios: -6 -4 -2 0 2 4 6

Show detected proteins only
Show all proteins
Filter by category:
ABC Transporter

Proteins found: 769

Test	Cutoff
***q*-Value**	**.005**
p-Value	

	Signif	Direction	Applies To
red	yes	+	ratios, bars
yellow	no	n/a	bars
green	yes	−	ratios, bars
pink	yes	+	*p*- , *q*-Values
light green	yes	−	*p*- , *q*-Values

Dot Plots Dot Plots

Hendrickson *et al.*

Protein	SgFn vs Sg Log2 Ratio	Log2 Sum	*q*-Value	*p*-Value	Raw *SgFn*	Raw *Sg*	Normalized *SgFn*	Normalized *Sg*	Description
SGO_0792	-1.830	6.869	0.0014	0.0031	7.000	38.000	15.2560	39.5470	hypothetical protein SGO_0792
					4.500	51.500	10.5604	51.5000	
SGO_0794	0.304	7.978	0.0206	0.0955	28.000	50.000	61.0238	52.0355	metallo-beta-lactamase family protein
					33.500	60.500	78.6164	60.5000	
SGO_0795	2.063	5.813	0.0022	0.0058	8.500	5.500	18.5251	5.7239	tributyrin esterase
					11.500	5.000	26.9877	5.0000	
SGO_0797	-0.613	4.048			3.000		6.5383		RocB protein, putative
						10.000		10.0000	
SGO_0798	-1.060	7.828	0.0009	0.0017	16.500	80.000	35.9605	83.2568	ABC transporter, ATP-binding protein SP1381
					16.000	70.500	37.5481	70.5000	
SGO_0800	-2.065	6.313	0.0072	0.0261	3.500	21.000	7.6280	21.8549	polysaccharide deacetylase family protein
					3.000	43.000	7.0403	43.0000	
SGO_0801	0.572	8.591	0.0014	0.0028	54.500	80.500	118.7785	83.7771	hom; homoserine dehydrogenase
					47.500	71.500	111.4710	71.5000	
SGO_0802	0.760	6.777	0.0003	0.0003	16.000	18.500	34.8708	19.2531	thrB; homoserine kinase
					14.500	21.500	34.0280	21.5000	
SGO_0804	0.475	5.314	0.0954	0.6141	4.000	4.000	8.7177	4.1628	murB; UDP-N-acetylenolpyruvoylglucosamine reductase
					5.500	14.000	12.9072	14.0000	
SGO_0805	-1.847	6.699	0.0005	0.0007	4.500	42.000	9.8074	43.7098	potA; spermidine/putrescine ABC transporter, ATP-binding subunit
					5.500	37.500	12.9072	37.5000	
SGO_0808	-3.175	6.779	0.0410	0.2259	2.500	33.500	5.4486	34.8638	potD; spermidine/putrescine ABC transporter
						69.500		69.5000	
SGO_0812	1.605	6.051	0.0014	0.0029	10.000	8.500	21.7942	8.8460	hypothetical protein SGO_0812
					12.000	7.500	28.1611	7.5000	

Log2 Ratios

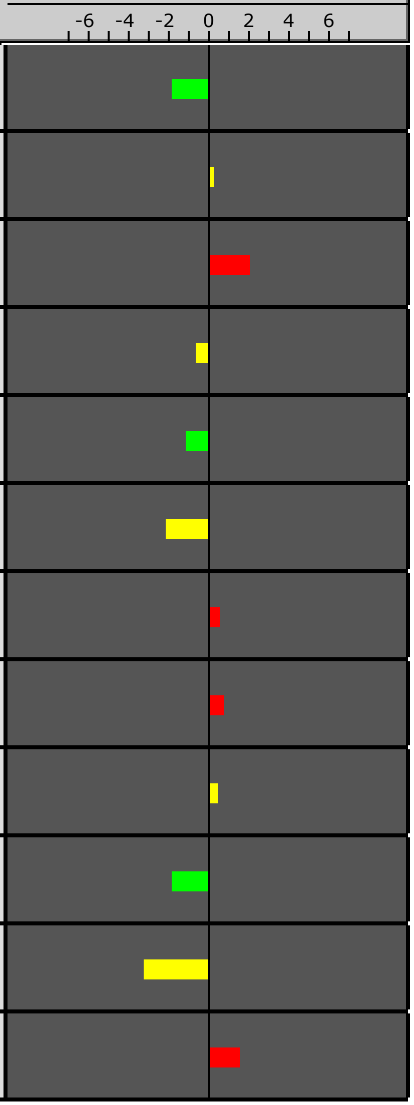

Show detected proteins only
Show all proteins
Filter by category:
ABC Transporter

Proteins found: 769

Test	Cutoff
***q*-Value**	**.005**
p-Value	

	Signif	Direction	Applies To
red	yes	+	ratios, bars
yellow	no	n/a	bars
green	yes	–	ratios, bars
light red	yes	+	*p*- , *q*-Values
light green	yes	–	*p*- , *q*-Values

Dot Plots Dot Plots

Protein	SgFn vs Sg Log$_2$ Ratio	Log$_2$ Sum	*q*-Value	*p*-Value	Raw *SgFn*	Raw *Sg*	Normalized *SgFn*	Normalized *Sg*	Description
SGO_0813	0.162	3.519	0.0963	0.6216	1.500		3.2691		fructose-bisphosphatase
					2.000	3.500	4.6935	3.5000	
SGO_0815	-1.223	7.201	0.0008	0.0014	9.000	46.000	19.6148	47.8726	thiI; thiamine biosynthesis protein ThiI
					10.500	55.000	24.6409	55.0000	
SGO_0818	-0.991	7.586	0.0392	0.2131	17.500	87.000	38.1399	90.5417	rplU; ribosomal protein L21
						63.500		63.5000	
SGO_0820	-0.660	7.245	0.0675	0.4089		72.500		75.4514	rpmA; ribosomal protein L27
					15.000	41.000	35.2014	41.0000	
SGO_0823	0.742	4.129	0.0368	0.1974	3.500		7.6280		Cof family protein
					2.500	4.000	5.8669	4.0000	
SGO_0824	0.590	7.350	0.0082	0.0310	22.500	25.000	49.0370	26.0177	lepA; GTP-binding protein LepA
					20.500	40.000	48.1085	40.0000	
SGO_0825	-2.331	6.193	0.0250	0.1222	3.000	27.000	6.5383	28.0992	lipoprotein, putative
						38.500		38.5000	
SGO_0830	1.409	3.045	0.0116	0.0479		1.500		1.5611	uvrC; excinuclease ABC, C subunit
					2.000	2.000	4.6935	2.0000	
SGO_0832	-3.238	6.446	0.0184	0.0838	2.000	44.500	4.3588	46.3116	hypothetical protein SGO_0832
						36.500		36.5000	
SGO_0834	-1.079	4.671	0.0550	0.3178		7.000		7.2850	hypothetical protein SGO_0834
					2.000	13.500	4.6935	13.5000	
SGO_0835	2.378	8.920	0.0001	0.0000	93.000	32.500	202.6862	33.8231	nitroreductase
					86.500	45.000	202.9945	45.0000	
SGO_0836	1.291	9.572	0.0004	0.0004	130.500	99.000	284.4146	103.0303	pepV; dipeptidase PepV
					109.000	118.000	255.7965	118.0000	

Log$_2$ Ratios: -6 -4 -2 0 2 4 6

Show detected proteins only
Show all proteins
Filter by category:
ABC Transporter

Proteins found: 769

Test	Cutoff
q-Value	.005
p-Value	

	Signif	Direction	Applies To
red	yes	+	ratios, bars
yellow	no	n/a	bars
green	yes	−	ratios, bars
pink	yes	+	*p*- , *q*-Values
light green	yes	−	*p*- , *q*-Values

Dot Plots Dot Plots

Hendrickson *et al.*

Protein	SgFn vs Sg Log_2 Ratio	Log_2 Sum	*q*-Value	*p*-Value	Raw *SgFn*	Raw *Sg*	Normalized *SgFn*	Normalized *Sg*	Description
SGO_0841	4.736	7.573	0.0111	0.0444	40.000		87.1769		oxidoreductase
					42.500	3.500	99.7372	3.5000	
SGO_0842	2.744	6.552	0.0028	0.0078	22.500	3.500	49.0370	3.6425	rhodanese family protein
					13.500	9.500	31.6812	9.5000	
SGO_0848	-0.727	7.350	0.0071	0.0254	18.000	48.500	39.2296	50.4744	rpmE; ribosomal protein L31
					10.000	50.000	23.4676	50.0000	
SGO_0849	0.752	6.415	0.0126	0.0528	15.000	13.000	32.6913	13.5292	DHH subfamily 1 protein
					9.000	18.000	21.1208	18.0000	
SGO_0850	0.386	6.736	0.0152	0.0673	16.000	22.500	34.8708	23.4160	flavodoxin
					11.000	22.500	25.8143	22.5000	
SGO_0851	1.157	4.915	0.0416	0.2304	7.000	10.000	15.2560	10.4071	putative permease
						4.500		4.5000	
SGO_0852	0.346	5.338	0.0035	0.0103	5.000	8.000	10.8971	8.3257	TPR domain protein
					5.000	9.500	11.7338	9.5000	
SGO_0853	-0.298	5.096	0.0590	0.3462	3.500	13.000	7.6280	13.5292	budA; alpha-acetolactate decarboxylase
					3.000	6.000	7.0403	6.0000	
SGO_0854	-2.737	9.701	0.0002	0.0001	33.500	336.500	73.0106	350.1988	cshA; surface-associated protein CshA
					17.000	369.500	39.8949	369.5000	
SGO_0855	-0.735	5.591	0.0050	0.0161	3.500	13.000	7.6280	13.5292	fbpA; fibronectin-binding protein A
					4.500	16.500	10.5604	16.5000	
SGO_0856	-3.308	8.206	0.0001	0.0000	5.500	124.500	11.9868	129.5683	ABC transporter, substrate binding protein
					6.500	138.500	15.2539	138.5000	
SGO_0859	0.405	7.841	0.0093	0.0357	29.000	54.500	63.2032	56.7187	pheS; phenylalanyl-tRNA synthetase, alpha subunit
					28.500	42.500	66.8826	42.5000	

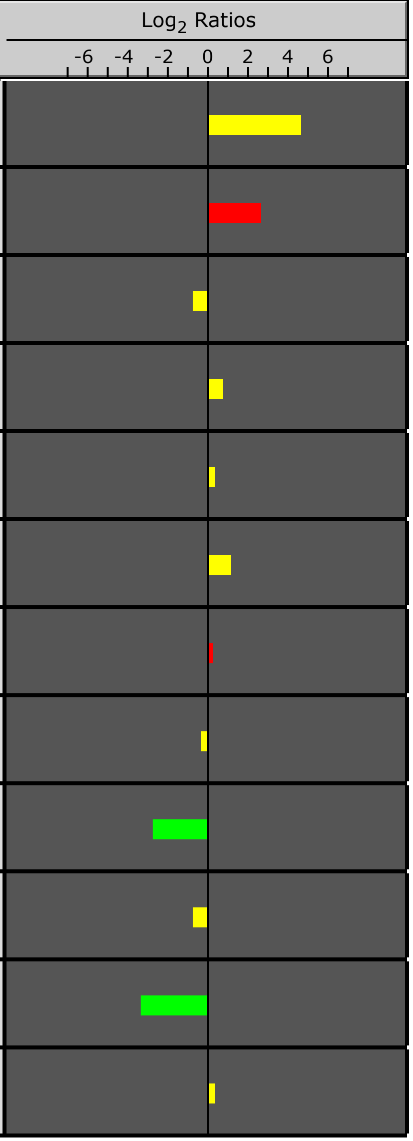

Show detected proteins only
Show all proteins
Filter by category:
ABC Transporter

Proteins found: 769

Test	Cutoff
q-Value	.005
p-Value	

	Signif	Direction	Applies To
red	yes	+	ratios, bars
yellow	no	n/a	bars
green	yes	−	ratios, bars
pink	yes	+	*p*- , *q*-Values
light green	yes	−	*p*- , *q*-Values

Dot Plots Dot Plots

Protein	SgFn vs Sg: Log_2 Ratio	Log_2 Sum	*q*-Value	*p*-Value	Raw: *SgFn*	Raw: *Sg*	Normalized: *SgFn*	Normalized: *Sg*	Description
SGO_0861	-0.152	8.497	0.0448	0.2521	35.500	99.000	77.3695	103.0303	pheT; phenylalanyl-tRNA synthetase, beta subunit
					40.000	87.000	93.8703	87.0000	
SGO_0885	0.700	6.064	0.0149	0.0652	11.500	10.000	25.0634	10.4071	cobyric acid synthase
					7.000	15.000	16.4273	15.0000	
SGO_0886	0.806	5.586	0.0240	0.1154	7.000	5.000	15.2560	5.2035	Mur ligase family protein
					6.000	13.500	14.0805	13.5000	
SGO_0889	-0.553	6.939	0.0003	0.0003	11.500	36.000	25.0634	37.4655	glmM; phosphoglucosamine mutase
					10.500	35.500	24.6409	35.5000	
SGO_0890	-1.796	8.439	0.0006	0.0010	24.000	126.000	52.3061	131.1294	LPXTG cell wall surface protein, collagen binding domain
					12.000	135.500	28.1611	135.5000	
SGO_0893	0.773	7.832	0.0005	0.0008	33.000	44.000	71.9209	45.7912	GTP-binding protein
					30.500	38.500	71.5761	38.5000	
SGO_0901	0.594	6.466	0.0011	0.0021	11.500	17.000	25.0634	17.6921	DNA-directed DNA polymerase III
					12.000	17.500	28.1611	17.5000	
SGO_0906	3.270	8.067	0.0000	0.0000	56.500	10.500	123.1373	10.9275	leuA; 2-isopropylmalate synthase
					51.000	14.500	119.6846	14.5000	
SGO_0911	1.606	7.986	0.0001	0.0000	45.000	29.000	98.0740	30.1806	hypothetical protein SGO_0911
					39.500	32.500	92.6969	32.5000	
SGO_0934	-0.121	3.967	0.0745	0.4594	2.500		5.4486		galE-2; UDP-glucose 4-epimerase
					2.000	5.500	4.6935	5.5000	
SGO_0946	0.714	7.288	0.0005	0.0007	23.000	30.000	50.1267	31.2213	Deblocking aminopeptidase
					20.000	28.000	46.9351	28.0000	
SGO_0948	0.086	6.180	0.0837	0.5279	9.000	19.000	19.6148	19.7735	pgdA; peptidoglycan N-acetylglucosamine deacetylase A
					7.500	15.500	17.6007	15.5000	

Show detected proteins only
Show all proteins
Filter by category:
ABC Transporter

Proteins found: 769

Test	Cutoff
q-Value	.005
p-Value	

Colour	Signif	Direction	Applies To
red	yes	+	ratios, bars
yellow	no	n/a	bars
green	yes	−	ratios, bars
pink	yes	+	*p*- , *q*-Values
light green	yes	−	*p*- , *q*-Values

Dot Plots | Dot Plots

Protein	SgFn vs Sg: Log_2 Ratio	SgFn vs Sg: Log_2 Sum	SgFn vs Sg: *q*-Value	SgFn vs Sg: *p*-Value	Raw: *SgFn*	Raw: *Sg*	Normalized: *SgFn*	Normalized: *Sg*	Description
SGO_0949	-0.475	5.295	0.0377	0.2032	5.000	12.500	10.8971	13.0089	deaD; DEAD RNA helicase
					2.500	9.500	5.8669	9.5000	
SGO_0950	1.300	5.753	0.0324	0.1703	12.000		26.1531		oxidoreductase
					8.000	9.000	18.7741	9.0000	
SGO_0951	0.064	6.011	0.1114	0.7288	6.500	13.000	14.1662	13.5292	udk; uridine kinase
					8.000	18.000	18.7741	18.0000	
SGO_0954	0.510	6.460	0.0041	0.0126	13.000	18.000	28.3325	18.7328	ATP-binding protein
					10.000	17.500	23.4676	17.5000	
SGO_0955	-0.040	4.797	0.1102	0.7184	3.000	5.500	6.5383	5.7239	transporter
					3.000	8.500	7.0403	8.5000	
SGO_0957	-2.699	5.625	0.0124	0.0518		20.500		21.3345	hypothetical protein SGO_0957
					1.500	24.500	3.5201	24.5000	
SGO_0959	2.052	5.945	0.0046	0.0144	14.000	8.000	30.5119	8.3257	copper homeostasis protein CutC
					8.000	4.000	18.7741	4.0000	
SGO_0966	-1.271	6.593	0.0003	0.0003	6.500	31.000	14.1662	32.2620	hsa; streptococcal hemagglutinin
					6.000	36.000	14.0805	36.0000	
SGO_0969	0.191	3.585	0.0379	0.2044	2.000	3.500	4.3588	3.6425	nss; nucleotide sugar synthetase-like protein
						4.000		4.0000	
SGO_0981	-1.139	4.303	0.0531	0.3053		5.500		5.7239	comE operon protein 2 family
					1.500	10.500	3.5201	10.5000	
SGO_0982	-2.577	8.302	0.0002	0.0002	10.000	121.500	21.7942	126.4462	amino acid ABC transporter, amino acid-binding protein
					10.000	144.000	23.4676	144.0000	
SGO_0987	0.344	7.742	0.0021	0.0052	28.000	48.000	61.0238	49.9541	metK; S-adenosylmethionine synthetase
					25.000	44.500	58.6689	44.5000	

Log_2 Ratios: -6 -4 -2 0 2 4 6

Show detected proteins only
Show all proteins
Filter by category: ABC Transporter

Proteins found: 769

Test	Cutoff
q-Value	.005
p-Value	

	Signif	Direction	Applies To
red	yes	+	ratios, bars
yellow	no	n/a	bars
green	yes	−	ratios, bars
pink	yes	+	*p*- , *q*-Values
light green	yes	−	*p*- , *q*-Values

Dot Plots Dot Plots

Streptococcus gordonii

Protein	SgFn vs Sg: Log_2 Ratio	Log_2 Sum	*q*-Value	*p*-Value	Raw: *SgFn*	Raw: *Sg*	Normalized: *SgFn*	Normalized: *Sg*	Description
SGO_0995	1.419	6.066	0.0067	0.0234	9.000	5.500	19.6148	5.7239	metallo-beta-lactamase superfamily protein
					12.000	13.500	28.1611	13.5000	
SGO_0997	-0.144	5.713	0.0609	0.3621	5.000	12.000	10.8971	12.4885	hypothetical protein SGO_0997
					6.000	15.000	14.0805	15.0000	
SGO_1000	2.968	5.438	0.0074	0.0270	9.000	2.500	19.6148	2.6018	recJ; single-stranded-DNA-specific exonuclease RecJ
					9.000		21.1208		
SGO_1001	1.289	8.028	0.0064	0.0223	33.000	42.500	71.9209	44.2302	apt; adenine phosphoribosyltransferase
					48.500	31.000	113.8177	31.0000	
SGO_1003	-0.915	5.035	0.0023	0.0059	2.500	11.500	5.4486	11.9682	DNA replication protein DnaD
					2.500	9.500	5.8669	9.5000	
SGO_1004	0.470	3.945	0.0697	0.4252	2.000		4.3588		glutathione S-transferase family protein
					3.000	4.000	7.0403	4.0000	
SGO_1005	0.530	5.015	0.0122	0.0505	5.000	5.500	10.8971	5.7239	Bcl-2 family protein
					3.500	7.500	8.2136	7.5000	
SGO_1006	0.802	2.778			2.000		4.3588		conserved hypothetical protein TIGR00486
						2.500		2.5000	
SGO_1009	2.074	9.379	0.0003	0.0003	115.000	57.000	250.6335	59.3204	rfbA-1; glucose-1-phosphate thymidylyltransferase
					122.500	68.500	287.4777	68.5000	
SGO_1010	0.815	7.342	0.0017	0.0041	26.000	28.000	56.6650	29.1399	rmlC; dTDP-4-keto-6-deoxyglucose-3,5-epimerase
					20.000	29.500	46.9351	29.5000	
SGO_1011	2.376	9.181	0.0022	0.0055	90.500	35.000	197.2377	36.4248	rfbB-1; dTDP-glucose 4,6-dehydratase
					123.000	58.000	288.6511	58.0000	
SGO_1012	1.633	9.235	0.0001	0.0000	102.500	72.000	223.3908	74.9311	galE-1; UDP-glucose 4-epimerase
					99.000	72.000	232.3289	72.0000	

Log_2 Ratios: -6 -4 -2 0 2 4 6

Show detected proteins only
Show all proteins
Filter by category: ABC Transporter

Proteins found: 769

Test	Cutoff
q-Value	.005
p-Value	

Color	Signif	Direction	Applies To
red	yes	+	ratios, bars
yellow	no	n/a	bars
green	yes	−	ratios, bars
pink	yes	+	*p*- , *q*-Values
light green	yes	−	*p*- , *q*-Values

Dot Plots Dot Plots

Hendrickson *et al.*

Protein	SgFn vs Sg Log$_2$ Ratio	Log$_2$ Sum	*q*-Value	*p*-Value	Raw *SgFn*	Raw *Sg*	Normalized *SgFn*	Normalized *Sg*	Description
SGO_1013	-2.184	6.159	0.0202	0.0930		34.500		35.9045	Glycosyltransferase involved in cell wall biogenesis
					3.000	28.500	7.0403	28.5000	
SGO_1016	-1.977	5.619	0.0303	0.1580	2.500	17.000	5.4486	17.6921	putative glycosyltransferase
						26.000		26.0000	
SGO_1019	-2.526	6.462	0.0035	0.0105		39.500		41.1080	glycosyl transferase
					3.000	40.000	7.0403	40.0000	
SGO_1020	1.139	7.938	0.0003	0.0002	37.000	37.500	80.6386	39.0266	rfbD; dTDP-4-dehydrorhamnose reductase
					37.500	37.500	88.0034	37.5000	
SGO_1024	-2.758	6.575	0.0023	0.0063	2.000	31.500	4.3588	32.7824	putative polysaccharide ABC transporter
					3.500	50.000	8.2136	50.0000	
SGO_1025	-1.071	6.306	0.0103	0.0406	8.500	21.500	18.5251	22.3753	rgp; glycosyltransferase
					3.500	30.000	8.2136	30.0000	
SGO_1026	-1.947	8.073	0.0048	0.0151	10.500	77.000	22.8839	80.1346	rhamnosyltransferase
					13.500	134.500	31.6812	134.5000	
SGO_1031	-0.494	5.534	0.0028	0.0079	4.000	13.500	8.7177	14.0496	cmk; cytidylate kinase
					4.500	13.000	10.5604	13.0000	
SGO_1033	-0.590	7.556	0.0312	0.1631	17.500	36.000	38.1399	37.4655	rpmI; ribosomal protein L35
					14.500	78.500	34.0280	78.5000	
SGO_1034	0.007	9.370	0.1242	0.8190	79.000	158.000	172.1743	164.4321	rplT; ribosomal protein L20
					68.000	165.500	159.5795	165.5000	
SGO_1035	1.252	6.060	0.0022	0.0058	10.000	7.000	21.7942	7.2850	gloA; lactoylglutathione lyase
					10.500	13.000	24.6409	13.0000	
SGO_1036	-0.270	7.310	0.0668	0.4036	21.000	34.500	45.7679	35.9045	amino acid ABC transporter, ATP-binding protein SP1242
					11.500	50.000	26.9877	50.0000	

Log$_2$ Ratios: -6 -4 -2 0 2 4 6

Show detected proteins only
Show all proteins
Filter by category:
ABC Transporter

Proteins found: 769

Test	Cutoff
q-Value	.005
p-Value	

	Signif	Direction	Applies To
red	yes	+	ratios, bars
yellow	no	n/a	bars
green	yes	−	ratios, bars
pink	yes	+	*p*- , *q*-Values
light green	yes	−	*p*- , *q*-Values

Dot Plots Dot Plots

Hendrickson *et al.*

Protein	SgFn vs Sg: Log_2 Ratio	Log_2 Sum	*q*-Value	*p*-Value	Raw: *SgFn*	Raw: *Sg*	Normalized: *SgFn*	Normalized: *Sg*	Description
**SGO_1037**	-2.158	5.957	0.0010	0.0019	1.500	23.500	3.2691	24.4567	glutamine ABC transporter permease and substrate binding protein
					4.000	25.000	9.3870	25.0000	
**SGO_1038**	1.093	5.266	0.0097	0.0377	7.000	4.000	15.2560	4.1628	uvrB; excinuclease ABC, B subunit
					4.500	8.500	10.5604	8.5000	
**SGO_1041**	1.365	4.298			6.500		14.1662		hypothetical protein SGO_1041
						5.500		5.5000	
**SGO_1044**	2.231	3.094							MutT/nudix family protein
					3.000	1.500	7.0403	1.5000	
**SGO_1047**	-2.388	5.361	0.0294	0.1515		14.500		15.0903	hypothetical protein SGO_1047
					1.500	22.500	3.5201	22.5000	
**SGO_1050**	1.494	3.254							ribF; riboflavin biosynthesis protein RibF
					3.000	2.500	7.0403	2.5000	
**SGO_1059**	-1.598	5.982	0.0001	0.0001	4.000	23.000	8.7177	23.9363	pstB; Phosphate import ATP-binding protein pstB 1 (Phosphate-transporting ATPase 1) (ABC phosphate transporter 1)
					3.000	23.500	7.0403	23.5000	
**SGO_1060**	-1.641	7.306	0.0009	0.0016	7.500	52.000	16.3457	54.1169	phosphate transport system regulatory protein
					9.500	65.500	22.2942	65.5000	
**SGO_1065**	-0.762	6.548	0.0014	0.0031	9.000	29.000	19.6148	30.1806	hypothetical protein SGO_1065
					6.500	28.500	15.2539	28.5000	
**SGO_1066**	-0.160	6.695	0.0523	0.2992	10.500	22.500	22.8839	23.4160	hypothetical protein SGO_1066
					11.000	31.500	25.8143	31.5000	
**SGO_1067**	0.317	2.974			2.000		4.3588		hypothetical protein SGO_1067
						3.500		3.5000	
**SGO_1069**	0.425	8.560	0.0023	0.0062	49.000	84.000	106.7917	87.4196	membrane alanyl aminopeptidase
					46.500	74.000	109.1242	74.0000	

Log2 Ratios: -6 -4 -2 0 2 4 6

Show detected proteins only
Show all proteins
Filter by category: ABC Transporter

Proteins found: 769

Test	Cutoff
q-Value	.005
p-Value	

Color	Signif	Direction	Applies To
red	yes	+	ratios, bars
yellow	no	n/a	bars
green	yes	−	ratios, bars
pink	yes	+	*p*- , *q*-Values
light green	yes	−	*p*- , *q*-Values

Dot Plots Dot Plots

Protein	SgFn vs Sg Log2 Ratio	Log2 Sum	q-Value	p-Value	Raw SgFn	Raw Sg	Normalized SgFn	Normalized Sg	Description
SGO_1072	3.899	6.269	0.0019	0.0046	17.000		37.0502		ciaR; Transcriptional regulatory protein CiaR
					16.000	2.500	37.5481	2.5000	
SGO_1073	-0.816	5.409	0.0184	0.0834		15.000		15.6106	Sensor protein CiaH
					4.000	17.500	9.3870	17.5000	
SGO_1075	2.151	5.320	0.0250	0.1226	9.500		20.7045		alpha-amylase precursor
					6.500	4.000	15.2539	4.0000	
SGO_1077	-0.574	5.673	0.1462	0.9937	1.500	11.500	3.2691	11.9682	coaA; pantothenate kinase
					9.500	13.500	22.2942	13.5000	
SGO_1079	0.866	7.696	0.0238	0.1139	20.000	27.500	43.5884	28.6195	pdp; pyrimidine-nucleoside phosphorylase
					39.500	42.500	92.6969	42.5000	
SGO_1080	-0.018	8.282	0.1194	0.7822	37.000	80.500	80.6386	83.7771	deoC; deoxyribose-phosphate aldolase
					31.500	73.000	73.9228	73.0000	
SGO_1082	-1.703	12.818	0.0002	0.0001	443.000	2606.500	965.4839	2712.6096	lipoprotein
					316.500	2801.500	742.7486	2801.5000	
SGO_1090	-0.494	5.182	0.0601	0.3543		10.500		10.9275	pseudouridine synthase, RluA family
					4.000	16.000	9.3870	16.0000	
SGO_1096	6.559	13.354	0.0001	0.0000	2269.000	49.500	4945.1085	51.5151	butA; acetoin dehydrogenase
					2308.500	58.500	5417.4882	58.5000	
SGO_1097	2.446	3.689			5.000		10.8971		proB; glutamate 5-kinase
						2.000		2.0000	
SGO_1098	3.518	6.387	0.0016	0.0037	18.500		40.3193		proA; gamma-glutamyl phosphate reductase
					17.000	3.500	39.8949	3.5000	
SGO_1099	2.177	5.178	0.0740	0.4560	2.000		4.3588		proC; pyrroline-5-carboxylate reductase
					12.500	2.500	29.3345	2.5000	

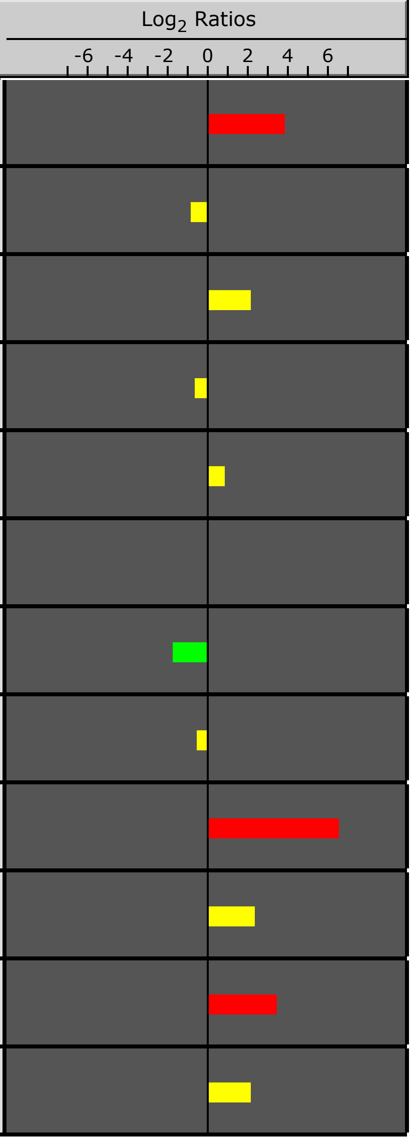

Show detected proteins only
Show all proteins
Filter by category:
ABC Transporter

Proteins found: 769

Test	Cutoff
q-Value	.005
p-Value	

	Signif	Direction	Applies To
red	yes	+	ratios, bars
yellow	no	n/a	bars
green	yes	–	ratios, bars
pink	yes	+	p- , q-Values
light green	yes	–	p- , q-Values

Dot Plots | Dot Plots

Hendrickson *et al.*

Protein	SgFn vs Sg Log_2 Ratio	Log_2 Sum	*q*-Value	*p*-Value	Raw *SgFn*	Raw *Sg*	Normalized *SgFn*	Normalized *Sg*	Description	Log_2 Ratios (-6 -4 -2 0 2 4 6)
SGO_1104	-0.461	4.724	0.0114	0.0463		9.500		9.8867	carB; carbamoyl-phosphate synthase, large subunit	
					3.000	9.500	7.0403	9.5000		
SGO_1107	-1.932	4.963	0.0370	0.1991		16.500		17.1717	PyrR bifunctional protein	
					1.500	10.500	3.5201	10.5000		
SGO_1109	-1.562	7.655	0.0005	0.0008	11.500	66.500	25.0634	69.2072	pyrB; aspartate carbamoyltransferase	
					11.000	81.500	25.8143	81.5000		
SGO_1110	-2.330	6.283	0.0068	0.0242		33.000		34.3434	surface antigen SCP-like domain	
					3.000	36.500	7.0403	36.5000		
SGO_1111	-2.986	6.930	0.0008	0.0012	3.000	45.500	6.5383	47.3523	fruR; phosphotransferase system repressor	
					3.000	61.000	7.0403	61.0000		
SGO_1112	-2.373	7.196	0.0001	0.0000	5.000	58.000	10.8971	60.3612	fruB; 1-phosphofructokinase	
					5.500	62.500	12.9072	62.5000		
SGO_1113	-4.663	11.177	0.0000	0.0000	30.000	1053.000	65.3827	1095.8672	fruA; PTS system, fructose specific IIABC components	
					12.500	1124.000	29.3345	1124.0000		
SGO_1114	-2.537	6.777	0.0003	0.0002	5.000	46.500	10.8971	48.3930	Protein of unknown function (DUF1149) superfamily	
					2.500	44.500	5.8669	44.5000		
SGO_1115	2.947	4.930							DegV family protein	
					11.500	3.500	26.9877	3.5000		
SGO_1116	2.419	6.592	0.0002	0.0001	19.500	6.500	42.4987	6.7646	dapB; dihydrodipicolinate reductase	
					16.500	8.500	38.7215	8.5000		
SGO_1118	1.378	4.957	0.0183	0.0825	5.500		11.9868		ABC transporter, ATP-binding protein SP1553	
					6.000	5.000	14.0805	5.0000		
SGO_1120	1.559	8.789	0.0013	0.0025	67.000	48.000	146.0213	49.9541	guaA; GMP synthase	
					78.500	62.000	184.2204	62.0000		

Show detected proteins only
Show all proteins
Filter by category:
ABC Transporter

Proteins found: 769

Test	Cutoff
***q*-Value**	**.005**
p-Value	

	Signif	Direction	Applies To
red	yes	+	ratios, bars
yellow	no	n/a	bars
green	yes	−	ratios, bars
pink	yes	+	*p*- , *q*-Values
light green	yes	−	*p*- , *q*-Values

Dot Plots Dot Plots

Streptococcus gordonii

Protein	SgFn vs Sg Log$_2$ Ratio	Log$_2$ Sum	*q*-Value	*p*-Value	Raw *SgFn*	Raw *Sg*	Normalized *SgFn*	Normalized *Sg*	Description
SGO_1123	0.954	7.773	0.0005	0.0007	35.000	35.000	76.2798	36.4248	ffh; signal recognition particle protein
					29.000	38.000	68.0559	38.0000	
SGO_1129	-0.262	6.672	0.0032	0.0095	10.500	28.000	22.8839	29.1399	lplA; lipoate protein ligase A
					10.000	26.500	23.4676	26.5000	
SGO_1130	-3.046	7.970	0.0000	0.0000	5.500	109.000	11.9868	113.4373	dihydrolipoamide dehydrogenase
					6.500	110.000	15.2539	110.0000	
SGO_1131	-2.627	6.185	0.0028	0.0079	2.500	32.000	5.4486	33.3027	sucB; dihydrolipoamide S-acetyltransferase
						34.000		34.0000	
SGO_1133	-0.959	6.337	0.0317	0.1660		35.000		36.4248	acoA; acetoin dehydrogenase
					7.000	28.000	16.4273	28.0000	
SGO_1134	2.321	6.119	0.0067	0.0238	9.000	4.500	19.6148	4.6832	hypothetical protein SGO_1134
					16.500	6.500	38.7215	6.5000	
SGO_1139	0.405	6.420	0.0116	0.0478	11.500	15.000	25.0634	15.6106	GTP-binding protein
					10.000	21.500	23.4676	21.5000	
SGO_1140	0.128	8.431	0.0388	0.2102	39.000	85.500	84.9975	88.9807	clpX; ATP-dependent Clp protease, ATP-binding subunit ClpX
					40.500	76.000	95.0437	76.0000	
SGO_1141	2.133	6.005	0.0060	0.0203	8.500	4.500	18.5251	4.6832	folA; dihydrofolate reductase
					14.500	7.000	34.0280	7.0000	
SGO_1143	0.391	5.180	0.0534	0.3075	3.500	5.500	7.6280	5.7239	thyA; thymidylate synthase
					5.500	10.000	12.9072	10.0000	
SGO_1144	2.268	9.727	0.0002	0.0001	152.500	66.500	332.3619	69.2072	glcK; glucokinase
					157.500	76.500	369.6142	76.5000	
SGO_1148	-2.650	8.527	0.0003	0.0003	12.500	164.000	27.2428	170.6764	cshB; surface-associated protein CshB
					10.000	147.500	23.4676	147.5000	

Log$_2$ Ratios: -6 -4 -2 0 2 4 6

Show detected proteins only
Show all proteins
Filter by category:
ABC Transporter

Proteins found: 769

Test	Cutoff
***q*-Value**	**.005**
p-Value	

	Signif	Direction	Applies To
(red)	yes	+	ratios, bars
(yellow)	no	n/a	bars
(green)	yes	−	ratios, bars
(pink)	yes	+	*p*- , *q*-Values
(light green)	yes	−	*p*- , *q*-Values

Dot Plots Dot Plots

Protein	SgFn vs Sg Log$_2$ Ratio	Log$_2$ Sum	*q*-Value	*p*-Value	Raw *SgFn*	Raw *Sg*	Normalized *SgFn*	Normalized *Sg*	Description
SGO_1150	0.600	4.826	0.0397	0.2158	5.500		11.9868		hypothetical protein SGO_1150
					4.000	7.000	9.3870	7.0000	
SGO_1151	2.287	9.772	0.0000	0.0000	165.000	68.500	359.6046	71.2886	glyA; serine hydroxymethyltransferase
					156.000	77.500	366.0941	77.5000	
SGO_1154	0.128	7.465	0.0957	0.6170	30.000	47.500	65.3827	49.4337	prfA; peptide chain release factor 1
					12.500	32.500	29.3345	32.5000	
SGO_1155	-1.087	5.504	0.0219	0.1031	4.500	9.500	9.8074	9.8867	tdk; thymidine kinase
					2.000	21.000	4.6935	21.0000	
SGO_1159	2.423	4.557	0.0157	0.0696	4.500		9.8074		guaC; guanosine monophosphate reductase
					5.000	2.000	11.7338	2.0000	
SGO_1167	-1.132	7.433	0.0004	0.0005	13.000	54.000	28.3325	56.1983	nox; NADH oxidase
					11.000	62.500	25.8143	62.5000	
SGO_1169	-0.745	6.887	0.0056	0.0186	12.500	37.500	27.2428	39.0266	NADPH-dependent FMN reductase
					7.500	34.500	17.6007	34.5000	
SGO_1170	-2.073	8.329	0.0001	0.0000	15.500	125.500	33.7810	130.6091	NADPH-dependent FMN reductase
					12.000	129.000	28.1611	129.0000	
SGO_1171	-3.650	7.609	0.0001	0.0001	5.000	89.000	10.8971	92.6232	thioredoxin family protein
					2.000	87.000	4.6935	87.0000	
SGO_1185	-0.148	4.148	0.1009	0.6525	2.500	7.000	5.4486	7.2850	acetyltransferase, GNAT family
						5.000		5.0000	
SGO_1189	-2.586	9.364	0.0003	0.0004	20.000	292.500	43.5884	304.4076	lipoprotein, putative
					21.500	260.500	50.4553	260.5000	
SGO_1191	-1.030	11.163	0.0007	0.0010	178.000	789.500	387.9371	821.6402	rplL; ribosomal protein L7/L12
					155.500	718.000	364.9207	718.0000	

Log$_2$ Ratios: -6 -4 -2 0 2 4 6

Show detected proteins only
Show all proteins
Filter by category:
ABC Transporter

Proteins found: 769

Test	Cutoff
q-Value	.005
p-Value	

	Signif	Direction	Applies To
red	yes	+	ratios, bars
yellow	no	n/a	bars
green	yes	–	ratios, bars
pink	yes	+	*p*-, *q*-Values
light green	yes	–	*p*-, *q*-Values

Dot Plots Dot Plots

Protein	SgFn vs Sg Log2 Ratio	Log2 Sum	*q*-Value	*p*-Value	Raw SgFn	Raw Sg	Normalized SgFn	Normalized Sg	Description
SGO_1192	-0.243	9.189	0.0417	0.2316	50.500	144.500	110.0608	150.3825	BL5; 50S ribosomal protein L10
					68.000	163.500	159.5795	163.5000	
SGO_1193	1.446	6.759	0.0004	0.0005	17.000	13.500	37.0502	14.0496	gid; Glucose inhibited division protein A
					18.000	15.000	42.2416	15.0000	
SGO_1197	-1.919	7.885	0.0011	0.0021	9.000	79.500	19.6148	82.7364	topA; DNA topoisomerase I
					13.000	103.500	30.5078	103.5000	
SGO_1202	0.838	5.772	0.0078	0.0290	6.500	9.000	14.1662	9.3664	GTP-binding protein
					9.000	10.000	21.1208	10.0000	
SGO_1203	1.365	7.031	0.0011	0.0022	19.500	15.500	42.4987	16.1310	anaerobic ribonucleotide reductase
					22.000	20.500	51.6287	20.5000	
SGO_1205	1.087	5.095	0.0245	0.1182	7.500	7.000	16.3457	7.2850	dapA; dihydrodipicolinate synthase
					3.000	3.500	7.0403	3.5000	
SGO_1206	0.207	7.665	0.0140	0.0604	23.500	42.500	51.2164	44.2302	asd; aspartate-semialdehyde dehydrogenase
					24.500	50.000	57.4955	50.0000	
SGO_1210	1.322	6.338	0.0019	0.0045	11.500	11.500	25.0634	11.9682	fhs-1; formate--tetrahydrofolate ligase
					14.000	11.000	32.8546	11.0000	
SGO_1213	-1.696	5.911	0.0075	0.0273	4.500	16.000	9.8074	16.6514	coaC; phosphopantothenoylcysteine decarboxylase
					2.000	29.000	4.6935	29.0000	
SGO_1215	0.991	10.269	0.0005	0.0007	178.000	190.000	387.9371	197.7348	manB; phosphomannomutase
					184.500	215.000	432.9766	215.0000	
SGO_1216	1.372	6.913	0.0009	0.0017	21.500	18.500	46.8576	19.2531	bta; Possible bacteriocin transport accessory protein
					17.000	14.500	39.8949	14.5000	
SGO_1219	0.989	8.278	0.0179	0.0802	31.500	44.000	68.6518	45.7912	pta; phosphate acetyltransferase
					60.500	54.000	141.9788	54.0000	

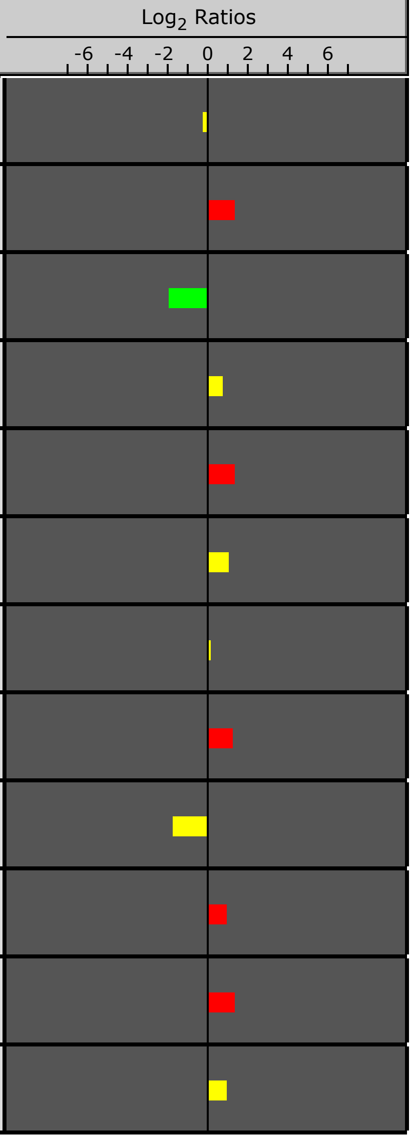

Show detected proteins only
Show all proteins
Filter by category:
ABC Transporter

Proteins found: 769

Test	Cutoff
q-Value	.005
p-Value	

Color	Signif	Direction	Applies To
red	yes	+	ratios, bars
yellow	no	n/a	bars
green	yes	–	ratios, bars
pink	yes	+	*p*- , *q*-Values
light green	yes	–	*p*- , *q*-Values

Dot Plots Dot Plots

Protein	SgFn vs Sg Log_2 Ratio	Log_2 Sum	*q*-Value	*p*-Value	Raw *SgFn*	Raw *Sg*	Normalized *SgFn*	Normalized *Sg*	Description
SGO_1221	-2.962	7.363	0.0009	0.0017	2.000	62.000	4.3588	64.5240	ppnK; Probable inorganic polyphosphate/ATP-NAD kinase
					8.000	77.000	18.7741	77.0000	
SGO_1222	-1.021	4.154	0.0070	0.0250		7.000		7.2850	GTP pyrophosphokinase-like protein
					1.500	7.000	3.5201	7.0000	
SGO_1224	-0.808	8.161	0.0001	0.0001	23.500	89.500	51.2164	93.1435	Ribose-phosphate pyrophosphokinase 2
					22.500	89.000	52.8020	89.0000	
SGO_1225	0.444	5.289	0.0259	0.1290	4.000	7.500	8.7177	7.8053	pyridoxal-phosphate dependent aminotransferase
					6.000	8.500	14.0805	8.5000	
SGO_1226	0.554	6.560	0.0306	0.1599	10.000	13.500	21.7942	14.0496	hypothetical protein SGO_1226
					14.500	24.500	34.0280	24.5000	
SGO_1228	1.028	5.079	0.0021	0.0053	5.500	6.500	11.9868	6.7646	DNA-binding protein BH0551
					4.500	4.500	10.5604	4.5000	
SGO_1231	-0.634	8.407	0.0061	0.0209	35.500	90.000	77.3695	93.6639	gyrA; DNA gyrase, A subunit
					24.000	112.000	56.3222	112.0000	
SGO_1232	2.961	10.386	0.0001	0.0000	277.000	65.000	603.6999	67.6461	L-lactate dehydrogenase
					247.500	85.500	580.8223	85.5000	
SGO_1234	-0.416	10.488	0.0136	0.0581	163.000	359.500	355.2458	374.1351	rpsA; 30S ribosomal protein S1
					112.000	444.000	262.8368	444.0000	
SGO_1238	1.196	7.844	0.0074	0.0267	46.000	35.000	100.2534	36.4248	ilvE; branched-chain amino acid aminotransferase
					26.000	32.000	61.0157	32.0000	
SGO_1239	-2.435	6.547	0.0006	0.0010	2.000	35.500	4.3588	36.9452	parC; DNA topoisomerase IV, A subunit
					5.000	40.500	11.7338	40.5000	
SGO_1247	-1.887	9.490	0.0001	0.0000	37.500	267.500	81.7283	278.3898	5'-nucleotidase family protein
					30.500	287.500	71.5761	287.5000	

Log_2 Ratios: -6 -4 -2 0 2 4 6

Show detected proteins only
Show all proteins
Filter by category:
ABC Transporter

Proteins found: 769

Test	Cutoff
q-Value	.005
p-Value	

	Signif	Direction	Applies To
red	yes	+	ratios, bars
yellow	no	n/a	bars
green	yes	–	ratios, bars
pink	yes	+	*p*- , *q*-Values
light green	yes	–	*p*- , *q*-Values

Dot Plots Dot Plots

Hendrickson *et al.*

Streptococcus gordonii

Hackett Laboratory UW

Protein	SgFn vs Sg				Raw		Normalized		Description
	Log_2 Ratio	Log_2 Sum	*q*-Value	*p*-Value	*SgFn*	*Sg*	*SgFn*	*Sg*	
SGO_1248	1.629	6.962	0.0102	0.0400	14.500	17.500	31.6016	18.2124	pyrC; dihydroorotase
					27.000	11.500	63.3624	11.5000	
SGO_1250	0.002	5.377	0.1241	0.8174	5.000	7.000	10.8971	7.2850	ung; uracil-DNA glycosylase
					4.000	14.000	9.3870	14.0000	
SGO_1253	-0.416	6.262	0.0462	0.2609	10.000	17.500	21.7942	18.2124	pyrE; orotate phosphoribosyltransferase
					5.000	25.000	11.7338	25.0000	
SGO_1257	1.286	5.778	0.0098	0.0384	11.500	6.000	25.0634	6.2443	probable transcriptional regulator (LysR family)
					6.000	9.500	14.0805	9.5000	
SGO_1258	-0.144	4.608	0.0885	0.5643	3.000	4.000	6.5383	4.1628	NAD-dependent deacetylase (Regulatory protein SIR2-like protein)
					2.000	9.000	4.6935	9.0000	
SGO_1260	-0.908	7.764	0.0001	0.0001	18.000	69.000	39.2296	71.8090	deoD; purine nucleoside phosphorylase
					15.500	70.000	36.3747	70.0000	
SGO_1263	-1.173	8.218	0.0007	0.0011	22.000	106.500	47.9473	110.8356	purine nucleoside phosphorylase I, inosine and guanosine-specific
					18.500	95.500	43.4150	95.5000	
SGO_1264	0.143	10.075	0.0658	0.3970	149.500	220.000	325.8236	228.9561	deoB; phosphopentomutase
					103.000	282.000	241.7160	282.0000	
SGO_1265	-0.125	7.240	0.0847	0.5356	15.500	26.000	33.7810	27.0585	rpiA; ribose 5-phosphate isomerase
					15.500	54.000	36.3747	54.0000	
SGO_1266	0.968	6.266	0.0005	0.0007	11.000	12.500	23.9736	13.0089	trmE; tRNA modification GTPase TrmE
					11.500	13.000	26.9877	13.0000	
SGO_1273	0.309	7.412	0.0431	0.2413	27.000	40.000	58.8444	41.6284	rpoD; RNA polymerase sigma factor
					15.500	33.500	36.3747	33.5000	
SGO_1276	-1.596	7.134	0.0001	0.0000	8.500	49.500	18.5251	51.5151	rpsU; ribosomal protein S21
					7.000	54.000	16.4273	54.0000	

Log_2 Ratios: -6 -4 -2 0 2 4 6

Show detected proteins only
Show all proteins
Filter by category:
ABC Transporter

Proteins found: 769

Test	Cutoff
q-Value	.005
p-Value	

Signif	Direction	Applies To
yes	+	ratios, bars
no	n/a	bars
yes	−	ratios, bars
yes	+	*p*-, *q*-Values
yes	−	

Dot Plots Dot Plots

Hendrickson *et al.*

Protein	SgFn vs Sg Log_2 Ratio	Log_2 Sum	*q*-Value	*p*-Value	Raw *SgFn*	Raw *Sg*	Normalized *SgFn*	Normalized *Sg*	Description
SGO_1281	-0.896	4.066	0.0337	0.1781		5.500		5.7239	penicillinase repressor, putative
					1.500	7.500	3.5201	7.5000	
SGO_1283	-0.373	7.916	0.0112	0.0452	27.500	68.500	59.9341	71.2886	oxidoreductase
					19.500	64.500	45.7618	64.5000	
SGO_1284	-0.375	5.791	0.0754	0.4661	3.500	15.500	7.6280	16.1310	thioredoxin-disulfide reductase
					7.500	14.000	17.6007	14.0000	
SGO_1293	0.127	7.656	0.0294	0.1508	22.500	44.500	49.0370	46.3116	asnS; asparaginyl-tRNA synthetase
					24.000	50.000	56.3222	50.0000	
SGO_1297	2.252	7.245	0.0019	0.0046	34.000	12.000	74.1003	12.4885	aspC; aspartate aminotransferase
					22.000	13.500	51.6287	13.5000	
SGO_1312	3.698	7.531	0.0000	0.0000	40.000	7.000	87.1769	7.2850	pepT; peptidase T
					36.000	6.000	84.4832	6.0000	
SGO_1315	1.317	3.611			4.000		8.7177		trmD; tRNA (guanine-N1)-methyltransferase
						3.500		3.5000	
SGO_1316	-0.094	4.561	0.0829	0.5220	2.500	7.000	5.4486	7.2850	16S rRNA processing protein RimM
					2.500	5.000	5.8669	5.0000	
SGO_1323	-0.001	7.305	0.1420	0.9590	19.500	42.500	42.4987	44.2302	rpsP; ribosomal protein S16
					15.500	35.000	36.3747	35.0000	
SGO_1327	-0.272	5.984	0.0425	0.2370	6.500	13.000	14.1662	13.5292	HAD-superfamily subfamily IIA hydrolase, TIGR01457
					6.000	21.500	14.0805	21.5000	
SGO_1336	0.376	6.355	0.0261	0.1308	8.500	17.000	18.5251	17.6921	pcrA; ATP-dependent DNA helicase PcrA
					12.000	17.500	28.1611	17.5000	
SGO_1338	-0.729	6.239	0.0050	0.0161	6.500	19.500	14.1662	20.2938	Signal peptidase I
					6.000	27.000	14.0805	27.0000	

Log_2 Ratios: -6 -4 -2 0 2 4 6

Show detected proteins only
Show all proteins
Filter by category:
ABC Transporter

Proteins found: 769

Test	Cutoff
q-Value	.005
p-Value	

	Signif	Direction	Applies To
red	yes	+	ratios, bars
yellow	no	n/a	bars
green	yes	−	ratios, bars
pink	yes	+	*p*- , *q*-Values
light green	yes	−	*p*- , *q*-Values

Dot Plots Dot Plots

Hendrickson *et al.*

Protein	SgFn vs Sg Log_2 Ratio	Log_2 Sum	*q*-Value	*p*-Value	Raw *SgFn*	Raw *Sg*	Normalized *SgFn*	Normalized *Sg*	Description
SGO_1339	0.635	11.550	0.0014	0.0030	447.500	551.000	975.2913	573.4310	pyk; pyruvate kinase
					362.500	600.000	850.6994	600.0000	
SGO_1340	1.240	10.517	0.0002	0.0001	227.000	213.500	494.7288	222.1915	Phosphofructokinase
					228.000	213.500	535.0606	213.5000	
SGO_1341	0.393	5.498	0.0437	0.2450	4.000	9.500	8.7177	9.8867	dnaE; DNA-directed DNA polymerase III alpha chain
					7.500	9.000	17.6007	9.0000	
SGO_1342	-0.526	10.529	0.0026	0.0071	155.000	423.500	337.8104	440.7405	ABC transporter, ATP-binding protein SP1715
					115.000	429.000	269.8770	429.0000	
SGO_1347	-3.010	6.238	0.0236	0.1122	2.000	39.500	4.3588	41.1080	membrane associated lipoprotein
						30.000		30.0000	
SGO_1355	0.352	4.188	0.0603	0.3566		4.500		4.6832	lipoprotein, putative
					3.000	6.500	7.0403	6.5000	
SGO_1361	-0.470	3.485							ADP-ribosylhydrolase
					2.000	6.500	4.6935	6.5000	
SGO_1364	-0.705	6.491	0.0080	0.0301	7.000	22.500	15.2560	23.4160	rumA-2; 23S rRNA (uracil-5-)-methyltransferase RumA
					8.000	32.500	18.7741	32.5000	
SGO_1365	-0.327	6.965	0.0064	0.0221	11.500	33.500	25.0634	34.8638	transcription regulator yrfE
					13.000	34.500	30.5078	34.5000	
SGO_1367	1.206	5.508	0.0015	0.0034	6.500	6.000	14.1662	6.2443	aroK; shikimate kinase
					7.500	7.500	17.6007	7.5000	
SGO_1368	2.491	5.517	0.0015	0.0034	9.000	1.500	19.6148	1.5611	aroA; 3-phosphoshikimate 1-carboxyvinyltransferase
					7.500	7.000	17.6007	7.0000	
SGO_1369	3.346	8.392	0.0001	0.0000	72.500	14.000	158.0081	14.5699	L-2-hydroxyisocaproate dehydrogenase
					63.000	15.500	147.8457	15.5000	

Log_2 Ratios: -6 -4 -2 0 2 4 6

Show detected proteins only
Show all proteins
Filter by category:
ABC Transporter

Proteins found: 769

Test	Cutoff
***q*-Value**	.005
p-Value	

	Signif	Direction	Applies To
(red)	yes	+	ratios, bars
(yellow)	no	n/a	bars
(green)	yes	−	ratios, bars
(pink)	yes	+	*p*- , *q*-Values
(light green)	yes	−	*p*- , *q*-Values

Dot Plots Dot Plots

Protein	SgFn vs Sg Log_2 Ratio	Log_2 Sum	*q*-Value	*p*-Value	Raw *SgFn*	Raw *Sg*	Normalized *SgFn*	Normalized *Sg*	Description
SGO_1370	1.319	6.800	0.0022	0.0058	21.000	15.500	45.7679	16.1310	Protein of unknown function (DUF964) superfamily
					14.500	15.500	34.0280	15.5000	
SGO_1372	0.153	4.305	0.0774	0.4805		5.500		5.7239	aroC; chorismate synthase
					3.000	7.000	7.0403	7.0000	
SGO_1373	0.481	4.716	0.0699	0.4266		5.500		5.7239	aroB; 3-dehydroquinate synthase
					4.500	10.000	10.5604	10.0000	
SGO_1375	-0.075	5.682	0.0821	0.5164	5.500	11.000	11.9868	11.4478	aroD; 3-dehydroquinate dehydratase, type I
					5.500	15.000	12.9072	15.0000	
SGO_1377	-1.512	8.293	0.0003	0.0003	19.000	105.000	41.4090	109.2745	sulfatase
					17.000	123.000	39.8949	123.0000	
SGO_1381	0.018	6.733	0.1240	0.8153	14.500	24.000	31.6016	24.9770	csn1; CRISPR-associated protein, Csn1 family
					9.500	27.500	22.2942	27.5000	
SGO_1383	0.097	8.709	0.0639	0.3823	44.500	91.500	96.9843	95.2249	rplS; ribosomal protein L19
					51.000	106.500	119.6846	106.5000	
SGO_1386	3.748	6.408	0.0246	0.1188	15.500		33.7810		chorismate mutase
					20.500	3.000	48.1085	3.0000	
SGO_1388	-0.919	5.475	0.0302	0.1570	2.000	9.500	4.3588	9.8867	pulA-1; pullulanase, type I
					5.000	18.500	11.7338	18.5000	
SGO_1389	-0.596	6.153	0.0095	0.0368	5.500	18.000	11.9868	18.7328	conserved hypothetical protein TIGR00147
					7.000	24.000	16.4273	24.0000	
SGO_1390	-0.644	6.055	0.0041	0.0125	5.000	20.000	10.8971	20.8142	ligA; DNA ligase, NAD-dependent
					6.500	19.500	15.2539	19.5000	
SGO_1397	0.483	6.995	0.0216	0.1016	15.000	20.000	32.6913	20.8142	map; methionine aminopeptidase, type I
					17.500	33.000	41.0682	33.0000	

Log_2 Ratios: -6 -4 -2 0 2 4 6

Show detected proteins only
Show all proteins
Filter by category: ABC Transporter

Proteins found: 769

Test	Cutoff
q-Value	.005
p-Value	

Signif	Direction	Applies To
yes	+	ratios, bars
no	n/a	bars
yes	–	ratios, bars
yes	+	*p*- , *q*-Values
yes	–	*p*- , *q*-Values

Dot Plots Dot Plots

Protein	SgFn vs Sg Log_2 Ratio	Log_2 Sum	*q*-Value	*p*-Value	Raw *SgFn*	Raw *Sg*	Normalized *SgFn*	Normalized *Sg*	Description
SGO_1398	-1.383	5.608	0.0018	0.0044	2.500	15.000	5.4486	15.6106	hypothetical protein SGO_1398
					3.500	19.500	8.2136	19.5000	
SGO_1400	-0.055	6.325	0.1011	0.6549	10.000	18.500	21.7942	19.2531	murA-2; UDP-N-acetylglucosamine 1-carboxyvinyltransferase
					7.500	21.500	17.6007	21.5000	
SGO_1413	0.215	5.654	0.0954	0.6144	3.500	7.000	7.6280	7.2850	first chain of major exonuclease RexA
					8.500	15.500	19.9474	15.5000	
SGO_1414	1.306	5.724	0.0082	0.0306	6.500	5.500	14.1662	5.7239	rexB; putative exonuclease RexB
					10.000	9.500	23.4676	9.5000	
SGO_1422	1.421	7.816	0.0049	0.0157	29.500	28.500	64.2929	29.6602	hypothetical protein SGO_1422
					43.000	30.500	100.9105	30.5000	
SGO_1426	0.877	13.890	0.0009	0.0017	2120.500	2771.500	4621.4644	2884.3267	eno; enolase
					2216.500	2470.500	5201.5866	2470.5000	
SGO_1431	-1.232	10.435	0.0057	0.0190	102.000	570.000	222.3010	593.2045	EzrA; Septation ring formation regulator ezrA
					79.000	383.500	185.3938	383.5000	
SGO_1432	-0.233	7.339	0.0479	0.2714	16.000	33.500	34.8708	34.8638	gyrB; DNA gyrase, B subunit
					16.500	53.500	38.7215	53.5000	
SGO_1434	1.420	4.000	0.0260	0.1301	3.500		7.6280		thiJ; 4-methyl-5(beta-hydroxyethyl)-thiazole monophosphate synthesis protein
					2.500	2.500	5.8669	2.5000	
SGO_1439	-1.950	7.156	0.0001	0.0000	7.000	55.500	15.2560	57.7594	ftsX; cell division protein FtsX
					6.000	55.500	14.0805	55.5000	
SGO_1440	-0.192	8.633	0.0018	0.0041	42.500	104.500	92.6254	108.7542	cell-division ATP-binding protein FtsE
					39.500	103.000	92.6969	103.0000	
SGO_1441	1.397	6.689	0.0036	0.0106	14.000	14.500	30.5119	15.0903	prfB; peptide chain release factor 2
					19.000	13.000	44.5884	13.0000	

Show detected proteins only
Show all proteins
Filter by category:
ABC Transporter

Proteins found: 769

Test	Cutoff
***q*-Value**	.005
p-Value	

	Signif	Direction	Applies To
red	yes	+	ratios, bars
yellow	no	n/a	bars
green	yes	–	ratios, bars
pink	yes	+	*p*- , *q*-Values
light green	yes	–	*p*- , *q*-Values

Dot Plots Dot Plots

Hendrickson *et al.*

Protein	SgFn vs Sg Log_2 Ratio	Log_2 Sum	*q*-Value	*p*-Value	Raw *SgFn*	Raw *Sg*	Normalized *SgFn*	Normalized *Sg*	Description
SGO_1446	0.050	6.385	0.1420	0.9578	8.500	15.000	18.5251	15.6106	murF; UDP-N-acetylmuramoyl-tripeptide--D-alanyl-D-alanine ligase
					10.000	26.000	23.4676	26.0000	
SGO_1447	0.192	6.727	0.0003	0.0002	13.000	23.500	28.3325	24.4567	ddlA; D-Ala-D-Ala ligase
					12.000	25.000	28.1611	25.0000	
SGO_1450	0.488	5.729	0.1066	0.6933	6.000	5.000	13.0765	5.2035	hypothetical protein SGO_1450
					6.500	19.500	15.2539	19.5000	
SGO_1451	-0.022	6.949	0.1451	0.9850	17.000	33.500	37.0502	34.8638	frr; ribosome recycling factor
					10.500	27.000	24.6409	27.0000	
SGO_1452	0.222	6.908	0.0294	0.1514	15.000	30.500	32.6913	31.7416	pyrH; uridylate kinase
					13.500	24.000	31.6812	24.0000	
SGO_1455	0.360	9.165	0.0027	0.0075	72.500	129.500	158.0081	134.7719	rplA; ribosomal protein L1
					70.000	117.000	164.2730	117.0000	
SGO_1456	-0.623	7.371	0.0080	0.0297	18.500	46.500	40.3193	48.3930	rplK; ribosomal protein L11
					11.000	51.000	25.8143	51.0000	
SGO_1458	-1.358	7.397	0.0062	0.0213	13.000	71.500	28.3325	74.4107	aha1; cation-transporting ATPase yfgQ
					8.000	47.000	18.7741	47.0000	
SGO_1460	-2.935	6.888	0.0009	0.0018	3.000	57.500	6.5383	59.8408	DNA translocase ftsK
					3.000	45.000	7.0403	45.0000	
SGO_1463	-2.423	7.258	0.0002	0.0002	7.000	59.500	15.2560	61.9222	peptidyl-prolyl cis-trans isomerase
					4.000	66.500	9.3870	66.5000	
SGO_1465	-1.851	6.642	0.0016	0.0037	4.500	32.000	9.8074	33.3027	ABC transporter, ATP-binding protein SP0770
					5.000	45.000	11.7338	45.0000	
SGO_1466	-0.133	5.958	0.0658	0.3968	6.500	18.500	14.1662	19.2531	MTA/SAH nucleosidase
					6.500	13.500	15.2539	13.5000	

Log_2 Ratios: -6 -4 -2 0 2 4 6

Show detected proteins only
Show all proteins
Filter by category:
ABC Transporter

Proteins found: 769

Test	Cutoff
***q*-Value**	**.005**
p-Value	

	Signif	Direction	Applies To
red	yes	+	ratios, bars
yellow	no	n/a	bars
green	yes	–	ratios, bars
pink	yes	+	*p*- , *q*-Values
light green	yes	–	*p*- , *q*-Values

Dot Plots Dot Plots

Protein	SgFn vs Sg Log_2 Ratio	Log_2 Sum	*q*-Value	*p*-Value	Raw *SgFn*	Raw *Sg*	Normalized *SgFn*	Normalized *Sg*	Description
SGO_1469	-0.341	7.526	0.0005	0.0007	19.000	48.500	41.4090	50.4744	glmU; UDP-N-acetylglucosamine pyrophosphorylase
					17.000	52.500	39.8949	52.5000	
SGO_1472	1.625	4.942	0.0553	0.3203	8.500		18.5251		acetyltransferase, GNAT family
					3.500	4.000	8.2136	4.0000	
SGO_1486	2.124	3.744			5.000		10.8971		beta-galactosidase
						2.500		2.5000	
SGO_1487	-0.501	7.346	0.0093	0.0359	17.500	41.000	38.1399	42.6691	LPXTG cell wall surface protein, Cna protein B-type domain
					12.500	52.500	29.3345	52.5000	
SGO_1493	-1.485	7.194	0.0009	0.0015	8.000	47.000	17.4354	48.9134	hypothetical protein SGO_1493
					9.000	59.000	21.1208	59.0000	
SGO_1512	1.099	4.069	0.0412	0.2271	2.500	3.000	5.4486	3.1221	lacG; 6-phospho-beta-galactosidase
					3.500		8.2136		
SGO_1529	-1.110	6.071	0.0007	0.0011	5.500	21.000	11.9868	21.8549	hypothetical protein SGO_1529
					4.000	24.000	9.3870	24.0000	
SGO_1530	-0.620	8.210	0.0111	0.0443	32.500	95.500	70.8312	99.3878	methionine-tRNA ligase
					20.000	79.000	46.9351	79.0000	
SGO_1531	0.311	5.961	0.0299	0.1548	9.500	13.000	20.7045	13.5292	xth; exodeoxyribonuclease III
					6.000	14.000	14.0805	14.0000	
SGO_1534	1.195	5.714	0.0220	0.1038	8.000	3.500	17.4354	3.6425	ArsC family
					7.000	15.000	16.4273	15.0000	
SGO_1536	-0.922	6.083	0.0065	0.0228	6.500	18.000	14.1662	18.7328	conserved hypothetical protein TIGR00096
					4.000	25.500	9.3870	25.5000	
SGO_1539	0.151	6.166	0.0639	0.3839	10.500	16.000	22.8839	16.6514	tmk; thymidylate kinase
					6.500	17.000	15.2539	17.0000	

Log_2 Ratios: -6 -4 -2 0 2 4 6

Show detected proteins only
Show all proteins
Filter by category:
ABC Transporter

Proteins found: 769

Test	Cutoff
q-Value	.005
p-Value	

	Signif	Direction	Applies To
red	yes	+	ratios, bars
yellow	no	n/a	bars
green	yes	−	ratios, bars
pink	yes	+	*p*- , *q*-Values
light green	yes	−	*p*- , *q*-Values

Dot Plots Dot Plots

Protein	SgFn vs Sg: Log_2 Ratio	Log_2 Sum	*q*-Value	*p*-Value	Raw: *SgFn*	Raw: *Sg*	Normalized: *SgFn*	Normalized: *Sg*	Description
SGO_1541	-0.363	8.032	0.0624	0.3731	18.000	60.500	39.2296	62.9629	atpC; ATP synthase F1, epsilon subunit
					33.500	81.000	78.6164	81.0000	
SGO_1542	0.619	11.040	0.0001	0.0001	290.500	410.500	633.1221	427.2113	atpD; ATP synthase F1, beta subunit
					273.500	403.500	641.8380	403.5000	
SGO_1543	0.383	8.135	0.0131	0.0553	41.500	60.500	90.4460	62.9629	atpG; ATP synthase F1, gamma subunit
					29.500	58.500	69.2293	58.5000	
SGO_1544	-0.415	10.250	0.0008	0.0014	125.500	330.000	273.5175	343.4342	atpA; ATP synthase F1, alpha subunit
					106.000	352.000	248.7562	352.0000	
SGO_1545	0.015	8.292	0.1306	0.8681	33.000	80.000	71.9209	83.2568	atpH; ATP synthase F1, delta subunit
					36.500	72.500	85.6566	72.5000	
SGO_1546	-1.156	8.155	0.0064	0.0223	30.000	86.500	65.3827	90.0214	atpF; ATP synthase F0, B subunit
					12.000	101.500	28.1611	101.5000	
SGO_1550	0.171	7.857	0.0596	0.3503	25.000	45.000	54.4856	46.8319	glgP-1; glycogen phosphorylase
					29.000	62.500	68.0559	62.5000	
SGO_1551	2.324	7.034	0.0017	0.0041	25.500	5.000	55.5753	5.2035	glgA; Glycogen synthase
					21.000	21.000	49.2819	21.0000	
SGO_1552	1.390	7.624	0.0016	0.0035	35.500	32.500	77.3695	33.8231	glgD; glucose-1-phosphate adenylyltransferase, GlgD subunit
					27.500	21.500	64.5358	21.5000	
SGO_1553	1.980	7.868	0.0023	0.0062	50.500	18.500	110.0608	19.2531	glgC; glucose-1-phosphate adenylyltransferase
					32.500	28.000	76.2696	28.0000	
SGO_1554	2.537	8.474	0.0005	0.0007	75.000	19.000	163.4567	19.7735	glgB; 1,4-alpha-glucan branching enzyme
					59.000	34.000	138.4587	34.0000	
SGO_1555	0.328	11.311	0.0025	0.0069	329.500	575.500	718.1195	598.9284	ptsI; phosphoenolpyruvate-protein phosphotransferase
					296.000	528.500	694.6400	528.5000	

Log_2 Ratios: -6 -4 -2 0 2 4 6

Show detected proteins only
Show all proteins
Filter by category:
ABC Transporter

Proteins found: 769

Test	Cutoff
q-Value	.005
p-Value	

Color	Signif	Direction	Applies To
red	yes	+	ratios, bars
yellow	no	n/a	bars
green	yes	–	ratios, bars
pink	yes	+	*p*- , *q*-Values
light green	yes	–	*p*- , *q*-Values

Dot Plots Dot Plots

Streptococcus gordonii

	SgFn vs Sg				Raw		Normalized		
Protein	Log_2 Ratio	Log_2 Sum	*q*-Value	*p*-Value	*SgFn*	*Sg*	*SgFn*	*Sg*	Description
**SGO_1556**	0.623	11.703	0.0038	0.0116	515.500	646.000	1123.4920	672.2984	phosphocarrier protein HPr
					384.500	635.500	902.3280	635.5000	
**SGO_1558**	0.723	8.951	0.0056	0.0187	81.000	83.000	176.5332	86.3789	nrdE; ribonucleoside-diphosphate reductase large chain
					56.500	99.500	132.5918	99.5000	
**SGO_1559**	0.091	7.735	0.0544	0.3137	24.500	45.500	53.3958	47.3523	ribonucleoside-diphosphate reductase, beta subunit
					24.000	56.000	56.3222	56.0000	
**SGO_1570**	-0.511	9.201	0.0126	0.0527	65.000	146.500	141.6624	152.4640	alaS; alanyl-tRNA synthetase
					43.500	192.500	102.0839	192.5000	
**SGO_1572**	-4.463	9.699	0.0000	0.0000	11.500	377.500	25.0634	392.8679	proteinase maturation protein, putative
					5.500	400.500	12.9072	400.5000	
**SGO_1573**	1.276	3.945			5.000		10.8971		O-methyltransferase family protein
						4.500		4.5000	
**SGO_1574**	0.665	8.801	0.0015	0.0034	67.500	81.000	147.1110	84.2975	pepF-1; oligoendopeptidase F
					54.000	88.000	126.7249	88.0000	
**SGO_1587**	0.505	6.189	0.0397	0.2167	8.000	10.000	17.4354	10.4071	queA; S-adenosylmethionine:tRNA ribosyltransferase-isomerase
					10.500	20.500	24.6409	20.5000	
**SGO_1589**	2.726	6.450	0.0001	0.0000	17.000	4.500	37.0502	4.6832	arcT; putative transaminase/peptidase
					16.500	7.000	38.7215	7.0000	
**SGO_1590**	-1.292	7.433	0.0008	0.0014	13.500	55.500	29.4222	57.7594	arcD; arginine-ornithine antiporter
					9.000	64.500	21.1208	64.5000	
**SGO_1591**	1.169	8.010	0.0009	0.0015	40.000	31.500	87.1769	32.7824	arcC; carbamate kinase
					38.500	47.500	90.3501	47.5000	
**SGO_1592**	2.366	11.142	0.0009	0.0016	379.500	174.500	827.0906	181.6038	arcB; ornithine carbamoyltransferase
					455.000	183.000	1067.7744	183.0000	

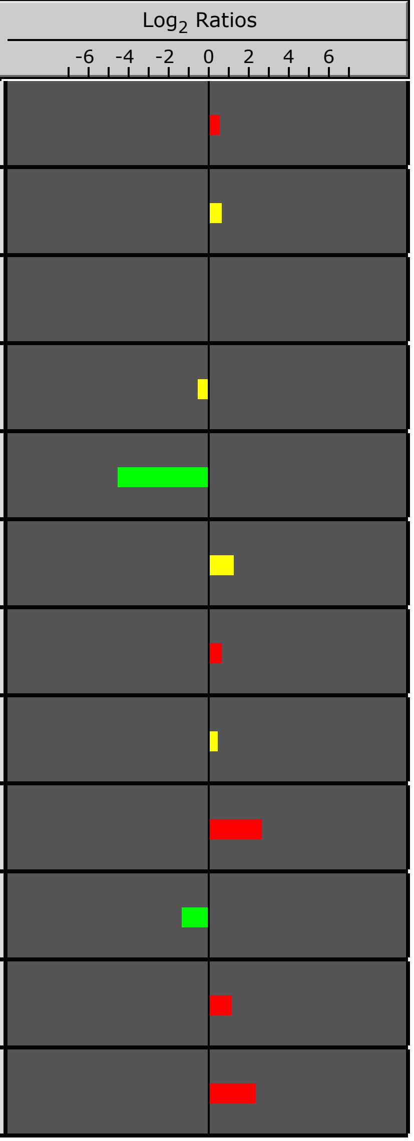

Show detected proteins only
Show all proteins
Filter by category:
ABC Transporter

Proteins found: 769

Test	Cutoff
***q*-Value**	**.005**
p-Value	

	Signif	Direction	Applies To
(red)	yes	+	ratios, bars
(yellow)	no	n/a	bars
(green)	yes	−	ratios, bars
(pink)	yes	+	***p*- , *q*-Values**
(light green)	yes	−	

Dot Plots Dot Plots

Protein	SgFn vs Sg Log_2 Ratio	Log_2 Sum	*q*-Value	*p*-Value	Raw *SgFn*	Raw *Sg*	Normalized *SgFn*	Normalized *Sg*	Description
SGO_1593	1.771	10.509	0.0002	0.0001	270.000	148.500	588.4439	154.5454	arcA; arginine deiminase
					229.500	176.000	538.5807	176.0000	
SGO_1599	2.054	10.580	0.0002	0.0002	298.500	135.500	650.5575	141.0162	sodA; manganese-dependent superoxide dismutase
					248.500	156.000	583.1691	156.0000	
SGO_1604	-1.738	6.821	0.0006	0.0009	6.000	38.000	13.0765	39.5470	acyltransferase family protein
					5.500	47.500	12.9072	47.5000	
SGO_1605	-3.386	6.286	0.0004	0.0005	1.500	31.000	3.2691	32.2620	P-type ATPase, metal cation transport
					1.500	39.000	3.5201	39.0000	
SGO_1609	-2.177	7.848	0.0001	0.0000	10.000	88.500	21.7942	92.1028	ATP-dependent RNA helicase, DEAD/DEAH box family
					8.500	96.500	19.9474	96.5000	
SGO_1617	0.495	6.407	0.0360	0.1921	9.500	12.000	20.7045	12.4885	prfC; peptide chain release factor 3
					12.000	23.500	28.1611	23.5000	
SGO_1619	-2.956	9.529	0.0009	0.0016	19.000	357.500	41.4090	372.0537	cation-transporting ATPase, E1-E2 family
					18.000	283.000	42.2416	283.0000	
SGO_1621	-0.202	5.929	0.0227	0.1077	6.000	14.500	13.0765	15.0903	HD domain protein
					6.500	17.500	15.2539	17.5000	
SGO_1622	1.367	6.408	0.0013	0.0026	15.000	14.000	32.6913	14.5699	Cof family protein
					12.000	9.500	28.1611	9.5000	
SGO_1623	-0.490	6.963	0.0014	0.0030	12.500	33.500	27.2428	34.8638	murM; MurM
					10.500	38.000	24.6409	38.0000	
SGO_1624	0.137	6.102	0.1018	0.6598	10.000	19.500	21.7942	20.2938	murN; MurN protein
					6.000	12.500	14.0805	12.5000	
SGO_1625	-0.506	8.384	0.0006	0.0009	30.000	95.000	65.3827	98.8674	acetoin utilization putative/CBS domain protein
					31.000	97.000	72.7495	97.0000	

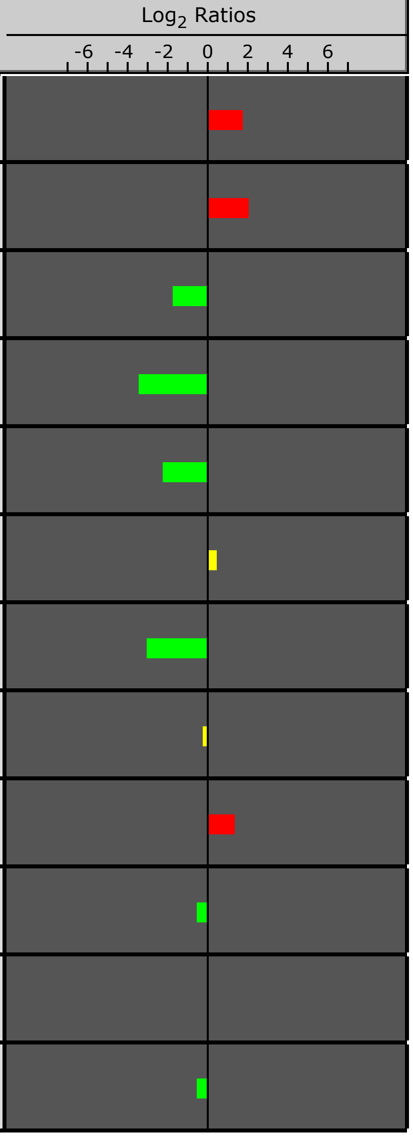

Show detected proteins only
Show all proteins
Filter by category:
ABC Transporter

Proteins found: 769

Test	Cutoff
q-Value	.005
p-Value	

	Signif	Direction	Applies To
red	yes	+	ratios, bars
yellow	no	n/a	bars
green	yes	–	ratios, bars
pink	yes	+	*p*- , *q*-Values
light green	yes	–	*p*- , *q*-Values

Dot Plots Dot Plots

Protein	SgFn vs Sg Log_2 Ratio	Log_2 Sum	*q*-Value	*p*-Value	Raw *SgFn*	Raw *Sg*	Normalized *SgFn*	Normalized *Sg*	Description	Log_2 Ratios -6 -4 -2 0 2 4 6
SGO_1626	-2.040	6.616	0.0213	0.0998		37.000		38.5063	branched-chain amino acid ABC transporter, ATP-binding protein	
					4.500	49.000	10.5604	49.0000		
SGO_1630	-3.259	10.217	0.0000	0.0000	27.500	509.000	59.9341	529.7212	branched-chain amino acid ABC transporter, amino acid-binding protein	
					22.500	547.500	52.8020	547.5000		
SGO_1632	1.202	8.084	0.0031	0.0090	36.500	37.500	79.5489	39.0266	clpP; ATP-dependent Clp protease, proteolytic subunit ClpP	
					47.000	42.500	110.2976	42.5000		
SGO_1633	1.254	8.358	0.0018	0.0044	60.000	45.000	130.7653	46.8319	upp; uracil phosphoribosyltransferase	
					43.000	49.500	100.9105	49.5000		
SGO_1634	-4.978	9.104	0.0000	0.0000	4.000	256.500	8.7177	266.9420	magnesium-translocating P-type ATPase	
					3.500	266.500	8.2136	266.5000		
SGO_1638	1.909	5.438	0.0023	0.0061	6.500	5.500	14.1662	5.7239	murE; UDP-N-acetylmuramoylalanyl-D-glutamate--2,6-diaminopimelate ligase	
					8.500	3.500	19.9474	3.5000		
SGO_1648	0.910	8.722	0.0009	0.0016	62.000	61.500	135.1242	64.0036	ppx1; inorganic pyrophosphatase, manganese-dependent	
					59.500	83.500	139.6320	83.5000		
SGO_1649	-0.303	6.470	0.0116	0.0477	9.000	26.000	19.6148	27.0585	act; pyruvate formate-lyase-activating enzyme	
					8.500	22.000	19.9474	22.0000		
SGO_1651	-2.162	9.827	0.0000	0.0000	39.000	357.000	84.9975	371.5333	LPXTG cell wall surface protein, nuclease/phosphatase domain	
					34.500	371.000	80.9631	371.0000		
SGO_1652	-0.823	6.776	0.0058	0.0195	10.000	28.500	21.7942	29.6602	intracellular glycosyl hydrolase	
					7.500	40.500	17.6007	40.5000		
SGO_1653	-2.067	8.997	0.0002	0.0002	20.500	187.500	44.6782	195.1331	trehalose PTS enzyme II	
					23.000	217.000	53.9754	217.0000		
SGO_1655	-1.918	7.001	0.0023	0.0060	4.500	40.500	9.8074	42.1487	CBS domain protein/possible hemolysin	
					7.500	58.500	17.6007	58.5000		

Show detected proteins only
Show all proteins
Filter by category:
ABC Transporter

Proteins found: 769

Test	Cutoff
***q*-Value**	**.005**
p-Value	

Color	Signif	Direction	Applies To
red	yes	+	ratios, bars
yellow	no	n/a	bars
green	yes	−	ratios, bars
pink	yes	+	*p*- , *q*-Values
light green	yes	−	*p*- , *q*-Values

Dot Plots Dot Plots

Protein	SgFn vs Sg Log2 Ratio	Log2 Sum	q-Value	p-Value	Raw SgFn	Raw Sg	Normalized SgFn	Normalized Sg	Description
SGO_1661	0.772	4.627	0.0794	0.4944		3.500		3.6425	radical SAM protein, TIGR01212 family
					4.500	10.500	10.5604	10.5000	
SGO_1666	0.784	7.821	0.0022	0.0057	33.000	33.500	71.9209	34.8638	trkA; potassium uptake protein, Trk family
					30.000	49.000	70.4027	49.0000	
SGO_1669	-0.888	7.256	0.0099	0.0386	15.000	38.500	32.6913	40.0673	ribosomal large subunit pseudouridine synthase B
					9.000	59.000	21.1208	59.0000	
SGO_1674	0.340	5.823	0.0050	0.0159	7.500	13.000	16.3457	13.5292	phosphodiesterase, MJ0936 family
					6.500	11.500	15.2539	11.5000	
SGO_1675	0.255	6.690	0.0603	0.3569	11.500	17.500	25.0634	18.2124	HAM1 protein-like protein
					13.000	29.500	30.5078	29.5000	
SGO_1676	0.999	5.350	0.0014	0.0030	6.500	5.500	14.1662	5.7239	murI; glutamate racemase
					5.500	8.000	12.9072	8.0000	
SGO_1678	1.280	6.005	0.0026	0.0070	9.000	7.500	19.6148	7.8053	lysA; diaminopimelate decarboxylase
					11.000	11.000	25.8143	11.0000	
SGO_1679	-2.373	10.552	0.0000	0.0000	60.000	608.000	130.7653	632.7514	phosphotransferase system enzyme II
					48.000	625.000	112.6443	625.0000	
SGO_1681	-2.678	10.136	0.0001	0.0000	30.500	471.500	66.4724	490.6946	PTS system, mannose/fructose/sorbose family, IID component
					37.000	481.500	86.8300	481.5000	
SGO_1683	0.183	8.135	0.0676	0.4105	44.500	59.500	96.9843	61.9222	serS; seryl-tRNA synthetase
					23.500	67.000	55.1488	67.0000	
SGO_1685	1.132	6.834	0.0008	0.0013	17.500	14.500	38.1399	15.0903	putative peroxidase / antioxidase
					17.000	21.000	39.8949	21.0000	
SGO_1687	-0.326	7.903	0.0149	0.0652	28.000	62.000	61.0238	64.5240	accA; acetyl-CoA carboxylase, carboxyl transferase, alpha subunit
					19.500	68.000	45.7618	68.0000	

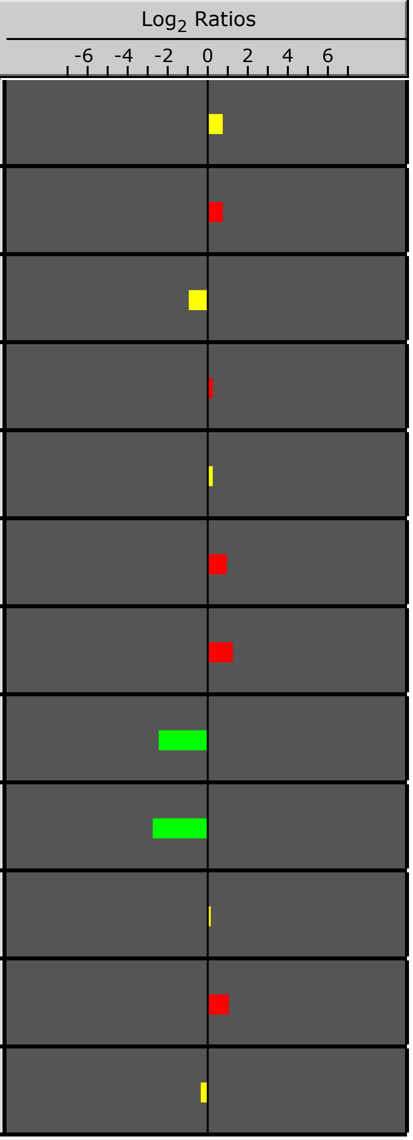

Show detected proteins only
Show all proteins
Filter by category:
ABC Transporter

Proteins found: 769

Test	Cutoff
q-Value	.005
p-Value	

Color	Signif	Direction	Applies To
red	yes	+	ratios, bars
yellow	no	n/a	bars
green	yes	–	ratios, bars
pink	yes	+	p- , q-Values
light green	yes	–	p- , q-Values

Dot Plots Dot Plots

Protein	SgFn vs Sg Log$_2$ Ratio	Log$_2$ Sum	*q*-Value	*p*-Value	Raw SgFn	Raw Sg	Normalized SgFn	Normalized Sg	Description
SGO_1688	0.081	6.923	0.1412	0.9507	14.000	20.500	30.5119	21.3345	accD; acetyl-CoA carboxylase, carboxyl transferase, beta subunit
					13.000	39.000	30.5078	39.0000	
SGO_1689	1.308	7.985	0.0008	0.0013	38.500	30.500	83.9077	31.7416	accC; acetyl-CoA carboxylase, biotin carboxylase
					41.000	41.500	96.2170	41.5000	
SGO_1691	-0.102	7.422	0.1200	0.7870	24.500	55.000	53.3958	57.2390	accB; acetyl-CoA carboxylase, biotin carboxyl carrier protein
					12.500	31.500	29.3345	31.5000	
SGO_1692	2.086	10.166	0.0004	0.0004	197.500	101.000	430.4358	105.1117	3-oxoacyl-[acyl-carrier-protein] synthase
					213.000	113.500	499.8592	113.5000	
SGO_1693	1.153	7.330	0.0003	0.0003	25.000	26.500	54.4856	27.5788	fabG; 3-oxoacyl-(acyl-carrier-protein) reductase
					24.000	22.500	56.3222	22.5000	
SGO_1694	0.376	6.814	0.0294	0.1515	12.000	20.000	26.1531	20.8142	fabD; malonyl CoA-acyl carrier protein transacylase
					16.000	28.000	37.5481	28.0000	
SGO_1695	0.088	9.310	0.0294	0.1501	71.000	147.500	154.7390	153.5047	enoyl-acyl carrier protein(ACP) reductase
					73.500	154.000	172.4866	154.0000	
SGO_1699	1.460	6.054	0.0002	0.0002	11.000	7.500	23.9736	7.8053	transcriptional regulator, MarR family
					10.500	10.000	24.6409	10.0000	
SGO_1700	2.949	8.270	0.0006	0.0010	55.500	17.000	120.9579	17.6921	enoyl-CoA hydratase/isomerase family protein
					65.000	17.500	152.5392	17.5000	
SGO_1701	0.868	7.787	0.0013	0.0027	30.000	36.500	65.3827	37.9859	aspartate kinase
					33.000	40.000	77.4430	40.0000	
SGO_1708	-2.226	9.714	0.0047	0.0146	36.500	424.000	79.5489	441.2609	amiF; Oligopeptide transport ATP-binding protein amiF
					27.500	254.500	64.5358	254.5000	
SGO_1709	-3.054	9.803	0.0000	0.0000	20.000	381.500	43.5884	397.0307	amiE; Oligopeptide transport ATP-binding protein
					22.500	400.000	52.8020	400.0000	

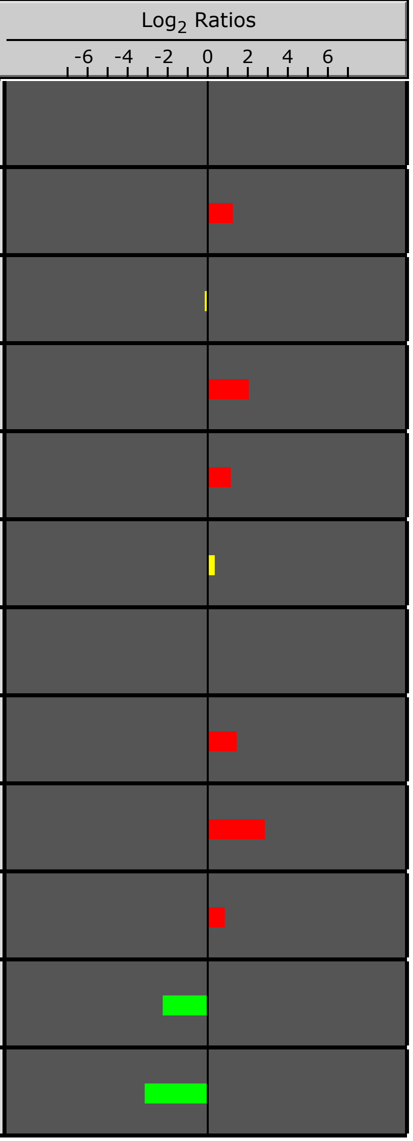

Show detected proteins only
Show all proteins
Filter by category:
ABC Transporter

Proteins found: 769

Test	Cutoff
q-Value	.005
p-Value	

	Signif	Direction	Applies To
red	yes	+	ratios, bars
yellow	no	n/a	bars
green	yes	–	ratios, bars
pink	yes	+	*p*- , *q*-Values
light green	yes	–	*p*- , *q*-Values

Dot Plots Dot Plots

Hendrickson *et al.*

Protein	SgFn vs Sg Log$_2$ Ratio	Log$_2$ Sum	*q*-Value	*p*-Value	Raw *SgFn*	Raw *Sg*	Normalized *SgFn*	Normalized *Sg*	Description
SGO_1711	-2.691	8.366	0.0007	0.0012	10.500	121.000	22.8839	125.9259	hppB; Oligopeptide transport system permease
					9.000	160.000	21.1208	160.0000	
SGO_1712	-1.923	10.854	0.0003	0.0003	111.000	712.500	241.9158	741.5056	hppA; oligopeptide-binding lipoprotein
					65.000	715.500	152.5392	715.5000	
SGO_1713	-1.214	10.762	0.0008	0.0013	137.500	615.000	299.6705	640.0364	hppG; oligopeptide-binding lipoprotein
					96.500	570.500	226.4620	570.5000	
SGO_1715	-1.347	9.552	0.0001	0.0000	51.000	263.500	111.1505	274.2270	hppH; oligopeptide-binding lipoprotein
					43.000	264.500	100.9105	264.5000	
SGO_1716	-0.893	9.573	0.0026	0.0072	70.000	215.000	152.5595	223.7526	oligopeptide binding protein
					49.000	270.500	114.9911	270.5000	
SGO_1717	-2.333	6.693	0.0016	0.0037	3.500	48.500	7.6280	50.4744	pbp3; penicillin-binding protein 3
					4.000	36.000	9.3870	36.0000	
SGO_1718	-0.376	7.137	0.0406	0.2227	11.000	31.000	23.9736	32.2620	sufB-1; FeS assembly protein SufB
					16.000	47.000	37.5481	47.0000	
SGO_1721	0.391	7.322	0.0192	0.0878	24.000	36.500	52.3061	37.9859	sufD; FeS assembly protein SufD
					16.500	31.000	38.7215	31.0000	
SGO_1722	0.722	6.904	0.0017	0.0039	18.000	19.500	39.2296	20.2938	sufC; FeS assembly ATPase SufC
					15.000	25.000	35.2014	25.0000	
SGO_1724	0.591	4.101	0.0794	0.4935		3.000		3.1221	mecA; Adapter protein mec
					3.000	7.000	7.0403	7.0000	
SGO_1727	-1.722	7.502	0.0003	0.0003	11.500	64.500	25.0634	67.1258	amino acid ABC transporter, amino acid-binding/permease protein
					7.500	71.500	17.6007	71.5000	
SGO_1728	-0.925	7.589	0.0068	0.0241	14.500	73.000	31.6016	75.9718	glnQ; glutamine ABC transporter ATP-binding protein
					14.500	51.000	34.0280	51.0000	

Log$_2$ Ratios: -6 -4 -2 0 2 4 6

Show detected proteins only
Show all proteins
Filter by category: ABC Transporter

Proteins found: 769

Test	Cutoff
q-Value	.005
p-Value	

	Signif	Direction	Applies To
red	yes	+	ratios, bars
yellow	no	n/a	bars
green	yes	–	ratios, bars
pink	yes	+	*p*- , *q*-Values
light green	yes	–	*p*- , *q*-Values

Dot Plots | Dot Plots

Streptococcus gordonii

Protein	SgFn vs Sg Log₂ Ratio	Log₂ Sum	*q*-Value	*p*-Value	Raw *SgFn*	Raw *Sg*	Normalized *SgFn*	Normalized *Sg*	Description
SGO_1730	0.138	10.208	0.0639	0.3830	163.500	292.500	356.3355	304.4076	SPFH domain/Band 7 family
					113.000	256.500	265.1835	256.5000	
SGO_1731	0.649	4.113	0.0366	0.1959	2.000	2.000	4.3588	2.0814	DNA-binding response regulator
					2.500	5.000	5.8669	5.0000	
SGO_1735	-0.145	9.384	0.0589	0.3452	82.500	155.500	179.8023	161.8303	hypothetical protein SGO_1735
					59.000	188.000	138.4587	188.0000	
SGO_1736	1.192	6.293	0.0068	0.0244	15.500	12.500	33.7810	13.0089	alkaline shock protein
					9.000	10.500	21.1208	10.5000	
SGO_1738	-0.253	4.817	0.0847	0.5362		11.500		11.9682	hypothetical protein SGO_1738
					3.500	8.000	8.2136	8.0000	
SGO_1739	-1.358	4.332	0.0238	0.1138	1.500	9.000	3.2691	9.3664	LytTr DNA-binding domain family
						7.500		7.5000	
SGO_1744	-0.225	4.863	0.0260	0.1304	4.000	9.500	8.7177	9.8867	ABC transporter, ATP-binding protein SP0636
						10.500		10.5000	
SGO_1745	0.223	11.665	0.0272	0.1384	422.000	810.500	919.7161	843.4951	fba; fructose-1,6-bisphosphate aldolase, class II
					351.000	659.500	823.7117	659.5000	
SGO_1748	-0.482	4.963	0.0203	0.0936		11.500		11.9682	pyrG; CTP synthase
					3.500	11.000	8.2136	11.0000	
SGO_1749	0.564	6.940	0.0184	0.0837	19.500	19.000	42.4987	19.7735	manA; mannose-6-phosphate isomerase, class I
					13.000	30.000	30.5078	30.0000	
SGO_1755	-0.956	6.493	0.0018	0.0044	7.000	25.500	15.2560	26.5381	scrK; fructokinase
					6.500	33.000	15.2539	33.0000	
SGO_1757	-0.656	8.406	0.0149	0.0651	38.000	84.500	82.8180	87.9400	glmS; glucosamine--fructose-6-phosphate aminotransferase
					21.500	118.000	50.4553	118.0000	

Log₂ Ratios: -6 -4 -2 0 2 4 6

Show detected proteins only
Show all proteins
Filter by category:
ABC Transporter

Proteins found: 769

Test	Cutoff
***q*-Value**	**.005**
p-Value	

	Signif	Direction	Applies To
	yes	+	ratios, bars
	no	n/a	bars
	yes	–	ratios, bars
	yes	+	*p*- , *q*-Values
	yes	–	*p*- , *q*-Values

Dot Plots | Dot Plots

Protein	SgFn vs Sg Log_2 Ratio	Log_2 Sum	*q*-Value	*p*-Value	Raw *SgFn*	Raw *Sg*	Normalized *SgFn*	Normalized *Sg*	Description
SGO_1763	-0.583	7.656	0.0056	0.0188	21.000	53.000	45.7679	55.1576	ABC transporter, substrate-binding protein SP0092
					15.000	65.500	35.2014	65.5000	
SGO_1768	3.248	6.256	0.0004	0.0004	17.000	5.000	37.0502	5.2035	glycosyl hydrolase, family 38
					13.500	2.500	31.6812	2.5000	
SGO_1774	1.620	6.691	0.0002	0.0002	18.500	11.000	40.3193	11.4478	alcohol dehydrogenase, zinc-containing
					16.000	14.000	37.5481	14.0000	
SGO_1780	1.231	3.065							hypothetical protein SGO_1780
					2.500	2.500	5.8669	2.5000	
SGO_1784	0.353	8.245	0.0126	0.0527	42.500	57.500	92.6254	59.8408	leuS; leucyl-tRNA synthetase
					33.000	73.500	77.4430	73.5000	
SGO_1792	0.124	2.648			1.500		3.2691		transcription regulator
						3.000		3.0000	
SGO_1799	2.086	9.301	0.0001	0.0000	117.500	50.500	256.0821	52.5558	endopeptidase O
					108.000	68.500	253.4497	68.5000	
SGO_1802	-6.178	11.051	0.0003	0.0003	9.000	1088.500	19.6148	1132.8124	Metal ABC transporter substrate-binding lipoprotein precursor
					4.500	958.000	10.5604	958.0000	
SGO_1803	1.164	6.483	0.0010	0.0018	15.500	13.000	33.7810	13.5292	tpx; thioredoxin peroxidase
					12.000	14.000	28.1611	14.0000	
SGO_1804	1.496	4.317	0.0079	0.0293	4.000		8.7177		hutI; imidazolonepropionase
					3.500	3.000	8.2136	3.0000	
SGO_1805	1.066	2.272			1.500	1.500	3.2691	1.5611	hutU; urocanate hydratase
SGO_1811	1.763	4.707	0.0030	0.0087		4.500		4.6832	hutH; histidine ammonia-lyase
					7.000	5.000	16.4273	5.0000	

Log_2 Ratios: -6 -4 -2 0 2 4 6

Show detected proteins only
Show all proteins
Filter by category:
ABC Transporter

Proteins found: 769

Test	Cutoff
***q*-Value**	**.005**
p-Value	

	Signif	Direction	Applies To
red	yes	+	ratios, bars
yellow	no	n/a	bars
green	yes	−	ratios, bars
pink	yes	+	*p*- , *q*-Values
light green	yes	−	*p*- , *q*-Values

Dot Plots Dot Plots

Hendrickson *et al.*

Protein	SgFn vs Sg Log_2 Ratio	Log_2 Sum	*q*-Value	*p*-Value	Raw *SgFn*	Raw *Sg*	Normalized *SgFn*	Normalized *Sg*	Description
SGO_1813	0.124	3.063			2.000		4.3588		hutG; formimidoylglutamase
						4.000		4.0000	
SGO_1814	1.632	5.980	0.0028	0.0079	9.000	8.500	19.6148	8.8460	putative regulatory protein
					12.000	6.500	28.1611	6.5000	
SGO_1816	0.877	5.790	0.0044	0.0135	9.500	9.000	20.7045	9.3664	scaR; ScaR Manganese-dependent regulator of scaCBA
					6.500	10.000	15.2539	10.0000	
SGO_1822	0.126	7.384	0.0609	0.3615	17.500	41.000	38.1399	42.6691	relA; GTP diphosphokinase
					21.000	37.000	49.2819	37.0000	
SGO_1824	0.490	5.870	0.0226	0.1071	6.500	9.500	14.1662	9.8867	prmA; ribosomal protein L11 methyltransferase
					8.500	14.500	19.9474	14.5000	
SGO_1827	0.838	5.024	0.0259	0.1289	7.000	7.000	15.2560	7.2850	hypothetical protein SGO_1827
						10.000		10.0000	
SGO_1828	0.994	5.002	0.0116	0.0472	6.000	6.500	13.0765	6.7646	ATPase, AAA family
					3.500	4.000	8.2136	4.0000	
SGO_1834	5.178	8.827	0.0008	0.0014	116.000	7.000	252.8130	7.2850	hypothetical protein SGO_1834
					80.500	5.000	188.9139	5.0000	
SGO_1835	2.787	4.567			9.500		20.7045		hypothetical protein SGO_1835
						3.000		3.0000	
SGO_1843	0.982	8.061	0.0002	0.0001	41.500	45.500	90.4460	47.3523	pepS; aminopeptidase PepS
					37.000	42.500	86.8300	42.5000	
SGO_1844	0.367	5.531	0.0241	0.1161	6.500	8.000	14.1662	8.3257	cbxX/cfqX family protein
					5.000	12.000	11.7338	12.0000	
SGO_1847	0.427	6.251	0.0392	0.2126	7.000	14.000	15.2560	14.5699	polC; DNA polymerase III, alpha subunit, Gram-positive type
					12.500	17.000	29.3345	17.0000	

Log2 Ratios: -6 -4 -2 0 2 4 6

Show detected proteins only
Show all proteins
Filter by category:
ABC Transporter

Proteins found: 769

Test	Cutoff
q-Value	.005
p-Value	

Colour	Signif	Direction	Applies To
red	yes	+	ratios, bars
yellow	no	n/a	bars
green	yes	−	ratios, bars
pink	yes	+	*p*- , *q*-Values
light green	yes	−	*p*- , *q*-Values

Dot Plots Dot Plots

Hendrickson *et al.*

Streptococcus gordonii

Protein	SgFn vs Sg Log2 Ratio	Log2 Sum	*q*-Value	*p*-Value	Raw *SgFn*	Raw *Sg*	Normalized *SgFn*	Normalized *Sg*	Description
SGO_1851	0.331	8.944	0.0137	0.0588	69.500	96.500	151.4698	100.4285	proS; prolyl-tRNA synthetase
					52.500	117.500	123.2047	117.5000	
SGO_1854	-1.824	6.877	0.0003	0.0003	5.000	45.500	10.8971	47.3523	uppS; undecaprenyl diphosphate synthase
					6.500	44.000	15.2539	44.0000	
SGO_1856	2.577	8.999	0.0055	0.0181	132.500	33.500	288.7734	34.8638	ATP-dependent proteinase ATP-binding chain
					65.000	35.500	152.5392	35.5000	
SGO_1858	2.081	4.249	0.0188	0.0855	3.500		7.6280		beta-fructofuranosidase/sucrose 6 phoshate hydrolase
					4.000	2.000	9.3870	2.0000	
SGO_1860	-0.612	9.299	0.0202	0.0926	74.500	204.500	162.3669	212.8251	5'-nucleotidase, lipoprotein e(P4) family
					39.000	163.000	91.5235	163.0000	
SGO_1862	2.719	8.218	0.0003	0.0004	54.500	21.000	118.7785	21.8549	alkaline shock protein
					59.500	17.500	139.6320	17.5000	
SGO_1863	-0.622	8.044	0.0044	0.0134	27.500	72.000	59.9341	74.9311	efp; Elongation factor P (EF-P)
					19.000	84.500	44.5884	84.5000	
SGO_1864	1.965	8.210	0.0001	0.0001	53.000	33.500	115.5094	34.8638	X-Pro aminopeptidase
					51.000	26.000	119.6846	26.0000	
SGO_1865	-0.360	7.560	0.0050	0.0160	17.500	48.500	38.1399	50.4744	uvrA; excinuclease ABC, A subunit
					19.000	55.500	44.5884	55.5000	
SGO_1867	-3.307	7.508	0.0003	0.0003	3.000	85.000	6.5383	88.4603	hypothetical protein SGO_1867
					4.500	76.500	10.5604	76.5000	
SGO_1878	0.367	4.391	0.0261	0.1310		6.500		6.7646	transcriptional regulator, TetR family domain protein
					3.500	6.000	8.2136	6.0000	
SGO_1879	0.354	7.776	0.0886	0.5656	23.500	28.000	51.2164	29.1399	rpsR; ribosomal protein S18
					28.500	72.000	66.8826	72.0000	

Log_2 Ratios

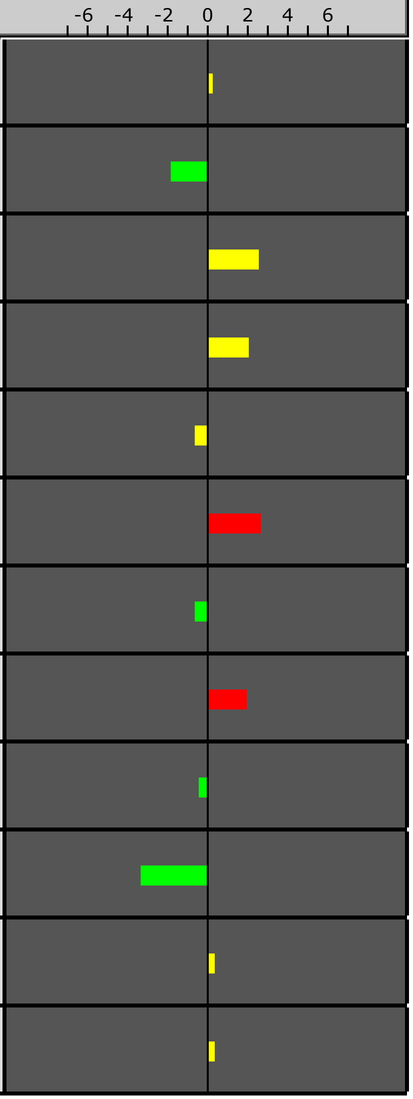

Show detected proteins only
Show all proteins
Filter by category:
ABC Transporter

Proteins found: 769

Test	Cutoff
q-Value	.005
p-Value	

	Signif	Direction	Applies To
(red)	yes	+	ratios, bars
(yellow)	no	n/a	bars
(green)	yes	–	ratios, bars
(pink)	yes	+	*p*- , *q*-Values
(light green)	yes	–	*p*- , *q*-Values

Dot Plots Dot Plots

Hendrickson *et al.*

Protein	SgFn vs Sg Log$_2$ Ratio	Log$_2$ Sum	*q*-Value	*p*-Value	Raw *SgFn*	Raw *Sg*	Normalized *SgFn*	Normalized *Sg*	Description
SGO_1880	-0.115	7.779	0.0501	0.2847	24.000	49.000	52.3061	50.9948	ssb-1; single-strand binding protein
					22.500	63.500	52.8020	63.5000	
SGO_1881	-0.382	9.153	0.0271	0.1376	44.500	146.500	96.9843	152.4640	rpsF; ribosomal protein S6
					65.500	166.000	153.7126	166.0000	
SGO_1882	1.171	5.749	0.0060	0.0204	10.000	6.000	21.7942	6.2443	folE; GTP cyclohydrolase I
					6.500	10.500	15.2539	10.5000	
SGO_1885	3.284	12.092	0.0000	0.0000	909.500	198.500	1982.1843	206.5809	groL; 60 kDa chaperonin/groEL protein
					842.500	200.000	1977.1427	200.0000	
SGO_1886	1.601	7.386	0.0137	0.0590	17.500	20.000	38.1399	20.8142	groES; chaperonin, 10 kDa
					38.500	18.000	90.3501	18.0000	
SGO_1892	2.240	8.409	0.0031	0.0089	79.000	24.500	172.1743	25.4974	PTS system, fructose(mannose)-specific IIB
					46.500	33.000	109.1242	33.0000	
SGO_1898	2.530	9.291	0.0004	0.0005	111.500	42.000	243.0056	43.7098	glutamyl aminopeptidase
					124.000	48.500	290.9979	48.5000	
SGO_1901	-0.053	6.001	0.1383	0.9288	10.000	17.000	21.7942	17.6921	tRNA binding domain
					4.500	14.000	10.5604	14.0000	
SGO_1903	0.470	8.382	0.0109	0.0434	51.000	64.000	111.1505	66.6054	ATP-dependent Zn protease
					35.500	72.500	83.3099	72.5000	
SGO_1912	2.439	4.026	0.0117	0.0484		1.500		1.5611	Bifunctional folate synthesis protein
					5.000	3.000	11.7338	3.0000	
SGO_1914	1.677	4.472	0.0060	0.0203	4.500		9.8074		folP; dihydropteroate synthase
					4.000	3.000	9.3870	3.0000	
SGO_1916	0.061	8.349	0.0764	0.4729	37.500	70.500	81.7283	73.3700	ackA; acetate kinase
					36.000	86.500	84.4832	86.5000	

Log$_2$ Ratios: -6 -4 -2 0 2 4 6

Show detected proteins only
Show all proteins
Filter by category:
ABC Transporter

Proteins found: 769

Test	Cutoff
q-Value	.005
p-Value	

Color	Signif	Direction	Applies To
red	yes	+	ratios, bars
yellow	no	n/a	bars
green	yes	–	ratios, bars
pink	yes	+	*p*- , *q*-Values
light green	yes	–	*p*- , *q*-Values

Dot Plots Dot Plots

Protein	SgFn vs Sg Log_2 Ratio	Log_2 Sum	*q*-Value	*p*-Value	Raw *SgFn*	Raw *Sg*	Normalized *SgFn*	Normalized *Sg*	Description	Log_2 Ratios (-6 -4 -2 0 2 4 6)
SGO_1917	0.787	5.009	0.0052	0.0167	4.000	6.000	8.7177	6.2443	hypothetical protein SGO_1917	
					5.000	5.500	11.7338	5.5000		
SGO_1926	-0.191	10.388	0.0027	0.0076	146.500	333.500	319.2853	347.0767	rpoC; DNA-directed RNA polymerase, beta chain	
					130.500	367.000	306.2518	367.0000		
SGO_1927	-0.581	10.200	0.0164	0.0731	141.000	347.000	307.2985	361.1262	rpoB; DNA-directed RNA polymerase, beta subunit	
					74.500	333.000	174.8334	333.0000		
SGO_1929	-0.009	8.729	0.1251	0.8259	48.000	97.000	104.6123	100.9488	tyrS; tyrosyl-tRNA synthetase	
					45.500	112.000	106.7774	112.0000		
SGO_1936	-3.425	8.816	0.0000	0.0000	9.000	193.500	19.6148	201.3773	adcA; metal-binding (Mn) permease precursor, lipoprotein	
					8.000	211.000	18.7741	211.0000		
SGO_1957	-0.388	5.582	0.0561	0.3268	3.500	9.000	7.6280	9.3664	hypothetical protein SGO_1957	
					5.500	18.000	12.9072	18.0000		
SGO_1958	-1.095	8.424	0.0024	0.0066	27.500	127.000	59.9341	132.1701	rplQ; ribosomal protein L17	
					21.000	102.000	49.2819	102.0000		
SGO_1959	0.029	9.157	0.1289	0.8527	61.000	152.000	132.9447	158.1879	rpoA; DNA-directed RNA polymerase, alpha subunit	
					66.000	125.000	154.8860	125.0000		
SGO_1960	-0.204	7.854	0.0710	0.4360	30.500	64.500	66.4724	67.1258	rpsK; ribosomal protein S11	
					18.000	55.500	42.2416	55.5000		
SGO_1961	-1.173	8.386	0.0006	0.0010	27.000	115.500	58.8444	120.2020	rpsM; ribosomal protein S13p/S18e	
					19.000	111.000	44.5884	111.0000		
SGO_1964	-0.394	8.593	0.0246	0.1193	47.500	106.500	103.5225	110.8356	adk; Adenylate kinase (ATP-AMP transphosphorylase)	
					28.000	106.000	65.7092	106.0000		
SGO_1966	0.711	8.581	0.0250	0.1218	75.000	64.500	163.4567	67.1258	rplO; ribosomal protein L15	
					34.000	72.500	79.7897	72.5000		

Show detected proteins only
Show all proteins
Filter by category:
ABC Transporter

Proteins found: 769

Test: ***q*-Value** / *p*-Value — Cutoff: .005

	Signif	Direction	Applies To
	yes	+	ratios, bars
	no	n/a	bars
	yes	−	ratios, bars
	yes	+	*p*- , *q*-Values
	yes	−	*p*- , *q*-Values

Dot Plots Dot Plots

Streptococcus gordonii

Hackett Laboratory UW

Protein	SgFn vs Sg Log$_2$ Ratio	Log$_2$ Sum	*q*-Value	*p*-Value	Raw *SgFn*	Raw *Sg*	Normalized *SgFn*	Normalized *Sg*	Description
SGO_1967	-0.238	7.392	0.0767	0.4756	13.000	42.000	28.3325	43.7098	50S ribosomal protein L30 -related protein
					21.500	45.500	50.4553	45.5000	
SGO_1968	-0.621	9.665	0.0078	0.0289	84.000	264.500	183.0714	275.2677	rpsE; ribosomal protein S5
					58.500	216.000	137.2853	216.0000	
SGO_1969	-0.461	9.203	0.0251	0.1230	71.000	182.000	154.7390	189.4092	rplR; ribosomal protein L18
					41.000	149.000	96.2170	149.0000	
SGO_1970	-1.812	9.837	0.0001	0.0001	52.000	349.000	113.3299	363.2077	BL10; 50S ribosomal protein L6
					38.500	347.500	90.3501	347.5000	
SGO_1971	-0.883	9.958	0.0005	0.0007	79.000	326.500	172.1743	339.7917	rpsH; ribosomal protein S8
					75.500	305.500	177.1801	305.5000	
SGO_1973	-0.823	9.445	0.0007	0.0011	60.500	202.500	131.8550	210.7437	BL6; 50S ribosomal protein L5
					51.000	234.500	119.6846	234.5000	
SGO_1974	-1.058	8.620	0.0046	0.0142	34.500	147.000	75.1901	152.9843	rplX; ribosomal protein L24
					22.500	112.500	52.8020	112.5000	
SGO_1975	-0.135	8.279	0.0814	0.5114	40.500	80.500	88.2666	83.7771	rplN; ribosomal protein L14
					26.000	77.500	61.0157	77.5000	
SGO_1976	0.116	7.182	0.1315	0.8772	14.500	45.500	31.6016	47.3523	BS16; 30S ribosomal protein
					18.000	24.000	42.2416	24.0000	
SGO_1977	-0.463	5.951	0.0523	0.2993	7.500	25.000	16.3457	26.0177	rpmC; ribosomal protein L29
						19.500		19.5000	
SGO_1978	-0.239	7.841	0.0607	0.3602	25.000	42.500	54.4856	44.2302	rplP; ribosomal protein L16
					20.500	82.500	48.1085	82.5000	
SGO_1979	-1.001	10.336	0.0006	0.0010	88.500	429.000	192.8788	446.4644	rpsC; ribosomal protein S3
					102.000	414.000	239.3692	414.0000	

Log$_2$ Ratios: -6 -4 -2 0 2 4 6

Show detected proteins only
Show all proteins
Filter by category:
ABC Transporter

Proteins found: 769

Test	Cutoff
***q*-Value**	**.005**
p-Value	

	Signif	Direction	Applies To
red	yes	+	ratios, bars
yellow	no	n/a	bars
green	yes	−	ratios, bars
pink	yes	+	*p*- , *q*-Values
light green	yes	−	*p*- , *q*-Values

Dot Plots Dot Plots

Hendrickson *et al.*

Protein	SgFn vs Sg Log2 Ratio	Log2 Sum	q-Value	p-Value	Raw SgFn	Raw Sg	Normalized SgFn	Normalized Sg	Description
SGO_1980	-0.272	8.907	0.0105	0.0417	53.500	134.000	116.5991	139.4551	rplV; ribosomal protein L22
					43.000	123.000	100.9105	123.0000	
SGO_1981	-0.372	9.339	0.0070	0.0251	64.000	159.000	139.4830	165.4728	rpsS; ribosomal protein S19
					60.500	200.500	141.9788	200.5000	
SGO_1982	0.174	9.007	0.0740	0.4556	78.500	99.500	171.0846	103.5506	rplB; ribosomal protein L2
					44.500	135.500	104.4307	135.5000	
SGO_1983	-1.445	8.527	0.0028	0.0079	14.500	130.000	31.6016	135.2923	rplW; ribosomal protein L23
					31.500	128.000	73.9228	128.0000	
SGO_1984	0.348	9.327	0.0089	0.0341	81.500	153.000	177.6229	159.2286	rplD; ribosomal protein L4/L1 family
					77.000	124.500	180.7003	124.5000	
SGO_1985	1.375	9.340	0.0021	0.0053	123.000	80.000	268.0689	83.2568	rplC; ribosomal protein L3
					85.500	96.000	200.6477	96.0000	
SGO_1986	-0.327	7.486	0.0238	0.1144	19.000	56.000	41.4090	58.2797	rpsJ; ribosomal protein S10
					16.000	42.000	37.5481	42.0000	
SGO_1988	1.168	5.598	0.0404	0.2208		7.000		7.2850	hydrolase, haloacid dehalogenase-like family
					10.500	16.500	24.6409	16.5000	
SGO_1989	0.601	9.267	0.0015	0.0034	89.500	109.500	195.0583	113.9577	purA; adenylosuccinate synthetase
					75.000	131.000	176.0068	131.0000	
SGO_1990	3.661	6.835	0.0005	0.0008	21.500	3.000	46.8576	3.1221	glutamate--cysteine ligase, putative/amino acid ligase, putative
					25.000	5.500	58.6689	5.5000	
SGO_1991	2.067	6.131	0.0009	0.0016	12.500	4.000	27.2428	4.1628	hslO; 33 kDa chaperonin /Heat shock protein 33-like protein
					12.000	10.500	28.1611	10.5000	
SGO_1993	-1.499	6.875	0.0024	0.0063	7.500	35.000	16.3457	36.4248	possible transcriptional regulator
					6.000	50.500	14.0805	50.5000	

Log2 Ratios: -6 -4 -2 0 2 4 6

Show detected proteins only
Show all proteins
Filter by category:
ABC Transporter

Proteins found: 769

Test	Cutoff
q-Value	.005
p-Value	

	Signif	Direction	Applies To
red	yes	+	ratios, bars
yellow	no	n/a	bars
green	yes	−	ratios, bars
pink	yes	+	p- , q-Values
light green	yes	−	p- , q-Values

Dot Plots Dot Plots

Protein	SgFn vs Sg: Log_2 Ratio	Log_2 Sum	*q*-Value	*p*-Value	Raw: *SgFn*	Raw: *Sg*	Normalized: *SgFn*	Normalized: *Sg*	Description
SGO_1995	0.615	5.975	0.0425	0.2374	6.000	16.500	13.0765	17.1717	MutT/nudix family protein
					10.500	8.000	24.6409	8.0000	
SGO_1998	-0.114	7.258	0.0299	0.1545	17.000	41.000	37.0502	42.6691	clpB; ATP-dependent Clp proteinase, ATP-binding chain
					15.500	37.000	36.3747	37.0000	
SGO_2000	0.216	10.871	0.0174	0.0778	247.500	443.500	539.4069	461.5547	tsf; translation elongation factor Ts
					199.000	404.500	467.0046	404.5000	
SGO_2001	-0.063	10.783	0.0858	0.5436	180.000	455.000	392.2960	473.5229	rpsB; ribosomal protein S2
					200.500	425.500	470.5248	425.5000	
SGO_2004	-2.079	7.088	0.0011	0.0023	5.000	46.000	10.8971	47.8726	LPXTG cell wall surface protein
					6.500	62.000	15.2539	62.0000	
SGO_2005	-5.995	9.561	0.0089	0.0340		379.000		394.4289	LPXTG cell wall surface protein
					2.500	355.000	5.8669	355.0000	
SGO_2007	0.413	7.410	0.0001	0.0001	22.500	35.500	49.0370	36.9452	nusG; transcription termination/antitermination factor NusG
					20.500	36.000	48.1085	36.0000	
SGO_2010	-1.834	6.616	0.0002	0.0002	4.500	38.500	9.8074	40.0673	pbp2a; penicillin-binding protein 2A
					5.000	36.500	11.7338	36.5000	
SGO_2013	-5.238	9.019	0.0000	0.0000	2.500	248.500	5.4486	258.6163	putative N-acetylmuramidase/lysin
					3.500	246.500	8.2136	246.5000	
SGO_2033	0.426	8.356	0.0017	0.0041	41.000	69.500	89.3563	72.3293	nrdD; ribonucleoside-triphosphate reductase
					42.000	67.500	98.5638	67.5000	
SGO_2034	-1.249	5.669	0.0365	0.1951	4.000	16.500	8.7177	17.1717	hypothetical protein SGO_2034
						25.000		25.0000	
SGO_2037	-0.611	6.209	0.0059	0.0199	5.500	21.500	11.9868	22.3753	cardiolipin synthase
					7.500	22.000	17.6007	22.0000	

Log_2 Ratios: -6 -4 -2 0 2 4 6

Show detected proteins only
Show all proteins
Filter by category:
ABC Transporter

Proteins found: 769

Test	Cutoff
***q*-Value**	**.005**
p-Value	

	Signif	Direction	Applies To
red	yes	+	ratios, bars
yellow	no	n/a	bars
green	yes	–	ratios, bars
light red	yes	+	*p*- , *q*-Values
light green	yes	–	*p*- , *q*-Values

Dot Plots Dot Plots

Protein	SgFn vs Sg Log_2 Ratio	Log_2 Sum	*q*-Value	*p*-Value	Raw *SgFn*	Raw *Sg*	Normalized *SgFn*	Normalized *Sg*	Description	Log_2 Ratios
SGO_2042	0.631	7.574	0.0260	0.1299	35.000	40.500	76.2798	42.1487	Bacterial protein of unknown function (DUF965) superfamily	
					17.500	31.000	41.0682	31.0000		
SGO_2045	-2.332	9.653	0.0005	0.0006	32.000	294.500	69.7415	306.4890	recA; recA protein	
					27.000	365.500	63.3624	365.5000		
SGO_2046	0.441	5.691	0.0257	0.1271	7.000	8.000	15.2560	8.3257	cinA; competence induced protein	
					6.000	14.000	14.0805	14.0000		
SGO_2050	0.126	3.423	0.1429	0.9676		5.000		5.2035	ruvA; Holliday junction DNA helicase RuvA	
					1.500	2.000	3.5201	2.0000		
SGO_2053	-0.307	6.428	0.0257	0.1272	9.500	19.500	20.7045	20.2938	DNA mismatch repair protein hexB	
					7.500	27.500	17.6007	27.5000		
SGO_2056	0.988	7.171	0.0049	0.0156	19.000	18.500	41.4090	19.2531	mutS; DNA mismatch repair protein MutS	
					23.000	29.500	53.9754	29.5000		
SGO_2058	0.776	10.075	0.0002	0.0001	152.500	185.500	332.3619	193.0516	argS; arginyl-tRNA synthetase	
					148.500	204.500	348.4934	204.5000		
SGO_2060	0.598	7.874	0.0093	0.0357	29.000	37.000	63.2032	38.5063	aspS-1; aspartyl-tRNA synthetase	
					33.000	55.500	77.4430	55.5000		
SGO_2062	1.266	8.848	0.0012	0.0025	80.500	55.000	175.4435	57.2390	hisS; histidyl-tRNA synthetase	
					63.500	79.000	149.0191	79.0000		
SGO_2064	1.217	8.670	0.0002	0.0002	67.000	54.500	146.0213	56.7187	ilvD; dihydroxy-acid dehydratase	
					59.000	66.000	138.4587	66.0000		
SGO_2066	-0.335	6.614	0.0814	0.5094	12.500	42.000	27.2428	43.7098	rpmG; ribosomal protein L33	
						27.000		27.0000		
SGO_2070	-0.754	6.302	0.0416	0.2298	8.500	12.500	18.5251	13.0089	hypothetical protein SGO_2070	
					4.000	38.000	9.3870	38.0000		

Log_2 Ratios axis: -6 -4 -2 0 2 4 6

Show detected proteins only
Show all proteins
Filter by category: ABC Transporter

Proteins found: 769

Test	Cutoff
q-Value	.005
p-Value	

	Signif	Direction	Applies To
red	yes	+	ratios, bars
yellow	no	n/a	bars
green	yes	−	ratios, bars
pink	yes	+	*p*- , *q*-Values
light green	yes	−	*p*- , *q*-Values

Dot Plots Dot Plots

Streptococcus gordonii

Hackett Laboratory UW

Protein	SgFn vs Sg				Raw		Normalized		Description
	Log_2 Ratio	Log_2 Sum	*q*-Value	*p*-Value	*SgFn*	*Sg*	*SgFn*	*Sg*	
**SGO_2082**	-2.782	5.615	0.0263	0.1323	1.500	18.000	3.2691	18.7328	lipoprotein, putative
						27.000		27.0000	
**SGO_2084**	0.089	5.219	0.0952	0.6113	3.500	9.500	7.6280	9.8867	NAD(P)H dehydrogenase, quinone family
					5.000	8.000	11.7338	8.0000	
**SGO_2085**	-0.782	8.011	0.0004	0.0004	22.500	75.500	49.0370	78.5736	purB; adenylosuccinate lyase
					19.500	84.500	45.7618	84.5000	
**SGO_2098**	0.507	11.004	0.0078	0.0287	313.000	388.500	682.1591	404.3157	rpsD; ribosomal protein S4
					224.500	440.000	526.8469	440.0000	
**SGO_2100**	-0.870	6.617	0.0014	0.0029	9.000	32.000	19.6148	33.3027	ABC transporter substrate-binding protein
					6.500	30.000	15.2539	30.0000	
**SGO_2103**	0.861	3.079			2.500		5.4486		hydrolase, haloacid dehalogenase-like family
						3.000		3.0000	
**SGO_2104**	-1.959	9.563	0.0003	0.0002	44.500	291.000	96.9843	302.8465	srtB; sortase B
					26.000	295.500	61.0157	295.5000	
**SGO_2105**	-1.919	12.653	0.0005	0.0008	303.000	2230.000	660.3649	2320.7824	abpA; amylase-binding protein AbpA
					291.000	2777.000	682.9063	2777.0000	
**SGO_2106**	0.371	8.552	0.0141	0.0613	55.500	79.000	120.9579	82.2161	ribose-phosphate diphosphokinase
					39.000	80.500	91.5235	80.5000	
**SGO_2133**	-0.914	10.415	0.0008	0.0014	121.000	411.000	263.7101	427.7317	Cell division protein ftsH-like protein
					90.000	462.500	211.2081	462.5000	
**SGO_2134**	1.391	8.622	0.0005	0.0008	63.500	62.500	138.3933	65.0444	hpt; hypoxanthine phosphoribosyltransferase
					62.000	45.000	145.4989	45.0000	
**SGO_2142**	-0.098	7.739	0.0609	0.3630	22.000	48.500	47.9473	50.4744	GTP-binding protein
					23.500	60.000	55.1488	60.0000	

Log_2 Ratios: -6 -4 -2 0 2 4 6

◉ Show detected proteins only
○ Show all proteins
☐ Filter by category:
ABC Transporter

Proteins found: 769

Test	Cutoff
***q*-Value**	**.005**
p-Value	

Color	Signif	Direction	Applies To
red	yes	+	ratios, bars
yellow	no	n/a	bars
green	yes	−	ratios, bars
pink	yes	+	*p*- , *q*-Values
light green	yes	−	*p*- , *q*-Values

Dot Plots Dot Plots

Hendrickson *et al.*

Protein	*SgFn* vs *Sg*				Raw		Normalized		Description	Log_2 Ratios
	Log_2 Ratio	Log_2 Sum	*q*-Value	*p*-Value	*SgFn*	*Sg*	*SgFn*	*Sg*		-6 -4 -2 0 2 4 6
**SGO_2150**	-0.999	8.352	0.0006	0.0008	27.500	108.500	59.9341	112.9170	degP; serine protease	
					21.000	104.500	49.2819	104.5000		

◉ Show detected proteins only
○ Show all proteins
☐ Filter by category:
ABC Transporter

Proteins found: 769

Test	Cutoff
***q*-Value**	**.005**
p-Value	

	Signif	Direction	Applies To
red	yes	+	ratios, bars
yellow	no	n/a	bars
green	yes	−	ratios, bars
pink	yes	+	*p*- , *q*-Values
light green	yes	−	*p*- , *q*-Values

Dot Plots Dot Plots
