# Supplementary material for: Proteomics of Streptococcus gordonii within a model developing oral microbial community
Source: BMC Microbiol. 2012 Sep 18;12:211. doi: 10.1186/1471-2180-12-211 (PMC3534352; doi:10.1186/1471-2180-12-211)
Supplement: Additional file 3 — SgPg_vs_Sg. A more detailed presentation of the relative abundance ratios for the comparison of SgPg and the Sg controls, including both raw and normalized spectral counts. Red and green highlights are used as in Additional file 1. [file 1471-2180-12-211-S3.pdf]

SgPg vs Sg

Streptococcus gordonii

Hackett Laboratory UW

Protein	SgPg vs Sg				Raw		Normalized		Description
	Log_2 Ratio	Log_2 Sum	*q*-Value	*p*-Value	*SgPg*	*Sg*	*SgPg*	*Sg*	
SGO_0001	-0.754	7.101	0.0159	0.0586	19.000	32.000	28.3302	33.3027	dnaA; chromosomal replication initiator protein DnaA
					15.500	53.500	22.1215	53.5000	
SGO_0002	0.550	7.204	0.0242	0.0969	30.000	21.000	44.7319	21.8549	dnaN; DNA polymerase III, beta subunit
					29.000	39.500	41.3886	39.5000	
SGO_0004	-1.979	8.713	0.0019	0.0040	36.500	137.000	54.4238	142.5772	putative lipoprotein
					22.500	190.500	32.1119	190.5000	
SGO_0005		4.640				10.500		10.9275	hypothetical protein SGO_0005
						14.000		14.0000	
SGO_0006	0.462	7.817	0.0017	0.0033	46.000	44.000	68.5889	45.7912	ABC transporter, ATP-binding protein
					43.500	49.000	62.0829	49.0000	
SGO_0007	1.153	6.977	0.0003	0.0002	29.000	17.000	43.2408	17.6921	trpS; tryptophanyl-tRNA synthetase
					30.500	21.500	43.5294	21.5000	
SGO_0008	1.064	9.833	0.0005	0.0007	214.000	156.500	319.0876	162.8711	inosine-5'-monophosphate dehydrogenase
					208.000	133.000	296.8562	133.0000	
SGO_0011	-1.064	5.482	0.0036	0.0090	6.000	13.000	8.9464	13.5292	proteinase, M16 family
					4.000	16.500	5.7088	16.5000	
SGO_0012		1.807							mpp; peptidase, M16 family
						3.500		3.5000	
SGO_0013	-0.643	5.212	0.0434	0.1881	6.000	15.000	8.9464	15.6106	hypothetical protein SGO_0013
						12.500		12.5000	
SGO_0015	-2.256	6.598	0.0001	0.0000	5.500	39.500	8.2009	41.1080	ABC transporter (ATP-binding protein)
					6.000	39.000	8.5632	39.0000	
SGO_0016	-0.241	4.669	0.0423	0.1825	3.500	7.500	5.2187	7.8053	cbiO; ABC transporter, ATP-binding protein
					4.500	6.000	6.4224	6.0000	

Show detected proteins only
Show all proteins
Filter by category:
ABC Transporter

Proteins found:
1179

Test	Cutoff
q-Value	.005
p-Value	

	Signif	Direction	Applies To
red	yes	+	ratios, bars
yellow	no	n/a	bars
green	yes	−	ratios, bars
pink	yes	+	*p*- , *q*-Values
light green	yes	−	*p*- , *q*-Values

Dot Plots Dot Plots

Hendrickson *et al.*

Protein	SgPg vs Sg Log_2 Ratio	Log_2 Sum	*q*-Value	*p*-Value	Raw *SgPg*	Raw *Sg*	Normalized *SgPg*	Normalized *Sg*	Description
SGO_0017		2.351				2.500		2.6018	ABC-type putative cobalt transport system, permease
						2.500		2.5000	
SGO_0019		2.807							sdhA; L-serine dehydratase, iron-sulfur-dependent, alpha subunit
						7.000		7.0000	
SGO_0020		2.585							sdhB; L-serine dehydratase, iron-sulfur-dependent, beta subunit
						6.000		6.0000	
SGO_0022	1.241	5.854	0.0025	0.0057	13.000	6.000	19.3838	6.2443	trmU; tRNA (5-methylaminomethyl-2-thiouridylate)-methyltransferase
					14.500	11.500	20.6943	11.5000	
SGO_0025	0.338	6.207	0.0477	0.2100	17.500	14.500	26.0936	15.0903	gidA; glucose inhibited division protein A
					11.000	17.000	15.6991	17.0000	
SGO_0026	-3.976	7.514	0.0005	0.0007	2.500	91.000	3.7277	94.7046	DHH subfamily 1 protein
					5.500	76.500	7.8496	76.5000	
SGO_0027	0.376	7.312	0.0046	0.0126	29.000	30.500	43.2408	31.7416	rplI; ribosomal protein L9
					32.500	37.500	46.3838	37.5000	
SGO_0028	-0.451	5.614	0.0507	0.2268	5.000	11.000	7.4553	11.4478	dnaC; replicative DNA helicase
					9.500	16.500	13.5583	16.5000	
SGO_0030	0.967	7.027	0.0008	0.0011	30.000	19.000	44.7319	19.7735	aspB; aspartate transaminase
					29.000	24.500	41.3886	24.5000	
SGO_0032	0.024	7.492	0.1697	0.8926	30.500	38.000	45.4774	39.5470	plsX; fatty acid/phospholipid synthesis protein PlsX
					31.500	50.000	44.9566	50.0000	
SGO_0033	0.994	5.306	0.0039	0.0102	10.000	5.500	14.9106	5.7239	acpP; acyl carrier protein
					8.000	7.500	11.4175	7.5000	
SGO_0035	-0.709	2.496							phosphoribosylformylglycinamidine synthase
					1.500	3.500	2.1408	3.5000	

Log_2 Ratios: -6 -4 -2 0 2 4 6

Show detected proteins only
Show all proteins
Filter by category:
ABC Transporter

Proteins found: 1179

Test	Cutoff
***q*-Value**	.005
p-Value	

	Signif	Direction	Applies To
red	yes	+	ratios, bars
yellow	no	n/a	bars
green	yes	–	ratios, bars
pink	yes	+	*p*- , *q*-Values
light green	yes	–	*p*- , *q*-Values

Dot Plots Dot Plots

SgPg vs Sg	Streptococcus gordonii	Hackett Laboratory UW

Summary Table | SgFn vs Sg | SgPg vs Sg | SgPgFn vs Sg | SgPg vs SgFn | SgPgFn vs SgFn | SgPgFn vs SgPg | Coverage

Protein	SgPg vs Sg Log2 Ratio	Log2 Sum	q-Value	p-Value	Raw SgPg	Raw Sg	Normalized SgPg	Normalized Sg	Description
SGO_0042	0.355	5.804	0.0083	0.0260	11.500	11.500	17.1472	11.9682	transcription regulator, GntR family
					10.000	12.500	14.2719	12.5000	
SGO_0044									PTS system, IIB component
SGO_0050		4.640				10.500		10.9275	hypothetical protein SGO_0050
						14.000		14.0000	
SGO_0051		3.351				5.000		5.2035	hypothetical protein SGO_0051
						5.000		5.0000	
SGO_0054	0.238	7.609	0.0136	0.0480	33.500	40.000	49.9506	41.6284	dltA; D-alanine-activating enzyme
					39.000	48.000	55.6605	48.0000	
SGO_0055		2.058				4.000		4.1628	dltB; integral membrane protein
SGO_0056		3.420				5.000		5.2035	dltC; D-alanyl carrier protein
						5.500		5.5000	
SGO_0057	-2.098	8.163	0.0008	0.0012	18.500	99.500	27.5847	103.5506	dltD protein
					18.500	129.000	26.4031	129.0000	
SGO_0059	1.097	12.538	0.0073	0.0222	1491.000	1257.500	2223.1761	1308.6923	pXO1; hypothetical protein SGO_0059
					1233.500	653.500	1760.4431	653.5000	
SGO_0060		8.043				118.000		122.8037	hypothetical protein SGO_0060
						141.000		141.0000	
SGO_0062		1.380				2.500		2.6018	hypothetical protein SGO_0062
SGO_0063	-1.765	7.875	0.0002	0.0001	15.500	86.000	23.1115	89.5010	hypothetical protein SGO_0063
					21.500	91.500	30.6847	91.5000	

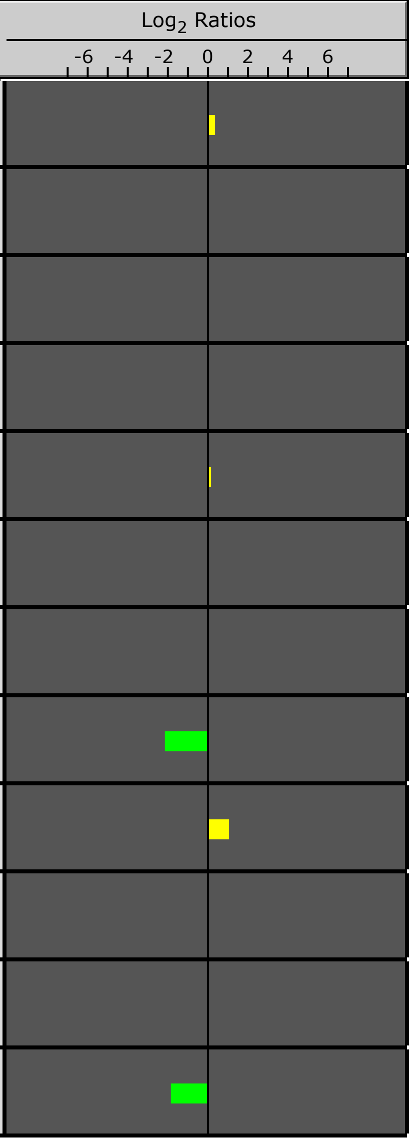

Show detected proteins only
Show all proteins
Filter by category: ABC Transporter

Proteins found: 1179

Test	Cutoff
q-Value	.005
p-Value	

Color	Signif	Direction	Applies To
red	yes	+	ratios, bars
yellow	no	n/a	bars
green	yes	−	ratios, bars
pink	yes	+	p- , q-Values
light green	yes	−	p- , q-Values

Dot Plots | Dot Plots

Hendrickson *et al.*

Protein	*SgPg vs Sg* Log_2 Ratio	Log_2 Sum	*q*-Value	*p*-Value	Raw *SgPg*	Raw *Sg*	Normalized *SgPg*	Normalized *Sg*	Description
SGO_0064	-1.647	9.154	0.0003	0.0002	40.000	214.000	59.6426	222.7119	FtsK/SpoIIIE family protein
					55.500	208.000	79.2092	208.0000	
SGO_0065	0.339	7.484	0.0009	0.0013	34.500	37.500	51.4417	39.0266	hypothetical protein SGO_0065
					34.000	40.000	48.5246	40.0000	
SGO_0066		4.494			6.500		9.6919		D-3-phosphoglycerate dehydrogenase, putative
					9.000		12.8447		
SGO_0067	0.784	7.024	0.0059	0.0173	32.000	23.000	47.7140	23.9363	protein with prophage function domain
					24.500	23.500	34.9662	23.5000	
SGO_0068	-0.510	6.655	0.0016	0.0031	13.500	30.000	20.1294	31.2213	lipoprotein, putative
					15.000	28.000	21.4079	28.0000	
SGO_0069	-0.728	6.084	0.0007	0.0009	9.000	19.500	13.4196	20.2938	hypothetical protein SGO_0069
					8.500	22.000	12.1311	22.0000	
SGO_0070	0.264	6.588	0.0177	0.0667	16.500	19.000	24.6026	19.7735	merozoite surface protein 1
					19.500	24.000	27.8303	24.0000	
SGO_0075		1.380				2.500		2.6018	hypothetical protein SGO_0075
SGO_0078	-1.267	6.328	0.0090	0.0292	9.500	34.000	14.1651	35.3841	hypothetical protein SGO_0078
					6.500	21.500	9.2768	21.5000	
SGO_0079		1.380				2.500		2.6018	hypothetical protein SGO_0079
SGO_0080	-0.712	7.484	0.0011	0.0018	24.500	51.000	36.5311	53.0762	hypothetical protein SGO_0080
					22.000	58.000	31.3983	58.0000	
SGO_0081	1.034	4.842	0.0181	0.0685	8.000	3.000	11.9285	3.1221	hypothetical protein SGO_0081
					5.000	6.500	7.1360	6.5000	

Log_2 Ratios: -6 -4 -2 0 2 4 6

Show detected proteins only
Show all proteins
Filter by category:
ABC Transporter

Proteins found: 1179

Test	Cutoff
q-Value	.005
p-Value	

Color	Signif	Direction	Applies To
red	yes	+	ratios, bars
yellow	no	n/a	bars
green	yes	−	ratios, bars
pink	yes	+	*p*- , *q*-Values
light green	yes	−	*p*- , *q*-Values

Dot Plots Dot Plots

Protein	SgPg vs Sg: Log_2 Ratio	Log_2 Sum	*q*-Value	*p*-Value	Raw: *SgPg*	Raw: *Sg*	Normalized: *SgPg*	Normalized: *Sg*	Description
SGO_0095		5.271			12.500		18.6383		mccF; microcin immunity protein MccF, putative
					14.000		19.9807		
SGO_0097		6.097				36.000		37.4655	hypothetical protein SGO_0097
						31.000		31.0000	
SGO_0098	0.979	6.324	0.0344	0.1448	19.000	15.500	28.3302	16.1310	ribonucleotide reductase-like protein
					25.000		35.6798		
SGO_0099		3.712			4.000		5.9643		pulA-2; pullulanase, type I
					5.000		7.1360		
SGO_0100	0.127	4.089	0.1827	0.9834		7.500		7.8053	maltose operon transcription repressor
					4.000	3.500	5.7088	3.5000	
SGO_0101		4.874				8.000		8.3257	malA; Maltodextrose utilization protein malA
						21.000		21.0000	
SGO_0104	-0.689	9.636	0.0001	0.0000	100.000	234.000	149.1064	243.5261	Maltose/maltodextrin-binding protein precursor
					109.000	247.500	155.5641	247.5000	
SGO_0105	0.873	6.200	0.0011	0.0019	16.500	14.000	24.6026	14.5699	malQ; 4-alpha-glucanotransferase
					16.000	11.500	22.8351	11.5000	
SGO_0106	1.969	7.831	0.0002	0.0001	61.500	19.000	91.7004	19.7735	glgP-2; maltodextrin phosphorylase
					62.500	27.000	89.1996	27.0000	
SGO_0107									LPXTG cell wall surface protein, collagen binding domain
SGO_0108		5.505				22.500		23.4160	ruvB; Holliday junction DNA helicase RuvB
						22.000		22.0000	
SGO_0110		2.898			5.000		7.4553		phosphotyrosine protein phosphatase

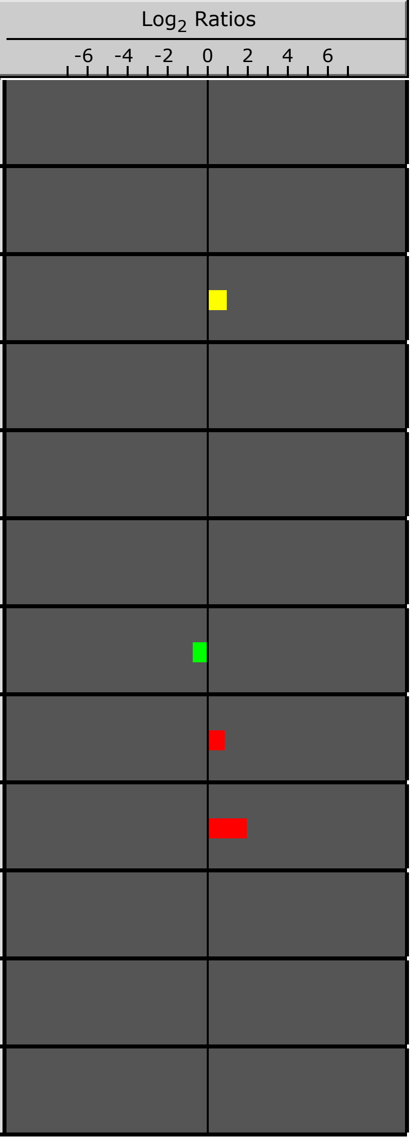

Show detected proteins only
Show all proteins
Filter by category:
ABC Transporter

Proteins found: 1179

Test	Cutoff
***q*-Value**	**.005**
p-Value	

Color	Signif	Direction	Applies To
red	yes	+	ratios, bars
yellow	no	n/a	bars
green	yes	−	ratios, bars
pink	yes	+	*p*- , *q*-Values
light green	yes	−	*p*- , *q*-Values

Dot Plots Dot Plots

Protein	SgPg vs Sg: Log_2 Ratio	Log_2 Sum	*q*-Value	*p*-Value	Raw: *SgPg*	Raw: *Sg*	Normalized: *SgPg*	Normalized: *Sg*	Description
SGO_0111		4.115				8.000		8.3257	MORN motif family protein
						9.000		9.0000	
SGO_0112		5.366				18.000		18.7328	putative acyltransferase
						22.500		22.5000	
SGO_0113	-1.505	13.143	0.0002	0.0001	838.500	3329.000	1250.2570	3464.5223	acdH; alcohol-acetaldehyde dehydrogenase
					777.000	3222.500	1108.9293	3222.5000	
SGO_0128		3.170							hypothetical protein SGO_0128
						9.000		9.0000	
SGO_0129	-3.290	6.881	0.0002	0.0001	3.500	48.500	5.2187	50.4744	atpI; v-type sodium ATP synthase, chain I
					4.000	56.500	5.7088	56.5000	
SGO_0131		5.425				11.000		11.4478	v-type sodium ATP synthase, chain E
						31.500		31.5000	
SGO_0132	-1.088	4.528	0.0494	0.2205	2.500		3.7277		ATP synthase (C/AC39) subunit
					5.500	11.500	7.8496	11.5000	
SGO_0134	0.106	5.225	0.1182	0.5924	5.000	7.500	7.4553	7.8053	acetyltransferase, GNAT family
					8.500	10.000	12.1311	10.0000	
SGO_0135	-0.954	7.926	0.0003	0.0003	25.500	77.500	38.0221	80.6550	v-type sodium ATP synthase, subunit A
					31.500	79.500	44.9566	79.5000	
SGO_0136	-1.309	8.383	0.0007	0.0010	29.500	106.000	43.9864	110.3152	v-type sodium ATP synthase, chain B
					36.500	127.500	52.0926	127.5000	
SGO_0137		3.653				2.000		2.0814	V-type ATPase, D subunit
						10.500		10.5000	
SGO_0138		2.459							LysM domain protein
						5.500		5.5000	

Log_2 Ratios: -6 -4 -2 0 2 4 6

Show detected proteins only
Show all proteins
Filter by category:
ABC Transporter

Proteins found: 1179

Test	Cutoff
q-Value	.005
p-Value	

Signif	Direction	Applies To
yes	+	ratios, bars
no	n/a	bars
yes	−	ratios, bars
yes	+	*p*- , *q*-Values
yes	−	*p*- , *q*-Values

Dot Plots Dot Plots

Hendrickson *et al.*

Protein	SgPg vs Sg Log$_2$ Ratio	Log$_2$ Sum	*q*-Value	*p*-Value	Raw *SgPg*	Raw *Sg*	Normalized *SgPg*	Normalized *Sg*	Description
SGO_0139	0.581	7.434	0.0007	0.0010	36.000	32.000	53.6783	33.3027	thrC; threonine synthase
					35.000	36.000	49.9518	36.0000	
SGO_0142		1.585							hypothetical protein SGO_0142
						3.000		3.0000	
SGO_0144	-2.511	5.213	0.0174	0.0651	2.000	15.000	2.9821	15.6106	hypothetical protein SGO_0144
						18.500		18.5000	
SGO_0145	-0.566	7.699	0.0007	0.0009	27.000	61.500	40.2587	64.0036	polI; DNA polymerase I
					30.500	60.000	43.5294	60.0000	
SGO_0146	0.659	6.116	0.0087	0.0278	16.000	11.000	23.8570	11.4478	CoA-binding domain protein
					13.000	15.500	18.5535	15.5000	
SGO_0148	-1.225	4.667	0.0139	0.0492	3.000	10.500	4.4732	10.9275	hypothetical protein SGO_0148
						10.000		10.0000	
SGO_0150		1.380				2.500		2.6018	fucA1; alpha-L-fucosidase
SGO_0152	1.009	6.079	0.0003	0.0003	14.500	10.500	21.6204	10.9275	tgt; queuine tRNA-ribosyltransferase
					16.500	11.500	23.5487	11.5000	
SGO_0154	0.517	10.445	0.0109	0.0367	307.000	309.500	457.7566	322.0996	pgi; glucose-6-phosphate isomerase
					254.000	251.500	362.5071	251.5000	
SGO_0155	-4.703	8.402	0.0009	0.0014	4.000	179.000	5.9643	186.2870	hypothetical protein SGO_0155
					4.500	139.500	6.4224	139.5000	
SGO_0156	-2.580	7.668	0.0004	0.0004	11.000	90.500	16.4017	94.1842	hypothetical protein SGO_0156
					9.000	80.000	12.8447	80.0000	
SGO_0157		3.357				6.000		6.2443	hypothetical protein SGO_0157
						4.000		4.0000	

Log$_2$ Ratios: -6 -4 -2 0 2 4 6

Show detected proteins only
Show all proteins
Filter by category:
ABC Transporter

Proteins found: 1179

Test	Cutoff
q-Value	.005
p-Value	

	Signif	Direction	Applies To
red	yes	+	ratios, bars
yellow	no	n/a	bars
green	yes	−	ratios, bars
pink	yes	+	*p*- , *q*-Values
light green	yes	−	*p*- , *q*-Values

Dot Plots Dot Plots

Protein	SgPg vs Sg Log$_2$ Ratio	Log$_2$ Sum	*q*-Value	*p*-Value	Raw *SgPg*	Raw *Sg*	Normalized *SgPg*	Normalized *Sg*	Description
SGO_0158	1.379	7.403	0.0009	0.0014	37.500	24.500	55.9149	25.4974	2,3,4,5-tetrahydropyridine-2-carboxylate N-succinyltransferase, putative
					46.500	21.500	66.3645	21.5000	
SGO_0159	-0.424	2.804			2.000		2.9821		hippurate hydrolase
						4.000		4.0000	
SGO_0163	0.746	7.631	0.0010	0.0016	41.000	32.000	61.1336	33.3027	galU; UTP-glucose-1-phosphate uridylyltransferase
					44.000	41.000	62.7965	41.0000	
SGO_0164	-0.280	7.349	0.0252	0.1009	28.000	40.000	41.7498	41.6284	Glycerol-3-phosphate dehydrogenase [NAD(P)+] (NAD(P)H-dependent glycerol-3-phosphate dehydrogenase)
					22.500	47.500	32.1119	47.5000	
SGO_0165		4.275				9.000		9.3664	ABC transporter, permease/ATP-binding protein SP2075
						10.000		10.0000	
SGO_0166	-2.723	6.342	0.0082	0.0256		35.000		36.4248	ABC transporter, permease/ATP-binding protein SP2073
					4.000	39.000	5.7088	39.0000	
SGO_0167		4.020				5.500		5.7239	glutamine amidotransferase
						10.500		10.5000	
SGO_0168		4.156				8.000		8.3257	hydrolase, NUDIX family
						9.500		9.5000	
SGO_0169	0.502	5.611	0.0352	0.1490	11.500	7.500	17.1472	7.8053	dut; dUTP diphosphatase
					8.000	12.500	11.4175	12.5000	
SGO_0171	-0.402	6.118	0.0338	0.1424	8.500	15.500	12.6740	16.1310	radA; DNA repair protein RadA
					12.000	23.500	17.1263	23.5000	
SGO_0172		5.614				24.000		24.9770	conserved hypothetical protein TIGR00266
						24.000		24.0000	
SGO_0173	-3.121	7.570	0.0001	0.0000	7.500	80.000	11.1830	83.2568	Carbonic anhydrase
					6.000	87.000	8.5632	87.0000	

Log$_2$ Ratios: -6 -4 -2 0 2 4 6

Show detected proteins only
Show all proteins
Filter by category:
ABC Transporter

Proteins found:
1179

Test	Cutoff
***q*-Value**	**.005**
p-Value	

	Signif	Direction	Applies To
(red)	yes	+	ratios, bars
(yellow)	no	n/a	bars
(green)	yes	−	ratios, bars
(pink)	yes	+	*p*- , *q*-Values
(light green)	yes	−	*p*- , *q*-Values

Dot Plots Dot Plots

Hendrickson *et al.*

Protein	SgPg vs Sg Log$_2$ Ratio	Log$_2$ Sum	*q*-Value	*p*-Value	Raw *SgPg*	Raw *Sg*	Normalized *SgPg*	Normalized *Sg*	Description
SGO_0174	1.114	8.678	0.0001	0.0000	94.500	60.500	140.9055	62.9629	gltX; glutamyl-tRNA synthetase
					97.500	66.500	139.1514	66.5000	
SGO_0179		2.585							Membrane protein oxaA 1 precursor
						6.000		6.0000	
SGO_0180	-0.132	7.007	0.1414	0.7291	26.500	24.000	39.5132	24.9770	jag; hypothetical protein SGO_0180
					15.500	42.000	22.1215	42.0000	
SGO_0181		5.217				17.000		17.6921	lipoprotein, putative
						19.500		19.5000	
SGO_0182		2.700							sapR; sakacin A production response regulator
						6.500		6.5000	
SGO_0188	-0.745	4.646	0.0473	0.2076		8.000		8.3257	hydrolase, TatD family
					4.000	11.000	5.7088	11.0000	
SGO_0189		1.585							primase-related protein
						3.000		3.0000	
SGO_0190	-1.685	5.563	0.0110	0.0370	2.000	12.500	2.9821	13.0089	hypothetical protein SGO_0190
					6.500	22.000	9.2768	22.0000	
SGO_0191	-1.774	4.548	0.0060	0.0179	2.000	10.000	2.9821	10.4071	hypothetical protein SGO_0191
						10.000		10.0000	
SGO_0193	0.844	5.139	0.0514	0.2304	6.500	3.000	9.6919	3.1221	ksgA; dimethyladenosine transferase
					8.000	11.000	11.4175	11.0000	
SGO_0197		3.541			3.500		5.2187		predicted ribosome small subunit-dependent GTPase A
					4.500		6.4224		
SGO_0198	0.896	7.170	0.0011	0.0017	32.000	27.500	47.7140	28.6195	rpe; ribulose-phosphate 3-epimerase
					32.000	22.000	45.6702	22.0000	

Log$_2$ Ratios: -6 -4 -2 0 2 4 6

Show detected proteins only
Show all proteins
Filter by category:
ABC Transporter

Proteins found: 1179

Test	Cutoff
q-Value	.005
p-Value	

	Signif	Direction	Applies To
red	yes	+	ratios, bars
yellow	no	n/a	bars
green	yes	–	ratios, bars
pink	yes	+	*p*- , *q*-Values
light green	yes	–	

Dot Plots Dot Plots

Hendrickson *et al.*

Protein	SgPg vs Sg Log_2 Ratio	Log_2 Sum	*q*-Value	*p*-Value	Raw *SgPg*	Raw *Sg*	Normalized *SgPg*	Normalized *Sg*	Description
SGO_0200	-1.166	7.858	0.0002	0.0001	24.500	74.500	36.5311	77.5329	competence-induced protein Ccs50
					24.500	83.000	34.9662	83.0000	
SGO_0201	-0.173	7.404	0.0277	0.1122	26.000	47.000	38.7677	48.9134	cmp-binding-factor 1
					28.500	41.000	40.6750	41.0000	
SGO_0202	-0.101	5.559	0.1063	0.5282	7.000	14.000	10.4374	14.5699	pur operon repressor
					8.500	10.000	12.1311	10.0000	
SGO_0203		2.700							hypothetical protein SGO_0203
						6.500		6.5000	
SGO_0204	0.094	8.177	0.0899	0.4380	56.500	63.500	84.2451	66.0851	rpsL; ribosomal protein S12
					46.000	73.500	65.6509	73.5000	
SGO_0205	0.726	9.456	0.0085	0.0269	124.000	113.000	184.8919	117.6002	rpsG; ribosomal protein S7
					178.000	146.000	254.0404	146.0000	
SGO_0206	0.873	12.482	0.0002	0.0001	1277.000	972.500	1904.0885	1012.0901	fusA; translation elongation factor G
					1258.000	1007.500	1795.4093	1007.5000	
SGO_0207	1.071	12.454	0.0007	0.0010	1267.000	748.500	1889.1778	778.9711	gap; glyceraldehyde-3-phosphate dehydrogenase, type I
					1330.000	1043.000	1898.1672	1043.0000	
SGO_0208		4.996				10.000		10.4071	LPXTG cell wall surface protein, glycosyl hydrolase family
						21.500		21.5000	
SGO_0209	2.191	13.204	0.0002	0.0001	2477.500	845.500	3694.1105	879.9200	pgk; phosphoglycerate kinase
					2836.000	814.500	4047.5205	814.5000	
SGO_0210	-5.424	7.976	0.0012	0.0020	1.500	98.500	2.2366	102.5099	sspA; streptococcal surface protein A
					2.500	143.500	3.5680	143.5000	
SGO_0211		9.177				254.000		264.3402	sspB; streptococcal surface protein B
						314.500		314.5000	

Log2 Ratios: -6 -4 -2 0 2 4 6

- Show detected proteins only
- Show all proteins
- Filter by category: ABC Transporter

Proteins found: 1179

Test	Cutoff
q-Value	.005
p-Value	

Color	Signif	Direction	Applies To
red	yes	+	ratios, bars
yellow	no	n/a	bars
green	yes	−	ratios, bars
pink	yes	+	*p*- , *q*-Values
light green	yes	−	*p*- , *q*-Values

Dot Plots Dot Plots

Streptococcus gordonii

Hackett Laboratory UW

Protein	SgPg vs Sg: Log_2 Ratio	SgPg vs Sg: Log_2 Sum	SgPg vs Sg: *q*-Value	SgPg vs Sg: *p*-Value	Raw: *SgPg*	Raw: *Sg*	Normalized: *SgPg*	Normalized: *Sg*	Description
SGO_0214		1.322							transcription regulator, MerR family
						2.500		2.5000	
SGO_0215	-0.087	8.628	0.0722	0.3407	70.000	95.500	104.3745	99.3878	glnA; glutamine synthetase, type I
					61.500	104.000	87.7724	104.0000	
SGO_0219	0.237	8.297	0.0080	0.0251	57.000	74.500	84.9906	77.5329	metallo-beta-lactamase superfamily protein 1
					59.500	67.000	84.9180	67.0000	
SGO_0220		4.294			5.500		8.2009		Protein of unknown function (DUF1447) superfamily
					8.000		11.4175		
SGO_0223	1.487	4.380	0.0087	0.0279		4.500		4.6832	glycoproteinase family protein
					8.500	4.000	12.1311	4.0000	
SGO_0230	0.665	5.843	0.0050	0.0141	12.500	9.000	18.6383	9.3664	Protein of unknown function, DUF536 family
					11.500	13.000	16.4127	13.0000	
SGO_0231		3.111				3.500		3.6425	glycerophosphoryl diester phosphodiesterase family protein
						5.000		5.0000	
SGO_0232	1.663	4.738	0.0558	0.2527	5.500		8.2009		conserved hypothetical protein TIGR00103
					10.500	3.500	14.9855	3.5000	
SGO_0233		5.976				35.000		36.4248	lipoprotein, putative
						26.500		26.5000	
SGO_0234	-0.219	7.669	0.0216	0.0849	35.000	53.000	52.1872	55.1576	pepX; X-Pro dipeptidyl-peptidase
					29.500	54.000	42.1022	54.0000	
SGO_0235	-1.563	7.157	0.0048	0.0133	10.500	62.500	15.6562	65.0444	glycerol uptake facilitator protein-like protein
					14.000	42.000	19.9807	42.0000	
SGO_0236		4.509				6.500		6.7646	hypothetical protein SGO_0236
						16.000		16.0000	

Log_2 Ratios: -6 -4 -2 0 2 4 6

Show detected proteins only
Show all proteins
Filter by category:
ABC Transporter

Proteins found: 1179

Test	Cutoff
q-Value	.005
p-Value	

	Signif	Direction	Applies To
red	yes	+	ratios, bars
yellow	no	n/a	bars
green	yes	−	ratios, bars
pink	yes	+	*p*- , *q*-Values
light green	yes	−	*p*- , *q*-Values

Dot Plots Dot Plots

Hendrickson *et al.*

Protein	SgPg vs Sg Log$_2$ Ratio	Log$_2$ Sum	*q*-Value	*p*-Value	Raw *SgPg*	Raw *Sg*	Normalized *SgPg*	Normalized *Sg*	Description
SGO_0237	1.319	5.790	0.0038	0.0097	12.500	5.000	18.6383	5.2035	ccpA; CcpA protein (proteinase)
					14.000	11.500	19.9807	11.5000	
SGO_0240		2.836				3.500		3.6425	mvaD; diphosphomevalonate decarboxylase
						3.500		3.5000	
SGO_0242		1.058				2.000		2.0814	FMN-dependent dehydrogenase family protein
SGO_0243		3.791			4.500		6.7098		hydroxymethylglutaryl-CoA reductase, degradative
					5.000		7.1360		
SGO_0244	2.252	5.461	0.0003	0.0002	11.500	3.500	17.1472	3.6425	hydroxymethylglutaryl-CoA synthase
					13.500	4.000	19.2671	4.0000	
SGO_0247	-1.086	11.004	0.0005	0.0006	213.500	709.000	318.3421	737.8631	pfl; formate acetyltransferase
					237.500	659.000	338.9584	659.0000	
SGO_0252	-0.542	4.617	0.0297	0.1218	4.500	6.500	6.7098	6.7646	possible TetR-type transcriptional regulator
					2.500	7.500	3.5680	7.5000	
SGO_0253		5.655				22.000		22.8956	hypothetical protein SGO_0253
						27.500		27.5000	
SGO_0254	-0.810	4.643	0.0299	0.1228	3.500	5.000	5.2187	5.2035	helicase, RecD/TraA family
					2.500	11.000	3.5680	11.0000	
SGO_0255	-1.618	7.630	0.0027	0.0065	16.000	84.500	23.8570	87.9400	Signal peptidase I
					17.000	62.000	24.2623	62.0000	
SGO_0256		4.191				6.500		6.7646	rnhC; ribonuclease HIII
						11.500		11.5000	
SGO_0258		1.585							hypothetical protein SGO_0258
						3.000		3.0000	

Log$_2$ Ratios: -6 -4 -2 0 2 4 6

Show detected proteins only
Show all proteins
Filter by category:
ABC Transporter

Proteins found: 1179

Test	Cutoff
q-Value	.005
p-Value	

	Signif	Direction	Applies To
red	yes	+	ratios, bars
yellow	no	n/a	bars
green	yes	−	ratios, bars
pink	yes	+	*p*-, *q*-Values
light green	yes	−	

Dot Plots Dot Plots

Streptococcus gordonii

Hackett Laboratory UW

Protein	SgPg vs Sg Log$_2$ Ratio	Log$_2$ Sum	*q*-Value	*p*-Value	Raw *SgPg*	Raw *Sg*	Normalized *SgPg*	Normalized *Sg*	Description	Log$_2$ Ratios (-6 -4 -2 0 2 4 6)
SGO_0260	-0.106	7.135	0.0376	0.1609	21.500	33.500	32.0579	34.8638	DNA mismatch binding protein MutS2	
					25.000	38.000	35.6798	38.0000		
SGO_0261		1.585							acetyltransferase, GNAT family	
						3.000		3.0000		
SGO_0262	0.829	7.073	0.0008	0.0012	30.500	24.500	45.4774	25.4974	dipeptidase	
					28.500	23.000	40.6750	23.0000		
SGO_0263	0.527	7.504	0.0012	0.0020	34.500	34.000	51.4417	35.3841	trx-1; thioredoxin	
					39.000	39.000	55.6605	39.0000		
SGO_0268	-1.945	5.229	0.0268	0.1082		18.000		18.7328	mechanosensitive transport protein	
					3.000	14.500	4.2816	14.5000		
SGO_0269		4.194				7.500		7.8053	hypothetical protein SGO_0269	
						10.500		10.5000		
SGO_0272	-0.248	6.402	0.0007	0.0009	13.000	22.500	19.3838	23.4160	hypothetical protein SGO_0272	
					13.500	22.500	19.2671	22.5000		
SGO_0276	0.383	9.225	0.0017	0.0034	114.000	132.500	169.9813	137.8940	gdhA; glutamate dehydrogenase (NADP)	
					118.000	122.000	168.4088	122.0000		
SGO_0277	-0.412	5.671	0.0422	0.1820	9.000	11.500	13.4196	11.9682	pyrA; Dihydroorotate dehydrogenase	
					6.000	17.000	8.5632	17.0000		
SGO_0278		1.000							msrA; Peptide methionine sulfoxide reductase msrA/msrB	
						2.000		2.0000		
SGO_0279		3.902				11.000		11.4478	lipoprotein, putative	
						3.500		3.5000		
SGO_0280	-0.591	6.340	0.0170	0.0629	14.000	24.000	20.8749	24.9770	trzA; ethylammeline chlorohydrolase	
					8.500	23.000	12.1311	23.0000		

Show detected proteins only
Show all proteins
Filter by category:
ABC Transporter

Proteins found: 1179

Test	Cutoff
***q*-Value**	**.005**
p-Value	

Color	Signif	Direction	Applies To
red	yes	+	ratios, bars
yellow	no	n/a	bars
green	yes	–	ratios, bars
pink	yes	+	*p*- , *q*-Values
light green	yes	–	*p*- , *q*-Values

Dot Plots Dot Plots

Hendrickson *et al.*

Protein	SgPg vs Sg Log2 Ratio	Log2 Sum	*q*-Value	*p*-Value	Raw *SgPg*	Raw *Sg*	Normalized *SgPg*	Normalized *Sg*	Description
SGO_0282		5.347				17.500		18.2124	cell wall polysaccharide biosynthesis protein
						22.500		22.5000	
SGO_0284		1.058				2.000		2.0814	transcriptional regulator
SGO_0286		5.487				20.500		21.3345	DNA mismatch repair protein MutS, putative
						23.500		23.5000	
SGO_0288		3.007			3.000		4.4732		GDSL-like lipase/acylhydrolase
					2.500		3.5680		
SGO_0290	-1.343	5.871	0.0144	0.0519	4.500	13.500	6.7098	14.0496	copper -translocating P-type ATPase
					6.500	28.500	9.2768	28.5000	
SGO_0291	-1.402	7.697	0.0008	0.0011	18.500	78.500	27.5847	81.6957	copper-translocating P-type ATPase
					20.500	69.000	29.2575	69.0000	
SGO_0292	0.881	9.177	0.0004	0.0005	120.000	100.000	178.9277	104.0710	spxB; pyruvate oxidase
					137.500	99.500	196.2391	99.5000	
SGO_0294		4.271				7.500		7.8053	ABC transporter, putative
						11.500		11.5000	
SGO_0297		2.459							6-phospho-beta-glucosidase
						5.500		5.5000	
SGO_0299		0.585							putative histidine kinase
						1.500		1.5000	
SGO_0301									ABC transporter ATP-binding protein-like protein
SGO_0306		3.886				7.000		7.2850	ABC transporter ATP-binding protein-like protein
						7.500		7.5000	

Log2 Ratios: -6 -4 -2 0 2 4 6

Show detected proteins only
Show all proteins
Filter by category: ABC Transporter

Proteins found: 1179

Test	Cutoff
q-Value	.005
p-Value	

	Signif	Direction	Applies To
red	yes	+	ratios, bars
yellow	no	n/a	bars
green	yes	−	ratios, bars
pink	yes	+	*p*- , *q*-Values
light green	yes	−	*p*- , *q*-Values

Dot Plots Dot Plots

Hendrickson *et al.*

Protein	*SgPg vs Sg* Log$_2$ Ratio	Log$_2$ Sum	*q*-Value	*p*-Value	Raw *SgPg*	Raw *Sg*	Normalized *SgPg*	Normalized *Sg*	Description	Log$_2$ Ratios (-6 -4 -2 0 2 4 6)
**SGO_0307**		2.718				2.000		2.0814	pabB; chorismate binding enzyme	
						4.500		4.5000		
**SGO_0310**	1.387	3.401	0.0144	0.0522	4.000	2.500	5.9643	2.6018	metE; 5-methyltetrahydropteroyltriglutamate--homocysteine S-methyltransferase	
						2.000		2.0000		
**SGO_0312**	0.735	11.059	0.0001	0.0000	441.500	387.000	658.3047	402.7546	xfp; D-xylulose 5-phosphate/D-fructose 6-phosphate phosphoketolase	
					472.500	398.000	674.3489	398.0000		
**SGO_0317**		8.639				177.500		184.7260	LPXTG cell wall surface protein, serine protease, subtilase family	
						214.000		214.0000		
**SGO_0321**	0.887	6.560	0.0098	0.0322	18.000	11.500	26.8391	11.9682	polypeptide deformylase	
					23.500	22.000	33.5390	22.0000		
**SGO_0323**	0.576	2.317			2.000		2.9821		pseudouridine synthase rRNA-specific	
						2.000		2.0000		
**SGO_0324**		5.618				27.500		28.6195	hypothetical protein SGO_0324	
						20.500		20.5000		
**SGO_0325**		3.058				8.000		8.3257	probable membrane protein, putative	
**SGO_0326**	-3.296	6.752	0.0097	0.0317	3.500	51.500	5.2187	53.5965	probable membrane protein, putative	
						49.000		49.0000		
**SGO_0327**	-0.856	3.376	0.0346	0.1462	1.500	3.500	2.2366	3.6425	Lipopolysaccharide N-acetylglucosaminyltransferase	
						4.500		4.5000		
**SGO_0328**		4.383				9.000		9.3664	transmembrane protein, putative	
						11.500		11.5000		
**SGO_0329**	-4.732	6.866	0.0187	0.0718		61.000		63.4833	hypothetical protein SGO_0329	
					1.500	51.000	2.1408	51.0000		

Show detected proteins only
Show all proteins
Filter by category:
ABC Transporter

Proteins found: 1179

Test	Cutoff
***q*-Value**	**.005**
p-Value	

Color	Signif	Direction	Applies To
red	yes	+	ratios, bars
yellow	no	n/a	bars
green	yes	−	ratios, bars
light red	yes	+	*p*- , *q*-Values
light green	yes	−	*p*- , *q*-Values

Dot Plots Dot Plots

Protein	*SgPg vs Sg* Log$_2$ Ratio	Log$_2$ Sum	*q*-Value	*p*-Value	Raw *SgPg*	Raw *Sg*	Normalized *SgPg*	Normalized *Sg*	Description
SGO_0330		5.031				17.000		17.6921	hypothetical protein SGO_0330
						15.000		15.0000	
SGO_0331		5.455				21.500		22.3753	hypothetical protein SGO_0331
						21.500		21.5000	
SGO_0332		5.722				19.000		19.7735	hypothetical protein SGO_0332
						33.000		33.0000	
SGO_0333	-0.089	8.419	0.0644	0.2989	55.000	77.500	82.0085	80.6550	rpsO; ribosomal protein S15
					58.500	96.000	83.4908	96.0000	
SGO_0337	2.161	3.452			6.000		8.9464		trx-2; thioredoxin
						2.000		2.0000	
SGO_0339	-0.424	2.389			1.500		2.2366		hypothetical protein SGO_0339
						3.000		3.0000	
SGO_0342	0.572	7.982	0.0036	0.0093	46.000	47.500	68.5889	49.4337	pepF-2; oligoendopeptidase
					58.000	52.000	82.7772	52.0000	
SGO_0344	-0.124	9.039	0.0049	0.0139	85.500	128.500	127.4860	133.7312	pnpA; polyribonucleotide nucleotidyltransferase
					87.000	140.500	124.1658	140.5000	
SGO_0345		3.663				4.000		4.1628	cysE; serine O-acetyltransferase
						8.500		8.5000	
SGO_0348	-1.348	4.537	0.0036	0.0090	3.500		5.2187		reductase
					3.500	13.000	4.9952	13.0000	
SGO_0349	0.002	6.611	0.1832	0.9875	16.500	22.000	24.6026	22.8956	cysS; cysteinyl-tRNA synthetase
					17.000	26.000	24.2623	26.0000	
SGO_0352	-0.745	7.834	0.0012	0.0022	31.500	66.000	46.9685	68.6868	ABC transporter, ATP-binding protein SP1580
					27.000	74.000	38.5342	74.0000	

Log$_2$ Ratios: -6 -4 -2 0 2 4 6

Show detected proteins only
Show all proteins
Filter by category:
ABC Transporter

Proteins found: 1179

Test	Cutoff
q-Value	.005
p-Value	

Color	Signif	Direction	Applies To
red	yes	+	ratios, bars
yellow	no	n/a	bars
green	yes	−	ratios, bars
pink	yes	+	*p*- , *q*-Values
light green	yes	−	*p*- , *q*-Values

Dot Plots Dot Plots

Protein	SgPg vs Sg: Log_2 Ratio	Log_2 Sum	*q*-Value	*p*-Value	Raw: *SgPg*	Raw: *Sg*	Normalized: *SgPg*	Normalized: *Sg*	Description	Log_2 Ratios (-6 -4 -2 0 2 4 6)
SGO_0353	0.905	4.160	0.0171	0.0639	5.000	3.500	7.4553	3.6425	transport protein	
					3.000	2.500	4.2816	2.5000		
SGO_0355	0.966	5.720	0.0046	0.0127	10.000	7.500	14.9106	7.8053	RNA methyltransferase, TrmH family, group 3	
					14.000	10.000	19.9807	10.0000		
SGO_0356		1.380				2.500		2.6018	hypothetical protein SGO_0356	
SGO_0357	1.449	7.485	0.0013	0.0025	46.000	17.500	68.5889	18.2124	degV; DegV family fatty acid binding protein	
					43.000	31.000	61.3693	31.0000		
SGO_0358	0.122	9.371	0.0784	0.3765	117.500	133.000	175.2000	138.4144	rplM; ribosomal protein L13	
					118.000	180.000	168.4088	180.0000		
SGO_0359	-0.466	8.319	0.0137	0.0485	43.000	76.000	64.1157	79.0939	rpsI; ribosomal protein S9	
					48.500	107.000	69.2189	107.0000		
SGO_0361	-0.553	4.546	0.0329	0.1384	4.000	8.000	5.9643	8.3257	immunity repressor protein	
					2.500	5.500	3.5680	5.5000		
SGO_0368		2.345				2.000		2.0814	merA; mercury(II) reductase	
						3.000		3.0000		
SGO_0371		2.000							putative transcriptional regulator	
						4.000		4.0000		
SGO_0372	1.166	6.049	0.0026	0.0062	15.000	13.000	22.3660	13.5292	malate oxidoreductase	
					16.000	7.500	22.8351	7.5000		
SGO_0374	-0.169	6.358	0.0363	0.1547	12.000	22.500	17.8928	23.4160	Response regulator of the LytR/AlgR family	
					14.500	20.000	20.6943	20.0000		
SGO_0376		3.322							ABC transporter ATP-binding protein	
						10.000		10.0000		

Show detected proteins only
Show all proteins
Filter by category:
ABC Transporter

Proteins found: 1179

Test	Cutoff
***q*-Value**	.005
p-Value	

	Signif	Direction	Applies To
red	yes	+	ratios, bars
yellow	no	n/a	bars
green	yes	−	ratios, bars
pink	yes	+	*p*-, *q*-Values
light green	yes	−	*p*-, *q*-Values

Dot Plots | Dot Plots

Protein	SgPg vs Sg Log_2 Ratio	Log_2 Sum	*q*-Value	*p*-Value	Raw *SgPg*	Raw *Sg*	Normalized *SgPg*	Normalized *Sg*	Description
SGO_0378									hypothetical protein SGO_0378
SGO_0379		4.594				3.500		3.6425	Protein of unknown function (DUF421) family
						20.500		20.5000	
SGO_0380		3.459							hypothetical protein SGO_0380
						11.000		11.0000	
SGO_0384	-0.385	5.773	0.0578	0.2637	6.500	10.500	9.6919	10.9275	putative carboxylate-amine/thiol ligase
					9.500	20.500	13.5583	20.5000	
SGO_0385		6.351				39.500		41.1080	exo-beta-D-fructosidase
						40.500		40.5000	
SGO_0387		4.194				7.500		7.8053	hypothetical protein SGO_0387
						10.500		10.5000	
SGO_0388		6.251				29.000		30.1806	LPXTG cell wall surface protein, zinc carboxypeptidase family
						46.000		46.0000	
SGO_0389		1.000							possible phosphoserine phosphatase
						2.000		2.0000	
SGO_0390	1.564	8.027	0.0010	0.0015	72.000	30.000	107.3566	31.2213	glycerol-3-phosphate dehydrogenase (NAD (P)+)
					61.500	34.500	87.7724	34.5000	
SGO_0392	2.875	5.566	0.0232	0.0922	13.000		19.3838		phosphoglycerate mutase
					17.500	3.000	24.9759	3.0000	
SGO_0393		5.105				10.000		10.4071	D-alanyl-D-alanine carboxypeptidase
						24.000		24.0000	
SGO_0396		2.322							Transcriptional regulator, PadR family
						5.000		5.0000	

Log_2 Ratios: -6 -4 -2 0 2 4 6

Show detected proteins only
Show all proteins
Filter by category:
ABC Transporter

Proteins found: 1179

Test	Cutoff
q-Value	.005
p-Value	

	Signif	Direction	Applies To
red	yes	+	ratios, bars
yellow	no	n/a	bars
green	yes	−	ratios, bars
pink	yes	+	*p*- , *q*-Values
light green	yes	−	

Dot Plots Dot Plots

Hendrickson *et al.*

Protein	SgPg vs Sg: Log_2 Ratio	Log_2 Sum	*q*-Value	*p*-Value	Raw: *SgPg*	Raw: *Sg*	Normalized: *SgPg*	Normalized: *Sg*	Description	Log_2 Ratios (-6 -4 -2 0 2 4 6)
SGO_0398	-2.695	6.928	0.0017	0.0033	7.500	58.500	11.1830	60.8815	ABC transporter ATP-binding protein	
					4.000	44.000	5.7088	44.0000		
SGO_0400		3.423				5.500		5.7239	hrcA; heat-inducible transcription repressor HrcA	
						5.000		5.0000		
SGO_0401	0.166	6.709	0.1742	0.9248	14.500	14.000	21.6204	14.5699	grpE; co-chaperone GrpE	
					22.000	37.000	31.3983	37.0000		
SGO_0402	0.461	11.142	0.0013	0.0022	424.500	434.500	632.9566	452.1883	dnaK; DnaK chaperone protein	
					473.000	499.000	675.0625	499.0000		
SGO_0404	-2.045	7.970	0.0057	0.0167	16.000	71.000	23.8570	73.8904	dnaJ; DnaJ chaparone protein	
					16.500	129.500	23.5487	129.5000		
SGO_0407	-0.231	2.696			2.000		2.9821		truA; tRNA pseudouridine synthase A	
						3.500		3.5000		
SGO_0408		8.890				232.000		241.4446	zmpB; zinc metalloproteinase B	
						233.000		233.0000		
SGO_0409	1.598	3.467	0.0233	0.0929	3.500	1.500	5.2187	1.5611	pyridoxine kinase	
					3.000		4.2816			
SGO_0411		4.454			8.000		11.9285		conserved hypothetical protein TIGR01440	
					7.000		9.9904			
SGO_0412	0.831	11.109	0.0005	0.0006	463.000	353.500	690.3625	367.8908	tig; trigger factor	
					506.000	428.500	722.1599	428.5000		
SGO_0413	2.022	7.008	0.0040	0.0107	27.000	15.500	40.2587	16.1310	DNA-directed RNA polymerase delta chain	
					44.000	9.500	62.7965	9.5000		
SGO_0415	-1.044	10.702	0.0005	0.0006	184.000	569.500	274.3557	592.6841	secA; preprotein translocase, SecA subunit	
					188.500	529.500	269.0260	529.5000		

Show detected proteins only
Show all proteins
Filter by category: ABC Transporter

Proteins found: 1179

Test	Cutoff
***q*-Value**	**.005**
p-Value	

Colour	Signif	Direction	Applies To
red	yes	+	ratios, bars
yellow	no	n/a	bars
green	yes	−	ratios, bars
pink	yes	+	*p*- , *q*-Values
light green	yes	−	*p*- , *q*-Values

Dot Plots Dot Plots

Protein	SgPg vs Sg Log2 Ratio	Log2 Sum	q-Value	p-Value	Raw SgPg	Raw Sg	Normalized SgPg	Normalized Sg	Description
SGO_0416	0.754	7.228	0.0087	0.0280	37.000	24.000	55.1694	24.9770	phospho-2-dehydro-3-deoxyheptonate aldolase
					27.500	30.500	39.2478	30.5000	
SGO_0418		3.870			5.500		8.2009		alr; alanine racemase
					4.500		6.4224		
SGO_0425	1.326	2.868			3.500	2.000	5.2187	2.0814	ansB; asparaginase
SGO_0426	-0.316	5.480	0.0492	0.2190	7.500	9.500	11.1830	9.8867	Cof family protein
					6.000	15.000	8.5632	15.0000	
SGO_0427	1.411	6.151	0.0006	0.0008	16.000	9.500	23.8570	9.8867	universal stress protein family
					19.500	9.500	27.8303	9.5000	
SGO_0429	-0.208	9.238	0.0673	0.3144	112.500	162.000	167.7447	168.5950	aspartate transaminase
					80.500	152.500	114.8891	152.5000	
SGO_0430		9.382				314.000		326.7828	LPXTG cell wall surface protein
						340.500		340.5000	
SGO_0431	0.147	5.310	0.1464	0.7587	6.500	6.500	9.6919	6.7646	GTP-sensing transcriptional pleiotropic repressor codY
					7.500	12.500	10.7040	12.5000	
SGO_0432	0.535	7.190	0.0066	0.0198	31.000	32.500	46.2230	33.8231	entB; isochorismatase family protein
					28.000	26.000	39.9614	26.0000	
SGO_0434	-0.559	7.587	0.0084	0.0265	23.000	49.000	34.2945	50.9948	aspS-2; aspartyl-tRNA synthetase
					30.500	63.500	43.5294	63.5000	
SGO_0435	-0.003	6.327	0.1779	0.9549	12.500	17.500	18.6383	18.2124	gatC; glutamyl-tRNA(Gln) amidotransferase, C subunit
					15.000	22.000	21.4079	22.0000	
SGO_0436	-0.300	9.301	0.0091	0.0294	99.500	179.500	148.3608	186.8074	gatA; glutamyl-tRNA(Gln) amidotransferase, A subunit
					94.000	161.500	134.1562	161.5000	

Log2 Ratios: -6 -4 -2 0 2 4 6

Show detected proteins only
Show all proteins
Filter by category: ABC Transporter

Proteins found: 1179

Test	Cutoff
q-Value	.005
p-Value	

	Signif	Direction	Applies To
red	yes	+	ratios, bars
yellow	no	n/a	bars
green	yes	−	ratios, bars
pink	yes	+	p- , q-Values
light green	yes	−	p- , q-Values

Dot Plots Dot Plots

Protein	*SgPg vs Sg* Log_2 Ratio	Log_2 Sum	*q*-Value	*p*-Value	Raw *SgPg*	Raw *Sg*	Normalized *SgPg*	Normalized *Sg*	Description	Log_2 Ratios
SGO_0437	0.549	8.996	0.0018	0.0038	95.000	100.000	141.6511	104.0710	gatB; glutamyl-tRNA(Gln) amidotransferase, B subunit	
					113.500	103.000	161.9865	103.0000		
SGO_0440	1.808	4.908	0.0017	0.0035	9.000	3.000	13.4196	3.1221	L-iditol 2-dehydrogenase BH3949	
					7.000	3.500	9.9904	3.5000		
SGO_0444		3.087							HAD superfamily (subfamily IIIA) phosphatase, TIGR01668	
						8.500		8.5000		
SGO_0445	0.374	6.781	0.0137	0.0486	23.000	21.000	34.2945	21.8549	GTP-binding protein	
					19.500	26.000	27.8303	26.0000		
SGO_0446		3.585							conserved hypothetical protein TIGR00253	
						12.000		12.0000		
SGO_0447	-0.822	5.135	0.0144	0.0518	6.000	10.000	8.9464	10.4071	nadD; nicotinate (nicotinamide) nucleotide adenylyltransferase	
					3.000	11.500	4.2816	11.5000		
SGO_0448	0.170	5.137	0.1330	0.6801	6.500	10.500	9.6919	10.9275	conserved hypothetical protein TIGR00488	
					6.000	6.000	8.5632	6.0000		
SGO_0449		1.585							isochorismatase family protein	
						3.000		3.0000		
SGO_0450	1.018	4.508	0.0199	0.0773	6.500		9.6919		iojap-related protein	
					6.000	4.500	8.5632	4.5000		
SGO_0451		3.791			4.500		6.7098		methyltransferase	
					5.000		7.1360			
SGO_0453		7.985				105.000		109.2745	lipoprotein, putative	
						144.000		144.0000		
SGO_0454	0.236	7.740	0.0327	0.1365	37.500	54.000	55.9149	56.1983	conserved hypothetical protein TIGR01033	
					41.500	42.500	59.2285	42.5000		

Log_2 Ratios axis: -6 -4 -2 0 2 4 6

Show detected proteins only
Show all proteins
Filter by category:
ABC Transporter

Proteins found: 1179

Test	Cutoff
***q*-Value**	**.005**
p-Value	

Color	Signif	Direction	Applies To
red	yes	+	ratios, bars
yellow	no	n/a	bars
green	yes	−	ratios, bars
pink	yes	+	*p*- , *q*-Values
light green	yes	−	*p*- , *q*-Values

Dot Plots Dot Plots

Streptococcus gordonii

Protein	SgPg vs Sg				Raw		Normalized		Description
	Log_2 Ratio	Log_2 Sum	*q*-Value	*p*-Value	*SgPg*	*Sg*	*SgPg*	*Sg*	
SGO_0455	-2.675	8.428	0.0004	0.0005	14.500	155.500	21.6204	161.8303	lipoprotein, putative
					17.500	136.000	24.9759	136.0000	
SGO_0456		3.732				7.000		7.2850	ILL5; amino acid aminohydrolase
						6.000		6.0000	
SGO_0457	-1.812	9.410	0.0002	0.0001	48.500	245.000	72.3166	254.9739	ABC transporter, substrate-binding protein SP0148
					55.000	274.500	78.4956	274.5000	
SGO_0458	-1.995	10.913	0.0005	0.0005	128.000	801.500	190.8562	834.1288	hlpA; lipoprotein
					136.500	708.500	194.8119	708.5000	
SGO_0459	-1.190	5.463	0.0169	0.0627	5.500	10.000	8.2009	10.4071	succinyl-diaminopimelate desuccinylase
					3.500	20.500	4.9952	20.5000	
SGO_0460	-2.214	7.691	0.0008	0.0013	13.000	91.000	19.3838	94.7046	ABC transporter, ATP-binding protein SP0151
					12.000	75.500	17.1263	75.5000	
SGO_0463		2.723				2.500		2.6018	cydD; putative ABC transporter (ATP-binding protein)
						4.000		4.0000	
SGO_0468	-0.141	6.233	0.1049	0.5199	11.000	12.000	16.4017	12.4885	hypothetical protein SGO_0468
					12.500	28.500	17.8399	28.5000	
SGO_0469		4.352				10.500		10.9275	integral membrane protein
						9.500		9.5000	
SGO_0476	1.201	6.277	0.0008	0.0012	19.500	12.000	29.0757	12.4885	rhodanese family protein
					17.500	11.000	24.9759	11.0000	
SGO_0480	2.150	5.482	0.0231	0.0917	15.000		22.3660		hypothetical protein SGO_0480
					12.500	4.500	17.8399	4.5000	
SGO_0483	0.911	6.419	0.0108	0.0362	15.000	17.000	22.3660	17.6921	hypothetical protein SGO_0483
					23.500	12.000	33.5390	12.0000	

Log_2 Ratios: -6 -4 -2 0 2 4 6

Show detected proteins only
Show all proteins
Filter by category:
ABC Transporter

Proteins found: 1179

Test	Cutoff
q-Value	.005
p-Value	

	Signif	Direction	Applies To
red	yes	+	ratios, bars
yellow	no	n/a	bars
green	yes	−	ratios, bars
pink	yes	+	*p*- , *q*-Values
light green	yes	−	*p*- , *q*-Values

Dot Plots Dot Plots

Protein	*SgPg vs Sg*				Raw		Normalized		Description	Log_2 Ratios
	Log_2 Ratio	Log_2 Sum	*q*-Value	*p*-Value	*SgPg*	*Sg*	*SgPg*	*Sg*		-6 -4 -2 0 2 4 6
SGO_0484		4.515				9.000		9.3664	sensor histidine kinase	
						13.500		13.5000		
SGO_0488	-2.054	6.608	0.0002	0.0001	6.000	39.500	8.9464	41.1080	ABC transporter, ATP-binding protein SP0483	
					7.000	37.500	9.9904	37.5000		
SGO_0491		3.343			2.500		3.7277		gidB; methyltransferase GidB	
					4.500		6.4224			
SGO_0494	1.064	6.136	0.0009	0.0013	16.500	9.500	24.6026	9.8867	lemA; LemA-like protein	
					16.000	13.000	22.8351	13.0000		
SGO_0495	-0.397	4.932	0.0987	0.4863	5.500	8.000	8.2009	8.3257	htpx; heat shock protein	
						14.000		14.0000		
SGO_0497									gtfG; glucosyltransferase G	
SGO_0500		2.058				4.000		4.1628	rggD; putative transcriptional regulator RggD	
SGO_0501	0.653	6.885	0.0004	0.0004	25.000	22.500	37.2766	23.4160	Uncharacterized ACR, COG1399	
					24.500	22.500	34.9662	22.5000		
SGO_0502	-0.710	9.878	0.0016	0.0032	133.000	291.000	198.3115	302.8465	floL; flotillin-like protein	
					112.000	280.000	159.8457	280.0000		
SGO_0503	0.873	10.962	0.0003	0.0002	420.000	350.000	626.2468	364.2484	gnd; 6-phosphogluconate dehydrogenase, decarboxylating	
					465.000	340.000	663.6449	340.0000		
SGO_0505	-3.384	10.986	0.0001	0.0000	53.500	911.000	79.7719	948.0865	PTS system, IIBC component	
					69.000	902.500	98.4763	902.5000		
SGO_0506	-1.165	3.532							rgfB; RgfB	
					2.500	8.000	3.5680	8.0000		

- Show detected proteins only
- Show all proteins
- Filter by category: ABC Transporter

Proteins found: 1179

Test	Cutoff
***q*-Value**	**.005**
p-Value	

Color	Signif	Direction	Applies To
red	yes	+	ratios, bars
yellow	no	n/a	bars
green	yes	−	ratios, bars
light red	yes	+	*p*- , *q*-Values
light green	yes	−	*p*- , *q*-Values

Dot Plots | Dot Plots

Streptococcus gordonii

Hackett Laboratory UW

Protein	SgPg vs Sg Log2 Ratio	Log2 Sum	*q*-Value	*p*-Value	Raw SgPg	Raw Sg	Normalized SgPg	Normalized Sg	Description
SGO_0508	-0.519	6.409	0.0010	0.0016	11.000	24.500	16.4017	25.4974	nrdR; transcriptional regulator, NrdR family
					13.000	24.500	18.5535	24.5000	
SGO_0509	-1.245	4.446	0.0735	0.3492		5.500		5.7239	Replication initiation and membrane attachment protein (DnaB) superfamily
					2.500	12.500	3.5680	12.5000	
SGO_0510	1.688	6.091	0.0010	0.0015	19.000	6.500	28.3302	6.7646	dnaI; primosomal protein DnaI
					16.500	9.500	23.5487	9.5000	
SGO_0511	0.194	5.117	0.1773	0.9470	6.000	4.500	8.9464	4.6832	NADPH-flavin oxidoreductase -like protein
					6.000	12.500	8.5632	12.5000	
SGO_0512	0.530	8.008	0.0100	0.0333	50.000	61.000	74.5532	63.4833	GTP-binding protein engA
					53.500	43.000	76.3548	43.0000	
SGO_0513		2.170							Snf2 family protein
						4.500		4.5000	
SGO_0514		3.791			4.500		6.7098		hypothetical protein SGO_0514
					5.000		7.1360		
SGO_0515	0.105	8.471	0.0142	0.0507	61.500	85.500	91.7004	88.9807	murC; UDP-N-acetylmuramate--alanine ligase
					64.500	82.000	92.0540	82.0000	
SGO_0518	-1.438	8.879	0.0001	0.0001	44.000	170.000	65.6068	176.9206	aminodeoxychorismate lyase-like protein
					43.000	167.000	61.3693	167.0000	
SGO_0519	0.526	7.415	0.0087	0.0280	38.000	31.000	56.6604	32.2620	greA; transcription elongation factor greA
					31.000	37.500	44.2430	37.5000	
SGO_0521	-1.082	6.092	0.0080	0.0248	9.000	18.000	13.4196	18.7328	Membrane protein oxaA 2 precursor
					6.000	27.500	8.5632	27.5000	
SGO_0523	0.164	5.107	0.1254	0.6344	6.500		9.6919		spoU rRNA Methylase family protein
					10.000	10.500	14.2719	10.5000	

Log2 Ratios: -6 -4 -2 0 2 4 6

Show detected proteins only
Show all proteins
Filter by category:
ABC Transporter

Proteins found: 1179

Test	Cutoff
q-Value	.005
p-Value	

	Signif	Direction	Applies To
red	yes	+	ratios, bars
yellow	no	n/a	bars
green	yes	−	ratios, bars
pink	yes	+	*p*- , *q*-Values
light green	yes	−	*p*- , *q*-Values

Dot Plots Dot Plots

SgPg vs Sg

Streptococcus gordonii

Hackett Laboratory UW

Summary Table | SgFn vs Sg | SgPg vs Sg | SgPgFn vs Sg | SgPg vs SgFn | SgPgFn vs SgFn | SgPgFn vs SgPg | Coverage

Protein	*SgPg vs Sg* Log$_2$ Ratio	Log$_2$ Sum	*q*-Value	*p*-Value	Raw *SgPg*	Raw *Sg*	Normalized *SgPg*	Normalized *Sg*	Description
**SGO_0526**	0.991	6.225	0.0078	0.0239	18.000	8.500	26.8391	8.8460	ilvB; acetolactate synthase, large subunit, biosynthetic type
					15.500	17.000	22.1215	17.0000	
**SGO_0527**	-0.395	5.587	0.0288	0.1179	5.500	12.500	8.2009	13.0089	ilvN; acetolactate synthase, small subunit
					9.000	14.000	12.8447	14.0000	
**SGO_0528**	0.581	8.528	0.0018	0.0039	79.000	68.000	117.7940	70.7683	ilvC; ketol-acid reductoisomerase
					72.500	77.000	103.4715	77.0000	
**SGO_0529**	1.401	4.686	0.0093	0.0302	5.000	2.000	7.4553	2.0814	ilvA; threonine dehydratase
					7.500	5.500	10.7040	5.5000	
**SGO_0530**	-1.277	5.023	0.0059	0.0174	2.000	10.000	2.9821	10.4071	Cof family protein
					5.000	12.000	7.1360	12.0000	
**SGO_0533**	0.871	2.143				1.500		1.5611	conserved hypothetical protein TIGR00150
					2.000		2.8544		
**SGO_0535**	-2.237	6.905	0.0008	0.0011	6.000	42.500	8.9464	44.2302	putative transcriptional regulator LytR
					8.500	54.500	12.1311	54.5000	
**SGO_0536**	-0.177	6.995	0.0193	0.0745	21.000	30.500	31.3123	31.7416	hypothetical protein SGO_0536
					20.000	36.000	28.5439	36.0000	
**SGO_0537**	1.124	6.487	0.0008	0.0011	20.500	11.500	30.5668	11.9682	HIT family protein
					21.500	16.500	30.6847	16.5000	
**SGO_0538**	-2.541	6.463	0.0012	0.0020	3.500	41.000	5.2187	42.6691	ABC transporter, ATP-binding protein SP0522
					5.500	32.500	7.8496	32.5000	
**SGO_0539**		4.667				10.000		10.4071	ABC transporter, permease protein
						15.000		15.0000	
**SGO_0540**	0.148	7.214	0.0131	0.0456	25.500	35.500	38.0221	36.9452	hypothetical protein SGO_0540
					28.000	33.500	39.9614	33.5000	

Log$_2$ Ratios: -6 -4 -2 0 2 4 6

◉ Show detected proteins only
○ Show all proteins
☐ Filter by category: ABC Transporter

Proteins found: 1179

Test	Cutoff
***q*-Value**	**.005**
p-Value	

	Signif	Direction	Applies To
red	yes	+	ratios, bars
yellow	no	n/a	bars
green	yes	–	ratios, bars
pink	yes	+	*p*- , *q*-Values
light green	yes	–	*p*- , *q*-Values

Dot Plots | Dot Plots

Hendrickson *et al.*

Protein	SgPg vs Sg: Log_2 Ratio	Log_2 Sum	*q*-Value	*p*-Value	Raw: *SgPg*	Raw: *Sg*	Normalized: *SgPg*	Normalized: *Sg*	Description
SGO_0541	-1.602	5.480	0.0030	0.0073	2.500	14.000	3.7277	14.5699	methyltransferase, putative
					5.500	18.500	7.8496	18.5000	
SGO_0542	-0.865	3.332							hypothetical protein SGO_0542
					2.500	6.500	3.5680	6.5000	
SGO_0543	-0.585	8.276	0.0002	0.0001	42.000	87.500	62.6247	91.0621	nusA; transcription termination factor NusA
					43.000	95.000	61.3693	95.0000	
SGO_0544		2.000							Protein of unknown function (DUF448) superfamily
						4.000		4.0000	
SGO_0545		1.807							ribosomal protein L7A family
						3.500		3.5000	
SGO_0546	-0.138	8.842	0.0729	0.3461	77.500	132.500	115.5574	137.8940	infB; Translation initiation factor IF-2
					71.500	103.500	102.0443	103.5000	
SGO_0547		1.098							rbfA; ribosome-binding factor A
					1.500		2.1408		
SGO_0548	-2.975	7.822	0.0004	0.0004	8.000	105.000	11.9285	109.2745	Na/Pi-cotransporter family protein
					9.500	91.500	13.5583	91.5000	
SGO_0549	3.756	5.724			33.000	3.500	49.2051	3.6425	nagA; N-acetylglucosamine-6-phosphate deacetylase
SGO_0552	2.595	6.355	0.0004	0.0004	22.500	3.500	33.5489	3.6425	oxidoreductase, aldo/keto reductase family
					25.000	9.000	35.6798	9.0000	
SGO_0554	-0.957	6.566	0.0297	0.1216	8.500	18.000	12.6740	18.7328	hsdR; type I site-specific deoxyribonuclease
					12.500	45.500	17.8399	45.5000	
SGO_0556		3.279				5.000		5.2035	DNA helicase-like protein, putative
						4.500		4.5000	

Log_2 Ratios: -6 -4 -2 0 2 4 6

Show detected proteins only
Show all proteins
Filter by category:
ABC Transporter

Proteins found: 1179

Test	Cutoff
***q*-Value**	**.005**
p-Value	

	Signif	Direction	Applies To
(red)	yes	+	ratios, bars
(yellow)	no	n/a	bars
(green)	yes	−	ratios, bars
(pink)	yes	+	*p*- , *q*-Values
(light green)	yes	−	

Dot Plots Dot Plots

Protein	SgPg vs Sg Log2 Ratio	Log2 Sum	q-Value	p-Value	Raw SgPg	Raw Sg	Normalized SgPg	Normalized Sg	Description
SGO_0557		1.807							HsdS specificity protein of type I restriction-modification system
						3.500		3.5000	
SGO_0558	0.356	4.186	0.1015	0.5010	5.500		8.2009		hypothetical protein SGO_0558
					3.500	5.000	4.9952	5.0000	
SGO_0560	0.516	5.703	0.0024	0.0054	11.000	10.000	16.4017	10.4071	hsdM; type I restriction-modification system, M subunit
					10.000	11.000	14.2719	11.0000	
SGO_0565	0.214	8.069	0.0140	0.0500	45.500	56.500	67.8434	58.8001	adhA; alcohol dehydrogenase
					53.500	65.500	76.3548	65.5000	
SGO_0566		7.073				53.000		55.1576	sgc; serine protease challisin
						79.500		79.5000	
SGO_0568	0.047	7.232	0.0949	0.4663	24.000	34.500	35.7855	35.9045	glyQ; glycyl-tRNA synthetase, alpha subunit
					28.500	38.000	40.6750	38.0000	
SGO_0569	0.474	8.445	0.0001	0.0001	67.000	70.000	99.9013	72.8497	glyS; glycyl-tRNA synthetase, beta subunit
					72.000	73.000	102.7579	73.0000	
SGO_0570	-0.455	4.468	0.0111	0.0375	4.500		6.7098		hypothetical protein SGO_0570
					4.500	9.000	6.4224	9.0000	
SGO_0571		1.322							hypothetical protein SGO_0571
						2.500		2.5000	
SGO_0573	-0.163	6.619	0.0727	0.3439	16.000	20.500	23.8570	21.3345	mraW; S-adenosyl-methyltransferase MraW
					15.500	31.000	22.1215	31.0000	
SGO_0575	-3.188	7.054	0.0001	0.0001	4.500	55.000	6.7098	57.2390	pbp2X; penicillin-binding protein 2X
					4.500	62.500	6.4224	62.5000	
SGO_0576		3.087							mraY; phospho-N-acetylmuramoyl-pentapeptide-transferase
						8.500		8.5000	

Log2 Ratios: -6 -4 -2 0 2 4 6

Show detected proteins only
Show all proteins
Filter by category:
ABC Transporter

Proteins found: 1179

Test	Cutoff
q-Value	.005
p-Value	

Color	Signif	Direction	Applies To
red	yes	+	ratios, bars
yellow	no	n/a	bars
green	yes	−	ratios, bars
pink	yes	+	p- , q-Values
light green	yes	−	p- , q-Values

Dot Plots Dot Plots

Streptococcus gordonii

Protein	SgPg vs Sg Log2 Ratio	Log2 Sum	q-Value	p-Value	Raw SgPg	Raw Sg	Normalized SgPg	Normalized Sg	Description
SGO_0577	0.720	7.329	0.0064	0.0189	35.500	23.500	52.9328	24.4567	ATP-dependent RNA helicase
					32.500	37.000	46.3838	37.0000	
SGO_0578		6.489				44.500		46.3116	amino acid ABC transporter permease protein
						43.500		43.5000	
SGO_0579	-2.330	7.076	0.0002	0.0001	9.000	55.500	13.4196	57.7594	amino acid ABC transporter ATP binding protein
					6.500	54.500	9.2768	54.5000	
SGO_0581	0.719	6.131	0.0084	0.0264	12.500	11.000	18.6383	11.4478	trxB; thioredoxin-disulfide reductase
					17.500	15.000	24.9759	15.0000	
SGO_0582	1.890	7.748	0.0025	0.0058	47.000	18.000	70.0800	18.7328	nicotinate phosphoribosyltransferase, putative
					69.500	27.000	99.1899	27.0000	
SGO_0583	1.345	5.936	0.0033	0.0083	14.000	5.500	20.8749	5.7239	nadE; NAD+ synthetase
					15.500	12.500	22.1215	12.5000	
SGO_0585	0.921	7.164	0.0065	0.0193	36.000	19.500	53.6783	20.2938	pepC; aminopeptidase C
					28.000	29.500	39.9614	29.5000	
SGO_0586	-1.402	8.842	0.0003	0.0003	47.000	154.500	70.0800	160.7896	pbp1a; penicillin-binding protein 1A
					39.500	171.500	56.3741	171.5000	
SGO_0589	1.105	7.429	0.0068	0.0204	31.500	24.000	46.9685	24.9770	methylase
					50.000	29.000	71.3597	29.0000	
SGO_0590	-0.529	5.876	0.0308	0.1271	10.000	13.500	14.9106	14.0496	Methyltransferase
					6.500	20.500	9.2768	20.5000	
SGO_0591	-2.495	8.975	0.0000	0.0000	25.500	208.000	38.0221	216.4676	hypothetical protein SGO_0591
					26.500	211.000	37.8206	211.0000	
SGO_0592	1.171	5.856	0.0003	0.0002	13.000	8.000	19.3838	8.3257	luxS; autoinducer-2 production protein LuxS
					14.500	9.500	20.6943	9.5000	

Log2 Ratios: -6 -4 -2 0 2 4 6

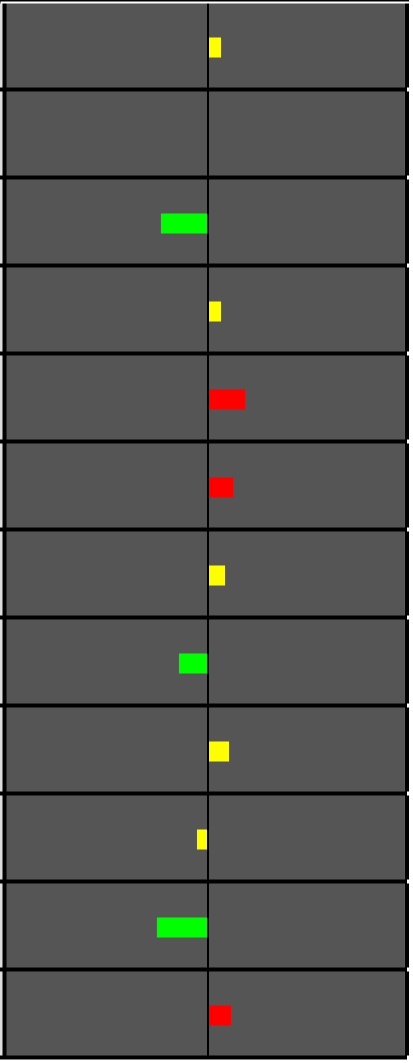

Show detected proteins only
Show all proteins
Filter by category:
ABC Transporter

Proteins found: 1179

Test	Cutoff
q-Value	.005
p-Value	

	Signif	Direction	Applies To
red	yes	+	ratios, bars
yellow	no	n/a	bars
green	yes	−	ratios, bars
pink	yes	+	p- , q-Values
light green	yes	−	p- , q-Values

Dot Plots Dot Plots

Hendrickson *et al.*

Protein	SgPg vs Sg Log2 Ratio	Log2 Sum	*q*-Value	*p*-Value	Raw *SgPg*	Raw *Sg*	Normalized *SgPg*	Normalized *Sg*	Description
SGO_0593	-2.047	9.043	0.0002	0.0001	37.500	196.000	55.9149	203.9791	HD/KH domain protein
					33.000	220.500	47.0974	220.5000	
SGO_0594	-0.071	6.280	0.1194	0.6001	11.500	21.500	17.1472	22.3753	gmk; Guanylate kinase (GMP kinase)
					14.500	17.500	20.6943	17.5000	
SGO_0595	-0.368	7.248	0.0003	0.0002	22.000	40.500	32.8034	42.1487	DNA-directed RNA polymerase, omega subunit
					23.500	43.500	33.5390	43.5000	
SGO_0596		1.643				3.000		3.1221	priA; primosomal protein N''
SGO_0597	1.413	5.785	0.0025	0.0057	14.000	5.000	20.8749	5.2035	fmt; methionyl-tRNA formyltransferase
					13.000	10.500	18.5535	10.5000	
SGO_0598	0.767	5.065	0.0098	0.0324	8.500	6.000	12.6740	6.2443	sun; sun protein
					6.000	6.000	8.5632	6.0000	
SGO_0599	-0.624	7.019	0.0012	0.0022	18.500	39.000	27.5847	40.5877	phosphoprotein phosphatase
					16.500	38.000	23.5487	38.0000	
SGO_0600	-2.409	8.059	0.0001	0.0000	15.500	105.000	23.1115	109.2745	serine/threonine protein kinase
					13.500	115.000	19.2671	115.0000	
SGO_0602		4.115				8.000		8.3257	histidine kinase
						9.000		9.0000	
SGO_0603	-0.916	5.074	0.0148	0.0537	2.500	9.500	3.7277	9.8867	response regulator
					6.000	11.500	8.5632	11.5000	
SGO_0604	0.579	8.119	0.0028	0.0068	54.000	47.500	80.5174	49.4337	hydrolase, haloacid dehalogenase family/peptidyl-prolyl cis-trans isomerase, cyclophilin type
					60.000	62.500	85.6316	62.5000	
SGO_0606	-0.319	9.299	0.0034	0.0086	89.000	162.000	132.7047	168.5950	cysK; cysteine synthase A
					103.500	181.000	147.7145	181.0000	

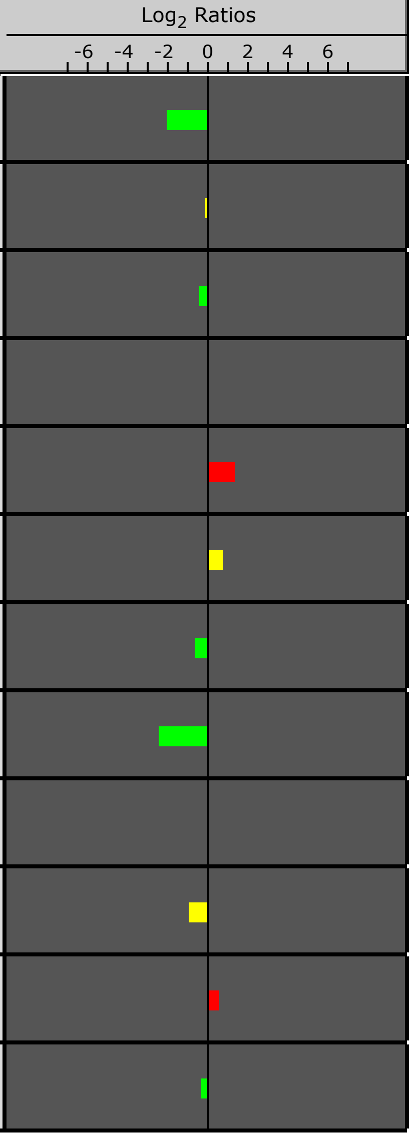

Show detected proteins only
Show all proteins
Filter by category:
ABC Transporter

Proteins found: 1179

Test	Cutoff
q-Value	.005
p-Value	

Color	Signif	Direction	Applies To
red	yes	+	ratios, bars
yellow	no	n/a	bars
green	yes	–	ratios, bars
light red	yes	+	*p*- , *q*-Values
light green	yes	–	*p*- , *q*-Values

Dot Plots Dot Plots

Protein	*SgPg* vs *Sg* Log$_2$ Ratio	Log$_2$ Sum	*q*-Value	*p*-Value	Raw *SgPg*	Raw *Sg*	Normalized *SgPg*	Normalized *Sg*	Description	Log$_2$ Ratios (-6 -4 -2 0 2 4 6)
**SGO_0608**		4.069				7.000		7.2850	comFA; competence ComFA-like protein	
						9.500		9.5000		
**SGO_0610**	0.727	8.578	0.0016	0.0031	84.500	75.000	125.9949	78.0532	ribosomal subunit interface protein	
					78.500	66.000	112.0347	66.0000		
**SGO_0626**	-1.293	5.901	0.0004	0.0005	5.000	20.000	7.4553	20.8142	recX; Regulatory protein recX	
					7.000	21.500	9.9904	21.5000		
**SGO_0628**		1.322							ymdC; 3''-aminoglycoside phosphotransferase-like protein (kanamycin kinase)	
						2.500		2.5000		
**SGO_0629**	-0.706	3.714	0.0791	0.3819	2.000	3.500	2.9821	3.6425	rumA-1; 23S rRNA (uracil-5-)-methyltransferase RumA	
						6.500		6.5000		
**SGO_0631**	3.710	6.407	0.0052	0.0151	28.000	3.000	41.7498	3.1221	alpha-glycerophosphate oxidase	
					28.000		39.9614			
**SGO_0632**		5.243			12.000		17.8928		glpK; glycerol kinase	
					14.000		19.9807			
**SGO_0635**		2.000							hypothetical protein SGO_0635	
						4.000		4.0000		
**SGO_0636**		2.930				3.000		3.1221	hypothetical protein SGO_0636	
						4.500		4.5000		
**SGO_0639**	0.402	8.251	0.0020	0.0044	55.500	65.500	82.7540	68.1665	valS; valyl-tRNA synthetase	
					63.500	63.000	90.6268	63.0000		
**SGO_0640**	-0.116	4.207	0.1194	0.5995	2.500	6.000	3.7277	6.2443	modification methylase	
					3.500	3.500	4.9952	3.5000		
**SGO_0641**	0.103	6.975	0.0807	0.3916	22.500	32.500	33.5489	33.8231	ATPase, histidine kinase-, DNA gyrase B-, and HSP90-like domain protein protein	
					22.000	27.000	31.3983	27.0000		

Show detected proteins only
Show all proteins
Filter by category:
ABC Transporter

Proteins found: 1179

Test	Cutoff
***q*-Value**	.005
p-Value	

	Signif	Direction	Applies To
red	yes	+	ratios, bars
yellow	no	n/a	bars
green	yes	–	ratios, bars
pink	yes	+	*p*- , *q*-Values
light green	yes	–	*p*- , *q*-Values

Dot Plots | Dot Plots

Hendrickson *et al.*

Protein	SgPg vs Sg Log$_2$ Ratio	SgPg vs Sg Log$_2$ Sum	SgPg vs Sg *q*-Value	SgPg vs Sg *p*-Value	Raw *SgPg*	Raw *Sg*	Normalized *SgPg*	Normalized *Sg*	Description
SGO_0642	-1.357	7.044	0.0027	0.0063	15.000	39.500	22.3660	41.1080	hypothetical protein SGO_0642
					10.500	53.500	14.9855	53.5000	
SGO_0643	-0.367	4.720	0.0490	0.2176	3.000	6.000	4.4732	6.2443	cytosine-specific methyltransferase
					5.000	8.500	7.1360	8.5000	
SGO_0644	1.368	6.862	0.0033	0.0081	33.000	16.000	49.2051	16.6514	hypothetical protein SGO_0644
					24.500	15.500	34.9662	15.5000	
SGO_0649		2.322							hypothetical protein SGO_0649
						5.000		5.0000	
SGO_0652	-3.768	7.285	0.0002	0.0001	6.500	71.000	9.6919	73.8904	hypothetical protein SGO_0652
					2.000	69.500	2.8544	69.5000	
SGO_0653	-0.400	4.660	0.0528	0.2379	4.500		6.7098		conserved hypothetical protein of unknown function (DUF1027)
					6.000	10.000	8.5632	10.0000	
SGO_0654	-0.815	8.095	0.0039	0.0102	30.000	74.000	44.7319	77.0125	radical SAM enzyme, Cfr family
					38.000	97.500	54.2333	97.5000	
SGO_0656	-0.846	5.780	0.0012	0.0020	7.500	17.000	11.1830	17.6921	trpB-2; tryptophan synthase, beta subunit
					6.000	17.500	8.5632	17.5000	
SGO_0657		1.322							trpE; anthranilate synthase component I
						2.500		2.5000	
SGO_0662		1.585							trpB-1; tryptophan synthase, beta subunit
						3.000		3.0000	
SGO_0663		1.000							trpA-2; tryptophan synthase, alpha subunit
						2.000		2.0000	
SGO_0665	-0.422	10.290	0.0218	0.0858	165.000	405.500	246.0255	422.0077	non-heme iron-containing ferritin
					200.000	298.500	285.4387	298.5000	

Log$_2$ Ratios: -6 -4 -2 0 2 4 6

Show detected proteins only
Show all proteins
Filter by category:
ABC Transporter

Proteins found: 1179

Test	Cutoff
q-Value	.005
p-Value	

	Signif	Direction	Applies To
red	yes	+	ratios, bars
yellow	no	n/a	bars
green	yes	−	ratios, bars
pink	yes	+	*p*- , *q*-Values
light green	yes	−	*p*- , *q*-Values

Dot Plots Dot Plots

Protein	SgPg vs Sg Log$_2$ Ratio	Log$_2$ Sum	*q*-Value	*p*-Value	Raw *SgPg*	Raw *Sg*	Normalized *SgPg*	Normalized *Sg*	Description
SGO_0667	-1.961	5.142	0.0011	0.0019	3.000	12.000	4.4732	12.4885	rhodanese family protein
					2.000	15.500	2.8544	15.5000	
SGO_0669	0.694	8.122	0.0002	0.0001	57.500	49.000	85.7362	50.9948	typA; GTP-binding protein TypA
					60.500	55.500	86.3452	55.5000	
SGO_0671	0.437	7.176	0.0084	0.0267	31.000	30.500	46.2230	31.7416	murD; UDP-N-acetylmuramoylalanine--D-glutamate ligase
					26.000	29.500	37.1070	29.5000	
SGO_0672	-1.494	6.719	0.0073	0.0224	5.500	42.500	8.2009	44.2302	murG; undecaprenyl-PP-MurNAc-pentapeptide-UDPGlcNAc GlcNAc transferase
					15.000	31.500	21.4079	31.5000	
SGO_0673	-2.932	6.419	0.0241	0.0961	3.500	33.500	5.2187	34.8638	DivIB; cell division protein DivIB
						45.500		45.5000	
SGO_0674	0.359	9.105	0.0012	0.0021	107.500	114.000	160.2894	118.6409	ftsA; cell division protein FtsA
					104.500	122.500	149.1417	122.5000	
SGO_0675	0.507	9.775	0.0084	0.0266	171.500	145.500	255.7174	151.4232	ftsZ; cell division protein FtsZ
					179.000	213.500	255.4676	213.5000	
SGO_0676	0.129	6.116	0.1080	0.5380	10.000	13.500	14.9106	14.0496	conserved hypothetical protein TIGR00044
					15.000	19.000	21.4079	19.0000	
SGO_0677	-0.679	8.372	0.0077	0.0236	37.500	111.000	55.9149	115.5188	ylmF protein
					50.000	88.500	71.3597	88.5000	
SGO_0680	0.819	9.042	0.0017	0.0035	103.000	93.500	153.5796	97.3063	cell division protein DivIVA
					128.500	93.000	183.3944	93.0000	
SGO_0681	0.809	8.932	0.0014	0.0026	106.000	74.500	158.0528	77.5329	ileS; isoleucyl-tRNA synthetase
					106.500	101.000	151.9961	101.0000	
SGO_0684	-1.462	8.112	0.0000	0.0000	25.000	97.000	37.2766	100.9488	hypothetical protein SGO_0684
					25.500	102.000	36.3934	102.0000	

Log$_2$ Ratios: -6 -4 -2 0 2 4 6

Show detected proteins only
Show all proteins
Filter by category:
ABC Transporter

Proteins found: 1179

Test	Cutoff
q-Value	.005
p-Value	

	Signif	Direction	Applies To
red	yes	+	ratios, bars
yellow	no	n/a	bars
green	yes	−	ratios, bars
pink	yes	+	*p*- , *q*-Values
light green	yes	−	*p*- , *q*-Values

Dot Plots Dot Plots

Streptococcus gordonii

Hackett Laboratory UW

Summary Table | SgFn vs Sg | SgPg vs Sg | SgPgFn vs Sg | SgPg vs SgFn | SgPgFn vs SgFn | SgPgFn vs SgPg | Coverage

	SgPg vs Sg				Raw		Normalized			Log_2 Ratios
Protein	Log_2 Ratio	Log_2 Sum	*q*-Value	*p*-Value	*SgPg*	*Sg*	*SgPg*	*Sg*	Description	-6 -4 -2 0 2 4 6
SGO_0685		2.170							MutT/nudix family protein	
						4.500		4.5000		
SGO_0688	-0.104	8.168	0.0159	0.0586	48.000	73.500	71.5711	76.4922	ATP dependent Clp protease, ATP-binding subunit, ClpE	
					47.000	72.500	67.0781	72.5000		
SGO_0689		3.170							hypothetical protein SGO_0689	
						9.000		9.0000		
SGO_0690	0.338	3.866	0.1779	0.9520		2.000		2.0814	folD; methenyltetrahydrofolate cyclohydrolase	
					3.500	7.500	4.9952	7.5000		
SGO_0693	-2.268	5.513	0.0024	0.0053	4.500	20.000	6.7098	20.8142	xseA; exodeoxyribonuclease VII, large subunit	
					1.500	16.000	2.1408	16.0000		
SGO_0694	0.972	4.814			12.500		18.6383		xseB; exodeoxyribonuclease VII, small subunit	
						9.500		9.5000		
SGO_0695		3.321							geranyltranstransferase	
					7.000		9.9904			
SGO_0696		3.029				4.000		4.1628	hemolysin-like protein	
						4.000		4.0000		
SGO_0697	0.936	3.253	0.0636	0.2947	3.000	1.500	4.4732	1.5611	transcription regulator	
						3.500		3.5000		
SGO_0698	0.402	5.779	0.0587	0.2686	9.500	8.000	14.1651	8.3257	recN; DNA repair protein RecN	
					11.500	16.000	16.4127	16.0000		
SGO_0699	0.342	4.783	0.0207	0.0805	5.500	5.000	8.2009	5.2035	Serine/threonine protein phosphatase	
					5.000	7.000	7.1360	7.0000		
SGO_0700	0.522	6.310	0.0140	0.0498	14.000	18.500	20.8749	19.2531	DegV family protein	
					18.000	13.500	25.6895	13.5000		

Show detected proteins only
Show all proteins
Filter by category:
ABC Transporter

Proteins found: 1179

Test	Cutoff
q-Value	.005
p-Value	

	Signif	Direction	Applies To
red	yes	+	ratios, bars
yellow	no	n/a	bars
green	yes	–	ratios, bars
pink	yes	+	*p*- , *q*-Values
light green	yes	–	*p*- , *q*-Values

Dot Plots Dot Plots

Protein	SgPg vs Sg Log$_2$ Ratio	Log$_2$ Sum	*q*-Value	*p*-Value	Raw *SgPg*	Raw *Sg*	Normalized *SgPg*	Normalized *Sg*	Description
SGO_0701	1.056	12.649	0.0002	0.0001	1475.000	1072.000	2199.3191	1115.6407	hup; DNA-binding histone-like protein HU
					1496.500	974.000	2135.7949	974.0000	
SGO_0704	1.791	11.120	0.0004	0.0004	559.500	193.000	834.2502	200.8570	gpmA; 2,3-bisphosphoglycerate-dependent phosphoglycerate mutase
					619.500	306.500	884.1463	306.5000	
SGO_0705		1.807							Protein of unknown function (DUF1250) superfamily
						3.500		3.5000	
SGO_0706	1.431	5.021	0.0017	0.0035	8.000	3.000	11.9285	3.1221	phoH-like protein
					8.000	6.000	11.4175	6.0000	
SGO_0707	-5.103	8.777	0.0000	0.0000	6.000	206.000	8.9464	214.3862	LPXTG cell wall surface protein
					3.000	211.000	4.2816	211.0000	
SGO_0708	0.702	9.607	0.0002	0.0001	162.000	137.000	241.5523	142.5772	ald; alanine dehydrogenase
					169.000	154.500	241.1957	154.5000	
SGO_0711		3.145				8.500		8.8460	conserved hypothetical protein TIGR00043
SGO_0713	0.586	7.177	0.0059	0.0173	32.500	29.500	48.4596	30.7009	sgg; GTP-binding protein Era
					27.000	27.000	38.5342	27.0000	
SGO_0715	-0.001	3.321							mutM; formamidopyrimidine-DNA glycosylase
					3.500	5.000	4.9952	5.0000	
SGO_0719	-2.061	5.620	0.0009	0.0013	3.500	17.000	5.2187	17.6921	rnr; ribonuclease R
					3.000	22.000	4.2816	22.0000	
SGO_0721	0.112	5.337	0.0947	0.4647	6.000	8.500	8.9464	8.8460	abpB-like dipeptidase lipoprotein
					8.500	10.500	12.1311	10.5000	
SGO_0722	0.624	6.153	0.0651	0.3027	14.000	7.500	20.8749	7.8053	tehB; tellurite resistance protein TehB
					14.000	22.500	19.9807	22.5000	

Log$_2$ Ratios: -6 -4 -2 0 2 4 6

Show detected proteins only
Show all proteins
Filter by category:
ABC Transporter

Proteins found: 1179

Test	Cutoff
***q*-Value**	.005
p-Value	

	Signif	Direction	Applies To
red	yes	+	ratios, bars
yellow	no	n/a	bars
green	yes	−	ratios, bars
pink	yes	+	*p*- , *q*-Values
light green	yes	−	*p*- , *q*-Values

Dot Plots Dot Plots

Protein	SgPg vs Sg Log2 Ratio	Log2 Sum	q-Value	p-Value	Raw SgPg	Raw Sg	Normalized SgPg	Normalized Sg	Description
SGO_0724	-2.408	6.437	0.0004	0.0003	4.000	32.500	5.9643	33.8231	dipeptidase
					5.500	39.000	7.8496	39.0000	
SGO_0728		2.000							ABC transporter, ATP-binding protein SP1653
						4.000		4.0000	
SGO_0729		3.022				3.000		3.1221	ABC transporter permease protein
						5.000		5.0000	
SGO_0731		4.120				9.500		9.8867	RRF2 family protein
						7.500		7.5000	
SGO_0736	-1.037	5.520	0.0033	0.0081	4.000	16.000	5.9643	16.6514	hprK; HPr(Ser) kinase/phosphatase
					6.500	14.000	9.2768	14.0000	
SGO_0737		4.348				9.000		9.3664	lgt; prolipoprotein diacylglyceryl transferase
						11.000		11.0000	
SGO_0738	-3.482	7.062	0.0005	0.0006	4.000	65.000	5.9643	67.6461	Bacterial protein of unknown function (DUF948) family
					3.500	55.000	4.9952	55.0000	
SGO_0739	0.984	5.964	0.0021	0.0046	12.500	11.000	18.6383	11.4478	hypothetical protein SGO_0739
					16.000	9.500	22.8351	9.5000	
SGO_0742	0.310	6.232	0.0039	0.0104	14.000	15.000	20.8749	15.6106	peptidase, U32 family
					14.500	18.000	20.6943	18.0000	
SGO_0743	-1.241	7.933	0.0005	0.0007	28.000	80.000	41.7498	83.2568	peptidase, U32 family
					22.000	88.000	31.3983	88.0000	
SGO_0745	2.842	7.443	0.0002	0.0001	48.500	8.000	72.3166	8.3257	hypothetical protein SGO_0745
					56.000	13.500	79.9228	13.5000	
SGO_0749	1.249	6.844	0.0018	0.0038	29.000	13.000	43.2408	13.5292	glutathione reductase
					26.000	21.000	37.1070	21.0000	

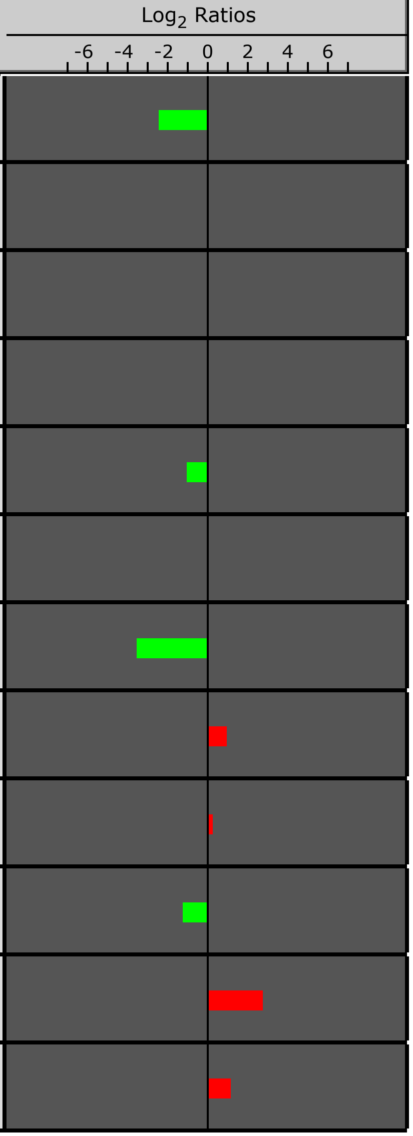

Show detected proteins only
Show all proteins
Filter by category: ABC Transporter

Proteins found: 1179

Test	Cutoff
q-Value	.005
p-Value	

Color	Signif	Direction	Applies To
red	yes	+	ratios, bars
yellow	no	n/a	bars
green	yes	−	ratios, bars
pink	yes	+	p- , q-Values
light green	yes	−	p- , q-Values

Dot Plots Dot Plots

Streptococcus gordonii

Protein	SgPg vs Sg: Log_2 Ratio	Log_2 Sum	*q*-Value	*p*-Value	Raw: *SgPg*	Raw: *Sg*	Normalized: *SgPg*	Normalized: *Sg*	Description
SGO_0750	-1.521	10.797	0.0013	0.0023	160.000	708.000	238.5702	736.8224	efflux transporter, RND family, MFP subunit subfamily
					153.500	584.500	219.0742	584.5000	
SGO_0751	0.115	8.412	0.0791	0.3822	67.500	82.500	100.6468	85.8585	ABC transporter, ATP-binding protein SP0786
					54.000	77.000	77.0684	77.0000	
SGO_0752	-1.394	7.621	0.0017	0.0034	18.000	60.000	26.8391	62.4426	ABC transporter, ATP-binding protein
					19.000	80.500	27.1167	80.5000	
SGO_0753	0.038	9.150	0.1267	0.6427	101.500	144.000	151.3430	149.8622	lysS; lysyl-tRNA synthetase
					95.500	130.500	136.2970	130.5000	
SGO_0754		1.835							phosphoglycerate mutase family protein
					2.500		3.5680		
SGO_0755	0.647	5.566	0.0123	0.0424	8.000	8.000	11.9285	8.3257	regulatory protein
					12.000	10.000	17.1263	10.0000	
SGO_0757		6.945				66.000		68.6868	peptidase, U32 family
						54.500		54.5000	
SGO_0760	0.615	9.269	0.0000	0.0000	125.500	117.000	187.1285	121.7630	ppc; phosphoenolpyruvate carboxylase
					130.500	122.000	186.2487	122.0000	
SGO_0761	0.462	13.435	0.0010	0.0017	2246.500	2223.000	3349.6748	2313.4975	tuf; translation elongation factor Tu
					2149.500	2341.000	3067.7522	2341.0000	
SGO_0762	0.868	11.183	0.0047	0.0131	581.000	384.500	866.3081	400.1528	tpiA; triosephosphate isomerase
					449.500	417.000	641.5234	417.0000	
SGO_0763	-1.037	8.173	0.0008	0.0011	29.500	87.500	43.9864	91.0621	murA-1; UDP-N-acetylglucosamine 1-carboxyvinyltransferase
					35.500	103.000	50.6654	103.0000	
SGO_0765		4.959				15.000		15.6106	endA; DNA-entry nuclease
						15.500		15.5000	

Log_2 Ratios: -6 -4 -2 0 2 4 6

Show detected proteins only
Show all proteins
Filter by category:
ABC Transporter

Proteins found: 1179

Test	Cutoff
***q*-Value**	**.005**
p-Value	

	Signif	Direction	Applies To
(red)	yes	+	ratios, bars
(yellow)	no	n/a	bars
(green)	yes	−	ratios, bars
(pink)	yes	+	*p*- , *q*-Values
(light green)	yes	−	*p*- , *q*-Values

Dot Plots Dot Plots

Protein	SgPg vs Sg Log_2 Ratio	Log_2 Sum	*q*-Value	*p*-Value	Raw SgPg	Raw Sg	Normalized SgPg	Normalized Sg	Description	Log_2 Ratios
SGO_0767	-0.180	5.377	0.0674	0.3152	5.500	11.000	8.2009	11.4478	transport protein	
					8.000	10.500	11.4175	10.5000		
SGO_0771	1.279	8.306	0.0013	0.0023	73.000	56.000	108.8477	58.2797	pepq; proline dipeptidase	
					79.500	36.000	113.4619	36.0000		
SGO_0773	-2.406	10.223	0.0002	0.0001	68.500	508.000	102.1379	528.6805	ccpA; catabolite control protein A	
					61.500	476.500	87.7724	476.5000		
SGO_0774	-1.574	7.142	0.0017	0.0036	14.500	56.500	21.6204	58.8001	glycosyl transferase, group 1 family protein	
					10.000	46.500	14.2719	46.5000		
SGO_0775	-0.719	6.433	0.0052	0.0149	9.500	28.500	14.1651	29.6602	glycosyl transferase, group 1	
					13.000	24.000	18.5535	24.0000		
SGO_0778	-0.132	9.237	0.0068	0.0204	94.000	148.000	140.1600	154.0250	thrS; threonyl-tRNA synthetase	
					103.500	161.500	147.7145	161.5000		
SGO_0779	0.258	6.808	0.0709	0.3336	23.500	19.500	35.0400	20.2938	response regulator	
					18.000	31.000	25.6895	31.0000		
SGO_0780	-2.189	5.620	0.0017	0.0034	3.000	22.500	4.4732	23.4160	histidine kinase	
					3.000	17.000	4.2816	17.0000		
SGO_0781		5.091				14.500		15.0903	vicX; Zn-dependent hydrolase (beta-lactamase superfamily)	
						19.000		19.0000		
SGO_0782		3.665				4.500		4.6832	Protein of unknown function (DUF454) family	
						8.000		8.0000		
SGO_0784	-1.759	8.327	0.0011	0.0018	24.000	105.500	35.7855	109.7949	smc; chromosome segregation protein SMC	
					26.000	138.500	37.1070	138.5000		
SGO_0785	-0.875	4.804	0.0299	0.1232	4.000	11.500	5.9643	11.9682	Cof family protein	
						10.000		10.0000		

Log_2 Ratios axis: -6 -4 -2 0 2 4 6

Show detected proteins only
Show all proteins
Filter by category:
ABC Transporter

Proteins found: 1179

Test	Cutoff
q-Value	.005
p-Value	

	Signif	Direction	Applies To
red	yes	+	ratios, bars
yellow	no	n/a	bars
green	yes	−	ratios, bars
pink	yes	+	*p*- , *q*-Values
light green	yes	−	*p*- , *q*-Values

Dot Plots | Dot Plots

Protein	SgPg vs Sg Log2 Ratio	Log2 Sum	q-Value	p-Value	Raw SgPg	Raw Sg	Normalized SgPg	Normalized Sg	Description
SGO_0786	0.800	6.061	0.0010	0.0015	14.500	10.500	21.6204	10.9275	Cof family protein
					14.500	13.500	20.6943	13.5000	
SGO_0787	-0.273	7.598	0.0142	0.0509	29.500	56.000	43.9864	58.2797	ftsY; cell division protein FtsY
					30.500	48.000	43.5294	48.0000	
SGO_0788	1.244	7.437	0.0003	0.0002	40.000	27.000	59.6426	28.0992	zwf; glucose-6-phosphate 1-dehydrogenase
					43.500	23.500	62.0829	23.5000	
SGO_0792	-0.585	7.246	0.0105	0.0350	23.000	38.000	34.2945	39.5470	hypothetical protein SGO_0792
					18.500	51.500	26.4031	51.5000	
SGO_0794	-0.049	7.789	0.1049	0.5188	35.000	50.000	52.1872	52.0355	metallo-beta-lactamase family protein
					39.500	60.500	56.3741	60.5000	
SGO_0795	1.371	5.264	0.0002	0.0001	9.000	5.500	13.4196	5.7239	tributyrin esterase
					10.000	5.000	14.2719	5.0000	
SGO_0797		3.322							RocB protein, putative
						10.000		10.0000	
SGO_0798	-1.256	7.768	0.0009	0.0013	22.500	80.000	33.5489	83.2568	ABC transporter, ATP-binding protein SP1381
					21.500	70.500	30.6847	70.5000	
SGO_0800	-1.446	6.450	0.0120	0.0409	7.000	21.000	10.4374	21.8549	polysaccharide deacetylase family protein
					8.500	43.000	12.1311	43.0000	
SGO_0801	1.139	8.955	0.0003	0.0002	111.500	80.500	166.2536	83.7771	hom; homoserine dehydrogenase
					122.500	71.500	174.8312	71.5000	
SGO_0802	0.767	6.782	0.0003	0.0002	23.000	18.500	34.2945	19.2531	thrB; homoserine kinase
					24.500	21.500	34.9662	21.5000	
SGO_0803	-1.866	7.889	0.0002	0.0001	16.500	86.000	24.6026	89.5010	hypothetical protein SGO_0803
					18.500	96.500	26.4031	96.5000	

Log2 Ratios: -6 -4 -2 0 2 4 6

Show detected proteins only
Show all proteins
Filter by category:
ABC Transporter

Proteins found: 1179

Test	Cutoff
q-Value	.005
p-Value	

	Signif	Direction	Applies To
red	yes	+	ratios, bars
yellow	no	n/a	bars
green	yes	–	ratios, bars
pink	yes	+	p- , q-Values
light green	yes	–	p- , q-Values

Dot Plots Dot Plots

Hendrickson *et al.*

Protein	*SgPg vs Sg* Log_2 Ratio	Log_2 Sum	*q*-Value	*p*-Value	Raw *SgPg*	Raw *Sg*	Normalized *SgPg*	Normalized *Sg*	Description	Log_2 Ratios
**SGO_0804**	-0.227	4.969	0.0921	0.4499	5.000	4.000	7.4553	4.1628	murB; UDP-N-acetylenolpyruvoylglucosamine reductase	
					4.000	14.000	5.7088	14.0000		
**SGO_0805**	-2.598	6.570	0.0004	0.0005	3.500	42.000	5.2187	43.7098	potA; spermidine/putrescine ABC transporter, ATP-binding subunit	
					6.000	37.500	8.5632	37.5000		
**SGO_0806**		4.271				7.500		7.8053	potB; spermidine/putrescine ABC transporter, permease protein	
						11.500		11.5000		
**SGO_0808**	-3.301	6.773	0.0501	0.2239		33.500		34.8638	potD; spermidine/putrescine ABC transporter	
					3.500	69.500	4.9952	69.5000		
**SGO_0812**	-0.235	4.916	0.0150	0.0547	4.500	8.500	6.7098	8.8460	hypothetical protein SGO_0812	
					5.000	7.500	7.1360	7.5000		
**SGO_0813**		1.807							fructose-bisphosphatase	
						3.500		3.5000		
**SGO_0814**	0.665	3.541							aminotransferase, class-V	
					5.000	4.500	7.1360	4.5000		
**SGO_0815**	-0.231	7.574	0.0182	0.0693	31.500	46.000	46.9685	47.8726	thiI; thiamine biosynthesis protein ThiI	
					28.500	55.000	40.6750	55.0000		
**SGO_0817**		3.556				6.500		6.7646	hypothetical protein SGO_0817	
						5.000		5.0000		
**SGO_0818**	2.010	9.582	0.0005	0.0006	190.500	87.000	284.0477	90.5417	rplU; ribosomal protein L21	
					230.000	63.500	328.2545	63.5000		
**SGO_0820**	0.025	7.846	0.1704	0.8971	35.000	72.500	52.1872	75.4514	rpmA; ribosomal protein L27	
					43.000	41.000	61.3693	41.0000		
**SGO_0822**		3.621				7.500		7.8053	lipoprotein, putative	
						4.500		4.5000		

Log2 Ratios axis: -6 -4 -2 0 2 4 6

Show detected proteins only
Show all proteins
Filter by category: ABC Transporter

Proteins found: 1179

Test	Cutoff
q-Value	.005
p-Value	

	Signif	Direction	Applies To
red	yes	+	ratios, bars
yellow	no	n/a	bars
green	yes	−	ratios, bars
pink	yes	+	*p*- , *q*-Values
light green	yes	−	*p*- , *q*-Values

Dot Plots Dot Plots

Protein	SgPg vs Sg Log$_2$ Ratio	Log$_2$ Sum	*q*-Value	*p*-Value	Raw *SgPg*	Raw *Sg*	Normalized *SgPg*	Normalized *Sg*	Description
SGO_0823	-0.165	2.920							Cof family protein
					2.500	4.000	3.5680	4.0000	
SGO_0824	1.254	7.781	0.0010	0.0017	51.500	25.000	76.7898	26.0177	lepA; GTP-binding protein LepA
					54.000	40.000	77.0684	40.0000	
SGO_0825	-1.794	6.419	0.0019	0.0042	6.500	27.000	9.6919	28.0992	lipoprotein, putative
					6.500	38.500	9.2768	38.5000	
SGO_0830	0.755	2.710	0.0282	0.1150	2.000	1.500	2.9821	1.5611	uvrC; excinuclease ABC, C subunit
						2.000		2.0000	
SGO_0832	-2.252	6.649	0.0010	0.0016	7.000	44.500	10.4374	46.3116	hypothetical protein SGO_0832
					5.000	36.500	7.1360	36.5000	
SGO_0833		3.120			2.000		2.9821		phosphoesterase-related gene
					4.000		5.7088		
SGO_0834		4.377				7.000		7.2850	hypothetical protein SGO_0834
						13.500		13.5000	
SGO_0835	1.275	8.064	0.0004	0.0004	63.500	32.500	94.6826	33.8231	nitroreductase
					66.000	45.000	94.1948	45.0000	
SGO_0836	0.793	9.237	0.0004	0.0004	130.000	99.000	193.8383	103.0303	pepV; dipeptidase PepV
					132.000	118.000	188.3895	118.0000	
SGO_0841		1.807							oxidoreductase
						3.500		3.5000	
SGO_0842	0.073	4.710	0.1825	0.9811	3.000	3.500	4.4732	3.6425	rhodanese family protein
					6.000	9.500	8.5632	9.5000	
SGO_0848	1.269	8.420	0.0000	0.0000	82.000	48.500	122.2672	50.4744	rpmE; ribosomal protein L31
					84.000	50.000	119.8842	50.0000	

Log$_2$ Ratios: -6 -4 -2 0 2 4 6

Show detected proteins only
Show all proteins
Filter by category:
ABC Transporter

Proteins found: 1179

Test	Cutoff
q-Value	.005
p-Value	

	Signif	Direction	Applies To
red	yes	+	ratios, bars
yellow	no	n/a	bars
green	yes	−	ratios, bars
pink	yes	+	*p*-, *q*-Values
light green	yes	−	*p*-, *q*-Values

Dot Plots Dot Plots

Protein	*SgPg* vs *Sg* Log$_2$ Ratio	Log$_2$ Sum	*q*-Value	*p*-Value	Raw *SgPg*	Raw *Sg*	Normalized *SgPg*	Normalized *Sg*	Description
SGO_0849	0.055	6.011	0.1464	0.7609	13.000	13.000	19.3838	13.5292	DHH subfamily 1 protein
					9.500	18.000	13.5583	18.0000	
SGO_0850	1.562	7.506	0.0004	0.0004	48.500	22.500	72.3166	23.4160	flavodoxin
					44.500	22.500	63.5101	22.5000	
SGO_0851	0.281	4.984	0.1267	0.6444	5.000	10.000	7.4553	10.4071	putative permease
					6.500	4.500	9.2768	4.5000	
SGO_0852	-0.862	4.813	0.0092	0.0300	4.500	8.000	6.7098	8.3257	TPR domain protein
					2.500	9.500	3.5680	9.5000	
SGO_0853	-0.638	4.965	0.0412	0.1775	4.500	13.000	6.7098	13.5292	budA; alpha-acetolactate decarboxylase
					3.500	6.000	4.9952	6.0000	
SGO_0854	-5.332	9.527	0.0001	0.0000	5.000	336.500	7.4553	350.1988	cshA; surface-associated protein CshA
					7.500	369.500	10.7040	369.5000	
SGO_0855	-0.453	5.699	0.0089	0.0287	8.000	13.000	11.9285	13.5292	fbpA; fibronectin-binding protein A
					7.000	16.500	9.9904	16.5000	
SGO_0856	-1.459	8.517	0.0003	0.0003	29.000	124.500	43.2408	129.5683	ABC transporter, substrate binding protein
					38.500	138.500	54.9469	138.5000	
SGO_0858		3.727				6.000		6.2443	ABC transporter, ATP-binding protein
						7.000		7.0000	
SGO_0859	-0.216	7.523	0.0440	0.1920	30.000	54.500	44.7319	56.7187	pheS; phenylalanyl-tRNA synthetase, alpha subunit
					28.000	42.500	39.9614	42.5000	
SGO_0861	0.743	8.986	0.0008	0.0012	103.500	99.000	154.3251	103.0303	pheT; phenylalanyl-tRNA synthetase, beta subunit
					114.000	87.000	162.7000	87.0000	
SGO_0862		3.475				3.000		3.1221	hypothetical protein SGO_0862
						8.000		8.0000	

Log$_2$ Ratios: -6 -4 -2 0 2 4 6

Show detected proteins only
Show all proteins
Filter by category:
ABC Transporter

Proteins found: 1179

Test	Cutoff
***q*-Value**	.005
p-Value	

	Signif	Direction	Applies To
red	yes	+	ratios, bars
yellow	no	n/a	bars
green	yes	−	ratios, bars
pink	yes	+	*p*- , *q*-Values
light green	yes	−	*p*- , *q*-Values

Dot Plots Dot Plots

Streptococcus gordonii

Hackett Laboratory UW

Protein	SgPg vs Sg Log2 Ratio	Log2 Sum	*q*-Value	*p*-Value	Raw *SgPg*	Raw *Sg*	Normalized *SgPg*	Normalized *Sg*	Description
SGO_0868		1.058				2.000		2.0814	hypothetical protein SGO_0868
SGO_0877		1.585							permease domain protein
						3.000		3.0000	
SGO_0885	-0.639	5.374	0.0115	0.0390	5.500	10.000	8.2009	10.4071	cobyric acid synthase
					5.500	15.000	7.8496	15.0000	
SGO_0886	-0.777	4.851	0.0434	0.1880	2.500	5.000	3.7277	5.2035	Mur ligase family protein
					4.500	13.500	6.4224	13.5000	
SGO_0887		7.303				84.000		87.4196	conserved hypothetical protein TIGR00159
						70.500		70.5000	
SGO_0888	-1.894	5.554	0.0025	0.0055	2.500	20.500	3.7277	21.3345	hypothetical protein SGO_0888
					4.500	15.500	6.4224	15.5000	
SGO_0889	0.013	7.195	0.1403	0.7211	24.000	36.000	35.7855	37.4655	glmM; phosphoglucosamine mutase
					26.500	35.500	37.8206	35.5000	
SGO_0890		8.059				126.000		131.1294	LPXTG cell wall surface protein, collagen binding domain
						135.500		135.5000	
SGO_0893	0.666	7.769	0.0040	0.0106	40.500	44.000	60.3881	45.7912	GTP-binding protein
					51.500	38.500	73.5005	38.5000	
SGO_0899		3.334				2.000		2.0814	putative hydrolase or acyltransferase
						8.000		8.0000	
SGO_0901	0.470	6.395	0.0052	0.0150	18.000	17.000	26.8391	17.6921	DNA-directed DNA polymerase III
					15.500	17.500	22.1215	17.5000	
SGO_0904		1.058				2.000		2.0814	purR; galactose operon repressor GalR

Log2 Ratios: -6 -4 -2 0 2 4 6

◉ Show detected proteins only
○ Show all proteins
☐ Filter by category:
ABC Transporter

Proteins found: 1179

Test	Cutoff
***q*-Value**	**.005**
p-Value	

Color	Signif	Direction	Applies To
red	yes	+	ratios, bars
yellow	no	n/a	bars
green	yes	–	ratios, bars
pink	yes	+	*p*- , *q*-Values
light green	yes	–	*p*- , *q*-Values

Dot Plots | Dot Plots

Hendrickson *et al.*

Protein	SgPg vs Sg: Log_2 Ratio	SgPg vs Sg: Log_2 Sum	SgPg vs Sg: *q*-Value	SgPg vs Sg: *p*-Value	Raw: *SgPg*	Raw: *Sg*	Normalized: *SgPg*	Normalized: *Sg*	Description
SGO_0905		4.857				12.000		12.4885	lipoprotein, putative
						16.500		16.5000	
SGO_0906	1.999	6.978	0.0001	0.0000	34.000	10.500	50.6962	10.9275	leuA; 2-isopropylmalate synthase
					35.000	14.500	49.9518	14.5000	
SGO_0907	0.546	3.597	0.0772	0.3701		2.500		2.6018	leuB; 3-isopropylmalate dehydrogenase
					3.500	4.500	4.9952	4.5000	
SGO_0909		2.683							leuC; 3-isopropylmalate dehydratase, large subunit
					4.500		6.4224		
SGO_0911	1.860	8.180	0.0001	0.0000	75.000	29.000	111.8298	30.1806	hypothetical protein SGO_0911
					81.000	32.500	115.6027	32.5000	
SGO_0915	-1.047	5.481	0.0012	0.0021	5.500	13.500	8.2009	14.0496	proV; ABC-type proline/glycine betaine transporter
					4.500	16.000	6.4224	16.0000	
SGO_0916		5.255				17.000		17.6921	proWX; ABC transporter membrane-spanning permease
						20.500		20.5000	
SGO_0932		2.845				4.500		4.6832	galK; galactokinase
						2.500		2.5000	
SGO_0934		2.459							galE-2; UDP-glucose 4-epimerase
						5.500		5.5000	
SGO_0936		2.036				2.500		2.6018	phosphoenolpyruvate synthase, putative
						1.500		1.5000	
SGO_0938									Phosphatidylserine decarboxylase proenzyme 2
SGO_0940		3.894				9.000		9.3664	ppsA; phosphoenolpyruvate synthase
						5.500		5.5000	

Log_2 Ratios: -6 -4 -2 0 2 4 6

Show detected proteins only
Show all proteins
Filter by category:
ABC Transporter

Proteins found: 1179

Test	Cutoff
q-Value	.005
p-Value	

	Signif	Direction	Applies To
red	yes	+	ratios, bars
yellow	no	n/a	bars
green	yes	−	ratios, bars
pink	yes	+	*p*- , *q*-Values
light green	yes	−	*p*- , *q*-Values

Dot Plots Dot Plots

Protein	*SgPg vs Sg* Log$_2$ Ratio	Log$_2$ Sum	*q*-Value	*p*-Value	Raw *SgPg*	Raw *Sg*	Normalized *SgPg*	Normalized *Sg*	Description	Log$_2$ Ratios (-6 -4 -2 0 2 4 6)
**SGO_0941**		1.322							hypothetical protein SGO_0941	
						2.500		2.5000		
**SGO_0942**		5.201				19.500		20.2938	zmpC; zinc metalloproteinase C	
						16.500		16.5000		
**SGO_0944**		5.012				6.500		6.7646	ABC transporter, ATP-binding protein	
						25.500		25.5000		
**SGO_0946**	-0.217	6.786	0.0285	0.1162	19.000	30.000	28.3302	31.2213	Deblocking aminopeptidase	
					16.000	28.000	22.8351	28.0000		
**SGO_0948**		5.141				19.000		19.7735	pgdA; peptidoglycan N-acetylglucosamine deacetylase A	
						15.500		15.5000		
**SGO_0949**	-0.476	5.268	0.0151	0.0552	5.000	12.500	7.4553	13.0089	deaD; DEAD RNA helicase	
					6.000	9.500	8.5632	9.5000		
**SGO_0950**	-0.275	4.609	0.1251	0.6319	6.500		9.6919		oxidoreductase	
					4.000	9.000	5.7088	9.0000		
**SGO_0951**	-0.138	5.905	0.0714	0.3362	9.000	13.000	13.4196	13.5292	udk; uridine kinase	
					10.500	18.000	14.9855	18.0000		
**SGO_0953**		5.155			10.500		15.6562		endoribonuclease L-PSP, putative	
					14.000		19.9807			
**SGO_0954**	0.095	6.263	0.1169	0.5847	9.500	18.000	14.1651	18.7328	ATP-binding protein	
					18.500	17.500	26.4031	17.5000		
**SGO_0955**	0.411	4.555	0.0759	0.3630		5.500		5.7239	transporter	
					6.500	8.500	9.2768	8.5000		
**SGO_0957**		5.518				20.500		21.3345	hypothetical protein SGO_0957	
						24.500		24.5000		

Show detected proteins only
Show all proteins
Filter by category:
ABC Transporter

Proteins found: 1179

Test	Cutoff
***q*-Value**	**.005**
p-Value	

	Signif	Direction	Applies To
(red)	yes	+	ratios, bars
(yellow)	no	n/a	bars
(green)	yes	−	ratios, bars
(pink)	yes	+	*p*- , *q*-Values
(light green)	yes	−	

Dot Plots Dot Plots

Protein	SgPg vs Sg Log$_2$ Ratio	Log$_2$ Sum	q-Value	p-Value	Raw SgPg	Raw Sg	Normalized SgPg	Normalized Sg	Description
SGO_0959	0.527	4.866	0.0492	0.2185	6.500	8.000	9.6919	8.3257	copper homeostasis protein CutC
					5.000	4.000	7.1360	4.0000	
SGO_0961		3.776				5.000		5.2035	ABC transporter, permease/ATP-binding protein
						8.500		8.5000	
SGO_0962		1.841				2.000		2.0814	ABC transporter, permease/ATP-binding protein
						1.500		1.5000	
SGO_0966		6.093				31.000		32.2620	hsa; streptococcal hemagglutinin
						36.000		36.0000	
SGO_0969		2.934				3.500		3.6425	nss; nucleotide sugar synthetase-like protein
						4.000		4.0000	
SGO_0971		2.609				2.500		2.6018	asp1; accessory secretory protein
						3.500		3.5000	
SGO_0972		1.585							asp2; accessory sescretory protein
						3.000		3.0000	
SGO_0975		3.272				4.000		4.1628	gtaA; glycosyl transferase, group 1 SP1758
						5.500		5.5000	
SGO_0979		3.279				5.000		5.2035	degV family protein
						4.500		4.5000	
SGO_0980		0.643				1.500		1.5611	transcriptional regulator, TetR family domain protein
SGO_0981	-0.793	4.371	0.0772	0.3697	3.000	5.500	4.4732	5.7239	comE operon protein 2 family
						10.500		10.5000	
SGO_0982	-2.052	8.392	0.0004	0.0003	19.500	121.500	29.0757	126.4462	amino acid ABC transporter, amino acid-binding protein
					25.500	144.000	36.3934	144.0000	

Log$_2$ Ratios: -6 -4 -2 0 2 4 6

Show detected proteins only
Show all proteins
Filter by category:
ABC Transporter

Proteins found: 1179

Test	Cutoff
q-Value	.005
p-Value	

	Signif	Direction	Applies To
red	yes	+	ratios, bars
yellow	no	n/a	bars
green	yes	−	ratios, bars
pink	yes	+	p- , q-Values
light green	yes	−	p- , q-Values

Dot Plots Dot Plots

Protein	SgPg vs Sg Log_2 Ratio	Log_2 Sum	*q*-Value	*p*-Value	Raw *SgPg*	Raw *Sg*	Normalized *SgPg*	Normalized *Sg*	Description
SGO_0983	-3.281	5.868	0.0096	0.0314		25.500		26.5381	amino acid ABC transporter, ATP-binding protein SP0709
					2.000	29.000	2.8544	29.0000	
SGO_0986		3.992				10.000		10.4071	hypothetical protein SGO_0986
						5.500		5.5000	
SGO_0987	0.274	7.714	0.0366	0.1563	32.500	48.000	48.4596	49.9541	metK; S-adenosylmethionine synthetase
					47.000	44.500	67.0781	44.5000	
SGO_0991	1.525	8.820				112.000		116.5595	hypothetical protein SGO_0991
					235.000		335.3905		
SGO_0993		2.098							GTP-binding protein HflX
					3.000		4.2816		
SGO_0994	0.996	4.243	0.0136	0.0479	3.500	2.000	5.2187	2.0814	hypothetical protein SGO_0994
					5.000	4.500	7.1360	4.5000	
SGO_0995	0.171	5.395	0.1330	0.6801	11.500	5.500	17.1472	5.7239	metallo-beta-lactamase superfamily protein
					4.000	13.500	5.7088	13.5000	
SGO_0996		3.351				5.000		5.2035	oxidoreductase, short-chain dehydrogenase/reductase family
						5.000		5.0000	
SGO_0997	-1.025	5.451	0.0317	0.1312	8.500	12.000	12.6740	12.4885	hypothetical protein SGO_0997
					2.500	15.000	3.5680	15.0000	
SGO_1000	1.958	4.529	0.0329	0.1379	8.000	2.500	11.9285	2.6018	recJ; single-stranded-DNA-specific exonuclease RecJ
					6.000		8.5632		
SGO_1001	1.111	7.883	0.0025	0.0058	48.500	42.500	72.3166	44.2302	apt; adenine phosphoribosyltransferase
					62.000	31.000	88.4860	31.0000	
SGO_1002		1.000							metA; homoserine O-succinyltransferase
						2.000		2.0000	

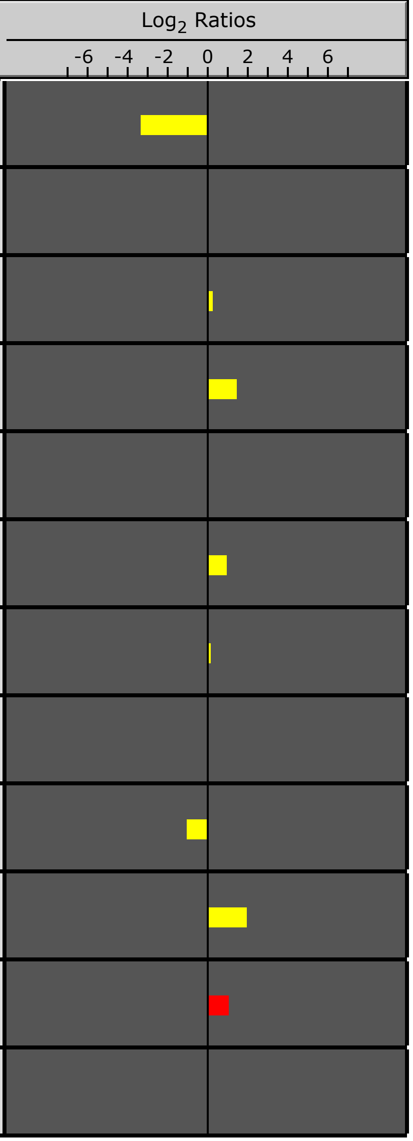

Show detected proteins only
Show all proteins
Filter by category:
ABC Transporter

Proteins found: 1179

Test	Cutoff
q-Value	.005
p-Value	

	Signif	Direction	Applies To
red	yes	+	ratios, bars
yellow	no	n/a	bars
green	yes	–	ratios, bars
pink	yes	+	*p*- , *q*-Values
light green	yes	–	

Dot Plots Dot Plots

Protein	*SgPg vs Sg* Log_2 Ratio	Log_2 Sum	*q*-Value	*p*-Value	Raw *SgPg*	Raw *Sg*	Normalized *SgPg*	Normalized *Sg*	Description
**SGO_1003**	0.078	5.464	0.1208	0.6096	8.500	11.500	12.6740	11.9682	DNA replication protein DnaD
					7.000	9.500	9.9904	9.5000	
**SGO_1004**	1.083	4.433	0.0635	0.2929	7.500		11.1830		glutathione S-transferase family protein
					4.500	4.000	6.4224	4.0000	
**SGO_1005**	0.994	5.306	0.0039	0.0102	10.000	5.500	14.9106	5.7239	Bcl-2 family protein
					8.000	7.500	11.4175	7.5000	
**SGO_1006**	0.787	3.497	0.0576	0.2617	3.500		5.2187		conserved hypothetical protein TIGR00486
					2.500	2.500	3.5680	2.5000	
**SGO_1007**		2.854			1.500		2.2366		oxidoreductase, DadA family protein SP1608
					3.500		4.9952		
**SGO_1009**	1.501	8.934	0.0003	0.0002	126.500	57.000	188.6196	59.3204	rfbA-1; glucose-1-phosphate thymidylyltransferase
					121.000	68.500	172.6904	68.5000	
**SGO_1010**	0.919	7.414	0.0033	0.0084	42.500	28.000	63.3702	29.1399	rmlC; dTDP-4-keto-6-deoxyglucose-3,5-epimerase
					34.000	29.500	48.5246	29.5000	
**SGO_1011**	1.150	8.223	0.0022	0.0049	73.000	35.000	108.8477	36.4248	rfbB-1; dTDP-glucose 4,6-dehydratase
					67.000	58.000	95.6220	58.0000	
**SGO_1012**	0.993	8.779	0.0001	0.0000	98.500	72.000	146.8698	74.9311	galE-1; UDP-glucose 4-epimerase
					102.000	72.000	145.5737	72.0000	
**SGO_1013**	-2.901	6.102	0.0214	0.0839		34.500		35.9045	Glycosyltransferase involved in cell wall biogenesis
					3.000	28.500	4.2816	28.5000	
**SGO_1016**	-1.298	5.936	0.0047	0.0130	6.500	17.000	9.6919	17.6921	putative glycosyltransferase
					5.500	26.000	7.8496	26.0000	
**SGO_1018**		3.669				5.500		5.7239	hypothetical protein SGO_1018
						7.000		7.0000	

Log_2 Ratios: -6 -4 -2 0 2 4 6

Show detected proteins only
Show all proteins
Filter by category:
ABC Transporter

Proteins found: 1179

Test	Cutoff
***q*-Value**	**.005**
p-Value	

	Signif	Direction	Applies To
red	yes	+	ratios, bars
yellow	no	n/a	bars
green	yes	–	ratios, bars
pink	yes	+	*p*- , *q*-Values
light green	yes	–	*p*- , *q*-Values

Dot Plots Dot Plots

Protein	SgPg vs Sg Log2 Ratio	Log2 Sum	q-Value	p-Value	Raw SgPg	Raw Sg	Normalized SgPg	Normalized Sg	Description
SGO_1019	-2.504	6.579	0.0001	0.0000	4.000	39.500	5.9643	41.1080	glycosyl transferase
					6.000	40.000	8.5632	40.0000	
SGO_1020	0.660	7.639	0.0101	0.0334	34.000	37.500	50.6962	39.0266	rfbD; dTDP-4-dehydrorhamnose reductase
					50.500	37.500	72.0733	37.5000	
SGO_1021		5.625				21.000		21.8549	rgpA; rhamnosyltransferase, putative
						27.500		27.5000	
SGO_1022		5.593				18.500		19.2531	rhamnosyltransferase
						29.000		29.0000	
SGO_1024	-0.897	6.994	0.0133	0.0467	18.500	31.500	27.5847	32.7824	putative polysaccharide ABC transporter
					12.000	50.000	17.1263	50.0000	
SGO_1025	-0.018	6.700	0.1714	0.9072	15.000	21.500	22.3660	22.3753	rgp; glycosyltransferase
					20.500	30.000	29.2575	30.0000	
SGO_1026	-2.178	8.025	0.0046	0.0126	15.000	77.000	22.3660	80.1346	rhamnosyltransferase
					16.500	134.500	23.5487	134.5000	
SGO_1027		5.739				22.500		23.4160	hypothetical protein SGO_1027
						30.000		30.0000	
SGO_1028		4.110				6.500		6.7646	hypothetical protein SGO_1028
						10.500		10.5000	
SGO_1030		4.129							hypothetical protein SGO_1030
						17.500		17.5000	
SGO_1031	-0.051	5.735	0.1206	0.6069	8.000	13.500	11.9285	14.0496	cmk; cytidylate kinase
					10.000	13.000	14.2719	13.0000	
SGO_1032	-0.408	5.564	0.0853	0.4151	10.000	12.000	14.9106	12.4885	infC; translation initiation factor IF-3
					4.500	13.500	6.4224	13.5000	

Log2 Ratios: -6 -4 -2 0 2 4 6

Show detected proteins only
Show all proteins
Filter by category: ABC Transporter

Proteins found: 1179

Test	Cutoff
q-Value	.005
p-Value	

Colour	Signif	Direction	Applies To
red	yes	+	ratios, bars
yellow	no	n/a	bars
green	yes	–	ratios, bars
pink	yes	+	p- , q-Values
light green	yes	–	p- , q-Values

Dot Plots Dot Plots

Hendrickson *et al.*

Protein	SgPg vs Sg: Log_2 Ratio	Log_2 Sum	*q*-Value	*p*-Value	Raw: *SgPg*	Raw: *Sg*	Normalized: *SgPg*	Normalized: *Sg*	Description
SGO_1033		6.858				36.000		37.4655	rpmI; ribosomal protein L35
						78.500		78.5000	
SGO_1034	1.019	9.964	0.0000	0.0000	222.000	158.000	331.0162	164.4321	rplT; ribosomal protein L20
					236.500	165.500	337.5312	165.5000	
SGO_1035	0.688	5.690	0.0127	0.0439	10.500	7.000	15.6562	7.2850	gloA; lactoylglutathione lyase
					11.000	13.000	15.6991	13.0000	
SGO_1036	-1.212	6.966	0.0083	0.0259	8.500	34.500	12.6740	35.9045	amino acid ABC transporter, ATP-binding protein SP1242
					18.500	50.000	26.4031	50.0000	
SGO_1037	-0.955	6.229	0.0002	0.0001	9.000	23.500	13.4196	24.4567	glutamine ABC transporter permease and substrate binding protein
					8.500	25.000	12.1311	25.0000	
SGO_1038	0.889	5.145	0.0229	0.0908	9.500	4.000	14.1651	4.1628	uvrB; excinuclease ABC, B subunit
					6.000	8.500	8.5632	8.5000	
SGO_1039		3.272				4.000		4.1628	hypothetical protein SGO_1039
						5.500		5.5000	
SGO_1041	0.719	4.572	0.0490	0.2172	7.000		10.4374		hypothetical protein SGO_1041
					5.500	5.500	7.8496	5.5000	
SGO_1044	2.045	3.796	0.0095	0.0310	4.000		5.9643		MutT/nudix family protein
					4.500	1.500	6.4224	1.5000	
SGO_1047	0.514	6.503	0.0197	0.0765	15.500	14.500	23.1115	15.0903	hypothetical protein SGO_1047
					21.000	22.500	29.9711	22.5000	
SGO_1049	2.154	5.188	0.0027	0.0065	9.000	1.500	13.4196	1.5611	tRNA pseudouridine synthase B
					10.500	6.500	14.9855	6.5000	
SGO_1050	1.530	4.146	0.0605	0.2783	3.500		5.2187		ribF; riboflavin biosynthesis protein RibF
					7.000	2.500	9.9904	2.5000	

Log_2 Ratios: -6 -4 -2 0 2 4 6

Show detected proteins only
Show all proteins
Filter by category:
ABC Transporter

Proteins found: 1179

Test	Cutoff
***q*-Value**	**.005**
p-Value	

Colour	Signif	Direction	Applies To
red	yes	+	ratios, bars
yellow	no	n/a	bars
green	yes	−	ratios, bars
pink	yes	+	*p*- , *q*-Values
light green	yes	−	*p*- , *q*-Values

Dot Plots Dot Plots

Protein	SgPg vs Sg Log_2 Ratio	SgPg vs Sg Log_2 Sum	SgPg vs Sg *q*-Value	SgPg vs Sg *p*-Value	Raw *SgPg*	Raw *Sg*	Normalized *SgPg*	Normalized *Sg*	Description
SGO_1051		3.576			8.000		11.9285		negative regulator of proteolysis
SGO_1054	0.144	3.662	0.1254	0.6353	3.000	4.500	4.4732	4.6832	NOL1/NOP2/sun family protein
						3.500		3.5000	
SGO_1055	-1.995	6.887	0.0009	0.0014	10.000	41.000	14.9106	42.6691	phosphate ABC transporter, phosphate-binding protein
					6.500	51.500	9.2768	51.5000	
SGO_1057		2.585							pstA; phosphate ABC transporter, permease protein PstA
						6.000		6.0000	
SGO_1058	-1.604	6.514	0.0002	0.0001	7.500	31.500	11.1830	32.7824	pstB; Phosphate import ATP-binding protein pstB 2 (Phosphate-transporting ATPase 2) (ABC phosphate transporter 2)
					8.000	36.000	11.4175	36.0000	
SGO_1059	0.545	6.866	0.0004	0.0004	22.500	23.000	33.5489	23.9363	pstB; Phosphate import ATP-binding protein pstB 1 (Phosphate-transporting ATPase 1) (ABC phosphate transporter 1)
					25.000	23.500	35.6798	23.5000	
SGO_1060	-0.234	7.789	0.0257	0.1035	36.500	52.000	54.4238	54.1169	phosphate transport system regulatory protein
					33.000	65.500	47.0974	65.5000	
SGO_1065	-0.937	6.481	0.0002	0.0001	10.500	29.000	15.6562	30.1806	hypothetical protein SGO_1065
					10.500	28.500	14.9855	28.5000	
SGO_1066	-1.426	6.237	0.0027	0.0065	8.000	22.500	11.9285	23.4160	hypothetical protein SGO_1066
					6.000	31.500	8.5632	31.5000	
SGO_1067		1.807							hypothetical protein SGO_1067
						3.500		3.5000	
SGO_1069	0.674	8.712	0.0038	0.0100	95.000	84.000	141.6511	87.4196	membrane alanyl aminopeptidase
					81.500	74.000	116.3163	74.0000	
SGO_1072	1.254	3.081			4.000		5.9643		ciaR; Transcriptional regulatory protein CiaR
						2.500		2.5000	

Log_2 Ratios: -6 -4 -2 0 2 4 6

Show detected proteins only
Show all proteins
Filter by category: ABC Transporter

Proteins found: 1179

Test	Cutoff
q-Value	.005
p-Value	

Color	Signif	Direction	Applies To
red	yes	+	ratios, bars
yellow	no	n/a	bars
green	yes	–	ratios, bars
pink	yes	+	*p*- , *q*-Values
light green	yes	–	*p*- , *q*-Values

Dot Plots | Dot Plots

Protein	SgPg vs Sg: Log_2 Ratio	Log_2 Sum	*q*-Value	*p*-Value	Raw: *SgPg*	Raw: *Sg*	Normalized: *SgPg*	Normalized: *Sg*	Description	Log_2 Ratios
SGO_1073	-2.149	5.203	0.0133	0.0468	2.500	15.000	3.7277	15.6106	Sensor protein CiaH	
						17.500		17.5000		
SGO_1075	0.098	3.050							alpha-amylase precursor	
					3.000	4.000	4.2816	4.0000		
SGO_1077	-0.543	5.427	0.0040	0.0106	6.500	11.500	9.6919	11.9682	coaA; pantothenate kinase	
					5.500	13.500	7.8496	13.5000		
SGO_1078		4.180			4.500		6.7098		methyltransferase domain protein	
					8.000		11.4175			
SGO_1079	-0.010	7.139	0.1705	0.8989	20.500	27.500	30.5668	28.6195	pdp; pyrimidine-nucleoside phosphorylase	
					27.500	42.500	39.2478	42.5000		
SGO_1080	0.227	8.409	0.0119	0.0406	64.000	80.500	95.4281	83.7771	deoC; deoxyribose-phosphate aldolase	
					61.500	73.000	87.7724	73.0000		
SGO_1081	-0.569	4.373	0.0588	0.2694	3.500	3.500	5.2187	3.6425	cdd; cytidine deaminase	
					2.000	9.000	2.8544	9.0000		
SGO_1082	-0.805	13.082	0.0002	0.0001	1026.000	2606.500	1529.8314	2712.6096	lipoprotein	
					1141.000	2801.500	1628.4277	2801.5000		
SGO_1083		8.625			149.500		222.9140		sugar ABC transporter, ATP-binding protein SP0846	
					120.500		171.9768			
SGO_1088	0.884	4.195	0.0275	0.1112	5.000	2.000	7.4553	2.0814	transcription regulator, LysR family	
					3.000	4.500	4.2816	4.5000		
SGO_1090	-0.245	5.633	0.0727	0.3447	9.000	10.500	13.4196	10.9275	pseudouridine synthase, RluA family	
					6.500	16.000	9.2768	16.0000		
SGO_1091		2.022				1.500		1.5611	hypothetical protein SGO_1091	
						2.500		2.5000		

Show detected proteins only
Show all proteins
Filter by category:
ABC Transporter

Proteins found: 1179

Test	Cutoff
***q*-Value**	**.005**
p-Value	

	Signif	Direction	Applies To
red	yes	+	ratios, bars
yellow	no	n/a	bars
green	yes	−	ratios, bars
pink	yes	+	*p*- , *q*-Values
light green	yes	−	*p*- , *q*-Values

Dot Plots Dot Plots

Protein	SgPg vs Sg Log_2 Ratio	Log_2 Sum	*q*-Value	*p*-Value	Raw *SgPg*	Raw *Sg*	Normalized *SgPg*	Normalized *Sg*	Description
SGO_1096	3.057	10.001	0.0001	0.0000	295.000	49.500	439.8638	51.5151	butA; acetoin dehydrogenase
					332.500	58.500	474.5418	58.5000	
SGO_1097		1.000							proB; glutamate 5-kinase
						2.000		2.0000	
SGO_1098	2.384	5.365	0.0434	0.1888	9.500		14.1651		proA; gamma-glutamyl phosphate reductase
					16.500	3.500	23.5487	3.5000	
SGO_1099		1.322							proC; pyrroline-5-carboxylate reductase
						2.500		2.5000	
SGO_1100		3.807							phnA; alkylphosphonate utilization operon protein PhnA
						14.000		14.0000	
SGO_1103		3.944				9.500		9.8867	carA; carbamoyl-phosphate synthase, small subunit
						5.500		5.5000	
SGO_1104		4.277				9.500		9.8867	carB; carbamoyl-phosphate synthase, large subunit
						9.500		9.5000	
SGO_1107	-0.775	5.151	0.0690	0.3237		16.500		17.1717	PyrR bifunctional protein
					5.500	10.500	7.8496	10.5000	
SGO_1109	-0.059	8.205	0.0928	0.4547	47.000	66.500	70.0800	69.2072	pyrB; aspartate carbamoyltransferase
					52.000	81.500	74.2141	81.5000	
SGO_1110		6.147				33.000		34.3434	surface antigen SCP-like domain
						36.500		36.5000	
SGO_1111	-2.353	7.016	0.0010	0.0016	6.500	45.500	9.6919	47.3523	fruR; phosphotransferase system repressor
					8.000	61.000	11.4175	61.0000	
SGO_1112	-1.735	7.327	0.0004	0.0004	10.000	58.000	14.9106	60.3612	fruB; 1-phosphofructokinase
					16.000	62.500	22.8351	62.5000	

Log_2 Ratios: -6 -4 -2 0 2 4 6

Show detected proteins only
Show all proteins
Filter by category:
ABC Transporter

Proteins found: 1179

Test	Cutoff
q-Value	.005
p-Value	

	Signif	Direction	Applies To
red	yes	+	ratios, bars
yellow	no	n/a	bars
green	yes	−	ratios, bars
pink	yes	+	*p*- , *q*-Values
light green	yes	−	*p*- , *q*-Values

Dot Plots Dot Plots

Hendrickson *et al.*

Protein	SgPg vs Sg Log$_2$ Ratio	Log$_2$ Sum	*q*-Value	*p*-Value	Raw *SgPg*	Raw *Sg*	Normalized *SgPg*	Normalized *Sg*	Description
SGO_1113	-3.955	11.208	0.0000	0.0000	58.000	1053.000	86.4817	1095.8672	fruA; PTS system, fructose specific IIABC components
					41.500	1124.000	59.2285	1124.0000	
SGO_1114	-1.137	7.080	0.0005	0.0005	15.500	46.500	23.1115	48.3930	Protein of unknown function (DUF1149) superfamily
					13.500	44.500	19.2671	44.5000	
SGO_1115	1.302	4.389	0.0357	0.1516	5.000		7.4553		DegV family protein
					7.000	3.500	9.9904	3.5000	
SGO_1116	2.860	6.972	0.0002	0.0001	39.000	6.500	58.1515	6.7646	dapB; dihydrodipicolinate reductase
					36.500	8.500	52.0926	8.5000	
SGO_1117		3.130			3.000		4.4732		pcnA; polynucleotide adenylyltransferase
					3.000		4.2816		
SGO_1118	-0.081	3.858	0.1344	0.6881	3.500		5.2187		ABC transporter, ATP-binding protein SP1553
					3.000	5.000	4.2816	5.0000	
SGO_1119		2.036				2.500		2.6018	Abi-alpha protein, putative
						1.500		1.5000	
SGO_1120	1.050	8.420	0.0004	0.0005	78.000	48.000	116.3030	49.9541	guaA; GMP synthase
					80.000	62.000	114.1755	62.0000	
SGO_1121	-0.972	3.402							transcription regulator, GntR family
					2.500	7.000	3.5680	7.0000	
SGO_1123	1.244	7.970	0.0002	0.0001	57.000	35.000	84.9906	36.4248	ffh; signal recognition particle protein
					64.000	38.000	91.3404	38.0000	
SGO_1124		3.025				3.500		3.6425	cell surface hydrolase
						4.500		4.5000	
SGO_1128		2.000							bacteriocin-associated integral membrane protein subfamily
						4.000		4.0000	

Log$_2$ Ratios: -6 -4 -2 0 2 4 6

Show detected proteins only
Show all proteins
Filter by category:
ABC Transporter

Proteins found: 1179

Test	Cutoff
q-Value	.005
p-Value	

Colour	Signif	Direction	Applies To
red	yes	+	ratios, bars
yellow	no	n/a	bars
green	yes	−	ratios, bars
pink	yes	+	*p*- , *q*-Values
light green	yes	−	*p*- , *q*-Values

Dot Plots Dot Plots

Protein	SgPg vs Sg Log_2 Ratio	Log_2 Sum	*q*-Value	*p*-Value	Raw *SgPg*	Raw *Sg*	Normalized *SgPg*	Normalized *Sg*	Description
SGO_1129	-0.806	6.457	0.0025	0.0058	12.500	28.000	18.6383	29.1399	lplA; lipoate protein ligase A
					9.500	26.500	13.5583	26.5000	
SGO_1130	-0.666	8.509	0.0002	0.0001	48.500	109.000	72.3166	113.4373	dihydrolipoamide dehydrogenase
					48.000	110.000	68.5053	110.0000	
SGO_1131	-0.208	6.974	0.0108	0.0364	21.000	32.000	31.3123	33.3027	sucB; dihydrolipoamide S-acetyltransferase
					19.000	34.000	27.1167	34.0000	
SGO_1132	-0.631	6.047	0.0181	0.0688	8.500	15.000	12.6740	15.6106	acetoin dehydrogenase
					9.000	25.000	12.8447	25.0000	
SGO_1133	-0.813	6.658	0.0048	0.0135	13.500	35.000	20.1294	36.4248	acoA; acetoin dehydrogenase
					11.500	28.000	16.4127	28.0000	
SGO_1134	-0.566	3.898	0.0626	0.2887	2.500	4.500	3.7277	4.6832	hypothetical protein SGO_1134
						6.500		6.5000	
SGO_1139	1.267	6.969	0.0006	0.0007	29.000	15.000	43.2408	15.6106	GTP-binding protein
					31.500	21.500	44.9566	21.5000	
SGO_1140	0.482	8.625	0.0017	0.0033	77.500	85.500	115.5574	88.9807	clpX; ATP-dependent Clp protease, ATP-binding subunit ClpX
					80.000	76.000	114.1755	76.0000	
SGO_1141	0.710	4.941	0.0144	0.0518	7.500	4.500	11.1830	4.6832	folA; dihydrofolate reductase
					5.500	7.000	7.8496	7.0000	
SGO_1143	1.268	5.705	0.0018	0.0037	12.000	5.500	17.8928	5.7239	thyA; thymidylate synthase
					13.000	10.000	18.5535	10.0000	
SGO_1144	1.912	9.437	0.0000	0.0000	184.000	66.500	274.3557	69.2072	glcK; glucokinase
					191.500	76.500	273.3075	76.5000	
SGO_1145		3.781				6.000		6.2443	cell surface hydrolase
						7.500		7.5000	

Log2 Ratios: -6 -4 -2 0 2 4 6

Hendrickson *et al.*

Protein	SgPg vs Sg: Log_2 Ratio	Log_2 Sum	*q*-Value	*p*-Value	Raw: *SgPg*	Raw: *Sg*	Normalized: *SgPg*	Normalized: *Sg*	Description
**SGO_1148**		8.314				164.000		170.6764	cshB; surface-associated protein CshB
						147.500		147.5000	
**SGO_1149**		3.830				5.500		5.7239	Pneumococcal vaccine antigen A-like protein
						8.500		8.5000	
**SGO_1150**	1.321	5.394	0.0177	0.0671	12.500		18.6383		hypothetical protein SGO_1150
					11.500	7.000	16.4127	7.0000	
**SGO_1151**	1.725	9.323	0.0000	0.0000	165.500	68.500	246.7711	71.2886	glyA; serine hydroxymethyltransferase
					171.500	77.500	244.7637	77.5000	
**SGO_1154**	0.348	7.523	0.0322	0.1340	33.500	47.500	49.9506	49.4337	prfA; peptide chain release factor 1
					36.500	32.500	52.0926	32.5000	
**SGO_1155**	0.258	6.054	0.1207	0.6079	9.000	9.500	13.4196	9.8867	tdk; thymidine kinase
					15.500	21.000	22.1215	21.0000	
**SGO_1159**	1.630	3.910	0.0590	0.2706	3.000		4.4732		guaC; guanosine monophosphate reductase
					6.000	2.000	8.5632	2.0000	
**SGO_1163**									Iron permease FTR1 family
**SGO_1166**		3.272				4.000		4.1628	voltage-gated chloride channel family protein
						5.500		5.5000	
**SGO_1167**	0.927	8.426	0.0002	0.0001	76.000	54.000	113.3208	56.1983	nox; NADH oxidase
					78.500	62.500	112.0347	62.5000	
**SGO_1169**	0.631	7.557	0.0079	0.0244	44.000	37.500	65.6068	39.0266	NADPH-dependent FMN reductase
					34.500	34.500	49.2382	34.5000	
**SGO_1170**	-0.581	8.759	0.0002	0.0001	60.000	125.500	89.4638	130.6091	NADPH-dependent FMN reductase
					59.000	129.000	84.2044	129.0000	

Log_2 Ratios: -6 -4 -2 0 2 4 6

Show detected proteins only
Show all proteins
Filter by category:
ABC Transporter

Proteins found: 1179

Test	Cutoff
q-Value	.005
p-Value	

	Signif	Direction	Applies To
red	yes	+	ratios, bars
yellow	no	n/a	bars
green	yes	−	ratios, bars
pink	yes	+	*p*- , *q*-Values
light green	yes	−	*p*- , *q*-Values

Dot Plots Dot Plots

Hendrickson *et al.*

Protein	SgPg vs Sg Log_2 Ratio	Log_2 Sum	*q*-Value	*p*-Value	Raw SgPg	Raw Sg	Normalized SgPg	Normalized Sg	Description	Log_2 Ratios
SGO_1171	-4.040	7.574	0.0001	0.0000	3.500	89.000	5.2187	92.6232	thioredoxin family protein	
					4.000	87.000	5.7088	87.0000		
SGO_1174		3.417				4.500		4.6832	histidine kinase	
						6.000		6.0000		
SGO_1176		0.585							peptide methionine sulfoxide reductase-like protein	
						1.500		1.5000		
SGO_1182		1.058				2.000		2.0814	LPXTG cell wall surface protein	
SGO_1184		3.412				3.500		3.6425	GdmH	
						7.000		7.0000		
SGO_1185	1.029	5.215	0.0048	0.0134	9.500	7.000	14.1651	7.2850	acetyltransferase, GNAT family	
					7.500	5.000	10.7040	5.0000		
SGO_1186		5.718				27.500		28.6195	ABC transporter, permease/ATP-binding protein	
						24.000		24.0000		
SGO_1187		6.301				33.500		34.8638	ABC transporter, permease/ATP-binding protein	
						44.000		44.0000		
SGO_1189	-3.706	9.249	0.0003	0.0003	12.500	292.500	18.6383	304.4076	lipoprotein, putative	
					17.500	260.500	24.9759	260.5000		
SGO_1191	0.125	11.651	0.0327	0.1371	580.500	789.500	865.5625	821.6402	rplL; ribosomal protein L7/L12	
					568.000	718.000	810.6459	718.0000		
SGO_1192	0.701	9.702	0.0111	0.0375	141.000	144.500	210.2400	150.3825	BL5; 50S ribosomal protein L10	
					216.500	163.500	308.9874	163.5000		
SGO_1193	0.996	6.452	0.0037	0.0096	22.500	13.500	33.5489	14.0496	gid; Glucose inhibited division protein A	
					17.500	15.000	24.9759	15.0000		

Log2 Ratios axis: -6 -4 -2 0 2 4 6

Show detected proteins only
Show all proteins
Filter by category:
ABC Transporter

Proteins found: 1179

Test	Cutoff
q-Value	.005
p-Value	

	Signif	Direction	Applies To
red	yes	+	ratios, bars
yellow	no	n/a	bars
green	yes	–	ratios, bars
pink	yes	+	*p*- , *q*-Values
light green	yes	–	*p*- , *q*-Values

Dot Plots Dot Plots

Hendrickson *et al.*

Protein	SgPg vs Sg Log$_2$ Ratio	Log$_2$ Sum	*q*-Value	*p*-Value	Raw SgPg	Raw Sg	Normalized SgPg	Normalized Sg	Description
SGO_1196		2.227				4.500		4.6832	satD; SatD-like protein
SGO_1197	-1.218	8.057	0.0018	0.0038	24.000	79.500	35.7855	82.7364	topA; DNA topoisomerase I
					31.000	103.500	44.2430	103.5000	
SGO_1198	-0.387	8.825	0.0055	0.0161	66.500	134.000	99.1557	139.4551	DNA processing Smf protein
					68.000	118.000	97.0492	118.0000	
SGO_1199	-0.356	3.409	0.0322	0.1336	2.000	3.500	2.9821	3.6425	acetyltransferase, CysE/LacA/LpxA/NodL family
						4.000		4.0000	
SGO_1202	0.592	5.619	0.0154	0.0563	8.000	9.000	11.9285	9.3664	GTP-binding protein
					12.500	10.000	17.8399	10.0000	
SGO_1203	0.746	6.612	0.0027	0.0066	19.000	15.500	28.3302	16.1310	anaerobic ribonucleotide reductase
					23.000	20.500	32.8254	20.5000	
SGO_1205	0.892	4.892	0.0147	0.0533	5.500	7.000	8.2009	7.2850	dapA; dihydrodipicolinate synthase
					7.500	3.500	10.7040	3.5000	
SGO_1206	-0.353	7.392	0.0040	0.0107	25.500	42.500	38.0221	44.2302	asd; aspartate-semialdehyde dehydrogenase
					25.000	50.000	35.6798	50.0000	
SGO_1208		3.604				4.000		4.1628	Hypothetical FAD-dependent oxidoreductase, putative
						8.000		8.0000	
SGO_1210	0.250	5.689	0.0723	0.3419	12.500	11.500	18.6383	11.9682	fhs-1; formate--tetrahydrofolate ligase
					7.000	11.000	9.9904	11.0000	
SGO_1213	-2.296	5.647	0.0472	0.2066	3.000	16.000	4.4732	16.6514	coaC; phosphopantothenoylcysteine decarboxylase
						29.000		29.0000	
SGO_1215	0.815	10.153	0.0002	0.0001	243.000	190.000	362.3285	197.7348	manB; phosphomannomutase
					254.500	215.000	363.2207	215.0000	

Log$_2$ Ratios: -6 -4 -2 0 2 4 6

Show detected proteins only
Show all proteins
Filter by category:
ABC Transporter

Proteins found: 1179

Test	Cutoff
q-Value	.005
p-Value	

	Signif	Direction	Applies To
red	yes	+	ratios, bars
yellow	no	n/a	bars
green	yes	−	ratios, bars
pink	yes	+	*p*- , *q*-Values
light green	yes	−	*p*- , *q*-Values

Dot Plots Dot Plots

Hendrickson *et al.*

Protein	SgPg vs Sg Log$_2$ Ratio	Log$_2$ Sum	*q*-Value	*p*-Value	Raw *SgPg*	Raw *Sg*	Normalized *SgPg*	Normalized *Sg*	Description
SGO_1216	1.790	7.228	0.0010	0.0016	43.000	18.500	64.1157	19.2531	bta; Possible bacteriocin transport accessory protein
					36.500	14.500	52.0926	14.5000	
SGO_1219	0.089	7.683	0.0652	0.3035	36.000	44.000	53.6783	45.7912	pta; phosphate acetyltransferase
					36.500	54.000	52.0926	54.0000	
SGO_1221		7.145				62.000		64.5240	ppnK; Probable inorganic polyphosphate/ATP-NAD kinase
						77.000		77.0000	
SGO_1222	-0.323	4.321	0.0170	0.0631		7.000		7.2850	GTP pyrophosphokinase-like protein
					4.000	7.000	5.7088	7.0000	
SGO_1224	0.647	8.869	0.0008	0.0012	100.500	89.500	149.8519	93.1435	Ribose-phosphate pyrophosphokinase 2
					95.000	89.000	135.5834	89.0000	
SGO_1225	0.546	5.333	0.0081	0.0252	7.000	7.500	10.4374	7.8053	pyridoxal-phosphate dependent aminotransferase
					9.500	8.500	13.5583	8.5000	
SGO_1226	0.436	6.473	0.0366	0.1558	16.000	13.500	23.8570	14.0496	hypothetical protein SGO_1226
					18.500	24.500	26.4031	24.5000	
SGO_1228	0.534	4.773	0.0171	0.0636	6.000	6.500	8.9464	6.7646	DNA-binding protein BH0551
					5.000	4.500	7.1360	4.5000	
SGO_1230	-2.690	6.240	0.0003	0.0003	4.000	33.500	5.9643	34.8638	srtA; sortase A
					3.000	30.500	4.2816	30.5000	
SGO_1231	-1.323	8.182	0.0024	0.0054	21.000	90.000	31.3123	93.6639	gyrA; DNA gyrase, A subunit
					37.500	112.000	53.5198	112.0000	
SGO_1232	2.941	10.367	0.0001	0.0000	398.500	65.000	594.1889	67.6461	L-lactate dehydrogenase
					402.000	85.500	573.7318	85.5000	
SGO_1234	0.414	10.895	0.0030	0.0073	368.500	359.500	549.4570	374.1351	rpsA; 30S ribosomal protein S1
					376.000	444.000	536.6247	444.0000	

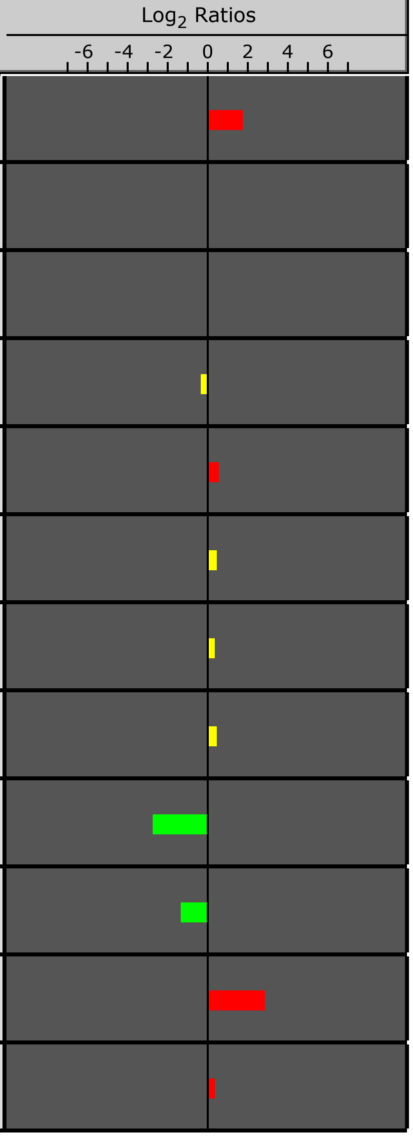

Show detected proteins only
Show all proteins
Filter by category:
ABC Transporter

Proteins found: 1179

Test	Cutoff
q-Value	.005
p-Value	

Color	Signif	Direction	Applies To
red	yes	+	ratios, bars
yellow	no	n/a	bars
green	yes	–	ratios, bars
pink	yes	+	*p*- , *q*-Values
light green	yes	–	*p*- , *q*-Values

Dot Plots Dot Plots

Protein	SgPg vs Sg Log$_2$ Ratio	SgPg vs Sg Log$_2$ Sum	SgPg vs Sg *q*-Value	SgPg vs Sg *p*-Value	Raw *SgPg*	Raw *Sg*	Normalized *SgPg*	Normalized *Sg*	Description
SGO_1237	0.373	6.228	0.0055	0.0159	13.500	17.000	20.1294	17.6921	hypothetical protein SGO_1237
					15.500	15.000	22.1215	15.0000	
SGO_1238	0.021	7.106	0.1464	0.7601	23.500	35.000	35.0400	36.4248	ilvE; branched-chain amino acid aminotransferase
					24.000	32.000	34.2526	32.0000	
SGO_1239	-1.117	6.822	0.0003	0.0003	11.500	35.500	17.1472	36.9452	parC; DNA topoisomerase IV, A subunit
					13.000	40.500	18.5535	40.5000	
SGO_1241		2.946				5.000		5.2035	aminoglycoside adenylyltransferase
						2.500		2.5000	
SGO_1242	-2.894	7.207	0.0175	0.0659		72.500		75.4514	lipoprotein, putative
					6.500	63.000	9.2768	63.0000	
SGO_1244	0.288	5.085	0.1646	0.8635	5.000	12.000	7.4553	12.4885	hypothetical protein SGO_1244
					7.000	4.000	9.9904	4.0000	
SGO_1245	-1.278	5.846	0.0026	0.0059	6.500	17.000	9.6919	17.6921	parE; DNA topoisomerase IV, B subunit
					5.000	23.000	7.1360	23.0000	
SGO_1246		3.940				8.500		8.8460	hypothetical protein SGO_1246
						6.500		6.5000	
SGO_1247		9.144				267.500		278.3898	5'-nucleotidase family protein
						287.500		287.5000	
SGO_1248	0.664	6.259	0.0221	0.0871	19.000	17.500	28.3302	18.2124	pyrC; dihydroorotase
					13.000	11.500	18.5535	11.5000	
SGO_1250		4.412				7.000		7.2850	ung; uracil-DNA glycosylase
						14.000		14.0000	
SGO_1251		1.841				2.000		2.0814	hypothetical protein SGO_1251
						1.500		1.5000	

Log$_2$ Ratios: -6 -4 -2 0 2 4 6

Show detected proteins only
Show all proteins
Filter by category:
ABC Transporter

Proteins found: 1179

Test	Cutoff
q-Value	.005
p-Value	

Color	Signif	Direction	Applies To
red	yes	+	ratios, bars
yellow	no	n/a	bars
green	yes	−	ratios, bars
pink	yes	+	*p*- , *q*-Values
light green	yes	−	*p*- , *q*-Values

Dot Plots Dot Plots

Hendrickson *et al.*

Protein	SgPg vs Sg Log$_2$ Ratio	Log$_2$ Sum	*q*-Value	*p*-Value	Raw *SgPg*	Raw *Sg*	Normalized *SgPg*	Normalized *Sg*	Description
SGO_1252		1.322							integral membrane protein, putative
						2.500		2.5000	
SGO_1253	0.695	6.813	0.0038	0.0099	22.000	17.500	32.8034	18.2124	pyrE; orotate phosphoribosyltransferase
					25.500	25.000	36.3934	25.0000	
SGO_1254	-1.011	5.018	0.0244	0.0978		11.500		11.9682	pyrF; orotidine 5'-phosphate decarboxylase
					4.500	14.000	6.4224	14.0000	
SGO_1255	-0.177	3.313	0.1403	0.7201		2.000		2.0814	pyrd; dihydroorotate dehydrogenase B
					2.000	5.000	2.8544	5.0000	
SGO_1257	0.296	5.116	0.0467	0.2042	6.000	6.000	8.9464	6.2443	probable transcriptional regulator (LysR family)
					7.000	9.500	9.9904	9.5000	
SGO_1258		3.718				4.000		4.1628	NAD-dependent deacetylase (Regulatory protein SIR2-like protein)
						9.000		9.0000	
SGO_1260	0.150	8.225	0.0058	0.0171	51.000	69.000	76.0443	71.8090	deoD; purine nucleoside phosphorylase
					57.000	70.000	81.3500	70.0000	
SGO_1261		1.807							glutamine amidotransferase, class I
						3.500		3.5000	
SGO_1263	0.330	8.861	0.0049	0.0137	84.000	106.500	125.2494	110.8356	purine nucleoside phosphorylase I, inosine and guanosine-specific
					93.500	95.500	133.4426	95.5000	
SGO_1264	0.019	10.003	0.1710	0.9034	177.500	220.000	264.6638	228.9561	deoB; phosphopentomutase
					175.500	282.000	250.4724	282.0000	
SGO_1265	0.958	7.844	0.0077	0.0238	48.000	26.000	71.5711	27.0585	rpiA; ribose 5-phosphate isomerase
					54.000	54.000	77.0684	54.0000	
SGO_1266	0.882	6.214	0.0025	0.0058	18.000	12.500	26.8391	13.0089	trmE; tRNA modification GTPase TrmE
					15.000	13.000	21.4079	13.0000	

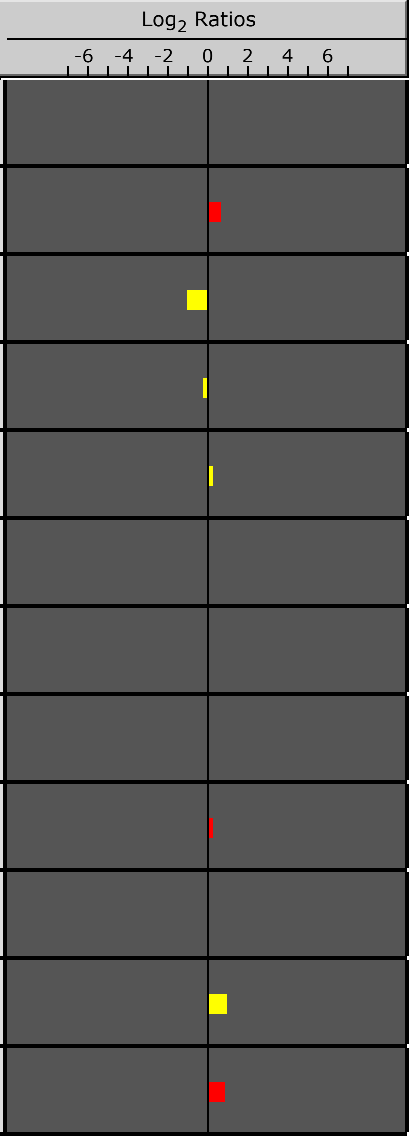

Show detected proteins only
Show all proteins
Filter by category:
ABC Transporter

Proteins found: 1179

Test	Cutoff
q-Value	.005
p-Value	

	Signif	Direction	Applies To
red	yes	+	ratios, bars
yellow	no	n/a	bars
green	yes	−	ratios, bars
pink	yes	+	*p*-, *q*-Values
light green	yes	−	*p*-, *q*-Values

Dot Plots Dot Plots

Protein	SgPg vs Sg Log2 Ratio	Log2 Sum	q-Value	p-Value	Raw SgPg	Raw Sg	Normalized SgPg	Normalized Sg	Description
SGO_1267	-1.825	5.596	0.0185	0.0707	4.000	22.000	5.9643	22.8956	thioredoxin family protein
						19.500		19.5000	
SGO_1273	1.903	8.469	0.0001	0.0000	93.000	40.000	138.6689	41.6284	rpoD; RNA polymerase sigma factor
					98.500	33.500	140.5786	33.5000	
SGO_1274	0.563	4.342	0.0490	0.2164	4.000	2.500	5.9643	2.6018	dnaG; DNA primase
					4.000	6.000	5.7088	6.0000	
SGO_1276	0.281	7.872	0.0142	0.0507	39.000	49.500	58.1515	51.5151	rpsU; ribosomal protein S21
					49.500	54.000	70.6461	54.0000	
SGO_1280		3.549				5.000		5.2035	putative membrane-associated Zn-dependent protease
						6.500		6.5000	
SGO_1281	0.982	5.300	0.0047	0.0128	7.500	5.500	11.1830	5.7239	penicillinase repressor, putative
					10.500	7.500	14.9855	7.5000	
SGO_1283	0.742	8.503	0.0003	0.0003	77.500	68.500	115.5574	71.2886	oxidoreductase
					78.000	64.500	111.3211	64.5000	
SGO_1284	0.132	6.048	0.1080	0.5381	17.000	15.500	25.3481	16.1310	thioredoxin-disulfide reductase
					7.500	14.000	10.7040	14.0000	
SGO_1290		4.483				9.000		9.3664	ABC transporter, permease/ATP-binding protein
						13.000		13.0000	
SGO_1291		3.115				4.000		4.1628	ABC transporter, permease/ATP-binding protein SP1358
						4.500		4.5000	
SGO_1293	0.430	7.822	0.0034	0.0085	46.500	44.500	69.3345	46.3116	asnS; asparaginyl-tRNA synthetase
					42.500	50.000	60.6557	50.0000	
SGO_1297	0.992	6.280	0.0004	0.0005	16.500	12.000	24.6026	12.4885	aspC; aspartate aminotransferase
					19.000	13.500	27.1167	13.5000	

Log2 Ratios: -6 -4 -2 0 2 4 6

Show detected proteins only
Show all proteins
Filter by category:
ABC Transporter

Proteins found: 1179

Test	Cutoff
q-Value	.005
p-Value	

	Signif	Direction	Applies To
red	yes	+	ratios, bars
yellow	no	n/a	bars
green	yes	−	ratios, bars
pink	yes	+	p- , q-Values
light green	yes	−	p- , q-Values

Dot Plots Dot Plots

Streptococcus gordonii

Protein	*SgPg vs Sg* Log_2 Ratio	Log_2 Sum	*q*-Value	*p*-Value	Raw *SgPg*	Raw *Sg*	Normalized *SgPg*	Normalized *Sg*	Description
**SGO_1298**		3.425				6.000		6.2443	hypothetical protein SGO_1298
						4.500		4.5000	
**SGO_1305**	-0.420	6.325	0.0004	0.0004	11.500	21.500	17.1472	22.3753	substrate-binding protein MsmE
					12.000	23.500	17.1263	23.5000	
**SGO_1312**	3.727	7.558	0.0001	0.0000	60.000	7.000	89.4638	7.2850	pepT; peptidase T
					60.000	6.000	85.6316	6.0000	
**SGO_1314**		1.807							metal binding lipoprotein
						3.500		3.5000	
**SGO_1315**	0.030	3.428	0.1661	0.8725	2.000		2.9821		trmD; tRNA (guanine-N1)-methyltransferase
					3.000	3.500	4.2816	3.5000	
**SGO_1316**	0.266	4.747	0.0494	0.2206	4.500	7.000	6.7098	7.2850	16S rRNA processing protein RimM
					5.500	5.000	7.8496	5.0000	
**SGO_1323**	0.691	7.691	0.0033	0.0082	39.500	42.500	58.8970	44.2302	rpsP; ribosomal protein S16
					48.000	35.000	68.5053	35.0000	
**SGO_1324**		5.595				20.500		21.3345	hypothetical protein SGO_1324
						27.000		27.0000	
**SGO_1327**	0.123	6.174	0.1330	0.6792	12.000	13.000	17.8928	13.5292	HAD-superfamily subfamily IIA hydrolase, TIGR01457
					13.500	21.500	19.2671	21.5000	
**SGO_1328**		1.585							hypothetical protein SGO_1328
						3.000		3.0000	
**SGO_1330**		3.772				4.000		4.1628	transporter
						9.500		9.5000	
**SGO_1331**		1.807							HlyB/MsbA family ABC transporter
						3.500		3.5000	

Log₂ Ratios: -6 -4 -2 0 2 4 6

◉ Show detected proteins only
○ Show all proteins
☐ Filter by category: ABC Transporter

Proteins found: 1179

Test	Cutoff
***q*-Value**	.005
p-Value	

Colour	Signif	Direction	Applies To
red	yes	+	ratios, bars
yellow	no	n/a	bars
green	yes	–	ratios, bars
pink	yes	+	*p*- , *q*-Values
light green	yes	–	*p*- , *q*-Values

Dot Plots | Dot Plots

Hendrickson *et al.*

Protein	SgPg vs Sg Log_2 Ratio	Log_2 Sum	*q*-Value	*p*-Value	Raw *SgPg*	Raw *Sg*	Normalized *SgPg*	Normalized *Sg*	Description
SGO_1336	0.050	6.164	0.0802	0.3879	13.000	17.000	19.3838	17.6921	pcrA; ATP-dependent DNA helicase PcrA
					12.000	17.500	17.1263	17.5000	
SGO_1337		1.585							cation efflux family protein
						3.000		3.0000	
SGO_1338	-3.036	5.648	0.0253	0.1018		19.500		20.2938	Signal peptidase I
					2.000	27.000	2.8544	27.0000	
SGO_1339	1.066	11.826	0.0001	0.0001	803.500	551.000	1198.0698	573.4310	pyk; pyruvate kinase
					882.500	600.000	1259.4982	600.0000	
SGO_1340	0.758	10.195	0.0001	0.0000	246.000	213.500	366.8017	222.1915	Phosphofructokinase
					259.000	213.500	369.6431	213.5000	
SGO_1341	-1.076	4.546	0.0154	0.0566	3.000	9.500	4.4732	9.8867	dnaE; DNA-directed DNA polymerase III alpha chain
						9.000		9.0000	
SGO_1342	-1.281	10.262	0.0001	0.0001	128.000	423.500	190.8562	440.7405	ABC transporter, ATP-binding protein SP1715
					117.500	429.000	167.6952	429.0000	
SGO_1343	-1.773	5.547	0.0085	0.0271	4.000	19.000	5.9643	19.7735	ABC transporter, ATP-binding protein SP1715
						21.000		21.0000	
SGO_1347		6.152				39.500		41.1080	membrane associated lipoprotein
						30.000		30.0000	
SGO_1355		3.483				4.500		4.6832	lipoprotein, putative
						6.500		6.5000	
SGO_1360		2.832				3.000		3.1221	ABC transporter domain protein
						4.000		4.0000	
SGO_1361		2.700							ADP-ribosylhydrolase
						6.500		6.5000	

Log2 Ratios: -6 -4 -2 0 2 4 6

Show detected proteins only
Show all proteins
Filter by category:
ABC Transporter

Proteins found: 1179

Test	Cutoff
q-Value	.005
p-Value	

Colour	Signif	Direction	Applies To
red	yes	+	ratios, bars
yellow	no	n/a	bars
green	yes	−	ratios, bars
pink	yes	+	*p*- , *q*-Values
light green	yes	−	*p*- , *q*-Values

Dot Plots Dot Plots

Protein	SgPg vs Sg Log_2 Ratio	Log_2 Sum	*q*-Value	*p*-Value	Raw *SgPg*	Raw *Sg*	Normalized *SgPg*	Normalized *Sg*	Description
SGO_1364	-0.053	6.770	0.1267	0.6436	17.500	22.500	26.0936	23.4160	rumA-2; 23S rRNA (uracil-5-)-methyltransferase RumA
					19.000	32.500	27.1167	32.5000	
SGO_1365	-3.071	6.289	0.0001	0.0001	4.000	33.500	5.9643	34.8638	transcription regulator yrfE
					2.000	34.500	2.8544	34.5000	
SGO_1366		3.719			5.000		7.4553		pheA; prephenate dehydratase
					4.000		5.7088		
SGO_1367	-0.645	4.492	0.0027	0.0065	3.000	6.000	4.4732	6.2443	aroK; shikimate kinase
					3.000	7.500	4.2816	7.5000	
SGO_1368	1.405	4.704	0.0175	0.0655	6.000	1.500	8.9464	1.5611	aroA; 3-phosphoshikimate 1-carboxyvinyltransferase
					6.000	7.000	8.5632	7.0000	
SGO_1369	-0.018	5.905	0.1710	0.9038	9.000	14.000	13.4196	14.5699	L-2-hydroxyisocaproate dehydrogenase
					11.500	15.500	16.4127	15.5000	
SGO_1370	1.245	6.740	0.0010	0.0015	27.500	15.500	41.0043	16.1310	Protein of unknown function (DUF964) superfamily
					24.000	15.500	34.2526	15.5000	
SGO_1372	1.553	5.640	0.0003	0.0002	12.000	5.500	17.8928	5.7239	aroC; chorismate synthase
					13.500	7.000	19.2671	7.0000	
SGO_1373	0.379	5.146	0.0473	0.2080	6.500	5.500	9.6919	5.7239	aroB; 3-dehydroquinate synthase
					7.000	10.000	9.9904	10.0000	
SGO_1374	0.232	4.333	0.0655	0.3054	4.500	4.000	6.7098	4.1628	aroE; shikimate 5-dehydrogenase
					3.000	5.000	4.2816	5.0000	
SGO_1375	0.563	6.044	0.0214	0.0838	16.000	11.000	23.8570	11.4478	aroD; 3-dehydroquinate dehydratase, type I
					11.000	15.000	15.6991	15.0000	
SGO_1376		3.982				7.500		7.8053	Methyltransferase
						8.000		8.0000	

Log_2 Ratios: -6 -4 -2 0 2 4 6

Show detected proteins only
Show all proteins
Filter by category:
ABC Transporter

Proteins found: 1179

Test	Cutoff
q-Value	.005
p-Value	

Colour	Signif	Direction	Applies To
red	yes	+	ratios, bars
yellow	no	n/a	bars
green	yes	–	ratios, bars
pink	yes	+	*p*- , *q*-Values
light green	yes	–	*p*- , *q*-Values

Dot Plots Dot Plots

SgPg vs Sg	# *Streptococcus gordonii*	Hackett Laboratory UW

Summary Table | SgFn vs Sg | SgPg vs Sg | SgPgFn vs Sg | SgPg vs SgFn | SgPgFn vs SgFn | SgPgFn vs SgPg | Coverage

Protein	*SgPg vs Sg* Log$_2$ Ratio	Log$_2$ Sum	*q*-Value	*p*-Value	Raw *SgPg*	Raw *Sg*	Normalized *SgPg*	Normalized *Sg*	Description
SGO_1377	-2.205	8.142	0.0003	0.0002	16.500	105.000	24.6026	109.2745	sulfatase
					18.000	123.000	25.6895	123.0000	
SGO_1381	-0.506	6.495	0.0150	0.0546	10.000	24.000	14.9106	24.9770	csn1; CRISPR-associated protein, Csn1 family
					16.000	27.500	22.8351	27.5000	
SGO_1383	0.692	9.044	0.0016	0.0032	102.000	91.500	152.0885	95.2249	rplS; ribosomal protein L19
					122.000	106.500	174.1176	106.5000	
SGO_1386	1.156	4.082	0.0635	0.2939	6.000		8.9464		chorismate mutase
					3.500	3.000	4.9952	3.0000	
SGO_1388		4.827				9.500		9.8867	pulA-1; pullulanase, type I
						18.500		18.5000	
SGO_1389		5.417				18.000		18.7328	conserved hypothetical protein TIGR00147
						24.000		24.0000	
SGO_1390	-0.884	5.960	0.0007	0.0009	8.000	20.000	11.9285	20.8142	ligA; DNA ligase, NAD-dependent
					7.000	19.500	9.9904	19.5000	
SGO_1394		4.059			4.000		5.9643		hypothetical protein SGO_1394
					7.500		10.7040		
SGO_1397	0.382	6.937	0.0390	0.1674	25.500	20.000	38.0221	20.8142	map; methionine aminopeptidase, type I
					21.500	33.000	30.6847	33.0000	
SGO_1398	-1.419	5.593	0.0015	0.0030	5.000	15.000	7.4553	15.6106	hypothetical protein SGO_1398
					4.000	19.500	5.7088	19.5000	
SGO_1400	-0.556	6.098	0.0023	0.0052	10.000	18.500	14.9106	19.2531	murA-2; UDP-N-acetylglucosamine 1-carboxyvinyltransferase
					9.000	21.500	12.8447	21.5000	
SGO_1405		1.835							hisA; phosphoribosylformimino-5-aminoimidazole carboxamide ribotide isomerase
					2.500		3.5680		

Log$_2$ Ratios: -6 -4 -2 0 2 4 6

Show detected proteins only
Show all proteins
Filter by category:
ABC Transporter

Proteins found: 1179

Test	Cutoff
***q*-Value**	**.005**
p-Value	

Color	Signif	Direction	Applies To
red	yes	+	ratios, bars
yellow	no	n/a	bars
green	yes	–	ratios, bars
pink	yes	+	*p-* , *q*-Values
light green	yes	–	*p-* , *q*-Values

Dot Plots | Dot Plots

Hendrickson *et al.*

Protein	*SgPg vs Sg* Log$_2$ Ratio	Log$_2$ Sum	*q*-Value	*p*-Value	Raw *SgPg*	Raw *Sg*	Normalized *SgPg*	Normalized *Sg*	Description
SGO_1413	-0.695	5.167	0.0323	0.1348	4.500	7.000	6.7098	7.2850	first chain of major exonuclease RexA
					4.500	15.500	6.4224	15.5000	
SGO_1414	-0.773	4.582	0.0176	0.0664	2.500	5.500	3.7277	5.7239	rexB; putative exonuclease RexB
					3.500	9.500	4.9952	9.5000	
SGO_1415		2.481				2.000		2.0814	LPXTG cell wall surface protein, X-prolyl dipeptidylaminopeptidase, putative
						3.500		3.5000	
SGO_1416		1.585							putative histidine kinase
						3.000		3.0000	
SGO_1417		2.973							response regulator protein
					5.500		7.8496		
SGO_1422	-0.278	6.778	0.0007	0.0009	17.000	28.500	25.3481	29.6602	hypothetical protein SGO_1422
					17.000	30.500	24.2623	30.5000	
SGO_1426	1.751	14.510	0.0001	0.0000	5900.500	2771.500	8798.0219	2884.3267	eno; enolase
					6433.500	2470.500	9181.8488	2470.5000	
SGO_1431	-1.476	10.366	0.0048	0.0136	117.500	570.000	175.2000	593.2045	EzrA; Septation ring formation regulator ezrA
					117.500	383.500	167.6952	383.5000	
SGO_1432	0.327	7.630	0.0534	0.2414	31.000	33.500	46.2230	34.8638	gyrB; DNA gyrase, B subunit
					44.500	53.500	63.5101	53.5000	
SGO_1434	1.419	4.025	0.0515	0.2317	3.500		5.2187		thiJ; 4-methyl-5(beta-hydroxyethyl)-thiazole monophosphate synthesis protein
					6.000	2.500	8.5632	2.5000	
SGO_1437		3.669				5.500		5.7239	DnaQ family exonuclease/DinG family helicase, putative
						7.000		7.0000	
SGO_1439	-1.888	7.169	0.0001	0.0000	11.000	55.500	16.4017	57.7594	ftsX; cell division protein FtsX
					10.000	55.500	14.2719	55.5000	

Log$_2$ Ratios: -6 -4 -2 0 2 4 6

Show detected proteins only
Show all proteins
Filter by category:
ABC Transporter

Proteins found: 1179

Test	Cutoff
q-Value	.005
p-Value	

	Signif	Direction	Applies To
red	yes	+	ratios, bars
yellow	no	n/a	bars
green	yes	−	ratios, bars
pink	yes	+	*p*- , *q*-Values
light green	yes	−	*p*- , *q*-Values

Dot Plots Dot Plots

Streptococcus gordonii

Protein	*SgPg vs Sg* Log$_2$ Ratio	Log$_2$ Sum	*q*-Value	*p*-Value	Raw *SgPg*	Raw *Sg*	Normalized *SgPg*	Normalized *Sg*	Description	Log$_2$ Ratios (-6 -4 -2 0 2 4 6)
SGO_1440	-1.350	8.204	0.0001	0.0000	27.000	104.500	40.2587	108.7542	cell-division ATP-binding protein FtsE	
					30.000	103.000	42.8158	103.0000		
SGO_1441	0.860	6.328	0.0120	0.0413	13.500	14.500	20.1294	15.0903	prfB; peptide chain release factor 2	
					22.500	13.000	32.1119	13.0000		
SGO_1446	0.547	6.652	0.0188	0.0726	18.500	15.000	27.5847	15.6106	murF; UDP-N-acetylmuramoyl-tripeptide--D-alanyl-D-alanine ligase	
					22.000	26.000	31.3983	26.0000		
SGO_1447	0.694	7.021	0.0026	0.0059	29.500	23.500	43.9864	24.4567	ddlA; D-Ala-D-Ala ligase	
					25.500	25.000	36.3934	25.0000		
SGO_1449	-2.494	7.277	0.0006	0.0007	7.500	69.500	11.1830	72.3293	pbp2b; penicillin-binding protein 2B	
					8.500	59.500	12.1311	59.5000		
SGO_1450	0.312	5.654	0.1740	0.9221	10.500	5.000	15.6562	5.2035	hypothetical protein SGO_1450	
					7.000	19.500	9.9904	19.5000		
SGO_1451	0.844	7.426	0.0011	0.0018	37.000	33.500	55.1694	34.8638	frr; ribosome recycling factor	
					38.500	27.000	54.9469	27.0000		
SGO_1452	1.012	7.403	0.0077	0.0237	45.500	30.500	67.8434	31.7416	pyrH; uridylate kinase	
					32.000	24.000	45.6702	24.0000		
SGO_1453		4.100			11.500		17.1472		glycosyl transferase, family 8 SP1766	
SGO_1454		4.989				18.500		19.2531	amino acid permease family protein	
						12.500		12.5000		
SGO_1455	1.547	9.960	0.0027	0.0064	208.000	129.500	310.1413	134.7719	rplA; ribosomal protein L1	
					304.000	117.000	433.8668	117.0000		
SGO_1456	0.563	7.956	0.0124	0.0428	41.500	46.500	61.8791	48.3930	rplK; ribosomal protein L11	
					61.000	51.000	87.0588	51.0000		

Show detected proteins only
Show all proteins
Filter by category:
ABC Transporter

Proteins found: 1179

Test	Cutoff
***q*-Value**	**.005**
p-Value	

	Signif	Direction	Applies To
red	yes	+	ratios, bars
yellow	no	n/a	bars
green	yes	−	ratios, bars
pink	yes	+	*p*- , *q*-Values
light green	yes	−	*p*- , *q*-Values

Dot Plots Dot Plots

Protein	SgPg vs Sg Log2 Ratio	Log2 Sum	q-Value	p-Value	Raw SgPg	Raw Sg	Normalized SgPg	Normalized Sg	Description	Log2 Ratios
SGO_1458	-3.777	7.025	0.0025	0.0057	3.500	71.500	5.2187	74.4107	aha1; cation-transporting ATPase yfgQ	
					2.500	47.000	3.5680	47.0000		
SGO_1459		1.161			1.500		2.2366		glyoxylase family protein	
SGO_1460	-1.693	7.097	0.0017	0.0034	11.000	57.500	16.4017	59.8408	DNA translocase ftsK	
					11.000	45.000	15.6991	45.0000		
SGO_1463	-1.582	7.422	0.0003	0.0002	16.000	59.500	23.8570	61.9222	peptidyl-prolyl cis-trans isomerase	
					13.500	66.500	19.2671	66.5000		
SGO_1464	-1.171	7.558	0.0021	0.0045	20.000	55.000	29.8213	57.2390	uncharacterized probable metal-binding protein	
					19.500	73.500	27.8303	73.5000		
SGO_1465	0.016	7.297	0.1771	0.9448	30.000	32.000	44.7319	33.3027	ABC transporter, ATP-binding protein SP0770	
					24.000	45.000	34.2526	45.0000		
SGO_1466	-0.616	5.754	0.0142	0.0510	8.000	18.500	11.9285	19.2531	MTA/SAH nucleosidase	
					6.500	13.500	9.2768	13.5000		
SGO_1468		1.585							MutT/nudix family protein	
						3.000		3.0000		
SGO_1469	0.365	7.880	0.0011	0.0018	43.000	48.500	64.1157	50.4744	glmU; UDP-N-acetylglucosamine pyrophosphorylase	
					48.000	52.500	68.5053	52.5000		
SGO_1472	0.998	4.385	0.0738	0.3513	7.500		11.1830		acetyltransferase, GNAT family	
					4.000	4.000	5.7088	4.0000		
SGO_1475		4.513				8.000		8.3257	lipoprotein, putative	
						14.500		14.5000		
SGO_1483		1.643				3.000		3.1221	ABC transporter, permease protein, probable SP0913	

Log2 Ratios axis: -6 -4 -2 0 2 4 6

Show detected proteins only
Show all proteins
Filter by category:
ABC Transporter

Proteins found: 1179

Test	Cutoff
q-Value	.005
p-Value	

	Signif	Direction	Applies To
red	yes	+	ratios, bars
yellow	no	n/a	bars
green	yes	−	ratios, bars
pink	yes	+	p- , q-Values
light green	yes	−	p- , q-Values

Dot Plots Dot Plots

SgPg vs Sg

Streptococcus gordonii

Hackett Laboratory UW

Summary Table | SgFn vs Sg | SgPg vs Sg | SgPgFn vs Sg | SgPg vs SgFn | SgPgFn vs SgFn | SgPgFn vs SgPg | Coverage

Protein	SgPg vs Sg: Log_2 Ratio	Log_2 Sum	*q*-Value	*p*-Value	Raw: *SgPg*	Raw: *Sg*	Normalized: *SgPg*	Normalized: *Sg*	Description	Log_2 Ratios (-6 -4 -2 0 2 4 6)
SGO_1484		3.135			3.500		5.2187		ABC transporter, ATP-binding protein	
					2.500		3.5680			
SGO_1486		1.322							beta-galactosidase	
						2.500		2.5000		
SGO_1487	-3.730	6.626	0.0185	0.0708		41.000		42.6691	LPXTG cell wall surface protein, Cna protein B-type domain	
					2.500	52.500	3.5680	52.5000		
SGO_1493	-2.649	6.864	0.0185	0.0704		47.000		48.9134	hypothetical protein SGO_1493	
					6.000	59.000	8.5632	59.0000		
SGO_1495		4.523				12.000		12.4885	putative transcriptional regulator	
						10.500		10.5000		
SGO_1498		1.643				3.000		3.1221	ABC transporter, permease protein, probable SP0913	
SGO_1504		3.785				7.000		7.2850	histidine kinase	
						6.500		6.5000		
SGO_1507		5.334				20.500		21.3345	hypothetical protein SGO_1507	
						19.000		19.0000		
SGO_1512	0.741	3.060			3.500	3.000	5.2187	3.1221	lacG; 6-phospho-beta-galactosidase	
SGO_1513		1.807							lacE; PTS system, lactose-specific IIBC component	
						3.500		3.5000		
SGO_1516	1.216	6.199	0.0050	0.0144	14.000	9.500	20.8749	9.8867	lacD-2; tagatose 1,6-diphosphate aldolase	
					21.500	12.000	30.6847	12.0000		
SGO_1518	0.892	4.926	0.0015	0.0030	7.000	4.500	10.4374	4.6832	lacB-2; galactose-6-phosphate isomerase, LacB subunit	
					6.500	6.000	9.2768	6.0000		

Show detected proteins only
Show all proteins
Filter by category:
ABC Transporter

Proteins found:
1179

Test	Cutoff
***q*-Value**	**.005**
p-Value	

	Signif	Direction	Applies To
red	yes	+	ratios, bars
yellow	no	n/a	bars
green	yes	−	ratios, bars
pink	yes	+	*p*- , *q*-Values
light green	yes	−	*p*- , *q*-Values

Dot Plots Dot Plots

Hendrickson *et al.*

Protein	SgPg vs Sg Log2 Ratio	Log2 Sum	q-Value	p-Value	Raw SgPg	Raw Sg	Normalized SgPg	Normalized Sg	Description
SGO_1520		3.058				8.000		8.3257	PTS system, IIC component
SGO_1521		2.170							PTS system IIB component (galactitol/fructose specific)
						4.500		4.5000	
SGO_1523									lacD-1; tagatose 1,6-diphosphate aldolase
SGO_1527		1.865				3.500		3.6425	Lactose phosphotransferase system repressor
SGO_1529	-1.199	6.053	0.0017	0.0037	8.500	21.000	12.6740	21.8549	hypothetical protein SGO_1529
					5.500	24.000	7.8496	24.0000	
SGO_1530	0.080	8.516	0.1062	0.5268	66.000	95.500	98.4102	99.3878	methionine-tRNA ligase
					62.500	79.000	89.1996	79.0000	
SGO_1531	0.988	6.363	0.0013	0.0023	20.000	13.000	29.8213	13.5292	xth; exodeoxyribonuclease III
					17.500	14.000	24.9759	14.0000	
SGO_1534	1.089	5.713	0.0490	0.2161	15.500	3.500	23.1115	3.6425	ArsC family
					7.500	15.000	10.7040	15.0000	
SGO_1536	-0.019	6.475	0.1779	0.9526	19.000	18.000	28.3302	18.7328	conserved hypothetical protein TIGR00096
					11.500	25.500	16.4127	25.5000	
SGO_1538		3.266				3.000		3.1221	DNA-directed DNA polymerase III, delta'' chain
						6.500		6.5000	
SGO_1539	0.530	6.365	0.0031	0.0075	15.000	16.000	22.3660	16.6514	tmk; thymidylate kinase
					18.500	17.000	26.4031	17.0000	
SGO_1540	-2.147	5.138	0.0124	0.0428		16.000		16.6514	YitT family protein
					2.500	15.000	3.5680	15.0000	

Log2 Ratios: -6 -4 -2 0 2 4 6

Show detected proteins only
Show all proteins
Filter by category:
ABC Transporter

Proteins found: 1179

Test	Cutoff
q-Value	.005
p-Value	

	Signif	Direction	Applies To
red	yes	+	ratios, bars
yellow	no	n/a	bars
green	yes	−	ratios, bars
pink	yes	+	p- , q-Values
light green	yes	−	p- , q-Values

Dot Plots Dot Plots

Protein	*SgPg vs Sg* Log$_2$ Ratio	Log$_2$ Sum	*q*-Value	*p*-Value	Raw *SgPg*	Raw *Sg*	Normalized *SgPg*	Normalized *Sg*	Description
**SGO_1541**	-1.636	7.569	0.0014	0.0025	15.500	60.500	23.1115	62.9629	atpC; ATP synthase F1, epsilon subunit
					16.000	81.000	22.8351	81.0000	
**SGO_1542**	-1.008	10.281	0.0002	0.0001	134.000	410.500	199.8025	427.2113	atpD; ATP synthase F1, beta subunit
					149.500	403.500	213.3654	403.5000	
**SGO_1543**	-1.291	7.418	0.0002	0.0001	17.000	60.500	25.3481	62.9629	atpG; ATP synthase F1, gamma subunit
					17.000	58.500	24.2623	58.5000	
**SGO_1544**	-0.726	10.124	0.0001	0.0001	137.500	330.000	205.0213	343.4342	atpA; ATP synthase F1, alpha subunit
					151.000	352.000	215.5062	352.0000	
**SGO_1545**	-1.669	7.677	0.0004	0.0004	16.500	80.000	24.6026	83.2568	atpH; ATP synthase F1, delta subunit
					17.000	72.500	24.2623	72.5000	
**SGO_1546**	-1.026	8.163	0.0016	0.0031	37.000	86.500	55.1694	90.0214	atpF; ATP synthase F0, B subunit
					28.000	101.500	39.9614	101.5000	
**SGO_1550**	0.394	7.975	0.0120	0.0408	49.500	45.000	73.8077	46.8319	glgP-1; glycogen phosphorylase
					48.000	62.500	68.5053	62.5000	
**SGO_1551**	0.606	5.888	0.1122	0.5606	14.000	5.000	20.8749	5.2035	glgA; Glycogen synthase
					8.500	21.000	12.1311	21.0000	
**SGO_1552**	0.946	7.357	0.0188	0.0722	47.000	32.500	70.0800	33.8231	glgD; glucose-1-phosphate adenylyltransferase, GlgD subunit
					27.000	21.500	38.5342	21.5000	
**SGO_1553**	1.515	7.492	0.0004	0.0005	45.000	18.500	67.0979	19.2531	glgC; glucose-1-phosphate adenylyltransferase
					46.000	28.000	65.6509	28.0000	
**SGO_1554**	1.332	7.526	0.0014	0.0027	44.500	19.000	66.3523	19.7735	glgB; 1,4-alpha-glucan branching enzyme
					45.000	34.000	64.2237	34.0000	
**SGO_1555**	0.101	11.189	0.0357	0.1513	410.000	575.500	611.3362	598.9284	ptsI; phosphoenolpyruvate-protein phosphotransferase
					417.500	528.500	595.8533	528.5000	

Log$_2$ Ratios: -6 -4 -2 0 2 4 6

Show detected proteins only
Show all proteins
Filter by category:
ABC Transporter

Proteins found: 1179

Test	Cutoff
***q*-Value**	**.005**
p-Value	

	Signif	Direction	Applies To
red	yes	+	ratios, bars
yellow	no	n/a	bars
green	yes	−	ratios, bars
light red	yes	+	*p*- , *q*-Values
light green	yes	−	*p*- , *q*-Values

Dot Plots Dot Plots

Protein	SgPg vs Sg Log2 Ratio	Log2 Sum	q-Value	p-Value	Raw SgPg	Raw Sg	Normalized SgPg	Normalized Sg	Description
SGO_1556	0.249	11.490	0.0302	0.1244	455.500	646.000	679.1796	672.2984	phosphocarrier protein HPr
					622.500	635.500	888.4279	635.5000	
SGO_1557		4.029				8.000		8.3257	NrdH-redoxin
						8.000		8.0000	
SGO_1558	0.059	8.566	0.0833	0.4048	64.500	83.000	96.1736	86.3789	nrdE; ribonucleoside-diphosphate reductase large chain
					68.000	99.500	97.0492	99.5000	
SGO_1559	0.935	8.231	0.0007	0.0009	69.000	45.500	102.8834	47.3523	ribonucleoside-diphosphate reductase, beta subunit
					66.000	56.000	94.1948	56.0000	
SGO_1561		3.674				6.500		6.7646	putative zinc metallopeptidase
						6.000		6.0000	
SGO_1562		4.516				9.500		9.8867	hypothetical protein SGO_1562
						13.000		13.0000	
SGO_1564	-0.168	7.022	0.0959	0.4718	20.000	46.000	29.8213	47.8726	transcription regulator, probable -related protein
					20.500	23.000	29.2575	23.0000	
SGO_1570	0.166	9.513	0.0562	0.2548	139.500	146.500	208.0034	152.4640	alaS; alanyl-tRNA synthetase
					124.500	192.500	177.6856	192.5000	
SGO_1571		4.888				15.000		15.6106	Protein of unknown function (DUF567) superfamily
						14.000		14.0000	
SGO_1572		9.632				377.500		392.8679	proteinase maturation protein, putative
						400.500		400.5000	
SGO_1573	0.121	3.873	0.1440	0.7432	2.500		3.7277		O-methyltransferase family protein
					4.500	4.500	6.4224	4.5000	
SGO_1574	1.082	9.069	0.0001	0.0000	123.500	81.000	184.1464	84.2975	pepF-1; oligoendopeptidase F
					126.500	88.000	180.5400	88.0000	

Log2 Ratios: -6 -4 -2 0 2 4 6

Show detected proteins only
Show all proteins
Filter by category:
ABC Transporter

Proteins found: 1179

Test	Cutoff
q-Value	.005
p-Value	

Color	Signif	Direction	Applies To
red	yes	+	ratios, bars
yellow	no	n/a	bars
green	yes	–	ratios, bars
pink	yes	+	p-, q-Values
light green	yes	–	p-, q-Values

Dot Plots Dot Plots

Protein	SgPg vs Sg Log_2 Ratio	SgPg vs Sg Log_2 Sum	SgPg vs Sg *q*-Value	SgPg vs Sg *p*-Value	Raw SgPg	Raw Sg	Normalized SgPg	Normalized Sg	Description
SGO_1576		2.832				3.000		3.1221	ptcC; PTS system, IIC component
						4.000		4.0000	
SGO_1580		4.634			9.000		13.4196		PTS system, Lactose/Cellobiose specific IIB subunit
					8.000		11.4175		
SGO_1585	-2.854	6.245	0.0039	0.0104	1.500	38.500	2.2366	40.0673	D-Alanyl-D-Alanine carboxypeptidase
					6.000	25.000	8.5632	25.0000	
SGO_1587	0.437	6.170	0.0692	0.3251	17.500	10.000	26.0936	10.4071	queA; S-adenosylmethionine:tRNA ribosyltransferase-isomerase
					10.500	20.500	14.9855	20.5000	
SGO_1588		3.890				8.000		8.3257	arcR; arginine repressor
						6.500		6.5000	
SGO_1589		3.546				4.500		4.6832	arcT; putative transaminase/peptidase
						7.000		7.0000	
SGO_1590		6.934				55.500		57.7594	arcD; arginine-ornithine antiporter
						64.500		64.5000	
SGO_1591	-1.436	6.786	0.0047	0.0131	12.500	31.500	18.6383	32.7824	arcC; carbamate kinase
					8.000	47.500	11.4175	47.5000	
SGO_1592	-0.713	9.197	0.0000	0.0000	74.500	174.500	111.0843	181.6038	arcB; ornithine carbamoyltransferase
					78.000	183.000	111.3211	183.0000	
SGO_1593	-0.870	8.998	0.0009	0.0015	58.000	148.500	86.4817	154.5454	arcA; arginine deiminase
					66.000	176.000	94.1948	176.0000	
SGO_1594		3.486				5.000		5.2035	Crp/Fnr family protein
						6.000		6.0000	
SGO_1598		2.836				3.500		3.6425	hypothetical protein SGO_1598
						3.500		3.5000	

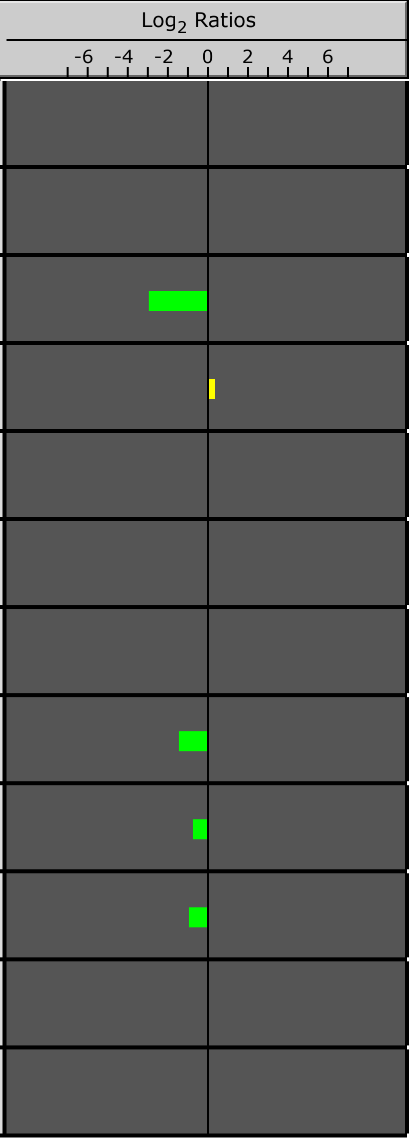

Show detected proteins only
Show all proteins
Filter by category:
ABC Transporter

Proteins found: 1179

Test	Cutoff
q-Value	.005
p-Value	

	Signif	Direction	Applies To
red	yes	+	ratios, bars
yellow	no	n/a	bars
green	yes	−	ratios, bars
pink	yes	+	*p*- , *q*-Values
light green	yes	−	*p*- , *q*-Values

Dot Plots Dot Plots

Protein	SgPg vs Sg				Raw		Normalized		Description
	Log_2 Ratio	Log_2 Sum	*q*-Value	*p*-Value	*SgPg*	*Sg*	*SgPg*	*Sg*	
SGO_1599	0.417	9.437	0.0020	0.0044	127.500	135.500	190.1106	141.0162	sodA; manganese-dependent superoxide dismutase
					144.500	156.000	206.2294	156.0000	
SGO_1600		3.975				5.500		5.7239	DNA polymerase III, delta chain
						10.000		10.0000	
SGO_1601		2.604				2.000		2.0814	celB; DNA internalization-related competence protein celB
						4.000		4.0000	
SGO_1604	-2.382	6.699	0.0006	0.0007	6.500	38.000	9.6919	39.5470	acyltransferase family protein
					5.000	47.500	7.1360	47.5000	
SGO_1605	-1.656	6.552	0.0008	0.0012	7.000	31.000	10.4374	32.2620	P-type ATPase, metal cation transport
					8.500	39.000	12.1311	39.0000	
SGO_1606	-0.708	4.993	0.0157	0.0575	5.500	9.000	8.2009	9.3664	DNA methyltransferase signature protein
					3.000	10.000	4.2816	10.0000	
SGO_1609	-0.739	8.236	0.0002	0.0001	37.000	88.500	55.1694	92.1028	ATP-dependent RNA helicase, DEAD/DEAH box family
					40.500	96.500	57.8013	96.5000	
SGO_1614		4.239				9.500		9.8867	magnesium and cobalt transporter CorA family
						9.000		9.0000	
SGO_1617	0.372	6.364	0.0788	0.3791	11.000	12.000	16.4017	12.4885	prfC; peptide chain release factor 3
					21.000	23.500	29.9711	23.5000	
SGO_1619	-1.786	9.723	0.0017	0.0033	73.000	357.500	108.8477	372.0537	cation-transporting ATPase, E1-E2 family
					57.000	283.000	81.3500	283.0000	
SGO_1621	-0.354	5.860	0.0078	0.0242	8.000	14.500	11.9285	15.0903	HD domain protein
					9.500	17.500	13.5583	17.5000	
SGO_1622	-0.155	5.499	0.0807	0.3914	7.000	14.000	10.4374	14.5699	Cof family protein
					7.500	9.500	10.7040	9.5000	

Log_2 Ratios: -6 -4 -2 0 2 4 6

Show detected proteins only
Show all proteins
Filter by category: ABC Transporter

Proteins found: 1179

Test	Cutoff
q-Value	.005
p-Value	

	Signif	Direction	Applies To
red	yes	+	ratios, bars
yellow	no	n/a	bars
green	yes	–	ratios, bars
pink	yes	+	*p*- , *q*-Values
light green	yes	–	*p*- , *q*-Values

Dot Plots Dot Plots

Hendrickson *et al.*

Protein	SgPg vs Sg: Log_2 Ratio	Log_2 Sum	*q*-Value	*p*-Value	Raw: *SgPg*	Raw: *Sg*	Normalized: *SgPg*	Normalized: *Sg*	Description
SGO_1623	-0.997	6.773	0.0003	0.0003	12.500	33.500	18.6383	34.8638	murM; MurM
					12.500	38.000	17.8399	38.0000	
SGO_1624	-0.926	5.632	0.0115	0.0389	6.000	19.500	8.9464	20.2938	murN; MurN protein
					5.500	12.500	7.8496	12.5000	
SGO_1625	-1.236	8.124	0.0001	0.0000	29.000	95.000	43.2408	98.8674	acetoin utilization putative/CBS domain protein
					28.000	97.000	39.9614	97.0000	
SGO_1626	-0.735	7.128	0.0047	0.0132	16.000	37.000	23.8570	38.5063	branched-chain amino acid ABC transporter, ATP-binding protein
					20.000	49.000	28.5439	49.0000	
SGO_1627	-1.163	7.382	0.0038	0.0099	18.500	64.500	27.5847	67.1258	branched-chain amino acid ABC transporter, ATP-binding protein
					16.500	48.500	23.5487	48.5000	
SGO_1630	-1.166	10.605	0.0001	0.0000	162.000	509.000	241.5523	529.7212	branched-chain amino acid ABC transporter, amino acid-binding protein
					167.000	547.500	238.3413	547.5000	
SGO_1631		3.428				6.500		6.7646	hypothetical protein SGO_1631
						4.000		4.0000	
SGO_1632	-0.250	7.229	0.0036	0.0092	22.500	37.500	33.5489	39.0266	clpP; ATP-dependent Clp protease, proteolytic subunit ClpP
					24.500	42.500	34.9662	42.5000	
SGO_1633	0.909	8.114	0.0001	0.0000	60.000	45.000	89.4638	46.8319	upp; uracil phosphoribosyltransferase
					64.000	49.500	91.3404	49.5000	
SGO_1634	-5.111	9.102	0.0000	0.0000	7.000	256.500	10.4374	266.9420	magnesium-translocating P-type ATPase
					4.000	266.500	5.7088	266.5000	
SGO_1638	1.421	5.055	0.0014	0.0027	7.500	5.500	11.1830	5.7239	murE; UDP-N-acetylmuramoylalanyl-D-glutamate--2,6-diaminopimelate ligase
					9.000	3.500	12.8447	3.5000	
SGO_1648	1.546	9.176	0.0020	0.0044	124.500	61.500	185.6374	64.0036	ppx1; inorganic pyrophosphatase, manganese-dependent
					172.000	83.500	245.4773	83.5000	

Log_2 Ratios

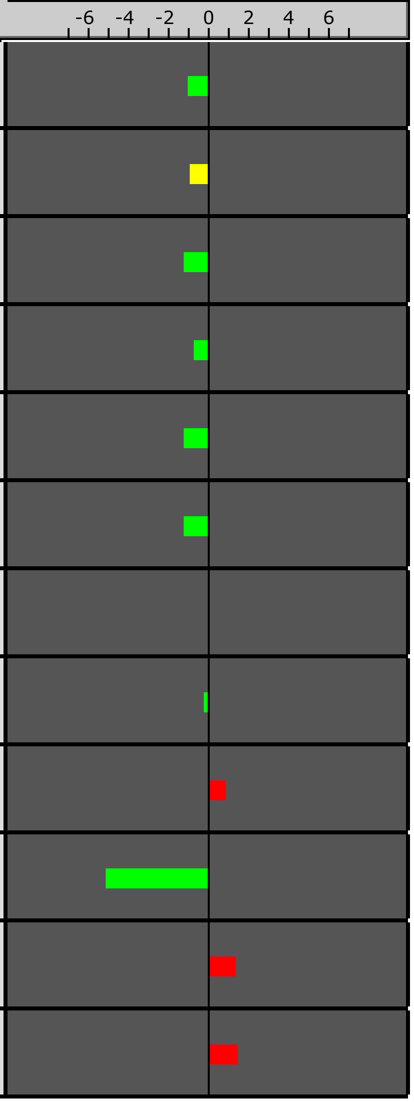

Show detected proteins only
Show all proteins
Filter by category:
ABC Transporter

Proteins found: 1179

Test	Cutoff
q-Value	.005
p-Value	

	Signif	Direction	Applies To
red	yes	+	ratios, bars
yellow	no	n/a	bars
green	yes	–	ratios, bars
pink	yes	+	*p*- , *q*-Values
light green	yes	–	*p*- , *q*-Values

Dot Plots Dot Plots

Protein	SgPg vs Sg: Log_2 Ratio	Log_2 Sum	*q*-Value	*p*-Value	Raw: *SgPg*	Raw: *Sg*	Normalized: *SgPg*	Normalized: *Sg*	Description
**SGO_1649**	-2.242	5.905	0.0010	0.0017	2.500	26.000	3.7277	27.0585	act; pyruvate formate-lyase-activating enzyme
					5.000	22.000	7.1360	22.0000	
**SGO_1650**		7.977				109.000		113.4373	LPXTG cell wall surface protein, collagen binding domain
						138.500		138.5000	
**SGO_1651**		9.536				357.000		371.5333	LPXTG cell wall surface protein, nuclease/phosphatase domain
						371.000		371.0000	
**SGO_1652**	0.184	7.218	0.0578	0.2631	26.500	28.500	39.5132	29.6602	intracellular glycosyl hydrolase
					27.500	40.500	39.2478	40.5000	
**SGO_1653**	-2.054	8.998	0.0002	0.0001	32.500	187.500	48.4596	195.1331	trehalose PTS enzyme II
					35.500	217.000	50.6654	217.0000	
**SGO_1655**	-2.739	6.857	0.0016	0.0030	4.000	40.500	5.9643	42.1487	CBS domain protein/possible hemolysin
					6.500	58.500	9.2768	58.5000	
**SGO_1657**		4.486				10.000		10.4071	membrane protein
						12.000		12.0000	
**SGO_1661**		3.822				3.500		3.6425	radical SAM protein, TIGR01212 family
						10.500		10.5000	
**SGO_1665**		2.585							rRNA methylase
						6.000		6.0000	
**SGO_1666**	-0.970	6.980	0.0056	0.0162	15.500	33.500	23.1115	34.8638	trkA; potassium uptake protein, Trk family
					13.500	49.000	19.2671	49.0000	
**SGO_1669**	-0.728	7.307	0.0126	0.0436	22.500	38.500	33.5489	40.0673	ribosomal large subunit pseudouridine synthase B
					18.000	59.000	25.6895	59.0000	
**SGO_1670**		2.379				5.000		5.2035	scpB; segregation and condensation protein B

Log_2 Ratios: -6 -4 -2 0 2 4 6

Show detected proteins only
Show all proteins
Filter by category:
ABC Transporter

Proteins found: 1179

Test	Cutoff
***q*-Value**	**.005**
p-Value	

	Signif	Direction	Applies To
red	yes	+	ratios, bars
yellow	no	n/a	bars
green	yes	–	ratios, bars
pink	yes	+	*p*- , *q*-Values
light green	yes	–	*p*- , *q*-Values

Dot Plots | Dot Plots

Hendrickson *et al.*

Protein	SgPg vs Sg Log$_2$ Ratio	Log$_2$ Sum	*q*-Value	*p*-Value	Raw SgPg	Raw Sg	Normalized SgPg	Normalized Sg	Description
SGO_1674	-0.643	5.361	0.0040	0.0107	6.000	13.000	8.9464	13.5292	phosphodiesterase, MJ0936 family
					5.000	11.500	7.1360	11.5000	
SGO_1675	-0.654	6.276	0.0214	0.0834	8.500	17.500	12.6740	18.2124	HAM1 protein-like protein
					12.000	29.500	17.1263	29.5000	
SGO_1676	1.692	5.845	0.0003	0.0002	14.500	5.500	21.6204	5.7239	murI; glutamate racemase
					15.500	8.000	22.1215	8.0000	
SGO_1678	0.029	5.328	0.1365	0.6999	10.500	7.500	15.6562	7.8053	lysA; diaminopimelate decarboxylase
					4.000	11.000	5.7088	11.0000	
SGO_1679	-1.278	10.798	0.0004	0.0003	198.000	608.000	295.2306	632.7514	phosphotransferase system enzyme II
					159.500	625.000	227.6373	625.0000	
SGO_1680	-2.691	8.003	0.0007	0.0010	11.000	119.000	16.4017	123.8444	phosphotransferase system enzyme II
					12.500	98.500	17.8399	98.5000	
SGO_1681	-1.826	10.284	0.0000	0.0000	93.000	471.500	138.6689	490.6946	PTS system, mannose/fructose/sorbose family, IID component
					95.000	481.500	135.5834	481.5000	
SGO_1683	0.563	8.319	0.0012	0.0021	67.000	59.500	99.9013	61.9222	serS; seryl-tRNA synthetase
					63.500	67.000	90.6268	67.0000	
SGO_1684	1.477	6.486	0.0012	0.0020	24.000	9.500	35.7855	9.8867	acyl-CoA dehydrogenase family
					21.000	14.000	29.9711	14.0000	
SGO_1685	2.779	8.132	0.0001	0.0000	84.000	14.500	125.2494	15.0903	putative peroxidase / antioxidase
					83.500	21.000	119.1707	21.0000	
SGO_1687	-0.031	8.036	0.1317	0.6708	46.500	62.000	69.3345	64.5240	accA; acetyl-CoA carboxylase, carboxyl transferase, alpha subunit
					42.500	68.000	60.6557	68.0000	
SGO_1688	-0.423	6.692	0.0434	0.1883	14.500	20.500	21.6204	21.3345	accD; acetyl-CoA carboxylase, carboxyl transferase, beta subunit
					15.000	39.000	21.4079	39.0000	

Log$_2$ Ratios: -6 -4 -2 0 2 4 6

Show detected proteins only
Show all proteins
Filter by category:
ABC Transporter

Proteins found: 1179

Test	Cutoff
***q*-Value**	**.005**
p-Value	

	Signif	Direction	Applies To
red	yes	+	ratios, bars
yellow	no	n/a	bars
green	yes	–	ratios, bars
pink	yes	+	*p*- , *q*-Values
light green	yes	–	*p*- , *q*-Values

Dot Plots Dot Plots

Protein	SgPg vs Sg Log$_2$ Ratio	Log$_2$ Sum	q-Value	p-Value	Raw SgPg	Raw Sg	Normalized SgPg	Normalized Sg	Description
SGO_1689	0.770	7.623	0.0018	0.0039	40.000	30.500	59.6426	31.7416	accC; acetyl-CoA carboxylase, biotin carboxylase
					45.000	41.500	64.2237	41.5000	
SGO_1690		5.878			16.000		23.8570		fabZ; beta-hydroxyacyl-(acyl-carrier-protein) dehydratase FabZ
					24.500		34.9662		
SGO_1691	1.379	8.276	0.0017	0.0036	77.500	55.000	115.5574	57.2390	accB; acetyl-CoA carboxylase, biotin carboxyl carrier protein
					74.000	31.500	105.6123	31.5000	
SGO_1692	1.429	9.656	0.0001	0.0000	196.000	101.000	292.2485	105.1117	3-oxoacyl-[acyl-carrier-protein] synthase
					207.500	113.500	296.1426	113.5000	
SGO_1693	0.871	7.143	0.0014	0.0026	32.500	26.500	48.4596	27.5788	fabG; 3-oxoacyl-(acyl-carrier-protein) reductase
					30.000	22.500	42.8158	22.5000	
SGO_1694	1.047	7.221	0.0027	0.0064	30.000	20.000	44.7319	20.8142	fabD; malonyl CoA-acyl carrier protein transacylase
					39.000	28.000	55.6605	28.0000	
SGO_1695	0.870	9.770	0.0028	0.0067	212.000	147.500	316.1055	153.5047	enoyl-acyl carrier protein(ACP) reductase
					175.000	154.000	249.7588	154.0000	
SGO_1698	-0.756	5.106	0.0515	0.2316	5.000	5.000	7.4553	5.2035	3-oxoacyl-[acyl-carrier-protein] synthase III
					3.000	17.500	4.2816	17.5000	
SGO_1699	0.913	5.685	0.0055	0.0160	13.000	7.500	19.3838	7.8053	transcriptional regulator, MarR family
					10.000	10.000	14.2719	10.0000	
SGO_1700	0.135	6.207	0.0097	0.0318	13.500	17.000	20.1294	17.6921	enoyl-CoA hydratase/isomerase family protein
					13.000	17.500	18.5535	17.5000	
SGO_1701	0.897	7.802	0.0001	0.0000	49.000	36.500	73.0621	37.9859	aspartate kinase
					50.500	40.000	72.0733	40.0000	
SGO_1708	-1.595	9.842	0.0073	0.0220	70.500	424.000	105.1200	441.2609	amiF; Oligopeptide transport ATP-binding protein amiF
					82.000	254.500	117.0299	254.5000	

Log$_2$ Ratios: -6 -4 -2 0 2 4 6

Show detected proteins only
Show all proteins
Filter by category:
ABC Transporter

Proteins found: 1179

Test	Cutoff
q-Value	.005
p-Value	

	Signif	Direction	Applies To
red	yes	+	ratios, bars
yellow	no	n/a	bars
green	yes	−	ratios, bars
pink	yes	+	p- , q-Values
light green	yes	−	p- , q-Values

Dot Plots Dot Plots

Hendrickson *et al.*

Protein	SgPg vs Sg Log2 Ratio	Log2 Sum	q-Value	p-Value	Raw SgPg	Raw Sg	Normalized SgPg	Normalized Sg	Description
SGO_1709	-1.584	10.055	0.0001	0.0000	83.500	381.500	124.5038	397.0307	amiE; Oligopeptide transport ATP-binding protein
					99.500	400.000	142.0057	400.0000	
SGO_1710	-2.268	6.883	0.0003	0.0002	8.000	49.500	11.9285	51.5151	amiD; Oligopeptide transport system permease protein
					6.000	46.000	8.5632	46.0000	
SGO_1711	-1.467	8.602	0.0014	0.0026	33.500	121.000	49.9506	125.9259	hppB; Oligopeptide transport system permease
					37.000	160.000	52.8062	160.0000	
SGO_1712	-1.011	11.090	0.0001	0.0000	243.000	712.500	362.3285	741.5056	hppA; oligopeptide-binding lipoprotein
					252.500	715.500	360.3663	715.5000	
SGO_1713	-1.386	10.708	0.0004	0.0004	160.000	615.000	238.5702	640.0364	hppG; oligopeptide-binding lipoprotein
					157.000	570.500	224.0694	570.5000	
SGO_1715	-1.406	9.535	0.0001	0.0000	66.000	263.500	98.4102	274.2270	hppH; oligopeptide-binding lipoprotein
					73.500	264.500	104.8987	264.5000	
SGO_1716	-0.939	9.553	0.0017	0.0034	90.000	215.000	134.1957	223.7526	oligopeptide binding protein
					86.000	270.500	122.7386	270.5000	
SGO_1717	-3.837	6.483	0.0278	0.1132	2.000	48.500	2.9821	50.4744	pbp3; penicillin-binding protein 3
						36.000		36.0000	
SGO_1718	0.192	7.395	0.0742	0.3537	31.500	31.000	46.9685	32.2620	sufB-1; FeS assembly protein SufB
					29.500	47.000	42.1022	47.0000	
SGO_1719		2.946				5.000		5.2035	SUF system FeS assembly protein, NifU family
						2.500		2.5000	
SGO_1720	1.646	5.980	0.0008	0.0011	15.500	5.500	23.1115	5.7239	aminotransferase, class-V
					17.000	10.000	24.2623	10.0000	
SGO_1721	0.589	7.430	0.0033	0.0083	32.500	36.500	48.4596	37.9859	sufD; FeS assembly protein SufD
					38.500	31.000	54.9469	31.0000	

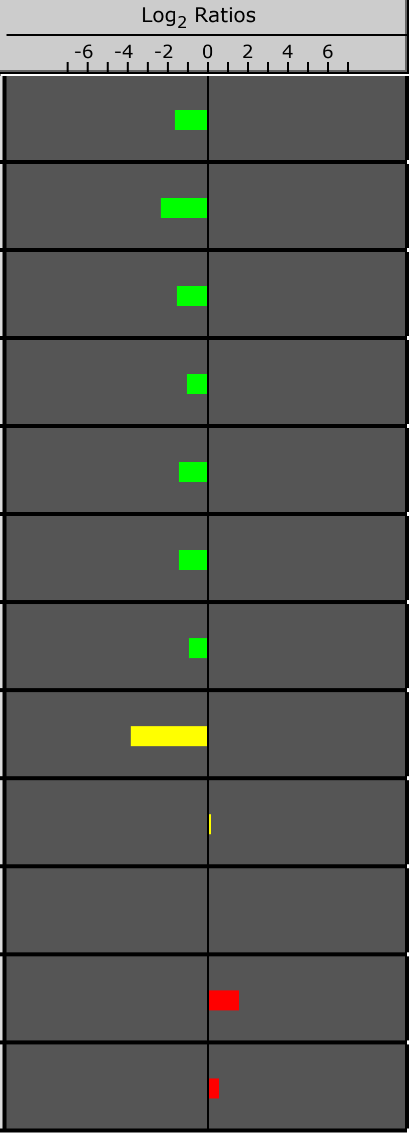

Show detected proteins only
Show all proteins
Filter by category:
ABC Transporter

Proteins found: 1179

Test	Cutoff
q-Value	.005
p-Value	

Color	Signif	Direction	Applies To
red	yes	+	ratios, bars
yellow	no	n/a	bars
green	yes	−	ratios, bars
pink	yes	+	p- , q-Values
light green	yes	−	p- , q-Values

Dot Plots Dot Plots

Protein	SgPg vs Sg Log_2 Ratio	Log_2 Sum	*q*-Value	*p*-Value	Raw *SgPg*	Raw *Sg*	Normalized *SgPg*	Normalized *Sg*	Description
SGO_1722	0.437	6.734	0.0079	0.0243	19.000	19.500	28.3302	20.2938	sufC; FeS assembly ATPase SufC
					23.000	25.000	32.8254	25.0000	
SGO_1724	-0.127	3.848	0.1462	0.7567		3.000		3.1221	mecA; Adapter protein mec
					3.000	7.000	4.2816	7.0000	
SGO_1725		4.038				10.500		10.9275	bacA; bacitracin resistance protein
						5.500		5.5000	
SGO_1726		4.724				10.500		10.9275	hypothetical protein SGO_1726
						15.500		15.5000	
SGO_1727	-0.949	7.719	0.0005	0.0007	22.000	64.500	32.8034	67.1258	amino acid ABC transporter, amino acid-binding/permease protein
					27.500	71.500	39.2478	71.5000	
SGO_1728	-1.549	7.411	0.0042	0.0114	17.000	73.000	25.3481	75.9718	glnQ; glutamine ABC transporter ATP-binding protein
					12.500	51.000	17.8399	51.0000	
SGO_1729		6.111			20.500		30.5668		hypothetical protein SGO_1729
					27.000		38.5342		
SGO_1730	-1.409	9.596	0.0011	0.0019	60.500	292.500	90.2094	304.4076	SPFH domain/Band 7 family
					86.000	256.500	122.7386	256.5000	
SGO_1731	2.963	5.857	0.0014	0.0026	14.500	2.000	21.6204	2.0814	DNA-binding response regulator
					20.500	5.000	29.2575	5.0000	
SGO_1732	0.137	5.243	0.1748	0.9291		6.000		6.2443	histidine kinase
					8.500	19.500	12.1311	19.5000	
SGO_1735	0.067	9.482	0.0791	0.3811	123.500	155.500	184.1464	161.8303	hypothetical protein SGO_1735
					127.000	188.000	181.2536	188.0000	
SGO_1736	0.919	6.090	0.0042	0.0115	17.000	12.500	25.3481	13.0089	alkaline shock protein
					13.500	10.500	19.2671	10.5000	

Log₂ Ratios: -6 -4 -2 0 2 4 6

Show detected proteins only
Show all proteins
Filter by category: ABC Transporter

Proteins found: 1179

Test	Cutoff
q-Value	.005
p-Value	

Color	Signif	Direction	Applies To
red	yes	+	ratios, bars
yellow	no	n/a	bars
green	yes	–	ratios, bars
pink	yes	+	*p*- , *q*-Values
light green	yes	–	*p*- , *q*-Values

Dot Plots Dot Plots

Protein	*SgPg vs Sg* Log$_2$ Ratio	Log$_2$ Sum	*q*-Value	*p*-Value	Raw *SgPg*	Raw *Sg*	Normalized *SgPg*	Normalized *Sg*	Description	Log$_2$ Ratios (-6 -4 -2 0 2 4 6)
SGO_1737		5.867				21.500		22.3753	rpmB; ribosomal protein L28	
						36.000		36.0000		
SGO_1738		4.320				11.500		11.9682	hypothetical protein SGO_1738	
						8.000		8.0000		
SGO_1739		4.076				9.000		9.3664	LytTr DNA-binding domain family	
						7.500		7.5000		
SGO_1744		4.350				9.500		9.8867	ABC transporter, ATP-binding protein SP0636	
						10.500		10.5000		
SGO_1745	1.138	12.225	0.0005	0.0006	1075.000	810.500	1602.8936	843.4951	fba; fructose-1,6-bisphosphate aldolase, class II	
					1177.500	659.500	1680.5202	659.5000		
SGO_1747		2.553			2.500		3.7277		hypothetical protein SGO_1747	
					1.500		2.1408			
SGO_1748	0.671	5.923	0.0190	0.0733	9.500	11.500	14.1651	11.9682	pyrG; CTP synthase	
					16.500	11.000	23.5487	11.0000		
SGO_1749	0.588	6.953	0.0186	0.0713	21.000	19.000	31.3123	19.7735	manA; mannose-6-phosphate isomerase, class I	
					30.000	30.000	42.8158	30.0000		
SGO_1750		5.518				20.000		20.8142	ABC-type multidrug/protein/lipid transport system, ATPase component	
						25.000		25.0000		
SGO_1751	-2.262	6.233	0.0014	0.0025	2.500	31.500	3.7277	32.7824	ABC-type multidrug/protein/lipid transport system, ATPase component	
					7.500	28.000	10.7040	28.0000		
SGO_1753		2.643				6.000		6.2443	hypothetical protein SGO_1753	
SGO_1755	0.917	7.427	0.0037	0.0097	42.500	25.500	63.3702	26.5381	scrK; fructokinase	
					34.500	33.000	49.2382	33.0000		

Protein	SgPg vs Sg Log$_2$ Ratio	Log$_2$ Sum	*q*-Value	*p*-Value	Raw *SgPg*	Raw *Sg*	Normalized *SgPg*	Normalized *Sg*	Description
SGO_1756		2.170							hypothetical protein SGO_1756
						4.500		4.5000	
SGO_1757	0.303	8.837	0.0214	0.0836	82.000	84.500	122.2672	87.9400	glmS; glucosamine--fructose-6-phosphate aminotransferase
					90.500	118.000	129.1610	118.0000	
SGO_1760		2.098							DNA-binding response regulator
					3.000		4.2816		
SGO_1761		4.631				7.000		7.2850	histidine kinase
						17.500		17.5000	
SGO_1763	0.005	7.920	0.1768	0.9410	36.000	53.000	53.6783	55.1576	ABC transporter, substrate-binding protein SP0092
					47.500	65.500	67.7917	65.5000	
SGO_1768	0.808	4.468	0.0490	0.2172	2.500	5.000	3.7277	5.2035	glycosyl hydrolase, family 38
					7.500	2.500	10.7040	2.5000	
SGO_1772		3.858							integral membrane protein
						14.500		14.5000	
SGO_1774	0.032	5.695	0.1520	0.7951	10.500	11.000	15.6562	11.4478	alcohol dehydrogenase, zinc-containing
					7.500	14.000	10.7040	14.0000	
SGO_1780		1.322							hypothetical protein SGO_1780
						2.500		2.5000	
SGO_1784	0.586	8.378	0.0027	0.0063	70.000	57.500	104.3745	59.8408	leuS; leucyl-tRNA synthetase
					66.500	73.500	94.9084	73.5000	
SGO_1792		1.585							transcription regulator
						3.000		3.0000	
SGO_1799	1.049	8.530	0.0008	0.0012	80.500	50.500	120.0306	52.5558	endopeptidase O
					90.000	68.500	128.4474	68.5000	

Log$_2$ Ratios: -6 -4 -2 0 2 4 6

Show detected proteins only
Show all proteins
Filter by category:
ABC Transporter

Proteins found: 1179

Test	Cutoff
q-Value	.005
p-Value	

	Signif	Direction	Applies To
red	yes	+	ratios, bars
yellow	no	n/a	bars
green	yes	–	ratios, bars
pink	yes	+	*p*-, *q*-Values
light green	yes	–	*p*-, *q*-Values

Dot Plots Dot Plots

Protein	SgPg vs Sg Log_2 Ratio	Log_2 Sum	*q*-Value	*p*-Value	Raw *SgPg*	Raw *Sg*	Normalized *SgPg*	Normalized *Sg*	Description
SGO_1800	-2.160	8.427	0.0005	0.0007	22.000	147.500	32.8034	153.5047	troB; manganese ABC transporter, ATP-binding protein SP1648
					21.000	128.000	29.9711	128.0000	
SGO_1802	-2.784	11.227	0.0004	0.0004	119.500	1088.500	178.1821	1132.8124	Metal ABC transporter substrate-binding lipoprotein precursor
					90.000	958.000	128.4474	958.0000	
SGO_1803	1.645	6.828	0.0001	0.0000	29.500	13.000	43.9864	13.5292	tpx; thioredoxin peroxidase
					29.500	14.000	42.1022	14.0000	
SGO_1804	2.632	5.330	0.0096	0.0315	13.000		19.3838		hutI; imidazolonepropionase
					12.500	3.000	17.8399	3.0000	
SGO_1805	4.930	6.617	0.0277	0.1125	38.000	1.500	56.6604	1.5611	hutU; urocanate hydratase
					28.000		39.9614		
SGO_1806		5.242			11.500		17.1472		ftcD; glutamate formiminotransferase
					14.500		20.6943		
SGO_1807		4.350			6.500		9.6919		serine cycle enzyme, putative
					7.500		10.7040		
SGO_1808		3.454			4.000		5.9643		fhs-2; formate--tetrahydrofolate ligase
					3.500		4.9952		
SGO_1811	1.223	5.013	0.0001	0.0000	7.500	4.500	11.1830	4.6832	hutH; histidine ammonia-lyase
					8.000	5.000	11.4175	5.0000	
SGO_1813		2.000							hutG; formimidoylglutamase
						4.000		4.0000	
SGO_1814		3.940				8.500		8.8460	putative regulatory protein
						6.500		6.5000	
SGO_1816	0.145	4.910	0.0440	0.1916		9.000		9.3664	scaR; ScaR Manganese-dependent regulator of scaCBA
					7.500	10.000	10.7040	10.0000	

Log_2 Ratios: -6 -4 -2 0 2 4 6

Show detected proteins only
Show all proteins
Filter by category: ABC Transporter

Proteins found: 1179

Test	Cutoff
q-Value	.005
p-Value	

	Signif	Direction	Applies To
red	yes	+	ratios, bars
yellow	no	n/a	bars
green	yes	–	ratios, bars
pink	yes	+	*p*-, *q*-Values
light green	yes	–	*p*-, *q*-Values

Dot Plots Dot Plots

Hendrickson *et al.*

Protein	*SgPg vs Sg*				Raw		Normalized		Description	Log_2 Ratios
	Log_2 Ratio	Log_2 Sum	*q*-Value	*p*-Value	*SgPg*	*Sg*	*SgPg*	*Sg*		-6 -4 -2 0 2 4 6
SGO_1822	-1.768	6.687	0.0004	0.0004	8.000	41.000	11.9285	42.6691	relA; GTP diphosphokinase	
					8.000	37.000	11.4175	37.0000		
SGO_1823		3.722			5.500		8.2009		conserved hypothetical protein TIGR00046	
					3.500		4.9952			
SGO_1824	0.820	6.059	0.0028	0.0069	14.000	9.500	20.8749	9.8867	prmA; ribosomal protein L11 methyltransferase	
					15.000	14.500	21.4079	14.5000		
SGO_1827	-0.256	4.992	0.0635	0.2934	4.000	7.000	5.9643	7.2850	hypothetical protein SGO_1827	
					6.000	10.000	8.5632	10.0000		
SGO_1828	0.056	4.482	0.1520	0.7952	2.500	6.500	3.7277	6.7646	ATPase, AAA family	
					5.500	4.000	7.8496	4.0000		
SGO_1834	2.739	6.583	0.0043	0.0117	35.500	7.000	52.9328	7.2850	hypothetical protein SGO_1834	
					21.500	5.000	30.6847	5.0000		
SGO_1835	0.863	3.800	0.0170	0.0631	3.500		5.2187		hypothetical protein SGO_1835	
					4.000	3.000	5.7088	3.0000		
SGO_1843	0.981	8.067	0.0026	0.0060	67.000	45.500	99.9013	47.3523	pepS; aminopeptidase PepS	
					55.000	42.500	78.4956	42.5000		
SGO_1844	0.775	5.772	0.0050	0.0141	12.500	8.000	18.6383	8.3257	cbxX/cfqX family protein	
					11.000	12.000	15.6991	12.0000		
SGO_1847	0.057	6.008	0.1039	0.5132	10.500	14.000	15.6562	14.5699	polC; DNA polymerase III, alpha subunit, Gram-positive type	
					12.000	17.000	17.1263	17.0000		
SGO_1848	-3.095	7.448	0.0004	0.0005	6.500	82.000	9.6919	85.3382	lipoprotein, putative	
					6.000	71.000	8.5632	71.0000		
SGO_1849		7.582				87.500		91.0621	hypothetical protein SGO_1849	
						100.500		100.5000		

Show detected proteins only
Show all proteins
Filter by category:
ABC Transporter

Proteins found:
1179

Test	Cutoff
***q*-Value**	**.005**
p-Value	

Color	Signif	Direction	Applies To
red	yes	+	ratios, bars
yellow	no	n/a	bars
green	yes	–	ratios, bars
pink	yes	+	*p*- , *q*-Values
light green	yes	–	*p*- , *q*-Values

Dot Plots Dot Plots

Hendrickson *et al.*

Protein	SgPg vs Sg Log_2 Ratio	Log_2 Sum	*q*-Value	*p*-Value	Raw *SgPg*	Raw *Sg*	Normalized *SgPg*	Normalized *Sg*	Description
SGO_1851	0.365	8.961	0.0066	0.0196	99.500	96.500	148.3608	100.4285	proS; prolyl-tRNA synthetase
					92.500	117.500	132.0154	117.5000	
SGO_1852	-1.841	6.734	0.0003	0.0003	9.000	38.000	13.4196	39.5470	membrane-associated zinc metalloprotease, putative
					7.000	43.500	9.9904	43.5000	
SGO_1854	-1.307	7.017	0.0015	0.0028	16.000	45.500	23.8570	47.3523	uppS; undecaprenyl diphosphate synthase
					10.000	44.000	14.2719	44.0000	
SGO_1855	-1.217	8.163	0.0022	0.0049	35.000	86.500	52.1872	90.0214	preprotein translocase, YajC subunit
					24.500	109.500	34.9662	109.5000	
SGO_1856	-0.896	6.760	0.0005	0.0007	14.000	33.500	20.8749	34.8638	ATP-dependent proteinase ATP-binding chain
					12.000	35.500	17.1263	35.5000	
SGO_1857	-2.113	4.877	0.0396	0.1700	2.000	10.000	2.9821	10.4071	PTS system, IIABC components
						16.000		16.0000	
SGO_1858	1.448	3.696	0.0223	0.0881	4.000		5.9643		beta-fructofuranosidase/sucrose 6 phoshate hydrolase
					3.500	2.000	4.9952	2.0000	
SGO_1860	-1.332	9.034	0.0020	0.0044	53.000	204.500	79.0264	212.8251	5'-nucleotidase, lipoprotein e(P4) family
					48.500	163.000	69.2189	163.0000	
SGO_1861	2.171	5.189	0.0108	0.0360	11.500	3.500	17.1472	3.6425	nusB; transcription antitermination factor NusB
					11.000		15.6991		
SGO_1862	0.941	6.840	0.0011	0.0017	26.500	21.000	39.5132	21.8549	alkaline shock protein
					25.000	17.500	35.6798	17.5000	
SGO_1863	0.394	8.526	0.0017	0.0034	69.000	72.000	102.8834	74.9311	efp; Elongation factor P (EF-P)
					74.500	84.500	106.3259	84.5000	
SGO_1864	0.586	7.241	0.0040	0.0105	30.000	33.500	44.7319	34.8638	X-Pro aminopeptidase
					32.000	26.000	45.6702	26.0000	

Log2 Ratios: -6 -4 -2 0 2 4 6

Show detected proteins only
Show all proteins
Filter by category:
ABC Transporter

Proteins found: 1179

Test	Cutoff
***q*-Value**	**.005**
p-Value	

	Signif	Direction	Applies To
red	yes	+	ratios, bars
yellow	no	n/a	bars
green	yes	−	ratios, bars
pink	yes	+	*p*- , *q*-Values
light green	yes	−	*p*- , *q*-Values

Dot Plots Dot Plots

Protein	SgPg vs Sg Log_2 Ratio	Log_2 Sum	*q*-Value	*p*-Value	Raw *SgPg*	Raw *Sg*	Normalized *SgPg*	Normalized *Sg*	Description	Log_2 Ratios
SGO_1865	-1.073	7.288	0.0003	0.0003	17.000	48.500	25.3481	50.4744	uvrA; excinuclease ABC, A subunit	
					17.500	55.500	24.9759	55.5000		
SGO_1866		2.384			3.500		5.2187		corA; magnesium and cobalt transporter	
SGO_1867	-0.785	8.025	0.0012	0.0021	31.500	85.000	46.9685	88.4603	hypothetical protein SGO_1867	
					34.000	76.500	48.5246	76.5000		
SGO_1869		6.100				27.000		28.0992	hypothetical protein SGO_1869	
						40.500		40.5000		
SGO_1870	-2.154	6.573	0.0017	0.0036	5.000	31.500	7.4553	32.7824	hypothetical protein SGO_1870	
					7.000	45.000	9.9904	45.0000		
SGO_1871		5.486				20.000		20.8142	lipoprotein, putative	
						24.000		24.0000		
SGO_1872	-2.042	5.394	0.0376	0.1612	3.000	14.500	4.4732	15.0903	hypothetical protein SGO_1872	
						22.500		22.5000		
SGO_1878		3.674				6.500		6.7646	transcriptional regulator, TetR family domain protein	
						6.000		6.0000		
SGO_1879	0.485	7.843	0.0748	0.3573	46.000	28.000	68.5889	29.1399	rpsR; ribosomal protein S18	
					42.000	72.000	59.9421	72.0000		
SGO_1880	1.077	8.471	0.0008	0.0011	76.500	49.000	114.0664	50.9948	ssb-1; single-strand binding protein	
					88.500	63.500	126.3066	63.5000		
SGO_1881	0.807	9.782	0.0045	0.0123	214.000	146.500	319.0876	152.4640	rpsF; ribosomal protein S6	
					170.000	166.000	242.6229	166.0000		
SGO_1882	1.566	6.032	0.0038	0.0099	13.500	6.000	20.1294	6.2443	folE; GTP cyclohydrolase I	
					20.000	10.500	28.5439	10.5000		

Show detected proteins only
Show all proteins
Filter by category:
ABC Transporter

Proteins found: 1179

Test	Cutoff
***q*-Value**	.005
p-Value	

	Signif	Direction	Applies To
red	yes	+	ratios, bars
yellow	no	n/a	bars
green	yes	–	ratios, bars
pink	yes	+	*p*- , *q*-Values
light green	yes	–	*p*- , *q*-Values

Dot Plots Dot Plots

Hendrickson *et al.*

Protein	SgPg vs Sg Log2 Ratio	Log2 Sum	q-Value	p-Value	Raw SgPg	Raw Sg	Normalized SgPg	Normalized Sg	Description
SGO_1884		4.239				9.500		9.8867	hypothetical protein SGO_1884
						9.000		9.0000	
SGO_1885	0.544	9.965	0.0001	0.0000	200.000	198.500	298.2128	206.5809	groL; 60 kDa chaperonin/groEL protein
					206.500	200.000	294.7154	200.0000	
SGO_1886	-0.062	6.280	0.1835	0.9903	17.000	20.000	25.3481	20.8142	groES; chaperonin, 10 kDa
					9.500	18.000	13.5583	18.0000	
SGO_1887		4.785				14.000		14.5699	ABC transporter, ATP-binding protein
						13.000		13.0000	
SGO_1888		3.936				7.500		7.8053	ABC transporter, permease protein
						7.500		7.5000	
SGO_1890		4.754				12.000		12.4885	PTS system, fructose(mannose)-specific IID
						14.500		14.5000	
SGO_1891		5.077				18.500		19.2531	PTS system, IIC component
						14.500		14.5000	
SGO_1892	-0.655	6.579	0.0076	0.0233	11.000	24.500	16.4017	25.4974	PTS system, fructose(mannose)-specific IIB
					14.500	33.000	20.6943	33.0000	
SGO_1894		3.832				6.000		6.2443	sugar-binding cell envelope protein
						8.000		8.0000	
SGO_1895		2.202				2.500		2.6018	response regulator
						2.000		2.0000	
SGO_1897		3.776				5.000		5.2035	rbsB; ribose ABC transporter (ribose-binding component) CAC1453
						8.500		8.5000	
SGO_1898	0.820	7.993	0.0003	0.0002	53.500	42.000	79.7719	43.7098	glutamyl aminopeptidase
					58.000	48.500	82.7772	48.5000	

Log2 Ratios: -6 -4 -2 0 2 4 6

Show detected proteins only
Show all proteins
Filter by category:
ABC Transporter

Proteins found: 1179

Test	Cutoff
q-Value	.005
p-Value	

	Signif	Direction	Applies To
red	yes	+	ratios, bars
yellow	no	n/a	bars
green	yes	−	ratios, bars
pink	yes	+	p- , q-Values
light green	yes	−	p- , q-Values

Dot Plots Dot Plots

Protein	*SgPg vs Sg* Log_2 Ratio	Log_2 Sum	*q*-Value	*p*-Value	Raw *SgPg*	Raw *Sg*	Normalized *SgPg*	Normalized *Sg*	Description	Log_2 Ratios (-6 -4 -2 0 2 4 6)
SGO_1899		2.700							hypothetical protein SGO_1899	
						6.500		6.5000		
SGO_1900	0.545	3.292	0.0127	0.0442	2.500		3.7277		thioredoxin family protein	
					2.500	2.500	3.5680	2.5000		
SGO_1901	1.151	6.682	0.0048	0.0136	28.000	17.000	41.7498	17.6921	tRNA binding domain	
					20.500	14.000	29.2575	14.0000		
SGO_1902	1.319	10.098	0.0015	0.0028	291.000	137.000	433.8996	142.5772	ssb; single-stranded DNA-binding protein	
					244.500	170.500	348.9488	170.5000		
SGO_1903	-0.014	8.113	0.1490	0.7754	44.500	64.000	66.3523	66.6054	ATP-dependent Zn protease	
					50.000	72.500	71.3597	72.5000		
SGO_1907		2.170							sensor protein	
						4.500		4.5000		
SGO_1912	1.890	4.365	0.0007	0.0009	5.500	1.500	8.2009	1.5611	Bifunctional folate synthesis protein	
					5.500	3.000	7.8496	3.0000		
SGO_1914	0.948	3.872	0.0322	0.1338	3.500		5.2187		folP; dihydropteroate synthase	
					4.500	3.000	6.4224	3.0000		
SGO_1916	0.394	8.528	0.0030	0.0074	70.500	70.500	105.1200	73.3700	ackA; acetate kinase	
					73.000	86.500	104.1851	86.5000		
SGO_1917	0.982	5.136	0.0039	0.0104	9.000	6.000	13.4196	6.2443	hypothetical protein SGO_1917	
					7.000	5.500	9.9904	5.5000		
SGO_1923		2.865				7.000		7.2850	comYB; competence protein	
SGO_1924	-2.018	8.576	0.0002	0.0001	21.500	142.000	32.0579	147.7808	comYA; competence protein comYA	
					31.000	157.500	44.2430	157.5000		

Show detected proteins only
Show all proteins
Filter by category:
ABC Transporter

Proteins found: 1179

Test	Cutoff
***q*-Value**	**.005**
p-Value	

	Signif	Direction	Applies To
red	yes	+	ratios, bars
yellow	no	n/a	bars
green	yes	−	ratios, bars
pink	yes	+	***p*- , *q*-Values**
light green	yes	−	***p*- , *q*-Values**

Dot Plots Dot Plots

Hendrickson *et al.*

Streptococcus gordonii

Hackett Laboratory UW

Protein	SgPg vs Sg Log$_2$ Ratio	Log$_2$ Sum	*q*-Value	*p*-Value	Raw *SgPg*	Raw *Sg*	Normalized *SgPg*	Normalized *Sg*	Description
SGO_1926	0.180	10.573	0.0033	0.0081	277.000	333.500	413.0247	347.0767	rpoC; DNA-directed RNA polymerase, beta chain
					277.500	367.000	396.0462	367.0000	
SGO_1927	-0.010	10.434	0.1496	0.7797	228.000	347.000	339.9625	361.1262	rpoB; DNA-directed RNA polymerase, beta subunit
					244.500	333.000	348.9488	333.0000	
SGO_1928	-2.965	7.544	0.0003	0.0003	7.000	73.500	10.4374	76.4922	pbp1b; penicillin-binding protein 1B
					7.500	89.000	10.7040	89.0000	
SGO_1929	1.093	9.384	0.0010	0.0015	164.500	97.000	245.2800	100.9488	tyrS; tyrosyl-tRNA synthetase
					147.000	112.000	209.7974	112.0000	
SGO_1930	1.085	4.520	0.0051	0.0145	5.500	2.500	8.2009	2.6018	rrmA; Ribosomal RNA large subunit methyltransferase A
					5.000	5.000	7.1360	5.0000	
SGO_1931		1.161			1.500		2.2366		lipoprotein, putative
SGO_1934		5.277				19.000		19.7735	copper-translocating P-type ATPase
						19.000		19.0000	
SGO_1935		2.496				3.500		3.6425	negative transcriptional regulator, CopY
						2.000		2.0000	
SGO_1936	-2.189	8.974	0.0001	0.0000	32.000	193.500	47.7140	201.3773	adcA; metal-binding (Mn) permease precursor, lipoprotein
					30.000	211.000	42.8158	211.0000	
SGO_1938	0.029	6.096	0.1771	0.9448	12.500	13.500	18.6383	14.0496	adcC; ATP-binding protein, Mn
					11.000	20.000	15.6991	20.0000	
SGO_1939		2.321							adcR; repressor protein adcR
					3.500		4.9952		
SGO_1957	-2.186	4.917	0.0534	0.2415		9.000		9.3664	hypothetical protein SGO_1957
					2.000	18.000	2.8544	18.0000	

Log$_2$ Ratios: -6 -4 -2 0 2 4 6

Show detected proteins only
Show all proteins
Filter by category:
ABC Transporter

Proteins found: 1179

Test	Cutoff
***q*-Value**	**.005**
p-Value	

	Signif	Direction	Applies To
red	yes	+	ratios, bars
yellow	no	n/a	bars
green	yes	−	ratios, bars
pink	yes	+	*p*- , *q*-Values
light green	yes	−	*p*- , *q*-Values

Dot Plots Dot Plots

Hendrickson *et al.*

Protein	SgPg vs Sg: Log_2 Ratio	Log_2 Sum	*q*-Value	*p*-Value	Raw: SgPg	Raw: Sg	Normalized: SgPg	Normalized: Sg	Description
SGO_1958	2.075	10.247	0.0004	0.0003	350.000	127.000	521.8723	132.1701	rplQ; ribosomal protein L17
					321.500	102.000	458.8427	102.0000	
SGO_1959	0.482	9.402	0.0056	0.0164	125.000	152.000	186.3830	158.1879	rpoA; DNA-directed RNA polymerase, alpha subunit
					145.000	125.000	206.9430	125.0000	
SGO_1960	0.545	8.234	0.0033	0.0080	56.500	64.500	84.2451	67.1258	rpsK; ribosomal protein S11
					66.000	55.500	94.1948	55.5000	
SGO_1961	1.499	9.790	0.0003	0.0003	231.500	115.500	345.1813	120.2020	rpsM; ribosomal protein S13p/S18e
					216.500	111.000	308.9874	111.0000	
SGO_1962		5.444			11.000		16.4017		rpmJ; ribosomal protein L36
					19.000		27.1167		
SGO_1964	0.047	8.784	0.0231	0.0918	75.500	106.500	112.5753	110.8356	adk; Adenylate kinase (ATP-AMP transphosphorylase)
					78.000	106.000	111.3211	106.0000	
SGO_1965	-1.375	6.740	0.0004	0.0005	9.000	39.000	13.4196	40.5877	Preprotein translocase secY subunit
					11.500	36.500	16.4127	36.5000	
SGO_1966	1.359	8.989	0.0068	0.0207	94.000	64.500	140.1600	67.1258	rplO; ribosomal protein L15
					160.000	72.500	228.3509	72.5000	
SGO_1967	1.872	8.702	0.0003	0.0002	116.000	42.000	172.9634	43.7098	50S ribosomal protein L30 -related protein
					108.000	45.500	154.1369	45.5000	
SGO_1968	0.640	10.290	0.0024	0.0053	263.000	264.500	392.1498	275.2677	rpsE; ribosomal protein S5
					258.000	216.000	368.2159	216.0000	
SGO_1969	1.259	10.158	0.0003	0.0003	270.500	182.000	403.3328	189.4092	rplR; ribosomal protein L18
					281.000	149.000	401.0414	149.0000	
SGO_1970	-0.442	10.269	0.0004	0.0004	178.500	349.000	266.1549	363.2077	BL10; 50S ribosomal protein L6
					180.000	347.500	256.8948	347.5000	

Log_2 Ratios: -6 -4 -2 0 2 4 6

Show detected proteins only
Show all proteins
Filter by category:
ABC Transporter

Proteins found: 1179

Test	Cutoff
q-Value	.005
p-Value	

	Signif	Direction	Applies To
red	yes	+	ratios, bars
yellow	no	n/a	bars
green	yes	–	ratios, bars
pink	yes	+	*p*- , *q*-Values
light green	yes	–	*p*- , *q*-Values

Dot Plots Dot Plots

Protein	SgPg vs Sg Log2 Ratio	Log2 Sum	q-Value	p-Value	Raw SgPg	Raw Sg	Normalized SgPg	Normalized Sg	Description
SGO_1971	0.875	10.836	0.0004	0.0005	411.500	326.500	613.5727	339.7917	rpsH; ribosomal protein S8
					398.500	305.500	568.7366	305.5000	
SGO_1972		5.340							rpsN; ribosomal protein S14p/S29e
						40.500		40.5000	
SGO_1973	0.314	9.963	0.0026	0.0060	189.500	202.500	282.5566	210.7437	BL6; 50S ribosomal protein L5
					189.500	234.500	270.4532	234.5000	
SGO_1974	-0.172	8.962	0.0595	0.2735	74.500	147.000	111.0843	152.9843	rplX; ribosomal protein L24
					85.500	112.500	122.0250	112.5000	
SGO_1975	0.740	8.750	0.0003	0.0003	92.500	80.500	137.9234	83.7771	rplN; ribosomal protein L14
					92.000	77.500	131.3018	77.5000	
SGO_1976	2.575	8.892	0.0008	0.0012	121.500	45.500	181.1642	47.3523	BS16; 30S ribosomal protein
					156.000	24.000	222.6422	24.0000	
SGO_1977	0.014	6.513	0.1779	0.9553	13.500	25.000	20.1294	26.0177	rpmC; ribosomal protein L29
					18.000	19.500	25.6895	19.5000	
SGO_1978	1.442	8.829	0.0015	0.0028	110.500	42.500	164.7625	44.2302	rplP; ribosomal protein L16
					114.500	82.500	163.4136	82.5000	
SGO_1979	-0.999	10.334	0.0003	0.0002	148.000	429.000	220.6774	446.4644	rpsC; ribosomal protein S3
					147.000	414.000	209.7974	414.0000	
SGO_1980	0.869	9.533	0.0003	0.0003	164.000	134.000	244.5345	139.4551	rplV; ribosomal protein L22
					164.000	123.000	234.0597	123.0000	
SGO_1981	-0.198	9.418	0.0320	0.1328	100.000	159.000	149.1064	165.4728	rpsS; ribosomal protein S19
					118.500	200.500	169.1224	200.5000	
SGO_1982	0.327	9.069	0.0196	0.0760	107.000	99.500	159.5438	103.5506	rplB; ribosomal protein L2
					97.000	135.500	138.4378	135.5000	

Log2 Ratios: -6 -4 -2 0 2 4 6

Show detected proteins only
Show all proteins
Filter by category:
ABC Transporter

Proteins found: 1179

Test	Cutoff
q-Value	.005
p-Value	

Color	Signif	Direction	Applies To
red	yes	+	ratios, bars
yellow	no	n/a	bars
green	yes	−	ratios, bars
pink	yes	+	p-, q-Values
light green	yes	−	p-, q-Values

Dot Plots Dot Plots

Hendrickson *et al.*

Protein	*SgPg vs Sg* Log$_2$ Ratio	Log$_2$ Sum	*q*-Value	*p*-Value	Raw *SgPg*	Raw *Sg*	Normalized *SgPg*	Normalized *Sg*	Description
SGO_1983	-0.214	8.948	0.0580	0.2650	91.000	130.000	135.6868	135.2923	rplW; ribosomal protein L23
					66.500	128.000	94.9084	128.0000	
SGO_1984	0.821	9.611	0.0017	0.0035	158.000	153.000	235.5881	159.2286	rplD; ribosomal protein L4/L1 family
					184.000	124.500	262.6036	124.5000	
SGO_1985	2.375	10.178	0.0072	0.0217	223.000	80.000	332.5072	83.2568	rplC; ribosomal protein L3
					453.000	96.000	646.5186	96.0000	
SGO_1986	0.450	7.881	0.0131	0.0458	42.500	56.000	63.3702	58.2797	rpsJ; ribosomal protein S10
					50.500	42.000	72.0733	42.0000	
SGO_1988		4.572				7.000		7.2850	hydrolase, haloacid dehalogenase-like family
						16.500		16.5000	
SGO_1989	1.403	9.800	0.0003	0.0002	226.500	109.500	337.7259	113.9577	purA; adenylosuccinate synthetase
					216.500	131.000	308.9874	131.0000	
SGO_1990	1.608	5.146	0.0122	0.0419	6.000	3.000	8.9464	3.1221	glutamate--cysteine ligase, putative/amino acid ligase, putative
					12.500	5.500	17.8399	5.5000	
SGO_1991	-0.388	4.635	0.0674	0.3156	3.000	4.000	4.4732	4.1628	hslO; 33 kDa chaperonin /Heat shock protein 33-like protein
					4.000	10.500	5.7088	10.5000	
SGO_1993	-0.453	7.226	0.0175	0.0657	22.500	35.000	33.5489	36.4248	possible transcriptional regulator
					20.500	50.500	29.2575	50.5000	
SGO_1994		1.380				2.500		2.6018	lipoprotein, putative
SGO_1995		4.654				16.500		17.1717	MutT/nudix family protein
						8.000		8.0000	
SGO_1998	-0.061	7.292	0.1442	0.7455	22.000	41.000	32.8034	42.6691	clpB; ATP-dependent Clp proteinase, ATP-binding chain
					31.000	37.000	44.2430	37.0000	

Log$_2$ Ratios: -6 -4 -2 0 2 4 6

Show detected proteins only
Show all proteins
Filter by category:
ABC Transporter

Proteins found: 1179

Test	Cutoff
q-Value	.005
p-Value	

Color	Signif	Direction	Applies To
red	yes	+	ratios, bars
yellow	no	n/a	bars
green	yes	−	ratios, bars
pink	yes	+	*p*- , *q*-Values
light green	yes	−	*p*- , *q*-Values

Dot Plots Dot Plots

Protein	SgPg vs Sg Log2 Ratio	Log2 Sum	q-Value	p-Value	Raw SgPg	Raw Sg	Normalized SgPg	Normalized Sg	Description
SGO_2000	0.557	11.062	0.0011	0.0018	437.500	443.500	652.3404	461.5547	tsf; translation elongation factor Ts
					434.000	404.500	619.4019	404.5000	
SGO_2001	0.163	10.895	0.0136	0.0480	347.500	455.000	518.1447	473.5229	rpsB; ribosomal protein S2
					341.500	425.500	487.3866	425.5000	
SGO_2004		6.780				46.000		47.8726	LPXTG cell wall surface protein
						62.000		62.0000	
SGO_2005	-4.964	9.595	0.0002	0.0001	7.500	379.000	11.1830	394.4289	LPXTG cell wall surface protein
					9.000	355.000	12.8447	355.0000	
SGO_2006		3.279				5.000		5.2035	thioredoxin signature protein
						4.500		4.5000	
SGO_2007	-0.015	7.182	0.1504	0.7846	25.500	35.500	38.0221	36.9452	nusG; transcription termination/antitermination factor NusG
					24.000	36.000	34.2526	36.0000	
SGO_2008		5.872				26.500		27.5788	preprotein translocase secE component - related protein
						31.000		31.0000	
SGO_2010		6.259				38.500		40.0673	pbp2a; penicillin-binding protein 2A
						36.500		36.5000	
SGO_2013		8.980				248.500		258.6163	putative N-acetylmuramidase/lysin
						246.500		246.5000	
SGO_2015		0.585							possible polysaccharide transport protein
						1.500		1.5000	
SGO_2016		2.170							nucleotide sugar dehydratase, putative
						4.500		4.5000	
SGO_2017	-0.539	3.456			3.000		4.4732		nucleotidyl transferase, putative
						6.500		6.5000	

Log2 Ratios: -6 -4 -2 0 2 4 6

Show detected proteins only
Show all proteins
Filter by category: ABC Transporter

Proteins found: 1179

Test	Cutoff
q-Value	.005
p-Value	

Color	Signif	Direction	Applies To
red	yes	+	ratios, bars
yellow	no	n/a	bars
green	yes	−	ratios, bars
pink	yes	+	p- , q-Values
light green	yes	−	p- , q-Values

Dot Plots Dot Plots

Hendrickson *et al.*

Streptococcus gordonii

Protein	SgPg vs Sg Log_2 Ratio	Log_2 Sum	*q*-Value	*p*-Value	Raw *SgPg*	Raw *Sg*	Normalized *SgPg*	Normalized *Sg*	Description
SGO_2019	-0.881	5.633	0.0003	0.0002	5.500	15.500	8.2009	16.1310	licD; licD3 protein
					6.500	16.000	9.2768	16.0000	
SGO_2020		2.585							glycosyltransferase
						6.000		6.0000	
SGO_2021		5.271				15.000		15.6106	Extracellular polysaccharide glycosyltransferase
						23.000		23.0000	
SGO_2022		2.000							UDP-glucose 4-epimerase BH3715
						4.000		4.0000	
SGO_2023		5.835				27.000		28.0992	galactosyltransferase
						29.000		29.0000	
SGO_2024	-1.523	7.385	0.0014	0.0026	13.500	52.500	20.1294	54.6373	Extracellular polysaccharide biosynthesis
					16.000	69.500	22.8351	69.5000	
SGO_2025	-0.304	6.869	0.0257	0.1036	20.500	30.000	30.5668	31.2213	wze; putative autophosphorylating protein tyrosine kinase
					15.500	33.000	22.1215	33.0000	
SGO_2026	-0.991	6.710	0.0019	0.0042	11.000	30.000	16.4017	31.2213	wzd; polysaccharide export protein, MPA1 family
					13.000	38.500	18.5535	38.5000	
SGO_2027		2.170							wzh; wzh/Putative phosphotyrosine-protein phosphatase
						4.500		4.5000	
SGO_2028	-2.941	6.130	0.0049	0.0138		31.000		32.2620	wzg; transcriptional regulator
					3.000	33.500	4.2816	33.5000	
SGO_2033	-0.854	7.763	0.0003	0.0002	26.500	69.500	39.5132	72.3293	nrdD; ribonucleoside-triphosphate reductase
					26.500	67.500	37.8206	67.5000	
SGO_2034	-1.989	5.567	0.0362	0.1540	3.500	16.500	5.2187	17.1717	hypothetical protein SGO_2034
						25.000		25.0000	

Log_2 Ratios: -6 -4 -2 0 2 4 6

Show detected proteins only
Show all proteins
Filter by category:
ABC Transporter

Proteins found: 1179

Test	Cutoff
q-Value	.005
p-Value	

	Signif	Direction	Applies To
red	yes	+	ratios, bars
yellow	no	n/a	bars
green	yes	−	ratios, bars
pink	yes	+	*p*- , *q*-Values
light green	yes	−	*p*- , *q*-Values

Dot Plots Dot Plots

Protein	SgPg vs Sg Log_2 Ratio	Log_2 Sum	*q*-Value	*p*-Value	Raw SgPg	Raw Sg	Normalized SgPg	Normalized Sg	Description
SGO_2037		5.472				21.500		22.3753	cardiolipin synthase
						22.000		22.0000	
SGO_2039	-1.419	3.789	0.0442	0.1931		4.500		4.6832	dihydrofolate synthase
					1.500	7.000	2.1408	7.0000	
SGO_2041	1.335	4.956	0.0032	0.0078	8.000	3.000	11.9285	3.1221	conserved hypothetical protein TIGR00250
					7.000	6.000	9.9904	6.0000	
SGO_2042	2.054	8.549	0.0006	0.0008	92.500	40.500	137.9234	42.1487	Bacterial protein of unknown function (DUF965) superfamily
					114.500	31.000	163.4136	31.0000	
SGO_2043	-2.954	5.560	0.0127	0.0442		20.000		20.8142	lipoprotein, putative
					2.000	23.500	2.8544	23.5000	
SGO_2045	0.828	10.862	0.0006	0.0008	389.500	294.500	580.7693	306.4890	recA; recA protein
					426.000	365.500	607.9844	365.5000	
SGO_2046	0.321	5.625	0.0568	0.2580	9.500	8.000	14.1651	8.3257	cinA; competence induced protein
					9.000	14.000	12.8447	14.0000	
SGO_2049		1.000							tagI; DNA-3-methyladenine glycosylase I
						2.000		2.0000	
SGO_2050	1.259	4.533	0.0163	0.0602	4.000	5.000	5.9643	5.2035	ruvA; Holliday junction DNA helicase RuvA
					7.000	2.000	9.9904	2.0000	
SGO_2052		3.000							hypothetical protein SGO_2052
						8.000		8.0000	
SGO_2053	-1.164	6.120	0.0053	0.0152	5.500	19.500	8.2009	20.2938	DNA mismatch repair protein hexB
					9.500	27.500	13.5583	27.5000	
SGO_2056	-0.084	6.562	0.1403	0.7223	12.500	18.500	18.6383	19.2531	mutS; DNA mismatch repair protein MutS
					19.000	29.500	27.1167	29.5000	

Log_2 Ratios: -6 -4 -2 0 2 4 6

Show detected proteins only
Show all proteins
Filter by category:
ABC Transporter

Proteins found: 1179

Test	Cutoff
***q*-Value**	**.005**
p-Value	

Color	Signif	Direction	Applies To
red	yes	+	ratios, bars
yellow	no	n/a	bars
green	yes	−	ratios, bars
pink	yes	+	*p*- , *q*-Values
light green	yes	−	*p*- , *q*-Values

Dot Plots Dot Plots

Protein	SgPg vs Sg				Raw		Normalized		Description
	Log_2 Ratio	Log_2 Sum	*q*-Value	*p*-Value	*SgPg*	*Sg*	*SgPg*	*Sg*	
SGO_2057		5.049				15.000		15.6106	argR; arginine repressor
						17.500		17.5000	
SGO_2058	0.451	9.878	0.0008	0.0011	188.000	185.500	280.3200	193.0516	argS; arginyl-tRNA synthetase
					184.500	204.500	263.3172	204.5000	
SGO_2059		2.058				4.000		4.1628	hypothetical protein SGO_2059
SGO_2060	0.996	8.121	0.0014	0.0027	61.500	37.000	91.7004	38.5063	aspS-1; aspartyl-tRNA synthetase
					65.000	55.500	92.7676	55.5000	
SGO_2062	0.686	8.464	0.0046	0.0126	77.500	55.000	115.5574	57.2390	hisS; histidyl-tRNA synthetase
					71.000	79.000	101.3307	79.0000	
SGO_2064	1.647	8.986	0.0005	0.0006	138.000	54.500	205.7668	56.7187	ilvD; dihydroxy-acid dehydratase
					125.000	66.000	178.3992	66.0000	
SGO_2065									rpmF; ribosomal protein L32
SGO_2066	1.393	7.977	0.0019	0.0041	66.500	42.000	99.1557	43.7098	rpmG; ribosomal protein L33
					57.500	27.000	82.0636	27.0000	
SGO_2070	-0.247	6.477	0.0921	0.4498	15.000	12.500	22.3660	13.0089	hypothetical protein SGO_2070
					11.000	38.000	15.6991	38.0000	
SGO_2073									FtsK/SpoIIIE family subfamily, putative
SGO_2080	-0.970	5.772	0.0103	0.0343	4.500	20.000	6.7098	20.8142	transcription activator plcR
					8.500	15.000	12.1311	15.0000	
SGO_2081	-2.513	6.148	0.0099	0.0328		30.000		31.2213	lipoprotein, putative
					4.000	34.000	5.7088	34.0000	

Log_2 Ratios: -6 -4 -2 0 2 4 6

Show detected proteins only
Show all proteins
Filter by category:
ABC Transporter

Proteins found: 1179

Test	Cutoff
q-Value	.005
p-Value	

	Signif	Direction	Applies To
red	yes	+	ratios, bars
yellow	no	n/a	bars
green	yes	–	ratios, bars
pink	yes	+	*p*- , *q*-Values
light green	yes	–	

Dot Plots Dot Plots

Protein	SgPg vs Sg: Log_2 Ratio	Log_2 Sum	*q*-Value	*p*-Value	Raw: *SgPg*	Raw: *Sg*	Normalized: *SgPg*	Normalized: *Sg*	Description
SGO_2082		5.515				18.000		18.7328	lipoprotein, putative
						27.000		27.0000	
SGO_2084	-0.180	4.686	0.0926	0.4531		9.500		9.8867	NAD(P)H dehydrogenase, quinone family
					5.500	8.000	7.8496	8.0000	
SGO_2085	-0.211	8.247	0.0036	0.0090	48.000	75.500	71.5711	78.5736	purB; adenylosuccinate lyase
					48.500	84.500	69.2189	84.5000	
SGO_2086		4.929				11.500		11.9682	hypothetical protein SGO_2086
						18.500		18.5000	
SGO_2094		5.483				17.500		18.2124	general stress protein GSP-781
						26.500		26.5000	
SGO_2096		8.836				210.000		218.5490	comB; transport protein ComB
						238.500		238.5000	
SGO_2097	-5.519	10.002	0.0001	0.0000	7.500	493.500	11.1830	513.5902	comA; ATP-binding Transport protein ComA
					7.500	490.000	10.7040	490.0000	
SGO_2098	0.885	11.230	0.0001	0.0001	519.500	388.500	774.6076	404.3157	rpsD; ribosomal protein S4
					548.500	440.000	782.8156	440.0000	
SGO_2099		3.098							ABC transporter ATP-binding protein
					6.000		8.5632		
SGO_2100	2.646	8.845	0.0002	0.0001	140.500	32.000	209.4945	33.3027	ABC transporter substrate-binding protein
					131.000	30.000	186.9623	30.0000	
SGO_2102		5.180			9.000		13.4196		hypothetical protein SGO_2102
					16.000		22.8351		
SGO_2103		1.585							hydrolase, haloacid dehalogenase-like family
						3.000		3.0000	

Log_2 Ratios: -6 -4 -2 0 2 4 6

Show detected proteins only
Show all proteins
Filter by category:
ABC Transporter

Proteins found: 1179

Test	Cutoff
***q*-Value**	**.005**
p-Value	

	Signif	Direction	Applies To
(red)	yes	+	ratios, bars
(yellow)	no	n/a	bars
(green)	yes	−	ratios, bars
(pink)	yes	+	*p*- , *q*-Values
(light green)	yes	−	*p*- , *q*-Values

Dot Plots Dot Plots

Protein	SgPg vs Sg Log2 Ratio	Log2 Sum	q-Value	p-Value	Raw SgPg	Raw Sg	Normalized SgPg	Normalized Sg	Description
SGO_2104	-1.730	9.606	0.0001	0.0000	55.000	291.000	82.0085	302.8465	srtB; sortase B
					69.500	295.500	99.1899	295.5000	
SGO_2105	-6.934	12.327	0.0003	0.0003	15.000	2230.000	22.3660	2320.7824	abpA; amylase-binding protein AbpA
					13.500	2777.000	19.2671	2777.0000	
SGO_2106	0.037	8.367	0.1120	0.5587	52.000	79.000	77.5353	82.2161	ribose-phosphate diphosphokinase
					63.000	80.500	89.9132	80.5000	
SGO_2107		3.785				7.000		7.2850	general stress protein GSP-781
						6.500		6.5000	
SGO_2108	-2.938	6.651	0.0001	0.0000	4.500	44.000	6.7098	45.7912	mreC; cell shape-determining protein MreC
					3.500	43.000	4.9952	43.0000	
SGO_2133	-1.361	10.272	0.0002	0.0001	116.000	411.000	172.9634	427.7317	Cell division protein ftsH-like protein
					121.500	462.500	173.4040	462.5000	
SGO_2134	-0.013	7.764	0.1588	0.8316	37.500	62.500	55.9149	65.0444	hpt; hypoxanthine phosphoribosyltransferase
					36.000	45.000	51.3790	45.0000	
SGO_2138		2.322							Septum formation initiator family
						5.000		5.0000	
SGO_2139		1.380				2.500		2.6018	S4 RNA-binding domain protein
SGO_2140		3.667				5.000		5.2035	transcription-repair coupling factor
						7.500		7.5000	
SGO_2141		1.000							pth; peptidyl-tRNA hydrolase
						2.000		2.0000	
SGO_2142	0.906	8.309	0.0011	0.0018	73.500	48.500	109.5932	50.4744	GTP-binding protein
					68.000	60.000	97.0492	60.0000	

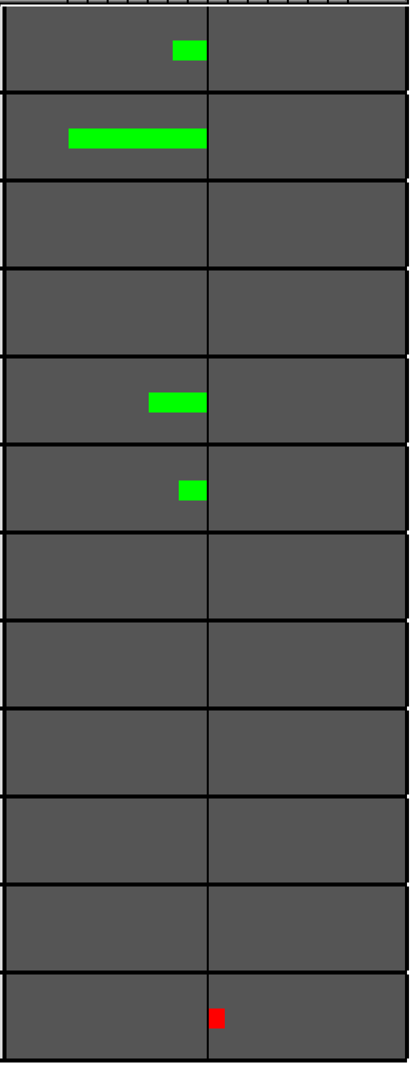

Show detected proteins only
Show all proteins
Filter by category:
ABC Transporter

Proteins found: 1179

Test	Cutoff
q-Value	.005
p-Value	

	Signif	Direction	Applies To
red	yes	+	ratios, bars
yellow	no	n/a	bars
green	yes	−	ratios, bars
pink	yes	+	p- , q-Values
light green	yes	−	p- , q-Values

Dot Plots Dot Plots

Streptococcus gordonii

Hackett Laboratory UW

Protein	SgPg vs Sg				Raw		Normalized		Description	Log_2 Ratios
	Log_2 Ratio	Log_2 Sum	*q*-Value	*p*-Value	*SgPg*	*Sg*	*SgPg*	*Sg*		-6 -4 -2 0 2 4 6
**SGO_2145**	-0.255	9.587	0.0085	0.0270	109.500	208.000	163.2715	216.4676	comE; competence response regulator ComE	
					131.500	201.500	187.6759	201.5000		
**SGO_2146**	-1.707	7.223	0.0003	0.0003	12.500	58.000	18.6383	60.3612	comD; histidine protein kinase ComD	
					11.500	54.000	16.4127	54.0000		
**SGO_2150**	-1.324	8.249	0.0002	0.0001	29.500	108.500	43.9864	112.9170	degP; serine protease	
					30.000	104.500	42.8158	104.5000		

Show detected proteins only
Show all proteins

Filter by category:
ABC Transporter

Proteins found:
1179

Test	Cutoff
***q*-Value**	**.005**
p-Value	

	Signif	Direction	Applies To
red	yes	+	ratios, bars
yellow	no	n/a	bars
green	yes	−	ratios, bars
pink	yes	+	*p*- , *q*-Values
light green	yes	−	*p*- , *q*-Values

Dot Plots Dot Plots

Hendrickson *et al.*
