# Supplementary material for: Proteomics of Streptococcus gordonii within a model developing oral microbial community
Source: BMC Microbiol. 2012 Sep 18;12:211. doi: 10.1186/1471-2180-12-211 (PMC3534352; doi:10.1186/1471-2180-12-211)
Supplement: Additional file 4 — SgPgFn_vs_Sg. A more detailed presentation of the relative abundance ratios for the comparison of SgPgFn and the Sg controls, including both raw and normalized spectral counts. Red and green highlights are used as in Additional file 1. [file 1471-2180-12-211-S4.pdf]

SgPgFn vs Sg

Streptococcus gordonii

Hackett Laboratory UW

Protein	SgPgFn vs Sg Log_2 Ratio	Log_2 Sum	*q*-Value	*p*-Value	Raw *SgPgFn*	Raw *Sg*	Normalized *SgPgFn*	Normalized *Sg*	Description
SGO_0001	-1.455	6.882	0.0038	0.0189	5.000	32.000	13.1600	33.3027	dnaA; chromosomal replication initiator protein DnaA
					6.000	53.500	18.0048	53.5000	
SGO_0002	-0.140	6.846	0.0591	0.5301	9.000	21.000	23.6880	21.8549	dnaN; DNA polymerase III, beta subunit
					10.000	39.500	30.0081	39.5000	
SGO_0004	-2.017	8.695	0.0009	0.0028	15.000	137.000	39.4800	142.5772	putative lipoprotein
					14.000	190.500	42.0113	190.5000	
SGO_0006	0.100	7.626	0.0516	0.4541	22.500	44.000	59.2200	45.7912	ABC transporter, ATP-binding protein
					14.500	49.000	43.5117	49.0000	
SGO_0007	0.785	6.734	0.0005	0.0012	13.000	17.000	34.2160	17.6921	trpS; tryptophanyl-tRNA synthetase
					11.000	21.500	33.0089	21.5000	
SGO_0008	0.961	9.763	0.0004	0.0008	105.500	156.500	277.6762	162.8711	inosine-5'-monophosphate dehydrogenase
					98.500	133.000	295.5796	133.0000	
SGO_0011	-0.825	5.581	0.0082	0.0486	4.500	13.000	11.8440	13.5292	proteinase, M16 family
					2.000	16.500	6.0016	16.5000	
SGO_0015	-2.738	6.428	0.0039	0.0197		39.500		41.1080	ABC transporter (ATP-binding protein)
					2.000	39.000	6.0016	39.0000	
SGO_0022	0.104	5.176	0.0906	0.8591	3.000	6.000	7.8960	6.2443	trmU; tRNA (5-methylaminomethyl-2-thiouridylate)-methyltransferase
					3.500	11.500	10.5028	11.5000	
SGO_0025	0.343	6.230	0.0332	0.2749	5.500	14.500	14.4760	15.0903	gidA; glucose inhibited division protein A
					9.500	17.000	28.5077	17.0000	
SGO_0026	-4.015	7.463	0.0109	0.0718	2.000	91.000	5.2640	94.7046	DHH subfamily 1 protein
						76.500		76.5000	
SGO_0027	0.520	7.410	0.0109	0.0722	15.500	30.500	40.7960	31.7416	rplI; ribosomal protein L9
					20.000	37.500	60.0162	37.5000	

Log_2 Ratios: -6 -4 -2 0 2 4 6

Show detected proteins only
Show all proteins
Filter by category:
ABC Transporter

Proteins found: 624

Test	Cutoff
q-Value	.005
p-Value	

Color	Signif	Direction	Applies To
red	yes	+	ratios, bars
yellow	no	n/a	bars
green	yes	–	ratios, bars
pink	yes	+	*p*- , *q*-Values
light green	yes	–	*p*- , *q*-Values

Dot Plots Dot Plots

Hendrickson *et al.*

Streptococcus gordonii

Hackett Laboratory UW

Protein	*SgPgFn vs Sg* Log$_2$ Ratio	Log$_2$ Sum	*q*-Value	*p*-Value	Raw *SgPgFn*	Raw *Sg*	Normalized *SgPgFn*	Normalized *Sg*	Description	Log$_2$ Ratios (-6 -4 -2 0 2 4 6)
SGO_0028	-0.610	5.208	0.0357	0.2993		11.000		11.4478	dnaC; replicative DNA helicase	
					3.000	16.500	9.0024	16.5000		
SGO_0030	0.689	6.851	0.0018	0.0069	14.500	19.000	38.1640	19.7735	aspB; aspartate transaminase	
					11.000	24.500	33.0089	24.5000		
SGO_0032	-0.245	7.365	0.0180	0.1364	15.500	38.000	40.7960	39.5470	plsX; fatty acid/phospholipid synthesis protein PlsX	
					11.500	50.000	34.5093	50.0000		
SGO_0033	1.403	5.651	0.0085	0.0517	9.500	5.500	25.0040	5.7239	acpP; acyl carrier protein	
					4.000	7.500	12.0032	7.5000		
SGO_0042	0.243	5.283	0.0113	0.0752	5.500	11.500	14.4760	11.9682	transcription regulator, GntR family	
						12.500		12.5000		
SGO_0054	-0.677	7.186	0.0010	0.0031	11.000	40.000	28.9520	41.6284	dltA; D-alanine-activating enzyme	
					9.000	48.000	27.0073	48.0000		
SGO_0057	-2.415	8.108	0.0005	0.0010	8.500	99.500	22.3720	103.5506	dltD protein	
					7.000	129.000	21.0057	129.0000		
SGO_0059	1.992	13.189	0.0006	0.0015	1304.000	1257.500	3432.1300	1308.6923	pXO1; hypothetical protein SGO_0059	
					1313.500	653.500	3941.5609	653.5000		
SGO_0063	-1.618	7.907	0.0000	0.0000	11.000	86.000	28.9520	89.5010	hypothetical protein SGO_0063	
					10.000	91.500	30.0081	91.5000		
SGO_0064	-2.484	8.988	0.0001	0.0000	15.000	214.000	39.4800	222.7119	FtsK/SpoIIIE family protein	
					12.500	208.000	37.5101	208.0000		
SGO_0065	0.159	7.386	0.0007	0.0020	17.000	37.500	44.7440	39.0266	hypothetical protein SGO_0065	
					14.500	40.000	43.5117	40.0000		
SGO_0067	-0.170	6.487	0.0082	0.0489	7.500	23.000	19.7400	23.9363	protein with prophage function domain	
					7.500	23.500	22.5061	23.5000		

- Show detected proteins only
- Show all proteins
- Filter by category: ABC Transporter

Proteins found: 624

Test	Cutoff
q-Value	.005
p-Value	

Color	Signif	Direction	Applies To
red	yes	+	ratios, bars
yellow	no	n/a	bars
green	yes	−	ratios, bars
pink	yes	+	*p*- , *q*-Values
light green	yes	−	*p*- , *q*-Values

Dot Plots Dot Plots

Hendrickson *et al.*

SgPgFn vs Sg

Streptococcus gordonii

Hackett Laboratory UW

Protein	*SgPgFn vs Sg* Log2 Ratio	Log2 Sum	*q*-Value	*p*-Value	Raw *SgPgFn*	Raw *Sg*	Normalized *SgPgFn*	Normalized *Sg*	Description
SGO_0068	-0.815	6.537	0.0004	0.0009	6.500	30.000	17.1080	31.2213	lipoprotein, putative
					5.500	28.000	16.5044	28.0000	
SGO_0069	-1.033	5.980	0.0006	0.0015	4.500	19.500	11.8440	20.2938	hypothetical protein SGO_0069
					3.000	22.000	9.0024	22.0000	
SGO_0070	0.351	6.637	0.0080	0.0470	11.500	19.000	30.2680	19.7735	merozoite surface protein 1
					8.500	24.000	25.5069	24.0000	
SGO_0080	-0.691	7.490	0.0004	0.0007	13.000	51.000	34.2160	53.0762	hypothetical protein SGO_0080
					11.500	58.000	34.5093	58.0000	
SGO_0104	-0.524	9.703	0.0008	0.0024	60.000	234.000	157.9201	243.5261	Maltose/maltodextrin-binding protein precursor
					61.500	247.500	184.5497	247.5000	
SGO_0105	-0.043	5.689	0.0920	0.8746	4.000	14.000	10.5280	14.5699	malQ; 4-alpha-glucanotransferase
					5.000	11.500	15.0040	11.5000	
SGO_0106	1.923	7.794	0.0001	0.0001	33.500	19.000	88.1721	19.7735	glgP-2; maltodextrin phosphorylase
					29.000	27.000	87.0234	27.0000	
SGO_0113	-1.648	13.107	0.0001	0.0001	416.500	3329.000	1096.2286	3464.5223	acdH; alcohol-acetaldehyde dehydrogenase
					345.500	3222.500	1036.7791	3222.5000	
SGO_0135	-1.286	7.825	0.0004	0.0008	10.500	77.500	27.6360	80.6550	v-type sodium ATP synthase, subunit A
					13.000	79.500	39.0105	79.5000	
SGO_0136	-1.600	8.309	0.0005	0.0011	12.500	106.000	32.9000	110.3152	v-type sodium ATP synthase, chain B
					15.500	127.500	46.5125	127.5000	
SGO_0139	-0.511	6.896	0.0090	0.0555	11.500	32.000	30.2680	33.3027	thrC; threonine synthase
					6.500	36.000	19.5052	36.0000	
SGO_0145	-0.848	7.593	0.0004	0.0009	12.000	61.500	31.5840	64.0036	polI; DNA polymerase I
					12.500	60.000	37.5101	60.0000	

Log2 Ratios: -6 -4 -2 0 2 4 6

Show detected proteins only
Show all proteins
Filter by category: ABC Transporter

Proteins found: 624

Test	Cutoff
q-Value	.005
p-Value	

	Signif	Direction	Applies To
red	yes	+	ratios, bars
yellow	no	n/a	bars
green	yes	−	ratios, bars
pink	yes	+	*p*- , *q*-Values
light green	yes	−	*p*- , *q*-Values

Dot Plots | Dot Plots

Hendrickson *et al.*

Protein	SgPgFn vs Sg Log_2 Ratio	Log_2 Sum	*q*-Value	*p*-Value	Raw *SgPgFn*	Raw *Sg*	Normalized *SgPgFn*	Normalized *Sg*	Description
SGO_0146	0.244	5.991	0.0505	0.4436	10.500	11.000	27.6360	11.4478	CoA-binding domain protein
					3.000	15.500	9.0024	15.5000	
SGO_0152	-0.431	5.291	0.0027	0.0119	3.500	10.500	9.2120	10.9275	tgt; queuine tRNA-ribosyltransferase
					2.500	11.500	7.5020	11.5000	
SGO_0154	0.826	10.628	0.0007	0.0018	193.000	309.500	507.9763	322.0996	pgi; glucose-6-phosphate isomerase
					167.000	251.500	501.1349	251.5000	
SGO_0156	-2.949	7.620	0.0003	0.0003	4.000	90.500	10.5280	94.1842	hypothetical protein SGO_0156
					4.000	80.000	12.0032	80.0000	
SGO_0158	1.833	7.756	0.0014	0.0050	37.500	24.500	98.7001	25.4974	2,3,4,5-tetrahydropyridine-2-carboxylate N-succinyltransferase, putative
					23.500	21.500	70.5190	21.5000	
SGO_0163	0.009	7.216	0.1026	0.9909	14.000	32.000	36.8480	33.3027	galU; UTP-glucose-1-phosphate uridylyltransferase
					12.500	41.000	37.5101	41.0000	
SGO_0164	-0.524	7.239	0.0018	0.0071	11.000	40.000	28.9520	41.6284	Glycerol-3-phosphate dehydrogenase [NAD (P)+] (NAD(P)H-dependent glycerol-3-phosphate dehydrogenase)
					11.000	47.500	33.0089	47.5000	
SGO_0169	0.646	5.680	0.0064	0.0364	5.500	7.500	14.4760	7.8053	dut; dUTP diphosphatase
					5.500	12.500	16.5044	12.5000	
SGO_0171	-1.565	5.530	0.0219	0.1728	2.500	15.500	6.5800	16.1310	radA; DNA repair protein RadA
						23.500		23.5000	
SGO_0174	0.684	8.400	0.0008	0.0023	37.000	60.500	97.3841	62.9629	gltX; glutamyl-tRNA synthetase
					37.000	66.500	111.0299	66.5000	
SGO_0180	-1.123	6.647	0.0120	0.0809	3.500	24.000	9.2120	24.9770	jag; hypothetical protein SGO_0180
					8.000	42.000	24.0065	42.0000	
SGO_0190	-1.173	5.410	0.0326	0.2689		12.500		13.0089	hypothetical protein SGO_0190
					2.500	22.000	7.5020	22.0000	

Log2 Ratios: -6 -4 -2 0 2 4 6

Show detected proteins only
Show all proteins
Filter by category:
ABC Transporter

Proteins found: 624

Test	Cutoff
q-Value	.005
p-Value	

Colour	Signif	Direction	Applies To
red	yes	+	ratios, bars
yellow	no	n/a	bars
green	yes	−	ratios, bars
pink	yes	+	*p*- , *q*-Values
light green	yes	−	*p*- , *q*-Values

Dot Plots | Dot Plots

Hendrickson *et al.*

Protein	SgPgFn vs Sg				Raw		Normalized		Description
	Log_2 Ratio	Log_2 Sum	*q*-Value	*p*-Value	*SgPgFn*	*Sg*	*SgPgFn*	*Sg*	
SGO_0198	0.364	6.853	0.0122	0.0827	11.000	27.500	28.9520	28.6195	rpe; ribulose-phosphate 3-epimerase
					12.000	22.000	36.0097	22.0000	
SGO_0200	-1.758	7.706	0.0003	0.0003	7.500	74.500	19.7400	77.5329	competence-induced protein Ccs50
					9.500	83.000	28.5077	83.0000	
SGO_0201	-1.090	7.048	0.0011	0.0034	7.000	47.000	18.4240	48.9134	cmp-binding-factor 1
					8.000	41.000	24.0065	41.0000	
SGO_0204	-0.064	8.092	0.0304	0.2491	25.000	63.500	65.8000	66.0851	rpsL; ribosomal protein S12
					22.500	73.500	67.5182	73.5000	
SGO_0205	0.797	9.499	0.0036	0.0174	75.000	113.000	197.4001	117.6002	rpsG; ribosomal protein S7
					87.500	146.000	262.5707	146.0000	
SGO_0206	1.012	12.576	0.0006	0.0015	837.000	972.500	2202.9853	1012.0901	fusA; translation elongation factor G
					627.500	1007.500	1883.0068	1007.5000	
SGO_0207	1.446	12.724	0.0010	0.0034	830.000	748.500	2184.5613	778.9711	gap; glyceraldehyde-3-phosphate dehydrogenase, type I
					919.500	1043.000	2759.2427	1043.0000	
SGO_0209	1.708	12.819	0.0001	0.0000	1021.000	845.500	2687.2735	879.9200	pgk; phosphoglycerate kinase
					948.000	814.500	2844.7657	814.5000	
SGO_0215	-0.427	8.481	0.0090	0.0555	34.500	95.500	90.8041	99.3878	glnA; glutamine synthetase, type I
					21.000	104.000	63.0170	104.0000	
SGO_0219	-0.152	8.101	0.0183	0.1387	26.000	74.500	68.4320	77.5329	metallo-beta-lactamase superfamily protein 1
					20.500	67.000	61.5166	67.0000	
SGO_0230	0.773	5.967	0.0163	0.1203	5.000	9.000	13.1600	9.3664	Protein of unknown function, DUF536 family
					9.000	13.000	27.0073	13.0000	
SGO_0232	1.561	4.606	0.0173	0.1289	4.500		11.8440		conserved hypothetical protein TIGR00103
					3.000	3.500	9.0024	3.5000	

Log_2 Ratios: -6 -4 -2 0 2 4 6

Show detected proteins only
Show all proteins
Filter by category:
ABC Transporter

Proteins found: 624

Test	Cutoff
q-Value	.005
p-Value	

	Signif	Direction	Applies To
(red)	yes	+	ratios, bars
(yellow)	no	n/a	bars
(green)	yes	−	ratios, bars
(pink)	yes	+	*p*- , *q*-Values
(light green)	yes	−	

Dot Plots Dot Plots

Protein	SgPgFn vs Sg Log$_2$ Ratio	Log$_2$ Sum	*q*-Value	*p*-Value	Raw SgPgFn	Raw Sg	Normalized SgPgFn	Normalized Sg	Description
SGO_0234	-0.741	7.449	0.0004	0.0009	13.500	53.000	35.5320	55.1576	pepX; X-Pro dipeptidyl-peptidase
					10.000	54.000	30.0081	54.0000	
SGO_0237	0.200	5.141	0.0774	0.7214	2.500	5.000	6.5800	5.2035	ccpA; CcpA protein (proteinase)
					4.000	11.500	12.0032	11.5000	
SGO_0244	2.049	4.551	0.0023	0.0095	6.000	3.500	15.7920	3.6425	hydroxymethylglutaryl-CoA synthase
						4.000		4.0000	
SGO_0247	-0.986	11.037	0.0004	0.0007	137.000	709.000	360.5842	737.8631	pfl; formate acetyltransferase
					114.500	659.000	343.5925	659.0000	
SGO_0252	-0.114	4.382	0.0432	0.3739	2.500	6.500	6.5800	6.7646	possible TetR-type transcriptional regulator
						7.500		7.5000	
SGO_0255	-2.151	7.519	0.0013	0.0045	7.000	84.500	18.4240	87.9400	Signal peptidase I
					5.000	62.000	15.0040	62.0000	
SGO_0258	1.585	3.585							hypothetical protein SGO_0258
					3.000	3.000	9.0024	3.0000	
SGO_0260	-1.380	6.661	0.0004	0.0008	4.500	33.500	11.8440	34.8638	DNA mismatch binding protein MutS2
					5.500	38.000	16.5044	38.0000	
SGO_0262	-0.877	6.228	0.0006	0.0017	5.500	24.500	14.4760	25.4974	dipeptidase
					4.000	23.000	12.0032	23.0000	
SGO_0263	1.216	7.973	0.0040	0.0199	41.000	34.000	107.9121	35.3841	trx-1; thioredoxin
					23.000	39.000	69.0186	39.0000	
SGO_0272	0.120	6.586	0.0310	0.2548	10.500	22.500	27.6360	23.4160	hypothetical protein SGO_0272
					7.500	22.500	22.5061	22.5000	
SGO_0276	0.460	9.271	0.0031	0.0143	62.500	132.500	164.5001	137.8940	gdhA; glutamate dehydrogenase (NADP)
					64.500	122.000	193.5521	122.0000	

Log$_2$ Ratios: -6 -4 -2 0 2 4 6

Show detected proteins only
Show all proteins
Filter by category: ABC Transporter

Proteins found: 624

Test	Cutoff
***q*-Value**	**.005**
p-Value	

	Signif	Direction	Applies To
red	yes	+	ratios, bars
yellow	no	n/a	bars
green	yes	−	ratios, bars
pink	yes	+	*p*- , *q*-Values
light green	yes	−	*p*- , *q*-Values

Dot Plots Dot Plots

Hendrickson *et al.*

Protein	SgPgFn vs Sg Log_2 Ratio	Log_2 Sum	*q*-Value	*p*-Value	Raw *SgPgFn*	Raw *Sg*	Normalized *SgPgFn*	Normalized *Sg*	Description
SGO_0286	-1.900	5.668	0.0073	0.0419		20.500		21.3345	DNA mismatch repair protein MutS, putative
					2.000	23.500	6.0016	23.5000	
SGO_0290	-1.342	5.657	0.0373	0.3152	3.000	13.500	7.8960	14.0496	copper -translocating P-type ATPase
						28.500		28.5000	
SGO_0291	-2.286	7.507	0.0004	0.0006	5.000	78.500	13.1600	81.6957	copper-translocating P-type ATPase
					6.000	69.000	18.0048	69.0000	
SGO_0292	1.040	9.282	0.0003	0.0005	84.000	100.000	221.0881	104.0710	spxB; pyruvate oxidase
					66.000	99.500	198.0533	99.5000	
SGO_0312	0.992	11.225	0.0001	0.0000	309.000	387.000	813.2885	402.7546	xfp; D-xylulose 5-phosphate/D-fructose 6-phosphate phosphoketolase
					260.000	398.000	780.2100	398.0000	
SGO_0317	-5.050	8.661	0.0082	0.0481		177.500		184.7260	LPXTG cell wall surface protein, serine protease, subtilase family
					2.000	214.000	6.0016	214.0000	
SGO_0321	0.594	6.384	0.0162	0.1194	8.000	11.500	21.0560	11.9682	polypeptide deformylase
					9.500	22.000	28.5077	22.0000	
SGO_0333	0.039	8.488	0.0801	0.7503	30.000	77.500	78.9600	80.6550	rpsO; ribosomal protein S15
					34.500	96.000	103.5279	96.0000	
SGO_0342	0.291	7.819	0.0031	0.0143	25.000	47.500	65.8000	49.4337	pepF-2; oligoendopeptidase
					19.500	52.000	58.5157	52.0000	
SGO_0344	-0.094	9.053	0.0059	0.0326	48.000	128.500	126.3361	133.7312	pnpA; polyribonucleotide nucleotidyltransferase
					43.500	140.500	130.5351	140.5000	
SGO_0349	0.188	6.710	0.0171	0.1270	11.500	22.000	30.2680	22.8956	cysS; cysteinyl-tRNA synthetase
					8.500	26.000	25.5069	26.0000	
SGO_0352	-0.788	7.816	0.0003	0.0003	16.000	66.000	42.1120	68.6868	ABC transporter, ATP-binding protein SP1580
					13.500	74.000	40.5109	74.0000	

Log_2 Ratios: -6 -4 -2 0 2 4 6

Show detected proteins only
Show all proteins
Filter by category:
ABC Transporter

Proteins found: 624

Test	Cutoff
q-Value	.005
p-Value	

	Signif	Direction	Applies To
red	yes	+	ratios, bars
yellow	no	n/a	bars
green	yes	−	ratios, bars
pink	yes	+	*p*- , *q*-Values
light green	yes	−	*p*- , *q*-Values

Dot Plots Dot Plots

Protein	SgPgFn vs Sg Log_2 Ratio	Log_2 Sum	*q*-Value	*p*-Value	Raw *SgPgFn*	Raw *Sg*	Normalized *SgPgFn*	Normalized *Sg*	Description
SGO_0357	1.313	7.388	0.0012	0.0041	21.000	17.500	55.2720	18.2124	degV; DegV family fatty acid binding protein
					21.000	31.000	63.0170	31.0000	
SGO_0358	0.343	9.490	0.0083	0.0498	73.000	133.000	192.1361	138.4144	rplM; ribosomal protein L13
					69.500	180.000	208.5561	180.0000	
SGO_0359	0.022	8.546	0.0981	0.9422	32.500	76.000	85.5400	79.0939	rpsI; ribosomal protein S9
					34.000	107.000	102.0275	107.0000	
SGO_0368	1.075	3.371	0.0148	0.1064	2.000	2.000	5.2640	2.0814	merA; mercury(II) reductase
						3.000		3.0000	
SGO_0372	0.792	5.806	0.0059	0.0319	7.000	13.000	18.4240	13.5292	malate oxidoreductase
					5.500	7.500	16.5044	7.5000	
SGO_0374	-1.123	6.002	0.0022	0.0094	5.000	22.500	13.1600	23.4160	Response regulator of the LytR/AlgR family
					2.500	20.000	7.5020	20.0000	
SGO_0376	-0.926	3.932			2.000		5.2640		ABC transporter ATP-binding protein
						10.000		10.0000	
SGO_0384	-0.243	5.827	0.0418	0.3577	4.500	10.500	11.8440	10.9275	putative carboxylate-amine/thiol ligase
					4.500	20.500	13.5036	20.5000	
SGO_0390	1.196	7.757	0.0003	0.0005	27.000	30.000	71.0640	31.2213	glycerol-3-phosphate dehydrogenase (NAD (P)+)
					26.500	34.500	79.5214	34.5000	
SGO_0392	2.271	4.127			5.500		14.4760		phosphoglycerate mutase
						3.000		3.0000	
SGO_0398	-2.749	6.912	0.0009	0.0028	2.500	58.500	6.5800	60.8815	ABC transporter ATP-binding protein
					3.000	44.000	9.0024	44.0000	
SGO_0401	0.875	7.096	0.0122	0.0835	17.000	14.000	44.7440	14.5699	grpE; co-chaperone GrpE
					13.500	37.000	40.5109	37.0000	

Log_2 Ratios: -6 -4 -2 0 2 4 6

Show detected proteins only
Show all proteins
Filter by category:
ABC Transporter

Proteins found: 624

Test	Cutoff
q-Value	.005
p-Value	

	Signif	Direction	Applies To
red	yes	+	ratios, bars
yellow	no	n/a	bars
green	yes	–	ratios, bars
pink	yes	+	*p*- , *q*-Values
light green	yes	–	*p*- , *q*-Values

Dot Plots Dot Plots

Hendrickson *et al.*

Protein	*SgPgFn vs Sg* Log$_2$ Ratio	Log$_2$ Sum	*q*-Value	*p*-Value	Raw *SgPgFn*	Raw *Sg*	Normalized *SgPgFn*	Normalized *Sg*	Description
SGO_0402	0.487	11.157	0.0007	0.0019	245.000	434.500	644.8404	452.1883	dnaK; DnaK chaperone protein
					229.000	499.000	687.1850	499.0000	
SGO_0404	-2.460	7.910	0.0032	0.0147	5.000	71.000	13.1600	73.8904	dnaJ; DnaJ chaparone protein
					8.000	129.500	24.0065	129.5000	
SGO_0408	-4.983	8.913	0.0027	0.0117		232.000		241.4446	zmpB; zinc metalloproteinase B
					2.500	233.000	7.5020	233.0000	
SGO_0412	1.089	11.280	0.0002	0.0002	328.500	353.500	864.6125	367.8908	tig; trigger factor
					275.000	428.500	825.2221	428.5000	
SGO_0413	1.445	6.540	0.0007	0.0018	12.500	15.500	32.9000	16.1310	DNA-directed RNA polymerase delta chain
					11.500	9.500	34.5093	9.5000	
SGO_0415	-1.184	10.658	0.0003	0.0005	89.500	569.500	235.5641	592.6841	secA; preprotein translocase, SecA subunit
					86.000	529.500	258.0695	529.5000	
SGO_0416	-0.211	6.715	0.0597	0.5367	12.000	24.000	31.5840	24.9770	phospho-2-dehydro-3-deoxyheptonate aldolase
					6.000	30.500	18.0048	30.5000	
SGO_0426	-0.210	5.146	0.0650	0.5907	4.000	9.500	10.5280	9.8867	Cof family protein
						15.000		15.0000	
SGO_0427	1.454	6.180	0.0001	0.0001	10.500	9.500	27.6360	9.8867	universal stress protein family
					8.500	9.500	25.5069	9.5000	
SGO_0429	-0.434	9.126	0.0012	0.0042	43.500	162.000	114.4921	168.5950	aspartate transaminase
					41.000	152.500	123.0331	152.5000	
SGO_0432	0.234	7.019	0.0184	0.1401	14.000	32.500	36.8480	33.8231	entB; isochorismatase family protein
					11.000	26.000	33.0089	26.0000	
SGO_0434	-0.962	7.443	0.0026	0.0113	13.500	49.000	35.5320	50.9948	aspS-2; aspartyl-tRNA synthetase
					8.000	63.500	24.0065	63.5000	

Log$_2$ Ratios: -6 -4 -2 0 2 4 6

Show detected proteins only
Show all proteins
Filter by category:
ABC Transporter

Proteins found: 624

Test	Cutoff
q-Value	.005
p-Value	

	Signif	Direction	Applies To
red	yes	+	ratios, bars
yellow	no	n/a	bars
green	yes	–	ratios, bars
pink	yes	+	*p*- , *q*-Values
light green	yes	–	*p*- , *q*-Values

Dot Plots Dot Plots

Hendrickson *et al.*

Protein	SgPgFn vs Sg Log_2 Ratio	Log_2 Sum	*q*-Value	*p*-Value	Raw *SgPgFn*	Raw *Sg*	Normalized *SgPgFn*	Normalized *Sg*	Description
SGO_0435	0.062	6.359	0.0647	0.5867	8.500	17.500	22.3720	18.2124	gatC; glutamyl-tRNA(Gln) amidotransferase, C subunit
					6.500	22.000	19.5052	22.0000	
SGO_0436	-0.390	9.261	0.0025	0.0108	50.000	179.500	131.6001	186.8074	gatA; glutamyl-tRNA(Gln) amidotransferase, A subunit
					44.500	161.500	133.5359	161.5000	
SGO_0437	0.341	8.903	0.0263	0.2113	65.000	100.000	171.0801	104.0710	gatB; glutamyl-tRNA(Gln) amidotransferase, B subunit
					33.500	103.000	100.5271	103.0000	
SGO_0440	1.671	4.100	0.0035	0.0167	4.000	3.000	10.5280	3.1221	L-iditol 2-dehydrogenase BH3949
						3.500		3.5000	
SGO_0445	-0.388	6.403	0.0100	0.0638	6.000	21.000	15.7920	21.8549	GTP-binding protein
					7.000	26.000	21.0057	26.0000	
SGO_0448	-1.036	4.384	0.0375	0.3180	1.500	10.500	3.9480	10.9275	conserved hypothetical protein TIGR00488
						6.000		6.0000	
SGO_0454	-0.263	7.542	0.0681	0.6242	10.500	54.000	27.6360	56.1983	conserved hypothetical protein TIGR01033
					20.000	42.500	60.0162	42.5000	
SGO_0455	-3.589	8.338	0.0003	0.0004	3.500	155.500	9.2120	161.8303	lipoprotein, putative
					5.500	136.000	16.5044	136.0000	
SGO_0457	-1.713	9.432	0.0001	0.0001	30.000	245.000	78.9600	254.9739	ABC transporter, substrate-binding protein SP0148
					27.500	274.500	82.5222	274.5000	
SGO_0458	-1.927	10.927	0.0003	0.0006	74.500	801.500	196.0841	834.1288	hlpA; lipoprotein
					69.500	708.500	208.5561	708.5000	
SGO_0460	-2.331	7.671	0.0005	0.0012	6.500	91.000	17.1080	94.7046	ABC transporter, ATP-binding protein SP0151
					5.500	75.500	16.5044	75.5000	
SGO_0468	-0.209	6.214	0.0553	0.4919	7.500	12.000	19.7400	12.4885	hypothetical protein SGO_0468
					4.500	28.500	13.5036	28.5000	

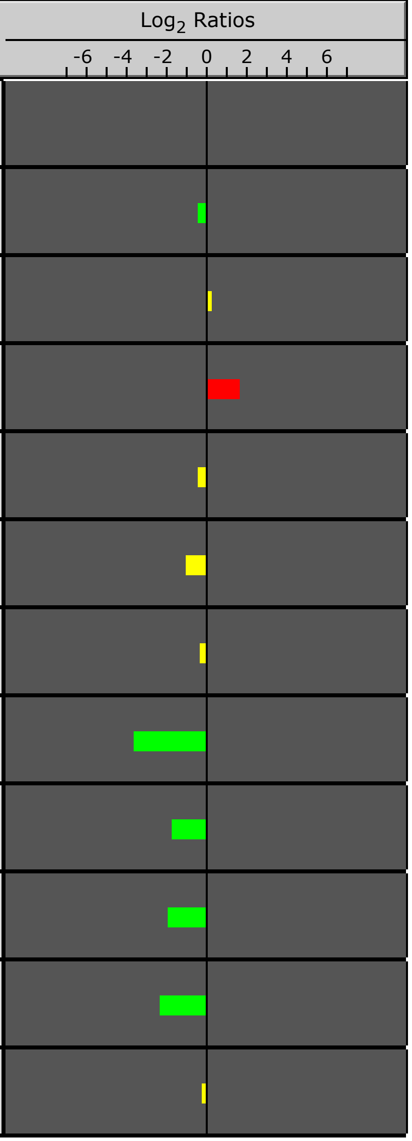

Show detected proteins only
Show all proteins
Filter by category:
ABC Transporter

Proteins found: 624

Test	Cutoff
q-Value	.005
p-Value	

	Signif	Direction	Applies To
red	yes	+	ratios, bars
yellow	no	n/a	bars
green	yes	–	ratios, bars
pink	yes	+	*p*- , *q*-Values
light green	yes	–	*p*- , *q*-Values

Dot Plots Dot Plots

Hendrickson *et al.*

Protein	*SgPgFn vs Sg*				Raw		Normalized		Description
	Log_2 Ratio	Log_2 Sum	*q*-Value	*p*-Value	*SgPgFn*	*Sg*	*SgPgFn*	*Sg*	
SGO_0476	1.297	6.342	0.0002	0.0002	10.500	12.000	27.6360	12.4885	rhodanese family protein
					10.000	11.000	30.0081	11.0000	
SGO_0480	0.737	3.585							hypothetical protein SGO_0480
					2.500	4.500	7.5020	4.5000	
SGO_0483	0.979	6.445	0.0011	0.0035	11.000	17.000	28.9520	17.6921	hypothetical protein SGO_0483
					9.500	12.000	28.5077	12.0000	
SGO_0501	0.967	7.084	0.0000	0.0000	17.000	22.500	44.7440	23.4160	Uncharacterized ACR, COG1399
					15.000	22.500	45.0121	22.5000	
SGO_0502	-1.046	9.757	0.0002	0.0002	52.500	291.000	138.1801	302.8465	floL; flotillin-like protein
					48.000	280.000	144.0388	280.0000	
SGO_0503	1.201	11.183	0.0002	0.0003	322.000	350.000	847.5045	364.2484	gnd; 6-phosphogluconate dehydrogenase, decarboxylating
					257.500	340.000	772.7080	340.0000	
SGO_0505	-3.763	10.957	0.0001	0.0000	24.000	911.000	63.1680	948.0865	PTS system, IIBC component
					24.500	902.500	73.5198	902.5000	
SGO_0508	-0.317	6.498	0.0061	0.0338	8.500	24.500	22.3720	25.4974	nrdR; transcriptional regulator, NrdR family
					6.000	24.500	18.0048	24.5000	
SGO_0510	1.171	5.714	0.0011	0.0038	7.500	6.500	19.7400	6.7646	dnaI; primosomal protein DnaI
					5.500	9.500	16.5044	9.5000	
SGO_0511	0.216	5.138	0.0919	0.8722	4.000	4.500	10.5280	4.6832	NADPH-flavin oxidoreductase -like protein
					2.500	12.500	7.5020	12.5000	
SGO_0512	0.771	8.160	0.0043	0.0222	38.000	61.000	100.0161	63.4833	GTP-binding protein engA
					26.500	43.000	79.5214	43.0000	
SGO_0515	0.490	8.689	0.0047	0.0250	40.500	85.500	106.5961	88.9807	murC; UDP-N-acetylmuramate--alanine ligase
					45.000	82.000	135.0363	82.0000	

Log_2 Ratios: -6 -4 -2 0 2 4 6

Show detected proteins only
Show all proteins
Filter by category:
ABC Transporter

Proteins found: 624

Test	Cutoff
q-Value	.005
p-Value	

	Signif	Direction	Applies To
red	yes	+	ratios, bars
yellow	no	n/a	bars
green	yes	−	ratios, bars
pink	yes	+	*p*- , *q*-Values
light green	yes	−	*p*- , *q*-Values

Dot Plots Dot Plots

Hendrickson *et al.*

Protein	SgPgFn vs Sg Log$_2$ Ratio	Log$_2$ Sum	*q*-Value	*p*-Value	Raw *SgPgFn*	Raw *Sg*	Normalized *SgPgFn*	Normalized *Sg*	Description
SGO_0518	-1.560	8.849	0.0002	0.0001	20.000	170.000	52.6400	176.9206	aminodeoxychorismate lyase-like protein
					21.500	167.000	64.5174	167.0000	
SGO_0519	0.177	7.214	0.0112	0.0744	14.500	31.000	38.1640	32.2620	greA; transcription elongation factor greA
					13.500	37.500	40.5109	37.5000	
SGO_0526	-0.429	5.467	0.0284	0.2290	3.000	8.500	7.8960	8.8460	ilvB; acetolactate synthase, large subunit, biosynthetic type
					3.500	17.000	10.5028	17.0000	
SGO_0527	-1.584	4.978	0.0063	0.0350		12.500		13.0089	ilvN; acetolactate synthase, small subunit
					1.500	14.000	4.5012	14.0000	
SGO_0528	0.292	8.366	0.0128	0.0887	39.000	68.000	102.6481	70.7683	ilvC; ketol-acid reductoisomerase
					26.500	77.000	79.5214	77.0000	
SGO_0529	1.149	3.915	0.0332	0.2748		2.000		2.0814	ilvA; threonine dehydratase
					2.500	5.500	7.5020	5.5000	
SGO_0535	-2.636	6.736	0.0117	0.0784	3.000	42.500	7.8960	44.2302	putative transcriptional regulator LytR
						54.500		54.5000	
SGO_0536	0.019	7.090	0.0847	0.7979	13.500	30.500	35.5320	31.7416	hypothetical protein SGO_0536
					11.000	36.000	33.0089	36.0000	
SGO_0537	0.678	6.200	0.0029	0.0130	8.000	11.500	21.0560	11.9682	HIT family protein
					8.000	16.500	24.0065	16.5000	
SGO_0540	-0.100	7.089	0.0204	0.1589	13.000	35.500	34.2160	36.9452	hypothetical protein SGO_0540
					10.500	33.500	31.5085	33.5000	
SGO_0543	-0.940	8.146	0.0003	0.0004	17.000	87.500	44.7440	91.0621	nusA; transcription termination factor NusA
					17.500	95.000	52.5141	95.0000	
SGO_0546	-0.475	8.700	0.0122	0.0836	27.500	132.500	72.3800	137.8940	infB; Translation initiation factor IF-2
					34.000	103.500	102.0275	103.5000	

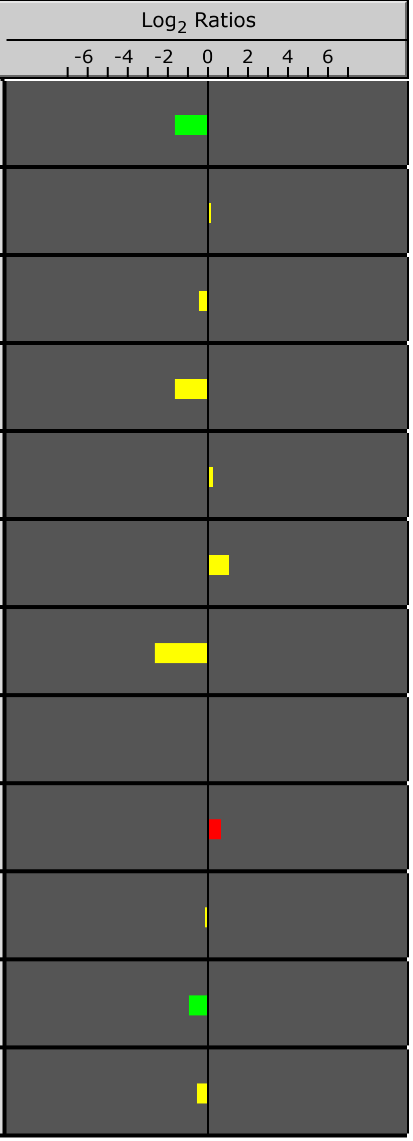

Show detected proteins only
Show all proteins
Filter by category:
ABC Transporter

Proteins found: 624

Test	Cutoff
q-Value	.005
p-Value	

	Signif	Direction	Applies To
red	yes	+	ratios, bars
yellow	no	n/a	bars
green	yes	−	ratios, bars
pink	yes	+	*p*-, *q*-Values
light green	yes	−	

Dot Plots Dot Plots

Hendrickson *et al.*

Streptococcus gordonii

Protein	*SgPgFn* vs *Sg* Log$_2$ Ratio	Log$_2$ Sum	*q*-Value	*p*-Value	Raw *SgPgFn*	Raw *Sg*	Normalized *SgPgFn*	Normalized *Sg*	Description	Log$_2$ Ratios (-6 -4 -2 0 2 4 6)
**SGO_0548**	-3.700	7.756	0.0003	0.0004	3.000	105.000	7.8960	109.2745	Na/Pi-cotransporter family protein	
					2.500	91.500	7.5020	91.5000		
**SGO_0552**	2.390	6.185	0.0004	0.0009	12.000	3.500	31.5840	3.6425	oxidoreductase, aldo/keto reductase family	
					9.500	9.000	28.5077	9.0000		
**SGO_0554**	-1.664	6.199	0.0395	0.3367	3.500	18.000	9.2120	18.7328	hsdR; type I site-specific deoxyribonuclease	
						45.500		45.5000		
**SGO_0558**	0.263	3.460							hypothetical protein SGO_0558	
					2.000	5.000	6.0016	5.0000		
**SGO_0560**	-0.051	5.401	0.0810	0.7600	4.500	10.000	11.8440	10.4071	hsdM; type I restriction-modification system, M subunit	
					3.000	11.000	9.0024	11.0000		
**SGO_0565**	0.010	7.962	0.0921	0.8777	23.000	56.500	60.5360	58.8001	adhA; alcohol dehydrogenase	
					21.500	65.500	64.5174	65.5000		
**SGO_0568**	-0.300	7.076	0.0184	0.1403	9.500	34.500	25.0040	35.9045	glyQ; glycyl-tRNA synthetase, alpha subunit	
					12.000	38.000	36.0097	38.0000		
**SGO_0569**	0.301	8.362	0.0214	0.1686	28.000	70.000	73.6960	72.8497	glyS; glycyl-tRNA synthetase, beta subunit	
					36.500	73.000	109.5295	73.0000		
**SGO_0573**	-1.240	6.233	0.0046	0.0239	3.000	20.500	7.8960	21.3345	mraW; S-adenosyl-methyltransferase MraW	
					5.000	31.000	15.0040	31.0000		
**SGO_0577**	0.118	7.003	0.0712	0.6568	10.000	23.500	26.3200	24.4567	ATP-dependent RNA helicase	
					13.500	37.000	40.5109	37.0000		
**SGO_0581**	-0.136	5.652	0.0353	0.2946	4.500	11.000	11.8440	11.4478	trxB; thioredoxin-disulfide reductase	
					4.000	15.000	12.0032	15.0000		
**SGO_0582**	1.063	7.143	0.0042	0.0212	21.500	18.000	56.5880	18.7328	nicotinate phosphoribosyltransferase, putative	
					13.000	27.000	39.0105	27.0000		

◉ Show detected proteins only
○ Show all proteins
☐ Filter by category:
ABC Transporter

Proteins found: 624

Test	Cutoff
***q*-Value**	**.005**
p-Value	

Color	Signif	Direction	Applies To
red	yes	+	ratios, bars
yellow	no	n/a	bars
green	yes	−	ratios, bars
pink	yes	+	*p*- , *q*-Values
light green	yes	−	*p*- , *q*-Values

Dot Plots | Dot Plots

Hendrickson *et al.*

Protein	*SgPgFn vs Sg* Log_2 Ratio	Log_2 Sum	*q*-Value	*p*-Value	Raw *SgPgFn*	Raw *Sg*	Normalized *SgPgFn*	Normalized *Sg*	Description
SGO_0583	0.732	5.536	0.0130	0.0915	5.000	5.500	13.1600	5.7239	nadE; NAD+ synthetase
					5.000	12.500	15.0040	12.5000	
SGO_0585	0.528	6.916	0.0092	0.0570	15.000	19.500	39.4800	20.2938	pepC; aminopeptidase C
					10.500	29.500	31.5085	29.5000	
SGO_0586	-1.931	8.713	0.0001	0.0001	15.500	154.500	40.7960	160.7896	pbp1a; penicillin-binding protein 1A
					15.500	171.500	46.5125	171.5000	
SGO_0589	0.840	7.241	0.0028	0.0124	21.000	24.000	55.2720	24.9770	methylase
					14.000	29.000	42.0113	29.0000	
SGO_0590	-1.104	5.408	0.0263	0.2109	3.000	13.500	7.8960	14.0496	Methyltransferase
						20.500		20.5000	
SGO_0591	-3.040	8.906	0.0000	0.0000	9.000	208.000	23.6880	216.4676	hypothetical protein SGO_0591
					9.500	211.000	28.5077	211.0000	
SGO_0592	0.243	4.825	0.0276	0.2220	4.000	8.000	10.5280	8.3257	luxS; autoinducer-2 production protein LuxS
						9.500		9.5000	
SGO_0593	-2.992	8.903	0.0001	0.0001	12.000	196.000	31.5840	203.9791	HD/KH domain protein
					7.500	220.500	22.5061	220.5000	
SGO_0594	-0.356	6.147	0.0097	0.0616	5.500	21.500	14.4760	22.3753	gmk; Guanylate kinase (GMP kinase)
					5.500	17.500	16.5044	17.5000	
SGO_0595	-0.733	7.101	0.0004	0.0006	10.500	40.500	27.6360	42.1487	DNA-directed RNA polymerase, omega subunit
					8.000	43.500	24.0065	43.5000	
SGO_0597	0.482	5.192	0.0290	0.2349	4.500	5.000	11.8440	5.2035	fmt; methionyl-tRNA formyltransferase
					3.000	10.500	9.0024	10.5000	
SGO_0599	-0.816	6.954	0.0019	0.0077	7.000	39.000	18.4240	40.5877	phosphoprotein phosphatase
					9.000	38.000	27.0073	38.0000	

Log_2 Ratios: -6 -4 -2 0 2 4 6

Show detected proteins only
Show all proteins
Filter by category:
ABC Transporter

Proteins found: 624

Test	Cutoff
q-Value	.005
p-Value	

	Signif	Direction	Applies To
(red)	yes	+	ratios, bars
(yellow)	no	n/a	bars
(green)	yes	−	ratios, bars
(pink)	yes	+	*p*- , *q*-Values
(light green)	yes	−	*p*- , *q*-Values

Dot Plots Dot Plots

Hendrickson *et al.*

Streptococcus gordonii

Protein	*SgPgFn vs Sg* Log$_2$ Ratio	Log$_2$ Sum	*q*-Value	*p*-Value	Raw *SgPgFn*	Raw *Sg*	Normalized *SgPgFn*	Normalized *Sg*	Description
SGO_0600	-3.243	7.883	0.0037	0.0182	4.500	105.000	11.8440	109.2745	serine/threonine protein kinase
						115.000		115.0000	
SGO_0604	0.298	7.959	0.0102	0.0659	27.500	47.500	72.3800	49.4337	hydrolase, haloacid dehalogenase family/peptidyl-prolyl cis-trans isomerase, cyclophilin type
					21.500	62.500	64.5174	62.5000	
SGO_0606	-0.436	9.248	0.0006	0.0014	48.000	162.000	126.3361	168.5950	cysK; cysteine synthase A
					44.000	181.000	132.0355	181.0000	
SGO_0610	0.525	8.478	0.0148	0.1065	50.000	75.000	131.6001	78.0532	ribosomal subunit interface protein
					27.000	66.000	81.0218	66.0000	
SGO_0626	-1.685	5.612	0.0032	0.0150	2.500	20.000	6.5800	20.8142	recX; Regulatory protein recX
						21.500		21.5000	
SGO_0631	2.433	5.206	0.0084	0.0510	6.000	3.000	15.7920	3.1221	alpha-glycerophosphate oxidase
					6.000		18.0048		
SGO_0639	-0.081	8.001	0.0655	0.5956	27.000	65.500	71.0640	68.1665	valS; valyl-tRNA synthetase
					18.000	63.000	54.0145	63.0000	
SGO_0640	2.061	4.870	0.0095	0.0595		6.000		6.2443	modification methylase
					6.500	3.500	19.5052	3.5000	
SGO_0641	-0.590	6.661	0.0043	0.0221	8.500	32.500	22.3720	33.8231	ATPase, histidine kinase-, DNA gyrase B-, and HSP90-like domain protein protein
					6.000	27.000	18.0048	27.0000	
SGO_0642	1.284	8.338	0.0006	0.0014	46.500	39.500	122.3881	41.1080	hypothetical protein SGO_0642
					35.500	53.500	106.5287	53.5000	
SGO_0643	-0.469	4.323	0.0371	0.3127	2.000	6.000	5.2640	6.2443	cytosine-specific methyltransferase
						8.500		8.5000	
SGO_0644	0.731	6.424	0.0024	0.0102	9.000	16.000	23.6880	16.6514	hypothetical protein SGO_0644
					10.000	15.500	30.0081	15.5000	

Log$_2$ Ratios: -6 -4 -2 0 2 4 6

Show detected proteins only
Show all proteins
Filter by category:
ABC Transporter

Proteins found: 624

Test	Cutoff
q-Value	.005
p-Value	

	Signif	Direction	Applies To
red	yes	+	ratios, bars
yellow	no	n/a	bars
green	yes	−	ratios, bars
pink	yes	+	*p*- , *q*-Values
light green	yes	−	*p*- , *q*-Values

Dot Plots Dot Plots

Hendrickson *et al.*

Protein	*SgPgFn* vs *Sg* Log2 Ratio	Log2 Sum	*q*-Value	*p*-Value	Raw *SgPgFn*	Raw *Sg*	Normalized *SgPgFn*	Normalized *Sg*	Description
SGO_0652	-3.269	7.309	0.0001	0.0000	3.500	71.000	9.2120	73.8904	hypothetical protein SGO_0652
					2.000	69.500	6.0016	69.5000	
SGO_0654	-1.356	7.923	0.0011	0.0036	14.500	74.000	38.1640	77.0125	radical SAM enzyme, Cfr family
					10.000	97.500	30.0081	97.5000	
SGO_0656	-0.917	5.799	0.0092	0.0574	5.500	17.000	14.4760	17.6921	trpB-2; tryptophan synthase, beta subunit
					2.000	17.500	6.0016	17.5000	
SGO_0665	-0.506	10.265	0.0144	0.1037	115.500	405.500	303.9962	422.0077	non-heme iron-containing ferritin
					68.500	298.500	205.5553	298.5000	
SGO_0669	0.640	8.090	0.0006	0.0015	30.000	49.000	78.9600	50.9948	typA; GTP-binding protein TypA
					29.000	55.500	87.0234	55.5000	
SGO_0671	-0.744	6.613	0.0005	0.0010	6.500	30.500	17.1080	31.7416	murD; UDP-N-acetylmuramoylalanine--D-glutamate ligase
					6.500	29.500	19.5052	29.5000	
SGO_0672	-3.241	6.316	0.0161	0.1181	1.500	42.500	3.9480	44.2302	murG; undecaprenyl-PP-MurNAc-pentapeptide-UDPGlcNAc GlcNAc transferase
						31.500		31.5000	
SGO_0673	-2.920	6.420	0.0136	0.0962	2.000	33.500	5.2640	34.8638	DivIB; cell division protein DivIB
						45.500		45.5000	
SGO_0674	0.429	9.145	0.0011	0.0037	58.500	114.000	153.9721	118.6409	ftsA; cell division protein FtsA
					57.000	122.500	171.0460	122.5000	
SGO_0675	0.860	9.992	0.0013	0.0044	121.000	145.500	318.4722	151.4232	ftsZ; cell division protein FtsZ
					111.500	213.500	334.5901	213.5000	
SGO_0676	-0.086	5.997	0.0553	0.4930	6.000	13.500	15.7920	14.0496	conserved hypothetical protein TIGR00044
					5.000	19.000	15.0040	19.0000	
SGO_0677	-0.787	8.346	0.0071	0.0406	29.000	111.000	76.3280	115.5188	ylmF protein
					15.000	88.500	45.0121	88.5000	

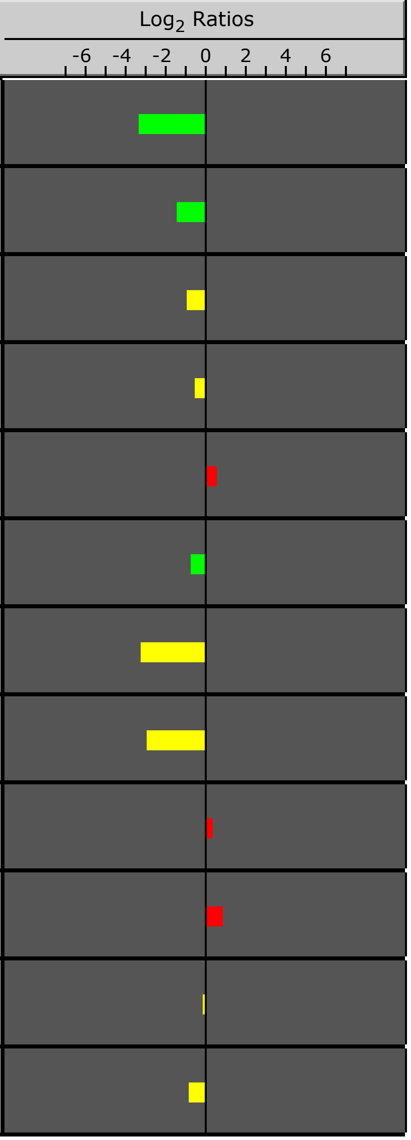

Show detected proteins only
Show all proteins
Filter by category:
ABC Transporter

Proteins found: 624

Test	Cutoff
q-Value	.005
p-Value	

	Signif	Direction	Applies To
red	yes	+	ratios, bars
yellow	no	n/a	bars
green	yes	−	ratios, bars
pink	yes	+	*p*- , *q*-Values
light green	yes	−	*p*- , *q*-Values

Dot Plots | Dot Plots

Hendrickson *et al.*

SgPgFn vs Sg	Streptococcus gordonii	Hackett Laboratory UW

Protein	SgPgFn vs Sg Log2 Ratio	Log2 Sum	q-Value	p-Value	Raw SgPgFn	Raw Sg	Normalized SgPgFn	Normalized Sg	Description
SGO_0680	0.360	8.769	0.0084	0.0506	41.000	93.500	107.9121	97.3063	cell division protein DivIVA
					46.000	93.000	138.0372	93.0000	
SGO_0681	0.282	8.622	0.0122	0.0830	42.500	74.500	111.8601	77.5329	ileS; isoleucyl-tRNA synthetase
					34.500	101.000	103.5279	101.0000	
SGO_0684	-0.754	8.337	0.0001	0.0000	23.500	97.000	61.8520	100.9488	hypothetical protein SGO_0684
					19.500	102.000	58.5157	102.0000	
SGO_0688	-0.604	7.948	0.0002	0.0003	19.000	73.500	50.0080	76.4922	ATP dependent Clp protease, ATP-binding subunit, ClpE
					16.000	72.500	48.0129	72.5000	
SGO_0693	-2.019	5.369	0.0151	0.1091		20.000		20.8142	xseA; exodeoxyribonuclease VII, large subunit
					1.500	16.000	4.5012	16.0000	
SGO_0700	-0.170	5.972	0.0741	0.6885	4.000	18.500	10.5280	19.2531	DegV family protein
					6.500	13.500	19.5052	13.5000	
SGO_0701	0.991	12.614	0.0019	0.0078	898.500	1072.000	2364.8534	1115.6407	hup; DNA-binding histone-like protein HU
					605.000	974.000	1815.4886	974.0000	
SGO_0704	2.431	11.636	0.0001	0.0001	501.500	193.000	1319.9488	200.8570	gpmA; 2,3-bisphosphoglycerate-dependent phosphoglycerate mutase
					452.000	306.500	1356.3651	306.5000	
SGO_0706	1.279	4.295	0.0196	0.1513		3.000		3.1221	phoH-like protein
					3.500	6.000	10.5028	6.0000	
SGO_0707	-4.949	8.783	0.0000	0.0000	4.000	206.000	10.5280	214.3862	LPXTG cell wall surface protein
					1.500	211.000	4.5012	211.0000	
SGO_0708	0.711	9.617	0.0018	0.0071	83.500	137.000	219.7721	142.5772	ald; alanine dehydrogenase
					89.500	154.500	268.5723	154.5000	
SGO_0713	0.405	7.070	0.0056	0.0304	16.000	29.500	42.1120	30.7009	sgg; GTP-binding protein Era
					11.500	27.000	34.5093	27.0000	

Log2 Ratios: -6 -4 -2 0 2 4 6

Show detected proteins only
Show all proteins
Filter by category:
ABC Transporter

Proteins found: 624

Test	Cutoff
q-Value	.005
p-Value	

	Signif	Direction	Applies To
red	yes	+	ratios, bars
yellow	no	n/a	bars
green	yes	−	ratios, bars
pink	yes	+	p- , q-Values
light green	yes	−	p- , q-Values

Dot Plots Dot Plots

Hendrickson *et al.*

Protein	*SgPgFn* vs *Sg* Log$_2$ Ratio	Log$_2$ Sum	*q*-Value	*p*-Value	Raw *SgPgFn*	Raw *Sg*	Normalized *SgPgFn*	Normalized *Sg*	Description
SGO_0721	0.287	5.501	0.0435	0.3781	7.000	8.500	18.4240	8.8460	abpB-like dipeptidase lipoprotein
					2.500	10.500	7.5020	10.5000	
SGO_0736	-1.951	5.113	0.0111	0.0738	1.500	16.000	3.9480	16.6514	hprK; HPr(Ser) kinase/phosphatase
						14.000		14.0000	
SGO_0739	0.854	5.875	0.0006	0.0015	7.500	11.000	19.7400	11.4478	hypothetical protein SGO_0739
					6.000	9.500	18.0048	9.5000	
SGO_0742	0.136	5.701	0.0463	0.4048	7.000	15.000	18.4240	15.6106	peptidase, U32 family
						18.000		18.0000	
SGO_0743	-1.304	7.921	0.0007	0.0019	10.500	80.000	27.6360	83.2568	peptidase, U32 family
					14.500	88.000	43.5117	88.0000	
SGO_0745	2.462	7.139	0.0015	0.0057	27.000	8.000	71.0640	8.3257	hypothetical protein SGO_0745
					16.000	13.500	48.0129	13.5000	
SGO_0749	0.577	6.407	0.0069	0.0395	10.000	13.000	26.3200	13.5292	glutathione reductase
					8.000	21.000	24.0065	21.0000	
SGO_0750	-2.349	10.625	0.0005	0.0012	52.500	708.000	138.1801	736.8224	efflux transporter, RND family, MFP subunit subfamily
					40.000	584.500	120.0323	584.5000	
SGO_0751	0.176	8.439	0.0142	0.1021	37.500	82.500	98.7001	85.8585	ABC transporter, ATP-binding protein SP0786
					28.500	77.000	85.5230	77.0000	
SGO_0752	-2.977	7.247	0.0130	0.0914		60.000		62.4426	ABC transporter, ATP-binding protein
					3.000	80.500	9.0024	80.5000	
SGO_0753	-0.113	9.075	0.0303	0.2481	46.000	144.000	121.0721	149.8622	lysS; lysyl-tRNA synthetase
					46.000	130.500	138.0372	130.5000	
SGO_0755	-0.282	4.691	0.0355	0.2972		8.000		8.3257	regulatory protein
					2.500	10.000	7.5020	10.0000	

Log$_2$ Ratios: -6 -4 -2 0 2 4 6

Show detected proteins only
Show all proteins
Filter by category:
ABC Transporter

Proteins found: 624

Test	Cutoff
q-Value	.005
p-Value	

	Signif	Direction	Applies To
red	yes	+	ratios, bars
yellow	no	n/a	bars
green	yes	−	ratios, bars
pink	yes	+	*p*- , *q*-Values
light green	yes	−	*p*- , *q*-Values

Dot Plots Dot Plots

Protein	SgPgFn vs Sg Log_2 Ratio	Log_2 Sum	*q*-Value	*p*-Value	Raw *SgPgFn*	Raw *Sg*	Normalized *SgPgFn*	Normalized *Sg*	Description
**SGO_0760**	0.431	9.163	0.0019	0.0079	67.000	117.000	176.3441	121.7630	ppc; phosphoenolpyruvate carboxylase
					51.000	122.000	153.0412	122.0000	
**SGO_0761**	0.656	13.549	0.0001	0.0000	1410.000	2223.000	3711.1221	2313.4975	tuf; translation elongation factor Tu
					1207.500	2341.000	3623.4753	2341.0000	
**SGO_0762**	1.175	11.402	0.0039	0.0197	436.000	384.500	1147.5527	400.1528	tpiA; triosephosphate isomerase
					247.000	417.000	741.1995	417.0000	
**SGO_0763**	-1.499	8.053	0.0010	0.0030	17.500	87.500	46.0600	91.0621	murA-1; UDP-N-acetylglucosamine 1-carboxyvinyltransferase
					8.500	103.000	25.5069	103.0000	
**SGO_0771**	0.699	7.925	0.0046	0.0243	28.000	56.000	73.6960	58.2797	pepq; proline dipeptidase
					25.000	36.000	75.0202	36.0000	
**SGO_0773**	-2.492	10.209	0.0001	0.0001	32.000	508.000	84.2240	528.6805	ccpA; catabolite control protein A
					31.500	476.500	94.5254	476.5000	
**SGO_0774**	-1.853	6.904	0.0142	0.1017	5.500	56.500	14.4760	58.8001	glycosyl transferase, group 1 family protein
						46.500		46.5000	
**SGO_0775**	-1.162	6.276	0.0009	0.0028	4.500	28.500	11.8440	29.6602	glycosyl transferase, group 1
					4.000	24.000	12.0032	24.0000	
**SGO_0778**	-0.413	9.110	0.0003	0.0004	45.000	148.000	118.4401	154.0250	thrS; threonyl-tRNA synthetase
					39.500	161.500	118.5319	161.5000	
**SGO_0779**	-1.147	6.246	0.0082	0.0488	6.500	19.500	17.1080	20.2938	response regulator
					2.500	31.000	7.5020	31.0000	
**SGO_0784**	-2.876	8.139	0.0005	0.0010	6.500	105.500	17.1080	109.7949	smc; chromosome segregation protein SMC
					5.500	138.500	16.5044	138.5000	
**SGO_0786**	0.377	5.816	0.0142	0.1012	7.000	10.500	18.4240	10.9275	Cof family protein
					4.500	13.500	13.5036	13.5000	

Log_2 Ratios: -6 -4 -2 0 2 4 6

Show detected proteins only
Show all proteins
Filter by category: ABC Transporter

Proteins found: 624

Test	Cutoff
***q*-Value**	**.005**
p-Value	

	Signif	Direction	Applies To
(red)	yes	+	ratios, bars
(yellow)	no	n/a	bars
(green)	yes	−	ratios, bars
(pink)	yes	+	*p*- , *q*-Values
(light green)	yes	−	*p*- , *q*-Values

Dot Plots Dot Plots

Protein	*SgPgFn vs Sg* Log$_2$ Ratio	Log$_2$ Sum	*q*-Value	*p*-Value	Raw *SgPgFn*	Raw *Sg*	Normalized *SgPgFn*	Normalized *Sg*	Description
SGO_0787	-0.953	7.335	0.0016	0.0064	9.000	56.000	23.6880	58.2797	ftsY; cell division protein FtsY
					10.500	48.000	31.5085	48.0000	
SGO_0788	1.161	7.380	0.0002	0.0002	22.000	27.000	57.9040	28.0992	zwf; glucose-6-phosphate 1-dehydrogenase
					19.000	23.500	57.0153	23.5000	
SGO_0792	-0.372	7.330	0.0097	0.0613	14.000	38.000	36.8480	39.5470	hypothetical protein SGO_0792
					11.000	51.500	33.0089	51.5000	
SGO_0794	-0.592	7.547	0.0015	0.0055	13.500	50.000	35.5320	52.0355	metallo-beta-lactamase family protein
					13.000	60.500	39.0105	60.5000	
SGO_0795	0.525	4.707	0.0008	0.0022	3.000	5.500	7.8960	5.7239	tributyrin esterase
					2.500	5.000	7.5020	5.0000	
SGO_0798	-1.346	7.742	0.0005	0.0011	11.500	80.000	30.2680	83.2568	ABC transporter, ATP-binding protein SP1381
					10.000	70.500	30.0081	70.5000	
SGO_0800	-1.735	6.211	0.0329	0.2721	3.500	21.000	9.2120	21.8549	polysaccharide deacetylase family protein
						43.000		43.0000	
SGO_0801	0.695	8.684	0.0080	0.0468	58.500	80.500	153.9721	83.7771	hom; homoserine dehydrogenase
					34.000	71.500	102.0275	71.5000	
SGO_0802	-0.222	6.252	0.0335	0.2779	5.500	18.500	14.4760	19.2531	thrB; homoserine kinase
					7.000	21.500	21.0057	21.5000	
SGO_0803	-2.851	7.738	0.0002	0.0002	7.000	86.000	18.4240	89.5010	hypothetical protein SGO_0803
					3.000	96.500	9.0024	96.5000	
SGO_0814	0.226	3.287			2.000		5.2640		aminotransferase, class-V
						4.500		4.5000	
SGO_0815	-0.667	7.389	0.0012	0.0041	11.500	46.000	30.2680	47.8726	thiI; thiamine biosynthesis protein ThiI
					11.500	55.000	34.5093	55.0000	

Log$_2$ Ratios: -6 -4 -2 0 2 4 6

Show detected proteins only
Show all proteins
Filter by category:
ABC Transporter

Proteins found: 624

Test	Cutoff
q-Value	.005
p-Value	

Color	Signif	Direction	Applies To
red	yes	+	ratios, bars
yellow	no	n/a	bars
green	yes	−	ratios, bars
pink	yes	+	*p*- , *q*-Values
light green	yes	−	*p*- , *q*-Values

Dot Plots Dot Plots

Hendrickson *et al.*

Protein	SgPgFn vs Sg Log$_2$ Ratio	Log$_2$ Sum	*q*-Value	*p*-Value	Raw *SgPgFn*	Raw *Sg*	Normalized *SgPgFn*	Normalized *Sg*	Description
SGO_0818	2.156	9.701	0.0004	0.0007	118.000	87.000	310.5762	90.5417	rplU; ribosomal protein L21
					122.500	63.500	367.5989	63.5000	
SGO_0820	0.074	7.887	0.0933	0.8915	28.000	72.500	73.6960	75.4514	rpmA; ribosomal protein L27
					15.500	41.000	46.5125	41.0000	
SGO_0824	0.727	7.433	0.0035	0.0168	19.500	25.000	51.3240	26.0177	lepA; GTP-binding protein LepA
					18.500	40.000	55.5149	40.0000	
SGO_0832	-2.454	6.497	0.0130	0.0915		44.500		46.3116	hypothetical protein SGO_0832
					2.500	36.500	7.5020	36.5000	
SGO_0835	1.176	8.008	0.0025	0.0110	28.500	32.500	75.0120	33.8231	nitroreductase
					34.500	45.000	103.5279	45.0000	
SGO_0836	0.471	9.046	0.0053	0.0283	65.000	99.000	171.0801	103.0303	pepV; dipeptidase PepV
					45.500	118.000	136.5367	118.0000	
SGO_0842	0.388	4.835	0.0615	0.5534	3.000	3.500	7.8960	3.6425	rhodanese family protein
					2.500	9.500	7.5020	9.5000	
SGO_0848	1.395	8.511	0.0001	0.0001	52.000	48.500	136.8641	50.4744	rpmE; ribosomal protein L31
					42.500	50.000	127.5343	50.0000	
SGO_0850	2.383	8.256	0.0063	0.0348	68.500	22.500	180.2921	23.4160	flavodoxin
					26.500	22.500	79.5214	22.5000	
SGO_0854	-5.415	9.525	0.0001	0.0000	3.000	336.500	7.8960	350.1988	cshA; surface-associated protein CshA
					3.000	369.500	9.0024	369.5000	
SGO_0856	-2.301	8.333	0.0001	0.0000	11.000	124.500	28.9520	129.5683	ABC transporter, substrate binding protein
					8.500	138.500	25.5069	138.5000	
SGO_0859	-0.807	7.283	0.0037	0.0179	9.500	54.500	25.0040	56.7187	pheS; phenylalanyl-tRNA synthetase, alpha subunit
					10.500	42.500	31.5085	42.5000	

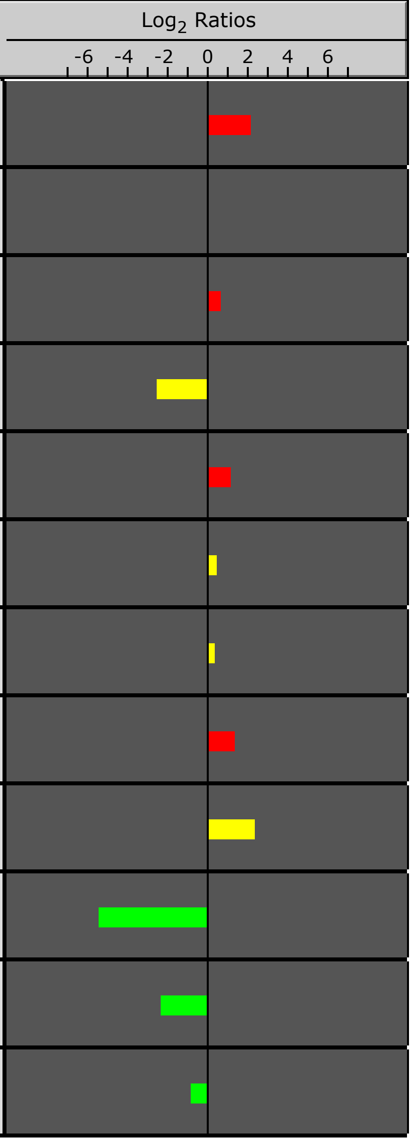

Show detected proteins only
Show all proteins
Filter by category:
ABC Transporter

Proteins found: 624

Test	Cutoff
q-Value	.005
p-Value	

	Signif	Direction	Applies To
red	yes	+	ratios, bars
yellow	no	n/a	bars
green	yes	−	ratios, bars
pink	yes	+	*p*- , *q*-Values
light green	yes	−	*p*- , *q*-Values

Dot Plots Dot Plots

Hendrickson *et al.*

Protein	*SgPgFn vs Sg* Log$_2$ Ratio	Log$_2$ Sum	*q*-Value	*p*-Value	Raw *SgPgFn*	Raw *Sg*	Normalized *SgPgFn*	Normalized *Sg*	Description
SGO_0861	0.727	8.977	0.0010	0.0033	56.000	99.000	147.3921	103.0303	pheT; phenylalanyl-tRNA synthetase, beta subunit
					55.500	87.000	166.5448	87.0000	
SGO_0885	-0.567	5.403	0.0086	0.0525	3.000	10.000	7.8960	10.4071	cobyric acid synthase
					3.000	15.000	9.0024	15.0000	
SGO_0888	-2.014	5.369	0.0175	0.1316		20.500		21.3345	hypothetical protein SGO_0888
					1.500	15.500	4.5012	15.5000	
SGO_0889	-0.844	6.839	0.0020	0.0083	9.500	36.000	25.0040	37.4655	glmM; phosphoglucosamine mutase
					5.500	35.500	16.5044	35.5000	
SGO_0893	0.522	7.678	0.0010	0.0033	23.000	44.000	60.5360	45.7912	GTP-binding protein
					20.000	38.500	60.0162	38.5000	
SGO_0901	-0.769	5.823	0.0046	0.0238	3.000	17.000	7.8960	17.6921	DNA-directed DNA polymerase III
					4.500	17.500	13.5036	17.5000	
SGO_0906	1.830	6.859	0.0013	0.0047	14.500	10.500	38.1640	10.9275	leuA; 2-isopropylmalate synthase
					17.500	14.500	52.5141	14.5000	
SGO_0911	1.485	7.903	0.0009	0.0027	29.500	29.000	77.6440	30.1806	hypothetical protein SGO_0911
					33.000	32.500	99.0267	32.5000	
SGO_0936	1.188	3.105	0.0184	0.1408		2.500		2.6018	phosphoenolpyruvate synthase, putative
					1.500	1.500	4.5012	1.5000	
SGO_0940	-0.447	4.331	0.0526	0.4634	2.000	9.000	5.2640	9.3664	ppsA; phosphoenolpyruvate synthase
						5.500		5.5000	
SGO_0946	-0.622	6.632	0.0098	0.0625	5.500	30.000	14.4760	31.2213	Deblocking aminopeptidase
					8.500	28.000	25.5069	28.0000	
SGO_0949	-0.757	4.862	0.0284	0.2286	2.500	12.500	6.5800	13.0089	deaD; DEAD RNA helicase
						9.500		9.5000	

Log$_2$ Ratios: -6 -4 -2 0 2 4 6

Show detected proteins only
Show all proteins
Filter by category:
ABC Transporter

Proteins found: 624

Test	Cutoff
q-Value	.005
p-Value	

Color	Signif	Direction	Applies To
red	yes	+	ratios, bars
yellow	no	n/a	bars
green	yes	–	ratios, bars
pink	yes	+	*p*- , *q*-Values
light green	yes	–	*p*- , *q*-Values

Dot Plots Dot Plots

Protein	SgPgFn vs Sg Log_2 Ratio	Log_2 Sum	*q*-Value	*p*-Value	Raw *SgPgFn*	Raw *Sg*	Normalized *SgPgFn*	Normalized *Sg*	Description
SGO_0950	-0.774	3.834			2.000		5.2640		oxidoreductase
						9.000		9.0000	
SGO_0951	-0.083	5.935	0.0662	0.6035	5.000	13.000	13.1600	13.5292	udk; uridine kinase
					5.500	18.000	16.5044	18.0000	
SGO_0954	0.496	6.475	0.0164	0.1211	7.500	18.000	19.7400	18.7328	ATP-binding protein
					11.000	17.500	33.0089	17.5000	
SGO_0982	-3.500	8.201	0.0002	0.0002	4.500	121.500	11.8440	126.4462	amino acid ABC transporter, amino acid-binding protein
					4.000	144.000	12.0032	144.0000	
SGO_0987	0.420	7.786	0.0008	0.0021	24.000	48.000	63.1680	49.9541	metK; S-adenosylmethionine synthetase
					21.000	44.500	63.0170	44.5000	
SGO_0995	-0.740	4.614	0.0526	0.4645	2.000	5.500	5.2640	5.7239	metallo-beta-lactamase superfamily protein
						13.500		13.5000	
SGO_1001	0.748	7.654	0.0066	0.0376	20.000	42.500	52.6400	44.2302	apt; adenine phosphoribosyltransferase
					24.500	31.000	73.5198	31.0000	
SGO_1005	0.682	5.098	0.0013	0.0047	4.000	5.500	10.5280	5.7239	Bcl-2 family protein
					3.500	7.500	10.5028	7.5000	
SGO_1009	1.149	8.693	0.0021	0.0088	46.000	57.000	121.0721	59.3204	rfbA-1; glucose-1-phosphate thymidylyltransferase
					55.000	68.500	165.0444	68.5000	
SGO_1010	0.915	7.403	0.0001	0.0001	21.500	28.000	56.5880	29.1399	rmlC; dTDP-4-keto-6-deoxyglucose-3,5-epimerase
					18.000	29.500	54.0145	29.5000	
SGO_1011	0.641	7.895	0.0064	0.0362	25.500	35.000	67.1160	36.4248	rfbB-1; dTDP-glucose 4,6-dehydratase
					25.500	58.000	76.5206	58.0000	
SGO_1012	0.585	8.521	0.0002	0.0002	41.000	72.000	107.9121	74.9311	galE-1; UDP-glucose 4-epimerase
					37.500	72.000	112.5303	72.0000	

Log_2 Ratios: -6 -4 -2 0 2 4 6

Show detected proteins only
Show all proteins
Filter by category:
ABC Transporter

Proteins found: 624

Test	Cutoff
q-Value	.005
p-Value	

Color	Signif	Direction	Applies To
red	yes	+	ratios, bars
yellow	no	n/a	bars
green	yes	–	ratios, bars
pink	yes	+	*p*- , *q*-Values
light green	yes	–	*p*- , *q*-Values

Dot Plots Dot Plots

Protein	*SgPgFn vs Sg*				Raw		Normalized		Description	Log_2 Ratios
	Log_2 Ratio	Log_2 Sum	*q*-Value	*p*-Value	*SgPgFn*	*Sg*	*SgPgFn*	*Sg*		-6 -4 -2 0 2 4 6
**SGO_1013**	-1.144	6.302	0.0175	0.1311	5.500	34.500	14.4760	35.9045	Glycosyltransferase involved in cell wall biogenesis	
						28.500		28.5000		
**SGO_1016**	-1.705	5.652	0.0215	0.1691	2.500	17.000	6.5800	17.6921	putative glycosyltransferase	
						26.000		26.0000		
**SGO_1019**	-3.361	6.410	0.0023	0.0096	1.500	39.500	3.9480	41.1080	glycosyl transferase	
						40.000		40.0000		
**SGO_1020**	0.517	7.544	0.0030	0.0137	23.000	37.500	60.5360	39.0266	rfbD; dTDP-4-dehydrorhamnose reductase	
					16.500	37.500	49.5133	37.5000		
**SGO_1025**	-0.576	6.464	0.0131	0.0922	8.500	21.500	22.3720	22.3753	rgp; glycosyltransferase	
					4.500	30.000	13.5036	30.0000		
**SGO_1026**	-3.207	7.890	0.0021	0.0088	4.000	77.000	10.5280	80.1346	rhamnosyltransferase	
					4.000	134.500	12.0032	134.5000		
**SGO_1031**	-0.973	5.369	0.0025	0.0107	2.000	13.500	5.2640	14.0496	cmk; cytidylate kinase	
					3.000	13.000	9.0024	13.0000		
**SGO_1032**	-0.623	5.422	0.0007	0.0018	3.000	12.000	7.8960	12.4885	infC; translation initiation factor IF-3	
					3.000	13.500	9.0024	13.5000		
**SGO_1033**	-0.863	7.498	0.0208	0.1629	7.500	36.000	19.7400	37.4655	rpmI; ribosomal protein L35	
					15.000	78.500	45.0121	78.5000		
**SGO_1034**	0.317	9.575	0.0337	0.2807	108.000	158.000	284.2562	164.4321	rplT; ribosomal protein L20	
					49.500	165.500	148.5400	165.5000		
**SGO_1035**	0.599	5.642	0.0130	0.0914	5.000	7.000	13.1600	7.2850	gloA; lactoylglutathione lyase	
					5.500	13.000	16.5044	13.0000		
**SGO_1036**	-2.329	6.684	0.0011	0.0035	3.000	34.500	7.8960	35.9045	amino acid ABC transporter, ATP-binding protein SP1242	
					3.000	50.000	9.0024	50.0000		

- Show detected proteins only
- Show all proteins
- Filter by category: ABC Transporter

Proteins found: 624

Test	Cutoff
***q*-Value**	**.005**
p-Value	

Colour	Signif	Direction	Applies To
red	yes	+	ratios, bars
yellow	no	n/a	bars
green	yes	−	ratios, bars
pink	yes	+	*p*- , *q*-Values
light green	yes	−	*p*- , *q*-Values

Dot Plots Dot Plots

Streptococcus gordonii

Hackett Laboratory UW

Protein	*SgPgFn vs Sg* Log_2 Ratio	Log_2 Sum	*q*-Value	*p*-Value	Raw *SgPgFn*	Raw *Sg*	Normalized *SgPgFn*	Normalized *Sg*	Description
**SGO_1038**	0.907	5.145	0.0101	0.0649	3.500	4.000	9.2120	4.1628	uvrB; excinuclease ABC, B subunit
					4.500	8.500	13.5036	8.5000	
**SGO_1047**	-0.691	5.932	0.0115	0.0768	5.500	14.500	14.4760	15.0903	hypothetical protein SGO_1047
					3.000	22.500	9.0024	22.5000	
**SGO_1049**	1.047	3.872	0.0552	0.4899	2.500	1.500	6.5800	1.5611	tRNA pseudouridine synthase B
						6.500		6.5000	
**SGO_1050**	1.263	3.088							ribF; riboflavin biosynthesis protein RibF
					2.000	2.500	6.0016	2.5000	
**SGO_1058**	-0.384	6.571	0.0169	0.1253	10.000	31.500	26.3200	32.7824	pstB; Phosphate import ATP-binding protein pstB 2 (Phosphate-transporting ATPase 2) (ABC phosphate transporter 2)
						36.000		36.0000	
**SGO_1059**	0.849	7.054	0.0001	0.0000	16.500	23.000	43.4280	23.9363	pstB; Phosphate import ATP-binding protein pstB 1 (Phosphate-transporting ATPase 1) (ABC phosphate transporter 1)
					14.000	23.500	42.0113	23.5000	
**SGO_1060**	-1.276	7.399	0.0007	0.0018	9.000	52.000	23.6880	54.1169	phosphate transport system regulatory protein
					8.500	65.500	25.5069	65.5000	
**SGO_1065**	-0.993	6.465	0.0004	0.0009	5.000	29.000	13.1600	30.1806	hypothetical protein SGO_1065
					5.500	28.500	16.5044	28.5000	
**SGO_1069**	0.331	8.508	0.0047	0.0248	36.500	84.000	96.0681	87.4196	membrane alanyl aminopeptidase
					35.500	74.000	106.5287	74.0000	
**SGO_1079**	-0.826	6.789	0.0056	0.0302	7.000	27.500	18.4240	28.6195	pdp; pyrimidine-nucleoside phosphorylase
					7.000	42.500	21.0057	42.5000	
**SGO_1080**	-0.127	8.229	0.0176	0.1329	26.500	80.500	69.7480	83.7771	deoC; deoxyribose-phosphate aldolase
					24.500	73.000	73.5198	73.0000	
**SGO_1081**	0.464	4.360	0.0716	0.6617	3.000	3.500	7.8960	3.6425	cdd; cytidine deaminase
						9.000		9.0000	

Log_2 Ratios: -6 -4 -2 0 2 4 6

Show detected proteins only
Show all proteins
Filter by category:
ABC Transporter

Proteins found: 624

Test	Cutoff
***q*-Value**	**.005**
p-Value	

Color	Signif	Direction	Applies To
red	yes	+	ratios, bars
yellow	no	n/a	bars
green	yes	−	ratios, bars
pink	yes	+	*p*- , *q*-Values
light green	yes	−	*p*- , *q*-Values

Dot Plots Dot Plots

Hendrickson *et al.*

Protein	*SgPgFn vs Sg* Log$_2$ Ratio	Log$_2$ Sum	*q*-Value	*p*-Value	Raw *SgPgFn*	Raw *Sg*	Normalized *SgPgFn*	Normalized *Sg*	Description	Log$_2$ Ratios (-6 -4 -2 0 2 4 6)
SGO_1082	-1.016	13.028	0.0024	0.0103	690.500	2606.500	1817.3970	2712.6096	lipoprotein	
					340.500	2801.500	1021.7750	2801.5000		
SGO_1088	0.367	3.396	0.0737	0.6830	1.500	2.000	3.9480	2.0814	transcription regulator, LysR family	
						4.500		4.5000		
SGO_1096	2.511	9.539	0.0010	0.0031	140.000	49.500	368.4802	51.5151	butA; acetoin dehydrogenase	
					88.500	58.500	265.5715	58.5000		
SGO_1098	1.476	4.525	0.0115	0.0771	4.000		10.5280		proA; gamma-glutamyl phosphate reductase	
					3.000	3.500	9.0024	3.5000		
SGO_1104	0.289	4.965	0.0092	0.0571	4.500	9.500	11.8440	9.8867	carB; carbamoyl-phosphate synthase, large subunit	
						9.500		9.5000		
SGO_1107	-1.162	5.074	0.0311	0.2563		16.500		17.1717	PyrR bifunctional protein	
					2.000	10.500	6.0016	10.5000		
SGO_1109	-0.527	7.996	0.0029	0.0129	21.500	66.500	56.5880	69.2072	pyrB; aspartate carbamoyltransferase	
					16.000	81.500	48.0129	81.5000		
SGO_1111	-3.352	6.828	0.0127	0.0882	2.000	45.500	5.2640	47.3523	fruR; phosphotransferase system repressor	
						61.000		61.0000		
SGO_1112	-3.355	7.010	0.0028	0.0123		58.000		60.3612	fruB; 1-phosphofructokinase	
					2.000	62.500	6.0016	62.5000		
SGO_1113	-3.884	11.211	0.0000	0.0000	26.000	1053.000	68.4320	1095.8672	fruA; PTS system, fructose specific IIABC components	
					27.500	1124.000	82.5222	1124.0000		
SGO_1114	-2.238	6.815	0.0001	0.0001	3.500	46.500	9.2120	48.3930	Protein of unknown function (DUF1149) superfamily	
					3.500	44.500	10.5028	44.5000		
SGO_1116	2.399	6.582	0.0006	0.0013	13.500	6.500	35.5320	6.7646	dapB; dihydrodipicolinate reductase	
					15.000	8.500	45.0121	8.5000		

Show detected proteins only
Show all proteins
Filter by category:
ABC Transporter

Proteins found: 624

Test	Cutoff
***q*-Value**	**.005**
p-Value	

	Signif	Direction	Applies To
red	yes	+	ratios, bars
yellow	no	n/a	bars
green	yes	−	ratios, bars
pink	yes	+	*p*- , *q*-Values
light green	yes	−	*p*- , *q*-Values

Dot Plots Dot Plots

Hendrickson *et al.*

Protein	*SgPgFn vs Sg* Log$_2$ Ratio	Log$_2$ Sum	*q*-Value	*p*-Value	Raw *SgPgFn*	Raw *Sg*	Normalized *SgPgFn*	Normalized *Sg*	Description	Log$_2$ Ratios
SGO_1120	0.698	8.203	0.0049	0.0263	29.500	48.000	77.6440	49.9541	guaA; GMP synthase	
					35.000	62.000	105.0283	62.0000		
SGO_1123	1.001	7.805	0.0004	0.0010	26.500	35.000	69.7480	36.4248	ffh; signal recognition particle protein	
					26.500	38.000	79.5214	38.0000		
SGO_1129	-0.672	6.501	0.0006	0.0016	7.000	28.000	18.4240	29.1399	lplA; lipoate protein ligase A	
					5.500	26.500	16.5044	26.5000		
SGO_1130	-0.959	8.402	0.0001	0.0000	22.000	109.000	57.9040	113.4373	dihydrolipoamide dehydrogenase	
					19.000	110.000	57.0153	110.0000		
SGO_1131	-0.836	6.722	0.0014	0.0050	6.000	32.000	15.7920	33.3027	sucB; dihydrolipoamide S-acetyltransferase	
					7.500	34.000	22.5061	34.0000		
SGO_1132	-1.908	5.520	0.0242	0.1926	2.000	15.000	5.2640	15.6106	acetoin dehydrogenase	
						25.000		25.0000		
SGO_1133	-1.214	6.539	0.0027	0.0117	4.000	35.000	10.5280	36.4248	acoA; acetoin dehydrogenase	
					6.000	28.000	18.0048	28.0000		
SGO_1134	0.121	4.103	0.0782	0.7301		4.500		4.6832	hypothetical protein SGO_1134	
					2.000	6.500	6.0016	6.5000		
SGO_1139	0.580	6.523	0.0034	0.0164	10.000	15.000	26.3200	15.6106	GTP-binding protein	
					9.500	21.500	28.5077	21.5000		
SGO_1140	-0.066	8.332	0.0435	0.3780	29.500	85.500	77.6440	88.9807	clpX; ATP-dependent Clp protease, ATP-binding subunit ClpX	
					26.500	76.000	79.5214	76.0000		
SGO_1141	0.201	4.191	0.0695	0.6387	2.500	4.500	6.5800	4.6832	folA; dihydrofolate reductase	
						7.000		7.0000		
SGO_1143	0.799	4.852	0.0299	0.2442	5.000	5.500	13.1600	5.7239	thyA; thymidylate synthase	
						10.000		10.0000		

Log$_2$ Ratios axis: -6 -4 -2 0 2 4 6

Show detected proteins only
Show all proteins
Filter by category:
ABC Transporter

Proteins found: 624

Test	Cutoff
q-Value	.005
p-Value	

	Signif	Direction	Applies To
red	yes	+	ratios, bars
yellow	no	n/a	bars
green	yes	−	ratios, bars
pink	yes	+	*p*- , *q*-Values
light green	yes	−	*p*- , *q*-Values

Dot Plots Dot Plots

Hendrickson *et al.*

Protein	SgPgFn vs Sg Log_2 Ratio	Log_2 Sum	*q*-Value	*p*-Value	Raw *SgPgFn*	Raw *Sg*	Normalized *SgPgFn*	Normalized *Sg*	Description
SGO_1144	1.707	9.282	0.0004	0.0009	98.500	66.500	259.2521	69.2072	glcK; glucokinase
					72.500	76.500	217.5586	76.5000	
SGO_1150	0.585	4.130							hypothetical protein SGO_1150
					3.500	7.000	10.5028	7.0000	
SGO_1151	1.614	9.238	0.0001	0.0000	84.000	68.500	221.0881	71.2886	glyA; serine hydroxymethyltransferase
					78.000	77.500	234.0630	77.5000	
SGO_1154	0.523	7.625	0.0085	0.0520	20.500	47.500	53.9560	49.4337	prfA; peptide chain release factor 1
					20.500	32.500	61.5166	32.5000	
SGO_1155	0.196	5.567	0.0922	0.8799		9.500		9.8867	tdk; thymidine kinase
					5.500	21.000	16.5044	21.0000	
SGO_1167	-0.299	7.772	0.0371	0.3123	14.000	54.000	36.8480	56.1983	nox; NADH oxidase
					21.000	62.500	63.0170	62.5000	
SGO_1169	0.557	7.504	0.0004	0.0009	20.500	37.500	53.9560	39.0266	NADPH-dependent FMN reductase
					18.000	34.500	54.0145	34.5000	
SGO_1170	-0.745	8.696	0.0002	0.0002	31.000	125.500	81.5920	130.6091	NADPH-dependent FMN reductase
					24.500	129.000	73.5198	129.0000	
SGO_1185	0.351	4.801	0.0232	0.1848	2.500	7.000	6.5800	7.2850	acetyltransferase, GNAT family
					3.000	5.000	9.0024	5.0000	
SGO_1189	-3.464	9.278	0.0003	0.0004	15.000	292.500	39.4800	304.4076	lipoprotein, putative
					5.500	260.500	16.5044	260.5000	
SGO_1191	0.717	11.990	0.0008	0.0022	454.000	789.500	1194.9287	821.6402	rplL; ribosomal protein L7/L12
					444.500	718.000	1333.8590	718.0000	
SGO_1192	-0.130	9.230	0.0106	0.0690	56.500	144.500	148.7081	150.3825	BL5; 50S ribosomal protein L10
					46.000	163.500	138.0372	163.5000	

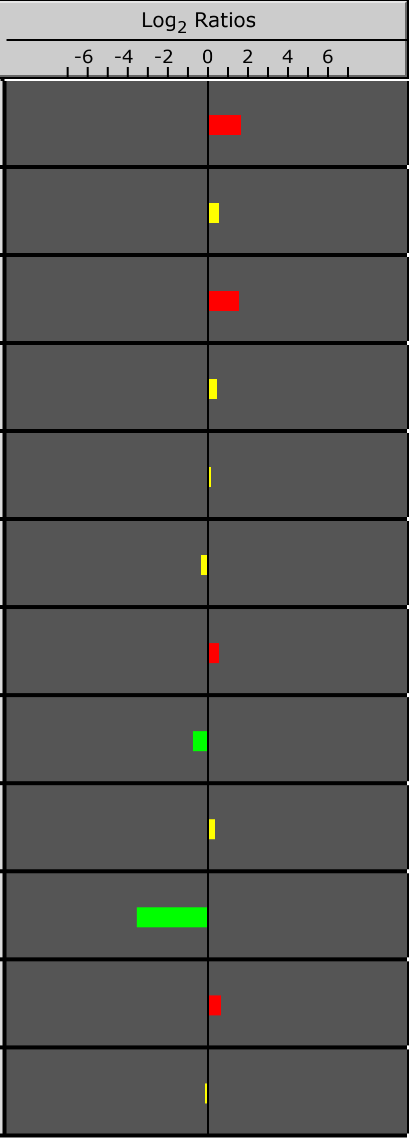

Show detected proteins only
Show all proteins
Filter by category:
ABC Transporter

Proteins found: 624

Test	Cutoff
q-Value	.005
p-Value	

Color	Signif	Direction	Applies To
red	yes	+	ratios, bars
yellow	no	n/a	bars
green	yes	−	ratios, bars
pink	yes	+	*p*- , *q*-Values
light green	yes	−	*p*- , *q*-Values

Dot Plots | Dot Plots

Hendrickson *et al.*

Protein	*SgPgFn* vs *Sg* Log$_2$ Ratio	Log$_2$ Sum	*q*-Value	*p*-Value	Raw *SgPgFn*	Raw *Sg*	Normalized *SgPgFn*	Normalized *Sg*	Description
SGO_1193	-0.784	5.522	0.0004	0.0007	3.000	13.500	7.8960	14.0496	gid; Glucose inhibited division protein A
					3.000	15.000	9.0024	15.0000	
SGO_1196	1.358	4.061				4.500		4.6832	satD; SatD-like protein
					4.000		12.0032		
SGO_1197	-1.756	7.913	0.0006	0.0017	10.000	79.500	26.3200	82.7364	topA; DNA topoisomerase I
					9.500	103.500	28.5077	103.5000	
SGO_1198	-0.145	8.938	0.0296	0.2408	48.000	134.000	126.3361	139.4551	DNA processing Smf protein
					35.500	118.000	106.5287	118.0000	
SGO_1203	0.435	6.426	0.0071	0.0404	8.500	15.500	22.3720	16.1310	anaerobic ribonucleotide reductase
					9.000	20.500	27.0073	20.5000	
SGO_1205	0.719	4.781	0.0129	0.0899	3.500	7.000	9.2120	7.2850	dapA; dihydrodipicolinate synthase
					2.500	3.500	7.5020	3.5000	
SGO_1206	0.164	7.642	0.0130	0.0915	19.000	42.500	50.0080	44.2302	asd; aspartate-semialdehyde dehydrogenase
					18.500	50.000	55.5149	50.0000	
SGO_1210	-0.802	4.885	0.0098	0.0626	2.500	11.500	6.5800	11.9682	fhs-1; formate--tetrahydrofolate ligase
						11.000		11.0000	
SGO_1215	0.239	9.813	0.0014	0.0051	92.500	190.000	243.4601	197.7348	manB; phosphomannomutase
					81.000	215.000	243.0654	215.0000	
SGO_1216	1.150	6.754	0.0004	0.0009	14.500	18.500	38.1640	19.2531	bta; Possible bacteriocin transport accessory protein
					12.000	14.500	36.0097	14.5000	
SGO_1219	-0.058	7.613	0.0681	0.6240	16.500	44.000	43.4280	45.7912	pta; phosphate acetyltransferase
					17.500	54.000	52.5141	54.0000	
SGO_1224	0.425	8.740	0.0033	0.0157	42.500	89.500	111.8601	93.1435	Ribose-phosphate pyrophosphokinase 2
					44.500	89.000	133.5359	89.0000	

Log$_2$ Ratios: -6 -4 -2 0 2 4 6

Show detected proteins only
Show all proteins
Filter by category:
ABC Transporter

Proteins found: 624

Test	Cutoff
q-Value	.005
p-Value	

Color	Signif	Direction	Applies To
red	yes	+	ratios, bars
yellow	no	n/a	bars
green	yes	–	ratios, bars
pink	yes	+	*p*- , *q*-Values
light green	yes	–	*p*- , *q*-Values

Dot Plots Dot Plots

Hendrickson *et al.*

Streptococcus gordonii

Hackett Laboratory UW

Protein	SgPgFn vs Sg: Log_2 Ratio	Log_2 Sum	*q*-Value	*p*-Value	Raw: *SgPgFn*	Raw: *Sg*	Normalized: *SgPgFn*	Normalized: *Sg*	Description
SGO_1225	0.465	5.279	0.0018	0.0070	4.000	7.500	10.5280	7.8053	pyridoxal-phosphate dependent aminotransferase
					4.000	8.500	12.0032	8.5000	
SGO_1226	0.231	6.360	0.0532	0.4709	8.000	13.500	21.0560	14.0496	hypothetical protein SGO_1226
					7.500	24.500	22.5061	24.5000	
SGO_1231	-1.868	8.032	0.0004	0.0008	10.500	90.000	27.6360	93.6639	gyrA; DNA gyrase, A subunit
					9.500	112.000	28.5077	112.0000	
SGO_1232	3.026	10.443	0.0000	0.0000	234.000	65.000	615.8884	67.6461	L-lactate dehydrogenase
					207.500	85.500	622.6676	85.5000	
SGO_1234	0.581	10.993	0.0008	0.0023	228.500	359.500	601.4123	374.1351	rpsA; 30S ribosomal protein S1
					206.000	444.000	618.1664	444.0000	
SGO_1237	0.822	6.561	0.0153	0.1106	7.500	17.000	19.7400	17.6921	hypothetical protein SGO_1237
					14.000	15.000	42.0113	15.0000	
SGO_1238	-0.322	6.950	0.0122	0.0839	9.000	35.000	23.6880	36.4248	ilvE; branched-chain amino acid aminotransferase
					10.500	32.000	31.5085	32.0000	
SGO_1239	-2.990	6.446	0.0001	0.0001	2.000	35.500	5.2640	36.9452	parC; DNA topoisomerase IV, A subunit
					1.500	40.500	4.5012	40.5000	
SGO_1242	-2.541	7.232	0.0106	0.0688	4.500	72.500	11.8440	75.4514	lipoprotein, putative
						63.000		63.0000	
SGO_1248	-0.668	5.583	0.0094	0.0593	3.500	17.500	9.2120	18.2124	pyrC; dihydroorotase
					3.000	11.500	9.0024	11.5000	
SGO_1253	0.357	6.621	0.0183	0.1387	9.000	17.500	23.6880	18.2124	pyrE; orotate phosphoribosyltransferase
					10.500	25.000	31.5085	25.0000	
SGO_1260	0.150	8.227	0.0123	0.0845	32.000	69.000	84.2240	71.8090	deoD; purine nucleoside phosphorylase
					24.500	70.000	73.5198	70.0000	

Log_2 Ratios: -6 -4 -2 0 2 4 6

Show detected proteins only
Show all proteins
Filter by category:
ABC Transporter

Proteins found: 624

Test	Cutoff
q-Value	.005
p-Value	

Color	Signif	Direction	Applies To
red	yes	+	ratios, bars
yellow	no	n/a	bars
green	yes	−	ratios, bars
pink	yes	+	*p*- , *q*-Values
light green	yes	−	*p*- , *q*-Values

Dot Plots Dot Plots

Hendrickson *et al.*

Protein	SgPgFn vs Sg Log$_2$ Ratio	Log$_2$ Sum	*q*-Value	*p*-Value	Raw *SgPgFn*	Raw *Sg*	Normalized *SgPgFn*	Normalized *Sg*	Description
SGO_1263	-0.436	8.496	0.0106	0.0693	24.000	106.500	63.1680	110.8356	purine nucleoside phosphorylase I, inosine and guanosine-specific
					30.500	95.500	91.5246	95.5000	
SGO_1264	-0.125	9.941	0.0572	0.5115	75.500	220.000	198.7161	228.9561	deoB; phosphopentomutase
					91.000	282.000	273.0735	282.0000	
SGO_1265	0.677	7.670	0.0125	0.0861	21.500	26.000	56.5880	27.0585	rpiA; ribose 5-phosphate isomerase
					22.000	54.000	66.0178	54.0000	
SGO_1266	0.563	6.020	0.0059	0.0320	8.500	12.500	22.3720	13.0089	trmE; tRNA modification GTPase TrmE
					5.500	13.000	16.5044	13.0000	
SGO_1273	1.987	8.558	0.0018	0.0070	68.500	40.000	180.2921	41.6284	rpoD; RNA polymerase sigma factor
					40.500	33.500	121.5327	33.5000	
SGO_1276	0.481	7.989	0.0059	0.0324	24.500	49.500	64.4840	51.5151	rpsU; ribosomal protein S21
					28.000	54.000	84.0226	54.0000	
SGO_1281	-0.127	4.265	0.0675	0.6167		5.500		5.7239	penicillinase repressor, putative
					2.000	7.500	6.0016	7.5000	
SGO_1283	0.335	8.261	0.0009	0.0029	32.500	68.500	85.5400	71.2886	oxidoreductase
					28.500	64.500	85.5230	64.5000	
SGO_1284	0.103	5.968	0.0455	0.3963	5.500	15.500	14.4760	16.1310	thioredoxin-disulfide reductase
					6.000	14.000	18.0048	14.0000	
SGO_1293	0.069	7.646	0.0692	0.6353	15.000	44.500	39.4800	46.3116	asnS; asparaginyl-tRNA synthetase
					21.500	50.000	64.5174	50.0000	
SGO_1297	1.112	6.360	0.0001	0.0000	10.500	12.000	27.6360	12.4885	aspC; aspartate aminotransferase
					9.500	13.500	28.5077	13.5000	
SGO_1305	-1.367	5.998	0.0004	0.0007	4.000	21.500	10.5280	22.3753	substrate-binding protein MsmE
					2.500	23.500	7.5020	23.5000	

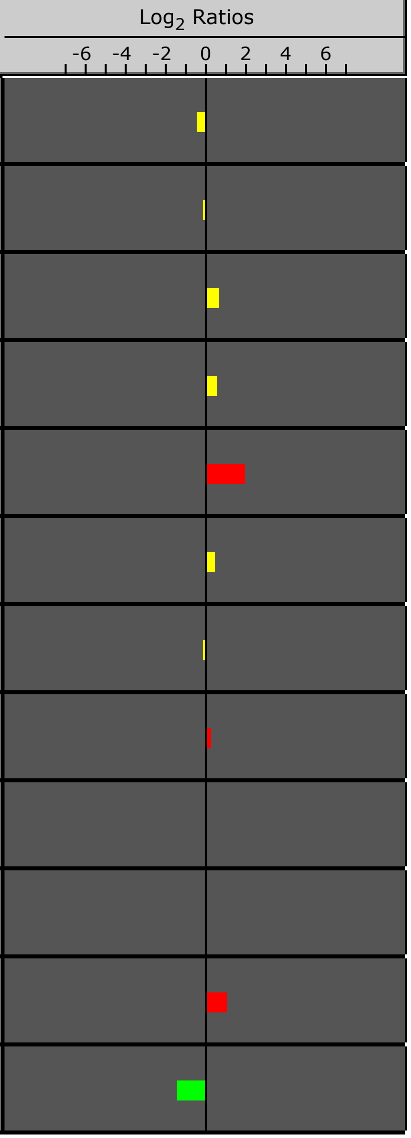

Show detected proteins only
Show all proteins
Filter by category:
ABC Transporter

Proteins found: 624

Test	Cutoff
q-Value	.005
p-Value	

	Signif	Direction	Applies To
red	yes	+	ratios, bars
yellow	no	n/a	bars
green	yes	−	ratios, bars
pink	yes	+	*p*- , *q*-Values
light green	yes	−	*p*- , *q*-Values

Dot Plots Dot Plots

Hendrickson *et al.*

Protein	*SgPgFn* vs *Sg*				Raw		Normalized		Description	Log_2 Ratios
	Log_2 Ratio	Log_2 Sum	*q*-Value	*p*-Value	*SgPgFn*	*Sg*	*SgPgFn*	*Sg*		-6 -4 -2 0 2 4 6
SGO_1312	3.152	7.040	0.0004	0.0009	25.000	7.000	65.8000	7.2850	pepT; peptidase T	
					17.500	6.000	52.5141	6.0000		
SGO_1323	1.424	8.182	0.0002	0.0003	41.500	42.500	109.2281	44.2302	rpsP; ribosomal protein S16	
					34.000	35.000	102.0275	35.0000		
SGO_1327	-0.794	5.775	0.0079	0.0463	3.500	13.000	9.2120	13.5292	HAD-superfamily subfamily IIA hydrolase, TIGR01457	
					3.500	21.500	10.5028	21.5000		
SGO_1336	-0.743	5.818	0.0010	0.0032	3.500	17.000	9.2120	17.6921	pcrA; ATP-dependent DNA helicase PcrA	
					4.000	17.500	12.0032	17.5000		
SGO_1339	0.963	11.757	0.0003	0.0003	454.000	551.000	1194.9287	573.4310	pyk; pyruvate kinase	
					364.500	600.000	1093.7944	600.0000		
SGO_1340	0.630	10.116	0.0002	0.0003	124.500	213.500	327.6842	222.1915	Phosphofructokinase	
					115.500	213.500	346.5933	213.5000		
SGO_1342	-1.216	10.281	0.0001	0.0000	71.000	423.500	186.8721	440.7405	ABC transporter, ATP-binding protein SP1715	
					62.500	429.000	187.5505	429.0000		
SGO_1364	-2.201	5.952	0.0174	0.1299		22.500		23.4160	rumA-2; 23S rRNA (uracil-5-)-methyltransferase RumA	
					2.000	32.500	6.0016	32.5000		
SGO_1365	-2.531	6.236	0.0012	0.0040		33.500		34.8638	transcription regulator yrfE	
					2.000	34.500	6.0016	34.5000		
SGO_1369	-0.928	5.247	0.0072	0.0414	3.000	14.000	7.8960	14.5699	L-2-hydroxyisocaproate dehydrogenase	
						15.500		15.5000		
SGO_1370	1.637	7.025	0.0003	0.0004	17.500	15.500	46.0600	16.1310	Protein of unknown function (DUF964) superfamily	
					17.500	15.500	52.5141	15.5000		
SGO_1372	0.923	4.628	0.0109	0.0717		5.500		5.7239	aroC; chorismate synthase	
					4.000	7.000	12.0032	7.0000		

Show detected proteins only
Show all proteins
Filter by category:
ABC Transporter

Proteins found:
624

Test	Cutoff
q-Value	.005
p-Value	

	Signif	Direction	Applies To
(red)	yes	+	ratios, bars
(yellow)	no	n/a	bars
(green)	yes	−	ratios, bars
(pink)	yes	+	*p*- , *q*-Values
(light green)	yes	−	

Dot Plots Dot Plots

Protein	*SgPgFn* vs *Sg*				Raw		Normalized		Description	Log_2 Ratios
	Log_2 Ratio	Log_2 Sum	*q*-Value	*p*-Value	*SgPgFn*	*Sg*	*SgPgFn*	*Sg*		-6 -4 -2 0 2 4 6
SGO_1373	0.062	4.562	0.1026	0.9899	3.000	5.500	7.8960	5.7239	aroB; 3-dehydroquinate synthase	
						10.000		10.0000		
SGO_1375	0.006	5.308	0.1015	0.9771	5.000	11.000	13.1600	11.4478	aroD; 3-dehydroquinate dehydratase, type I	
						15.000		15.0000		
SGO_1377	-3.781	7.966	0.0002	0.0001	4.500	105.000	11.8440	109.2745	sulfatase	
					2.000	123.000	6.0016	123.0000		
SGO_1381	-0.907	6.330	0.0003	0.0006	5.500	24.000	14.4760	24.9770	csn1; CRISPR-associated protein, Csn1 family	
					4.500	27.500	13.5036	27.5000		
SGO_1383	0.813	9.118	0.0003	0.0004	65.500	91.500	172.3961	95.2249	rplS; ribosomal protein L19	
					60.500	106.500	181.5489	106.5000		
SGO_1390	-0.819	5.995	0.0031	0.0140	5.500	20.000	14.4760	20.8142	ligA; DNA ligase, NAD-dependent	
					3.000	19.500	9.0024	19.5000		
SGO_1397	-0.134	6.668	0.0535	0.4738	8.500	20.000	22.3720	20.8142	map; methionine aminopeptidase, type I	
					8.500	33.000	25.5069	33.0000		
SGO_1400	-1.006	5.997	0.0111	0.0740	6.500	18.500	17.1080	19.2531	murA-2; UDP-N-acetylglucosamine 1-carboxyvinyltransferase	
					2.000	21.500	6.0016	21.5000		
SGO_1414	0.673	5.281	0.0084	0.0507	5.000	5.500	13.1600	5.7239	rexB; putative exonuclease RexB	
					3.500	9.500	10.5028	9.5000		
SGO_1422	0.474	7.184	0.0121	0.0817	13.000	28.500	34.2160	29.6602	hypothetical protein SGO_1422	
					17.000	30.500	51.0137	30.5000		
SGO_1426	2.044	14.741	0.0001	0.0000	4114.000	2771.500	10828.054	2884.3267	eno; enolase	
					3729.500	2470.500	11191.512	2470.5000		
SGO_1431	-1.818	10.285	0.0024	0.0100	51.500	570.000	135.5481	593.2045	EzrA; Septation ring formation regulator ezrA	
					45.000	383.500	135.0363	383.5000		

Show detected proteins only
Show all proteins
Filter by category:
ABC Transporter

Proteins found: 624

Test	Cutoff
q-Value	.005
p-Value	

	Signif	Direction	Applies To
(red)	yes	+	ratios, bars
(yellow)	no	n/a	bars
(green)	yes	–	ratios, bars
(pink)	yes	+	*p*- , *q*-Values
(light green)	yes	–	

Dot Plots Dot Plots

Hendrickson *et al.*

Protein	*SgPgFn vs Sg* Log_2 Ratio	Log_2 Sum	*q*-Value	*p*-Value	Raw *SgPgFn*	Raw *Sg*	Normalized *SgPgFn*	Normalized *Sg*	Description
SGO_1432	-0.175	7.394	0.0706	0.6504	19.500	33.500	51.3240	34.8638	gyrB; DNA gyrase, B subunit
					9.500	53.500	28.5077	53.5000	
SGO_1434	1.074	2.957			2.000		5.2640		thiJ; 4-methyl-5(beta-hydroxyethyl)-thiazole monophosphate synthesis protein
						2.500		2.5000	
SGO_1439	-1.087	7.383	0.0003	0.0006	9.000	55.500	23.6880	57.7594	ftsX; cell division protein FtsX
					10.000	55.500	30.0081	55.5000	
SGO_1440	-2.111	8.027	0.0001	0.0000	9.500	104.500	25.0040	108.7542	cell-division ATP-binding protein FtsE
					8.000	103.000	24.0065	103.0000	
SGO_1441	0.530	6.167	0.0258	0.2063	11.500	14.500	30.2680	15.0903	prfB; peptide chain release factor 2
					4.500	13.000	13.5036	13.0000	
SGO_1446	-1.757	5.755	0.0038	0.0190	3.000	15.000	7.8960	15.6106	murF; UDP-N-acetylmuramoyl-tripeptide--D-alanyl-D-alanine ligase
					1.500	26.000	4.5012	26.0000	
SGO_1447	-0.234	6.516	0.0002	0.0002	8.000	23.500	21.0560	24.4567	ddlA; D-Ala-D-Ala ligase
					7.000	25.000	21.0057	25.0000	
SGO_1450	-0.049	5.584	0.0945	0.9050	2.000	5.000	5.2640	5.2035	hypothetical protein SGO_1450
					6.000	19.500	18.0048	19.5000	
SGO_1451	1.596	7.952	0.0003	0.0004	33.500	33.500	88.1721	34.8638	frr; ribosome recycling factor
					32.500	27.000	97.5262	27.0000	
SGO_1452	0.769	7.227	0.0011	0.0036	17.500	30.500	46.0600	31.7416	pyrH; uridylate kinase
					16.000	24.000	48.0129	24.0000	
SGO_1455	1.539	9.954	0.0016	0.0063	117.500	129.500	309.2602	134.7719	rplA; ribosomal protein L1
					143.500	117.000	430.6159	117.0000	
SGO_1456	1.340	8.456	0.0002	0.0002	45.500	46.500	119.7561	48.3930	rplK; ribosomal protein L11
					44.000	51.000	132.0355	51.0000	

Log_2 Ratios: -6 -4 -2 0 2 4 6

Show detected proteins only
Show all proteins
Filter by category:
ABC Transporter

Proteins found: 624

Test	Cutoff
q-Value	.005
p-Value	

	Signif	Direction	Applies To
red	yes	+	ratios, bars
yellow	no	n/a	bars
green	yes	−	ratios, bars
pink	yes	+	*p*- , *q*-Values
light green	yes	−	*p*- , *q*-Values

Dot Plots Dot Plots

Hendrickson *et al.*

Protein	SgPgFn vs Sg				Raw		Normalized		Description
	Log_2 Ratio	Log_2 Sum	*q*-Value	*p*-Value	*SgPgFn*	*Sg*	*SgPgFn*	*Sg*	
SGO_1458	-3.490	6.985	0.0199	0.1543	2.000	71.500	5.2640	74.4107	aha1; cation-transporting ATPase yfgQ
						47.000		47.0000	
SGO_1460	-2.301	6.850	0.0154	0.1116	4.000	57.500	10.5280	59.8408	DNA translocase ftsK
						45.000		45.0000	
SGO_1463	-1.721	7.388	0.0001	0.0001	8.000	59.500	21.0560	61.9222	peptidyl-prolyl cis-trans isomerase
					6.000	66.500	18.0048	66.5000	
SGO_1464	-1.964	7.359	0.0007	0.0020	7.000	55.000	18.4240	57.2390	uncharacterized probable metal-binding protein
					5.000	73.500	15.0040	73.5000	
SGO_1465	-0.670	6.990	0.0048	0.0254	10.000	32.000	26.3200	33.3027	ABC transporter, ATP-binding protein SP0770
					7.500	45.000	22.5061	45.0000	
SGO_1469	0.313	7.853	0.0043	0.0221	22.500	48.500	59.2200	50.4744	glmU; UDP-N-acetylglucosamine pyrophosphorylase
					23.000	52.500	69.0186	52.5000	
SGO_1472	0.170	3.088							acetyltransferase, GNAT family
					1.500	4.000	4.5012	4.0000	
SGO_1486	1.330	3.932	0.0225	0.1786	2.000		5.2640		beta-galactosidase
					2.500	2.500	7.5020	2.5000	
SGO_1516	0.291	5.601	0.0057	0.0306	5.000	9.500	13.1600	9.8867	lacD-2; tagatose 1,6-diphosphate aldolase
					4.500	12.000	13.5036	12.0000	
SGO_1530	-0.321	8.323	0.0092	0.0575	26.000	95.500	68.4320	99.3878	methionine-tRNA ligase
					24.500	79.000	73.5198	79.0000	
SGO_1531	0.273	5.947	0.0295	0.2398	5.000	13.000	13.1600	13.5292	xth; exodeoxyribonuclease III
					7.000	14.000	21.0057	14.0000	
SGO_1534	1.284	5.196	0.0428	0.3687		3.500		3.6425	ArsC family
					6.000	15.000	18.0048	15.0000	

Log_2 Ratios: -6 -4 -2 0 2 4 6

Show detected proteins only
Show all proteins
Filter by category:
ABC Transporter

Proteins found: 624

Test	Cutoff
***q*-Value**	**.005**
p-Value	

	Signif	Direction	Applies To
red	yes	+	ratios, bars
yellow	no	n/a	bars
green	yes	−	ratios, bars
pink	yes	+	*p*- , *q*-Values
light green	yes	−	*p*- , *q*-Values

Dot Plots Dot Plots

Hendrickson *et al.*

Protein	SgPgFn vs Sg Log_2 Ratio	Log_2 Sum	*q*-Value	*p*-Value	Raw *SgPgFn*	Raw *Sg*	Normalized *SgPgFn*	Normalized *Sg*	Description
SGO_1536	-0.798	6.124	0.0053	0.0282	4.000	18.000	10.5280	18.7328	conserved hypothetical protein TIGR00096
					5.000	25.500	15.0040	25.5000	
SGO_1539	0.614	6.430	0.0085	0.0520	8.000	16.000	21.0560	16.6514	tmk; thymidylate kinase
					10.500	17.000	31.5085	17.0000	
SGO_1541	-2.232	7.454	0.0008	0.0022	4.500	60.500	11.8440	62.9629	atpC; ATP synthase F1, epsilon subunit
					6.500	81.000	19.5052	81.0000	
SGO_1542	-1.022	10.277	0.0002	0.0002	73.000	410.500	192.1361	427.2113	atpD; ATP synthase F1, beta subunit
					72.500	403.500	217.5586	403.5000	
SGO_1543	-2.555	7.153	0.0001	0.0001	4.500	60.500	11.8440	62.9629	atpG; ATP synthase F1, gamma subunit
					3.000	58.500	9.0024	58.5000	
SGO_1544	-0.675	10.144	0.0002	0.0003	79.000	330.000	207.9281	343.4342	atpA; ATP synthase F1, alpha subunit
					76.000	352.000	228.0614	352.0000	
SGO_1545	-2.474	7.521	0.0002	0.0003	5.500	80.000	14.4760	83.2568	atpH; ATP synthase F1, delta subunit
					4.500	72.500	13.5036	72.5000	
SGO_1546	-1.162	8.114	0.0003	0.0005	16.500	86.500	43.4280	90.0214	atpF; ATP synthase F0, B subunit
					14.000	101.500	42.0113	101.5000	
SGO_1550	-0.160	7.692	0.0416	0.3558	16.500	45.000	43.4280	46.8319	glgP-1; glycogen phosphorylase
					18.000	62.500	54.0145	62.5000	
SGO_1551	0.335	5.734	0.0978	0.9376	4.000	5.000	10.5280	5.2035	glgA; Glycogen synthase
					5.500	21.000	16.5044	21.0000	
SGO_1552	1.172	7.482	0.0036	0.0174	27.500	32.500	72.3800	33.8231	glgD; glucose-1-phosphate adenylyltransferase, GlgD subunit
					17.000	21.500	51.0137	21.5000	
SGO_1553	1.201	7.268	0.0007	0.0018	19.500	18.500	51.3240	19.2531	glgC; glucose-1-phosphate adenylyltransferase
					18.500	28.000	55.5149	28.0000	

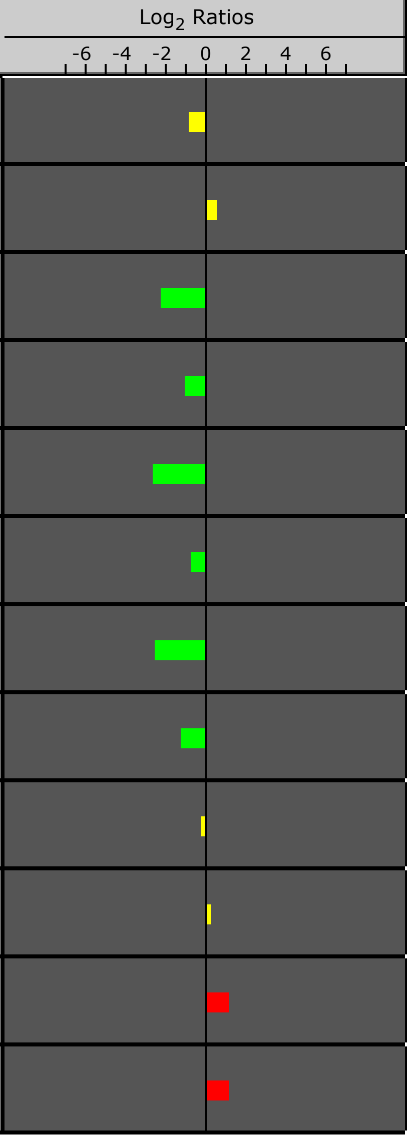

- Show detected proteins only
- Show all proteins
- Filter by category: ABC Transporter

Proteins found: 624

Test	Cutoff
q-Value	.005
p-Value	

	Signif	Direction	Applies To
red	yes	+	ratios, bars
yellow	no	n/a	bars
green	yes	–	ratios, bars
pink	yes	+	*p*- , *q*-Values
light green	yes	–	*p*- , *q*-Values

Dot Plots Dot Plots

Hendrickson *et al.*

Protein	SgPgFn vs Sg Log_2 Ratio	Log_2 Sum	*q*-Value	*p*-Value	Raw *SgPgFn*	Raw *Sg*	Normalized *SgPgFn*	Normalized *Sg*	Description
SGO_1554	1.318	7.518	0.0012	0.0042	23.000	19.000	60.5360	19.7735	glgB; 1,4-alpha-glucan branching enzyme
					23.000	34.000	69.0186	34.0000	
SGO_1555	-0.270	11.011	0.0082	0.0487	163.500	575.500	430.3322	598.9284	ptsI; phosphoenolpyruvate-protein phosphotransferase
					168.500	528.500	505.6361	528.5000	
SGO_1556	1.139	12.044	0.0021	0.0088	467.500	646.000	1230.4607	672.2984	phosphocarrier protein HPr
					561.500	635.500	1684.9535	635.5000	
SGO_1558	-0.012	8.530	0.0856	0.8069	34.500	83.000	90.8041	86.3789	nrdE; ribonucleoside-diphosphate reductase large chain
					31.000	99.500	93.0250	99.5000	
SGO_1559	1.078	8.326	0.0002	0.0002	41.000	45.500	107.9121	47.3523	ribonucleoside-diphosphate reductase, beta subunit
					36.500	56.000	109.5295	56.0000	
SGO_1570	-0.251	9.309	0.0180	0.1358	50.000	146.500	131.6001	152.4640	alaS; alanyl-tRNA synthetase
					52.500	192.500	157.5424	192.5000	
SGO_1572	-5.725	9.645	0.0016	0.0062		377.500		392.8679	proteinase maturation protein, putative
					2.500	400.500	7.5020	400.5000	
SGO_1574	0.810	8.890	0.0002	0.0002	55.500	81.000	146.0761	84.2975	pepF-1; oligoendopeptidase F
					52.000	88.000	156.0420	88.0000	
SGO_1585	-2.399	6.151	0.0222	0.1761		38.500		40.0673	D-Alanyl-D-Alanine carboxypeptidase
					2.000	25.000	6.0016	25.0000	
SGO_1587	-0.217	5.809	0.0458	0.3997	5.000	10.000	13.1600	10.4071	queA; S-adenosylmethionine:tRNA ribosyltransferase-isomerase
					4.000	20.500	12.0032	20.5000	
SGO_1591	-1.727	6.702	0.0018	0.0070	4.500	31.500	11.8440	32.7824	arcC; carbamate kinase
					4.000	47.500	12.0032	47.5000	
SGO_1592	-0.397	9.329	0.0032	0.0149	58.500	174.500	153.9721	181.6038	arcB; ornithine carbamoyltransferase
					41.500	183.000	124.5335	183.0000	

Log_2 Ratios: -6 -4 -2 0 2 4 6

Show detected proteins only
Show all proteins
Filter by category:
ABC Transporter

Proteins found: 624

Test	Cutoff
q-Value	.005
p-Value	

	Signif	Direction	Applies To
red	yes	+	ratios, bars
yellow	no	n/a	bars
green	yes	−	ratios, bars
pink	yes	+	*p*- , *q*-Values
light green	yes	−	*p*- , *q*-Values

Dot Plots Dot Plots

Hendrickson *et al.*

Protein	*SgPgFn vs Sg* Log$_2$ Ratio	Log$_2$ Sum	*q*-Value	*p*-Value	Raw *SgPgFn*	Raw *Sg*	Normalized *SgPgFn*	Normalized *Sg*	Description
SGO_1593	-0.377	9.191	0.0021	0.0090	47.500	148.500	125.0201	154.5454	arcA; arginine deiminase
					43.000	176.000	129.0347	176.0000	
SGO_1599	0.277	9.360	0.0057	0.0308	73.000	135.500	192.1361	141.0162	sodA; manganese-dependent superoxide dismutase
					56.000	156.000	168.0452	156.0000	
SGO_1604	-3.267	6.516	0.0101	0.0647		38.000		39.5470	acyltransferase family protein
					1.500	47.500	4.5012	47.5000	
SGO_1605	-2.978	6.243	0.0106	0.0686		31.000		32.2620	P-type ATPase, metal cation transport
					1.500	39.000	4.5012	39.0000	
SGO_1609	-0.912	8.175	0.0003	0.0004	20.500	88.500	53.9560	92.1028	ATP-dependent RNA helicase, DEAD/DEAH box family
					15.500	96.500	46.5125	96.5000	
SGO_1617	0.146	6.211	0.0823	0.7733	6.500	12.000	17.1080	12.4885	prfC; peptide chain release factor 3
					7.000	23.500	21.0057	23.5000	
SGO_1619	-1.811	9.715	0.0009	0.0029	38.000	357.500	100.0161	372.0537	cation-transporting ATPase, E1-E2 family
					28.500	283.000	85.5230	283.0000	
SGO_1621	-1.626	5.242	0.0106	0.0693	2.000	14.500	5.2640	15.0903	HD domain protein
						17.500		17.5000	
SGO_1622	0.164	5.675	0.0620	0.5594	4.000	14.000	10.5280	14.5699	Cof family protein
					5.500	9.500	16.5044	9.5000	
SGO_1623	-1.620	6.404	0.0071	0.0405	4.500	33.500	11.8440	34.8638	murM; MurM
						38.000		38.0000	
SGO_1624	-0.601	5.436	0.0431	0.3719		19.500		20.2938	murN; MurN protein
					3.500	12.500	10.5028	12.5000	
SGO_1625	-2.134	7.916	0.0001	0.0001	10.500	95.000	27.6360	98.8674	acetoin utilization putative/CBS domain protein
					6.000	97.000	18.0048	97.0000	

Log$_2$ Ratios: -6 -4 -2 0 2 4 6

Show detected proteins only
Show all proteins
Filter by category:
ABC Transporter

Proteins found: 624

Test	Cutoff
q-Value	.005
p-Value	

	Signif	Direction	Applies To
red	yes	+	ratios, bars
yellow	no	n/a	bars
green	yes	−	ratios, bars
pink	yes	+	*p*- , *q*-Values
light green	yes	−	*p*- , *q*-Values

Dot Plots Dot Plots

Hendrickson *et al.*

Protein	*SgPgFn vs Sg* Log$_2$ Ratio	Log$_2$ Sum	*q*-Value	*p*-Value	Raw *SgPgFn*	Raw *Sg*	Normalized *SgPgFn*	Normalized *Sg*	Description
SGO_1626	-0.741	7.124	0.0024	0.0104	9.500	37.000	25.0040	38.5063	branched-chain amino acid ABC transporter, ATP-binding protein
					9.000	49.000	27.0073	49.0000	
SGO_1627	-1.098	7.407	0.0031	0.0142	12.000	64.500	31.5840	67.1258	branched-chain amino acid ABC transporter, ATP-binding protein
					7.500	48.500	22.5061	48.5000	
SGO_1630	-1.610	10.482	0.0001	0.0000	68.500	509.000	180.2921	529.7212	branched-chain amino acid ABC transporter, amino acid-binding protein
					57.500	547.500	172.5464	547.5000	
SGO_1632	0.368	7.633	0.0375	0.3174	12.500	37.500	32.9000	39.0266	clpP; ATP-dependent Clp protease, proteolytic subunit ClpP
					28.000	42.500	84.0226	42.5000	
SGO_1633	0.812	8.067	0.0046	0.0242	38.500	45.000	101.3321	46.8319	upp; uracil phosphoribosyltransferase
					23.500	49.500	70.5190	49.5000	
SGO_1648	1.285	8.978	0.0004	0.0007	70.500	61.500	185.5561	64.0036	ppx1; inorganic pyrophosphatase, manganese-dependent
					57.000	83.500	171.0460	83.5000	
SGO_1649	-2.213	5.763	0.0122	0.0831	2.000	26.000	5.2640	27.0585	act; pyruvate formate-lyase-activating enzyme
						22.000		22.0000	
SGO_1652	-0.012	7.118	0.0881	0.8335	13.000	28.500	34.2160	29.6602	intracellular glycosyl hydrolase
					11.500	40.500	34.5093	40.5000	
SGO_1653	-2.355	8.961	0.0004	0.0007	10.500	187.500	27.6360	195.1331	trehalose PTS enzyme II
					19.500	217.000	58.5157	217.0000	
SGO_1666	-2.475	6.630	0.0011	0.0038	3.500	33.500	9.2120	34.8638	trkA; potassium uptake protein, Trk family
					2.000	49.000	6.0016	49.0000	
SGO_1669	-0.722	7.304	0.0063	0.0352	11.000	38.500	28.9520	40.0673	ribosomal large subunit pseudouridine synthase B
					10.000	59.000	30.0081	59.0000	
SGO_1675	-0.814	6.224	0.0104	0.0676	4.000	17.500	10.5280	18.2124	HAM1 protein-like protein
					5.500	29.500	16.5044	29.5000	

Log$_2$ Ratios: -6 -4 -2 0 2 4 6

Show detected proteins only
Show all proteins
Filter by category: ABC Transporter

Proteins found: 624

Test	Cutoff
***q*-Value**	**.005**
p-Value	

Color	Signif	Direction	Applies To
red	yes	+	ratios, bars
yellow	no	n/a	bars
green	yes	–	ratios, bars
light red	yes	+	*p*- , *q*-Values
light green	yes	–	*p*- , *q*-Values

Dot Plots | Dot Plots

Protein	SgPgFn vs Sg Log$_2$ Ratio	Log$_2$ Sum	*q*-Value	*p*-Value	Raw *SgPgFn*	Raw *Sg*	Normalized *SgPgFn*	Normalized *Sg*	Description
SGO_1676	1.047	5.395	0.0034	0.0164	4.500	5.500	11.8440	5.7239	murI; glutamate racemase
					5.500	8.000	16.5044	8.0000	
SGO_1678	-0.494	4.666	0.0386	0.3278	2.500	7.500	6.5800	7.8053	lysA; diaminopimelate decarboxylase
						11.000		11.0000	
SGO_1679	-1.444	10.749	0.0001	0.0000	82.000	608.000	215.8241	632.7514	phosphotransferase system enzyme II
					82.500	625.000	247.5666	625.0000	
SGO_1680	-1.867	7.981	0.0139	0.0989	11.500	119.000	30.2680	123.8444	phosphotransferase system enzyme II
						98.500		98.5000	
SGO_1681	-1.822	10.287	0.0001	0.0001	46.000	471.500	121.0721	490.6946	PTS system, mannose/fructose/sorbose family, IID component
					52.000	481.500	156.0420	481.5000	
SGO_1683	0.372	8.211	0.0043	0.0219	34.500	59.500	90.8041	61.9222	serS; seryl-tRNA synthetase
					25.500	67.000	76.5206	67.0000	
SGO_1684	1.486	6.497	0.0013	0.0044	11.000	9.500	28.9520	9.8867	acyl-CoA dehydrogenase family
					12.500	14.000	37.5101	14.0000	
SGO_1685	3.050	8.386	0.0008	0.0023	65.500	14.500	172.3961	15.0903	putative peroxidase / antioxidase
					42.000	21.000	126.0339	21.0000	
SGO_1687	0.122	8.112	0.0045	0.0231	28.000	62.000	73.6960	64.5240	accA; acetyl-CoA carboxylase, carboxyl transferase, alpha subunit
					23.500	68.000	70.5190	68.0000	
SGO_1688	-1.406	6.366	0.0063	0.0357	5.000	20.500	13.1600	21.3345	accD; acetyl-CoA carboxylase, carboxyl transferase, beta subunit
					3.000	39.000	9.0024	39.0000	
SGO_1689	0.660	7.554	0.0016	0.0063	22.500	30.500	59.2200	31.7416	accC; acetyl-CoA carboxylase, biotin carboxylase
					18.500	41.500	55.5149	41.5000	
SGO_1691	0.766	7.873	0.0089	0.0549	24.000	55.000	63.1680	57.2390	accB; acetyl-CoA carboxylase, biotin carboxyl carrier protein
					27.500	31.500	82.5222	31.5000	

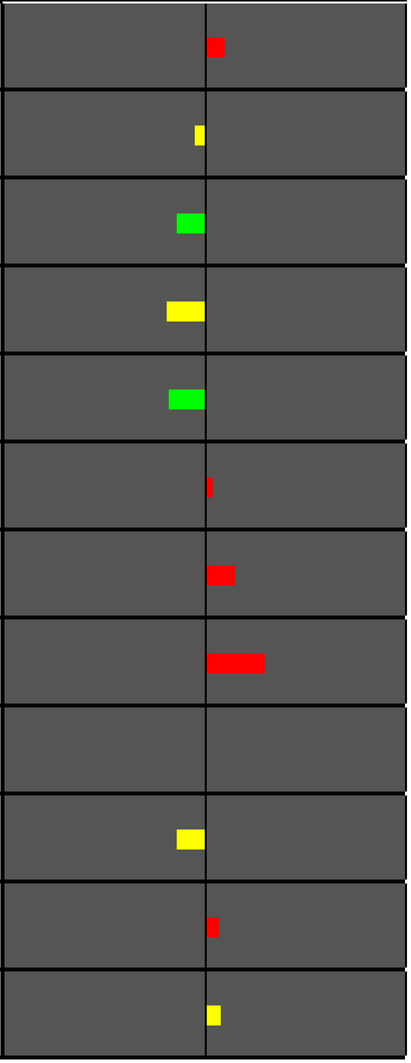

Show detected proteins only
Show all proteins
Filter by category:
ABC Transporter

Proteins found: 624

Test	Cutoff
q-Value	.005
p-Value	

Color	Signif	Direction	Applies To
red	yes	+	ratios, bars
yellow	no	n/a	bars
green	yes	−	ratios, bars
pink	yes	+	*p*- , *q*-Values
light green	yes	−	*p*- , *q*-Values

Dot Plots Dot Plots

Hendrickson *et al.*

Protein	SgPgFn vs Sg Log2 Ratio	Log2 Sum	q-Value	p-Value	Raw SgPgFn	Raw Sg	Normalized SgPgFn	Normalized Sg	Description
SGO_1692	0.957	9.329	0.0002	0.0002	78.000	101.000	205.2961	105.1117	3-oxoacyl-[acyl-carrier-protein] synthase
					73.000	113.500	219.0590	113.5000	
SGO_1693	0.329	6.844	0.0297	0.2420	15.500	26.500	40.7960	27.5788	fabG; 3-oxoacyl-(acyl-carrier-protein) reductase
					8.000	22.500	24.0065	22.5000	
SGO_1694	0.254	6.757	0.0396	0.3378	14.000	20.000	36.8480	20.8142	fabD; malonyl CoA-acyl carrier protein transacylase
					7.500	28.000	22.5061	28.0000	
SGO_1695	0.383	9.478	0.0104	0.0673	88.500	147.500	232.9321	153.5047	enoyl-acyl carrier protein(ACP) reductase
					57.500	154.000	172.5464	154.0000	
SGO_1699	0.250	4.823	0.0440	0.3827		7.500		7.8053	transcriptional regulator, MarR family
					3.500	10.000	10.5028	10.0000	
SGO_1700	-1.156	5.429	0.0016	0.0063	3.000	17.000	7.8960	17.6921	enoyl-CoA hydratase/isomerase family protein
						17.500		17.5000	
SGO_1701	0.450	7.536	0.0079	0.0461	17.500	36.500	46.0600	37.9859	aspartate kinase
					20.500	40.000	61.5166	40.0000	
SGO_1708	-1.843	9.787	0.0037	0.0183	32.500	424.000	85.5400	441.2609	amiF; Oligopeptide transport ATP-binding protein amiF
					34.000	254.500	102.0275	254.5000	
SGO_1709	-1.751	10.017	0.0001	0.0001	39.000	381.500	102.6481	397.0307	amiE; Oligopeptide transport ATP-binding protein
					45.500	400.000	136.5367	400.0000	
SGO_1710	-1.800	6.971	0.0002	0.0002	5.500	49.500	14.4760	51.5151	amiD; Oligopeptide transport system permease protein
					4.500	46.000	13.5036	46.0000	
SGO_1711	-1.236	8.671	0.0013	0.0049	20.000	121.000	52.6400	125.9259	hppB; Oligopeptide transport system permease
					23.000	160.000	69.0186	160.0000	
SGO_1712	-0.769	11.175	0.0001	0.0001	167.000	712.500	439.5443	741.5056	hppA; oligopeptide-binding lipoprotein
					138.500	715.500	415.6119	715.5000	

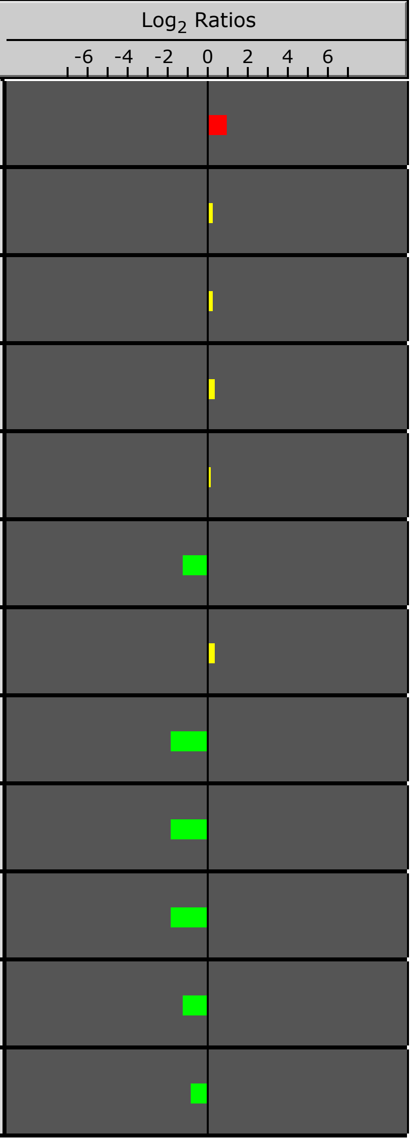

Show detected proteins only
Show all proteins
Filter by category:
ABC Transporter

Proteins found: 624

Test	Cutoff
q-Value	.005
p-Value	

Color	Signif	Direction	Applies To
red	yes	+	ratios, bars
yellow	no	n/a	bars
green	yes	−	ratios, bars
pink	yes	+	p- , q-Values
light green	yes	−	p- , q-Values

Dot Plots Dot Plots

Hendrickson *et al.*

Streptococcus gordonii

Protein	*SgPgFn vs Sg* Log$_2$ Ratio	Log$_2$ Sum	*q*-Value	*p*-Value	Raw *SgPgFn*	Raw *Sg*	Normalized *SgPgFn*	Normalized *Sg*	Description
SGO_1713	-1.142	10.780	0.0003	0.0005	101.500	615.000	267.1482	640.0364	hppG; oligopeptide-binding lipoprotein
					93.500	570.500	280.5755	570.5000	
SGO_1715	-1.403	9.537	0.0001	0.0000	36.500	263.500	96.0681	274.2270	hppH; oligopeptide-binding lipoprotein
					36.000	264.500	108.0291	264.5000	
SGO_1716	-0.817	9.616	0.0059	0.0323	70.500	215.000	185.5561	223.7526	oligopeptide binding protein
					35.000	270.500	105.0283	270.5000	
SGO_1718	-0.096	7.275	0.0867	0.8195	10.500	31.000	27.6360	32.2620	sufB-1; FeS assembly protein SufB
					16.000	47.000	48.0129	47.0000	
SGO_1720	0.593	5.293	0.0194	0.1486	5.500	5.500	14.4760	5.7239	aminotransferase, class-V
					3.000	10.000	9.0024	10.0000	
SGO_1721	0.411	7.324	0.0029	0.0128	17.000	36.500	44.7440	37.9859	sufD; FeS assembly protein SufD
					15.500	31.000	46.5125	31.0000	
SGO_1722	0.109	6.599	0.0647	0.5864	6.500	19.500	17.1080	20.2938	sufC; FeS assembly ATPase SufC
					11.500	25.000	34.5093	25.0000	
SGO_1727	-1.039	7.688	0.0002	0.0003	12.000	64.500	31.5840	67.1258	amino acid ABC transporter, amino acid-binding/permease protein
					12.000	71.500	36.0097	71.5000	
SGO_1728	-1.188	7.505	0.0032	0.0153	10.500	73.000	27.6360	75.9718	glnQ; glutamine ABC transporter ATP-binding protein
					9.000	51.000	27.0073	51.0000	
SGO_1730	-1.177	9.659	0.0006	0.0016	45.000	292.500	118.4401	304.4076	SPFH domain/Band 7 family
					43.000	256.500	129.0347	256.5000	
SGO_1731	2.608	5.540	0.0004	0.0007	7.000	2.000	18.4240	2.0814	DNA-binding response regulator
					7.000	5.000	21.0057	5.0000	
SGO_1735	-0.245	9.332	0.0086	0.0525	59.000	155.500	155.2881	161.8303	hypothetical protein SGO_1735
					46.500	188.000	139.5376	188.0000	

Log$_2$ Ratios: -6 -4 -2 0 2 4 6

Show detected proteins only
Show all proteins
Filter by category:
ABC Transporter

Proteins found: 624

Test	Cutoff
***q*-Value**	**.005**
p-Value	

	Signif	Direction	Applies To
red	yes	+	ratios, bars
yellow	no	n/a	bars
green	yes	−	ratios, bars
light red	yes	+	*p*- , *q*-Values
light green	yes	−	*p*- , *q*-Values

Dot Plots Dot Plots

Hendrickson *et al.*

Protein	SgPgFn vs Sg: Log_2 Ratio	Log_2 Sum	*q*-Value	*p*-Value	Raw: *SgPgFn*	Raw: *Sg*	Normalized: *SgPgFn*	Normalized: *Sg*	Description
SGO_1736	-0.151	5.089	0.0568	0.5072	4.000	12.500	10.5280	13.0089	alkaline shock protein
						10.500		10.5000	
SGO_1737	2.134	7.515	0.0078	0.0454		21.500		22.3753	rpmB; ribosomal protein L28
					41.500	36.000	124.5335	36.0000	
SGO_1745	1.424	12.436	0.0013	0.0044	665.500	810.500	1751.5970	843.4951	fba; fructose-1,6-bisphosphate aldolase, class II
					762.000	659.500	2286.6155	659.5000	
SGO_1748	0.167	5.622	0.0423	0.3624	6.000	11.500	15.7920	11.9682	pyrG; CTP synthase
					3.500	11.000	10.5028	11.0000	
SGO_1749	-0.386	6.448	0.0200	0.1552	8.000	19.000	21.0560	19.7735	manA; mannose-6-phosphate isomerase, class I
					5.500	30.000	16.5044	30.0000	
SGO_1755	0.304	7.053	0.0095	0.0602	13.000	25.500	34.2160	26.5381	scrK; fructokinase
					13.000	33.000	39.0105	33.0000	
SGO_1757	-0.347	8.518	0.0137	0.0974	28.000	84.500	73.6960	87.9400	glmS; glucosamine--fructose-6-phosphate aminotransferase
					29.000	118.000	87.0234	118.0000	
SGO_1763	0.099	7.963	0.0335	0.2787	25.000	53.000	65.8000	55.1576	ABC transporter, substrate-binding protein SP0092
					21.000	65.500	63.0170	65.5000	
SGO_1774	0.393	5.898	0.0210	0.1646	5.000	11.000	13.1600	11.4478	alcohol dehydrogenase, zinc-containing
					7.000	14.000	21.0057	14.0000	
SGO_1784	0.812	8.516	0.0005	0.0011	44.000	57.500	115.8081	59.8408	leuS; leucyl-tRNA synthetase
					39.000	73.500	117.0315	73.5000	
SGO_1799	0.779	8.359	0.0031	0.0142	44.000	50.500	115.8081	52.5558	endopeptidase O
					30.500	68.500	91.5246	68.5000	
SGO_1800	-2.837	8.327	0.0003	0.0005	6.500	147.500	17.1080	153.5047	troB; manganese ABC transporter, ATP-binding protein SP1648
					7.500	128.000	22.5061	128.0000	

Log_2 Ratios: -6 -4 -2 0 2 4 6

Show detected proteins only
Show all proteins
Filter by category:
ABC Transporter

Proteins found: 624

Test	Cutoff
q-Value	.005
p-Value	

Signif	Direction	Applies To
yes	+	ratios, bars
no	n/a	bars
yes	−	ratios, bars
yes	+	*p*- , *q*-Values
yes	−	*p*- , *q*-Values

Dot Plots Dot Plots

Hendrickson *et al.*

Protein	SgPgFn vs Sg Log_2 Ratio	Log_2 Sum	*q*-Value	*p*-Value	Raw *SgPgFn*	Raw *Sg*	Normalized *SgPgFn*	Normalized *Sg*	Description
SGO_1802	-3.417	11.160	0.0003	0.0003	43.000	1088.500	113.1761	1132.8124	Metal ABC transporter substrate-binding lipoprotein precursor
					28.000	958.000	84.0226	958.0000	
SGO_1803	1.530	6.761	0.0021	0.0088	12.500	13.000	32.9000	13.5292	tpx; thioredoxin peroxidase
					16.000	14.000	48.0129	14.0000	
SGO_1805	3.472	5.269	0.0288	0.2332	9.500	1.500	25.0040	1.5611	hutU; urocanate hydratase
					4.000		12.0032		
SGO_1811	0.311	3.971	0.0125	0.0864		4.500		4.6832	hutH; histidine ammonia-lyase
					2.000	5.000	6.0016	5.0000	
SGO_1822	-1.916	6.495	0.0097	0.0614	4.000	41.000	10.5280	42.6691	relA; GTP diphosphokinase
						37.000		37.0000	
SGO_1824	0.344	5.781	0.0208	0.1628	6.500	9.500	17.1080	9.8867	prmA; ribosomal protein L11 methyltransferase
					4.500	14.500	13.5036	14.5000	
SGO_1828	0.528	4.191	0.0428	0.3679		6.500		6.7646	ATPase, AAA family
					2.500	4.000	7.5020	4.0000	
SGO_1834	3.228	6.972	0.0001	0.0001	22.500	7.000	59.2200	7.2850	hypothetical protein SGO_1834
					18.000	5.000	54.0145	5.0000	
SGO_1835	2.701	4.492							hypothetical protein SGO_1835
					6.500	3.000	19.5052	3.0000	
SGO_1843	0.922	8.025	0.0010	0.0032	29.500	45.500	77.6440	47.3523	pepS; aminopeptidase PepS
					31.000	42.500	93.0250	42.5000	
SGO_1844	-0.266	5.225	0.0432	0.3728	2.500	8.000	6.5800	8.3257	cbxX/cfqX family protein
					3.500	12.000	10.5028	12.0000	
SGO_1847	-0.806	5.342	0.0155	0.1128		14.000		14.5699	polC; DNA polymerase III, alpha subunit, Gram-positive type
					3.000	17.000	9.0024	17.0000	

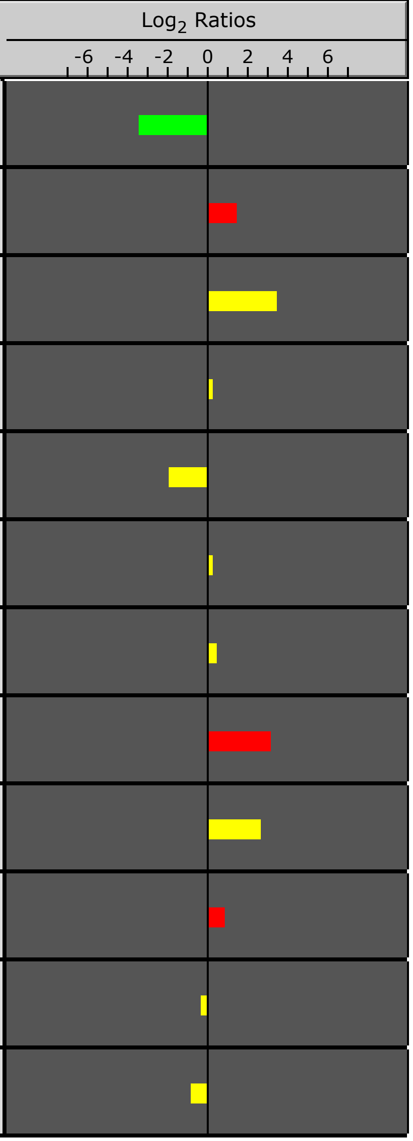

Show detected proteins only
Show all proteins
Filter by category:
ABC Transporter

Proteins found: 624

Test	Cutoff
q-Value	.005
p-Value	

	Signif	Direction	Applies To
red	yes	+	ratios, bars
yellow	no	n/a	bars
green	yes	−	ratios, bars
pink	yes	+	*p*- , *q*-Values
light green	yes	−	

Dot Plots Dot Plots

Hendrickson *et al.*

Protein	SgPgFn vs Sg Log$_2$ Ratio	Log$_2$ Sum	*q*-Value	*p*-Value	Raw *SgPgFn*	Raw *Sg*	Normalized *SgPgFn*	Normalized *Sg*	Description
SGO_1848	-3.131	7.451	0.0003	0.0006	2.500	82.000	6.5800	85.3382	lipoprotein, putative
					4.000	71.000	12.0032	71.0000	
SGO_1851	0.354	8.954	0.0037	0.0181	55.500	96.500	146.0761	100.4285	proS; prolyl-tRNA synthetase
					44.000	117.500	132.0355	117.5000	
SGO_1852	-1.808	6.568	0.0073	0.0423	4.500	38.000	11.8440	39.5470	membrane-associated zinc metalloprotease, putative
						43.500		43.5000	
SGO_1854	-1.185	7.051	0.0011	0.0035	6.000	45.500	15.7920	47.3523	uppS; undecaprenyl diphosphate synthase
					8.500	44.000	25.5069	44.0000	
SGO_1856	-0.872	6.771	0.0009	0.0027	8.500	33.500	22.3720	34.8638	ATP-dependent proteinase ATP-binding chain
					5.500	35.500	16.5044	35.5000	
SGO_1860	-1.747	8.928	0.0009	0.0028	19.500	204.500	51.3240	212.8251	5'-nucleotidase, lipoprotein e(P4) family
					20.000	163.000	60.0162	163.0000	
SGO_1861	0.305	3.026				3.500		3.6425	nusB; transcription antitermination factor NusB
					1.500		4.5012		
SGO_1862	1.008	6.883	0.0004	0.0008	14.500	21.000	38.1640	21.8549	alkaline shock protein
					13.500	17.500	40.5109	17.5000	
SGO_1863	0.360	8.507	0.0017	0.0065	40.000	72.000	105.2801	74.9311	efp; Elongation factor P (EF-P)
					33.000	84.500	99.0267	84.5000	
SGO_1864	0.554	7.258	0.0189	0.1444	22.500	33.500	59.2200	34.8638	X-Pro aminopeptidase
					11.000	26.000	33.0089	26.0000	
SGO_1865	-1.655	7.125	0.0002	0.0001	6.500	48.500	17.1080	50.4744	uvrA; excinuclease ABC, A subunit
					5.500	55.500	16.5044	55.5000	
SGO_1867	-0.450	8.165	0.0084	0.0500	27.000	85.000	71.0640	88.4603	hypothetical protein SGO_1867
					17.000	76.500	51.0137	76.5000	

Log$_2$ Ratios: -6 -4 -2 0 2 4 6

Show detected proteins only
Show all proteins
Filter by category:
ABC Transporter

Proteins found: 624

Test	Cutoff
q-Value	.005
p-Value	

	Signif	Direction	Applies To
red	yes	+	ratios, bars
yellow	no	n/a	bars
green	yes	−	ratios, bars
pink	yes	+	*p*- , *q*-Values
light green	yes	−	*p*- , *q*-Values

Dot Plots Dot Plots

Hendrickson *et al.*

Protein	*SgPgFn* vs *Sg* Log$_2$ Ratio	Log$_2$ Sum	*q*-Value	*p*-Value	Raw *SgPgFn*	Raw *Sg*	Normalized *SgPgFn*	Normalized *Sg*	Description
SGO_1879	0.700	7.966	0.0195	0.1505	27.500	28.000	72.3800	29.1399	rpsR; ribosomal protein S18
					25.500	72.000	76.5206	72.0000	
SGO_1880	0.689	8.219	0.0009	0.0027	35.500	49.000	93.4361	50.9948	ssb-1; single-strand binding protein
					30.000	63.500	90.0242	63.5000	
SGO_1881	0.575	9.632	0.0010	0.0031	95.500	146.500	251.3561	152.4640	rpsF; ribosomal protein S6
					74.500	166.000	223.5602	166.0000	
SGO_1882	0.473	5.296	0.0155	0.1125	4.000	6.000	10.5280	6.2443	folE; GTP cyclohydrolase I
					4.000	10.500	12.0032	10.5000	
SGO_1885	0.330	9.842	0.0005	0.0013	94.500	198.500	248.7241	206.5809	groL; 60 kDa chaperonin/groEL protein
					87.500	200.000	262.5707	200.0000	
SGO_1886	-0.907	5.912	0.0036	0.0171	3.000	20.000	7.8960	20.8142	groES; chaperonin, 10 kDa
					4.500	18.000	13.5036	18.0000	
SGO_1892	-0.914	6.496	0.0045	0.0231	7.500	24.500	19.7400	25.4974	PTS system, fructose(mannose)-specific IIB
					4.000	33.000	12.0032	33.0000	
SGO_1898	0.145	7.601	0.0155	0.1136	20.500	42.000	53.9560	43.7098	glutamyl aminopeptidase
					16.000	48.500	48.0129	48.5000	
SGO_1901	0.799	6.435	0.0009	0.0026	10.000	17.000	26.3200	17.6921	tRNA binding domain
					9.500	14.000	28.5077	14.0000	
SGO_1902	0.981	9.865	0.0016	0.0059	131.000	137.000	344.7922	142.5772	ssb; single-stranded DNA-binding protein
					91.500	170.500	274.5739	170.5000	
SGO_1903	0.042	8.146	0.0725	0.6714	24.000	64.000	63.1680	66.6054	ATP-dependent Zn protease
					27.000	72.500	81.0218	72.5000	
SGO_1914	1.000	3.170							folP; dihydropteroate synthase
					2.000	3.000	6.0016	3.0000	

Log$_2$ Ratios: -6 -4 -2 0 2 4 6

Show detected proteins only
Show all proteins
Filter by category:
ABC Transporter

Proteins found: 624

Test	Cutoff
q-Value	.005
p-Value	

	Signif	Direction	Applies To
red	yes	+	ratios, bars
yellow	no	n/a	bars
green	yes	−	ratios, bars
pink	yes	+	*p*- , *q*-Values
light green	yes	−	*p*- , *q*-Values

Dot Plots | Dot Plots

Hendrickson *et al.*

Protein	*SgPgFn vs Sg* Log_2 Ratio	Log_2 Sum	*q*-Value	*p*-Value	Raw *SgPgFn*	Raw *Sg*	Normalized *SgPgFn*	Normalized *Sg*	Description
SGO_1916	0.287	8.469	0.0046	0.0243	38.000	70.500	100.0161	73.3700	ackA; acetate kinase
					31.500	86.500	94.5254	86.5000	
SGO_1917	1.025	5.153	0.0001	0.0001	4.500	6.000	11.8440	6.2443	hypothetical protein SGO_1917
					4.000	5.500	12.0032	5.5000	
SGO_1924	-2.489	8.495	0.0001	0.0001	8.500	142.000	22.3720	147.7808	comYA; competence protein comYA
					11.000	157.500	33.0089	157.5000	
SGO_1926	0.220	10.594	0.0018	0.0071	153.500	333.500	404.0122	347.0767	rpoC; DNA-directed RNA polymerase, beta chain
					142.500	367.000	427.6151	367.0000	
SGO_1927	0.140	10.512	0.0173	0.1287	135.500	347.000	356.6362	361.1262	rpoB; DNA-directed RNA polymerase, beta subunit
					136.500	333.000	409.6102	333.0000	
SGO_1928	-3.385	7.438	0.0087	0.0531	3.000	73.500	7.8960	76.4922	pbp1b; penicillin-binding protein 1B
						89.000		89.0000	
SGO_1929	0.438	8.971	0.0031	0.0143	50.500	97.000	132.9161	100.9488	tyrS; tyrosyl-tRNA synthetase
					52.000	112.000	156.0420	112.0000	
SGO_1934	-1.691	5.485	0.0037	0.0184		19.000		19.7735	copper-translocating P-type ATPase
					2.000	19.000	6.0016	19.0000	
SGO_1936	-2.739	8.890	0.0001	0.0000	10.500	193.500	27.6360	201.3773	adcA; metal-binding (Mn) permease precursor, lipoprotein
					11.500	211.000	34.5093	211.0000	
SGO_1958	1.594	9.873	0.0005	0.0012	122.500	127.000	322.4202	132.1701	rplQ; ribosomal protein L17
					127.000	102.000	381.1026	102.0000	
SGO_1959	0.337	9.318	0.0063	0.0352	66.000	152.000	173.7121	158.1879	rpoA; DNA-directed RNA polymerase, alpha subunit
					60.500	125.000	181.5489	125.0000	
SGO_1960	0.585	8.257	0.0012	0.0041	36.000	64.500	94.7521	67.1258	rpsK; ribosomal protein S11
					29.500	55.500	88.5238	55.5000	

Log_2 Ratios: -6 -4 -2 0 2 4 6

Show detected proteins only
Show all proteins
Filter by category:
ABC Transporter

Proteins found: 624

Test	Cutoff
q-Value	.005
p-Value	

Color	Signif	Direction	Applies To
red	yes	+	ratios, bars
yellow	no	n/a	bars
green	yes	−	ratios, bars
pink	yes	+	*p*- , *q*-Values
light green	yes	−	*p*- , *q*-Values

Dot Plots | Dot Plots

Hendrickson *et al.*

Protein	*SgPgFn* vs *Sg* Log$_2$ Ratio	Log$_2$ Sum	*q*-Value	*p*-Value	Raw *SgPgFn*	Raw *Sg*	Normalized *SgPgFn*	Normalized *Sg*	Description
SGO_1961	0.380	9.056	0.0015	0.0056	54.500	115.500	143.4441	120.2020	rpsM; ribosomal protein S13p/S18e
					52.500	111.000	157.5424	111.0000	
SGO_1964	0.431	8.993	0.0013	0.0045	52.500	106.500	138.1801	110.8356	adk; Adenylate kinase (ATP-AMP transphosphorylase)
					51.500	106.000	154.5416	106.0000	
SGO_1965	-1.253	6.773	0.0003	0.0003	6.000	39.000	15.7920	40.5877	Preprotein translocase secY subunit
					5.500	36.500	16.5044	36.5000	
SGO_1966	1.278	8.907	0.0009	0.0028	57.500	64.500	151.3401	67.1258	rplO; ribosomal protein L15
					63.000	72.500	189.0509	72.5000	
SGO_1967	0.975	8.055	0.0016	0.0058	29.500	42.000	77.6440	43.7098	50S ribosomal protein L30 -related protein
					33.000	45.500	99.0267	45.5000	
SGO_1968	0.201	10.039	0.0195	0.1505	110.500	264.500	290.8362	275.2677	rpsE; ribosomal protein S5
					90.000	216.000	270.0727	216.0000	
SGO_1969	1.285	10.176	0.0002	0.0003	156.500	182.000	411.9082	189.4092	rplR; ribosomal protein L18
					135.500	149.000	406.6094	149.0000	
SGO_1970	-0.307	10.335	0.0120	0.0805	126.500	349.000	332.9482	363.2077	BL10; 50S ribosomal protein L6
					82.500	347.500	247.5666	347.5000	
SGO_1971	1.268	11.111	0.0015	0.0056	339.500	326.500	893.5645	339.7917	rpsH; ribosomal protein S8
					224.500	305.500	673.6813	305.5000	
SGO_1973	0.582	10.121	0.0019	0.0079	137.500	202.500	361.9002	210.7437	BL6; 50S ribosomal protein L5
					102.000	234.500	306.0824	234.5000	
SGO_1974	0.659	9.413	0.0039	0.0194	71.500	147.000	188.1881	152.9843	rplX; ribosomal protein L24
					76.000	112.500	228.0614	112.5000	
SGO_1975	1.293	9.123	0.0005	0.0012	69.000	80.500	181.6081	83.7771	rplN; ribosomal protein L14
					71.500	77.500	214.5577	77.5000	

Show detected proteins only
Show all proteins
Filter by category:
ABC Transporter

Proteins found: 624

Test	Cutoff
q-Value	.005
p-Value	

Color	Signif	Direction	Applies To
red	yes	+	ratios, bars
yellow	no	n/a	bars
green	yes	−	ratios, bars
pink	yes	+	*p*- , *q*-Values
light green	yes	−	*p*- , *q*-Values

Dot Plots Dot Plots

Protein	SgPgFn vs Sg: Log_2 Ratio	Log_2 Sum	*q*-Value	*p*-Value	Raw: *SgPgFn*	Raw: *Sg*	Normalized: *SgPgFn*	Normalized: *Sg*	Description
SGO_1976	1.712	8.214	0.0030	0.0137	51.500	45.500	135.5481	47.3523	BS16; 30S ribosomal protein
					30.000	24.000	90.0242	24.0000	
SGO_1977	0.375	6.717	0.0222	0.1763	9.000	25.000	23.6880	26.0177	rpmC; ribosomal protein L29
					12.000	19.500	36.0097	19.5000	
SGO_1978	0.912	8.468	0.0040	0.0201	42.500	42.500	111.8601	44.2302	rplP; ribosomal protein L16
					38.500	82.500	115.5311	82.5000	
SGO_1979	-0.865	10.382	0.0004	0.0010	82.500	429.000	217.1401	446.4644	rpsC; ribosomal protein S3
					85.500	414.000	256.5691	414.0000	
SGO_1980	1.192	9.751	0.0003	0.0005	108.000	134.000	284.2562	139.4551	rplV; ribosomal protein L22
					105.000	123.000	315.0848	123.0000	
SGO_1981	-0.058	9.485	0.0642	0.5800	62.000	159.000	163.1841	165.4728	rpsS; ribosomal protein S19
					62.500	200.500	187.5505	200.5000	
SGO_1982	0.562	9.205	0.0042	0.0214	61.000	99.500	160.5521	103.5506	rplB; ribosomal protein L2
					63.500	135.500	190.5513	135.5000	
SGO_1983	-0.221	8.939	0.0161	0.1179	38.000	130.000	100.0161	135.2923	rplW; ribosomal protein L23
					42.500	128.000	127.5343	128.0000	
SGO_1984	1.443	10.038	0.0004	0.0009	136.000	153.000	357.9522	159.2286	rplD; ribosomal protein L4/L1 family
					136.500	124.500	409.6102	124.5000	
SGO_1985	2.068	9.860	0.0001	0.0000	139.000	80.000	365.8482	83.2568	rplC; ribosomal protein L3
					128.000	96.000	384.1034	96.0000	
SGO_1986	0.996	8.217	0.0008	0.0022	38.500	56.000	101.3321	58.2797	rpsJ; ribosomal protein S10
					32.000	42.000	96.0258	42.0000	
SGO_1989	1.422	9.815	0.0003	0.0004	117.500	109.500	309.2602	113.9577	purA; adenylosuccinate synthetase
					115.500	131.000	346.5933	131.0000	

Log_2 Ratios: -6 -4 -2 0 2 4 6

Show detected proteins only
Show all proteins
Filter by category:
ABC Transporter

Proteins found: 624

Test	Cutoff
q-Value	.005
p-Value	

Color	Signif	Direction	Applies To
red	yes	+	ratios, bars
yellow	no	n/a	bars
green	yes	−	ratios, bars
pink	yes	+	*p*- , *q*-Values
light green	yes	−	*p*- , *q*-Values

Dot Plots | Dot Plots

Protein	*SgPgFn vs Sg* Log$_2$ Ratio	Log$_2$ Sum	*q*-Value	*p*-Value	Raw *SgPgFn*	Raw *Sg*	Normalized *SgPgFn*	Normalized *Sg*	Description
SGO_1990	1.153	4.157	0.0196	0.1515	3.500	3.000	9.2120	3.1221	glutamate--cysteine ligase, putative/amino acid ligase, putative
						5.500		5.5000	
SGO_1993	-0.658	7.153	0.0087	0.0534	8.500	35.000	22.3720	36.4248	possible transcriptional regulator
					11.000	50.500	33.0089	50.5000	
SGO_1995	-0.155	5.158	0.0782	0.7315	4.000	16.500	10.5280	17.1717	MutT/nudix family protein
						8.000		8.0000	
SGO_1998	-0.905	6.939	0.0016	0.0059	9.500	41.000	25.0040	42.6691	clpB; ATP-dependent Clp proteinase, ATP-binding chain
					6.000	37.000	18.0048	37.0000	
SGO_2000	0.572	11.072	0.0014	0.0052	260.000	443.500	684.3204	461.5547	tsf; translation elongation factor Ts
					201.000	404.500	603.1623	404.5000	
SGO_2001	0.252	10.944	0.0063	0.0353	191.000	455.000	502.7123	473.5229	rpsB; ribosomal protein S2
					189.500	425.500	568.6531	425.5000	
SGO_2005	-4.242	9.625	0.0001	0.0001	9.000	379.000	23.6880	394.4289	LPXTG cell wall surface protein
					5.500	355.000	16.5044	355.0000	
SGO_2007	0.048	7.214	0.0366	0.3079	15.000	35.500	39.4800	36.9452	nusG; transcription termination/antitermination factor NusG
					12.000	36.000	36.0097	36.0000	
SGO_2024	-1.812	7.317	0.0008	0.0021	6.000	52.500	15.7920	54.6373	Extracellular polysaccharide biosynthesis
					6.500	69.500	19.5052	69.5000	
SGO_2025	-1.636	6.420	0.0005	0.0011	3.000	30.000	7.8960	31.2213	wze; putative autophosphorylating protein tyrosine kinase
					4.500	33.000	13.5036	33.0000	
SGO_2033	-1.279	7.627	0.0002	0.0003	10.000	69.500	26.3200	72.3293	nrdD; ribonucleoside-triphosphate reductase
					10.500	67.500	31.5085	67.5000	
SGO_2041	0.282	3.847	0.0764	0.7107	2.000	3.000	5.2640	3.1221	conserved hypothetical protein TIGR00250
						6.000		6.0000	

Log$_2$ Ratios: -6 -4 -2 0 2 4 6

Show detected proteins only
Show all proteins
Filter by category:
ABC Transporter

Proteins found: 624

Test	Cutoff
***q*-Value**	**.005**
p-Value	

	Signif	Direction	Applies To
red	yes	+	ratios, bars
yellow	no	n/a	bars
green	yes	−	ratios, bars
light red	yes	+	*p*- , *q*-Values
light green	yes	−	*p*- , *q*-Values

Dot Plots Dot Plots

Streptococcus gordonii

Protein	SgPgFn vs Sg Log$_2$ Ratio	SgPgFn vs Sg Log$_2$ Sum	SgPgFn vs Sg *q*-Value	SgPgFn vs Sg *p*-Value	Raw *SgPgFn*	Raw *Sg*	Normalized *SgPgFn*	Normalized *Sg*	Description
SGO_2042	2.436	8.867	0.0006	0.0013	83.500	40.500	219.7721	42.1487	Bacterial protein of unknown function (DUF965) superfamily
					58.000	31.000	174.0468	31.0000	
SGO_2045	0.911	10.917	0.0007	0.0018	254.000	294.500	668.5284	306.4890	recA; recA protein
					197.500	365.500	592.6595	365.5000	
SGO_2046	-0.649	5.179	0.0131	0.0925	3.000	8.000	7.8960	8.3257	cinA; competence induced protein
					2.000	14.000	6.0016	14.0000	
SGO_2053	-1.264	6.077	0.0018	0.0070	3.500	19.500	9.2120	20.2938	DNA mismatch repair protein hexB
					3.500	27.500	10.5028	27.5000	
SGO_2056	-0.777	6.303	0.0176	0.1321	3.500	18.500	9.2120	19.2531	mutS; DNA mismatch repair protein MutS
					7.000	29.500	21.0057	29.5000	
SGO_2058	0.830	10.111	0.0006	0.0014	143.000	185.500	376.3762	193.0516	argS; arginyl-tRNA synthetase
					110.500	204.500	331.5892	204.5000	
SGO_2060	-0.098	7.496	0.0584	0.5233	17.500	37.000	46.0600	38.5063	aspS-1; aspartyl-tRNA synthetase
					13.500	55.500	40.5109	55.5000	
SGO_2062	0.880	8.587	0.0018	0.0073	51.000	55.000	134.2321	57.2390	hisS; histidyl-tRNA synthetase
					38.000	79.000	114.0307	79.0000	
SGO_2064	1.405	8.804	0.0002	0.0003	64.500	54.500	169.7641	56.7187	ilvD; dihydroxy-acid dehydratase
					51.500	66.000	154.5416	66.0000	
SGO_2066	1.056	7.749	0.0037	0.0182	31.500	42.000	82.9080	43.7098	rpmG; ribosomal protein L33
					20.500	27.000	61.5166	27.0000	
SGO_2070	-0.962	6.219	0.0204	0.1592	5.500	12.500	14.4760	13.0089	hypothetical protein SGO_2070
					3.000	38.000	9.0024	38.0000	
SGO_2085	-0.164	8.270	0.0050	0.0265	28.500	75.500	75.0120	78.5736	purB; adenylosuccinate lyase
					23.500	84.500	70.5190	84.5000	

Log$_2$ Ratios: -6 -4 -2 0 2 4 6

Show detected proteins only
Show all proteins
Filter by category: ABC Transporter

Proteins found: 624

Test	Cutoff
q-Value	.005
p-Value	

Color	Signif	Direction	Applies To
red	yes	+	ratios, bars
yellow	no	n/a	bars
green	yes	−	ratios, bars
pink	yes	+	*p*- , *q*-Values
light green	yes	−	*p*- , *q*-Values

Dot Plots Dot Plots

Protein	*SgPgFn vs Sg* Log$_2$ Ratio	Log$_2$ Sum	*q*-Value	*p*-Value	Raw *SgPgFn*	Raw *Sg*	Normalized *SgPgFn*	Normalized *Sg*	Description
SGO_2097	-6.574	9.979	0.0032	0.0151	2.000	493.500	5.2640	513.5902	comA; ATP-binding Transport protein ComA
						490.000		490.0000	
SGO_2098	0.782	11.174	0.0034	0.0159	319.500	388.500	840.9245	404.3157	rpsD; ribosomal protein S4
					208.500	440.000	625.6684	440.0000	
SGO_2100	2.702	8.891	0.0001	0.0000	76.500	32.000	201.3481	33.3027	ABC transporter substrate-binding protein
					70.000	30.000	210.0565	30.0000	
SGO_2104	-1.198	9.753	0.0005	0.0011	59.000	291.000	155.2881	302.8465	srtB; sortase B
					36.500	295.500	109.5295	295.5000	
SGO_2105	-6.361	12.333	0.0002	0.0003	11.500	2230.000	30.2680	2320.7824	abpA; amylase-binding protein AbpA
					10.500	2777.000	31.5085	2777.0000	
SGO_2106	-0.130	8.283	0.0006	0.0015	28.000	79.000	73.6960	82.2161	ribose-phosphate diphosphokinase
					25.000	80.500	75.0202	80.5000	
SGO_2133	-1.206	10.318	0.0002	0.0002	76.500	411.000	201.3481	427.7317	Cell division protein ftsH-like protein
					61.500	462.500	184.5497	462.5000	
SGO_2134	-0.203	7.684	0.0476	0.4172	21.500	62.500	56.5880	65.0444	hpt; hypoxanthine phosphoribosyltransferase
					13.000	45.000	39.0105	45.0000	
SGO_2142	0.662	8.157	0.0025	0.0106	30.000	48.500	78.9600	50.4744	GTP-binding protein
					32.000	60.000	96.0258	60.0000	
SGO_2145	-0.254	9.586	0.0013	0.0045	67.000	208.000	176.3441	216.4676	comE; competence response regulator ComE
					58.000	201.500	174.0468	201.5000	
SGO_2146	-2.665	6.947	0.0073	0.0420		58.000		60.3612	comD; histidine protein kinase ComD
					3.000	54.000	9.0024	54.0000	
SGO_2150	-2.772	7.969	0.0002	0.0001	8.000	108.500	21.0560	112.9170	degP; serine protease
					4.000	104.500	12.0032	104.5000	

Log$_2$ Ratios: -6 -4 -2 0 2 4 6

Show detected proteins only
Show all proteins
Filter by category:
ABC Transporter

Proteins found: 624

Test	Cutoff
q-Value	.005
p-Value	

Color	Signif	Direction	Applies To
red	yes	+	ratios, bars
yellow	no	n/a	bars
green	yes	–	ratios, bars
pink	yes	+	*p*- , *q*-Values
light green	yes	–	*p*- , *q*-Values

Dot Plots Dot Plots
