# Supplementary material for: Proteomics of Streptococcus gordonii within a model developing oral microbial community
Source: BMC Microbiol. 2012 Sep 18;12:211. doi: 10.1186/1471-2180-12-211 (PMC3534352; doi:10.1186/1471-2180-12-211)
Supplement: Additional file 5 — SgPg_vs_SgFn. A more detailed presentation of the relative abundance ratios for the comparison of SgPg and SgFn, including both raw and normalized spectral counts. Red and green highlights are used as in Additional file 1. [file 1471-2180-12-211-S5.pdf]

Streptococcus gordonii

Hackett Laboratory UW

Protein	SgPg vs SgFn Log_2 Ratio	Log_2 Sum	*q*-Value	*p*-Value	Raw *SgPg*	Raw *SgFn*	Normalized *SgPg*	Normalized *SgFn*	Description
**SGO_0001**	0.148	6.069	0.0468	0.3152	19.000	9.500	19.8503	14.5072	dnaA; chromosomal replication initiator protein DnaA
					15.500	10.500	15.5000	17.2653	
**SGO_0002**	-0.090	6.961	0.0299	0.1843	30.000	20.000	31.3426	30.5414	dnaN; DNA polymerase III, beta subunit
					29.000	20.500	29.0000	33.7085	
**SGO_0004**	-0.318	7.080	0.0514	0.3501	36.500	29.500	38.1335	45.0485	putative lipoprotein
					22.500	18.000	22.5000	29.5977	
**SGO_0006**	0.304	7.378	0.0161	0.0882	46.000	28.000	48.0586	42.7579	ABC transporter, ATP-binding protein
					43.500	19.500	43.5000	32.0642	
**SGO_0007**	1.447	6.385	0.0006	0.0008	29.000	9.000	30.2978	13.7436	trpS; tryptophanyl-tRNA synthetase
					30.500	5.500	30.5000	9.0437	
**SGO_0008**	0.755	9.426	0.0009	0.0014	214.000	77.000	223.5770	117.5843	inosine-5'-monophosphate dehydrogenase
					208.000	84.500	208.0000	138.9447	
**SGO_0011**	-0.201	4.002	0.0922	0.6812	6.000		6.2685		proteinase, M16 family
					4.000	3.500	4.0000	5.7551	
**SGO_0015**	0.514	3.987	0.0097	0.0458	5.500		5.7461		ABC transporter (ATP-binding protein)
					6.000	2.500	6.0000	4.1108	
**SGO_0022**	-2.421	7.479	0.0000	0.0000	13.000	50.000	13.5818	76.3534	trmU; tRNA (5-methylaminomethyl-2-thiouridylate)-methyltransferase
					14.500	45.000	14.5000	73.9942	
**SGO_0025**	0.908	5.502	0.0157	0.0846	17.500	3.500	18.2832	5.3447	gidA; glucose inhibited division protein A
					11.000	6.500	11.0000	10.6881	
**SGO_0027**	0.756	6.646	0.0011	0.0021	29.000	11.000	30.2978	16.7978	rplI; ribosomal protein L9
					32.500	12.500	32.5000	20.5539	
**SGO_0028**	0.991	4.452	0.0119	0.0590	5.000	2.000	5.2238	3.0541	dnaC; replicative DNA helicase
					9.500	2.500	9.5000	4.1108	

Log_2 Ratios: -6 -4 -2 0 2 4 6

Show detected proteins only
Show all proteins
Filter by category:
ABC Transporter

Proteins found: 713

Test	Cutoff
***q*-Value**	**.005**
p-Value	

Colour	Signif	Direction	Applies To
red	yes	+	ratios, bars
yellow	no	n/a	bars
green	yes	−	ratios, bars
pink	yes	+	*p*- , *q*-Values
light green	yes	−	*p*- , *q*-Values

Dot Plots | Dot Plots

Hendrickson *et al.*

Streptococcus gordonii

Hackett Laboratory UW

Protein	SgPg vs SgFn Log_2 Ratio	Log_2 Sum	*q*-Value	*p*-Value	Raw *SgPg*	Raw *SgFn*	Normalized *SgPg*	Normalized *SgFn*	Description
SGO_0030	-1.184	7.625	0.0001	0.0000	30.000	44.500	31.3426	67.9545	aspB; aspartate transaminase
					29.000	42.000	29.0000	69.0613	
SGO_0032	0.968	6.584	0.0006	0.0007	30.500	9.500	31.8649	14.5072	plsX; fatty acid/phospholipid synthesis protein PlsX
					31.500	11.000	31.5000	18.0875	
SGO_0035	-3.076	4.751	0.0137	0.0713		7.500		11.4530	phosphoribosylformylglycinamidine synthase
					1.500	8.500	1.5000	13.9767	
SGO_0042	-0.563	5.766	0.0019	0.0045	11.500	11.000	12.0147	16.7978	transcription regulator, GntR family
					10.000	9.500	10.0000	15.6210	
SGO_0054	-1.327	8.019	0.0002	0.0001	33.500	59.000	34.9992	90.0970	dltA; D-alanine-activating enzyme
					39.000	58.000	39.0000	95.3703	
SGO_0057	-0.280	6.397	0.0215	0.1239	18.500	17.500	19.3279	26.7237	dltD protein
					18.500	12.000	18.5000	19.7318	
SGO_0059	3.589	11.564	0.0007	0.0009	1491.000	93.500	1557.7259	142.7809	pXO1; hypothetical protein SGO_0059
					1233.500	56.500	1233.5000	92.9039	
SGO_0063	0.204	6.135	0.0400	0.2615	15.500	9.500	16.1937	14.5072	hypothetical protein SGO_0063
					21.500	11.000	21.5000	18.0875	
SGO_0064	1.133	7.144	0.0027	0.0079	40.000	16.000	41.7901	24.4331	FtsK/SpoIIIE family protein
					55.500	12.000	55.5000	19.7318	
SGO_0065	0.298	6.991	0.0071	0.0294	34.500	17.000	36.0440	25.9602	hypothetical protein SGO_0065
					34.000	19.000	34.0000	31.2420	
SGO_0066	1.034	4.293	0.0278	0.1684	6.500	2.500	6.7909	3.8177	D-3-phosphoglycerate dehydrogenase, putative
					9.000		9.0000		
SGO_0067	2.110	6.156	0.0015	0.0035	32.000	5.000	33.4321	7.6353	protein with prophage function domain
					24.500	3.500	24.5000	5.7551	

Show detected proteins only
Show all proteins
Filter by category:
ABC Transporter

Proteins found: 713

Test	Cutoff
***q*-Value**	**.005**
p-Value	

	Signif	Direction	Applies To
red	yes	+	ratios, bars
yellow	no	n/a	bars
green	yes	−	ratios, bars
pink	yes	+	***p*- , *q*-Values**
light green	yes	−	***p*- , *q*-Values**

Dot Plots Dot Plots

Streptococcus gordonii

Hackett Laboratory UW

Summary Table | SgFn vs Sg | SgPg vs Sg | SgPgFn vs Sg | SgPg vs SgFn | SgPgFn vs SgFn | SgPgFn vs SgPg | Coverage

Protein	*SgPg vs SgFn* Log$_2$ Ratio	Log$_2$ Sum	*q*-Value	*p*-Value	Raw *SgPg*	Raw *SgFn*	Normalized *SgPg*	Normalized *SgFn*	Description
**SGO_0068**	1.338	5.123	0.0075	0.0324	13.500		14.1042		lipoprotein, putative
					15.000	3.500	15.0000	5.7551	
**SGO_0078**	0.966	4.360	0.0387	0.2518	9.500		9.9251		hypothetical protein SGO_0078
					6.500	2.500	6.5000	4.1108	
**SGO_0080**	0.527	6.346	0.0125	0.0629	24.500	13.500	25.5964	20.6154	hypothetical protein SGO_0080
					22.000	8.000	22.0000	13.1545	
**SGO_0081**	-0.142	4.788	0.0913	0.6734	8.000	4.500	8.3580	6.8718	hypothetical protein SGO_0081
					5.000	4.500	5.0000	7.3994	
**SGO_0095**	-1.125	6.431	0.0009	0.0016	12.500	21.000	13.0594	32.0684	mccF; microcin immunity protein MccF, putative
					14.000	16.500	14.0000	27.1312	
**SGO_0099**	-0.483	4.566	0.0416	0.2746	4.000	2.500	4.1790	3.8177	pulA-2; pullulanase, type I
					5.000	6.500	5.0000	10.6881	
**SGO_0100**	-0.717	3.403							maltose operon transcription repressor
					4.000	4.000	4.0000	6.5773	
**SGO_0104**	-0.447	8.985	0.0077	0.0334	100.000	108.000	104.4752	164.9234	Maltose/maltodextrin-binding protein precursor
					109.000	78.000	109.0000	128.2566	
**SGO_0105**	-0.225	6.182	0.0330	0.2061	16.500	15.000	17.2384	22.9060	malQ; 4-alpha-glucanotransferase
					16.000	10.000	16.0000	16.4432	
**SGO_0106**	-0.879	8.496	0.0019	0.0044	61.500	84.500	64.2523	129.0373	glgP-2; maltodextrin phosphorylase
					62.500	64.000	62.5000	105.2362	
**SGO_0110**	0.668	3.090			5.000		5.2238		phosphotyrosine protein phosphatase
						2.000		3.2886	
**SGO_0113**	0.584	11.427	0.0014	0.0031	838.500	375.000	876.0249	572.6507	acdH; alcohol-acetaldehyde dehydrogenase
					777.000	321.500	777.0000	528.6476	

Log$_2$ Ratios: -6 -4 -2 0 2 4 6

- Show detected proteins only
- Show all proteins
- Filter by category: ABC Transporter

Proteins found: 713

Test	Cutoff
***q*-Value**	**.005**
p-Value	

	Signif	Direction	Applies To
(red)	yes	+	ratios, bars
(yellow)	no	n/a	bars
(green)	yes	−	ratios, bars
(pink)	yes	+	*p*- , *q*-Values
(light green)	yes	−	*p*- , *q*-Values

Dot Plots Dot Plots

Protein	*SgPg vs SgFn* Log2 Ratio	Log2 Sum	*q*-Value	*p*-Value	Raw *SgPg*	Raw *SgFn*	Normalized *SgPg*	Normalized *SgFn*	Description
SGO_0134	0.434	4.222	0.0641	0.4482	5.000		5.2238		acetyltransferase, GNAT family
					8.500	3.000	8.5000	4.9329	
SGO_0135	-0.937	7.410	0.0031	0.0095	25.500	41.500	26.6412	63.3733	v-type sodium ATP synthase, subunit A
					31.500	29.500	31.5000	48.5073	
SGO_0136	-0.613	7.410	0.0019	0.0047	29.500	35.500	30.8202	54.2109	v-type sodium ATP synthase, chain B
					36.500	29.500	36.5000	48.5073	
SGO_0139	0.074	7.146	0.0368	0.2375	36.000	21.500	37.6111	32.8320	thrC; threonine synthase
					35.000	22.000	35.0000	36.1750	
SGO_0144	-1.478	3.796	0.0265	0.1585	2.000	4.500	2.0895	6.8718	hypothetical protein SGO_0144
						3.000		4.9329	
SGO_0145	-0.841	7.372	0.0061	0.0248	27.000	41.500	28.2083	63.3733	polI; DNA polymerase I
					30.500	26.500	30.5000	43.5744	
SGO_0146	-0.397	6.110	0.0195	0.1112	16.000	15.000	16.7160	22.9060	CoA-binding domain protein
					13.000	10.000	13.0000	16.4432	
SGO_0152	0.622	5.762	0.0322	0.1998	14.500	10.500	15.1489	16.0342	tgt; queuine tRNA-ribosyltransferase
					16.500	4.000	16.5000	6.5773	
SGO_0154	-1.270	10.934	0.0011	0.0022	307.000	493.000	320.7390	752.8447	pgi; glucose-6-phosphate isomerase
					254.000	382.500	254.0000	628.9508	
SGO_0158	-1.423	8.300	0.0006	0.0007	37.500	70.000	39.1782	106.8948	2,3,4,5-tetrahydropyridine-2-carboxylate N-succinyltransferase, putative
					46.500	74.500	46.5000	122.5015	
SGO_0159	-1.718	3.164			2.000	4.500	2.0895	6.8718	hippurate hydrolase
SGO_0163	0.182	7.353	0.0069	0.0286	41.000	26.500	42.8348	40.4673	galU; UTP-glucose-1-phosphate uridylyltransferase
					44.000	22.000	44.0000	36.1750	

Log2 Ratios: -6 -4 -2 0 2 4 6

Show detected proteins only
Show all proteins

Filter by category: ABC Transporter

Proteins found: 713

Test	Cutoff
***q*-Value**	**.005**
p-Value	

	Signif	Direction	Applies To
(red)	yes	+	ratios, bars
(yellow)	no	n/a	bars
(green)	yes	−	ratios, bars
(pink)	yes	+	*p*- , *q*-Values
(light green)	yes	−	*p*- , *q*-Values

Dot Plots | Dot Plots

Hendrickson *et al.*

Streptococcus gordonii

Hackett Laboratory UW

Protein	*SgPg vs SgFn* Log$_2$ Ratio	Log$_2$ Sum	*q*-Value	*p*-Value	Raw *SgPg*	Raw *SgFn*	Normalized *SgPg*	Normalized *SgFn*	Description
SGO_0164	-0.577	7.010	0.0080	0.0354	28.000	22.000	29.2531	33.5955	Glycerol-3-phosphate dehydrogenase [NAD(P)+] (NAD(P)H-dependent glycerol-3-phosphate dehydrogenase)
					22.500	26.500	22.5000	43.5744	
SGO_0169	-1.427	6.200	0.0032	0.0101	11.500	20.500	12.0147	31.3049	dut; dUTP diphosphatase
					8.000	13.500	8.0000	22.1983	
SGO_0171	0.587	4.795	0.0400	0.2623	8.500	4.500	8.8804	6.8718	radA; DNA repair protein RadA
					12.000		12.0000		
SGO_0173	-1.884	6.016	0.0008	0.0013	7.500	15.000	7.8356	22.9060	Carbonic anhydrase
					6.000	17.000	6.0000	27.9534	
SGO_0174	0.356	8.459	0.0157	0.0841	94.500	60.000	98.7291	91.6241	gltX; glutamyl-tRNA synthetase
					97.500	39.000	97.5000	64.1283	
SGO_0180	0.815	6.068	0.0151	0.0803	26.500	6.500	27.6859	9.9259	jag; hypothetical protein SGO_0180
					15.500	8.500	15.5000	13.9767	
SGO_0188	-0.729	4.120	0.0340	0.2137		5.000		7.6353	hydrolase, TatD family
					4.000	3.500	4.0000	5.7551	
SGO_0193	0.840	4.614	0.0309	0.1910	6.500	1.500	6.7909	2.2906	ksgA; dimethyladenosine transferase
					8.000	4.500	8.0000	7.3994	
SGO_0197	-0.890	4.539	0.0020	0.0048	3.500	4.500	3.6566	6.8718	predicted ribosome small subunit-dependent GTPase A
					4.500	5.000	4.5000	8.2216	
SGO_0198	-1.704	8.123	0.0002	0.0001	32.000	67.000	33.4321	102.3136	rpe; ribulose-phosphate 3-epimerase
					32.000	67.500	32.0000	110.9913	
SGO_0200	0.255	6.525	0.0010	0.0018	24.500	13.500	25.5964	20.6154	competence-induced protein Ccs50
					24.500	13.000	24.5000	21.3761	
SGO_0201	-1.470	7.726	0.0022	0.0060	26.000	43.000	27.1636	65.6639	cmp-binding-factor 1
					28.500	55.000	28.5000	90.4374	

Log$_2$ Ratios: -6 -4 -2 0 2 4 6

Show detected proteins only
Show all proteins
Filter by category:
ABC Transporter

Proteins found: 713

Test	Cutoff
q-Value	.005
p-Value	

	Signif	Direction	Applies To
red	yes	+	ratios, bars
yellow	no	n/a	bars
green	yes	−	ratios, bars
pink	yes	+	*p*- , *q*-Values
light green	yes	−	*p*- , *q*-Values

Dot Plots Dot Plots

Streptococcus gordonii

Hackett Laboratory UW

Protein	SgPg vs SgFn				Raw		Normalized		Description
	Log_2 Ratio	Log_2 Sum	*q*-Value	*p*-Value	*SgPg*	*SgFn*	*SgPg*	*SgFn*	
SGO_0202	-0.250	5.128	0.0384	0.2496	7.000	5.000	7.3133	7.6353	pur operon repressor
					8.500	7.000	8.5000	11.5102	
SGO_0204	0.174	7.649	0.0829	0.6053	56.500	39.500	59.0285	60.3192	rpsL; ribosomal protein S12
					46.000	21.500	46.0000	35.3528	
SGO_0205	0.345	9.103	0.0281	0.1707	124.000	92.500	129.5493	141.2538	rpsG; ribosomal protein S7
					178.000	61.500	178.0000	101.1254	
SGO_0206	0.424	12.143	0.0008	0.0013	1277.000	652.000	1334.1488	995.6486	fusA; translation elongation factor G
					1258.000	569.500	1258.0000	936.4379	
SGO_0207	-0.118	12.434	0.0006	0.0008	1267.000	934.000	1323.7013	1426.2819	gap; glyceraldehyde-3-phosphate dehydrogenase, type I
					1330.000	884.000	1330.0000	1453.5753	
SGO_0209	0.736	13.083	0.0007	0.0009	2477.500	1110.000	2588.3741	1695.0460	pgk; phosphoglycerate kinase
					2836.000	949.000	2836.0000	1560.4558	
SGO_0210	-4.474	6.540	0.0009	0.0016	1.500	33.500	1.5671	51.1568	sspA; streptococcal surface protein A
					2.500	23.000	2.5000	37.8193	
SGO_0215	-0.240	8.195	0.0085	0.0389	70.000	51.500	73.1327	78.6440	glnA; glutamine synthetase, type I
					61.500	48.500	61.5000	79.7493	
SGO_0219	0.373	7.721	0.0007	0.0008	57.000	29.000	59.5509	44.2850	metallo-beta-lactamase superfamily protein 1
					59.500	29.000	59.5000	47.6852	
SGO_0220	-0.171	4.418	0.0848	0.6214	5.500	5.000	5.7461	7.6353	Protein of unknown function (DUF1447) superfamily
					8.000		8.0000		
SGO_0223	0.669	3.791				3.500		5.3447	glycoproteinase family protein
					8.500		8.5000		
SGO_0230	0.940	5.270	0.0117	0.0564	12.500	6.500	13.0594	9.9259	Protein of unknown function, DUF536 family
					11.500	2.500	11.5000	4.1108	

Log_2 Ratios: -6 -4 -2 0 2 4 6

Show detected proteins only
Show all proteins
Filter by category:
ABC Transporter

Proteins found: 713

Test	Cutoff
q-Value	.005
p-Value	

	Signif	Direction	Applies To
red	yes	+	ratios, bars
yellow	no	n/a	bars
green	yes	–	ratios, bars
pink	yes	+	*p*- , *q*-Values
light green	yes	–	*p*- , *q*-Values

Dot Plots Dot Plots

Hendrickson *et al.*

Protein	SgPg vs SgFn Log_2 Ratio	Log_2 Sum	*q*-Value	*p*-Value	Raw *SgPg*	Raw *SgFn*	Normalized *SgPg*	Normalized *SgFn*	Description
**SGO_0234**	-0.442	7.280	0.0043	0.0153	35.000	30.500	36.5663	46.5756	pepX; X-Pro dipeptidyl-peptidase
					29.500	26.000	29.5000	42.7522	
**SGO_0237**	0.508	5.557	0.0255	0.1510	12.500	4.500	13.0594	6.8718	ccpA; CcpA protein (proteinase)
					14.000	8.000	14.0000	13.1545	
**SGO_0243**	-1.190	4.998	0.0013	0.0029	4.500	6.500	4.7014	9.9259	hydroxymethylglutaryl-CoA reductase, degradative
					5.000	7.500	5.0000	12.3324	
**SGO_0244**	0.032	5.664	0.1189	0.9011	11.500	9.500	12.0147	14.5072	hydroxymethylglutaryl-CoA synthase
					13.500	6.500	13.5000	10.6881	
**SGO_0247**	-0.854	10.354	0.0067	0.0274	213.500	223.500	223.0546	341.2998	pfl; formate acetyltransferase
					237.500	308.500	237.5000	507.2715	
**SGO_0252**	-1.020	4.417	0.0086	0.0395	4.500	5.500	4.7014	8.3989	possible TetR-type transcriptional regulator
					2.500	3.500	2.5000	5.7551	
**SGO_0255**	0.297	5.938	0.0082	0.0371	16.000	10.000	16.7160	15.2707	Signal peptidase I
					17.000	7.500	17.0000	12.3324	
**SGO_0260**	0.597	6.310	0.0062	0.0249	21.500	8.500	22.4622	12.9801	DNA mismatch binding protein MutS2
					25.000	11.500	25.0000	18.9096	
**SGO_0262**	0.261	6.793	0.0115	0.0553	30.500	18.000	31.8649	27.4872	dipeptidase
					28.500	14.000	28.5000	23.0204	
**SGO_0263**	0.431	7.051	0.0237	0.1383	34.500	14.000	36.0440	21.3790	trx-1; thioredoxin
					39.000	22.000	39.0000	36.1750	
**SGO_0272**	-0.367	5.966	0.0163	0.0892	13.000	13.500	13.5818	20.6154	hypothetical protein SGO_0272
					13.500	9.000	13.5000	14.7988	
**SGO_0276**	-0.334	9.070	0.0083	0.0379	114.000	108.500	119.1018	165.6869	gdhA; glutamate dehydrogenase (NADP)
					118.000	82.000	118.0000	134.8339	

Log_2 Ratios: -6 -4 -2 0 2 4 6

Show detected proteins only
Show all proteins
Filter by category:
ABC Transporter

Proteins found: 713

Test	Cutoff
***q*-Value**	**.005**
p-Value	

	Signif	Direction	Applies To
(red)	yes	+	ratios, bars
(yellow)	no	n/a	bars
(green)	yes	–	ratios, bars
(pink)	yes	+	*p*- , *q*-Values
(light green)	yes	–	*p*- , *q*-Values

Dot Plots Dot Plots

Protein	SgPg vs SgFn Log$_2$ Ratio	Log$_2$ Sum	*q*-Value	*p*-Value	Raw *SgPg*	Raw *SgFn*	Normalized *SgPg*	Normalized *SgFn*	Description
SGO_0277	-0.766	5.384	0.0153	0.0815	9.000	6.500	9.4028	9.9259	pyrA; Dihydroorotate dehydrogenase
					6.000	10.000	6.0000	16.4432	
SGO_0280	-1.269	6.326	0.0124	0.0620	14.000	25.000	14.6265	38.1767	trzA; ethylammeline chlorohydrolase
					8.500	11.500	8.5000	18.9096	
SGO_0288	-1.232	3.610	0.0111	0.0536	3.000		3.1343		GDSL-like lipase/acylhydrolase
					2.500	4.000	2.5000	6.5773	
SGO_0291	0.297	6.175	0.0019	0.0045	18.500	11.000	19.3279	16.7978	copper-translocating P-type ATPase
					20.500	9.500	20.5000	15.6210	
SGO_0292	2.395	8.289	0.0002	0.0001	120.000	15.500	125.3703	23.6696	spxB; pyruvate oxidase
					137.500	16.000	137.5000	26.3091	
SGO_0310	-0.916	4.329	0.0290	0.1782	4.000	4.500	4.1790	6.8718	metE; 5-methyltetrahydropteroyltriglutamate--homocysteine S-methyltransferase
						5.500		9.0437	
SGO_0312	-0.479	11.127	0.0008	0.0013	441.500	444.000	461.2582	678.0184	xfp; D-xylulose 5-phosphate/D-fructose 6-phosphate phosphoketolase
					472.500	380.000	472.5000	624.8401	
SGO_0321	-0.690	6.785	0.0021	0.0052	18.000	23.500	18.8055	35.8861	polypeptide deformylase
					23.500	19.500	23.5000	32.0642	
SGO_0327	-1.654	2.700			1.500		1.5671		Lipopolysaccharide N-acetylglucosaminyltransferase
						3.000		4.9329	
SGO_0333	0.234	7.759	0.0419	0.2768	55.000	39.500	57.4614	60.3192	rpsO; ribosomal protein S15
					58.500	24.500	58.5000	40.2857	
SGO_0339	-3.474	5.190	0.0118	0.0574	1.500	10.500	1.5671	16.0342	hypothetical protein SGO_0339
						11.500		18.9096	
SGO_0342	-0.629	8.075	0.0021	0.0050	46.000	56.500	48.0586	86.2794	pepF-2; oligoendopeptidase
					58.000	47.000	58.0000	77.2828	

Log$_2$ Ratios: -6 -4 -2 0 2 4 6

Show detected proteins only
Show all proteins
Filter by category:
ABC Transporter

Proteins found: 713

Test	Cutoff
q-Value	.005
p-Value	

Color	Signif	Direction	Applies To
red	yes	+	ratios, bars
yellow	no	n/a	bars
green	yes	–	ratios, bars
pink	yes	+	*p*- , *q*-Values
light green	yes	–	*p*- , *q*-Values

Dot Plots Dot Plots

Streptococcus gordonii

Protein	SgPg vs SgFn Log$_2$ Ratio	Log$_2$ Sum	*q*-Value	*p*-Value	Raw *SgPg*	Raw *SgFn*	Normalized *SgPg*	Normalized *SgFn*	Description	Log$_2$ Ratios (-6 -4 -2 0 2 4 6)
SGO_0344	-0.343	8.646	0.0046	0.0170	85.500	79.000	89.3263	120.6384	pnpA; polyribonucleotide nucleotidyltransferase	
					87.000	63.000	87.0000	103.5919		
SGO_0348	-0.942	3.810	0.0043	0.0151	3.500	4.500	3.6566	6.8718	reductase	
					3.500		3.5000			
SGO_0349	-0.796	6.568	0.0072	0.0301	16.500	16.000	17.2384	24.4331	cysS; cysteinyl-tRNA synthetase	
					17.000	22.000	17.0000	36.1750		
SGO_0352	-0.396	7.112	0.0042	0.0144	31.500	25.000	32.9097	38.1767	ABC transporter, ATP-binding protein SP1580	
					27.000	24.500	27.0000	40.2857		
SGO_0353	-0.501	4.341	0.0414	0.2728	5.000	2.500	5.2238	3.8177	transport protein	
					3.000	5.000	3.0000	8.2216		
SGO_0355	0.236	5.496	0.0349	0.2222	10.000	6.000	10.4475	9.1624	RNA methyltransferase, TrmH family, group 3	
					14.000	7.000	14.0000	11.5102		
SGO_0357	0.351	7.343	0.0025	0.0070	46.000	23.000	48.0586	35.1226	degV; DegV family fatty acid binding protein	
					43.000	22.000	43.0000	36.1750		
SGO_0358	-0.321	9.085	0.0082	0.0369	117.500	108.500	122.7584	165.6869	rplM; ribosomal protein L13	
					118.000	83.000	118.0000	136.4782		
SGO_0359	0.184	7.477	0.0730	0.5246	43.000	34.500	44.9244	52.6839	rpsI; ribosomal protein S9	
					48.500	19.500	48.5000	32.0642		
SGO_0361	-0.849	4.222	0.0132	0.0679	4.000	3.000	4.1790	4.5812	immunity repressor protein	
					2.500	4.500	2.5000	7.3994		
SGO_0372	-0.545	6.289	0.0048	0.0177	15.000	17.000	15.6713	25.9602	malate oxidoreductase	
					16.000	12.500	16.0000	20.5539		
SGO_0374	-0.908	6.283	0.0019	0.0047	12.000	15.000	12.5370	22.9060	Response regulator of the LytR/AlgR family	
					14.500	17.000	14.5000	27.9534		

Show detected proteins only
Show all proteins
Filter by category:
ABC Transporter

Proteins found: 713

Test	Cutoff
***q*-Value**	.005
p-Value	

	Signif	Direction	Applies To
red	yes	+	ratios, bars
yellow	no	n/a	bars
green	yes	−	ratios, bars
pink	yes	+	*p*- , *q*-Values
light green	yes	−	*p*- , *q*-Values

Dot Plots Dot Plots

Hendrickson *et al.*

Protein	SgPg vs SgFn Log$_2$ Ratio	Log$_2$ Sum	*q*-Value	*p*-Value	Raw *SgPg*	Raw *SgFn*	Normalized *SgPg*	Normalized *SgFn*	Description
SGO_0384	-0.094	5.081	0.0912	0.6706	6.500	4.500	6.7909	6.8718	putative carboxylate-amine/thiol ligase
					9.500	6.500	9.5000	10.6881	
SGO_0390	-0.374	8.298	0.0161	0.0876	72.000	66.500	75.2222	101.5501	glycerol-3-phosphate dehydrogenase (NAD (P)+)
					61.500	46.500	61.5000	76.4607	
SGO_0392	-0.946	6.501	0.0011	0.0021	13.000	18.500	13.5818	28.2508	phosphoglycerate mutase
					17.500	19.000	17.5000	31.2420	
SGO_0398	-0.794	4.991	0.0183	0.1028	7.500	5.000	7.8356	7.6353	ABC transporter ATP-binding protein
					4.000	7.500	4.0000	12.3324	
SGO_0401	-0.561	6.510	0.0076	0.0327	14.500	16.500	15.1489	25.1966	grpE; co-chaperone GrpE
					22.000	17.500	22.0000	28.7755	
SGO_0402	-0.741	11.257	0.0002	0.0001	424.500	492.500	443.4974	752.0812	dnaK; DnaK chaperone protein
					473.000	474.000	473.0000	779.4058	
SGO_0404	-0.036	6.073	0.0706	0.5026	16.000	10.500	16.7160	16.0342	dnaJ; DnaJ chaparone protein
					16.500	11.000	16.5000	18.0875	
SGO_0407	-1.686	3.963	0.0161	0.0876	2.000	4.000	2.0895	6.1083	truA; tRNA pseudouridine synthase A
						4.500		7.3994	
SGO_0409	-5.919	8.682	0.0007	0.0010	3.500	115.000	3.6566	175.6129	pyridoxine kinase
					3.000	139.000	3.0000	228.5599	
SGO_0411	-4.261	8.407	0.0072	0.0303	8.000	61.000	8.3580	93.1512	conserved hypothetical protein TIGR01440
					7.000	140.500	7.0000	231.0264	
SGO_0412	0.673	10.654	0.0006	0.0008	463.000	216.500	483.7204	330.6103	tig; trigger factor
					506.000	177.000	506.0000	291.0439	
SGO_0413	-0.184	7.251	0.0657	0.4612	27.000	25.000	28.2083	38.1767	DNA-directed RNA polymerase delta chain
					44.000	25.500	44.0000	41.9301	

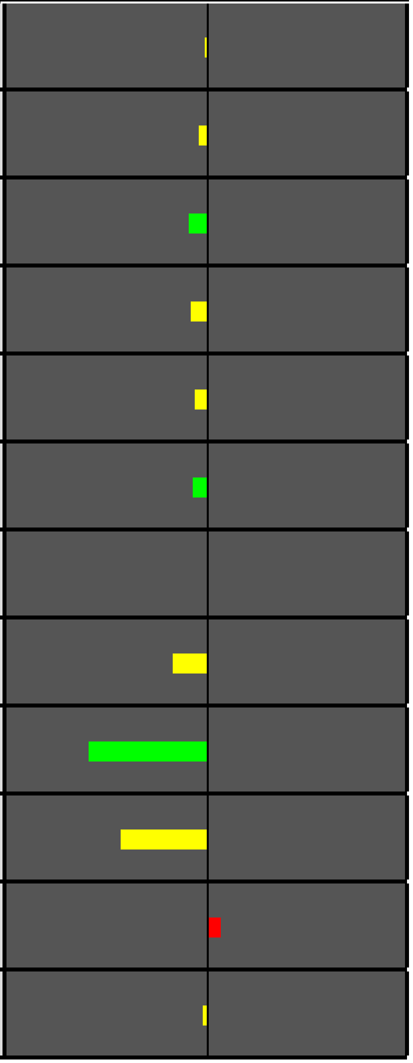

Show detected proteins only
Show all proteins
Filter by category:
ABC Transporter

Proteins found: 713

Test	Cutoff
q-Value	.005
p-Value	

	Signif	Direction	Applies To
red	yes	+	ratios, bars
yellow	no	n/a	bars
green	yes	−	ratios, bars
pink	yes	+	*p*- , *q*-Values
light green	yes	−	

Dot Plots Dot Plots

Protein	SgPg vs SgFn Log2 Ratio	Log2 Sum	*q*-Value	*p*-Value	Raw *SgPg*	Raw *SgFn*	Normalized *SgPg*	Normalized *SgFn*	Description
**SGO_0415**	0.090	9.530	0.0349	0.2222	184.000	125.500	192.2344	191.6471	secA; preprotein translocase, SecA subunit
					188.500	101.500	188.5000	166.8981	
**SGO_0416**	-0.702	7.428	0.0028	0.0085	37.000	35.000	38.6558	53.4474	phospho-2-dehydro-3-deoxyheptonate aldolase
					27.500	32.000	27.5000	52.6181	
**SGO_0418**	-1.405	5.217	0.0004	0.0004	5.500	8.500	5.7461	12.9801	alr; alanine racemase
					4.500	8.500	4.5000	13.9767	
**SGO_0425**	-0.910	3.396			3.500	4.500	3.6566	6.8718	ansB; asparaginase
**SGO_0426**	-1.201	5.513	0.0025	0.0071	7.500	9.000	7.8356	13.7436	Cof family protein
					6.000	11.000	6.0000	18.0875	
**SGO_0427**	-1.809	7.352	0.0008	0.0012	16.000	37.500	16.7160	57.2651	universal stress protein family
					19.500	42.500	19.5000	69.8834	
**SGO_0429**	-0.121	8.678	0.0795	0.5757	112.500	67.000	117.5346	102.3136	aspartate transaminase
					80.500	66.500	80.5000	109.3470	
**SGO_0431**	-0.204	4.493	0.0326	0.2026	6.500		6.7909		GTP-sensing transcriptional pleiotropic repressor codY
					7.500	5.000	7.5000	8.2216	
**SGO_0432**	1.873	6.100	0.0126	0.0633	31.000		32.3873		entB; isochorismatase family protein
					28.000	5.000	28.0000	8.2216	
**SGO_0434**	0.066	6.756	0.1206	0.9195	23.000	13.000	24.0293	19.8519	aspS-2; aspartyl-tRNA synthetase
					30.500	20.500	30.5000	33.7085	
**SGO_0435**	0.055	5.782	0.0639	0.4458	12.500	8.500	13.0594	12.9801	gatC; glutamyl-tRNA(Gln) amidotransferase, C subunit
					15.000	8.500	15.0000	13.9767	
**SGO_0436**	0.568	8.373	0.0013	0.0027	99.500	46.000	103.9529	70.2451	gatA; glutamyl-tRNA(Gln) amidotransferase, A subunit
					94.000	38.500	94.0000	63.3062	

Log2 Ratios: -6 -4 -2 0 2 4 6

- Show detected proteins only
- Show all proteins
- Filter by category: ABC Transporter

Proteins found: 713

Test	Cutoff
***q*-Value**	.005
p-Value	

	Signif	Direction	Applies To
red	yes	+	ratios, bars
yellow	no	n/a	bars
green	yes	−	ratios, bars
pink	yes	+	*p*- , *q*-Values
light green	yes	−	*p*- , *q*-Values

Dot Plots Dot Plots

Protein	*SgPg vs SgFn* Log_2 Ratio	Log_2 Sum	*q*-Value	*p*-Value	Raw *SgPg*	Raw *SgFn*	Normalized *SgPg*	Normalized *SgFn*	Description
**SGO_0437**	0.601	8.467	0.0037	0.0121	95.000	52.000	99.2515	79.4076	gatB; glutamyl-tRNA(Gln) amidotransferase, B subunit
					113.500	37.500	113.5000	61.6618	
**SGO_0440**	-2.526	6.782	0.0004	0.0003	9.000	28.500	9.4028	43.5215	L-iditol 2-dehydrogenase BH3949
					7.000	30.500	7.0000	50.1516	
**SGO_0445**	-0.617	6.781	0.0019	0.0044	23.000	22.500	24.0293	34.3590	GTP-binding protein
					19.500	19.500	19.5000	32.0642	
**SGO_0447**	-1.713	5.261	0.0044	0.0155	6.000	11.500	6.2685	17.5613	nadD; nicotinate (nicotinamide) nucleotide adenylyltransferase
					3.000	7.000	3.0000	11.5102	
**SGO_0448**	-1.326	5.495	0.0019	0.0046	6.500	12.000	6.7909	18.3248	conserved hypothetical protein TIGR00488
					6.000	8.500	6.0000	13.9767	
**SGO_0450**	-0.570	4.989	0.0013	0.0029	6.500	6.500	6.7909	9.9259	iojap-related protein
					6.000	5.500	6.0000	9.0437	
**SGO_0454**	0.070	7.311	0.1040	0.7740	37.500	21.000	39.1782	32.0684	conserved hypothetical protein TIGR01033
					41.500	28.000	41.5000	46.0408	
**SGO_0455**	2.829	5.127	0.0111	0.0532	14.500	1.500	15.1489	2.2906	lipoprotein, putative
					17.500		17.5000		
**SGO_0457**	1.359	7.198	0.0002	0.0001	48.500	13.500	50.6705	20.6154	ABC transporter, substrate-binding protein SP0148
					55.000	12.500	55.0000	20.5539	
**SGO_0458**	0.889	8.708	0.0015	0.0034	128.000	56.500	133.7283	86.2794	hlpA; lipoprotein
					136.500	37.500	136.5000	61.6618	
**SGO_0459**	-1.303	4.977	0.0023	0.0064	5.500	6.500	5.7461	9.9259	succinyl-diaminopimelate desuccinylase
					3.500	7.500	3.5000	12.3324	
**SGO_0460**	0.425	5.479	0.0025	0.0072	13.000	6.000	13.5818	9.1624	ABC transporter, ATP-binding protein SP0151
					12.000	6.000	12.0000	9.8659	

Log_2 Ratios: -6 -4 -2 0 2 4 6

Show detected proteins only
Show all proteins
Filter by category:
ABC Transporter

Proteins found: 713

Test	Cutoff
***q*-Value**	.005
p-Value	

	Signif	Direction	Applies To
red	yes	+	ratios, bars
yellow	no	n/a	bars
green	yes	−	ratios, bars
pink	yes	+	*p*- , *q*-Values
light green	yes	−	*p*- , *q*-Values

Dot Plots Dot Plots

Protein	SgPg vs SgFn Log2 Ratio	Log2 Sum	*q*-Value	*p*-Value	Raw SgPg	Raw SgFn	Normalized SgPg	Normalized SgFn	Description
SGO_0468	-1.602	6.634	0.0051	0.0196	11.000	31.000	11.4923	47.3391	hypothetical protein SGO_0468
					12.500	17.000	12.5000	27.9534	
SGO_0476	2.521	5.363	0.0119	0.0583	19.500		20.3727		rhodanese family protein
					17.500	2.000	17.5000	3.2886	
SGO_0480	-4.542	9.506	0.0050	0.0191	15.000	146.500	15.6713	223.7155	hypothetical protein SGO_0480
					12.500	289.000	12.5000	475.2073	
SGO_0483	0.245	6.162	0.0367	0.2361	15.000	11.000	15.6713	16.7978	hypothetical protein SGO_0483
					23.500	9.500	23.5000	15.6210	
SGO_0488	0.160	4.799	0.1077	0.8086	6.000	2.000	6.2685	3.0541	ABC transporter, ATP-binding protein SP0483
					7.000	7.000	7.0000	11.5102	
SGO_0491	-1.048	4.414	0.0033	0.0108	2.500	5.000	2.6119	7.6353	gidB; methyltransferase GidB
					4.500	4.000	4.5000	6.5773	
SGO_0494	-2.354	7.679	0.0013	0.0025	16.500	64.500	17.2384	98.4959	lemA; LemA-like protein
					16.000	44.500	16.0000	73.1721	
SGO_0495	-0.838	4.717	0.0118	0.0577	5.500	7.000	5.7461	10.6895	htpx; heat shock protein
						6.000		9.8659	
SGO_0501	2.358	5.796	0.0062	0.0253	25.000		26.1188		Uncharacterized ACR, COG1399
					24.500	3.000	24.5000	4.9329	
SGO_0502	1.192	8.502	0.0023	0.0064	133.000	44.000	138.9521	67.1910	floL; flotillin-like protein
					112.000	27.000	112.0000	44.3965	
SGO_0503	-0.832	11.295	0.0004	0.0003	420.000	545.500	438.7960	833.0158	gnd; 6-phosphogluconate dehydrogenase, decarboxylating
					465.000	472.000	465.0000	776.1171	
SGO_0505	0.210	7.872	0.0512	0.3479	53.500	42.500	55.8943	64.9004	PTS system, IIBC component
					69.000	27.000	69.0000	44.3965	

Log2 Ratios: -6 -4 -2 0 2 4 6

Show detected proteins only
Show all proteins
Filter by category: ABC Transporter

Proteins found: 713

Test	Cutoff
q-Value / *p*-Value	.005

	Signif	Direction	Applies To
red	yes	+	ratios, bars
yellow	no	n/a	bars
green	yes	−	ratios, bars
pink	yes	+	*p*- , *q*-Values
light green	yes	−	*p*- , *q*-Values

Dot Plots Dot Plots

Hendrickson *et al.*

Protein	SgPg vs SgFn Log$_2$ Ratio	Log$_2$ Sum	*q*-Value	*p*-Value	Raw *SgPg*	Raw *SgFn*	Normalized *SgPg*	Normalized *SgFn*	Description
SGO_0508	-0.087	5.228	0.0712	0.5085	11.000	8.500	11.4923	12.9801	nrdR; transcriptional regulator, NrdR family
					13.000		13.0000		
SGO_0510	0.348	6.032	0.0275	0.1659	19.000	11.500	19.8503	17.5613	dnaI; primosomal protein DnaI
					16.500	7.000	16.5000	11.5102	
SGO_0511	-1.112	5.300	0.0054	0.0209	6.000	7.000	6.2685	10.6895	NADPH-flavin oxidoreductase -like protein
					6.000	10.000	6.0000	16.4432	
SGO_0512	0.329	7.571	0.0043	0.0149	50.000	30.000	52.2376	45.8121	GTP-binding protein engA
					53.500	23.500	53.5000	38.6414	
SGO_0515	-0.507	8.288	0.0038	0.0126	61.500	66.000	64.2523	100.7865	murC; UDP-N-acetylmuramate--alanine ligase
					64.500	50.500	64.5000	83.0380	
SGO_0518	1.227	6.988	0.0002	0.0001	44.000	12.500	45.9691	19.0884	aminodeoxychorismate lyase-like protein
					43.000	11.500	43.0000	18.9096	
SGO_0519	0.476	6.929	0.0132	0.0679	38.000	19.500	39.7006	29.7778	greA; transcription elongation factor greA
					31.000	13.000	31.0000	21.3761	
SGO_0521	-0.509	4.706	0.0488	0.3295	9.000	7.000	9.4028	10.6895	Membrane protein oxaA 2 precursor
					6.000		6.0000		
SGO_0523	-1.995	6.366	0.0002	0.0001	6.500	21.500	6.7909	32.8320	spoU rRNA Methylase family protein
					10.000	20.000	10.0000	32.8863	
SGO_0526	0.264	5.998	0.0611	0.4254	18.000	7.000	18.8055	10.6895	ilvB; acetolactate synthase, large subunit, biosynthetic type
					15.500	11.500	15.5000	18.9096	
SGO_0527	-0.403	5.078	0.0196	0.1122	5.500	6.000	5.7461	9.1624	ilvN; acetolactate synthase, small subunit
					9.000	6.000	9.0000	9.8659	
SGO_0528	-0.445	8.520	0.0078	0.0339	79.000	77.500	82.5354	118.3478	ilvC; ketol-acid reductoisomerase
					72.500	57.000	72.5000	93.7260	

Log$_2$ Ratios: -6 -4 -2 0 2 4 6

Show detected proteins only
Show all proteins
Filter by category:
ABC Transporter

Proteins found: 713

Test	Cutoff
***q*-Value**	.005
p-Value	

	Signif	Direction	Applies To
red	yes	+	ratios, bars
yellow	no	n/a	bars
green	yes	−	ratios, bars
pink	yes	+	*p*-, *q*-Values
light green	yes	−	*p*-, *q*-Values

Dot Plots Dot Plots

Hendrickson *et al.*

Protein	SgPg vs SgFn Log$_2$ Ratio	Log$_2$ Sum	*q*-Value	*p*-Value	Raw *SgPg*	Raw *SgFn*	Normalized *SgPg*	Normalized *SgFn*	Description
SGO_0529	-0.507	4.959	0.0259	0.1541	5.000	4.500	5.2238	6.8718	ilvA; threonine dehydratase
					7.500	7.000	7.5000	11.5102	
SGO_0530	-0.849	4.253	0.0220	0.1276	2.000	3.000	2.0895	4.5812	Cof family protein
					5.000	4.500	5.0000	7.3994	
SGO_0533	-1.611	3.019				4.000		6.1083	conserved hypothetical protein TIGR00150
					2.000		2.0000		
SGO_0535	0.257	4.384	0.0652	0.4574	6.000	4.000	6.2685	6.1083	putative transcriptional regulator LytR
					8.500		8.5000		
SGO_0537	-0.118	6.485	0.0126	0.0637	20.500	16.000	21.4174	24.4331	HIT family protein
					21.500	13.500	21.5000	22.1983	
SGO_0538	-1.633	5.245	0.0054	0.0210	3.500	7.000	3.6566	10.6895	ABC transporter, ATP-binding protein SP0522
					5.500	11.000	5.5000	18.0875	
SGO_0540	-0.986	7.348	0.0004	0.0003	25.500	37.000	26.6412	56.5015	hypothetical protein SGO_0540
					28.000	31.500	28.0000	51.7960	
SGO_0541	-1.057	4.580	0.0057	0.0224	2.500	5.500	2.6119	8.3989	methyltransferase, putative
					5.500	4.500	5.5000	7.3994	
SGO_0543	1.107	6.991	0.0000	0.0000	42.000	13.500	43.8796	20.6154	nusA; transcription termination factor NusA
					43.000	12.000	43.0000	19.7318	
SGO_0546	0.170	8.171	0.0265	0.1583	77.500	40.500	80.9683	61.8463	infB; Translation initiation factor IF-2
					71.500	45.000	71.5000	73.9942	
SGO_0548	1.438	4.402	0.0127	0.0642	8.000		8.3580		Na/Pi-cotransporter family protein
					9.500	2.000	9.5000	3.2886	
SGO_0549	3.912	5.200			33.000	1.500	34.4768	2.2906	nagA; N-acetylglucosamine-6-phosphate deacetylase

Log$_2$ Ratios: -6 -4 -2 0 2 4 6

Show detected proteins only
Show all proteins
Filter by category:
ABC Transporter

Proteins found: 713

Test	Cutoff
q-Value	.005
p-Value	

	Signif	Direction	Applies To
red	yes	+	ratios, bars
yellow	no	n/a	bars
green	yes	–	ratios, bars
pink	yes	+	*p*- , *q*-Values
light green	yes	–	*p*- , *q*-Values

Dot Plots Dot Plots

Hendrickson *et al.*

Protein	SgPg vs SgFn Log2 Ratio	Log2 Sum	q-Value	p-Value	Raw SgPg	Raw SgFn	Normalized SgPg	Normalized SgFn	Description
SGO_0552	-0.752	7.028	0.0022	0.0058	22.500	29.500	23.5069	45.0485	oxidoreductase, aldo/keto reductase family
					25.000	22.500	25.0000	36.9971	
SGO_0554	-1.018	6.007	0.0028	0.0083	8.500	12.500	8.8804	19.0884	hsdR; type I site-specific deoxyribonuclease
					12.500	14.500	12.5000	23.8426	
SGO_0558	-0.137	3.826	0.1100	0.8286	5.500		5.7461		hypothetical protein SGO_0558
					3.500	3.000	3.5000	4.9329	
SGO_0560	2.120	4.582	0.0118	0.0572	11.000		11.4923		hsdM; type I restriction-modification system, M subunit
					10.000	1.500	10.0000	2.4665	
SGO_0565	-1.797	8.825	0.0009	0.0015	45.500	128.000	47.5362	195.4648	adhA; alcohol dehydrogenase
					53.500	95.500	53.5000	157.0322	
SGO_0568	1.448	6.209	0.0014	0.0031	24.000	8.500	25.0741	12.9801	glyQ; glycyl-tRNA synthetase, alpha subunit
					28.500	4.500	28.5000	7.3994	
SGO_0569	0.084	8.115	0.0814	0.5918	67.000	38.000	69.9984	58.0286	glyS; glycyl-tRNA synthetase, beta subunit
					72.000	47.000	72.0000	77.2828	
SGO_0573	-0.176	6.126	0.0588	0.4067	16.000	15.500	16.7160	23.6696	mraW; S-adenosyl-methyltransferase MraW
					15.500	8.500	15.5000	13.9767	
SGO_0577	0.804	6.796	0.0075	0.0319	35.500	17.500	37.0887	26.7237	ATP-dependent RNA helicase
					32.500	9.000	32.5000	14.7988	
SGO_0579	0.718	4.668	0.0075	0.0323	9.000	3.000	9.4028	4.5812	amino acid ABC transporter ATP binding protein
					6.500	3.000	6.5000	4.9329	
SGO_0581	1.121	5.490	0.0046	0.0168	12.500	3.500	13.0594	5.3447	trxB; thioredoxin-disulfide reductase
					17.500	5.500	17.5000	9.0437	
SGO_0582	-1.346	8.699	0.0006	0.0007	47.000	96.000	49.1034	146.5986	nicotinate phosphoribosyltransferase, putative
					69.500	91.500	69.5000	150.4549	

Log2 Ratios: -6 -4 -2 0 2 4 6

Show detected proteins only
Show all proteins
Filter by category:
ABC Transporter

Proteins found: 713

Test	Cutoff
q-Value	.005
p-Value	

Color	Signif	Direction	Applies To
red	yes	+	ratios, bars
yellow	no	n/a	bars
green	yes	−	ratios, bars
pink	yes	+	p- , q-Values
light green	yes	−	p- , q-Values

Dot Plots Dot Plots

Protein	SgPg vs SgFn Log2 Ratio	Log2 Sum	*q*-Value	*p*-Value	Raw SgPg	Raw SgFn	Normalized SgPg	Normalized SgFn	Description
SGO_0583	-0.967	6.490	0.0045	0.0163	14.000	23.000	14.6265	35.1226	nadE; NAD+ synthetase
					15.500	15.000	15.5000	24.6647	
SGO_0585	-1.956	8.313	0.0004	0.0004	36.000	77.500	37.6111	118.3478	pepC; aminopeptidase C
					28.000	81.500	28.0000	134.0117	
SGO_0586	0.341	7.309	0.0144	0.0754	47.000	20.500	49.1034	31.3049	pbp1a; penicillin-binding protein 1A
					39.500	23.500	39.5000	38.6414	
SGO_0589	-0.571	7.688	0.0196	0.1119	31.500	49.000	32.9097	74.8264	methylase
					50.000	29.500	50.0000	48.5073	
SGO_0590	-0.895	5.577	0.0044	0.0159	10.000	11.000	10.4475	16.7978	Methyltransferase
					6.500	8.500	6.5000	13.9767	
SGO_0591	-0.081	6.773	0.0005	0.0005	25.500	18.500	26.6412	28.2508	hypothetical protein SGO_0591
					26.500	17.000	26.5000	27.9534	
SGO_0592	0.401	5.626	0.0027	0.0078	13.000	7.500	13.5818	11.4530	luxS; autoinducer-2 production protein LuxS
					14.500	6.000	14.5000	9.8659	
SGO_0593	1.189	6.701	0.0013	0.0027	37.500	9.000	39.1782	13.7436	HD/KH domain protein
					33.000	11.000	33.0000	18.0875	
SGO_0594	-0.633	6.078	0.0028	0.0086	11.500	14.500	12.0147	22.1425	gmk; Guanylate kinase (GMP kinase)
					14.500	11.500	14.5000	18.9096	
SGO_0595	0.997	6.131	0.0011	0.0021	22.000	9.000	22.9846	13.7436	DNA-directed RNA polymerase, omega subunit
					23.500	6.000	23.5000	9.8659	
SGO_0597	-0.682	6.173	0.0032	0.0104	14.000	13.000	14.6265	19.8519	fmt; methionyl-tRNA formyltransferase
					13.000	15.000	13.0000	24.6647	
SGO_0598	0.150	4.423	0.0894	0.6563	8.500		8.8804		sun; sun protein
					6.000	4.000	6.0000	6.5773	

Log2 Ratios: -6 -4 -2 0 2 4 6

Show detected proteins only
Show all proteins
Filter by category:
ABC Transporter

Proteins found: 713

Test	Cutoff
q-Value	.005
p-Value	

	Signif	Direction	Applies To
red	yes	+	ratios, bars
yellow	no	n/a	bars
green	yes	−	ratios, bars
pink	yes	+	*p*- , *q*-Values
light green	yes	−	*p*- , *q*-Values

Dot Plots Dot Plots

Protein	SgPg vs SgFn Log2 Ratio	Log2 Sum	q-Value	p-Value	Raw SgPg	Raw SgFn	Normalized SgPg	Normalized SgFn	Description
SGO_0599	-1.709	7.255	0.0004	0.0003	18.500	40.500	19.3279	61.8463	phosphoprotein phosphatase
					16.500	33.500	16.5000	55.0846	
SGO_0600	-0.306	6.059	0.0240	0.1406	15.500	14.000	16.1937	21.3790	serine/threonine protein kinase
					13.500	9.500	13.5000	15.6210	
SGO_0603	-1.375	4.866	0.0031	0.0096	2.500	7.000	2.6119	10.6895	response regulator
					6.000	6.000	6.0000	9.8659	
SGO_0604	-1.206	8.588	0.0001	0.0000	54.000	87.000	56.4166	132.8550	hydrolase, haloacid dehalogenase family/peptidyl-prolyl cis-trans isomerase, cyclophilin type
					60.000	82.500	60.0000	135.6561	
SGO_0606	0.287	8.481	0.0032	0.0103	89.000	52.000	92.9830	79.4076	cysK; cysteine synthase A
					103.500	49.500	103.5000	81.3936	
SGO_0610	-0.434	8.621	0.0095	0.0444	84.500	84.000	88.2816	128.2737	ribosomal subunit interface protein
					78.500	60.000	78.5000	98.6590	
SGO_0626	0.400	4.071	0.0495	0.3347	5.000	3.000	5.2238	4.5812	recX; Regulatory protein recX
					7.000		7.0000		
SGO_0631	2.906	5.932	0.0044	0.0161	28.000	2.500	29.2531	3.8177	alpha-glycerophosphate oxidase
					28.000		28.0000		
SGO_0639	0.330	7.771	0.0060	0.0237	55.500	29.000	57.9838	44.2850	valS; valyl-tRNA synthetase
					63.500	32.000	63.5000	52.6181	
SGO_0641	0.081	6.473	0.0774	0.5594	22.500	16.000	23.5069	24.4331	ATPase, histidine kinase-, DNA gyrase B-, and HSP90-like domain protein protein
					22.000	11.500	22.0000	18.9096	
SGO_0642	0.432	5.530	0.0425	0.2821	15.000	4.500	15.6713	6.8718	hypothetical protein SGO_0642
					10.500	8.000	10.5000	13.1545	
SGO_0643	-1.201	4.722	0.0021	0.0051	3.000	5.500	3.1343	8.3989	cytosine-specific methyltransferase
					5.000	6.000	5.0000	9.8659	

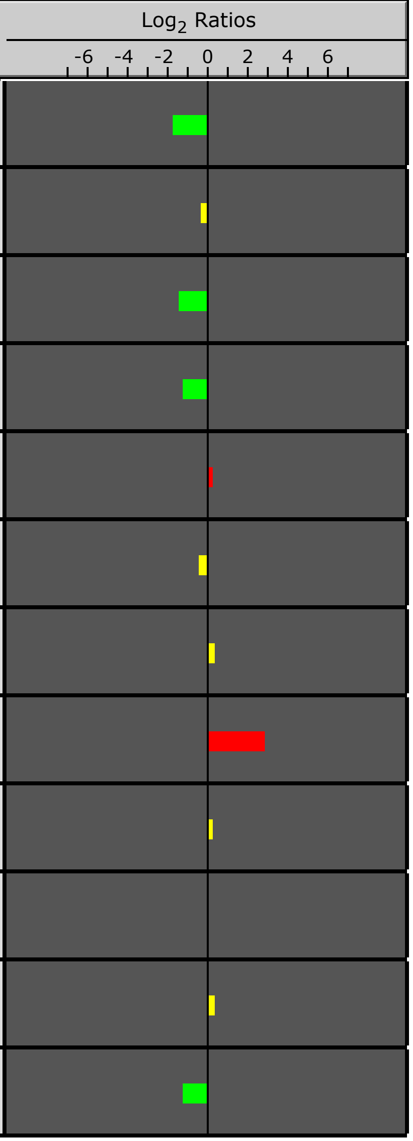

Show detected proteins only
Show all proteins
Filter by category:
ABC Transporter

Proteins found: 713

Test	Cutoff
q-Value	.005
p-Value	

Color	Signif	Direction	Applies To
red	yes	+	ratios, bars
yellow	no	n/a	bars
green	yes	−	ratios, bars
pink	yes	+	p- , q-Values
light green	yes	−	p- , q-Values

Dot Plots Dot Plots

Protein	SgPg vs SgFn Log_2 Ratio	Log_2 Sum	*q*-Value	*p*-Value	Raw *SgPg*	Raw *SgFn*	Normalized *SgPg*	Normalized *SgFn*	Description
SGO_0644	1.467	6.326	0.0028	0.0083	33.000	8.000	34.4768	12.2165	hypothetical protein SGO_0644
					24.500	5.500	24.5000	9.0437	
SGO_0652	0.057	3.991	0.0810	0.5877	6.500	2.500	6.7909	3.8177	hypothetical protein SGO_0652
					2.000	2.000	2.0000	3.2886	
SGO_0654	2.498	6.237	0.0141	0.0739	30.000	4.000	31.3426	6.1083	radical SAM enzyme, Cfr family
					38.000		38.0000		
SGO_0656	-1.633	5.978	0.0164	0.0904	7.500	8.000	7.8356	12.2165	trpB-2; tryptophan synthase, beta subunit
					6.000	22.500	6.0000	36.9971	
SGO_0665	0.384	9.366	0.0119	0.0585	165.000	107.500	172.3842	164.1599	non-heme iron-containing ferritin
					200.000	75.000	200.0000	123.3237	
SGO_0669	-0.097	7.966	0.0429	0.2855	57.500	38.500	60.0733	58.7921	typA; GTP-binding protein TypA
					60.500	43.000	60.5000	70.7056	
SGO_0671	-1.199	7.584	0.0008	0.0012	31.000	46.500	32.3873	71.0087	murD; UDP-N-acetylmuramoylalanine--D-glutamate ligase
					26.000	38.000	26.0000	62.4840	
SGO_0672	-1.375	6.114	0.0055	0.0216	5.500	14.000	5.7461	21.3790	murG; undecaprenyl-PP-MurNAc-pentapeptide-UDPGlcNAc GlcNAc transferase
					15.000	16.500	15.0000	27.1312	
SGO_0673	-1.282	4.462	0.0387	0.2526	3.500	4.500	3.6566	6.8718	DivIB; cell division protein DivIB
						7.000		11.5102	
SGO_0674	-0.210	8.869	0.0037	0.0120	107.500	84.500	112.3109	129.0373	ftsA; cell division protein FtsA
					104.500	74.000	104.5000	121.6794	
SGO_0675	-0.149	9.573	0.0515	0.3515	171.500	155.500	179.1750	237.4591	ftsZ; cell division protein FtsZ
					179.000	101.000	179.0000	166.0759	
SGO_0676	0.397	5.507	0.0465	0.3128	10.000	4.500	10.4475	6.8718	conserved hypothetical protein TIGR00044
					15.000	8.000	15.0000	13.1545	

Log_2 Ratios: -6 -4 -2 0 2 4 6

Show detected proteins only
Show all proteins
Filter by category:
ABC Transporter

Proteins found: 713

Test	Cutoff
***q*-Value**	**.005**
p-Value	

	Signif	Direction	Applies To
red	yes	+	ratios, bars
yellow	no	n/a	bars
green	yes	−	ratios, bars
pink	yes	+	*p*- , *q*-Values
light green	yes	−	*p*- , *q*-Values

Dot Plots | Dot Plots

Protein	*SgPg vs SgFn* Log$_2$ Ratio	Log$_2$ Sum	*q*-Value	*p*-Value	Raw *SgPg*	Raw *SgFn*	Normalized *SgPg*	Normalized *SgFn*	Description
SGO_0677	0.062	7.443	0.0778	0.5626	37.500	26.500	39.1782	40.4673	ylmF protein
					50.000	27.000	50.0000	44.3965	
SGO_0680	0.801	8.548	0.0049	0.0186	103.000	55.000	107.6095	83.9888	cell division protein DivIVA
					128.500	33.000	128.5000	54.2624	
SGO_0681	1.077	8.323	0.0001	0.0000	106.000	33.500	110.7438	51.1568	ileS; isoleucyl-tRNA synthetase
					106.500	31.500	106.5000	51.7960	
SGO_0684	-1.632	7.728	0.0004	0.0004	25.000	49.000	26.1188	74.8264	hypothetical protein SGO_0684
					25.500	52.000	25.5000	85.5044	
SGO_0688	-0.332	7.804	0.0336	0.2103	48.000	31.000	50.1481	47.3391	ATP dependent Clp protease, ATP-binding subunit, ClpE
					47.000	48.000	47.0000	78.9272	
SGO_0690	-1.471	4.553	0.0349	0.2212		5.000		7.6353	folD; methenyltetrahydrofolate cyclohydrolase
					3.500	7.500	3.5000	12.3324	
SGO_0693	-3.021	5.697	0.0055	0.0215	4.500	10.000	4.7014	15.2707	xseA; exodeoxyribonuclease VII, large subunit
					1.500	18.500	1.5000	30.4198	
SGO_0695	0.389	3.626				3.500		5.3447	geranyltranstransferase
					7.000		7.0000		
SGO_0698	-0.769	5.857	0.0019	0.0043	9.500	11.000	9.9251	16.7978	recN; DNA repair protein RecN
					11.500	12.000	11.5000	19.7318	
SGO_0699	-0.938	4.974	0.0023	0.0060	5.500	6.000	5.7461	9.1624	Serine/threonine protein phosphatase
					5.000	7.000	5.0000	11.5102	
SGO_0700	0.903	5.663	0.0060	0.0237	14.000	7.500	14.6265	11.4530	DegV family protein
					18.000	4.000	18.0000	6.5773	
SGO_0701	0.251	12.458	0.0282	0.1725	1475.000	705.500	1541.0098	1077.3468	hup; DNA-binding histone-like protein HU
					1496.500	919.000	1496.5000	1511.1263	

Log$_2$ Ratios: -6 -4 -2 0 2 4 6

Show detected proteins only
Show all proteins
Filter by category: ABC Transporter

Proteins found: 713

Test	Cutoff
q-Value	.005
p-Value	

Color	Signif	Direction	Applies To
red	yes	+	ratios, bars
yellow	no	n/a	bars
green	yes	−	ratios, bars
pink	yes	+	*p*- , *q*-Values
light green	yes	−	*p*- , *q*-Values

Dot Plots Dot Plots

Hendrickson *et al.*

Protein	SgPg vs SgFn Log$_2$ Ratio	Log$_2$ Sum	*q*-Value	*p*-Value	Raw *SgPg*	Raw *SgFn*	Normalized *SgPg*	Normalized *SgFn*	Description
SGO_0704	0.300	11.092	0.0038	0.0127	559.500	341.500	584.5390	521.4939	gpmA; 2,3-bisphosphoglycerate-dependent phosphoglycerate mutase
					619.500	278.500	619.5000	457.9420	
SGO_0706	-1.515	5.980	0.0001	0.0000	8.000	15.000	8.3580	22.9060	phoH-like protein
					8.000	14.500	8.0000	23.8426	
SGO_0707	-5.234	8.398	0.0005	0.0005	6.000	118.500	6.2685	180.9576	LPXTG cell wall surface protein
					3.000	89.500	3.0000	147.1663	
SGO_0708	-2.339	11.001	0.0000	0.0000	162.000	564.500	169.2499	862.0301	ald; alanine dehydrogenase
					169.000	516.500	169.0000	849.2892	
SGO_0713	-0.744	7.343	0.0012	0.0024	32.500	32.500	33.9545	49.6297	sgg; GTP-binding protein Era
					27.000	31.500	27.0000	51.7960	
SGO_0719	-1.516	4.686	0.0013	0.0029	3.500	5.500	3.6566	8.3989	rnr; ribonuclease R
					3.000	6.500	3.0000	10.6881	
SGO_0721	-0.439	5.111	0.0072	0.0300	6.000	6.500	6.2685	9.9259	abpB-like dipeptidase lipoprotein
					8.500	6.000	8.5000	9.8659	
SGO_0722	0.853	5.475	0.0002	0.0001	14.000	5.000	14.6265	7.6353	tehB; tellurite resistance protein TehB
					14.000	5.000	14.0000	8.2216	
SGO_0724	-0.780	4.723	0.0077	0.0334	4.000	4.500	4.1790	6.8718	dipeptidase
					5.500	6.000	5.5000	9.8659	
SGO_0736	-1.954	5.676	0.0005	0.0005	4.000	12.500	4.1790	19.0884	hprK; HPr(Ser) kinase/phosphatase
					6.500	13.000	6.5000	21.3761	
SGO_0739	1.551	5.087	0.0174	0.0968	12.500		13.0594		hypothetical protein SGO_0739
					16.000	3.000	16.0000	4.9329	
SGO_0742	-0.443	6.102	0.0000	0.0000	14.000	13.000	14.6265	19.8519	peptidase, U32 family
					14.500	12.000	14.5000	19.7318	

Log$_2$ Ratios: -6 -4 -2 0 2 4 6

Show detected proteins only
Show all proteins
Filter by category:
ABC Transporter

Proteins found: 713

Test	Cutoff
q-Value	.005
p-Value	

	Signif	Direction	Applies To
red	yes	+	ratios, bars
yellow	no	n/a	bars
green	yes	−	ratios, bars
pink	yes	+	*p*- , *q*-Values
light green	yes	−	*p*- , *q*-Values

Dot Plots Dot Plots

Protein	SgPg vs SgFn: Log_2 Ratio	Log_2 Sum	*q*-Value	*p*-Value	Raw: SgPg	Raw: SgFn	Normalized: SgPg	Normalized: SgFn	Description
SGO_0743	1.012	6.258	0.0031	0.0096	28.000	9.000	29.2531	13.7436	peptidase, U32 family
					22.000	7.000	22.0000	11.5102	
SGO_0745	1.433	6.982	0.0106	0.0504	48.500		50.6705		hypothetical protein SGO_0745
					56.000	12.000	56.0000	19.7318	
SGO_0749	-1.065	7.467	0.0068	0.0278	29.000	30.500	30.2978	46.5756	glutathione reductase
					26.000	45.000	26.0000	73.9942	
SGO_0750	3.963	8.415	0.0001	0.0000	160.000	6.000	167.1604	9.1624	efflux transporter, RND family, MFP subunit subfamily
					153.500	7.000	153.5000	11.5102	
SGO_0751	3.150	7.118	0.0009	0.0015	67.500	3.500	70.5208	5.3447	ABC transporter, ATP-binding protein SP0786
					54.000	5.500	54.0000	9.0437	
SGO_0753	0.776	8.336	0.0061	0.0244	101.500	50.000	106.0424	76.3534	lysS; lysyl-tRNA synthetase
					95.500	27.500	95.5000	45.2187	
SGO_0754	-1.203	3.045							phosphoglycerate mutase family protein
					2.500	3.500	2.5000	5.7551	
SGO_0755	0.430	5.150	0.0253	0.1495	8.000	4.000	8.3580	6.1083	regulatory protein
					12.000	5.500	12.0000	9.0437	
SGO_0760	0.092	8.988	0.0338	0.2120	125.500	75.000	131.1164	114.5301	ppc; phosphoenolpyruvate carboxylase
					130.500	80.000	130.5000	131.5453	
SGO_0761	-0.524	13.422	0.0022	0.0058	2246.500	1976.000	2347.0363	3017.4872	tuf; translation elongation factor Tu
					2149.500	2103.500	2149.5000	3458.8186	
SGO_0762	0.996	10.630	0.0038	0.0129	581.000	150.500	607.0012	229.8238	tpiA; triosephosphate isomerase
					449.500	181.500	449.5000	298.4433	
SGO_0763	-0.153	7.128	0.0151	0.0803	29.500	24.500	30.8202	37.4132	murA-1; UDP-N-acetylglucosamine 1-carboxyvinyltransferase
					35.500	22.000	35.5000	36.1750	

Log_2 Ratios: -6 -4 -2 0 2 4 6

Show detected proteins only
Show all proteins
Filter by category:
ABC Transporter

Proteins found: 713

Test	Cutoff
q-Value	.005
p-Value	

Color	Signif	Direction	Applies To
red	yes	+	ratios, bars
yellow	no	n/a	bars
green	yes	−	ratios, bars
pink	yes	+	*p*- , *q*-Values
light green	yes	−	*p*- , *q*-Values

Dot Plots Dot Plots

Hendrickson *et al.*

Protein	SgPg vs SgFn Log2 Ratio	Log2 Sum	q-Value	p-Value	Raw SgPg	Raw SgFn	Normalized SgPg	Normalized SgFn	Description
SGO_0767	-0.829	5.279	0.0144	0.0755	5.500	10.500	5.7461	16.0342	transport protein
					8.000	5.500	8.0000	9.0437	
SGO_0771	-0.078	8.325	0.0501	0.3396	73.000	58.500	76.2669	89.3335	pepq; proline dipeptidase
					79.500	46.000	79.5000	75.6385	
SGO_0773	-0.087	8.100	0.0601	0.4177	68.500	42.500	71.5655	64.9004	ccpA; catabolite control protein A
					61.500	46.500	61.5000	76.4607	
SGO_0774	0.257	5.516	0.0357	0.2284	14.500	6.500	15.1489	9.9259	glycosyl transferase, group 1 family protein
					10.000	6.500	10.0000	10.6881	
SGO_0775	-0.186	5.622	0.0562	0.3873	9.500	7.000	9.9251	10.6895	glycosyl transferase, group 1
					13.000	9.500	13.0000	15.6210	
SGO_0778	0.500	8.428	0.0005	0.0005	94.000	45.500	98.2067	69.4816	thrS; threonyl-tRNA synthetase
					103.500	44.500	103.5000	73.1721	
SGO_0779	-1.473	7.317	0.0007	0.0008	23.500	40.500	24.5517	61.8463	response regulator
					18.000	33.500	18.0000	55.0846	
SGO_0780	-1.070	4.283	0.0082	0.0369	3.000	5.500	3.1343	8.3989	histidine kinase
					3.000	3.000	3.0000	4.9329	
SGO_0784	-0.115	6.752	0.0695	0.4919	24.000	14.500	25.0741	22.1425	smc; chromosome segregation protein SMC
					26.000	21.000	26.0000	34.5306	
SGO_0785	-0.548	3.363			4.000	4.000	4.1790	6.1083	Cof family protein
SGO_0786	1.850	5.077	0.0050	0.0193	14.500		15.1489		Cof family protein
					14.500	2.500	14.5000	4.1108	
SGO_0787	0.448	6.741	0.0087	0.0402	29.500	17.500	30.8202	26.7237	ftsY; cell division protein FtsY
					30.500	11.500	30.5000	18.9096	

Log2 Ratios: -6 -4 -2 0 2 4 6

Show detected proteins only
Show all proteins
Filter by category: ABC Transporter

Proteins found: 713

Test	Cutoff
q-Value	.005
p-Value	

	Signif	Direction	Applies To
red	yes	+	ratios, bars
yellow	no	n/a	bars
green	yes	−	ratios, bars
pink	yes	+	p-, q-Values
light green	yes	−	p-, q-Values

Dot Plots Dot Plots

Hendrickson *et al.*

Protein	*SgPg vs SgFn* Log_2 Ratio	Log_2 Sum	*q*-Value	*p*-Value	Raw *SgPg*	Raw *SgFn*	Normalized *SgPg*	Normalized *SgFn*	Description
SGO_0788	-1.017	8.020	0.0028	0.0083	40.000	49.000	41.7901	74.8264	zwf; glucose-6-phosphate 1-dehydrogenase
					43.500	60.500	43.5000	99.4811	
SGO_0792	1.245	5.922	0.0025	0.0071	23.000	7.000	24.0293	10.6895	hypothetical protein SGO_0792
					18.500	4.500	18.5000	7.3994	
SGO_0794	-0.353	7.442	0.0119	0.0588	35.000	28.000	36.5663	42.7579	metallo-beta-lactamase family protein
					39.500	33.500	39.5000	55.0846	
SGO_0795	-0.692	5.681	0.0081	0.0360	9.000	8.500	9.4028	12.9801	tributyrin esterase
					10.000	11.500	10.0000	18.9096	
SGO_0798	-0.196	6.593	0.0045	0.0162	22.500	16.500	23.5069	25.1966	ABC transporter, ATP-binding protein SP1381
					21.500	16.000	21.5000	26.3091	
SGO_0800	0.619	4.705	0.0019	0.0047	7.000	3.500	7.3133	5.3447	polysaccharide deacetylase family protein
					8.500	3.000	8.5000	4.9329	
SGO_0801	0.567	8.645	0.0005	0.0004	111.500	54.500	116.4899	83.2252	hom; homoserine dehydrogenase
					122.500	47.500	122.5000	78.1050	
SGO_0802	0.008	6.597	0.0826	0.6016	23.000	16.000	24.0293	24.4331	thrB; homoserine kinase
					24.500	14.500	24.5000	23.8426	
SGO_0804	-0.701	4.607	0.0102	0.0483	5.000	4.000	5.2238	6.1083	murB; UDP-N-acetylenolpyruvoylglucosamine reductase
					4.000	5.500	4.0000	9.0437	
SGO_0805	-0.751	4.677	0.0092	0.0427	3.500	4.500	3.6566	6.8718	potA; spermidine/putrescine ABC transporter, ATP-binding subunit
					6.000	5.500	6.0000	9.0437	
SGO_0808	-0.125	2.871				2.500		3.8177	potD; spermidine/putrescine ABC transporter
					3.500		3.5000		
SGO_0812	-1.840	5.482	0.0012	0.0023	4.500	10.000	4.7014	15.2707	hypothetical protein SGO_0812
					5.000	12.000	5.0000	19.7318	

◉ Show detected proteins only
○ Show all proteins
☐ Filter by category:
ABC Transporter

Proteins found:
713

Test	Cutoff
***q*-Value**	**.005**
p-Value	

	Signif	Direction	Applies To
(red)	yes	+	ratios, bars
(yellow)	no	n/a	bars
(green)	yes	−	ratios, bars
(pink)	yes	+	*p*- , *q*-Values
(light green)	yes	−	*p*- , *q*-Values

Dot Plots | Dot Plots

Protein	SgPg vs SgFn Log$_2$ Ratio	Log$_2$ Sum	*q*-Value	*p*-Value	Raw *SgPg*	Raw *SgFn*	Normalized *SgPg*	Normalized *SgFn*	Description
**SGO_0815**	0.991	6.530	0.0013	0.0026	31.500	9.000	32.9097	13.7436	thiI; thiamine biosynthesis protein ThiI
					28.500	10.500	28.5000	17.2653	
**SGO_0818**	3.001	8.832	0.0110	0.0524	190.500	17.500	199.0253	26.7237	rplU; ribosomal protein L21
					230.000		230.0000		
**SGO_0820**	0.685	6.704	0.0229	0.1335	35.000		36.5663		rpmA; ribosomal protein L27
					43.000	15.000	43.0000	24.6647	
**SGO_0823**	-0.907	3.580	0.0282	0.1720		3.500		5.3447	Cof family protein
					2.500	2.500	2.5000	4.1108	
**SGO_0824**	0.663	7.458	0.0000	0.0000	51.500	22.500	53.8047	34.3590	lepA; GTP-binding protein LepA
					54.000	20.500	54.0000	33.7085	
**SGO_0825**	0.536	4.160	0.0096	0.0448	6.500	3.000	6.7909	4.5812	lipoprotein, putative
					6.500		6.5000		
**SGO_0830**	-0.654	2.427			2.000		2.0895		uvrC; excinuclease ABC, C subunit
						2.000		3.2886	
**SGO_0832**	0.985	3.942	0.0356	0.2272	7.000	2.000	7.3133	3.0541	hypothetical protein SGO_0832
					5.000		5.0000		
**SGO_0833**	-2.479	5.265	0.0007	0.0009	2.000	11.500	2.0895	17.5613	phosphoesterase-related gene
					4.000	9.000	4.0000	14.7988	
**SGO_0835**	-1.103	8.702	0.0000	0.0000	63.500	93.000	66.3418	142.0174	nitroreductase
					66.000	86.500	66.0000	142.2333	
**SGO_0836**	-0.497	9.336	0.0013	0.0026	130.000	130.500	135.8178	199.2824	pepV; dipeptidase PepV
					132.000	109.000	132.0000	179.2304	
**SGO_0842**	-2.671	6.038	0.0025	0.0072	3.000	22.500	3.1343	34.3590	rhodanese family protein
					6.000	13.500	6.0000	22.1983	

Log$_2$ Ratios: -6 -4 -2 0 2 4 6

Show detected proteins only
Show all proteins
Filter by category:
ABC Transporter

Proteins found: 713

Test	Cutoff
***q*-Value**	**.005**
p-Value	

	Signif	Direction	Applies To
(red)	yes	+	ratios, bars
(yellow)	no	n/a	bars
(green)	yes	−	ratios, bars
(pink)	yes	+	*p*- , *q*-Values
(light green)	yes	−	*p*- , *q*-Values

Dot Plots Dot Plots

Protein	SgPg vs SgFn Log$_2$ Ratio	Log$_2$ Sum	*q*-Value	*p*-Value	Raw *SgPg*	Raw *SgFn*	Normalized *SgPg*	Normalized *SgFn*	Description
SGO_0848	1.996	7.739	0.0004	0.0003	82.000	18.000	85.6697	27.4872	rpmE; ribosomal protein L31
					84.000	10.000	84.0000	16.4432	
SGO_0849	-0.697	5.926	0.0132	0.0684	13.000	15.000	13.5818	22.9060	DHH subfamily 1 protein
					9.500	9.000	9.5000	14.7988	
SGO_0850	1.176	7.105	0.0011	0.0020	48.500	16.000	50.6705	24.4331	flavodoxin
					44.500	11.000	44.5000	18.0875	
SGO_0851	-0.875	4.486	0.0156	0.0837	5.000	7.000	5.2238	10.6895	putative permease
					6.500		6.5000		
SGO_0852	-1.209	4.527	0.0025	0.0071	4.500	5.000	4.7014	7.6353	TPR domain protein
					2.500	5.000	2.5000	8.2216	
SGO_0853	-0.340	4.208	0.0129	0.0661	4.500	3.500	4.7014	5.3447	budA; alpha-acetolactate decarboxylase
					3.500	3.000	3.5000	4.9329	
SGO_0854	-2.595	6.521	0.0044	0.0160	5.000	33.500	5.2238	51.1568	cshA; surface-associated protein CshA
					7.500	17.000	7.5000	27.9534	
SGO_0855	0.282	4.813	0.0271	0.1633	8.000	3.500	8.3580	5.3447	fbpA; fibronectin-binding protein A
					7.000	4.500	7.0000	7.3994	
SGO_0856	1.850	6.458	0.0011	0.0021	29.000	5.500	30.2978	8.3989	ABC transporter, substrate binding protein
					38.500	6.500	38.5000	10.6881	
SGO_0859	-0.621	7.234	0.0007	0.0010	30.000	29.000	31.3426	44.2850	pheS; phenylalanyl-tRNA synthetase, alpha subunit
					28.000	28.500	28.0000	46.8630	
SGO_0861	0.895	8.418	0.0007	0.0009	103.500	35.500	108.1319	54.2109	pheT; phenylalanyl-tRNA synthetase, beta subunit
					114.000	40.000	114.0000	65.7726	
SGO_0885	-1.339	5.333	0.0042	0.0146	5.500	11.500	5.7461	17.5613	cobyric acid synthase
					5.500	7.000	5.5000	11.5102	

Log$_2$ Ratios: -6 -4 -2 0 2 4 6

Show detected proteins only
Show all proteins
Filter by category:
ABC Transporter

Proteins found: 713

Test	Cutoff
***q*-Value**	**.005**
p-Value	

Colour	Signif	Direction	Applies To
red	yes	+	ratios, bars
yellow	no	n/a	bars
green	yes	−	ratios, bars
pink	yes	+	*p*- , *q*-Values
light green	yes	−	*p*- , *q*-Values

Dot Plots Dot Plots

Hendrickson *et al.*

Protein	SgPg vs SgFn Log_2 Ratio	Log_2 Sum	*q*-Value	*p*-Value	Raw *SgPg*	Raw *SgFn*	Normalized *SgPg*	Normalized *SgFn*	Description
SGO_0886	-1.583	4.790	0.0009	0.0015	2.500	7.000	2.6119	10.6895	Mur ligase family protein
					4.500	6.000	4.5000	9.8659	
SGO_0889	0.566	6.433	0.0003	0.0003	24.000	11.500	25.0741	17.5613	glmM; phosphoglucosamine mutase
					26.500	10.500	26.5000	17.2653	
SGO_0893	-0.107	7.603	0.0440	0.2940	40.500	33.000	42.3125	50.3933	GTP-binding protein
					51.500	30.500	51.5000	50.1516	
SGO_0901	-0.125	6.162	0.0425	0.2819	18.000	11.500	18.8055	17.5613	DNA-directed DNA polymerase III
					15.500	12.000	15.5000	19.7318	
SGO_0906	-1.270	7.911	0.0000	0.0000	34.000	56.500	35.5216	86.2794	leuA; 2-isopropylmalate synthase
					35.000	51.000	35.0000	83.8601	
SGO_0909	-0.372	4.027	0.0561	0.3851		4.500		6.8718	leuC; 3-isopropylmalate dehydratase, large subunit
					4.500	3.000	4.5000	4.9329	
SGO_0911	0.254	8.195	0.0012	0.0024	75.000	45.000	78.3564	68.7181	hypothetical protein SGO_0911
					81.000	39.500	81.0000	64.9505	
SGO_0946	-0.931	6.698	0.0008	0.0011	19.000	23.000	19.8503	35.1226	Deblocking aminopeptidase
					16.000	20.000	16.0000	32.8863	
SGO_0949	-0.001	4.522	0.1088	0.8182	5.000	5.000	5.2238	7.6353	deaD; DEAD RNA helicase
					6.000	2.500	6.0000	4.1108	
SGO_0950	-1.575	5.402	0.0029	0.0089	6.500	12.000	6.7909	18.3248	oxidoreductase
					4.000	8.000	4.0000	13.1545	
SGO_0951	-0.202	5.426	0.0329	0.2049	9.000	6.500	9.4028	9.9259	udk; uridine kinase
					10.500	8.000	10.5000	13.1545	
SGO_0954	-0.415	6.016	0.0367	0.2361	9.500	13.000	9.9251	19.8519	ATP-binding protein
					18.500	10.000	18.5000	16.4432	

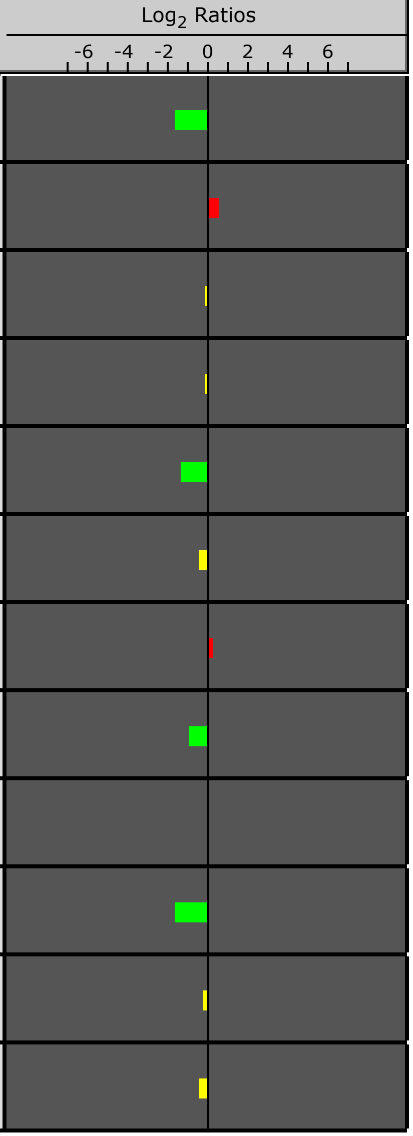

Show detected proteins only
Show all proteins
Filter by category:
ABC Transporter

Proteins found: 713

Test	Cutoff
***q*-Value**	**.005**
p-Value	

	Signif	Direction	Applies To
(red)	yes	+	ratios, bars
(yellow)	no	n/a	bars
(green)	yes	−	ratios, bars
(pink)	yes	+	*p*- , *q*-Values
(light green)	yes	−	

Dot Plots Dot Plots

Protein	SgPg vs SgFn Log_2 Ratio	Log_2 Sum	*q*-Value	*p*-Value	Raw *SgPg*	Raw *SgFn*	Normalized *SgPg*	Normalized *SgFn*	Description
SGO_0955	0.451	4.001	0.0127	0.0640		3.000		4.5812	transporter
					6.500	3.000	6.5000	4.9329	
SGO_0959	-1.525	5.534	0.0049	0.0184	6.500	14.000	6.7909	21.3790	copper homeostasis protein CutC
					5.000	8.000	5.0000	13.1545	
SGO_0981	0.346	2.486			3.000		3.1343		comE operon protein 2 family
						1.500		2.4665	
SGO_0982	0.524	6.278	0.0049	0.0186	19.500	10.000	20.3727	15.2707	amino acid ABC transporter, amino acid-binding protein
					25.500	10.000	25.5000	16.4432	
SGO_0987	-0.070	7.365	0.0982	0.7269	32.500	28.000	33.9545	42.7579	metK; S-adenosylmethionine synthetase
					47.000	25.000	47.0000	41.1079	
SGO_0993	-0.644	3.652	0.0469	0.3163		2.500		3.8177	GTP-binding protein HflX
					3.000	3.500	3.0000	5.7551	
SGO_0995	-1.248	5.629	0.0117	0.0566	11.500	9.000	12.0147	13.7436	metallo-beta-lactamase superfamily protein
					4.000	12.000	4.0000	19.7318	
SGO_0997	-0.881	4.852	0.0342	0.2148	8.500	5.000	8.8804	7.6353	hypothetical protein SGO_0997
					2.500	6.000	2.5000	9.8659	
SGO_1000	-1.010	5.423	0.0013	0.0025	8.000	9.000	8.3580	13.7436	recJ; single-stranded-DNA-specific exonuclease RecJ
					6.000	9.000	6.0000	14.7988	
SGO_1001	-0.178	7.924	0.0588	0.4071	48.500	33.000	50.6705	50.3933	apt; adenine phosphoribosyltransferase
					62.000	48.500	62.0000	79.7493	
SGO_1003	0.993	4.573	0.0021	0.0054	8.500	2.500	8.8804	3.8177	DNA replication protein DnaD
					7.000	2.500	7.0000	4.1108	
SGO_1004	0.613	4.345	0.0244	0.1438	7.500	2.000	7.8356	3.0541	glutathione S-transferase family protein
					4.500	3.000	4.5000	4.9329	

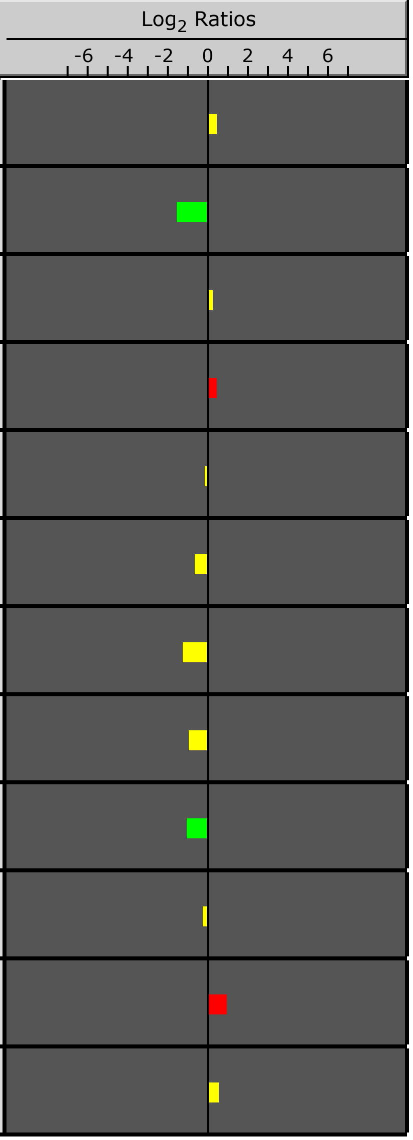

Show detected proteins only
Show all proteins
Filter by category:
ABC Transporter

Proteins found: 713

Test	Cutoff
q-Value	.005
p-Value	

	Signif	Direction	Applies To
red	yes	+	ratios, bars
yellow	no	n/a	bars
green	yes	−	ratios, bars
pink	yes	+	*p*- , *q*-Values
light green	yes	−	

Dot Plots Dot Plots

Hendrickson *et al.*

Protein	SgPg vs SgFn Log$_2$ Ratio	Log$_2$ Sum	*q*-Value	*p*-Value	Raw SgPg	Raw SgFn	Normalized SgPg	Normalized SgFn	Description
SGO_1005	0.464	4.993	0.0129	0.0658	10.000	5.000	10.4475	7.6353	Bcl-2 family protein
					8.000	3.500	8.0000	5.7551	
SGO_1006	-0.015	3.203	0.1268	0.9734	3.500	2.000	3.6566	3.0541	conserved hypothetical protein TIGR00486
					2.500		2.5000		
SGO_1009	-0.573	9.300	0.0019	0.0047	126.500	115.000	132.1612	175.6129	rfbA-1; glucose-1-phosphate thymidylyltransferase
					121.000	122.500	121.0000	201.4287	
SGO_1010	0.105	7.238	0.0678	0.4775	42.500	26.000	44.4020	39.7038	rmlC; dTDP-4-keto-6-deoxyglucose-3,5-epimerase
					34.000	20.000	34.0000	32.8863	
SGO_1011	-1.226	8.918	0.0039	0.0133	73.000	90.500	76.2669	138.1997	rfbB-1; dTDP-glucose 4,6-dehydratase
					67.000	123.000	67.0000	202.2509	
SGO_1012	-0.640	9.034	0.0002	0.0001	98.500	102.500	102.9081	156.5245	galE-1; UDP-glucose 4-epimerase
					102.000	99.000	102.0000	162.7873	
SGO_1013	-0.717	2.988							Glycosyltransferase involved in cell wall biogenesis
					3.000	3.000	3.0000	4.9329	
SGO_1016	0.679	4.010	0.0282	0.1722	6.500	2.500	6.7909	3.8177	putative glycosyltransferase
					5.500		5.5000		
SGO_1019	0.022	3.918	0.1180	0.8916	4.000		4.1790		glycosyl transferase
					6.000	3.000	6.0000	4.9329	
SGO_1020	-0.479	7.674	0.0085	0.0391	34.000	37.000	35.5216	56.5015	rfbD; dTDP-4-dehydrorhamnose reductase
					50.500	37.500	50.5000	61.6618	
SGO_1024	1.861	5.327	0.0044	0.0154	18.500	2.000	19.3279	3.0541	putative polysaccharide ABC transporter
					12.000	3.500	12.0000	5.7551	
SGO_1025	1.052	5.779	0.0087	0.0402	15.000	8.500	15.6713	12.9801	rgp; glycosyltransferase
					20.500	3.500	20.5000	5.7551	

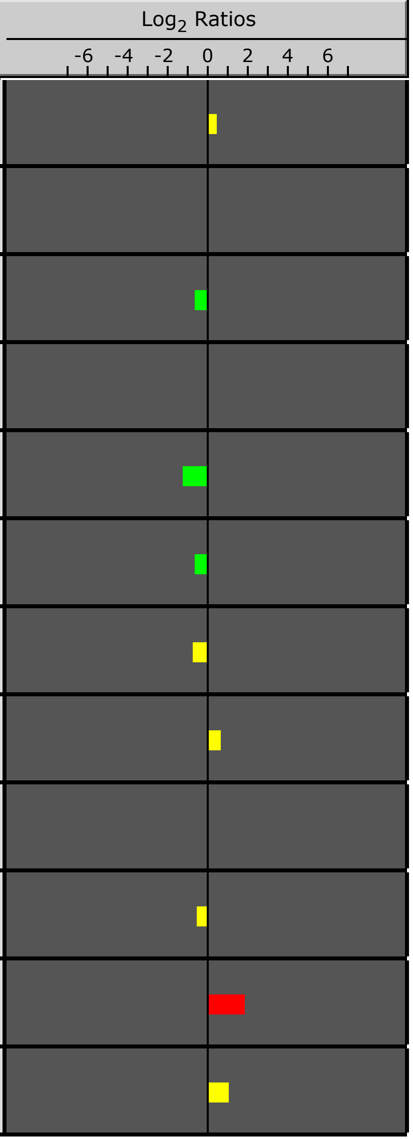

Show detected proteins only
Show all proteins
Filter by category:
ABC Transporter

Proteins found: 713

Test	Cutoff
q-Value	.005
p-Value	

Color	Signif	Direction	Applies To
red	yes	+	ratios, bars
yellow	no	n/a	bars
green	yes	−	ratios, bars
light red	yes	+	*p*-, *q*-Values
light green	yes	−	*p*-, *q*-Values

Dot Plots Dot Plots

Protein	SgPg vs SgFn Log_2 Ratio	Log_2 Sum	*q*-Value	*p*-Value	Raw *SgPg*	Raw *SgFn*	Normalized *SgPg*	Normalized *SgFn*	Description	Log_2 Ratios (-6 -4 -2 0 2 4 6)
SGO_1026	-0.231	6.138	0.0308	0.1900	15.000	10.500	15.6713	16.0342	rhamnosyltransferase	
					16.500	13.500	16.5000	22.1983		
SGO_1031	0.443	4.994	0.0067	0.0276	8.000	4.000	8.3580	6.1083	cmk; cytidylate kinase	
					10.000	4.500	10.0000	7.3994		
SGO_1034	1.012	9.453	0.0001	0.0000	222.000	79.000	231.9350	120.6384	rplT; ribosomal protein L20	
					236.500	68.000	236.5000	111.8135		
SGO_1035	-0.564	5.768	0.0013	0.0027	10.500	10.000	10.9699	15.2707	gloA; lactoylglutathione lyase	
					11.000	10.500	11.0000	17.2653		
SGO_1036	-0.942	6.292	0.0161	0.0871	8.500	21.000	8.8804	32.0684	amino acid ABC transporter, ATP-binding protein SP1242	
					18.500	11.500	18.5000	18.9096		
SGO_1037	1.204	4.743	0.0083	0.0375	9.000	1.500	9.4028	2.2906	glutamine ABC transporter permease and substrate binding protein	
					8.500	4.000	8.5000	6.5773		
SGO_1038	-0.205	5.088	0.0722	0.5173	9.500	7.000	9.9251	10.6895	uvrB; excinuclease ABC, B subunit	
					6.000	4.500	6.0000	7.3994		
SGO_1041	-0.646	4.507	0.0268	0.1605	7.000	6.500	7.3133	9.9259	hypothetical protein SGO_1041	
					5.500		5.5000			
SGO_1044	-0.186	3.767	0.0278	0.1681	4.000		4.1790		MutT/nudix family protein	
					4.500	3.000	4.5000	4.9329		
SGO_1047	2.902	5.310	0.0170	0.0942	15.500		16.1937		hypothetical protein SGO_1047	
					21.000	1.500	21.0000	2.4665		
SGO_1050	0.036	3.963	0.1129	0.8522	3.500		3.6566		ribF; riboflavin biosynthesis protein RibF	
					7.000	3.000	7.0000	4.9329		
SGO_1059	2.143	5.896	0.0001	0.0001	22.500	4.000	23.5069	6.1083	pstB; Phosphate import ATP-binding protein pstB 1 (Phosphate-transporting ATPase 1) (ABC phosphate transporter 1)	
					25.000	3.000	25.0000	4.9329		

Show detected proteins only
Show all proteins
Filter by category:
ABC Transporter

Proteins found: 713

Test	Cutoff
q-Value	.005
p-Value	

	Signif	Direction	Applies To
red	yes	+	ratios, bars
yellow	no	n/a	bars
green	yes	−	ratios, bars
pink	yes	+	*p*- , *q*-Values
light green	yes	−	*p*- , *q*-Values

Dot Plots Dot Plots

Protein	*SgPg vs SgFn* Log$_2$ Ratio	Log$_2$ Sum	*q*-Value	*p*-Value	Raw *SgPg*	Raw *SgFn*	Normalized *SgPg*	Normalized *SgFn*	Description
SGO_1060	1.407	6.618	0.0009	0.0014	36.500	7.500	38.1335	11.4530	phosphate transport system regulatory protein
					33.000	9.500	33.0000	15.6210	
SGO_1065	-0.175	5.520	0.0316	0.1956	10.500	9.000	10.9699	13.7436	hypothetical protein SGO_1065
					10.500	6.500	10.5000	10.6881	
SGO_1066	-1.266	5.599	0.0009	0.0016	8.000	10.500	8.3580	16.0342	hypothetical protein SGO_1066
					6.000	11.000	6.0000	18.0875	
SGO_1069	0.250	8.375	0.0127	0.0644	95.000	49.000	99.2515	74.8264	membrane alanyl aminopeptidase
					81.500	46.500	81.5000	76.4607	
SGO_1072	-2.645	5.819	0.0021	0.0051	4.000	17.000	4.1790	25.9602	ciaR; Transcriptional regulatory protein CiaR
						16.000		26.3091	
SGO_1073	-1.332	3.200			2.500		2.6119		Sensor protein CiaH
						4.000		6.5773	
SGO_1075	-2.053	4.817	0.0216	0.1250		9.500		14.5072	alpha-amylase precursor
					3.000	6.500	3.0000	10.6881	
SGO_1077	0.031	4.917	0.0724	0.5199	6.500	1.500	6.7909	2.2906	coaA; pantothenate kinase
					5.500	9.500	5.5000	15.6210	
SGO_1078	-1.252	5.397	0.0030	0.0090	4.500	8.500	4.7014	12.9801	methyltransferase domain protein
					8.000	10.000	8.0000	16.4432	
SGO_1079	-0.876	7.174	0.0185	0.1042	20.500	20.000	21.4174	30.5414	pdp; pyrimidine-nucleoside phosphorylase
					27.500	39.500	27.5000	64.9505	
SGO_1080	0.245	7.887	0.0045	0.0165	64.000	37.000	66.8642	56.5015	deoC; deoxyribose-phosphate aldolase
					61.500	31.500	61.5000	51.7960	
SGO_1082	0.898	11.735	0.0011	0.0019	1026.000	443.000	1071.9160	676.4913	lipoprotein
					1141.000	316.500	1141.0000	520.4260	

Log$_2$ Ratios: -6 -4 -2 0 2 4 6

Show detected proteins only
Show all proteins
Filter by category:
ABC Transporter

Proteins found: 713

Test	Cutoff
***q*-Value**	**.005**
p-Value	

	Signif	Direction	Applies To
(red)	yes	+	ratios, bars
(yellow)	no	n/a	bars
(green)	yes	−	ratios, bars
(pink)	yes	+	*p*- , *q*-Values
(light green)	yes	−	*p*- , *q*-Values

Dot Plots Dot Plots

Protein	*SgPg vs SgFn* Log_2 Ratio	Log_2 Sum	*q*-Value	*p*-Value	Raw *SgPg*	Raw *SgFn*	Normalized *SgPg*	Normalized *SgFn*	Description
**SGO_1083**	1.096	8.662	0.0021	0.0055	149.500	41.500	156.1905	63.3733	sugar ABC transporter, ATP-binding protein SP0846
					120.500	39.500	120.5000	64.9505	
**SGO_1090**	0.249	4.491	0.0722	0.5174	9.000		9.4028		pseudouridine synthase, RluA family
					6.500	4.000	6.5000	6.5773	
**SGO_1096**	-3.502	12.948	0.0001	0.0001	295.000	2269.000	308.2020	3464.9183	butA; acetoin dehydrogenase
					332.500	2308.500	332.5000	3795.9033	
**SGO_1098**	-1.135	6.369	0.0019	0.0043	9.500	18.500	9.9251	28.2508	proA; gamma-glutamyl phosphate reductase
					16.500	17.000	16.5000	27.9534	
**SGO_1107**	1.157	2.994							PyrR bifunctional protein
					5.500	1.500	5.5000	2.4665	
**SGO_1109**	1.503	7.095	0.0001	0.0000	47.000	11.500	49.1034	17.5613	pyrB; aspartate carbamoyltransferase
					52.000	11.000	52.0000	18.0875	
**SGO_1111**	0.633	4.603	0.0021	0.0054	6.500	3.000	6.7909	4.5812	fruR; phosphotransferase system repressor
					8.000	3.000	8.0000	4.9329	
**SGO_1112**	0.638	5.431	0.0121	0.0598	10.000	5.000	10.4475	7.6353	fruB; 1-phosphofructokinase
					16.000	5.500	16.0000	9.0437	
**SGO_1113**	0.709	7.396	0.0246	0.1456	58.000	30.000	60.5956	45.8121	fruA; PTS system, fructose specific IIABC components
					41.500	12.500	41.5000	20.5539	
**SGO_1114**	1.400	5.373	0.0022	0.0060	15.500	5.000	16.1937	7.6353	Protein of unknown function (DUF1149) superfamily
					13.500	2.500	13.5000	4.1108	
**SGO_1115**	-1.645	4.960	0.0095	0.0441	5.000		5.2238		DegV family protein
					7.000	11.500	7.0000	18.9096	
**SGO_1116**	0.440	7.068	0.0022	0.0059	39.000	19.500	40.7453	29.7778	dapB; dihydrodipicolinate reductase
					36.500	16.500	36.5000	27.1312	

Log_2 Ratios: -6 -4 -2 0 2 4 6

Show detected proteins only
Show all proteins
Filter by category:
ABC Transporter

Proteins found: 713

Test	Cutoff
***q*-Value**	**.005**
p-Value	

Color	Signif	Direction	Applies To
red	yes	+	ratios, bars
yellow	no	n/a	bars
green	yes	−	ratios, bars
light red	yes	+	*p*- , *q*-Values
light green	yes	−	*p*- , *q*-Values

Dot Plots Dot Plots

Hendrickson *et al.*

Streptococcus gordonii

Protein	SgPg vs SgFn Log_2 Ratio	Log_2 Sum	*q*-Value	*p*-Value	Raw *SgPg*	Raw *SgFn*	Normalized *SgPg*	Normalized *SgFn*	Description
SGO_1117	-0.898	4.274	0.0269	0.1619	3.000	6.500	3.1343	9.9259	pcnA; polynucleotide adenylyltransferase
					3.000	2.000	3.0000	3.2886	
SGO_1118	-1.459	4.639	0.0008	0.0011	3.500	5.500	3.6566	8.3989	ABC transporter, ATP-binding protein SP1553
					3.000	6.000	3.0000	9.8659	
SGO_1120	-0.509	8.618	0.0052	0.0203	78.000	67.000	81.4907	102.3136	guaA; GMP synthase
					80.000	78.500	80.0000	129.0788	
SGO_1123	0.290	7.812	0.0038	0.0129	57.000	35.000	59.5509	53.4474	ffh; signal recognition particle protein
					64.000	29.000	64.0000	47.6852	
SGO_1129	-0.544	5.782	0.0047	0.0173	12.500	10.500	13.0594	16.0342	lplA; lipoate protein ligase A
					9.500	10.000	9.5000	16.4432	
SGO_1130	2.380	6.880	0.0001	0.0000	48.500	5.500	50.6705	8.3989	dihydrolipoamide dehydrogenase
					48.000	6.500	48.0000	10.6881	
SGO_1131	2.419	5.484	0.0116	0.0560	21.000	2.500	21.9398	3.8177	sucB; dihydrolipoamide S-acetyltransferase
					19.000		19.0000		
SGO_1133	0.146	5.214	0.0706	0.5025	13.500		14.1042		acoA; acetoin dehydrogenase
					11.500	7.000	11.5000	11.5102	
SGO_1134	-2.886	5.443	0.0357	0.2287	2.500	9.000	2.6119	13.7436	hypothetical protein SGO_1134
						16.500		27.1312	
SGO_1139	0.862	6.582	0.0002	0.0001	29.000	11.500	30.2978	17.5613	GTP-binding protein
					31.500	10.000	31.5000	16.4432	
SGO_1140	0.354	8.166	0.0015	0.0034	77.500	39.000	80.9683	59.5557	clpX; ATP-dependent Clp protease, ATP-binding subunit ClpX
					80.000	40.500	80.0000	66.5948	
SGO_1141	-1.422	5.648	0.0080	0.0352	7.500	8.500	7.8356	12.9801	folA; dihydrofolate reductase
					5.500	14.500	5.5000	23.8426	

Show detected proteins only
Show all proteins
Filter by category:
ABC Transporter

Proteins found: 713

Test	Cutoff
q-Value	.005
p-Value	

	Signif	Direction	Applies To
red	yes	+	ratios, bars
yellow	no	n/a	bars
green	yes	–	ratios, bars
pink	yes	+	*p*- , *q*-Values
light green	yes	–	*p*- , *q*-Values

Dot Plots Dot Plots

Streptococcus gordonii

Protein	*SgPg vs SgFn* Log2 Ratio	Log2 Sum	*q*-Value	*p*-Value	Raw *SgPg*	Raw *SgFn*	Normalized *SgPg*	Normalized *SgFn*	Description
SGO_1143	0.877	5.319	0.0041	0.0140	12.000	3.500	12.5370	5.3447	thyA; thymidylate synthase
					13.000	5.500	13.0000	9.0437	
SGO_1144	-0.356	9.774	0.0022	0.0056	184.000	152.500	192.2344	232.8779	glcK; glucokinase
					191.500	157.500	191.5000	258.9798	
SGO_1150	0.721	5.305	0.0023	0.0062	12.500	5.500	13.0594	8.3989	hypothetical protein SGO_1150
					11.500	4.000	11.5000	6.5773	
SGO_1151	-0.562	9.736	0.0001	0.0000	165.500	165.000	172.9065	251.9663	glyA; serine hydroxymethyltransferase
					171.500	156.000	171.5000	256.5133	
SGO_1154	0.220	7.107	0.1010	0.7485	33.500	30.000	34.9992	45.8121	prfA; peptide chain release factor 1
					36.500	12.500	36.5000	20.5539	
SGO_1155	1.345	5.132	0.0082	0.0365	9.000	4.500	9.4028	6.8718	tdk; thymidine kinase
					15.500	2.000	15.5000	3.2886	
SGO_1159	-0.794	4.599	0.0100	0.0472	3.000	4.500	3.1343	6.8718	guaC; guanosine monophosphate reductase
					6.000	5.000	6.0000	8.2216	
SGO_1167	2.059	7.614	0.0000	0.0000	76.000	13.000	79.4012	19.8519	nox; NADH oxidase
					78.500	11.000	78.5000	18.0875	
SGO_1169	1.376	6.806	0.0027	0.0078	44.000	12.500	45.9691	19.0884	NADPH-dependent FMN reductase
					34.500	7.500	34.5000	12.3324	
SGO_1170	1.493	7.367	0.0002	0.0001	60.000	15.500	62.6851	23.6696	NADPH-dependent FMN reductase
					59.000	12.000	59.0000	19.7318	
SGO_1171	-0.390	4.216	0.0429	0.2851	3.500	5.000	3.6566	7.6353	thioredoxin family protein
					4.000	2.000	4.0000	3.2886	
SGO_1185	1.176	4.409	0.0260	0.1546	9.500	2.500	9.9251	3.8177	acetyltransferase, GNAT family
					7.500		7.5000		

Log2 Ratios: -6 -4 -2 0 2 4 6

Show detected proteins only
Show all proteins
Filter by category:
ABC Transporter

Proteins found: 713

Test	Cutoff
q-Value	.005
p-Value	

Signif	Direction	Applies To
yes	+	ratios, bars
no	n/a	bars
yes	−	ratios, bars
yes	+	*p*- , *q*-Values
yes	−	

Dot Plots Dot Plots

Protein	SgPg vs SgFn Log_2 Ratio	Log_2 Sum	*q*-Value	*p*-Value	Raw *SgPg*	Raw *SgFn*	Normalized *SgPg*	Normalized *SgFn*	Description
SGO_1189	-1.120	6.592	0.0013	0.0026	12.500	20.000	13.0594	30.5414	lipoprotein, putative
					17.500	21.500	17.5000	35.3528	
SGO_1191	1.155	10.733	0.0002	0.0001	580.500	178.000	606.4788	271.8182	rplL; ribosomal protein L7/L12
					568.000	155.500	568.0000	255.6911	
SGO_1192	0.944	9.110	0.0071	0.0297	141.000	50.500	147.3101	77.1170	BL5; 50S ribosomal protein L10
					216.500	68.000	216.5000	111.8135	
SGO_1193	-0.451	6.593	0.0082	0.0371	22.500	17.000	23.5069	25.9602	gid; Glucose inhibited division protein A
					17.500	18.000	17.5000	29.5977	
SGO_1197	0.702	6.511	0.0076	0.0330	24.000	9.000	25.0741	13.7436	topA; DNA topoisomerase I
					31.000	13.000	31.0000	21.3761	
SGO_1202	-0.246	5.510	0.0543	0.3719	8.000	6.500	8.3580	9.9259	GTP-binding protein
					12.500	9.000	12.5000	14.7988	
SGO_1203	-0.619	6.766	0.0034	0.0112	19.000	19.500	19.8503	29.7778	anaerobic ribonucleotide reductase
					23.000	22.000	23.0000	36.1750	
SGO_1205	-0.195	4.889	0.0680	0.4795	5.500	7.500	5.7461	11.4530	dapA; dihydrodipicolinate synthase
					7.500	3.000	7.5000	4.9329	
SGO_1206	-0.559	6.998	0.0013	0.0029	25.500	23.500	26.6412	35.8861	asd; aspartate-semialdehyde dehydrogenase
					25.000	24.500	25.0000	40.2857	
SGO_1210	-1.072	5.922	0.0057	0.0223	12.500	11.500	13.0594	17.5613	fhs-1; formate--tetrahydrofolate ligase
					7.000	14.000	7.0000	23.0204	
SGO_1213	-0.601	3.733	0.0674	0.4737	3.000	4.500	3.1343	6.8718	coaC; phosphopantothenoylcysteine decarboxylase
						2.000		3.2886	
SGO_1215	-0.176	10.082	0.0080	0.0351	243.000	178.000	253.8748	271.8182	manB; phosphomannomutase
					254.500	184.500	254.5000	303.3763	

Log_2 Ratios: -6 -4 -2 0 2 4 6

Show detected proteins only
Show all proteins
Filter by category: ABC Transporter

Proteins found: 713

Test	Cutoff
q-Value	.005
p-Value	

Color	Signif	Direction	Applies To
red	yes	+	ratios, bars
yellow	no	n/a	bars
green	yes	–	ratios, bars
pink	yes	+	*p*-, *q*-Values
light green	yes	–	*p*-, *q*-Values

Dot Plots Dot Plots

Protein	*SgPg vs SgFn* Log_2 Ratio	Log_2 Sum	*q*-Value	*p*-Value	Raw *SgPg*	Raw *SgFn*	Normalized *SgPg*	Normalized *SgFn*	Description
SGO_1216	0.419	7.152	0.0080	0.0349	43.000	21.500	44.9244	32.8320	bta; Possible bacteriocin transport accessory protein
					36.500	17.000	36.5000	27.9534	
SGO_1219	-0.901	7.792	0.0163	0.0896	36.000	31.500	37.6111	48.1027	pta; phosphate acetyltransferase
					36.500	60.500	36.5000	99.4811	
SGO_1222	0.698	2.693							GTP pyrophosphokinase-like protein
					4.000	1.500	4.0000	2.4665	
SGO_1224	1.455	8.092	0.0003	0.0002	100.500	23.500	104.9976	35.8861	Ribose-phosphate pyrophosphokinase 2
					95.000	22.500	95.0000	36.9971	
SGO_1225	0.103	5.035	0.1024	0.7602	7.000	4.000	7.3133	6.1083	pyridoxal-phosphate dependent aminotransferase
					9.500	6.000	9.5000	9.8659	
SGO_1226	-0.118	6.216	0.0701	0.4969	16.000	10.000	16.7160	15.2707	hypothetical protein SGO_1226
					18.500	14.500	18.5000	23.8426	
SGO_1228	-0.494	4.758	0.0045	0.0167	6.000	5.500	6.2685	8.3989	DNA-binding protein BH0551
					5.000	4.500	5.0000	7.3994	
SGO_1231	-0.689	7.258	0.0135	0.0698	21.000	35.500	21.9398	54.2109	gyrA; DNA gyrase, A subunit
					37.500	24.000	37.5000	39.4636	
SGO_1232	-0.020	10.687	0.0601	0.4179	398.500	277.000	416.3338	422.9980	L-lactate dehydrogenase
					402.000	247.500	402.0000	406.9682	
SGO_1234	0.829	10.222	0.0014	0.0032	368.500	163.000	384.9913	248.9122	rpsA; 30S ribosomal protein S1
					376.000	112.000	376.0000	184.1634	
SGO_1238	-1.175	7.336	0.0066	0.0269	23.500	46.000	24.5517	70.2451	ilvE; branched-chain amino acid aminotransferase
					24.000	26.000	24.0000	42.7522	
SGO_1239	1.318	5.182	0.0052	0.0202	11.500	2.000	12.0147	3.0541	parC; DNA topoisomerase IV, A subunit
					13.000	5.000	13.0000	8.2216	

Log_2 Ratios: -6 -4 -2 0 2 4 6

Show detected proteins only
Show all proteins
Filter by category: ABC Transporter

Proteins found: 713

Test	Cutoff
q-Value	.005
p-Value	

	Signif	Direction	Applies To
red	yes	+	ratios, bars
yellow	no	n/a	bars
green	yes	−	ratios, bars
pink	yes	+	*p*- , *q*-Values
light green	yes	−	*p*- , *q*-Values

Dot Plots Dot Plots

Protein	SgPg vs SgFn Log2 Ratio	Log2 Sum	q-Value	p-Value	Raw SgPg	Raw SgFn	Normalized SgPg	Normalized SgFn	Description
SGO_1248	-0.965	6.635	0.0161	0.0872	19.000	14.500	19.8503	22.1425	pyrC; dihydroorotase
					13.000	27.000	13.0000	44.3965	
SGO_1253	1.111	6.169	0.0032	0.0103	22.000	10.000	22.9846	15.2707	pyrE; orotate phosphoribosyltransferase
					25.500	5.000	25.5000	8.2216	
SGO_1257	-0.991	5.347	0.0106	0.0504	6.000	11.500	6.2685	17.5613	probable transcriptional regulator (LysR family)
					7.000	6.000	7.0000	9.8659	
SGO_1260	1.058	7.351	0.0002	0.0002	51.000	18.000	53.2824	27.4872	deoD; purine nucleoside phosphorylase
					57.000	15.500	57.0000	25.4869	
SGO_1263	1.503	7.938	0.0002	0.0001	84.000	22.000	87.7592	33.5955	purine nucleoside phosphorylase I, inosine and guanosine-specific
					93.500	18.500	93.5000	30.4198	
SGO_1264	-0.124	9.567	0.0535	0.3653	177.500	149.500	185.4436	228.2967	deoB; phosphopentomutase
					175.500	103.000	175.5000	169.3645	
SGO_1265	1.083	7.260	0.0003	0.0002	48.000	15.500	50.1481	23.6696	rpiA; ribose 5-phosphate isomerase
					54.000	15.500	54.0000	25.4869	
SGO_1266	-0.086	6.119	0.0707	0.5041	18.000	11.000	18.8055	16.7978	trmE; tRNA modification GTPase TrmE
					15.000	11.500	15.0000	18.9096	
SGO_1273	1.594	8.036	0.0006	0.0008	93.000	27.000	97.1620	41.2308	rpoD; RNA polymerase sigma factor
					98.500	15.500	98.5000	25.4869	
SGO_1276	1.877	6.842	0.0007	0.0010	39.000	8.500	40.7453	12.9801	rpsU; ribosomal protein S21
					49.500	7.000	49.5000	11.5102	
SGO_1281	1.879	4.379	0.0216	0.1249	7.500		7.8356		penicillinase repressor, putative
					10.500	1.500	10.5000	2.4665	
SGO_1283	1.115	7.864	0.0006	0.0008	77.500	27.500	80.9683	41.9944	oxidoreductase
					78.000	19.500	78.0000	32.0642	

Log2 Ratios: -6 -4 -2 0 2 4 6

Show detected proteins only
Show all proteins
Filter by category:
ABC Transporter

Proteins found: 713

Test	Cutoff
q-Value	.005
p-Value	

	Signif	Direction	Applies To
red	yes	+	ratios, bars
yellow	no	n/a	bars
green	yes	–	ratios, bars
pink	yes	+	p-, q-Values
light green	yes	–	p-, q-Values

Dot Plots Dot Plots

Protein	SgPg vs SgFn Log$_2$ Ratio	Log$_2$ Sum	*q*-Value	*p*-Value	Raw *SgPg*	Raw *SgFn*	Normalized *SgPg*	Normalized *SgFn*	Description
SGO_1284	0.508	5.424	0.0537	0.3677	17.000	3.500	17.7608	5.3447	thioredoxin-disulfide reductase
					7.500	7.500	7.5000	12.3324	
SGO_1293	0.303	7.365	0.0076	0.0328	46.500	22.500	48.5810	34.3590	asnS; asparaginyl-tRNA synthetase
					42.500	24.000	42.5000	39.4636	
SGO_1297	-1.260	6.958	0.0033	0.0109	16.500	34.000	17.2384	51.9203	aspC; aspartate aminotransferase
					19.000	22.000	19.0000	36.1750	
SGO_1312	0.028	7.925	0.0440	0.2936	60.000	40.000	62.6851	61.0827	pepT; peptidase T
					60.000	36.000	60.0000	59.1954	
SGO_1315	-1.287	3.485	0.0152	0.0809	2.000	4.000	2.0895	6.1083	trmD; tRNA (guanine-N1)-methyltransferase
					3.000		3.0000		
SGO_1316	0.360	4.180	0.0050	0.0190	4.500	2.500	4.7014	3.8177	16S rRNA processing protein RimM
					5.500	2.500	5.5000	4.1108	
SGO_1323	0.692	7.175	0.0021	0.0051	39.500	19.500	41.2677	29.7778	rpsP; ribosomal protein S16
					48.000	15.500	48.0000	25.4869	
SGO_1327	0.395	5.518	0.0009	0.0015	12.000	6.500	12.5370	9.9259	HAD-superfamily subfamily IIA hydrolase, TIGR01457
					13.500	6.000	13.5000	9.8659	
SGO_1336	-0.326	5.865	0.0282	0.1730	13.000	8.500	13.5818	12.9801	pcrA; ATP-dependent DNA helicase PcrA
					12.000	12.000	12.0000	19.7318	
SGO_1338	-2.307	4.446	0.0012	0.0024		6.500		9.9259	Signal peptidase I
					2.000	6.000	2.0000	9.8659	
SGO_1339	0.431	11.551	0.0019	0.0043	803.500	447.500	839.4586	683.3631	pyk; pyruvate kinase
					882.500	362.500	882.5000	596.0645	
SGO_1340	-0.483	10.273	0.0008	0.0011	246.000	227.000	257.0091	346.6445	Phosphofructokinase
					259.000	228.000	259.0000	374.9040	

Log$_2$ Ratios: -6 -4 -2 0 2 4 6

Show detected proteins only
Show all proteins
Filter by category: ABC Transporter

Proteins found: 713

Test	Cutoff
q-Value	.005
p-Value	

Color	Signif	Direction	Applies To
red	yes	+	ratios, bars
yellow	no	n/a	bars
green	yes	–	ratios, bars
pink	yes	+	*p*- , *q*-Values
light green	yes	–	*p*- , *q*-Values

Dot Plots Dot Plots

Hendrickson *et al.*

Protein	SgPg vs SgFn Log_2 Ratio	Log_2 Sum	*q*-Value	*p*-Value	Raw *SgPg*	Raw *SgFn*	Normalized *SgPg*	Normalized *SgFn*	Description
SGO_1341	-1.469	4.431	0.0448	0.3009	3.000	4.000	3.1343	6.1083	dnaE; DNA-directed DNA polymerase III alpha chain
						7.500		12.3324	
SGO_1342	-0.755	9.403	0.0030	0.0092	128.000	155.000	133.7283	236.6956	ABC transporter, ATP-binding protein SP1715
					117.500	115.000	117.5000	189.0963	
SGO_1364	0.652	5.934	0.0013	0.0028	17.500	7.000	18.2832	10.6895	rumA-2; 23S rRNA (uracil-5-)-methyltransferase RumA
					19.000	8.000	19.0000	13.1545	
SGO_1365	-2.745	5.496	0.0007	0.0010	4.000	11.500	4.1790	17.5613	transcription regulator yrfE
					2.000	13.000	2.0000	21.3761	
SGO_1367	-1.851	4.827	0.0009	0.0014	3.000	6.500	3.1343	9.9259	aroK; shikimate kinase
					3.000	7.500	3.0000	12.3324	
SGO_1368	-1.086	5.261	0.0005	0.0005	6.000	9.000	6.2685	13.7436	aroA; 3-phosphoshikimate 1-carboxyvinyltransferase
					6.000	7.500	6.0000	12.3324	
SGO_1369	-3.364	7.878	0.0001	0.0000	9.000	72.500	9.4028	110.7125	L-2-hydroxyisocaproate dehydrogenase
					11.500	63.000	11.5000	103.5919	
SGO_1370	-0.075	6.763	0.0826	0.6023	27.500	21.000	28.7307	32.0684	Protein of unknown function (DUF964) superfamily
					24.000	14.500	24.0000	23.8426	
SGO_1372	1.399	4.953	0.0083	0.0379	12.000		12.5370		aroC; chorismate synthase
					13.500	3.000	13.5000	4.9329	
SGO_1373	-0.102	4.405	0.0224	0.1302	6.500		6.7909		aroB; 3-dehydroquinate synthase
					7.000	4.500	7.0000	7.3994	
SGO_1375	0.638	5.497	0.0111	0.0536	16.000	5.500	16.7160	8.3989	aroD; 3-dehydroquinate dehydratase, type I
					11.000	5.500	11.0000	9.0437	
SGO_1377	-0.693	6.527	0.0002	0.0001	16.500	19.000	17.2384	29.0143	sulfatase
					18.000	17.000	18.0000	27.9534	

Log_2 Ratios: -6 -4 -2 0 2 4 6

Show detected proteins only
Show all proteins
Filter by category: ABC Transporter

Proteins found: 713

Test	Cutoff
q-Value	.005
p-Value	

Colour	Signif	Direction	Applies To
red	yes	+	ratios, bars
yellow	no	n/a	bars
green	yes	–	ratios, bars
pink	yes	+	*p*- , *q*-Values
light green	yes	–	*p*- , *q*-Values

Dot Plots | Dot Plots

Hendrickson *et al.*

Protein	*SgPg vs SgFn* Log$_2$ Ratio	Log$_2$ Sum	*q*-Value	*p*-Value	Raw *SgPg*	Raw *SgFn*	Normalized *SgPg*	Normalized *SgFn*	Description	Log$_2$ Ratios -6 -4 -2 0 2 4 6
**SGO_1381**	-0.525	6.005	0.0188	0.1061	10.000	14.500	10.4475	22.1425	csn1; CRISPR-associated protein, Csn1 family	
					16.000	9.500	16.0000	15.6210		
**SGO_1383**	0.595	8.571	0.0030	0.0093	102.000	44.500	106.5647	67.9545	rplS; ribosomal protein L19	
					122.000	51.000	122.0000	83.8601		
**SGO_1386**	-2.592	6.069	0.0018	0.0042	6.000	15.500	6.2685	23.6696	chorismate mutase	
					3.500	20.500	3.5000	33.7085		
**SGO_1390**	-0.240	5.074	0.0349	0.2218	8.000	5.000	8.3580	7.6353	ligA; DNA ligase, NAD-dependent	
					7.000	6.500	7.0000	10.6881		
**SGO_1394**	-0.126	4.153	0.1186	0.8978	4.000	4.000	4.1790	6.1083	hypothetical protein SGO_1394	
					7.500		7.5000			
**SGO_1397**	-0.101	6.641	0.0692	0.4893	25.500	15.000	26.6412	22.9060	map; methionine aminopeptidase, type I	
					21.500	17.500	21.5000	28.7755		
**SGO_1398**	-0.036	4.232	0.1077	0.8090	5.000	2.500	5.2238	3.8177	hypothetical protein SGO_1398	
					4.000	3.500	4.0000	5.7551		
**SGO_1400**	-0.501	5.556	0.0058	0.0230	10.000	10.000	10.4475	15.2707	murA-2; UDP-N-acetylglucosamine 1-carboxyvinyltransferase	
					9.000	7.500	9.0000	12.3324		
**SGO_1413**	-0.910	4.834	0.0232	0.1353	4.500	3.500	4.7014	5.3447	first chain of major exonuclease RexA	
					4.500	8.500	4.5000	13.9767		
**SGO_1414**	-2.079	5.022	0.0038	0.0129	2.500	6.500	2.6119	9.9259	rexB; putative exonuclease RexB	
					3.500	10.000	3.5000	16.4432		
**SGO_1422**	-1.700	7.234	0.0037	0.0120	17.000	29.500	17.7608	45.0485	hypothetical protein SGO_1422	
					17.000	43.000	17.0000	70.7056		
**SGO_1426**	0.874	14.250	0.0003	0.0003	5900.500	2120.500	6164.5617	3238.1486	eno; enolase	
					6433.500	2216.500	6433.5000	3644.6263		

Show detected proteins only
Show all proteins
Filter by category:
ABC Transporter

Proteins found: 713

Test	Cutoff
***q*-Value**	**.005**
p-Value	

Color	Signif	Direction	Applies To
red	yes	+	ratios, bars
yellow	no	n/a	bars
green	yes	−	ratios, bars
pink	yes	+	*p*- , *q*-Values
light green	yes	−	*p*- , *q*-Values

Dot Plots Dot Plots

Protein	SgPg vs SgFn Log_2 Ratio	Log_2 Sum	*q*-Value	*p*-Value	Raw *SgPg*	Raw *SgFn*	Normalized *SgPg*	Normalized *SgFn*	Description
SGO_1431	-0.244	9.039	0.0119	0.0586	117.500	102.000	122.7584	155.7610	EzrA; Septation ring formation regulator ezrA
					117.500	79.000	117.5000	129.9010	
SGO_1432	0.560	7.005	0.0084	0.0385	31.000	16.000	32.3873	24.4331	gyrB; DNA gyrase, B subunit
					44.500	16.500	44.5000	27.1312	
SGO_1434	-0.001	4.256	0.1190	0.9037	3.500	3.500	3.6566	5.3447	thiJ; 4-methyl-5(beta-hydroxyethyl)-thiazole monophosphate synthesis protein
					6.000	2.500	6.0000	4.1108	
SGO_1439	0.062	5.394	0.0593	0.4113	11.000	7.000	11.4923	10.6895	ftsX; cell division protein FtsX
					10.000	6.000	10.0000	9.8659	
SGO_1440	-1.158	7.555	0.0000	0.0000	27.000	42.500	28.2083	64.9004	cell-division ATP-binding protein FtsE
					30.000	39.500	30.0000	64.9505	
SGO_1441	-0.537	6.479	0.0212	0.1216	13.500	14.000	14.1042	21.3790	prfB; peptide chain release factor 2
					22.500	19.000	22.5000	31.2420	
SGO_1446	0.497	6.145	0.0048	0.0181	18.500	8.500	19.3279	12.9801	murF; UDP-N-acetylmuramoyl-tripeptide--D-alanyl-D-alanine ligase
					22.000	10.000	22.0000	16.4432	
SGO_1447	0.502	6.584	0.0037	0.0122	29.500	13.000	30.8202	19.8519	ddlA; D-Ala-D-Ala ligase
					25.500	12.000	25.5000	19.7318	
SGO_1450	-0.175	5.241	0.0703	0.4995	10.500	6.000	10.9699	9.1624	hypothetical protein SGO_1450
					7.000	6.500	7.0000	10.6881	
SGO_1451	0.866	6.911	0.0024	0.0066	37.000	17.000	38.6558	25.9602	frr; ribosome recycling factor
					38.500	10.500	38.5000	17.2653	
SGO_1452	0.790	6.962	0.0074	0.0313	45.500	15.000	47.5362	22.9060	pyrH; uridylate kinase
					32.000	13.500	32.0000	22.1983	
SGO_1453	1.869	3.936			11.500		12.0147		glycosyl transferase, family 8 SP1766
						2.000		3.2886	

Log_2 Ratios: -6 -4 -2 0 2 4 6

Show detected proteins only
Show all proteins
Filter by category:
ABC Transporter

Proteins found: 713

Test	Cutoff
q-Value	.005
p-Value	

	Signif	Direction	Applies To
red	yes	+	ratios, bars
yellow	no	n/a	bars
green	yes	−	ratios, bars
pink	yes	+	*p*- , *q*-Values
light green	yes	−	*p*- , *q*-Values

Dot Plots Dot Plots

Hendrickson *et al.*

Protein	SgPg vs SgFn Log2 Ratio	SgPg vs SgFn Log2 Sum	SgPg vs SgFn *q*-Value	SgPg vs SgFn *p*-Value	Raw *SgPg*	Raw *SgFn*	Normalized *SgPg*	Normalized *SgFn*	Description	Log2 Ratios (-6 -4 -2 0 2 4 6)
SGO_1455	1.187	9.545	0.0031	0.0097	208.000	72.500	217.3085	110.7125	rplA; ribosomal protein L1	(bar)
					304.000	70.000	304.0000	115.1021		
SGO_1456	1.186	7.235	0.0044	0.0159	41.500	18.500	43.3572	28.2508	rplK; ribosomal protein L11	(bar)
					61.000	11.000	61.0000	18.0875		
SGO_1458	-2.418	5.291	0.0023	0.0064	3.500	13.000	3.6566	19.8519	aha1; cation-transporting ATPase yfgQ	(bar)
					2.500	8.000	2.5000	13.1545		
SGO_1459	-3.101	4.837	0.0133	0.0686	1.500	8.000	1.5671	12.2165	glyoxylase family protein	(bar)
						9.000		14.7988		
SGO_1460	1.242	5.000	0.0001	0.0000	11.000	3.000	11.4923	4.5812	DNA translocase ftsK	(bar)
					11.000	3.000	11.0000	4.9329		
SGO_1463	0.841	5.569	0.0059	0.0232	16.000	7.000	16.7160	10.6895	peptidyl-prolyl cis-trans isomerase	(bar)
					13.500	4.000	13.5000	6.5773		
SGO_1465	1.867	6.138	0.0013	0.0026	30.000	4.500	31.3426	6.8718	ABC transporter, ATP-binding protein SP0770	(bar)
					24.000	5.000	24.0000	8.2216		
SGO_1466	-0.483	5.149	0.0044	0.0157	8.000	6.500	8.3580	9.9259	MTA/SAH nucleosidase	(bar)
					6.500	6.500	6.5000	10.6881		
SGO_1469	0.705	7.228	0.0004	0.0003	43.000	19.000	44.9244	29.0143	glmU; UDP-N-acetylglucosamine pyrophosphorylase	(bar)
					48.000	17.000	48.0000	27.9534		
SGO_1472	-0.627	4.934	0.0371	0.2402	7.500	8.500	7.8356	12.9801	acetyltransferase, GNAT family	(bar)
					4.000	3.500	4.0000	5.7551		
SGO_1487	-3.229	5.637	0.0167	0.0923		17.500		26.7237	LPXTG cell wall surface protein, Cna protein B-type domain	(bar)
					2.500	12.500	2.5000	20.5539		
SGO_1493	-1.164	5.045	0.0191	0.1084		8.000		12.2165	hypothetical protein SGO_1493	(bar)
					6.000	9.000	6.0000	14.7988		

- Show detected proteins only
- Show all proteins
- Filter by category: ABC Transporter

Proteins found: 713

Test	Cutoff
***q*-Value**	.005
p-Value	

	Signif	Direction	Applies To
red	yes	+	ratios, bars
yellow	no	n/a	bars
green	yes	−	ratios, bars
pink	yes	+	*p*- , *q*-Values
light green	yes	−	*p*- , *q*-Values

Dot Plots | Dot Plots

Hendrickson *et al.*

Protein	SgPg vs SgFn Log_2 Ratio	Log_2 Sum	*q*-Value	*p*-Value	Raw *SgPg*	Raw *SgFn*	Normalized *SgPg*	Normalized *SgFn*	Description
**SGO_1512**	-0.358	3.726	0.0645	0.4512	3.500	2.500	3.6566	3.8177	lacG; 6-phospho-beta-galactosidase
						3.500		5.7551	
**SGO_1529**	-0.089	4.876	0.1076	0.8056	8.500	5.500	8.8804	8.3989	hypothetical protein SGO_1529
					5.500	4.000	5.5000	6.5773	
**SGO_1530**	0.700	7.741	0.0048	0.0180	66.000	32.500	68.9537	49.6297	methionine-tRNA ligase
					62.500	20.000	62.5000	32.8863	
**SGO_1531**	0.677	5.972	0.0060	0.0243	20.000	9.500	20.8950	14.5072	xth; exodeoxyribonuclease III
					17.500	6.000	17.5000	9.8659	
**SGO_1534**	-0.106	5.567	0.1294	0.9952	15.500	8.000	16.1937	12.2165	ArsC family
					7.500	7.000	7.5000	11.5102	
**SGO_1536**	0.903	5.581	0.0127	0.0647	19.000	6.500	19.8503	9.9259	conserved hypothetical protein TIGR00096
					11.500	4.000	11.5000	6.5773	
**SGO_1539**	0.379	5.928	0.0213	0.1226	15.000	10.500	15.6713	16.0342	tmk; thymidylate kinase
					18.500	6.500	18.5000	10.6881	
**SGO_1541**	-1.273	6.843	0.0107	0.0508	15.500	18.000	16.1937	27.4872	atpC; ATP synthase F1, epsilon subunit
					16.000	33.500	16.0000	55.0846	
**SGO_1542**	-1.626	10.208	0.0000	0.0000	134.000	290.500	139.9968	443.6134	atpD; ATP synthase F1, beta subunit
					149.500	273.500	149.5000	449.7204	
**SGO_1543**	-1.674	7.196	0.0013	0.0029	17.000	41.500	17.7608	63.3733	atpG; ATP synthase F1, gamma subunit
					17.000	29.500	17.0000	48.5073	
**SGO_1544**	-0.311	9.368	0.0025	0.0072	137.500	125.500	143.6535	191.6471	atpA; ATP synthase F1, alpha subunit
					151.000	106.000	151.0000	174.2975	
**SGO_1545**	-1.684	7.176	0.0007	0.0008	16.500	33.000	17.2384	50.3933	atpH; ATP synthase F1, delta subunit
					17.000	36.500	17.0000	60.0175	

Log_2 Ratios: -6 -4 -2 0 2 4 6

Show detected proteins only
Show all proteins
Filter by category:
ABC Transporter

Proteins found: 713

Test	Cutoff
***q*-Value**	**.005**
p-Value	

	Signif	Direction	Applies To
red	yes	+	ratios, bars
yellow	no	n/a	bars
green	yes	−	ratios, bars
pink	yes	+	*p*- , *q*-Values
light green	yes	−	*p*- , *q*-Values

Dot Plots | Dot Plots

Protein	SgPg vs SgFn Log2 Ratio	Log2 Sum	q-Value	p-Value	Raw SgPg	Raw SgFn	Normalized SgPg	Normalized SgFn	Description
SGO_1546	0.130	7.047	0.1240	0.9498	37.000	30.000	38.6558	45.8121	atpF; ATP synthase F0, B subunit
					28.000	12.000	28.0000	19.7318	
SGO_1550	0.224	7.536	0.0179	0.1003	49.500	25.000	51.7152	38.1767	glgP-1; glycogen phosphorylase
					48.000	29.000	48.0000	47.6852	
SGO_1551	-1.718	6.594	0.0009	0.0014	14.000	25.500	14.6265	38.9402	glgA; Glycogen synthase
					8.500	21.000	8.5000	34.5306	
SGO_1552	-0.443	7.456	0.0307	0.1890	47.000	35.500	49.1034	54.2109	glgD; glucose-1-phosphate adenylyltransferase, GlgD subunit
					27.000	27.500	27.0000	45.2187	
SGO_1553	-0.465	7.805	0.0137	0.0711	45.000	50.500	47.0139	77.1170	glgC; glucose-1-phosphate adenylyltransferase
					46.000	32.500	46.0000	53.4403	
SGO_1554	-1.204	8.243	0.0008	0.0013	44.500	75.000	46.4915	114.5301	glgB; 1,4-alpha-glucan branching enzyme
					45.000	59.000	45.0000	97.0146	
SGO_1555	-0.227	10.842	0.0007	0.0011	410.000	329.500	428.3485	503.1690	ptsI; phosphoenolpyruvate-protein phosphotransferase
					417.500	296.000	417.5000	486.7175	
SGO_1556	-0.374	11.298	0.0151	0.0799	455.500	515.500	475.8847	787.2038	phosphocarrier protein HPr
					622.500	384.500	622.5000	632.2395	
SGO_1558	-0.663	8.459	0.0051	0.0197	64.500	81.000	67.3865	123.6925	nrdE; ribonucleoside-diphosphate reductase large chain
					68.000	56.500	68.0000	92.9039	
SGO_1559	0.844	7.748	0.0005	0.0005	69.000	24.500	72.0879	37.4132	ribonucleoside-diphosphate reductase, beta subunit
					66.000	24.000	66.0000	39.4636	
SGO_1570	0.677	8.785	0.0044	0.0160	139.500	65.000	145.7430	99.2594	alaS; alanyl-tRNA synthetase
					124.500	43.500	124.5000	71.5277	
SGO_1573	-1.155	3.882	0.0245	0.1448	2.500	5.000	2.6119	7.6353	O-methyltransferase family protein
					4.500		4.5000		

Log2 Ratios: -6 -4 -2 0 2 4 6

Show detected proteins only
Show all proteins
Filter by category:
ABC Transporter

Proteins found: 713

Test	Cutoff
q-Value	.005
p-Value	

	Signif	Direction	Applies To
red	yes	+	ratios, bars
yellow	no	n/a	bars
green	yes	−	ratios, bars
pink	yes	+	p- , q-Values
light green	yes	−	p- , q-Values

Dot Plots Dot Plots

Protein	*SgPg vs SgFn* Log$_2$ Ratio	Log$_2$ Sum	*q*-Value	*p*-Value	Raw *SgPg*	Raw *SgFn*	Normalized *SgPg*	Normalized *SgFn*	Description
SGO_1574	0.417	8.805	0.0019	0.0047	123.500	67.500	129.0269	103.0771	pepF-1; oligoendopeptidase F
					126.500	54.000	126.5000	88.7931	
SGO_1580	1.452	4.570	0.0007	0.0009	9.000	2.000	9.4028	3.0541	PTS system, Lactose/Cellobiose specific IIB subunit
					8.000	2.000	8.0000	3.2886	
SGO_1587	-0.068	5.865	0.1190	0.9045	17.500	8.000	18.2832	12.2165	queA; S-adenosylmethionine:tRNA ribosyltransferase-isomerase
					10.500	10.500	10.5000	17.2653	
SGO_1591	-2.605	7.184	0.0002	0.0001	12.500	40.000	13.0594	61.0827	arcC; carbamate kinase
					8.000	38.500	8.0000	63.3062	
SGO_1592	-3.079	10.535	0.0008	0.0012	74.500	379.500	77.8341	579.5225	arcB; ornithine carbamoyltransferase
					78.000	455.000	78.0000	748.1637	
SGO_1593	-2.641	9.840	0.0002	0.0001	58.000	270.000	60.5956	412.3085	arcA; arginine deiminase
					66.000	229.500	66.0000	377.3705	
SGO_1599	-1.637	10.158	0.0003	0.0002	127.500	298.500	133.2059	455.8299	sodA; manganese-dependent superoxide dismutase
					144.500	248.500	144.5000	408.6125	
SGO_1604	-0.644	4.907	0.0028	0.0085	6.500	6.000	6.7909	9.1624	acyltransferase family protein
					5.000	5.500	5.0000	9.0437	
SGO_1605	1.730	4.362	0.0005	0.0005	7.000	1.500	7.3133	2.2906	P-type ATPase, metal cation transport
					8.500	1.500	8.5000	2.4665	
SGO_1609	1.437	6.760	0.0001	0.0000	37.000	10.000	38.6558	15.2707	ATP-dependent RNA helicase, DEAD/DEAH box family
					40.500	8.500	40.5000	13.9767	
SGO_1617	-0.123	6.060	0.1068	0.7985	11.000	9.500	11.4923	14.5072	prfC; peptide chain release factor 3
					21.000	12.000	21.0000	19.7318	
SGO_1619	1.170	7.584	0.0024	0.0068	73.000	19.000	76.2669	29.0143	cation-transporting ATPase, E1-E2 family
					57.000	18.000	57.0000	29.5977	

Log$_2$ Ratios: -6 -4 -2 0 2 4 6

Show detected proteins only
Show all proteins
Filter by category: ABC Transporter

Proteins found: 713

Test	Cutoff
q-Value	.005
p-Value	

Color	Signif	Direction	Applies To
red	yes	+	ratios, bars
yellow	no	n/a	bars
green	yes	−	ratios, bars
pink	yes	+	*p*- , *q*-Values
light green	yes	−	*p*- , *q*-Values

Dot Plots Dot Plots

Hendrickson *et al.*

Protein	*SgPg vs SgFn* Log$_2$ Ratio	Log$_2$ Sum	*q*-Value	*p*-Value	Raw *SgPg*	Raw *SgFn*	Normalized *SgPg*	Normalized *SgFn*	Description
**SGO_1621**	-0.151	5.237	0.0278	0.1678	8.000	6.000	8.3580	9.1624	HD domain protein
					9.500	6.500	9.5000	10.6881	
**SGO_1622**	-1.521	5.844	0.0006	0.0006	7.000	15.000	7.3133	22.9060	Cof family protein
					7.500	12.000	7.5000	19.7318	
**SGO_1623**	-0.507	5.952	0.0012	0.0023	12.500	12.500	13.0594	19.0884	murM; MurM
					12.500	10.500	12.5000	17.2653	
**SGO_1624**	-1.064	5.206	0.0060	0.0240	6.000	10.000	6.2685	15.2707	murN; MurN protein
					5.500	6.000	5.5000	9.8659	
**SGO_1625**	-0.730	7.277	0.0008	0.0013	29.000	30.000	30.2978	45.8121	acetoin utilization putative/CBS domain protein
					28.000	31.000	28.0000	50.9738	
**SGO_1626**	1.305	5.463	0.0170	0.0947	16.000		16.7160		branched-chain amino acid ABC transporter, ATP-binding protein
					20.000	4.500	20.0000	7.3994	
**SGO_1630**	2.093	8.698	0.0000	0.0000	162.000	27.500	169.2499	41.9944	branched-chain amino acid ABC transporter, amino acid-binding protein
					167.000	22.500	167.0000	36.9971	
**SGO_1632**	-1.451	7.500	0.0023	0.0064	22.500	36.500	23.5069	55.7380	clpP; ATP-dependent Clp protease, proteolytic subunit ClpP
					24.500	47.000	24.5000	77.2828	
**SGO_1633**	-0.346	8.175	0.0121	0.0602	60.000	60.000	62.6851	91.6241	upp; uracil phosphoribosyltransferase
					64.000	43.000	64.0000	70.7056	
**SGO_1634**	-0.133	4.535	0.1063	0.7935	7.000	4.000	7.3133	6.1083	magnesium-translocating P-type ATPase
					4.000	3.500	4.0000	5.7551	
**SGO_1638**	-0.488	5.348	0.0125	0.0623	7.500	6.500	7.8356	9.9259	murE; UDP-N-acetylmuramoylalanyl-D-glutamate--2,6-diaminopimelate ligase
					9.000	8.500	9.0000	13.9767	
**SGO_1648**	0.636	8.950	0.0052	0.0203	124.500	62.000	130.0717	94.6782	ppx1; inorganic pyrophosphatase, manganese-dependent
					172.000	59.500	172.0000	97.8368	

Log$_2$ Ratios: -6 -4 -2 0 2 4 6

Show detected proteins only
Show all proteins
Filter by category: ABC Transporter

Proteins found: 713

Test	Cutoff
***q*-Value**	**.005**
p-Value	

Color	Signif	Direction	Applies To
red	yes	+	ratios, bars
yellow	no	n/a	bars
green	yes	−	ratios, bars
pink	yes	+	*p*-, *q*-Values
light green	yes	−	*p*-, *q*-Values

Dot Plots | Dot Plots

Hendrickson *et al.*

Protein	*SgPg vs SgFn* Log$_2$ Ratio	Log$_2$ Sum	*q*-Value	*p*-Value	Raw *SgPg*	Raw *SgFn*	Normalized *SgPg*	Normalized *SgFn*	Description	Log$_2$ Ratios
SGO_1649	-1.939	5.143	0.0006	0.0007	2.500	9.000	2.6119	13.7436	act; pyruvate formate-lyase-activating enzyme	
					5.000	8.500	5.0000	13.9767		
SGO_1652	1.008	6.371	0.0005	0.0005	26.500	10.000	27.6859	15.2707	intracellular glycosyl hydrolase	
					27.500	7.500	27.5000	12.3324		
SGO_1653	0.013	7.115	0.1226	0.9373	32.500	20.500	33.9545	31.3049	trehalose PTS enzyme II	
					35.500	23.000	35.5000	37.8193		
SGO_1655	-0.821	4.901	0.0162	0.0886	4.000	4.500	4.1790	6.8718	CBS domain protein/possible hemolysin	
					6.500	7.500	6.5000	12.3324		
SGO_1666	-1.754	7.016	0.0001	0.0000	15.500	33.000	16.1937	50.3933	trkA; potassium uptake protein, Trk family	
					13.500	30.000	13.5000	49.3295		
SGO_1669	0.160	6.308	0.0762	0.5497	22.500	15.000	23.5069	22.9060	ribosomal large subunit pseudouridine synthase B	
					18.000	9.000	18.0000	14.7988		
SGO_1674	-0.983	5.062	0.0007	0.0010	6.000	7.500	6.2685	11.4530	phosphodiesterase, MJ0936 family	
					5.000	6.500	5.0000	10.6881		
SGO_1675	-0.908	5.903	0.0027	0.0079	8.500	11.500	8.8804	17.5613	HAM1 protein-like protein	
					12.000	13.000	12.0000	21.3761		
SGO_1676	0.694	5.633	0.0003	0.0002	14.500	6.500	15.1489	9.9259	murI; glutamate racemase	
					15.500	5.500	15.5000	9.0437		
SGO_1678	-1.251	5.548	0.0083	0.0379	10.500	9.000	10.9699	13.7436	lysA; diaminopimelate decarboxylase	
					4.000	11.000	4.0000	18.0875		
SGO_1679	1.095	9.069	0.0023	0.0062	198.000	60.000	206.8610	91.6241	phosphotransferase system enzyme II	
					159.500	48.000	159.5000	78.9272		
SGO_1681	0.852	8.227	0.0011	0.0020	93.000	30.500	97.1620	46.5756	PTS system, mannose/fructose/sorbose family, IID component	
					95.000	37.000	95.0000	60.8397		

Log$_2$ Ratios axis: -6 -4 -2 0 2 4 6

Show detected proteins only
Show all proteins
Filter by category:
ABC Transporter

Proteins found: 713

Test	Cutoff
***q*-Value**	**.005**
p-Value	

	Signif	Direction	Applies To
red	yes	+	ratios, bars
yellow	no	n/a	bars
green	yes	−	ratios, bars
pink	yes	+	*p*- , *q*-Values
light green	yes	−	*p*- , *q*-Values

Dot Plots Dot Plots

Protein	SgPg vs SgFn Log_2 Ratio	Log_2 Sum	*q*-Value	*p*-Value	Raw *SgPg*	Raw *SgFn*	Normalized *SgPg*	Normalized *SgFn*	Description
SGO_1683	0.380	7.907	0.0346	0.2185	67.000	44.500	69.9984	67.9545	serS; seryl-tRNA synthetase
					63.500	23.500	63.5000	38.6414	
SGO_1685	1.647	7.820	0.0001	0.0000	84.000	17.500	87.7592	26.7237	putative peroxidase / antioxidase
					83.500	17.000	83.5000	27.9534	
SGO_1687	0.295	7.374	0.0188	0.1060	46.500	28.000	48.5810	42.7579	accA; acetyl-CoA carboxylase, carboxyl transferase, alpha subunit
					42.500	19.500	42.5000	32.0642	
SGO_1688	-0.504	6.188	0.0000	0.0000	14.500	14.000	15.1489	21.3790	accD; acetyl-CoA carboxylase, carboxyl transferase, beta subunit
					15.000	13.000	15.0000	21.3761	
SGO_1689	-0.538	7.735	0.0021	0.0051	40.000	38.500	41.7901	58.7921	accC; acetyl-CoA carboxylase, biotin carboxylase
					45.000	41.000	45.0000	67.4170	
SGO_1690	0.686	6.080	0.0188	0.1070	16.000	6.000	16.7160	9.1624	fabZ; beta-hydroxyacyl-(acyl-carrier-protein) dehydratase FabZ
					24.500	10.500	24.5000	17.2653	
SGO_1691	1.481	7.734	0.0013	0.0027	77.500	24.500	80.9683	37.4132	accB; acetyl-CoA carboxylase, biotin carboxyl carrier protein
					74.000	12.500	74.0000	20.5539	
SGO_1692	-0.657	10.055	0.0015	0.0034	196.000	197.500	204.7715	301.5960	3-oxoacyl-[acyl-carrier-protein] synthase
					207.500	213.000	207.5000	350.2393	
SGO_1693	-0.282	7.146	0.0033	0.0107	32.500	25.000	33.9545	38.1767	fabG; 3-oxoacyl-(acyl-carrier-protein) reductase
					30.000	24.000	30.0000	39.4636	
SGO_1694	0.671	6.845	0.0067	0.0276	30.000	12.000	31.3426	18.3248	fabD; malonyl CoA-acyl carrier protein transacylase
					39.000	16.000	39.0000	26.3091	
SGO_1695	0.782	9.289	0.0030	0.0092	212.000	71.000	221.4875	108.4219	enoyl-acyl carrier protein(ACP) reductase
					175.000	73.500	175.0000	120.8572	
SGO_1699	-0.547	5.849	0.0043	0.0152	13.000	11.000	13.5818	16.7978	transcriptional regulator, MarR family
					10.000	10.500	10.0000	17.2653	

Log_2 Ratios: -6 -4 -2 0 2 4 6

Show detected proteins only
Show all proteins
Filter by category:
ABC Transporter

Proteins found: 713

Test	Cutoff
q-Value	.005
p-Value	

	Signif	Direction	Applies To
red	yes	+	ratios, bars
yellow	no	n/a	bars
green	yes	−	ratios, bars
pink	yes	+	*p*- , *q*-Values
light green	yes	−	*p*- , *q*-Values

Dot Plots Dot Plots

Hendrickson *et al.*

Protein	SgPg vs SgFn Log_2 Ratio	Log_2 Sum	*q*-Value	*p*-Value	Raw *SgPg*	Raw *SgFn*	Normalized *SgPg*	Normalized *SgFn*	Description
SGO_1700	-2.813	7.773	0.0007	0.0010	13.500	55.500	14.1042	84.7523	enoyl-CoA hydratase/isomerase family protein
					13.000	65.000	13.0000	106.8805	
SGO_1701	0.028	7.657	0.1026	0.7627	49.000	30.000	51.1929	45.8121	aspartate kinase
					50.500	33.000	50.5000	54.2624	
SGO_1708	0.630	8.003	0.0022	0.0059	70.500	36.500	73.6550	55.7380	amiF; Oligopeptide transport ATP-binding protein amiF
					82.000	27.500	82.0000	45.2187	
SGO_1709	1.471	7.990	0.0006	0.0007	83.500	20.000	87.2368	30.5414	amiE; Oligopeptide transport ATP-binding protein
					99.500	22.500	99.5000	36.9971	
SGO_1711	1.224	6.684	0.0002	0.0001	33.500	10.500	34.9992	16.0342	hppB; Oligopeptide transport system permease
					37.000	9.000	37.0000	14.7988	
SGO_1712	0.912	9.612	0.0027	0.0079	243.000	111.000	253.8748	169.5046	hppA; oligopeptide-binding lipoprotein
					252.500	65.000	252.5000	106.8805	
SGO_1713	-0.172	9.436	0.0368	0.2370	160.000	137.500	167.1604	209.9719	hppG; oligopeptide-binding lipoprotein
					157.000	96.500	157.0000	158.6765	
SGO_1715	-0.060	8.185	0.0447	0.2998	66.000	51.000	68.9537	77.8805	hppH; oligopeptide-binding lipoprotein
					73.500	43.000	73.5000	70.7056	
SGO_1716	-0.045	8.522	0.0912	0.6716	90.000	70.000	94.0277	106.8948	oligopeptide binding protein
					86.000	49.000	86.0000	80.5715	
SGO_1717	-1.505	3.809	0.0180	0.1005	2.000	3.500	2.0895	5.3447	pbp3; penicillin-binding protein 3
						4.000		6.5773	
SGO_1718	0.568	6.721	0.0097	0.0454	31.500	11.000	32.9097	16.7978	sufB-1; FeS assembly protein SufB
					29.500	16.000	29.5000	26.3091	
SGO_1721	0.197	7.090	0.0384	0.2496	32.500	24.000	33.9545	36.6496	sufD; FeS assembly protein SufD
					38.500	16.500	38.5000	27.1312	

Log_2 Ratios: -6 -4 -2 0 2 4 6

Show detected proteins only
Show all proteins
Filter by category: ABC Transporter

Proteins found: 713

Test	Cutoff
q-Value	.005
p-Value	

	Signif	Direction	Applies To
red	yes	+	ratios, bars
yellow	no	n/a	bars
green	yes	−	ratios, bars
pink	yes	+	*p*- , *q*-Values
light green	yes	−	*p*- , *q*-Values

Dot Plots Dot Plots

Protein	SgPg vs SgFn Log2 Ratio	SgPg vs SgFn Log2 Sum	SgPg vs SgFn *q*-Value	SgPg vs SgFn *p*-Value	Raw *SgPg*	Raw *SgFn*	Normalized *SgPg*	Normalized *SgFn*	Description
SGO_1722	-0.285	6.570	0.0075	0.0318	19.000	18.000	19.8503	27.4872	sufC; FeS assembly ATPase SufC
					23.000	15.000	23.0000	24.6647	
SGO_1724	-0.717	2.988							mecA; Adapter protein mec
					3.000	3.000	3.0000	4.9329	
SGO_1727	0.773	6.329	0.0041	0.0141	22.000	11.500	22.9846	17.5613	amino acid ABC transporter, amino acid-binding/permease protein
					27.500	7.500	27.5000	12.3324	
SGO_1728	-0.625	6.253	0.0044	0.0160	17.000	14.500	17.7608	22.1425	glnQ; glutamine ABC transporter ATP-binding protein
					12.500	14.500	12.5000	23.8426	
SGO_1729	-2.961	8.759	0.0025	0.0072	20.500	97.000	21.4174	148.1256	hypothetical protein SGO_1729
					27.000	144.000	27.0000	236.7815	
SGO_1730	-1.547	9.192	0.0021	0.0053	60.500	163.500	63.2075	249.6757	SPFH domain/Band 7 family
					86.000	113.000	86.0000	185.8077	
SGO_1731	2.314	5.420	0.0013	0.0029	14.500	2.000	15.1489	3.0541	DNA-binding response regulator
					20.500	2.500	20.5000	4.1108	
SGO_1735	0.211	8.904	0.0244	0.1434	123.500	82.500	129.0269	125.9831	hypothetical protein SGO_1735
					127.000	59.000	127.0000	97.0146	
SGO_1736	-0.273	6.124	0.0440	0.2941	17.000	15.500	17.7608	23.6696	alkaline shock protein
					13.500	9.000	13.5000	14.7988	
SGO_1745	0.915	11.782	0.0003	0.0002	1075.000	422.000	1123.1089	644.4229	fba; fructose-1,6-bisphosphate aldolase, class II
					1177.500	351.000	1177.5000	577.1549	
SGO_1747	-1.902	3.525	0.0129	0.0660	2.500		2.6119		hypothetical protein SGO_1747
					1.500	4.500	1.5000	7.3994	
SGO_1748	1.153	5.008	0.0403	0.2643	9.500		9.9251		pyrG; CTP synthase
					16.500	3.500	16.5000	5.7551	

Show detected proteins only
Show all proteins
Filter by category:
ABC Transporter

Proteins found: 713

Test	Cutoff
q-Value	.005
p-Value	

Color	Signif	Direction	Applies To
red	yes	+	ratios, bars
yellow	no	n/a	bars
green	yes	−	ratios, bars
pink	yes	+	*p*- , *q*-Values
light green	yes	−	*p*- , *q*-Values

Dot Plots | Dot Plots

Hendrickson *et al.*

Protein	SgPg vs SgFn Log2 Ratio	Log2 Sum	q-Value	p-Value	Raw SgPg	Raw SgFn	Normalized SgPg	Normalized SgFn	Description
SGO_1749	0.024	6.688	0.1201	0.9143	21.000	19.500	21.9398	29.7778	manA; mannose-6-phosphate isomerase, class I
					30.000	13.000	30.0000	21.3761	
SGO_1755	1.873	6.648	0.0011	0.0021	42.500	7.000	44.4020	10.6895	scrK; fructokinase
					34.500	6.500	34.5000	10.6881	
SGO_1757	0.959	8.074	0.0028	0.0085	82.000	38.000	85.6697	58.0286	glmS; glucosamine--fructose-6-phosphate aminotransferase
					90.500	21.500	90.5000	35.3528	
SGO_1760	-1.133	3.260							DNA-binding response regulator
					3.000	4.000	3.0000	6.5773	
SGO_1763	0.588	7.148	0.0069	0.0284	36.000	21.000	37.6111	32.0684	ABC transporter, substrate-binding protein SP0092
					47.500	15.000	47.5000	24.6647	
SGO_1768	-2.439	5.865	0.0010	0.0018	2.500	17.000	2.6119	25.9602	glycosyl hydrolase, family 38
					7.500	13.500	7.5000	22.1983	
SGO_1774	-1.588	6.190	0.0005	0.0006	10.500	18.500	10.9699	28.2508	alcohol dehydrogenase, zinc-containing
					7.500	16.000	7.5000	26.3091	
SGO_1784	0.233	8.016	0.0129	0.0663	70.000	42.500	73.1327	64.9004	leuS; leucyl-tRNA synthetase
					66.500	33.000	66.5000	54.2624	
SGO_1799	-1.037	9.053	0.0001	0.0000	80.500	117.500	84.1026	179.4305	endopeptidase O
					90.000	108.000	90.0000	177.5861	
SGO_1802	3.394	7.883	0.0013	0.0025	119.500	9.000	124.8479	13.7436	Metal ABC transporter substrate-binding lipoprotein precursor
					90.000	4.500	90.0000	7.3994	
SGO_1803	0.481	6.697	0.0022	0.0059	29.500	15.500	30.8202	23.6696	tpx; thioredoxin peroxidase
					29.500	12.000	29.5000	19.7318	
SGO_1804	1.136	5.246	0.0003	0.0002	13.000	4.000	13.5818	6.1083	hutI; imidazolonepropionase
					12.500	3.500	12.5000	5.7551	

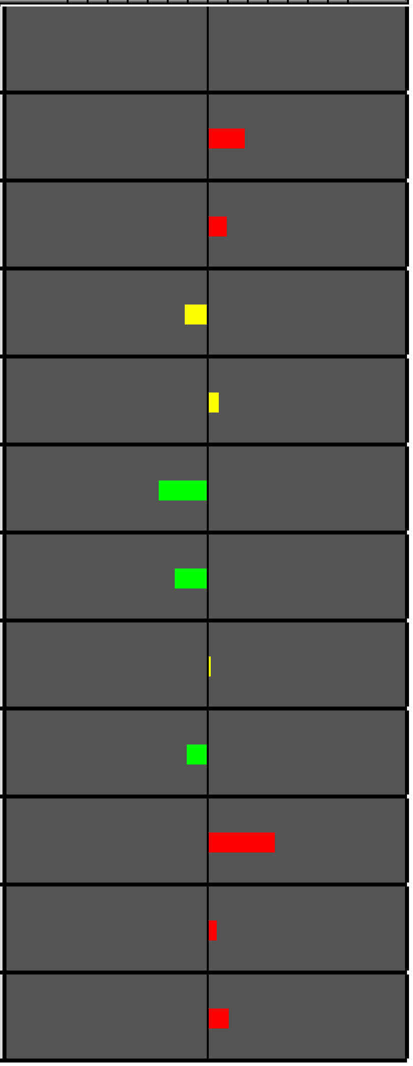

Show detected proteins only
Show all proteins
Filter by category: ABC Transporter

Proteins found: 713

Test	Cutoff
q-Value	.005
p-Value	

	Signif	Direction	Applies To
red	yes	+	ratios, bars
yellow	no	n/a	bars
green	yes	−	ratios, bars
pink	yes	+	p- , q-Values
light green	yes	−	

Dot Plots Dot Plots

Protein	SgPg vs SgFn Log_2 Ratio	Log_2 Sum	*q*-Value	*p*-Value	Raw *SgPg*	Raw *SgFn*	Normalized *SgPg*	Normalized *SgFn*	Description	Log_2 Ratios
**SGO_1805**	3.863	6.129	0.0204	0.1167	38.000	1.500	39.7006	2.2906	hutU; urocanate hydratase	
					28.000		28.0000			
**SGO_1808**	-1.367	4.133	0.0081	0.0358	4.000		4.1790		fhs-2; formate--tetrahydrofolate ligase	
					3.500	6.000	3.5000	9.8659		
**SGO_1811**	-0.540	4.773	0.0042	0.0146	7.500		7.8356		hutH; histidine ammonia-lyase	
					8.000	7.000	8.0000	11.5102		
**SGO_1816**	-0.731	5.031	0.0357	0.2282		9.500		14.5072	scaR; ScaR Manganese-dependent regulator of scaCBA	
					7.500	6.500	7.5000	10.6881		
**SGO_1822**	-1.893	6.278	0.0011	0.0022	8.000	17.500	8.3580	26.7237	relA; GTP diphosphokinase	
					8.000	21.000	8.0000	34.5306		
**SGO_1824**	0.331	5.742	0.0167	0.0927	14.000	6.500	14.6265	9.9259	prmA; ribosomal protein L11 methyltransferase	
					15.000	8.500	15.0000	13.9767		
**SGO_1827**	-1.094	4.383	0.0183	0.1026	4.000	7.000	4.1790	10.6895	hypothetical protein SGO_1827	
					6.000		6.0000			
**SGO_1828**	-0.938	4.525	0.0147	0.0777	2.500	6.000	2.6119	9.1624	ATPase, AAA family	
					5.500	3.500	5.5000	5.7551		
**SGO_1834**	-2.439	8.524	0.0013	0.0027	35.500	116.000	37.0887	177.1399	hypothetical protein SGO_1834	
					21.500	80.500	21.5000	132.3674		
**SGO_1835**	-1.923	4.470	0.0032	0.0102	3.500	9.500	3.6566	14.5072	hypothetical protein SGO_1835	
					4.000		4.0000			
**SGO_1843**	-0.001	7.961	0.1224	0.9344	67.000	41.500	69.9984	63.3733	pepS; aminopeptidase PepS	
					55.000	37.000	55.0000	60.8397		
**SGO_1844**	0.408	5.399	0.0074	0.0314	12.500	6.500	13.0594	9.9259	cbxX/cfqX family protein	
					11.000	5.000	11.0000	8.2216		

Show detected proteins only
Show all proteins
Filter by category:
ABC Transporter

Proteins found: 713

Test	Cutoff
***q*-Value**	**.005**
p-Value	

	Signif	Direction	Applies To
(red)	yes	+	ratios, bars
(yellow)	no	n/a	bars
(green)	yes	–	ratios, bars
(pink)	yes	+	*p*- , *q*-Values
(light green)	yes	–	*p*- , *q*-Values

Dot Plots Dot Plots

Protein	SgPg vs SgFn Log$_2$ Ratio	Log$_2$ Sum	*q*-Value	*p*-Value	Raw *SgPg*	Raw *SgFn*	Normalized *SgPg*	Normalized *SgFn*	Description
SGO_1847	-0.370	5.761	0.0377	0.2442	10.500	7.000	10.9699	10.6895	polC; DNA polymerase III, alpha subunit, Gram-positive type
					12.000	12.500	12.0000	20.5539	
SGO_1851	0.035	8.603	0.1049	0.7821	99.500	69.500	103.9529	106.1313	proS; prolyl-tRNA synthetase
					92.500	52.500	92.5000	86.3266	
SGO_1854	0.517	5.493	0.0244	0.1434	16.000	5.000	16.7160	7.6353	uppS; undecaprenyl diphosphate synthase
					10.000	6.500	10.0000	10.6881	
SGO_1856	-3.472	8.392	0.0042	0.0144	14.000	132.500	14.6265	202.3366	ATP-dependent proteinase ATP-binding chain
					12.000	65.000	12.0000	106.8805	
SGO_1858	-0.633	4.293	0.0040	0.0137	4.000	3.500	4.1790	5.3447	beta-fructofuranosidase/sucrose 6 phoshate hydrolase
					3.500	4.000	3.5000	6.5773	
SGO_1860	-0.721	8.138	0.0156	0.0833	53.000	74.500	55.3719	113.7666	5'-nucleotidase, lipoprotein e(P4) family
					48.500	39.000	48.5000	64.1283	
SGO_1862	-1.778	7.869	0.0006	0.0006	26.500	54.500	27.6859	83.2252	alkaline shock protein
					25.000	59.500	25.0000	97.8368	
SGO_1863	1.017	7.780	0.0009	0.0014	69.000	27.500	72.0879	41.9944	efp; Elongation factor P (EF-P)
					74.500	19.000	74.5000	31.2420	
SGO_1864	-1.379	7.834	0.0001	0.0000	30.000	53.000	31.3426	80.9346	X-Pro aminopeptidase
					32.000	51.000	32.0000	83.8601	
SGO_1865	-0.713	6.543	0.0014	0.0032	17.000	17.500	17.7608	26.7237	uvrA; excinuclease ABC, A subunit
					17.500	19.000	17.5000	31.2420	
SGO_1867	2.522	6.302	0.0002	0.0001	31.500	3.000	32.9097	4.5812	hypothetical protein SGO_1867
					34.000	4.500	34.0000	7.3994	
SGO_1879	0.132	7.433	0.0562	0.3870	46.000	23.500	48.0586	35.8861	rpsR; ribosomal protein S18
					42.000	28.500	42.0000	46.8630	

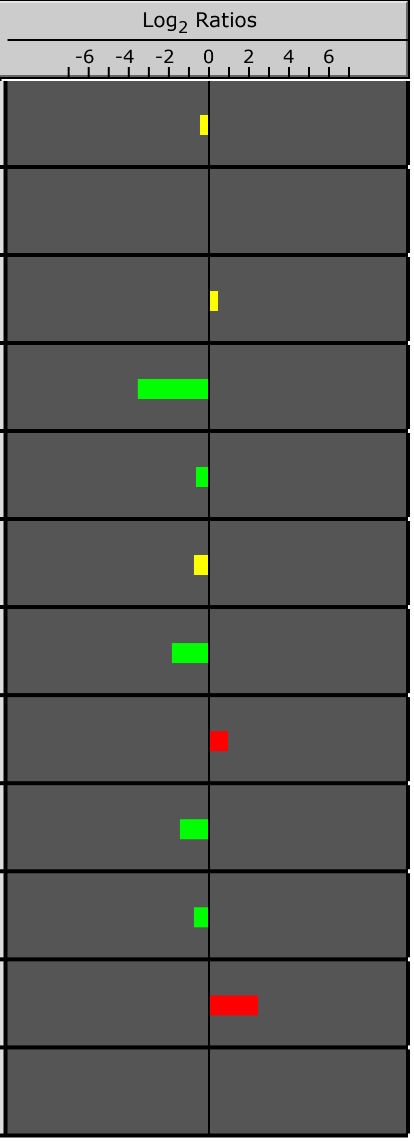

Show detected proteins only
Show all proteins
Filter by category:
ABC Transporter

Proteins found: 713

Test	Cutoff
***q*-Value**	**.005**
p-Value	

	Signif	Direction	Applies To
red	yes	+	ratios, bars
yellow	no	n/a	bars
green	yes	−	ratios, bars
pink	yes	+	*p*- , *q*-Values
light green	yes	−	

Dot Plots Dot Plots

Protein	SgPg vs SgFn				Raw		Normalized		Description
	Log_2 Ratio	Log_2 Sum	*q*-Value	*p*-Value	*SgPg*	*SgFn*	*SgPg*	*SgFn*	
SGO_1880	1.192	7.919	0.0004	0.0003	76.500	24.000	79.9236	36.6496	ssb-1; single-strand binding protein
					88.500	22.500	88.5000	36.9971	
SGO_1881	1.188	9.153	0.0034	0.0109	214.000	44.500	223.5770	67.9545	rpsF; ribosomal protein S6
					170.000	65.500	170.0000	107.7027	
SGO_1882	0.395	5.908	0.0260	0.1553	13.500	10.000	14.1042	15.2707	folE; GTP cyclohydrolase I
					20.000	6.500	20.0000	10.6881	
SGO_1885	-2.739	11.639	0.0000	0.0000	200.000	909.500	208.9505	1388.8687	groL; 60 kDa chaperonin/groEL protein
					206.500	842.500	206.5000	1385.3362	
SGO_1886	-1.663	6.874	0.0125	0.0626	17.000	17.500	17.7608	26.7237	groES; chaperonin, 10 kDa
					9.500	38.500	9.5000	63.3062	
SGO_1892	-2.895	7.801	0.0024	0.0068	11.000	79.000	11.4923	120.6384	PTS system, fructose(mannose)-specific IIB
					14.500	46.500	14.5000	76.4607	
SGO_1898	-1.710	8.931	0.0007	0.0009	53.500	111.500	55.8943	170.2681	glutamyl aminopeptidase
					58.000	124.000	58.0000	203.8952	
SGO_1901	1.204	6.178	0.0069	0.0283	28.000	10.000	29.2531	15.2707	tRNA binding domain
					20.500	4.500	20.5000	7.3994	
SGO_1903	-0.484	7.863	0.0087	0.0402	44.500	51.000	46.4915	77.8805	ATP-dependent Zn protease
					50.000	35.500	50.0000	58.3732	
SGO_1912	-0.548	4.283	0.0072	0.0301	5.500		5.7461		Bifunctional folate synthesis protein
					5.500	5.000	5.5000	8.2216	
SGO_1914	-0.729	4.433	0.0011	0.0020	3.500	4.500	3.6566	6.8718	folP; dihydropteroate synthase
					4.500	4.000	4.5000	6.5773	
SGO_1916	0.333	8.040	0.0002	0.0001	70.500	37.500	73.6550	57.2651	ackA; acetate kinase
					73.000	36.000	73.0000	59.1954	

Log_2 Ratios: -6 -4 -2 0 2 4 6

Show detected proteins only
Show all proteins
Filter by category:
ABC Transporter

Proteins found: 713

Test	Cutoff
q-Value	.005
p-Value	

	Signif	Direction	Applies To
red	yes	+	ratios, bars
yellow	no	n/a	bars
green	yes	−	ratios, bars
pink	yes	+	*p*- , *q*-Values
light green	yes	−	*p*- , *q*-Values

Dot Plots Dot Plots

Hendrickson *et al.*

SgPg vs SgFn

Streptococcus gordonii

Hackett Laboratory UW

Protein	SgPg vs SgFn Log2 Ratio	Log2 Sum	*q*-Value	*p*-Value	Raw SgPg	Raw SgFn	Normalized SgPg	Normalized SgFn	Description
SGO_1917	0.195	4.942	0.0506	0.3436	9.000	4.000	9.4028	6.1083	hypothetical protein SGO_1917
					7.000	5.000	7.0000	8.2216	
SGO_1926	0.371	9.973	0.0006	0.0007	277.000	146.500	289.3964	223.7155	rpoC; DNA-directed RNA polymerase, beta chain
					277.500	130.500	277.5000	214.5832	
SGO_1927	0.571	9.680	0.0141	0.0739	228.000	141.000	238.2036	215.3166	rpoB; DNA-directed RNA polymerase, beta subunit
					244.500	74.500	244.5000	122.5015	
SGO_1929	1.102	8.867	0.0008	0.0013	164.500	48.000	171.8618	73.2993	tyrS; tyrosyl-tRNA synthetase
					147.000	45.500	147.0000	74.8164	
SGO_1936	1.236	6.497	0.0004	0.0004	32.000	9.000	33.4321	13.7436	adcA; metal-binding (Mn) permease precursor, lipoprotein
					30.000	8.000	30.0000	13.1545	
SGO_1957	-1.798	4.035	0.0345	0.2178		3.500		5.3447	hypothetical protein SGO_1957
					2.000	5.500	2.0000	9.0437	
SGO_1958	3.171	9.577	0.0002	0.0002	350.000	27.500	365.6633	41.9944	rplQ; ribosomal protein L17
					321.500	21.000	321.5000	34.5306	
SGO_1959	0.453	8.899	0.0029	0.0089	125.000	61.000	130.5941	93.1512	rpoA; DNA-directed RNA polymerase, alpha subunit
					145.000	66.000	145.0000	108.5249	
SGO_1960	0.749	7.652	0.0050	0.0192	56.500	30.500	59.0285	46.5756	rpsK; ribosomal protein S11
					66.000	18.000	66.0000	29.5977	
SGO_1961	2.673	9.052	0.0002	0.0001	231.500	27.000	241.8602	41.2308	rpsM; ribosomal protein S13p/S18e
					216.500	19.000	216.5000	31.2420	
SGO_1964	0.441	8.106	0.0161	0.0875	75.500	47.500	78.8788	72.5358	adk; Adenylate kinase (ATP-AMP transphosphorylase)
					78.000	28.000	78.0000	46.0408	
SGO_1966	0.648	8.744	0.0282	0.1723	94.000	75.000	98.2067	114.5301	rplO; ribosomal protein L15
					160.000	34.000	160.0000	55.9067	

Log2 Ratios: -6 -4 -2 0 2 4 6

Show detected proteins only
Show all proteins
Filter by category:
ABC Transporter

Proteins found: 713

Test	Cutoff
q-Value	.005
p-Value	

	Signif	Direction	Applies To
(red)	yes	+	ratios, bars
(yellow)	no	n/a	bars
(green)	yes	−	ratios, bars
(pink)	yes	+	*p*- , *q*-Values
(light green)	yes	−	*p*- , *q*-Values

Dot Plots Dot Plots

Hendrickson *et al.*

Streptococcus gordonii

Protein	SgPg vs SgFn Log_2 Ratio	Log_2 Sum	*q*-Value	*p*-Value	Raw *SgPg*	Raw *SgFn*	Normalized *SgPg*	Normalized *SgFn*	Description
SGO_1967	2.111	8.152	0.0006	0.0007	116.000	13.000	121.1913	19.8519	50S ribosomal protein L30 -related protein
					108.000	21.500	108.0000	35.3528	
SGO_1968	1.261	9.565	0.0006	0.0007	263.000	84.000	274.7699	128.2737	rpsE; ribosomal protein S5
					258.000	58.500	258.0000	96.1925	
SGO_1969	1.721	9.530	0.0005	0.0005	270.500	71.000	282.6055	108.4219	rplR; ribosomal protein L18
					281.000	41.000	281.0000	67.4170	
SGO_1970	1.370	8.992	0.0003	0.0002	178.500	52.000	186.4883	79.4076	BL10; 50S ribosomal protein L6
					180.000	38.500	180.0000	63.3062	
SGO_1971	1.758	10.068	0.0002	0.0001	411.500	79.000	429.9156	120.6384	rpsH; ribosomal protein S8
					398.500	75.500	398.5000	124.1459	
SGO_1973	1.138	9.139	0.0002	0.0001	189.500	60.500	197.9806	92.3876	BL6; 50S ribosomal protein L5
					189.500	51.000	189.5000	83.8601	
SGO_1974	0.886	7.983	0.0021	0.0053	74.500	34.500	77.8341	52.6839	rplX; ribosomal protein L24
					85.500	22.500	85.5000	36.9971	
SGO_1975	0.875	8.196	0.0021	0.0051	92.500	40.500	96.6396	61.8463	rplN; ribosomal protein L14
					92.000	26.000	92.0000	42.7522	
SGO_1976	2.459	8.387	0.0007	0.0009	121.500	14.500	126.9374	22.1425	BS16; 30S ribosomal protein
					156.000	18.000	156.0000	29.5977	
SGO_1977	0.476	5.445	0.0390	0.2551	13.500	7.500	14.1042	11.4530	rpmC; ribosomal protein L29
					18.000		18.0000		
SGO_1978	1.680	8.238	0.0001	0.0000	110.500	25.000	115.4451	38.1767	rplP; ribosomal protein L16
					114.500	20.500	114.5000	33.7085	
SGO_1979	0.002	9.240	0.1243	0.9528	148.000	88.500	154.6234	135.1456	rpsC; ribosomal protein S3
					147.000	102.000	147.0000	167.7202	

Log_2 Ratios: -6 -4 -2 0 2 4 6

Show detected proteins only
Show all proteins
Filter by category:
ABC Transporter

Proteins found: 713

Test	Cutoff
***q*-Value**	**.005**
p-Value	

	Signif	Direction	Applies To
(red)	yes	+	ratios, bars
(yellow)	no	n/a	bars
(green)	yes	–	ratios, bars
(pink)	yes	+	*p*- , *q*-Values
(light green)	yes	–	*p*- , *q*-Values

Dot Plots Dot Plots

Hendrickson *et al.*

Protein	*SgPg vs SgFn* Log$_2$ Ratio	Log$_2$ Sum	*q*-Value	*p*-Value	Raw *SgPg*	Raw *SgFn*	Normalized *SgPg*	Normalized *SgFn*	Description
**SGO_1980**	1.141	8.930	0.0002	0.0002	164.000	53.500	171.3394	81.6982	rplV; ribosomal protein L22
					164.000	43.000	164.0000	70.7056	
**SGO_1981**	0.174	8.715	0.0107	0.0510	100.000	64.000	104.4752	97.7324	rpsS; ribosomal protein S19
					118.500	60.500	118.5000	99.4811	
**SGO_1982**	0.153	8.650	0.0843	0.6166	107.000	78.500	111.7885	119.8749	rplB; ribosomal protein L2
					97.000	44.500	97.0000	73.1721	
**SGO_1983**	1.231	7.880	0.0079	0.0346	91.000	14.500	95.0725	22.1425	rplW; ribosomal protein L23
					66.500	31.500	66.5000	51.7960	
**SGO_1984**	0.473	9.229	0.0014	0.0030	158.000	81.500	165.0709	124.4561	rplD; ribosomal protein L4/L1 family
					184.000	77.000	184.0000	126.6123	
**SGO_1985**	0.999	9.986	0.0136	0.0706	223.000	123.000	232.9798	187.8294	rplC; ribosomal protein L3
					453.000	85.500	453.0000	140.5890	
**SGO_1986**	0.777	7.231	0.0011	0.0020	42.500	19.000	44.4020	29.0143	rpsJ; ribosomal protein S10
					50.500	16.000	50.5000	26.3091	
**SGO_1989**	0.802	9.478	0.0007	0.0008	226.500	89.500	236.6364	136.6726	purA; adenylosuccinate synthetase
					216.500	75.000	216.5000	123.3237	
**SGO_1990**	-2.053	6.535	0.0013	0.0027	6.000	21.500	6.2685	32.8320	glutamate--cysteine ligase, putative/amino acid ligase, putative
					12.500	25.000	12.5000	41.1079	
**SGO_1991**	-2.454	5.522	0.0001	0.0000	3.000	12.500	3.1343	19.0884	hslO; 33 kDa chaperonin /Heat shock protein 33-like protein
					4.000	12.000	4.0000	19.7318	
**SGO_1993**	1.046	6.030	0.0009	0.0014	22.500	7.500	23.5069	11.4530	possible transcriptional regulator
					20.500	6.000	20.5000	9.8659	
**SGO_1998**	0.053	6.720	0.0848	0.6222	22.000	17.000	22.9846	25.9602	clpB; ATP-dependent Clp proteinase, ATP-binding chain
					31.000	15.500	31.0000	25.4869	

Log$_2$ Ratios: -6 -4 -2 0 2 4 6

Show detected proteins only
Show all proteins
Filter by category:
ABC Transporter

Proteins found: 713

Test	Cutoff
***q*-Value**	**.005**
p-Value	

Color	Signif	Direction	Applies To
red	yes	+	ratios, bars
yellow	no	n/a	bars
green	yes	–	ratios, bars
pink	yes	+	*p*- , *q*-Values
light green	yes	–	*p*- , *q*-Values

Dot Plots Dot Plots

Protein	*SgPg vs SgFn* Log$_2$ Ratio	Log$_2$ Sum	*q*-Value	*p*-Value	Raw *SgPg*	Raw *SgFn*	Normalized *SgPg*	Normalized *SgFn*	Description	Log$_2$ Ratios (-6 -4 -2 0 2 4 6)
**SGO_2000**	0.341	10.640	0.0032	0.0103	437.500	247.500	457.0792	377.9494	tsf; translation elongation factor Ts	
					434.000	199.000	434.0000	327.2189		
**SGO_2001**	0.226	10.354	0.0122	0.0605	347.500	180.000	363.0515	274.8723	rpsB; ribosomal protein S2	
					341.500	200.500	341.5000	329.6853		
**SGO_2005**	1.031	4.389	0.0158	0.0855	7.500		7.8356		LPXTG cell wall surface protein	
					9.000	2.500	9.0000	4.1108		
**SGO_2007**	-0.429	6.891	0.0009	0.0016	25.500	22.500	26.6412	34.3590	nusG; transcription termination/antitermination factor NusG	
					24.000	20.500	24.0000	33.7085		
**SGO_2033**	-1.280	7.538	0.0003	0.0003	26.500	41.000	27.6859	62.6098	nrdD; ribonucleoside-triphosphate reductase	
					26.500	42.000	26.5000	69.0613		
**SGO_2034**	-0.740	3.288			3.500	4.000	3.6566	6.1083	hypothetical protein SGO_2034	
**SGO_2042**	1.423	8.197	0.0021	0.0052	92.500	35.000	96.6396	53.4474	Bacterial protein of unknown function (DUF965) superfamily	
					114.500	17.500	114.5000	28.7755		
**SGO_2045**	3.160	9.855	0.0000	0.0000	389.500	32.000	406.9311	48.8662	recA; recA protein	
					426.000	27.000	426.0000	44.3965		
**SGO_2046**	-0.120	5.303	0.0188	0.1070	9.500	7.000	9.9251	10.6895	cinA; competence induced protein	
					9.000	6.000	9.0000	9.8659		
**SGO_2050**	1.133	3.770	0.0411	0.2701	4.000		4.1790		ruvA; Holliday junction DNA helicase RuvA	
					7.000	1.500	7.0000	2.4665		
**SGO_2053**	-0.856	5.395	0.0050	0.0190	5.500	9.500	5.7461	14.5072	DNA mismatch repair protein hexB	
					9.500	7.500	9.5000	12.3324		
**SGO_2056**	-1.072	6.628	0.0033	0.0109	12.500	19.000	13.0594	29.0143	mutS; DNA mismatch repair protein MutS	
					19.000	23.000	19.0000	37.8193		

◉ Show detected proteins only
○ Show all proteins
☐ Filter by category:
ABC Transporter

Proteins found: 713

Test	Cutoff
***q*-Value**	.005
p-Value	

	Signif	Direction	Applies To
red	yes	+	ratios, bars
yellow	no	n/a	bars
green	yes	−	ratios, bars
pink	yes	+	*p*- , *q*-Values
light green	yes	−	*p*- , *q*-Values

Dot Plots Dot Plots

SgPg vs SgFn	Streptococcus gordonii	Hackett Laboratory UW

Protein	SgPg vs SgFn Log$_2$ Ratio	Log$_2$ Sum	*q*-Value	*p*-Value	Raw SgPg	Raw SgFn	Normalized SgPg	Normalized SgFn	Description
SGO_2058	-0.325	9.745	0.0011	0.0020	188.000	152.500	196.4135	232.8779	argS; arginyl-tRNA synthetase
					184.500	148.500	184.5000	244.1809	
SGO_2060	0.399	7.832	0.0038	0.0130	61.500	29.000	64.2523	44.2850	aspS-1; aspartyl-tRNA synthetase
					65.000	33.000	65.0000	54.2624	
SGO_2062	-0.579	8.567	0.0028	0.0084	77.500	80.500	80.9683	122.9290	hisS; histidyl-tRNA synthetase
					71.000	63.500	71.0000	104.4141	
SGO_2064	0.430	8.872	0.0029	0.0089	138.000	67.000	144.1758	102.3136	ilvD; dihydroxy-acid dehydratase
					125.000	59.000	125.0000	97.0146	
SGO_2066	1.727	7.190	0.0158	0.0853	66.500	12.500	69.4760	19.0884	rpmG; ribosomal protein L33
					57.500		57.5000		
SGO_2070	0.507	5.531	0.0345	0.2179	15.000	8.500	15.6713	12.9801	hypothetical protein SGO_2070
					11.000	4.000	11.0000	6.5773	
SGO_2084	-0.269	4.253	0.0745	0.5363		3.500		5.3447	NAD(P)H dehydrogenase, quinone family
					5.500	5.000	5.5000	8.2216	
SGO_2085	0.571	7.367	0.0003	0.0003	48.000	22.500	50.1481	34.3590	purB; adenylosuccinate lyase
					48.500	19.500	48.5000	32.0642	
SGO_2098	0.377	10.921	0.0072	0.0303	519.500	313.000	542.7489	477.9724	rpsD; ribosomal protein S4
					548.500	224.500	548.5000	369.1489	
SGO_2100	3.516	8.239	0.0002	0.0001	140.500	9.000	146.7877	13.7436	ABC transporter substrate-binding protein
					131.000	6.500	131.0000	10.6881	
SGO_2104	0.230	7.893	0.0562	0.3877	55.000	44.500	57.4614	67.9545	srtB; sortase B
					69.500	26.000	69.5000	42.7522	
SGO_2105	-5.016	9.922	0.0000	0.0000	15.000	303.000	15.6713	462.7017	abpA; amylase-binding protein AbpA
					13.500	291.000	13.5000	478.4959	

Log$_2$ Ratios: -6 -4 -2 0 2 4 6

Show detected proteins only
Show all proteins
Filter by category:
ABC Transporter

Proteins found: 713

Test	Cutoff
q-Value	.005
p-Value	

Color	Signif	Direction	Applies To
red	yes	+	ratios, bars
yellow	no	n/a	bars
green	yes	−	ratios, bars
pink	yes	+	*p*- , *q*-Values
light green	yes	−	*p*- , *q*-Values

Dot Plots Dot Plots

Hendrickson *et al.*

Streptococcus gordonii

Protein	*SgPg* vs *SgFn* Log$_2$ Ratio	Log$_2$ Sum	*q*-Value	*p*-Value	Raw *SgPg*	Raw *SgFn*	Normalized *SgPg*	Normalized *SgFn*	Description
**SGO_2106**	-0.334	8.056	0.0167	0.0923	52.000	55.500	54.3271	84.7523	ribose-phosphate diphosphokinase
					63.000	39.000	63.0000	64.1283	
**SGO_2133**	-0.447	9.169	0.0060	0.0240	116.000	121.000	121.1913	184.7753	Cell division protein ftsH-like protein
					121.500	90.000	121.5000	147.9884	
**SGO_2134**	-1.405	8.099	0.0001	0.0000	37.500	63.500	39.1782	96.9688	hpt; hypoxanthine phosphoribosyltransferase
					36.000	62.000	36.0000	101.9476	
**SGO_2142**	1.004	7.762	0.0008	0.0011	73.500	22.000	76.7893	33.5955	GTP-binding protein
					68.000	23.500	68.0000	38.6414	
**SGO_2150**	-0.325	7.102	0.0081	0.0362	29.500	27.500	30.8202	41.9944	degP; serine protease
					30.000	21.000	30.0000	34.5306	

Log$_2$ Ratios: -6 -4 -2 0 2 4 6

Show detected proteins only
Show all proteins
Filter by category:
ABC Transporter

Proteins found: 713

Test	Cutoff
q-Value	.005
p-Value	

	Signif	Direction	Applies To
red	yes	+	ratios, bars
yellow	no	n/a	bars
green	yes	−	ratios, bars
pink	yes	+	*p*- , *q*-Values
light green	yes	−	*p*- , *q*-Values

Dot Plots Dot Plots
