# Supplementary material for: Proteomics of Streptococcus gordonii within a model developing oral microbial community
Source: BMC Microbiol. 2012 Sep 18;12:211. doi: 10.1186/1471-2180-12-211 (PMC3534352; doi:10.1186/1471-2180-12-211)
Supplement: Additional file 6 — SgPgFn_vs_SgFn. A more detailed presentation of the relative abundance ratios for the comparison of SgPgFn and SgFn, including both raw and normalized spectral counts. Red and green highlights are used as in Additional file 1. [file 1471-2180-12-211-S6.pdf]

Streptococcus gordonii

Hackett Laboratory UW

Protein	*SgPgFn vs SgFn* Log$_2$ Ratio	Log$_2$ Sum	*q*-Value	*p*-Value	Raw *SgPgFn*	Raw *SgFn*	Normalized *SgPgFn*	Normalized *SgFn*	Description
**SGO_0001**	-0.553	5.134	0.0078	0.0292	5.000	9.500	6.0383	9.5000	dnaA; chromosomal replication initiator protein DnaA
					6.000	10.500	8.2613	11.3062	
**SGO_0002**	-0.780	6.060	0.0016	0.0035	9.000	20.000	10.8689	20.0000	dnaN; DNA polymerase III, beta subunit
					10.000	20.500	13.7688	22.0740	
**SGO_0004**	-0.356	6.431	0.0274	0.1457	15.000	29.500	18.1149	29.5000	putative lipoprotein
					14.000	18.000	19.2764	19.3820	
**SGO_0006**	-0.058	6.587	0.1084	0.7696	22.500	28.000	27.1724	28.0000	ABC transporter, ATP-binding protein
					14.500	19.500	19.9648	20.9972	
**SGO_0007**	1.079	5.516	0.0015	0.0031	13.000	9.000	15.6996	9.0000	trpS; tryptophanyl-tRNA synthetase
					11.000	5.500	15.1457	5.9223	
**SGO_0008**	0.651	8.752	0.0013	0.0020	105.500	77.000	127.4082	77.0000	inosine-5'-monophosphate dehydrogenase
					98.500	84.500	135.6229	90.9879	
**SGO_0011**	0.038	3.580	0.1191	0.8484	4.500		5.4345		proteinase, M16 family
					2.000	3.500	2.7538	3.7687	
**SGO_0015**	0.033	2.445							ABC transporter (ATP-binding protein)
					2.000	2.500	2.7538	2.6919	
**SGO_0022**	-3.558	6.740	0.0001	0.0000	3.000	50.000	3.6230	50.0000	trmU; tRNA (5-methylaminomethyl-2-thiouridylate)-methyltransferase
					3.500	45.000	4.8191	48.4551	
**SGO_0025**	0.913	4.918	0.0230	0.1173	5.500	3.500	6.6421	3.5000	gidA; glucose inhibited division protein A
					9.500	6.500	13.0804	6.9991	
**SGO_0027**	0.900	6.144	0.0071	0.0259	15.500	11.000	18.7187	11.0000	rplI; ribosomal protein L9
					20.000	12.500	27.5376	13.4597	
**SGO_0028**	0.832	3.141	0.0235	0.1219		2.000		2.0000	dnaC; replicative DNA helicase
					3.000	2.500	4.1306	2.6919	

Log$_2$ Ratios: -6 -4 -2 0 2 4 6

Show detected proteins only
Show all proteins
Filter by category: ABC Transporter

Proteins found: 584

Test	Cutoff
***q*-Value**	.005
p-Value	

	Signif	Direction	Applies To
red	yes	+	ratios, bars
yellow	no	n/a	bars
green	yes	–	ratios, bars
pink	yes	+	*p*- , *q*-Values
light green	yes	–	*p*- , *q*-Values

Dot Plots Dot Plots

Protein	SgPgFn vs SgFn Log$_2$ Ratio	Log$_2$ Sum	*q*-Value	*p*-Value	Raw *SgPgFn*	Raw *SgFn*	Normalized *SgPgFn*	Normalized *SgFn*	Description
SGO_0030	-1.462	6.935	0.0001	0.0000	14.500	44.500	17.5111	44.5000	aspB; aspartate transaminase
					11.000	42.000	15.1457	45.2247	
SGO_0032	0.699	5.805	0.0031	0.0086	15.500	9.500	18.7187	9.5000	plsX; fatty acid/phospholipid synthesis protein PlsX
					11.500	11.000	15.8341	11.8446	
SGO_0042	-0.675	4.801	0.0136	0.0616	5.500	11.000	6.6421	11.0000	transcription regulator, GntR family
						9.500		10.2294	
SGO_0054	-2.242	7.201	0.0001	0.0000	11.000	59.000	13.2843	59.0000	dltA; D-alanine-activating enzyme
					9.000	58.000	12.3919	62.4532	
SGO_0057	-0.596	5.653	0.0078	0.0291	8.500	17.500	10.2651	17.5000	dltD protein
					7.000	12.000	9.6382	12.9214	
SGO_0059	4.484	11.789	0.0004	0.0002	1304.000	93.500	1574.7891	93.5000	pXO1; hypothetical protein SGO_0059
					1313.500	56.500	1808.5350	60.8380	
SGO_0063	0.350	5.597	0.0071	0.0258	11.000	9.500	13.2843	9.5000	hypothetical protein SGO_0063
					10.000	11.000	13.7688	11.8446	
SGO_0064	0.296	6.006	0.0101	0.0407	15.000	16.000	18.1149	16.0000	FtsK/SpoIIIE family protein
					12.500	12.000	17.2110	12.9214	
SGO_0065	0.118	6.285	0.0397	0.2305	17.000	17.000	20.5302	17.0000	hypothetical protein SGO_0065
					14.500	19.000	19.9648	20.4588	
SGO_0066	1.422	4.018	0.0352	0.2001	4.500	2.500	5.4345	2.5000	D-3-phosphoglycerate dehydrogenase, putative
					6.000		8.2613		
SGO_0067	1.156	4.815	0.0012	0.0019	7.500	5.000	9.0575	5.0000	protein with prophage function domain
					7.500	3.500	10.3266	3.7687	
SGO_0068	1.033	4.262	0.0063	0.0223	6.500		7.8498		lipoprotein, putative
					5.500	3.500	7.5729	3.7687	

Log$_2$ Ratios: -6 -4 -2 0 2 4 6

Show detected proteins only
Show all proteins
Filter by category:
ABC Transporter

Proteins found: 584

Test	Cutoff
***q*-Value**	**.005**
p-Value	

	Signif	Direction	Applies To
red	yes	+	ratios, bars
yellow	no	n/a	bars
green	yes	−	ratios, bars
pink	yes	+	***p*- , *q*-Values**
light green	yes	−	

Dot Plots Dot Plots

Protein	SgPgFn vs SgFn Log_2 Ratio	Log_2 Sum	*q*-Value	*p*-Value	Raw *SgPgFn*	Raw *SgFn*	Normalized *SgPgFn*	Normalized *SgFn*	Description
SGO_0080	0.548	5.745	0.0105	0.0445	13.000	13.500	15.6996	13.5000	hypothetical protein SGO_0080
					11.500	8.000	15.8341	8.6142	
SGO_0099	-0.792	3.575	0.0732	0.4882	2.000	2.500	2.4153	2.5000	pulA-2; pullulanase, type I
						6.500		6.9991	
SGO_0104	-0.282	8.448	0.0219	0.1109	60.000	108.000	72.4596	108.0000	Maltose/maltodextrin-binding protein precursor
					61.500	78.000	84.6783	83.9888	
SGO_0105	-1.140	5.228	0.0045	0.0140	4.000	15.000	4.8306	15.0000	malQ; 4-alpha-glucanotransferase
					5.000	10.000	6.8844	10.7678	
SGO_0106	-0.925	7.869	0.0018	0.0039	33.500	84.500	40.4566	84.5000	glgP-2; maltodextrin phosphorylase
					29.000	64.000	39.9296	68.9139	
SGO_0113	0.441	10.731	0.0011	0.0015	416.500	375.000	502.9905	375.0000	acdH; alcohol-acetaldehyde dehydrogenase
					345.500	321.500	475.7129	346.1846	
SGO_0135	-1.269	6.698	0.0026	0.0068	10.500	41.500	12.6804	41.5000	v-type sodium ATP synthase, subunit A
					13.000	29.500	17.8995	31.7650	
SGO_0136	-0.904	6.696	0.0022	0.0052	12.500	35.500	15.0958	35.5000	v-type sodium ATP synthase, chain B
					15.500	29.500	21.3417	31.7650	
SGO_0139	-1.017	6.088	0.0023	0.0056	11.500	21.500	13.8881	21.5000	thrC; threonine synthase
					6.500	22.000	8.9497	23.6891	
SGO_0145	-1.124	6.669	0.0048	0.0153	12.000	41.500	14.4919	41.5000	polI; DNA polymerase I
					12.500	26.500	17.2110	28.5347	
SGO_0146	-0.812	5.412	0.0355	0.2024	10.500	15.000	12.6804	15.0000	CoA-binding domain protein
					3.000	10.000	4.1306	10.7678	
SGO_0152	-0.818	4.490	0.0268	0.1420	3.500	10.500	4.2268	10.5000	tgt; queuine tRNA-ribosyltransferase
					2.500	4.000	3.4422	4.3071	

Log_2 Ratios: -6 -4 -2 0 2 4 6

Show detected proteins only
Show all proteins
Filter by category: ABC Transporter

Proteins found: 584

Test	Cutoff
q-Value	.005
p-Value	

	Signif	Direction	Applies To
(red)	yes	+	ratios, bars
(yellow)	no	n/a	bars
(green)	yes	−	ratios, bars
(pink)	yes	+	*p*- , *q*-Values
(light green)	yes	−	*p*- , *q*-Values

Dot Plots Dot Plots

Protein	SgPgFn vs SgFn Log_2 Ratio	Log_2 Sum	*q*-Value	*p*-Value	Raw *SgPgFn*	Raw *SgFn*	Normalized *SgPgFn*	Normalized *SgFn*	Description
SGO_0154	-0.961	10.418	0.0014	0.0025	193.000	493.000	233.0784	493.0000	pgi; glucose-6-phosphate isomerase
					167.000	382.500	229.9394	411.8681	
SGO_0158	-0.969	7.832	0.0020	0.0047	37.500	70.000	45.2873	70.0000	2,3,4,5-tetrahydropyridine-2-carboxylate N-succinyltransferase, putative
					23.500	74.500	32.3567	80.2201	
SGO_0163	-0.555	6.398	0.0013	0.0022	14.000	26.500	16.9072	26.5000	galU; UTP-glucose-1-phosphate uridylyltransferase
					12.500	22.000	17.2110	23.6891	
SGO_0164	-0.821	6.303	0.0038	0.0111	11.000	22.000	13.2843	22.0000	Glycerol-3-phosphate dehydrogenase [NAD (P)+] (NAD(P)H-dependent glycerol-3-phosphate dehydrogenase)
					11.000	26.500	15.1457	28.5347	
SGO_0169	-1.283	5.622	0.0033	0.0094	5.500	20.500	6.6421	20.5000	dut; dUTP diphosphatase
					5.500	13.500	7.5729	14.5365	
SGO_0171	-0.576	2.911			2.500	4.500	3.0192	4.5000	radA; DNA repair protein RadA
SGO_0174	-0.073	7.627	0.0875	0.6035	37.000	60.000	44.6834	60.0000	gltX; glutamyl-tRNA synthetase
					37.000	39.000	50.9446	41.9944	
SGO_0180	-0.177	4.949	0.1287	0.9284	3.500	6.500	4.2268	6.5000	jag; hypothetical protein SGO_0180
					8.000	8.500	11.0151	9.1526	
SGO_0197	-0.253	3.809	0.0515	0.3175		4.500		4.5000	predicted ribosome small subunit-dependent GTPase A
					3.000	5.000	4.1306	5.3839	
SGO_0198	-2.236	7.405	0.0002	0.0001	11.000	67.000	13.2843	67.0000	rpe; ribulose-phosphate 3-epimerase
					12.000	67.500	16.5226	72.6826	
SGO_0200	-0.337	5.633	0.0212	0.1059	7.500	13.500	9.0575	13.5000	competence-induced protein Ccs50
					9.500	13.000	13.0804	13.9981	
SGO_0201	-2.387	6.927	0.0016	0.0032	7.000	43.000	8.4536	43.0000	cmp-binding-factor 1
					8.000	55.000	11.0151	59.2229	

Log_2 Ratios: -6 -4 -2 0 2 4 6

- Show detected proteins only
- Show all proteins
- Filter by category: ABC Transporter

Proteins found: 584

Test	Cutoff
***q*-Value**	.005
p-Value	

Color	Signif	Direction	Applies To
red	yes	+	ratios, bars
yellow	no	n/a	bars
green	yes	−	ratios, bars
pink	yes	+	*p*- , *q*-Values
light green	yes	−	*p*- , *q*-Values

Dot Plots Dot Plots

Hendrickson *et al.*

Streptococcus gordonii

Hackett Laboratory UW

Protein	*SgPgFn vs SgFn* Log$_2$ Ratio	Log$_2$ Sum	*q*-Value	*p*-Value	Raw *SgPgFn*	Raw *SgFn*	Normalized *SgPgFn*	Normalized *SgFn*	Description
SGO_0204	0.016	6.952	0.1239	0.8855	25.000	39.500	30.1915	39.5000	rpsL; ribosomal protein S12
					22.500	21.500	30.9799	23.1508	
SGO_0205	0.417	8.530	0.0214	0.1072	75.000	92.500	90.5745	92.5000	rpsG; ribosomal protein S7
					87.500	61.500	120.4772	66.2219	
SGO_0206	0.564	11.617	0.0024	0.0061	837.000	652.000	1010.8117	652.0000	fusA; translation elongation factor G
					627.500	569.500	863.9937	613.2259	
SGO_0207	0.257	12.020	0.0183	0.0872	830.000	934.000	1002.3581	934.0000	gap; glyceraldehyde-3-phosphate dehydrogenase, type I
					919.500	884.000	1266.0433	951.8731	
SGO_0209	0.252	12.189	0.0031	0.0085	1021.000	1110.000	1233.0212	1110.0000	pgk; phosphoglycerate kinase
					948.000	949.000	1305.2845	1021.8637	
SGO_0215	-0.579	7.445	0.0059	0.0205	34.500	51.500	41.6643	51.5000	glnA; glutamine synthetase, type I
					21.000	48.500	28.9145	52.2238	
SGO_0219	-0.016	6.905	0.1133	0.8056	26.000	29.000	31.3992	29.0000	metallo-beta-lactamase superfamily protein 1
					20.500	29.000	28.2261	31.2266	
SGO_0230	1.048	4.788	0.0233	0.1196	5.000	6.500	6.0383	6.5000	Protein of unknown function, DUF536 family
					9.000	2.500	12.3919	2.6919	
SGO_0234	-0.964	6.469	0.0008	0.0008	13.500	30.500	16.3034	30.5000	pepX; X-Pro dipeptidyl-peptidase
					10.000	26.000	13.7688	27.9963	
SGO_0237	-0.611	4.436	0.0348	0.1970	2.500	4.500	3.0192	4.5000	ccpA; CcpA protein (proteinase)
					4.000	8.000	5.5075	8.6142	
SGO_0243	-0.226	4.377	0.0612	0.3979		6.500		6.5000	hydroxymethylglutaryl-CoA reductase, degradative
					4.500	7.500	6.1960	8.0758	
SGO_0244	-0.170	4.570	0.0834	0.5695	6.000	9.500	7.2460	9.5000	hydroxymethylglutaryl-CoA synthase
						6.500		6.9991	

Log$_2$ Ratios: -6 -4 -2 0 2 4 6

Show detected proteins only
Show all proteins
Filter by category:
ABC Transporter

Proteins found: 584

Test	Cutoff
q-Value	.005
p-Value	

Color	Signif	Direction	Applies To
red	yes	+	ratios, bars
yellow	no	n/a	bars
green	yes	−	ratios, bars
pink	yes	+	*p*- , *q*-Values
light green	yes	−	*p*- , *q*-Values

Dot Plots Dot Plots

Hendrickson *et al.*

Protein	*SgPgFn vs SgFn* Log_2 Ratio	Log_2 Sum	*q*-Value	*p*-Value	Raw *SgPgFn*	Raw *SgFn*	Normalized *SgPgFn*	Normalized *SgFn*	Description
**SGO_0247**	-0.755	9.779	0.0088	0.0343	137.000	223.500	165.4495	223.5000	pfl; formate acetyltransferase
					114.500	308.500	157.6530	332.1865	
**SGO_0252**	-0.593	3.619	0.0510	0.3132	2.500	5.500	3.0192	5.5000	possible TetR-type transcriptional regulator
						3.500		3.7687	
**SGO_0255**	-0.236	5.062	0.0282	0.1521	7.000	10.000	8.4536	10.0000	Signal peptidase I
					5.000	7.500	6.8844	8.0758	
**SGO_0258**	0.355	2.880							hypothetical protein SGO_0258
					3.000	3.000	4.1306	3.2303	
**SGO_0260**	-0.677	5.083	0.0123	0.0543	4.500	8.500	5.4345	8.5000	DNA mismatch binding protein MutS2
					5.500	11.500	7.5729	12.3830	
**SGO_0262**	-1.445	5.499	0.0010	0.0014	5.500	18.000	6.6421	18.0000	dipeptidase
					4.000	14.000	5.5075	15.0749	
**SGO_0263**	1.121	6.893	0.0087	0.0340	41.000	14.000	49.5141	14.0000	trx-1; thioredoxin
					23.000	22.000	31.6683	23.6891	
**SGO_0272**	0.001	5.530	0.1304	0.9477	10.500	13.500	12.6804	13.5000	hypothetical protein SGO_0272
					7.500	9.000	10.3266	9.6910	
**SGO_0276**	-0.258	8.496	0.0206	0.1025	62.500	108.500	75.4788	108.5000	gdhA; glutamate dehydrogenase (NADP)
					64.500	82.000	88.8089	88.2959	
**SGO_0286**	-0.453	2.705							DNA mismatch repair protein MutS, putative
					2.000	3.500	2.7538	3.7687	
**SGO_0291**	-0.587	5.151	0.0046	0.0146	5.000	11.000	6.0383	11.0000	copper-translocating P-type ATPase
					6.000	9.500	8.2613	10.2294	
**SGO_0292**	2.555	7.814	0.0003	0.0001	84.000	15.500	101.4435	15.5000	spxB; pyruvate oxidase
					66.000	16.000	90.8742	17.2285	

Log_2 Ratios: -6 -4 -2 0 2 4 6

Show detected proteins only
Show all proteins
Filter by category:
ABC Transporter

Proteins found: 584

Test	Cutoff
***q*-Value**	**.005**
p-Value	

	Signif	Direction	Applies To
red	yes	+	ratios, bars
yellow	no	n/a	bars
green	yes	−	ratios, bars
light red	yes	+	*p*- , *q*-Values
light green	yes	−	*p*- , *q*-Values

Dot Plots Dot Plots

Hendrickson *et al.*

Protein	SgPgFn vs SgFn Log_2 Ratio	Log_2 Sum	*q*-Value	*p*-Value	Raw *SgPgFn*	Raw *SgFn*	Normalized *SgPgFn*	Normalized *SgFn*	Description
SGO_0312	-0.222	10.630	0.0039	0.0115	309.000	444.000	373.1670	444.0000	xfp; D-xylulose 5-phosphate/D-fructose 6-phosphate phosphoketolase
					260.000	380.000	357.9894	409.1762	
SGO_0321	-0.983	6.071	0.0015	0.0030	8.000	23.500	9.6613	23.5000	polypeptide deformylase
					9.500	19.500	13.0804	20.9972	
SGO_0333	0.362	7.225	0.0310	0.1718	30.000	39.500	36.2298	39.5000	rpsO; ribosomal protein S15
					34.500	24.500	47.5024	26.3811	
SGO_0342	-0.909	7.359	0.0009	0.0010	25.000	56.500	30.1915	56.5000	pepF-2; oligoendopeptidase
					19.500	47.000	26.8492	50.6086	
SGO_0344	-0.313	8.048	0.0061	0.0214	48.000	79.000	57.9677	79.0000	pnpA; polyribonucleotide nucleotidyltransferase
					43.500	63.000	59.8944	67.8371	
SGO_0349	-0.611	6.029	0.0125	0.0552	11.500	16.000	13.8881	16.0000	cysS; cysteinyl-tRNA synthetase
					8.500	22.000	11.7035	23.6891	
SGO_0352	-0.438	6.480	0.0007	0.0007	16.000	25.000	19.3226	25.0000	ABC transporter, ATP-binding protein SP1580
					13.500	24.500	18.5879	26.3811	
SGO_0357	0.214	6.658	0.0091	0.0360	21.000	23.000	25.3609	23.0000	degV; DegV family fatty acid binding protein
					21.000	22.000	28.9145	23.6891	
SGO_0358	-0.100	8.576	0.0520	0.3226	73.000	108.500	88.1592	108.5000	rplM; ribosomal protein L13
					69.500	83.000	95.6933	89.3727	
SGO_0359	0.671	7.145	0.0101	0.0418	32.500	34.500	39.2490	34.5000	rpsI; ribosomal protein S9
					34.000	19.500	46.8140	20.9972	
SGO_0368	-0.659	3.359	0.0554	0.3497	2.000	3.000	2.4153	3.0000	merA; mercury(II) reductase
						4.500		4.8455	
SGO_0372	-0.919	5.539	0.0025	0.0064	7.000	17.000	8.4536	17.0000	malate oxidoreductase
					5.500	12.500	7.5729	13.4597	

Log_2 Ratios: -6 -4 -2 0 2 4 6

Show detected proteins only
Show all proteins
Filter by category:
ABC Transporter

Proteins found: 584

Test	Cutoff
q-Value	.005
p-Value	

	Signif	Direction	Applies To
red	yes	+	ratios, bars
yellow	no	n/a	bars
green	yes	−	ratios, bars
pink	yes	+	*p*- , *q*-Values
light green	yes	−	*p*- , *q*-Values

Dot Plots Dot Plots

Hendrickson *et al.*

Protein	SgPgFn vs SgFn Log$_2$ Ratio	Log$_2$ Sum	*q*-Value	*p*-Value	Raw SgPgFn	Raw SgFn	Normalized SgPgFn	Normalized SgFn	Description
SGO_0374	-1.862	5.419	0.0013	0.0022	5.000	15.000	6.0383	15.0000	Response regulator of the LytR/AlgR family
					2.500	17.000	3.4422	18.3053	
SGO_0384	0.048	4.532	0.1293	0.9361	4.500	4.500	5.4345	4.5000	putative carboxylate-amine/thiol ligase
					4.500	6.500	6.1960	6.9991	
SGO_0390	-0.742	7.537	0.0050	0.0165	27.000	66.500	32.6068	66.5000	glycerol-3-phosphate dehydrogenase (NAD (P)+)
					26.500	46.500	36.4874	50.0702	
SGO_0392	-1.550	5.511	0.0113	0.0485	5.500	18.500	6.6421	18.5000	phosphoglycerate mutase
						19.000		20.4588	
SGO_0398	-0.848	4.338	0.0119	0.0517	2.500	5.000	3.0192	5.0000	ABC transporter ATP-binding protein
					3.000	7.500	4.1306	8.0758	
SGO_0401	0.148	6.218	0.0233	0.1210	17.000	16.500	20.5302	16.5000	grpE; co-chaperone GrpE
					13.500	17.500	18.5879	18.8436	
SGO_0402	-0.715	10.656	0.0003	0.0001	245.000	492.500	295.8768	492.5000	dnaK; DnaK chaperone protein
					229.000	474.000	315.3061	510.3935	
SGO_0404	-0.451	5.300	0.0313	0.1735	5.000	10.500	6.0383	10.5000	dnaJ; DnaJ chaparone protein
					8.000	11.000	11.0151	11.8446	
SGO_0411	-4.144	7.766	0.0451	0.2683	4.500	61.000	5.4345	61.0000	conserved hypothetical protein TIGR01440
						140.500		151.2875	
SGO_0412	0.932	10.208	0.0004	0.0003	328.500	216.500	396.7164	216.5000	tig; trigger factor
					275.000	177.000	378.6426	190.5900	
SGO_0413	-0.761	6.382	0.0007	0.0007	12.500	25.000	15.0958	25.0000	DNA-directed RNA polymerase delta chain
					11.500	25.500	15.8341	27.4579	
SGO_0415	-0.050	8.850	0.0760	0.5089	89.500	125.500	108.0856	125.5000	secA; preprotein translocase, SecA subunit
					86.000	101.500	118.4119	109.2931	

Show detected proteins only
Show all proteins
Filter by category:
ABC Transporter

Proteins found: 584

Test	Cutoff
***q*-Value**	.005
p-Value	

	Signif	Direction	Applies To
	yes	+	ratios, bars
	no	n/a	bars
	yes	−	ratios, bars
	yes	+	*p*- , *q*-Values
	yes	−	

Dot Plots Dot Plots

Hendrickson *et al.*

Protein	SgPgFn vs SgFn Log2 Ratio	Log2 Sum	*q*-Value	*p*-Value	Raw SgPgFn	Raw SgFn	Normalized SgPgFn	Normalized SgFn	Description
SGO_0416	-1.666	6.527	0.0009	0.0010	12.000	35.000	14.4919	35.0000	phospho-2-dehydro-3-deoxyheptonate aldolase
					6.000	32.000	8.2613	34.4569	
SGO_0426	-1.096	4.682	0.0291	0.1586	4.000	9.000	4.8306	9.0000	Cof family protein
						11.000		11.8446	
SGO_0427	-1.766	6.750	0.0009	0.0012	10.500	37.500	12.6804	37.5000	universal stress protein family
					8.500	42.500	11.7035	45.7631	
SGO_0429	-0.347	7.952	0.0016	0.0034	43.500	67.000	52.5332	67.0000	aspartate transaminase
					41.000	66.500	56.4522	71.6058	
SGO_0432	1.572	5.226	0.0121	0.0526	14.000		16.9072		entB; isochorismatase family protein
					11.000	5.000	15.1457	5.3839	
SGO_0434	-0.338	5.963	0.0483	0.2909	13.500	13.000	16.3034	13.0000	aspS-2; aspartyl-tRNA synthetase
					8.000	20.500	11.0151	22.0740	
SGO_0435	0.120	5.204	0.0298	0.1626	8.500	8.500	10.2651	8.5000	gatC; glutamyl-tRNA(Gln) amidotransferase, C subunit
					6.500	8.500	8.9497	9.1526	
SGO_0436	0.478	7.708	0.0009	0.0010	50.000	46.000	60.3830	46.0000	gatA; glutamyl-tRNA(Gln) amidotransferase, A subunit
					44.500	38.500	61.2713	41.4560	
SGO_0437	0.393	7.762	0.0355	0.2029	65.000	52.000	78.4979	52.0000	gatB; glutamyl-tRNA(Gln) amidotransferase, B subunit
					33.500	37.500	46.1256	40.3792	
SGO_0440	-2.663	6.048	0.0122	0.0534	4.000	28.500	4.8306	28.5000	L-iditol 2-dehydrogenase BH3949
						30.500		32.8418	
SGO_0445	-1.379	5.916	0.0006	0.0005	6.000	22.500	7.2460	22.5000	GTP-binding protein
					7.000	19.500	9.6382	20.9972	
SGO_0448	-2.532	4.521	0.0206	0.1025	1.500	12.000	1.8115	12.0000	conserved hypothetical protein TIGR00488
						8.500		9.1526	

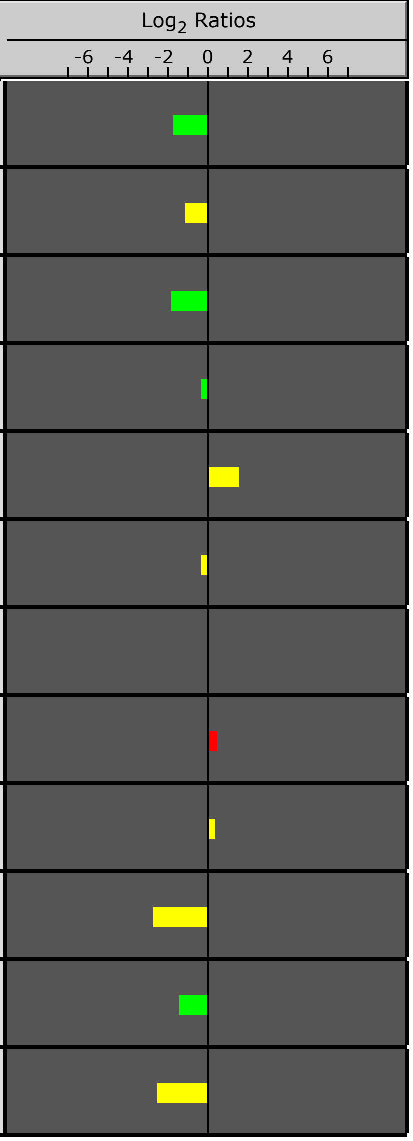

Show detected proteins only
Show all proteins
Filter by category:
ABC Transporter

Proteins found: 584

Test	Cutoff
q-Value	.005
p-Value	

	Signif	Direction	Applies To
red	yes	+	ratios, bars
yellow	no	n/a	bars
green	yes	−	ratios, bars
pink	yes	+	*p*- , *q*-Values
light green	yes	−	*p*- , *q*-Values

Dot Plots Dot Plots

Hendrickson *et al.*

Protein	*SgPgFn* vs *SgFn* Log$_2$ Ratio	Log$_2$ Sum	*q*-Value	*p*-Value	Raw *SgPgFn*	Raw *SgFn*	Normalized *SgPgFn*	Normalized *SgFn*	Description
SGO_0454	-0.429	6.514	0.0563	0.3573	10.500	21.000	12.6804	21.0000	conserved hypothetical protein TIGR01033
					20.000	28.000	27.5376	30.1498	
SGO_0455	1.915	3.733	0.0398	0.2313	3.500	1.500	4.2268	1.5000	lipoprotein, putative
					5.500		7.5729		
SGO_0457	1.458	6.659	0.0001	0.0000	30.000	13.500	36.2298	13.5000	ABC transporter, substrate-binding protein SP0148
					27.500	12.500	37.8643	13.4597	
SGO_0458	0.958	8.142	0.0015	0.0029	74.500	56.500	89.9707	56.5000	hlpA; lipoprotein
					69.500	37.500	95.6933	40.3792	
SGO_0460	0.308	4.801	0.0014	0.0024	6.500	6.000	7.8498	6.0000	ABC transporter, ATP-binding protein SP0151
					5.500	6.000	7.5729	6.4607	
SGO_0468	-1.669	6.013	0.0058	0.0201	7.500	31.000	9.0575	31.0000	hypothetical protein SGO_0468
					4.500	17.000	6.1960	18.3053	
SGO_0476	2.617	4.838	0.0082	0.0313	10.500		12.6804		rhodanese family protein
					10.000	2.000	13.7688	2.1536	
SGO_0480	-5.955	8.849	0.0388	0.2230		146.500		146.5000	hypothetical protein SGO_0480
					2.500	289.000	3.4422	311.1893	
SGO_0483	0.313	5.573	0.0011	0.0015	11.000	11.000	13.2843	11.0000	hypothetical protein SGO_0483
					9.500	9.500	13.0804	10.2294	
SGO_0497	-6.245	8.111	0.0022	0.0054	1.500	138.500	1.8115	138.5000	gtfG; glucosyltransferase G
						126.500		136.2126	
SGO_0501	2.672	5.473	0.0013	0.0023	17.000		20.5302		Uncharacterized ACR, COG1399
					15.000	3.000	20.6532	3.2303	
SGO_0502	0.856	7.662	0.0028	0.0076	52.500	44.000	63.4022	44.0000	floL; flotillin-like protein
					48.000	27.000	66.0904	29.0730	

Log$_2$ Ratios: -6 -4 -2 0 2 4 6

Show detected proteins only
Show all proteins
Filter by category:
ABC Transporter

Proteins found: 584

Test	Cutoff
q-Value	.005
p-Value	

	Signif	Direction	Applies To
red	yes	+	ratios, bars
yellow	no	n/a	bars
green	yes	−	ratios, bars
pink	yes	+	*p*- , *q*-Values
light green	yes	−	*p*- , *q*-Values

Dot Plots Dot Plots

Hendrickson *et al.*

Protein	*SgPgFn vs SgFn* Log$_2$ Ratio	Log$_2$ Sum	*q*-Value	*p*-Value	Raw *SgPgFn*	Raw *SgFn*	Normalized *SgPgFn*	Normalized *SgFn*	Description	Log$_2$ Ratios (-6 -4 -2 0 2 4 6)
SGO_0503	-0.504	10.811	0.0012	0.0018	322.000	545.500	388.8666	545.5000	gnd; 6-phosphogluconate dehydrogenase, decarboxylating	
					257.500	472.000	354.5472	508.2399		
SGO_0505	-0.169	7.069	0.0565	0.3604	24.000	42.500	28.9838	42.5000	PTS system, IIBC component	
					24.500	27.000	33.7336	29.0730		
SGO_0508	0.116	4.756	0.0856	0.5856	8.500	8.500	10.2651	8.5000	nrdR; transcriptional regulator, NrdR family	
					6.000		8.2613			
SGO_0510	-0.169	5.157	0.0612	0.3971	7.500	11.500	9.0575	11.5000	dnaI; primosomal protein DnaI	
					5.500	7.000	7.5729	7.5375		
SGO_0511	-1.090	4.703	0.0072	0.0263	4.000	7.000	4.8306	7.0000	NADPH-flavin oxidoreductase -like protein	
					2.500	10.000	3.4422	10.7678		
SGO_0512	0.571	7.105	0.0060	0.0210	38.000	30.000	45.8911	30.0000	GTP-binding protein engA	
					26.500	23.500	36.4874	25.3043		
SGO_0515	-0.122	7.853	0.0635	0.4155	40.500	66.000	48.9102	66.0000	murC; UDP-N-acetylmuramate--alanine ligase	
					45.000	50.500	61.9597	54.3774		
SGO_0518	1.104	6.297	0.0015	0.0027	20.000	12.500	24.1532	12.5000	aminodeoxychorismate lyase-like protein	
					21.500	11.500	29.6030	12.3830		
SGO_0519	0.127	6.121	0.0722	0.4804	14.500	19.500	17.5111	19.5000	greA; transcription elongation factor greA	
					13.500	13.000	18.5879	13.9981		
SGO_0526	-1.156	4.798	0.0101	0.0413	3.000	7.000	3.6230	7.0000	ilvB; acetolactate synthase, large subunit, biosynthetic type	
					3.500	11.500	4.8191	12.3830		
SGO_0527	-1.592	3.861	0.0089	0.0352		6.000		6.0000	ilvN; acetolactate synthase, small subunit	
					1.500	6.000	2.0653	6.4607		
SGO_0528	-0.734	7.797	0.0048	0.0157	39.000	77.500	47.0988	77.5000	ilvC; ketol-acid reductoisomerase	
					26.500	57.000	36.4874	61.3764		

Show detected proteins only
Show all proteins
Filter by category:
ABC Transporter

Proteins found: 584

Test	Cutoff
q-Value	.005
p-Value	

Color	Signif	Direction	Applies To
red	yes	+	ratios, bars
yellow	no	n/a	bars
green	yes	−	ratios, bars
pink	yes	+	*p*- , *q*-Values
light green	yes	−	*p*- , *q*-Values

Dot Plots Dot Plots

Streptococcus gordonii

Hackett Laboratory UW

Protein	SgPgFn vs SgFn				Raw		Normalized		Description
	Log_2 Ratio	Log_2 Sum	*q*-Value	*p*-Value	*SgPgFn*	*SgFn*	*SgPgFn*	*SgFn*	
SGO_0529	-0.759	3.952	0.0542	0.3391		4.500		4.5000	ilvA; threonine dehydratase
					2.500	7.000	3.4422	7.5375	
SGO_0535	-0.143	2.930			3.000	4.000	3.6230	4.0000	putative transcriptional regulator LytR
SGO_0537	-0.564	5.678	0.0016	0.0033	8.000	16.000	9.6613	16.0000	HIT family protein
					8.000	13.500	11.0151	14.5365	
SGO_0540	-1.234	6.659	0.0004	0.0002	13.000	37.000	15.6996	37.0000	hypothetical protein SGO_0540
					10.500	31.500	14.4573	33.9186	
SGO_0543	0.752	6.151	0.0016	0.0032	17.000	13.500	20.5302	13.5000	nusA; transcription termination factor NusA
					17.500	12.000	24.0954	12.9214	
SGO_0546	-0.168	7.401	0.0612	0.3987	27.500	40.500	33.2107	40.5000	infB; Translation initiation factor IF-2
					34.000	45.000	46.8140	48.4551	
SGO_0548	0.714	3.205	0.0101	0.0417	3.000		3.6230		Na/Pi-cotransporter family protein
					2.500	2.000	3.4422	2.1536	
SGO_0552	-0.957	6.345	0.0017	0.0037	12.000	29.500	14.4919	29.5000	oxidoreductase, aldo/keto reductase family
					9.500	22.500	13.0804	24.2275	
SGO_0554	-1.725	5.015	0.0204	0.1000	3.500	12.500	4.2268	12.5000	hsdR; type I site-specific deoxyribonuclease
						14.500		15.6133	
SGO_0558	-0.230	2.581							hypothetical protein SGO_0558
					2.000	3.000	2.7538	3.2303	
SGO_0560	1.553	3.483	0.0247	0.1292	4.500		5.4345		hsdM; type I restriction-modification system, M subunit
					3.000	1.500	4.1306	1.6152	
SGO_0565	-2.000	8.171	0.0010	0.0013	23.000	128.000	27.7762	128.0000	adhA; alcohol dehydrogenase
					21.500	95.500	29.6030	102.8324	

Log_2 Ratios: -6 -4 -2 0 2 4 6

Show detected proteins only
Show all proteins
Filter by category:
ABC Transporter

Proteins found: 584

Test	Cutoff
***q*-Value**	.005
p-Value	

	Signif	Direction	Applies To
(red)	yes	+	ratios, bars
(yellow)	no	n/a	bars
(green)	yes	−	ratios, bars
(pink)	yes	+	*p*- , *q*-Values
(light green)	yes	−	*p*- , *q*-Values

Dot Plots Dot Plots

Hendrickson *et al.*

Streptococcus gordonii

Hackett Laboratory UW

Protein	SgPgFn vs SgFn				Raw		Normalized		Description
	Log_2 Ratio	Log_2 Sum	*q*-Value	*p*-Value	*SgPgFn*	*SgFn*	*SgPgFn*	*SgFn*	
SGO_0568	1.101	5.369	0.0073	0.0268	9.500	8.500	11.4728	8.5000	glyQ; glycyl-tRNA synthetase, alpha subunit
					12.000	4.500	16.5226	4.8455	
SGO_0569	-0.089	7.432	0.1037	0.7297	28.000	38.000	33.8145	38.0000	glyS; glycyl-tRNA synthetase, beta subunit
					36.500	47.000	50.2562	50.6086	
SGO_0573	-1.254	5.136	0.0101	0.0414	3.000	15.500	3.6230	15.5000	mraW; S-adenosyl-methyltransferase MraW
					5.000	8.500	6.8844	9.1526	
SGO_0577	0.202	5.854	0.0868	0.5957	10.000	17.500	12.0766	17.5000	ATP-dependent RNA helicase
					13.500	9.000	18.5879	9.6910	
SGO_0581	0.265	4.348	0.0563	0.3569	4.500	3.500	5.4345	3.5000	trxB; thioredoxin-disulfide reductase
					4.000	5.500	5.5075	5.9223	
SGO_0582	-2.174	7.897	0.0002	0.0001	21.500	96.000	25.9647	96.0000	nicotinate phosphoribosyltransferase, putative
					13.000	91.500	17.8995	98.5253	
SGO_0583	-1.580	5.703	0.0027	0.0071	5.000	23.000	6.0383	23.0000	nadE; NAD+ synthetase
					5.000	15.000	6.8844	16.1517	
SGO_0585	-2.349	7.628	0.0004	0.0002	15.000	77.500	18.1149	77.5000	pepC; aminopeptidase C
					10.500	81.500	14.4573	87.7575	
SGO_0586	-0.188	6.424	0.0303	0.1667	15.500	20.500	18.7187	20.5000	pbp1a; penicillin-binding protein 1A
					15.500	23.500	21.3417	25.3043	
SGO_0589	-0.835	6.970	0.0101	0.0417	21.000	49.000	25.3609	49.0000	methylase
					14.000	29.500	19.2764	31.7650	
SGO_0590	-1.470	4.571	0.0188	0.0905	3.000	11.000	3.6230	11.0000	Methyltransferase
						8.500		9.1526	
SGO_0591	-0.626	5.925	0.0013	0.0021	9.000	18.500	10.8689	18.5000	hypothetical protein SGO_0591
					9.500	17.000	13.0804	18.3053	

Log_2 Ratios

-6 -4 -2 0 2 4 6

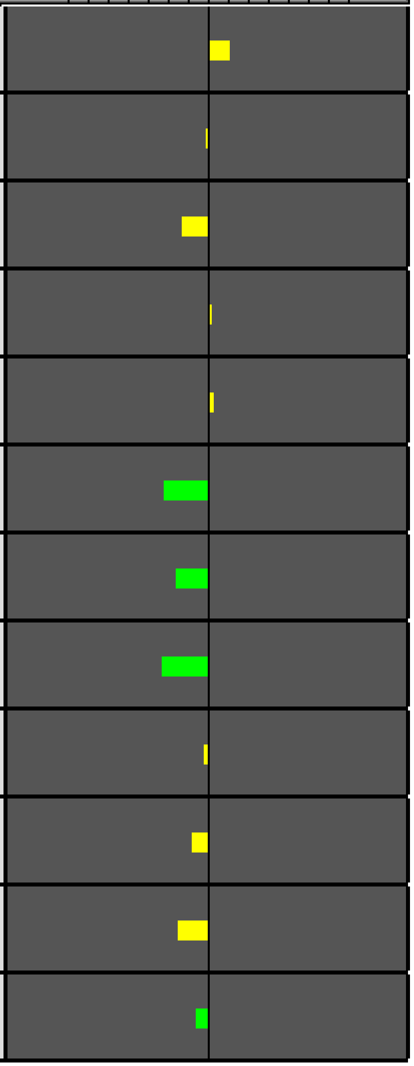

Show detected proteins only
Show all proteins
Filter by category:
ABC Transporter

Proteins found: 584

Test	Cutoff
q-Value	.005
p-Value	

	Signif	Direction	Applies To
(red)	yes	+	ratios, bars
(yellow)	no	n/a	bars
(green)	yes	−	ratios, bars
(pink)	yes	+	*p*- , *q*-Values
(light green)	yes	−	

Dot Plots Dot Plots

Hendrickson *et al.*

Protein	SgPgFn vs SgFn: Log_2 Ratio	Log_2 Sum	*q*-Value	*p*-Value	Raw: *SgPgFn*	Raw: *SgFn*	Normalized: *SgPgFn*	Normalized: *SgFn*	Description	Log_2 Ratios (-6 -4 -2 0 2 4 6)
**SGO_0592**	-0.527	4.232	0.0281	0.1510	4.000	7.500	4.8306	7.5000	luxS; autoinducer-2 production protein LuxS	
						6.000		6.4607		
**SGO_0593**	0.245	5.513	0.0449	0.2657	12.000	9.000	14.4919	9.0000	HD/KH domain protein	
					7.500	11.000	10.3266	11.8446		
**SGO_0594**	-0.918	5.361	0.0014	0.0025	5.500	14.500	6.6421	14.5000	gmk; Guanylate kinase (GMP kinase)	
					5.500	11.500	7.5729	12.3830		
**SGO_0595**	0.632	5.291	0.0054	0.0183	10.500	9.000	12.6804	9.0000	DNA-directed RNA polymerase, omega subunit	
					8.000	6.000	11.0151	6.4607		
**SGO_0597**	-1.613	5.275	0.0013	0.0022	4.500	13.000	5.4345	13.0000	fmt; methionyl-tRNA formyltransferase	
					3.000	15.000	4.1306	16.1517		
**SGO_0599**	-1.901	6.606	0.0006	0.0005	7.000	40.500	8.4536	40.5000	phosphoprotein phosphatase	
					9.000	33.500	12.3919	36.0721		
**SGO_0600**	-1.139	4.891	0.0315	0.1751	4.500	14.000	5.4345	14.0000	serine/threonine protein kinase	
						9.500		10.2294		
**SGO_0604**	-1.487	7.899	0.0001	0.0000	27.500	87.000	33.2107	87.0000	hydrolase, haloacid dehalogenase family/peptidyl-prolyl cis-trans isomerase, cyclophilin type	
					21.500	82.500	29.6030	88.8343		
**SGO_0606**	0.171	7.806	0.0019	0.0043	48.000	52.000	57.9677	52.0000	cysK; cysteine synthase A	
					44.000	49.500	60.5828	53.3006		
**SGO_0610**	-0.637	7.943	0.0136	0.0613	50.000	84.000	60.3830	84.0000	ribosomal subunit interface protein	
					27.000	60.000	37.1758	64.6068		
**SGO_0626**	0.009	2.590			2.500	3.000	3.0192	3.0000	recX; Regulatory protein recX	
**SGO_0631**	1.630	4.171	0.0136	0.0613	6.000	2.500	7.2460	2.5000	alpha-glycerophosphate oxidase	
					6.000		8.2613			

Show detected proteins only
Show all proteins
Filter by category:
ABC Transporter

Proteins found: 584

Test	Cutoff
***q*-Value**	**.005**
p-Value	

	Signif	Direction	Applies To
red	yes	+	ratios, bars
yellow	no	n/a	bars
green	yes	−	ratios, bars
pink	yes	+	*p*- , *q*-Values
light green	yes	−	*p*- , *q*-Values

Dot Plots Dot Plots

Protein	SgPgFn vs SgFn Log_2 Ratio	Log_2 Sum	*q*-Value	*p*-Value	Raw *SgPgFn*	Raw *SgFn*	Normalized *SgPgFn*	Normalized *SgFn*	Description
SGO_0639	-0.153	6.917	0.0555	0.3511	27.000	29.000	32.6068	29.0000	valS; valyl-tRNA synthetase
					18.000	32.000	24.7839	34.4569	
SGO_0641	-0.612	5.552	0.0071	0.0258	8.500	16.000	10.2651	16.0000	ATPase, histidine kinase-, DNA gyrase B-, and HSP90-like domain protein protein
					6.000	11.500	8.2613	12.3830	
SGO_0642	3.073	6.884	0.0005	0.0003	46.500	4.500	56.1562	4.5000	hypothetical protein SGO_0642
					35.500	8.000	48.8793	8.6142	
SGO_0643	-1.303	3.846	0.0181	0.0853	2.000	5.500	2.4153	5.5000	cytosine-specific methyltransferase
						6.000		6.4607	
SGO_0644	0.830	5.269	0.0045	0.0138	9.000	8.000	10.8689	8.0000	hypothetical protein SGO_0644
					10.000	5.500	13.7688	5.9223	
SGO_0652	0.556	3.540	0.0164	0.0761	3.500	2.500	4.2268	2.5000	hypothetical protein SGO_0652
					2.000	2.000	2.7538	2.1536	
SGO_0654	1.957	5.141	0.0205	0.1015	14.500	4.000	17.5111	4.0000	radical SAM enzyme, Cfr family
					10.000		13.7688		
SGO_0656	-1.703	5.379	0.0202	0.0985	5.500	8.000	6.6421	8.0000	trpB-2; tryptophan synthase, beta subunit
					2.000	22.500	2.7538	24.2275	
SGO_0665	0.300	8.721	0.0397	0.2299	115.500	107.500	139.4848	107.5000	non-heme iron-containing ferritin
					68.500	75.000	94.3164	80.7585	
SGO_0669	-0.151	7.331	0.0325	0.1814	30.000	38.500	36.2298	38.5000	typA; GTP-binding protein TypA
					29.000	43.000	39.9296	46.3015	
SGO_0671	-2.380	6.703	0.0004	0.0002	6.500	46.500	7.8498	46.5000	murD; UDP-N-acetylmuramoylalanine--D-glutamate ligase
					6.500	38.000	8.9497	40.9176	
SGO_0672	-3.122	5.069	0.0180	0.0847	1.500	14.000	1.8115	14.0000	murG; undecaprenyl-PP-MurNAc-pentapeptide-UDPGlcNAc GlcNAc transferase
						16.500		17.7669	

Log_2 Ratios: -6 -4 -2 0 2 4 6

Show detected proteins only
Show all proteins
Filter by category:
ABC Transporter

Proteins found: 584

Test	Cutoff
***q*-Value**	**.005**
p-Value	

	Signif	Direction	Applies To
red	yes	+	ratios, bars
yellow	no	n/a	bars
green	yes	–	ratios, bars
pink	yes	+	*p*- , *q*-Values
light green	yes	–	*p*- , *q*-Values

Dot Plots Dot Plots

Streptococcus gordonii

Protein	*SgPgFn vs SgFn* Log$_2$ Ratio	Log$_2$ Sum	*q*-Value	*p*-Value	Raw *SgPgFn*	Raw *SgFn*	Normalized *SgPgFn*	Normalized *SgFn*	Description
SGO_0673	-1.270	3.853	0.0431	0.2539	2.000	4.500	2.4153	4.5000	DivIB; cell division protein DivIB
						7.000		7.5375	
SGO_0674	-0.140	8.291	0.0145	0.0659	58.500	84.500	70.6481	84.5000	ftsA; cell division protein FtsA
					57.000	74.000	78.4823	79.6817	
SGO_0675	0.204	9.139	0.0478	0.2859	121.000	155.500	146.1269	155.5000	ftsZ; cell division protein FtsZ
					111.500	101.000	153.5224	108.7547	
SGO_0676	0.182	4.768	0.0997	0.6987	6.000	4.500	7.2460	4.5000	conserved hypothetical protein TIGR00044
					5.000	8.000	6.8844	8.6142	
SGO_0677	-0.046	6.798	0.1359	0.9911	29.000	26.500	35.0221	26.5000	ylmF protein
					15.000	27.000	20.6532	29.0730	
SGO_0680	0.341	7.668	0.0356	0.2039	41.000	55.000	49.5141	55.0000	cell division protein DivIVA
					46.000	33.000	63.3366	35.5337	
SGO_0681	0.551	7.377	0.0007	0.0008	42.500	33.500	51.3256	33.5000	ileS; isoleucyl-tRNA synthetase
					34.500	31.500	47.5024	33.9186	
SGO_0684	-0.924	7.324	0.0010	0.0012	23.500	49.000	28.3800	49.0000	hypothetical protein SGO_0684
					19.500	52.000	26.8492	55.9925	
SGO_0688	-0.832	6.996	0.0118	0.0511	19.000	31.000	22.9455	31.0000	ATP dependent Clp protease, ATP-binding subunit, ClpE
					16.000	48.000	22.0301	51.6854	
SGO_0693	-2.773	4.999	0.0401	0.2338		10.000		10.0000	xseA; exodeoxyribonuclease VII, large subunit
					1.500	18.500	2.0653	19.9204	
SGO_0700	0.210	4.677	0.0820	0.5588	4.000	7.500	4.8306	7.5000	DegV family protein
					6.500	4.000	8.9497	4.3071	
SGO_0701	0.186	11.819	0.0600	0.3839	898.500	705.500	1085.0828	705.5000	hup; DNA-binding histone-like protein HU
					605.000	919.000	833.0138	989.5603	

Log$_2$ Ratios: -6 -4 -2 0 2 4 6

Show detected proteins only
Show all proteins
Filter by category:
ABC Transporter

Proteins found: 584

Test	Cutoff
q-Value	.005
p-Value	

Color	Signif	Direction	Applies To
red	yes	+	ratios, bars
yellow	no	n/a	bars
green	yes	−	ratios, bars
pink	yes	+	*p*- , *q*-Values
light green	yes	−	*p*- , *q*-Values

Dot Plots Dot Plots

Streptococcus gordonii

Hackett Laboratory UW

Protein	SgPgFn vs SgFn				Raw		Normalized		Description	Log_2 Ratios
	Log_2 Ratio	Log_2 Sum	*q*-Value	*p*-Value	*SgPgFn*	*SgFn*	*SgPgFn*	*SgFn*		-6 -4 -2 0 2 4 6
SGO_0704	0.940	10.868	0.0004	0.0002	501.500	341.500	605.6417	341.5000	gpmA; 2,3-bisphosphoglycerate-dependent phosphoglycerate mutase	
					452.000	278.500	622.3508	299.8831		
SGO_0706	-1.667	5.147	0.0054	0.0186		15.000		15.0000	phoH-like protein	
					3.500	14.500	4.8191	15.6133		
SGO_0707	-5.080	7.793	0.0006	0.0005	4.000	118.500	4.8306	118.5000	LPXTG cell wall surface protein	
					1.500	89.500	2.0653	96.3718		
SGO_0708	-2.330	10.393	0.0001	0.0000	83.500	564.500	100.8396	564.5000	ald; alanine dehydrogenase	
					89.500	516.500	123.2310	556.1566		
SGO_0713	-0.925	6.666	0.0007	0.0007	16.000	32.500	19.3226	32.5000	sgg; GTP-binding protein Era	
					11.500	31.500	15.8341	33.9186		
SGO_0721	-0.265	4.636	0.1044	0.7373	7.000	6.500	8.4536	6.5000	abpB-like dipeptidase lipoprotein	
					2.500	6.000	3.4422	6.4607		
SGO_0736	-2.868	4.823	0.0101	0.0416	1.500	12.500	1.8115	12.5000	hprK; HPr(Ser) kinase/phosphatase	
						13.000		13.9981		
SGO_0739	1.421	4.361	0.0109	0.0466	7.500		9.0575		hypothetical protein SGO_0739	
					6.000	3.000	8.2613	3.2303		
SGO_0742	-0.616	5.103	0.0023	0.0056	7.000	13.000	8.4536	13.0000	peptidase, U32 family	
						12.000		12.9214		
SGO_0743	0.950	5.620	0.0085	0.0330	10.500	9.000	12.6804	9.0000	peptidase, U32 family	
					14.500	7.000	19.9648	7.5375		
SGO_0745	1.053	6.078	0.0389	0.2241	27.000		32.6068		hypothetical protein SGO_0745	
					16.000	12.000	22.0301	12.9214		
SGO_0749	-1.737	6.673	0.0041	0.0126	10.000	30.500	12.0766	30.5000	glutathione reductase	
					8.000	45.000	11.0151	48.4551		

Show detected proteins only
Show all proteins
Filter by category:
ABC Transporter

Proteins found: 584

Test	Cutoff
***q*-Value**	**.005**
p-Value	

	Signif	Direction	Applies To
red	yes	+	ratios, bars
yellow	no	n/a	bars
green	yes	−	ratios, bars
pink	yes	+	*p*- , *q*-Values
light green	yes	−	*p*- , *q*-Values

Dot Plots Dot Plots

Hendrickson *et al.*

Protein	SgPgFn vs SgFn Log$_2$ Ratio	Log$_2$ Sum	*q*-Value	*p*-Value	Raw *SgPgFn*	Raw *SgFn*	Normalized *SgPgFn*	Normalized *SgFn*	Description
SGO_0750	3.135	7.045	0.0004	0.0002	52.500	6.000	63.4022	6.0000	efflux transporter, RND family, MFP subunit subfamily
					40.000	7.000	55.0753	7.5375	
SGO_0751	3.211	6.554	0.0004	0.0003	37.500	3.500	45.2873	3.5000	ABC transporter, ATP-binding protein SP0786
					28.500	5.500	39.2411	5.9223	
SGO_0753	0.624	7.633	0.0121	0.0526	46.000	50.000	55.5524	50.0000	lysS; lysyl-tRNA synthetase
					46.000	27.500	63.3366	29.6114	
SGO_0755	-0.500	3.740	0.0565	0.3592		4.000		4.0000	regulatory protein
					2.500	5.500	3.4422	5.9223	
SGO_0760	-0.093	8.287	0.0546	0.3434	67.000	75.000	80.9132	75.0000	ppc; phosphoenolpyruvate carboxylase
					51.000	80.000	70.2210	86.1424	
SGO_0761	-0.330	12.893	0.0045	0.0138	1410.000	1976.000	1702.8011	1976.0000	tuf; translation elongation factor Tu
					1207.500	2103.500	1662.5854	2265.0056	
SGO_0762	1.303	10.244	0.0054	0.0182	436.000	150.500	526.5399	150.5000	tpiA; triosephosphate isomerase
					247.000	181.500	340.0899	195.4355	
SGO_0763	-0.615	6.340	0.0148	0.0673	17.500	24.500	21.1341	24.5000	murA-1; UDP-N-acetylglucosamine 1-carboxyvinyltransferase
					8.500	22.000	11.7035	23.6891	
SGO_0771	-0.658	7.462	0.0020	0.0046	28.000	58.500	33.8145	58.5000	pepq; proline dipeptidase
					25.000	46.000	34.4221	49.5319	
SGO_0773	-0.172	7.448	0.0253	0.1330	32.000	42.500	38.6451	42.5000	ccpA; catabolite control protein A
					31.500	46.500	43.3718	50.0702	
SGO_0774	-0.022	4.332	0.1048	0.7413	5.500	6.500	6.6421	6.5000	glycosyl transferase, group 1 family protein
						6.500		6.9991	
SGO_0775	-0.629	4.816	0.0103	0.0434	4.500	7.000	5.4345	7.0000	glycosyl transferase, group 1
					4.000	9.500	5.5075	10.2294	

Log$_2$ Ratios: -6 -4 -2 0 2 4 6

Show detected proteins only
Show all proteins
Filter by category:
ABC Transporter

Proteins found: 584

Test	Cutoff
***q*-Value** / *p*-Value	.005

	Signif	Direction	Applies To
red	yes	+	ratios, bars
yellow	no	n/a	bars
green	yes	–	ratios, bars
pink	yes	+	*p*- , *q*-Values
light green	yes	–	*p*- , *q*-Values

Dot Plots Dot Plots

SgPgFn vs SgFn

Streptococcus gordonii

Hackett Laboratory UW

Summary Table | SgFn vs Sg | SgPg vs Sg | SgPgFn vs Sg | SgPg vs SgFn | SgPgFn vs SgFn | SgPgFn vs SgPg | Coverage

Protein	SgPgFn vs SgFn Log_2 Ratio	Log_2 Sum	*q*-Value	*p*-Value	Raw SgPgFn	Raw SgFn	Normalized SgPgFn	Normalized SgFn	Description
SGO_0778	0.220	7.659	0.0011	0.0016	45.000	45.500	54.3447	45.5000	thrS; threonyl-tRNA synthetase
					39.500	44.500	54.3869	47.9167	
SGO_0779	-2.878	6.457	0.0005	0.0004	6.500	40.500	7.8498	40.5000	response regulator
					2.500	33.500	3.4422	36.0721	
SGO_0784	-1.232	5.715	0.0055	0.0190	6.500	14.500	7.8498	14.5000	smc; chromosome segregation protein SMC
					5.500	21.000	7.5729	22.6124	
SGO_0786	1.427	4.116	0.0282	0.1522	7.000		8.4536		Cof family protein
					4.500	2.500	6.1960	2.6919	
SGO_0787	-0.232	5.787	0.0490	0.2959	9.000	17.500	10.8689	17.5000	ftsY; cell division protein FtsY
					10.500	11.500	14.4573	12.3830	
SGO_0788	-1.100	7.383	0.0027	0.0071	22.000	49.000	26.5685	49.0000	zwf; glucose-6-phosphate 1-dehydrogenase
					19.000	60.500	26.1608	65.1452	
SGO_0792	1.458	5.456	0.0009	0.0011	14.000	7.000	16.9072	7.0000	hypothetical protein SGO_0792
					11.000	4.500	15.1457	4.8455	
SGO_0794	-0.896	6.619	0.0029	0.0081	13.500	28.000	16.3034	28.0000	metallo-beta-lactamase family protein
					13.000	33.500	17.8995	36.0721	
SGO_0795	-1.539	4.805	0.0031	0.0086	3.000	8.500	3.6230	8.5000	tributyrin esterase
					2.500	11.500	3.4422	12.3830	
SGO_0798	-0.286	5.940	0.0007	0.0007	11.500	16.500	13.8881	16.5000	ABC transporter, ATP-binding protein SP1381
					10.000	16.000	13.7688	17.2285	
SGO_0800	0.330	3.454	0.0202	0.0988	3.500	3.500	4.2268	3.5000	polysaccharide deacetylase family protein
						3.000		3.2303	
SGO_0801	0.123	7.802	0.0693	0.4577	58.500	54.500	70.6481	54.5000	hom; homoserine dehydrogenase
					34.000	47.500	46.8140	51.1470	

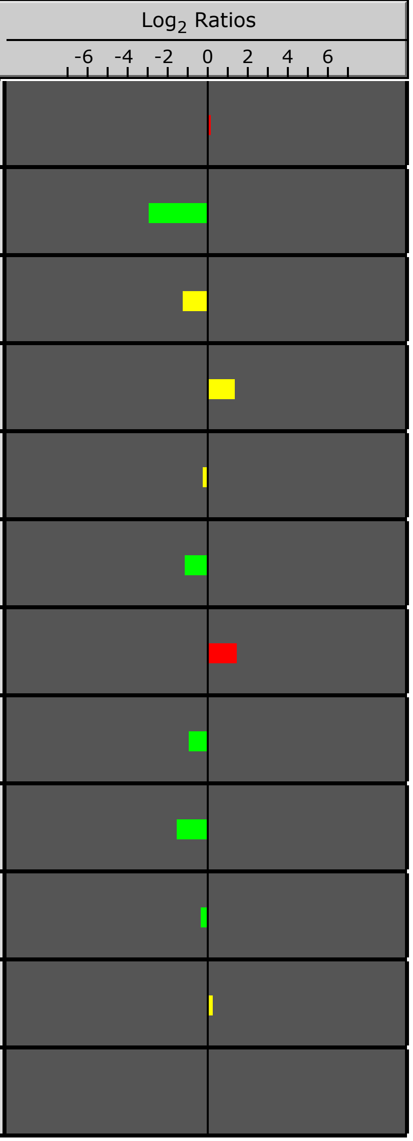

Show detected proteins only
Show all proteins
Filter by category:
ABC Transporter

Proteins found: 584

Test	Cutoff
q-Value	.005
p-Value	

Color	Signif	Direction	Applies To
red	yes	+	ratios, bars
yellow	no	n/a	bars
green	yes	–	ratios, bars
pink	yes	+	*p*- , *q*-Values
light green	yes	–	*p*- , *q*-Values

Dot Plots Dot Plots

Hendrickson *et al.*

Protein	*SgPgFn vs SgFn* Log_2 Ratio	Log_2 Sum	*q*-Value	*p*-Value	Raw *SgPgFn*	Raw *SgFn*	Normalized *SgPgFn*	Normalized *SgFn*	Description
**SGO_0802**	-0.982	5.582	0.0015	0.0031	5.500	16.000	6.6421	16.0000	thrB; homoserine kinase
					7.000	14.500	9.6382	15.6133	
**SGO_0815**	0.556	5.645	0.0041	0.0124	11.500	9.000	13.8881	9.0000	thiI; thiamine biosynthesis protein ThiI
					11.500	10.500	15.8341	11.3062	
**SGO_0818**	3.147	8.361	0.0134	0.0601	118.000	17.500	142.5039	17.5000	rplU; ribosomal protein L21
					122.500		168.6681		
**SGO_0820**	0.734	6.156	0.0515	0.3181	28.000		33.8145		rpmA; ribosomal protein L27
					15.500	15.000	21.3417	16.1517	
**SGO_0824**	0.136	6.548	0.0078	0.0297	19.500	22.500	23.5494	22.5000	lepA; GTP-binding protein LepA
					18.500	20.500	25.4723	22.0740	
**SGO_0832**	0.783	2.444				2.000		2.0000	hypothetical protein SGO_0832
					2.500		3.4422		
**SGO_0835**	-1.203	8.066	0.0008	0.0008	28.500	93.000	34.4183	93.0000	nitroreductase
					34.500	86.500	47.5024	93.1414	
**SGO_0836**	-0.820	8.604	0.0015	0.0029	65.000	130.500	78.4979	130.5000	pepV; dipeptidase PepV
					45.500	109.000	62.6481	117.3690	
**SGO_0842**	-2.356	5.463	0.0027	0.0073	3.000	22.500	3.6230	22.5000	rhodanese family protein
					2.500	13.500	3.4422	14.5365	
**SGO_0848**	2.122	7.230	0.0005	0.0003	52.000	18.000	62.7983	18.0000	rpmE; ribosomal protein L31
					42.500	10.000	58.5175	10.7678	
**SGO_0850**	1.997	7.200	0.0101	0.0422	68.500	16.000	82.7247	16.0000	flavodoxin
					26.500	11.000	36.4874	11.8446	
**SGO_0854**	-2.678	5.896	0.0048	0.0152	3.000	33.500	3.6230	33.5000	cshA; surface-associated protein CshA
					3.000	17.000	4.1306	18.3053	

Log_2 Ratios: -6 -4 -2 0 2 4 6

Show detected proteins only
Show all proteins
Filter by category:
ABC Transporter

Proteins found: 584

Test	Cutoff
***q*-Value**	**.005**
p-Value	

Color	Signif	Direction	Applies To
red	yes	+	ratios, bars
yellow	no	n/a	bars
green	yes	−	ratios, bars
light red	yes	+	*p*- , *q*-Values
light green	yes	−	*p*- , *q*-Values

Dot Plots Dot Plots

Hendrickson *et al.*

Protein	SgPgFn vs SgFn: Log_2 Ratio	Log_2 Sum	*q*-Value	*p*-Value	Raw: *SgPgFn*	Raw: *SgFn*	Normalized: *SgPgFn*	Normalized: *SgFn*	Description
**SGO_0856**	1.007	5.228	0.0013	0.0022	11.000	5.500	13.2843	5.5000	ABC transporter, substrate binding protein
					8.500	6.500	11.7035	6.9991	
**SGO_0859**	-1.212	6.420	0.0006	0.0004	9.500	29.000	11.4728	29.0000	pheS; phenylalanyl-tRNA synthetase, alpha subunit
					10.500	28.500	14.4573	30.6882	
**SGO_0861**	0.878	7.798	0.0013	0.0023	56.000	35.500	67.6290	35.5000	pheT; phenylalanyl-tRNA synthetase, beta subunit
					55.500	40.000	76.4170	43.0712	
**SGO_0885**	-1.267	4.744	0.0049	0.0163	3.000	11.500	3.6230	11.5000	cobyric acid synthase
					3.000	7.000	4.1306	7.5375	
**SGO_0889**	-0.291	5.387	0.0344	0.1939	9.500	11.500	11.4728	11.5000	glmM; phosphoglucosamine mutase
					5.500	10.500	7.5729	11.3062	
**SGO_0893**	-0.251	6.921	0.0001	0.0000	23.000	33.000	27.7762	33.0000	GTP-binding protein
					20.000	30.500	27.5376	32.8418	
**SGO_0901**	-1.363	5.098	0.0016	0.0033	3.000	11.500	3.6230	11.5000	DNA-directed DNA polymerase III
					4.500	12.000	6.1960	12.9214	
**SGO_0906**	-1.439	7.258	0.0005	0.0004	14.500	56.500	17.5111	56.5000	leuA; 2-isopropylmalate synthase
					17.500	51.000	24.0954	54.9158	
**SGO_0911**	-0.121	7.397	0.0554	0.3488	29.500	45.000	35.6260	45.0000	hypothetical protein SGO_0911
					33.000	39.500	45.4371	42.5328	
**SGO_0946**	-1.336	5.975	0.0016	0.0033	5.500	23.000	6.6421	23.0000	Deblocking aminopeptidase
					8.500	20.000	11.7035	21.5356	
**SGO_0949**	-0.281	3.421	0.0875	0.6042	2.500	5.000	3.0192	5.0000	deaD; DEAD RNA helicase
						2.500		2.6919	
**SGO_0950**	-2.074	4.525	0.0255	0.1345	2.000	12.000	2.4153	12.0000	oxidoreductase
						8.000		8.6142	

Log2 Ratios: -6 -4 -2 0 2 4 6

Show detected proteins only
Show all proteins
Filter by category:
ABC Transporter

Proteins found: 584

Test	Cutoff
***q*-Value**	**.005**
p-Value	

	Signif	Direction	Applies To
red	yes	+	ratios, bars
yellow	no	n/a	bars
green	yes	–	ratios, bars
pink	yes	+	*p*- , *q*-Values
light green	yes	–	*p*- , *q*-Values

Dot Plots | Dot Plots

Hendrickson *et al.*

Protein	SgPgFn vs SgFn Log$_2$ Ratio	Log$_2$ Sum	*q*-Value	*p*-Value	Raw *SgPgFn*	Raw *SgFn*	Normalized *SgPgFn*	Normalized *SgFn*	Description
SGO_0951	-0.146	4.844	0.0608	0.3924	5.000	6.500	6.0383	6.5000	udk; uridine kinase
					5.500	8.000	7.5729	8.6142	
SGO_0954	-0.015	5.584	0.1274	0.9148	7.500	13.000	9.0575	13.0000	ATP-binding protein
					11.000	10.000	15.1457	10.7678	
SGO_0982	-0.924	4.987	0.0004	0.0002	4.500	10.000	5.4345	10.0000	amino acid ABC transporter, amino acid-binding protein
					4.000	10.000	5.5075	10.7678	
SGO_0987	0.076	6.818	0.0052	0.0176	24.000	28.000	28.9838	28.0000	metK; S-adenosylmethionine synthetase
					21.000	25.000	28.9145	26.9195	
SGO_0993	-0.346	3.118	0.0694	0.4602	2.000	2.500	2.4153	2.5000	GTP-binding protein HflX
						3.500		3.7687	
SGO_0995	-2.159	4.605	0.0271	0.1436	2.000	9.000	2.4153	9.0000	metallo-beta-lactamase superfamily protein
						12.000		12.9214	
SGO_1001	-0.540	7.161	0.0225	0.1147	20.000	33.000	24.1532	33.0000	apt; adenine phosphoribosyltransferase
					24.500	48.500	33.7336	52.2238	
SGO_1005	0.152	4.203	0.0501	0.3034	4.000	5.000	4.8306	5.0000	Bcl-2 family protein
					3.500	3.500	4.8191	3.7687	
SGO_1009	-0.925	8.563	0.0020	0.0047	46.000	115.000	55.5524	115.0000	rfbA-1; glucose-1-phosphate thymidylyltransferase
					55.000	122.500	75.7285	131.9055	
SGO_1010	0.100	6.619	0.0511	0.3146	21.500	26.000	25.9647	26.0000	rmlC; dTDP-4-keto-6-deoxyglucose-3,5-epimerase
					18.000	20.000	24.7839	21.5356	
SGO_1011	-1.735	8.174	0.0028	0.0076	25.500	90.500	30.7953	90.5000	rfbB-1; dTDP-glucose 4,6-dehydratase
					25.500	123.000	35.1105	132.4439	
SGO_1012	-1.048	8.277	0.0001	0.0000	41.000	102.500	49.5141	102.5000	galE-1; UDP-glucose 4-epimerase
					37.500	99.000	51.6331	106.6012	

Log$_2$ Ratios: -6 -4 -2 0 2 4 6

Show detected proteins only
Show all proteins
Filter by category:
ABC Transporter

Proteins found: 584

Test	Cutoff
q-Value	.005
p-Value	

	Signif	Direction	Applies To
red	yes	+	ratios, bars
yellow	no	n/a	bars
green	yes	−	ratios, bars
pink	yes	+	*p*- , *q*-Values
light green	yes	−	*p*- , *q*-Values

Dot Plots Dot Plots

Protein	SgPgFn vs SgFn Log$_2$ Ratio	Log$_2$ Sum	*q*-Value	*p*-Value	Raw SgPgFn	Raw SgFn	Normalized SgPgFn	Normalized SgFn	Description
SGO_1013	1.040	3.303			5.500		6.6421		Glycosyltransferase involved in cell wall biogenesis
						3.000		3.2303	
SGO_1016	0.272	2.464			2.500	2.500	3.0192	2.5000	putative glycosyltransferase
SGO_1019	-0.835	2.334			1.500		1.8115		glycosyl transferase
						3.000		3.2303	
SGO_1020	-0.622	6.999	0.0020	0.0046	23.000	37.000	27.7762	37.0000	rfbD; dTDP-4-dehydrorhamnose reductase
					16.500	37.500	22.7186	40.3792	
SGO_1025	0.495	4.844	0.0527	0.3287	8.500	8.500	10.2651	8.5000	rgp; glycosyltransferase
					4.500	3.500	6.1960	3.7687	
SGO_1026	-1.260	5.145	0.0030	0.0084	4.000	10.500	4.8306	10.5000	rhamnosyltransferase
					4.000	13.500	5.5075	14.5365	
SGO_1031	-0.479	3.944	0.0246	0.1287	2.000	4.000	2.4153	4.0000	cmk; cytidylate kinase
					3.000	4.500	4.1306	4.8455	
SGO_1033	-0.273	5.973	0.0937	0.6504	7.500	17.500	9.0575	17.5000	rpmI; ribosomal protein L35
					15.000	14.500	20.6532	15.6133	
SGO_1034	0.310	8.455	0.0481	0.2893	108.000	79.000	130.4273	79.0000	rplT; ribosomal protein L20
					49.500	68.000	68.1557	73.2210	
SGO_1035	-0.653	5.126	0.0027	0.0070	5.000	10.000	6.0383	10.0000	gloA; lactoylglutathione lyase
					5.500	10.500	7.5729	11.3062	
SGO_1036	-2.060	5.362	0.0045	0.0142	3.000	21.000	3.6230	21.0000	amino acid ABC transporter, ATP-binding protein SP1242
					3.000	11.500	4.1306	12.3830	
SGO_1038	-0.187	4.477	0.0694	0.4605	3.500	7.000	4.2268	7.0000	uvrB; excinuclease ABC, B subunit
					4.500	4.500	6.1960	4.8455	

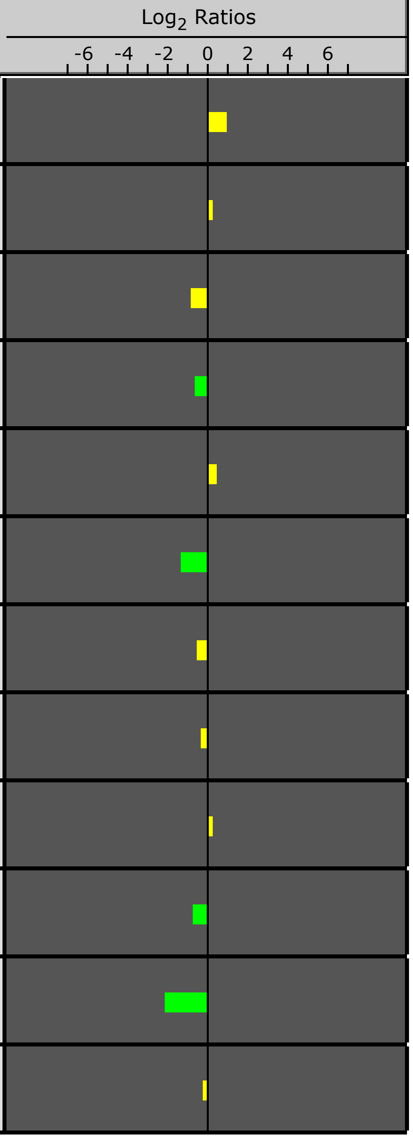

Show detected proteins only
Show all proteins
Filter by category:
ABC Transporter

Proteins found: 584

Test	Cutoff
q-Value	.005
p-Value	

	Signif	Direction	Applies To
red	yes	+	ratios, bars
yellow	no	n/a	bars
green	yes	−	ratios, bars
pink	yes	+	*p*- , *q*-Values
light green	yes	−	*p*- , *q*-Values

Dot Plots Dot Plots

Protein	SgPgFn vs SgFn Log$_2$ Ratio	Log$_2$ Sum	*q*-Value	*p*-Value	Raw *SgPgFn*	Raw *SgFn*	Normalized *SgPgFn*	Normalized *SgFn*	Description
SGO_1047	1.697	3.631	0.0356	0.2046	5.500		6.6421		hypothetical protein SGO_1047
					3.000	1.500	4.1306	1.6152	
SGO_1050	-0.230	2.581							ribF; riboflavin biosynthesis protein RibF
					2.000	3.000	2.7538	3.2303	
SGO_1059	2.447	5.537	0.0001	0.0000	16.500	4.000	19.9264	4.0000	pstB; Phosphate import ATP-binding protein pstB 1 (Phosphate-transporting ATPase 1) (ABC phosphate transporter 1)
					14.000	3.000	19.2764	3.2303	
SGO_1060	0.365	5.333	0.0135	0.0606	9.000	7.500	10.8689	7.5000	phosphate transport system regulatory protein
					8.500	9.500	11.7035	10.2294	
SGO_1065	-0.231	4.888	0.0351	0.1995	5.000	9.000	6.0383	9.0000	hypothetical protein SGO_1065
					5.500	6.500	7.5729	6.9991	
SGO_1069	-0.094	7.585	0.0233	0.1204	36.500	49.000	44.0796	49.0000	membrane alanyl aminopeptidase
					35.500	46.500	48.8793	50.0702	
SGO_1079	-1.692	6.333	0.0101	0.0421	7.000	20.000	8.4536	20.0000	pdp; pyrimidine-nucleoside phosphorylase
					7.000	39.500	9.6382	42.5328	
SGO_1080	-0.109	7.094	0.0180	0.0847	26.500	37.000	32.0030	37.0000	deoC; deoxyribose-phosphate aldolase
					24.500	31.500	33.7336	33.9186	
SGO_1082	0.686	11.027	0.0202	0.0984	690.500	443.000	833.8895	443.0000	lipoprotein
					340.500	316.500	468.8284	340.8007	
SGO_1083	0.463	7.643	0.0001	0.0000	48.000	41.500	57.9677	41.5000	sugar ABC transporter, ATP-binding protein SP0846
					42.000	39.500	57.8291	42.5328	
SGO_1096	-4.048	12.301	0.0002	0.0001	140.000	2269.000	169.0724	2269.0000	butA; acetoin dehydrogenase
					88.500	2308.500	121.8541	2485.7454	
SGO_1098	-2.043	5.516	0.0001	0.0000	4.000	18.500	4.8306	18.5000	proA; gamma-glutamyl phosphate reductase
					3.000	17.000	4.1306	18.3053	

Log$_2$ Ratios: -6 -4 -2 0 2 4 6

◉ Show detected proteins only
○ Show all proteins
☐ Filter by category:
ABC Transporter

Proteins found: 584

Test	Cutoff
q-Value	.005
p-Value	

	Signif	Direction	Applies To
red	yes	+	ratios, bars
yellow	no	n/a	bars
green	yes	−	ratios, bars
pink	yes	+	*p*- , *q*-Values
light green	yes	−	*p*- , *q*-Values

Dot Plots | Dot Plots

Streptococcus gordonii

Protein	SgPgFn vs SgFn: Log_2 Ratio	Log_2 Sum	*q*-Value	*p*-Value	Raw: *SgPgFn*	Raw: *SgFn*	Normalized: *SgPgFn*	Normalized: *SgFn*	Description
SGO_1104	0.750	3.115			4.500		5.4345		carB; carbamoyl-phosphate synthase, large subunit
						3.000		3.2303	
SGO_1107	0.770	2.127							PyrR bifunctional protein
					2.000	1.500	2.7538	1.6152	
SGO_1109	1.035	6.157	0.0011	0.0017	21.500	11.500	25.9647	11.5000	pyrB; aspartate carbamoyltransferase
					16.000	11.000	22.0301	11.8446	
SGO_1111	-0.366	3.112	0.0209	0.1038	2.000	3.000	2.4153	3.0000	fruR; phosphotransferase system repressor
						3.000		3.2303	
SGO_1112	-0.983	3.774	0.0214	0.1074		5.000		5.0000	fruB; 1-phosphofructokinase
					2.000	5.500	2.7538	5.9223	
SGO_1113	0.779	6.817	0.0182	0.0864	26.000	30.000	31.3992	30.0000	fruA; PTS system, fructose specific IIABC components
					27.500	12.500	37.8643	13.4597	
SGO_1114	0.299	4.065	0.0612	0.3976	3.500	5.000	4.2268	5.0000	Protein of unknown function (DUF1149) superfamily
					3.500	2.500	4.8191	2.6919	
SGO_1116	-0.021	6.214	0.1274	0.9159	13.500	19.500	16.3034	19.5000	dapB; dihydrodipicolinate reductase
					15.000	16.500	20.6532	17.7669	
SGO_1120	-0.861	7.879	0.0041	0.0122	29.500	67.000	35.6260	67.0000	guaA; GMP synthase
					35.000	78.500	48.1909	84.5272	
SGO_1123	0.048	7.074	0.0814	0.5514	26.500	35.000	32.0030	35.0000	ffh; signal recognition particle protein
					26.500	29.000	36.4874	31.2266	
SGO_1129	-0.410	5.221	0.0013	0.0022	7.000	10.500	8.4536	10.5000	lplA; lipoate protein ligase A
					5.500	10.000	7.5729	10.7678	
SGO_1130	2.087	6.027	0.0001	0.0000	22.000	5.500	26.5685	5.5000	dihydrolipoamide dehydrogenase
					19.000	6.500	26.1608	6.9991	

Log_2 Ratios: -6 -4 -2 0 2 4 6

Show detected proteins only
Show all proteins
Filter by category:
ABC Transporter

Proteins found: 584

Test	Cutoff
q-Value	.005
p-Value	

Color	Signif	Direction	Applies To
red	yes	+	ratios, bars
yellow	no	n/a	bars
green	yes	−	ratios, bars
pink	yes	+	*p*- , *q*-Values
light green	yes	−	*p*- , *q*-Values

Dot Plots Dot Plots

Protein	*SgPgFn vs SgFn* Log$_2$ Ratio	Log$_2$ Sum	*q*-Value	*p*-Value	Raw *SgPgFn*	Raw *SgFn*	Normalized *SgPgFn*	Normalized *SgFn*	Description
SGO_1131	1.791	4.327	0.0283	0.1530	6.000	2.500	7.2460	2.5000	sucB; dihydrolipoamide S-acetyltransferase
					7.500		10.3266		
SGO_1133	-0.255	4.367	0.0958	0.6663	4.000		4.8306		acoA; acetoin dehydrogenase
					6.000	7.000	8.2613	7.5375	
SGO_1134	-2.199	4.884	0.0423	0.2490		9.000		9.0000	hypothetical protein SGO_1134
					2.000	16.500	2.7538	17.7669	
SGO_1139	0.176	5.568	0.0074	0.0275	10.000	11.500	12.0766	11.5000	GTP-binding protein
					9.500	10.000	13.0804	10.7678	
SGO_1140	-0.194	7.274	0.0080	0.0304	29.500	39.000	35.6260	39.0000	clpX; ATP-dependent Clp protease, ATP-binding subunit ClpX
					26.500	40.500	36.4874	43.6096	
SGO_1141	-1.932	4.762	0.0407	0.2387	2.500	8.500	3.0192	8.5000	folA; dihydrofolate reductase
						14.500		15.6133	
SGO_1143	0.407	3.951	0.0709	0.4709	5.000	3.500	6.0383	3.5000	thyA; thymidylate synthase
						5.500		5.9223	
SGO_1144	-0.562	9.079	0.0024	0.0060	98.500	152.500	118.9545	152.5000	glcK; glucokinase
					72.500	157.500	99.8240	169.5928	
SGO_1150	-0.014	3.870	0.1271	0.9104		5.500		5.5000	hypothetical protein SGO_1150
					3.500	4.000	4.8191	4.3071	
SGO_1151	-0.674	9.082	0.0002	0.0001	84.000	165.000	101.4435	165.0000	glyA; serine hydroxymethyltransferase
					78.000	156.000	107.3968	167.9776	
SGO_1154	0.396	6.592	0.0614	0.4010	20.500	30.000	24.7570	30.0000	prfA; peptide chain release factor 1
					20.500	12.500	28.2261	13.4597	
SGO_1155	1.283	3.830	0.0310	0.1716		4.500		4.5000	tdk; thymidine kinase
					5.500	2.000	7.5729	2.1536	

Log$_2$ Ratios: -6 -4 -2 0 2 4 6

Show detected proteins only
Show all proteins
Filter by category:
ABC Transporter

Proteins found: 584

Test	Cutoff
***q*-Value**	**.005**
p-Value	

	Signif	Direction	Applies To
red	yes	+	ratios, bars
yellow	no	n/a	bars
green	yes	−	ratios, bars
pink	yes	+	*p*-, *q*-Values
light green	yes	−	*p*-, *q*-Values

Dot Plots | Dot Plots

Streptococcus gordonii

Hackett Laboratory UW

Protein	SgPgFn vs SgFn				Raw		Normalized		Description
	Log_2 Ratio	Log_2 Sum	*q*-Value	*p*-Value	*SgPgFn*	*SgFn*	*SgPgFn*	*SgFn*	
**SGO_1167**	0.833	6.143	0.0129	0.0571	14.000	13.000	16.9072	13.0000	nox; NADH oxidase
					21.000	11.000	28.9145	11.8446	
**SGO_1169**	1.302	6.132	0.0011	0.0015	20.500	12.500	24.7570	12.5000	NADPH-dependent FMN reductase
					18.000	7.500	24.7839	8.0758	
**SGO_1170**	1.328	6.638	0.0006	0.0005	31.000	15.500	37.4375	15.5000	NADPH-dependent FMN reductase
					24.500	12.000	33.7336	12.9214	
**SGO_1185**	0.498	3.270	0.0501	0.3038	2.500	2.500	3.0192	2.5000	acetyltransferase, GNAT family
					3.000		4.1306		
**SGO_1189**	-0.877	6.105	0.0154	0.0708	15.000	20.000	18.1149	20.0000	lipoprotein, putative
					5.500	21.500	7.5729	23.1508	
**SGO_1191**	1.746	10.556	0.0004	0.0002	454.000	178.000	548.2778	178.0000	rplL; ribosomal protein L7/L12
					444.500	155.500	612.0242	167.4392	
**SGO_1192**	0.112	7.996	0.0872	0.5997	56.500	50.500	68.2328	50.5000	BL5; 50S ribosomal protein L10
					46.000	68.000	63.3366	73.2210	
**SGO_1193**	-2.230	5.464	0.0004	0.0003	3.000	17.000	3.6230	17.0000	gid; Glucose inhibited division protein A
					3.000	18.000	4.1306	19.3820	
**SGO_1197**	0.163	5.590	0.0770	0.5164	10.000	9.000	12.0766	9.0000	topA; DNA topoisomerase I
					9.500	13.000	13.0804	13.9981	
**SGO_1203**	-0.930	6.041	0.0020	0.0048	8.500	19.500	10.2651	19.5000	anaerobic ribonucleotide reductase
					9.000	22.000	12.3919	23.6891	
**SGO_1205**	-0.368	4.202	0.0507	0.3092	3.500	7.500	4.2268	7.5000	dapA; dihydrodipicolinate synthase
					2.500	3.000	3.4422	3.2303	
**SGO_1206**	-0.043	6.619	0.0817	0.5556	19.000	23.500	22.9455	23.5000	asd; aspartate-semialdehyde dehydrogenase
					18.500	24.500	25.4723	26.3811	

Log_2 Ratios: -6 -4 -2 0 2 4 6

- Show detected proteins only
- Show all proteins
- Filter by category: ABC Transporter

Proteins found: 584

Test	Cutoff
***q*-Value**	**.005**
p-Value	

Color	Signif	Direction	Applies To
red	yes	+	ratios, bars
yellow	no	n/a	bars
green	yes	−	ratios, bars
pink	yes	+	***p*- , *q*-Values**
light green	yes	−	***p*- , *q*-Values**

Dot Plots Dot Plots

Hendrickson *et al.*

Protein	*SgPgFn* vs *SgFn* Log2 Ratio	Log2 Sum	*q*-Value	*p*-Value	Raw *SgPgFn*	Raw *SgFn*	Normalized *SgPgFn*	Normalized *SgFn*	Description
SGO_1210	-2.125	4.887	0.0218	0.1097	2.500	11.500	3.0192	11.5000	fhs-1; formate--tetrahydrofolate ligase
						14.000		15.0749	
SGO_1215	-0.753	9.229	0.0009	0.0010	92.500	178.000	111.7086	178.0000	manB; phosphomannomutase
					81.000	184.500	111.5275	198.6658	
SGO_1216	-0.222	6.206	0.0131	0.0582	14.500	21.500	17.5111	21.5000	bta; Possible bacteriocin transport accessory protein
					12.000	17.000	16.5226	18.3053	
SGO_1219	-1.048	7.136	0.0161	0.0745	16.500	31.500	19.9264	31.5000	pta; phosphate acetyltransferase
					17.500	60.500	24.0954	65.1452	
SGO_1224	1.233	7.325	0.0011	0.0015	42.500	23.500	51.3256	23.5000	Ribose-phosphate pyrophosphokinase 2
					44.500	22.500	61.2713	24.2275	
SGO_1225	0.021	4.378	0.1295	0.9390	4.000	4.000	4.8306	4.0000	pyridoxal-phosphate dependent aminotransferase
					4.000	6.000	5.5075	6.4607	
SGO_1226	-0.323	5.511	0.0327	0.1832	8.000	10.000	9.6613	10.0000	hypothetical protein SGO_1226
					7.500	14.500	10.3266	15.6133	
SGO_1231	-1.234	6.445	0.0028	0.0078	10.500	35.500	12.6804	35.5000	gyrA; DNA gyrase, A subunit
					9.500	24.000	13.0804	25.8427	
SGO_1232	0.065	10.119	0.0078	0.0295	234.000	277.000	282.5925	277.0000	L-lactate dehydrogenase
					207.500	247.500	285.7031	266.5029	
SGO_1234	0.997	9.720	0.0011	0.0016	228.500	163.000	275.9504	163.0000	rpsA; 30S ribosomal protein S1
					206.000	112.000	283.6378	120.5993	
SGO_1238	-1.517	6.634	0.0056	0.0194	9.000	46.000	10.8689	46.0000	ilvE; branched-chain amino acid aminotransferase
					10.500	26.000	14.4573	27.9963	
SGO_1239	-0.555	3.569	0.0403	0.2359	2.000	2.000	2.4153	2.0000	parC; DNA topoisomerase IV, A subunit
					1.500	5.000	2.0653	5.3839	

Log2 Ratios: -6 -4 -2 0 2 4 6

Show detected proteins only
Show all proteins
Filter by category:
ABC Transporter

Proteins found: 584

Test	Cutoff
***q*-Value**	**.005**
p-Value	

	Signif	Direction	Applies To
red	yes	+	ratios, bars
yellow	no	n/a	bars
green	yes	−	ratios, bars
pink	yes	+	*p*- , *q*-Values
light green	yes	−	*p*- , *q*-Values

Dot Plots Dot Plots

Protein	SgPgFn vs SgFn Log2 Ratio	Log2 Sum	q-Value	p-Value	Raw SgPgFn	Raw SgFn	Normalized SgPgFn	Normalized SgFn	Description
SGO_1248	-2.297	5.699	0.0069	0.0249	3.500	14.500	4.2268	14.5000	pyrC; dihydroorotase
					3.000	27.000	4.1306	29.0730	
SGO_1253	0.773	5.347	0.0134	0.0603	9.000	10.000	10.8689	10.0000	pyrE; orotate phosphoribosyltransferase
					10.500	5.000	14.4573	5.3839	
SGO_1260	1.059	6.742	0.0009	0.0010	32.000	18.000	38.6451	18.0000	deoD; purine nucleoside phosphorylase
					24.500	15.500	33.7336	16.6901	
SGO_1263	0.737	6.819	0.0083	0.0316	24.000	22.000	28.9838	22.0000	purine nucleoside phosphorylase I, inosine and guanosine-specific
					30.500	18.500	41.9949	19.9204	
SGO_1264	-0.269	8.897	0.0403	0.2361	75.500	149.500	91.1784	149.5000	deoB; phosphopentomutase
					91.000	103.000	125.2963	110.9083	
SGO_1265	0.802	6.467	0.0014	0.0026	21.500	15.500	25.9647	15.5000	rpiA; ribose 5-phosphate isomerase
					22.000	15.500	30.2914	16.6901	
SGO_1266	-0.405	5.365	0.0117	0.0504	8.500	11.000	10.2651	11.0000	trmE; tRNA modification GTPase TrmE
					5.500	11.500	7.5729	12.3830	
SGO_1273	1.678	7.509	0.0037	0.0108	68.500	27.000	82.7247	27.0000	rpoD; RNA polymerase sigma factor
					40.500	15.500	55.7637	16.6901	
SGO_1276	2.077	6.395	0.0013	0.0021	24.500	8.500	29.5877	8.5000	rpsU; ribosomal protein S21
					28.000	7.000	38.5527	7.5375	
SGO_1281	0.770	2.127							penicillinase repressor, putative
					2.000	1.500	2.7538	1.6152	
SGO_1283	0.708	6.989	0.0018	0.0041	32.500	27.500	39.2490	27.5000	oxidoreductase
					28.500	19.500	39.2411	20.9972	
SGO_1284	0.479	4.727	0.0518	0.3205	5.500	3.500	6.6421	3.5000	thioredoxin-disulfide reductase
					6.000	7.500	8.2613	8.0758	

Log2 Ratios: -6 -4 -2 0 2 4 6

Show detected proteins only
Show all proteins
Filter by category:
ABC Transporter

Proteins found: 584

Test	Cutoff
q-Value	.005
p-Value	

	Signif	Direction	Applies To
red	yes	+	ratios, bars
yellow	no	n/a	bars
green	yes	−	ratios, bars
pink	yes	+	p- , q-Values
light green	yes	−	p- , q-Values

Dot Plots Dot Plots

Hendrickson *et al.*

Protein	*SgPgFn vs SgFn* Log$_2$ Ratio	Log$_2$ Sum	*q*-Value	*p*-Value	Raw *SgPgFn*	Raw *SgFn*	Normalized *SgPgFn*	Normalized *SgFn*	Description
SGO_1293	-0.058	6.586	0.1292	0.9336	15.000	22.500	18.1149	22.5000	asnS; asparaginyl-tRNA synthetase
					21.500	24.000	29.6030	25.8427	
SGO_1297	-1.140	6.383	0.0042	0.0127	10.500	34.000	12.6804	34.0000	aspC; aspartate aminotransferase
					9.500	22.000	13.0804	23.6891	
SGO_1312	-0.546	7.056	0.0025	0.0065	25.000	40.000	30.1915	40.0000	pepT; peptidase T
					17.500	36.000	24.0954	38.7641	
SGO_1323	1.425	7.057	0.0003	0.0002	41.500	19.500	50.1179	19.5000	rpsP; ribosomal protein S16
					34.000	15.500	46.8140	16.6901	
SGO_1327	-0.522	4.460	0.0011	0.0014	3.500	6.500	4.2268	6.5000	HAD-superfamily subfamily IIA hydrolase, TIGR01457
					3.500	6.000	4.8191	6.4607	
SGO_1336	-1.119	4.961	0.0062	0.0218	3.500	8.500	4.2268	8.5000	pcrA; ATP-dependent DNA helicase PcrA
					4.000	12.000	5.5075	12.9214	
SGO_1339	0.328	10.883	0.0048	0.0154	454.000	447.500	548.2778	447.5000	pyk; pyruvate kinase
					364.500	362.500	501.8736	390.3326	
SGO_1340	-0.610	9.611	0.0008	0.0008	124.500	227.000	150.3537	227.0000	Phosphofructokinase
					115.500	228.000	159.0299	245.5057	
SGO_1342	-0.690	8.816	0.0033	0.0095	71.000	155.000	85.7439	155.0000	ABC transporter, ATP-binding protein SP1715
					62.500	115.000	86.0551	123.8296	
SGO_1364	-1.496	4.199	0.0205	0.1008		7.000		7.0000	rumA-2; 23S rRNA (uracil-5-)-methyltransferase RumA
					2.000	8.000	2.7538	8.6142	
SGO_1365	-2.204	4.820	0.0169	0.0791		11.500		11.5000	transcription regulator yrfE
					2.000	13.000	2.7538	13.9981	
SGO_1369	-4.275	7.170	0.0063	0.0223	3.000	72.500	3.6230	72.5000	L-2-hydroxyisocaproate dehydrogenase
						63.000		67.8371	

Log$_2$ Ratios: -6 -4 -2 0 2 4 6

Show detected proteins only
Show all proteins
Filter by category:
ABC Transporter

Proteins found: 584

Test	Cutoff
q-Value	.005
p-Value	

	Signif	Direction	Applies To
red	yes	+	ratios, bars
yellow	no	n/a	bars
green	yes	−	ratios, bars
pink	yes	+	*p*- , *q*-Values
light green	yes	−	*p*- , *q*-Values

Dot Plots Dot Plots

Protein	SgPgFn vs SgFn Log_2 Ratio	Log_2 Sum	*q*-Value	*p*-Value	Raw *SgPgFn*	Raw *SgFn*	Normalized *SgPgFn*	Normalized *SgFn*	Description
SGO_1370	0.318	6.355	0.0193	0.0935	17.500	21.000	21.1341	21.0000	Protein of unknown function (DUF964) superfamily
					17.500	14.500	24.0954	15.6133	
SGO_1372	0.770	3.127							aroC; chorismate synthase
					4.000	3.000	5.5075	3.2303	
SGO_1373	-0.419	3.082			3.000		3.6230		aroB; 3-dehydroquinate synthase
						4.500		4.8455	
SGO_1375	0.081	4.126	0.0571	0.3649	5.000	5.500	6.0383	5.5000	aroD; 3-dehydroquinate dehydratase, type I
						5.500		5.9223	
SGO_1377	-2.269	5.508	0.0005	0.0004	4.500	19.000	5.4345	19.0000	sulfatase
					2.000	17.000	2.7538	18.3053	
SGO_1381	-0.925	5.231	0.0052	0.0172	5.500	14.500	6.6421	14.5000	csn1; CRISPR-associated protein, Csn1 family
					4.500	9.500	6.1960	10.2294	
SGO_1383	0.716	8.032	0.0013	0.0023	65.500	44.500	79.1018	44.5000	rplS; ribosomal protein L19
					60.500	51.000	83.3014	54.9158	
SGO_1390	-0.176	4.509	0.0817	0.5555	5.500	5.000	6.6421	5.0000	ligA; DNA ligase, NAD-dependent
					3.000	6.500	4.1306	6.9991	
SGO_1397	-0.617	5.803	0.0048	0.0153	8.500	15.000	10.2651	15.0000	map; methionine aminopeptidase, type I
					8.500	17.500	11.7035	18.8436	
SGO_1400	-0.951	4.842	0.0202	0.0980	6.500	10.000	7.8498	10.0000	murA-2; UDP-N-acetylglucosamine 1-carboxyvinyltransferase
					2.000	7.500	2.7538	8.0758	
SGO_1414	-0.633	4.814	0.0183	0.0876	5.000	6.500	6.0383	6.5000	rexB; putative exonuclease RexB
					3.500	10.000	4.8191	10.7678	
SGO_1422	-0.947	6.844	0.0101	0.0413	13.000	29.500	15.6996	29.5000	hypothetical protein SGO_1422
					17.000	43.000	23.4070	46.3015	

Log_2 Ratios

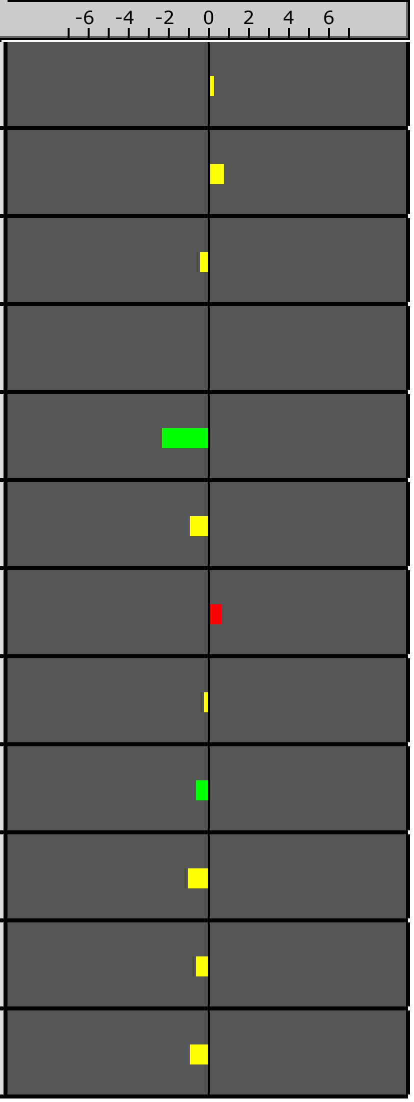

Show detected proteins only
Show all proteins
Filter by category:
ABC Transporter

Proteins found: 584

Test	Cutoff
***q*-Value**	**.005**
p-Value	

	Signif	Direction	Applies To
(red)	yes	+	ratios, bars
(yellow)	no	n/a	bars
(green)	yes	–	ratios, bars
(pink)	yes	+	*p*- , *q*-Values
(light green)	yes	–	*p*- , *q*-Values

Dot Plots Dot Plots

Protein	*SgPgFn vs SgFn* Log$_2$ Ratio	Log$_2$ Sum	*q*-Value	*p*-Value	Raw *SgPgFn*	Raw *SgFn*	Normalized *SgPgFn*	Normalized *SgFn*	Description	Log$_2$ Ratios
SGO_1426	1.167	13.835	0.0002	0.0001	4114.000	2120.500	4968.3146	2120.5000	eno; enolase	
					3729.500	2216.500	5135.0827	2386.6817		
SGO_1431	-0.585	8.282	0.0028	0.0076	51.500	102.000	62.1945	102.0000	EzrA; Septation ring formation regulator ezrA	
					45.000	79.000	61.9597	85.0656		
SGO_1432	0.058	6.137	0.0965	0.6723	19.500	16.000	23.5494	16.0000	gyrB; DNA gyrase, B subunit	
					9.500	16.500	13.0804	17.7669		
SGO_1434	-0.346	3.106	0.0544	0.3410	2.000	3.500	2.4153	3.5000	thiJ; 4-methyl-5(beta-hydroxyethyl)-thiazole monophosphate synthesis protein	
						2.500		2.6919		
SGO_1439	0.863	5.252	0.0027	0.0072	9.000	7.000	10.8689	7.0000	ftsX; cell division protein FtsX	
					10.000	6.000	13.7688	6.4607		
SGO_1440	-1.919	6.748	0.0000	0.0000	9.500	42.500	11.4728	42.5000	cell-division ATP-binding protein FtsE	
					8.000	39.500	11.0151	42.5328		
SGO_1441	-0.867	5.769	0.0186	0.0894	11.500	14.000	13.8881	14.0000	prfB; peptide chain release factor 2	
					4.500	19.000	6.1960	20.4588		
SGO_1446	-1.806	4.641	0.0016	0.0034	3.000	8.500	3.6230	8.5000	murF; UDP-N-acetylmuramoyl-tripeptide--D-alanyl-D-alanine ligase	
					1.500	10.000	2.0653	10.7678		
SGO_1447	-0.426	5.499	0.0000	0.0000	8.000	13.000	9.6613	13.0000	ddlA; D-Ala-D-Ala ligase	
					7.000	12.000	9.6382	12.9214		
SGO_1450	-0.537	4.565	0.0809	0.5462	2.000	6.000	2.4153	6.0000	hypothetical protein SGO_1450	
					6.000	6.500	8.2613	6.9991		
SGO_1451	1.618	6.827	0.0008	0.0008	33.500	17.000	40.4566	17.0000	frr; ribosome recycling factor	
					32.500	10.500	44.7487	11.3062		
SGO_1452	0.547	6.184	0.0004	0.0002	17.500	15.000	21.1341	15.0000	pyrH; uridylate kinase	
					16.000	13.500	22.0301	14.5365		

Log$_2$ Ratios axis: -6 -4 -2 0 2 4 6

Show detected proteins only
Show all proteins
Filter by category:
ABC Transporter

Proteins found: 584

Test	Cutoff
***q*-Value**	**.005**
p-Value	

Colour	Signif	Direction	Applies To
red	yes	+	ratios, bars
yellow	no	n/a	bars
green	yes	−	ratios, bars
pink	yes	+	*p*- , *q*-Values
light green	yes	−	*p*- , *q*-Values

Dot Plots Dot Plots

Protein	SgPgFn vs SgFn Log$_2$ Ratio	Log$_2$ Sum	*q*-Value	*p*-Value	Raw *SgPgFn*	Raw *SgFn*	Normalized *SgPgFn*	Normalized *SgFn*	Description
SGO_1453	2.151	4.446	0.0325	0.1818	10.000		12.0766		glycosyl transferase, family 8 SP1766
					5.500	2.000	7.5729	2.1536	
SGO_1455	1.180	8.929	0.0033	0.0095	117.500	72.500	141.9001	72.5000	rplA; ribosomal protein L1
					143.500	70.000	197.5826	75.3746	
SGO_1456	1.963	7.189	0.0006	0.0004	45.500	18.500	54.9485	18.5000	rplK; ribosomal protein L11
					44.000	11.000	60.5828	11.8446	
SGO_1458	-2.131	4.587	0.0298	0.1627	2.000	13.000	2.4153	13.0000	aha1; cation-transporting ATPase yfgQ
						8.000		8.6142	
SGO_1460	0.634	3.467	0.0102	0.0427	4.000	3.000	4.8306	3.0000	DNA translocase ftsK
						3.000		3.2303	
SGO_1463	0.702	4.869	0.0085	0.0325	8.000	7.000	9.6613	7.0000	peptidyl-prolyl cis-trans isomerase
					6.000	4.000	8.2613	4.3071	
SGO_1465	1.182	5.013	0.0011	0.0016	10.000	4.500	12.0766	4.5000	ABC transporter, ATP-binding protein SP0770
					7.500	5.000	10.3266	5.3839	
SGO_1469	0.653	6.587	0.0017	0.0038	22.500	19.000	27.1724	19.0000	glmU; UDP-N-acetylglucosamine pyrophosphorylase
					23.000	17.000	31.6683	18.3053	
SGO_1472	-1.454	3.841	0.0537	0.3353		8.500		8.5000	acetyltransferase, GNAT family
					1.500	3.500	2.0653	3.7687	
SGO_1486	-0.794	3.441	0.0286	0.1547	2.000	5.000	2.4153	5.0000	beta-galactosidase
					2.500		3.4422		
SGO_1530	0.299	6.897	0.0330	0.1849	26.000	32.500	31.3992	32.5000	methionine-tRNA ligase
					24.500	20.000	33.7336	21.5356	
SGO_1531	-0.038	4.984	0.1282	0.9234	5.000	9.500	6.0383	9.5000	xth; exodeoxyribonuclease III
					7.000	6.000	9.6382	6.4607	

Log$_2$ Ratios: -6 -4 -2 0 2 4 6

Show detected proteins only
Show all proteins
Filter by category:
ABC Transporter

Proteins found: 584

Test	Cutoff
q-Value	.005
p-Value	

Color	Signif	Direction	Applies To
red	yes	+	ratios, bars
yellow	no	n/a	bars
green	yes	−	ratios, bars
pink	yes	+	*p*- , *q*-Values
light green	yes	−	*p*- , *q*-Values

Dot Plots Dot Plots

Streptococcus gordonii

Hackett Laboratory UW

Protein	SgPgFn vs SgFn Log_2 Ratio	Log_2 Sum	*q*-Value	*p*-Value	Raw *SgPgFn*	Raw *SgFn*	Normalized *SgPgFn*	Normalized *SgFn*	Description
**SGO_1534**	0.089	4.573	0.0468	0.2795		8.000		8.0000	ArsC family
					6.000	7.000	8.2613	7.5375	
**SGO_1536**	0.124	4.493	0.0921	0.6369	4.000	6.500	4.8306	6.5000	conserved hypothetical protein TIGR00096
					5.000	4.000	6.8844	4.3071	
**SGO_1539**	0.463	5.379	0.0278	0.1490	8.000	10.500	9.6613	10.5000	tmk; thymidylate kinase
					10.500	6.500	14.4573	6.9991	
**SGO_1541**	-1.869	6.097	0.0086	0.0335	4.500	18.000	5.4345	18.0000	atpC; ATP synthase F1, epsilon subunit
					6.500	33.500	8.9497	36.0721	
**SGO_1542**	-1.641	9.594	0.0001	0.0000	73.000	290.500	88.1592	290.5000	atpD; ATP synthase F1, beta subunit
					72.500	273.500	99.8240	294.4992	
**SGO_1543**	-2.938	6.372	0.0011	0.0015	4.500	41.500	5.4345	41.5000	atpG; ATP synthase F1, gamma subunit
					3.000	29.500	4.1306	31.7650	
**SGO_1544**	-0.260	8.780	0.0054	0.0184	79.000	125.500	95.4052	125.5000	atpA; ATP synthase F1, alpha subunit
					76.000	106.000	104.6431	114.1386	
**SGO_1545**	-2.489	6.412	0.0006	0.0005	5.500	33.000	6.6421	33.0000	atpH; ATP synthase F1, delta subunit
					4.500	36.500	6.1960	39.3025	
**SGO_1546**	-0.007	6.360	0.1038	0.7314	16.500	30.000	19.9264	30.0000	atpF; ATP synthase F0, B subunit
					14.000	12.000	19.2764	12.9214	
**SGO_1550**	-0.330	6.657	0.0181	0.0857	16.500	25.000	19.9264	25.0000	glgP-1; glycogen phosphorylase
					18.000	29.000	24.7839	31.2266	
**SGO_1551**	-1.989	5.919	0.0006	0.0006	4.000	25.500	4.8306	25.5000	glgA; Glycogen synthase
					5.500	21.000	7.5729	22.6124	
**SGO_1552**	-0.218	6.928	0.0481	0.2886	27.500	35.500	33.2107	35.5000	glgD; glucose-1-phosphate adenylyltransferase, GlgD subunit
					17.000	27.500	23.4070	29.6114	

Log2 Ratios

Show detected proteins only
Show all proteins
Filter by category:
ABC Transporter

Proteins found: 584

Test	Cutoff
***q*-Value**	**.005**
p-Value	

Color	Signif	Direction	Applies To
red	yes	+	ratios, bars
yellow	no	n/a	bars
green	yes	–	ratios, bars
pink	yes	+	*p*- , *q*-Values
light green	yes	–	*p*- , *q*-Values

Dot Plots Dot Plots

Hendrickson *et al.*

Protein	SgPgFn vs SgFn Log$_2$ Ratio	Log$_2$ Sum	*q*-Value	*p*-Value	Raw SgPgFn	Raw SgFn	Normalized SgPgFn	Normalized SgFn	Description
SGO_1553	-0.779	7.072	0.0074	0.0272	19.500	50.500	23.5494	50.5000	glgC; glucose-1-phosphate adenylyltransferase
					18.500	32.500	25.4723	34.9953	
SGO_1554	-1.219	7.629	0.0011	0.0015	23.000	75.000	27.7762	75.0000	glgB; 1,4-alpha-glucan branching enzyme
					23.000	59.000	31.6683	63.5300	
SGO_1555	-0.598	10.074	0.0012	0.0019	163.500	329.500	197.4525	329.5000	ptsI; phosphoenolpyruvate-protein phosphotransferase
					168.500	296.000	232.0047	318.7267	
SGO_1556	0.516	11.147	0.0126	0.0555	467.500	515.500	564.5812	515.5000	phosphocarrier protein HPr
					561.500	384.500	773.1194	414.0217	
SGO_1558	-0.735	7.821	0.0049	0.0160	34.500	81.000	41.6643	81.0000	nrdE; ribonucleoside-diphosphate reductase large chain
					31.000	56.500	42.6834	60.8380	
SGO_1559	0.987	7.230	0.0001	0.0000	41.000	24.500	49.5141	24.5000	ribonucleoside-diphosphate reductase, beta subunit
					36.500	24.000	50.2562	25.8427	
SGO_1570	0.260	7.934	0.0345	0.1952	50.000	65.000	60.3830	65.0000	alaS; alanyl-tRNA synthetase
					52.500	43.500	72.2863	46.8399	
SGO_1572	-1.262	4.383	0.0507	0.3099		11.500		11.5000	proteinase maturation protein, putative
					2.500	5.500	3.4422	5.9223	
SGO_1574	0.145	8.046	0.0233	0.1197	55.500	67.500	67.0251	67.5000	pepF-1; oligoendopeptidase F
					52.000	54.000	71.5979	58.1461	
SGO_1580	1.302	3.866	0.0039	0.0118	3.500	2.000	4.2268	2.0000	PTS system, Lactose/Cellobiose specific IIB subunit
					4.500	2.000	6.1960	2.1536	
SGO_1587	-0.722	4.947	0.0075	0.0278	5.000	8.000	6.0383	8.0000	queA; S-adenosylmethionine:tRNA ribosyltransferase-isomerase
					4.000	10.500	5.5075	11.3062	
SGO_1591	-2.896	6.530	0.0001	0.0000	4.500	40.000	5.4345	40.0000	arcC; carbamate kinase
					4.000	38.500	5.5075	41.4560	

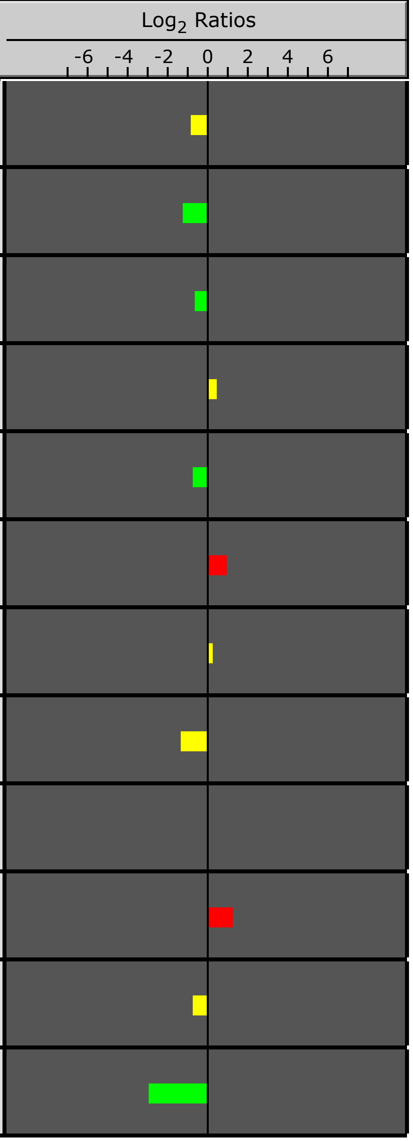

Show detected proteins only
Show all proteins
Filter by category:
ABC Transporter

Proteins found: 584

Test	Cutoff
q-Value	.005
p-Value	

Color	Signif	Direction	Applies To
red	yes	+	ratios, bars
yellow	no	n/a	bars
green	yes	−	ratios, bars
pink	yes	+	*p*- , *q*-Values
light green	yes	−	*p*- , *q*-Values

Dot Plots Dot Plots

Protein	SgPgFn vs SgFn Log_2 Ratio	Log_2 Sum	*q*-Value	*p*-Value	Raw *SgPgFn*	Raw *SgFn*	Normalized *SgPgFn*	Normalized *SgFn*	Description
SGO_1592	-2.763	9.962	0.0010	0.0014	58.500	379.500	70.6481	379.5000	arcB; ornithine carbamoyltransferase
					41.500	455.000	57.1406	489.9347	
SGO_1593	-2.148	9.308	0.0002	0.0001	47.500	270.000	57.3639	270.0000	arcA; arginine deiminase
					43.000	229.500	59.2059	247.1209	
SGO_1599	-1.777	9.514	0.0004	0.0002	73.000	298.500	88.1592	298.5000	sodA; manganese-dependent superoxide dismutase
					56.000	248.500	77.1054	267.5797	
SGO_1604	-1.529	3.806	0.0025	0.0063		6.000		6.0000	acyltransferase family protein
					1.500	5.500	2.0653	5.9223	
SGO_1605	0.408	2.373	0.0156	0.0719		1.500		1.5000	P-type ATPase, metal cation transport
					1.500	1.500	2.0653	1.6152	
SGO_1609	1.265	6.028	0.0008	0.0009	20.500	10.000	24.7570	10.0000	ATP-dependent RNA helicase, DEAD/DEAH box family
					15.500	8.500	21.3417	9.1526	
SGO_1617	-0.349	5.319	0.0224	0.1137	6.500	9.500	7.8498	9.5000	prfC; peptide chain release factor 3
					7.000	12.000	9.6382	12.9214	
SGO_1619	1.145	6.949	0.0009	0.0012	38.000	19.000	45.8911	19.0000	cation-transporting ATPase, E1-E2 family
					28.500	18.000	39.2411	19.3820	
SGO_1621	-1.424	3.946	0.0167	0.0775	2.000	6.000	2.4153	6.0000	HD domain protein
						6.500		6.9991	
SGO_1622	-1.203	5.334	0.0019	0.0044	4.000	15.000	4.8306	15.0000	Cof family protein
					5.500	12.000	7.5729	12.9214	
SGO_1623	-1.129	4.870	0.0131	0.0586	4.500	12.500	5.4345	12.5000	murM; MurM
						10.500		11.3062	
SGO_1624	-0.738	4.411	0.0501	0.3047		10.000		10.0000	murN; MurN protein
					3.500	6.000	4.8191	6.4607	

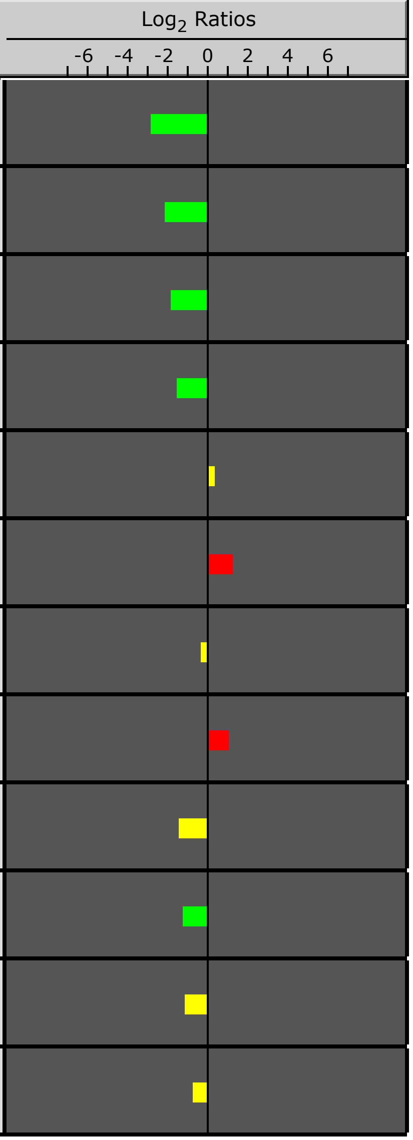

Show detected proteins only
Show all proteins
Filter by category:
ABC Transporter

Proteins found: 584

Test	Cutoff
q-Value	.005
p-Value	

	Signif	Direction	Applies To
red	yes	+	ratios, bars
yellow	no	n/a	bars
green	yes	−	ratios, bars
pink	yes	+	*p*- , *q*-Values
light green	yes	−	*p*- , *q*-Values

Dot Plots Dot Plots

Protein	SgPgFn vs SgFn Log$_2$ Ratio	Log$_2$ Sum	*q*-Value	*p*-Value	Raw *SgPgFn*	Raw *SgFn*	Normalized *SgPgFn*	Normalized *SgFn*	Description
**SGO_1625**	-1.628	6.398	0.0008	0.0009	10.500	30.000	12.6804	30.0000	acetoin utilization putative/CBS domain protein
					6.000	31.000	8.2613	33.3802	
**SGO_1626**	1.299	4.843	0.0101	0.0412	9.500		11.4728		branched-chain amino acid ABC transporter, ATP-binding protein
					9.000	4.500	12.3919	4.8455	
**SGO_1630**	1.649	7.739	0.0001	0.0000	68.500	27.500	82.7247	27.5000	branched-chain amino acid ABC transporter, amino acid-binding protein
					57.500	22.500	79.1707	24.2275	
**SGO_1632**	-0.833	7.137	0.0239	0.1245	12.500	36.500	15.0958	36.5000	clpP; ATP-dependent Clp protease, proteolytic subunit ClpP
					28.000	47.000	38.5527	50.6086	
**SGO_1633**	-0.442	7.533	0.0195	0.0946	38.500	60.000	46.4949	60.0000	upp; uracil phosphoribosyltransferase
					23.500	43.000	32.3567	46.3015	
**SGO_1648**	0.375	8.178	0.0014	0.0026	70.500	62.000	85.1401	62.0000	ppx1; inorganic pyrophosphatase, manganese-dependent
					57.000	59.500	78.4823	64.0684	
**SGO_1649**	-1.910	4.362	0.0027	0.0073	2.000	9.000	2.4153	9.0000	act; pyruvate formate-lyase-activating enzyme
						8.500		9.1526	
**SGO_1652**	0.811	5.633	0.0010	0.0012	13.000	10.000	15.6996	10.0000	intracellular glycosyl hydrolase
					11.500	7.500	15.8341	8.0758	
**SGO_1653**	-0.288	6.406	0.0813	0.5500	10.500	20.500	12.6804	20.5000	trehalose PTS enzyme II
					19.500	23.000	26.8492	24.7659	
**SGO_1666**	-3.259	6.176	0.0001	0.0000	3.500	33.000	4.2268	33.0000	trkA; potassium uptake protein, Trk family
					2.000	30.000	2.7538	32.3034	
**SGO_1669**	0.166	5.693	0.0746	0.4987	11.000	15.000	13.2843	15.0000	ribosomal large subunit pseudouridine synthase B
					10.000	9.000	13.7688	9.6910	
**SGO_1675**	-1.069	5.244	0.0031	0.0088	4.000	11.500	4.8306	11.5000	HAM1 protein-like protein
					5.500	13.000	7.5729	13.9981	

Log$_2$ Ratios: -6 -4 -2 0 2 4 6

Show detected proteins only
Show all proteins
Filter by category:
ABC Transporter

Proteins found: 584

Test	Cutoff
***q*-Value**	**.005**
p-Value	

	Signif	Direction	Applies To
red	yes	+	ratios, bars
yellow	no	n/a	bars
green	yes	−	ratios, bars
pink	yes	+	*p*- , *q*-Values
light green	yes	−	*p*- , *q*-Values

Dot Plots Dot Plots

Protein	SgPgFn vs SgFn Log$_2$ Ratio	Log$_2$ Sum	*q*-Value	*p*-Value	Raw *SgPgFn*	Raw *SgFn*	Normalized *SgPgFn*	Normalized *SgFn*	Description
SGO_1676	0.048	4.668	0.0972	0.6784	4.500	6.500	5.4345	6.5000	murI; glutamate racemase
					5.500	5.500	7.5729	5.9223	
SGO_1678	-1.774	4.577	0.0233	0.1208	2.500	9.000	3.0192	9.0000	lysA; diaminopimelate decarboxylase
						11.000		11.8446	
SGO_1679	0.929	8.341	0.0012	0.0019	82.000	60.000	99.0281	60.0000	phosphotransferase system enzyme II
					82.500	48.000	113.5928	51.6854	
SGO_1681	0.855	7.626	0.0043	0.0131	46.000	30.500	55.5524	30.5000	PTS system, mannose/fructose/sorbose family, IID component
					52.000	37.000	71.5979	39.8408	
SGO_1683	0.189	7.196	0.0866	0.5936	34.500	44.500	41.6643	44.5000	serS; seryl-tRNA synthetase
					25.500	23.500	35.1105	25.3043	
SGO_1685	1.918	7.432	0.0017	0.0038	65.500	17.500	79.1018	17.5000	putative peroxidase / antioxidase
					42.000	17.000	57.8291	18.3053	
SGO_1687	0.448	6.848	0.0070	0.0253	28.000	28.000	33.8145	28.0000	accA; acetyl-CoA carboxylase, carboxyl transferase, alpha subunit
					23.500	19.500	32.3567	20.9972	
SGO_1688	-1.487	5.254	0.0006	0.0005	5.000	14.000	6.0383	14.0000	accD; acetyl-CoA carboxylase, carboxyl transferase, beta subunit
					3.000	13.000	4.1306	13.9981	
SGO_1689	-0.648	7.080	0.0015	0.0031	22.500	38.500	27.1724	38.5000	accC; acetyl-CoA carboxylase, biotin carboxylase
					18.500	41.000	25.4723	44.1480	
SGO_1690	-0.770	4.468	0.0601	0.3863	4.000	6.000	4.8306	6.0000	fabZ; beta-hydroxyacyl-(acyl-carrier-protein) dehydratase FabZ
						10.500		11.3062	
SGO_1691	0.867	6.712	0.0097	0.0386	24.000	24.500	28.9838	24.5000	accB; acetyl-CoA carboxylase, biotin carboxyl carrier protein
					27.500	12.500	37.8643	13.4597	
SGO_1692	-1.129	9.280	0.0009	0.0011	78.000	197.500	94.1975	197.5000	3-oxoacyl-[acyl-carrier-protein] synthase
					73.000	213.000	100.5124	229.3540	

Log$_2$ Ratios: -6 -4 -2 0 2 4 6

Show detected proteins only
Show all proteins
Filter by category:
ABC Transporter

Proteins found: 584

Test	Cutoff
q-Value	.005
p-Value	

Color	Signif	Direction	Applies To
red	yes	+	ratios, bars
yellow	no	n/a	bars
green	yes	−	ratios, bars
pink	yes	+	*p*- , *q*-Values
light green	yes	−	*p*- , *q*-Values

Dot Plots Dot Plots

Protein	*SgPgFn vs SgFn* Log₂ Ratio	Log₂ Sum	*q*-Value	*p*-Value	Raw *SgPgFn*	Raw *SgFn*	Normalized *SgPgFn*	Normalized *SgFn*	Description
SGO_1693	-0.824	6.332	0.0053	0.0180	15.500	25.000	18.7187	25.0000	fabG; 3-oxoacyl-(acyl-carrier-protein) reductase
					8.000	24.000	11.0151	25.8427	
SGO_1694	-0.122	5.819	0.1009	0.7086	14.000	12.000	16.9072	12.0000	fabD; malonyl CoA-acyl carrier protein transacylase
					7.500	16.000	10.3266	17.2285	
SGO_1695	0.295	8.393	0.0233	0.1203	88.500	71.000	106.8779	71.0000	enoyl-acyl carrier protein(ACP) reductase
					57.500	73.500	79.1707	79.1433	
SGO_1699	-1.210	4.762	0.0048	0.0154		11.000		11.0000	transcriptional regulator, MarR family
					3.500	10.500	4.8191	11.3062	
SGO_1700	-4.105	7.012	0.0167	0.0776	3.000	55.500	3.6230	55.5000	enoyl-CoA hydratase/isomerase family protein
						65.000		69.9907	
SGO_1701	-0.419	6.844	0.0121	0.0527	17.500	30.000	21.1341	30.0000	aspartate kinase
					20.500	33.000	28.2261	35.5337	
SGO_1708	0.383	7.250	0.0103	0.0432	32.500	36.500	39.2490	36.5000	amiF; Oligopeptide transport ATP-binding protein amiF
					34.000	27.500	46.8140	29.6114	
SGO_1709	1.303	7.267	0.0024	0.0059	39.000	20.000	47.0988	20.0000	amiE; Oligopeptide transport ATP-binding protein
					45.500	22.500	62.6481	24.2275	
SGO_1711	1.455	6.248	0.0017	0.0038	20.000	10.500	24.1532	10.5000	hppB; Oligopeptide transport system permease
					23.000	9.000	31.6683	9.6910	
SGO_1712	1.154	9.163	0.0016	0.0033	167.000	111.000	201.6793	111.0000	hppA; oligopeptide-binding lipoprotein
					138.500	65.000	190.6982	69.9907	
SGO_1713	0.072	8.945	0.0937	0.6499	101.500	137.500	122.5775	137.5000	hppG; oligopeptide-binding lipoprotein
					93.500	96.500	128.7385	103.9092	
SGO_1715	-0.056	7.577	0.0676	0.4455	36.500	51.000	44.0796	51.0000	hppH; oligopeptide-binding lipoprotein
					36.000	43.000	49.5678	46.3015	

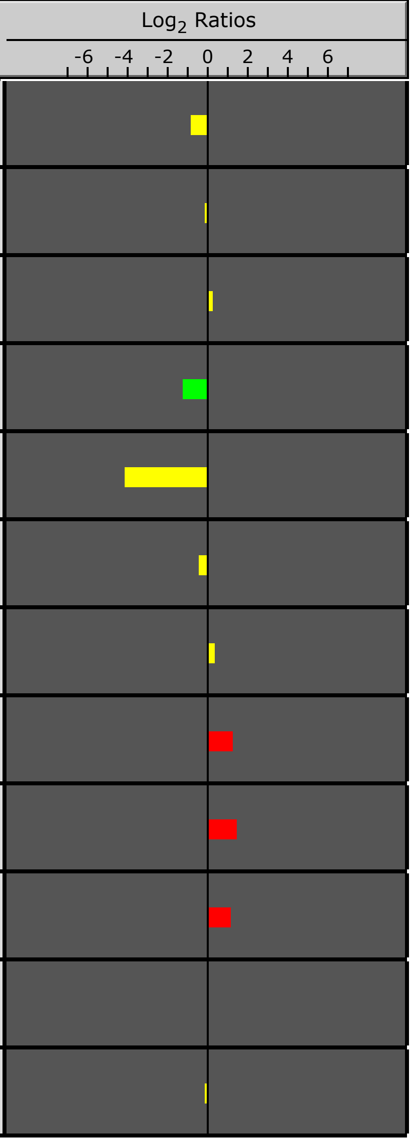

Show detected proteins only
Show all proteins
Filter by category:
ABC Transporter

Proteins found: 584

Test	Cutoff
q-Value	.005
p-Value	

	Signif	Direction	Applies To
red	yes	+	ratios, bars
yellow	no	n/a	bars
green	yes	−	ratios, bars
pink	yes	+	*p*- , *q*-Values
light green	yes	−	*p*- , *q*-Values

Dot Plots Dot Plots

Protein	SgPgFn vs SgFn				Raw		Normalized		Description
	Log_2 Ratio	Log_2 Sum	*q*-Value	*p*-Value	*SgPgFn*	*SgFn*	*SgPgFn*	*SgFn*	
SGO_1716	0.076	8.001	0.0978	0.6839	70.500	70.000	85.1401	70.0000	oligopeptide binding protein
					35.000	49.000	48.1909	52.7622	
SGO_1718	0.280	5.976	0.0608	0.3913	10.500	11.000	12.6804	11.0000	sufB-1; FeS assembly protein SufB
					16.000	16.000	22.0301	17.2285	
SGO_1721	0.020	6.386	0.1345	0.9787	17.000	24.000	20.5302	24.0000	sufD; FeS assembly protein SufD
					15.500	16.500	21.3417	17.7669	
SGO_1722	-0.613	5.854	0.0224	0.1139	6.500	18.000	7.8498	18.0000	sufC; FeS assembly ATPase SufC
					11.500	15.000	15.8341	16.1517	
SGO_1727	0.683	5.661	0.0048	0.0156	12.000	11.500	14.4919	11.5000	amino acid ABC transporter, amino acid-binding/permease protein
					12.000	7.500	16.5226	8.0758	
SGO_1728	-0.263	5.786	0.0020	0.0047	10.500	14.500	12.6804	14.5000	glnQ; glutamine ABC transporter ATP-binding protein
					9.000	14.500	12.3919	15.6133	
SGO_1729	-3.517	8.099	0.0025	0.0064	11.500	97.000	13.8881	97.0000	hypothetical protein SGO_1729
					6.000	144.000	8.2613	155.0562	
SGO_1730	-1.314	8.639	0.0024	0.0058	45.000	163.500	54.3447	163.5000	SPFH domain/Band 7 family
					43.000	113.000	59.2059	121.6761	
SGO_1731	1.960	4.510	0.0006	0.0004	7.000	2.000	8.4536	2.0000	DNA-binding response regulator
					7.000	2.500	9.6382	2.6919	
SGO_1735	-0.100	8.136	0.0649	0.4266	59.000	82.500	71.2520	82.5000	hypothetical protein SGO_1735
					46.500	59.000	64.0250	63.5300	
SGO_1736	-1.343	4.908	0.0394	0.2279	4.000	15.500	4.8306	15.5000	alkaline shock protein
						9.000		9.6910	
SGO_1745	1.201	11.373	0.0022	0.0053	665.500	422.000	803.6980	422.0000	fba; fructose-1,6-bisphosphate aldolase, class II
					762.000	351.000	1049.1843	377.9496	

Show detected proteins only
Show all proteins
Filter by category:
ABC Transporter

Proteins found: 584

Test	Cutoff
q-Value	.005
p-Value	

	Signif	Direction	Applies To
red	yes	+	ratios, bars
yellow	no	n/a	bars
green	yes	−	ratios, bars
pink	yes	+	*p*- , *q*-Values
light green	yes	−	*p*- , *q*-Values

Dot Plots Dot Plots

Streptococcus gordonii

Protein	SgPgFn vs SgFn Log$_2$ Ratio	Log$_2$ Sum	*q*-Value	*p*-Value	Raw *SgPgFn*	Raw *SgFn*	Normalized *SgPgFn*	Normalized *SgFn*	Description
SGO_1747	-0.419	3.082			3.000		3.6230		hypothetical protein SGO_1747
						4.500		4.8455	
SGO_1748	0.649	3.985	0.0510	0.3132	6.000		7.2460		pyrG; CTP synthase
					3.500	3.500	4.8191	3.7687	
SGO_1749	-0.950	5.665	0.0052	0.0173	8.000	19.500	9.6613	19.5000	manA; mannose-6-phosphate isomerase, class I
					5.500	13.000	7.5729	13.9981	
SGO_1755	1.260	5.573	0.0006	0.0006	13.000	7.000	15.6996	7.0000	scrK; fructokinase
					13.000	6.500	17.8995	6.9991	
SGO_1757	0.309	7.076	0.0451	0.2675	28.000	38.000	33.8145	38.0000	glmS; glucosamine--fructose-6-phosphate aminotransferase
					29.000	21.500	39.9296	23.1508	
SGO_1763	0.682	6.589	0.0020	0.0048	25.000	21.000	30.1915	21.0000	ABC transporter, substrate-binding protein SP0092
					21.000	15.000	28.9145	16.1517	
SGO_1774	-1.227	5.684	0.0015	0.0028	5.000	18.500	6.0383	18.5000	alcohol dehydrogenase, zinc-containing
					7.000	16.000	9.6382	17.2285	
SGO_1784	0.459	7.530	0.0023	0.0057	44.000	42.500	53.1371	42.5000	leuS; leucyl-tRNA synthetase
					39.000	33.000	53.6984	35.5337	
SGO_1799	-1.307	8.362	0.0004	0.0002	44.000	117.500	53.1371	117.5000	endopeptidase O
					30.500	108.000	41.9949	116.2922	
SGO_1802	2.760	6.705	0.0014	0.0025	43.000	9.000	51.9294	9.0000	Metal ABC transporter substrate-binding lipoprotein precursor
					28.000	4.500	38.5527	4.8455	
SGO_1803	0.366	6.034	0.0255	0.1343	12.500	15.500	15.0958	15.5000	tpx; thioredoxin peroxidase
					16.000	12.000	22.0301	12.9214	
SGO_1805	2.406	4.208	0.0435	0.2567	9.500	1.500	11.4728	1.5000	hutU; urocanate hydratase
					4.000		5.5075		

Log$_2$ Ratios: -6 -4 -2 0 2 4 6

Show detected proteins only
Show all proteins
Filter by category:
ABC Transporter

Proteins found: 584

Test	Cutoff
q-Value	.005
p-Value	

	Signif	Direction	Applies To
red	yes	+	ratios, bars
yellow	no	n/a	bars
green	yes	−	ratios, bars
pink	yes	+	*p*- , *q*-Values
light green	yes	−	*p*- , *q*-Values

Dot Plots Dot Plots

Protein	*SgPgFn vs SgFn* Log$_2$ Ratio	Log$_2$ Sum	*q*-Value	*p*-Value	Raw *SgPgFn*	Raw *SgFn*	Normalized *SgPgFn*	Normalized *SgFn*	Description	Log$_2$ Ratios (-6 -4 -2 0 2 4 6)
**SGO_1808**	0.110	4.382	0.1057	0.7490	4.500		5.4345		fhs-2; formate--tetrahydrofolate ligase	
					6.500	6.000	8.9497	6.4607		
**SGO_1811**	-1.453	3.363							hutH; histidine ammonia-lyase	
					2.000	7.000	2.7538	7.5375		
**SGO_1822**	-2.042	5.490	0.0212	0.1059	4.000	17.500	4.8306	17.5000	relA; GTP diphosphokinase	
						21.000		22.6124		
**SGO_1824**	-0.145	4.892	0.0667	0.4389	6.500	6.500	7.8498	6.5000	prmA; ribosomal protein L11 methyltransferase	
					4.500	8.500	6.1960	9.1526		
**SGO_1828**	-0.466	3.724	0.0639	0.4192		6.000		6.0000	ATPase, AAA family	
					2.500	3.500	3.4422	3.7687		
**SGO_1834**	-1.950	7.992	0.0015	0.0030	22.500	116.000	27.1724	116.0000	hypothetical protein SGO_1834	
					18.000	80.500	24.7839	86.6807		
**SGO_1835**	-0.086	4.206				9.500		9.5000	hypothetical protein SGO_1835	
					6.500		8.9497			
**SGO_1843**	-0.060	7.319	0.0775	0.5213	29.500	41.500	35.6260	41.5000	pepS; aminopeptidase PepS	
					31.000	37.000	42.6834	39.8408		
**SGO_1844**	-0.633	4.302	0.0107	0.0454	2.500	6.500	3.0192	6.5000	cbxX/cfqX family protein	
					3.500	5.000	4.8191	5.3839		
**SGO_1847**	-1.233	4.620	0.0507	0.3100		7.000		7.0000	polC; DNA polymerase III, alpha subunit, Gram-positive type	
					3.000	12.500	4.1306	13.4597		
**SGO_1851**	0.024	7.987	0.1208	0.8624	55.500	69.500	67.0251	69.5000	proS; prolyl-tRNA synthetase	
					44.000	52.500	60.5828	56.5309		
**SGO_1854**	0.638	4.952	0.0188	0.0905	6.000	5.000	7.2460	5.0000	uppS; undecaprenyl diphosphate synthase	
					8.500	6.500	11.7035	6.9991		

- Show detected proteins only
- Show all proteins
- Filter by category: ABC Transporter

Proteins found: 584

Test	Cutoff
***q*-Value**	**.005**
p-Value	

Color	Signif	Direction	Applies To
red	yes	+	ratios, bars
yellow	no	n/a	bars
green	yes	−	ratios, bars
pink	yes	+	*p*- , *q*-Values
light green	yes	−	*p*- , *q*-Values

Dot Plots Dot Plots

Protein	SgPgFn vs SgFn Log$_2$ Ratio	Log$_2$ Sum	*q*-Value	*p*-Value	Raw *SgPgFn*	Raw *SgFn*	Normalized *SgPgFn*	Normalized *SgFn*	Description
SGO_1856	-3.449	7.784	0.0046	0.0145	8.500	132.500	10.2651	132.5000	ATP-dependent proteinase ATP-binding chain
					5.500	65.000	7.5729	69.9907	
SGO_1860	-1.135	7.389	0.0101	0.0407	19.500	74.500	23.5494	74.5000	5'-nucleotidase, lipoprotein e(P4) family
					20.000	39.000	27.5376	41.9944	
SGO_1862	-1.712	7.273	0.0007	0.0007	14.500	54.500	17.5111	54.5000	alkaline shock protein
					13.500	59.500	18.5879	64.0684	
SGO_1863	0.982	7.147	0.0012	0.0019	40.000	27.500	48.3064	27.5000	efp; Elongation factor P (EF-P)
					33.000	19.000	45.4371	20.4588	
SGO_1864	-1.411	7.231	0.0014	0.0026	22.500	53.000	27.1724	53.0000	X-Pro aminopeptidase
					11.000	51.000	15.1457	54.9158	
SGO_1865	-1.295	5.738	0.0008	0.0010	6.500	17.500	7.8498	17.5000	uvrA; excinuclease ABC, A subunit
					5.500	19.000	7.5729	20.4588	
SGO_1867	2.857	5.997	0.0015	0.0030	27.000	3.000	32.6068	3.0000	hypothetical protein SGO_1867
					17.000	4.500	23.4070	4.8455	
SGO_1879	0.347	6.937	0.0108	0.0460	27.500	23.500	33.2107	23.5000	rpsR; ribosomal protein S18
					25.500	28.500	35.1105	30.6882	
SGO_1880	0.803	7.049	0.0001	0.0000	35.500	24.000	42.8719	24.0000	ssb-1; single-strand binding protein
					30.000	22.500	41.3065	24.2275	
SGO_1881	0.957	8.379	0.0031	0.0087	95.500	44.500	115.3316	44.5000	rpsF; ribosomal protein S6
					74.500	65.500	102.5777	70.5291	
SGO_1882	-0.698	4.773	0.0085	0.0331	4.000	10.000	4.8306	10.0000	folE; GTP cyclohydrolase I
					4.000	6.500	5.5075	6.9991	
SGO_1885	-2.954	11.002	0.0000	0.0000	94.500	909.500	114.1239	909.5000	groL; 60 kDa chaperonin/groEL protein
					87.500	842.500	120.4772	907.1867	

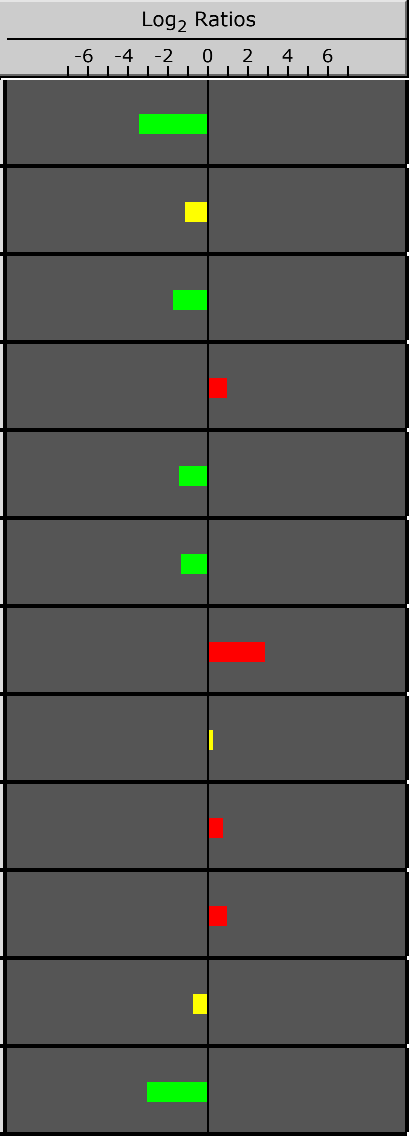

Show detected proteins only
Show all proteins
Filter by category:
ABC Transporter

Proteins found: 584

Test	Cutoff
q-Value	.005
p-Value	

	Signif	Direction	Applies To
(red)	yes	+	ratios, bars
(yellow)	no	n/a	bars
(green)	yes	−	ratios, bars
(pink)	yes	+	*p*- , *q*-Values
(light green)	yes	−	

Dot Plots Dot Plots

Protein	SgPgFn vs SgFn Log$_2$ Ratio	Log$_2$ Sum	*q*-Value	*p*-Value	Raw *SgPgFn*	Raw *SgFn*	Normalized *SgPgFn*	Normalized *SgFn*	Description
SGO_1886	-2.507	6.104	0.0097	0.0386	3.000	17.500	3.6230	17.5000	groES; chaperonin, 10 kDa
					4.500	38.500	6.1960	41.4560	
SGO_1892	-3.155	7.166	0.0025	0.0065	7.500	79.000	9.0575	79.0000	PTS system, fructose(mannose)-specific IIB
					4.000	46.500	5.5075	50.0702	
SGO_1898	-2.385	8.189	0.0006	0.0006	20.500	111.500	24.7570	111.5000	glutamyl aminopeptidase
					16.000	124.000	22.0301	133.5207	
SGO_1901	0.852	5.322	0.0102	0.0424	10.000	10.000	12.0766	10.0000	tRNA binding domain
					9.500	4.500	13.0804	4.8455	
SGO_1903	-0.428	7.280	0.0167	0.0781	24.000	51.000	28.9838	51.0000	ATP-dependent Zn protease
					27.000	35.500	37.1758	38.2257	
SGO_1914	-0.677	3.531	0.0094	0.0372		4.500		4.5000	folP; dihydropteroate synthase
					2.000	4.000	2.7538	4.3071	
SGO_1916	0.227	7.371	0.0018	0.0041	38.000	37.500	45.8911	37.5000	ackA; acetate kinase
					31.500	36.000	43.3718	38.7641	
SGO_1917	0.237	4.345	0.0275	0.1467	4.500	4.000	5.4345	4.0000	hypothetical protein SGO_1917
					4.000	5.000	5.5075	5.3839	
SGO_1926	0.411	9.385	0.0008	0.0009	153.500	146.500	185.3759	146.5000	rpoC; DNA-directed RNA polymerase, beta chain
					142.500	130.500	196.2057	140.5197	
SGO_1927	0.722	9.162	0.0101	0.0410	135.500	141.000	163.6380	141.0000	rpoB; DNA-directed RNA polymerase, beta subunit
					136.500	74.500	187.9444	80.2201	
SGO_1929	0.446	7.843	0.0035	0.0103	50.500	48.000	60.9868	48.0000	tyrS; tyrosyl-tRNA synthetase
					52.000	45.500	71.5979	48.9935	
SGO_1936	0.686	5.528	0.0033	0.0095	10.500	9.000	12.6804	9.0000	adcA; metal-binding (Mn) permease precursor, lipoprotein
					11.500	8.000	15.8341	8.6142	

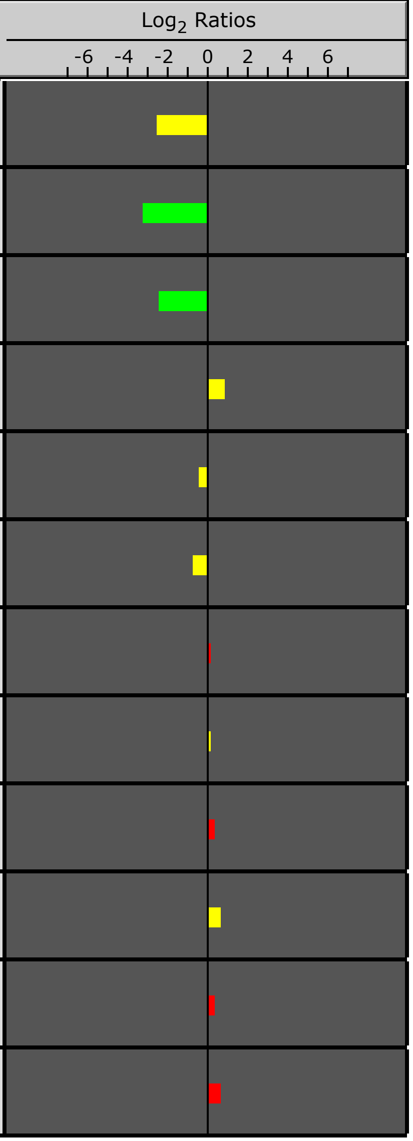

Show detected proteins only
Show all proteins
Filter by category:
ABC Transporter

Proteins found: 584

Test	Cutoff
q-Value	.005
p-Value	

Color	Signif	Direction	Applies To
red	yes	+	ratios, bars
yellow	no	n/a	bars
green	yes	–	ratios, bars
pink	yes	+	*p*- , *q*-Values
light green	yes	–	*p*- , *q*-Values

Dot Plots Dot Plots

Hendrickson *et al.*

Protein	SgPgFn vs SgFn Log$_2$ Ratio	Log$_2$ Sum	q-Value	p-Value	Raw SgPgFn	Raw SgFn	Normalized SgPgFn	Normalized SgFn	Description
SGO_1958	2.689	8.543	0.0006	0.0004	122.500	27.500	147.9384	27.5000	rplQ; ribosomal protein L17
					127.000	21.000	174.8641	22.6124	
SGO_1959	0.308	8.205	0.0048	0.0153	66.000	61.000	79.7056	61.0000	rpoA; DNA-directed RNA polymerase, alpha subunit
					60.500	66.000	83.3014	71.0674	
SGO_1960	0.789	7.066	0.0045	0.0141	36.000	30.500	43.4758	30.5000	rpsK; ribosomal protein S11
					29.500	18.000	40.6180	19.3820	
SGO_1961	1.553	7.536	0.0006	0.0004	54.500	27.000	65.8175	27.0000	rpsM; ribosomal protein S13p/S18e
					52.500	19.000	72.2863	20.4588	
SGO_1964	0.825	7.728	0.0045	0.0139	52.500	47.500	63.4022	47.5000	adk; Adenylate kinase (ATP-AMP transphosphorylase)
					51.500	28.000	70.9094	30.1498	
SGO_1966	0.567	8.065	0.0299	0.1641	57.500	75.000	69.4405	75.0000	rplO; ribosomal protein L15
					63.000	34.000	86.7436	36.6105	
SGO_1967	1.214	6.873	0.0039	0.0118	29.500	13.000	35.6260	13.0000	50S ribosomal protein L30 -related protein
					33.000	21.500	45.4371	23.1508	
SGO_1968	0.822	8.659	0.0017	0.0037	110.500	84.000	133.4465	84.0000	rpsE; ribosomal protein S5
					90.000	58.500	123.9194	62.9916	
SGO_1969	1.746	8.939	0.0006	0.0005	156.500	71.000	188.9988	71.0000	rplR; ribosomal protein L18
					135.500	41.000	186.5676	44.1480	
SGO_1970	1.504	8.491	0.0022	0.0051	126.500	52.000	152.7690	52.0000	BL10; 50S ribosomal protein L6
					82.500	38.500	113.5928	41.4560	
SGO_1971	2.151	9.780	0.0014	0.0024	339.500	79.000	410.0007	79.0000	rpsH; ribosomal protein S8
					224.500	75.500	309.1101	81.2969	
SGO_1973	1.406	8.721	0.0009	0.0011	137.500	60.500	166.0533	60.5000	BL6; 50S ribosomal protein L5
					102.000	51.000	140.4420	54.9158	

Log$_2$ Ratios: -6 -4 -2 0 2 4 6

Show detected proteins only
Show all proteins
Filter by category:
ABC Transporter

Proteins found: 584

Test	Cutoff
q-Value	.005
p-Value	

	Signif	Direction	Applies To
red	yes	+	ratios, bars
yellow	no	n/a	bars
green	yes	–	ratios, bars
pink	yes	+	p- , q-Values
light green	yes	–	p- , q-Values

Dot Plots Dot Plots

Hendrickson *et al.*

Protein	SgPgFn vs SgFn Log$_2$ Ratio	Log$_2$ Sum	*q*-Value	*p*-Value	Raw *SgPgFn*	Raw *SgFn*	Normalized *SgPgFn*	Normalized *SgFn*	Description
SGO_1974	1.717	7.964	0.0011	0.0016	71.500	34.500	86.3477	34.5000	rplX; ribosomal protein L24
					76.000	22.500	104.6431	24.2275	
SGO_1975	1.428	7.967	0.0013	0.0021	69.000	40.500	83.3286	40.5000	rplN; ribosomal protein L14
					71.500	26.000	98.4471	27.9963	
SGO_1976	1.596	7.102	0.0038	0.0111	51.500	14.500	62.1945	14.5000	BS16; 30S ribosomal protein
					30.000	18.000	41.3065	19.3820	
SGO_1977	0.837	5.125	0.0457	0.2725	9.000	7.500	10.8689	7.5000	rpmC; ribosomal protein L29
					12.000		16.5226		
SGO_1978	1.151	7.242	0.0002	0.0001	42.500	25.000	51.3256	25.0000	rplP; ribosomal protein L16
					38.500	20.500	53.0100	22.0740	
SGO_1979	0.136	8.699	0.0521	0.3237	82.500	88.500	99.6320	88.5000	rpsC; ribosomal protein S3
					85.500	102.000	117.7234	109.8315	
SGO_1980	1.464	8.550	0.0005	0.0003	108.000	53.500	130.4273	53.5000	rplV; ribosomal protein L22
					105.000	43.000	144.5726	46.3015	
SGO_1981	0.314	8.180	0.0049	0.0163	62.000	64.000	74.8749	64.0000	rpsS; ribosomal protein S19
					62.500	60.500	86.0551	65.1452	
SGO_1982	0.388	8.167	0.0310	0.1712	61.000	78.500	73.6673	78.5000	rplB; ribosomal protein L2
					63.500	44.500	87.4320	47.9167	
SGO_1983	1.224	7.256	0.0069	0.0248	38.000	14.500	45.8911	14.5000	rplW; ribosomal protein L23
					42.500	31.500	58.5175	33.9186	
SGO_1984	1.096	9.013	0.0008	0.0008	136.000	81.500	164.2418	81.5000	rplD; ribosomal protein L4/L1 family
					136.500	77.000	187.9444	82.9120	
SGO_1985	0.693	9.127	0.0024	0.0061	139.000	123.000	167.8648	123.0000	rplC; ribosomal protein L3
					128.000	85.500	176.2409	92.0646	

Log$_2$ Ratios: -6 -4 -2 0 2 4 6

Show detected proteins only
Show all proteins
Filter by category:
ABC Transporter

Proteins found: 584

Test	Cutoff
***q*-Value**	**.005**
p-Value	

	Signif	Direction	Applies To
red	yes	+	ratios, bars
yellow	no	n/a	bars
green	yes	−	ratios, bars
pink	yes	+	*p*- , *q*-Values
light green	yes	−	*p*- , *q*-Values

Dot Plots | Dot Plots

Hendrickson *et al.*

Protein	SgPgFn vs SgFn Log$_2$ Ratio	Log$_2$ Sum	*q*-Value	*p*-Value	Raw *SgPgFn*	Raw *SgFn*	Normalized *SgPgFn*	Normalized *SgFn*	Description
SGO_1986	1.323	6.986	0.0002	0.0001	38.500	19.000	46.4949	19.0000	rpsJ; ribosomal protein S10
					32.000	16.000	44.0602	17.2285	
SGO_1989	0.821	8.880	0.0010	0.0013	117.500	89.500	141.9001	89.5000	purA; adenylosuccinate synthetase
					115.500	75.000	159.0299	80.7585	
SGO_1990	-2.509	5.718	0.0181	0.0858	3.500	21.500	4.2268	21.5000	glutamate--cysteine ligase, putative/amino acid ligase, putative
						25.000		26.9195	
SGO_1993	0.841	5.299	0.0076	0.0285	8.500	7.500	10.2651	7.5000	possible transcriptional regulator
					11.000	6.000	15.1457	6.4607	
SGO_1995	-0.770	4.468	0.0601	0.3863	4.000	6.000	4.8306	6.0000	MutT/nudix family protein
						10.500		11.3062	
SGO_1998	-0.791	5.739	0.0021	0.0049	9.500	17.000	11.4728	17.0000	clpB; ATP-dependent Clp proteinase, ATP-binding chain
					6.000	15.500	8.2613	16.6901	
SGO_2000	0.356	10.040	0.0060	0.0208	260.000	247.500	313.9917	247.5000	tsf; translation elongation factor Ts
					201.000	199.000	276.7534	214.2791	
SGO_2001	0.316	9.794	0.0097	0.0386	191.000	180.000	230.6631	180.0000	rpsB; ribosomal protein S2
					189.500	200.500	260.9192	215.8943	
SGO_2005	1.753	4.401	0.0290	0.1574	9.000		10.8689		LPXTG cell wall surface protein
					5.500	2.500	7.5729	2.6919	
SGO_2007	-0.365	6.308	0.0012	0.0019	15.000	22.500	18.1149	22.5000	nusG; transcription termination/antitermination factor NusG
					12.000	20.500	16.5226	22.0740	
SGO_2033	-1.704	6.817	0.0004	0.0002	10.000	41.000	12.0766	41.0000	nrdD; ribonucleoside-triphosphate reductase
					10.500	42.000	14.4573	45.2247	
SGO_2042	1.805	7.874	0.0017	0.0037	83.500	35.000	100.8396	35.0000	Bacterial protein of unknown function (DUF965) superfamily
					58.000	17.500	79.8592	18.8436	

Log$_2$ Ratios: -6 -4 -2 0 2 4 6

Show detected proteins only
Show all proteins
Filter by category:
ABC Transporter

Proteins found: 584

Test	Cutoff
***q*-Value**	**.005**
p-Value	

	Signif	Direction	Applies To
red	yes	+	ratios, bars
yellow	no	n/a	bars
green	yes	−	ratios, bars
pink	yes	+	*p*- , *q*-Values
light green	yes	−	*p*- , *q*-Values

Dot Plots Dot Plots

Protein	SgPgFn vs SgFn Log_2 Ratio	Log_2 Sum	*q*-Value	*p*-Value	Raw *SgPgFn*	Raw *SgFn*	Normalized *SgPgFn*	Normalized *SgFn*	Description
SGO_2045	3.243	9.321	0.0003	0.0001	254.000	32.000	306.7457	32.0000	recA; recA protein
					197.500	27.000	271.9343	29.0730	
SGO_2046	-1.090	4.310	0.0010	0.0012	3.000	7.000	3.6230	7.0000	cinA; competence induced protein
					2.000	6.000	2.7538	6.4607	
SGO_2053	-0.957	4.735	0.0014	0.0024	3.500	9.500	4.2268	9.5000	DNA mismatch repair protein hexB
					3.500	7.500	4.8191	8.0758	
SGO_2056	-1.765	5.849	0.0027	0.0072	3.500	19.000	4.2268	19.0000	mutS; DNA mismatch repair protein MutS
					7.000	23.000	9.6382	24.7659	
SGO_2058	0.054	9.316	0.0612	0.3967	143.000	152.500	172.6954	152.5000	argS; arginyl-tRNA synthetase
					110.500	148.500	152.1455	159.9018	
SGO_2060	-0.696	6.704	0.0031	0.0087	17.500	29.000	21.1341	29.0000	aspS-1; aspartyl-tRNA synthetase
					13.500	33.000	18.5879	35.5337	
SGO_2062	-0.386	8.038	0.0077	0.0286	51.000	80.500	61.5907	80.5000	hisS; histidyl-tRNA synthetase
					38.000	63.500	52.3215	68.3755	
SGO_2064	0.188	8.126	0.0073	0.0270	64.500	67.000	77.8941	67.0000	ilvD; dihydroxy-acid dehydratase
					51.500	59.000	70.9094	63.5300	
SGO_2066	1.390	6.300	0.0278	0.1487	31.500	12.500	38.0413	12.5000	rpmG; ribosomal protein L33
					20.500		28.2261		
SGO_2070	-0.208	4.559	0.0776	0.5231	5.500	8.500	6.6421	8.5000	hypothetical protein SGO_2070
					3.000	4.000	4.1306	4.3071	
SGO_2085	0.619	6.785	0.0006	0.0006	28.500	22.500	34.4183	22.5000	purB; adenylosuccinate lyase
					23.500	19.500	32.3567	20.9972	
SGO_2098	0.275	10.262	0.0340	0.1914	319.500	313.000	385.8475	313.0000	rpsD; ribosomal protein S4
					208.500	224.500	287.0800	241.7370	

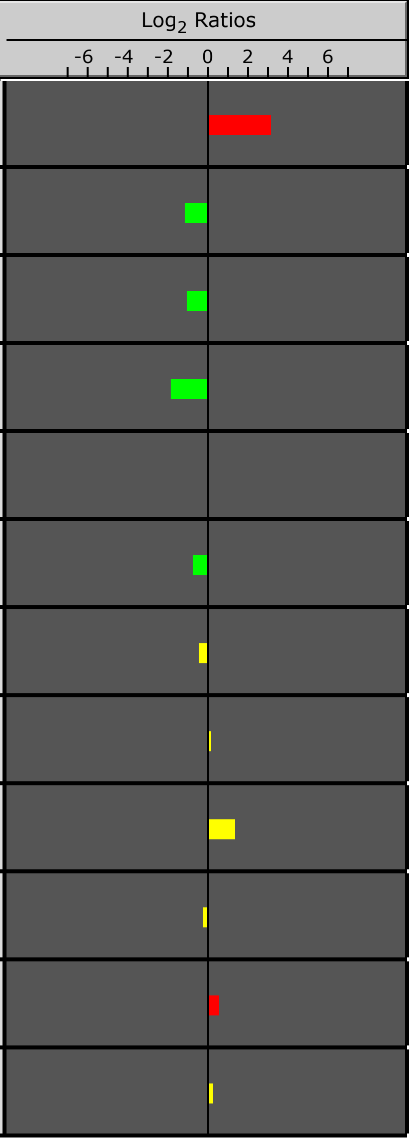

Show detected proteins only
Show all proteins
Filter by category:
ABC Transporter

Proteins found: 584

Test	Cutoff
q-Value	.005
p-Value	

Color	Signif	Direction	Applies To
red	yes	+	ratios, bars
yellow	no	n/a	bars
green	yes	−	ratios, bars
pink	yes	+	*p*- , *q*-Values
light green	yes	−	*p*- , *q*-Values

Dot Plots Dot Plots

Streptococcus gordonii

Protein	SgPgFn vs SgFn Log$_2$ Ratio	Log$_2$ Sum	*q*-Value	*p*-Value	Raw *SgPgFn*	Raw *SgFn*	Normalized *SgPgFn*	Normalized *SgFn*	Description
SGO_2100	3.572	7.678	0.0001	0.0000	76.500	9.000	92.3860	9.0000	ABC transporter substrate-binding protein
					70.000	6.500	96.3818	6.9991	
SGO_2104	0.762	7.600	0.0117	0.0501	59.000	44.500	71.2520	44.5000	srtB; sortase B
					36.500	26.000	50.2562	27.9963	
SGO_2105	-4.443	9.332	0.0000	0.0000	11.500	303.000	13.8881	303.0000	abpA; amylase-binding protein AbpA
					10.500	291.000	14.4573	313.3428	
SGO_2106	-0.501	7.373	0.0085	0.0332	28.000	55.500	33.8145	55.5000	ribose-phosphate diphosphokinase
					25.000	39.000	34.4221	41.9944	
SGO_2133	-0.292	8.626	0.0149	0.0680	76.500	121.000	92.3860	121.0000	Cell division protein ftsH-like protein
					61.500	90.000	84.6783	96.9102	
SGO_2134	-1.595	7.444	0.0006	0.0004	21.500	63.500	25.9647	63.5000	hpt; hypoxanthine phosphoribosyltransferase
					13.000	62.000	17.8995	66.7603	
SGO_2142	0.760	6.995	0.0026	0.0067	30.000	22.000	36.2298	22.0000	GTP-binding protein
					32.000	23.500	44.0602	25.3043	
SGO_2150	-1.773	6.029	0.0014	0.0025	8.000	27.500	9.6613	27.5000	degP; serine protease
					4.000	21.000	5.5075	22.6124	

Log$_2$ Ratios: -6 -4 -2 0 2 4 6

Show detected proteins only
Show all proteins
Filter by category:
ABC Transporter

Proteins found: 584

Test	Cutoff
q-Value	.005
p-Value	

Color	Signif	Direction	Applies To
red	yes	+	ratios, bars
yellow	no	n/a	bars
green	yes	–	ratios, bars
light red	yes	+	*p*- , *q*-Values
light green	yes	–	*p*- , *q*-Values

Dot Plots Dot Plots
