# Supplementary material for: Proteomics of Streptococcus gordonii within a model developing oral microbial community
Source: BMC Microbiol. 2012 Sep 18;12:211. doi: 10.1186/1471-2180-12-211 (PMC3534352; doi:10.1186/1471-2180-12-211)
Supplement: Additional file 7 — SgPgFn_vs_SgPg. A more detailed presentation of the relative abundance ratios for the comparison of SgPgFn and SgPg, including both raw and normalized spectral counts. Red and green highlights are used as in Additional file 1. [file 1471-2180-12-211-S7.pdf]

Streptococcus gordonii

Hackett Laboratory UW

Protein	SgPgFn vs SgPg: Log_2 Ratio	Log_2 Sum	*q*-Value	*p*-Value	Raw: *SgPgFn*	Raw: *SgPg*	Normalized: *SgPgFn*	Normalized: *SgPg*	Description
SGO_0001	-0.702	5.838	0.0183	0.0240	5.000	19.000	9.2209	19.8503	dnaA; chromosomal replication initiator protein DnaA
					6.000	15.500	12.6156	15.5000	
SGO_0002	-0.691	6.614	0.0067	0.0043	9.000	30.000	16.5976	31.3426	dnaN; DNA polymerase III, beta subunit
					10.000	29.000	21.0259	29.0000	
SGO_0004	-0.038	6.879	0.2009	0.7232	15.000	36.500	27.6627	38.1335	putative lipoprotein
					14.000	22.500	29.4363	22.5000	
SGO_0006	-0.362	7.354	0.0348	0.0653	22.500	46.000	41.4940	48.0586	ABC transporter, ATP-binding protein
					14.500	43.500	30.4876	43.5000	
SGO_0007	-0.368	6.754	0.0012	0.0001	13.000	29.000	23.9743	30.2978	trpS; tryptophanyl-tRNA synthetase
					11.000	30.500	23.1285	30.5000	
SGO_0008	-0.103	9.703	0.0397	0.0811	105.500	214.000	194.5610	223.5770	inosine-5'-monophosphate dehydrogenase
					98.500	208.000	207.1055	208.0000	
SGO_0011	0.238	4.509	0.1464	0.4687	4.500	6.000	8.2988	6.2685	proteinase, M16 family
					2.000	4.000	4.2052	4.0000	
SGO_0015	-0.482	3.996	0.0283	0.0484		5.500		5.7461	ABC transporter (ATP-binding protein)
					2.000	6.000	4.2052	6.0000	
SGO_0022	-1.137	5.357	0.0037	0.0010	3.000	13.000	5.5325	13.5818	trmU; tRNA (5-methylaminomethyl-2-thiouridylate)-methyltransferase
					3.500	14.500	7.3591	14.5000	
SGO_0025	0.005	5.892	0.2382	0.9134	5.500	17.500	10.1430	18.2832	gidA; glucose inhibited division protein A
					9.500	11.000	19.9746	11.0000	
SGO_0026	-0.039	3.561	0.2247	0.8413	2.000	2.500	3.6884	2.6119	DHH subfamily 1 protein
						5.500		5.5000	
SGO_0027	0.144	7.060	0.1271	0.3931	15.500	29.000	28.5848	30.2978	rplI; ribosomal protein L9
					20.000	32.500	42.0519	32.5000	

Log_2 Ratios: -6 -4 -2 0 2 4 6

Show detected proteins only
Show all proteins
Filter by category:
ABC Transporter

Proteins found: 627

Test	Cutoff
q-Value	.005
p-Value	

	Signif	Direction	Applies To
red	yes	+	ratios, bars
yellow	no	n/a	bars
green	yes	–	ratios, bars
pink	yes	+	*p*- , *q*-Values
light green	yes	–	*p*- , *q*-Values

Dot Plots Dot Plots

Hendrickson *et al.*

Protein	SgPgFn vs SgPg				Raw		Normalized		Description	Log_2 Ratios
	Log_2 Ratio	Log_2 Sum	*q*-Value	*p*-Value	*SgPgFn*	*SgPg*	*SgPgFn*	*SgPg*		-6 -4 -2 0 2 4 6
**SGO_0028**	-0.159	4.394	0.1982	0.7084		5.000		5.2238	dnaC; replicative DNA helicase	
					3.000	9.500	6.3078	9.5000		
**SGO_0030**	-0.278	6.784	0.0184	0.0244	14.500	30.000	26.7406	31.3426	aspB; aspartate transaminase	
					11.000	29.000	23.1285	29.0000		
**SGO_0032**	-0.269	6.860	0.0188	0.0254	15.500	30.500	28.5848	31.8649	plsX; fatty acid/phospholipid synthesis protein PlsX	
					11.500	31.500	24.1798	31.5000		
**SGO_0033**	0.409	5.472	0.0935	0.2631	9.500	10.000	17.5197	10.4475	acpP; acyl carrier protein	
					4.000	8.000	8.4104	8.0000		
**SGO_0042**	-0.112	5.007	0.1627	0.5485	5.500	11.500	10.1430	12.0147	transcription regulator, GntR family	
						10.000		10.0000		
**SGO_0054**	-0.915	6.823	0.0031	0.0007	11.000	33.500	20.2860	34.9992	dltA; D-alanine-activating enzyme	
					9.000	39.000	18.9233	39.0000		
**SGO_0057**	-0.316	6.092	0.0050	0.0020	8.500	18.500	15.6755	19.3279	dltD protein	
					7.000	18.500	14.7182	18.5000		
**SGO_0059**	0.895	12.958	0.0058	0.0034	1304.000	1491.000	2404.8107	1557.7259	pXO1; hypothetical protein SGO_0059	
					1313.500	1233.500	2761.7566	1233.5000		
**SGO_0063**	0.146	6.304	0.1104	0.3266	11.000	15.500	20.2860	16.1937	hypothetical protein SGO_0063	
					10.000	21.500	21.0259	21.5000		
**SGO_0064**	-0.837	7.241	0.0120	0.0121	15.000	40.000	27.6627	41.7901	FtsK/SpoIIIE family protein	
					12.500	55.500	26.2824	55.5000		
**SGO_0065**	-0.179	7.043	0.0094	0.0077	17.000	34.500	31.3511	36.0440	hypothetical protein SGO_0065	
					14.500	34.000	30.4876	34.0000		
**SGO_0066**	0.388	5.198	0.0661	0.1647	4.500	6.500	8.2988	6.7909	D-3-phosphoglycerate dehydrogenase, putative	
					6.000	9.000	12.6156	9.0000		

Show detected proteins only
Show all proteins
Filter by category:
ABC Transporter

Proteins found: 627

Test	Cutoff
***q*-Value**	**.005**
p-Value	

	Signif	Direction	Applies To
(red)	yes	+	ratios, bars
(yellow)	no	n/a	bars
(green)	yes	−	ratios, bars
(pink)	yes	+	*p*- , *q*-Values
(light green)	yes	−	*p*- , *q*-Values

Dot Plots Dot Plots

Streptococcus gordonii

Hackett Laboratory UW

Summary Table | SgFn vs Sg | SgPg vs Sg | SgPgFn vs Sg | SgPg vs SgFn | SgPgFn vs SgFn | SgPgFn vs SgPg | Coverage

Protein	SgPgFn vs SgPg Log_2 Ratio	Log_2 Sum	*q*-Value	*p*-Value	Raw *SgPgFn*	Raw *SgPg*	Normalized *SgPgFn*	Normalized *SgPg*	Description
SGO_0067	-0.954	6.452	0.0122	0.0126	7.500	32.000	13.8313	33.4321	protein with prophage function domain
					7.500	24.500	15.7695	24.5000	
SGO_0068	-0.305	5.719	0.0051	0.0023	6.500	13.500	11.9872	14.1042	lipoprotein, putative
					5.500	15.000	11.5643	15.0000	
SGO_0069	-0.305	5.023	0.0396	0.0796	4.500	9.000	8.2988	9.4028	hypothetical protein SGO_0069
					3.000	8.500	6.3078	8.5000	
SGO_0070	0.087	6.244	0.1261	0.3884	11.500	16.500	21.2081	17.2384	merozoite surface protein 1
					8.500	19.500	17.8720	19.5000	
SGO_0080	0.021	6.581	0.2181	0.8060	13.000	24.500	23.9743	25.5964	hypothetical protein SGO_0080
					11.500	22.000	24.1798	22.0000	
SGO_0099	-0.310	3.686	0.0956	0.2721	2.000	4.000	3.6884	4.1790	pulA-2; pullulanase, type I
						5.000		5.0000	
SGO_0104	0.165	8.825	0.0450	0.0968	60.000	100.000	110.6508	104.4752	Maltose/maltodextrin-binding protein precursor
					61.500	109.000	129.3095	109.0000	
SGO_0105	-0.915	5.676	0.0067	0.0043	4.000	16.500	7.3767	17.2384	malQ; 4-alpha-glucanotransferase
					5.000	16.000	10.5130	16.0000	
SGO_0106	-0.046	7.963	0.0238	0.0370	33.500	61.500	61.7800	64.2523	glgP-2; maltodextrin phosphorylase
					29.000	62.500	60.9752	62.5000	
SGO_0113	-0.143	11.620	0.0405	0.0836	416.500	838.500	768.1010	876.0249	acdH; alcohol-acetaldehyde dehydrogenase
					345.500	777.000	726.4461	777.0000	
SGO_0135	-0.332	6.712	0.0532	0.1238	10.500	25.500	19.3639	26.6412	v-type sodium ATP synthase, subunit A
					13.000	31.500	27.3337	31.5000	
SGO_0136	-0.291	6.942	0.0664	0.1660	12.500	29.500	23.0522	30.8202	v-type sodium ATP synthase, chain B
					15.500	36.500	32.5902	36.5000	

Log_2 Ratios: -6 -4 -2 0 2 4 6

Show detected proteins only
Show all proteins
Filter by category: ABC Transporter

Proteins found: 627

Test	Cutoff
q-Value	.005
p-Value	

Color	Signif	Direction	Applies To
red	yes	+	ratios, bars
yellow	no	n/a	bars
green	yes	−	ratios, bars
pink	yes	+	*p*- , *q*-Values
light green	yes	−	*p*- , *q*-Values

Dot Plots Dot Plots

Hendrickson *et al.*

Streptococcus gordonii

Hackett Laboratory UW

Protein	*SgPgFn vs SgPg* Log$_2$ Ratio	Log$_2$ Sum	*q*-Value	*p*-Value	Raw *SgPgFn*	Raw *SgPg*	Normalized *SgPgFn*	Normalized *SgPg*	Description
**SGO_0139**	-1.092	6.748	0.0063	0.0038	11.500	36.000	21.2081	37.6111	thrC; threonine synthase
					6.500	35.000	13.6669	35.0000	
**SGO_0145**	-0.282	6.743	0.0224	0.0329	12.000	27.000	22.1302	28.2083	polI; DNA polymerase I
					12.500	30.500	26.2824	30.5000	
**SGO_0146**	-0.416	5.791	0.1828	0.6415	10.500	16.000	19.3639	16.7160	CoA-binding domain protein
					3.000	13.000	6.3078	13.0000	
**SGO_0152**	-1.441	5.438	0.0019	0.0003	3.500	14.500	6.4546	15.1489	tgt; queuine tRNA-ribosyltransferase
					2.500	16.500	5.2565	16.5000	
**SGO_0154**	0.309	10.324	0.0256	0.0417	193.000	307.000	355.9267	320.7390	pgi; glucose-6-phosphate isomerase
					167.000	254.000	351.1331	254.0000	
**SGO_0156**	-0.369	5.181	0.0319	0.0567	4.000	11.000	7.3767	11.4923	hypothetical protein SGO_0156
					4.000	9.000	8.4104	9.0000	
**SGO_0158**	0.454	7.674	0.0376	0.0735	37.500	37.500	69.1567	39.1782	2,3,4,5-tetrahydropyridine-2-carboxylate N-succinyltransferase, putative
					23.500	46.500	49.4109	46.5000	
**SGO_0163**	-0.737	7.118	0.0007	0.0000	14.000	41.000	25.8185	42.8348	galU; UTP-glucose-1-phosphate uridylyltransferase
					12.500	44.000	26.2824	44.0000	
**SGO_0164**	-0.244	6.572	0.0594	0.1433	11.000	28.000	20.2860	29.2531	Glycerol-3-phosphate dehydrogenase [NAD(P)+] (NAD(P)H-dependent glycerol-3-phosphate dehydrogenase)
					11.000	22.500	23.1285	22.5000	
**SGO_0169**	0.144	5.383	0.1609	0.5407	5.500	11.500	10.1430	12.0147	dut; dUTP diphosphatase
					5.500	8.000	11.5643	8.0000	
**SGO_0171**	-1.163	4.672	0.0664	0.1664	2.500	8.500	4.6104	8.8804	radA; DNA repair protein RadA
						12.000		12.0000	
**SGO_0174**	-0.429	8.419	0.0055	0.0029	37.000	94.500	68.2347	98.7291	gltX; glutamyl-tRNA synthetase
					37.000	97.500	77.7960	97.5000	

Log$_2$ Ratios: -6 -4 -2 0 2 4 6

Show detected proteins only
Show all proteins
Filter by category:
ABC Transporter

Proteins found: 627

Test	Cutoff
***q*-Value**	**.005**
p-Value	

Color	Signif	Direction	Applies To
red	yes	+	ratios, bars
yellow	no	n/a	bars
green	yes	−	ratios, bars
pink	yes	+	*p*- , *q*-Values
light green	yes	−	*p*- , *q*-Values

Dot Plots Dot Plots

Protein	SgPgFn vs SgPg Log_2 Ratio	Log_2 Sum	*q*-Value	*p*-Value	Raw *SgPgFn*	Raw *SgPg*	Normalized *SgPgFn*	Normalized *SgPg*	Description
SGO_0180	-0.991	6.054	0.0521	0.1201	3.500	26.500	6.4546	27.6859	jag; hypothetical protein SGO_0180
					8.000	15.500	16.8207	15.5000	
SGO_0190	0.512	3.791	0.2040	0.7382		2.000		2.0895	hypothetical protein SGO_0190
					2.500	6.500	5.2565	6.5000	
SGO_0197	0.637	3.854	0.0520	0.1190		3.500		3.6566	predicted ribosome small subunit-dependent GTPase A
					3.000	4.500	6.3078	4.5000	
SGO_0198	-0.532	6.794	0.0089	0.0068	11.000	32.000	20.2860	33.4321	rpe; ribulose-phosphate 3-epimerase
					12.000	32.000	25.2311	32.0000	
SGO_0200	-0.591	6.391	0.0165	0.0202	7.500	24.500	13.8313	25.5964	competence-induced protein Ccs50
					9.500	24.500	19.9746	24.5000	
SGO_0201	-0.917	6.416	0.0048	0.0017	7.000	26.000	12.9093	27.1636	cmp-binding-factor 1
					8.000	28.500	16.8207	28.5000	
SGO_0204	-0.158	7.633	0.0830	0.2217	25.000	56.500	46.1045	59.0285	rpsL; ribosomal protein S12
					22.500	46.000	47.3084	46.0000	
SGO_0205	0.071	9.299	0.2016	0.7269	75.000	124.000	138.3135	129.5493	rpsG; ribosomal protein S7
					87.500	178.000	183.9769	178.0000	
SGO_0206	0.140	12.413	0.0590	0.1419	837.000	1277.000	1543.5786	1334.1488	fusA; translation elongation factor G
					627.500	1258.000	1319.3775	1258.0000	
SGO_0207	0.375	12.579	0.0248	0.0399	830.000	1267.000	1530.6694	1323.7013	gap; glyceraldehyde-3-phosphate dehydrogenase, type I
					919.500	1330.000	1933.3348	1330.0000	
SGO_0209	-0.484	13.183	0.0050	0.0022	1021.000	2477.500	1882.9077	2588.3741	pgk; phosphoglycerate kinase
					948.000	2836.000	1993.2587	2836.0000	
SGO_0215	-0.339	7.921	0.0562	0.1327	34.500	70.000	63.6242	73.1327	glnA; glutamine synthetase, type I
					21.000	61.500	44.1545	61.5000	

Log_2 Ratios: -6 -4 -2 0 2 4 6

Show detected proteins only
Show all proteins
Filter by category:
ABC Transporter

Proteins found: 627

Test	Cutoff
q-Value	.005
p-Value	

	Signif	Direction	Applies To
red	yes	+	ratios, bars
yellow	no	n/a	bars
green	yes	−	ratios, bars
pink	yes	+	*p*- , *q*-Values
light green	yes	−	*p*- , *q*-Values

Dot Plots Dot Plots

Hendrickson *et al.*

Protein	*SgPgFn vs SgPg* Log$_2$ Ratio	Log$_2$ Sum	*q*-Value	*p*-Value	Raw *SgPgFn*	Raw *SgPg*	Normalized *SgPgFn*	Normalized *SgPg*	Description
**SGO_0219**	-0.389	7.715	0.0050	0.0021	26.000	57.000	47.9487	59.5509	metallo-beta-lactamase superfamily protein 1
					20.500	59.500	43.1032	59.5000	
**SGO_0230**	0.108	5.720	0.1672	0.5724	5.000	12.500	9.2209	13.0594	Protein of unknown function, DUF536 family
					9.000	11.500	18.9233	11.5000	
**SGO_0232**	-0.102	4.947	0.1775	0.6200	4.500	5.500	8.2988	5.7461	conserved hypothetical protein TIGR00103
					3.000	10.500	6.3078	10.5000	
**SGO_0234**	-0.522	6.807	0.0178	0.0227	13.500	35.000	24.8964	36.5663	pepX; X-Pro dipeptidyl-peptidase
					10.000	29.500	21.0259	29.5000	
**SGO_0237**	-1.119	5.325	0.0099	0.0084	2.500	12.500	4.6104	13.0594	ccpA; CcpA protein (proteinase)
					4.000	14.000	8.4104	14.0000	
**SGO_0243**	0.965	4.260	0.0166	0.0206		4.500		4.7014	hydroxymethylglutaryl-CoA reductase, degradative
					4.500	5.000	9.4617	5.0000	
**SGO_0244**	-0.203	5.193	0.0935	0.2633	6.000	11.500	11.0651	12.0147	hydroxymethylglutaryl-CoA synthase
						13.500		13.5000	
**SGO_0247**	0.100	9.898	0.0316	0.0559	137.000	213.500	252.6527	223.0546	pfl; formate acetyltransferase
					114.500	237.500	240.7470	237.5000	
**SGO_0252**	0.427	3.562	0.1586	0.5274	2.500	4.500	4.6104	4.7014	possible TetR-type transcriptional regulator
						2.500		2.5000	
**SGO_0255**	-0.533	5.836	0.0073	0.0051	7.000	16.000	12.9093	16.7160	Signal peptidase I
					5.000	17.000	10.5130	17.0000	
**SGO_0260**	-1.274	6.073	0.0045	0.0014	4.500	21.500	8.2988	22.4622	DNA mismatch binding protein MutS2
					5.500	25.000	11.5643	25.0000	
**SGO_0262**	-1.706	6.302	0.0019	0.0003	5.500	30.500	10.1430	31.8649	dipeptidase
					4.000	28.500	8.4104	28.5000	

Log$_2$ Ratios: -6 -4 -2 0 2 4 6

Show detected proteins only
Show all proteins
Filter by category:
ABC Transporter

Proteins found: 627

Test	Cutoff
***q*-Value**	**.005**
p-Value	

	Signif	Direction	Applies To
(red)	yes	+	ratios, bars
(yellow)	no	n/a	bars
(green)	yes	−	ratios, bars
(pink)	yes	+	*p*- , *q*-Values
(light green)	yes	−	*p*- , *q*-Values

Dot Plots Dot Plots

Hendrickson *et al.*

Protein	SgPgFn vs SgPg Log$_2$ Ratio	Log$_2$ Sum	*q*-Value	*p*-Value	Raw *SgPgFn*	Raw *SgPg*	Normalized *SgPgFn*	Normalized *SgPg*	Description
SGO_0263	0.690	7.637	0.0306	0.0536	41.000	34.500	75.6114	36.0440	trx-1; thioredoxin
					23.000	39.000	48.3597	39.0000	
SGO_0272	0.368	5.959	0.0212	0.0303	10.500	13.000	19.3639	13.5818	hypothetical protein SGO_0272
					7.500	13.500	15.7695	13.5000	
SGO_0276	0.077	8.931	0.1104	0.3263	62.500	114.000	115.2612	119.1018	gdhA; glutamate dehydrogenase (NADP)
					64.500	118.000	135.6173	118.0000	
SGO_0290	0.001	4.065	0.2458	0.9518	3.000	4.500	5.5325	4.7014	copper -translocating P-type ATPase
						6.500		6.5000	
SGO_0291	-0.884	5.946	0.0057	0.0032	5.000	18.500	9.2209	19.3279	copper-translocating P-type ATPase
					6.000	20.500	12.6156	20.5000	
SGO_0292	0.159	9.120	0.0391	0.0774	84.000	120.000	154.9111	125.3703	spxB; pyruvate oxidase
					66.000	137.500	138.7712	137.5000	
SGO_0312	0.258	11.002	0.0042	0.0012	309.000	441.500	569.8516	461.2582	xfp; D-xylulose 5-phosphate/D-fructose 6-phosphate phosphoketolase
					260.000	472.500	546.6743	472.5000	
SGO_0321	-0.292	6.267	0.0650	0.1584	8.000	18.000	14.7534	18.8055	polypeptide deformylase
					9.500	23.500	19.9746	23.5000	
SGO_0333	0.128	7.930	0.1089	0.3178	30.000	55.000	55.3254	57.4614	rpsO; ribosomal protein S15
					34.500	58.500	72.5395	58.5000	
SGO_0342	-0.280	7.594	0.0330	0.0606	25.000	46.000	46.1045	48.0586	pepF-2; oligoendopeptidase
					19.500	58.000	41.0006	58.0000	
SGO_0344	0.030	8.477	0.0735	0.1898	48.000	85.500	88.5206	89.3263	pnpA; polyribonucleotide nucleotidyltransferase
					43.500	87.000	91.4628	87.0000	
SGO_0349	0.186	6.196	0.0419	0.0871	11.500	16.500	21.2081	17.2384	cysS; cysteinyl-tRNA synthetase
					8.500	17.000	17.8720	17.0000	

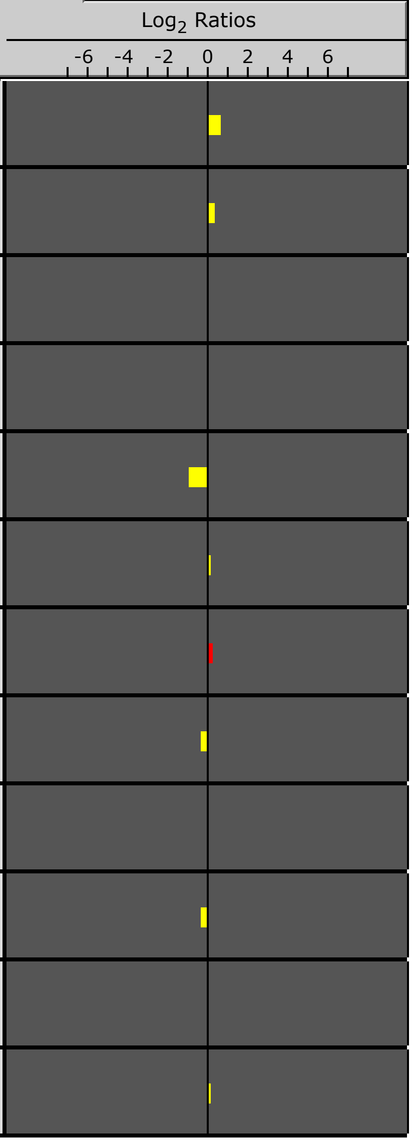

Show detected proteins only
Show all proteins
Filter by category:
ABC Transporter

Proteins found: 627

Test	Cutoff
q-Value	.005
p-Value	

	Signif	Direction	Applies To
red	yes	+	ratios, bars
yellow	no	n/a	bars
green	yes	–	ratios, bars
pink	yes	+	*p*- , *q*-Values
light green	yes	–	*p*- , *q*-Values

Dot Plots Dot Plots

Hendrickson *et al.*

Protein	SgPgFn vs SgPg Log_2 Ratio	Log_2 Sum	*q*-Value	*p*-Value	Raw *SgPgFn*	Raw *SgPg*	Normalized *SgPgFn*	Normalized *SgPg*	Description
SGO_0352	-0.043	6.880	0.1733	0.6020	16.000	31.500	29.5069	32.9097	ABC transporter, ATP-binding protein SP1580
					13.500	27.000	28.3850	27.0000	
SGO_0357	-0.137	7.442	0.0627	0.1523	21.000	46.000	38.7278	48.0586	degV; DegV family fatty acid binding protein
					21.000	43.000	44.1545	43.0000	
SGO_0358	0.221	9.027	0.0116	0.0115	73.000	117.500	134.6251	122.7584	rplM; ribosomal protein L13
					69.500	118.000	146.1303	118.0000	
SGO_0359	0.488	7.813	0.0120	0.0122	32.500	43.000	59.9358	44.9244	rpsI; ribosomal protein S9
					34.000	48.500	71.4882	48.5000	
SGO_0372	-0.374	5.811	0.0055	0.0029	7.000	15.000	12.9093	15.6713	malate oxidoreductase
					5.500	16.000	11.5643	16.0000	
SGO_0374	-0.954	5.376	0.0145	0.0161	5.000	12.000	9.2209	12.5370	Response regulator of the LytR/AlgR family
					2.500	14.500	5.2565	14.5000	
SGO_0384	0.142	5.090	0.1416	0.4514	4.500	6.500	8.2988	6.7909	putative carboxylate-amine/thiol ligase
					4.500	9.500	9.4617	9.5000	
SGO_0390	-0.369	7.920	0.0236	0.0363	27.000	72.000	49.7929	75.2222	glycerol-3-phosphate dehydrogenase (NAD (P)+)
					26.500	61.500	55.7187	61.5000	
SGO_0392	-0.604	5.365	0.0830	0.2216	5.500	13.000	10.1430	13.5818	phosphoglycerate mutase
						17.500		17.5000	
SGO_0398	-0.054	4.508	0.2021	0.7300	2.500	7.500	4.6104	7.8356	ABC transporter ATP-binding protein
					3.000	4.000	6.3078	4.0000	
SGO_0401	0.708	6.598	0.0127	0.0135	17.000	14.500	31.3511	15.1489	grpE; co-chaperone GrpE
					13.500	22.000	28.3850	22.0000	
SGO_0402	0.026	10.853	0.1603	0.5363	245.000	424.500	451.8241	443.4974	dnaK; DnaK chaperone protein
					229.000	473.000	481.4939	473.0000	

Log2 Ratios: -6 -4 -2 0 2 4 6

Show detected proteins only
Show all proteins
Filter by category:
ABC Transporter

Proteins found: 627

Test	Cutoff
q-Value	.005
p-Value	

	Signif	Direction	Applies To
red	yes	+	ratios, bars
yellow	no	n/a	bars
green	yes	−	ratios, bars
pink	yes	+	*p*- , *q*-Values
light green	yes	−	

Dot Plots Dot Plots

Hendrickson *et al.*

Protein	SgPgFn vs SgPg				Raw		Normalized		Description
	Log_2 Ratio	Log_2 Sum	*q*-Value	*p*-Value	*SgPgFn*	*SgPg*	*SgPgFn*	*SgPg*	
**SGO_0404**	-0.415	5.889	0.0769	0.2007	5.000	16.000	9.2209	16.7160	dnaJ; DnaJ chaparone protein
					8.000	16.500	16.8207	16.5000	
**SGO_0411**	0.118	4.564	0.1587	0.5290	4.500	8.000	8.2988	8.3580	conserved hypothetical protein TIGR01440
						7.000		7.0000	
**SGO_0412**	0.259	11.086	0.0051	0.0025	328.500	463.000	605.8131	483.7204	tig; trigger factor
					275.000	506.000	578.2132	506.0000	
**SGO_0413**	-0.577	6.900	0.0368	0.0718	12.500	27.000	23.0522	28.2083	DNA-directed RNA polymerase delta chain
					11.500	44.000	24.1798	44.0000	
**SGO_0415**	-0.140	9.505	0.0226	0.0337	89.500	184.000	165.0541	192.2344	secA; preprotein translocase, SecA subunit
					86.000	188.500	180.8230	188.5000	
**SGO_0416**	-0.964	6.657	0.0227	0.0340	12.000	37.000	22.1302	38.6558	phospho-2-dehydro-3-deoxyheptonate aldolase
					6.000	27.500	12.6156	27.5000	
**SGO_0426**	0.105	4.407	0.1979	0.7048	4.000	7.500	7.3767	7.8356	Cof family protein
						6.000		6.0000	
**SGO_0427**	0.043	6.199	0.1767	0.6150	10.500	16.000	19.3639	16.7160	universal stress protein family
					8.500	19.500	17.8720	19.5000	
**SGO_0429**	-0.226	8.510	0.0878	0.2406	43.500	112.500	80.2218	117.5346	aspartate transaminase
					41.000	80.500	86.2063	80.5000	
**SGO_0432**	-0.301	6.773	0.0214	0.0309	14.000	31.000	25.8185	32.3873	entB; isochorismatase family protein
					11.000	28.000	23.1285	28.0000	
**SGO_0434**	-0.404	6.589	0.0524	0.1213	13.500	23.000	24.8964	24.0293	aspS-2; aspartyl-tRNA synthetase
					8.000	30.500	16.8207	30.5000	
**SGO_0435**	0.065	5.843	0.1504	0.4844	8.500	12.500	15.6755	13.0594	gatC; glutamyl-tRNA(Gln) amidotransferase, C subunit
					6.500	15.000	13.6669	15.0000	

Log_2 Ratios: -6 -4 -2 0 2 4 6

Show detected proteins only
Show all proteins
Filter by category:
ABC Transporter

Proteins found: 627

Test	Cutoff
***q*-Value**	**.005**
p-Value	

	Signif	Direction	Applies To
red	yes	+	ratios, bars
yellow	no	n/a	bars
green	yes	−	ratios, bars
pink	yes	+	*p*- , *q*-Values
light green	yes	−	*p*- , *q*-Values

Dot Plots Dot Plots

Protein	SgPgFn vs SgPg Log_2 Ratio	Log_2 Sum	*q*-Value	*p*-Value	Raw *SgPgFn*	Raw *SgPg*	Normalized *SgPgFn*	Normalized *SgPg*	Description
SGO_0436	-0.090	8.584	0.0541	0.1266	50.000	99.500	92.2090	103.9529	gatA; glutamyl-tRNA(Gln) amidotransferase, A subunit
					44.500	94.000	93.5654	94.0000	
SGO_0437	-0.208	8.655	0.1558	0.5048	65.000	95.000	119.8717	99.2515	gatB; glutamyl-tRNA(Gln) amidotransferase, B subunit
					33.500	113.500	70.4369	113.5000	
SGO_0440	-0.137	4.572	0.1770	0.6170	4.000	9.000	7.3767	9.4028	L-iditol 2-dehydrogenase BH3949
						7.000		7.0000	
SGO_0445	-0.762	6.115	0.0125	0.0132	6.000	23.000	11.0651	24.0293	GTP-binding protein
					7.000	19.500	14.7182	19.5000	
SGO_0448	-1.206	3.960	0.0358	0.0691	1.500	6.500	2.7663	6.7909	conserved hypothetical protein TIGR00488
						6.000		6.0000	
SGO_0454	-0.499	7.151	0.0878	0.2397	10.500	37.500	19.3639	39.1782	conserved hypothetical protein TIGR01033
					20.000	41.500	42.0519	41.5000	
SGO_0455	-0.914	5.663	0.0166	0.0204	3.500	14.500	6.4546	15.1489	lipoprotein, putative
					5.500	17.500	11.5643	17.5000	
SGO_0457	0.099	7.774	0.0397	0.0810	30.000	48.500	55.3254	50.6705	ABC transporter, substrate-binding protein SP0148
					27.500	55.000	57.8213	55.0000	
SGO_0458	0.069	9.113	0.0419	0.0868	74.500	128.000	137.3914	133.7283	hlpA; lipoprotein
					69.500	136.500	146.1303	136.5000	
SGO_0460	-0.117	5.619	0.0524	0.1210	6.500	13.000	11.9872	13.5818	ABC transporter, ATP-binding protein SP0151
					5.500	12.000	11.5643	12.0000	
SGO_0468	-0.067	5.563	0.2181	0.8046	7.500	11.000	13.8313	11.4923	hypothetical protein SGO_0468
					4.500	12.500	9.4617	12.5000	
SGO_0476	0.096	6.290	0.0979	0.2804	10.500	19.500	19.3639	20.3727	rhodanese family protein
					10.000	17.500	21.0259	17.5000	

Log2 Ratios: -6 -4 -2 0 2 4 6

- Show detected proteins only
- Show all proteins
- Filter by category: ABC Transporter

Proteins found: 627

Test	Cutoff
q-Value	.005
p-Value	

	Signif	Direction	Applies To
red	yes	+	ratios, bars
yellow	no	n/a	bars
green	yes	–	ratios, bars
pink	yes	+	*p*- , *q*-Values
light green	yes	–	*p*- , *q*-Values

Dot Plots Dot Plots

Protein	*SgPgFn vs SgPg* Log$_2$ Ratio	Log$_2$ Sum	*q*-Value	*p*-Value	Raw *SgPgFn*	Raw *SgPg*	Normalized *SgPgFn*	Normalized *SgPg*	Description	Log$_2$ Ratios (-6 -4 -2 0 2 4 6)
**SGO_0480**	-1.413	5.063	0.0499	0.1131		15.000		15.6713	hypothetical protein SGO_0480	
					2.500	12.500	5.2565	12.5000		
**SGO_0483**	0.069	6.312	0.2214	0.8252	11.000	15.000	20.2860	15.6713	hypothetical protein SGO_0483	
					9.500	23.500	19.9746	23.5000		
**SGO_0501**	0.314	6.827	0.0037	0.0010	17.000	25.000	31.3511	26.1188	Uncharacterized ACR, COG1399	
					15.000	24.500	31.5389	24.5000		
**SGO_0502**	-0.336	8.810	0.0263	0.0431	52.500	133.000	96.8194	138.9521	floL; flotillin-like protein	
					48.000	112.000	100.9245	112.0000		
**SGO_0503**	0.328	10.994	0.0086	0.0064	322.000	420.000	593.8260	438.7960	gnd; 6-phosphogluconate dehydrogenase, decarboxylating	
					257.500	465.000	541.4178	465.0000		
**SGO_0505**	-0.379	7.786	0.0264	0.0435	24.000	53.500	44.2603	55.8943	PTS system, IIBC component	
					24.500	69.000	51.5135	69.0000		
**SGO_0508**	0.202	5.722	0.0616	0.1493	8.500	11.000	15.6755	11.4923	nrdR; transcriptional regulator, NrdR family	
					6.000	13.000	12.6156	13.0000		
**SGO_0510**	-0.517	5.948	0.0158	0.0183	7.500	19.000	13.8313	19.8503	dnaI; primosomal protein DnaI	
					5.500	16.500	11.5643	16.5000		
**SGO_0511**	0.022	4.638	0.2139	0.7868	4.000	6.000	7.3767	6.2685	NADPH-flavin oxidoreductase -like protein	
					2.500	6.000	5.2565	6.0000		
**SGO_0512**	0.241	7.855	0.0443	0.0950	38.000	50.000	70.0788	52.2376	GTP-binding protein engA	
					26.500	53.500	55.7187	53.5000		
**SGO_0515**	0.385	8.219	0.0246	0.0388	40.500	61.500	74.6893	64.2523	murC; UDP-N-acetylmuramate--alanine ligase	
					45.000	64.500	94.6167	64.5000		
**SGO_0518**	-0.123	7.418	0.0956	0.2702	20.000	44.000	36.8836	45.9691	aminodeoxychorismate lyase-like protein	
					21.500	43.000	45.2058	43.0000		

Show detected proteins only
Show all proteins
Filter by category:
ABC Transporter

Proteins found:
627

Test	Cutoff
***q*-Value**	**.005**
p-Value	

Color	Signif	Direction	Applies To
red	yes	+	ratios, bars
yellow	no	n/a	bars
green	yes	−	ratios, bars
pink	yes	+	*p*- , *q*-Values
light green	yes	−	*p*- , *q*-Values

Dot Plots | Dot Plots

Protein	*SgPgFn vs SgPg*				Raw		Normalized		Description
	Log_2 Ratio	Log_2 Sum	*q*-Value	*p*-Value	*SgPgFn*	*SgPg*	*SgPgFn*	*SgPg*	
**SGO_0519**	-0.349	6.975	0.0315	0.0556	14.500	38.000	26.7406	39.7006	greA; transcription elongation factor greA
					13.500	31.000	28.3850	31.0000	
**SGO_0526**	-1.420	5.561	0.0050	0.0022	3.000	18.000	5.5325	18.8055	ilvB; acetolactate synthase, large subunit, biosynthetic type
					3.500	15.500	7.3591	15.5000	
**SGO_0527**	-1.189	4.162	0.0867	0.2343		5.500		5.7461	ilvN; acetolactate synthase, small subunit
					1.500	9.000	3.1539	9.0000	
**SGO_0528**	-0.289	8.143	0.0423	0.0885	39.000	79.000	71.9230	82.5354	ilvC; ketol-acid reductoisomerase
					26.500	72.500	55.7187	72.5000	
**SGO_0529**	-0.252	4.168	0.1566	0.5093		5.000		5.2238	ilvA; threonine dehydratase
					2.500	7.500	5.2565	7.5000	
**SGO_0535**	-0.400	4.343	0.1153	0.3452	3.000	6.000	5.5325	6.2685	putative transcriptional regulator LytR
						8.500		8.5000	
**SGO_0536**	0.196	6.491	0.0198	0.0277	13.500	21.000	24.8964	21.9398	hypothetical protein SGO_0536
					11.000	20.000	23.1285	20.0000	
**SGO_0537**	-0.446	6.219	0.0051	0.0025	8.000	20.500	14.7534	21.4174	HIT family protein
					8.000	21.500	16.8207	21.5000	
**SGO_0540**	-0.248	6.654	0.0094	0.0078	13.000	25.500	23.9743	26.6412	hypothetical protein SGO_0540
					10.500	28.000	22.0772	28.0000	
**SGO_0543**	-0.355	7.276	0.0105	0.0098	17.000	42.000	31.3511	43.8796	nusA; transcription termination factor NusA
					17.500	43.000	36.7954	43.0000	
**SGO_0546**	-0.338	8.102	0.0476	0.1054	27.500	77.500	50.7149	80.9683	infB; Translation initiation factor IF-2
					34.000	71.500	71.4882	71.5000	
**SGO_0548**	-0.725	4.840	0.0050	0.0019	3.000	8.000	5.5325	8.3580	Na/Pi-cotransporter family protein
					2.500	9.500	5.2565	9.5000	

Log_2 Ratios: -6 -4 -2 0 2 4 6

Show detected proteins only
Show all proteins
Filter by category:
ABC Transporter

Proteins found: 627

Test	Cutoff
***q*-Value**	**.005**
p-Value	

	Signif	Direction	Applies To
(red)	yes	+	ratios, bars
(yellow)	no	n/a	bars
(green)	yes	–	ratios, bars
(pink)	yes	+	*p*- , *q*-Values
(light green)	yes	–	*p*- , *q*-Values

Dot Plots Dot Plots

Hendrickson *et al.*

Protein	SgPgFn vs SgPg Log2 Ratio	Log2 Sum	q-Value	p-Value	Raw SgPgFn	Raw SgPg	Normalized SgPgFn	Normalized SgPg	Description	Log2 Ratios (-6 -4 -2 0 2 4 6)
SGO_0552	-0.205	6.502	0.0184	0.0242	12.000	22.500	22.1302	23.5069	oxidoreductase, aldo/keto reductase family	
					9.500	25.000	19.9746	25.0000		
SGO_0554	-0.707	4.799	0.0921	0.2571	3.500	8.500	6.4546	8.8804	hsdR; type I site-specific deoxyribonuclease	
						12.500		12.5000		
SGO_0558	-0.093	3.750	0.2118	0.7732		5.500		5.7461	hypothetical protein SGO_0558	
					2.000	3.500	4.2052	3.5000		
SGO_0560	-0.567	5.174	0.0151	0.0173	4.500	11.000	8.2988	11.4923	hsdM; type I restriction-modification system, M subunit	
					3.000	10.000	6.3078	10.0000		
SGO_0565	-0.204	7.560	0.0246	0.0387	23.000	45.500	42.4161	47.5362	adhA; alcohol dehydrogenase	
					21.500	53.500	45.2058	53.5000		
SGO_0568	-0.346	6.590	0.0499	0.1129	9.500	24.000	17.5197	25.0741	glyQ; glycyl-tRNA synthetase, alpha subunit	
					12.000	28.500	25.2311	28.5000		
SGO_0569	-0.173	8.079	0.1338	0.4182	28.000	67.000	51.6370	69.9984	glyS; glycyl-tRNA synthetase, beta subunit	
					36.500	72.000	76.7447	72.0000		
SGO_0573	-1.078	5.593	0.0120	0.0121	3.000	16.000	5.5325	16.7160	mraW; S-adenosyl-methyltransferase MraW	
					5.000	15.500	10.5130	15.5000		
SGO_0577	-0.602	6.863	0.0238	0.0368	10.000	35.500	18.4418	37.0887	ATP-dependent RNA helicase	
					13.500	32.500	28.3850	32.5000		
SGO_0581	-0.856	5.563	0.0121	0.0124	4.500	12.500	8.2988	13.0594	trxB; thioredoxin-disulfide reductase	
					4.000	17.500	8.4104	17.5000		
SGO_0582	-0.827	7.536	0.0224	0.0331	21.500	47.000	39.6499	49.1034	nicotinate phosphoribosyltransferase, putative	
					13.000	69.500	27.3337	69.5000		
SGO_0583	-0.613	5.640	0.0045	0.0014	5.000	14.000	9.2209	14.6265	nadE; NAD+ synthetase	
					5.000	15.500	10.5130	15.5000		

Show detected proteins only
Show all proteins
Filter by category:
ABC Transporter

Proteins found: 627

Test	Cutoff
q-Value	.005
p-Value	

	Signif	Direction	Applies To
red	yes	+	ratios, bars
yellow	no	n/a	bars
green	yes	−	ratios, bars
pink	yes	+	p- , q-Values
light green	yes	−	p- , q-Values

Dot Plots Dot Plots

Protein	SgPgFn vs SgPg Log2 Ratio	Log2 Sum	q-Value	p-Value	Raw SgPgFn	Raw SgPg	Normalized SgPgFn	Normalized SgPg	Description
SGO_0585	-0.393	6.850	0.0426	0.0898	15.000	36.000	27.6627	37.6111	pepC; aminopeptidase C
					10.500	28.000	22.0772	28.0000	
SGO_0586	-0.529	7.227	0.0163	0.0197	15.500	47.000	28.5848	49.1034	pbp1a; penicillin-binding protein 1A
					15.500	39.500	32.5902	39.5000	
SGO_0589	-0.265	7.239	0.0979	0.2806	21.000	31.500	38.7278	32.9097	methylase
					14.000	50.000	29.4363	50.0000	
SGO_0590	-0.575	4.491	0.1229	0.3763	3.000	10.000	5.5325	10.4475	Methyltransferase
						6.500		6.5000	
SGO_0591	-0.545	6.487	0.0058	0.0034	9.000	25.500	16.5976	26.6412	hypothetical protein SGO_0591
					9.500	26.500	19.9746	26.5000	
SGO_0592	-0.928	5.148	0.0264	0.0438	4.000	13.000	7.3767	13.5818	luxS; autoinducer-2 production protein LuxS
						14.500		14.5000	
SGO_0593	-0.945	6.782	0.0089	0.0068	12.000	37.500	22.1302	39.1782	HD/KH domain protein
					7.500	33.000	15.7695	33.0000	
SGO_0594	-0.285	5.592	0.0336	0.0621	5.500	11.500	10.1430	12.0147	gmk; Guanylate kinase (GMP kinase)
					5.500	14.500	11.5643	14.5000	
SGO_0595	-0.365	6.369	0.0086	0.0063	10.500	22.000	19.3639	22.9846	DNA-directed RNA polymerase, omega subunit
					8.000	23.500	16.8207	23.5000	
SGO_0597	-0.930	5.400	0.0057	0.0031	4.500	14.000	8.2988	14.6265	fmt; methionyl-tRNA formyltransferase
					3.000	13.000	6.3078	13.0000	
SGO_0599	-0.192	6.080	0.1225	0.3743	7.000	18.500	12.9093	19.3279	phosphoprotein phosphatase
					9.000	16.500	18.9233	16.5000	
SGO_0600	-0.833	5.248	0.0549	0.1291	4.500	15.500	8.2988	16.1937	serine/threonine protein kinase
						13.500		13.5000	

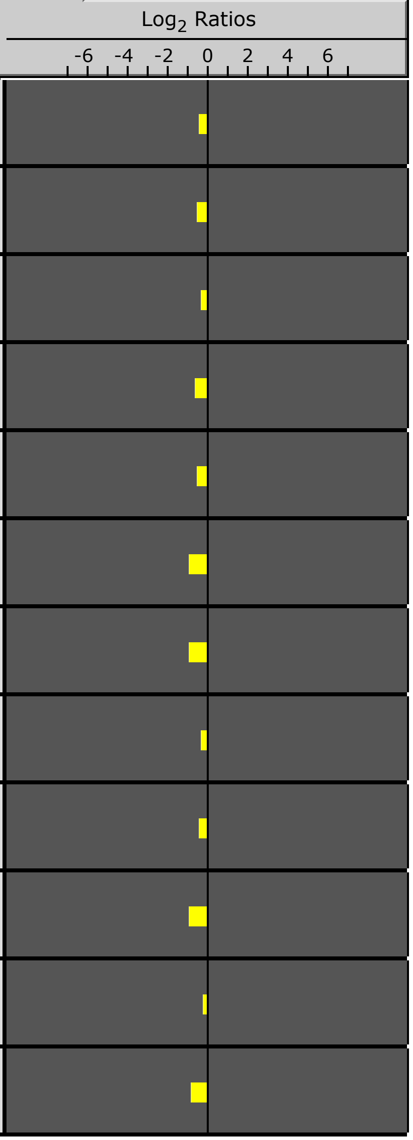

Show detected proteins only
Show all proteins
Filter by category:
ABC Transporter

Proteins found: 627

Test	Cutoff
q-Value	.005
p-Value	

	Signif	Direction	Applies To
red	yes	+	ratios, bars
yellow	no	n/a	bars
green	yes	−	ratios, bars
pink	yes	+	p- , q-Values
light green	yes	−	p- , q-Values

Dot Plots Dot Plots

Streptococcus gordonii

Protein	SgPgFn vs SgPg: Log_2 Ratio	Log_2 Sum	*q*-Value	*p*-Value	Raw: *SgPgFn*	Raw: *SgPg*	Normalized: *SgPgFn*	Normalized: *SgPg*	Description	Log_2 Ratios (-6 -4 -2 0 2 4 6)
SGO_0604	-0.281	7.730	0.0121	0.0124	27.500	54.000	50.7149	56.4166	hydrolase, haloacid dehalogenase family/peptidyl-prolyl cis-trans isomerase, cyclophilin type	
					21.500	60.000	45.2058	60.0000		
SGO_0606	-0.116	8.560	0.0452	0.0978	48.000	89.000	88.5206	92.9830	cysK; cysteine synthase A	
					44.000	103.500	92.5141	103.5000		
SGO_0610	-0.202	8.303	0.1450	0.4632	50.000	84.500	92.2090	88.2816	ribosomal subunit interface protein	
					27.000	78.500	56.7700	78.5000		
SGO_0626	-0.391	4.073	0.1141	0.3401	2.500	5.000	4.6104	5.2238	recX; Regulatory protein recX	
						7.000		7.0000		
SGO_0631	-1.276	6.339	0.0012	0.0001	6.000	28.000	11.0651	29.2531	alpha-glycerophosphate oxidase	
					6.000	28.000	12.6156	28.0000		
SGO_0632	-0.428	5.532	0.0101	0.0091	5.000	12.000	9.2209	12.5370	glpK; glycerol kinase	
					5.000	14.000	10.5130	14.0000		
SGO_0639	-0.483	7.708	0.0168	0.0211	27.000	55.500	49.7929	57.9838	valS; valyl-tRNA synthetase	
					18.000	63.500	37.8467	63.5000		
SGO_0640	2.176	4.306	0.0195	0.0266		2.500		2.6119	modification methylase	
					6.500	3.500	13.6669	3.5000		
SGO_0641	-0.693	6.206	0.0057	0.0032	8.500	22.500	15.6755	23.5069	ATPase, histidine kinase-, DNA gyrase B-, and HSP90-like domain protein protein	
					6.000	22.000	12.6156	22.0000		
SGO_0642	2.641	7.544	0.0019	0.0003	46.500	15.000	85.7544	15.6713	hypothetical protein SGO_0642	
					35.500	10.500	74.6421	10.5000		
SGO_0643	-0.102	3.563	0.2074	0.7545	2.000	3.000	3.6884	3.1343	cytosine-specific methyltransferase	
						5.000		5.0000		
SGO_0644	-0.638	6.594	0.0262	0.0428	9.000	33.000	16.5976	34.4768	hypothetical protein SGO_0644	
					10.000	24.500	21.0259	24.5000		

◉ Show detected proteins only
○ Show all proteins
☐ Filter by category: ABC Transporter

Proteins found: 627

Test	Cutoff
***q*-Value**	**.005**
p-Value	

Color	Signif	Direction	Applies To
red	yes	+	ratios, bars
yellow	no	n/a	bars
green	yes	−	ratios, bars
pink	yes	+	*p*- , *q*-Values
light green	yes	−	*p*- , *q*-Values

Dot Plots | Dot Plots

Protein	SgPgFn vs SgPg Log$_2$ Ratio	Log$_2$ Sum	*q*-Value	*p*-Value	Raw *SgPgFn*	Raw *SgPg*	Normalized *SgPgFn*	Normalized *SgPg*	Description
SGO_0652	0.499	4.282	0.1697	0.5840	3.500	6.500	6.4546	6.7909	hypothetical protein SGO_0652
					2.000	2.000	4.2052	2.0000	
SGO_0654	-0.541	6.872	0.0183	0.0237	14.500	30.000	26.7406	31.3426	radical SAM enzyme, Cfr family
					10.000	38.000	21.0259	38.0000	
SGO_0656	-0.070	4.817	0.2354	0.8955	5.500	7.500	10.1430	7.8356	trpB-2; tryptophan synthase, beta subunit
					2.000	6.000	4.2052	6.0000	
SGO_0665	-0.084	9.511	0.2049	0.7441	115.500	165.000	213.0028	172.3842	non-heme iron-containing ferritin
					68.500	200.000	144.0277	200.0000	
SGO_0669	-0.054	7.888	0.0984	0.2826	30.000	57.500	55.3254	60.0733	typA; GTP-binding protein TypA
					29.000	60.500	60.9752	60.5000	
SGO_0671	-1.181	6.393	0.0058	0.0033	6.500	31.000	11.9872	32.3873	murD; UDP-N-acetylmuramoylalanine--D-glutamate ligase
					6.500	26.000	13.6669	26.0000	
SGO_0672	-1.747	4.555	0.1156	0.3479	1.500	5.500	2.7663	5.7461	murG; undecaprenyl-PP-MurNAc-pentapeptide-UDPGlcNAc GlcNAc transferase
						15.000		15.0000	
SGO_0673	0.012	2.877			2.000	3.500	3.6884	3.6566	DivIB; cell division protein DivIB
SGO_0674	0.070	8.796	0.0972	0.2773	58.500	107.500	107.8845	112.3109	ftsA; cell division protein FtsA
					57.000	104.500	119.8478	104.5000	
SGO_0675	0.353	9.672	0.0028	0.0006	121.000	171.500	223.1458	179.1750	ftsZ; cell division protein FtsZ
					111.500	179.000	234.4392	179.0000	
SGO_0676	-0.215	5.555	0.0878	0.2400	6.000	10.000	11.0651	10.4475	conserved hypothetical protein TIGR00044
					5.000	15.000	10.5130	15.0000	
SGO_0677	-0.108	7.445	0.2139	0.7877	29.000	37.500	53.4812	39.1782	ylmF protein
					15.000	50.000	31.5389	50.0000	

Log$_2$ Ratios: -6 -4 -2 0 2 4 6

Show detected proteins only
Show all proteins
Filter by category: ABC Transporter

Proteins found: 627

Test	Cutoff
q-Value	.005
p-Value	

	Signif	Direction	Applies To
red	yes	+	ratios, bars
yellow	no	n/a	bars
green	yes	−	ratios, bars
pink	yes	+	*p*- , *q*-Values
light green	yes	−	*p*- , *q*-Values

Dot Plots | Dot Plots

SgPgFn vs SgPg

Streptococcus gordonii

Hackett Laboratory UW

Summary Table | SgFn vs Sg | SgPg vs Sg | SgPgFn vs Sg | SgPg vs SgFn | SgPgFn vs SgFn | SgPgFn vs SgPg | Coverage

Protein	SgPgFn vs SgPg Log_2 Ratio	Log_2 Sum	*q*-Value	*p*-Value	Raw *SgPgFn*	Raw *SgPg*	Normalized *SgPgFn*	Normalized *SgPg*	Description
SGO_0680	-0.460	8.674	0.0226	0.0338	41.000	103.000	75.6114	107.6095	cell division protein DivIVA
					46.000	128.500	96.7193	128.5000	
SGO_0681	-0.526	8.524	0.0026	0.0005	42.500	106.000	78.3776	110.7438	ileS; isoleucyl-tRNA synthetase
					34.500	106.500	72.5395	106.5000	
SGO_0684	0.708	7.087	0.0016	0.0002	23.500	25.000	43.3382	26.1188	hypothetical protein SGO_0684
					19.500	25.500	41.0006	25.5000	
SGO_0688	-0.500	7.374	0.0031	0.0007	19.000	48.000	35.0394	50.1481	ATP dependent Clp protease, ATP-binding subunit, ClpE
					16.000	47.000	33.6415	47.0000	
SGO_0693	0.248	3.226	0.2508	0.9789		4.500		4.7014	xseA; exodeoxyribonuclease VII, large subunit
					1.500	1.500	3.1539	1.5000	
SGO_0700	-0.692	5.746	0.0354	0.0673	4.000	14.000	7.3767	14.6265	DegV family protein
					6.500	18.000	13.6669	18.0000	
SGO_0701	-0.065	12.543	0.1866	0.6611	898.500	1475.000	1656.9957	1541.0098	hup; DNA-binding histone-like protein HU
					605.000	1496.500	1272.0691	1496.5000	
SGO_0704	0.640	11.588	0.0012	0.0001	501.500	559.500	924.8563	584.5390	gpmA; 2,3-bisphosphoglycerate-dependent phosphoglycerate mutase
					452.000	619.500	950.3723	619.5000	
SGO_0706	-0.152	4.568	0.0575	0.1368		8.000		8.3580	phoH-like protein
					3.500	8.000	7.3591	8.0000	
SGO_0707	0.154	4.307	0.1982	0.7097	4.000	6.000	7.3767	6.2685	LPXTG cell wall surface protein
					1.500	3.000	3.1539	3.0000	
SGO_0708	0.009	9.410	0.2272	0.8553	83.500	162.000	153.9890	169.2499	ald; alanine dehydrogenase
					89.500	169.000	188.1821	169.0000	
SGO_0713	-0.181	6.841	0.0893	0.2463	16.000	32.500	29.5069	33.9545	sgg; GTP-binding protein Era
					11.500	27.000	24.1798	27.0000	

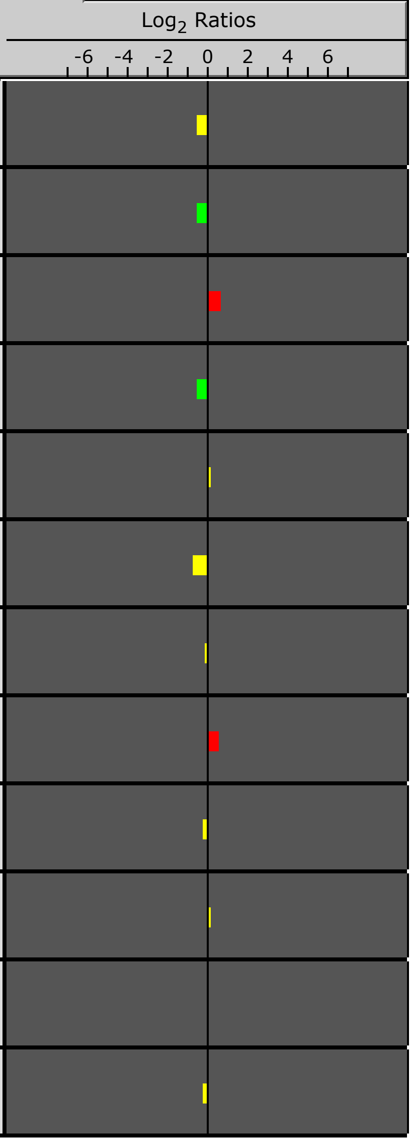

- Show detected proteins only
- Show all proteins
- Filter by category: ABC Transporter

Proteins found: 627

Test	Cutoff
q-Value	.005
p-Value	

	Signif	Direction	Applies To
red	yes	+	ratios, bars
yellow	no	n/a	bars
green	yes	−	ratios, bars
pink	yes	+	*p*- , *q*-Values
light green	yes	−	

Dot Plots Dot Plots

Hendrickson *et al.*

Streptococcus gordonii

Hackett Laboratory UW

Protein	*SgPgFn* vs *SgPg* Log$_2$ Ratio	Log$_2$ Sum	*q*-Value	*p*-Value	Raw *SgPgFn*	Raw *SgPg*	Normalized *SgPgFn*	Normalized *SgPg*	Description	Log$_2$ Ratios (-6 -4 -2 0 2 4 6)
**SGO_0721**	0.174	5.042	0.1577	0.5139	7.000	6.000	12.9093	6.2685	abpB-like dipeptidase lipoprotein	
					2.500	8.500	5.2565	8.5000		
**SGO_0736**	-0.914	3.749	0.0956	0.2697	1.500	4.000	2.7663	4.1790	hprK; HPr(Ser) kinase/phosphatase	
						6.500		6.5000		
**SGO_0739**	-0.130	5.795	0.0902	0.2502	7.500	12.500	13.8313	13.0594	hypothetical protein SGO_0739	
					6.000	16.000	12.6156	16.0000		
**SGO_0742**	-0.174	5.394	0.0184	0.0243	7.000	14.000	12.9093	14.6265	peptidase, U32 family	
						14.500		14.5000		
**SGO_0743**	-0.062	6.660	0.2296	0.8666	10.500	28.000	19.3639	29.2531	peptidase, U32 family	
					14.500	22.000	30.4876	22.0000		
**SGO_0745**	-0.380	7.571	0.0455	0.0988	27.000	48.500	49.7929	50.6705	hypothetical protein SGO_0745	
					16.000	56.000	33.6415	56.0000		
**SGO_0749**	-0.672	6.517	0.0067	0.0042	10.000	29.000	18.4418	30.2978	glutathione reductase	
					8.000	26.000	16.8207	26.0000		
**SGO_0750**	-0.828 [green]	8.970	0.0037 [light green]	0.0010	52.500	160.000	96.8194	167.1604	efflux transporter, RND family, MFP subunit subfamily	
					40.000	153.500	84.1037	153.5000		
**SGO_0751**	0.061	7.986	0.1979	0.7045	37.500	67.500	69.1567	70.5208	ABC transporter, ATP-binding protein SP0786	
					28.500	54.000	59.9239	54.0000		
**SGO_0752**	-1.583	5.463	0.0073	0.0049		18.000		18.8055	ABC transporter, ATP-binding protein	
					3.000	19.000	6.3078	19.0000		
**SGO_0753**	-0.152	8.582	0.0514	0.1174	46.000	101.500	84.8323	106.0424	lysS; lysyl-tRNA synthetase	
					46.000	95.500	96.7193	95.5000		
**SGO_0755**	-0.930	4.679	0.0843	0.2256		8.000		8.3580	regulatory protein	
					2.500	12.000	5.2565	12.0000		

Show detected proteins only
Show all proteins
Filter by category:
ABC Transporter

Proteins found:
627

Test	Cutoff
***q*-Value**	**.005**
p-Value	

	Signif	Direction	Applies To
red	yes	+	ratios, bars
yellow	no	n/a	bars
green	yes	−	ratios, bars
pink	yes	+	*p*- , *q*-Values
light green	yes	−	*p*- , *q*-Values

Dot Plots Dot Plots

Protein	SgPgFn vs SgPg Log_2 Ratio	Log_2 Sum	*q*-Value	*p*-Value	Raw *SgPgFn*	Raw *SgPg*	Normalized *SgPgFn*	Normalized *SgPg*	Description
SGO_0760	-0.184	8.944	0.0275	0.0469	67.000	125.500	123.5601	131.1164	ppc; phosphoenolpyruvate carboxylase
					51.000	130.500	107.2323	130.5000	
SGO_0761	0.194	13.234	0.0122	0.0126	1410.000	2246.500	2600.2938	2347.0363	tuf; translation elongation factor Tu
					1207.500	2149.500	2538.8817	2149.5000	
SGO_0762	0.307	11.217	0.0902	0.2505	436.000	581.000	804.0625	607.0012	tpiA; triosephosphate isomerase
					247.000	449.500	519.3406	449.5000	
SGO_0763	-0.462	6.864	0.0656	0.1614	17.500	29.500	32.2731	30.8202	murA-1; UDP-N-acetylglucosamine 1-carboxyvinyltransferase
					8.500	35.500	17.8720	35.5000	
SGO_0771	-0.580	8.022	0.0012	0.0001	28.000	73.000	51.6370	76.2669	pepq; proline dipeptidase
					25.000	79.500	52.5648	79.5000	
SGO_0773	-0.086	8.013	0.1168	0.3541	32.000	68.500	59.0138	71.5655	ccpA; catabolite control protein A
					31.500	61.500	66.2317	61.5000	
SGO_0774	-0.279	5.141	0.1579	0.5182	5.500	14.500	10.1430	15.1489	glycosyl transferase, group 1 family protein
						10.000		10.0000	
SGO_0775	-0.443	5.309	0.0248	0.0395	4.500	9.500	8.2988	9.9251	glycosyl transferase, group 1
					4.000	13.000	8.4104	13.0000	
SGO_0778	-0.280	8.523	0.0045	0.0014	45.000	94.000	82.9881	98.2067	thrS; threonyl-tRNA synthetase
					39.500	103.500	83.0524	103.5000	
SGO_0779	-1.405	5.902	0.0158	0.0186	6.500	23.500	11.9872	24.5517	response regulator
					2.500	18.000	5.2565	18.0000	
SGO_0784	-1.117	6.222	0.0007	0.0000	6.500	24.000	11.9872	25.0741	smc; chromosome segregation protein SMC
					5.500	26.000	11.5643	26.0000	
SGO_0786	-0.423	5.701	0.0238	0.0369	7.000	14.500	12.9093	15.1489	Cof family protein
					4.500	14.500	9.4617	14.5000	

Log_2 Ratios: -6 -4 -2 0 2 4 6

Show detected proteins only
Show all proteins
Filter by category:
ABC Transporter

Proteins found: 627

Test	Cutoff
q-Value	.005
p-Value	

	Signif	Direction	Applies To
red	yes	+	ratios, bars
yellow	no	n/a	bars
green	yes	−	ratios, bars
pink	yes	+	*p*- , *q*-Values
light green	yes	−	*p*- , *q*-Values

Dot Plots Dot Plots

Protein	*SgPgFn vs SgPg* Log_2 Ratio	Log_2 Sum	*q*-Value	*p*-Value	Raw *SgPgFn*	Raw *SgPg*	Normalized *SgPgFn*	Normalized *SgPg*	Description
**SGO_0787**	-0.680	6.644	0.0078	0.0056	9.000	29.500	16.5976	30.8202	ftsY; cell division protein FtsY
					10.500	30.500	22.0772	30.5000	
**SGO_0788**	-0.083	7.373	0.0165	0.0200	22.000	40.000	40.5720	41.7901	zwf; glucose-6-phosphate 1-dehydrogenase
					19.000	43.500	39.9493	43.5000	
**SGO_0792**	0.213	6.515	0.0667	0.1684	14.000	23.000	25.8185	24.0293	hypothetical protein SGO_0792
					11.000	18.500	23.1285	18.5000	
**SGO_0794**	-0.543	7.003	0.0048	0.0017	13.500	35.000	24.8964	36.5663	metallo-beta-lactamase family protein
					13.000	39.500	27.3337	39.5000	
**SGO_0795**	-0.846	4.916	0.0016	0.0002	3.000	9.000	5.5325	9.4028	tributyrin esterase
					2.500	10.000	5.2565	10.0000	
**SGO_0798**	-0.090	6.447	0.0451	0.0973	11.500	22.500	21.2081	23.5069	ABC transporter, ATP-binding protein SP1381
					10.000	21.500	21.0259	21.5000	
**SGO_0800**	-0.289	4.477	0.0893	0.2470	3.500	7.000	6.4546	7.3133	polysaccharide deacetylase family protein
						8.500		8.5000	
**SGO_0801**	-0.444	8.709	0.0357	0.0679	58.500	111.500	107.8845	116.4899	hom; homoserine dehydrogenase
					34.000	122.500	71.4882	122.5000	
**SGO_0802**	-0.990	6.198	0.0056	0.0030	5.500	23.000	10.1430	24.0293	thrB; homoserine kinase
					7.000	24.500	14.7182	24.5000	
**SGO_0803**	-0.985	5.780	0.0183	0.0238	7.000	16.500	12.9093	17.2384	hypothetical protein SGO_0803
					3.000	18.500	6.3078	18.5000	
**SGO_0814**	-0.439	3.119			2.000		3.6884		aminotransferase, class-V
						5.000		5.0000	
**SGO_0815**	-0.436	6.739	0.0128	0.0137	11.500	31.500	21.2081	32.9097	thiI; thiamine biosynthesis protein ThiI
					11.500	28.500	24.1798	28.5000	

Log_2 Ratios: -6 -4 -2 0 2 4 6

- Show detected proteins only
- Show all proteins
- Filter by category: ABC Transporter

Proteins found: 627

Test	Cutoff
***q*-Value**	**.005**
p-Value	

	Signif	Direction	Applies To
red	yes	+	ratios, bars
yellow	no	n/a	bars
green	yes	−	ratios, bars
pink	yes	+	*p*- , *q*-Values
light green	yes	−	*p*- , *q*-Values

Dot Plots Dot Plots

Hendrickson *et al.*

Protein	SgPgFn vs SgPg Log$_2$ Ratio	Log$_2$ Sum	*q*-Value	*p*-Value	Raw *SgPgFn*	Raw *SgPg*	Normalized *SgPgFn*	Normalized *SgPg*	Description
SGO_0818	0.146	9.821	0.0805	0.2119	118.000	190.500	217.6132	199.0253	rplU; ribosomal protein L21
					122.500	230.000	257.5677	230.0000	
SGO_0820	0.049	7.356	0.1989	0.7149	28.000	35.000	51.6370	36.5663	rpmA; ribosomal protein L27
					15.500	43.000	32.5902	43.0000	
SGO_0824	-0.527	7.513	0.0019	0.0003	19.500	51.500	35.9615	53.8047	lepA; GTP-binding protein LepA
					18.500	54.000	38.8980	54.0000	
SGO_0832	-0.202	4.135	0.1685	0.5790		7.000		7.3133	hypothetical protein SGO_0832
					2.500	5.000	5.2565	5.0000	
SGO_0835	-0.100	8.008	0.1676	0.5747	28.500	63.500	52.5591	66.3418	nitroreductase
					34.500	66.000	72.5395	66.0000	
SGO_0836	-0.322	8.917	0.0227	0.0344	65.000	130.000	119.8717	135.8178	pepV; dipeptidase PepV
					45.500	132.000	95.6680	132.0000	
SGO_0842	0.314	4.316	0.1271	0.3929	3.000	3.000	5.5325	3.1343	rhodanese family protein
					2.500	6.000	5.2565	6.0000	
SGO_0848	0.126	8.471	0.0199	0.0280	52.000	82.000	95.8974	85.6697	rpmE; ribosomal protein L31
					42.500	84.000	89.3602	84.0000	
SGO_0850	0.821	8.115	0.0532	0.1238	68.500	48.500	126.3263	50.6705	flavodoxin
					26.500	44.500	55.7187	44.5000	
SGO_0854	-0.083	4.618	0.1668	0.5698	3.000	5.000	5.5325	5.2238	cshA; surface-associated protein CshA
					3.000	7.500	6.3078	7.5000	
SGO_0856	-0.843	6.741	0.0099	0.0084	11.000	29.000	20.2860	30.2978	ABC transporter, substrate binding protein
					8.500	38.500	17.8720	38.5000	
SGO_0859	-0.591	6.628	0.0101	0.0091	9.500	30.000	17.5197	31.3426	pheS; phenylalanyl-tRNA synthetase, alpha subunit
					10.500	28.000	22.0772	28.0000	

Log$_2$ Ratios: -6 -4 -2 0 2 4 6

Show detected proteins only
Show all proteins
Filter by category:
ABC Transporter

Proteins found: 627

Test	Cutoff
q-Value	.005
p-Value	

	Signif	Direction	Applies To
red	yes	+	ratios, bars
yellow	no	n/a	bars
green	yes	−	ratios, bars
pink	yes	+	*p*- , *q*-Values
light green	yes	−	*p*- , *q*-Values

Dot Plots Dot Plots

Protein	*SgPgFn* vs *SgPg*				Raw		Normalized		Description
	Log_2 Ratio	Log_2 Sum	*q*-Value	*p*-Value	*SgPgFn*	*SgPg*	*SgPgFn*	*SgPg*	
**SGO_0861**	-0.016	8.788	0.2193	0.8146	56.000	103.500	103.2741	108.1319	pheT; phenylalanyl-tRNA synthetase, beta subunit
					55.500	114.000	116.6939	114.0000	
**SGO_0885**	0.072	4.529	0.1023	0.2951	3.000	5.500	5.5325	5.7461	cobyric acid synthase
					3.000	5.500	6.3078	5.5000	
**SGO_0888**	-0.120	3.360	0.2049	0.7437		2.500		2.6119	hypothetical protein SGO_0888
					1.500	4.500	3.1539	4.5000	
**SGO_0889**	-0.857	6.334	0.0094	0.0079	9.500	24.000	17.5197	25.0741	glmM; phosphoglucosamine mutase
					5.500	26.500	11.5643	26.5000	
**SGO_0893**	-0.144	7.478	0.0694	0.1766	23.000	40.500	42.4161	42.3125	GTP-binding protein
					20.000	51.500	42.0519	51.5000	
**SGO_0901**	-1.239	5.624	0.0092	0.0073	3.000	18.000	5.5325	18.8055	DNA-directed DNA polymerase III
					4.500	15.500	9.4617	15.5000	
**SGO_0906**	-0.169	7.067	0.1085	0.3157	14.500	34.000	26.7406	35.5216	leuA; 2-isopropylmalate synthase
					17.500	35.000	36.7954	35.0000	
**SGO_0911**	-0.375	8.145	0.0196	0.0271	29.500	75.000	54.4033	78.3564	hypothetical protein SGO_0911
					33.000	81.000	69.3856	81.0000	
**SGO_0946**	-0.405	5.997	0.0811	0.2141	5.500	19.000	10.1430	19.8503	Deblocking aminopeptidase
					8.500	16.000	17.8720	16.0000	
**SGO_0949**	-0.280	3.985	0.0869	0.2354	2.500	5.000	4.6104	5.2238	deaD; DEAD RNA helicase
						6.000		6.0000	
**SGO_0950**	-0.499	3.856	0.1382	0.4363	2.000	6.500	3.6884	6.7909	oxidoreductase
						4.000		4.0000	
**SGO_0951**	0.056	5.347	0.1726	0.5963	5.000	9.000	9.2209	9.4028	udk; uridine kinase
					5.500	10.500	11.5643	10.5000	

Log_2 Ratios: -6 -4 -2 0 2 4 6

Show detected proteins only
Show all proteins
Filter by category:
ABC Transporter

Proteins found: 627

Test	Cutoff
***q*-Value**	**.005**
p-Value	

	Signif	Direction	Applies To
(red)	yes	+	ratios, bars
(yellow)	no	n/a	bars
(green)	yes	−	ratios, bars
(pink)	yes	+	*p*- , *q*-Values
(light green)	yes	−	

Dot Plots Dot Plots

Protein	*SgPgFn vs SgPg* Log_2 Ratio	Log_2 Sum	*q*-Value	*p*-Value	Raw *SgPgFn*	Raw *SgPg*	Normalized *SgPgFn*	Normalized *SgPg*	Description
**SGO_0954**	0.400	6.031	0.1104	0.3269	7.500	9.500	13.8313	9.9251	ATP-binding protein
					11.000	18.500	23.1285	18.5000	
**SGO_0982**	-1.448	5.968	0.0050	0.0022	4.500	19.500	8.2988	20.3727	amino acid ABC transporter, amino acid-binding protein
					4.000	25.500	8.4104	25.5000	
**SGO_0987**	0.146	7.404	0.1271	0.3950	24.000	32.500	44.2603	33.9545	metK; S-adenosylmethionine synthetase
					21.000	47.000	44.1545	47.0000	
**SGO_0993**	0.298	2.742			2.000		3.6884		GTP-binding protein HflX
						3.000		3.0000	
**SGO_0995**	-0.910	4.300	0.1485	0.4762	2.000	11.500	3.6884	12.0147	metallo-beta-lactamase superfamily protein
						4.000		4.0000	
**SGO_1001**	-0.363	7.652	0.0482	0.1077	20.000	48.500	36.8836	50.6705	apt; adenine phosphoribosyltransferase
					24.500	62.000	51.5135	62.0000	
**SGO_1005**	-0.311	5.052	0.0394	0.0786	4.000	10.000	7.3767	10.4475	Bcl-2 family protein
					3.500	8.000	7.3591	8.0000	
**SGO_1009**	-0.352	8.825	0.0358	0.0687	46.000	126.500	84.8323	132.1612	rfbA-1; glucose-1-phosphate thymidylyltransferase
					55.000	121.000	115.6426	121.0000	
**SGO_1010**	-0.004	7.284	0.2348	0.8913	21.500	42.500	39.6499	44.4020	rmlC; dTDP-4-keto-6-deoxyglucose-3,5-epimerase
					18.000	34.000	37.8467	34.0000	
**SGO_1011**	-0.510	7.930	0.0092	0.0074	25.500	73.000	47.0266	76.2669	rfbB-1; dTDP-glucose 4,6-dehydratase
					25.500	67.000	53.6161	67.0000	
**SGO_1012**	-0.408	8.489	0.0012	0.0001	41.000	98.500	75.6114	102.9081	galE-1; UDP-glucose 4-epimerase
					37.500	102.000	78.8473	102.0000	
**SGO_1013**	1.757	3.716			5.500		10.1430		Glycosyltransferase involved in cell wall biogenesis
						3.000		3.0000	

Log_2 Ratios: -6 -4 -2 0 2 4 6

Show detected proteins only
Show all proteins
Filter by category:
ABC Transporter

Proteins found: 627

Test	Cutoff
***q*-Value**	**.005**
p-Value	

	Signif	Direction	Applies To
(red)	yes	+	ratios, bars
(yellow)	no	n/a	bars
(green)	yes	–	ratios, bars
(pink)	yes	+	*p*- , *q*-Values
(light green)	yes	–	*p*- , *q*-Values

Dot Plots | Dot Plots

Hendrickson *et al.*

Protein	SgPgFn vs SgPg				Raw		Normalized		Description
	Log_2 Ratio	Log_2 Sum	*q*-Value	*p*-Value	*SgPgFn*	*SgPg*	*SgPgFn*	*SgPg*	
SGO_1016	-0.407	4.079	0.0910	0.2534	2.500	6.500	4.6104	6.7909	putative glycosyltransferase
						5.500		5.5000	
SGO_1019	-0.856	3.694	0.0874	0.2378	1.500	4.000	2.7663	4.1790	glycosyl transferase
						6.000		6.0000	
SGO_1020	-0.143	7.350	0.1361	0.4272	23.000	34.000	42.4161	35.5216	rfbD; dTDP-4-dehydrorhamnose reductase
					16.500	50.500	34.6928	50.5000	
SGO_1025	-0.558	5.938	0.0438	0.0934	8.500	15.000	15.6755	15.6713	rgp; glycosyltransferase
					4.500	20.500	9.4617	20.5000	
SGO_1026	-1.030	5.584	0.0017	0.0002	4.000	15.000	7.3767	15.6713	rhamnosyltransferase
					4.000	16.500	8.4104	16.5000	
SGO_1031	-0.922	4.825	0.0158	0.0184	2.000	8.000	3.6884	8.3580	cmk; cytidylate kinase
					3.000	10.000	6.3078	10.0000	
SGO_1032	-0.215	4.744	0.1382	0.4357	3.000	10.000	5.5325	10.4475	infC; translation initiation factor IF-3
					3.000	4.500	6.3078	4.5000	
SGO_1034	-0.702	9.592	0.0321	0.0574	108.000	222.000	199.1714	231.9350	rplT; ribosomal protein L20
					49.500	236.500	104.0784	236.5000	
SGO_1035	-0.089	5.418	0.1403	0.4457	5.000	10.500	9.2209	10.9699	gloA; lactoylglutathione lyase
					5.500	11.000	11.5643	11.0000	
SGO_1036	-1.118	5.294	0.0358	0.0685	3.000	8.500	5.5325	8.8804	amino acid ABC transporter, ATP-binding protein SP1242
					3.000	18.500	6.3078	18.5000	
SGO_1038	0.018	4.993	0.2552	0.9977	3.500	9.500	6.4546	9.9251	uvrB; excinuclease ABC, B subunit
					4.500	6.000	9.4617	6.0000	
SGO_1047	-1.205	5.745	0.0107	0.0100	5.500	15.500	10.1430	16.1937	hypothetical protein SGO_1047
					3.000	21.000	6.3078	21.0000	

Log_2 Ratios: -6 -4 -2 0 2 4 6

Show detected proteins only
Show all proteins
Filter by category:
ABC Transporter

Proteins found:
627

Test	Cutoff
q-Value	.005
p-Value	

	Signif	Direction	Applies To
red	yes	+	ratios, bars
yellow	no	n/a	bars
green	yes	–	ratios, bars
pink	yes	+	*p*- , *q*-Values
light green	yes	–	*p*- , *q*-Values

Dot Plots Dot Plots

Streptococcus gordonii

Hackett Laboratory UW

Protein	*SgPgFn* vs *SgPg* Log$_2$ Ratio	Log$_2$ Sum	*q*-Value	*p*-Value	Raw *SgPgFn*	Raw *SgPg*	Normalized *SgPgFn*	Normalized *SgPg*	Description
SGO_1049	-1.108	4.615	0.0348	0.0652	2.500	9.000	4.6104	9.4028	tRNA pseudouridine synthase B
						10.500		10.5000	
SGO_1050	-0.267	3.894	0.1782	0.6234		3.500		3.6566	ribF; riboflavin biosynthesis protein RibF
					2.000	7.000	4.2052	7.0000	
SGO_1051	-1.595	3.476			1.500	8.000	2.7663	8.3580	negative regulator of proteolysis
SGO_1058	1.220	5.099	0.0073	0.0050	10.000	7.500	18.4418	7.8356	pstB; Phosphate import ATP-binding protein pstB 2 (Phosphate-transporting ATPase 2) (ABC phosphate transporter 2)
						8.000		8.0000	
SGO_1059	0.304	6.760	0.0048	0.0016	16.500	22.500	30.4290	23.5069	pstB; Phosphate import ATP-binding protein pstB 1 (Phosphate-transporting ATPase 1) (ABC phosphate transporter 1)
					14.000	25.000	29.4363	25.0000	
SGO_1060	-1.042	6.723	0.0043	0.0012	9.000	36.500	16.5976	38.1335	phosphate transport system regulatory protein
					8.500	33.000	17.8720	33.0000	
SGO_1065	-0.056	5.401	0.1849	0.6538	5.000	10.500	9.2209	10.9699	hypothetical protein SGO_1065
					5.500	10.500	11.5643	10.5000	
SGO_1069	-0.344	8.334	0.0248	0.0395	36.500	95.000	67.3126	99.2515	membrane alanyl aminopeptidase
					35.500	81.500	74.6421	81.5000	
SGO_1079	-0.816	6.258	0.0107	0.0101	7.000	20.500	12.9093	21.4174	pdp; pyrimidine-nucleoside phosphorylase
					7.000	27.500	14.7182	27.5000	
SGO_1080	-0.354	7.838	0.0064	0.0039	26.500	64.000	48.8708	66.8642	deoC; deoxyribose-phosphate aldolase
					24.500	61.500	51.5135	61.5000	
SGO_1081	1.033	3.484	0.0735	0.1892	3.000	3.500	5.5325	3.6566	cdd; cytidine deaminase
						2.000		2.0000	
SGO_1082	-0.212	12.037	0.1609	0.5401	690.500	1026.000	1273.4063	1071.9160	lipoprotein
					340.500	1141.000	715.9331	1141.0000	

Log$_2$ Ratios

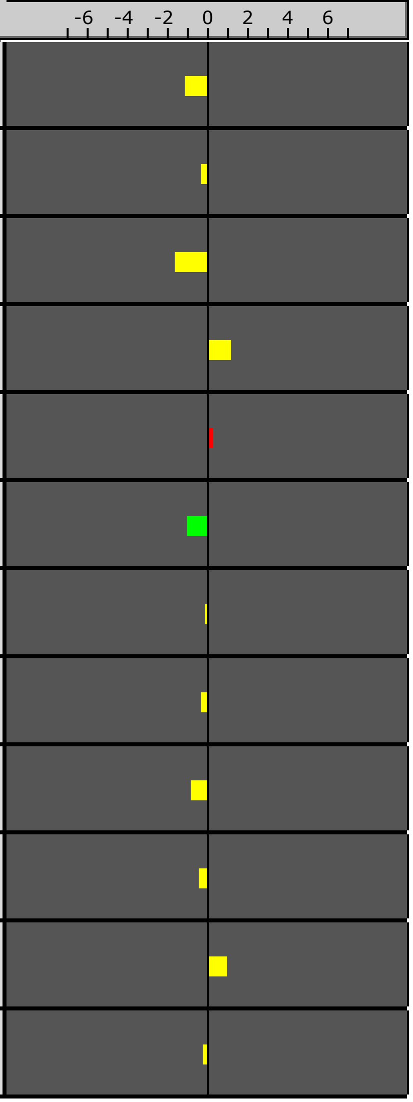

Show detected proteins only
Show all proteins
Filter by category:
ABC Transporter

Proteins found: 627

Test	Cutoff
***q*-Value**	**.005**
p-Value	

	Signif	Direction	Applies To
(red)	yes	+	ratios, bars
(yellow)	no	n/a	bars
(green)	yes	−	ratios, bars
(pink)	yes	+	*p*- , *q*-Values
(light green)	yes	−	*p*- , *q*-Values

Dot Plots Dot Plots

Hendrickson *et al.*

Protein	SgPgFn vs SgPg				Raw		Normalized		Description	Log_2 Ratios
	Log_2 Ratio	Log_2 Sum	*q*-Value	*p*-Value	*SgPgFn*	*SgPg*	*SgPgFn*	*SgPg*		-6 -4 -2 0 2 4 6
**SGO_1083**	-0.634	8.825	0.0148	0.0168	48.000	149.500	88.5206	156.1905	sugar ABC transporter, ATP-binding protein SP0846	
					42.000	120.500	88.3089	120.5000		
**SGO_1088**	-0.517	3.458	0.1390	0.4396	1.500	5.000	2.7663	5.2238	transcription regulator, LysR family	
						3.000		3.0000		
**SGO_1096**	-0.546	10.083	0.0167	0.0208	140.000	295.000	258.1852	308.2020	butA; acetoin dehydrogenase	
					88.500	332.500	186.0795	332.5000		
**SGO_1098**	-0.908	5.326	0.0271	0.0453	4.000	9.500	7.3767	9.9251	proA; gamma-glutamyl phosphate reductase	
					3.000	16.500	6.3078	16.5000		
**SGO_1107**	-0.387	3.279							PyrR bifunctional protein	
					2.000	5.500	4.2052	5.5000		
**SGO_1109**	-0.468	7.446	0.0077	0.0055	21.500	47.000	39.6499	49.1034	pyrB; aspartate carbamoyltransferase	
					16.000	52.000	33.6415	52.0000		
**SGO_1111**	-0.999	4.208	0.0466	0.1029	2.000	6.500	3.6884	6.7909	fruR; phosphotransferase system repressor	
						8.000		8.0000		
**SGO_1112**	-1.620	4.938	0.0735	0.1901		10.000		10.4475	fruB; 1-phosphofructokinase	
					2.000	16.000	4.2052	16.0000		
**SGO_1113**	0.070	7.700	0.2139	0.7865	26.000	58.000	47.9487	60.5956	fruA; PTS system, fructose specific IIABC components	
					27.500	41.500	57.8213	41.5000		
**SGO_1114**	-1.101	5.443	0.0051	0.0023	3.500	15.500	6.4546	16.1937	Protein of unknown function (DUF1149) superfamily	
					3.500	13.500	7.3591	13.5000		
**SGO_1116**	-0.461	7.063	0.0163	0.0196	13.500	39.000	24.8964	40.7453	dapB; dihydrodipicolinate reductase	
					15.000	36.500	31.5389	36.5000		
**SGO_1120**	-0.352	8.177	0.0319	0.0570	29.500	78.000	54.4033	81.4907	guaA; GMP synthase	
					35.000	80.000	73.5908	80.0000		

Show detected proteins only
Show all proteins
Filter by category:
ABC Transporter

Proteins found: 627

Test	Cutoff
***q*-Value**	**.005**
p-Value	

	Signif	Direction	Applies To
(red)	yes	+	ratios, bars
(yellow)	no	n/a	bars
(green)	yes	−	ratios, bars
(pink)	yes	+	*p*- , *q*-Values
(light green)	yes	−	

Dot Plots | Dot Plots

Hendrickson *et al.*

Protein	*SgPgFn vs SgPg* Log2 Ratio	Log2 Sum	*q*-Value	*p*-Value	Raw *SgPgFn*	Raw *SgPg*	Normalized *SgPgFn*	Normalized *SgPg*	Description
**SGO_1123**	-0.243	7.834	0.0198	0.0276	26.500	57.000	48.8708	59.5509	ffh; signal recognition particle protein
					26.500	64.000	55.7187	64.0000	
**SGO_1129**	0.133	5.556	0.1407	0.4477	7.000	12.500	12.9093	13.0594	lplA; lipoate protein ligase A
					5.500	9.500	11.5643	9.5000	
**SGO_1130**	-0.293	7.485	0.0045	0.0014	22.000	48.500	40.5720	50.6705	dihydrolipoamide dehydrogenase
					19.000	48.000	39.9493	48.0000	
**SGO_1131**	-0.628	6.083	0.0172	0.0217	6.000	21.000	11.0651	21.9398	sucB; dihydrolipoamide S-acetyltransferase
					7.500	19.000	15.7695	19.0000	
**SGO_1132**	-1.277	4.431	0.0092	0.0072	2.000	8.500	3.6884	8.8804	acetoin dehydrogenase
						9.000		9.0000	
**SGO_1133**	-0.401	5.511	0.0753	0.1952	4.000	13.500	7.3767	14.1042	acoA; acetoin dehydrogenase
					6.000	11.500	12.6156	11.5000	
**SGO_1134**	0.687	2.769				2.500		2.6119	hypothetical protein SGO_1134
					2.000		4.2052		
**SGO_1139**	-0.687	6.647	0.0017	0.0002	10.000	29.000	18.4418	30.2978	GTP-binding protein
					9.500	31.500	19.9746	31.5000	
**SGO_1140**	-0.548	8.083	0.0007	0.0000	29.500	77.500	54.4033	80.9683	clpX; ATP-dependent Clp protease, ATP-binding subunit ClpX
					26.500	80.000	55.7187	80.0000	
**SGO_1141**	-0.510	4.166	0.1108	0.3287	2.500	7.500	4.6104	7.8356	folA; dihydrofolate reductase
						5.500		5.5000	
**SGO_1143**	-0.469	5.119	0.0256	0.0415	5.000	12.000	9.2209	12.5370	thyA; thymidylate synthase
						13.000		13.0000	
**SGO_1144**	-0.205	9.487	0.0330	0.0604	98.500	184.000	181.6517	192.2344	glcK; glucokinase
					72.500	191.500	152.4380	191.5000	

Log2 Ratios: -6 -4 -2 0 2 4 6

Show detected proteins only
Show all proteins
Filter by category:
ABC Transporter

Proteins found: 627

Test	Cutoff
***q*-Value**	**.005**
p-Value	

	Signif	Direction	Applies To
red	yes	+	ratios, bars
yellow	no	n/a	bars
green	yes	−	ratios, bars
pink	yes	+	*p*- , *q*-Values
light green	yes	−	*p*- , *q*-Values

Dot Plots | Dot Plots

Hendrickson *et al.*

Protein	SgPgFn vs SgPg Log2 Ratio	Log2 Sum	q-Value	p-Value	Raw SgPgFn	Raw SgPg	Normalized SgPgFn	Normalized SgPg	Description
SGO_1150	-0.736	4.996	0.0458	0.1000		12.500		13.0594	hypothetical protein SGO_1150
					3.500	11.500	7.3591	11.5000	
SGO_1151	-0.112	9.374	0.0151	0.0172	84.000	165.500	154.9111	172.9065	glyA; serine hydroxymethyltransferase
					78.000	171.500	164.0023	171.5000	
SGO_1154	0.176	7.252	0.0328	0.0595	20.500	33.500	37.8057	34.9992	prfA; peptide chain release factor 1
					20.500	36.500	43.1032	36.5000	
SGO_1155	-0.062	5.189	0.2203	0.8197		9.000		9.4028	tdk; thymidine kinase
					5.500	15.500	11.5643	15.5000	
SGO_1167	-1.225	7.832	0.0061	0.0037	14.000	76.000	25.8185	79.4012	nox; NADH oxidase
					21.000	78.500	44.1545	78.5000	
SGO_1169	-0.074	7.287	0.1579	0.5196	20.500	44.000	37.8057	45.9691	NADPH-dependent FMN reductase
					18.000	34.500	37.8467	34.5000	
SGO_1170	-0.164	7.848	0.0268	0.0445	31.000	60.000	57.1696	62.6851	NADPH-dependent FMN reductase
					24.500	59.000	51.5135	59.0000	
SGO_1185	-0.678	4.825	0.0219	0.0318	2.500	9.500	4.6104	9.9251	acetyltransferase, GNAT family
					3.000	7.500	6.3078	7.5000	
SGO_1189	0.243	6.125	0.1382	0.4349	15.000	12.500	27.6627	13.0594	lipoprotein, putative
					5.500	17.500	11.5643	17.5000	
SGO_1191	0.592	11.525	0.0050	0.0022	454.000	580.500	837.2577	606.4788	rplL; ribosomal protein L7/L12
					444.500	568.000	934.6028	568.0000	
SGO_1192	-0.831	9.141	0.0196	0.0270	56.500	141.000	104.1962	147.3101	BL5; 50S ribosomal protein L10
					46.000	216.500	96.7193	216.5000	
SGO_1193	-1.780	5.724	0.0061	0.0036	3.000	22.500	5.5325	23.5069	gid; Glucose inhibited division protein A
					3.000	17.500	6.3078	17.5000	

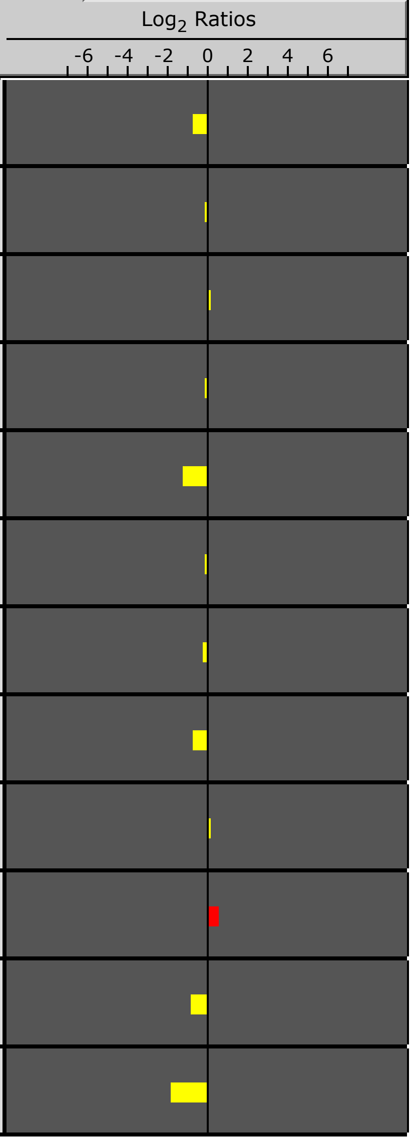

Show detected proteins only
Show all proteins
Filter by category:
ABC Transporter

Proteins found: 627

Test	Cutoff
q-Value	.005
p-Value	

Color	Signif	Direction	Applies To
red	yes	+	ratios, bars
yellow	no	n/a	bars
green	yes	−	ratios, bars
pink	yes	+	p- , q-Values
light green	yes	−	p- , q-Values

Dot Plots Dot Plots

Protein	SgPgFn vs SgPg Log2 Ratio	Log2 Sum	q-Value	p-Value	Raw SgPgFn	Raw SgPg	Normalized SgPgFn	Normalized SgPg	Description
SGO_1197	-0.539	6.562	0.0140	0.0154	10.000	24.000	18.4418	25.0741	topA; DNA topoisomerase I
					9.500	31.000	19.9746	31.0000	
SGO_1198	0.242	8.232	0.0289	0.0498	48.000	66.500	88.5206	69.4760	DNA processing Smf protein
					35.500	68.000	74.6421	68.0000	
SGO_1203	-0.311	6.275	0.0295	0.0509	8.500	19.000	15.6755	19.8503	anaerobic ribonucleotide reductase
					9.000	23.000	18.9233	23.0000	
SGO_1205	-0.173	4.641	0.1033	0.2994	3.500	5.500	6.4546	5.7461	dapA; dihydrodipicolinate synthase
					2.500	7.500	5.2565	7.5000	
SGO_1206	0.517	6.972	0.0053	0.0027	19.000	25.500	35.0394	26.6412	asd; aspartate-semialdehyde dehydrogenase
					18.500	25.000	38.8980	25.0000	
SGO_1210	-1.052	4.625	0.1103	0.3245	2.500	12.500	4.6104	13.0594	fhs-1; formate--tetrahydrofolate ligase
						7.000		7.0000	
SGO_1215	-0.577	9.730	0.0000	0.0000	92.500	243.000	170.5866	253.8748	manB; phosphomannomutase
					81.000	254.500	170.3101	254.5000	
SGO_1216	-0.641	7.060	0.0103	0.0094	14.500	43.000	26.7406	44.9244	bta; Possible bacteriocin transport accessory protein
					12.000	36.500	25.2311	36.5000	
SGO_1219	-0.147	7.143	0.0656	0.1621	16.500	36.000	30.4290	37.6111	pta; phosphate acetyltransferase
					17.500	36.500	36.7954	36.5000	
SGO_1224	-0.222	8.539	0.0384	0.0755	42.500	100.500	78.3776	104.9976	Ribose-phosphate pyrophosphokinase 2
					44.500	95.000	93.5654	95.0000	
SGO_1225	-0.082	5.027	0.1579	0.5157	4.000	7.000	7.3767	7.3133	pyridoxal-phosphate dependent aminotransferase
					4.000	9.500	8.4104	9.5000	
SGO_1226	-0.205	6.039	0.0203	0.0288	8.000	16.000	14.7534	16.7160	hypothetical protein SGO_1226
					7.500	18.500	15.7695	18.5000	

Log2 Ratios: -6 -4 -2 0 2 4 6

Show detected proteins only
Show all proteins
Filter by category:
ABC Transporter

Proteins found: 627

Test	Cutoff
q-Value	.005
p-Value	

Color	Signif	Direction	Applies To
red	yes	+	ratios, bars
yellow	no	n/a	bars
green	yes	−	ratios, bars
pink	yes	+	p- , q-Values
light green	yes	−	p- , q-Values

Dot Plots Dot Plots

Streptococcus gordonii

Protein	*SgPgFn vs SgPg* Log$_2$ Ratio	Log$_2$ Sum	*q*-Value	*p*-Value	Raw *SgPgFn*	Raw *SgPg*	Normalized *SgPgFn*	Normalized *SgPg*	Description
**SGO_1231**	-0.544	6.626	0.0495	0.1114	10.500	21.000	19.3639	21.9398	gyrA; DNA gyrase, A subunit
					9.500	37.500	19.9746	37.5000	
**SGO_1232**	0.085	10.720	0.0113	0.0108	234.000	398.500	431.5381	416.3338	L-lactate dehydrogenase
					207.500	402.000	436.2882	402.0000	
**SGO_1234**	0.167	10.658	0.0048	0.0016	228.500	368.500	421.3951	384.9913	rpsA; 30S ribosomal protein S1
					206.000	376.000	433.1343	376.0000	
**SGO_1237**	0.449	6.187	0.0848	0.2281	7.500	13.500	13.8313	14.1042	hypothetical protein SGO_1237
					14.000	15.500	29.4363	15.5000	
**SGO_1238**	-0.343	6.447	0.0305	0.0530	9.000	23.500	16.5976	24.5517	ilvE; branched-chain amino acid aminotransferase
					10.500	24.000	22.0772	24.0000	
**SGO_1239**	-1.874	4.994	0.0012	0.0001	2.000	11.500	3.6884	12.0147	parC; DNA topoisomerase IV, A subunit
					1.500	13.000	3.1539	13.0000	
**SGO_1242**	0.352	3.887			4.500		8.2988		lipoprotein, putative
						6.500		6.5000	
**SGO_1248**	-1.332	5.511	0.0136	0.0148	3.500	19.000	6.4546	19.8503	pyrC; dihydroorotase
					3.000	13.000	6.3078	13.0000	
**SGO_1253**	-0.339	6.446	0.0353	0.0668	9.000	22.000	16.5976	22.9846	pyrE; orotate phosphoribosyltransferase
					10.500	25.500	22.0772	25.5000	
**SGO_1260**	0.001	7.787	0.2478	0.9628	32.000	51.000	59.0138	53.2824	deoD; purine nucleoside phosphorylase
					24.500	57.000	51.5135	57.0000	
**SGO_1263**	-0.766	8.178	0.0100	0.0088	24.000	84.000	44.2603	87.7592	purine nucleoside phosphorylase I, inosine and guanosine-specific
					30.500	93.500	64.1291	93.5000	
**SGO_1264**	-0.144	9.434	0.1271	0.3944	75.500	177.500	139.2356	185.4436	deoB; phosphopentomutase
					91.000	175.500	191.3360	175.5000	

Log$_2$ Ratios: -6 -4 -2 0 2 4 6

Show detected proteins only
Show all proteins
Filter by category:
ABC Transporter

Proteins found: 627

Test	Cutoff
***q*-Value**	**.005**
p-Value	

	Signif	Direction	Applies To
red	yes	+	ratios, bars
yellow	no	n/a	bars
green	yes	−	ratios, bars
pink	yes	+	*p*- , *q*-Values
light green	yes	−	*p*- , *q*-Values

Dot Plots Dot Plots

Hendrickson *et al.*

Streptococcus gordonii

Protein	SgPgFn vs SgPg Log2 Ratio	Log2 Sum	q-Value	p-Value	Raw SgPgFn	Raw SgPg	Normalized SgPgFn	Normalized SgPg	Description
SGO_1265	-0.281	7.570	0.0190	0.0257	21.500	48.000	39.6499	50.1481	rpiA; ribose 5-phosphate isomerase
					22.000	54.000	46.2571	54.0000	
SGO_1266	-0.319	5.932	0.0570	0.1353	8.500	18.000	15.6755	18.8055	trmE; tRNA modification GTPase TrmE
					5.500	15.000	11.5643	15.0000	
SGO_1273	0.084	8.669	0.1635	0.5535	68.500	93.000	126.3263	97.1620	rpoD; RNA polymerase sigma factor
					40.500	98.500	85.1550	98.5000	
SGO_1276	0.200	7.602	0.0874	0.2374	24.500	39.000	45.1824	40.7453	rpsU; ribosomal protein S21
					28.000	49.500	58.8726	49.5000	
SGO_1281	-1.109	4.494	0.0665	0.1670		7.500		7.8356	penicillinase repressor, putative
					2.000	10.500	4.2052	10.5000	
SGO_1283	-0.407	8.123	0.0016	0.0002	32.500	77.500	59.9358	80.9683	oxidoreductase
					28.500	78.000	59.9239	78.0000	
SGO_1284	-0.029	5.586	0.1982	0.7089	5.500	17.000	10.1430	17.7608	thioredoxin-disulfide reductase
					6.000	7.500	12.6156	7.5000	
SGO_1293	-0.362	7.357	0.0731	0.1879	15.000	46.500	27.6627	48.5810	asnS; asparaginyl-tRNA synthetase
					21.500	42.500	45.2058	42.5000	
SGO_1297	0.120	6.240	0.0340	0.0635	10.500	16.500	19.3639	17.2384	aspC; aspartate aminotransferase
					9.500	19.000	19.9746	19.0000	
SGO_1305	-0.947	5.196	0.0053	0.0026	4.000	11.500	7.3767	12.0147	substrate-binding protein MsmE
					2.500	12.000	5.2565	12.0000	
SGO_1312	-0.574	7.684	0.0079	0.0057	25.000	60.000	46.1045	62.6851	pepT; peptidase T
					17.500	60.000	36.7954	60.0000	
SGO_1323	0.733	7.891	0.0043	0.0012	41.500	39.500	76.5335	41.2677	rpsP; ribosomal protein S16
					34.000	48.000	71.4882	48.0000	

Log2 Ratios: -6 -4 -2 0 2 4 6

- Show detected proteins only
- Show all proteins
- Filter by category: ABC Transporter

Proteins found: 627

Test	Cutoff
q-Value	.005
p-Value	

	Signif	Direction	Applies To
red	yes	+	ratios, bars
yellow	no	n/a	bars
green	yes	−	ratios, bars
pink	yes	+	p- , q-Values
light green	yes	−	p- , q-Values

Dot Plots Dot Plots

Hendrickson *et al.*

Protein	SgPgFn vs SgPg Log_2 Ratio	Log_2 Sum	*q*-Value	*p*-Value	Raw *SgPgFn*	Raw *SgPg*	Normalized *SgPgFn*	Normalized *SgPg*	Description
SGO_1327	-0.917	5.317	0.0026	0.0005	3.500	12.000	6.4546	12.5370	HAD-superfamily subfamily IIA hydrolase, TIGR01457
					3.500	13.500	7.3591	13.5000	
SGO_1336	-0.793	5.338	0.0073	0.0051	3.500	13.000	6.4546	13.5818	pcrA; ATP-dependent DNA helicase PcrA
					4.000	12.000	8.4104	12.0000	
SGO_1339	-0.104	11.699	0.0426	0.0899	454.000	803.500	837.2577	839.4586	pyk; pyruvate kinase
					364.500	882.500	766.3954	882.5000	
SGO_1340	-0.128	9.949	0.0114	0.0111	124.500	246.000	229.6004	257.0091	Phosphofructokinase
					115.500	259.000	242.8496	259.0000	
SGO_1342	0.066	9.004	0.1095	0.3209	71.000	128.000	130.9368	133.7283	ABC transporter, ATP-binding protein SP1715
					62.500	117.500	131.4121	117.5000	
SGO_1364	-2.148	5.375	0.0143	0.0158		17.500		18.2832	rumA-2; 23S rRNA (uracil-5-)-methyltransferase RumA
					2.000	19.000	4.2052	19.0000	
SGO_1365	0.541	3.376	0.1526	0.4924		4.000		4.1790	transcription regulator yrfE
					2.000	2.000	4.2052	2.0000	
SGO_1369	-0.910	4.724	0.0565	0.1337	3.000	9.000	5.5325	9.4028	L-2-hydroxyisocaproate dehydrogenase
						11.500		11.5000	
SGO_1370	0.392	6.928	0.0178	0.0228	17.500	27.500	32.2731	28.7307	Protein of unknown function (DUF964) superfamily
					17.500	24.000	36.7954	24.0000	
SGO_1372	-0.629	5.106	0.0352	0.0663		12.000		12.5370	aroC; chorismate synthase
					4.000	13.500	8.4104	13.5000	
SGO_1373	-0.318	4.272	0.0284	0.0487	3.000	6.500	5.5325	6.7909	aroB; 3-dehydroquinate synthase
						7.000		7.0000	
SGO_1375	-0.556	5.207	0.1164	0.3516	5.000	16.000	9.2209	16.7160	aroD; 3-dehydroquinate dehydratase, type I
						11.000		11.0000	

Log2 Ratios: -6 -4 -2 0 2 4 6

- Show detected proteins only
- Show all proteins
- Filter by category: ABC Transporter

Proteins found: 627

Test	Cutoff
***q*-Value**	**.005**
p-Value	

	Signif	Direction	Applies To
red	yes	+	ratios, bars
yellow	no	n/a	bars
green	yes	−	ratios, bars
pink	yes	+	*p*- , *q*-Values
light green	yes	−	*p*- , *q*-Values

Dot Plots Dot Plots

Protein	SgPgFn vs SgPg Log2 Ratio	Log2 Sum	q-Value	p-Value	Raw SgPgFn	Raw SgPg	Normalized SgPgFn	Normalized SgPg	Description
SGO_1377	-1.576	5.577	0.0051	0.0025	4.500	16.500	8.2988	17.2384	sulfatase
					2.000	18.000	4.2052	18.0000	
SGO_1381	-0.400	5.525	0.0533	0.1243	5.500	10.000	10.1430	10.4475	csn1; CRISPR-associated protein, Csn1 family
					4.500	16.000	9.4617	16.0000	
SGO_1383	0.121	8.897	0.0576	0.1374	65.500	102.000	120.7938	106.5647	rplS; ribosomal protein L19
					60.500	122.000	127.2069	122.0000	
SGO_1390	0.065	4.991	0.1897	0.6735	5.500	8.000	10.1430	8.3580	ligA; DNA ligase, NAD-dependent
					3.000	7.000	6.3078	7.0000	
SGO_1397	-0.516	6.352	0.0165	0.0202	8.500	25.500	15.6755	26.6412	map; methionine aminopeptidase, type I
					8.500	21.500	17.8720	21.5000	
SGO_1400	-0.450	5.155	0.1586	0.5277	6.500	10.000	11.9872	10.4475	murA-2; UDP-N-acetylglucosamine 1-carboxyvinyltransferase
					2.000	9.000	4.2052	9.0000	
SGO_1414	1.446	4.504	0.0057	0.0031	5.000	2.500	9.2209	2.6119	rexB; putative exonuclease RexB
					3.500	3.500	7.3591	3.5000	
SGO_1422	0.752	6.562	0.0229	0.0351	13.000	17.000	23.9743	17.7608	hypothetical protein SGO_1422
					17.000	17.000	35.7441	17.0000	
SGO_1426	0.293	14.775	0.0037	0.0009	4114.000	5900.500	7586.9564	6164.5617	eno; enolase
					3729.500	6433.500	7841.6227	6433.5000	
SGO_1431	-0.341	8.748	0.0026	0.0005	51.500	117.500	94.9753	122.7584	EzrA; Septation ring formation regulator ezrA
					45.000	117.500	94.6167	117.5000	
SGO_1432	-0.502	7.053	0.0667	0.1681	19.500	31.000	35.9615	32.3873	gyrB; DNA gyrase, B subunit
					9.500	44.500	19.9746	44.5000	
SGO_1434	-0.345	3.738	0.1566	0.5087	2.000	3.500	3.6884	3.6566	thiJ; 4-methyl-5(beta-hydroxyethyl)-thiazole monophosphate synthesis protein
						6.000		6.0000	

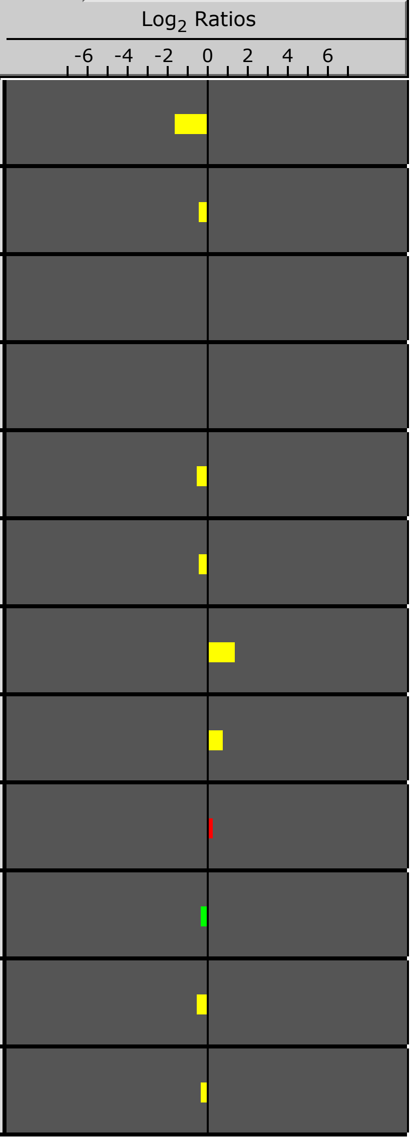

Show detected proteins only
Show all proteins
Filter by category:
ABC Transporter

Proteins found: 627

Test	Cutoff
q-Value	.005
p-Value	

	Signif	Direction	Applies To
red	yes	+	ratios, bars
yellow	no	n/a	bars
green	yes	–	ratios, bars
pink	yes	+	p- , q-Values
light green	yes	–	p- , q-Values

Dot Plots Dot Plots

Streptococcus gordonii

Hackett Laboratory UW

Protein	SgPgFn vs SgPg Log_2 Ratio	Log_2 Sum	*q*-Value	*p*-Value	Raw *SgPgFn*	Raw *SgPg*	Normalized *SgPgFn*	Normalized *SgPg*	Description	Log_2 Ratios (-6 -4 -2 0 2 4 6)
SGO_1439	0.801	5.885	0.0103	0.0094	9.000	11.000	16.5976	11.4923	ftsX; cell division protein FtsX	
					10.000	10.000	21.0259	10.0000		
SGO_1440	-0.761	6.532	0.0017	0.0002	9.500	27.000	17.5197	28.2083	cell-division ATP-binding protein FtsE	
					8.000	30.000	16.8207	30.0000		
SGO_1441	-0.331	6.072	0.1586	0.5279	11.500	13.500	21.2081	14.1042	prfB; peptide chain release factor 2	
					4.500	22.500	9.4617	22.5000		
SGO_1446	-2.303	5.644	0.0026	0.0006	3.000	18.500	5.5325	19.3279	murF; UDP-N-acetylmuramoyl-tripeptide--D-alanyl-D-alanine ligase	
					1.500	22.000	3.1539	22.0000		
SGO_1447	-0.928	6.423	0.0057	0.0032	8.000	29.500	14.7534	30.8202	ddlA; D-Ala-D-Ala ligase	
					7.000	25.500	14.7182	25.5000		
SGO_1450	-0.361	5.099	0.2139	0.7870	2.000	10.500	3.6884	10.9699	hypothetical protein SGO_1450	
					6.000	7.000	12.6156	7.0000		
SGO_1451	0.752	7.695	0.0032	0.0008	33.500	37.000	61.7800	38.6558	frr; ribosome recycling factor	
					32.500	38.500	68.3343	38.5000		
SGO_1452	-0.243	7.184	0.0848	0.2275	17.500	45.500	32.2731	47.5362	pyrH; uridylate kinase	
					16.000	32.000	33.6415	32.0000		
SGO_1453	0.282	5.393	0.1618	0.5445	10.000	11.500	18.4418	12.0147	glycosyl transferase, family 8 SP1766	
					5.500		11.5643			
SGO_1455	-0.007	10.022	0.2492	0.9697	117.500	208.000	216.6911	217.3085	rplA; ribosomal protein L1	
					143.500	304.000	301.7222	304.0000		
SGO_1456	0.777	8.133	0.0094	0.0079	45.500	41.500	83.9102	43.3572	rplK; ribosomal protein L11	
					44.000	61.000	92.5141	61.0000		
SGO_1458	0.287	3.299	0.1503	0.4830	2.000	3.500	3.6884	3.6566	aha1; cation-transporting ATPase yfgQ	
						2.500		2.5000		

Show detected proteins only
Show all proteins
Filter by category:
ABC Transporter

Proteins found: 627

Test	Cutoff
q-Value	.005
p-Value	

Color	Signif	Direction	Applies To
red	yes	+	ratios, bars
yellow	no	n/a	bars
green	yes	−	ratios, bars
pink	yes	+	*p*- , *q*-Values
light green	yes	−	*p*- , *q*-Values

Dot Plots Dot Plots

Hendrickson *et al.*

Protein	SgPgFn vs SgPg Log2 Ratio	Log2 Sum	q-Value	p-Value	Raw SgPgFn	Raw SgPg	Normalized SgPgFn	Normalized SgPg	Description
SGO_1460	-0.608	4.901	0.0251	0.0404	4.000	11.000	7.3767	11.4923	DNA translocase ftsK
						11.000		11.0000	
SGO_1463	-0.139	5.848	0.1013	0.2915	8.000	16.000	14.7534	16.7160	peptidyl-prolyl cis-trans isomerase
					6.000	13.500	12.6156	13.5000	
SGO_1464	-0.793	5.996	0.0050	0.0018	7.000	20.000	12.9093	20.8950	uncharacterized probable metal-binding protein
					5.000	19.500	10.5130	19.5000	
SGO_1465	-0.686	6.485	0.0158	0.0184	10.000	30.000	18.4418	31.3426	ABC transporter, ATP-binding protein SP0770
					7.500	24.000	15.7695	24.0000	
SGO_1469	-0.052	7.514	0.1588	0.5306	22.500	43.000	41.4940	44.9244	glmU; UDP-N-acetylglucosamine pyrophosphorylase
					23.000	48.000	48.3597	48.0000	
SGO_1472	-0.828	3.906	0.1256	0.3862		7.500		7.8356	acetyltransferase, GNAT family
					1.500	4.000	3.1539	4.0000	
SGO_1516	-0.925	5.776	0.0172	0.0219	5.000	14.000	9.2209	14.6265	lacD-2; tagatose 1,6-diphosphate aldolase
					4.500	21.500	9.4617	21.5000	
SGO_1530	-0.402	7.851	0.0073	0.0049	26.000	66.000	47.9487	68.9537	methionine-tRNA ligase
					24.500	62.500	51.5135	62.5000	
SGO_1531	-0.715	5.962	0.0212	0.0304	5.000	20.000	9.2209	20.8950	xth; exodeoxyribonuclease III
					7.000	17.500	14.7182	17.5000	
SGO_1534	0.195	5.182	0.2345	0.8886		15.500		16.1937	ArsC family
					6.000	7.500	12.6156	7.5000	
SGO_1536	-0.779	5.622	0.0396	0.0794	4.000	19.000	7.3767	19.8503	conserved hypothetical protein TIGR00096
					5.000	11.500	10.5130	11.5000	
SGO_1539	0.084	6.150	0.1727	0.5986	8.000	15.000	14.7534	15.6713	tmk; thymidylate kinase
					10.500	18.500	22.0772	18.5000	

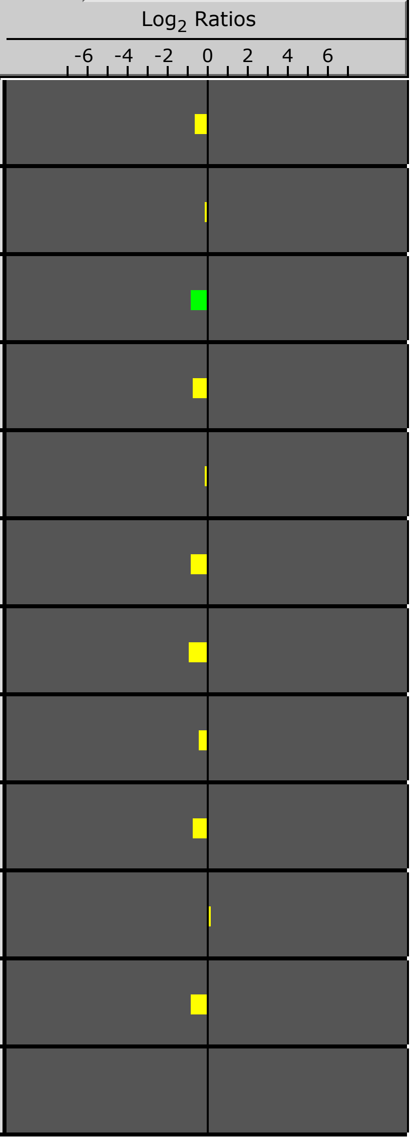

Show detected proteins only
Show all proteins
Filter by category:
ABC Transporter

Proteins found: 627

Test	Cutoff
q-Value	.005
p-Value	

	Signif	Direction	Applies To
red	yes	+	ratios, bars
yellow	no	n/a	bars
green	yes	–	ratios, bars
pink	yes	+	p- , q-Values
light green	yes	–	

Dot Plots Dot Plots

Hendrickson *et al.*

Protein	SgPgFn vs SgPg Log$_2$ Ratio	Log$_2$ Sum	*q*-Value	*p*-Value	Raw *SgPgFn*	Raw *SgPg*	Normalized *SgPgFn*	Normalized *SgPg*	Description
SGO_1541	-0.596	5.759	0.0271	0.0458	4.500	15.500	8.2988	16.1937	atpC; ATP synthase F1, epsilon subunit
					6.500	16.000	13.6669	16.0000	
SGO_1542	-0.014	9.171	0.2260	0.8480	73.000	134.000	134.6251	139.9968	atpD; ATP synthase F1, beta subunit
					72.500	149.500	152.4380	149.5000	
SGO_1543	-1.264	5.625	0.0026	0.0005	4.500	17.000	8.2988	17.7608	atpG; ATP synthase F1, gamma subunit
					3.000	17.000	6.3078	17.0000	
SGO_1544	0.051	9.229	0.1101	0.3231	79.000	137.500	145.6902	143.6535	atpA; ATP synthase F1, alpha subunit
					76.000	151.000	159.7971	151.0000	
SGO_1545	-0.805	5.751	0.0010	0.0001	5.500	16.500	10.1430	17.2384	atpH; ATP synthase F1, delta subunit
					4.500	17.000	9.4617	17.0000	
SGO_1546	-0.137	6.983	0.1164	0.3521	16.500	37.000	30.4290	38.6558	atpF; ATP synthase F0, B subunit
					14.000	28.000	29.4363	28.0000	
SGO_1550	-0.554	7.392	0.0092	0.0072	16.500	49.500	30.4290	51.7152	glgP-1; glycogen phosphorylase
					18.000	48.000	37.8467	48.0000	
SGO_1551	-0.272	5.395	0.1285	0.4007	4.000	14.000	7.3767	14.6265	glgA; Glycogen synthase
					5.500	8.500	11.5643	8.5000	
SGO_1552	0.226	7.345	0.1627	0.5498	27.500	47.000	50.7149	49.1034	glgD; glucose-1-phosphate adenylyltransferase, GlgD subunit
					17.000	27.000	35.7441	27.0000	
SGO_1553	-0.314	7.391	0.0050	0.0021	19.500	45.000	35.9615	47.0139	glgC; glucose-1-phosphate adenylyltransferase
					18.500	46.000	38.8980	46.0000	
SGO_1554	-0.014	7.510	0.2269	0.8526	23.000	44.500	42.4161	46.4915	glgB; 1,4-alpha-glucan branching enzyme
					23.000	45.000	48.3597	45.0000	
SGO_1555	-0.372	10.552	0.0100	0.0088	163.500	410.000	301.5234	428.3485	ptsI; phosphoenolpyruvate-protein phosphotransferase
					168.500	417.500	354.2870	417.5000	

Log$_2$ Ratios: -6 -4 -2 0 2 4 6

Show detected proteins only
Show all proteins
Filter by category:
ABC Transporter

Proteins found: 627

Test	Cutoff
q-Value	.005
p-Value	

	Signif	Direction	Applies To
red	yes	+	ratios, bars
yellow	no	n/a	bars
green	yes	−	ratios, bars
pink	yes	+	*p*- , *q*-Values
light green	yes	−	*p*- , *q*-Values

Dot Plots Dot Plots

Hendrickson *et al.*

Protein	*SgPgFn vs SgPg* Log$_2$ Ratio	Log$_2$ Sum	*q*-Value	*p*-Value	Raw *SgPgFn*	Raw *SgPg*	Normalized *SgPgFn*	Normalized *SgPg*	Description
**SGO_1556**	0.890	11.617	0.0158	0.0186	467.500	455.500	862.1541	475.8847	phosphocarrier protein HPr
					561.500	622.500	1180.6063	622.5000	
**SGO_1558**	-0.072	8.045	0.0087	0.0065	34.500	64.500	63.6242	67.3865	nrdE; ribonucleoside-diphosphate reductase large chain
					31.000	68.000	65.1804	68.0000	
**SGO_1559**	0.143	8.182	0.0200	0.0282	41.000	69.000	75.6114	72.0879	ribonucleoside-diphosphate reductase, beta subunit
					36.500	66.000	76.7447	66.0000	
**SGO_1570**	-0.417	8.885	0.0185	0.0248	50.000	139.500	92.2090	145.7430	alaS; alanyl-tRNA synthetase
					52.500	124.500	110.3862	124.5000	
**SGO_1574**	-0.272	8.868	0.0050	0.0020	55.500	123.500	102.3520	129.0269	pepF-1; oligoendopeptidase F
					52.000	126.500	109.3349	126.5000	
**SGO_1580**	-0.150	5.058	0.1529	0.4943	3.500	9.000	6.4546	9.4028	PTS system, Lactose/Cellobiose specific IIB subunit
					4.500	8.000	9.4617	8.0000	
**SGO_1585**	0.456	3.557	0.2327	0.8803		1.500		1.5671	D-Alanyl-D-Alanine carboxypeptidase
					2.000	6.000	4.2052	6.0000	
**SGO_1587**	-0.654	5.537	0.0426	0.0902	5.000	17.500	9.2209	18.2832	queA; S-adenosylmethionine:tRNA ribosyltransferase-isomerase
					4.000	10.500	8.4104	10.5000	
**SGO_1591**	-0.291	5.239	0.0865	0.2333	4.500	12.500	8.2988	13.0594	arcC; carbamate kinase
					4.000	8.000	8.4104	8.0000	
**SGO_1592**	0.316	8.455	0.0271	0.0457	58.500	74.500	107.8845	77.8341	arcB; ornithine carbamoyltransferase
					41.500	78.000	87.2576	78.0000	
**SGO_1593**	0.493	8.251	0.0030	0.0007	47.500	58.000	87.5985	60.5956	arcA; arginine deiminase
					43.000	66.000	90.4115	66.0000	
**SGO_1599**	-0.140	9.050	0.0520	0.1195	73.000	127.500	134.6251	133.2059	sodA; manganese-dependent superoxide dismutase
					56.000	144.500	117.7452	144.5000	

Log$_2$ Ratios: -6 -4 -2 0 2 4 6

Show detected proteins only
Show all proteins
Filter by category:
ABC Transporter

Proteins found: 627

Test	Cutoff
***q*-Value**	**.005**
p-Value	

	Signif	Direction	Applies To
red	yes	+	ratios, bars
yellow	no	n/a	bars
green	yes	–	ratios, bars
pink	yes	+	*p*- , *q*-Values
light green	yes	–	*p*- , *q*-Values

Dot Plots Dot Plots

Protein	*SgPgFn vs SgPg* Log$_2$ Ratio	Log$_2$ Sum	*q*-Value	*p*-Value	Raw *SgPgFn*	Raw *SgPg*	Normalized *SgPgFn*	Normalized *SgPg*	Description	Log$_2$ Ratios (-6 -4 -2 0 2 4 6)
**SGO_1604**	-0.886	3.902	0.0769	0.2010		6.500		6.7909	acyltransferase family protein	
					1.500	5.000	3.1539	5.0000		
**SGO_1605**	-1.322	4.245	0.0396	0.0791		7.000		7.3133	P-type ATPase, metal cation transport	
					1.500	8.500	3.1539	8.5000		
**SGO_1609**	-0.173	7.225	0.0366	0.0712	20.500	37.000	37.8057	38.6558	ATP-dependent RNA helicase, DEAD/DEAH box family	
					15.500	40.500	32.5902	40.5000		
**SGO_1617**	-0.226	5.887	0.1256	0.3856	6.500	11.000	11.9872	11.4923	prfC; peptide chain release factor 3	
					7.000	21.000	14.7182	21.0000		
**SGO_1619**	-0.025	8.040	0.2193	0.8120	38.000	73.000	70.0788	76.2669	cation-transporting ATPase, E1-E2 family	
					28.500	57.000	59.9239	57.0000		
**SGO_1621**	-1.273	4.429	0.0358	0.0691	2.000	8.000	3.6884	8.3580	HD domain protein	
						9.500		9.5000		
**SGO_1622**	0.319	5.077	0.0728	0.1866	4.000	7.000	7.3767	7.3133	Cof family protein	
					5.500	7.500	11.5643	7.5000		
**SGO_1623**	-0.623	5.081	0.0248	0.0397	4.500	12.500	8.2988	13.0594	murM; MurM	
						12.500		12.5000		
**SGO_1624**	0.326	4.258	0.0656	0.1623		6.000		6.2685	murN; MurN protein	
					3.500	5.500	7.3591	5.5000		
**SGO_1625**	-0.898	6.496	0.0094	0.0077	10.500	29.000	19.3639	30.2978	acetoin utilization putative/CBS domain protein	
					6.000	28.000	12.6156	28.0000		
**SGO_1626**	-0.006	6.193	0.2367	0.9031	9.500	16.000	17.5197	16.7160	branched-chain amino acid ABC transporter, ATP-binding protein	
					9.000	20.000	18.9233	20.0000		
**SGO_1627**	0.065	6.204	0.1828	0.6418	12.000	18.500	22.1302	19.3279	branched-chain amino acid ABC transporter, ATP-binding protein	
					7.500	16.500	15.7695	16.5000		

Show detected proteins only
Show all proteins
Filter by category:
ABC Transporter

Proteins found: 627

Test	Cutoff
***q*-Value**	**.005**
p-Value	

	Signif	Direction	Applies To
(red)	yes	+	ratios, bars
(yellow)	no	n/a	bars
(green)	yes	−	ratios, bars
(pink)	yes	+	*p*- , *q*-Values
(light green)	yes	−	*p*- , *q*-Values

Dot Plots Dot Plots

SgPgFn vs SgPg

Streptococcus gordonii

Hackett Laboratory UW

Protein	SgPgFn vs SgPg Log2 Ratio	Log2 Sum	q-Value	p-Value	Raw SgPgFn	Raw SgPg	Normalized SgPgFn	Normalized SgPg	Description
SGO_1630	-0.444	9.189	0.0012	0.0001	68.500	162.000	126.3263	169.2499	branched-chain amino acid ABC transporter, amino acid-binding protein
					57.500	167.000	120.8991	167.0000	
SGO_1632	0.618	7.022	0.0768	0.1997	12.500	22.500	23.0522	23.5069	clpP; ATP-dependent Clp protease, proteolytic subunit ClpP
					28.000	24.500	58.8726	24.5000	
SGO_1633	-0.097	7.949	0.1841	0.6500	38.500	60.000	71.0009	62.6851	upp; uracil phosphoribosyltransferase
					23.500	64.000	49.4109	64.0000	
SGO_1648	-0.261	9.108	0.0541	0.1270	70.500	124.500	130.0147	130.0717	ppx1; inorganic pyrophosphatase, manganese-dependent
					57.000	172.000	119.8478	172.0000	
SGO_1649	0.029	3.498	0.2429	0.9375	2.000	2.500	3.6884	2.6119	act; pyruvate formate-lyase-activating enzyme
						5.000		5.0000	
SGO_1652	-0.197	6.691	0.0007	0.0000	13.000	26.500	23.9743	27.6859	intracellular glycosyl hydrolase
					11.500	27.500	24.1798	27.5000	
SGO_1653	-0.301	7.020	0.1579	0.5204	10.500	32.500	19.3639	33.9545	trehalose PTS enzyme II
					19.500	35.500	41.0006	35.5000	
SGO_1666	-1.505	5.335	0.0052	0.0026	3.500	15.500	6.4546	16.1937	trkA; potassium uptake protein, Trk family
					2.000	13.500	4.2052	13.5000	
SGO_1669	0.006	6.372	0.2463	0.9553	11.000	22.500	20.2860	23.5069	ribosomal large subunit pseudouridine synthase B
					10.000	18.000	21.0259	18.0000	
SGO_1675	-0.161	5.315	0.1661	0.5658	4.000	8.500	7.3767	8.8804	HAM1 protein-like protein
					5.500	12.000	11.5643	12.0000	
SGO_1676	-0.645	5.659	0.0113	0.0108	4.500	14.500	8.2988	15.1489	murI; glutamate racemase
					5.500	15.500	11.5643	15.5000	
SGO_1678	-0.523	4.291	0.1651	0.5609	2.500	10.500	4.6104	10.9699	lysA; diaminopimelate decarboxylase
						4.000		4.0000	

Log2 Ratios: -6 -4 -2 0 2 4 6

Show detected proteins only
Show all proteins
Filter by category:
ABC Transporter

Proteins found: 627

Test	Cutoff
q-Value	.005
p-Value	

	Signif	Direction	Applies To
red	yes	+	ratios, bars
yellow	no	n/a	bars
green	yes	−	ratios, bars
pink	yes	+	p- , q-Values
light green	yes	−	p- , q-Values

Dot Plots | Dot Plots

Hendrickson *et al.*

Protein	SgPgFn vs SgPg				Raw		Normalized		Description
	Log_2 Ratio	Log_2 Sum	*q*-Value	*p*-Value	*SgPgFn*	*SgPg*	*SgPgFn*	*SgPg*	
SGO_1679	-0.165	9.433	0.0933	0.2616	82.000	198.000	151.2228	206.8610	phosphotransferase system enzyme II
					82.500	159.500	173.4640	159.5000	
SGO_1680	0.823	5.498	0.0228	0.0348	11.500	11.000	21.2081	11.4923	phosphotransferase system enzyme II
						12.500		12.5000	
SGO_1681	0.003	8.594	0.2354	0.8967	46.000	93.000	84.8323	97.1620	PTS system, mannose/fructose/sorbose family, IID component
					52.000	95.000	109.3349	95.0000	
SGO_1683	-0.191	7.970	0.0457	0.0994	34.500	67.000	63.6242	69.9984	serS; seryl-tRNA synthetase
					25.500	63.500	53.6161	63.5000	
SGO_1684	0.009	6.534	0.2382	0.9134	11.000	24.000	20.2860	25.0741	acyl-CoA dehydrogenase family
					12.500	21.000	26.2824	21.0000	
SGO_1685	0.271	8.571	0.0582	0.1392	65.500	84.000	120.7938	87.7592	putative peroxidase / antioxidase
					42.000	83.500	88.3089	83.5000	
SGO_1687	0.153	7.586	0.0385	0.0760	28.000	46.500	51.6370	48.5810	accA; acetyl-CoA carboxylase, carboxyl transferase, alpha subunit
					23.500	42.500	49.4109	42.5000	
SGO_1688	-0.983	5.513	0.0057	0.0032	5.000	14.500	9.2209	15.1489	accD; acetyl-CoA carboxylase, carboxyl transferase, beta subunit
					3.000	15.000	6.3078	15.0000	
SGO_1689	-0.110	7.385	0.0381	0.0748	22.500	40.000	41.4940	41.7901	accC; acetyl-CoA carboxylase, biotin carboxylase
					18.500	45.000	38.8980	45.0000	
SGO_1690	-1.456	5.603	0.0713	0.1821	4.000	16.000	7.3767	16.7160	fabZ; beta-hydroxyacyl-(acyl-carrier-protein) dehydratase FabZ
						24.500		24.5000	
SGO_1691	-0.614	8.006	0.0102	0.0092	24.000	77.500	44.2603	80.9683	accB; acetyl-CoA carboxylase, biotin carboxyl carrier protein
					27.500	74.000	57.8213	74.0000	
SGO_1692	-0.472	9.471	0.0019	0.0003	78.000	196.000	143.8460	204.7715	3-oxoacyl-[acyl-carrier-protein] synthase
					73.000	207.500	153.4893	207.5000	

Log_2 Ratios: -6 -4 -2 0 2 4 6

Show detected proteins only
Show all proteins
Filter by category: ABC Transporter

Proteins found: 627

Test	Cutoff
q-Value	.005
p-Value	

	Signif	Direction	Applies To
red	yes	+	ratios, bars
yellow	no	n/a	bars
green	yes	−	ratios, bars
pink	yes	+	*p*- , *q*-Values
light green	yes	−	*p*- , *q*-Values

Dot Plots Dot Plots

Hendrickson *et al.*

Streptococcus gordonii

Protein	SgPgFn vs SgPg: Log_2 Ratio	Log_2 Sum	*q*-Value	*p*-Value	Raw: *SgPgFn*	Raw: *SgPg*	Normalized: *SgPgFn*	Normalized: *SgPg*	Description	Log_2 Ratios (-6 -4 -2 0 2 4 6)
**SGO_1693**	-0.542	6.773	0.0397	0.0812	15.500	32.500	28.5848	33.9545	fabG; 3-oxoacyl-(acyl-carrier-protein) reductase	
					8.000	30.000	16.8207	30.0000		
**SGO_1694**	-0.793	6.806	0.0204	0.0291	14.000	30.000	25.8185	31.3426	fabD; malonyl CoA-acyl carrier protein transacylase	
					7.500	39.000	15.7695	39.0000		
**SGO_1695**	-0.487	9.411	0.0306	0.0534	88.500	212.000	163.2099	221.4875	enoyl-acyl carrier protein(ACP) reductase	
					57.500	175.000	120.8991	175.0000		
**SGO_1699**	-0.663	4.951	0.0888	0.2445		13.000		13.5818	transcriptional regulator, MarR family	
					3.500	10.000	7.3591	10.0000		
**SGO_1700**	-1.291	5.028	0.0264	0.0438	3.000	13.500	5.5325	14.1042	enoyl-CoA hydratase/isomerase family protein	
						13.000		13.0000		
**SGO_1701**	-0.447	7.468	0.0184	0.0246	17.500	49.000	32.2731	51.1929	aspartate kinase	
					20.500	50.500	43.1032	50.5000		
**SGO_1708**	-0.248	8.165	0.0330	0.0603	32.500	70.500	59.9358	73.6550	amiF; Oligopeptide transport ATP-binding protein amiF	
					34.000	82.000	71.4882	82.0000		
**SGO_1709**	-0.168	8.469	0.1043	0.3029	39.000	83.500	71.9230	87.2368	amiE; Oligopeptide transport ATP-binding protein	
					45.500	99.500	95.6680	99.5000		
**SGO_1710**	0.468	5.086	0.0227	0.0342	5.500	8.000	10.1430	8.3580	amiD; Oligopeptide transport system permease protein	
					4.500	6.000	9.4617	6.0000		
**SGO_1711**	0.231	7.297	0.0594	0.1436	20.000	33.500	36.8836	34.9992	hppB; Oligopeptide transport system permease	
					23.000	37.000	48.3597	37.0000		
**SGO_1712**	0.242	10.111	0.0051	0.0024	167.000	243.000	307.9781	253.8748	hppA; oligopeptide-binding lipoprotein	
					138.500	252.500	291.2092	252.5000		
**SGO_1713**	0.244	9.467	0.0073	0.0050	101.500	160.000	187.1843	167.1604	hppG; oligopeptide-binding lipoprotein	
					93.500	157.000	196.5925	157.0000		

◉ Show detected proteins only
○ Show all proteins
☐ Filter by category:
ABC Transporter

Proteins found: 627

Test	Cutoff
***q*-Value**	**.005**
p-Value	

	Signif	Direction	Applies To
red	yes	+	ratios, bars
yellow	no	n/a	bars
green	yes	–	ratios, bars
pink	yes	+	*p*- , *q*-Values
light green	yes	–	*p*- , *q*-Values

Dot Plots Dot Plots

Hendrickson *et al.*

Protein	SgPgFn vs SgPg				Raw		Normalized		Description	Log_2 Ratios
	Log_2 Ratio	Log_2 Sum	*q*-Value	*p*-Value	*SgPgFn*	*SgPg*	*SgPgFn*	*SgPg*		-6 -4 -2 0 2 4 6
**SGO_1715**	0.004	8.157	0.2404	0.9264	36.500	66.000	67.3126	68.9537	hppH; oligopeptide-binding lipoprotein	
					36.000	73.500	75.6934	73.5000		
**SGO_1716**	0.121	8.584	0.1586	0.5254	70.500	90.000	130.0147	94.0277	oligopeptide binding protein	
					35.000	86.000	73.5908	86.0000		
**SGO_1718**	-0.288	6.851	0.1156	0.3481	10.500	31.500	19.3639	32.9097	sufB-1; FeS assembly protein SufB	
					16.000	29.500	33.6415	29.5000		
**SGO_1720**	-1.053	5.634	0.0073	0.0051	5.500	15.500	10.1430	16.1937	aminotransferase, class-V	
					3.000	17.000	6.3078	17.0000		
**SGO_1721**	-0.178	7.092	0.0300	0.0521	17.000	32.500	31.3511	33.9545	sufD; FeS assembly protein SufD	
					15.500	38.500	32.5902	38.5000		
**SGO_1722**	-0.328	6.304	0.1360	0.4258	6.500	19.000	11.9872	19.8503	sufC; FeS assembly ATPase SufC	
					11.500	23.000	24.1798	23.0000		
**SGO_1727**	-0.089	6.612	0.1273	0.3962	12.000	22.000	22.1302	22.9846	amino acid ABC transporter, amino acid-binding/permease protein	
					12.000	27.500	25.2311	27.5000		
**SGO_1728**	0.361	6.099	0.0393	0.0781	10.500	17.000	19.3639	17.7608	glnQ; glutamine ABC transporter ATP-binding protein	
					9.000	12.500	18.9233	12.5000		
**SGO_1729**	-0.556	6.362	0.0426	0.0903	11.500	20.500	21.2081	21.4174	hypothetical protein SGO_1729	
					6.000	27.000	12.6156	27.0000		
**SGO_1730**	0.232	8.334	0.0701	0.1788	45.000	60.500	82.9881	63.2075	SPFH domain/Band 7 family	
					43.000	86.000	90.4115	86.0000		
**SGO_1731**	-0.354	5.984	0.0428	0.0909	7.000	14.500	12.9093	15.1489	DNA-binding response regulator	
					7.000	20.500	14.7182	20.5000		
**SGO_1735**	-0.312	8.854	0.0071	0.0047	59.000	123.500	108.8066	129.0269	hypothetical protein SGO_1735	
					46.500	127.000	97.7706	127.0000		

Show detected proteins only
Show all proteins
Filter by category:
ABC Transporter

Proteins found: 627

Test	Cutoff
***q*-Value**	**.005**
p-Value	

	Signif	Direction	Applies To
red	yes	+	ratios, bars
yellow	no	n/a	bars
green	yes	−	ratios, bars
pink	yes	+	*p*- , *q*-Values
light green	yes	−	*p*- , *q*-Values

Dot Plots Dot Plots

Protein	SgPgFn vs SgPg Log2 Ratio	Log2 Sum	q-Value	p-Value	Raw SgPgFn	Raw SgPg	Normalized SgPgFn	Normalized SgPg	Description
SGO_1736	-1.070	5.272	0.0656	0.1608	4.000	17.000	7.3767	17.7608	alkaline shock protein
						13.500		13.5000	
SGO_1745	0.286	12.325	0.0441	0.0943	665.500	1075.000	1227.3018	1123.1089	fba; fructose-1,6-bisphosphate aldolase, class II
					762.000	1177.500	1602.1763	1177.5000	
SGO_1747	1.483	3.270	0.0461	0.1009	3.000	2.500	5.5325	2.6119	hypothetical protein SGO_1747
						1.500		1.5000	
SGO_1748	-0.504	5.487	0.0660	0.1637	6.000	9.500	11.0651	9.9251	pyrG; CTP synthase
					3.500	16.500	7.3591	16.5000	
SGO_1749	-0.974	6.290	0.0133	0.0144	8.000	21.000	14.7534	21.9398	manA; mannose-6-phosphate isomerase, class I
					5.500	30.000	11.5643	30.0000	
SGO_1755	-0.613	7.025	0.0163	0.0196	13.000	42.500	23.9743	44.4020	scrK; fructokinase
					13.000	34.500	27.3337	34.5000	
SGO_1757	-0.650	8.174	0.0050	0.0019	28.000	82.000	51.6370	85.6697	glmS; glucosamine--fructose-6-phosphate aminotransferase
					29.000	90.500	60.9752	90.5000	
SGO_1763	0.094	7.454	0.1393	0.4415	25.000	36.000	46.1045	37.6111	ABC transporter, substrate-binding protein SP0092
					21.000	47.500	44.1545	47.5000	
SGO_1774	0.361	5.406	0.0878	0.2411	5.000	10.500	9.2209	10.9699	alcohol dehydrogenase, zinc-containing
					7.000	7.500	14.7182	7.5000	
SGO_1784	0.226	8.242	0.0100	0.0089	44.000	70.000	81.1439	73.1327	leuS; leucyl-tRNA synthetase
					39.000	66.500	82.0011	66.5000	
SGO_1799	-0.270	8.319	0.0358	0.0694	44.000	80.500	81.1439	84.1026	endopeptidase O
					30.500	90.000	64.1291	90.0000	
SGO_1800	-0.676	6.165	0.0092	0.0071	6.500	22.000	11.9872	22.9846	troB; manganese ABC transporter, ATP-binding protein SP1648
					7.500	21.000	15.7695	21.0000	

Log2 Ratios: -6 -4 -2 0 2 4 6

Show detected proteins only
Show all proteins
Filter by category: ABC Transporter

Proteins found: 627

Test	Cutoff
q-Value	.005
p-Value	

Color	Signif	Direction	Applies To
red	yes	+	ratios, bars
yellow	no	n/a	bars
green	yes	−	ratios, bars
pink	yes	+	p- , q-Values
light green	yes	−	p- , q-Values

Dot Plots | Dot Plots

Protein	*SgPgFn vs SgPg* Log_2 Ratio	Log_2 Sum	*q*-Value	*p*-Value	Raw *SgPgFn*	Raw *SgPg*	Normalized *SgPgFn*	Normalized *SgPg*	Description	Log_2 Ratios (-6 -4 -2 0 2 4 6)
**SGO_1802**	-0.634	8.464	0.0272	0.0462	43.000	119.500	79.2997	124.8479	Metal ABC transporter substrate-binding lipoprotein precursor	
					28.000	90.000	58.8726	90.0000		
**SGO_1803**	-0.115	6.871	0.1727	0.5975	12.500	29.500	23.0522	30.8202	tpx; thioredoxin peroxidase	
					16.000	29.500	33.6415	29.5000		
**SGO_1805**	-1.458	6.549	0.0146	0.0165	9.500	38.000	17.5197	39.7006	hutU; urocanate hydratase	
					4.000	28.000	8.4104	28.0000		
**SGO_1806**	-0.584	5.473	0.0226	0.0335	4.000	11.500	7.3767	12.0147	ftcD; glutamate formiminotransferase	
					5.000	14.500	10.5130	14.5000		
**SGO_1808**	1.477	4.890	0.0163	0.0196	4.500	4.000	8.2988	4.1790	fhs-2; formate--tetrahydrofolate ligase	
					6.500	3.500	13.6669	3.5000		
**SGO_1811**	-0.913	4.325	0.0131	0.0141		7.500		7.8356	hutH; histidine ammonia-lyase	
					2.000	8.000	4.2052	8.0000		
**SGO_1822**	-0.149	4.569	0.0583	0.1398	4.000	8.000	7.3767	8.3580	relA; GTP diphosphokinase	
						8.000		8.0000		
**SGO_1824**	-0.476	5.675	0.0116	0.0115	6.500	14.000	11.9872	14.6265	prmA; ribosomal protein L11 methyltransferase	
					4.500	15.000	9.4617	15.0000		
**SGO_1828**	0.472	3.741	0.1647	0.5585		2.500		2.6119	ATPase, AAA family	
					2.500	5.500	5.2565	5.5000		
**SGO_1834**	0.489	7.108	0.0493	0.1104	22.500	35.500	41.4940	37.0887	hypothetical protein SGO_1834	
					18.000	21.500	37.8467	21.5000		
**SGO_1835**	1.837	4.414	0.0114	0.0111		3.500		3.6566	hypothetical protein SGO_1835	
					6.500	4.000	13.6669	4.0000		
**SGO_1843**	-0.059	7.934	0.1835	0.6466	29.500	67.000	54.4033	69.9984	pepS; aminopeptidase PepS	
					31.000	55.000	65.1804	55.0000		

◉ Show detected proteins only
○ Show all proteins
☐ Filter by category:
ABC Transporter

Proteins found: 627

Test	Cutoff
***q*-Value**	**.005**
p-Value	

	Signif	Direction	Applies To
red	yes	+	ratios, bars
yellow	no	n/a	bars
green	yes	−	ratios, bars
pink	yes	+	*p*- , *q*-Values
light green	yes	−	*p*- , *q*-Values

Dot Plots | Dot Plots

Protein	SgPgFn vs SgPg Log$_2$ Ratio	Log$_2$ Sum	*q*-Value	*p*-Value	Raw *SgPgFn*	Raw *SgPg*	Normalized *SgPgFn*	Normalized *SgPg*	Description
**SGO_1844**	-1.041	5.171	0.0100	0.0089	2.500	12.500	4.6104	13.0594	cbxX/cfqX family protein
					3.500	11.000	7.3591	11.0000	
**SGO_1847**	-0.863	4.872	0.0339	0.0631		10.500		10.9699	polC; DNA polymerase III, alpha subunit, Gram-positive type
					3.000	12.000	6.3078	12.0000	
**SGO_1848**	-0.036	4.690	0.2404	0.9247	2.500	6.500	4.6104	6.7909	lipoprotein, putative
					4.000	6.000	8.4104	6.0000	
**SGO_1851**	-0.011	8.612	0.2296	0.8672	55.500	99.500	102.3520	103.9529	proS; prolyl-tRNA synthetase
					44.000	92.500	92.5141	92.5000	
**SGO_1852**	0.033	4.627	0.2454	0.9485	4.500	9.000	8.2988	9.4028	membrane-associated zinc metalloprotease, putative
						7.000		7.0000	
**SGO_1854**	0.121	5.798	0.1989	0.7144	6.000	16.000	11.0651	16.7160	uppS; undecaprenyl diphosphate synthase
					8.500	10.000	17.8720	10.0000	
**SGO_1856**	0.023	5.751	0.2247	0.8416	8.500	14.000	15.6755	14.6265	ATP-dependent proteinase ATP-binding chain
					5.500	12.000	11.5643	12.0000	
**SGO_1860**	-0.414	7.507	0.0146	0.0165	19.500	53.000	35.9615	55.3719	5'-nucleotidase, lipoprotein e(P4) family
					20.000	48.500	42.0519	48.5000	
**SGO_1861**	-1.866	4.710	0.0246	0.0386		11.500		12.0147	nusB; transcription antitermination factor NusB
					1.500	11.000	3.1539	11.0000	
**SGO_1862**	0.067	6.752	0.0956	0.2722	14.500	26.500	26.7406	27.6859	alkaline shock protein
					13.500	25.000	28.3850	25.0000	
**SGO_1863**	-0.035	8.179	0.1094	0.3198	40.000	69.000	73.7672	72.0879	efp; Elongation factor P (EF-P)
					33.000	74.500	69.3856	74.5000	
**SGO_1864**	-0.032	7.000	0.2382	0.9116	22.500	30.000	41.4940	31.3426	X-Pro aminopeptidase
					11.000	32.000	23.1285	32.0000	

Log$_2$ Ratios: -6 -4 -2 0 2 4 6

Show detected proteins only
Show all proteins
Filter by category: ABC Transporter

Proteins found: 627

Test	Cutoff
***q*-Value**	**.005**
p-Value	

	Signif	Direction	Applies To
red	yes	+	ratios, bars
yellow	no	n/a	bars
green	yes	–	ratios, bars
pink	yes	+	*p*- , *q*-Values
light green	yes	–	*p*- , *q*-Values

Dot Plots | Dot Plots

Streptococcus gordonii

Protein	SgPgFn vs SgPg Log2 Ratio	Log2 Sum	*q*-Value	*p*-Value	Raw *SgPgFn*	Raw *SgPg*	Normalized *SgPgFn*	Normalized *SgPg*	Description
SGO_1865	-0.582	5.878	0.0007	0.0000	6.500	17.000	11.9872	17.7608	uvrA; excinuclease ABC, A subunit
					5.500	17.500	11.5643	17.5000	
SGO_1866	1.334	3.687			5.000	3.500	9.2209	3.6566	corA; magnesium and cobalt transporter
SGO_1867	0.335	7.252	0.0477	0.1060	27.000	31.500	49.7929	32.9097	hypothetical protein SGO_1867
					17.000	34.000	35.7441	34.0000	
SGO_1879	0.215	7.603	0.0228	0.0348	27.500	46.000	50.7149	48.0586	rpsR; ribosomal protein S18
					25.500	42.000	53.6161	42.0000	
SGO_1880	-0.388	8.214	0.0069	0.0045	35.500	76.500	65.4684	79.9236	ssb-1; single-strand binding protein
					30.000	88.500	63.0778	88.5000	
SGO_1881	-0.231	9.505	0.0656	0.1618	95.500	214.000	176.1192	223.5770	rpsF; ribosomal protein S6
					74.500	170.000	156.6432	170.0000	
SGO_1882	-1.092	5.641	0.0125	0.0131	4.000	13.500	7.3767	14.1042	folE; GTP cyclohydrolase I
					4.000	20.000	8.4104	20.0000	
SGO_1885	-0.214	9.596	0.0050	0.0022	94.500	200.000	174.2750	208.9505	groL; 60 kDa chaperonin/groEL protein
					87.500	206.500	183.9769	206.5000	
SGO_1886	-0.844	5.401	0.0466	0.1028	3.000	17.000	5.5325	17.7608	groES; chaperonin, 10 kDa
					4.500	9.500	9.4617	9.5000	
SGO_1892	-0.259	5.592	0.1219	0.3717	7.500	11.000	13.8313	11.4923	PTS system, fructose(mannose)-specific IIB
					4.000	14.500	8.4104	14.5000	
SGO_1898	-0.675	7.534	0.0026	0.0006	20.500	53.500	37.8057	55.8943	glutamyl aminopeptidase
					16.000	58.000	33.6415	58.0000	
SGO_1901	-0.352	6.462	0.0503	0.1141	10.000	28.000	18.4418	29.2531	tRNA binding domain
					9.500	20.500	19.9746	20.5000	

Log2 Ratios: -6 -4 -2 0 2 4 6

Show detected proteins only
Show all proteins
Filter by category:
ABC Transporter

Proteins found: 627

Test	Cutoff
q-Value	.005
p-Value	

	Signif	Direction	Applies To
red	yes	+	ratios, bars
yellow	no	n/a	bars
green	yes	−	ratios, bars
pink	yes	+	*p*- , *q*-Values
light green	yes	−	*p*- , *q*-Values

Dot Plots Dot Plots

Protein	*SgPgFn vs SgPg* Log$_2$ Ratio	Log$_2$ Sum	*q*-Value	*p*-Value	Raw *SgPgFn*	Raw *SgPg*	Normalized *SgPgFn*	Normalized *SgPg*	Description
SGO_1902	-0.339	9.940	0.0402	0.0826	131.000	291.000	241.5876	304.0230	ssb; single-stranded DNA-binding protein
					91.500	244.500	192.3873	244.5000	
SGO_1903	0.056	7.626	0.1705	0.5878	24.000	44.500	44.2603	46.4915	ATP-dependent Zn protease
					27.000	50.000	56.7700	50.0000	
SGO_1914	0.052	3.628	0.2193	0.8139		3.500		3.6566	folP; dihydropteroate synthase
					2.000	4.500	4.2052	4.5000	
SGO_1916	-0.106	8.144	0.0163	0.0194	38.000	70.500	70.0788	73.6550	ackA; acetate kinase
					31.500	73.000	66.2317	73.0000	
SGO_1917	0.042	5.049	0.2247	0.8395	4.500	9.000	8.2988	9.4028	hypothetical protein SGO_1917
					4.000	7.000	8.4104	7.0000	
SGO_1924	-0.471	6.528	0.0494	0.1109	8.500	21.500	15.6755	22.4622	comYA; competence protein comYA
					11.000	31.000	23.1285	31.0000	
SGO_1926	0.039	10.167	0.0956	0.2716	153.500	277.000	283.0816	289.3964	rpoC; DNA-directed RNA polymerase, beta chain
					142.500	277.500	299.6196	277.5000	
SGO_1927	0.150	9.994	0.0423	0.0883	135.500	228.000	249.8864	238.2036	rpoB; DNA-directed RNA polymerase, beta subunit
					136.500	244.500	287.0040	244.5000	
SGO_1928	-0.421	4.347	0.0219	0.0317	3.000	7.000	5.5325	7.3133	pbp1b; penicillin-binding protein 1B
						7.500		7.5000	
SGO_1929	-0.655	9.026	0.0087	0.0065	50.500	164.500	93.1311	171.8618	tyrS; tyrosyl-tRNA synthetetase
					52.000	147.000	109.3349	147.0000	
SGO_1936	-0.550	6.741	0.0107	0.0101	10.500	32.000	19.3639	33.4321	adcA; metal-binding (Mn) permease precursor, lipoprotein
					11.500	30.000	24.1798	30.0000	
SGO_1958	-0.481	10.205	0.0116	0.0114	122.500	350.000	225.9120	365.6633	rplQ; ribosomal protein L17
					127.000	321.500	267.0294	321.5000	

Log$_2$ Ratios: -6 -4 -2 0 2 4 6

Show detected proteins only
Show all proteins
Filter by category: ABC Transporter

Proteins found: 627

Test	Cutoff
q-Value	.005
p-Value	

	Signif	Direction	Applies To
red	yes	+	ratios, bars
yellow	no	n/a	bars
green	yes	−	ratios, bars
pink	yes	+	*p*- , *q*-Values
light green	yes	−	*p*- , *q*-Values

Dot Plots Dot Plots

Streptococcus gordonii

Protein	SgPgFn vs SgPg Log_2 Ratio	Log_2 Sum	*q*-Value	*p*-Value	Raw *SgPgFn*	Raw *SgPg*	Normalized *SgPgFn*	Normalized *SgPg*	Description
SGO_1959	-0.145	9.035	0.0322	0.0578	66.000	125.000	121.7159	130.5941	rpoA; DNA-directed RNA polymerase, alpha subunit
					60.500	145.000	127.2069	145.0000	
SGO_1960	0.040	7.986	0.1586	0.5270	36.000	56.500	66.3905	59.0285	rpsK; ribosomal protein S11
					29.500	66.000	62.0265	66.0000	
SGO_1961	-1.119	9.386	0.0026	0.0006	54.500	231.500	100.5078	241.8602	rpsM; ribosomal protein S13p/S18e
					52.500	216.500	110.3862	216.5000	
SGO_1962	0.805	5.815	0.0818	0.2172	14.000	11.000	25.8185	11.4923	rpmJ; ribosomal protein L36
						19.000		19.0000	
SGO_1964	0.384	8.500	0.0076	0.0054	52.500	75.500	96.8194	78.8788	adk; Adenylate kinase (ATP-AMP transphosphorylase)
					51.500	78.000	108.2836	78.0000	
SGO_1965	0.121	5.444	0.0928	0.2595	6.000	9.000	11.0651	9.4028	Preprotein translocase secY subunit
					5.500	11.500	11.5643	11.5000	
SGO_1966	-0.081	8.956	0.1835	0.6466	57.500	94.000	106.0403	98.2067	rplO; ribosomal protein L15
					63.000	160.000	132.4634	160.0000	
SGO_1967	-0.897	8.463	0.0054	0.0028	29.500	116.000	54.4033	121.1913	50S ribosomal protein L30 -related protein
					33.000	108.000	69.3856	108.0000	
SGO_1968	-0.439	9.855	0.0048	0.0017	110.500	263.000	203.7819	274.7699	rpsE; ribosomal protein S5
					90.000	258.000	189.2334	258.0000	
SGO_1969	0.025	10.151	0.0183	0.0240	156.500	270.500	288.6142	282.6055	rplR; ribosomal protein L18
					135.500	281.000	284.9014	281.0000	
SGO_1970	0.135	9.595	0.1111	0.3304	126.500	178.500	233.2888	186.4883	BL10; 50S ribosomal protein L6
					82.500	180.000	173.4640	180.0000	
SGO_1971	0.393	10.912	0.0327	0.0590	339.500	411.500	626.0991	429.9156	rpsH; ribosomal protein S8
					224.500	398.500	472.0323	398.5000	

Log_2 Ratios: -6 -4 -2 0 2 4 6

Show detected proteins only
Show all proteins
Filter by category:
ABC Transporter

Proteins found: 627

Test	Cutoff
q-Value	.005
p-Value	

	Signif	Direction	Applies To
(red)	yes	+	ratios, bars
(yellow)	no	n/a	bars
(green)	yes	–	ratios, bars
(pink)	yes	+	*p*- , *q*-Values
(light green)	yes	–	*p*- , *q*-Values

Dot Plots Dot Plots

Protein	SgPgFn vs SgPg: Log_2 Ratio	Log_2 Sum	*q*-Value	*p*-Value	Raw: *SgPgFn*	Raw: *SgPg*	Normalized: *SgPgFn*	Normalized: *SgPg*	Description
SGO_1973	0.268	9.741	0.0248	0.0399	137.500	189.500	253.5747	197.9806	BL6; 50S ribosomal protein L5
					102.000	189.500	214.4645	189.5000	
SGO_1974	0.831	8.830	0.0071	0.0046	71.500	74.500	131.8589	77.8341	rplX; ribosomal protein L24
					76.000	85.500	159.7971	85.5000	
SGO_1975	0.553	8.865	0.0092	0.0073	69.000	92.500	127.2484	96.6396	rplN; ribosomal protein L14
					71.500	92.000	150.3354	92.0000	
SGO_1976	-0.862	8.785	0.0140	0.0153	51.500	121.500	94.9753	126.9374	BS16; 30S ribosomal protein
					30.000	156.000	63.0778	156.0000	
SGO_1977	0.361	6.208	0.0684	0.1734	9.000	13.500	16.5976	14.1042	rpmC; ribosomal protein L29
					12.000	18.000	25.2311	18.0000	
SGO_1978	-0.529	8.605	0.0007	0.0000	42.500	110.500	78.3776	115.4451	rplP; ribosomal protein L16
					38.500	114.500	80.9499	114.5000	
SGO_1979	0.134	9.307	0.0661	0.1645	82.500	148.000	152.1448	154.6234	rpsC; ribosomal protein S3
					85.500	147.000	179.7717	147.0000	
SGO_1980	0.323	9.561	0.0094	0.0076	108.000	164.000	199.1714	171.3394	rplV; ribosomal protein L22
					105.000	164.000	220.7723	164.0000	
SGO_1981	0.140	8.873	0.0680	0.1722	62.000	100.000	114.3392	104.4752	rpsS; ribosomal protein S19
					62.500	118.500	131.4121	118.5000	
SGO_1982	0.235	8.829	0.0419	0.0871	61.000	107.000	112.4950	111.7885	rplB; ribosomal protein L2
					63.500	97.000	133.5147	97.0000	
SGO_1983	-0.007	8.326	0.2399	0.9214	38.000	91.000	70.0788	95.0725	rplW; ribosomal protein L23
					42.500	66.500	89.3602	66.5000	
SGO_1984	0.622	9.793	0.0066	0.0041	136.000	158.000	250.8085	165.0709	rplD; ribosomal protein L4/L1 family
					136.500	184.000	287.0040	184.0000	

Log_2 Ratios: -6 -4 -2 0 2 4 6

Show detected proteins only
Show all proteins
Filter by category:
ABC Transporter

Proteins found: 627

Test	Cutoff
q-Value	.005
p-Value	

	Signif	Direction	Applies To
red	yes	+	ratios, bars
yellow	no	n/a	bars
green	yes	–	ratios, bars
pink	yes	+	*p*- , *q*-Values
light green	yes	–	*p*- , *q*-Values

Dot Plots Dot Plots

Protein	SgPgFn vs SgPg Log$_2$ Ratio	Log$_2$ Sum	*q*-Value	*p*-Value	Raw *SgPgFn*	Raw *SgPg*	Normalized *SgPgFn*	Normalized *SgPg*	Description
SGO_1985	-0.307	10.243	0.1025	0.2963	139.000	223.000	256.3410	232.9798	rplC; ribosomal protein L3
					128.000	453.000	269.1320	453.0000	
SGO_1986	0.546	7.865	0.0050	0.0018	38.500	42.500	71.0009	44.4020	rpsJ; ribosomal protein S10
					32.000	50.500	67.2830	50.5000	
SGO_1989	0.019	9.834	0.2083	0.7590	117.500	226.500	216.6911	236.6364	purA; adenylosuccinate synthetase
					115.500	216.500	242.8496	216.5000	
SGO_1990	-0.456	4.657	0.1579	0.5196	3.500	6.000	6.4546	6.2685	glutamate--cysteine ligase, putative/amino acid ligase, putative
						12.500		12.5000	
SGO_1993	-0.205	6.372	0.1151	0.3438	8.500	22.500	15.6755	23.5069	possible transcriptional regulator
					11.000	20.500	23.1285	20.5000	
SGO_1998	-0.844	6.394	0.0172	0.0218	9.500	22.000	17.5197	22.9846	clpB; ATP-dependent Clp proteinase, ATP-binding chain
					6.000	31.000	12.6156	31.0000	
SGO_2000	0.015	10.808	0.2122	0.7760	260.000	437.500	479.4868	457.0792	tsf; translation elongation factor Ts
					201.000	434.000	422.6213	434.0000	
SGO_2001	0.089	10.507	0.0813	0.2151	191.000	347.500	352.2384	363.0515	rpsB; ribosomal protein S2
					189.500	341.500	398.4415	341.5000	
SGO_2005	0.722	5.492	0.0219	0.0321	9.000	7.500	16.5976	7.8356	LPXTG cell wall surface protein
					5.500	9.000	11.5643	9.0000	
SGO_2007	0.063	6.694	0.1174	0.3566	15.000	25.500	27.6627	26.6412	nusG; transcription termination/antitermination factor NusG
					12.000	24.000	25.2311	24.0000	
SGO_2024	-0.289	5.777	0.0339	0.0630	6.000	13.500	11.0651	14.1042	Extracellular polysaccharide biosynthesis
					6.500	16.000	13.6669	16.0000	
SGO_2025	-1.332	5.698	0.0123	0.0128	3.000	20.500	5.5325	21.4174	wze; putative autophosphorylating protein tyrosine kinase
					4.500	15.500	9.4617	15.5000	

Log$_2$ Ratios: -6 -4 -2 0 2 4 6

Show detected proteins only
Show all proteins
Filter by category:
ABC Transporter

Proteins found: 627

Test	Cutoff
q-Value	.005
p-Value	

	Signif	Direction	Applies To
red	yes	+	ratios, bars
yellow	no	n/a	bars
green	yes	−	ratios, bars
pink	yes	+	*p*- , *q*-Values
light green	yes	−	*p*- , *q*-Values

Dot Plots Dot Plots

Streptococcus gordonii

Hackett Laboratory UW

Protein	SgPgFn vs SgPg Log2 Ratio	Log2 Sum	q-Value	p-Value	Raw SgPgFn	Raw SgPg	Normalized SgPgFn	Normalized SgPg	Description
SGO_2033	-0.425	6.565	0.0099	0.0085	10.000	26.500	18.4418	27.6859	nrdD; ribonucleoside-triphosphate reductase
					10.500	26.500	22.0772	26.5000	
SGO_2041	-1.052	4.251	0.0482	0.1073	2.000	8.000	3.6884	8.3580	conserved hypothetical protein TIGR00250
						7.000		7.0000	
SGO_2042	0.382	8.928	0.0313	0.0550	83.500	92.500	153.9890	96.6396	Bacterial protein of unknown function (DUF965) superfamily
					58.000	114.500	121.9504	114.5000	
SGO_2045	0.083	10.745	0.0818	0.2174	254.000	389.500	468.4217	406.9311	recA; recA protein
					197.500	426.000	415.2622	426.0000	
SGO_2046	-0.970	4.841	0.0050	0.0022	3.000	9.500	5.5325	9.9251	cinA; competence induced protein
					2.000	9.000	4.2052	9.0000	
SGO_2053	-0.100	4.861	0.1661	0.5663	3.500	5.500	6.4546	5.7461	DNA mismatch repair protein hexB
					3.500	9.500	7.3591	9.5000	
SGO_2056	-0.693	5.734	0.0656	0.1610	3.500	12.500	6.4546	13.0594	mutS; DNA mismatch repair protein MutS
					7.000	19.000	14.7182	19.0000	
SGO_2058	0.379	9.776	0.0103	0.0095	143.000	188.000	263.7177	196.4135	argS; arginyl-tRNA synthetase
					110.500	184.500	232.3366	184.5000	
SGO_2060	-1.094	7.569	0.0012	0.0001	17.500	61.500	32.2731	64.2523	aspS-1; aspartyl-tRNA synthetase
					13.500	65.000	28.3850	65.0000	
SGO_2062	0.193	8.348	0.0506	0.1152	51.000	77.500	94.0532	80.9683	hisS; histidyl-tRNA synthetase
					38.000	71.000	79.8986	71.0000	
SGO_2064	-0.242	8.955	0.0271	0.0454	64.500	138.000	118.9496	144.1758	ilvD; dihydroxy-acid dehydratase
					51.500	125.000	108.2836	125.0000	
SGO_2066	-0.337	7.834	0.0466	0.1024	31.500	66.500	58.0917	69.4760	rpmG; ribosomal protein L33
					20.500	57.500	43.1032	57.5000	

Log2 Ratios: -6 -4 -2 0 2 4 6

Show detected proteins only
Show all proteins
Filter by category:
ABC Transporter

Proteins found: 627

Test	Cutoff
q-Value	.005
p-Value	

	Signif	Direction	Applies To
red	yes	+	ratios, bars
yellow	no	n/a	bars
green	yes	−	ratios, bars
pink	yes	+	p- , q-Values
light green	yes	−	p- , q-Values

Dot Plots Dot Plots

Hendrickson *et al.*

Protein	SgPgFn vs SgPg Log_2 Ratio	Log_2 Sum	*q*-Value	*p*-Value	Raw *SgPgFn*	Raw *SgPg*	Normalized *SgPgFn*	Normalized *SgPg*	Description
SGO_2070	-0.715	5.430	0.0332	0.0611	5.500	15.000	10.1430	15.6713	hypothetical protein SGO_2070
					3.000	11.000	6.3078	11.0000	
SGO_2085	0.047	7.648	0.0778	0.2038	28.500	48.000	52.5591	50.1481	purB; adenylosuccinate lyase
					23.500	48.500	49.4109	48.5000	
SGO_2097	-1.055	4.250	0.0195	0.0268	2.000	7.500	3.6884	7.8356	comA; ATP-binding Transport protein ComA
						7.500		7.5000	
SGO_2098	-0.102	11.049	0.1579	0.5181	319.500	519.500	589.2155	542.7489	rpsD; ribosomal protein S4
					208.500	548.500	438.3908	548.5000	
SGO_2100	0.055	9.145	0.1191	0.3624	76.500	140.500	141.0798	146.7877	ABC transporter substrate-binding protein
					70.000	131.000	147.1815	131.0000	
SGO_2102	-1.959	4.836	0.0805	0.2118		9.000		9.4028	hypothetical protein SGO_2102
					1.500	16.000	3.1539	16.0000	
SGO_2104	0.532	8.288	0.0328	0.0594	59.000	55.000	108.8066	57.4614	srtB; sortase B
					36.500	69.500	76.7447	69.5000	
SGO_2105	0.573	6.179	0.0050	0.0019	11.500	15.000	21.2081	15.6713	abpA; amylase-binding protein AbpA
					10.500	13.500	22.0772	13.5000	
SGO_2106	-0.167	7.791	0.0396	0.0799	28.000	52.000	51.6370	54.3271	ribose-phosphate diphosphokinase
					25.000	63.000	52.5648	63.0000	
SGO_2133	0.155	9.003	0.0195	0.0267	76.500	116.000	141.0798	121.1913	Cell division protein ftsH-like protein
					61.500	121.500	129.3095	121.5000	
SGO_2134	-0.190	7.151	0.1153	0.3458	21.500	37.500	39.6499	39.1782	hpt; hypoxanthine phosphoribosyltransferase
					13.000	36.000	27.3337	36.0000	
SGO_2142	-0.244	8.063	0.0397	0.0812	30.000	73.500	55.3254	76.7893	GTP-binding protein
					32.000	68.000	67.2830	68.0000	

Show detected proteins only
Show all proteins
Filter by category:
ABC Transporter

Proteins found: 627

Test	Cutoff
***q*-Value**	**.005**
p-Value	

Color	Signif	Direction	Applies To
red	yes	+	ratios, bars
yellow	no	n/a	bars
green	yes	−	ratios, bars
pink	yes	+	*p*- , *q*-Values
light green	yes	−	*p*- , *q*-Values

Dot Plots Dot Plots

Protein	SgPgFn vs SgPg: Log_2 Ratio	Log_2 Sum	*q*-Value	*p*-Value	Raw: *SgPgFn*	Raw: *SgPg*	Normalized: *SgPgFn*	Normalized: *SgPg*	Description	Log_2 Ratios (-6 -4 -2 0 2 4 6)
SGO_2145	0.001	8.941	0.2492	0.9711	67.000	109.500	123.5601	114.4004	comE; competence response regulator ComE	
					58.000	131.500	121.9504	131.5000		
SGO_2146	-0.958	4.948	0.0402	0.0827		12.500		13.0594	comD; histidine protein kinase ComD	
					3.000	11.500	6.3078	11.5000		
SGO_2150	-1.449	6.392	0.0050	0.0020	8.000	29.500	14.7534	30.8202	degP; serine protease	
					4.000	30.000	8.4104	30.0000		

Show detected proteins only
Show all proteins
Filter by category:
ABC Transporter

Proteins found:
627

Test	Cutoff
q-Value	.005
p-Value	

	Signif	Direction	Applies To
red	yes	+	ratios, bars
yellow	no	n/a	bars
green	yes	−	ratios, bars
pink	yes	+	*p*- , *q*-Values
light green	yes	−	*p*- , *q*-Values

Dot Plots Dot Plots

Hendrickson *et al.*
