# Supplementary material for: Proteomics of Streptococcus gordonii within a model developing oral microbial community
Source: BMC Microbiol. 2012 Sep 18;12:211. doi: 10.1186/1471-2180-12-211 (PMC3534352; doi:10.1186/1471-2180-12-211)
Supplement: Additional file 8 — Coverage. Coverage statistics for individual proteins based on recovered tryptic fragments and the inferred sequences from the annotated genome for S. gordonii[36]. Gray shading indicates the percentage of the protein covered by the detected peptides. Black shading indicates the undetected percentage. [file 1471-2180-12-211-S8.pdf]

Coverage

Streptococcus gordonii

Hackett Laboratory UW

Protein	*Sg* n	*Sg* Coverage	*SgFn* n	*SgFn* Coverage	*SgPg* n	*SgPg* Coverage	*SgPgFn* n	*SgPgFn* Coverage	Description
**SGO_0001**	16	48.2%	7	26.0%	9	33.3%	4	15.6%	dnaA; chromosomal replication initiator protein DnaA
	24	57.3%	10	34.2%	9	32.9%	7	26.4%	
**SGO_0002**	9	27.5%	7	24.9%	11	35.4%	4	11.9%	dnaN; DNA polymerase III, beta subunit
	10	30.7%	7	27.8%	11	33.3%	4	11.9%	
**SGO_0004**	15	62.2%	11	51.7%	11	52.3%	8	43.6%	putative lipoprotein
	14	59.3%	9	35.5%	12	52.9%	9	41.3%	
**SGO_0005**	8	10.9%							hypothetical protein SGO_0005
	11	16.0%							
**SGO_0006**	17	41.5%	17	44.8%	21	52.2%	10	26.7%	ABC transporter, ATP-binding protein
	20	44.8%	15	43.5%	18	45.7%	9	26.7%	
**SGO_0007**	8	34.7%	7	28.8%	9	34.4%	7	28.2%	trpS; tryptophanyl-tRNA synthetase
	9	35.0%	7	29.7%	8	34.7%	5	20.6%	
**SGO_0008**	23	54.8%	22	62.9%	30	77.7%	20	52.3%	inosine-5'-monophosphate dehydrogenase
	26	58.2%	26	64.5%	28	70.4%	20	57.0%	
**SGO_0011**	7	22.7%			3	11.3%	8	25.1%	proteinase, M16 family
	10	27.2%	5	17.6%	4	11.6%	4	13.7%	
**SGO_0012**			3	13.7%					mpp; peptidase, M16 family
	3	8.4%	4	17.2%					
**SGO_0013**	4	18.1%			4	18.1%			hypothetical protein SGO_0013
	5	21.0%							
**SGO_0015**	8	41.6%			5	24.8%			ABC transporter (ATP-binding protein)
	9	42.3%	3	15.0%	6	33.6%	3	15.7%	
**SGO_0016**	4	24.7%			4	24.0%			cbiO; ABC transporter, ATP-binding protein
	4	17.9%			4	16.8%			

Sg Rep I Rep II | *SgFn* Rep I Rep II | *SgPg* Rep I Rep II | *SgPgFn* Rep I Rep II

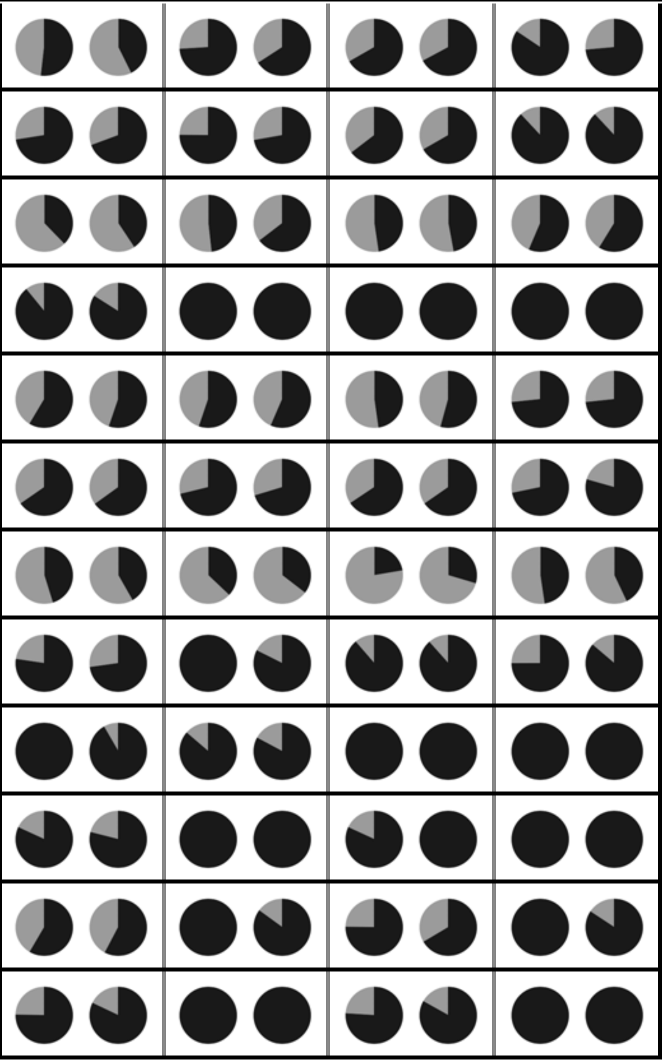

◉ Show detected proteins only
○ Show all proteins

☐ Filter by category:
ABC Transporter

Proteins found:
1179

Dot Plots Dot Plots

Hendrickson *et al.*

Protein	*Sg* n	*Sg* Coverage	*SgFn* n	*SgFn* Coverage	*SgPg* n	*SgPg* Coverage	*SgPgFn* n	*SgPgFn* Coverage	Description
**SGO_0017**	4	12.5%							ABC-type putative cobalt transport system, permease
	4	13.3%							
**SGO_0019**									sdhA; L-serine dehydratase, iron-sulfur-dependent, alpha subunit
	6	24.1%							
**SGO_0020**			3	26.9%					sdhB; L-serine dehydratase, iron-sulfur-dependent, beta subunit
	3	19.7%	3	24.2%					
**SGO_0022**	6	20.4%	15	55.2%	8	28.4%	5	20.6%	trmU; tRNA (5-methylaminomethyl-2-thiouridylate)-methyltransferase
	8	29.5%	15	56.3%	11	31.1%	3	15.0%	
**SGO_0025**	10	23.8%	4	9.5%	11	23.9%	4	8.1%	gidA; glucose inhibited division protein A
	10	22.8%	6	13.8%	10	22.1%	7	14.9%	
**SGO_0026**	20	32.3%			4	9.1%	4	6.8%	DHH subfamily 1 protein
	21	33.1%			5	7.3%			
**SGO_0027**	7	54.7%	7	46.7%	8	63.3%	7	58.7%	rplI; ribosomal protein L9
	11	72.0%	9	64.0%	8	55.3%	8	52.7%	
**SGO_0028**	9	30.6%	4	11.8%	5	17.7%			dnaC; replicative DNA helicase
	13	41.9%	4	13.7%	7	27.5%	3	9.5%	
**SGO_0030**	12	47.3%	18	54.2%	15	57.7%	11	40.8%	aspB; aspartate transaminase
	13	46.1%	19	59.7%	14	49.9%	11	42.8%	
**SGO_0032**	11	36.9%	7	30.0%	12	44.1%	5	18.0%	plsX; fatty acid/phospholipid synthesis protein PlsX
	16	42.6%	7	27.9%	11	39.3%	4	11.1%	
**SGO_0033**	4	41.8%			3	40.5%	3	40.5%	acpP; acyl carrier protein
	6	57.0%			4	51.9%	4	51.9%	
**SGO_0035**			4	5.5%					phosphoribosylformylglycinamidine synthase
	3	3.6%	5	6.4%	3	4.2%			

Pie-chart columns: *Sg* Rep I, Rep II; *SgFn* Rep I, Rep II; *SgPg* Rep I, Rep II; *SgPgFn* Rep I, Rep II

Show detected proteins only
Show all proteins
Filter by category:
ABC Transporter

Proteins found: 1179

Dot Plots Dot Plots

Streptococcus gordonii

Hackett Laboratory UW

Protein	*Sg* n	*Sg* Coverage	*SgFn* n	*SgFn* Coverage	*SgPg* n	*SgPg* Coverage	*SgPgFn* n	*SgPgFn* Coverage	Description
SGO_0042	3	15.1%	6	43.3%	5	33.2%	3	17.6%	transcription regulator, GntR family
	5	26.5%	5	35.3%	5	29.8%			
SGO_0044									PTS system, IIB component
SGO_0050	7	33.5%							hypothetical protein SGO_0050
	10	40.4%							
SGO_0051	3	16.1%							hypothetical protein SGO_0051
	4	28.6%							
SGO_0054	16	40.8%	13	36.3%	13	31.8%	6	20.9%	dltA; D-alanine-activating enzyme
	16	38.5%	13	37.7%	16	38.9%	7	20.3%	
SGO_0055	3	9.9%							dltB; integral membrane protein
SGO_0056	3	39.2%	3	31.6%					dltC; D-alanyl carrier protein
	4	36.7%							
SGO_0057	20	58.3%	14	48.8%	13	43.8%	7	25.4%	dltD protein
	26	64.0%	9	32.9%	12	40.0%	6	19.9%	
SGO_0059	4	45.3%	4	45.3%	4	45.3%	4	45.3%	pXO1; hypothetical protein SGO_0059
	4	45.3%	4	45.3%	5	45.3%	5	45.3%	
SGO_0060	36	45.3%							hypothetical protein SGO_0060
	45	49.0%	3	5.0%					
SGO_0062	3	51.2%							hypothetical protein SGO_0062
SGO_0063	14	32.1%	4	10.8%	7	22.3%	5	14.4%	hypothetical protein SGO_0063
	17	42.9%	7	18.9%	9	27.3%	7	17.5%	

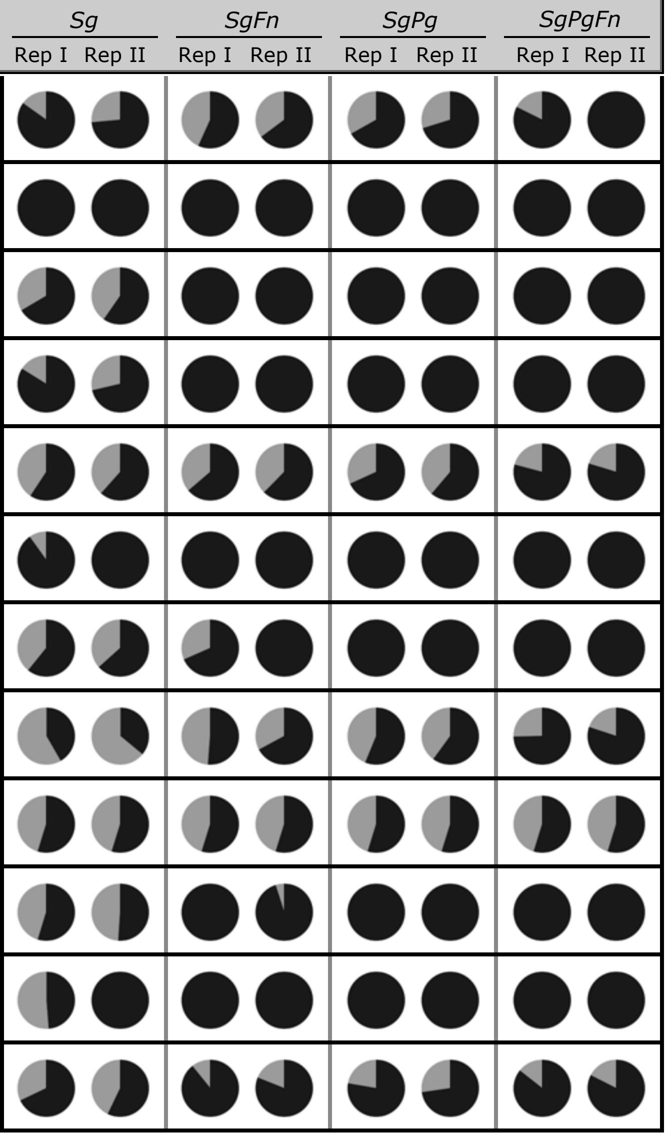

Hendrickson *et al.*

Coverage

Streptococcus gordonii

Hackett Laboratory UW

Protein	*Sg* n	*Sg* Coverage	*SgFn* n	*SgFn* Coverage	*SgPg* n	*SgPg* Coverage	*SgPgFn* n	*SgPgFn* Coverage	Description	*Sg* Rep I	*Sg* Rep II	*SgFn* Rep I	*SgFn* Rep II	*SgPg* Rep I	*SgPg* Rep II	*SgPgFn* Rep I	*SgPgFn* Rep II
**SGO_0064**	50	43.8%	9	8.5%	29	28.5%	14	13.6%	FtsK/SpoIIIE family protein								
	52	42.8%	11	11.8%	30	30.6%	17	16.4%									
**SGO_0065**	16	49.9%	9	33.7%	13	48.2%	10	35.9%	hypothetical protein SGO_0065								
	18	50.4%	11	38.1%	15	56.2%	12	37.0%									
**SGO_0066**			3	36.6%	4	36.6%	3	36.6%	D-3-phosphoglycerate dehydrogenase, putative								
					5	58.4%	3	36.6%									
**SGO_0067**	8	25.7%	4	14.6%	13	37.3%	8	28.1%	protein with prophage function domain								
	9	29.7%	5	17.1%	14	42.6%	6	20.0%									
**SGO_0068**	7	40.7%			7	40.7%	3	24.3%	lipoprotein, putative								
	7	40.7%	4	28.2%	6	40.1%	3	23.2%									
**SGO_0069**	5	38.6%			5	38.6%	3	25.5%	hypothetical protein SGO_0069								
	7	45.7%			4	31.0%	4	31.0%									
**SGO_0070**	4	33.6%			4	34.3%	4	38.8%	merozoite surface protein 1								
	5	39.6%			4	34.3%	4	39.6%									
**SGO_0075**	4	23.6%							hypothetical protein SGO_0075								
**SGO_0078**	7	38.2%			5	25.0%			hypothetical protein SGO_0078								
	7	38.6%	3	20.9%	4	25.0%											
**SGO_0079**	3	27.6%							hypothetical protein SGO_0079								
**SGO_0080**	17	38.7%	9	20.8%	14	31.8%	10	24.4%	hypothetical protein SGO_0080								
	22	49.8%	7	18.0%	12	29.7%	10	23.3%									
**SGO_0081**	3	11.5%	5	21.6%	3	11.5%			hypothetical protein SGO_0081								
	3	11.5%	4	13.3%	6	29.8%											

◉ Show detected proteins only
○ Show all proteins

☐ Filter by category:
ABC Transporter

Proteins found:
1179

Dot Plots Dot Plots

Coverage

Streptococcus gordonii

Hackett Laboratory UW

Summary Table | SgFn vs Sg | SgPg vs Sg | SgPgFn vs Sg | SgPg vs SgFn | SgPgFn vs SgFn | SgPgFn vs SgPg | Coverage

Protein	*Sg* n	*Sg* Coverage	*SgFn* n	*SgFn* Coverage	*SgPg* n	*SgPg* Coverage	*SgPgFn* n	*SgPgFn* Coverage	Description
**SGO_0095**			8	39.1%	8	38.8%			mccF; microcin immunity protein MccF, putative
			9	47.3%	6	34.4%			
**SGO_0097**	3	12.4%							hypothetical protein SGO_0097
	5	22.5%							
**SGO_0098**	3	21.7%			3	21.7%			ribonucleotide reductase-like protein
					3	21.7%			
**SGO_0099**			4	11.3%	7	16.6%	3	5.6%	pulA-2; pullulanase, type I
			7	15.8%	6	16.6%			
**SGO_0100**	6	27.4%							maltose operon transcription repressor
	4	20.7%	4	21.6%	3	11.9%			
**SGO_0101**	5	25.8%							malA; Maltodextrose utilization protein malA
	10	37.5%							
**SGO_0104**	23	59.6%	19	56.8%	23	61.8%	20	57.7%	Maltose/maltodextrin-binding protein precursor
	26	65.1%	18	56.8%	23	61.8%	17	50.1%	
**SGO_0105**	8	19.6%	8	24.6%	9	21.5%	4	9.1%	malQ; 4-alpha-glucanotransferase
	9	19.8%	8	22.3%	9	21.7%	3	6.6%	
**SGO_0106**	12	20.5%	27	49.1%	23	42.0%	17	31.9%	glgP-2; maltodextrin phosphorylase
	15	22.7%	24	47.5%	28	51.1%	15	29.3%	
**SGO_0107**									LPXTG cell wall surface protein, collagen binding domain
							4	5.6%	
**SGO_0108**	8	30.7%	4	15.4%					ruvB; Holliday junction DNA helicase RuvB
	9	30.7%	3	13.9%					
**SGO_0110**					3	40.7%			phosphotyrosine protein phosphatase
			4	46.4%					

Sg Rep I Rep II | *SgFn* Rep I Rep II | *SgPg* Rep I Rep II | *SgPgFn* Rep I Rep II

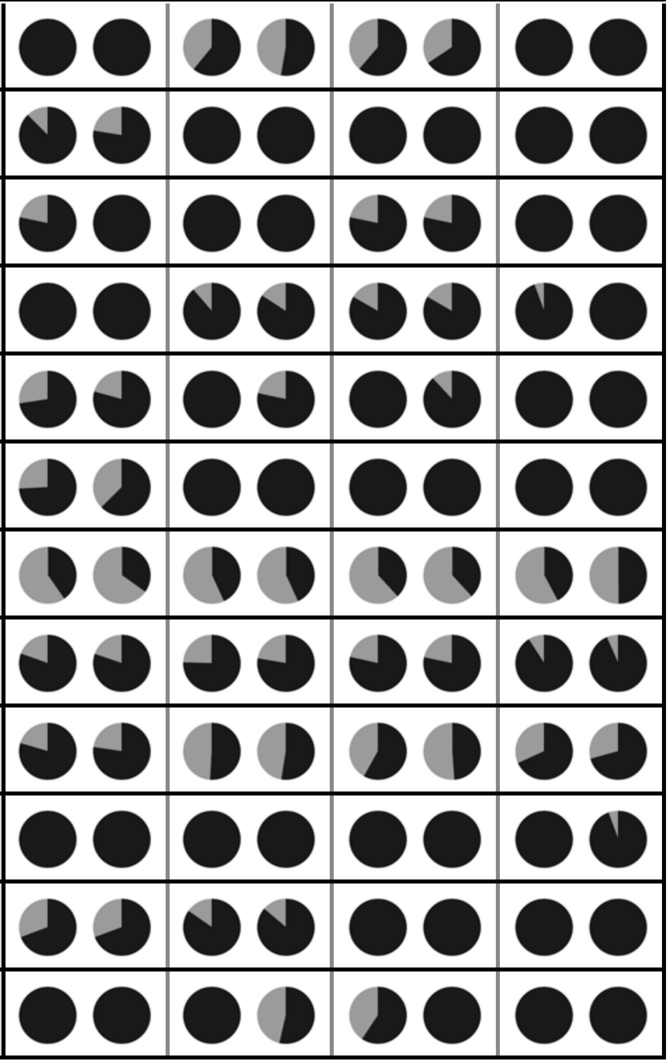

Show detected proteins only
Show all proteins
Filter by category:
ABC Transporter

Proteins found: 1179

Dot Plots Dot Plots

Hendrickson *et al.*

Protein	*Sg* n	*Sg* Coverage	*SgFn* n	*SgFn* Coverage	*SgPg* n	*SgPg* Coverage	*SgPgFn* n	*SgPgFn* Coverage	Description
SGO_0111	4	27.0%							MORN motif family protein
	6	36.5%							
SGO_0112	10	22.0%							putative acyltransferase
	9	22.2%							
SGO_0113	53	63.8%	53	66.8%	47	60.5%	45	59.1%	acdH; alcohol-acetaldehyde dehydrogenase
	57	66.0%	51	76.3%	48	60.9%	45	60.2%	
SGO_0128									hypothetical protein SGO_0128
	5	60.7%							
SGO_0129	13	29.2%			4	11.1%			atpI; v-type sodium ATP synthase, chain I
	16	31.6%			4	8.8%			
SGO_0131	6	34.7%	3	27.6%					v-type sodium ATP synthase, chain E
	6	35.2%	4	29.1%					
SGO_0132					3	12.8%			ATP synthase (C/AC39) subunit
	3	13.1%			4	15.4%			
SGO_0134	6	27.3%			6	26.4%			acetyltransferase, GNAT family
	8	39.0%	4	17.7%	7	35.1%			
SGO_0135	22	49.0%	16	34.9%	13	28.4%	8	16.1%	v-type sodium ATP synthase, subunit A
	24	49.5%	10	23.3%	18	48.5%	9	20.0%	
SGO_0136	22	54.7%	16	48.9%	12	43.5%	10	36.2%	v-type sodium ATP synthase, chain B
	22	54.5%	14	44.6%	13	45.9%	10	36.2%	
SGO_0137	3	16.4%							V-type ATPase, D subunit
	4	15.5%							
SGO_0138									LysM domain protein
	4	17.7%	5	22.0%					

Sg Rep I Rep II | *SgFn* Rep I Rep II | *SgPg* Rep I Rep II | *SgPgFn* Rep I Rep II

Show detected proteins only
Show all proteins
Filter by category:
ABC Transporter

Proteins found:
1179

Dot Plots Dot Plots

Hendrickson *et al.*

Streptococcus gordonii

Protein	*Sg* n	*Sg* Coverage	*SgFn* n	*SgFn* Coverage	*SgPg* n	*SgPg* Coverage	*SgPgFn* n	*SgPgFn* Coverage	Description
**SGO_0139**	11	37.0%	14	44.9%	12	43.5%	10	30.0%	thrC; threonine synthase
	12	38.3%	11	35.8%	13	42.9%	6	19.8%	
**SGO_0142**									hypothetical protein SGO_0142
	3	15.5%							
**SGO_0144**	14	15.6%	4	4.2%	3	1.9%			hypothetical protein SGO_0144
	18	18.1%	3	3.1%					
**SGO_0145**	31	45.3%	25	41.1%	21	34.8%	11	19.9%	polI; DNA polymerase I
	25	37.6%	21	34.7%	22	38.1%	11	22.0%	
**SGO_0146**	5	41.4%	7	58.6%	7	58.6%	5	41.4%	CoA-binding domain protein
	6	46.2%	7	66.9%	5	46.2%	3	23.4%	
**SGO_0148**	5	33.3%			3	21.1%			hypothetical protein SGO_0148
	7	38.2%							
**SGO_0150**	3	10.0%							fucA1; alpha-L-fucosidase
**SGO_0152**	4	21.1%	6	31.3%	5	27.4%	3	17.9%	tgt; queuine tRNA-ribosyltransferase
	4	21.1%	4	17.4%	7	36.3%	4	19.7%	
**SGO_0154**	16	47.9%	32	71.3%	26	63.3%	18	53.9%	pgi; glucose-6-phosphate isomerase
	21	48.6%	33	74.6%	23	61.0%	20	54.8%	
**SGO_0155**	15	52.6%			3	16.6%			hypothetical protein SGO_0155
	15	57.0%			5	16.6%			
**SGO_0156**	9	41.0%			3	16.4%	3	16.4%	hypothetical protein SGO_0156
	11	42.5%			3	16.4%	3	17.2%	
**SGO_0157**	4	16.8%							hypothetical protein SGO_0157
	5	20.6%							

Sg Rep I / Rep II | *SgFn* Rep I / Rep II | *SgPg* Rep I / Rep II | *SgPgFn* Rep I / Rep II

Hendrickson *et al.*

Protein	*Sg* n	*Sg* Coverage	*SgFn* n	*SgFn* Coverage	*SgPg* n	*SgPg* Coverage	*SgPgFn* n	*SgPgFn* Coverage	Description
SGO_0158	8	42.2%	7	38.8%	7	41.4%	6	37.9%	2,3,4,5-tetrahydropyridine-2-carboxylate N-succinyltransferase, putative
	8	42.2%	6	34.9%	8	41.4%	8	45.3%	
SGO_0159			3	10.6%	3	10.6%			hippurate hydrolase
	5	19.9%							
SGO_0163	7	36.4%	11	48.3%	10	45.4%	6	29.1%	galU; UTP-glucose-1-phosphate uridylyltransferase
	8	40.1%	10	48.7%	9	41.7%	8	40.7%	
SGO_0164	12	55.9%	14	69.2%	11	56.2%	8	38.2%	Glycerol-3-phosphate dehydrogenase [NAD(P)+] (NAD(P)H-dependent glycerol-3-phosphate dehydrogenase)
	13	61.5%	12	60.7%	11	58.6%	9	41.1%	
SGO_0165	9	22.3%							ABC transporter, permease/ATP-binding protein SP2075
	8	17.8%							
SGO_0166	11	26.5%							ABC transporter, permease/ATP-binding protein SP2073
	10	25.1%			3	3.7%			
SGO_0167	5	27.1%							glutamine amidotransferase
	5	32.3%							
SGO_0168	4	30.2%							hydrolase, NUDIX family
	5	39.6%	3	22.8%					
SGO_0169	5	41.5%	5	42.9%	4	42.9%	5	42.9%	dut; dUTP diphosphatase
	5	52.4%	4	28.6%	4	44.2%	4	37.4%	
SGO_0171	7	21.7%	5	20.2%	9	31.8%	3	11.4%	radA; DNA repair protein RadA
	12	40.1%			10	35.0%			
SGO_0172	7	44.1%	3	25.4%					conserved hypothetical protein TIGR00266
	7	44.5%							
SGO_0173	7	56.7%	3	34.1%	4	36.0%			Carbonic anhydrase
	7	56.7%	5	50.0%	4	37.2%			

Sg Rep I Rep II | *SgFn* Rep I Rep II | *SgPg* Rep I Rep II | *SgPgFn* Rep I Rep II

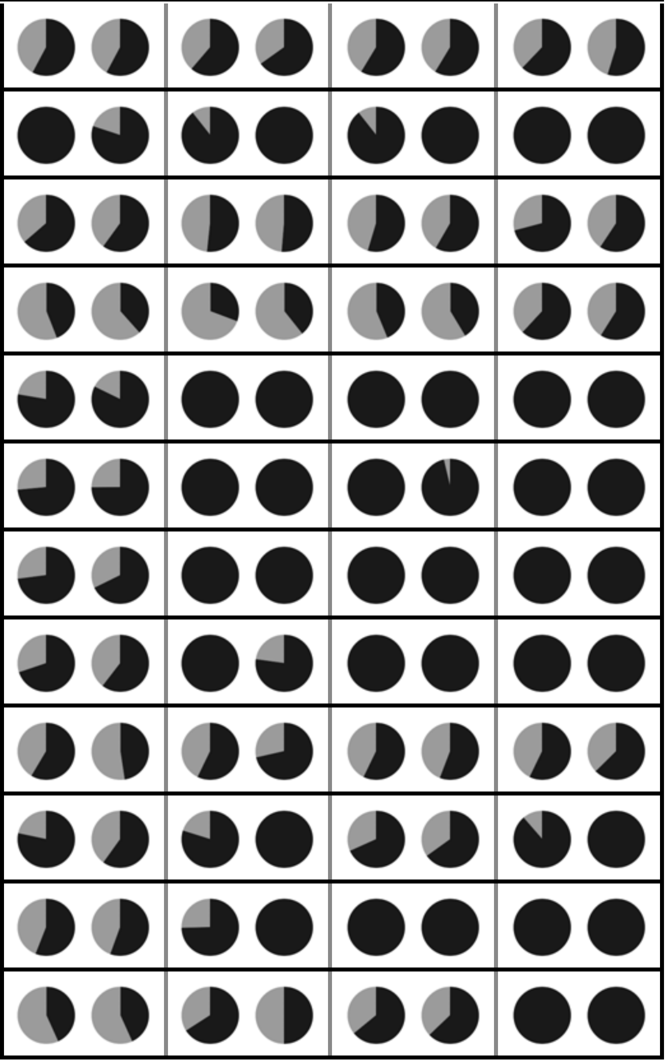

Hendrickson *et al.*

Protein	*Sg* n	*Sg* Coverage	*SgFn* n	*SgFn* Coverage	*SgPg* n	*SgPg* Coverage	*SgPgFn* n	*SgPgFn* Coverage	Description
**SGO_0174**	22	52.2%	19	45.4%	27	57.9%	17	44.9%	gltX; glutamyl-tRNA synthetase
	22	53.2%	23	54.8%	23	56.9%	18	47.4%	
**SGO_0179**									Membrane protein oxaA 1 precursor
	3	7.0%							
**SGO_0180**	11	34.1%	7	30.9%	8	30.9%	3	10.3%	jag; hypothetical protein SGO_0180
	13	38.4%	4	19.7%	7	25.3%	8	28.1%	
**SGO_0181**	12	40.4%							lipoprotein, putative
	15	55.1%							
**SGO_0182**			3	20.5%					sapR; sakacin A production response regulator
	3	21.0%	4	32.4%					
**SGO_0188**	5	20.7%	6	35.9%					hydrolase, TatD family
	6	27.0%	3	17.6%	3	14.5%			
**SGO_0189**									primase-related protein
	3	20.4%							
**SGO_0190**	9	34.2%			3	15.8%			hypothetical protein SGO_0190
	16	51.8%			4	20.1%	3	15.5%	
**SGO_0191**	7	17.8%			4	14.9%			hypothetical protein SGO_0191
	9	27.0%							
**SGO_0193**	3	14.1%	3	12.8%	7	32.8%			ksgA; dimethyladenosine transferase
	5	20.7%	4	17.9%	6	24.5%			
**SGO_0197**			4	25.7%	3	20.2%			predicted ribosome small subunit-dependent GTPase A
			6	39.7%	5	37.3%	3	17.8%	
**SGO_0198**	8	51.1%	10	71.2%	10	65.3%	7	35.6%	rpe; ribulose-phosphate 3-epimerase
	7	37.9%	10	74.4%	9	58.9%	5	29.7%	

Streptococcus gordonii

Protein	*Sg* n	*Sg* Coverage	*SgFn* n	*SgFn* Coverage	*SgPg* n	*SgPg* Coverage	*SgPgFn* n	*SgPgFn* Coverage	Description
SGO_0200	15	42.6%	10	38.8%	12	45.0%	5	20.3%	competence-induced protein Ccs50
	21	51.9%	9	38.3%	11	45.2%	7	30.9%	
SGO_0201	10	46.3%	12	48.2%	11	47.9%	6	26.2%	cmp-binding-factor 1
	12	51.1%	13	49.2%	13	52.1%	7	26.8%	
SGO_0202	3	20.3%	5	33.6%	4	25.5%			pur operon repressor
	4	25.8%	5	32.1%	5	33.2%			
SGO_0203									hypothetical protein SGO_0203
	4	27.9%							
SGO_0204	7	25.5%	8	45.3%	7	39.4%	8	45.3%	rpsL; ribosomal protein S12
	7	25.5%	6	43.8%	7	39.4%	8	45.3%	
SGO_0205	12	67.3%	11	62.8%	11	59.6%	9	60.3%	rpsG; ribosomal protein S7
	14	74.4%	13	80.1%	10	59.0%	10	53.8%	
SGO_0206	41	83.5%	38	79.2%	42	83.5%	38	77.5%	fusA; translation elongation factor G
	46	86.0%	44	97.4%	45	83.8%	38	79.8%	
SGO_0207	14	47.9%	25	69.3%	19	62.2%	16	58.6%	gap; glyceraldehyde-3-phosphate dehydrogenase, type I
	15	50.6%	27	91.1%	18	59.5%	18	58.9%	
SGO_0208	12	9.7%	13	11.3%					LPXTG cell wall surface protein, glycosyl hydrolase family
	16	14.3%	12	9.9%					
SGO_0209	26	72.2%	29	76.7%	27	81.5%	26	80.5%	pgk; phosphoglycerate kinase
	29	72.9%	29	87.2%	28	82.5%	24	72.2%	
SGO_0210	17	14.3%	20	21.1%	3	2.1%			sspA; streptococcal surface protein A
	28	20.6%	15	18.4%	4	4.3%			
SGO_0211	20	13.4%	4	3.0%					sspB; streptococcal surface protein B
	18	13.2%	5	4.5%					

Hendrickson *et al.*

Protein	*Sg* n	*Sg* Coverage	*SgFn* n	*SgFn* Coverage	*SgPg* n	*SgPg* Coverage	*SgPgFn* n	*SgPgFn* Coverage	Description
**SGO_0214**									transcription regulator, MerR family
	4	30.6%							
**SGO_0215**	16	47.1%	17	48.7%	18	54.5%	15	49.6%	glnA; glutamine synthetase, type I
	20	52.7%	19	56.3%	16	52.5%	12	34.4%	
**SGO_0219**	17	44.3%	18	45.0%	20	51.3%	10	23.2%	metallo-beta-lactamase superfamily protein 1
	17	44.5%	15	38.8%	23	58.9%	11	27.0%	
**SGO_0220**			3	53.9%	4	59.2%			Protein of unknown function (DUF1447) superfamily
					5	68.4%			
**SGO_0223**	3	11.3%	3	11.6%					glycoproteinase family protein
	3	11.3%			3	12.2%			
**SGO_0230**	4	25.6%	6	40.0%	6	37.8%	4	27.2%	Protein of unknown function, DUF536 family
	8	43.3%	5	31.1%	5	31.1%	7	44.4%	
**SGO_0231**	4	11.1%							glycerophosphoryl diester phosphodiesterase family protein
	6	17.1%							
**SGO_0232**					3	26.9%	3	26.9%	conserved hypothetical protein TIGR00103
	4	33.6%			3	26.9%	3	26.9%	
**SGO_0233**	7	31.9%	3	26.7%					lipoprotein, putative
	10	48.9%	6	45.2%					
**SGO_0234**	20	35.3%	18	35.6%	19	34.8%	13	23.7%	pepX; X-Pro dipeptidyl-peptidase
	21	36.2%	19	38.5%	18	30.8%	5	10.0%	
**SGO_0235**	3	11.3%			4	21.9%			glycerol uptake facilitator protein-like protein
	4	14.1%			3	18.7%			
**SGO_0236**	6	12.0%							hypothetical protein SGO_0236
	6	11.7%							

Sg Rep I Rep II | *SgFn* Rep I Rep II | *SgPg* Rep I Rep II | *SgPgFn* Rep I Rep II

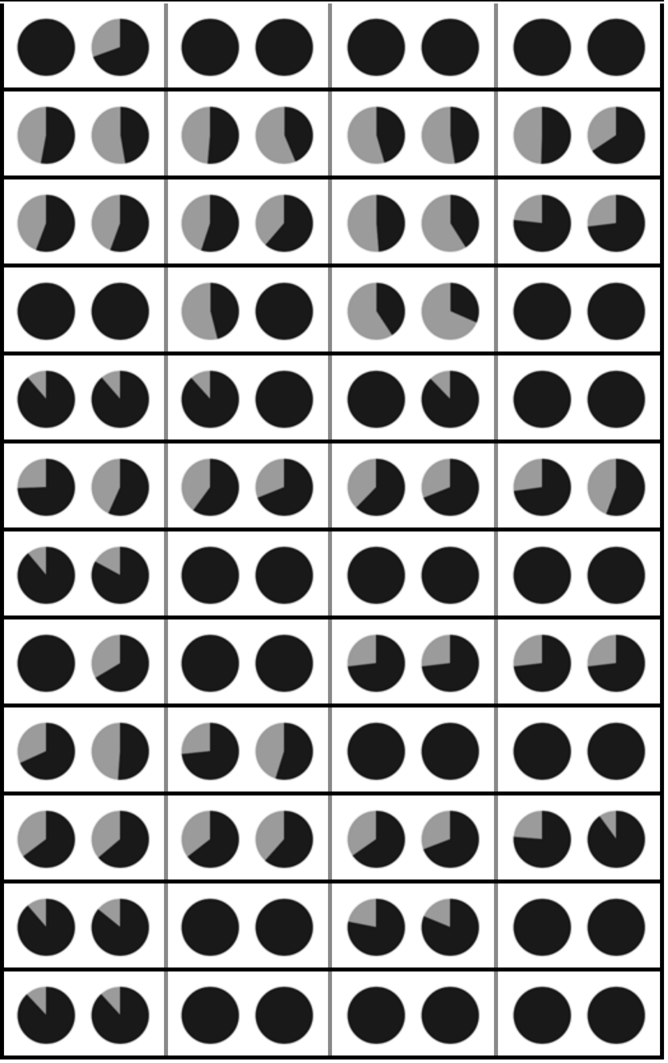

Hendrickson *et al.*

Protein	*Sg* n	*Sg* Coverage	*SgFn* n	*SgFn* Coverage	*SgPg* n	*SgPg* Coverage	*SgPgFn* n	*SgPgFn* Coverage	Description
**SGO_0237**	3	18.0%	4	26.1%	7	38.0%	3	17.1%	ccpA; CcpA protein (proteinase)
	8	42.4%	6	36.7%	8	45.7%	3	18.0%	
**SGO_0240**	5	23.8%							mvaD; diphosphomevalonate decarboxylase
	4	21.3%							
**SGO_0242**	3	10.8%	3	14.7%					FMN-dependent dehydrogenase family protein
**SGO_0243**			3	6.6%	3	12.3%			hydroxymethylglutaryl-CoA reductase, degradative
			4	9.4%	4	15.8%	5	14.2%	
**SGO_0244**	3	8.7%	7	20.9%	8	31.6%	6	19.9%	hydroxymethylglutaryl-CoA synthase
	5	18.1%	7	28.3%	8	28.1%			
**SGO_0247**	45	67.2%	43	66.4%	39	66.7%	33	51.1%	pfl; formate acetyltransferase
	47	68.1%	43	69.3%	38	62.6%	30	51.9%	
**SGO_0252**	7	32.1%	4	16.0%	6	26.9%	3	9.4%	possible TetR-type transcriptional regulator
	6	26.9%	3	14.6%	3	14.6%			
**SGO_0253**	10	42.7%							hypothetical protein SGO_0253
	12	36.9%							
**SGO_0254**	6	13.1%			6	12.1%			helicase, RecD/TraA family
	11	21.3%			4	9.5%			
**SGO_0255**	11	48.5%	7	46.5%	4	29.0%	5	19.0%	Signal peptidase I
	13	48.5%	5	33.0%	6	39.5%	5	26.5%	
**SGO_0256**	4	17.9%							rnhC; ribonuclease HIII
	6	22.3%	3	16.9%					
**SGO_0258**									hypothetical protein SGO_0258
	3	23.8%	3	56.4%			3	27.7%	

Sg Rep I Rep II | *SgFn* Rep I Rep II | *SgPg* Rep I Rep II | *SgPgFn* Rep I Rep II

Show detected proteins only
Show all proteins
Filter by category:
ABC Transporter

Proteins found: 1179

Dot Plots Dot Plots

Hendrickson *et al.*

Protein	*Sg* n	*Sg* Coverage	*SgFn* n	*SgFn* Coverage	*SgPg* n	*SgPg* Coverage	*SgPgFn* n	*SgPgFn* Coverage	Description
**SGO_0260**	16	29.7%	8	17.5%	13	24.3%	6	8.4%	DNA mismatch binding protein MutS2
	23	40.2%	11	22.4%	15	28.7%	7	10.8%	
**SGO_0261**									acetyltransferase, GNAT family
	3	16.4%							
**SGO_0262**	11	34.1%	9	29.7%	12	32.8%	7	19.9%	dipeptidase
	9	30.5%	9	32.4%	12	30.3%	5	15.9%	
**SGO_0263**	4	42.3%	4	42.3%	4	42.3%	4	42.3%	trx-1; thioredoxin
	4	42.3%	6	67.3%	4	42.3%	3	41.3%	
**SGO_0268**	10	45.5%							mechanosensitive transport protein
	8	38.7%			4	17.2%			
**SGO_0269**	3	22.5%							hypothetical protein SGO_0269
	4	31.9%							
**SGO_0272**	6	55.7%	7	54.9%	5	42.6%	4	39.3%	hypothetical protein SGO_0272
	5	48.4%	6	49.2%	5	42.6%	4	39.3%	
**SGO_0276**	19	44.9%	24	63.2%	23	59.2%	16	42.4%	gdhA; glutamate dehydrogenase (NADP)
	24	48.2%	22	62.9%	25	65.4%	20	55.6%	
**SGO_0277**	5	31.7%	4	28.8%	5	35.3%			pyrA; Dihydroorotate dehydrogenase
	5	31.7%	5	35.6%	4	27.9%			
**SGO_0278**			3	15.8%					msrA; Peptide methionine sulfoxide reductase msrA/msrB
	3	14.8%							
**SGO_0279**	6	29.4%							lipoprotein, putative
	4	23.7%							
**SGO_0280**	6	20.4%	10	41.0%	7	20.9%			trzA; ethylammeline chlorohydrolase
	7	23.4%	6	23.6%	6	20.6%			

Show detected proteins only
Show all proteins
Filter by category:
ABC Transporter

Proteins found:
1179

Dot Plots Dot Plots

Hendrickson *et al.*

Protein	*Sg* n	*Sg* Coverage	*SgFn* n	*SgFn* Coverage	*SgPg* n	*SgPg* Coverage	*SgPgFn* n	*SgPgFn* Coverage	Description
**SGO_0282**	9	27.1%							cell wall polysaccharide biosynthesis protein
	11	37.6%							
**SGO_0284**	3	9.3%							transcriptional regulator
**SGO_0286**	7	16.0%							DNA mismatch repair protein MutS, putative
	10	24.5%	3	7.7%			3	8.3%	
**SGO_0288**					3	21.1%			GDSL-like lipase/acylhydrolase
			3	21.6%	4	30.9%			
**SGO_0290**	3	28.5%			3	28.5%	3	28.5%	copper -translocating P-type ATPase
	3	28.5%			3	28.5%			
**SGO_0291**	16	31.1%	8	19.3%	9	20.7%	7	16.6%	copper-translocating P-type ATPase
	16	33.5%	6	13.4%	9	20.8%	6	13.9%	
**SGO_0292**	19	45.9%	14	34.7%	28	64.1%	23	55.5%	spxB; pyruvate oxidase
	24	51.1%	11	35.4%	29	64.1%	24	60.2%	
**SGO_0294**	4	8.6%							ABC transporter, putative
	5	10.1%							
**SGO_0297**			6	15.9%					6-phospho-beta-glucosidase
	4	11.5%	9	27.6%					
**SGO_0299**									putative histidine kinase
	3	11.0%							
**SGO_0301**							3	14.2%	ABC transporter ATP-binding protein-like protein
**SGO_0306**	4	17.9%							ABC transporter ATP-binding protein-like protein
	6	21.9%							

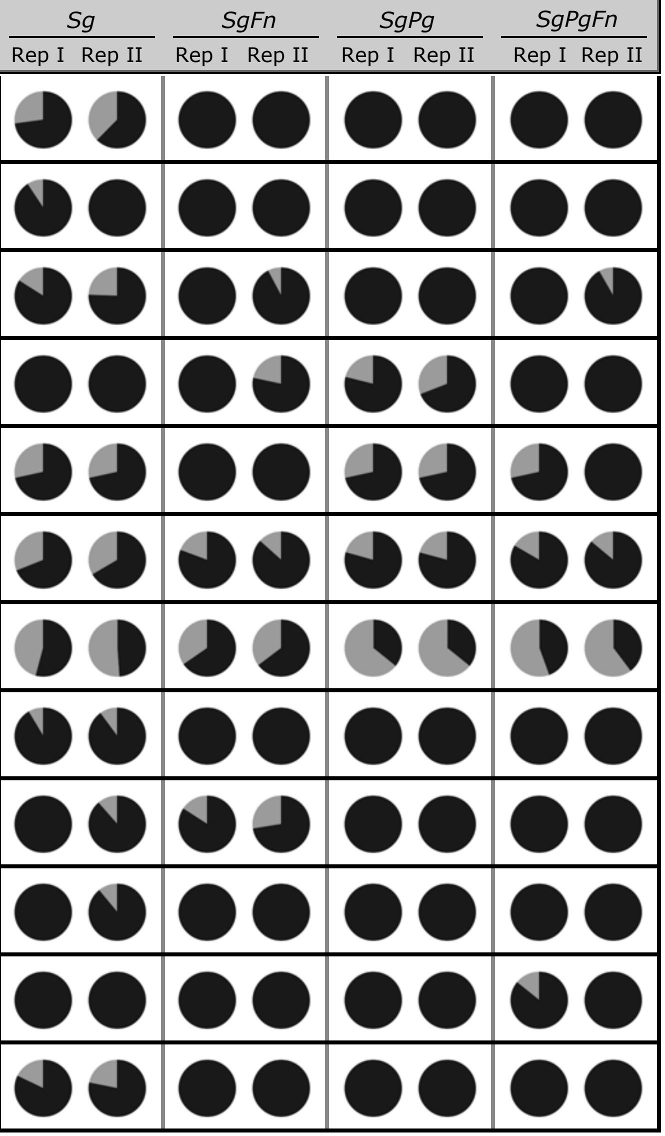

Show detected proteins only
Show all proteins
Filter by category:
ABC Transporter

Proteins found:
1179

Dot Plots Dot Plots

Hendrickson *et al.*

Protein	*Sg* n	*Sg* Coverage	*SgFn* n	*SgFn* Coverage	*SgPg* n	*SgPg* Coverage	*SgPgFn* n	*SgPgFn* Coverage	Description
**SGO_0307**	4	8.8%							pabB; chorismate binding enzyme
	6	12.5%							
**SGO_0310**	3	5.5%	4	8.7%	4	6.8%			metE; 5-methyltetrahydropteroyltriglutamate--homocysteine S-methyltransferase
	3	4.9%	5	8.3%					
**SGO_0312**	33	46.3%	45	58.4%	40	56.9%	34	53.5%	xfp; D-xylulose 5-phosphate/D-fructose 6-phosphate phosphoketolase
	34	48.5%	55	71.2%	41	57.2%	38	55.5%	
**SGO_0317**	43	46.8%							LPXTG cell wall surface protein, serine protease, subtilase family
	52	50.1%					3	2.6%	
**SGO_0321**	8	57.8%	8	61.3%	7	51.5%	5	42.2%	polypeptide deformylase
	10	71.1%	8	57.4%	8	56.4%	6	42.2%	
**SGO_0323**					3	18.6%			pseudouridine synthase rRNA-specific
	3	14.9%							
**SGO_0324**	10	22.6%							hypothetical protein SGO_0324
	10	22.3%							
**SGO_0325**	7	25.4%							probable membrane protein, putative
**SGO_0326**	19	38.9%			3	8.5%			probable membrane protein, putative
	23	46.5%							
**SGO_0327**	5	17.2%			3	11.4%			Lipopolysaccharide N-acetylglucosaminyltransferase
	4	13.7%	4	12.4%					
**SGO_0328**	5	9.8%							transmembrane protein, putative
	5	9.8%							
**SGO_0329**	20	47.0%							hypothetical protein SGO_0329
	21	49.4%			3	8.6%			

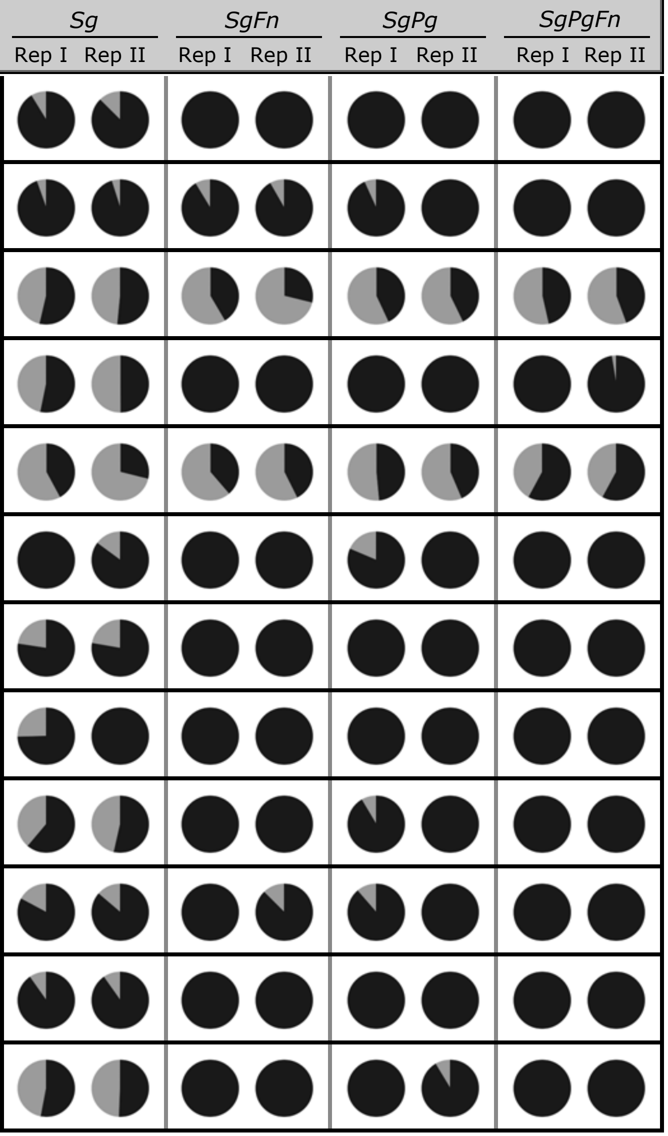

Show detected proteins only
Show all proteins
Filter by category:
ABC Transporter

Proteins found: 1179

Dot Plots Dot Plots

Hendrickson *et al.*

Protein	*Sg* n	*Sg* Coverage	*SgFn* n	*SgFn* Coverage	*SgPg* n	*SgPg* Coverage	*SgPgFn* n	*SgPgFn* Coverage	Description
SGO_0330	8	40.1%							hypothetical protein SGO_0330
	8	38.5%							
SGO_0331	4	19.7%							hypothetical protein SGO_0331
	6	23.3%							
SGO_0332	12	40.6%							hypothetical protein SGO_0332
	14	40.8%							
SGO_0333	8	34.8%	9	68.5%	11	68.5%	10	38.2%	rpsO; ribosomal protein S15
	11	68.5%	8	66.3%	11	68.5%	10	66.3%	
SGO_0337					3	33.7%			trx-2; thioredoxin
	3	34.6%							
SGO_0339			7	27.0%	3	11.8%			hypothetical protein SGO_0339
	4	16.3%	7	31.9%					
SGO_0342	15	34.6%	19	44.5%	17	39.1%	12	26.9%	pepF-2; oligoendopeptidase
	15	33.4%	21	45.0%	18	43.0%	10	19.2%	
SGO_0344	27	48.1%	29	47.3%	28	48.4%	22	38.6%	pnpA; polyribonucleotide nucleotidyltransferase
	31	48.5%	27	44.1%	26	50.4%	16	26.0%	
SGO_0345	3	16.1%							cysE; serine O-acetyltransferase
	6	23.4%							
SGO_0348			5	48.0%	3	31.8%			reductase
	4	36.4%			3	31.8%			
SGO_0349	11	32.2%	10	28.6%	12	36.5%	10	25.7%	cysS; cysteinyl-tRNA synthetase
	11	31.8%	11	31.1%	11	32.7%	7	18.3%	
SGO_0352	14	42.3%	12	40.7%	13	48.4%	7	26.1%	ABC transporter, ATP-binding protein SP1580
	16	47.6%	12	37.8%	12	39.6%	6	21.5%	

Show detected proteins only
Show all proteins
Filter by category:
ABC Transporter

Proteins found: 1179

Dot Plots Dot Plots

Hendrickson *et al.*

Protein	*Sg* n	*Sg* Coverage	*SgFn* n	*SgFn* Coverage	*SgPg* n	*SgPg* Coverage	*SgPgFn* n	*SgPgFn* Coverage	Description
**SGO_0353**	3	6.0%	4	7.6%	5	7.8%			transport protein
	3	5.2%	3	6.3%	3	6.0%			
**SGO_0355**	5	27.6%	4	20.6%	6	35.0%			RNA methyltransferase, TrmH family, group 3
	7	28.0%	4	23.5%	5	30.9%			
**SGO_0356**	3	16.4%							hypothetical protein SGO_0356
**SGO_0357**	8	44.3%	10	59.6%	11	59.6%	8	57.1%	degV; DegV family fatty acid binding protein
	9	47.4%	6	39.0%	12	63.8%	9	47.7%	
**SGO_0358**	11	78.4%	10	73.0%	9	65.5%	8	70.3%	rplM; ribosomal protein L13
	11	79.1%	12	77.7%	11	79.1%	10	75.7%	
**SGO_0359**	5	27.7%	6	28.5%	5	21.5%	5	21.5%	rpsI; ribosomal protein S9
	6	28.5%	4	20.8%	5	21.5%	5	21.5%	
**SGO_0361**	6	33.1%	4	35.5%	5	40.7%			immunity repressor protein
	5	33.1%	5	35.5%	3	28.5%			
**SGO_0368**	3	9.8%	5	11.9%			3	9.4%	merA; mercury(II) reductase
	4	10.9%	5	13.2%					
**SGO_0371**									putative transcriptional regulator
	4	39.7%							
**SGO_0372**	6	18.9%	12	25.7%	8	30.1%	7	25.1%	malate oxidoreductase
	8	22.2%	10	24.0%	7	23.1%	6	17.4%	
**SGO_0374**	7	29.5%	9	40.6%	8	40.6%	5	23.4%	Response regulator of the LytR/AlgR family
	8	34.4%	9	46.3%	9	41.8%	4	14.3%	
**SGO_0376**							4	24.3%	ABC transporter ATP-binding protein
	5	23.9%							

Sg Rep I Rep II | *SgFn* Rep I Rep II | *SgPg* Rep I Rep II | *SgPgFn* Rep I Rep II

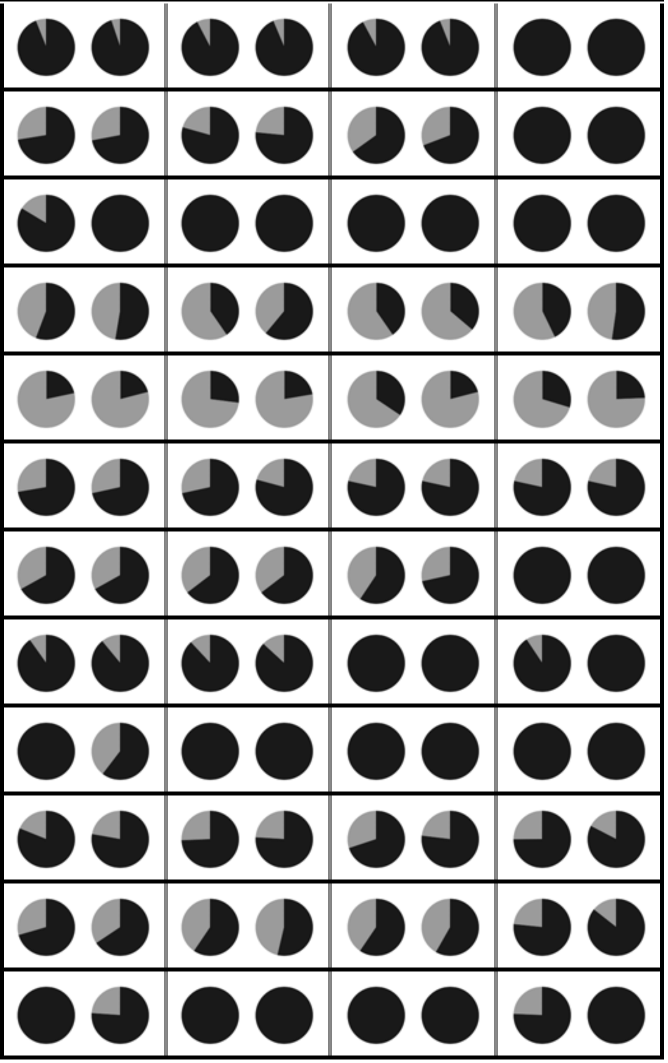

Show detected proteins only
Show all proteins
Filter by category:
ABC Transporter

Proteins found: 1179

Dot Plots Dot Plots

Protein	*Sg* n	*Sg* Coverage	*SgFn* n	*SgFn* Coverage	*SgPg* n	*SgPg* Coverage	*SgPgFn* n	*SgPgFn* Coverage	Description
**SGO_0378**			3	29.9%					hypothetical protein SGO_0378
**SGO_0379**	6	31.6%							Protein of unknown function (DUF421) family
	7	34.9%							
**SGO_0380**									hypothetical protein SGO_0380
	3	23.3%							
**SGO_0384**	3	15.7%	4	23.1%	3	15.7%	4	25.3%	putative carboxylate-amine/thiol ligase
	5	22.8%	6	29.2%	6	34.3%	5	29.2%	
**SGO_0385**	19	19.9%	21	21.7%					exo-beta-D-fructosidase
	24	27.5%	18	19.0%					
**SGO_0387**	4	22.1%							hypothetical protein SGO_0387
	6	31.7%							
**SGO_0388**	19	25.3%	16	26.6%					LPXTG cell wall surface protein, zinc carboxypeptidase family
	27	36.4%	13	18.7%					
**SGO_0389**			3	37.5%					possible phosphoserine phosphatase
	3	28.4%	3	45.5%					
**SGO_0390**	14	43.4%	19	51.2%	23	56.4%	11	34.4%	glycerol-3-phosphate dehydrogenase (NAD(P)+)
	18	50.3%	16	48.8%	18	49.7%	13	37.1%	
**SGO_0392**			7	36.4%	6	32.5%	3	14.5%	phosphoglycerate mutase
	4	25.4%	5	28.9%	6	32.5%			
**SGO_0393**	8	53.7%	3	16.3%					D-alanyl-D-alanine carboxypeptidase
	10	63.8%							
**SGO_0396**									Transcriptional regulator, PadR family
	3	25.2%							

Show detected proteins only
Show all proteins
Filter by category:
ABC Transporter

Proteins found: 1179

Dot Plots Dot Plots

Hendrickson *et al.*

Streptococcus gordonii

Protein	*Sg* n	*Sg* Coverage	*SgFn* n	*SgFn* Coverage	*SgPg* n	*SgPg* Coverage	*SgPgFn* n	*SgPgFn* Coverage	Description
**SGO_0398**	12	49.5%	4	16.1%	5	20.4%	4	17.5%	ABC transporter ATP-binding protein
	10	35.1%	5	20.7%	3	14.0%	4	17.9%	
**SGO_0400**	4	12.8%	8	27.6%					hrcA; heat-inducible transcription repressor HrcA
	4	12.5%	4	16.6%					
**SGO_0401**	8	41.8%	9	46.9%	7	33.9%	6	33.9%	grpE; co-chaperone GrpE
	10	53.7%	9	53.7%	8	41.8%	7	33.9%	
**SGO_0402**	29	56.8%	40	71.3%	32	64.3%	29	61.6%	dnaK; DnaK chaperone protein
	36	63.8%	42	74.6%	33	64.4%	33	64.6%	
**SGO_0404**	15	42.0%	6	23.6%	8	28.9%	6	19.7%	dnaJ; DnaJ chaparone protein
	21	50.1%	9	26.2%	13	35.7%	8	27.8%	
**SGO_0407**			3	17.3%	3	19.7%			truA; tRNA pseudouridine synthase A
	3	16.9%	3	17.3%					
**SGO_0408**	65	42.9%							zmpB; zinc metalloproteinase B
	68	42.0%					4	3.6%	
**SGO_0409**	3	17.0%	14	67.2%	3	28.5%			pyridoxine kinase
			13	70.8%	4	26.9%			
**SGO_0411**			12	80.7%	3	12.8%	3	12.8%	conserved hypothetical protein TIGR01440
			11	84.5%	3	12.8%			
**SGO_0412**	28	73.8%	33	83.8%	32	81.0%	27	73.1%	tig; trigger factor
	35	85.9%	30	84.3%	32	81.0%	27	73.1%	
**SGO_0413**	5	45.1%	6	46.2%	5	33.3%	4	32.3%	DNA-directed RNA polymerase delta chain
	3	22.6%	7	35.9%	5	33.3%	5	33.3%	
**SGO_0415**	41	59.2%	34	53.6%	39	59.8%	29	46.2%	secA; preprotein translocase, SecA subunit
	45	61.2%	30	50.6%	34	54.9%	33	55.5%	

Sg Rep I Rep II | *SgFn* Rep I Rep II | *SgPg* Rep I Rep II | *SgPgFn* Rep I Rep II

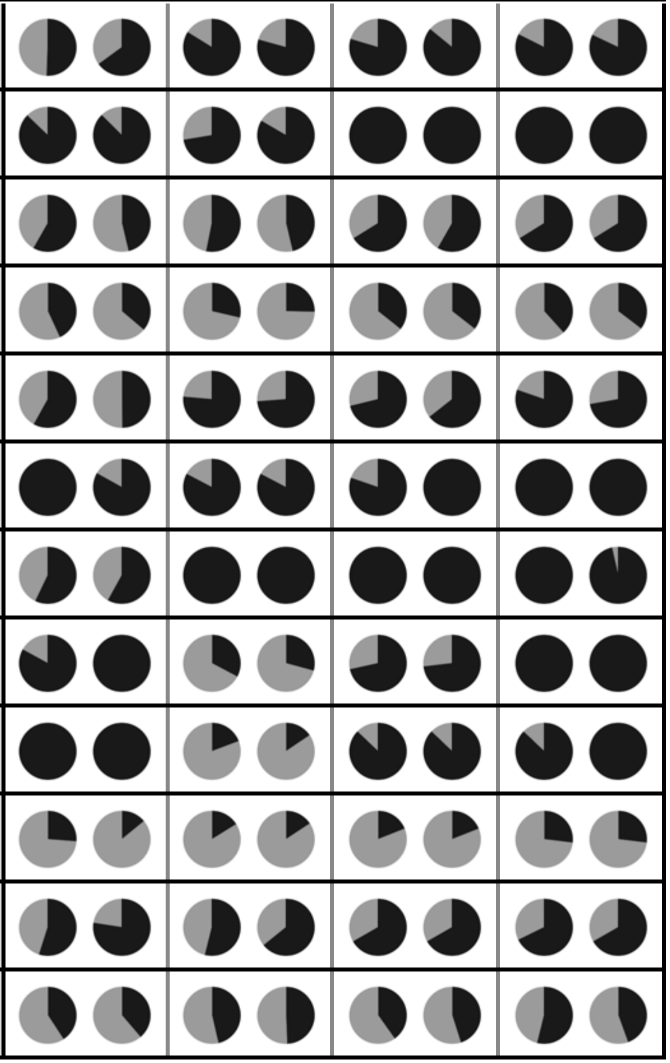

Show detected proteins only
Show all proteins
Filter by category:
ABC Transporter

Proteins found:
1179

Dot Plots Dot Plots

Hendrickson *et al.*

Protein	*Sg*		*SgFn*		*SgPg*		*SgPgFn*		Description
	n	Coverage	n	Coverage	n	Coverage	n	Coverage	
**SGO_0416**	12	44.9%	15	51.0%	15	59.5%	11	42.3%	phospho-2-dehydro-3-deoxyheptonate aldolase
	14	53.6%	14	50.7%	12	46.4%	6	27.4%	
**SGO_0418**			7	26.4%	4	18.5%			alr; alanine racemase
			6	22.8%	4	12.5%			
**SGO_0425**	3	16.9%	4	22.5%	3	16.9%			ansB; asparaginase
**SGO_0426**	10	24.6%	6	16.6%	7	14.9%	5	14.3%	Cof family protein
	13	31.3%	11	32.2%	6	13.2%			
**SGO_0427**	5	38.7%	8	62.7%	6	57.3%	4	30.7%	universal stress protein family
	7	44.7%	8	76.7%	6	57.3%	6	51.3%	
**SGO_0429**	18	56.7%	18	57.2%	20	61.6%	15	51.7%	aspartate transaminase
	18	59.2%	17	56.7%	19	59.9%	16	51.5%	
**SGO_0430**	29	48.6%	17	29.0%					LPXTG cell wall surface protein
	38	57.1%	14	25.3%					
**SGO_0431**	8	32.1%			6	28.6%			GTP-sensing transcriptional pleiotropic repressor codY
	6	23.7%	4	16.4%	5	20.6%			
**SGO_0432**	4	36.2%			3	29.2%	3	29.2%	entB; isochorismatase family protein
	5	40.0%	4	36.2%	3	29.2%	3	29.2%	
**SGO_0434**	19	47.6%	12	33.2%	14	40.1%	11	26.6%	aspS-2; aspartyl-tRNA synthetase
	22	49.7%	17	42.0%	13	36.3%	8	22.5%	
**SGO_0435**	5	59.0%	5	80.0%	5	79.0%	4	41.0%	gatC; glutamyl-tRNA(Gln) amidotransferase, C subunit
	5	51.0%	5	89.0%	5	78.0%	4	49.0%	
**SGO_0436**	22	55.9%	16	42.2%	21	52.7%	16	43.6%	gatA; glutamyl-tRNA(Gln) amidotransferase, A subunit
	25	54.9%	18	43.9%	19	50.6%	19	52.7%	

Columns at right (pie charts): *Sg* Rep I, Rep II; *SgFn* Rep I, Rep II; *SgPg* Rep I, Rep II; *SgPgFn* Rep I, Rep II

Hendrickson *et al.*

Protein	*Sg* n	*Sg* Coverage	*SgFn* n	*SgFn* Coverage	*SgPg* n	*SgPg* Coverage	*SgPgFn* n	*SgPgFn* Coverage	Description
**SGO_0437**	20	47.0%	19	55.6%	22	57.2%	17	43.8%	gatB; glutamyl-tRNA(Gln) amidotransferase, B subunit
	21	48.8%	19	52.0%	21	56.6%	13	30.2%	
**SGO_0440**	4	21.6%	11	49.7%	7	37.1%	4	29.0%	L-iditol 2-dehydrogenase BH3949
	5	26.1%	13	62.4%	4	25.3%			
**SGO_0444**									HAD superfamily (subfamily IIIA) phosphatase, TIGR01668
	3	19.5%							
**SGO_0445**	11	42.1%	12	42.9%	14	54.6%	6	22.0%	GTP-binding protein
	13	48.1%	10	38.6%	14	45.9%	6	21.2%	
**SGO_0446**									conserved hypothetical protein TIGR00253
	3	53.4%							
**SGO_0447**	4	34.3%	6	43.8%	4	38.1%			nadD; nicotinate (nicotinamide) nucleotide adenylyltransferase
	4	24.8%	5	48.6%	3	30.0%			
**SGO_0448**	5	32.7%	9	50.8%	5	35.2%	3	22.1%	conserved hypothetical protein TIGR00488
	4	28.1%	6	40.7%	7	49.2%			
**SGO_0449**									isochorismatase family protein
	3	18.6%							
**SGO_0450**			3	24.0%	4	32.2%			iojap-related protein
	4	38.0%	4	30.6%	3	32.2%			
**SGO_0451**					4	20.9%			methyltransferase
					4	20.9%			
**SGO_0453**	18	56.2%							lipoprotein, putative
	26	64.5%							
**SGO_0454**	6	31.9%	4	25.6%	6	32.8%	3	20.2%	conserved hypothetical protein TIGR01033
	8	40.3%	4	25.6%	4	26.9%	3	20.2%	

Hendrickson *et al.*

Coverage

Streptococcus gordonii

Hackett Laboratory UW

Protein	*Sg* n	*Sg* Coverage	*SgFn* n	*SgFn* Coverage	*SgPg* n	*SgPg* Coverage	*SgPgFn* n	*SgPgFn* Coverage	Description
SGO_0455	21	61.0%	3	8.7%	10	40.9%	6	24.4%	lipoprotein, putative
	21	59.9%			9	35.5%	7	27.6%	
SGO_0456	4	13.8%	5	26.8%					ILL5; amino acid aminohydrolase
	5	17.0%	9	42.2%					
SGO_0457	17	48.8%	6	27.7%	11	36.8%	9	33.0%	ABC transporter, substrate-binding protein SP0148
	23	57.5%	7	34.0%	12	40.0%	6	20.4%	
SGO_0458	24	71.4%	17	67.9%	17	61.3%	15	59.2%	hlpA; lipoprotein
	27	70.7%	11	55.1%	16	61.0%	16	60.6%	
SGO_0459	8	30.9%	6	21.2%	8	24.7%			succinyl-diaminopimelate desuccinylase
	10	35.2%	7	25.2%	4	14.2%			
SGO_0460	12	34.2%	5	13.8%	10	31.4%	6	14.7%	ABC transporter, ATP-binding protein SP0151
	12	33.6%	6	17.8%	8	23.7%	6	18.1%	
SGO_0463	3	6.5%							cydD; putative ABC transporter (ATP-binding protein)
	5	10.9%							
SGO_0468	7	26.3%	7	26.3%	7	29.2%	6	28.5%	hypothetical protein SGO_0468
	8	31.0%	7	26.3%	11	42.3%	5	24.9%	
SGO_0469	3	10.2%							integral membrane protein
	3	10.2%							
SGO_0476	7	31.7%			12	46.6%	9	38.1%	rhodanese family protein
	6	25.0%	3	15.5%	11	48.2%	7	30.5%	
SGO_0480			8	62.8%	3	30.7%			hypothetical protein SGO_0480
	3	32.8%	6	58.4%	3	30.7%	3	30.7%	
SGO_0483	11	45.7%	7	37.1%	7	33.1%	5	26.2%	hypothetical protein SGO_0483
	10	40.1%	5	33.8%	12	55.3%	7	32.1%	

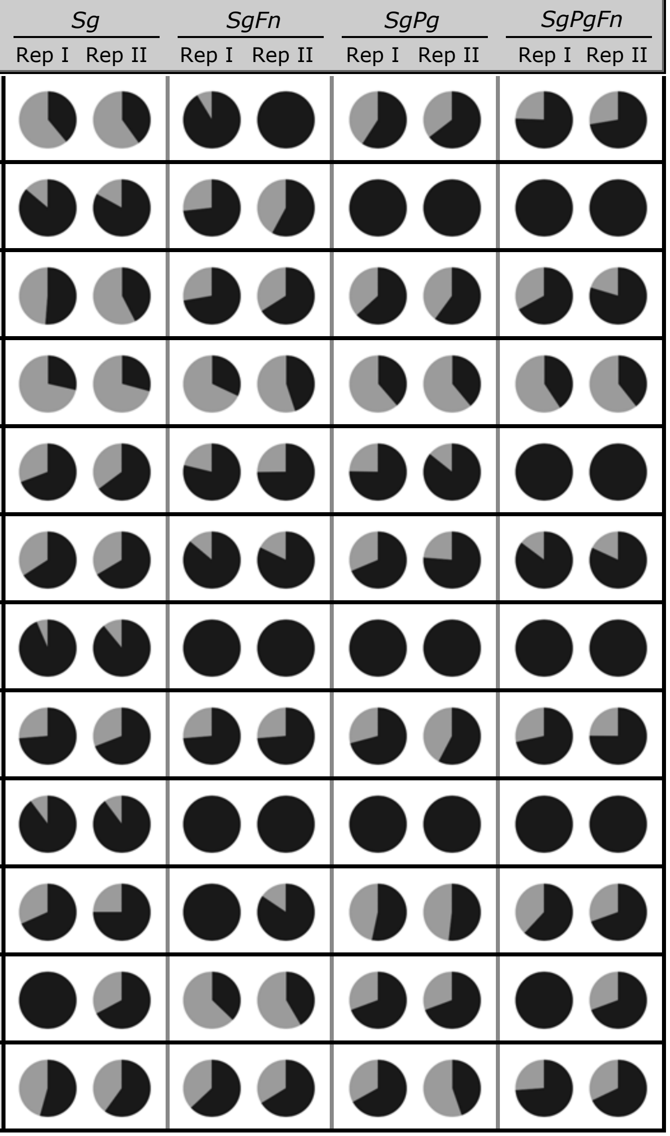

Show detected proteins only
Show all proteins
Filter by category:
ABC Transporter

Proteins found: 1179

Dot Plots Dot Plots

Hendrickson *et al.*

Streptococcus gordonii

Protein	*Sg* n	*Sg* Coverage	*SgFn* n	*SgFn* Coverage	*SgPg* n	*SgPg* Coverage	*SgPgFn* n	*SgPgFn* Coverage	Description	*Sg* Rep I	*Sg* Rep II	*SgFn* Rep I	*SgFn* Rep II	*SgPg* Rep I	*SgPg* Rep II	*SgPgFn* Rep I	*SgPgFn* Rep II
**SGO_0484**	5	18.0%	4	12.9%					sensor histidine kinase								
	6	18.5%															
**SGO_0488**	19	39.8%	4	10.9%	8	21.9%			ABC transporter, ATP-binding protein SP0483								
	18	35.7%	7	21.9%	6	19.1%											
**SGO_0491**			5	31.6%	4	21.1%			gidB; methyltransferase GidB								
			4	22.4%	4	26.6%											
**SGO_0494**	5	27.5%	15	72.0%	8	51.3%			lemA; LemA-like protein								
	8	50.8%	11	68.3%	9	53.4%											
**SGO_0495**	4	21.2%	4	23.2%	3	19.2%			htpx; heat shock protein								
	6	28.6%	5	32.3%													
**SGO_0497**			44	38.3%			3	2.5%	gtfG; glucosyltransferase G								
			45	39.5%													
**SGO_0500**	3	12.1%							rggD; putative transcriptional regulator RggD								
**SGO_0501**	7	43.2%			8	43.2%	6	34.1%	Uncharacterized ACR, COG1399								
	8	50.0%	3	22.7%	7	43.2%	6	34.1%									
**SGO_0502**	29	58.4%	20	47.5%	25	56.2%	22	51.1%	floL; flotillin-like protein								
	34	61.9%	15	37.1%	27	57.6%	21	51.9%									
**SGO_0503**	23	62.2%	33	77.6%	28	74.3%	25	69.2%	gnd; 6-phosphogluconate dehydrogenase, decarboxylating								
	27	65.2%	32	81.9%	31	78.7%	27	66.9%									
**SGO_0505**	21	35.2%	16	34.2%	16	31.0%	10	21.6%	PTS system, IIBC component								
	22	37.5%	14	31.8%	20	36.2%	11	23.6%									
**SGO_0506**									rgfB; RgfB								
	3	15.5%			3	14.8%											

Hendrickson *et al.*

Protein	*Sg* n	*Sg* Coverage	*SgFn* n	*SgFn* Coverage	*SgPg* n	*SgPg* Coverage	*SgPgFn* n	*SgPgFn* Coverage	Description
**SGO_0508**	3	19.7%	4	22.3%	3	19.7%	3	19.7%	nrdR; transcriptional regulator, NrdR family
	3	19.7%			3	19.7%	4	22.3%	
**SGO_0509**	5	16.9%							Replication initiation and membrane attachment protein (DnaB) superfamily
	10	23.7%			4	11.5%			
**SGO_0510**	7	30.1%	8	31.1%	13	60.2%	8	34.4%	dnaI; primosomal protein DnaI
	7	26.1%	7	34.4%	10	37.8%	6	15.1%	
**SGO_0511**	3	16.4%	6	38.2%	5	29.4%	3	17.2%	NADPH-flavin oxidoreductase -like protein
	8	36.6%	7	42.9%	7	34.9%	3	17.2%	
**SGO_0512**	13	35.1%	11	31.0%	11	34.2%	10	33.5%	GTP-binding protein engA
	17	42.0%	8	27.8%	11	36.9%	10	32.8%	
**SGO_0513**			6	10.2%					Snf2 family protein
	4	5.3%	3	4.6%					
**SGO_0514**					4	14.4%			hypothetical protein SGO_0514
					4	24.6%			
**SGO_0515**	12	35.4%	16	47.4%	15	50.3%	8	26.0%	murC; UDP-N-acetylmuramate--alanine ligase
	14	41.5%	15	51.5%	15	50.3%	9	31.8%	
**SGO_0518**	21	48.4%	9	21.0%	15	42.7%	10	26.3%	aminodeoxychorismate lyase-like protein
	26	51.4%	8	23.2%	17	45.9%	9	26.9%	
**SGO_0519**	8	51.3%	6	61.9%	10	71.3%	5	51.3%	greA; transcription elongation factor greA
	10	58.1%	4	39.4%	9	58.8%	6	53.1%	
**SGO_0521**	5	18.7%	3	8.4%	3	8.4%			Membrane protein oxaA 2 precursor
	7	22.6%			3	11.0%			
**SGO_0523**			7	52.4%	6	34.6%			spoU rRNA Methylase family protein
	6	33.7%	9	59.3%	4	26.4%			

Sg Rep I Rep II | *SgFn* Rep I Rep II | *SgPg* Rep I Rep II | *SgPgFn* Rep I Rep II

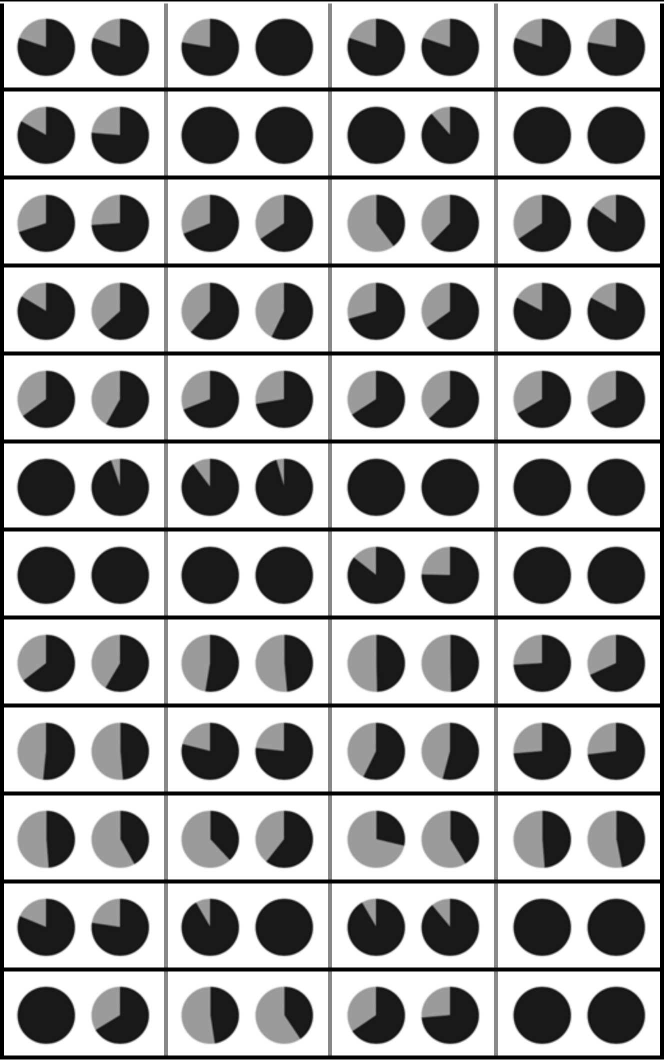

Show detected proteins only
Show all proteins
Filter by category:
ABC Transporter

Proteins found:
1179

Dot Plots Dot Plots

Hendrickson *et al.*

Protein	*Sg* n	*Sg* Coverage	*SgFn* n	*SgFn* Coverage	*SgPg* n	*SgPg* Coverage	*SgPgFn* n	*SgPgFn* Coverage	Description
**SGO_0526**	11	29.4%	6	14.2%	12	31.5%	3	5.4%	ilvB; acetolactate synthase, large subunit, biosynthetic type
	13	29.6%	9	19.4%	13	28.9%	4	9.6%	
**SGO_0527**	4	25.3%	3	25.9%	3	19.0%			ilvN; acetolactate synthase, small subunit
	5	32.3%	3	12.7%	3	20.3%	3	20.9%	
**SGO_0528**	12	40.9%	14	42.9%	16	50.6%	10	38.5%	ilvC; ketol-acid reductoisomerase
	13	43.2%	14	42.4%	17	50.9%	10	33.2%	
**SGO_0529**	3	11.5%	5	16.8%	6	28.1%			ilvA; threonine dehydratase
	7	19.2%	7	26.9%	7	27.6%	3	12.5%	
**SGO_0530**	6	33.3%	5	29.6%	4	27.8%			Cof family protein
	7	37.8%	7	40.0%	6	33.3%			
**SGO_0533**	3	18.8%	3	21.3%					conserved hypothetical protein TIGR00150
					3	18.8%			
**SGO_0535**	10	33.3%	4	16.4%	4	16.9%	3	12.4%	putative transcriptional regulator LytR
	13	40.2%			5	18.9%			
**SGO_0536**	6	60.0%			7	73.0%	7	79.0%	hypothetical protein SGO_0536
	6	60.0%			6	60.0%	4	57.0%	
**SGO_0537**	4	38.7%	3	32.9%	4	38.7%	4	42.8%	HIT family protein
	6	43.4%	4	50.9%	4	38.7%	5	44.5%	
**SGO_0538**	8	39.9%	6	28.0%	4	20.2%			ABC transporter, ATP-binding protein SP0522
	7	39.9%	8	31.7%	4	22.2%			
**SGO_0539**	6	13.1%							ABC transporter, permease protein
	7	16.5%							
**SGO_0540**	11	55.7%	12	58.0%	14	73.5%	10	47.3%	hypothetical protein SGO_0540
	11	53.0%	13	59.1%	12	59.5%	10	46.2%	

Sg Rep I Rep II | *SgFn* Rep I Rep II | *SgPg* Rep I Rep II | *SgPgFn* Rep I Rep II

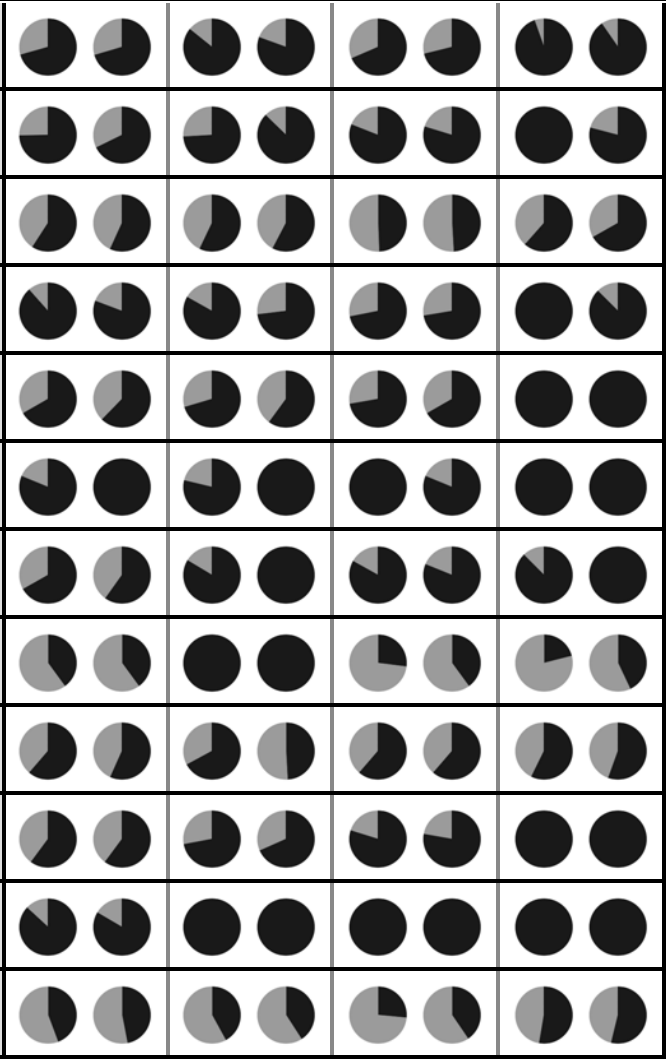

Show detected proteins only
Show all proteins

Filter by category:
ABC Transporter

Proteins found:
1179

Dot Plots Dot Plots

Hendrickson *et al.*

Protein	*Sg* n	*Sg* Coverage	*SgFn* n	*SgFn* Coverage	*SgPg* n	*SgPg* Coverage	*SgPgFn* n	*SgPgFn* Coverage	Description
**SGO_0541**	6	36.3%	5	32.1%	4	20.3%			methyltransferase, putative
	9	49.5%	5	39.2%	7	49.1%			
**SGO_0542**									hypothetical protein SGO_0542
	3	14.2%			4	24.7%			
**SGO_0543**	17	40.0%	9	26.9%	15	37.0%	10	27.6%	nusA; transcription termination factor NusA
	18	41.9%	8	25.4%	16	44.1%	11	30.5%	
**SGO_0544**									Protein of unknown function (DUF448) superfamily
	3	24.5%							
**SGO_0545**									ribosomal protein L7A family
	3	39.4%							
**SGO_0546**	25	33.4%	22	29.9%	26	31.5%	15	22.1%	infB; Translation initiation factor IF-2
	32	39.0%	21	35.0%	27	32.8%	15	19.7%	
**SGO_0547**									rbfA; ribosome-binding factor A
					3	36.5%			
**SGO_0548**	12	27.4%			7	14.7%	4	9.0%	Na/Pi-cotransporter family protein
	14	26.3%	3	9.2%	6	14.5%	4	6.6%	
**SGO_0549**	3	9.1%	3	14.4%	3	11.0%			nagA; N-acetylglucosamine-6-phosphate deacetylase
**SGO_0552**	4	20.6%	15	67.3%	10	53.0%	6	33.8%	oxidoreductase, aldo/keto reductase family
	7	34.5%	14	60.1%	8	42.3%	7	37.4%	
**SGO_0554**	19	26.5%	10	15.4%	8	12.2%	4	5.8%	hsdR; type I site-specific deoxyribonuclease
	24	32.5%	13	20.9%	13	21.0%			
**SGO_0556**	6	7.0%							DNA helicase-like protein, putative
	5	8.5%							

Sg Rep I Rep II | *SgFn* Rep I Rep II | *SgPg* Rep I Rep II | *SgPgFn* Rep I Rep II

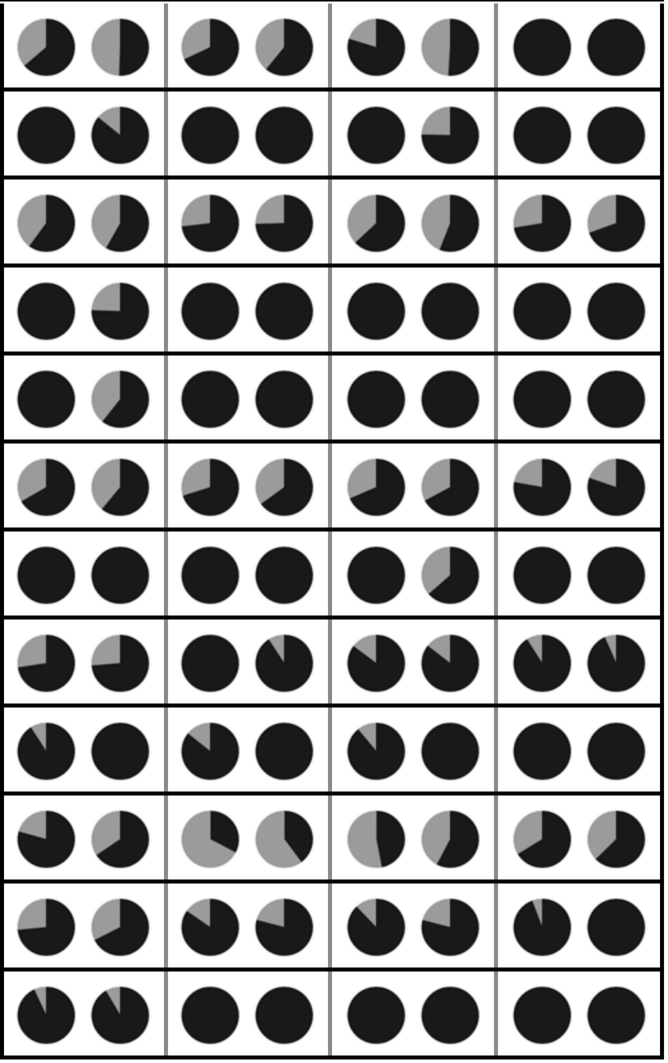

◉ Show detected proteins only
○ Show all proteins
☐ Filter by category:
ABC Transporter

Proteins found: 1179

Dot Plots Dot Plots

Hendrickson *et al.*

Protein	*Sg* n	*Sg* Coverage	*SgFn* n	*SgFn* Coverage	*SgPg* n	*SgPg* Coverage	*SgPgFn* n	*SgPgFn* Coverage	Description
**SGO_0557**									HsdS specificity protein of type I restriction-modification system
	4	13.9%							
**SGO_0558**					4	26.0%			hypothetical protein SGO_0558
	5	32.7%	3	20.5%	3	20.9%	3	18.5%	
**SGO_0560**	6	20.7%			5	18.1%	5	17.8%	hsdM; type I restriction-modification system, M subunit
	7	18.1%	3	12.1%	4	15.7%	4	15.3%	
**SGO_0565**	14	70.0%	19	73.2%	14	60.3%	11	46.8%	adhA; alcohol dehydrogenase
	18	74.7%	18	72.6%	12	60.3%	11	52.4%	
**SGO_0566**	34	28.4%	23	21.9%					sgc; serine protease challisin
	43	32.7%	23	23.1%					
**SGO_0568**	9	41.6%	6	25.6%	11	42.3%	7	28.9%	glyQ; glycyl-tRNA synthetase, alpha subunit
	8	36.1%	5	19.0%	12	51.1%	9	35.1%	
**SGO_0569**	21	35.3%	19	34.8%	27	49.5%	19	33.3%	glyS; glycyl-tRNA synthetase, beta subunit
	23	35.9%	16	30.0%	27	47.4%	22	40.8%	
**SGO_0570**					3	29.4%			hypothetical protein SGO_0570
	4	37.6%			3	29.4%			
**SGO_0571**									hypothetical protein SGO_0571
	3	7.8%							
**SGO_0573**	7	36.4%	5	23.7%	6	32.3%	3	18.4%	mraW; S-adenosyl-methyltransferase MraW
	10	50.6%	5	28.5%	6	33.5%	4	19.0%	
**SGO_0575**	25	47.8%			3	5.6%			pbp2X; penicillin-binding protein 2X
	23	46.7%			3	6.5%			
**SGO_0576**									mraY; phospho-N-acetylmuramoyl-pentapeptide-transferase
	3	11.0%							

Sg Rep I Rep II | *SgFn* Rep I Rep II | *SgPg* Rep I Rep II | *SgPgFn* Rep I Rep II

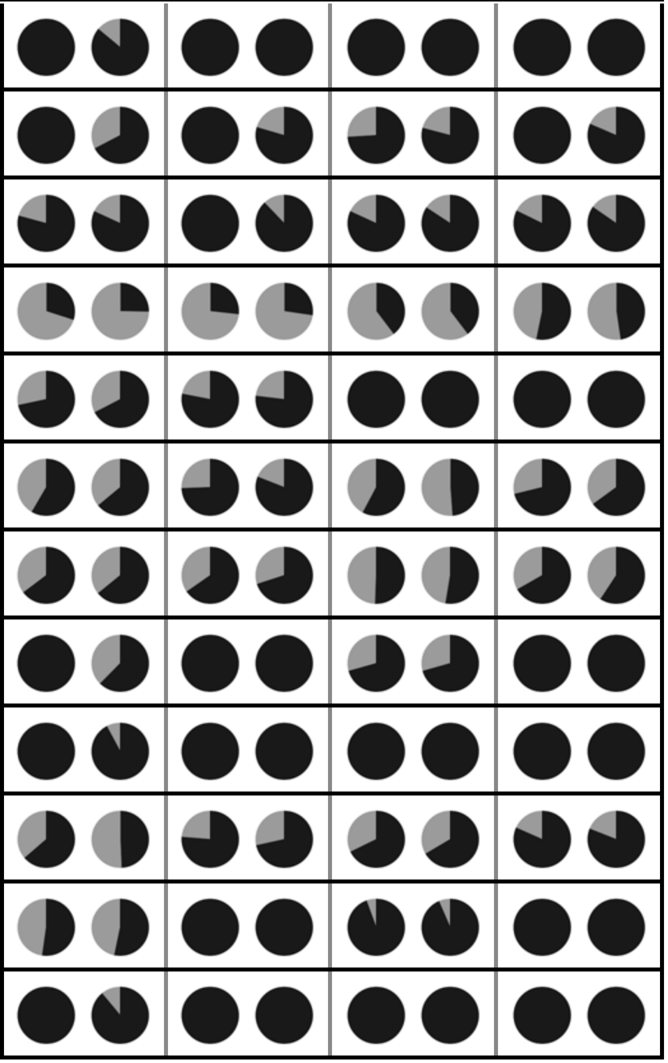

Hendrickson *et al.*

Streptococcus gordonii

Hackett Laboratory UW

Protein	Sg n	Sg Coverage	SgFn n	SgFn Coverage	SgPg n	SgPg Coverage	SgPgFn n	SgPgFn Coverage	Description
SGO_0577	14	42.1%	12	35.6%	16	46.3%	8	26.8%	ATP-dependent RNA helicase
	17	45.2%	10	27.5%	17	52.3%	6	21.3%	
SGO_0578	5	24.5%							amino acid ABC transporter permease protein
	6	29.7%							
SGO_0579	8	54.3%	3	24.7%	9	51.8%			amino acid ABC transporter ATP binding protein
	12	63.2%	3	21.9%	4	28.7%			
SGO_0581	7	25.7%	5	24.3%	6	29.9%	6	25.0%	trxB; thioredoxin-disulfide reductase
	8	29.9%	5	17.8%	9	38.5%	4	16.1%	
SGO_0582	14	28.2%	22	48.8%	17	45.9%	8	23.5%	nicotinate phosphoribosyltransferase, putative
	16	35.2%	18	39.9%	19	47.3%	12	34.4%	
SGO_0583	6	29.9%	7	29.6%	9	42.3%	4	19.3%	nadE; NAD+ synthetase
	5	23.4%	7	32.1%	6	29.2%	5	25.5%	
SGO_0585	6	21.2%	22	53.2%	11	37.6%	6	21.8%	pepC; aminopeptidase C
	13	30.2%	16	44.6%	13	39.6%	5	20.9%	
SGO_0586	26	50.0%	13	20.8%	19	41.6%	9	21.8%	pbp1a; penicillin-binding protein 1A
	26	48.5%	12	23.3%	17	35.0%	12	25.7%	
SGO_0589	6	61.2%	4	52.6%	6	61.2%	5	61.2%	methylase
	6	61.2%	4	45.7%	6	61.2%	6	61.2%	
SGO_0590	7	23.2%	9	22.9%	7	18.8%	4	12.6%	Methyltransferase
	10	25.0%	10	26.0%	7	20.9%			
SGO_0591	24	60.3%	11	39.7%	14	40.8%	7	23.7%	hypothetical protein SGO_0591
	26	60.7%	9	26.9%	15	44.7%	8	19.9%	
SGO_0592	5	30.6%	3	18.1%	7	56.9%	5	33.8%	luxS; autoinducer-2 production protein LuxS
	4	25.6%	4	34.4%	7	55.6%			

Sg: Rep I, Rep II | SgFn: Rep I, Rep II | SgPg: Rep I, Rep II | SgPgFn: Rep I, Rep II

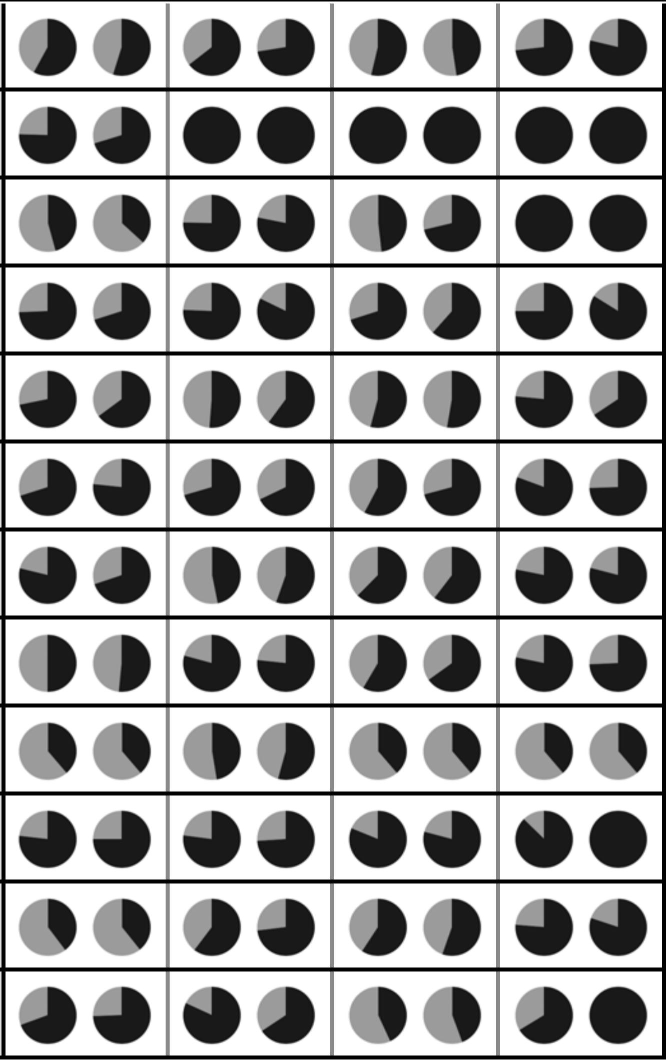

Show detected proteins only
Show all proteins
Filter by category:
ABC Transporter

Proteins found: 1179

Dot Plots Dot Plots

Hendrickson *et al.*

Protein	Sg n	Sg Coverage	SgFn n	SgFn Coverage	SgPg n	SgPg Coverage	SgPgFn n	SgPgFn Coverage	Description
SGO_0593	25	45.2%	8	21.3%	21	43.9%	11	27.5%	HD/KH domain protein
	30	47.3%	10	23.4%	20	39.3%	8	16.3%	
SGO_0594	8	40.4%	8	48.1%	8	48.6%	5	20.2%	gmk; Guanylate kinase (GMP kinase)
	8	40.4%	7	41.3%	8	55.8%	5	30.3%	
SGO_0595	5	43.7%	4	43.7%	6	59.2%	6	58.3%	DNA-directed RNA polymerase, omega subunit
	6	59.2%	4	43.7%	7	59.2%	6	59.2%	
SGO_0596	5	8.8%	6	14.0%					priA; primosomal protein N''
			5	8.7%					
SGO_0597	6	32.5%	10	37.6%	11	46.9%	3	14.8%	fmt; methionyl-tRNA formyltransferase
	6	27.7%	9	39.9%	8	39.5%	3	16.7%	
SGO_0598	7	20.4%			10	30.2%			sun; sun protein
	6	17.8%	6	20.4%	6	17.4%			
SGO_0599	11	48.0%	15	64.6%	9	39.8%	5	20.3%	phosphoprotein phosphatase
	11	43.9%	13	52.4%	7	35.4%	6	30.9%	
SGO_0600	22	48.8%	9	19.8%	12	28.5%	5	13.6%	serine/threonine protein kinase
	24	52.5%	7	16.9%	12	32.4%			
SGO_0602	7	24.0%							histidine kinase
	7	27.4%							
SGO_0603	4	21.5%	7	35.0%	5	25.7%			response regulator
	5	25.7%	6	30.8%	4	20.1%			
SGO_0604	14	46.9%	20	62.1%	18	59.3%	8	19.3%	hydrolase, haloacid dehalogenase family/peptidyl-prolyl cis-trans isomerase, cyclophilin type
	18	54.8%	21	69.0%	20	63.6%	12	35.8%	
SGO_0606	17	70.2%	14	77.7%	15	75.1%	16	82.5%	cysK; cysteine synthase A
	15	69.6%	13	76.7%	16	81.2%	15	81.9%	

Sg: Rep I, Rep II | SgFn: Rep I, Rep II | SgPg: Rep I, Rep II | SgPgFn: Rep I, Rep II

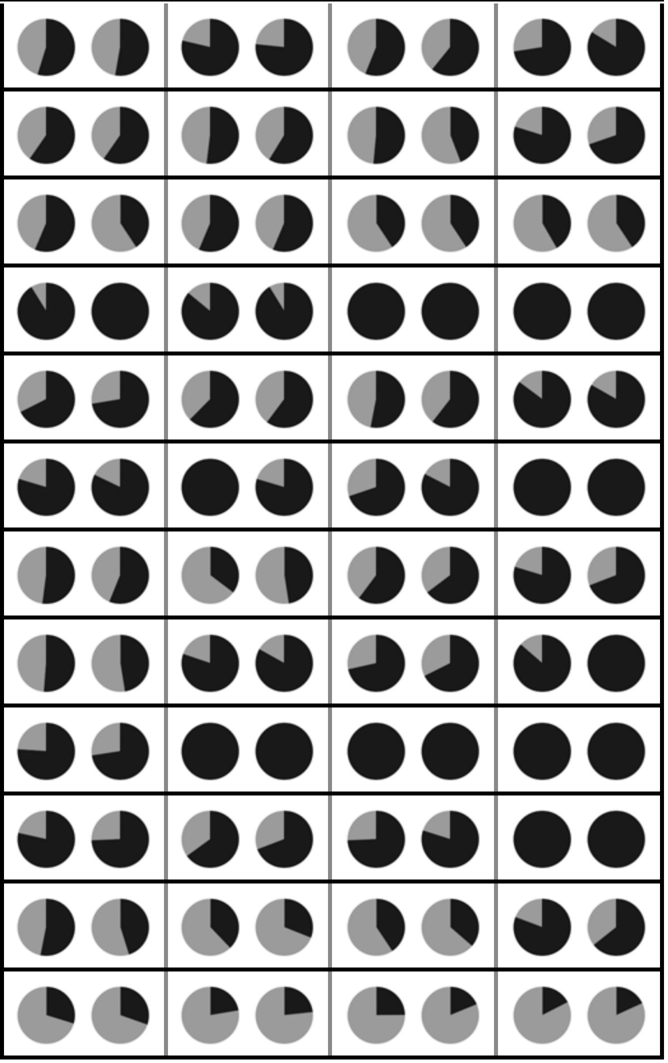

Show detected proteins only
Show all proteins
Filter by category:
ABC Transporter

Proteins found:
1179

Dot Plots Dot Plots

Hendrickson *et al.*

Protein	*Sg* n	*Sg* Coverage	*SgFn* n	*SgFn* Coverage	*SgPg* n	*SgPg* Coverage	*SgPgFn* n	*SgPgFn* Coverage	Description
**SGO_0608**	4	13.4%							comFA; competence ComFA-like protein
	4	14.1%							
**SGO_0610**	8	48.9%	11	55.6%	9	52.8%	8	51.7%	ribosomal subunit interface protein
	9	53.9%	9	54.4%	9	52.8%	6	37.8%	
**SGO_0626**	7	42.2%	3	13.6%	4	18.6%	3	15.5%	recX; Regulatory protein recX
	4	23.6%			5	22.9%			
**SGO_0628**									ymdC; 3''-aminoglycoside phosphotransferase-like protein (kanamycin kinase)
	3	21.1%							
**SGO_0629**	4	15.9%			3	10.6%			rumA-1; 23S rRNA (uracil-5-)-methyltransferase RumA
	6	23.1%							
**SGO_0631**	4	10.0%	4	9.4%	18	36.2%	6	14.6%	alpha-glycerophosphate oxidase
					17	36.7%	5	10.0%	
**SGO_0632**					6	17.9%	5	19.9%	glpK; glycerol kinase
					6	18.9%	4	13.9%	
**SGO_0635**									hypothetical protein SGO_0635
	5	21.4%	4	17.4%					
**SGO_0636**	3	41.4%							hypothetical protein SGO_0636
	3	39.1%							
**SGO_0639**	23	35.6%	16	27.8%	30	46.0%	19	31.3%	valS; valyl-tRNA synthetase
	23	38.5%	19	37.8%	29	44.4%	17	28.9%	
**SGO_0640**	4	17.7%			4	12.6%			modification methylase
	4	16.2%			3	13.4%	4	14.3%	
**SGO_0641**	17	30.0%	17	34.6%	21	40.1%	9	16.9%	ATPase, histidine kinase-, DNA gyrase B-, and HSP90-like domain protein protein
	15	27.8%	13	24.5%	18	37.7%	6	11.0%	

Coverage columns: *Sg* Rep I, Rep II; *SgFn* Rep I, Rep II; *SgPg* Rep I, Rep II; *SgPgFn* Rep I, Rep II

Hendrickson *et al.*

Protein	*Sg* n	*Sg* Coverage	*SgFn* n	*SgFn* Coverage	*SgPg* n	*SgPg* Coverage	*SgPgFn* n	*SgPgFn* Coverage	Description
**SGO_0642**	15	35.5%	5	14.7%	10	23.4%	7	21.2%	hypothetical protein SGO_0642
	19	37.2%	4	11.8%	10	24.4%	6	15.9%	
**SGO_0643**	8	24.6%	3	12.1%	5	15.0%	3	8.9%	cytosine-specific methyltransferase
	7	24.6%	3	9.9%	7	25.9%			
**SGO_0644**	9	28.1%	7	20.5%	16	42.5%	9	30.6%	hypothetical protein SGO_0644
	11	34.3%	8	23.7%	14	39.1%	11	34.0%	
**SGO_0649**									hypothetical protein SGO_0649
	3	19.1%							
**SGO_0652**	16	39.5%	3	11.8%	7	21.6%	4	13.8%	hypothetical protein SGO_0652
	19	48.7%	4	14.7%	4	15.3%	4	11.0%	
**SGO_0653**					4	24.2%			conserved hypothetical protein of unknown function (DUF1027)
	5	28.1%			3	19.1%			
**SGO_0654**	13	48.1%	4	17.7%	10	35.4%	6	21.3%	radical SAM enzyme, Cfr family
	20	53.9%			10	35.4%	5	18.2%	
**SGO_0656**	7	23.9%	8	29.0%	5	20.4%	5	18.6%	trpB-2; tryptophan synthase, beta subunit
	8	28.8%	10	30.5%	6	22.4%	4	17.6%	
**SGO_0657**									trpE; anthranilate synthase component I
	4	10.0%							
**SGO_0662**			5	19.9%					trpB-1; tryptophan synthase, beta subunit
	3	9.8%	3	10.3%					
**SGO_0663**									trpA-2; tryptophan synthase, alpha subunit
	3	13.1%							
**SGO_0665**	9	71.3%	8	66.7%	8	66.7%	6	53.8%	non-heme iron-containing ferritin
	8	66.7%	10	74.3%	8	64.3%	6	53.8%	

Sg Rep I Rep II | *SgFn* Rep I Rep II | *SgPg* Rep I Rep II | *SgPgFn* Rep I Rep II

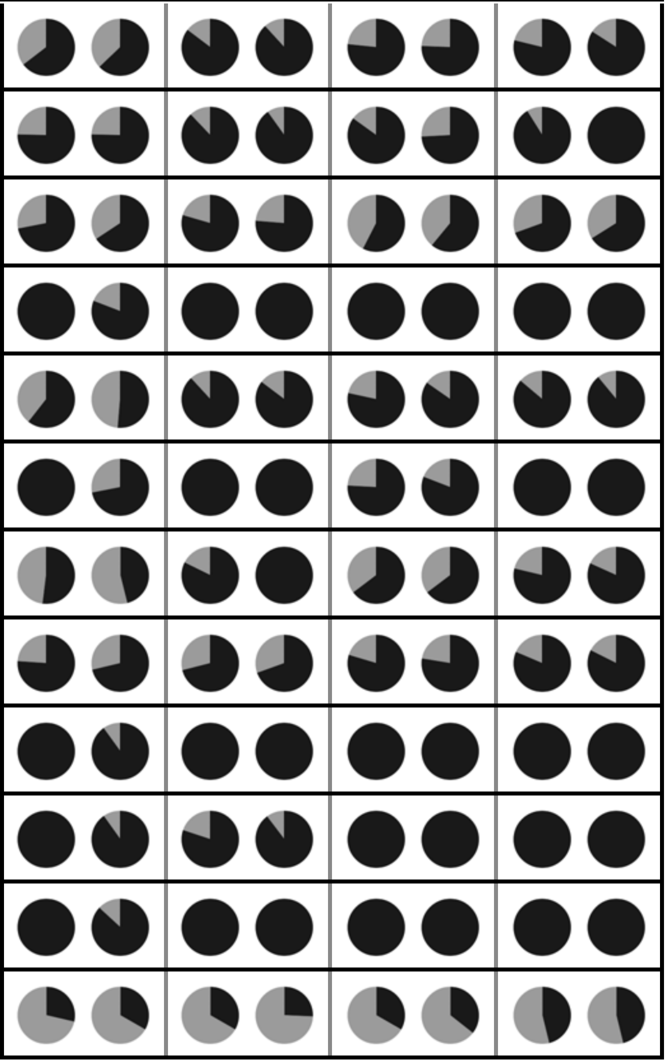

Show detected proteins only
Show all proteins
Filter by category:
ABC Transporter

Proteins found:
1179

Dot Plots Dot Plots

Hendrickson *et al.*

Protein	*Sg* n	*Sg* Coverage	*SgFn* n	*SgFn* Coverage	*SgPg* n	*SgPg* Coverage	*SgPgFn* n	*SgPgFn* Coverage	Description
SGO_0667	4	38.3%			4	38.3%			rhodanese family protein
	5	39.1%			3	29.7%			
SGO_0669	15	33.3%	12	27.6%	18	35.1%	14	30.1%	typA; GTP-binding protein TypA
	16	35.8%	17	38.2%	16	34.8%	12	26.7%	
SGO_0671	11	31.6%	19	58.4%	18	54.0%	3	13.6%	murD; UDP-N-acetylmuramoylalanine--D-glutamate ligase
	15	39.1%	14	46.7%	17	51.3%	5	17.3%	
SGO_0672	18	61.5%	8	32.6%	7	28.4%	3	9.6%	murG; undecaprenyl-PP-MurNAc-pentapeptide-UDPGlcNAc GlcNAc transferase
	16	54.2%	10	37.6%	10	39.3%			
SGO_0673	12	29.2%	5	17.4%	4	15.1%	3	10.1%	DivIB; cell division protein DivIB
	18	32.2%	5	16.4%					
SGO_0674	19	52.4%	19	53.0%	18	54.3%	15	47.9%	ftsA; cell division protein FtsA
	23	59.2%	17	52.1%	19	55.4%	15	46.6%	
SGO_0675	17	49.2%	20	52.3%	17	48.9%	15	43.9%	ftsZ; cell division protein FtsZ
	19	49.4%	18	50.6%	17	50.8%	15	46.1%	
SGO_0676	5	32.7%	5	27.4%	4	24.7%	4	31.4%	conserved hypothetical protein TIGR00044
	7	39.0%	5	32.3%	6	38.1%	3	17.9%	
SGO_0677	8	50.3%	7	55.6%	4	37.0%	3	31.7%	ylmF protein
	9	63.5%	6	53.4%	7	56.6%	3	31.7%	
SGO_0680	13	57.3%	12	56.5%	15	57.3%	13	52.4%	cell division protein DivIVA
	13	57.3%	9	43.9%	15	57.3%	12	54.5%	
SGO_0681	23	32.5%	22	33.9%	30	40.6%	21	33.2%	ileS; isoleucyl-tRNA synthetase
	28	36.1%	21	39.6%	29	41.0%	19	30.8%	
SGO_0684	4	41.4%	5	41.4%	5	41.4%	4	41.4%	hypothetical protein SGO_0684
	5	42.4%	6	42.4%	4	41.4%	4	41.4%	

Show detected proteins only
Show all proteins
Filter by category:
ABC Transporter

Proteins found: 1179

Dot Plots Dot Plots

Protein	*Sg* n	*Sg* Coverage	*SgFn* n	*SgFn* Coverage	*SgPg* n	*SgPg* Coverage	*SgPgFn* n	*SgPgFn* Coverage	Description
**SGO_0685**									MutT/nudix family protein
	3	32.7%	3	34.0%					
**SGO_0688**	27	48.1%	15	24.7%	24	41.7%	14	27.6%	ATP dependent Clp protease, ATP-binding subunit, ClpE
	35	50.2%	16	31.6%	26	48.2%	13	23.4%	
**SGO_0689**									hypothetical protein SGO_0689
	4	31.6%							
**SGO_0690**	3	17.3%	3	17.3%					folD; methenyltetrahydrofolate cyclohydrolase
	4	20.8%	5	25.0%	3	17.3%			
**SGO_0693**	12	33.6%	8	24.7%	3	10.8%			xseA; exodeoxyribonuclease VII, large subunit
	14	33.2%	15	39.2%	3	10.8%	3	8.5%	
**SGO_0694**					3	45.7%			xseB; exodeoxyribonuclease VII, small subunit
	3	61.4%							
**SGO_0695**			3	9.6%					geranyltranstransferase
					3	16.5%			
**SGO_0696**	3	10.3%							hemolysin-like protein
	4	18.1%							
**SGO_0697**	3	34.3%			3	32.9%			transcription regulator
	4	34.3%							
**SGO_0698**	8	24.6%	10	27.0%	6	17.8%			recN; DNA repair protein RecN
	14	38.2%	7	18.3%	12	33.3%			
**SGO_0699**	3	26.4%	5	41.7%	3	31.4%			Serine/threonine protein phosphatase
	3	25.2%	6	35.1%	4	35.1%			
**SGO_0700**	7	39.4%	6	31.4%	8	42.2%	3	11.6%	DegV family protein
	7	33.6%	4	16.2%	9	45.1%	4	18.1%	

Hendrickson *et al.*

Protein	*Sg* n	*Sg* Coverage	*SgFn* n	*SgFn* Coverage	*SgPg* n	*SgPg* Coverage	*SgPgFn* n	*SgPgFn* Coverage	Description
SGO_0701	7	59.3%	8	73.6%	6	49.5%	7	62.6%	hup; DNA-binding histone-like protein HU
	10	73.6%	8	70.3%	7	62.6%	9	64.8%	
SGO_0704	15	70.9%	17	79.1%	21	80.4%	18	81.3%	gpmA; 2,3-bisphosphoglycerate-dependent phosphoglycerate mutase
	20	72.6%	18	83.9%	22	82.6%	18	77.4%	
SGO_0705									Protein of unknown function (DUF1250) superfamily
	3	45.5%							
SGO_0706	3	12.0%	11	47.3%	7	32.8%			phoH-like protein
	6	24.7%	10	42.8%	7	32.8%	5	17.8%	
SGO_0707	36	28.7%	32	25.8%	10	11.6%	6	4.9%	LPXTG cell wall surface protein
	46	33.0%	30	25.5%	4	3.2%	3	2.3%	
SGO_0708	18	65.1%	23	92.7%	19	77.6%	16	71.9%	ald; alanine dehydrogenase
	20	76.8%	28	98.4%	22	88.6%	17	68.9%	
SGO_0711	3	12.7%							conserved hypothetical protein TIGR00043
			4	20.6%					
SGO_0713	14	51.8%	15	61.9%	14	54.8%	7	29.1%	sgg; GTP-binding protein Era
	10	41.5%	16	59.5%	12	45.5%	9	36.8%	
SGO_0715									mutM; formamidopyrimidine-DNA glycosylase
	3	18.2%			3	19.0%			
SGO_0719	14	22.9%	6	11.3%	4	8.6%			rnr; ribonuclease R
	15	25.3%	6	11.4%	5	10.9%			
SGO_0721	7	13.7%	4	8.0%	4	7.5%	6	11.1%	abpB-like dipeptidase lipoprotein
	10	19.8%	4	6.6%	3	6.3%	3	5.4%	
SGO_0722	4	19.2%	5	22.0%	5	19.6%			tehB; tellurite resistance protein TehB
	9	37.1%	5	22.0%	5	19.9%			

Sg Rep I Rep II | *SgFn* Rep I Rep II | *SgPg* Rep I Rep II | *SgPgFn* Rep I Rep II

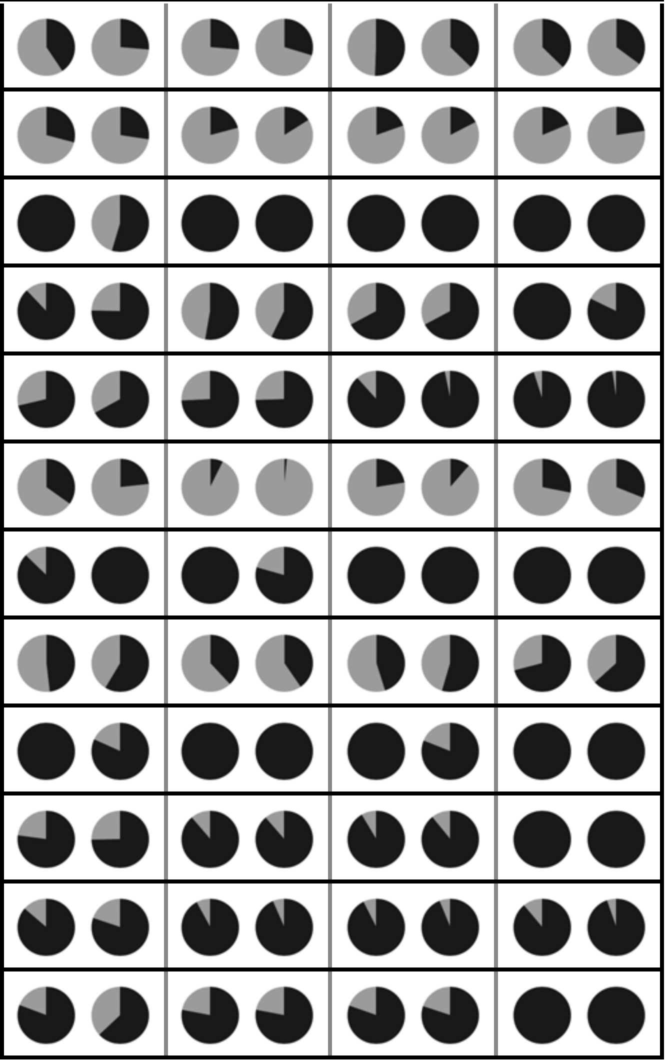

Show detected proteins only
Show all proteins
Filter by category:
ABC Transporter

Proteins found: 1179

Dot Plots Dot Plots

Protein	*Sg* n	*Sg* Coverage	*SgFn* n	*SgFn* Coverage	*SgPg* n	*SgPg* Coverage	*SgPgFn* n	*SgPgFn* Coverage	Description
**SGO_0724**	12	32.5%	6	20.5%	5	16.1%			dipeptidase
	14	36.0%	5	14.4%	6	18.1%			
**SGO_0728**									ABC transporter, ATP-binding protein SP1653
	4	27.0%							
**SGO_0729**	4	5.2%							ABC transporter permease protein
	7	9.7%							
**SGO_0731**	4	41.6%							RRF2 family protein
	4	41.6%							
**SGO_0736**	9	43.1%	10	46.3%	5	22.8%	3	11.9%	hprK; HPr(Ser) kinase/phosphatase
	8	37.9%	9	42.8%	5	21.5%			
**SGO_0737**	3	14.5%							lgt; prolipoprotein diacylglyceryl transferase
	3	14.5%							
**SGO_0738**	5	57.5%			4	51.2%			Bacterial protein of unknown function (DUF948) family
	5	57.5%			3	28.3%			
**SGO_0739**	7	43.5%			8	63.9%	4	43.5%	hypothetical protein SGO_0739
	7	46.9%	4	28.6%	7	63.9%	6	55.8%	
**SGO_0742**	4	17.2%	7	29.8%	4	18.8%	4	16.8%	peptidase, U32 family
	5	20.7%	6	29.4%	5	25.9%			
**SGO_0743**	13	38.1%	9	27.3%	10	30.1%	7	21.5%	peptidase, U32 family
	16	48.8%	6	17.8%	10	28.7%	7	24.5%	
**SGO_0745**	3	44.3%			4	44.3%	4	44.3%	hypothetical protein SGO_0745
	3	44.3%	3	38.6%	4	44.3%	4	44.3%	
**SGO_0749**	5	19.2%	12	48.6%	11	45.9%	7	20.3%	glutathione reductase
	9	29.0%	12	45.4%	9	38.8%	6	21.4%	

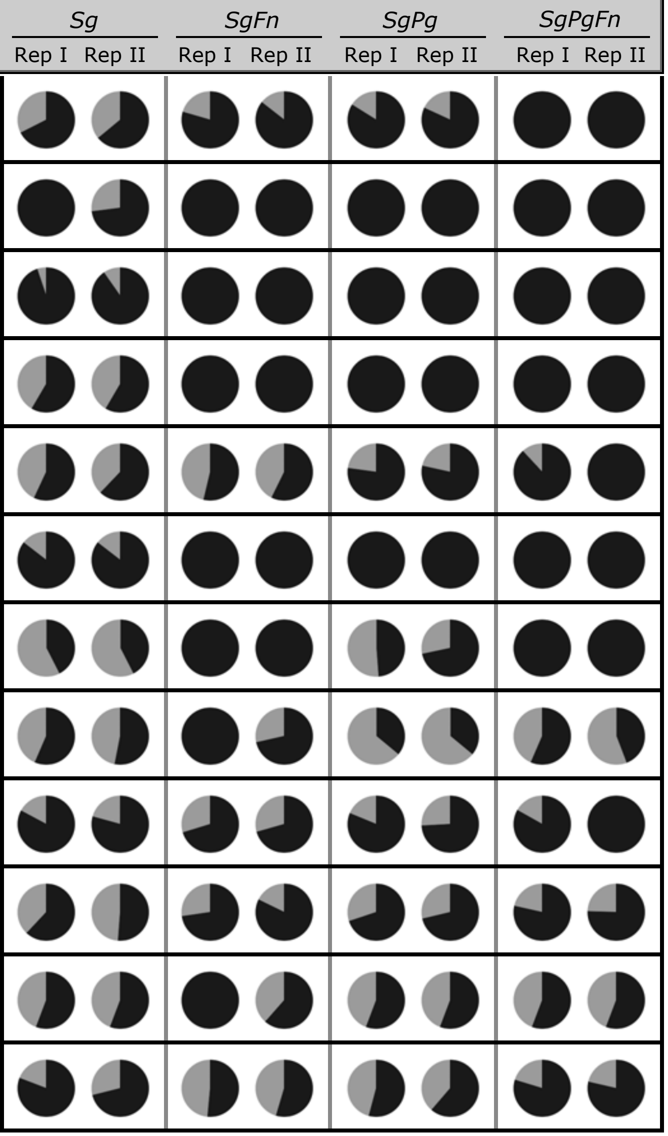

Show detected proteins only
Show all proteins
Filter by category:
ABC Transporter

Proteins found: 1179

Dot Plots Dot Plots

Hendrickson *et al.*

Protein	*Sg* n	*Sg* Coverage	*SgFn* n	*SgFn* Coverage	*SgPg* n	*SgPg* Coverage	*SgPgFn* n	*SgPgFn* Coverage	Description
SGO_0750	21	64.0%	5	16.8%	16	53.8%	12	49.0%	efflux transporter, RND family, MFP subunit subfamily
	20	61.9%	4	18.8%	18	60.9%	12	41.4%	
SGO_0751	9	45.0%	3	20.8%	13	74.5%	7	44.6%	ABC transporter, ATP-binding protein SP0786
	11	56.3%	5	34.2%	11	68.4%	7	40.3%	
SGO_0752	10	28.5%			5	17.5%			ABC transporter, ATP-binding protein
	14	36.6%			6	16.7%	5	13.0%	
SGO_0753	22	45.8%	20	38.1%	24	48.0%	16	32.5%	lysS; lysyl-tRNA synthetase
	26	45.4%	19	40.3%	22	45.8%	16	33.3%	
SGO_0754									phosphoglycerate mutase family protein
			4	42.3%	3	27.7%			
SGO_0755	6	57.0%	4	39.2%	4	43.7%			regulatory protein
	6	55.1%	5	44.9%	6	55.1%	4	41.8%	
SGO_0757	10	49.2%							peptidase, U32 family
	9	52.4%							
SGO_0760	37	44.2%	34	42.7%	40	50.7%	18	24.1%	ppc; phosphoenolpyruvate carboxylase
	41	44.4%	29	40.4%	38	45.9%	18	23.8%	
SGO_0761	31	78.1%	42	82.2%	37	79.9%	36	79.4%	tuf; translation elongation factor Tu
	32	78.1%	52	98.0%	36	79.9%	36	81.2%	
SGO_0762	12	54.0%	13	55.2%	14	55.2%	12	54.0%	tpiA; triosephosphate isomerase
	12	54.0%	13	54.0%	13	55.2%	12	54.0%	
SGO_0763	19	59.9%	11	41.1%	14	45.5%	9	26.3%	murA-1; UDP-N-acetylglucosamine 1-carboxyvinyltransferase
	17	58.2%	12	40.4%	15	54.2%	8	24.4%	
SGO_0765	4	21.9%							endA; DNA-entry nuclease
	8	49.1%							

Columns at right: *Sg* Rep I, Rep II; *SgFn* Rep I, Rep II; *SgPg* Rep I, Rep II; *SgPgFn* Rep I, Rep II

Hendrickson *et al.*

Protein	*Sg* n	*Sg* Coverage	*SgFn* n	*SgFn* Coverage	*SgPg* n	*SgPg* Coverage	*SgPgFn* n	*SgPgFn* Coverage	Description	*Sg* / *SgFn* / *SgPg* / *SgPgFn* Rep I / Rep II
SGO_0767	9	40.5%	11	50.5%	6	33.2%			transport protein	
	10	46.4%	6	33.2%	8	42.2%				
SGO_0771	12	45.0%	16	64.7%	12	58.9%	12	48.1%	pepq; proline dipeptidase	
	13	52.8%	14	60.3%	15	65.3%	10	41.4%		
SGO_0773	25	82.3%	14	65.3%	18	74.6%	13	60.5%	ccpA; catabolite control protein A	
	26	82.6%	14	65.3%	17	74.3%	12	61.7%		
SGO_0774	12	38.0%	5	15.9%	5	20.6%	4	17.1%	glycosyl transferase, group 1 family protein	
	17	47.0%	6	20.9%	7	31.5%				
SGO_0775	13	32.3%	11	30.9%	9	23.8%	4	12.4%	glycosyl transferase, group 1	
	12	29.3%	10	30.2%	8	23.6%	7	19.7%		
SGO_0778	23	46.7%	24	56.0%	25	53.8%	18	33.2%	thrS; threonyl-tRNA synthetase	
	25	43.7%	21	50.4%	28	56.1%	19	39.7%		
SGO_0779	7	45.5%	16	70.0%	13	65.2%	5	30.0%	response regulator	
	12	54.1%	15	65.2%	13	61.4%	3	14.2%		
SGO_0780	14	37.7%	7	19.4%	3	5.3%			histidine kinase	
	12	36.9%	4	14.8%	4	13.2%				
SGO_0781	11	43.8%							vicX; Zn-dependent hydrolase (beta-lactamase superfamily)	
	13	51.3%	3	23.2%						
SGO_0782	3	16.9%							Protein of unknown function (DUF454) family	
	3	16.9%								
SGO_0784	53	48.3%	18	21.9%	24	24.9%	9	10.0%	smc; chromosome segregation protein SMC	
	57	50.8%	18	21.6%	27	30.6%	8	10.0%		
SGO_0785	4	20.4%	3	15.8%	3	20.0%			Cof family protein	
	6	33.6%								

◉ Show detected proteins only
○ Show all proteins
☐ Filter by category:
ABC Transporter

Proteins found:
1179

Dot Plots Dot Plots

Hendrickson *et al.*

Protein	*Sg* n	*Sg* Coverage	*SgFn* n	*SgFn* Coverage	*SgPg* n	*SgPg* Coverage	*SgPgFn* n	*SgPgFn* Coverage	Description
**SGO_0786**	6	39.4%			6	39.4%	5	33.8%	Cof family protein
	5	33.8%	4	25.7%	7	46.1%	5	33.8%	
**SGO_0787**	20	47.1%	14	38.9%	12	31.4%	6	15.3%	ftsY; cell division protein FtsY
	24	54.6%	9	23.8%	14	33.5%	7	17.4%	
**SGO_0788**	7	18.5%	19	47.5%	15	40.7%	7	19.8%	zwf; glucose-6-phosphate 1-dehydrogenase
	12	30.5%	18	47.5%	13	38.5%	9	20.6%	
**SGO_0792**	21	34.6%	7	13.5%	21	35.4%	11	18.5%	hypothetical protein SGO_0792
	20	34.9%	6	12.7%	15	23.5%	9	18.0%	
**SGO_0794**	16	38.9%	19	43.4%	20	35.6%	9	20.4%	metallo-beta-lactamase family protein
	19	41.6%	15	39.2%	19	39.4%	10	25.9%	
**SGO_0795**	4	24.0%	6	31.2%	5	32.3%	4	26.6%	tributyrin esterase
	5	31.2%	9	44.9%	6	41.8%	3	21.7%	
**SGO_0797**			4	12.3%					RocB protein, putative
	6	12.8%							
**SGO_0798**	11	64.8%	8	49.0%	11	63.2%	5	29.6%	ABC transporter, ATP-binding protein SP1381
	10	55.1%	8	48.2%	11	60.3%	6	35.2%	
**SGO_0800**	11	48.2%	4	23.0%	6	29.7%	3	15.7%	polysaccharide deacetylase family protein
	11	48.2%	4	16.3%	5	23.3%			
**SGO_0801**	15	48.4%	16	54.9%	18	60.0%	16	50.5%	hom; homoserine dehydrogenase
	16	53.5%	17	55.4%	17	54.2%	12	42.5%	
**SGO_0802**	10	54.9%	8	43.1%	10	55.6%	5	28.1%	thrB; homoserine kinase
	12	60.1%	13	63.5%	10	60.1%	8	41.0%	
**SGO_0803**	7	20.8%			7	21.8%	3	15.3%	hypothetical protein SGO_0803
	8	23.8%			8	23.8%	4	15.3%	

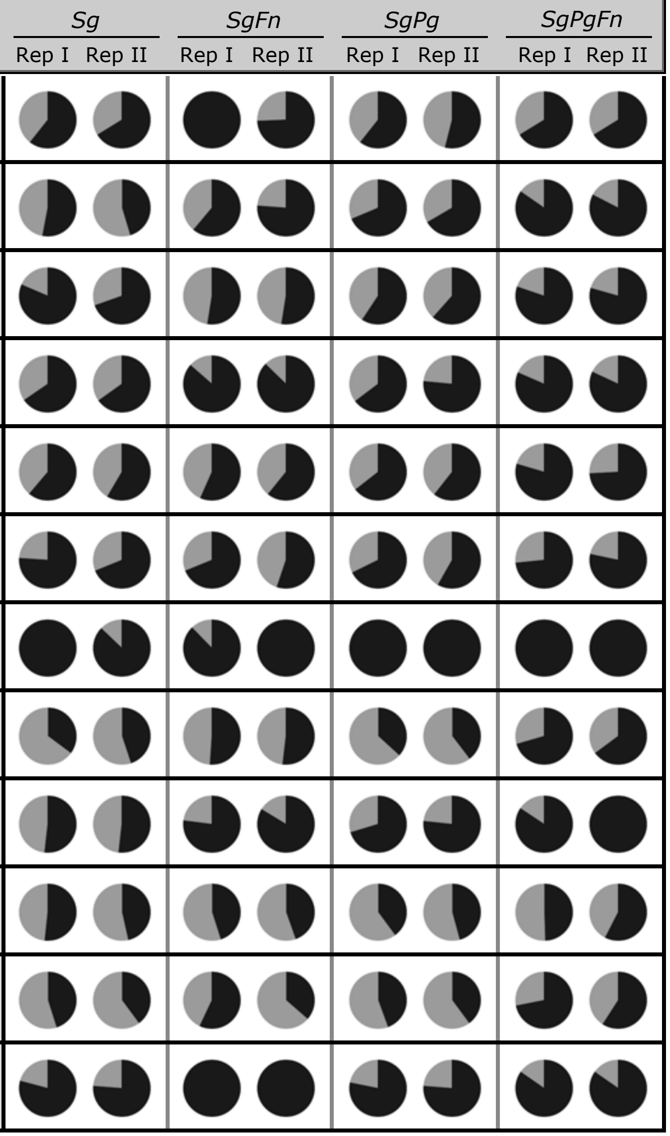

Show detected proteins only
Show all proteins
Filter by category:
ABC Transporter

Proteins found: 1179

Dot Plots Dot Plots

Hendrickson *et al.*

Protein	*Sg* n	*Sg* Coverage	*SgFn* n	*SgFn* Coverage	*SgPg* n	*SgPg* Coverage	*SgPgFn* n	*SgPgFn* Coverage	Description
**SGO_0804**	5	17.2%	4	20.5%	4	11.9%			murB; UDP-N-acetylenolpyruvoylglucosamine reductase
	6	25.2%	4	20.5%	4	22.2%			
**SGO_0805**	12	30.9%	4	21.0%	3	13.8%			potA; spermidine/putrescine ABC transporter, ATP-binding subunit
	12	28.1%	4	21.8%	5	11.7%			
**SGO_0806**	4	15.7%							potB; spermidine/putrescine ABC transporter, permease protein
	4	15.7%							
**SGO_0808**	13	57.0%	3	12.4%					potD; spermidine/putrescine ABC transporter
	16	57.3%			6	29.2%			
**SGO_0812**	7	35.6%	5	31.5%	5	25.6%			hypothetical protein SGO_0812
	8	39.6%	7	38.9%	5	30.0%			
**SGO_0813**			3	3.8%					fructose-bisphosphatase
	3	4.4%	3	7.7%					
**SGO_0814**							3	7.4%	aminotransferase, class-V
	5	27.4%			3	13.9%			
**SGO_0815**	15	51.2%	8	31.4%	14	47.3%	6	22.0%	thiI; thiamine biosynthesis protein ThiI
	18	59.2%	9	33.4%	13	41.6%	7	26.5%	
**SGO_0817**	5	22.3%							hypothetical protein SGO_0817
	3	18.8%							
**SGO_0818**	3	49.0%	3	49.0%	4	57.7%	3	49.0%	rplU; ribosomal protein L21
	4	54.8%			3	49.0%	3	49.0%	
**SGO_0820**	3	37.1%			5	49.5%	3	37.1%	rpmA; ribosomal protein L27
	3	37.1%	3	37.1%	5	49.5%	3	37.1%	
**SGO_0822**	3	30.3%							lipoprotein, putative
	5	36.0%							

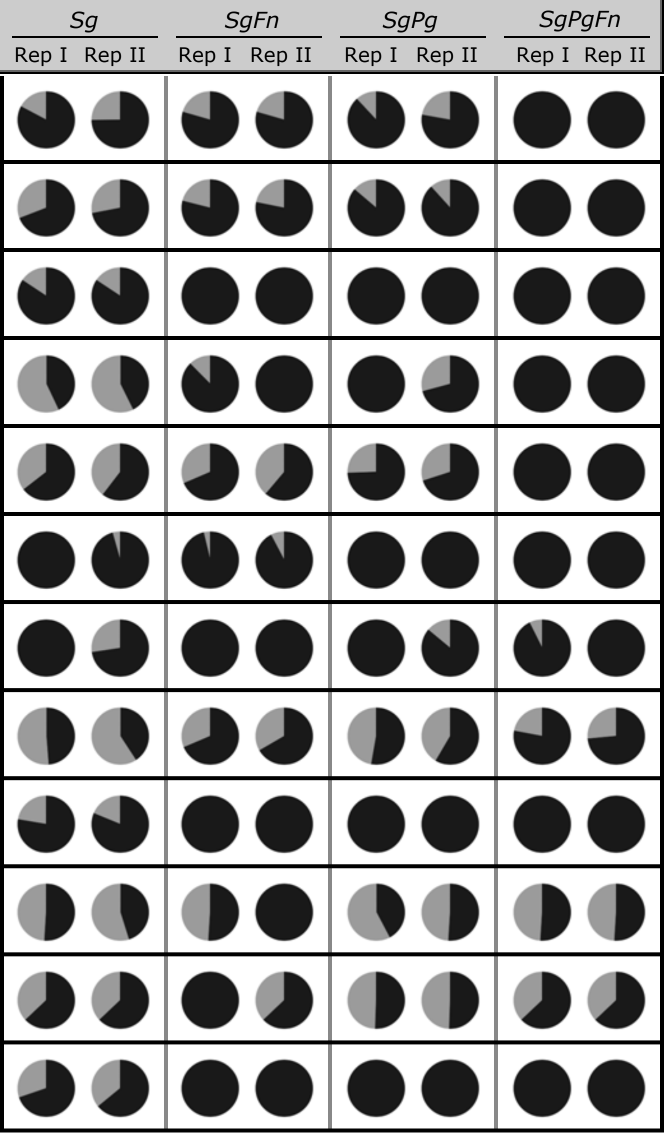

Hendrickson *et al.*

Streptococcus gordonii

Protein	*Sg* n	*Sg* Coverage	*SgFn* n	*SgFn* Coverage	*SgPg* n	*SgPg* Coverage	*SgPgFn* n	*SgPgFn* Coverage	Description
**SGO_0823**			4	21.5%					Cof family protein
	4	23.7%	3	13.7%	4	25.9%			
**SGO_0824**	11	31.3%	10	26.5%	15	38.9%	11	29.8%	lepA; GTP-binding protein LepA
	19	46.5%	13	32.1%	12	34.3%	10	27.8%	
**SGO_0825**	9	54.0%	3	20.4%	4	27.5%			lipoprotein, putative
	7	35.1%			3	20.4%			
**SGO_0830**	3	7.0%			3	8.5%			uvrC; excinuclease ABC, C subunit
	3	7.4%	3	10.8%					
**SGO_0832**	7	73.7%	3	43.0%	3	39.5%			hypothetical protein SGO_0832
	8	78.9%			3	47.4%	3	40.4%	
**SGO_0833**			7	40.2%	3	19.2%			phosphoesterase-related gene
			7	31.3%	5	28.1%			
**SGO_0834**	4	8.1%							hypothetical protein SGO_0834
	5	9.2%	3	7.4%					
**SGO_0835**	8	39.8%	12	62.7%	12	58.7%	10	57.2%	nitroreductase
	11	63.7%	11	62.7%	11	58.7%	10	61.2%	
**SGO_0836**	17	52.2%	21	60.0%	21	65.5%	15	54.4%	pepV; dipeptidase PepV
	16	46.5%	22	69.2%	22	66.0%	13	48.4%	
**SGO_0841**			14	51.8%					oxidoreductase
	4	13.9%	15	56.1%					
**SGO_0842**	4	51.0%	8	62.2%	3	40.8%	3	34.7%	rhodanese family protein
	4	51.0%	5	58.2%	5	58.2%	3	34.7%	
**SGO_0848**	3	59.6%	5	60.6%	5	60.6%	5	60.6%	rpmE; ribosomal protein L31
	3	59.6%	3	59.6%	6	61.7%	4	60.6%	

Sg Rep I Rep II | *SgFn* Rep I Rep II | *SgPg* Rep I Rep II | *SgPgFn* Rep I Rep II

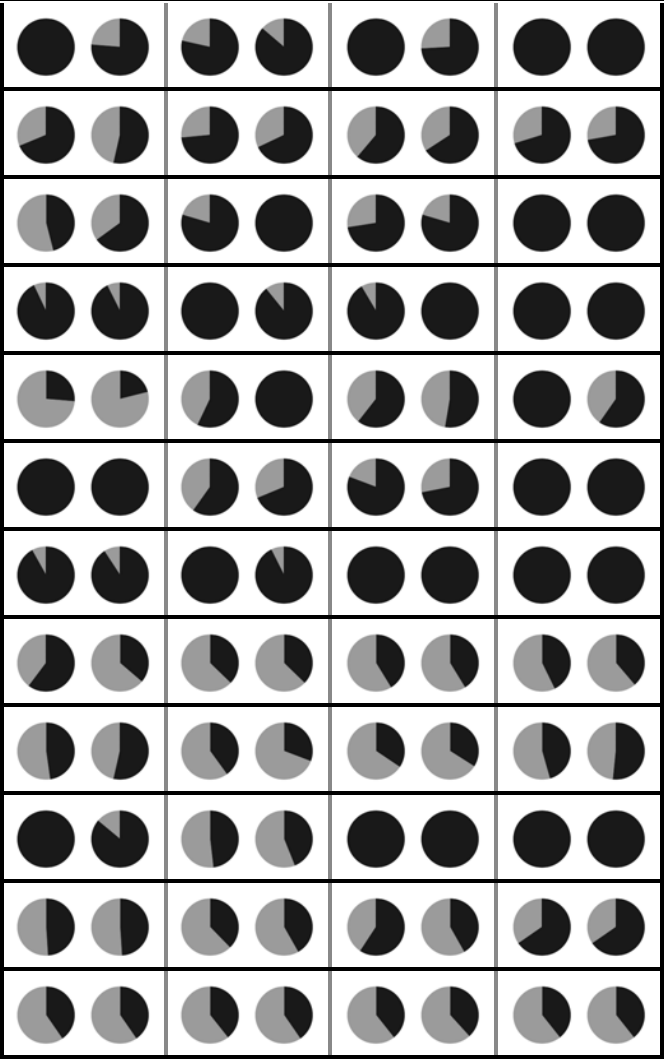

Show detected proteins only
Show all proteins
Filter by category:
ABC Transporter

Proteins found:
1179

Dot Plots Dot Plots

Protein	*Sg* n	*Sg* Coverage	*SgFn* n	*SgFn* Coverage	*SgPg* n	*SgPg* Coverage	*SgPgFn* n	*SgPgFn* Coverage	Description
SGO_0849	9	31.2%	10	41.1%	10	43.3%			DHH subfamily 1 protein
	12	43.0%	7	32.5%	8	34.7%			
SGO_0850	4	51.7%	5	55.8%	4	51.7%	4	51.7%	flavodoxin
	3	38.1%	4	51.7%	4	51.7%	4	51.7%	
SGO_0851	6	18.8%	3	15.5%	3	15.2%			putative permease
	3	11.1%			3	15.7%			
SGO_0852	8	29.9%	4	15.8%	4	14.8%			TPR domain protein
	7	24.2%	5	17.8%	3	12.3%			
SGO_0853	6	37.2%	3	21.8%	3	19.7%			budA; alpha-acetolactate decarboxylase
	4	25.1%	4	24.7%	3	22.2%			
SGO_0854	65	33.6%	23	18.9%	7	5.9%	4	3.1%	cshA; surface-associated protein CshA
	71	35.1%	18	15.7%	10	9.1%	4	2.6%	
SGO_0855	10	24.2%	6	14.0%	9	24.5%			fbpA; fibronectin-binding protein A
	12	28.9%	6	18.2%	8	18.4%			
SGO_0856	14	68.3%	6	32.3%	12	60.8%	6	27.8%	ABC transporter, substrate binding protein
	19	73.1%	5	24.6%	12	56.3%	6	27.2%	
SGO_0858	4	16.7%							ABC transporter, ATP-binding protein
	7	33.7%							
SGO_0859	13	44.4%	10	34.7%	11	39.0%	6	20.6%	pheS; phenylalanyl-tRNA synthetase, alpha subunit
	13	42.1%	11	36.1%	11	39.3%	9	31.2%	
SGO_0861	25	42.9%	15	29.0%	26	50.2%	16	30.3%	pheT; phenylalanyl-tRNA synthetase, beta subunit
	27	40.2%	15	33.6%	26	44.9%	16	29.8%	
SGO_0862	3	14.3%							hypothetical protein SGO_0862
	4	24.5%							

Sg: Rep I, Rep II; SgFn: Rep I, Rep II; SgPg: Rep I, Rep II; SgPgFn: Rep I, Rep II

Hendrickson *et al.*

Protein	*Sg* n	*Sg* Coverage	*SgFn* n	*SgFn* Coverage	*SgPg* n	*SgPg* Coverage	*SgPgFn* n	*SgPgFn* Coverage	Description
SGO_0868	3	59.7%							hypothetical protein SGO_0868
SGO_0877									permease domain protein
	3	13.9%							
SGO_0885	7	44.4%	6	40.6%	4	24.9%	3	20.3%	cobyric acid synthase
	8	47.9%	6	39.1%	4	24.9%	3	17.6%	
SGO_0886	4	13.4%	6	18.1%	4	12.8%			Mur ligase family protein
	8	21.5%	5	13.6%	5	17.7%			
SGO_0887	7	24.6%							conserved hypothetical protein TIGR00159
	10	28.5%							
SGO_0888	6	41.5%			3	25.8%			hypothetical protein SGO_0888
	8	48.0%			3	25.8%	3	25.8%	
SGO_0889	14	42.0%	6	19.3%	14	40.9%	9	24.2%	glmM; phosphoglucosamine mutase
	15	49.1%	9	28.9%	14	47.1%	4	12.9%	
SGO_0890	7	11.0%	9	17.3%					LPXTG cell wall surface protein, collagen binding domain
	9	12.5%	8	15.7%					
SGO_0893	16	53.2%	15	56.7%	15	54.4%	10	39.0%	GTP-binding protein
	18	56.9%	14	53.9%	18	58.3%	11	42.0%	
SGO_0899	4	15.0%							putative hydrolase or acyltransferase
	7	24.6%							
SGO_0901	10	28.1%	10	29.9%	10	26.8%	3	10.3%	DNA-directed DNA polymerase III
	8	21.0%	8	23.4%	11	32.9%	6	18.3%	
SGO_0904	3	10.5%							purR; galactose operon repressor GalR

Show detected proteins only
Show all proteins
Filter by category:
ABC Transporter

Proteins found:
1179

Dot Plots Dot Plots

Protein	*Sg* n	*Sg* Coverage	*SgFn* n	*SgFn* Coverage	*SgPg* n	*SgPg* Coverage	*SgPgFn* n	*SgPgFn* Coverage	Description
**SGO_0905**	5	40.8%							lipoprotein, putative
	6	45.9%							
**SGO_0906**	9	25.7%	15	33.3%	16	36.0%	10	29.3%	leuA; 2-isopropylmalate synthase
	9	24.1%	15	35.1%	14	35.1%	13	38.1%	
**SGO_0907**	3	11.6%							leuB; 3-isopropylmalate dehydrogenase
	3	12.8%			3	11.6%			
**SGO_0909**			4	18.7%					leuC; 3-isopropylmalate dehydratase, large subunit
			3	16.1%	4	10.4%			
**SGO_0911**	6	36.3%	12	65.3%	14	69.1%	9	54.4%	hypothetical protein SGO_0911
	12	57.9%	11	58.7%	10	57.1%	9	48.3%	
**SGO_0915**	6	43.4%			3	21.9%			proV; ABC-type proline/glycine betaine transporter
	8	47.5%			3	25.2%			
**SGO_0916**	12	32.4%							proWX; ABC transporter membrane-spanning permease
	13	36.4%							
**SGO_0932**	6	15.3%							galK; galactokinase
	5	17.1%							
**SGO_0934**			3	15.9%					galE-2; UDP-glucose 4-epimerase
	5	22.2%	3	15.9%					
**SGO_0936**	4	6.4%							phosphoenolpyruvate synthase, putative
	3	4.9%					3	5.5%	
**SGO_0938**							3	15.2%	Phosphatidylserine decarboxylase proenzyme 2
**SGO_0940**	8	16.1%					3	7.6%	ppsA; phosphoenolpyruvate synthase
	5	8.6%							

Sg Rep I Rep II | *SgFn* Rep I Rep II | *SgPg* Rep I Rep II | *SgPgFn* Rep I Rep II

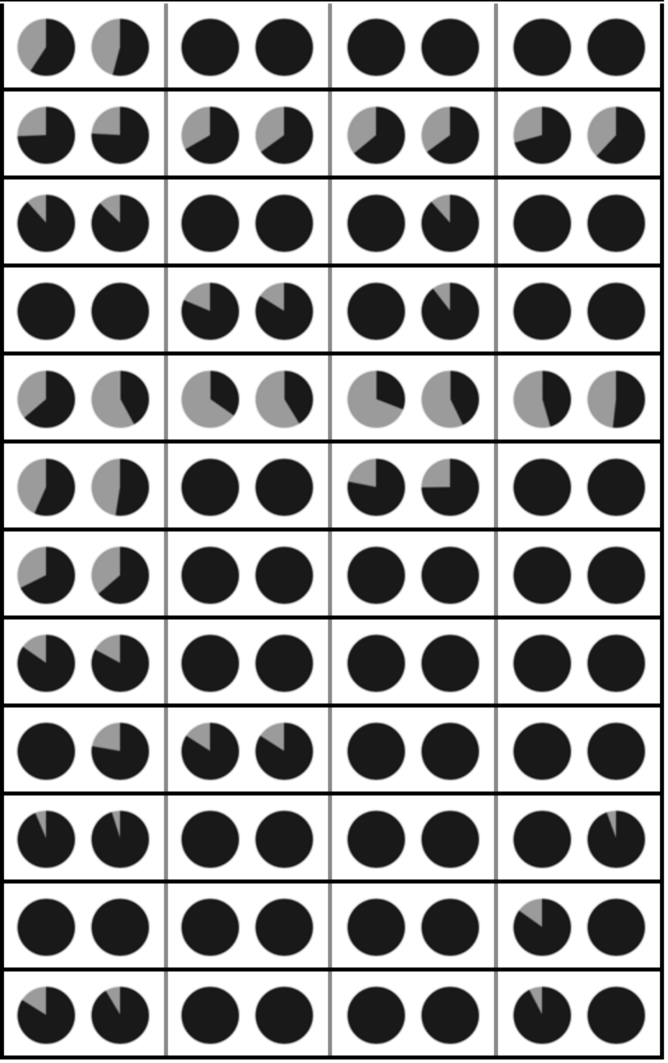

Show detected proteins only
Show all proteins
Filter by category:
ABC Transporter

Proteins found:
1179

Dot Plots Dot Plots

Hendrickson *et al.*

Protein	*Sg* n	*Sg* Coverage	*SgFn* n	*SgFn* Coverage	*SgPg* n	*SgPg* Coverage	*SgPgFn* n	*SgPgFn* Coverage	Description	*Sg* Rep I	*Sg* Rep II	*SgFn* Rep I	*SgFn* Rep II	*SgPg* Rep I	*SgPg* Rep II	*SgPgFn* Rep I	*SgPgFn* Rep II
**SGO_0941**									hypothetical protein SGO_0941								
	4	18.2%															
**SGO_0942**	17	8.7%							zmpC; zinc metalloproteinase C								
	17	10.7%															
**SGO_0944**	3	20.4%							ABC transporter, ATP-binding protein								
	4	27.4%															
**SGO_0946**	6	21.2%	7	25.8%	7	24.6%	4	15.4%	Deblocking aminopeptidase								
	7	26.1%	8	32.5%	4	15.1%	5	18.3%									
**SGO_0948**	9	28.0%	7	16.1%					pgdA; peptidoglycan N-acetylglucosamine deacetylase A								
	8	20.6%	10	29.2%													
**SGO_0949**	8	27.0%	5	24.0%	5	17.0%	3	10.0%	deaD; DEAD RNA helicase								
	8	24.0%	4	15.9%	5	18.6%											
**SGO_0950**			7	31.9%	3	16.9%	3	22.4%	oxidoreductase								
	6	28.8%	5	23.3%	3	16.9%											
**SGO_0951**	7	43.8%	6	44.8%	7	44.3%	4	27.6%	udk; uridine kinase								
	8	48.1%	6	44.8%	7	54.8%	3	22.9%									
**SGO_0953**					3	42.1%			endoribonuclease L-PSP, putative								
					3	42.1%											
**SGO_0954**	8	32.1%	7	26.0%	6	28.7%	3	11.8%	ATP-binding protein								
	7	30.4%	6	26.4%	9	37.5%	4	18.6%									
**SGO_0955**	5	22.8%	5	21.3%					transporter								
	6	26.5%	4	17.3%	6	27.2%											
**SGO_0957**	6	46.6%							hypothetical protein SGO_0957								
	6	46.6%	3	24.5%													

Show detected proteins only
Show all proteins
Filter by category:
ABC Transporter

Proteins found:
1179

Dot Plots Dot Plots

Hendrickson *et al.*

Protein	*Sg* n	*Sg* Coverage	*SgFn* n	*SgFn* Coverage	*SgPg* n	*SgPg* Coverage	*SgPgFn* n	*SgPgFn* Coverage	Description
**SGO_0959**	5	36.2%	6	45.7%	5	36.2%			copper homeostasis protein CutC
	3	24.8%	6	37.6%	5	36.2%			
**SGO_0961**	5	15.4%							ABC transporter, permease/ATP-binding protein
	7	15.9%							
**SGO_0962**	3	7.6%							ABC transporter, permease/ATP-binding protein
	3	8.3%							
**SGO_0966**	9	5.6%	5	4.2%					hsa; streptococcal hemagglutinin
	10	6.3%	5	3.8%					
**SGO_0969**	4	17.1%	3	13.8%					nss; nucleotide sugar synthetase-like protein
	3	17.1%							
**SGO_0971**	4	9.1%							asp1; accessory secretory protein
	4	9.9%							
**SGO_0972**									asp2; accessory sescretory protein
	5	12.0%							
**SGO_0975**	6	16.6%							gtaA; glycosyl transferase, group 1 SP1758
	4	10.3%							
**SGO_0979**	4	15.7%							degV family protein
	4	16.7%							
**SGO_0980**	3	18.0%							transcriptional regulator, TetR family domain protein
**SGO_0981**	6	40.6%			4	31.6%			comE operon protein 2 family
	5	41.9%	3	20.6%					
**SGO_0982**	19	68.0%	4	25.4%	11	47.9%	4	24.3%	amino acid ABC transporter, amino acid-binding protein
	23	70.8%	3	17.6%	10	49.6%	4	25.4%	

Show detected proteins only
Show all proteins
Filter by category:
ABC Transporter

Proteins found: 1179

Dot Plots Dot Plots

Streptococcus gordonii

Protein	*Sg* n	*Sg* Coverage	*SgFn* n	*SgFn* Coverage	*SgPg* n	*SgPg* Coverage	*SgPgFn* n	*SgPgFn* Coverage	Description
SGO_0983	5	36.9%							amino acid ABC transporter, ATP-binding protein SP0709
	5	36.9%			3	21.0%			
SGO_0986	5	26.5%							hypothetical protein SGO_0986
	5	26.5%							
SGO_0987	12	30.6%	16	38.4%	16	40.4%	11	35.9%	metK; S-adenosylmethionine synthetase
	13	34.1%	14	39.4%	10	30.3%	10	28.0%	
SGO_0991	3	53.6%							hypothetical protein SGO_0991
					3	53.6%			
SGO_0993			3	8.5%			4	12.9%	GTP-binding protein HflX
			4	10.2%	5	13.8%			
SGO_0994	3	21.7%			3	24.6%			hypothetical protein SGO_0994
	5	39.1%			3	24.6%			
SGO_0995	7	23.0%	4	22.7%	13	56.0%	4	20.4%	metallo-beta-lactamase superfamily protein
	7	31.4%	6	28.5%	6	27.5%			
SGO_0996	5	25.5%							oxidoreductase, short-chain dehydrogenase/reductase family
	6	38.6%							
SGO_0997	7	34.6%	3	17.5%	4	23.1%			hypothetical protein SGO_0997
	7	34.6%	3	17.5%	3	17.5%			
SGO_1000	4	8.1%	9	19.2%	9	17.9%			recJ; single-stranded-DNA-specific exonuclease RecJ
			11	22.9%	7	14.1%			
SGO_1001	11	67.1%	9	67.6%	11	78.2%	12	72.9%	apt; adenine phosphoribosyltransferase
	11	77.6%	8	68.8%	13	82.9%	10	75.9%	
SGO_1002									metA; homoserine O-succinyltransferase
	4	15.3%							

Hendrickson *et al.*

Protein	*Sg* n	*Sg* Coverage	*SgFn* n	*SgFn* Coverage	*SgPg* n	*SgPg* Coverage	*SgPgFn* n	*SgPgFn* Coverage	Description
SGO_1003	7	38.5%	3	16.4%	5	29.6%			DNA replication protein DnaD
	5	29.6%	4	23.9%	6	32.3%			
SGO_1004			3	14.1%	4	17.2%			glutathione S-transferase family protein
	3	14.1%	5	25.2%	3	13.4%			
SGO_1005	3	21.0%	5	34.5%	5	27.1%	4	27.5%	Bcl-2 family protein
	5	30.1%	5	27.1%	6	39.3%	3	23.1%	
SGO_1006			3	15.8%	4	22.6%			conserved hypothetical protein TIGR00486
	3	14.3%			3	15.8%			
SGO_1007					3	11.5%			oxidoreductase, DadA family protein SP1608
					3	11.5%			
SGO_1009	13	63.3%	23	87.9%	17	77.2%	15	77.5%	rfbA-1; glucose-1-phosphate thymidylyltransferase
	16	65.4%	21	92.0%	19	85.1%	15	70.6%	
SGO_1010	7	42.6%	8	47.2%	7	42.6%	7	42.6%	rmlC; dTDP-4-keto-6-deoxyglucose-3,5-epimerase
	9	47.2%	7	48.2%	7	42.6%	6	38.1%	
SGO_1011	10	38.2%	20	64.4%	16	51.7%	14	47.7%	rfbB-1; dTDP-glucose 4,6-dehydratase
	16	53.2%	17	57.5%	17	51.7%	13	49.1%	
SGO_1012	14	50.1%	17	56.9%	14	47.5%	11	43.4%	galE-1; UDP-glucose 4-epimerase
	12	48.1%	18	62.8%	14	57.8%	14	55.8%	
SGO_1013	6	37.3%					3	17.8%	Glycosyltransferase involved in cell wall biogenesis
	7	41.5%	3	16.9%	3	23.7%			
SGO_1016	8	41.5%	3	12.3%	5	26.9%	3	15.8%	putative glycosyltransferase
	9	46.9%			5	27.3%			
SGO_1018	4	9.1%							hypothetical protein SGO_1018
	3	8.1%							

Sg Rep I Rep II | *SgFn* Rep I Rep II | *SgPg* Rep I Rep II | *SgPgFn* Rep I Rep II

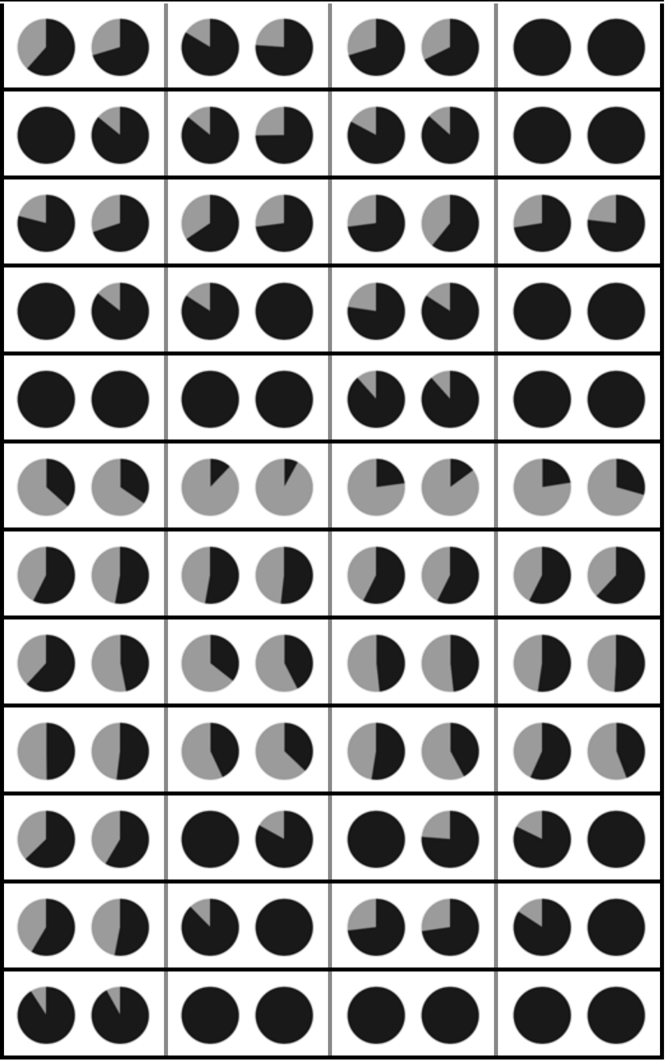

Hendrickson *et al.*

Coverage	*Streptococcus gordonii*	Hackett Laboratory	UW

Summary Table | SgFn vs Sg | SgPg vs Sg | SgPgFn vs Sg | SgPg vs SgFn | SgPgFn vs SgFn | SgPgFn vs SgPg | Coverage

Protein	*Sg*		*SgFn*		*SgPg*		*SgPgFn*		Description
	n	Coverage	n	Coverage	n	Coverage	n	Coverage	
**SGO_1019**	7	29.2%			4	19.2%	3	13.6%	glycosyl transferase
	8	29.2%	4	24.0%	4	19.5%			
**SGO_1020**	13	55.1%	15	62.5%	12	57.2%	8	32.9%	rfbD; dTDP-4-dehydrorhamnose reductase
	14	60.1%	15	67.8%	15	67.1%	7	29.0%	
**SGO_1021**	9	38.2%							rgpA; rhamnosyltransferase, putative
	14	47.6%							
**SGO_1022**	8	35.8%							rhamnosyltransferase
	7	31.3%							
**SGO_1024**	15	37.8%	4	15.5%	9	26.5%			putative polysaccharide ABC transporter
	17	40.3%	5	17.4%	7	17.7%			
**SGO_1025**	12	39.6%	8	30.7%	9	28.1%	9	24.7%	rgp; glycosyltransferase
	15	45.4%	5	22.7%	10	31.6%	6	19.8%	
**SGO_1026**	22	34.7%	9	16.0%	8	19.2%	3	5.8%	rhamnosyltransferase
	29	40.4%	10	17.7%	12	26.5%	3	5.8%	
**SGO_1027**	11	20.2%							hypothetical protein SGO_1027
	13	23.5%							
**SGO_1028**	3	28.4%							hypothetical protein SGO_1028
	4	28.4%							
**SGO_1030**									hypothetical protein SGO_1030
	3	28.9%							
**SGO_1031**	6	39.1%	6	43.1%	7	48.0%	3	23.1%	cmk; cytidylate kinase
	7	45.3%	5	34.2%	7	51.1%	3	23.6%	
**SGO_1032**	4	29.0%			4	30.7%	4	24.4%	infC; translation initiation factor IF-3
	4	29.0%			4	30.7%	3	20.5%	

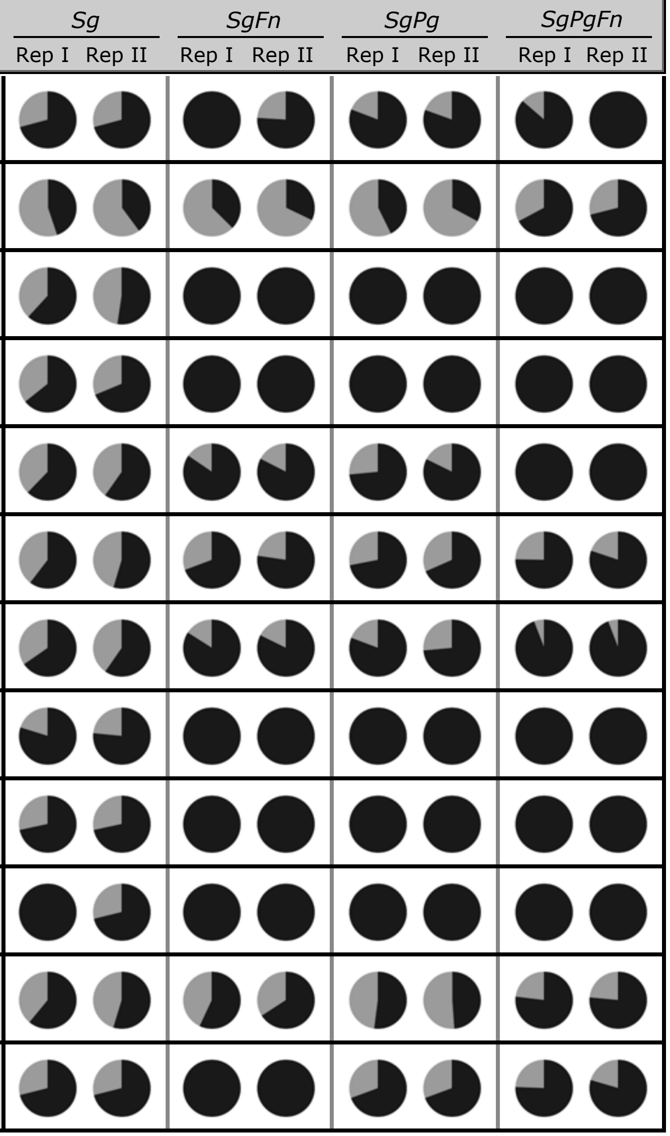

- (selected) Show detected proteins only
- Show all proteins

☐ Filter by category:

ABC Transporter

Proteins found: 1179

Dot Plots | Dot Plots

Hendrickson *et al.*

Protein	*Sg* n	*Sg* Coverage	*SgFn* n	*SgFn* Coverage	*SgPg* n	*SgPg* Coverage	*SgPgFn* n	*SgPgFn* Coverage	Description
SGO_1033	3	18.2%	5	33.3%			3	18.2%	rpmI; ribosomal protein L35
	5	30.3%	4	18.2%			4	18.2%	
SGO_1034	7	52.1%	6	54.6%	7	52.1%	5	47.1%	rplT; ribosomal protein L20
	7	52.1%	8	73.9%	7	52.1%	5	51.3%	
SGO_1035	4	34.1%	5	36.5%	3	28.6%	3	28.6%	gloA; lactoylglutathione lyase
	4	34.1%	5	38.1%	3	28.6%	3	28.6%	
SGO_1036	10	54.9%	13	69.1%	7	45.5%	3	17.5%	amino acid ABC transporter, ATP-binding protein SP1242
	9	52.0%	6	35.8%	10	64.6%	3	20.3%	
SGO_1037	9	26.6%	3	8.0%	5	9.5%			glutamine ABC transporter permease and substrate binding protein
	13	37.1%	4	14.2%	8	21.6%			
SGO_1038	6	10.6%	9	19.2%	7	14.8%	6	10.1%	uvrB; excinuclease ABC, B subunit
	8	15.6%	9	19.9%	8	16.0%	7	13.1%	
SGO_1039	4	18.2%							hypothetical protein SGO_1039
	3	10.5%							
SGO_1041			3	32.0%	3	27.1%			hypothetical protein SGO_1041
	3	24.3%			4	40.3%			
SGO_1044					3	26.5%			MutT/nudix family protein
	3	22.6%	3	23.9%	4	32.9%			
SGO_1047	10	29.2%			8	22.2%	5	14.6%	hypothetical protein SGO_1047
	14	35.4%	3	9.7%	9	27.1%	3	9.4%	
SGO_1049	3	18.8%			4	21.6%	3	20.5%	tRNA pseudouridine synthase B
	6	29.8%			4	22.6%			
SGO_1050					4	15.4%			ribF; riboflavin biosynthesis protein RibF
	3	9.8%	3	10.5%	3	12.1%	4	16.4%	

Sg Rep I Rep II | *SgFn* Rep I Rep II | *SgPg* Rep I Rep II | *SgPgFn* Rep I Rep II

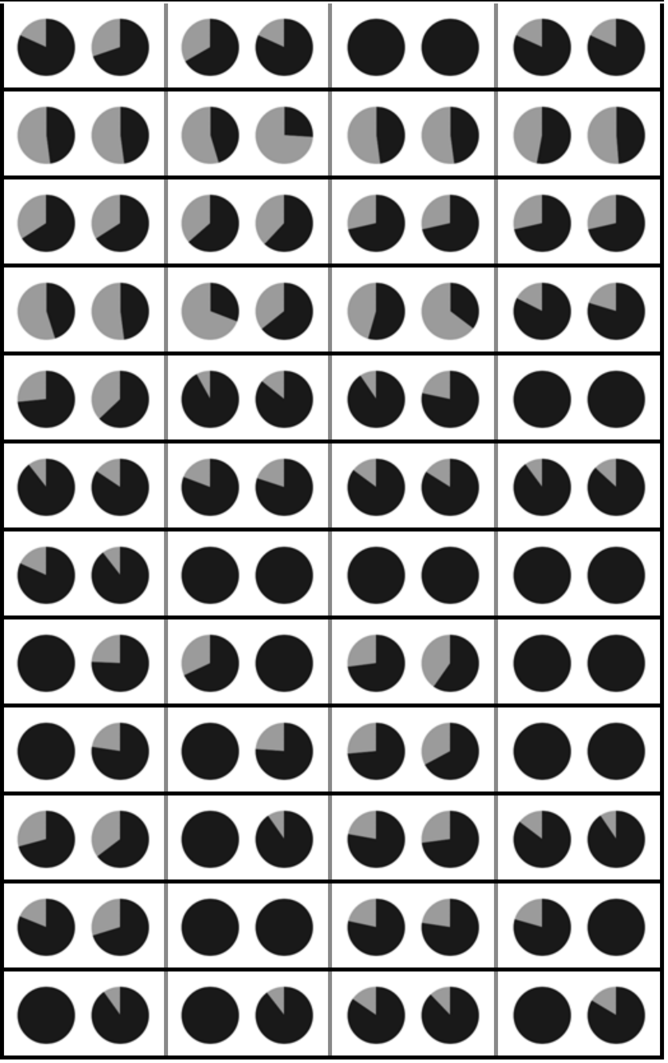

Show detected proteins only
Show all proteins
Filter by category:
ABC Transporter

Proteins found:
1179

Dot Plots Dot Plots

Protein	*Sg* n	*Sg* Coverage	*SgFn* n	*SgFn* Coverage	*SgPg* n	*SgPg* Coverage	*SgPgFn* n	*SgPgFn* Coverage	Description
**SGO_1051**					5	43.6%	3	21.8%	negative regulator of proteolysis
**SGO_1054**	4	13.8%			3	14.5%			NOL1/NOP2/sun family protein
	5	18.2%							
**SGO_1055**	12	67.2%			4	21.2%			phosphate ABC transporter, phosphate-binding protein
	13	71.3%			5	25.9%			
**SGO_1057**									pstA; phosphate ABC transporter, permease protein PstA
	4	10.2%							
**SGO_1058**	12	40.8%			5	17.6%	4	13.1%	pstB; Phosphate import ATP-binding protein pstB 2 (Phosphate-transporting ATPase 2) (ABC phosphate transporter 2)
	14	46.1%			4	15.4%			
**SGO_1059**	9	46.4%	3	17.9%	7	48.4%	7	44.8%	pstB; Phosphate import ATP-binding protein pstB 1 (Phosphate-transporting ATPase 1) (ABC phosphate transporter 1)
	9	46.4%	3	17.9%	8	48.8%	6	38.5%	
**SGO_1060**	11	55.8%	7	39.2%	9	54.4%	6	37.8%	phosphate transport system regulatory protein
	11	55.8%	7	41.0%	12	57.6%	6	37.8%	
**SGO_1065**	8	41.7%	7	42.8%	4	32.1%	4	32.1%	hypothetical protein SGO_1065
	8	43.3%	4	32.1%	4	32.1%	3	27.8%	
**SGO_1066**	12	37.3%	8	23.9%	5	12.0%			hypothetical protein SGO_1066
	14	37.0%	10	25.0%	4	11.1%			
**SGO_1067**			3	64.3%					hypothetical protein SGO_1067
	3	57.1%							
**SGO_1069**	32	45.6%	27	44.3%	36	53.5%	21	37.5%	membrane alanyl aminopeptidase
	27	40.0%	35	54.9%	31	51.0%	20	35.1%	
**SGO_1072**			5	31.3%	4	21.4%			ciaR; Transcriptional regulatory protein CiaR
	3	14.7%	7	34.4%					

Show detected proteins only
Show all proteins
Filter by category:
ABC Transporter

Proteins found:
1179

Dot Plots Dot Plots

Hendrickson *et al.*

Protein	*Sg* n	*Sg* Coverage	*SgFn* n	*SgFn* Coverage	*SgPg* n	*SgPg* Coverage	*SgPgFn* n	*SgPgFn* Coverage	Description
SGO_1073	6	17.6%			4	9.9%			Sensor protein CiaH
	6	17.6%	5	15.2%					
SGO_1075			7	22.8%					alpha-amylase precursor
	4	12.2%	5	15.3%	3	11.2%			
SGO_1077	5	19.6%	3	13.7%	4	14.4%			coaA; pantothenate kinase
	6	25.2%	5	18.6%	5	14.4%			
SGO_1078			3	35.0%	3	35.0%			methyltransferase domain protein
			4	42.6%	3	35.0%			
SGO_1079	10	31.3%	9	29.2%	12	39.8%	5	20.0%	pdp; pyrimidine-nucleoside phosphorylase
	11	28.9%	11	36.5%	10	35.5%	7	25.6%	
SGO_1080	11	68.6%	12	70.9%	12	70.9%	13	72.3%	deoC; deoxyribose-phosphate aldolase
	12	70.9%	10	61.4%	13	72.3%	12	70.9%	
SGO_1081	3	38.8%			4	58.1%	3	44.2%	cdd; cytidine deaminase
	5	55.0%			3	44.2%			
SGO_1082	22	66.5%	22	65.9%	19	65.9%	20	65.9%	lipoprotein
	31	75.0%	20	62.2%	20	63.4%	18	63.4%	
SGO_1083			19	53.8%	28	61.1%	19	43.4%	sugar ABC transporter, ATP-binding protein SP0846
			20	49.1%	24	58.1%	18	47.6%	
SGO_1088	3	17.9%			7	28.5%	3	23.2%	transcription regulator, LysR family
	5	24.8%			4	20.9%			
SGO_1090	7	31.1%			9	33.1%			pseudouridine synthase, RluA family
	8	33.1%	4	20.9%	8	33.4%			
SGO_1091	3	20.5%							hypothetical protein SGO_1091
	4	29.0%							

Sg Rep I Rep II | *SgFn* Rep I Rep II | *SgPg* Rep I Rep II | *SgPgFn* Rep I Rep II

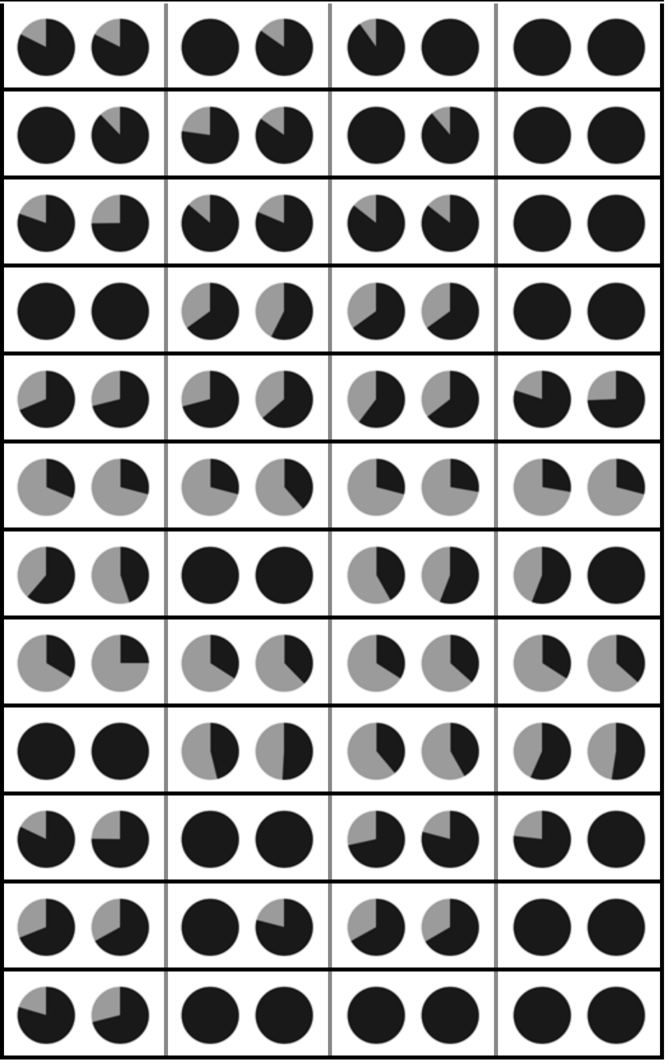

Show detected proteins only
Show all proteins
Filter by category:
ABC Transporter

Proteins found: 1179

Dot Plots Dot Plots

Protein	*Sg* n	*Sg* Coverage	*SgFn* n	*SgFn* Coverage	*SgPg* n	*SgPg* Coverage	*SgPgFn* n	*SgPgFn* Coverage	Description
**SGO_1096**	10	53.1%	15	64.6%	12	61.4%	12	61.4%	butA; acetoin dehydrogenase
	10	58.3%	20	82.7%	12	61.4%	11	61.4%	
**SGO_1097**			3	9.8%					proB; glutamate 5-kinase
	3	8.7%							
**SGO_1098**			8	31.4%	6	22.9%	3	13.8%	proA; gamma-glutamyl phosphate reductase
	4	11.9%	6	21.4%	7	26.4%	3	14.3%	
**SGO_1099**			4	18.7%					proC; pyrroline-5-carboxylate reductase
	3	19.0%	5	23.9%					
**SGO_1100**									phnA; alkylphosphonate utilization operon protein PhnA
	4	34.8%							
**SGO_1103**	3	13.4%							carA; carbamoyl-phosphate synthase, small subunit
	3	14.8%							
**SGO_1104**	14	20.8%					4	4.2%	carB; carbamoyl-phosphate synthase, large subunit
	9	11.2%	4	5.9%					
**SGO_1107**	7	49.1%							PyrR bifunctional protein
	7	49.1%	3	28.3%	4	33.5%	3	26.6%	
**SGO_1109**	14	50.3%	9	38.6%	14	48.0%	10	45.1%	pyrB; aspartate carbamoyltransferase
	15	52.9%	10	38.9%	16	57.8%	8	37.3%	
**SGO_1110**	15	26.4%							surface antigen SCP-like domain
	21	35.4%	4	7.9%					
**SGO_1111**	13	59.1%	4	21.5%	7	40.1%	4	21.9%	fruR; phosphotransferase system repressor
	14	60.3%	3	18.2%	4	22.7%			
**SGO_1112**	11	51.8%	5	32.7%	8	37.3%			fruB; 1-phosphofructokinase
	14	60.4%	4	22.4%	11	59.4%	3	14.9%	

Sg Rep I Rep II | *SgFn* Rep I Rep II | *SgPg* Rep I Rep II | *SgPgFn* Rep I Rep II

Hendrickson *et al.*

Protein	*Sg* n	*Sg* Coverage	*SgFn* n	*SgFn* Coverage	*SgPg* n	*SgPg* Coverage	*SgPgFn* n	*SgPgFn* Coverage	Description	*Sg* Rep I	*Sg* Rep II	*SgFn* Rep I	*SgFn* Rep II	*SgPg* Rep I	*SgPg* Rep II	*SgPgFn* Rep I	*SgPgFn* Rep II
**SGO_1113**	21	34.8%	9	16.8%	13	27.6%	8	18.7%	fruA; PTS system, fructose specific IIABC components								
	26	41.3%	5	10.7%	14	27.4%	11	24.2%									
**SGO_1114**	7	42.7%	5	42.7%	7	51.6%	4	31.5%	Protein of unknown function (DUF1149) superfamily								
	6	36.3%	3	29.8%	5	31.5%	3	25.0%									
**SGO_1115**					3	13.4%			DegV family protein								
	3	12.0%	3	13.4%	3	12.0%											
**SGO_1116**	6	43.1%	7	39.6%	7	43.5%	6	43.1%	dapB; dihydrodipicolinate reductase								
	6	43.5%	6	37.6%	7	43.5%	6	43.1%									
**SGO_1117**			7	30.8%	4	9.8%			pcnA; polynucleotide adenylyltransferase								
			4	17.3%	4	15.8%											
**SGO_1118**			5	12.4%	4	8.9%			ABC transporter, ATP-binding protein SP1553								
	6	12.7%	8	14.8%	5	9.7%											
**SGO_1119**	4	9.4%							Abi-alpha protein, putative								
	3	5.8%															
**SGO_1120**	21	50.4%	20	49.0%	21	54.4%	15	46.3%	guaA; GMP synthase								
	25	57.3%	25	64.3%	24	54.1%	17	45.8%									
**SGO_1121**									transcription regulator, GntR family								
	5	16.8%			3	22.4%											
**SGO_1123**	11	28.4%	16	47.1%	20	54.4%	13	41.2%	ffh; signal recognition particle protein								
	14	38.7%	14	43.3%	21	54.4%	14	44.7%									
**SGO_1124**	3	18.0%							cell surface hydrolase								
	4	22.5%															
**SGO_1128**									bacteriocin-associated integral membrane protein subfamily								
	6	10.3%															

Streptococcus gordonii

Protein	*Sg* n	*Sg* Coverage	*SgFn* n	*SgFn* Coverage	*SgPg* n	*SgPg* Coverage	*SgPgFn* n	*SgPgFn* Coverage	Description
SGO_1129	9	33.1%	6	14.9%	8	35.3%	7	26.4%	lplA; lipoate protein ligase A
	11	35.6%	4	14.9%	8	33.7%	6	26.4%	
SGO_1130	21	39.7%	4	8.1%	14	28.6%	13	30.0%	dihydrolipoamide dehydrogenase
	20	42.0%	5	14.8%	17	35.4%	11	24.2%	
SGO_1131	10	34.9%	4	18.4%	8	32.3%	4	19.3%	sucB; dihydrolipoamide S-acetyltransferase
	16	40.3%			9	35.4%	5	23.9%	
SGO_1132	5	22.4%			6	26.1%	3	17.6%	acetoin dehydrogenase
	7	28.5%			5	26.1%			
SGO_1133	7	30.1%			6	26.7%	6	29.2%	acoA; acetoin dehydrogenase
	8	33.2%	5	15.8%	7	30.7%	4	20.5%	
SGO_1134	6	16.5%	7	20.9%	4	14.4%			hypothetical protein SGO_1134
	6	22.9%	12	37.6%			4	12.4%	
SGO_1139	9	58.9%	10	55.8%	13	73.1%	7	49.2%	GTP-binding protein
	12	64.5%	8	49.7%	14	78.7%	8	43.7%	
SGO_1140	15	43.8%	15	46.9%	20	67.5%	12	35.9%	clpX; ATP-dependent Clp protease, ATP-binding subunit ClpX
	20	49.1%	16	46.0%	20	67.2%	14	45.7%	
SGO_1141	5	40.6%	4	36.5%	4	36.5%	3	23.5%	folA; dihydrofolate reductase
	4	25.9%	7	53.5%	4	36.5%			
SGO_1143	3	16.5%	3	17.2%	4	21.1%	3	16.5%	thyA; thymidylate synthase
	4	21.1%	4	23.7%	4	19.7%			
SGO_1144	14	45.5%	19	79.6%	19	72.1%	13	58.6%	glcK; glucokinase
	19	63.9%	19	77.1%	18	69.0%	13	64.3%	
SGO_1145	4	21.7%							cell surface hydrolase
	4	17.8%							

Show detected proteins only
Show all proteins
Filter by category:
ABC Transporter
Proteins found: 1179
Dot Plots Dot Plots

Hendrickson *et al.*

Protein	*Sg* n	*Sg* Coverage	*SgFn* n	*SgFn* Coverage	*SgPg* n	*SgPg* Coverage	*SgPgFn* n	*SgPgFn* Coverage	Description	*Sg* Rep I	*Sg* Rep II	*SgFn* Rep I	*SgFn* Rep II	*SgPg* Rep I	*SgPg* Rep II	*SgPgFn* Rep I	*SgPgFn* Rep II
**SGO_1148**	46	28.8%	11	9.0%					cshB; surface-associated protein CshB								
	48	29.5%	11	8.8%													
**SGO_1149**	5	36.0%							Pneumococcal vaccine antigen A-like protein								
	6	42.0%															
**SGO_1150**			8	32.1%	8	37.0%			hypothetical protein SGO_1150								
	9	37.3%	5	22.8%	9	38.0%	3	12.7%									
**SGO_1151**	15	45.0%	25	68.6%	19	55.0%	16	49.8%	glyA; serine hydroxymethyltransferase								
	16	46.0%	22	67.4%	22	58.3%	14	44.0%									
**SGO_1154**	10	36.2%	10	46.2%	11	37.6%	11	38.2%	prfA; peptide chain release factor 1								
	12	44.3%	8	33.1%	13	46.5%	10	38.4%									
**SGO_1155**	7	41.9%	4	29.8%	5	24.1%			tdk; thymidine kinase								
	8	46.1%	4	28.8%	5	26.2%	4	16.2%									
**SGO_1159**			3	12.2%	4	21.1%			guaC; guanosine monophosphate reductase								
	3	18.3%	3	12.2%	5	30.9%											
**SGO_1163**							3	7.8%	Iron permease FTR1 family								
**SGO_1166**	3	9.1%							voltage-gated chloride channel family protein								
	3	9.9%															
**SGO_1167**	13	31.7%	8	27.9%	17	48.9%	15	42.4%	nox; NADH oxidase								
	18	39.7%	7	21.6%	16	43.2%	14	39.7%									
**SGO_1169**	12	53.7%	4	29.9%	12	53.2%	6	35.3%	NADPH-dependent FMN reductase								
	11	48.8%	4	29.9%	11	46.8%	7	40.8%									
**SGO_1170**	13	42.8%	6	21.5%	15	45.4%	8	30.4%	NADPH-dependent FMN reductase								
	15	43.5%	8	31.4%	13	42.8%	10	35.5%									

Show detected proteins only
Show all proteins
Filter by category:
ABC Transporter

Proteins found: 1179

Dot Plots Dot Plots

Streptococcus gordonii

Hackett Laboratory UW

Protein	*Sg* n	*Sg* Coverage	*SgFn* n	*SgFn* Coverage	*SgPg* n	*SgPg* Coverage	*SgPgFn* n	*SgPgFn* Coverage	Description
SGO_1171	12	76.9%	4	36.3%	5	46.3%			thioredoxin family protein
	14	76.9%	4	39.4%	4	35.0%			
SGO_1174	3	7.1%							histidine kinase
	6	12.0%							
SGO_1176									peptide methionine sulfoxide reductase-like protein
	3	11.0%							
SGO_1182	3	8.9%							LPXTG cell wall surface protein
SGO_1184	4	23.2%							GdmH
	4	19.4%							
SGO_1185	4	21.7%	3	26.1%	5	27.7%	3	15.8%	acetyltransferase, GNAT family
	4	21.7%			4	21.7%	3	16.3%	
SGO_1186	12	30.5%							ABC transporter, permease/ATP-binding protein
	14	34.6%							
SGO_1187	13	27.2%							ABC transporter, permease/ATP-binding protein
	16	34.4%							
SGO_1189	8	45.8%	6	50.3%	4	28.8%	3	22.2%	lipoprotein, putative
	12	60.8%	3	20.3%	5	30.1%	3	22.2%	
SGO_1191	12	93.4%	13	82.8%	15	89.3%	13	86.9%	rplL; ribosomal protein L7/L12
	11	82.8%	10	82.8%	13	89.3%	12	82.8%	
SGO_1192	9	53.6%	5	47.0%	12	75.9%	10	68.7%	BL5; 50S ribosomal protein L10
	11	64.5%	8	60.8%	13	72.9%	10	63.3%	
SGO_1193	9	31.1%	12	51.4%	12	48.2%	4	16.9%	gid; Glucose inhibited division protein A
	10	38.1%	14	62.4%	14	62.2%	4	15.8%	

Sg Rep I Rep II | *SgFn* Rep I Rep II | *SgPg* Rep I Rep II | *SgPgFn* Rep I Rep II

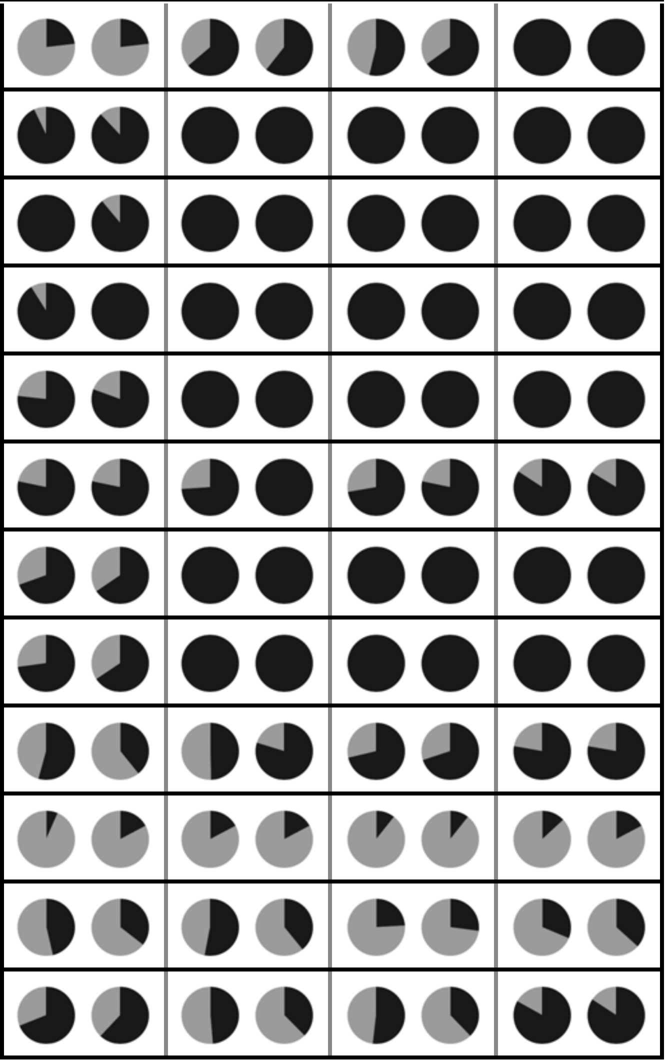

Show detected proteins only
Show all proteins
Filter by category:
ABC Transporter

Proteins found:
1179

Dot Plots Dot Plots

Streptococcus gordonii

Sg: Rep I, Rep II | SgFn: Rep I, Rep II | SgPg: Rep I, Rep II | SgPgFn: Rep I, Rep II

Protein	*Sg* n	*Sg* Coverage	*SgFn* n	*SgFn* Coverage	*SgPg* n	*SgPg* Coverage	*SgPgFn* n	*SgPgFn* Coverage	Description
**SGO_1196**	3	23.3%							satD; SatD-like protein
							3	17.2%	
**SGO_1197**	29	49.9%	9	21.2%	14	30.0%	10	21.1%	topA; DNA topoisomerase I
	33	52.2%	9	23.4%	18	35.7%	10	19.5%	
**SGO_1198**	14	50.4%			13	51.8%	12	46.1%	DNA processing Smf protein
	16	57.5%			10	44.3%	10	45.7%	
**SGO_1199**	3	26.0%			3	26.0%			acetyltransferase, CysE/LacA/LpxA/NodL family
	5	34.4%							
**SGO_1202**	6	26.9%	7	33.9%	8	37.1%			GTP-binding protein
	9	34.6%	5	19.8%	10	44.5%			
**SGO_1203**	4	36.6%	7	77.7%	6	63.4%	4	51.8%	anaerobic ribonucleotide reductase
	6	67.0%	9	79.5%	8	78.6%	4	51.8%	
**SGO_1205**	4	17.0%	7	24.4%	6	24.8%	4	15.8%	dapA; dihydrodipicolinate synthase
	5	20.3%	3	13.8%	6	26.7%	4	19.6%	
**SGO_1206**	9	41.3%	9	36.6%	11	48.6%	8	36.9%	asd; aspartate-semialdehyde dehydrogenase
	12	49.4%	10	46.4%	10	41.3%	7	29.3%	
**SGO_1208**	3	25.8%							Hypothetical FAD-dependent oxidoreductase, putative
	5	36.8%							
**SGO_1210**	6	13.3%	11	25.7%	6	13.3%	3	9.2%	fhs-1; formate--tetrahydrofolate ligase
	6	14.4%	9	25.4%	4	8.8%			
**SGO_1213**	7	55.2%	5	49.7%	4	37.7%			coaC; phosphopantothenoylcysteine decarboxylase
	9	62.8%	3	25.7%					
**SGO_1215**	30	71.9%	28	74.3%	31	78.0%	26	69.8%	manB; phosphomannomutase
	32	75.0%	32	78.3%	35	79.5%	26	66.1%	

Show detected proteins only
Show all proteins
Filter by category:
ABC Transporter

Proteins found:
1179

Dot Plots Dot Plots

Protein	*Sg* n	*Sg* Coverage	*SgFn* n	*SgFn* Coverage	*SgPg* n	*SgPg* Coverage	*SgPgFn* n	*SgPgFn* Coverage	Description
SGO_1216	6	62.6%	7	73.9%	7	60.9%	7	68.7%	bta; Possible bacteriocin transport accessory protein
	5	53.0%	5	53.0%	8	78.3%	7	60.9%	
SGO_1219	9	42.5%	11	52.9%	10	45.5%	8	33.8%	pta; phosphate acetyltransferase
	11	45.8%	11	50.8%	11	52.9%	7	30.8%	
SGO_1221	12	57.3%	3	14.6%					ppnK; Probable inorganic polyphosphate/ATP-NAD kinase
	17	70.4%	9	42.3%					
SGO_1222	4	21.3%							GTP pyrophosphokinase-like protein
	6	33.5%	3	18.6%	4	26.2%			
SGO_1224	17	58.4%	13	51.6%	16	54.0%	11	39.8%	Ribose-phosphate pyrophosphokinase 2
	17	59.6%	10	41.3%	14	52.2%	14	51.6%	
SGO_1225	6	24.3%	6	27.2%	7	34.1%	4	20.0%	pyridoxal-phosphate dependent aminotransferase
	6	24.5%	5	21.1%	8	36.8%	7	33.9%	
SGO_1226	6	50.4%	5	45.2%	5	45.2%	4	45.2%	hypothetical protein SGO_1226
	5	45.2%	4	40.9%	5	45.2%	4	40.9%	
SGO_1228	6	34.4%	5	21.4%	6	27.9%			DNA-binding protein BH0551
	7	36.3%	5	30.7%	6	35.3%			
SGO_1230	9	46.6%			4	23.9%			srtA; sortase A
	9	34.7%			3	22.7%			
SGO_1231	28	40.0%	20	37.0%	17	26.9%	11	14.0%	gyrA; DNA gyrase, A subunit
	34	44.3%	12	19.6%	23	34.0%	10	14.9%	
SGO_1232	12	51.5%	18	59.8%	20	58.5%	14	55.2%	L-lactate dehydrogenase
	12	49.7%	15	51.5%	18	57.6%	15	55.2%	
SGO_1234	23	56.6%	31	69.3%	28	67.3%	26	63.6%	rpsA; 30S ribosomal protein S1
	28	57.9%	24	60.3%	26	58.1%	27	62.6%	

Show detected proteins only
Show all proteins
Filter by category:
ABC Transporter

Proteins found: 1179

Dot Plots Dot Plots

Protein	*Sg* n	*Sg* Coverage	*SgFn* n	*SgFn* Coverage	*SgPg* n	*SgPg* Coverage	*SgPgFn* n	*SgPgFn* Coverage	Description
SGO_1237	5	82.9%			6	82.9%	4	44.7%	hypothetical protein SGO_1237
	6	82.9%			6	82.9%	5	82.9%	
SGO_1238	7	30.3%	11	47.4%	9	37.6%	7	30.0%	ilvE; branched-chain amino acid aminotransferase
	8	35.3%	10	40.9%	11	47.1%	8	32.9%	
SGO_1239	18	26.6%	3	6.4%	11	19.7%	3	4.5%	parC; DNA topoisomerase IV, A subunit
	19	29.4%	7	12.7%	9	14.3%	3	5.6%	
SGO_1241	4	18.4%							aminoglycoside adenylyltransferase
	3	15.9%							
SGO_1242	6	42.2%					3	36.2%	lipoprotein, putative
	8	49.1%			3	36.2%			
SGO_1244	9	22.3%			5	18.9%			hypothetical protein SGO_1244
	5	15.5%			5	17.0%			
SGO_1245	12	22.7%			6	12.3%			parE; DNA topoisomerase IV, B subunit
	12	23.1%			6	12.8%			
SGO_1246	3	13.6%							hypothetical protein SGO_1246
	3	13.6%							
SGO_1247	36	55.2%	25	40.7%					5'-nucleotidase family protein
	41	61.8%	21	49.6%					
SGO_1248	5	13.5%	9	32.2%	7	16.6%	4	12.6%	pyrC; dihydroorotase
	7	18.0%	8	24.6%	7	18.2%	4	13.7%	
SGO_1250	5	38.7%	3	21.7%					ung; uracil-DNA glycosylase
	5	35.9%	4	33.2%					
SGO_1251	3	16.4%							hypothetical protein SGO_1251
	3	16.4%							

Show detected proteins only
Show all proteins
Filter by category:
ABC Transporter

Proteins found: 1179

Dot Plots Dot Plots

Hendrickson *et al.*

Protein	*Sg* n	*Sg* Coverage	*SgFn* n	*SgFn* Coverage	*SgPg* n	*SgPg* Coverage	*SgPgFn* n	*SgPgFn* Coverage	Description
**SGO_1252**									integral membrane protein, putative
	3	7.0%							
**SGO_1253**	5	42.1%	8	55.0%	9	72.2%	4	38.8%	pyrE; orotate phosphoribosyltransferase
	7	43.5%	5	43.5%	7	62.2%	4	37.8%	
**SGO_1254**	6	42.2%							pyrF; orotidine 5'-phosphate decarboxylase
	7	49.1%			4	33.9%			
**SGO_1255**	4	31.1%							pyrd; dihydroorotate dehydrogenase B
	4	26.6%			4	30.8%			
**SGO_1257**	4	15.2%	8	31.1%	6	27.8%			probable transcriptional regulator (LysR family)
	7	22.5%	6	28.8%	8	30.1%			
**SGO_1258**	3	16.5%	4	22.2%					NAD-dependent deacetylase (Regulatory protein SIR2-like protein)
	5	25.5%	4	27.2%					
**SGO_1260**	10	53.2%	8	52.3%	10	61.6%	7	40.1%	deoD; purine nucleoside phosphorylase
	10	51.9%	9	58.2%	9	57.8%	7	40.1%	
**SGO_1261**									glutamine amidotransferase, class I
	5	27.5%							
**SGO_1263**	9	45.7%	6	40.1%	6	40.5%	7	43.5%	purine nucleoside phosphorylase I, inosine and guanosine-specific
	7	43.5%	8	49.8%	6	40.5%	10	48.7%	
**SGO_1264**	22	65.0%	22	64.5%	23	66.7%	21	65.0%	deoB; phosphopentomutase
	22	65.3%	18	66.7%	23	66.7%	22	66.5%	
**SGO_1265**	9	59.1%	6	30.2%	6	41.8%	5	28.0%	rpiA; ribose 5-phosphate isomerase
	9	59.1%	4	23.6%	9	59.1%	5	28.0%	
**SGO_1266**	12	33.0%	9	31.9%	8	19.7%	6	12.0%	trmE; tRNA modification GTPase TrmE
	10	26.7%	9	23.6%	11	27.1%	6	10.3%	

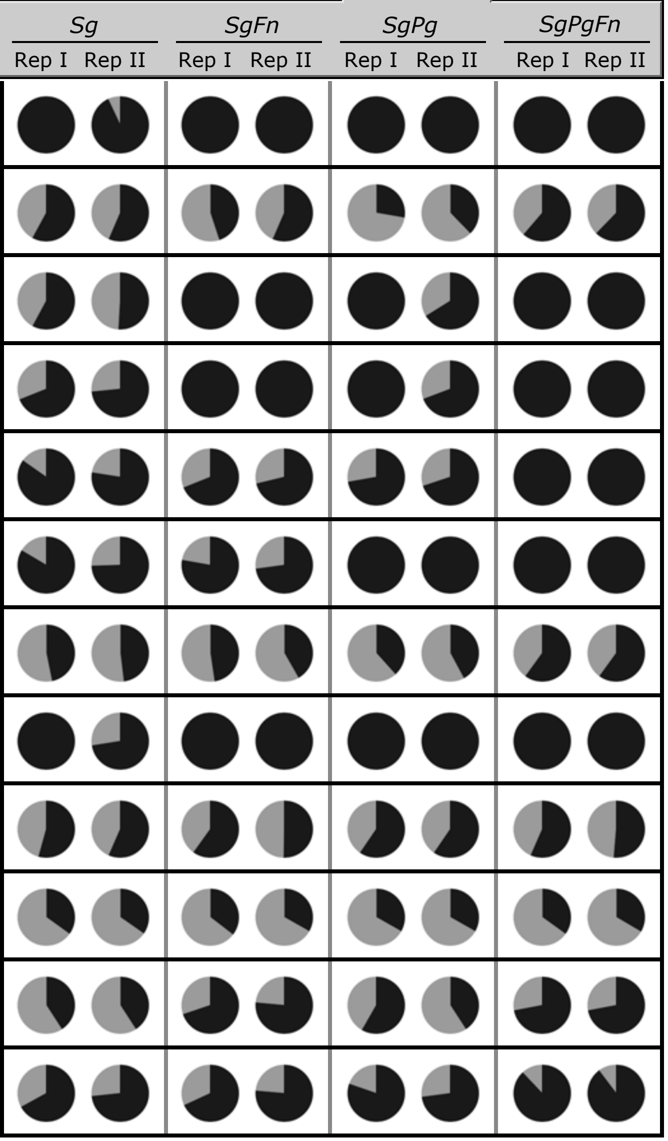

Show detected proteins only
Show all proteins
Filter by category:
ABC Transporter

Proteins found: 1179

Dot Plots Dot Plots

Protein	*Sg* n	*Sg* Coverage	*SgFn* n	*SgFn* Coverage	*SgPg* n	*SgPg* Coverage	*SgPgFn* n	*SgPgFn* Coverage	Description
SGO_1267	6	48.5%			3	28.1%			thioredoxin family protein
	7	54.5%							
SGO_1273	15	51.1%	13	47.6%	17	56.8%	17	56.5%	rpoD; RNA polymerase sigma factor
	15	51.9%	8	34.9%	17	57.6%	13	49.2%	
SGO_1274	4	8.6%			5	12.6%			dnaG; DNA primase
	7	12.6%			7	17.2%			
SGO_1276	4	36.2%	3	34.5%	5	36.2%	4	36.2%	rpsU; ribosomal protein S21
	5	37.9%	4	37.9%	5	37.9%	4	36.2%	
SGO_1280	4	20.4%							putative membrane-associated Zn-dependent protease
	4	17.9%							
SGO_1281	4	44.8%			5	59.2%			penicillinase repressor, putative
	4	44.8%	3	36.8%	5	59.2%	3	36.8%	
SGO_1283	13	45.2%	8	27.4%	18	59.6%	11	39.5%	oxidoreductase
	14	44.1%	8	32.2%	16	52.1%	10	35.8%	
SGO_1284	7	33.2%	6	33.9%	9	45.0%	4	17.4%	thioredoxin-disulfide reductase
	9	45.3%	10	43.5%	7	34.8%	6	28.9%	
SGO_1290	6	17.1%							ABC transporter, permease/ATP-binding protein
	7	21.6%							
SGO_1291	6	13.8%							ABC transporter, permease/ATP-binding protein SP1358
	3	5.0%							
SGO_1293	13	31.4%	11	30.3%	16	37.8%	8	20.4%	asnS; asparaginyl-tRNA synthetase
	15	38.1%	13	36.1%	14	34.4%	9	25.6%	
SGO_1297	9	28.8%	10	42.3%	9	44.6%	8	30.9%	aspC; aspartate aminotransferase
	8	27.0%	10	42.3%	10	42.1%	8	30.4%	

Sg Rep I Rep II | *SgFn* Rep I Rep II | *SgPg* Rep I Rep II | *SgPgFn* Rep I Rep II

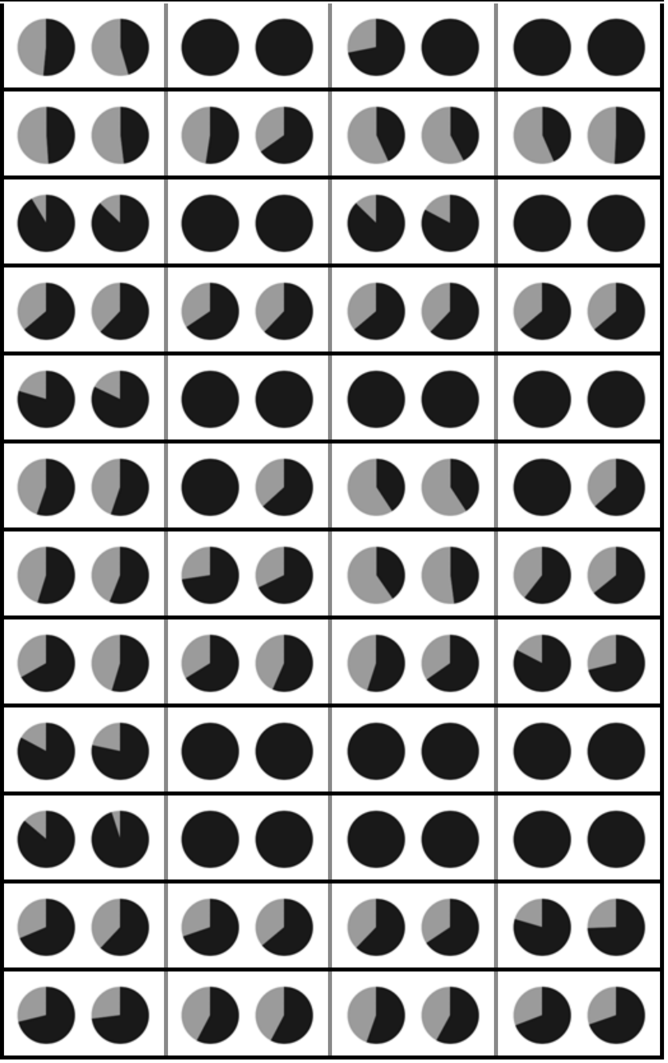

Show detected proteins only
Show all proteins
Filter by category:
ABC Transporter

Proteins found: 1179

Dot Plots Dot Plots

Hendrickson *et al.*

Streptococcus gordonii

Protein	*Sg* n	*Sg* Coverage	*SgFn* n	*SgFn* Coverage	*SgPg* n	*SgPg* Coverage	*SgPgFn* n	*SgPgFn* Coverage	Description
SGO_1298	5	28.4%							hypothetical protein SGO_1298
	4	25.9%							
SGO_1305	15	42.7%			12	38.2%	6	22.7%	substrate-binding protein MsmE
	18	45.2%			12	35.0%	4	14.5%	
SGO_1312	6	19.1%	14	49.5%	17	60.5%	13	46.6%	pepT; peptidase T
	8	26.2%	15	56.6%	16	56.6%	12	40.4%	
SGO_1314									metal binding lipoprotein
	5	21.9%							
SGO_1315			5	36.6%	3	20.2%			trmD; tRNA (guanine-N1)-methyltransferase
	4	26.7%			4	23.5%			
SGO_1316	6	37.2%	3	26.2%	5	34.9%			16S rRNA processing protein RimM
	6	38.4%	3	18.6%	4	33.1%			
SGO_1323	3	43.3%	4	50.0%	3	43.3%	3	43.3%	rpsP; ribosomal protein S16
	3	43.3%	3	43.3%	3	43.3%	3	43.3%	
SGO_1324	5	30.6%							hypothetical protein SGO_1324
	6	30.6%							
SGO_1327	7	35.5%	6	24.2%	8	38.7%	3	15.6%	HAD-superfamily subfamily IIA hydrolase, TIGR01457
	10	43.4%	4	18.0%	8	38.7%	5	21.1%	
SGO_1328									hypothetical protein SGO_1328
	3	11.8%							
SGO_1330	3	9.1%							transporter
	7	16.5%							
SGO_1331									HlyB/MsbA family ABC transporter
	5	13.1%							

Sg Rep I Rep II | *SgFn* Rep I Rep II | *SgPg* Rep I Rep II | *SgPgFn* Rep I Rep II

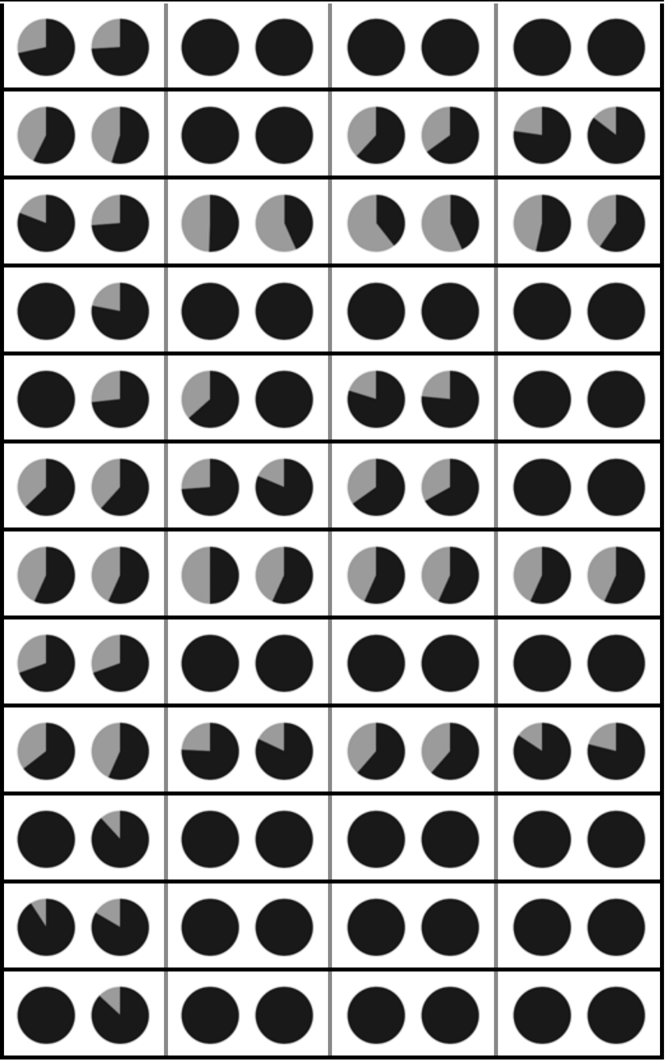

Show detected proteins only
Show all proteins
Filter by category:
ABC Transporter

Proteins found:
1179

Dot Plots Dot Plots

Hendrickson *et al.*

Protein	*Sg*		*SgFn*		*SgPg*		*SgPgFn*		Description
	n	Coverage	n	Coverage	n	Coverage	n	Coverage	
**SGO_1336**	12	23.9%	7	14.0%	16	33.5%	5	7.1%	pcrA; ATP-dependent DNA helicase PcrA
	15	28.0%	8	16.3%	14	27.8%	6	9.7%	
**SGO_1337**									cation efflux family protein
	3	9.1%							
**SGO_1338**	8	42.2%	4	30.8%					Signal peptidase I
	11	60.5%	4	30.8%	3	15.7%			
**SGO_1339**	25	56.1%	35	79.2%	29	72.5%	27	69.1%	pyk; pyruvate kinase
	32	69.5%	36	81.0%	34	78.6%	28	74.7%	
**SGO_1340**	14	43.8%	20	52.7%	20	61.3%	15	42.9%	Phosphofructokinase
	20	60.4%	19	50.3%	20	60.1%	15	42.9%	
**SGO_1341**	10	14.4%	4	5.8%	4	5.7%			dnaE; DNA-directed DNA polymerase III alpha chain
	8	12.2%	7	10.0%					
**SGO_1342**	18	68.4%	18	67.5%	18	65.4%	16	65.0%	ABC transporter, ATP-binding protein SP1715
	18	68.4%	19	74.8%	17	65.4%	16	65.0%	
**SGO_1343**	5	15.4%			3	10.5%			ABC transporter, ATP-binding protein SP1715
	5	15.4%							
**SGO_1347**	10	37.4%	3	13.5%					membrane associated lipoprotein
	13	45.6%							
**SGO_1355**	4	27.9%							lipoprotein, putative
	4	22.1%	3	16.2%					
**SGO_1360**	3	9.0%							ABC transporter domain protein
	6	14.6%							
**SGO_1361**									ADP-ribosylhydrolase
	3	21.1%	3	23.2%					

Sg Rep I Rep II | *SgFn* Rep I Rep II | *SgPg* Rep I Rep II | *SgPgFn* Rep I Rep II

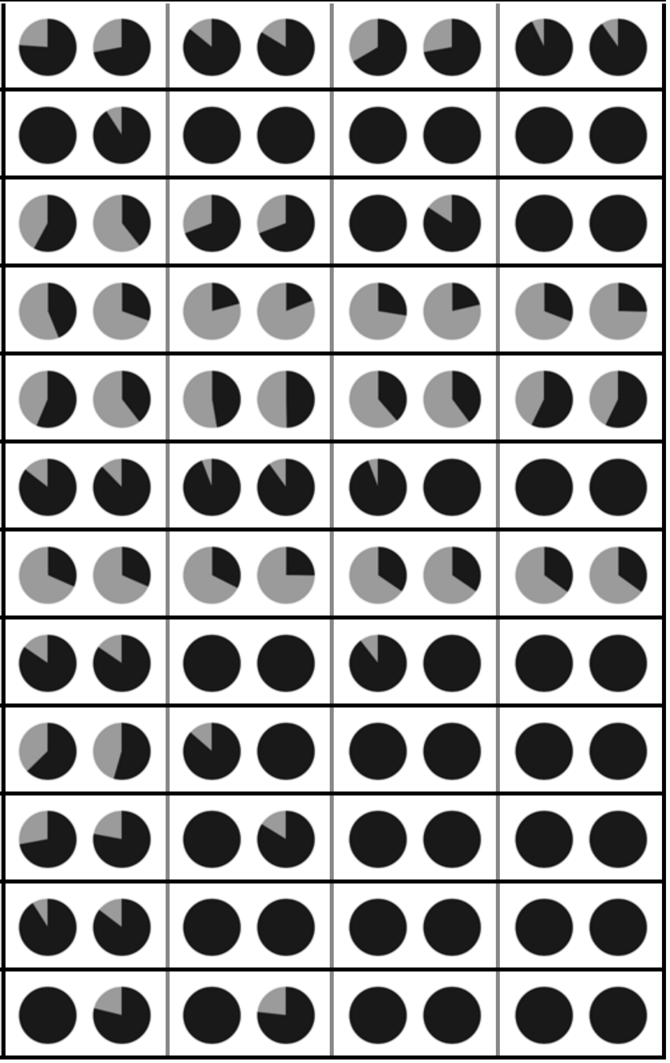

Show detected proteins only
Show all proteins
Filter by category:
ABC Transporter

Proteins found:
1179

Dot Plots Dot Plots

Hendrickson *et al.*

Protein	*Sg* n	*Sg* Coverage	*SgFn* n	*SgFn* Coverage	*SgPg* n	*SgPg* Coverage	*SgPgFn* n	*SgPgFn* Coverage	Description
**SGO_1364**	13	33.3%	4	16.4%	13	42.1%			rumA-2; 23S rRNA (uracil-5-)-methyltransferase RumA
	17	44.5%	7	27.0%	13	43.6%	4	12.3%	
**SGO_1365**	14	40.2%	5	13.8%	5	19.4%			transcription regulator yrfE
	18	45.6%	6	14.0%	3	8.4%	3	13.5%	
**SGO_1366**					6	34.5%			pheA; prephenate dehydratase
					4	21.5%			
**SGO_1367**	5	42.2%	6	39.1%	3	21.1%			aroK; shikimate kinase
	6	40.4%	6	42.2%	3	18.0%			
**SGO_1368**	3	8.7%	8	30.9%	5	20.4%			aroA; 3-phosphoshikimate 1-carboxyvinyltransferase
	6	20.4%	8	32.1%	5	24.1%			
**SGO_1369**	6	28.3%	9	40.8%	5	25.3%	3	15.5%	L-2-hydroxyisocaproate dehydrogenase
	8	32.2%	9	43.4%	6	35.9%			
**SGO_1370**	6	73.2%	6	73.2%	6	73.2%	3	33.9%	Protein of unknown function (DUF964) superfamily
	6	73.2%	5	56.3%	4	51.8%	4	51.8%	
**SGO_1372**	3	10.1%			6	30.4%			aroC; chorismate synthase
	8	33.8%	3	15.2%	11	41.8%	3	12.6%	
**SGO_1373**	3	15.8%			5	26.8%	3	17.2%	aroB; 3-dehydroquinate synthase
	6	25.9%	3	16.9%	5	26.8%			
**SGO_1374**	3	13.0%			6	28.4%			aroE; shikimate 5-dehydrogenase
	4	16.8%			3	16.8%			
**SGO_1375**	7	41.3%	4	26.2%	6	37.8%	4	23.1%	aroD; 3-dehydroquinate dehydratase, type I
	8	50.7%	3	17.8%	6	38.2%			
**SGO_1376**	5	17.6%							Methyltransferase
	5	18.3%							

Sg Rep I Rep II | *SgFn* Rep I Rep II | *SgPg* Rep I Rep II | *SgPgFn* Rep I Rep II

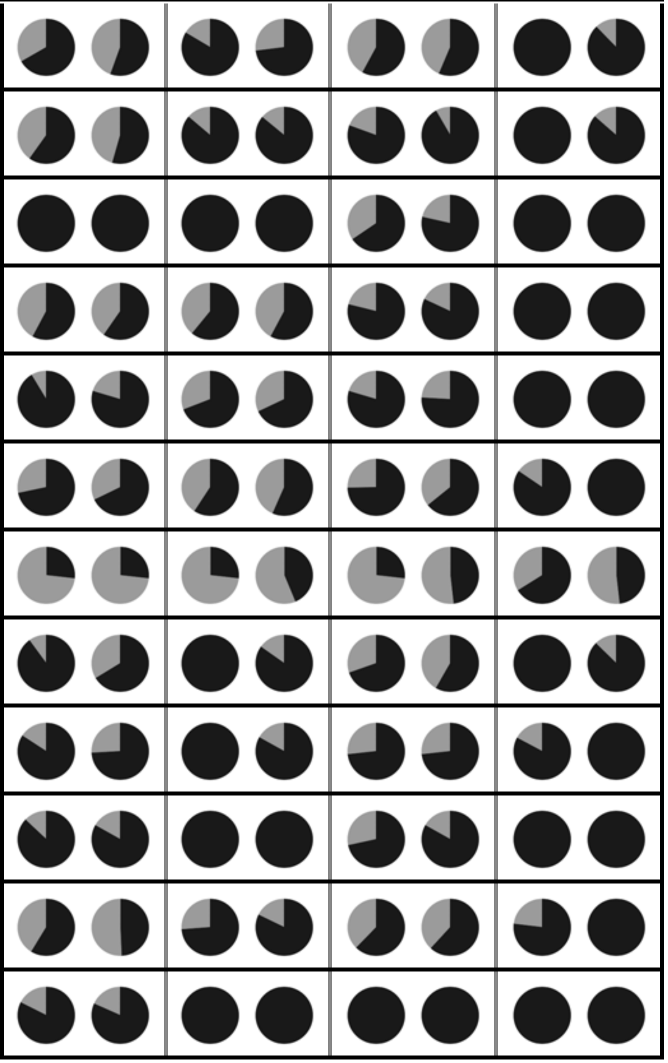

Show detected proteins only
Show all proteins
Filter by category:
ABC Transporter

Proteins found:
1179

Dot Plots Dot Plots

Hendrickson *et al.*

Streptococcus gordonii

Protein	*Sg* n	*Sg* Coverage	*SgFn* n	*SgFn* Coverage	*SgPg* n	*SgPg* Coverage	*SgPgFn* n	*SgPgFn* Coverage	Description
**SGO_1377**	22	43.8%	11	24.7%	11	23.6%	4	6.7%	sulfatase
	27	49.0%	14	31.8%	13	26.2%	4	8.8%	
**SGO_1381**	18	20.0%	12	13.9%	9	11.6%	3	3.1%	csn1; CRISPR-associated protein, Csn1 family
	28	27.6%	13	13.4%	12	14.8%	4	4.4%	
**SGO_1383**	9	53.0%	8	47.0%	6	46.1%	6	40.0%	rplS; ribosomal protein L19
	10	60.9%	10	57.4%	6	40.0%	9	53.0%	
**SGO_1386**			8	72.7%	5	62.5%			chorismate mutase
	3	30.7%	7	68.2%	3	48.9%			
**SGO_1388**	9	16.9%	3	6.4%					pulA-1; pullulanase, type I
	14	27.1%	6	13.1%					
**SGO_1389**	6	21.4%	3	11.9%					conserved hypothetical protein TIGR00147
	6	20.1%	4	14.2%					
**SGO_1390**	13	26.8%	8	16.0%	7	15.5%	6	12.7%	ligA; DNA ligase, NAD-dependent
	13	29.9%	10	23.6%	7	15.3%	6	12.7%	
**SGO_1394**			6	46.8%	3	14.5%			hypothetical protein SGO_1394
					3	16.2%			
**SGO_1397**	8	42.8%	9	41.8%	8	45.6%	6	30.2%	map; methionine aminopeptidase, type I
	9	48.1%	8	39.6%	8	44.2%	5	26.3%	
**SGO_1398**	15	47.8%	3	8.9%	5	14.1%			hypothetical protein SGO_1398
	14	38.4%	3	8.5%	4	10.8%			
**SGO_1400**	6	22.9%	7	20.7%	9	29.0%	6	22.6%	murA-2; UDP-N-acetylglucosamine 1-carboxyvinyltransferase
	11	37.6%	6	24.8%	7	23.8%	3	14.8%	
**SGO_1405**									hisA; phosphoribosylformimino-5-aminoimidazole carboxamide ribotide isomerase
					3	13.8%			

Show detected proteins only
Show all proteins
Filter by category:
ABC Transporter

Proteins found: 1179

Dot Plots Dot Plots

Protein	*Sg* n	*Sg* Coverage	*SgFn* n	*SgFn* Coverage	*SgPg* n	*SgPg* Coverage	*SgPgFn* n	*SgPgFn* Coverage	Description
**SGO_1413**	8	8.7%	6	7.4%	8	8.6%			first chain of major exonuclease RexA
	16	18.0%	12	15.9%	7	7.7%			
**SGO_1414**	8	11.5%	6	8.3%	4	5.6%	3	4.8%	rexB; putative exonuclease RexB
	10	14.7%	8	12.2%	6	9.0%	4	5.9%	
**SGO_1415**	4	5.7%							LPXTG cell wall surface protein, X-prolyl dipeptidylaminopeptidase, putative
	5	6.3%							
**SGO_1416**									putative histidine kinase
	5	14.8%							
**SGO_1417**									response regulator protein
					4	27.1%			
**SGO_1422**	9	28.9%	10	39.0%	9	34.2%	6	20.8%	hypothetical protein SGO_1422
	10	29.8%	10	31.3%	8	25.9%	6	20.8%	
**SGO_1426**	31	75.1%	32	77.9%	32	77.2%	27	75.1%	eno; enolase
	31	77.0%	33	85.7%	30	77.2%	31	77.2%	
**SGO_1431**	31	53.5%	21	43.6%	25	47.7%	18	39.0%	EzrA; Septation ring formation regulator ezrA
	28	50.3%	20	41.3%	27	49.0%	20	40.1%	
**SGO_1432**	18	33.4%	12	26.5%	16	33.3%	6	10.9%	gyrB; DNA gyrase, B subunit
	20	38.7%	14	34.1%	19	37.9%	6	9.4%	
**SGO_1434**			4	29.1%	4	40.1%	3	22.0%	thiJ; 4-methyl-5(beta-hydroxyethyl)-thiazole monophosphate synthesis protein
	4	32.4%	3	28.0%	3	25.8%			
**SGO_1437**	6	9.2%							DnaQ family exonuclease/DinG family helicase, putative
	7	11.0%							
**SGO_1439**	8	23.4%	5	19.5%	5	17.5%	3	17.2%	ftsX; cell division protein FtsX
	10	25.6%	5	21.4%	4	17.2%	3	17.2%	

Sg Rep I Rep II | *SgFn* Rep I Rep II | *SgPg* Rep I Rep II | *SgPgFn* Rep I Rep II

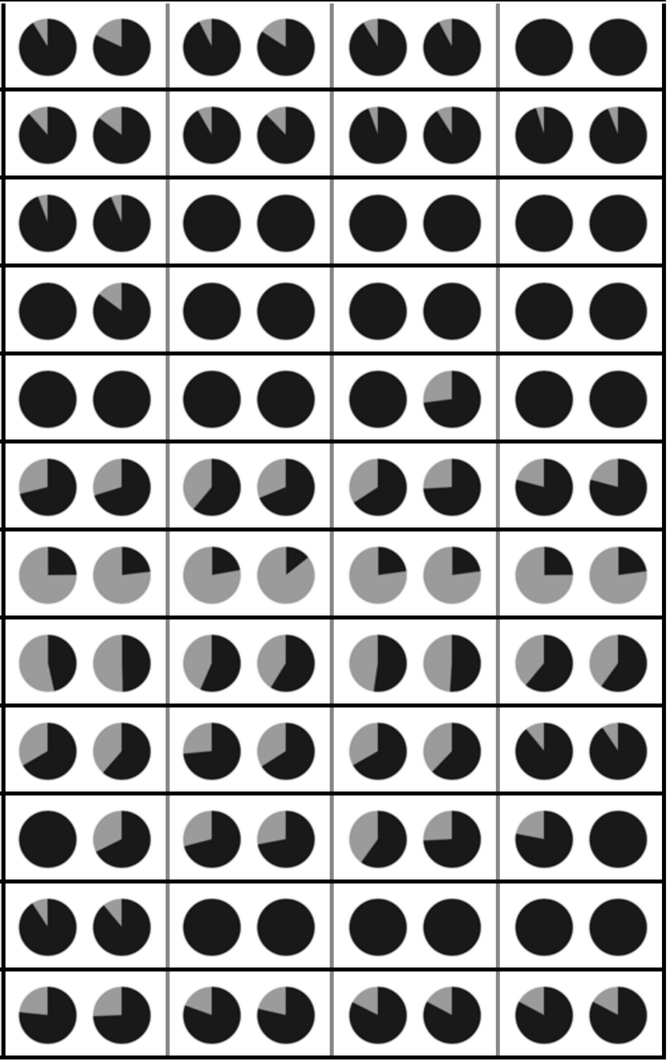

Show detected proteins only
Show all proteins

Filter by category:
ABC Transporter

Proteins found:
1179

Dot Plots Dot Plots

Hendrickson *et al.*

Protein	*Sg* n	*Sg* Coverage	*SgFn* n	*SgFn* Coverage	*SgPg* n	*SgPg* Coverage	*SgPgFn* n	*SgPgFn* Coverage	Description
SGO_1440	14	69.6%	15	69.1%	10	59.1%	7	43.5%	cell-division ATP-binding protein FtsE
	18	74.3%	13	67.4%	10	55.2%	6	45.7%	
SGO_1441	8	29.9%	8	30.2%	9	30.2%	5	20.3%	prfB; peptide chain release factor 2
	6	22.8%	7	27.5%	11	35.2%	3	11.8%	
SGO_1446	12	36.0%	9	27.6%	12	37.3%	4	11.2%	murF; UDP-N-acetylmuramoyl-tripeptide--D-alanyl-D-alanine ligase
	14	39.7%	8	25.2%	12	34.2%	3	11.4%	
SGO_1447	13	45.1%	8	32.2%	14	52.0%	6	26.1%	ddlA; D-Ala-D-Ala ligase
	15	53.2%	8	28.7%	14	50.6%	5	23.3%	
SGO_1449	24	47.7%			9	21.9%			pbp2b; penicillin-binding protein 2B
	26	50.6%			11	26.3%			
SGO_1450	6	27.1%	4	19.0%	6	28.2%	3	14.8%	hypothetical protein SGO_1450
	7	30.6%	5	23.6%	5	22.2%	6	28.2%	
SGO_1451	6	43.8%	4	33.5%	6	42.7%	6	40.0%	frr; ribosome recycling factor
	7	44.3%	4	33.5%	7	43.8%	6	39.5%	
SGO_1452	10	46.9%	6	38.6%	7	45.6%	9	54.4%	pyrH; uridylate kinase
	9	45.2%	4	27.4%	7	45.6%	6	34.0%	
SGO_1453					4	11.7%	3	6.4%	glycosyl transferase, family 8 SP1766
			3	8.7%			3	8.3%	
SGO_1454	3	6.6%							amino acid permease family protein
	3	6.6%							
SGO_1455	8	39.3%	10	59.4%	9	45.0%	9	49.3%	rplA; ribosomal protein L1
	12	50.7%	10	55.9%	9	49.3%	9	45.0%	
SGO_1456	4	30.5%	4	35.5%	4	30.5%	6	44.7%	rplK; ribosomal protein L11
	5	37.6%	5	51.8%	4	30.5%	6	42.6%	

Sg Rep I Rep II | *SgFn* Rep I Rep II | *SgPg* Rep I Rep II | *SgPgFn* Rep I Rep II

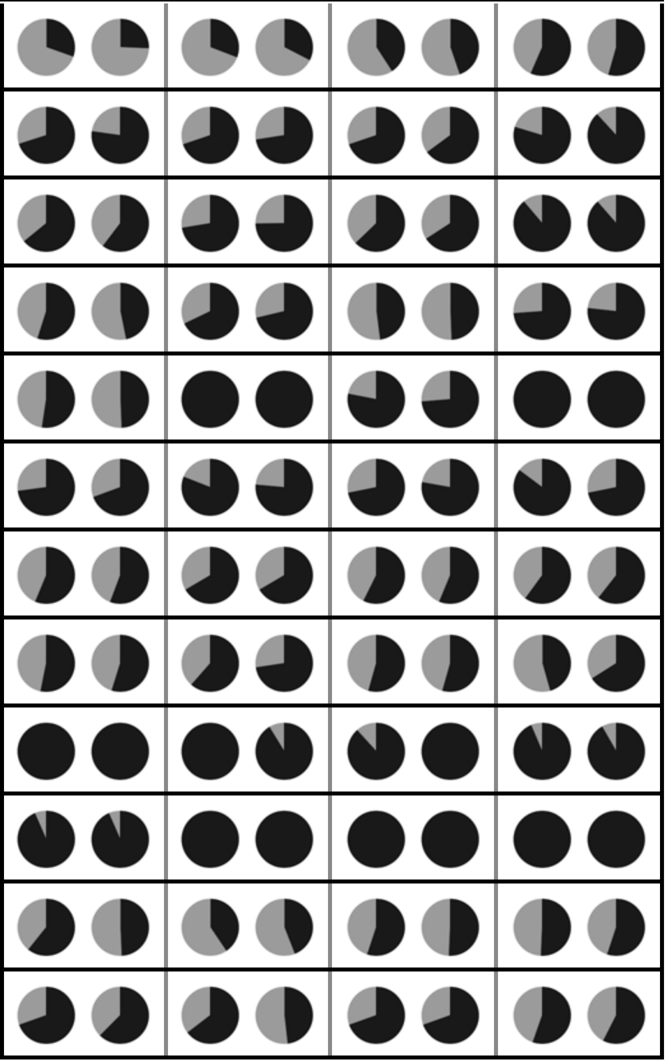

Show detected proteins only
Show all proteins
Filter by category:
ABC Transporter

Proteins found:
1179

Dot Plots Dot Plots

Hendrickson *et al.*

Protein	*Sg* n	*Sg* Coverage	*SgFn* n	*SgFn* Coverage	*SgPg* n	*SgPg* Coverage	*SgPgFn* n	*SgPgFn* Coverage	Description
SGO_1458	19	33.8%	11	20.8%	5	8.2%	4	7.7%	aha1; cation-transporting ATPase yfgQ
	21	38.2%	8	15.1%	4	9.0%			
SGO_1459			6	67.2%	3	35.2%			glyoxylase family protein
			5	65.6%					
SGO_1460	17	32.5%	4	8.7%	11	23.0%	4	6.4%	DNA translocase ftsK
	19	34.3%	4	8.5%	7	12.3%			
SGO_1463	14	61.4%	5	27.0%	11	52.1%	5	27.0%	peptidyl-prolyl cis-trans isomerase
	15	61.4%	4	17.6%	7	40.1%	6	28.5%	
SGO_1464	10	94.9%			8	85.9%	5	84.6%	uncharacterized probable metal-binding protein
	11	100.0%			7	85.9%	4	50.0%	
SGO_1465	17	43.9%	8	25.5%	13	34.3%	8	26.7%	ABC transporter, ATP-binding protein SP0770
	20	50.5%	6	19.1%	10	31.4%	6	21.4%	
SGO_1466	7	40.0%	6	33.0%	7	42.2%			MTA/SAH nucleosidase
	4	19.6%	6	27.0%	6	37.0%			
SGO_1468									MutT/nudix family protein
	3	20.2%							
SGO_1469	12	35.9%	10	31.2%	13	48.4%	13	44.0%	glmU; UDP-N-acetylglucosamine pyrophosphorylase
	13	43.1%	7	23.7%	14	45.8%	13	38.6%	
SGO_1472			5	24.7%	5	32.0%			acetyltransferase, GNAT family
	3	20.6%	3	15.0%	5	30.4%	3	24.7%	
SGO_1475	7	22.1%							lipoprotein, putative
	9	32.7%							
SGO_1483	4	9.8%							ABC transporter, permease protein, probable SP0913

Sg Rep I Rep II | *SgFn* Rep I Rep II | *SgPg* Rep I Rep II | *SgPgFn* Rep I Rep II

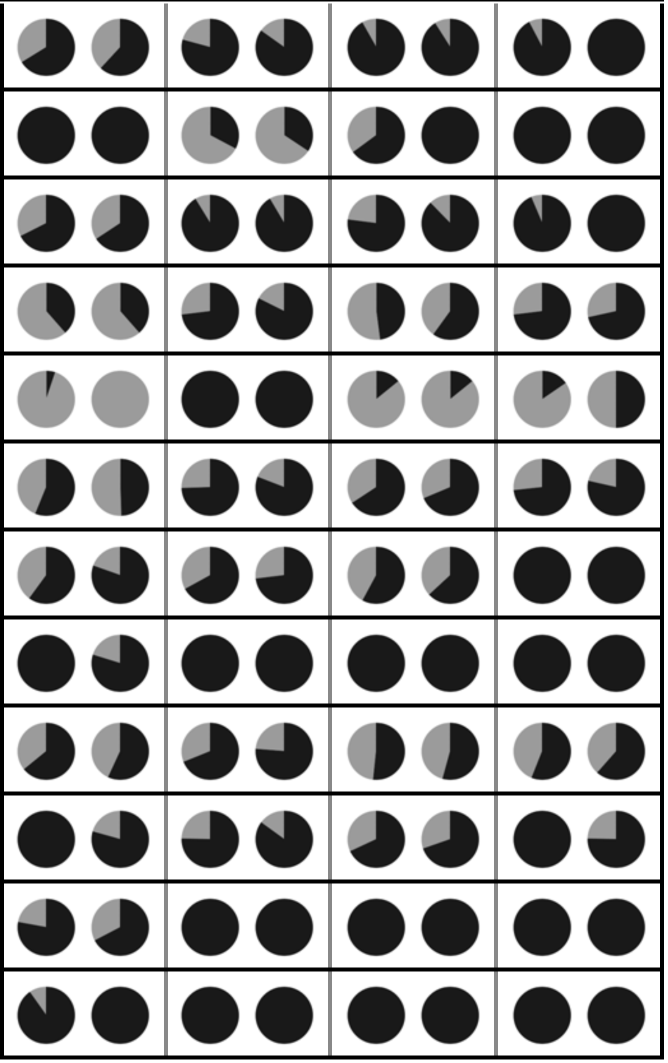

Show detected proteins only
Show all proteins
Filter by category:
ABC Transporter

Proteins found:
1179

Dot Plots Dot Plots

Hendrickson *et al.*

Protein	*Sg* n	*Sg* Coverage	*SgFn* n	*SgFn* Coverage	*SgPg* n	*SgPg* Coverage	*SgPgFn* n	*SgPgFn* Coverage	Description	*Sg* Rep I	*Sg* Rep II	*SgFn* Rep I	*SgFn* Rep II	*SgPg* Rep I	*SgPg* Rep II	*SgPgFn* Rep I	*SgPgFn* Rep II
**SGO_1484**					3	20.8%			ABC transporter, ATP-binding protein								
					3	20.8%											
**SGO_1486**			8	5.9%			4	3.2%	beta-galactosidase								
	5	3.1%					5	3.9%									
**SGO_1487**	12	9.4%	11	10.5%					LPXTG cell wall surface protein, Cna protein B-type domain								
	18	12.8%	7	5.0%	4	4.0%											
**SGO_1493**	10	25.3%	4	12.7%					hypothetical protein SGO_1493								
	14	38.9%	6	19.9%	4	16.1%											
**SGO_1495**	3	34.6%							putative transcriptional regulator								
	6	53.4%															
**SGO_1498**	3	5.7%							ABC transporter, permease protein, probable SP0913								
**SGO_1504**	3	13.8%							histidine kinase								
	5	20.9%															
**SGO_1507**	7	23.6%							hypothetical protein SGO_1507								
	11	30.5%															
**SGO_1512**	3	9.2%	4	18.2%	5	13.7%			lacG; 6-phospho-beta-galactosidase								
			5	18.4%													
**SGO_1513**									lacE; PTS system, lactose-specific IIBC component								
	3	5.5%															
**SGO_1516**	8	37.1%			9	44.2%	4	22.4%	lacD-2; tagatose 1,6-diphosphate aldolase								
	9	31.3%			13	53.4%	6	31.3%									
**SGO_1518**	4	28.1%			6	46.2%			lacB-2; galactose-6-phosphate isomerase, LacB subunit								
	6	33.9%			6	46.2%											

Hendrickson *et al.*

Protein	*Sg* n	*Sg* Coverage	*SgFn* n	*SgFn* Coverage	*SgPg* n	*SgPg* Coverage	*SgPgFn* n	*SgPgFn* Coverage	Description
SGO_1520	4	10.4%							PTS system, IIC component
SGO_1521									PTS system IIB component (galactitol/fructose specific)
	4	47.5%							
SGO_1523									lacD-1; tagatose 1,6-diphosphate aldolase
SGO_1527	3	17.9%							Lactose phosphotransferase system repressor
SGO_1529	6	32.3%	6	27.4%	5	27.7%			hypothetical protein SGO_1529
	10	46.7%	6	34.7%	3	18.2%			
SGO_1530	23	46.4%	22	47.0%	26	52.9%	17	36.6%	methionine-tRNA ligase
	27	53.5%	16	33.3%	24	50.2%	15	35.0%	
SGO_1531	11	54.9%	6	29.1%	10	60.0%	6	34.9%	xth; exodeoxyribonuclease III
	11	59.6%	5	26.5%	8	49.5%	5	33.1%	
SGO_1534	5	35.9%	4	46.2%	8	68.4%			ArsC family
	6	49.6%	5	57.3%	6	62.4%	4	34.2%	
SGO_1536	10	40.5%	4	19.4%	9	43.0%	4	17.3%	conserved hypothetical protein TIGR00096
	11	45.8%	5	26.1%	10	42.3%	4	18.7%	
SGO_1538	4	20.4%							DNA-directed DNA polymerase III, delta'' chain
	3	16.7%							
SGO_1539	7	42.0%	7	36.3%	8	48.1%	4	22.2%	tmk; thymidylate kinase
	7	41.5%	5	29.7%	9	48.6%	6	42.0%	
SGO_1540	3	12.2%							YitT family protein
	6	19.2%			4	23.0%			

Columns *Sg*, *SgFn*, *SgPg*, *SgPgFn*: Rep I, Rep II

Show detected proteins only
Show all proteins
Filter by category:
ABC Transporter

Proteins found:
1179

Dot Plots Dot Plots

Coverage									*Streptococcus gordonii*	Hackett Laboratory	UW

Protein	*Sg* n	*Sg* Coverage	*SgFn* n	*SgFn* Coverage	*SgPg* n	*SgPg* Coverage	*SgPgFn* n	*SgPgFn* Coverage	Description	*Sg* Rep I	*Sg* Rep II	*SgFn* Rep I	*SgFn* Rep II	*SgPg* Rep I	*SgPg* Rep II	*SgPgFn* Rep I	*SgPgFn* Rep II
**SGO_1541**	4	33.3%	8	40.6%	5	32.6%	3	27.5%	atpC; ATP synthase F1, epsilon subunit								
	5	33.3%	6	31.2%	4	32.6%	6	39.9%									
**SGO_1542**	25	73.3%	30	75.9%	24	64.1%	19	58.5%	atpD; ATP synthase F1, beta subunit								
	30	75.4%	29	72.6%	22	62.2%	20	58.8%									
**SGO_1543**	15	55.6%	12	55.6%	10	36.9%	4	21.2%	atpG; ATP synthase F1, gamma subunit								
	16	50.2%	10	44.4%	11	47.1%	4	19.8%									
**SGO_1544**	17	41.9%	15	43.7%	17	45.1%	9	26.1%	atpA; ATP synthase F1, alpha subunit								
	16	41.9%	17	48.3%	17	45.1%	9	22.8%									
**SGO_1545**	5	42.1%	6	39.9%	4	28.7%	4	33.7%	atpH; ATP synthase F1, delta subunit								
	6	46.6%	8	58.4%	4	33.7%	4	33.7%									
**SGO_1546**	14	56.1%	15	61.6%	10	49.4%	10	47.0%	atpF; ATP synthase F0, B subunit								
	15	61.6%	8	39.0%	11	49.4%	9	48.8%									
**SGO_1550**	22	38.1%	19	30.2%	25	45.0%	13	22.4%	glgP-1; glycogen phosphorylase								
	27	44.4%	26	42.7%	27	44.2%	20	32.7%									
**SGO_1551**	5	17.4%	14	38.0%	9	25.0%	3	9.0%	glgA; Glycogen synthase								
	14	36.8%	15	42.6%	6	16.8%	5	14.7%									
**SGO_1552**	8	28.6%	13	47.7%	12	41.7%	8	26.6%	glgD; glucose-1-phosphate adenylyltransferase, GlgD subunit								
	9	26.9%	11	42.0%	12	43.7%	8	33.4%									
**SGO_1553**	7	23.9%	14	46.1%	14	45.5%	7	27.6%	glgC; glucose-1-phosphate adenylyltransferase								
	10	33.9%	18	53.4%	15	50.5%	7	27.1%									
**SGO_1554**	13	30.8%	24	47.4%	16	33.6%	12	28.9%	glgB; 1,4-alpha-glucan branching enzyme								
	15	34.6%	22	48.0%	20	38.5%	8	21.5%									
**SGO_1555**	25	62.2%	28	68.3%	28	66.6%	24	55.3%	ptsI; phosphoenolpyruvate-protein phosphotransferase								
	31	69.8%	31	74.5%	26	65.3%	21	54.4%									

◉ Show detected proteins only
○ Show all proteins
☐ Filter by category:
ABC Transporter

Proteins found:
1179

Dot Plots | Dot Plots

Hendrickson *et al.*

Protein	*Sg* n	*Sg* Coverage	*SgFn* n	*SgFn* Coverage	*SgPg* n	*SgPg* Coverage	*SgPgFn* n	*SgPgFn* Coverage	Description	*Sg* Rep I	*Sg* Rep II	*SgFn* Rep I	*SgFn* Rep II	*SgPg* Rep I	*SgPg* Rep II	*SgPgFn* Rep I	*SgPgFn* Rep II
SGO_1556	3	39.1%	5	41.4%	3	39.1%	3	39.1%	phosphocarrier protein HPr								
	5	41.4%	4	39.1%	3	39.1%	3	39.1%									
SGO_1557	3	68.1%							NrdH-redoxin								
	3	68.1%															
SGO_1558	27	48.0%	31	53.8%	25	47.3%	19	38.8%	nrdE; ribonucleoside-diphosphate reductase large chain								
	32	49.4%	23	40.3%	30	52.0%	16	30.7%									
SGO_1559	12	40.4%	11	46.4%	13	46.4%	9	33.9%	ribonucleoside-diphosphate reductase, beta subunit								
	13	41.1%	10	38.9%	13	46.4%	9	28.2%									
SGO_1561	3	13.6%							putative zinc metallopeptidase								
	3	13.6%															
SGO_1562	4	10.6%							hypothetical protein SGO_1562								
	3	10.6%															
SGO_1564	3	53.7%			3	53.7%			transcription regulator, probable -related protein								
	3	53.7%			3	53.7%											
SGO_1570	33	43.5%	29	42.4%	37	47.0%	23	35.4%	alaS; alanyl-tRNA synthetase								
	41	50.2%	24	38.6%	34	42.4%	29	37.4%									
SGO_1571	6	43.5%							Protein of unknown function (DUF567) superfamily								
	7	44.7%															
SGO_1572	26	69.3%	8	32.0%					proteinase maturation protein, putative								
	26	70.9%	5	20.7%			4	17.8%									
SGO_1573			3	23.2%	3	29.4%			O-methyltransferase family protein								
	4	17.5%			5	39.5%											
SGO_1574	17	35.5%	22	44.2%	21	49.8%	15	29.8%	pepF-1; oligoendopeptidase F								
	18	37.0%	21	44.7%	24	52.7%	15	30.8%									

Hendrickson *et al.*

Protein	*Sg* n	*Sg* Coverage	*SgFn* n	*SgFn* Coverage	*SgPg* n	*SgPg* Coverage	*SgPgFn* n	*SgPgFn* Coverage	Description
**SGO_1576**	3	8.9%							ptcC; PTS system, IIC component
	3	8.2%							
**SGO_1580**			3	45.2%	4	49.0%	4	49.0%	PTS system, Lactose/Cellobiose specific IIB subunit
			3	49.0%	4	49.0%	3	49.0%	
**SGO_1585**	14	40.4%			3	13.3%			D-Alanyl-D-Alanine carboxypeptidase
	13	37.8%			5	19.6%	3	6.7%	
**SGO_1587**	5	26.9%	4	23.4%	4	24.3%	4	24.3%	queA; S-adenosylmethionine:tRNA ribosyltransferase-isomerase
	9	42.1%	8	33.0%	4	24.3%	3	19.0%	
**SGO_1588**	4	48.1%							arcR; arginine repressor
	5	48.1%							
**SGO_1589**	5	13.5%	11	29.3%					arcT; putative transaminase/peptidase
	7	19.8%	10	30.0%					
**SGO_1590**	5	9.9%	4	9.1%					arcD; arginine-ornithine antiporter
	6	11.7%	3	9.1%					
**SGO_1591**	10	43.5%	17	70.8%	9	39.0%	5	27.6%	arcC; carbamate kinase
	13	54.3%	17	65.4%	7	27.6%	6	32.4%	
**SGO_1592**	15	53.8%	25	64.8%	12	49.4%	10	42.3%	arcB; ornithine carbamoyltransferase
	15	52.7%	26	73.1%	12	46.7%	11	45.3%	
**SGO_1593**	28	72.6%	42	88.3%	20	62.3%	18	60.4%	arcA; arginine deiminase
	30	76.5%	38	84.8%	24	66.7%	17	53.3%	
**SGO_1594**	5	22.8%							Crp/Fnr family protein
	6	26.3%							
**SGO_1598**	3	11.8%							hypothetical protein SGO_1598
	5	17.4%							

Sg Rep I Rep II | *SgFn* Rep I Rep II | *SgPg* Rep I Rep II | *SgPgFn* Rep I Rep II

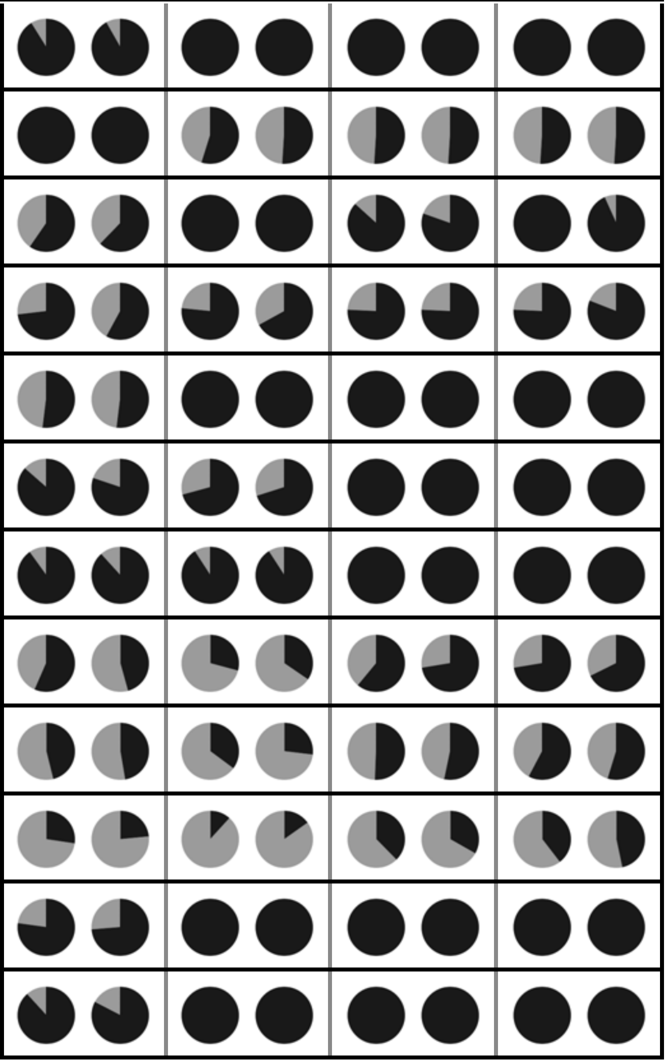

Show detected proteins only
Show all proteins
Filter by category:
ABC Transporter

Proteins found:
1179

Dot Plots Dot Plots

Hendrickson *et al.*

Protein	*Sg* n	*Sg* Coverage	*SgFn* n	*SgFn* Coverage	*SgPg* n	*SgPg* Coverage	*SgPgFn* n	*SgPgFn* Coverage	Description
**SGO_1599**	10	56.2%	15	57.2%	10	52.2%	10	56.2%	sodA; manganese-dependent superoxide dismutase
	9	52.2%	13	57.2%	10	52.2%	11	56.2%	
**SGO_1600**	6	25.8%							DNA polymerase III, delta chain
	7	27.8%							
**SGO_1601**	3	4.0%							celB; DNA internalization-related competence protein celB
	4	5.6%							
**SGO_1604**	12	48.3%	6	30.5%	6	28.4%			acyltransferase family protein
	15	55.9%	5	32.6%	5	23.7%	3	18.6%	
**SGO_1605**	16	29.3%	3	6.5%	5	11.0%			P-type ATPase, metal cation transport
	20	31.3%	3	8.0%	5	11.9%	3	6.1%	
**SGO_1606**	4	25.0%			4	25.0%			DNA methyltransferase signature protein
	4	25.0%			3	19.2%			
**SGO_1609**	18	45.9%	7	17.2%	14	38.4%	12	35.2%	ATP-dependent RNA helicase, DEAD/DEAH box family
	22	51.2%	6	17.0%	15	42.8%	13	35.0%	
**SGO_1614**	9	32.5%							magnesium and cobalt transporter CorA family
	8	25.2%							
**SGO_1617**	12	37.0%	8	20.6%	10	23.9%	9	24.1%	prfC; peptide chain release factor 3
	19	48.2%	10	20.4%	16	38.1%	9	25.9%	
**SGO_1619**	26	34.0%	10	17.4%	21	32.7%	12	19.7%	cation-transporting ATPase, E1-E2 family
	26	34.0%	15	26.8%	20	35.2%	11	18.3%	
**SGO_1621**	8	25.2%	6	21.7%	5	16.9%	3	10.4%	HD domain protein
	9	26.3%	9	31.2%	7	25.2%			
**SGO_1622**	9	40.5%	6	30.5%	6	29.0%	6	29.0%	Cof family protein
	10	43.1%	4	21.2%	6	29.7%	4	20.1%	

Columns: *Sg* Rep I / Rep II, *SgFn* Rep I / Rep II, *SgPg* Rep I / Rep II, *SgPgFn* Rep I / Rep II

Hendrickson *et al.*

Protein	*Sg* n	*Sg* Coverage	*SgFn* n	*SgFn* Coverage	*SgPg* n	*SgPg* Coverage	*SgPgFn* n	*SgPgFn* Coverage	Description
SGO_1623	10	31.5%	8	23.2%	11	37.2%	5	20.5%	murM; MurM
	15	48.7%	8	24.9%	11	39.1%			
SGO_1624	8	25.1%	7	17.8%	5	15.6%			murN; MurN protein
	10	25.1%	7	24.1%	5	13.1%	5	14.8%	
SGO_1625	15	77.1%	11	61.9%	13	64.2%	9	57.8%	acetoin utilization putative/CBS domain protein
	16	77.5%	10	55.5%	12	59.6%	6	39.4%	
SGO_1626	9	50.8%			8	45.3%	4	23.7%	branched-chain amino acid ABC transporter, ATP-binding protein
	9	46.6%	5	32.2%	8	42.8%	6	35.2%	
SGO_1627	7	41.7%			6	37.0%	5	33.1%	branched-chain amino acid ABC transporter, ATP-binding protein
	10	46.9%			5	27.6%	5	33.1%	
SGO_1630	25	62.9%	15	50.0%	20	61.1%	17	52.3%	branched-chain amino acid ABC transporter, amino acid-binding protein
	30	70.4%	14	45.6%	20	59.3%	16	47.9%	
SGO_1631	3	28.1%							hypothetical protein SGO_1631
	3	29.2%							
SGO_1632	4	15.8%	5	39.8%	5	37.8%	6	36.7%	clpP; ATP-dependent Clp protease, proteolytic subunit ClpP
	5	21.4%	5	39.8%	5	37.8%	4	15.8%	
SGO_1633	9	58.9%	14	67.5%	14	67.5%	12	66.5%	upp; uracil phosphoribosyltransferase
	12	59.8%	14	72.2%	13	67.5%	10	54.1%	
SGO_1634	34	42.7%	5	9.5%	8	13.9%			magnesium-translocating P-type ATPase
	35	44.0%	5	6.5%	6	8.7%			
SGO_1638	6	15.4%	9	22.2%	8	25.6%			murE; UDP-N-acetylmuramoylalanyl-D-glutamate--2,6-diaminopimelate ligase
	6	15.6%	10	31.4%	6	16.8%			
SGO_1648	10	43.4%	12	59.5%	12	58.8%	10	43.1%	ppx1; inorganic pyrophosphatase, manganese-dependent
	12	46.6%	11	53.4%	12	58.8%	8	36.0%	

Sg Rep I Rep II | *SgFn* Rep I Rep II | *SgPg* Rep I Rep II | *SgPgFn* Rep I Rep II

Show detected proteins only
Show all proteins
Filter by category:
ABC Transporter

Proteins found:
1179

Dot Plots Dot Plots

Hendrickson *et al.*

Protein	*Sg* n	*Sg* Coverage	*SgFn* n	*SgFn* Coverage	*SgPg* n	*SgPg* Coverage	*SgPgFn* n	*SgPgFn* Coverage	Description
**SGO_1649**	9	45.7%	6	33.1%	3	14.1%	3	14.1%	act; pyruvate formate-lyase-activating enzyme
	12	58.0%	10	48.0%	5	23.4%			
**SGO_1650**	14	22.5%							LPXTG cell wall surface protein, collagen binding domain
	16	24.8%							
**SGO_1651**	28	45.6%	20	32.7%					LPXTG cell wall surface protein, nuclease/phosphatase domain
	31	46.6%	18	34.1%					
**SGO_1652**	13	31.2%	5	13.9%	12	31.1%	7	18.3%	intracellular glycosyl hydrolase
	12	28.7%	4	10.9%	10	29.4%	7	19.2%	
**SGO_1653**	20	32.4%	9	18.5%	12	25.9%	5	10.9%	trehalose PTS enzyme II
	25	35.0%	5	9.6%	12	24.5%	7	14.1%	
**SGO_1655**	10	27.1%	5	16.1%	4	12.1%			CBS domain protein/possible hemolysin
	15	33.0%	6	20.2%	4	13.0%			
**SGO_1657**	4	5.3%							membrane protein
	6	8.0%							
**SGO_1661**	3	11.2%							radical SAM protein, TIGR01212 family
	6	31.7%	6	26.9%					
**SGO_1665**									rRNA methylase
	4	35.7%							
**SGO_1666**	16	39.5%	14	34.8%	13	35.8%	6	19.6%	trkA; potassium uptake protein, Trk family
	20	47.0%	14	38.3%	13	36.9%	4	14.3%	
**SGO_1669**	12	45.0%	11	55.8%	11	40.5%	10	47.1%	ribosomal large subunit pseudouridine synthase B
	17	57.4%	8	36.4%	13	45.9%	8	33.9%	
**SGO_1670**	4	30.9%							scpB; segregation and condensation protein B

Sg Rep I Rep II | *SgFn* Rep I Rep II | *SgPg* Rep I Rep II | *SgPgFn* Rep I Rep II

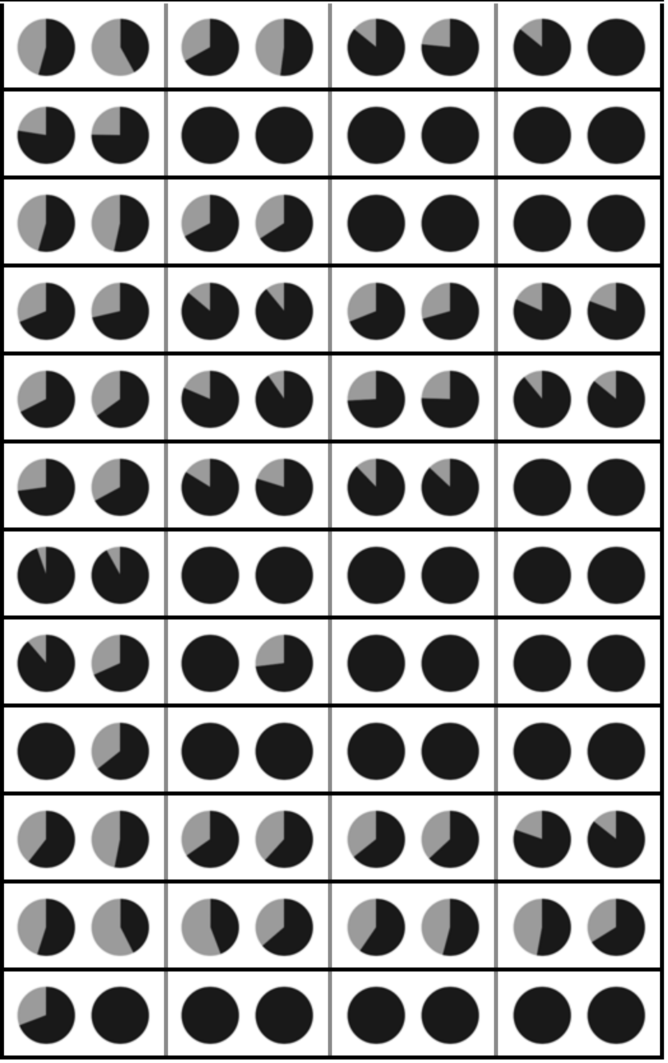

Show detected proteins only
Show all proteins
Filter by category:
ABC Transporter

Proteins found:
1179

Dot Plots Dot Plots

Hendrickson *et al.*

Protein	*Sg* n	*Sg* Coverage	*SgFn* n	*SgFn* Coverage	*SgPg* n	*SgPg* Coverage	*SgPgFn* n	*SgPgFn* Coverage	Description
**SGO_1674**	5	32.4%	4	29.5%	5	27.7%			phosphodiesterase, MJ0936 family
	5	32.4%	5	37.0%	3	22.5%			
**SGO_1675**	7	26.2%	6	20.9%	9	40.3%	3	12.6%	HAM1 protein-like protein
	11	37.5%	8	31.7%	7	35.4%	4	16.9%	
**SGO_1676**	4	22.3%	4	23.5%	9	45.5%	4	23.1%	murI; glutamate racemase
	4	25.4%	4	21.6%	11	59.8%	6	29.9%	
**SGO_1678**	4	13.9%	6	24.0%	6	23.8%	3	13.0%	lysA; diaminopimelate decarboxylase
	5	19.2%	6	22.4%	3	13.0%			
**SGO_1679**	19	60.8%	15	64.7%	17	60.8%	16	61.4%	phosphotransferase system enzyme II
	23	68.1%	19	68.4%	16	59.9%	15	59.6%	
**SGO_1680**	4	18.7%			4	18.7%	4	18.7%	phosphotransferase system enzyme II
	4	18.7%			4	18.7%			
**SGO_1681**	13	44.2%	10	41.9%	10	41.9%	9	38.6%	PTS system, mannose/fructose/sorbose family, IID component
	15	44.2%	7	35.6%	10	41.9%	9	38.6%	
**SGO_1683**	19	55.1%	17	60.2%	18	61.9%	11	40.0%	serS; seryl-tRNA synthetase
	19	56.5%	13	50.6%	19	63.3%	13	43.5%	
**SGO_1684**	7	26.3%			12	46.6%	7	28.0%	acyl-CoA dehydrogenase family
	6	24.3%			12	43.8%	9	36.7%	
**SGO_1685**	4	33.0%	5	34.1%	5	37.4%	4	26.4%	putative peroxidase / antioxidase
	4	26.9%	4	33.0%	5	37.4%	3	25.3%	
**SGO_1687**	10	48.0%	11	48.4%	10	48.0%	7	35.9%	accA; acetyl-CoA carboxylase, carboxyl transferase, alpha subunit
	8	36.3%	8	35.2%	10	44.5%	9	39.1%	
**SGO_1688**	7	25.4%	8	36.2%	10	43.6%	5	22.3%	accD; acetyl-CoA carboxylase, carboxyl transferase, beta subunit
	13	41.1%	9	35.2%	11	45.3%	4	17.1%	

Sg Rep I Rep II | *SgFn* Rep I Rep II | *SgPg* Rep I Rep II | *SgPgFn* Rep I Rep II

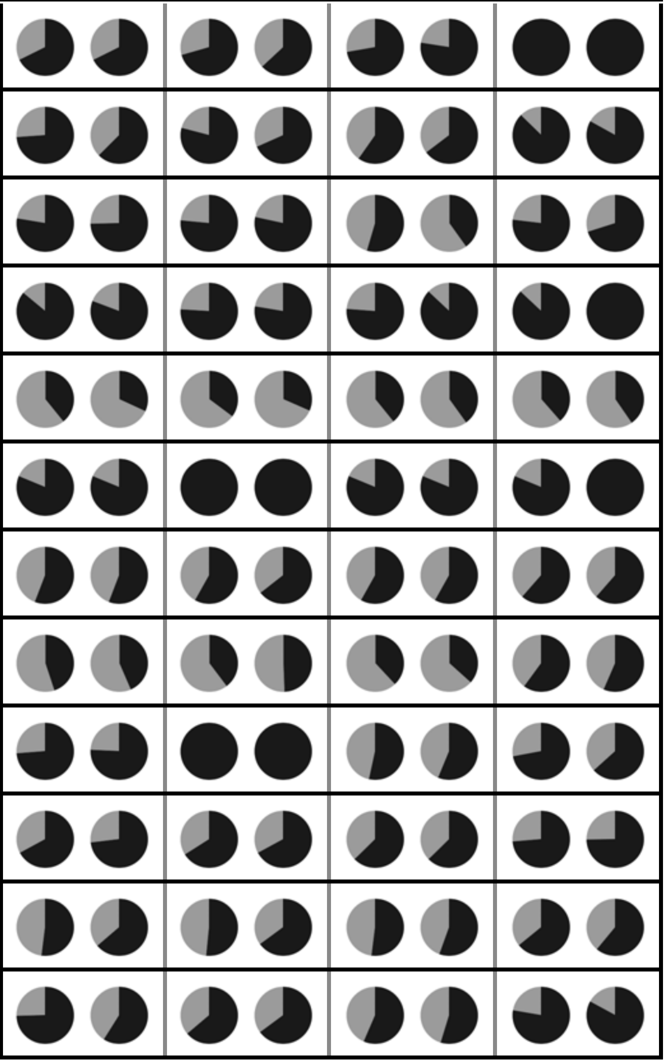

Hendrickson *et al.*

Protein	*Sg* n	*Sg* Coverage	*SgFn* n	*SgFn* Coverage	*SgPg* n	*SgPg* Coverage	*SgPgFn* n	*SgPgFn* Coverage	Description
**SGO_1689**	14	48.1%	13	48.4%	13	44.9%	9	29.0%	accC; acetyl-CoA carboxylase, biotin carboxylase
	14	45.8%	14	52.3%	15	51.0%	9	32.2%	
**SGO_1690**			3	27.1%	6	40.0%	4	34.3%	fabZ; beta-hydroxyacyl-(acyl-carrier-protein) dehydratase FabZ
			5	40.7%	7	46.4%			
**SGO_1691**	4	25.3%	8	42.0%	7	39.5%	6	27.8%	accB; acetyl-CoA carboxylase, biotin carboxyl carrier protein
	5	28.4%	9	47.5%	8	46.3%	6	39.5%	
**SGO_1692**	15	65.4%	25	74.1%	22	78.8%	17	63.7%	3-oxoacyl-[acyl-carrier-protein] synthase
	17	62.0%	27	77.3%	21	71.2%	16	61.2%	
**SGO_1693**	7	39.3%	11	55.7%	15	73.0%	9	50.0%	fabG; 3-oxoacyl-(acyl-carrier-protein) reductase
	11	47.5%	12	59.4%	13	63.5%	4	20.9%	
**SGO_1694**	10	45.4%	9	43.5%	11	58.5%	10	43.8%	fabD; malonyl CoA-acyl carrier protein transacylase
	13	55.9%	9	42.2%	13	60.8%	8	38.2%	
**SGO_1695**	12	52.2%	15	57.1%	17	60.8%	13	54.9%	enoyl-acyl carrier protein(ACP) reductase
	13	52.8%	12	52.2%	13	50.0%	10	45.4%	
**SGO_1698**	4	22.8%			6	27.2%			3-oxoacyl-[acyl-carrier-protein] synthase III
	7	32.1%			4	22.5%			
**SGO_1699**	4	48.3%	3	40.8%	4	47.5%			transcriptional regulator, MarR family
	5	48.3%	4	50.8%	4	48.3%	3	40.8%	
**SGO_1700**	7	33.8%	9	45.6%	7	30.4%	4	18.6%	enoyl-CoA hydratase/isomerase family protein
	6	25.5%	9	47.9%	6	30.4%			
**SGO_1701**	11	28.7%	12	40.8%	17	52.4%	11	31.6%	aspartate kinase
	14	37.3%	16	45.6%	19	55.9%	10	27.4%	
**SGO_1708**	16	62.9%	11	42.7%	14	65.5%	9	43.6%	amiF; Oligopeptide transport ATP-binding protein amiF
	20	74.6%	10	51.1%	13	65.1%	9	49.5%	

Sg Rep I Rep II | *SgFn* Rep I Rep II | *SgPg* Rep I Rep II | *SgPgFn* Rep I Rep II

Show detected proteins only
Show all proteins
Filter by category:
ABC Transporter

Proteins found: 1179

Dot Plots Dot Plots

Hendrickson *et al.*

Streptococcus gordonii

Protein	*Sg* n	*Sg* Coverage	*SgFn* n	*SgFn* Coverage	*SgPg* n	*SgPg* Coverage	*SgPgFn* n	*SgPgFn* Coverage	Description
**SGO_1709**	16	49.6%	9	34.1%	11	40.0%	12	42.5%	amiE; Oligopeptide transport ATP-binding protein
	17	51.8%	10	33.2%	11	40.0%	12	44.5%	
**SGO_1710**	6	16.2%			5	15.6%	4	13.0%	amiD; Oligopeptide transport system permease protein
	9	24.7%			4	15.3%	4	17.9%	
**SGO_1711**	19	31.1%	7	14.9%	11	23.1%	10	20.1%	hppB; Oligopeptide transport system permease
	22	38.0%	7	15.1%	14	30.1%	12	25.5%	
**SGO_1712**	36	53.2%	27	45.9%	31	51.3%	31	53.5%	hppA; oligopeptide-binding lipoprotein
	42	57.0%	21	45.6%	31	50.1%	27	49.2%	
**SGO_1713**	35	57.1%	29	54.2%	33	58.0%	28	54.4%	hppG; oligopeptide-binding lipoprotein
	38	58.5%	22	47.6%	30	52.6%	20	43.6%	
**SGO_1715**	32	61.7%	23	49.5%	31	62.4%	21	42.9%	hppH; oligopeptide-binding lipoprotein
	35	64.1%	23	48.5%	29	57.5%	23	47.9%	
**SGO_1716**	40	68.0%	24	47.2%	26	50.7%	21	46.1%	oligopeptide binding protein
	38	66.9%	18	38.6%	29	58.3%	17	37.8%	
**SGO_1717**	16	58.5%	4	16.3%	4	13.3%			pbp3; penicillin-binding protein 3
	16	59.5%	4	13.6%					
**SGO_1718**	16	36.4%	10	30.2%	16	51.5%	9	23.6%	sufB-1; FeS assembly protein SufB
	20	50.9%	14	36.8%	13	39.1%	11	26.4%	
**SGO_1719**	4	33.8%							SUF system FeS assembly protein, NifU family
	3	33.8%							
**SGO_1720**	4	15.1%			8	34.4%	4	19.3%	aminotransferase, class-V
	9	38.5%			6	25.6%	3	16.1%	
**SGO_1721**	9	28.8%	8	28.6%	10	31.2%	10	31.2%	sufD; FeS assembly protein SufD
	8	26.0%	8	28.6%	11	34.3%	6	23.3%	

Sg Rep I Rep II | *SgFn* Rep I Rep II | *SgPg* Rep I Rep II | *SgPgFn* Rep I Rep II

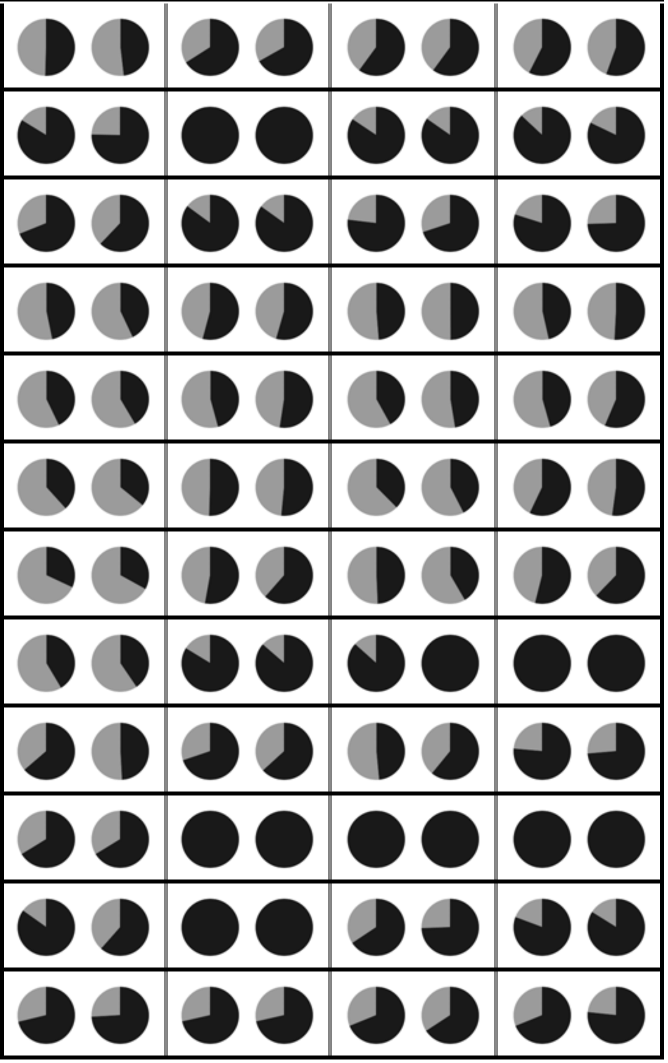

Show detected proteins only

Show all proteins

Filter by category:

ABC Transporter

Proteins found: 1179

Dot Plots Dot Plots

Hendrickson *et al.*

Streptococcus gordonii

Protein	*Sg* n	*Sg* Coverage	*SgFn* n	*SgFn* Coverage	*SgPg* n	*SgPg* Coverage	*SgPgFn* n	*SgPgFn* Coverage	Description
**SGO_1722**	9	34.8%	11	50.0%	14	56.3%	7	25.8%	sufC; FeS assembly ATPase SufC
	9	39.1%	5	25.4%	10	48.4%	5	19.5%	
**SGO_1724**	3	13.6%							mecA; Adapter protein mec
	6	32.6%	3	23.6%	4	24.8%			
**SGO_1725**	3	12.4%							bacA; bacitracin resistance protein
	3	12.4%							
**SGO_1726**	8	16.7%							hypothetical protein SGO_1726
	10	18.3%							
**SGO_1727**	16	44.3%	7	24.8%	8	28.4%	4	16.9%	amino acid ABC transporter, amino acid-binding/permease protein
	16	43.6%	7	23.8%	10	33.2%	4	16.7%	
**SGO_1728**	8	44.7%	6	45.1%	7	44.3%	7	33.3%	glnQ; glutamine ABC transporter ATP-binding protein
	9	47.6%	6	40.7%	6	40.7%	6	38.6%	
**SGO_1729**			5	68.1%	4	61.1%	3	50.0%	hypothetical protein SGO_1729
			4	61.1%	4	61.1%	4	61.1%	
**SGO_1730**	26	75.6%	25	71.5%	19	61.7%	13	51.9%	SPFH domain/Band 7 family
	32	78.3%	25	70.5%	21	63.4%	19	54.9%	
**SGO_1731**	3	23.6%	4	22.1%	6	38.2%	4	26.1%	DNA-binding response regulator
	3	18.6%	3	24.1%	8	49.7%	6	37.2%	
**SGO_1732**	6	19.1%							histidine kinase
	7	23.0%			3	9.6%			
**SGO_1735**	22	61.0%	21	58.5%	21	56.0%	18	50.7%	hypothetical protein SGO_1735
	23	64.6%	25	60.6%	21	56.0%	16	47.3%	
**SGO_1736**	4	33.1%	4	33.1%	4	33.1%	3	28.9%	alkaline shock protein
	4	33.1%	4	33.1%	4	33.1%			

Sg Rep I Rep II | *SgFn* Rep I Rep II | *SgPg* Rep I Rep II | *SgPgFn* Rep I Rep II

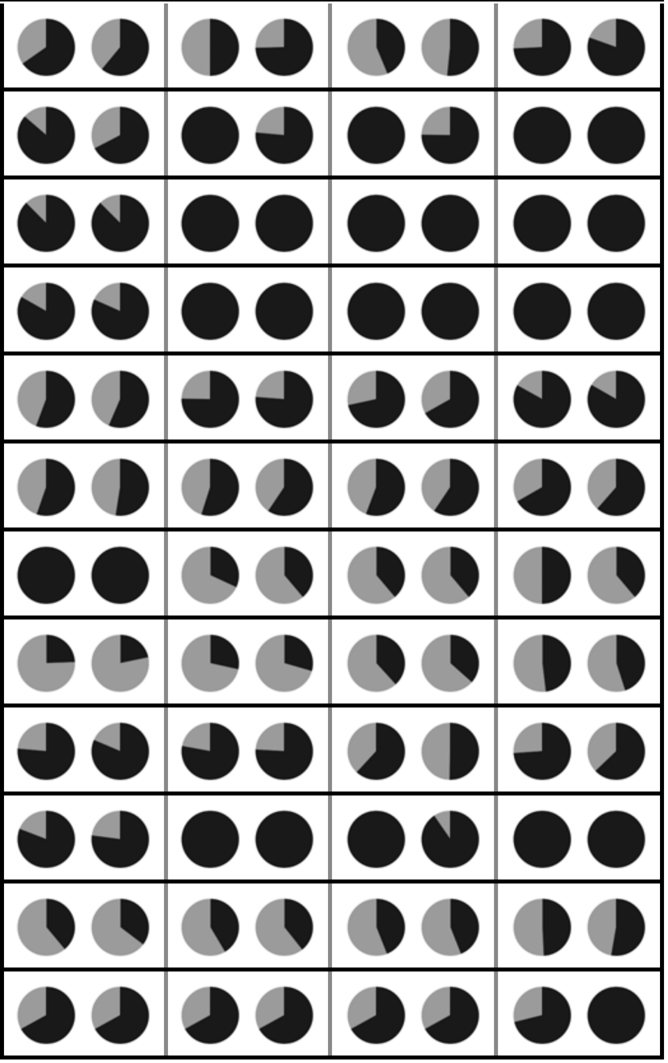

◉ Show detected proteins only
○ Show all proteins

☐ Filter by category:

ABC Transporter

Proteins found:
1179

Dot Plots | Dot Plots

Hendrickson *et al.*

Protein	*Sg* n	*Sg* Coverage	*SgFn* n	*SgFn* Coverage	*SgPg* n	*SgPg* Coverage	*SgPgFn* n	*SgPgFn* Coverage	Description
**SGO_1737**	3	38.7%							rpmB; ribosomal protein L28
	4	40.3%					4	40.3%	
**SGO_1738**	4	21.2%							hypothetical protein SGO_1738
	4	21.2%	3	14.0%					
**SGO_1739**	6	36.5%	3	27.7%					LytTr DNA-binding domain family
	7	36.5%							
**SGO_1744**	9	34.8%	7	25.8%					ABC transporter, ATP-binding protein SP0636
	8	34.2%							
**SGO_1745**	15	51.9%	18	54.9%	14	51.2%	16	54.9%	fba; fructose-1,6-bisphosphate aldolase, class II
	17	56.7%	22	63.8%	15	51.2%	16	54.9%	
**SGO_1747**					3	25.0%	3	27.2%	hypothetical protein SGO_1747
			3	27.2%	3	33.1%			
**SGO_1748**	9	25.0%			7	23.8%	6	21.0%	pyrG; CTP synthase
	11	24.2%	3	11.4%	12	36.5%	6	17.9%	
**SGO_1749**	7	36.6%	6	28.0%	8	43.6%	3	11.5%	manA; mannose-6-phosphate isomerase, class I
	9	42.7%	10	47.1%	7	36.6%	6	28.0%	
**SGO_1750**	11	21.4%							ABC-type multidrug/protein/lipid transport system, ATPase component
	13	25.1%							
**SGO_1751**	13	27.0%			3	6.1%			ABC-type multidrug/protein/lipid transport system, ATPase component
	13	28.2%			6	11.4%			
**SGO_1753**	3	22.8%							hypothetical protein SGO_1753
**SGO_1755**	8	47.2%	6	39.5%	11	67.6%	11	67.6%	scrK; fructokinase
	8	43.5%	7	47.2%	12	67.6%	9	57.5%	

Sg Rep I Rep II | *SgFn* Rep I Rep II | *SgPg* Rep I Rep II | *SgPgFn* Rep I Rep II

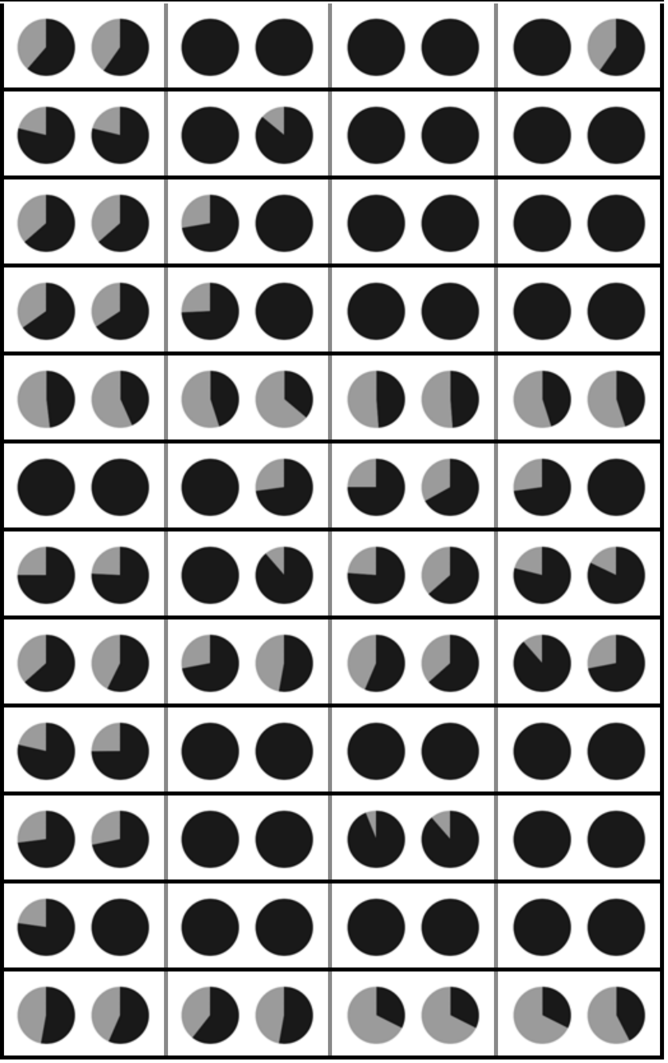

Show detected proteins only
Show all proteins
Filter by category:
ABC Transporter

Proteins found:
1179

Dot Plots Dot Plots

Hendrickson *et al.*

Protein	*Sg* n	*Sg* Coverage	*SgFn* n	*SgFn* Coverage	*SgPg* n	*SgPg* Coverage	*SgPgFn* n	*SgPgFn* Coverage	Description
**SGO_1756**									hypothetical protein SGO_1756
	6	49.4%							
**SGO_1757**	19	44.4%	13	34.3%	24	54.6%	14	39.3%	glmS; glucosamine--fructose-6-phosphate aminotransferase
	24	48.4%	12	35.0%	25	61.0%	17	47.6%	
**SGO_1760**									DNA-binding response regulator
			3	9.6%	4	14.3%			
**SGO_1761**	6	13.8%							histidine kinase
	10	18.9%							
**SGO_1763**	18	48.4%	17	46.1%	17	54.5%	15	53.9%	ABC transporter, substrate-binding protein SP0092
	20	56.6%	11	39.3%	19	56.4%	12	40.7%	
**SGO_1768**	6	13.8%	12	23.7%	3	7.9%			glycosyl hydrolase, family 38
	5	10.5%	13	26.6%	8	15.5%			
**SGO_1772**									integral membrane protein
	3	11.8%							
**SGO_1774**	5	27.5%	8	36.5%	6	30.4%	4	21.2%	alcohol dehydrogenase, zinc-containing
	8	40.6%	8	39.4%	6	29.0%	4	23.8%	
**SGO_1780**									hypothetical protein SGO_1780
	3	16.7%	3	14.3%					
**SGO_1784**	14	20.6%	20	28.6%	16	25.1%	12	20.9%	leuS; leucyl-tRNA synthetase
	18	25.1%	12	21.4%	16	26.9%	16	27.7%	
**SGO_1792**			3	12.8%					transcription regulator
	3	13.7%							
**SGO_1799**	19	42.5%	32	72.1%	26	63.3%	23	59.8%	endopeptidase O
	25	51.6%	27	65.1%	28	64.3%	16	41.7%	

◉ Show detected proteins only
○ Show all proteins

☐ Filter by category:
ABC Transporter

Proteins found:
1179

Dot Plots Dot Plots

Streptococcus gordonii

Protein	*Sg* n	*Sg* Coverage	*SgFn* n	*SgFn* Coverage	*SgPg* n	*SgPg* Coverage	*SgPgFn* n	*SgPgFn* Coverage	Description
**SGO_1800**	18	76.2%			10	56.1%	6	34.3%	troB; manganese ABC transporter, ATP-binding protein SP1648
	19	75.7%			10	53.1%	6	30.1%	
**SGO_1802**	26	75.2%	7	38.4%	21	70.6%	16	71.6%	Metal ABC transporter substrate-binding lipoprotein precursor
	32	85.8%	4	27.4%	18	66.8%	16	72.6%	
**SGO_1803**	4	37.4%	5	51.5%	5	51.5%	4	29.4%	tpx; thioredoxin peroxidase
	5	38.0%	5	51.5%	7	52.1%	3	28.8%	
**SGO_1804**			6	23.0%	10	40.1%			hutI; imidazolonepropionase
	4	18.1%	4	17.8%	10	37.1%			
**SGO_1805**	3	7.0%	3	7.1%	16	29.4%	8	16.3%	hutU; urocanate hydratase
					20	39.1%	3	5.6%	
**SGO_1806**					9	38.5%	5	20.7%	ftcD; glutamate formiminotransferase
					9	29.4%	4	16.4%	
**SGO_1807**					3	23.6%			serine cycle enzyme, putative
					3	23.6%			
**SGO_1808**					6	18.7%	3	7.9%	fhs-2; formate--tetrahydrofolate ligase
			4	9.5%	6	18.3%	5	13.5%	
**SGO_1811**	4	12.3%			5	15.4%			hutH; histidine ammonia-lyase
	5	15.4%	3	10.7%	7	22.2%	4	12.3%	
**SGO_1813**			3	12.8%					hutG; formimidoylglutamase
	3	11.6%							
**SGO_1814**	6	7.9%	6	7.7%					putative regulatory protein
	5	6.5%	5	8.8%					
**SGO_1816**	3	15.8%	6	33.0%					scaR; ScaR Manganese-dependent regulator of scaCBA
	4	23.7%	5	24.7%	5	27.9%			

Show detected proteins only
Show all proteins

Filter by category:
ABC Transporter

Proteins found:
1179

Dot Plots Dot Plots

Hendrickson *et al.*

Protein	*Sg* n	*Sg* Coverage	*SgFn* n	*SgFn* Coverage	*SgPg* n	*SgPg* Coverage	*SgPgFn* n	*SgPgFn* Coverage	Description
**SGO_1822**	22	33.7%	9	15.3%	10	19.5%	4	7.0%	relA; GTP diphosphokinase
	25	39.8%	10	15.6%	5	11.0%			
**SGO_1823**					4	28.3%			conserved hypothetical protein TIGR00046
					3	19.0%			
**SGO_1824**	6	34.6%	4	25.5%	4	25.5%	5	30.8%	prmA; ribosomal protein L11 methyltransferase
	8	40.6%	5	27.7%	6	36.2%	5	29.6%	
**SGO_1827**	5	35.3%	3	27.6%	3	27.6%			hypothetical protein SGO_1827
	4	27.6%			3	27.6%			
**SGO_1828**	6	19.7%	5	15.4%	3	12.6%			ATPase, AAA family
	3	12.8%	3	10.4%	4	15.6%	3	11.4%	
**SGO_1834**	5	27.6%	11	69.9%	6	43.4%	8	54.1%	hypothetical protein SGO_1834
	5	28.1%	11	73.5%	7	52.6%	8	55.6%	
**SGO_1835**			3	29.9%	3	29.9%			hypothetical protein SGO_1835
	3	29.9%			3	29.9%	3	47.8%	
**SGO_1843**	11	33.4%	14	49.4%	13	38.0%	10	31.2%	pepS; aminopeptidase PepS
	12	40.0%	11	43.8%	13	38.0%	10	29.5%	
**SGO_1844**	5	7.9%	6	10.5%	10	18.9%	3	6.5%	cbxX/cfqX family protein
	12	20.0%	9	16.1%	12	18.6%	5	10.0%	
**SGO_1847**	13	12.5%	10	8.7%	10	9.4%			polC; DNA polymerase III, alpha subunit, Gram-positive type
	15	14.6%	11	12.0%	14	13.9%	6	4.8%	
**SGO_1848**	9	47.8%			4	23.9%	5	22.2%	lipoprotein, putative
	10	48.3%			4	23.9%	4	17.8%	
**SGO_1849**	8	68.4%							hypothetical protein SGO_1849
	7	63.2%							

Sg: Rep I, Rep II | SgFn: Rep I, Rep II | SgPg: Rep I, Rep II | SgPgFn: Rep I, Rep II

◉ Show detected proteins only
○ Show all proteins
☐ Filter by category:
ABC Transporter

Proteins found: 1179

Dot Plots Dot Plots

Hendrickson *et al.*

Protein	*Sg* n	*Sg* Coverage	*SgFn* n	*SgFn* Coverage	*SgPg* n	*SgPg* Coverage	*SgPgFn* n	*SgPgFn* Coverage	Description
**SGO_1851**	25	54.5%	21	45.9%	25	54.7%	19	37.2%	proS; prolyl-tRNA synthetase
	28	55.8%	23	53.7%	28	60.2%	20	45.0%	
**SGO_1852**	9	27.6%			5	17.7%	4	16.8%	membrane-associated zinc metalloprotease, putative
	10	29.7%			5	18.5%			
**SGO_1854**	12	57.0%	6	34.5%	6	32.1%	6	32.1%	uppS; undecaprenyl diphosphate synthase
	12	50.6%	6	32.1%	7	39.4%	6	28.5%	
**SGO_1855**	4	47.3%			3	47.3%			preprotein translocase, YajC subunit
	4	47.3%			3	47.3%			
**SGO_1856**	17	35.8%	37	69.2%	12	22.9%	8	18.2%	ATP-dependent proteinase ATP-binding chain
	19	34.7%	26	54.8%	12	26.7%	4	9.8%	
**SGO_1857**	4	11.3%			3	8.5%			PTS system, IIABC components
	7	15.3%							
**SGO_1858**			4	17.8%	5	17.6%			beta-fructofuranosidase/sucrose 6 phoshate hydrolase
	3	9.3%	5	16.7%	5	19.6%			
**SGO_1860**	10	47.4%	17	61.8%	11	48.1%	7	41.4%	5'-nucleotidase, lipoprotein e(P4) family
	14	55.8%	13	55.8%	9	47.0%	5	29.5%	
**SGO_1861**	3	23.4%			4	40.1%			nusB; transcription antitermination factor NusB
					4	40.1%	3	23.4%	
**SGO_1862**	3	40.3%	4	46.5%	3	38.8%	3	40.3%	alkaline shock protein
	3	40.3%	5	63.6%	3	38.8%	4	47.3%	
**SGO_1863**	8	58.1%	7	61.3%	7	50.5%	6	41.4%	efp; Elongation factor P (EF-P)
	8	58.1%	5	46.2%	8	66.1%	6	41.4%	
**SGO_1864**	10	46.2%	13	58.4%	10	43.1%	6	25.2%	X-Pro aminopeptidase
	13	51.6%	13	63.7%	10	52.7%	6	25.2%	

Sg Rep I Rep II | SgFn Rep I Rep II | SgPg Rep I Rep II | SgPgFn Rep I Rep II

Show detected proteins only
Show all proteins
Filter by category:
ABC Transporter

Proteins found: 1179

Dot Plots Dot Plots

Hendrickson *et al.*

Streptococcus gordonii

Hackett Laboratory UW

Protein	*Sg* n	*Sg* Coverage	*SgFn* n	*SgFn* Coverage	*SgPg* n	*SgPg* Coverage	*SgPgFn* n	*SgPgFn* Coverage	Description
**SGO_1865**	26	35.1%	15	28.7%	17	29.2%	8	16.5%	uvrA; excinuclease ABC, A subunit
	30	43.4%	14	28.2%	20	35.9%	8	16.3%	
**SGO_1866**					3	11.1%	3	8.3%	corA; magnesium and cobalt transporter
**SGO_1867**	5	23.2%	3	15.6%	4	22.8%	3	20.1%	hypothetical protein SGO_1867
	5	23.2%	4	22.8%	4	22.8%	4	22.8%	
**SGO_1869**	9	29.8%							hypothetical protein SGO_1869
	13	46.3%							
**SGO_1870**	14	28.8%			4	8.9%			hypothetical protein SGO_1870
	16	34.4%			3	6.3%			
**SGO_1871**	11	34.0%							lipoprotein, putative
	11	34.3%							
**SGO_1872**	6	25.1%			5	21.0%			hypothetical protein SGO_1872
	11	40.2%							
**SGO_1878**	3	20.6%							transcriptional regulator, TetR family domain protein
	3	17.2%	4	29.9%					
**SGO_1879**	7	44.3%	4	43.0%	5	41.8%	3	31.6%	rpsR; ribosomal protein S18
	7	44.3%	7	59.5%	6	41.8%	5	44.3%	
**SGO_1880**	8	79.8%	7	69.3%	8	79.8%	8	79.8%	ssb-1; single-strand binding protein
	8	79.8%	6	57.1%	8	79.8%	7	73.0%	
**SGO_1881**	7	74.0%	8	76.0%	8	74.0%	8	78.1%	rpsF; ribosomal protein S6
	8	82.3%	9	78.1%	7	72.9%	8	74.0%	
**SGO_1882**	3	19.8%	4	20.3%	4	20.3%	3	19.8%	folE; GTP cyclohydrolase I
	5	46.0%	5	40.1%	4	20.3%	4	25.1%	

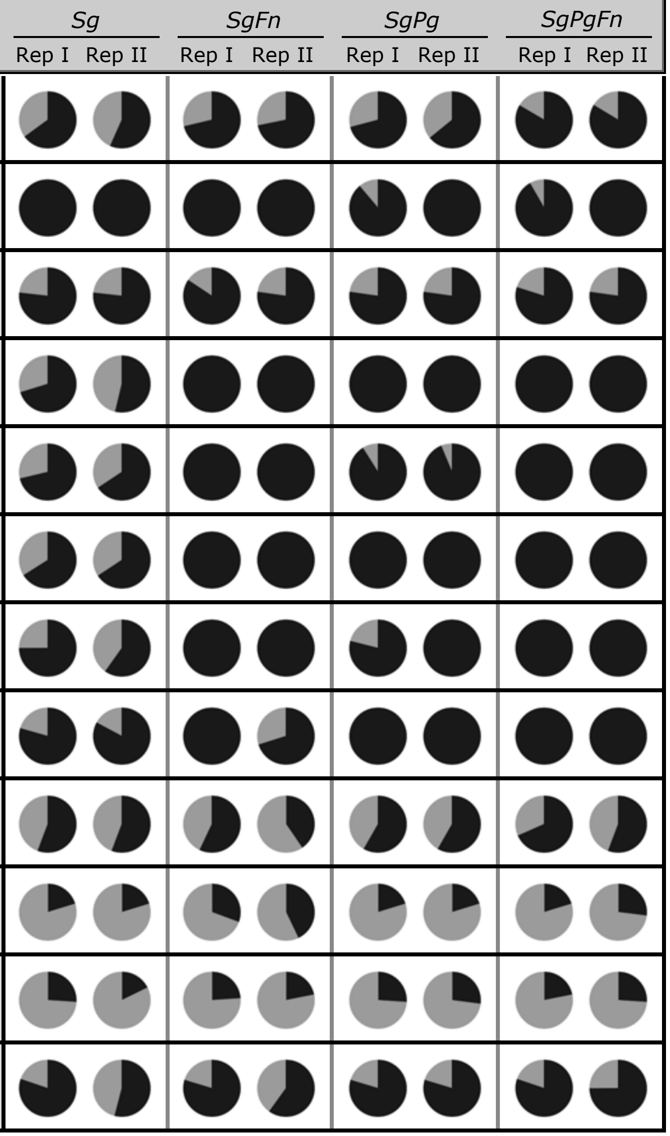

Show detected proteins only
Show all proteins
Filter by category:
ABC Transporter

Proteins found: 1179

Dot Plots Dot Plots

Hendrickson *et al.*

Protein	Sg n	Sg Coverage	SgFn n	SgFn Coverage	SgPg n	SgPg Coverage	SgPgFn n	SgPgFn Coverage	Description
**SGO_1884**	6	30.6%							hypothetical protein SGO_1884
	8	43.3%							
**SGO_1885**	30	60.7%	40	73.5%	29	63.5%	25	56.7%	groL; 60 kDa chaperonin/groEL protein
	36	65.2%	35	75.4%	34	70.6%	24	61.1%	
**SGO_1886**	4	54.8%	4	54.8%	4	54.8%	3	41.9%	groES; chaperonin, 10 kDa
	4	54.8%	4	51.6%	3	41.9%	3	41.9%	
**SGO_1887**	3	13.9%							ABC transporter, ATP-binding protein
	7	29.6%							
**SGO_1888**	5	11.6%							ABC transporter, permease protein
	7	12.8%							
**SGO_1890**	3	11.2%							PTS system, fructose(mannose)-specific IID
	3	11.2%							
**SGO_1891**	3	13.8%							PTS system, IIC component
	4	24.7%							
**SGO_1892**	5	52.4%	7	56.1%	5	52.4%	5	52.4%	PTS system, fructose(mannose)-specific IIB
	6	56.1%	6	59.8%	5	52.4%	4	42.7%	
**SGO_1894**	3	12.4%							sugar-binding cell envelope protein
	7	27.4%							
**SGO_1895**	3	15.0%							response regulator
	3	14.5%							
**SGO_1897**	3	12.9%							rbsB; ribose ABC transporter (ribose-binding component) CAC1453
	6	22.7%							
**SGO_1898**	9	40.6%	15	71.3%	13	63.4%	11	49.9%	glutamyl aminopeptidase
	10	49.3%	17	70.1%	13	63.4%	9	40.6%	

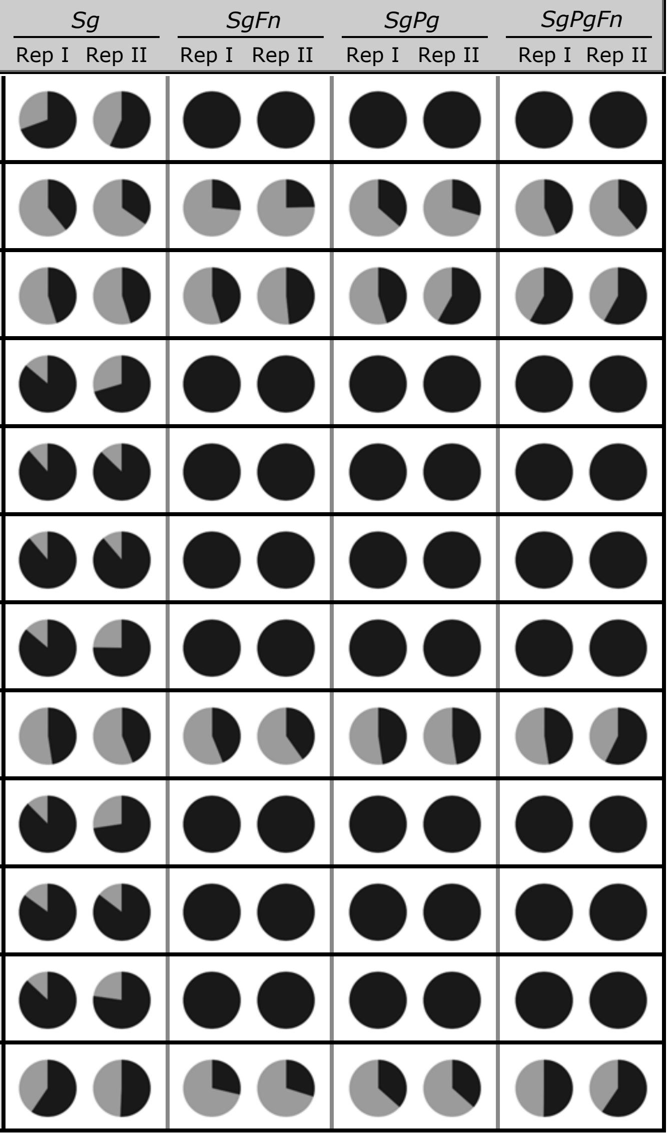

Show detected proteins only
Show all proteins
Filter by category:
ABC Transporter

Proteins found:
1179

Dot Plots Dot Plots

Streptococcus gordonii

Protein	*Sg* n	*Sg* Coverage	*SgFn* n	*SgFn* Coverage	*SgPg* n	*SgPg* Coverage	*SgPgFn* n	*SgPgFn* Coverage	Description
**SGO_1899**									hypothetical protein SGO_1899
	3	30.5%							
**SGO_1900**					3	36.2%			thioredoxin family protein
	4	48.6%			3	43.8%			
**SGO_1901**	6	36.1%	4	22.6%	7	44.7%	5	31.7%	tRNA binding domain
	5	31.7%	5	26.4%	7	36.1%	5	30.3%	
**SGO_1902**	5	57.3%			6	59.5%	6	59.5%	ssb; single-stranded DNA-binding protein
	6	64.1%			6	59.5%	6	59.5%	
**SGO_1903**	17	39.1%	25	50.0%	20	46.4%	15	44.2%	ATP-dependent Zn protease
	19	39.3%	20	49.0%	23	52.8%	13	39.5%	
**SGO_1907**									sensor protein
	3	15.7%							
**SGO_1912**	3	20.4%			5	29.3%			Bifunctional folate synthesis protein
	4	27.4%	4	25.6%	3	20.7%			
**SGO_1914**			4	15.0%	3	14.6%			folP; dihydropteroate synthase
	4	15.6%	5	21.2%	4	18.1%	3	11.8%	
**SGO_1916**	19	61.4%	13	41.4%	19	61.4%	14	48.0%	ackA; acetate kinase
	19	60.6%	13	47.7%	21	68.7%	12	46.7%	
**SGO_1917**	3	17.7%	3	18.6%	6	37.5%	5	29.3%	hypothetical protein SGO_1917
	4	20.5%	6	34.4%	6	37.5%	3	18.9%	
**SGO_1923**	5	17.2%							comYB; competence protein
**SGO_1924**	17	54.9%			11	41.1%	7	23.5%	comYA; competence protein comYA
	16	54.9%			9	34.5%	5	15.4%	

Hendrickson *et al.*

Coverage

Streptococcus gordonii

Hackett Laboratory UW

Summary Table | SgFn vs Sg | SgPg vs Sg | SgPgFn vs Sg | SgPg vs SgFn | SgPgFn vs SgFn | SgPgFn vs SgPg | Coverage

Protein	*Sg*		*SgFn*		*SgPg*		*SgPgFn*		Description
	n	Coverage	n	Coverage	n	Coverage	n	Coverage	
SGO_1926	55	49.6%	51	52.1%	69	57.5%	55	48.3%	rpoC; DNA-directed RNA polymerase, beta chain
	69	57.0%	56	57.9%	73	62.6%	55	50.3%	
SGO_1927	50	53.6%	44	55.0%	61	63.4%	44	52.1%	rpoB; DNA-directed RNA polymerase, beta subunit
	53	54.5%	37	49.8%	57	63.7%	43	51.9%	
SGO_1928	27	36.4%			9	17.0%	5	10.4%	pbp1b; penicillin-binding protein 1B
	30	42.4%			12	24.0%			
SGO_1929	11	37.6%	17	56.2%	19	56.0%	13	41.6%	tyrS; tyrosyl-tRNA synthetase
	16	40.2%	16	57.4%	21	60.3%	13	46.7%	
SGO_1930	4	15.2%			3	12.1%			rrmA; Ribosomal RNA large subunit methyltransferase A
	6	24.5%			3	12.1%			
SGO_1931					3	30.9%			lipoprotein, putative
SGO_1934	11	26.9%							copper-translocating P-type ATPase
	14	32.5%					3	5.4%	
SGO_1935	5	49.0%							negative transcriptional regulator, CopY
	3	27.3%							
SGO_1936	25	58.2%	8	24.2%	18	50.8%	5	16.2%	adcA; metal-binding (Mn) permease precursor, lipoprotein
	35	65.6%	8	24.8%	15	41.2%	6	18.2%	
SGO_1938	6	43.2%			3	22.9%			adcC; ATP-binding protein, Mn
	8	48.3%			3	22.9%			
SGO_1939									adcR; repressor protein adcR
					4	30.6%			
SGO_1957	8	30.8%	4	12.0%					hypothetical protein SGO_1957
	12	41.3%	6	19.5%	3	13.4%			

Rep I / Rep II columns: *Sg*, *SgFn*, *SgPg*, *SgPgFn*

Show detected proteins only
Show all proteins
Filter by category:
ABC Transporter

Proteins found: 1179

Dot Plots Dot Plots

Hendrickson *et al.*

Protein	*Sg* n	*Sg* Coverage	*SgFn* n	*SgFn* Coverage	*SgPg* n	*SgPg* Coverage	*SgPgFn* n	*SgPgFn* Coverage	Description	*Sg* Rep I	*Sg* Rep II	*SgFn* Rep I	*SgFn* Rep II	*SgPg* Rep I	*SgPg* Rep II	*SgPgFn* Rep I	*SgPgFn* Rep II
**SGO_1958**	5	47.7%	5	47.7%	6	48.4%	6	54.7%	rplQ; ribosomal protein L17								
	7	54.7%	5	51.6%	6	48.4%	5	47.7%									
**SGO_1959**	17	50.3%	20	59.0%	20	68.3%	15	54.8%	rpoA; DNA-directed RNA polymerase, alpha subunit								
	19	55.8%	15	56.7%	19	62.2%	18	63.5%									
**SGO_1960**	3	22.8%	4	33.9%	5	50.4%	4	33.9%	rpsK; ribosomal protein S11								
	4	33.1%	5	47.2%	4	33.9%	3	22.8%									
**SGO_1961**	16	69.4%	11	62.8%	17	69.4%	13	59.5%	rpsM; ribosomal protein S13p/S18e								
	16	69.4%	8	47.9%	14	69.4%	14	69.4%									
**SGO_1962**					3	60.5%	3	60.5%	rpmJ; ribosomal protein L36								
					3	60.5%											
**SGO_1964**	10	64.6%	13	62.7%	13	68.9%	12	67.9%	adk; Adenylate kinase (ATP-AMP transphosphorylase)								
	11	64.6%	11	62.3%	14	79.2%	9	58.0%									
**SGO_1965**	8	20.2%			6	15.4%	3	8.5%	Preprotein translocase secY subunit								
	9	20.6%			5	13.8%	3	8.5%									
**SGO_1966**	6	49.3%	7	50.7%	7	50.7%	12	56.2%	rplO; ribosomal protein L15								
	5	28.8%	5	47.3%	8	50.7%	9	50.7%									
**SGO_1967**	4	54.1%	3	54.1%	4	54.1%	5	54.1%	50S ribosomal protein L30 -related protein								
	4	54.1%	3	35.1%	4	54.1%	4	54.1%									
**SGO_1968**	11	65.9%	8	60.4%	10	71.3%	12	72.0%	rpsE; ribosomal protein S5								
	12	67.1%	9	76.2%	11	71.3%	11	72.0%									
**SGO_1969**	4	39.8%	4	39.8%	4	39.8%	5	47.5%	rplR; ribosomal protein L18								
	5	50.0%	4	39.8%	4	39.8%	4	39.8%									
**SGO_1970**	9	59.6%	12	71.9%	9	59.6%	9	59.6%	BL10; 50S ribosomal protein L6								
	10	65.7%	6	49.4%	11	66.3%	8	59.6%									

- (•) Show detected proteins only
- ( ) Show all proteins
- [ ] Filter by category: ABC Transporter

Proteins found: 1179

Dot Plots Dot Plots

Streptococcus gordonii

Hackett Laboratory UW

Protein	*Sg* n	*Sg* Coverage	*SgFn* n	*SgFn* Coverage	*SgPg* n	*SgPg* Coverage	*SgPgFn* n	*SgPgFn* Coverage	Description
SGO_1971	12	59.8%	13	59.8%	12	58.3%	12	58.3%	rpsH; ribosomal protein S8
	12	59.8%	10	50.0%	13	59.8%	11	58.3%	
SGO_1972									rpsN; ribosomal protein S14p/S29e
	3	49.2%							
SGO_1973	18	83.9%	15	70.6%	16	80.6%	14	77.2%	BL6; 50S ribosomal protein L5
	17	81.7%	15	88.3%	17	78.3%	15	75.0%	
SGO_1974	8	47.5%	9	63.4%	9	52.5%	8	47.5%	rplX; ribosomal protein L24
	9	63.4%	6	54.5%	9	52.5%	8	47.5%	
SGO_1975	4	27.0%	4	22.1%	5	34.4%	5	32.0%	rplN; ribosomal protein L14
	6	36.1%	4	22.1%	4	27.0%	5	32.0%	
SGO_1976	4	25.6%	3	25.6%	5	38.4%	5	38.4%	BS16; 30S ribosomal protein
	4	25.6%	6	53.5%	6	58.1%	5	38.4%	
SGO_1977	4	42.6%	4	50.0%	5	45.6%	4	57.4%	rpmC; ribosomal protein L29
	8	73.5%			5	58.8%	3	50.0%	
SGO_1978	4	33.6%	6	42.3%	6	44.5%	4	33.6%	rplP; ribosomal protein L16
	8	52.6%	4	40.1%	6	44.5%	4	37.2%	
SGO_1979	12	55.8%	14	58.1%	11	53.9%	10	49.8%	rpsC; ribosomal protein S3
	14	59.4%	13	69.6%	10	53.5%	9	53.9%	
SGO_1980	8	58.8%	6	50.9%	7	57.0%	6	54.4%	rplV; ribosomal protein L22
	9	69.3%	5	49.1%	8	61.4%	7	60.5%	
SGO_1981	8	57.0%	8	48.4%	8	57.0%	6	55.9%	rpsS; ribosomal protein S19
	9	58.1%	10	64.5%	9	57.0%	8	57.0%	
SGO_1982	10	47.3%	10	52.0%	11	56.0%	10	50.2%	rplB; ribosomal protein L2
	12	52.7%	11	54.2%	11	45.5%	12	56.0%	

Show detected proteins only
Show all proteins
Filter by category:
ABC Transporter

Proteins found:
1179

Dot Plots Dot Plots

Coverage

Streptococcus gordonii

Hackett Laboratory UW

Summary Table | SgFn vs Sg | SgPg vs Sg | SgPgFn vs Sg | SgPg vs SgFn | SgPgFn vs SgFn | SgPgFn vs SgPg | Coverage

Protein	*Sg* n	*Sg* Coverage	*SgFn* n	*SgFn* Coverage	*SgPg* n	*SgPg* Coverage	*SgPgFn* n	*SgPgFn* Coverage	Description
**SGO_1983**	7	69.4%	6	59.2%	8	69.4%	7	69.4%	rplW; ribosomal protein L23
	9	84.7%	6	68.4%	7	69.4%	8	76.5%	
**SGO_1984**	7	37.7%	9	47.8%	8	51.7%	7	51.7%	rplD; ribosomal protein L4/L1 family
	8	41.5%	9	49.8%	8	51.7%	7	51.7%	
**SGO_1985**	8	45.2%	11	63.9%	9	53.8%	10	59.6%	rplC; ribosomal protein L3
	10	48.1%	10	64.9%	10	56.7%	10	59.6%	
**SGO_1986**	5	38.2%	5	38.2%	5	38.2%	6	45.1%	rpsJ; ribosomal protein S10
	5	38.2%	5	45.1%	5	38.2%	6	45.1%	
**SGO_1988**	6	26.6%							hydrolase, haloacid dehalogenase-like family
	6	25.7%	5	20.6%					
**SGO_1989**	25	67.0%	24	61.9%	31	69.5%	26	67.0%	purA; adenylosuccinate synthetase
	26	67.2%	25	63.3%	29	65.8%	26	66.7%	
**SGO_1990**	3	6.4%	10	23.7%	7	15.3%	4	8.7%	glutamate--cysteine ligase, putative/amino acid ligase, putative
	7	10.9%	12	26.4%	8	19.8%			
**SGO_1991**	3	12.8%	6	23.1%	3	16.2%			hslO; 33 kDa chaperonin /Heat shock protein 33-like protein
	8	32.4%	5	19.7%	4	19.7%			
**SGO_1993**	10	49.8%	6	35.6%	9	49.8%	3	22.4%	possible transcriptional regulator
	14	70.3%	6	45.1%	8	49.8%	5	29.7%	
**SGO_1994**	3	25.9%							lipoprotein, putative
**SGO_1995**	4	16.0%	3	11.8%			3	13.4%	MutT/nudix family protein
	4	16.0%	4	22.1%					
**SGO_1998**	19	30.2%	13	20.6%	21	32.3%	10	17.2%	clpB; ATP-dependent Clp proteinase, ATP-binding chain
	24	32.6%	15	24.2%	23	34.7%	8	13.6%	

Sg Rep I Rep II | *SgFn* Rep I Rep II | *SgPg* Rep I Rep II | *SgPgFn* Rep I Rep II

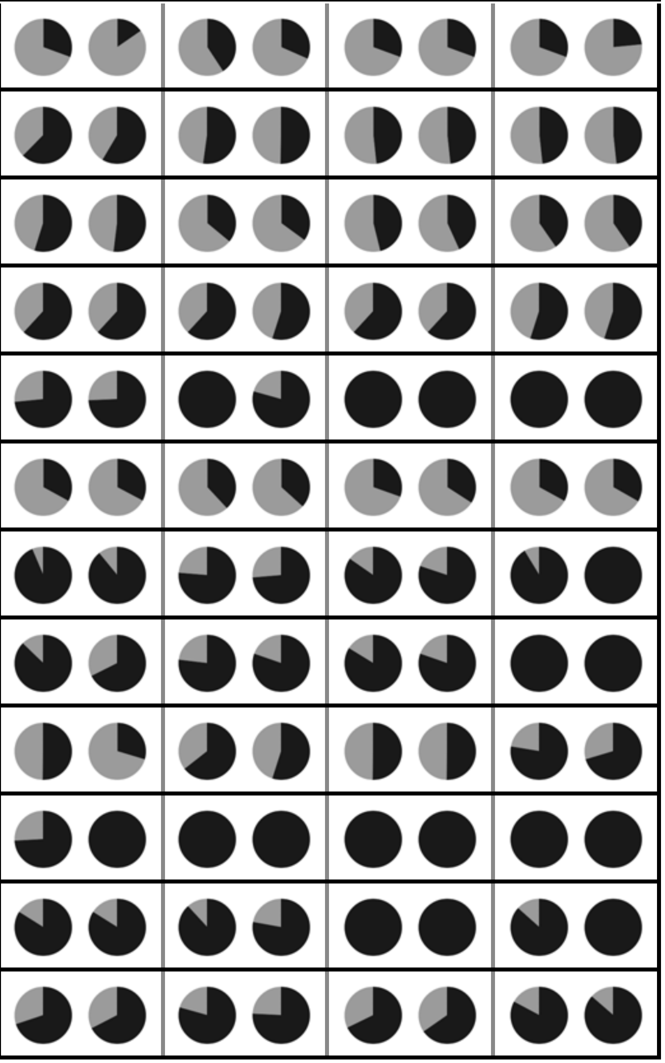

Show detected proteins only
Show all proteins
Filter by category:
ABC Transporter

Proteins found:
1179

Dot Plots Dot Plots

Hendrickson *et al.*

Protein	*Sg* n	*Sg* Coverage	*SgFn* n	*SgFn* Coverage	*SgPg* n	*SgPg* Coverage	*SgPgFn* n	*SgPgFn* Coverage	Description
**SGO_2000**	16	67.4%	17	68.6%	20	79.8%	16	66.9%	tsf; translation elongation factor Ts
	16	67.4%	19	78.4%	18	78.1%	15	61.7%	
**SGO_2001**	17	63.8%	23	69.2%	19	65.8%	19	66.9%	rpsB; ribosomal protein S2
	19	68.5%	23	80.8%	18	63.1%	20	69.2%	
**SGO_2004**	20	41.9%	5	13.1%					LPXTG cell wall surface protein
	25	41.3%	6	14.3%					
**SGO_2005**	46	19.3%			5	1.8%	9	3.6%	LPXTG cell wall surface protein
	54	21.0%	4	1.9%	5	2.3%	5	1.9%	
**SGO_2006**	3	27.3%							thioredoxin signature protein
	3	28.9%							
**SGO_2007**	7	47.8%	8	56.7%	6	43.3%	5	36.5%	nusG; transcription termination/antitermination factor NusG
	8	53.4%	7	48.9%	7	55.6%	7	47.8%	
**SGO_2008**	3	31.0%							preprotein translocase secE component -related protein
	3	31.0%							
**SGO_2010**	17	33.1%	6	17.3%					pbp2a; penicillin-binding protein 2A
	17	33.2%	4	8.4%					
**SGO_2013**	35	48.2%	5	6.0%					putative N-acetylmuramidase/lysin
	40	53.4%	5	6.6%					
**SGO_2015**									possible polysaccharide transport protein
	3	6.4%							
**SGO_2016**									nucleotide sugar dehydratase, putative
	4	10.5%							
**SGO_2017**					3	29.6%			nucleotidyl transferase, putative
	5	41.3%							

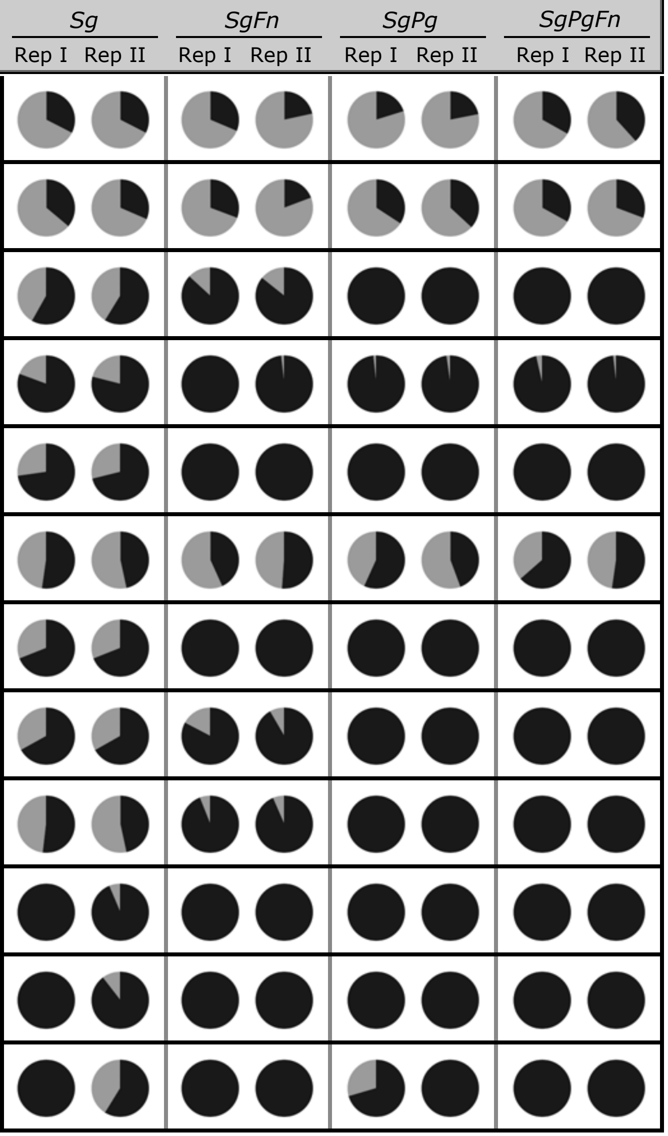

Hendrickson *et al.*

Protein	*Sg* n	*Sg* Coverage	*SgFn* n	*SgFn* Coverage	*SgPg* n	*SgPg* Coverage	*SgPgFn* n	*SgPgFn* Coverage	Description
**SGO_2019**	8	34.7%			6	22.6%			licD; licD3 protein
	9	38.2%			4	18.8%			
**SGO_2020**									glycosyltransferase
	4	16.0%							
**SGO_2021**	7	34.9%							Extracellular polysaccharide glycosyltransferase
	11	45.5%							
**SGO_2022**									UDP-glucose 4-epimerase BH3715
	4	17.7%							
**SGO_2023**	7	33.3%							galactosyltransferase
	6	33.3%							
**SGO_2024**	19	32.3%			10	18.9%	3	6.5%	Extracellular polysaccharide biosynthesis
	24	38.8%			8	14.9%	4	10.0%	
**SGO_2025**	13	60.2%			10	43.7%	3	16.9%	wze; putative autophosphorylating protein tyrosine kinase
	12	54.5%			9	44.2%	5	28.6%	
**SGO_2026**	5	31.6%			6	37.7%			wzd; polysaccharide export protein, MPA1 family
	6	31.6%			5	31.6%			
**SGO_2027**									wzh; wzh/Putative phosphotyrosine-protein phosphatase
	5	12.3%							
**SGO_2028**	12	29.8%							wzg; transcriptional regulator
	13	31.5%			3	8.6%			
**SGO_2033**	22	40.2%	23	39.0%	14	33.4%	7	17.7%	nrdD; ribonucleoside-triphosphate reductase
	31	48.2%	16	30.4%	14	31.9%	9	21.1%	
**SGO_2034**	8	14.0%	3	7.1%	4	7.6%			hypothetical protein SGO_2034
	11	19.3%							

Hendrickson *et al.*

Protein	*Sg* n	*Sg* Coverage	*SgFn* n	*SgFn* Coverage	*SgPg* n	*SgPg* Coverage	*SgPgFn* n	*SgPgFn* Coverage	Description
**SGO_2037**	8	21.0%	5	11.4%					cardiolipin synthase
	9	22.4%	6	14.0%					
**SGO_2039**	5	16.7%							dihydrofolate synthase
	6	18.7%			3	8.7%			
**SGO_2041**	4	33.8%			6	43.9%	3	23.0%	conserved hypothetical protein TIGR00250
	5	38.1%			4	33.8%			
**SGO_2042**	5	67.0%	5	68.2%	3	53.4%	4	63.6%	Bacterial protein of unknown function (DUF965) superfamily
	4	63.6%	4	63.6%	3	53.4%	4	63.6%	
**SGO_2043**	8	58.9%							lipoprotein, putative
	7	44.7%			3	21.6%			
**SGO_2045**	18	52.2%	16	49.3%	18	53.3%	17	51.4%	recA; recA protein
	22	54.8%	13	41.0%	19	55.6%	17	54.3%	
**SGO_2046**	7	19.9%	5	14.4%	4	10.0%	4	8.6%	cinA; competence induced protein
	5	12.2%	5	10.0%	4	9.6%	4	9.8%	
**SGO_2049**									tagI; DNA-3-methyladenine glycosylase I
	3	29.3%							
**SGO_2050**	5	37.2%			3	25.5%			ruvA; Holliday junction DNA helicase RuvA
	3	17.3%	3	23.5%	4	26.0%			
**SGO_2052**									hypothetical protein SGO_2052
	4	25.4%							
**SGO_2053**	9	20.2%	9	23.5%	5	13.8%	5	13.3%	DNA mismatch repair protein hexB
	12	28.4%	6	14.4%	10	27.5%	7	17.3%	
**SGO_2056**	15	20.2%	13	21.3%	11	21.4%	5	8.0%	mutS; DNA mismatch repair protein MutS
	17	28.1%	18	31.5%	14	28.6%	10	22.6%	

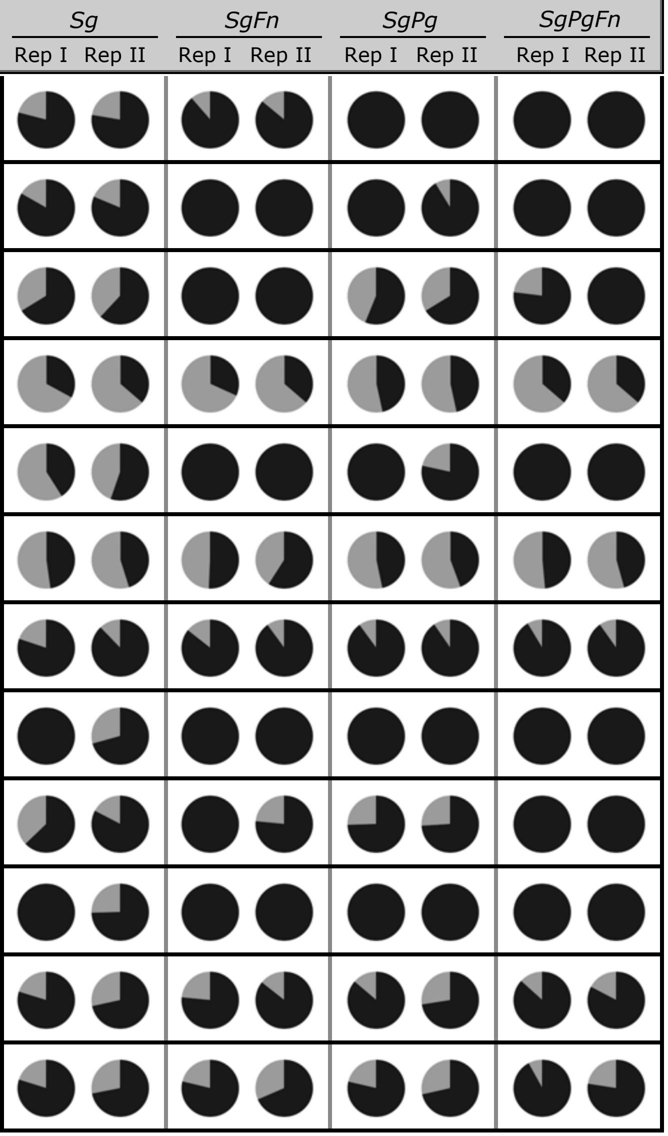

Show detected proteins only
Show all proteins
Filter by category:
ABC Transporter

Proteins found: 1179

Dot Plots Dot Plots

Protein	*Sg* n	*Sg* Coverage	*SgFn* n	*SgFn* Coverage	*SgPg* n	*SgPg* Coverage	*SgPgFn* n	*SgPgFn* Coverage	Description
SGO_2057	7	59.3%							argR; arginine repressor
	6	49.0%							
SGO_2058	31	59.8%	39	69.4%	37	66.2%	25	56.6%	argS; arginyl-tRNA synthetase
	37	65.7%	36	70.5%	36	64.1%	24	52.0%	
SGO_2059	5	19.0%							hypothetical protein SGO_2059
SGO_2060	15	31.5%	12	28.1%	21	43.0%	8	16.6%	aspS-1; aspartyl-tRNA synthetase
	22	42.0%	15	37.0%	19	39.4%	8	19.7%	
SGO_2062	16	45.8%	19	63.1%	21	66.0%	14	37.1%	hisS; histidyl-tRNA synthetase
	17	48.6%	18	55.2%	20	62.4%	14	40.4%	
SGO_2064	16	40.0%	19	48.8%	22	54.0%	15	41.9%	ilvD; dihydroxy-acid dehydratase
	18	40.3%	18	47.7%	24	60.4%	14	40.3%	
SGO_2065									rpmF; ribosomal protein L32
SGO_2066	3	34.7%	5	65.3%	4	44.9%	3	34.7%	rpmG; ribosomal protein L33
	4	53.1%			3	34.7%	4	44.9%	
SGO_2070	7	31.4%	7	37.1%	9	48.8%	7	34.8%	hypothetical protein SGO_2070
	9	40.1%	3	15.7%	8	42.8%	3	12.4%	
SGO_2073							3	13.0%	FtsK/SpoIIIE family subfamily, putative
SGO_2080	7	29.7%			4	20.6%			transcription activator plcR
	6	29.7%			5	22.9%			
SGO_2081	10	47.4%							lipoprotein, putative
	15	60.9%			3	24.3%			

Sg Rep I Rep II | *SgFn* Rep I Rep II | *SgPg* Rep I Rep II | *SgPgFn* Rep I Rep II

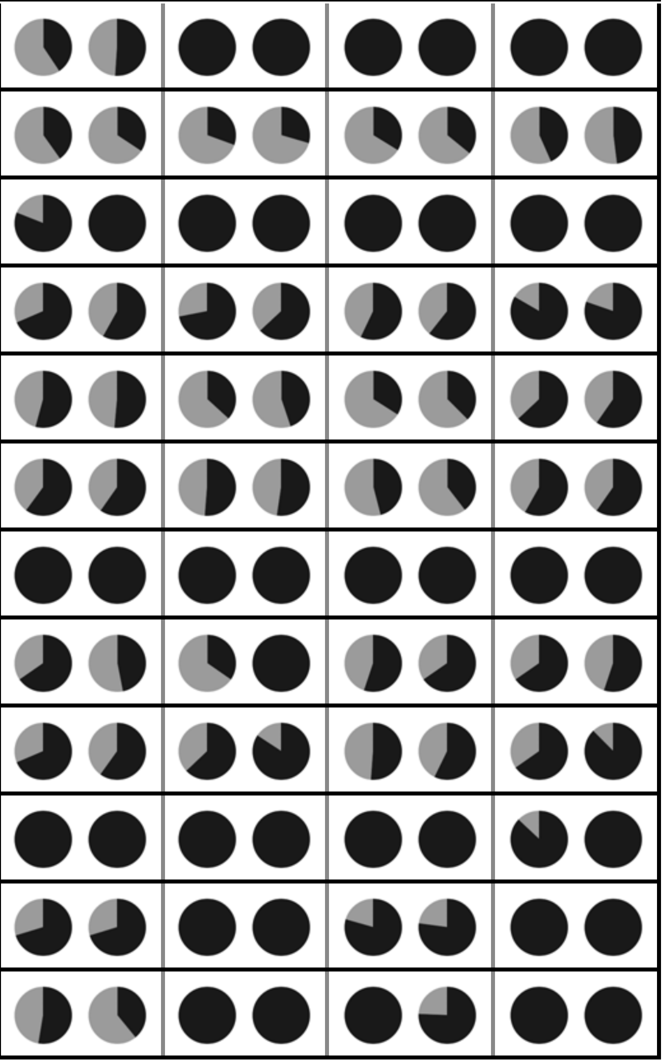

Show detected proteins only
Show all proteins
Filter by category:
ABC Transporter

Proteins found:
1179

Dot Plots Dot Plots

Hendrickson *et al.*

Protein	*Sg* n	*Sg* Coverage	*SgFn* n	*SgFn* Coverage	*SgPg* n	*SgPg* Coverage	*SgPgFn* n	*SgPgFn* Coverage	Description
SGO_2082	9	43.0%	3	17.7%					lipoprotein, putative
	12	50.6%							
SGO_2084	4	27.7%	3	13.3%					NAD(P)H dehydrogenase, quinone family
	5	27.7%	4	25.5%	5	42.0%			
SGO_2085	21	58.7%	12	37.4%	16	50.6%	13	46.4%	purB; adenylosuccinate lyase
	23	56.8%	13	43.6%	17	53.4%	9	32.7%	
SGO_2086	6	15.6%							hypothetical protein SGO_2086
	8	18.9%							
SGO_2094	8	26.0%							general stress protein GSP-781
	11	37.7%							
SGO_2096	16	51.8%							comB; transport protein ComB
	21	60.6%							
SGO_2097	30	47.3%			10	16.0%	3	2.9%	comA; ATP-binding Transport protein ComA
	32	49.7%			8	17.6%			
SGO_2098	17	65.0%	16	69.0%	20	61.6%	21	75.4%	rpsD; ribosomal protein S4
	21	65.5%	16	58.6%	21	66.5%	22	76.4%	
SGO_2099									ABC transporter ATP-binding protein
					4	22.9%			
SGO_2100	14	45.8%	7	27.1%	25	60.5%	18	49.7%	ABC transporter substrate-binding protein
	12	42.5%	5	22.3%	22	59.3%	18	54.2%	
SGO_2102					5	60.4%			hypothetical protein SGO_2102
					5	60.4%	3	45.8%	
SGO_2103			3	21.5%					hydrolase, haloacid dehalogenase-like family
	4	21.0%							

Sg Rep I Rep II | *SgFn* Rep I Rep II | *SgPg* Rep I Rep II | *SgPgFn* Rep I Rep II

Show detected proteins only
Show all proteins
Filter by category:
ABC Transporter

Proteins found:
1179

Dot Plots Dot Plots

Hendrickson *et al.*

Protein	*Sg* n	*Sg* Coverage	*SgFn* n	*SgFn* Coverage	*SgPg* n	*SgPg* Coverage	*SgPgFn* n	*SgPgFn* Coverage	Description
**SGO_2104**	15	48.4%	13	53.7%	13	54.4%	9	34.7%	srtB; sortase B
	18	57.9%	13	51.9%	13	57.2%	9	35.4%	
**SGO_2105**	9	55.9%	11	55.9%	5	39.0%	6	48.2%	abpA; amylase-binding protein AbpA
	12	69.7%	12	69.7%	7	48.2%	6	44.6%	
**SGO_2106**	13	44.2%	14	40.8%	14	43.0%	14	45.5%	ribose-phosphate diphosphokinase
	16	48.0%	15	44.9%	14	40.8%	10	34.3%	
**SGO_2107**	4	22.7%							general stress protein GSP-781
	4	23.5%							
**SGO_2108**	10	55.1%			5	32.4%			mreC; cell shape-determining protein MreC
	11	57.8%			3	20.6%			
**SGO_2133**	32	50.5%	33	53.6%	25	47.4%	19	37.6%	Cell division protein ftsH-like protein
	40	57.6%	26	47.3%	22	40.9%	18	37.9%	
**SGO_2134**	6	26.7%	12	52.8%	11	46.7%	8	38.3%	hpt; hypoxanthine phosphoribosyltransferase
	7	33.3%	12	52.8%	10	46.7%	6	37.2%	
**SGO_2138**									Septum formation initiator family
	5	42.6%							
**SGO_2139**	3	43.2%							S4 RNA-binding domain protein
**SGO_2140**	6	6.6%							transcription-repair coupling factor
	8	9.5%							
**SGO_2141**									pth; peptidyl-tRNA hydrolase
	3	18.5%							
**SGO_2142**	15	56.9%	14	53.4%	16	61.2%	13	44.7%	GTP-binding protein
	17	62.0%	16	58.5%	18	61.2%	15	56.9%	

Sg Rep I Rep II | *SgFn* Rep I Rep II | *SgPg* Rep I Rep II | *SgPgFn* Rep I Rep II

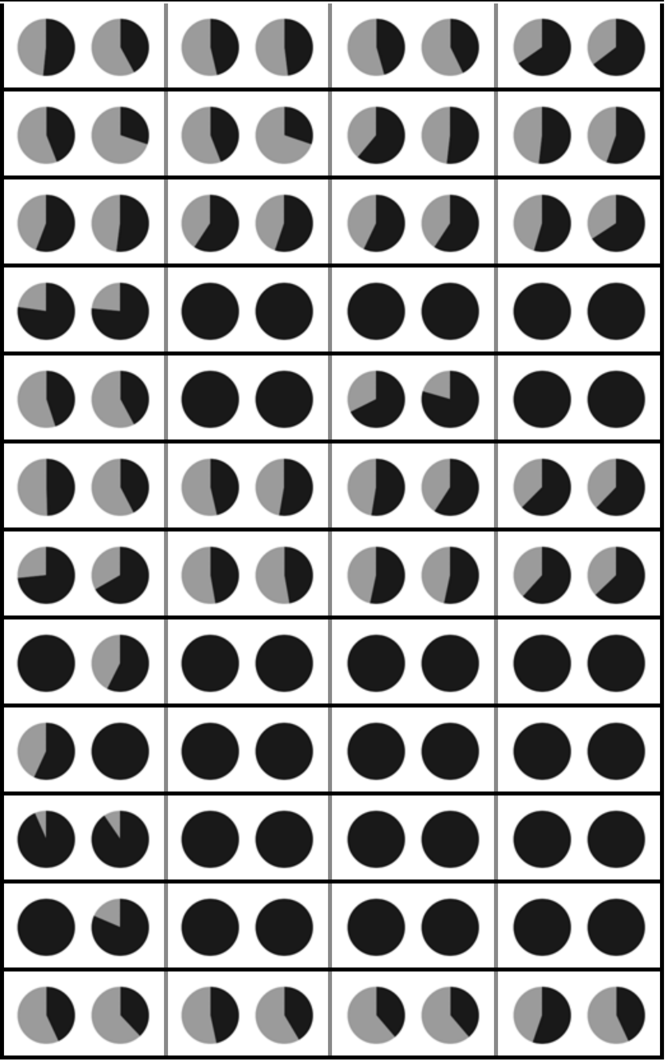

Show detected proteins only
Show all proteins
Filter by category:
ABC Transporter

Proteins found: 1179

Dot Plots Dot Plots

Protein	*Sg*		*SgFn*		*SgPg*		*SgPgFn*		Description	*Sg* / *SgFn* / *SgPg* / *SgPgFn* (Rep I, Rep II)
	n	Coverage	n	Coverage	n	Coverage	n	Coverage		
**SGO_2145**	17	56.5%			15	56.1%	15	56.9%	comE; competence response regulator ComE	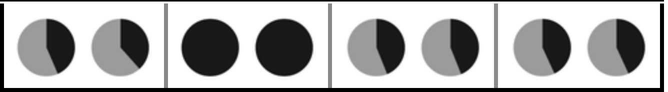
	20	61.6%			17	56.1%	16	56.9%		
**SGO_2146**	11	29.4%			7	23.2%			comD; histidine protein kinase ComD	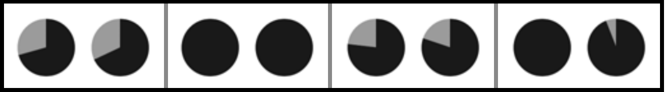
	11	32.0%			6	19.9%	3	6.0%		
**SGO_2150**	11	44.8%	9	34.5%	8	39.3%	7	34.8%	degP; serine protease	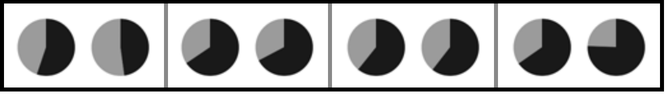
	14	52.1%	9	32.7%	9	39.5%	5	24.4%		

Hendrickson *et al.*
