# Supplementary figures and images for: Proteomics of Streptococcus gordonii within a model developing oral microbial community
Source: BMC Microbiol. 2012 Sep 18;12:211. doi: 10.1186/1471-2180-12-211 (PMC3534352; doi:10.1186/1471-2180-12-211)

q-Value < 0.005

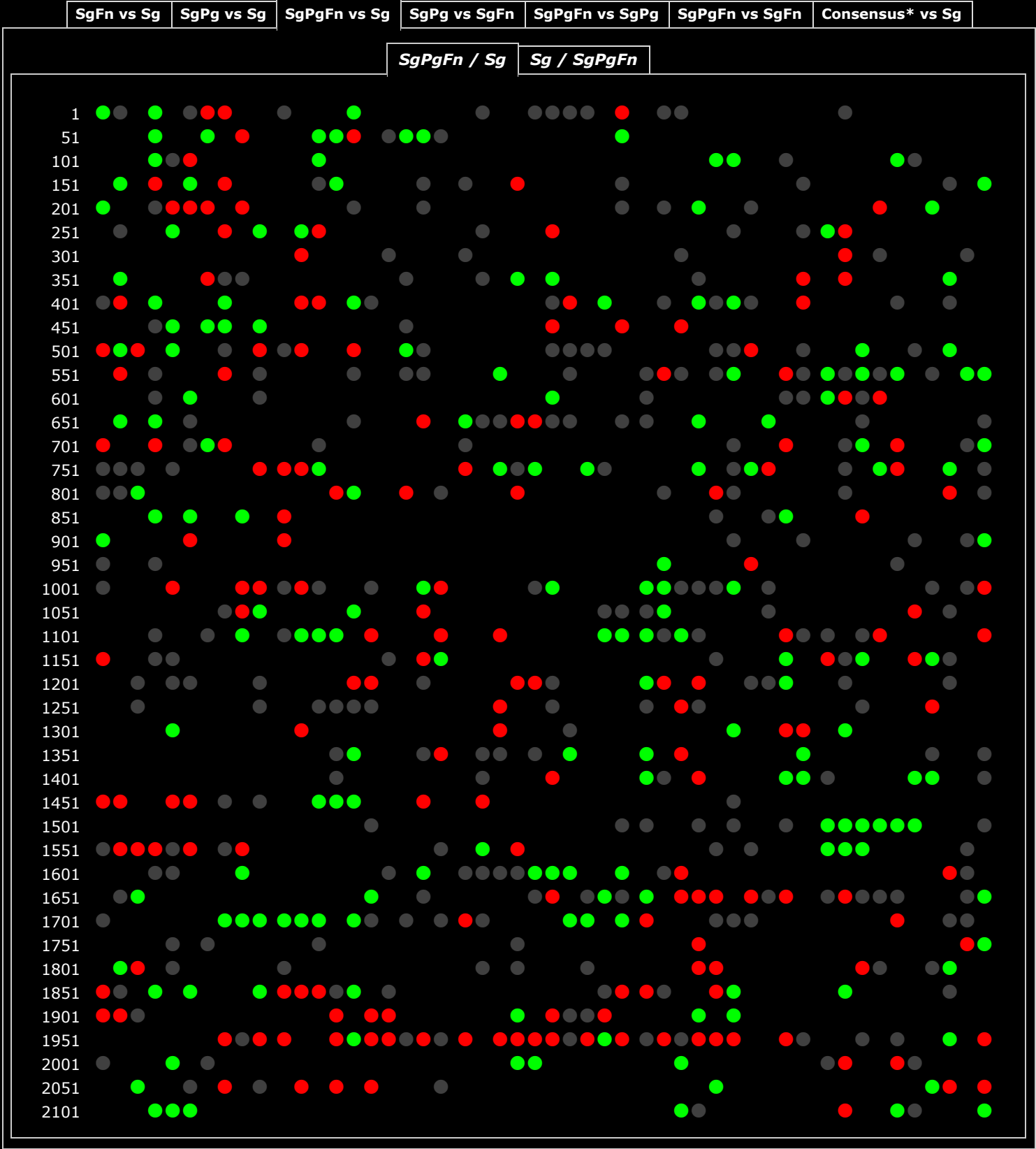

\*a consensus view of those proteins that trend the same in SgPg/Sg and SgPgFn/Sg

Supplement: Additional file 9 — Geneplot_SgPgFn_vs_Sg. A genomic plot of all data collected for S. gordonii protein relative abundance calculations used in the comparison of SgPgFn and the Sg controls. The color code for each SGO number [36] follows that used in the data tables (see Additional files 1, 2, 3, 4, 5, 6, 7), where data was acquired. ORFs coded black were either not used in the annotation or no tryptic fragments were observed. Grey indicates qualitative detection only. [file 1471-2180-12-211-S9.pdf]
