# Supplementary material for: Proteomics of Streptococcus gordonii within a model developing oral microbial community
Source: BMC Microbiol. 2012 Sep 18;12:211. doi: 10.1186/1471-2180-12-211 (PMC3534352; doi:10.1186/1471-2180-12-211)
Supplement: Additional file 10 — Regressplots.pdf. XY regression plots demonstrating the reproducibility of the spectral counting mass spectrometry data for the technical and biological replicates, with an explanatory note. [file 1471-2180-12-211-S10.pdf]

Introductory comments for regression plots of technical and biological replicates, with comments on the normalization procedure

Sample reproducibility is vital with respect to distinguishing significant changes between different biological states from random variation, be it variation due to the measurement process itself or due to minor variations in protein expression that are real but not of great biological interest. Regression plots are shown below to demonstrate the reproducilibity of both the technical and biological replicates used in this study. Log_2 spectral count numbers are displayed for either the two technical replicates used for each biological replicate or the two biological replicates for each experimental condition. As discussed in the references cited in the main text, spectral counts for the biological replicates were derived by averaging the values of the technical replicates, so the technical replicate comparisons shown here use the raw spectral counts. However, biological replicates were compared individually across conditions using spectral counts normalized to the highest total spectral counts. To be consistent with the data analysis in the main text the biological replicate comparisons below use normalized spectral counts. The data showed strong correlations and tight grouping across all biological and technical comparisons, allowing sensitive determination of protein relative abundance differences between conditions. As is normally the case, lower overall spectral counts, at the bottom left of the figures, were not as reproducible as high counts and tend towards a limited number of quantized values. Under our conditions, proteins with count values of less than 16 on a linear scale are considered qualitatively detected, but seldom yield a meaningful or reproducible relative abundance ratio. As noted in the context of microarray data by Wei *et al*., (2004) we find trends to be reproducible, but the actual magnitudes of the relative abundance ratios can vary considerably. Reproducible trends, using the proteomic methods and organisms described here, can be accomplished with two or three biological replicates. Based on formal power analyses, to achieve significant level of reproducibility for the mean magnitudes (+/- 10%) would require potentially tens of biological replicates, similarly to what Wei *et al*. observed for their array data. Random variation in the mass spectra might prove to be a limiting factor. Obviously, given the expense involved and the limited additional information that would be gained, it makes more sense to deeply sample (Hackett, 2007) a smaller number of replicates and to emphasize trends rather than absolute magnitudes of abundance ratios.

The normalization scheme is necessary to compensate for different detection levels between the samples. In pure *S. gordonii* samples the entire biomass consists of *S. gordonii* cells and all of the recovered peptides should belong to this organism. In the triple species samples only a third of the biomass comes from *S. gordonii*. As expected, the *S. gordonii* peptide recovery from the three species samples were only around a third of those recovered from the pure samples. Without normalization virtually every protein would be considered reduced when comparing the three organism sample to *S. gordonii* alone. However, this would not represent a biological change but rather an artifact of the sampling procedure. The normalization assumes that total protein mass within the cells is fairly constant between samples. Barring a

measurement of the total protein within individual cells for each sample, this is the only plausible assumption for the calculations.

For samples with dramatically different peptide recoveries dynamic range can be a problem. Falling outside of the optimal dynamic range can cause distortions at the high and low ends of the detection range. For this reason, we do not use samples that would require large normalization factors (10 fold or more) for analysis. The largest normalization used in the paper is the expected 3-fold normalization comparing Sg Pg Fn with the pure Sg sample. It is possible that distortions exist even at this level of normalization. However, given the nature of the samples a 3-fold normalization is simply a factor that has to be accepted.

A convenient example of the effect of normalization on the readout for a specific ORF is SGO_0007, the trpS; tryptophanyl-tRNA synthetase, in the SgPgFn comparison to SgFn, see Table 10 in the main text. As mentioned in the methods, the portion dealing with protein abundance ratio calculations, the significance is not derived from a single comparison, but rather a cross comparison of each of the two SgPgFn biological replicates with each of the two SgFn replicates using a *t*-test, as these are independent rather than paired samples. While the non-normalized 13 counts might seem little different from 9 counts, it seems much larger compared to the 5.5 counts of the other SgFn biological replicate, especially after normalization. The four comparisons produce an average ratio between SgPgFn and SgFn as well as a *p*-value derived from the *t* statistic. In this case the ratio was a $\log_2$ of 1.079 with a *p*-value of 0.0031. As mentioned in the methods section, the *p*-value was used to calculate a *q*-value in order to adjust for multiple hypothesis testing and a *q*-value cutoff for statistical significance was used of 0.005, a fairly conservative cutoff. Additionally, prior to the application of statistical calculations to transcriptome microarrays, the intellectual forefather of whole cell proteomic data analysis, a two-fold change was considered sufficient in and of itself to identify a potential candidate for altered levels between treatments.

While the recovery of specific proteins, rather than the proteome as a whole, might differ between extractions or biological replicates it would be impossible to detect without an entirely separate measurement of protein levels for each individual protein. In addition, the plots of the biological replicates against each other provided in the supplemental material below indicate that the extractions themselves were relatively reproducible. Different conditions might result in a reproducible change in extraction efficiency of a specific protein, resulting in an error. However, given the signal/noise ratios of these measurements such errors seem unlikely to alter the overall picture and subsequent interpretation.

References

Wei C, Li J, Bumgarner RE: **Sample size for detecting differentially expressed genes in microarray experiments.** *BMC Genomics* 2004, **5**:87.

Hackett M: **Science, marketing and wishful thinking in quantitative proteomics.** *Proteomics* 2008, **8**: 4618-4623.

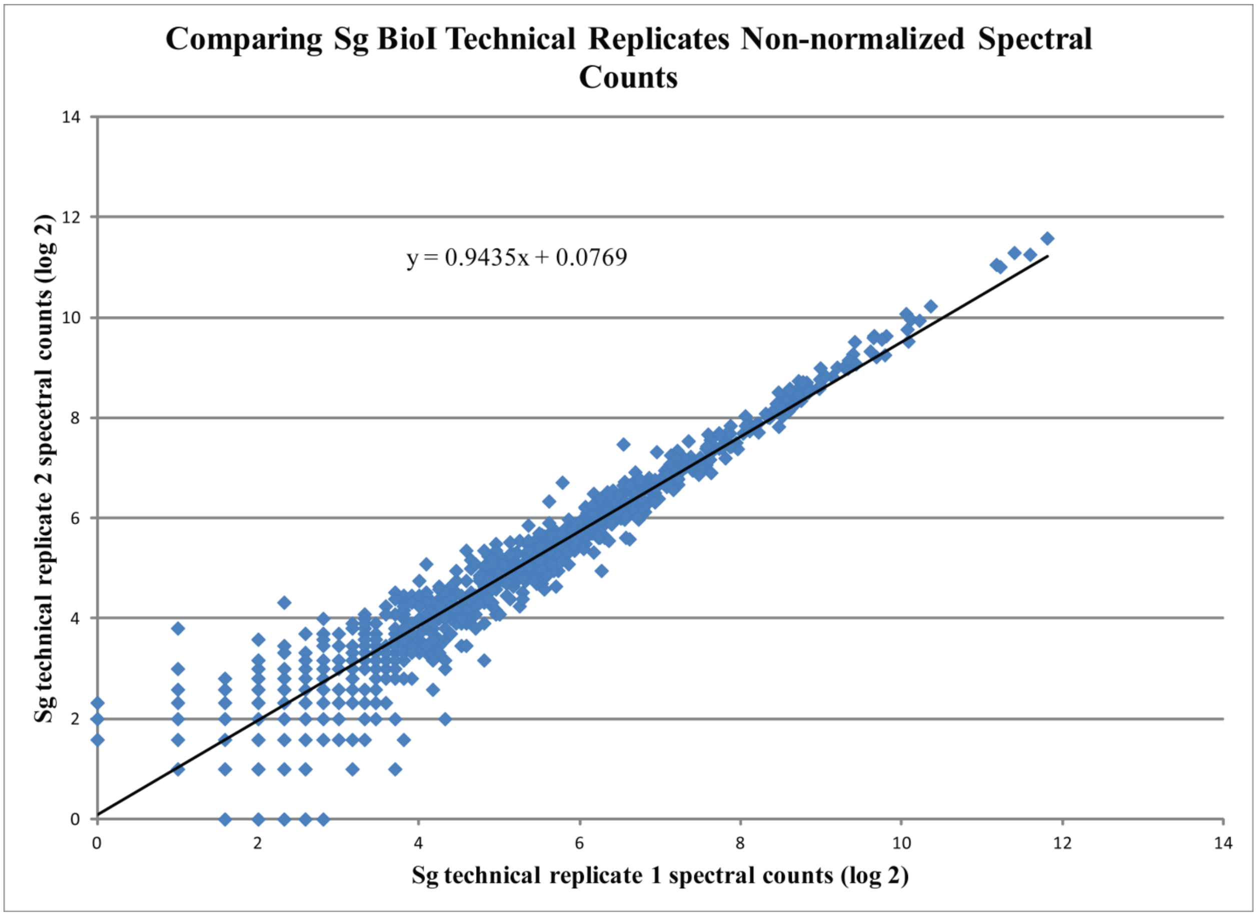
Comparing Sg BioI Technical Replicates Non-normalized Spectral Counts
y = 0.9435x + 0.0769
Sg technical replicate 2 specetral counts (log 2)
Sg technical replicate 1 spectral counts (log 2)
0
2
4
6
8
10
12
14

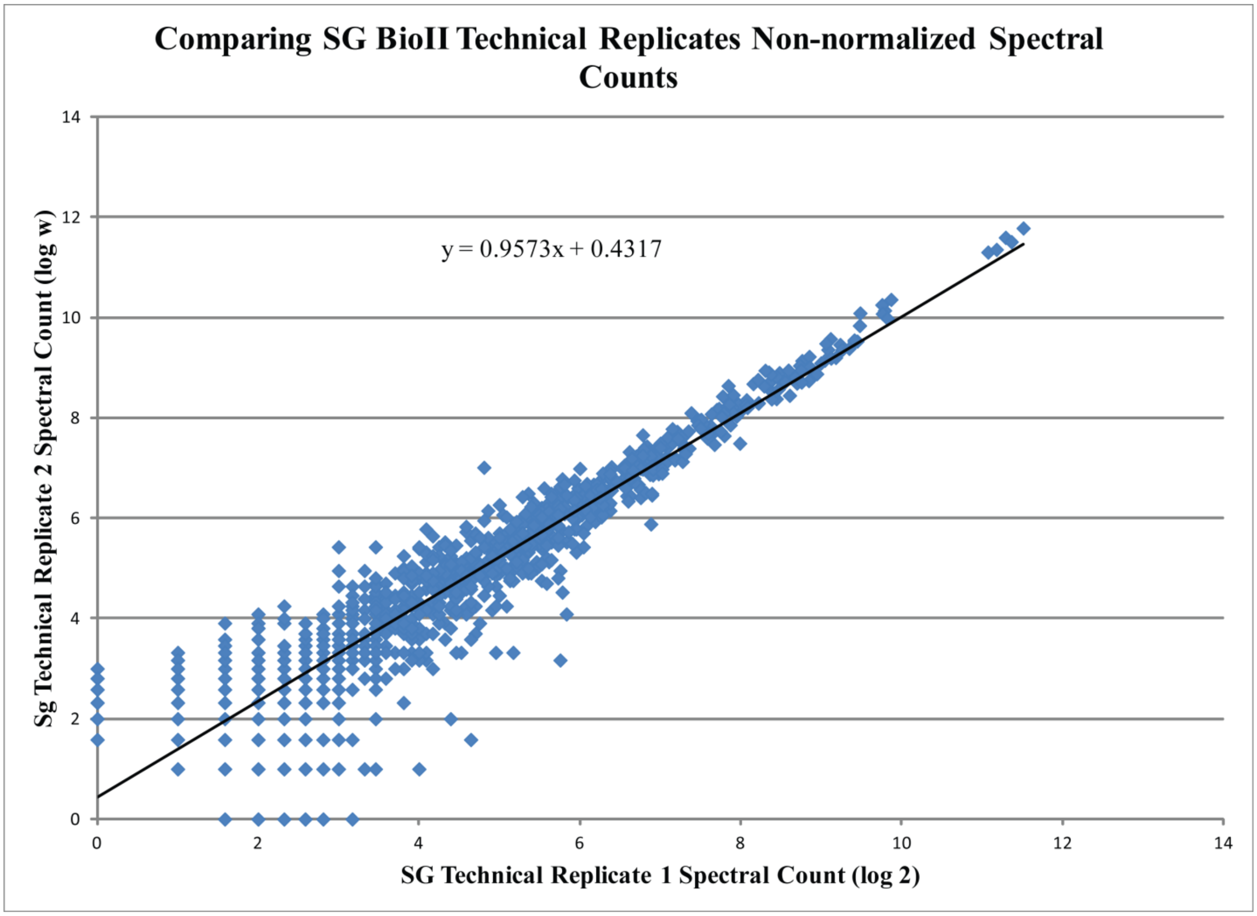
Comparing SG BioII Technical Replicates Non-normalized Spectral Counts
y = 0.9573x + 0.4317
Sg Technical Replicate 2 Spectral Count (log w)
SG Technical Replicate 1 Spectral Count (log 2)
0
2
4
6
8
10
12
14

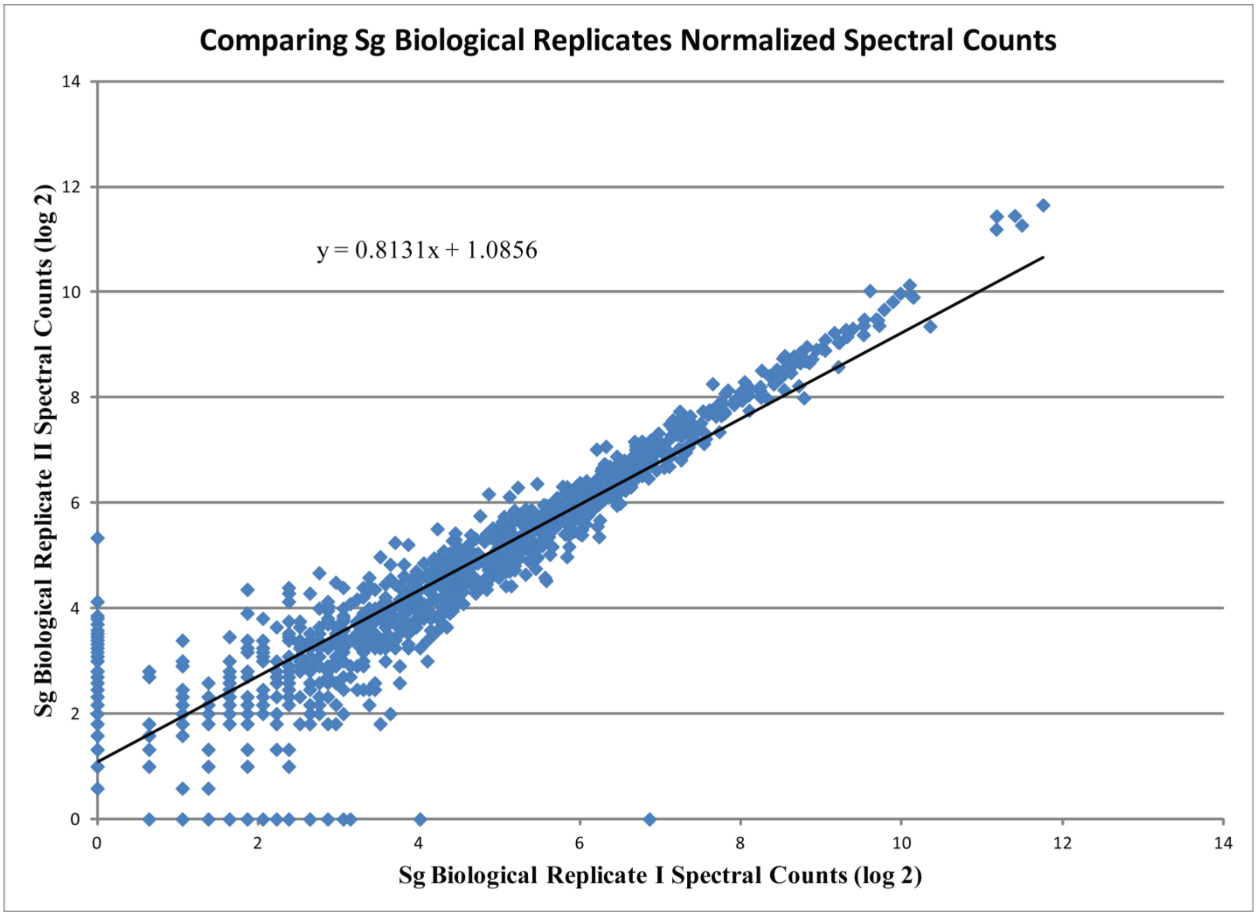
Comparing Sg Biological Replicates Normalized Spectral Counts
y = 0.8131x + 1.0856
Sg Biological Replicate II Spectral Counts (log 2)
Sg Biological Replicate I Spectral Counts (log 2)
0
2
4
6
8
10
12
14

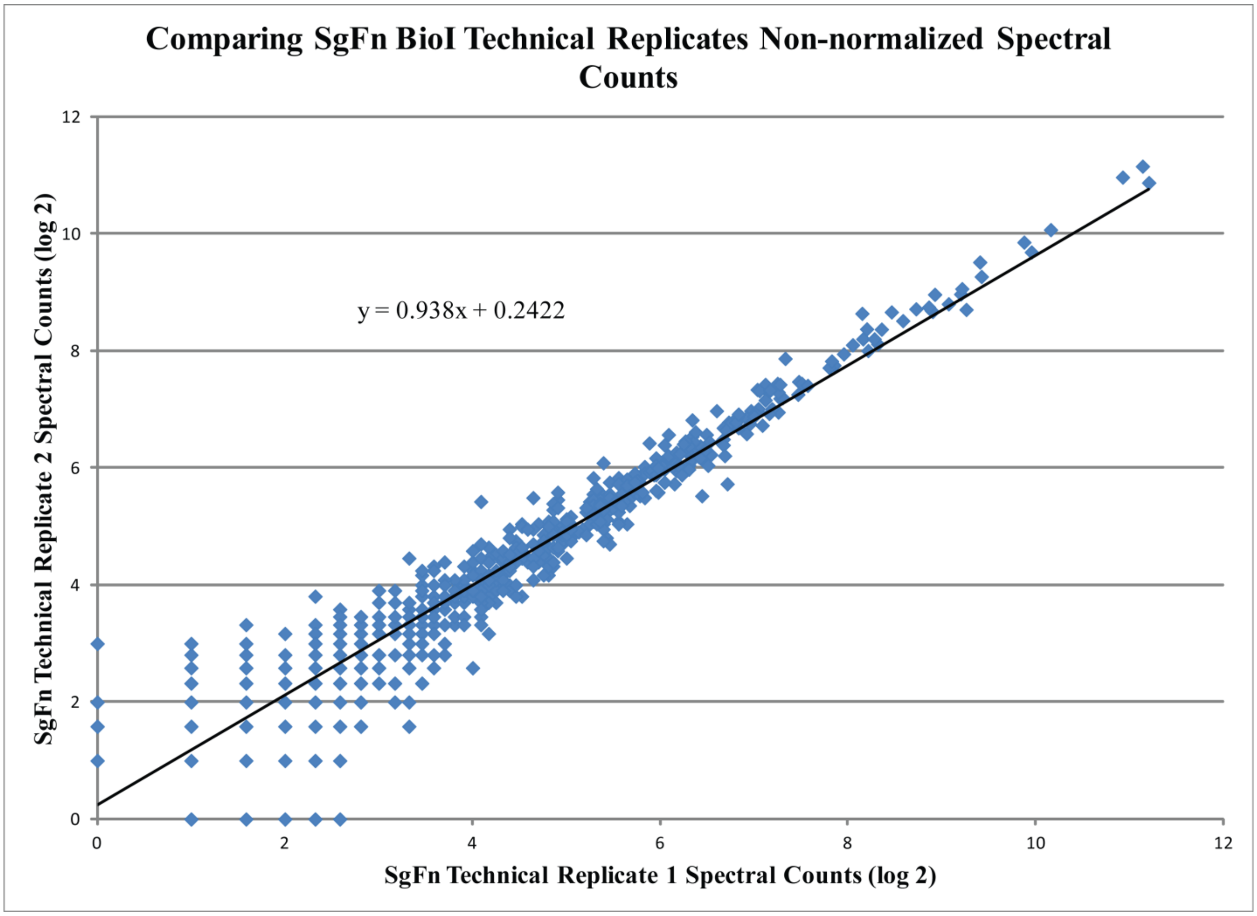

Comparing SgFn BioI Technical Replicates Non-normalized Spectral Counts
y = 0.938x + 0.2422
SgFn Technical Replicate 2 Spectral Counts (log 2)
SgFn Technical Replicate 1 Spectral Counts (log 2)
0
2
4
6
8
10
12

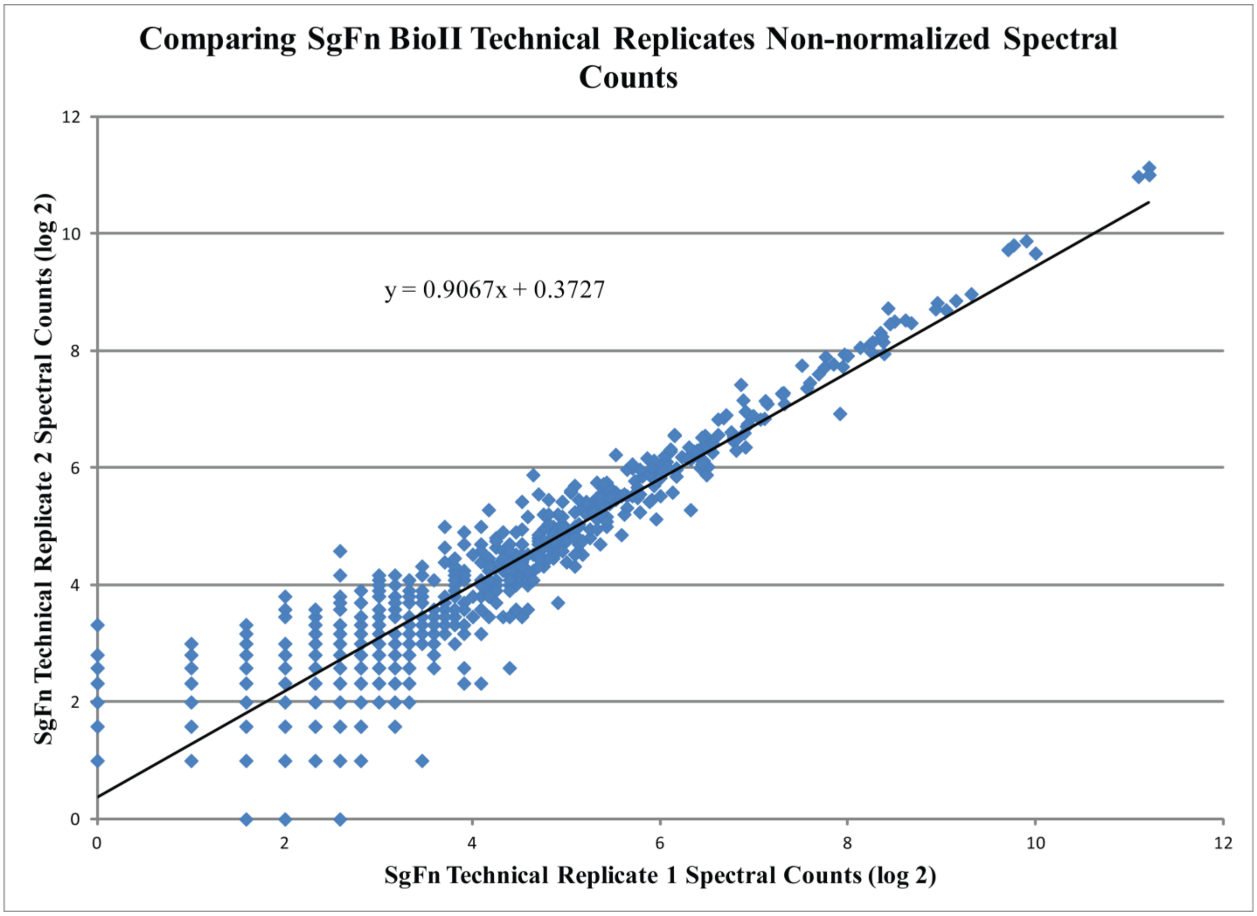
Comparing SgFn BioII Technical Replicates Non-normalized Spectral Counts
y = 0.9067x + 0.3727
SgFn Technical Replicate 2 Spectral Counts (log 2)
SgFn Technical Replicate 1 Spectral Counts (log 2)
0
2
4
6
8
10
12

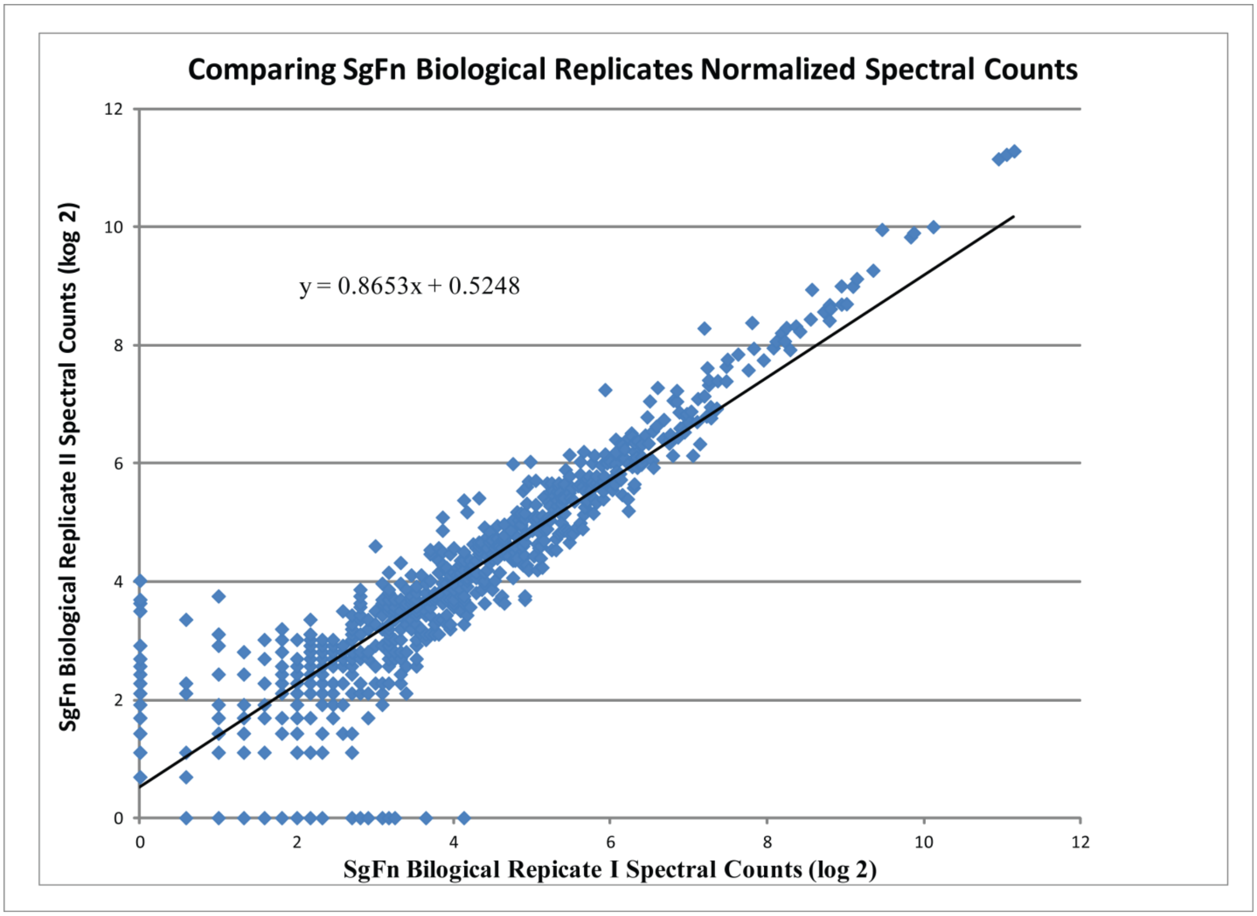
Comparing SgFn Biological Replicates Normalized Spectral Counts
y = 0.8653x + 0.5248
SgFn Biological Replicate II Spectral Counts (kog 2)
SgFn Bilogical Repicate I Spectral Counts (log 2)
0
2
4
6
8
10
12

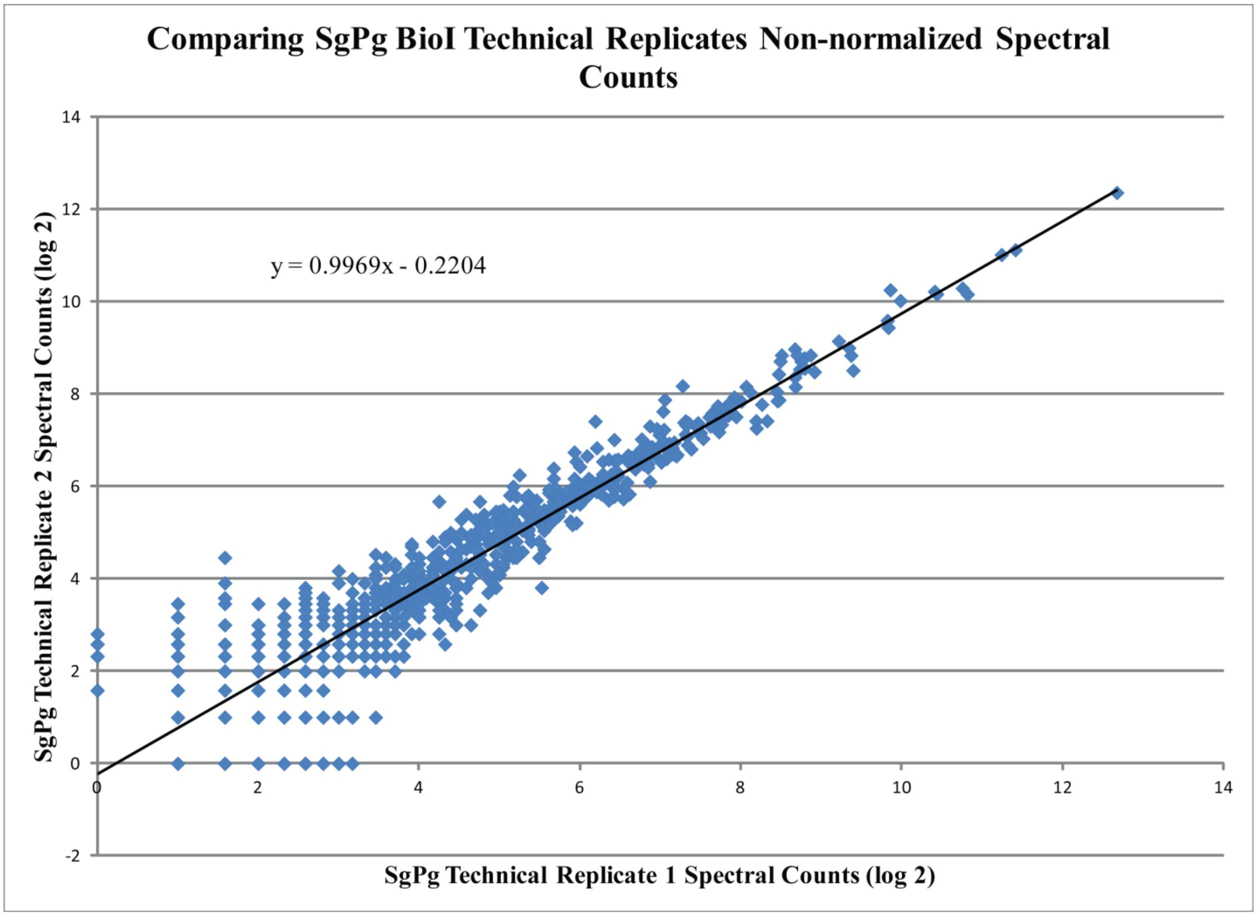

Comparing SgPg BioI Technical Replicates Non-normalized Spectral Counts
y = 0.9969x - 0.2204
SgPg Technical Replicate 2 Spectral Counts (log 2)
SgPg Technical Replicate 1 Spectral Counts (log 2)
14
12
10
8
6
4
2
0
-2
0
2
4
6
8
10
12
14

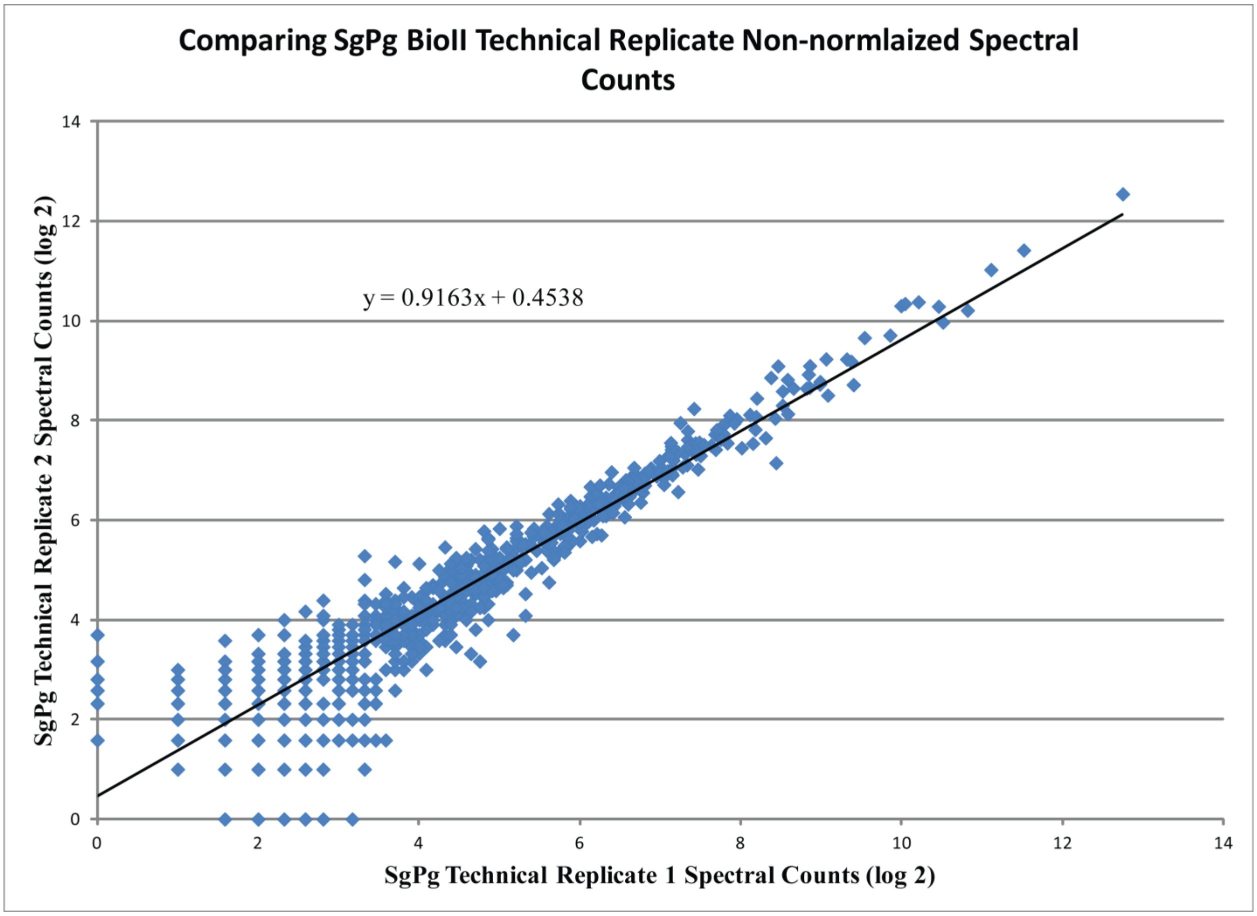
Comparing SgPg BioII Technical Replicate Non-normlaized Spectral Counts
y = 0.9163x + 0.4538
SgPg Technical Replicate 2 Spectral Counts (log 2)
SgPg Technical Replicate 1 Spectral Counts (log 2)
0
2
4
6
8
10
12
14

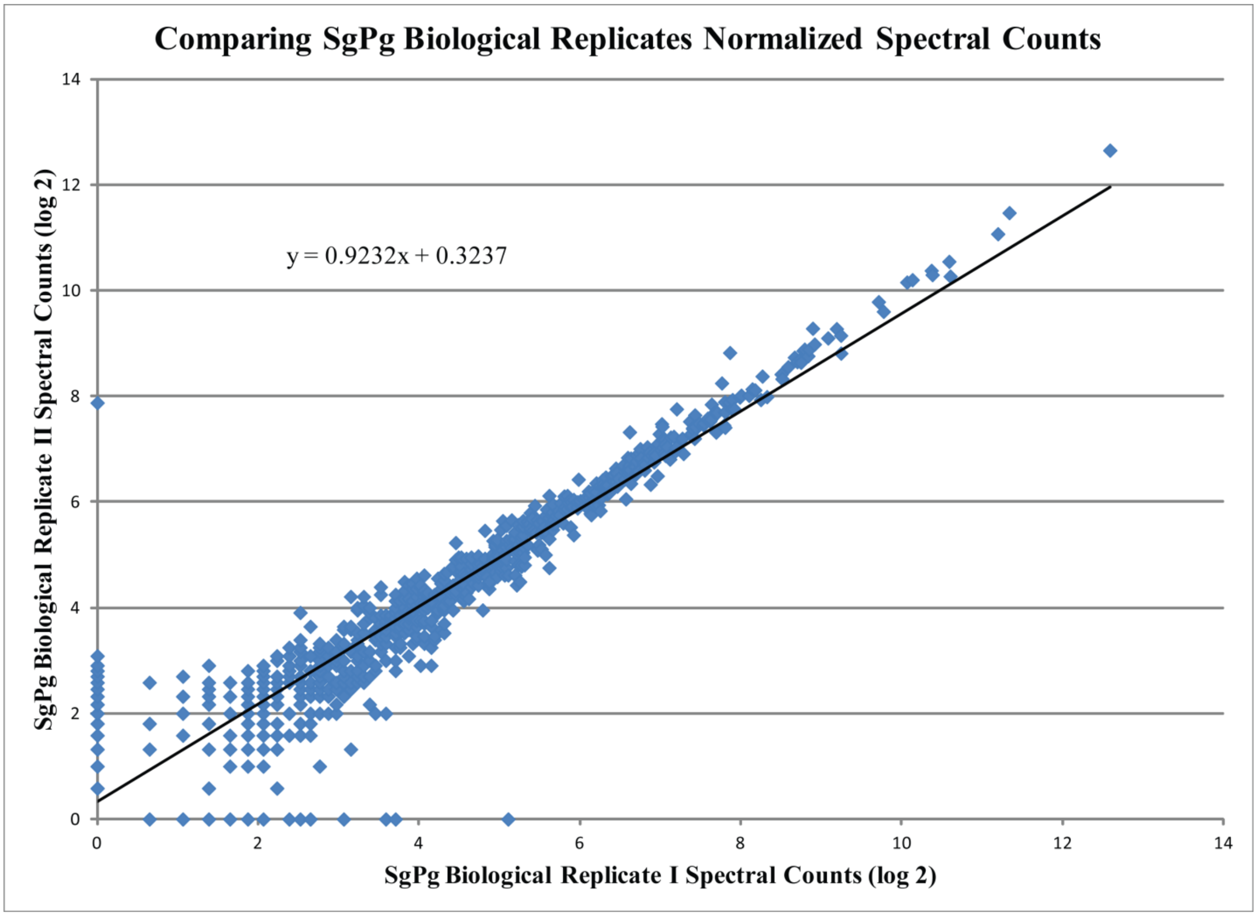
Comparing SgPg Biological Replicates Normalized Spectral Counts
y = 0.9232x + 0.3237
SgPg Biological Replicate II Spectral Counts (log 2)
SgPg Biological Replicate I Spectral Counts (log 2)
0
2
4
6
8
10
12
14

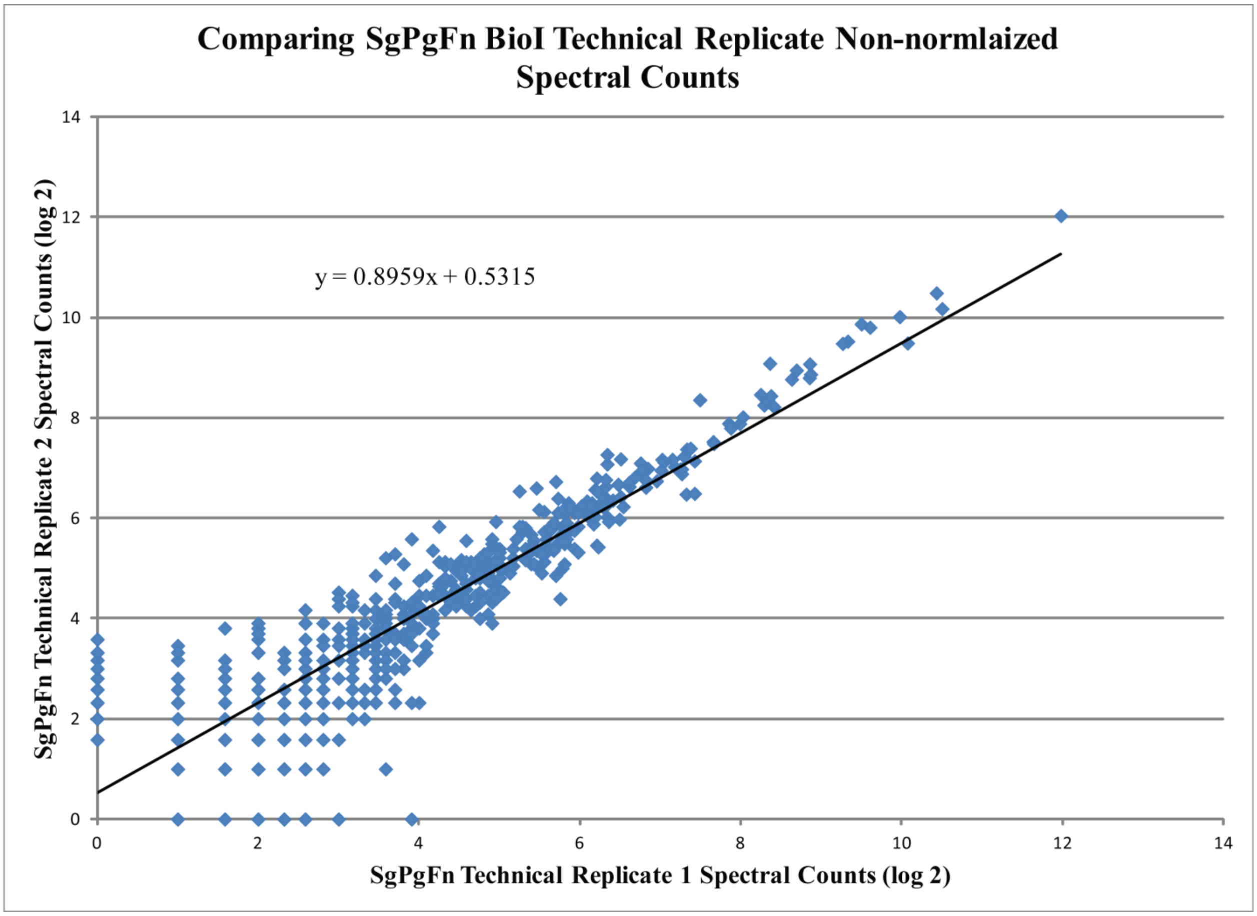
Comparing SgPgFn BioI Technical Replicate Non-normlaized
Spectral Counts
y = 0.8959x + 0.5315
SgPgFn Technical Replicate 2 Spectral Counts (log 2)
SgPgFn Technical Replicate 1 Spectral Counts (log 2)
0
2
4
6
8
10
12
14

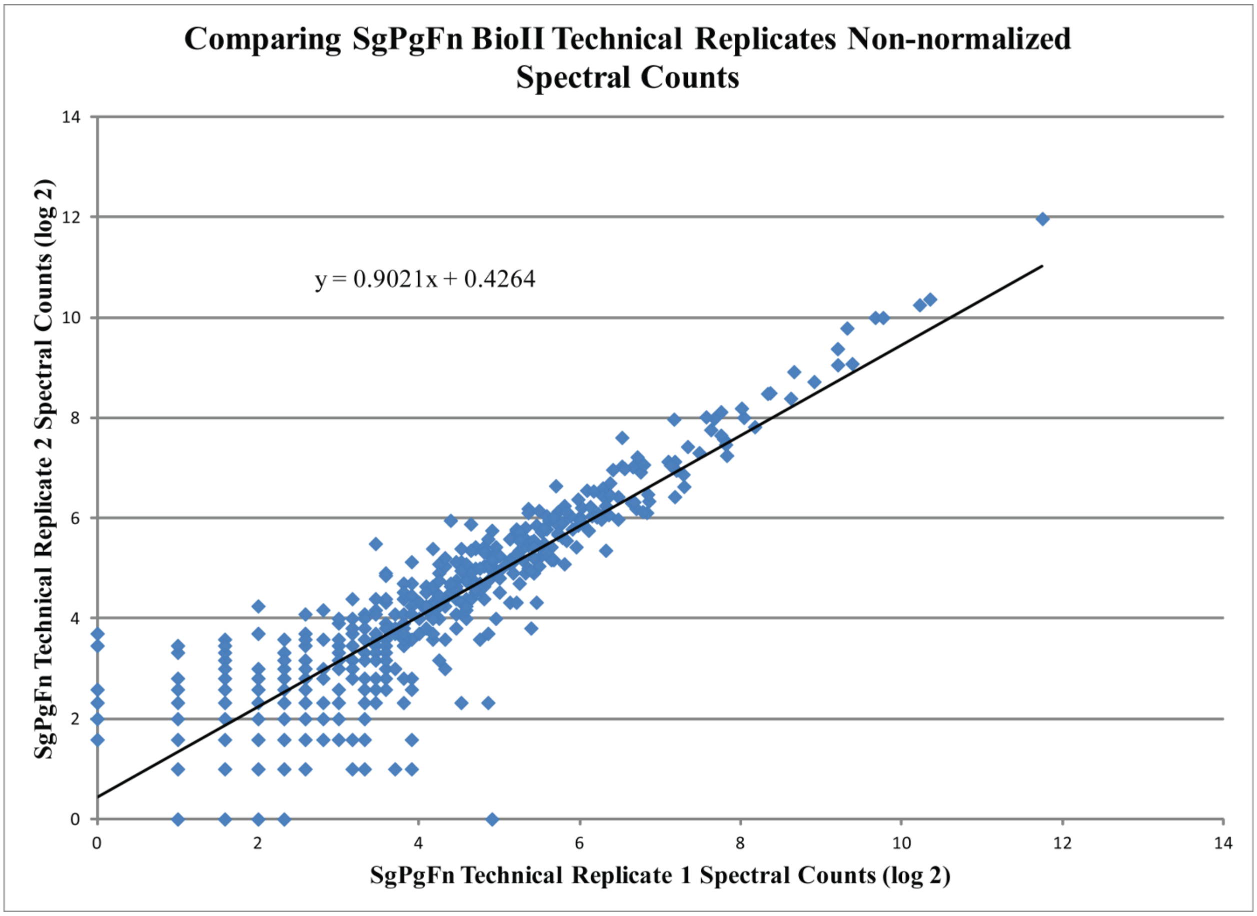
Comparing SgPgFn BioII Technical Replicates Non-normalized Spectral Counts
y = 0.9021x + 0.4264
SgPgFn Technical Replicate 2 Spectral Counts (log 2)
SgPgFn Technical Replicate 1 Spectral Counts (log 2)
0
2
4
6
8
10
12
14

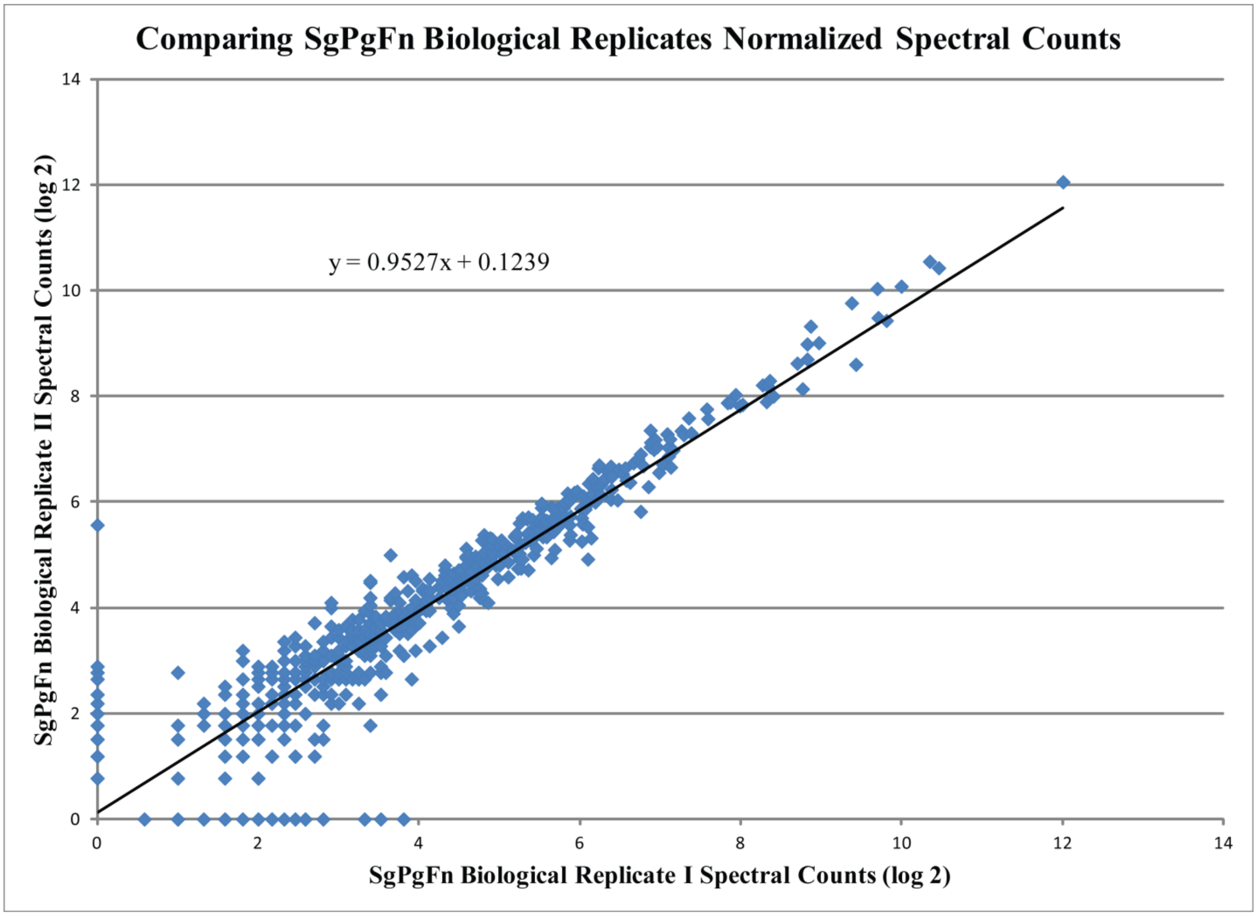

Comparing SgPgFn Biological Replicates Normalized Spectral Counts
y = 0.9527x + 0.1239
SgPgFn Biological Replicate II Spectral Counts (log 2)
SgPgFn Biological Replicate I Spectral Counts (log 2)
0
2
4
6
8
10
12
14
